# Supplementary figures and images for: Nuclear translocation of SIRT4 mediates deacetylation of U2AF2 to modulate renal fibrosis through alternative splicing-mediated upregulation of CCN2 (part 1 of 9)
Source: eLife. 2024 Nov 4;13:RP98524. doi: 10.7554/eLife.98524 (PMC11534337; doi:10.7554/eLife.98524)

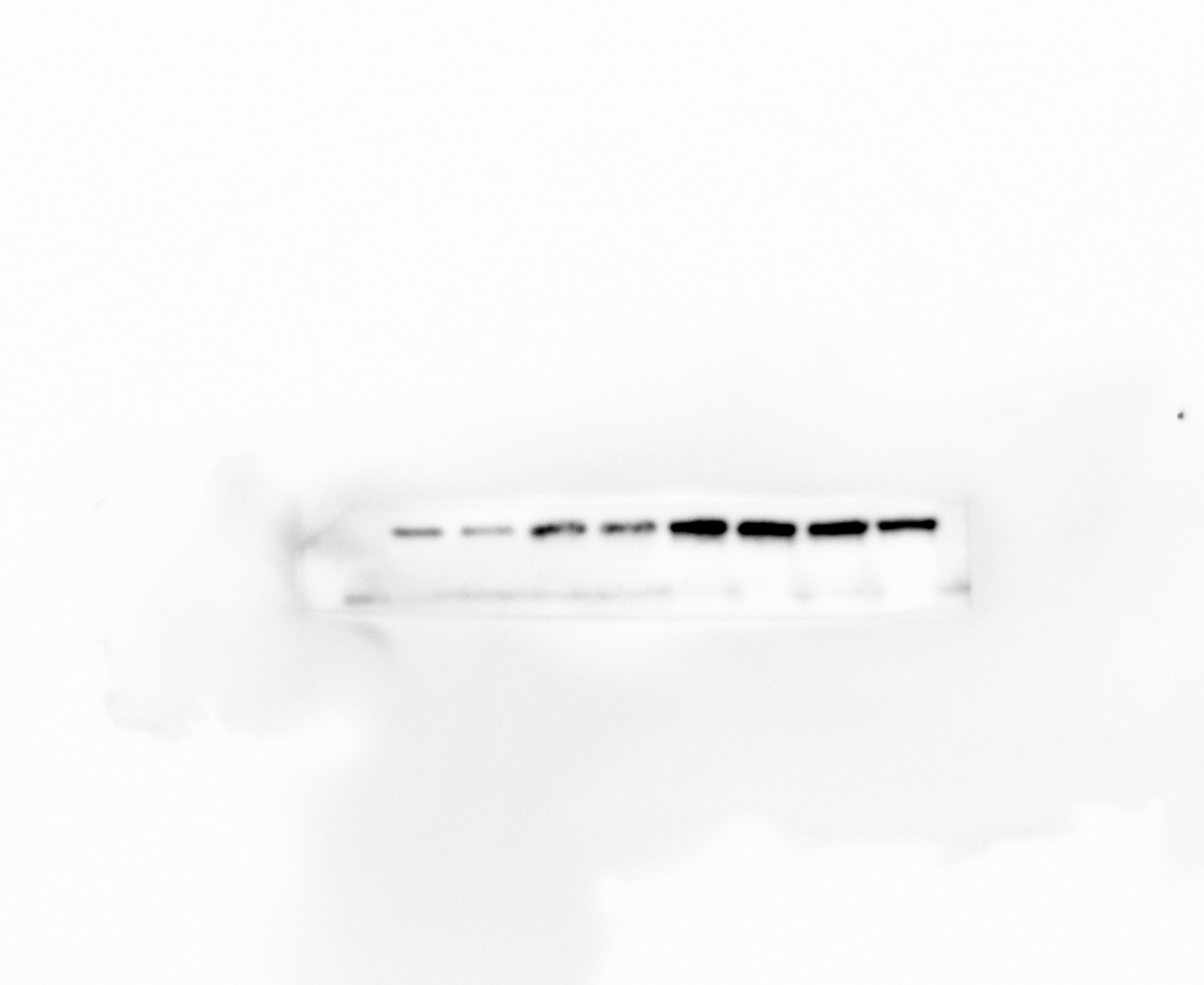

Supplement: Figure 1—source data 1. [file elife-98524-fig1-data1.zip › Fig 1-data1-v1/1A/bottom/COL1A1.tif]

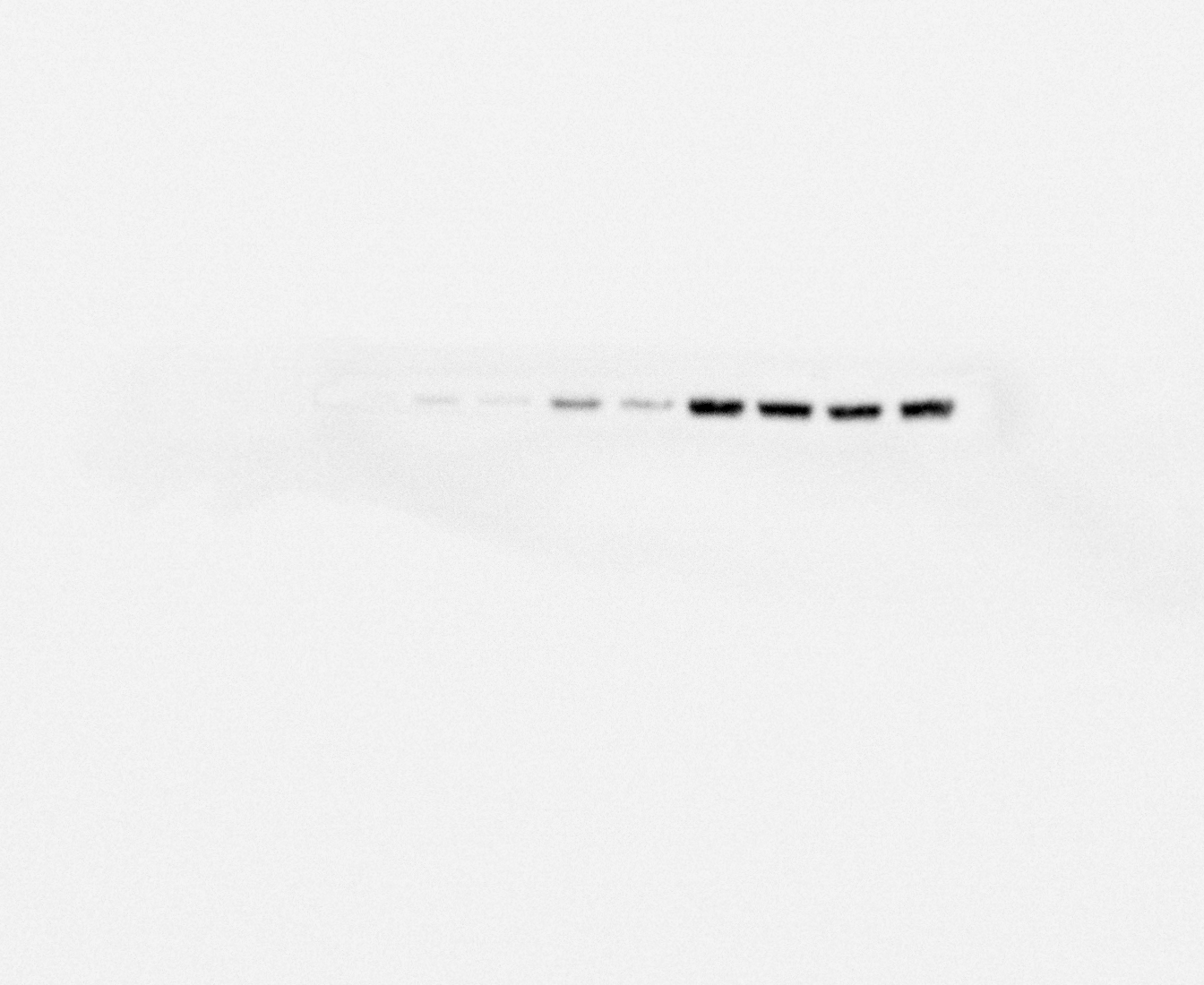

Supplement: Figure 1—source data 1. [file elife-98524-fig1-data1.zip › Fig 1-data1-v1/1A/bottom/FN1.tif]

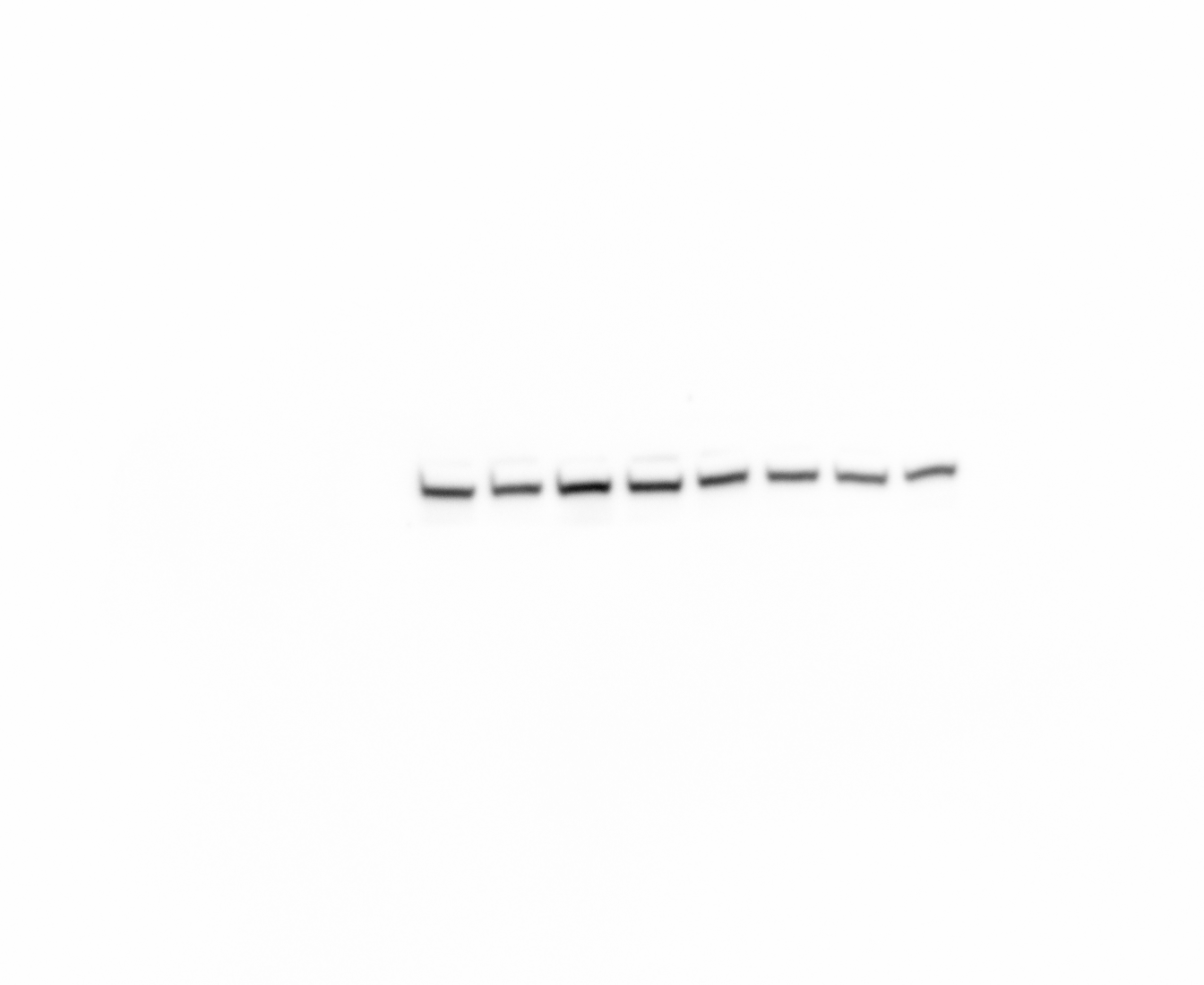

Supplement: Figure 1—source data 1. [file elife-98524-fig1-data1.zip › Fig 1-data1-v1/1A/bottom/SIRT4.tif]

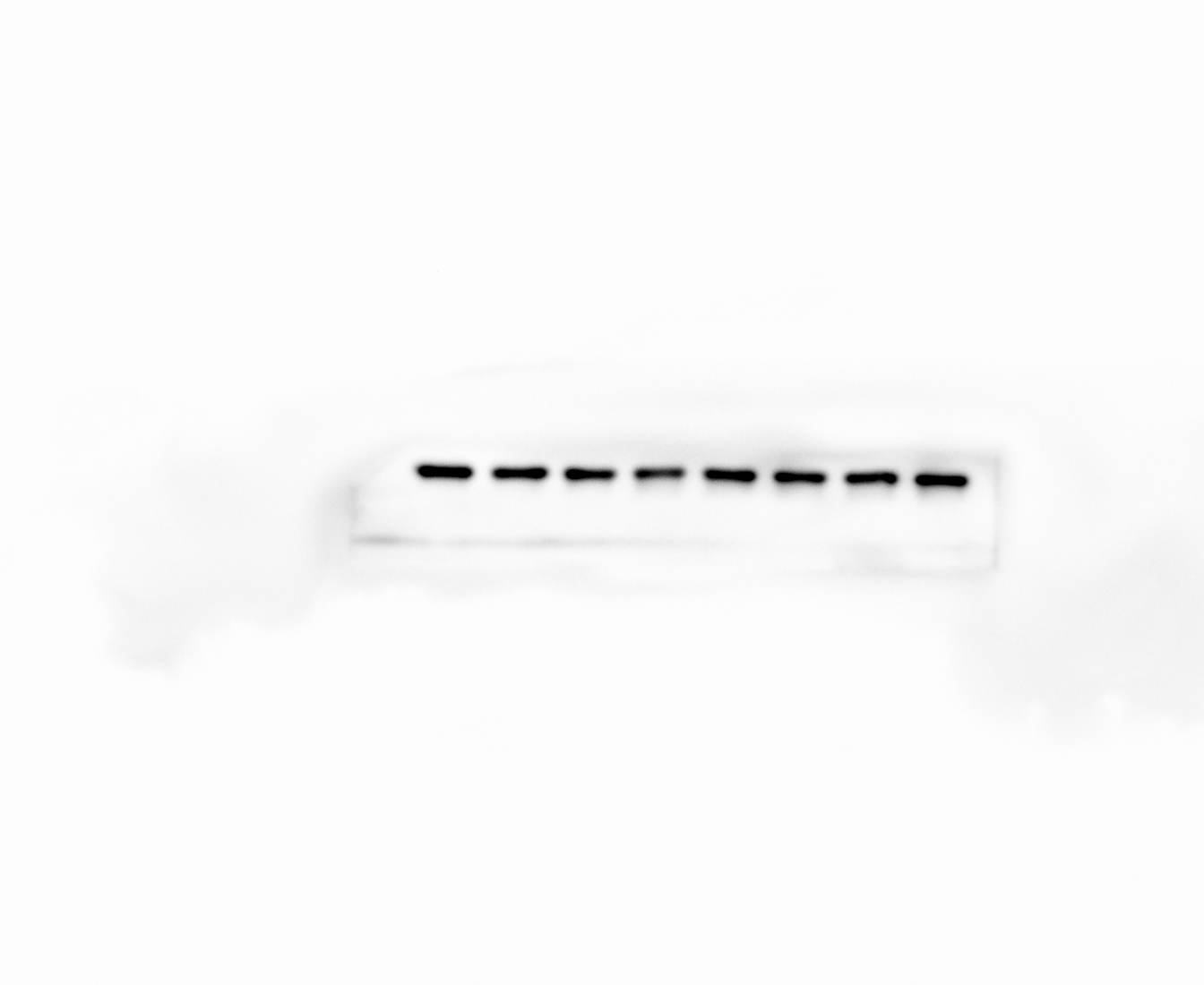

Supplement: Figure 1—source data 1. [file elife-98524-fig1-data1.zip › Fig 1-data1-v1/1A/bottom/Tubulin.tif]

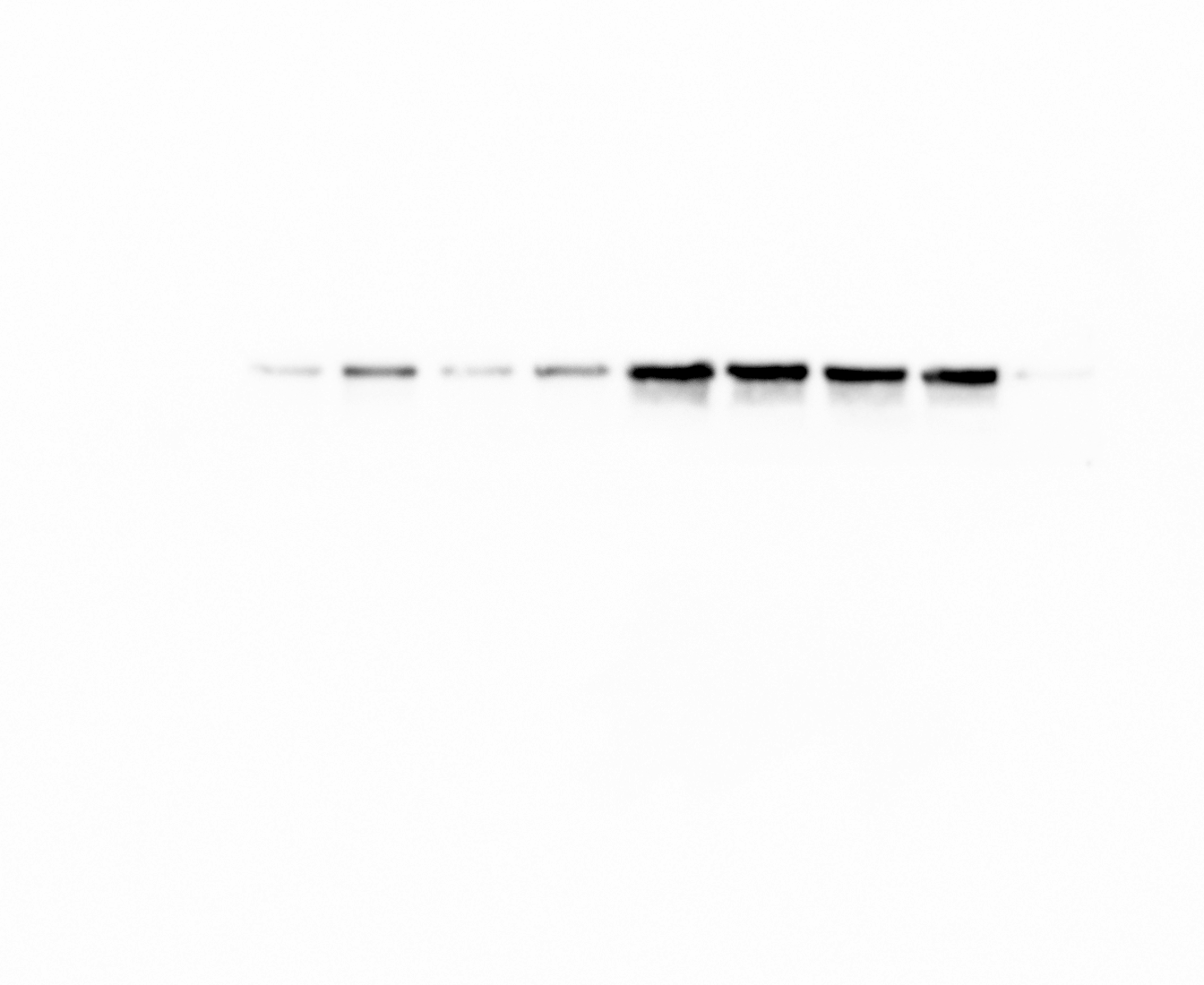

Supplement: Figure 1—source data 1. [file elife-98524-fig1-data1.zip › Fig 1-data1-v1/1A/upper/COL1A1.tif]

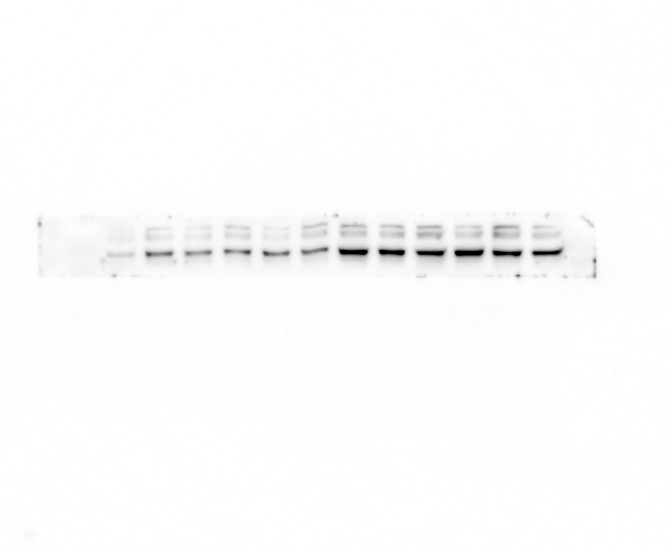

Supplement: Figure 1—source data 1. [file elife-98524-fig1-data1.zip › Fig 1-data1-v1/1A/upper/FN1.tif]

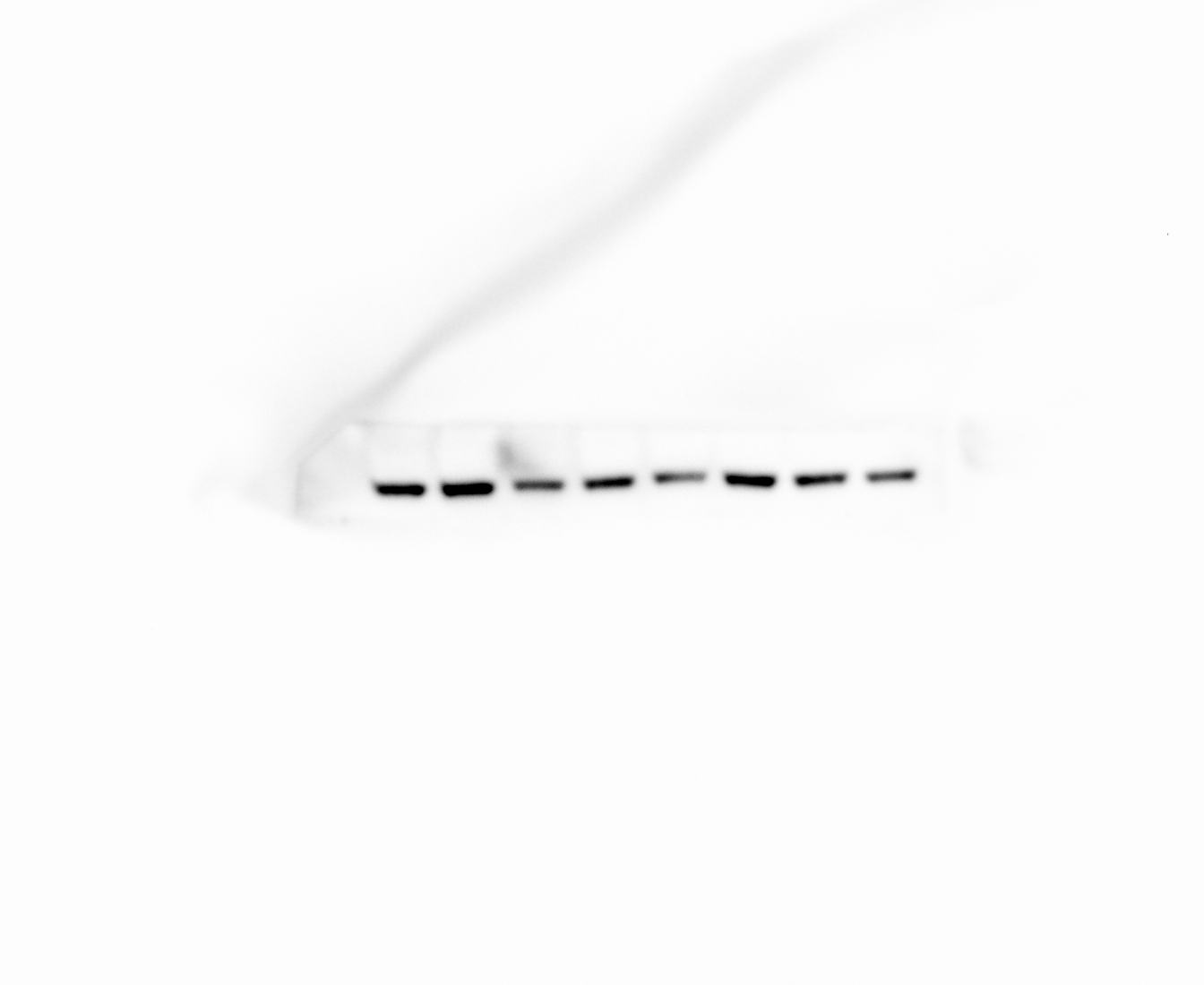

Supplement: Figure 1—source data 1. [file elife-98524-fig1-data1.zip › Fig 1-data1-v1/1A/upper/SIRT4.tif]

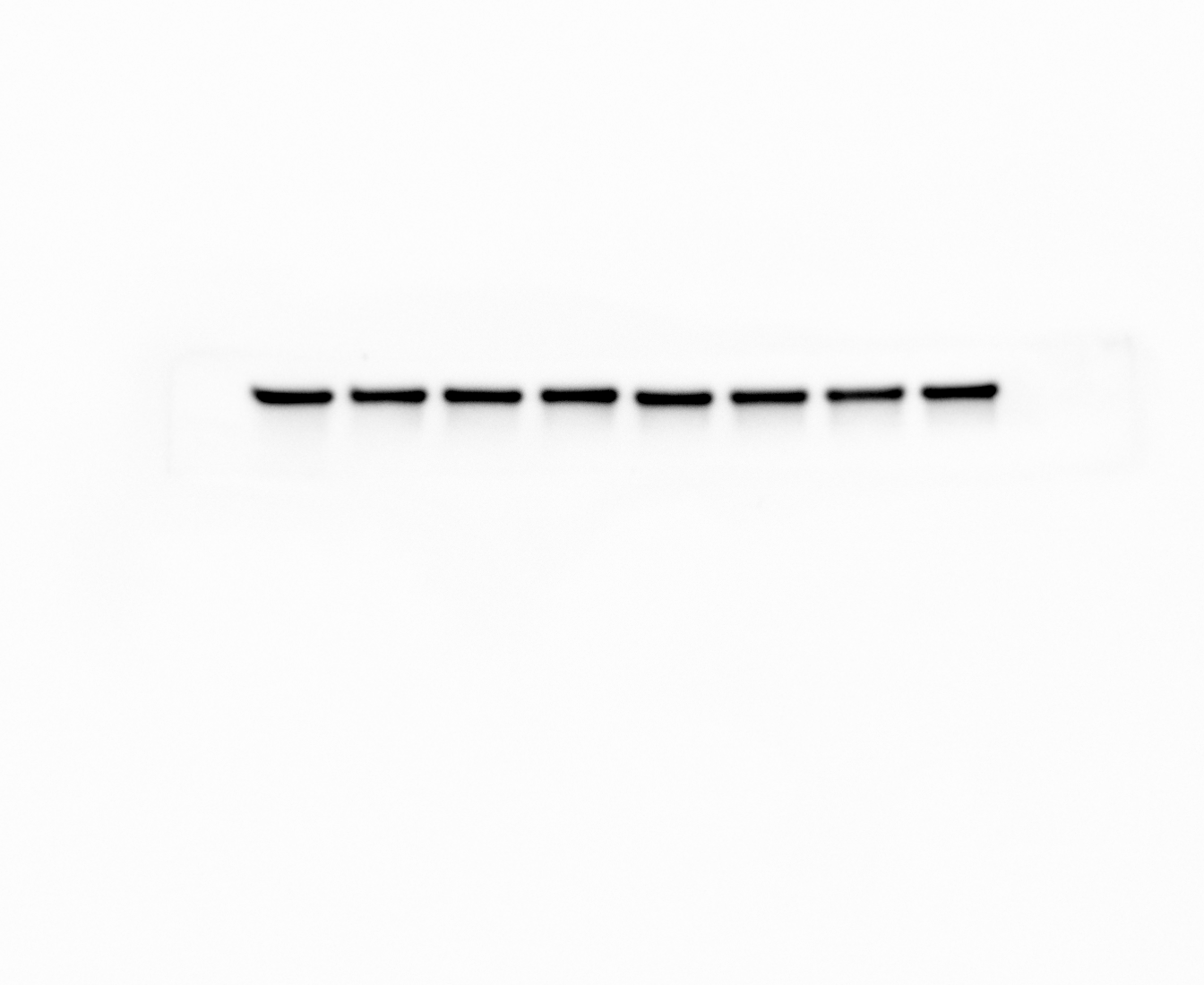

Supplement: Figure 1—source data 1. [file elife-98524-fig1-data1.zip › Fig 1-data1-v1/1A/upper/Tubulin.tif]

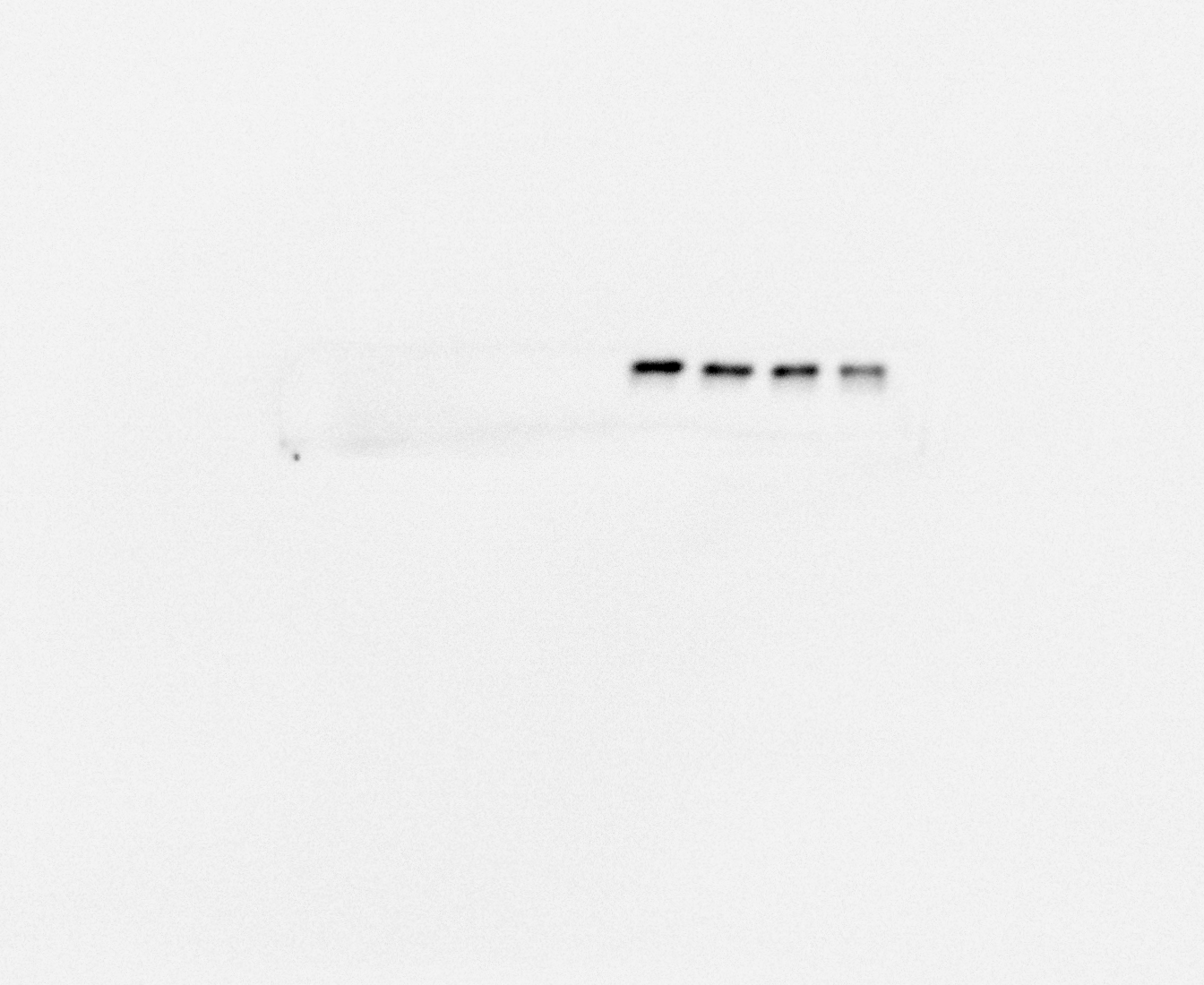

Supplement: Figure 1—source data 1. [file elife-98524-fig1-data1.zip › Fig 1-data1-v1/2B/bottom/PCNA.tif]

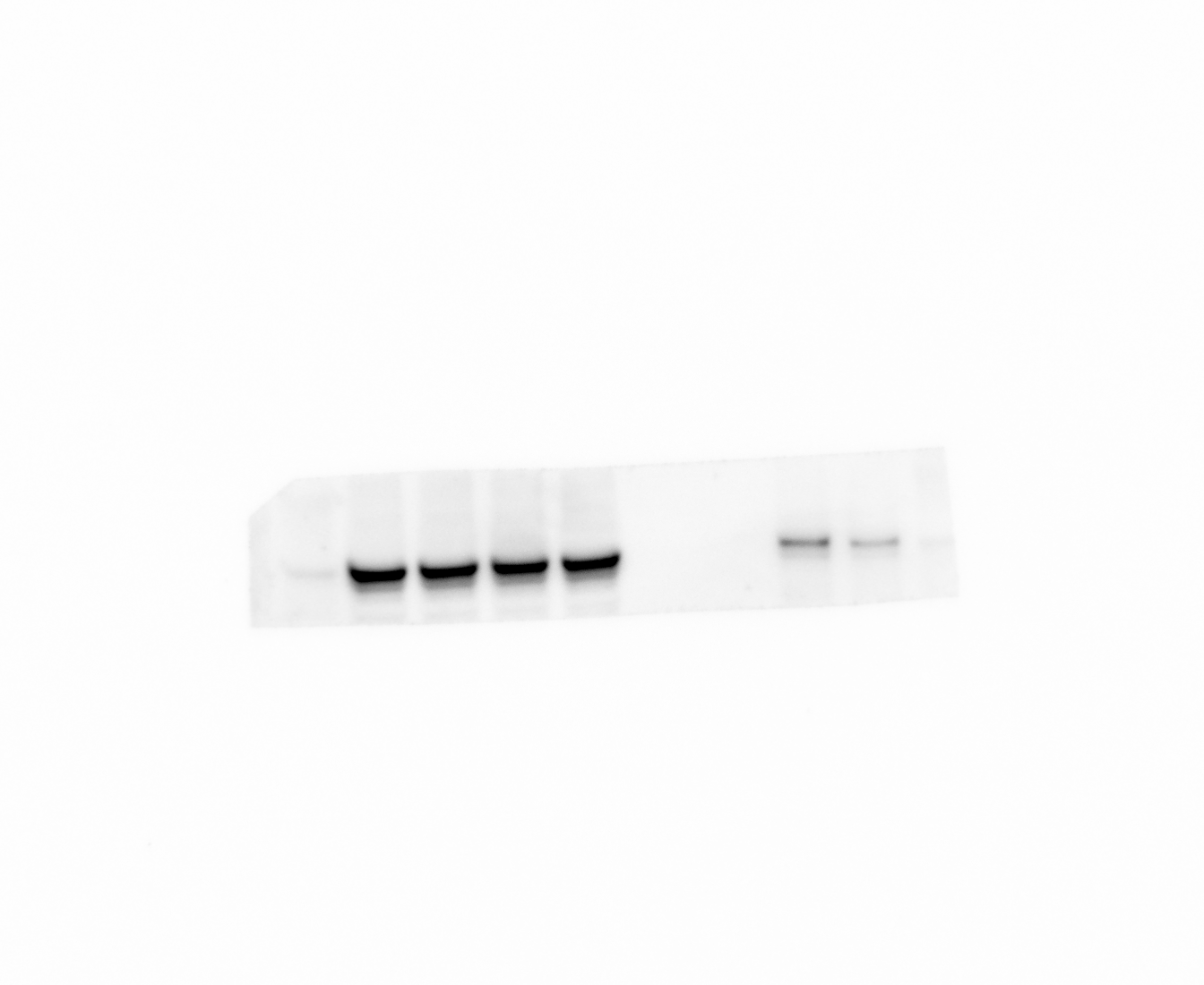

Supplement: Figure 1—source data 1. [file elife-98524-fig1-data1.zip › Fig 1-data1-v1/2B/bottom/SIRT4.tif]

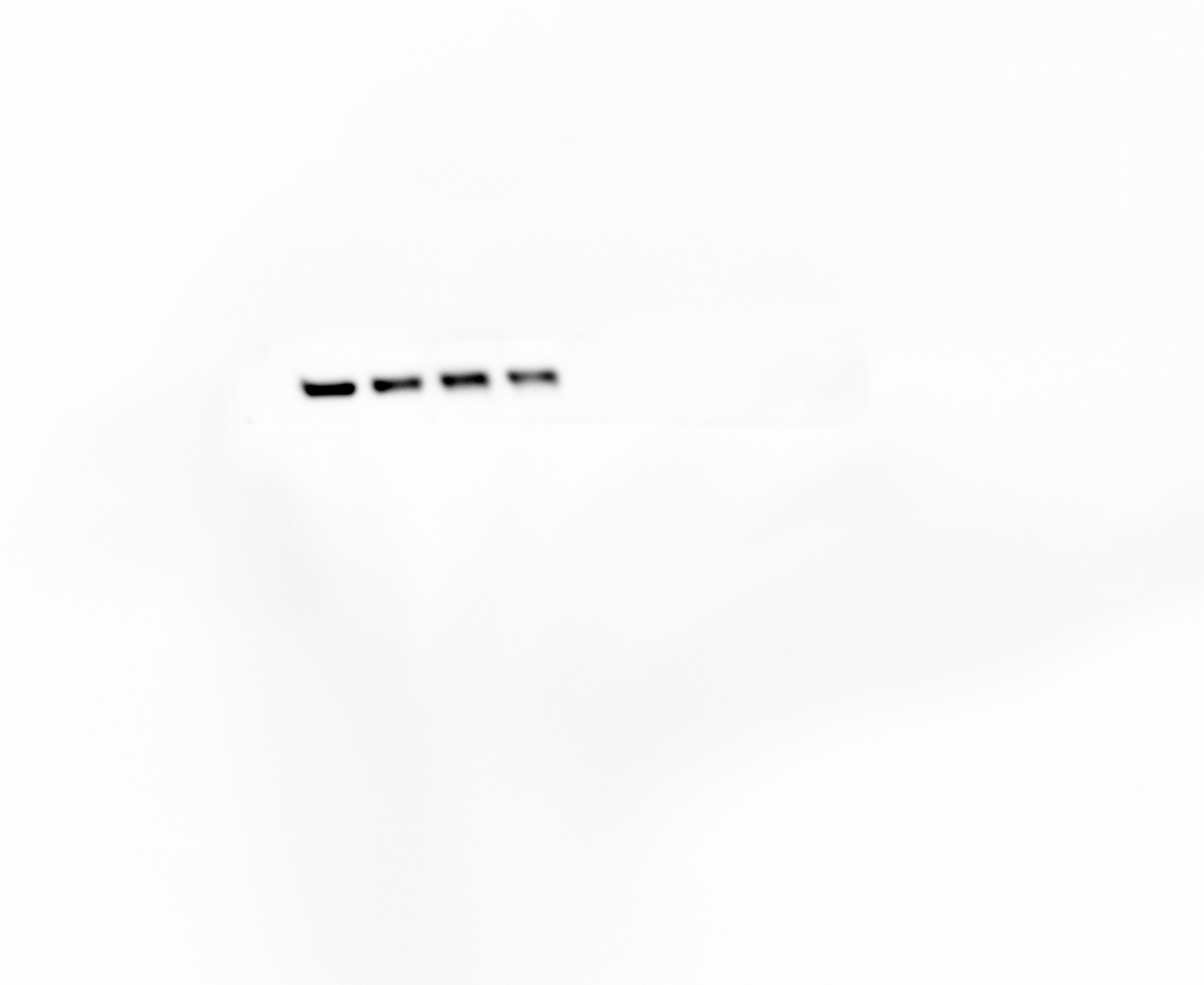

Supplement: Figure 1—source data 1. [file elife-98524-fig1-data1.zip › Fig 1-data1-v1/2B/bottom/Tubulin.tif]

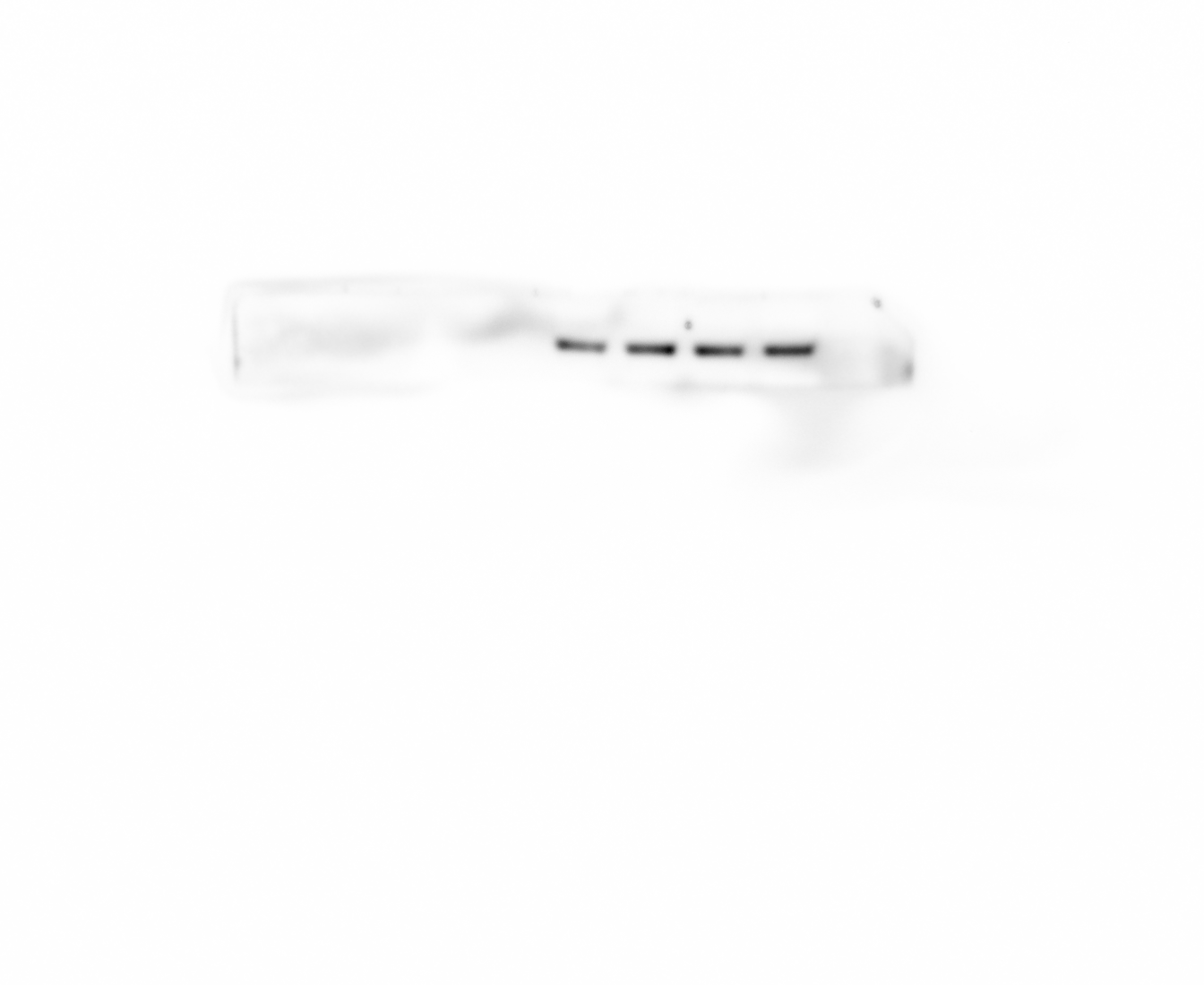

Supplement: Figure 1—source data 1. [file elife-98524-fig1-data1.zip › Fig 1-data1-v1/2B/upper/PCNA upper.tif]

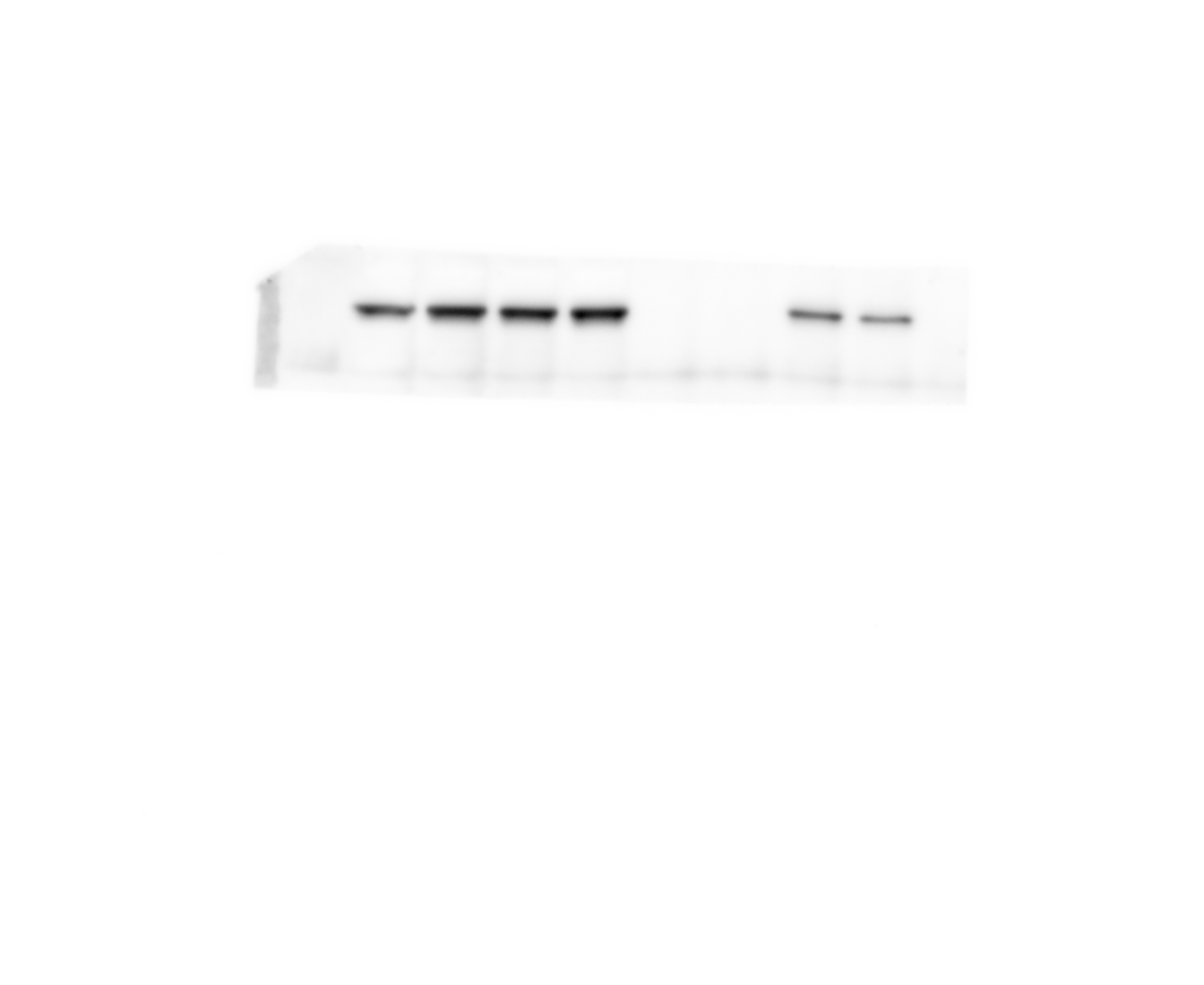

Supplement: Figure 1—source data 1. [file elife-98524-fig1-data1.zip › Fig 1-data1-v1/2B/upper/SIRT4 upper.tif]

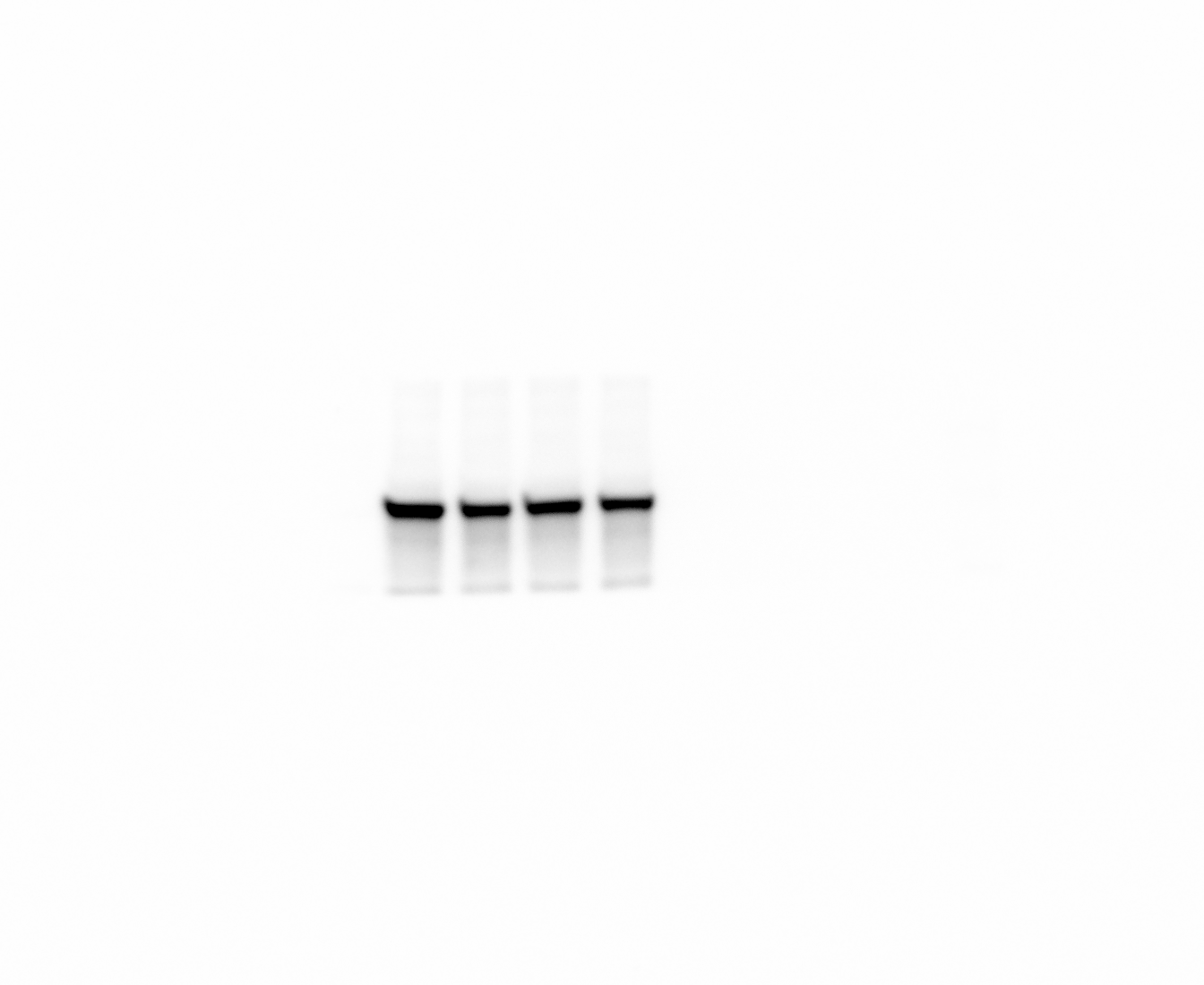

Supplement: Figure 1—source data 1. [file elife-98524-fig1-data1.zip › Fig 1-data1-v1/2B/upper/Tubulin upper.tif]

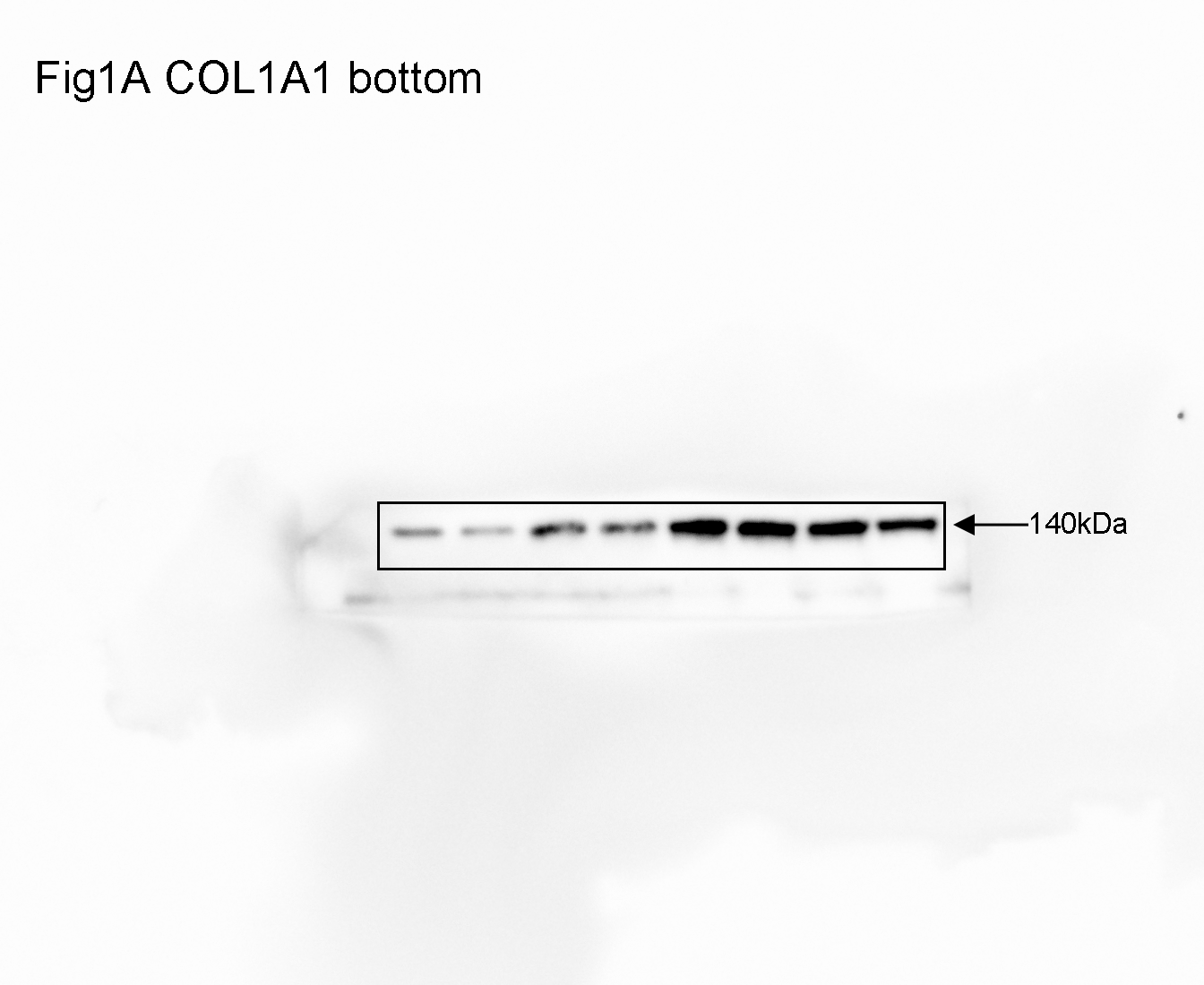

Supplement: Figure 1—source data 2. [file elife-98524-fig1-data2.zip › Fig 1-data2-v1/1A/bottom/COL1A1 bottom.tif]

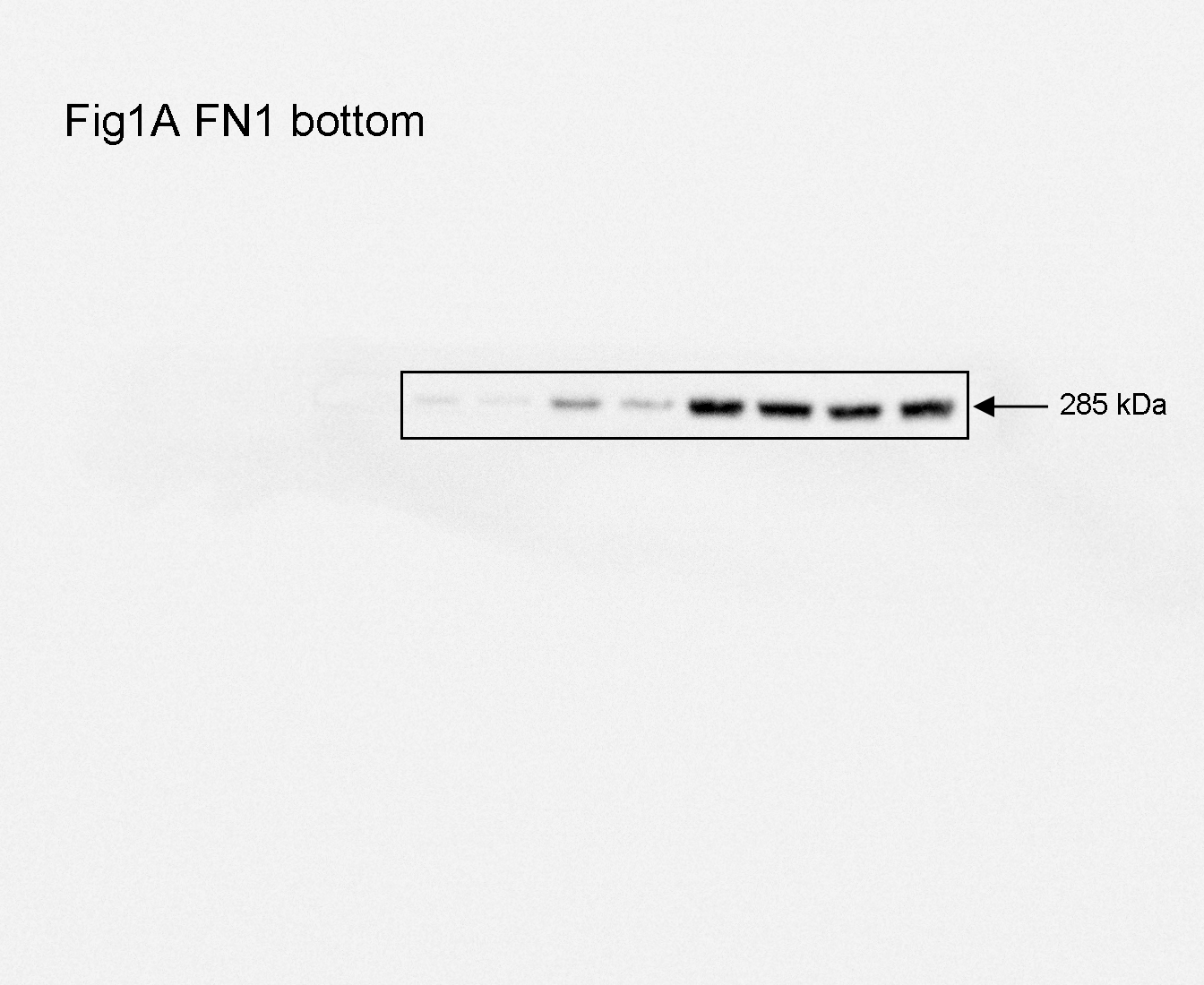

Supplement: Figure 1—source data 2. [file elife-98524-fig1-data2.zip › Fig 1-data2-v1/1A/bottom/FN1 bottom.tif]

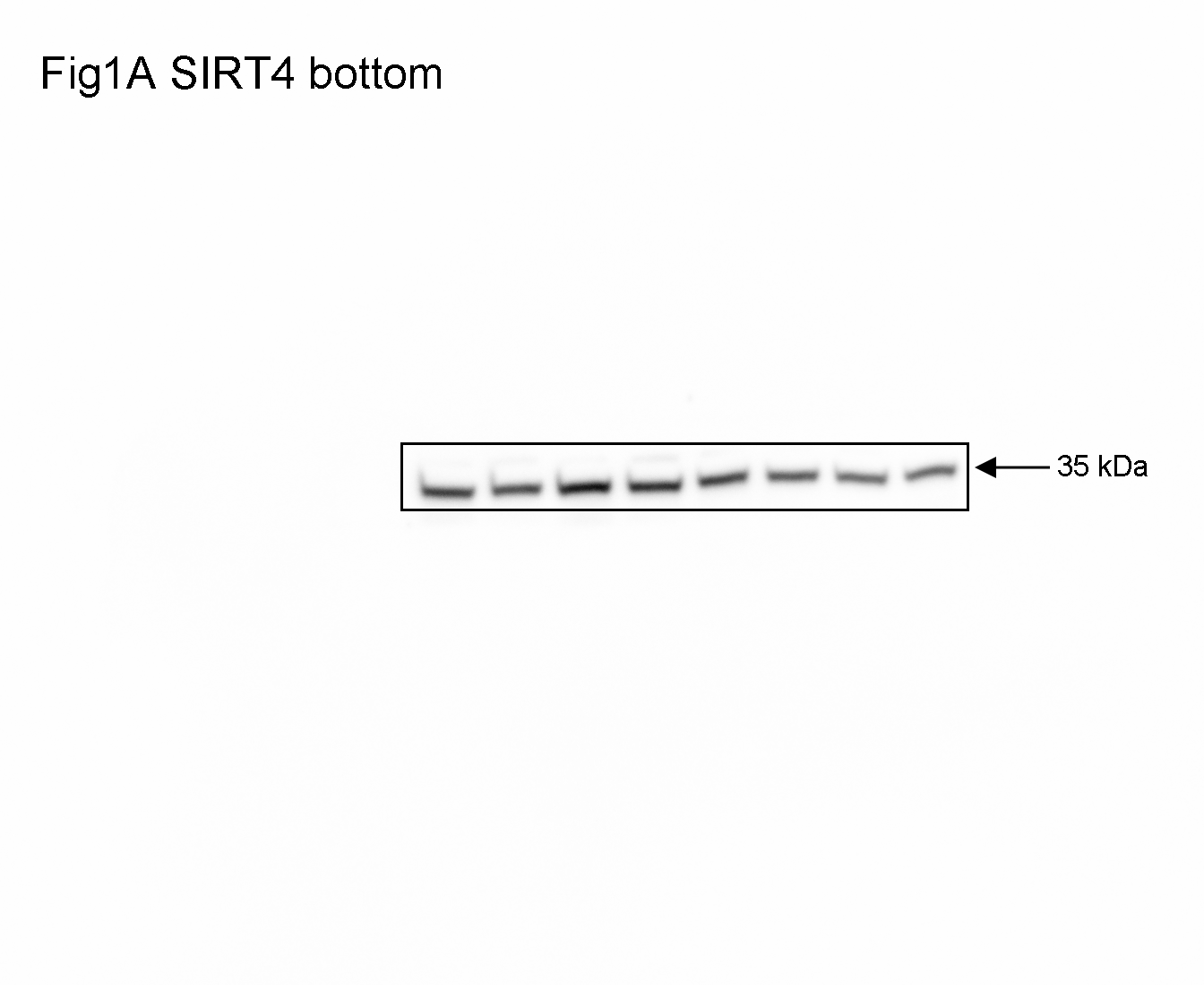

Supplement: Figure 1—source data 2. [file elife-98524-fig1-data2.zip › Fig 1-data2-v1/1A/bottom/SIRT4 bottom.tif]

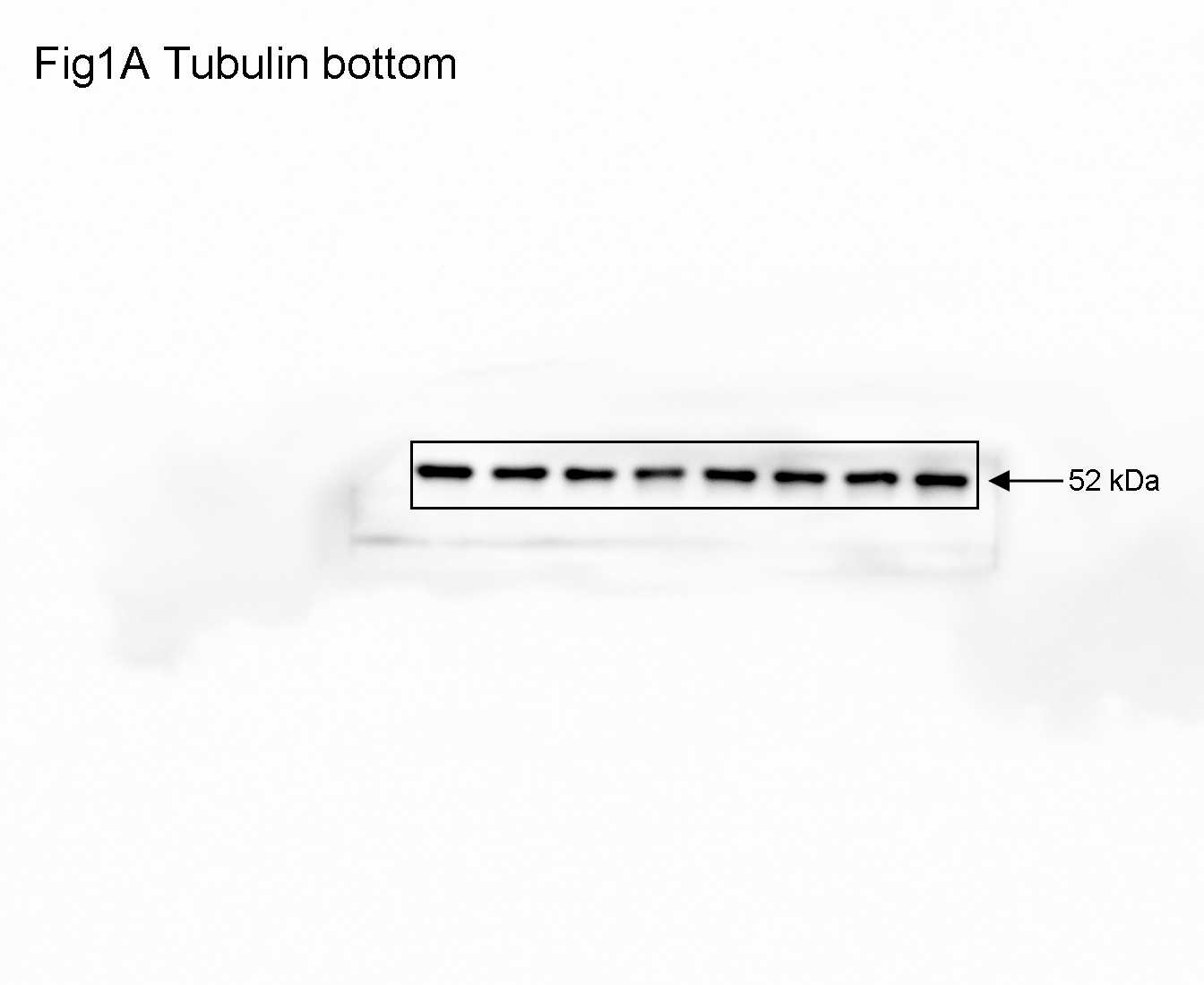

Supplement: Figure 1—source data 2. [file elife-98524-fig1-data2.zip › Fig 1-data2-v1/1A/bottom/Tubulin bottom.tif]

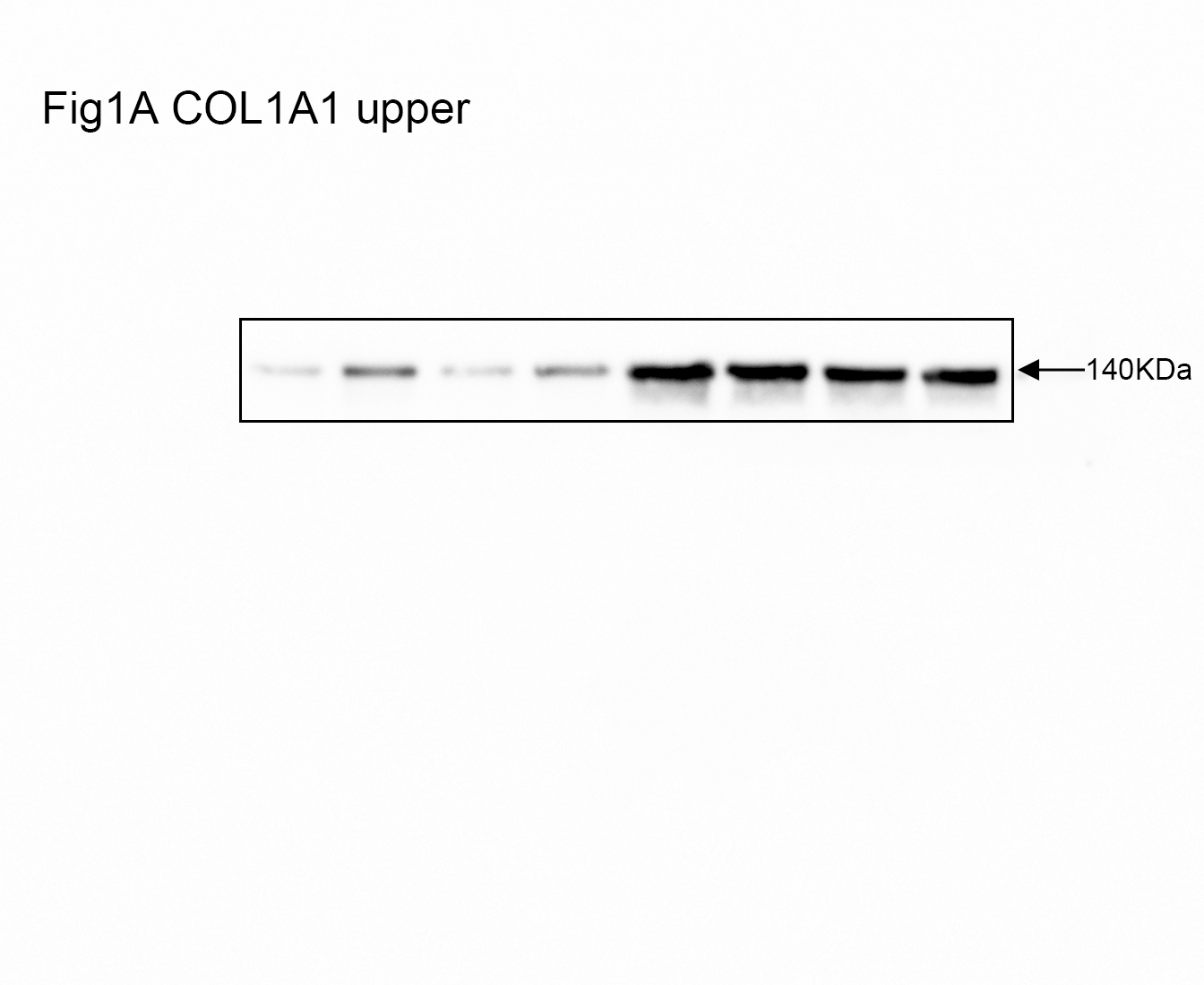

Supplement: Figure 1—source data 2. [file elife-98524-fig1-data2.zip › Fig 1-data2-v1/1A/upper/COL1A1 upper.tif]

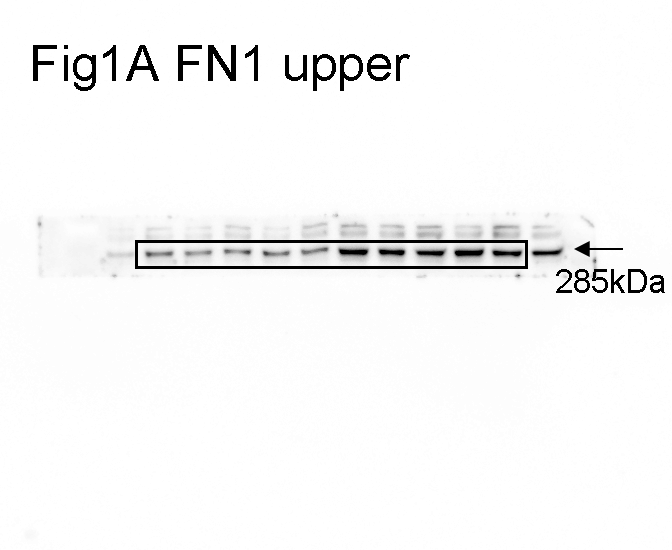

Supplement: Figure 1—source data 2. [file elife-98524-fig1-data2.zip › Fig 1-data2-v1/1A/upper/FN1 upper.tif]

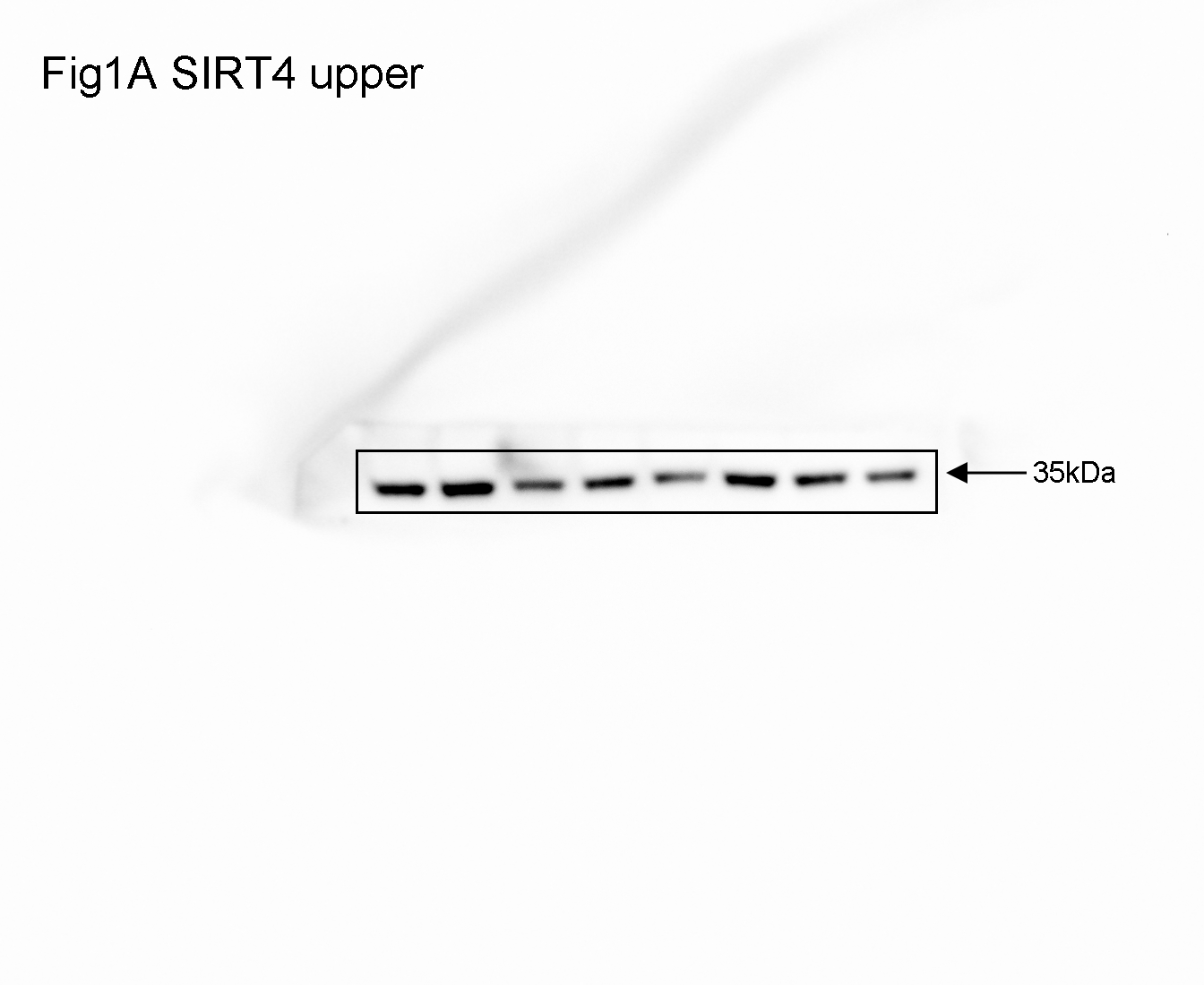

Supplement: Figure 1—source data 2. [file elife-98524-fig1-data2.zip › Fig 1-data2-v1/1A/upper/SIRT4 upper.tif]

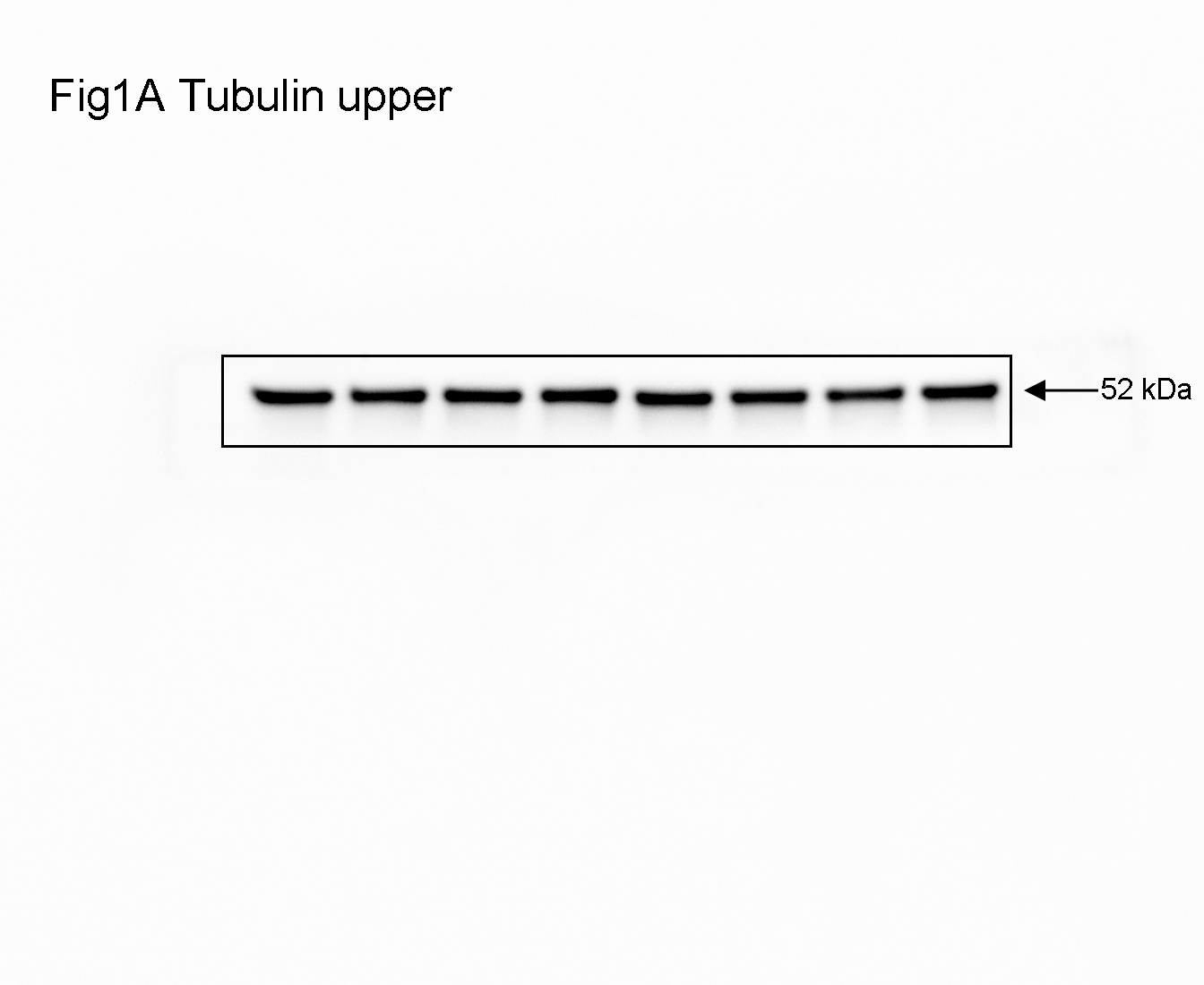

Supplement: Figure 1—source data 2. [file elife-98524-fig1-data2.zip › Fig 1-data2-v1/1A/upper/Tubulin upper.tif]

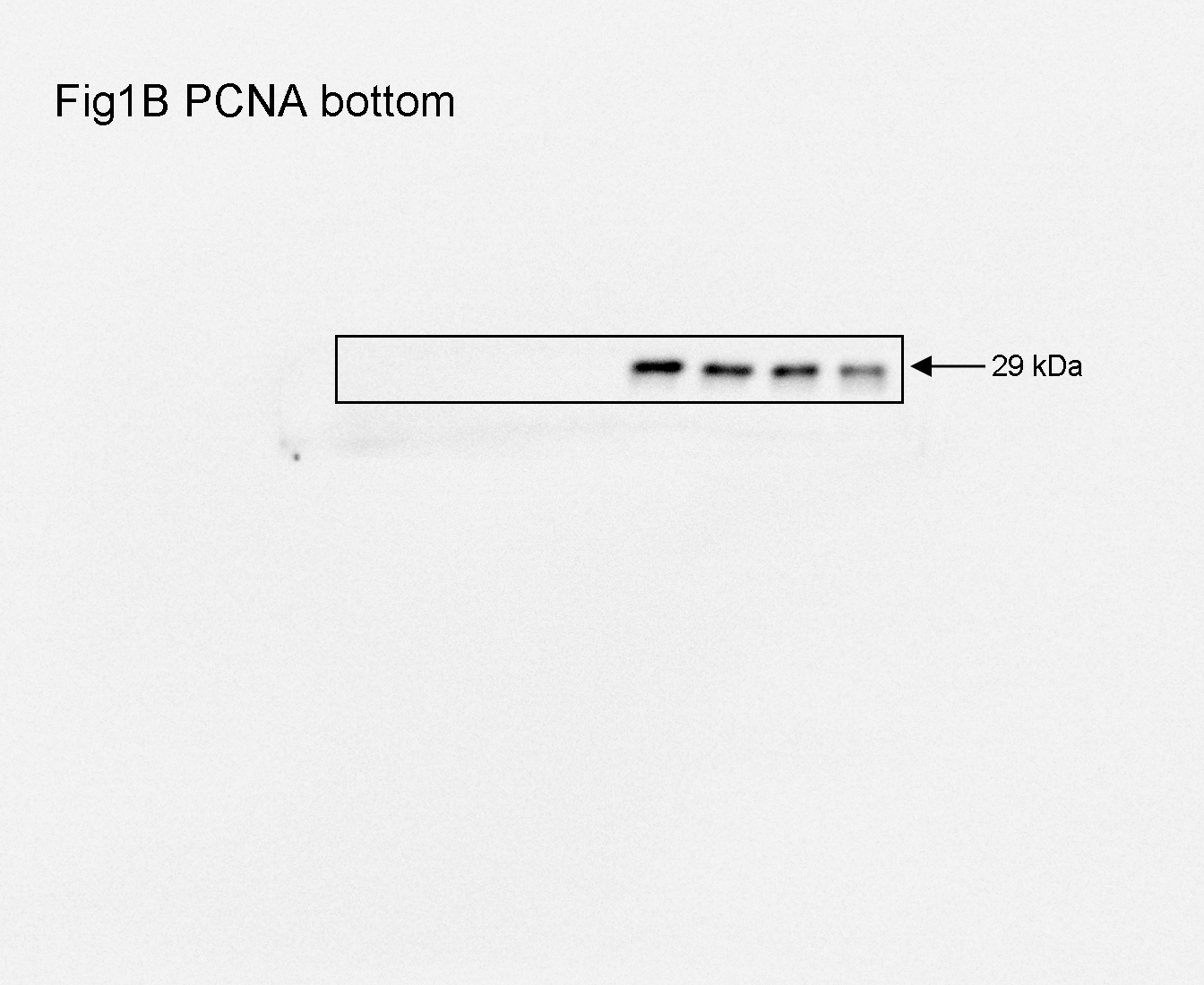

Supplement: Figure 1—source data 2. [file elife-98524-fig1-data2.zip › Fig 1-data2-v1/1B/bottom/PCNA.tif]

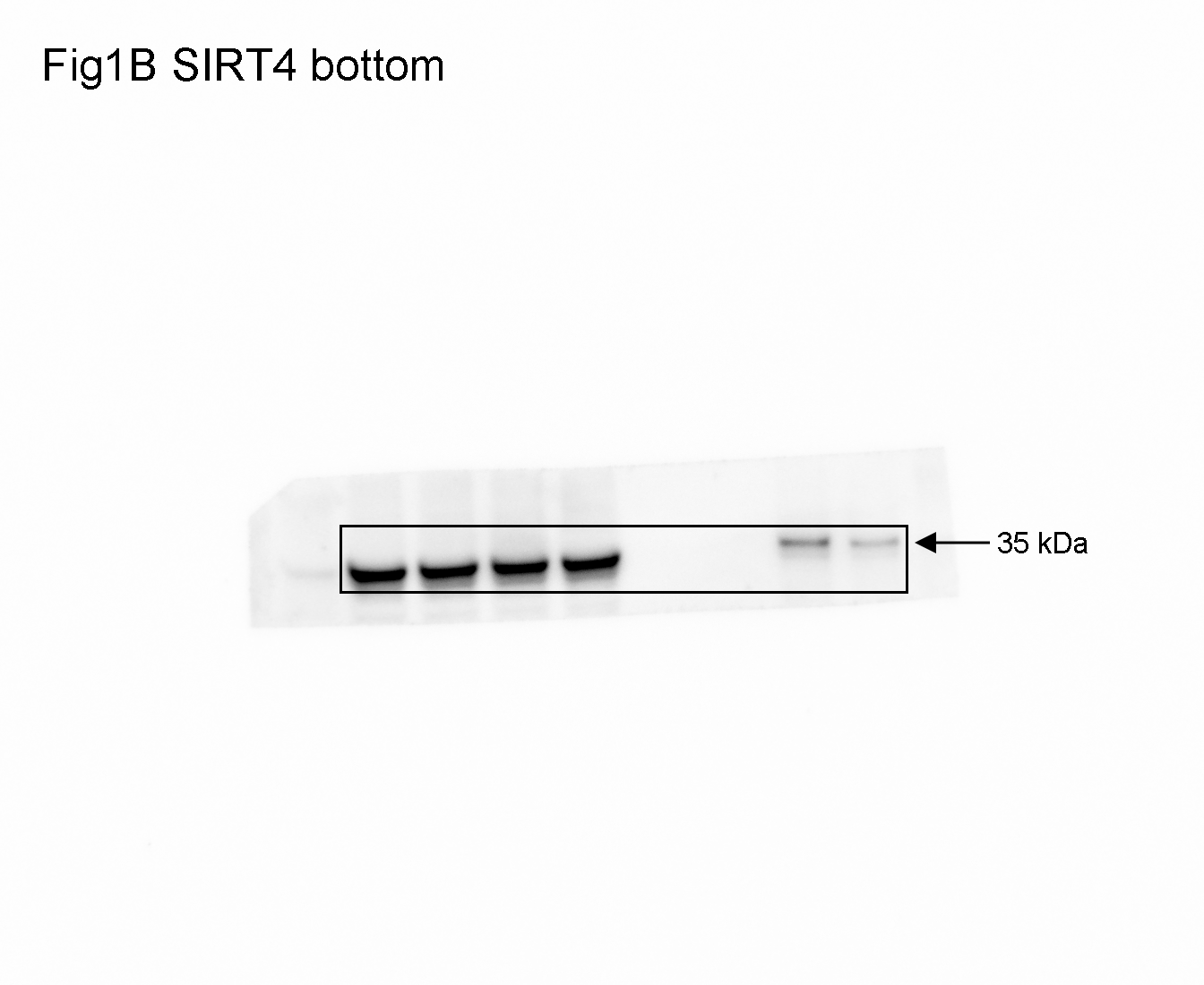

Supplement: Figure 1—source data 2. [file elife-98524-fig1-data2.zip › Fig 1-data2-v1/1B/bottom/SIRT4.tif]

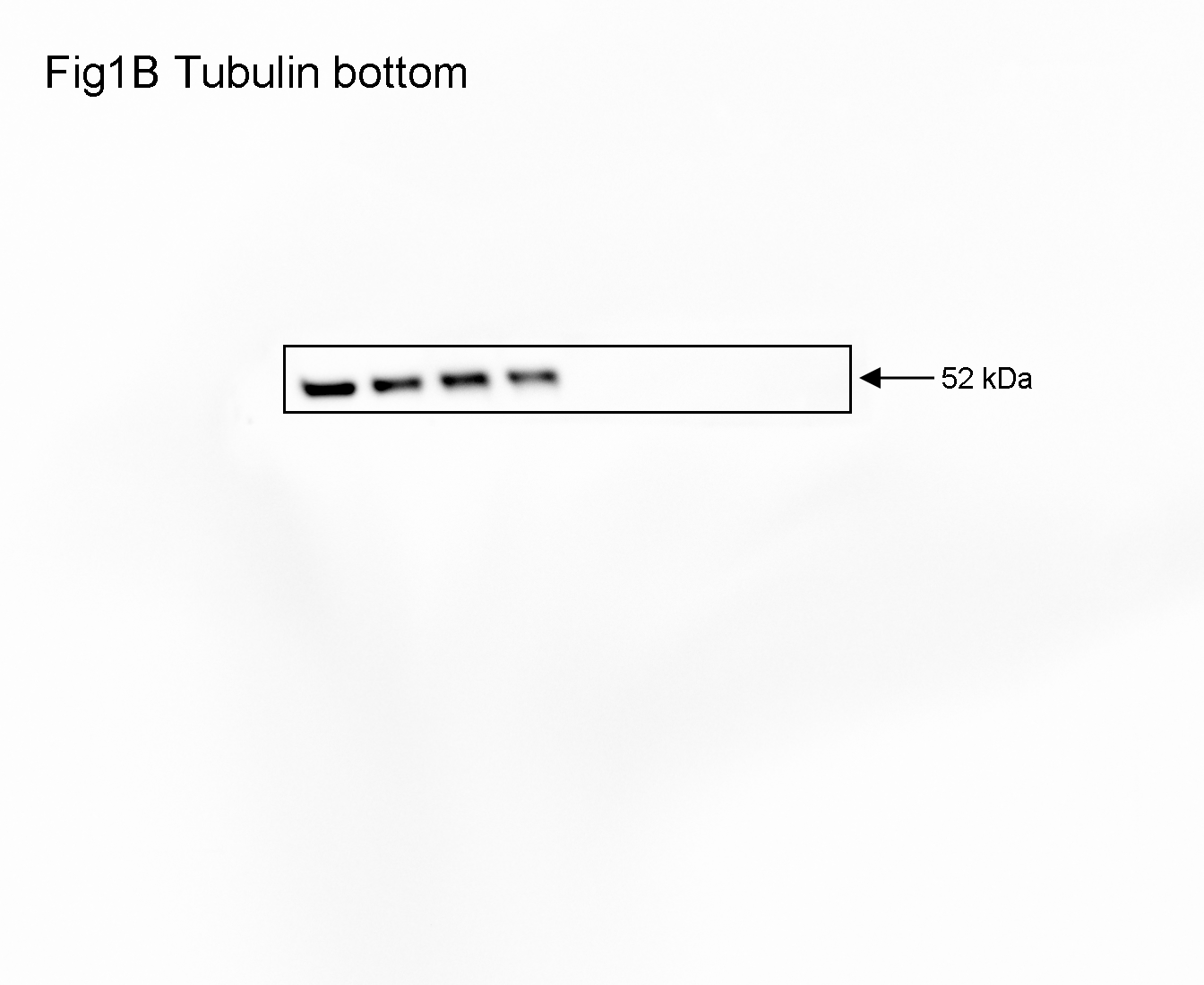

Supplement: Figure 1—source data 2. [file elife-98524-fig1-data2.zip › Fig 1-data2-v1/1B/bottom/Tubulin.tif]

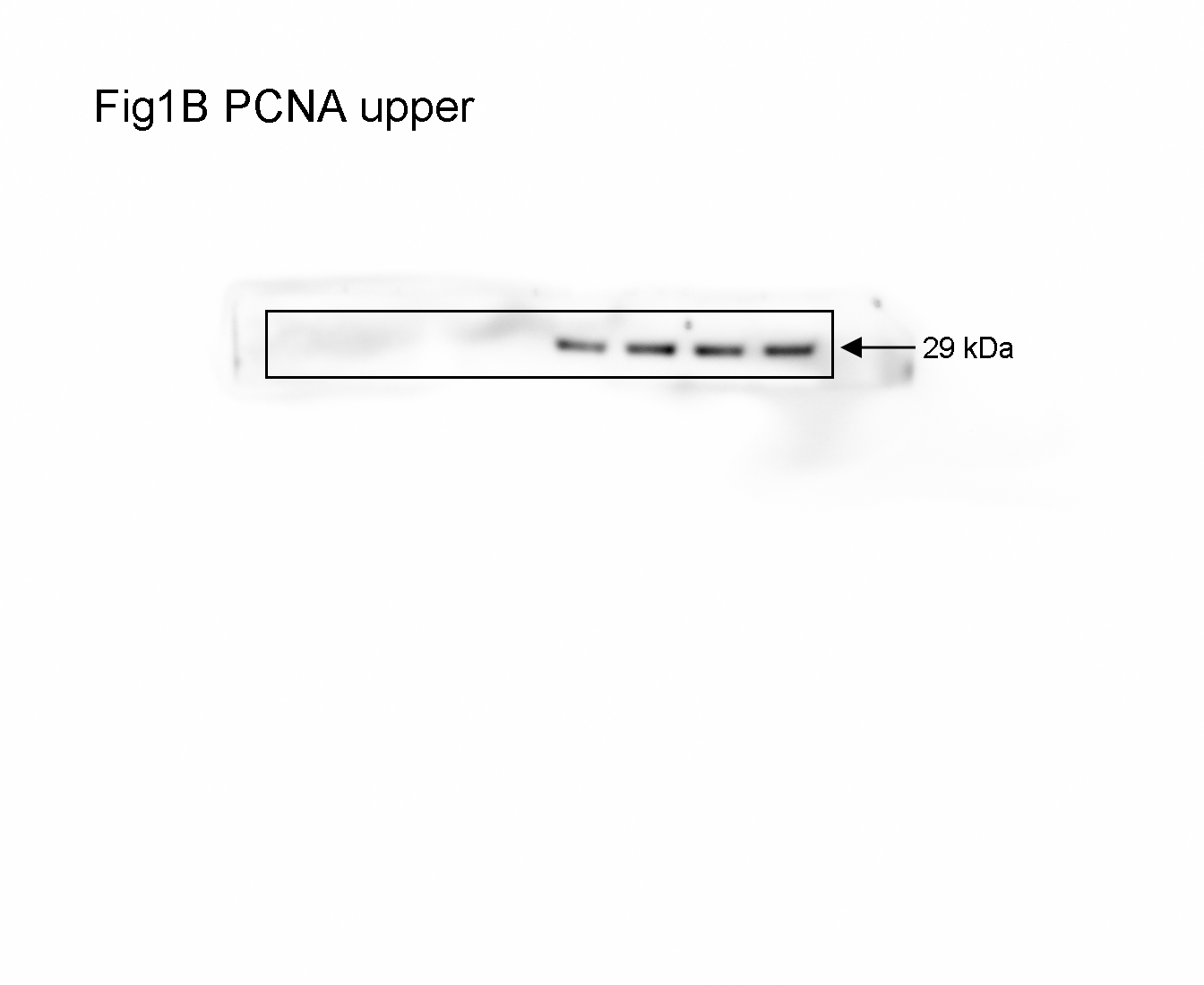

Supplement: Figure 1—source data 2. [file elife-98524-fig1-data2.zip › Fig 1-data2-v1/1B/upper/PCNA.tif]

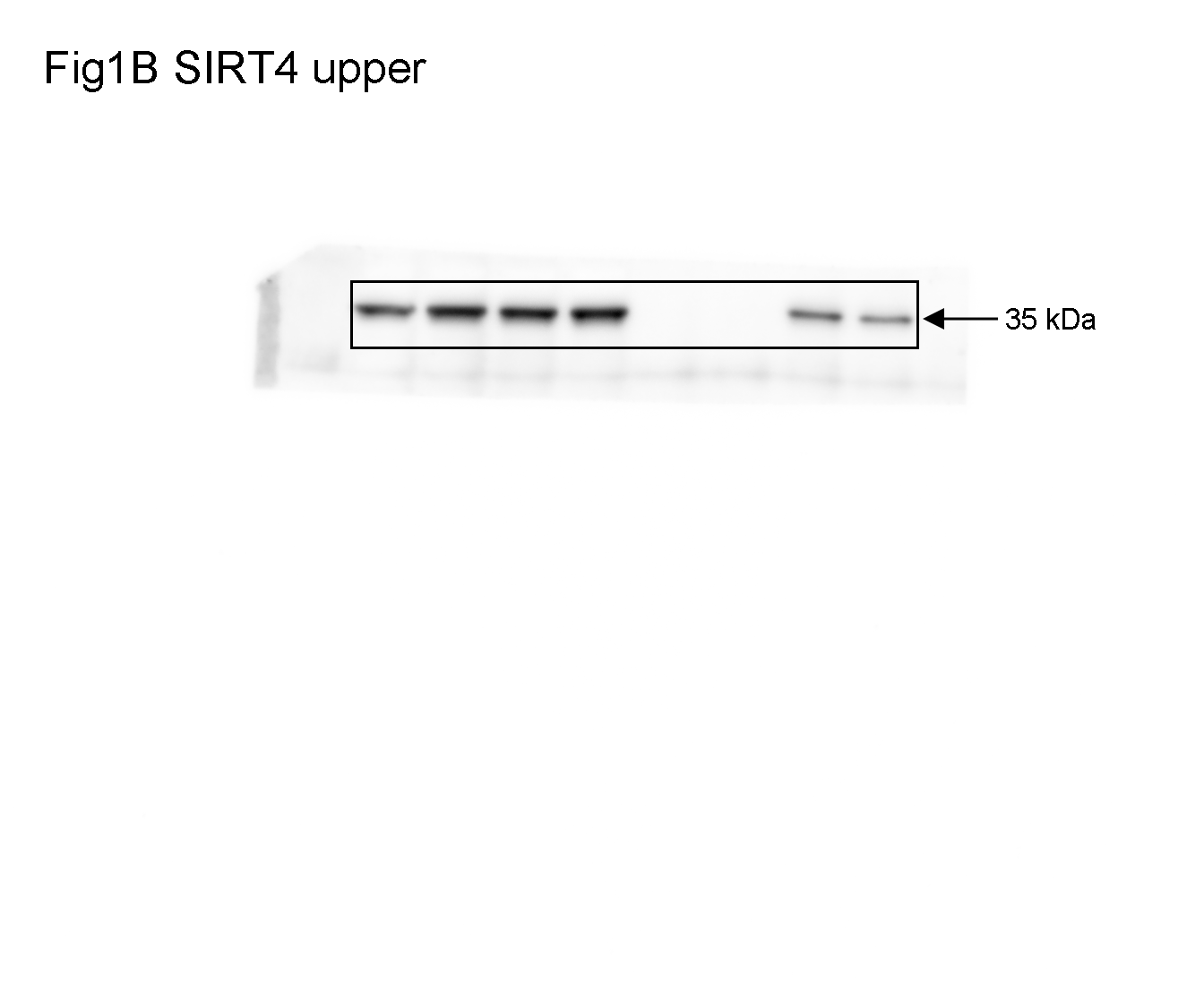

Supplement: Figure 1—source data 2. [file elife-98524-fig1-data2.zip › Fig 1-data2-v1/1B/upper/SIRT4.tif]

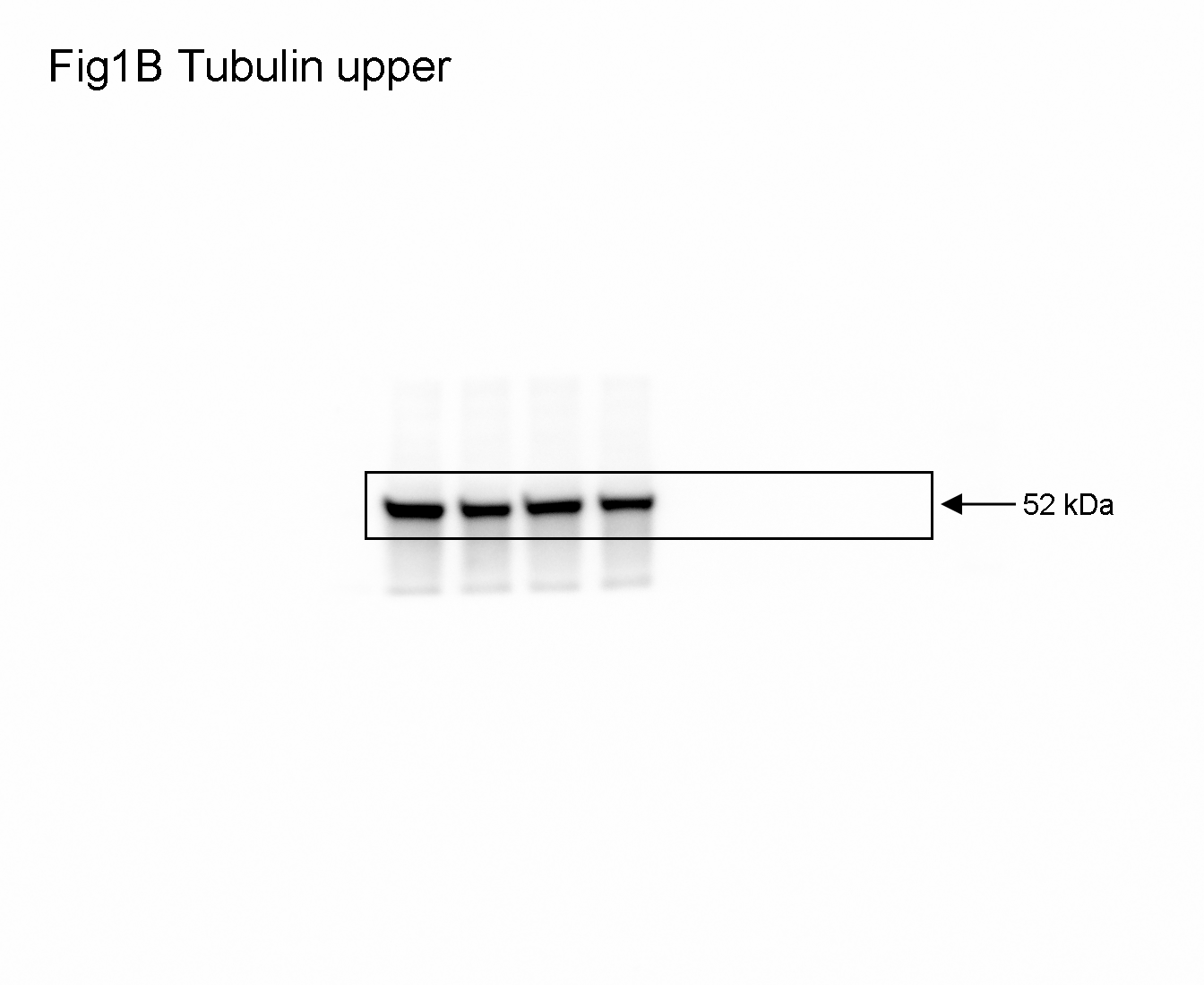

Supplement: Figure 1—source data 2. [file elife-98524-fig1-data2.zip › Fig 1-data2-v1/1B/upper/Tubulin.tif]

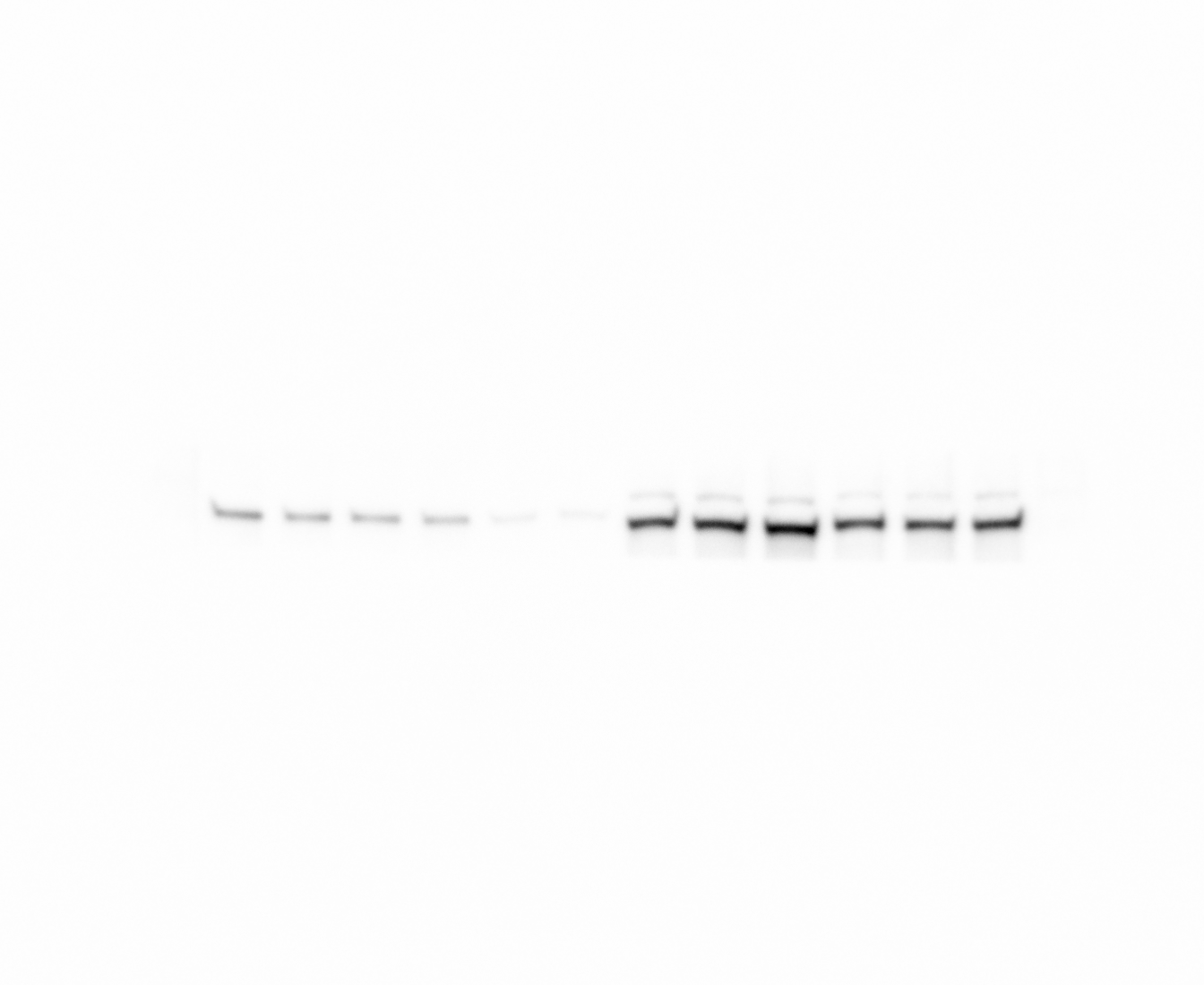

Supplement: Figure 2—source data 1. [file elife-98524-fig2-data1.zip › Fig 2-data1-v1/2B/CCN2.tif]

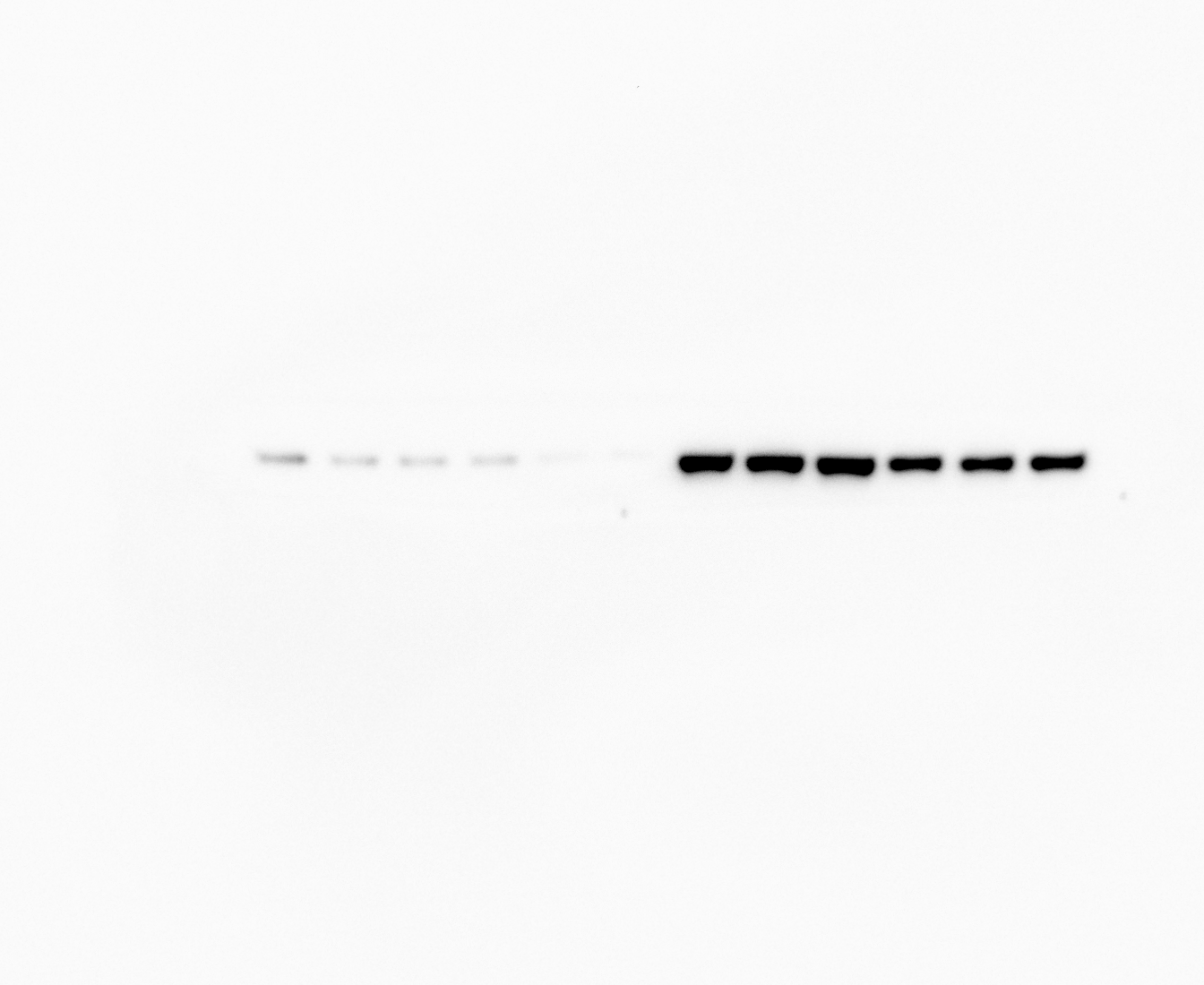

Supplement: Figure 2—source data 1. [file elife-98524-fig2-data1.zip › Fig 2-data1-v1/2B/COL1A1.tif]

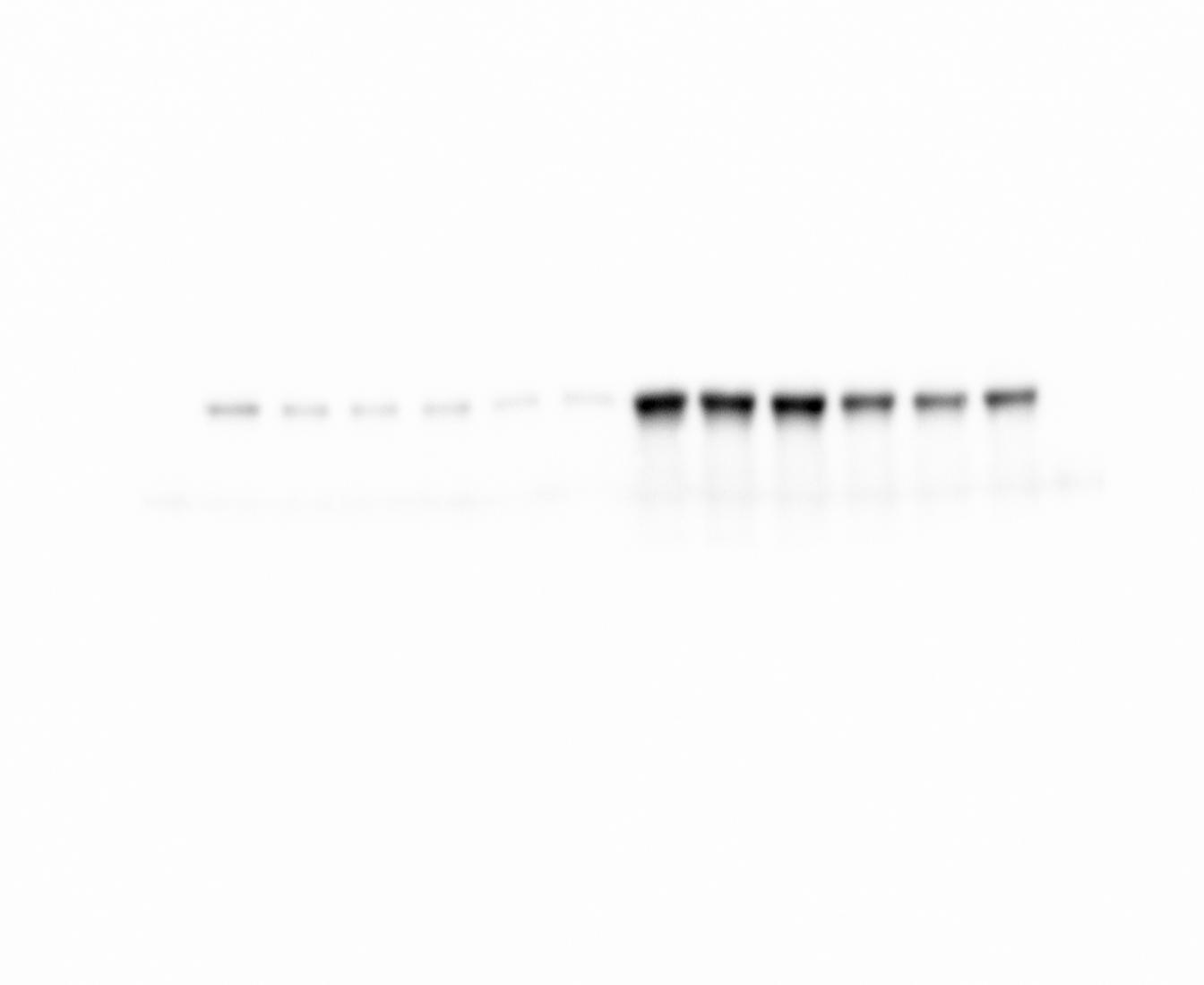

Supplement: Figure 2—source data 1. [file elife-98524-fig2-data1.zip › Fig 2-data1-v1/2B/COL3A1.tif]

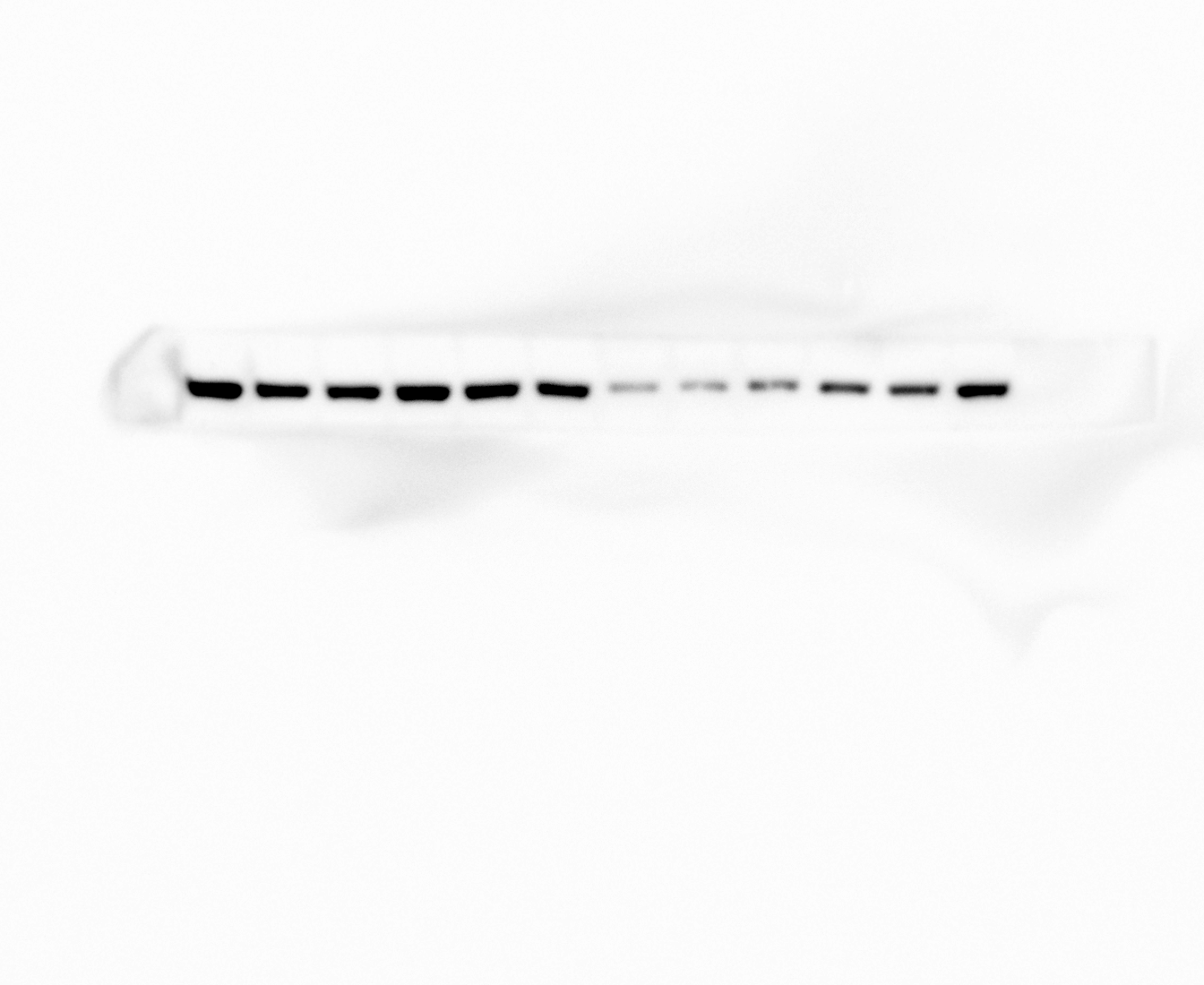

Supplement: Figure 2—source data 1. [file elife-98524-fig2-data1.zip › Fig 2-data1-v1/2B/E-cadherin.tif]

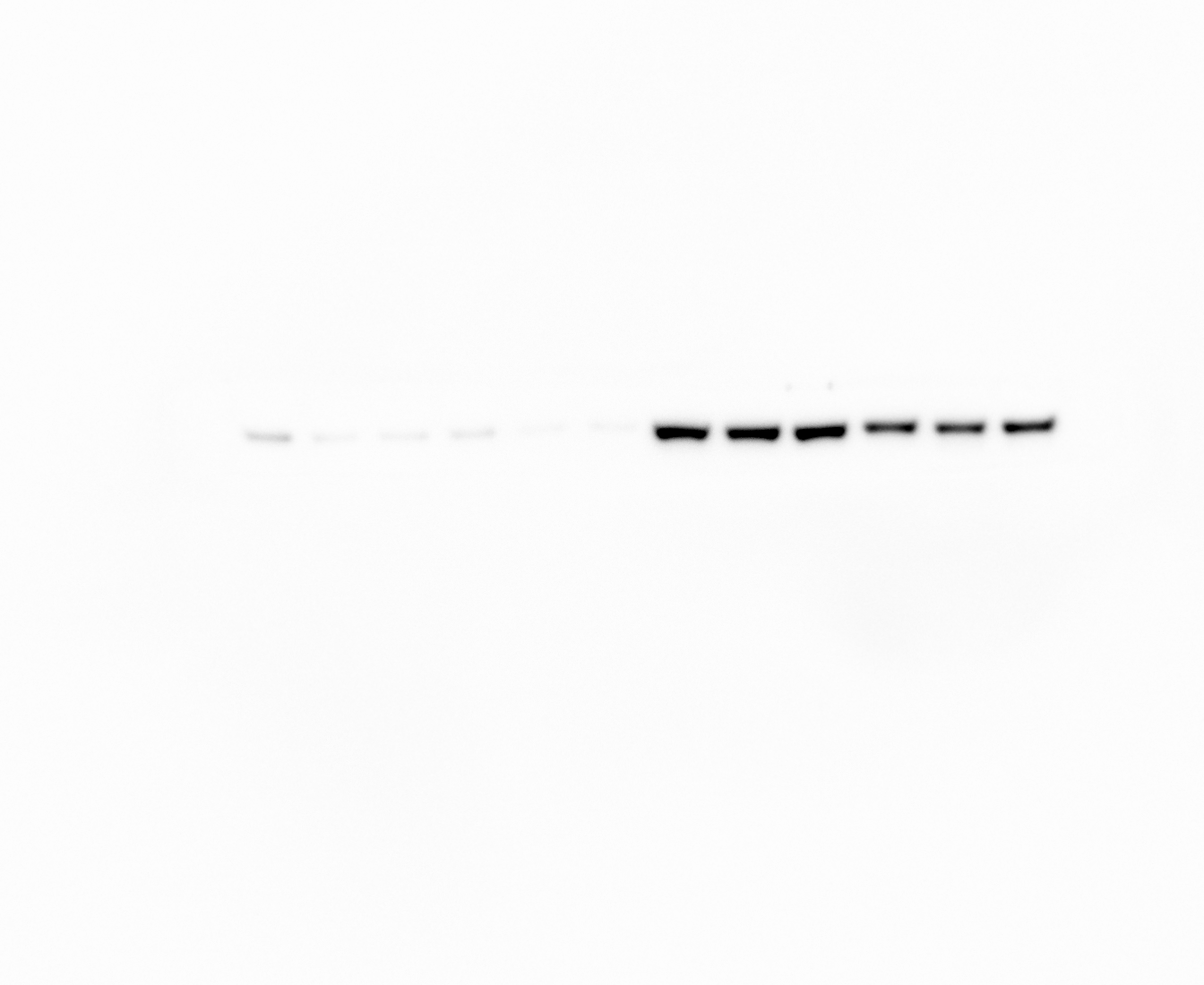

Supplement: Figure 2—source data 1. [file elife-98524-fig2-data1.zip › Fig 2-data1-v1/2B/FN1.tif]

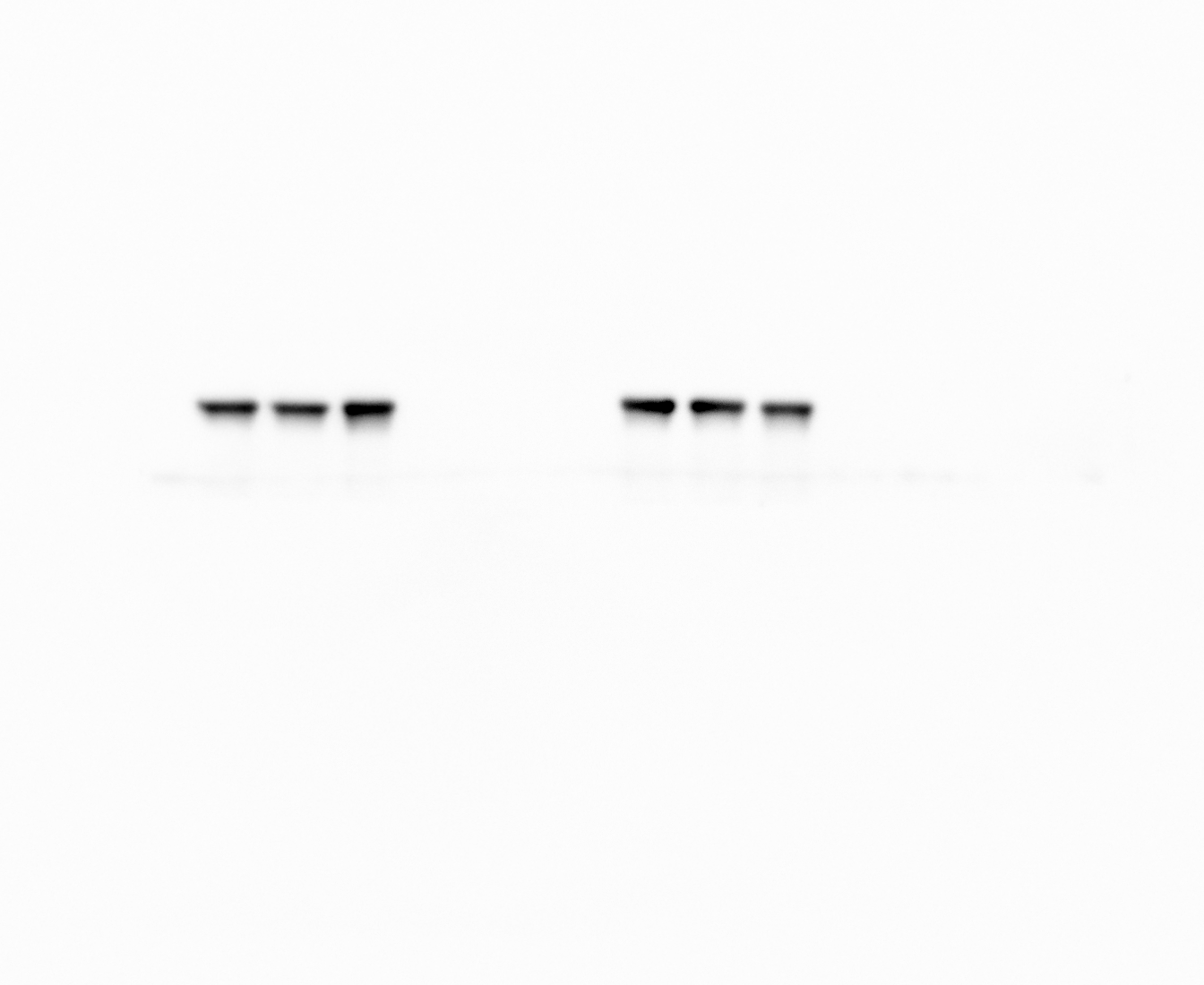

Supplement: Figure 2—source data 1. [file elife-98524-fig2-data1.zip › Fig 2-data1-v1/2B/SIRT4.tif]

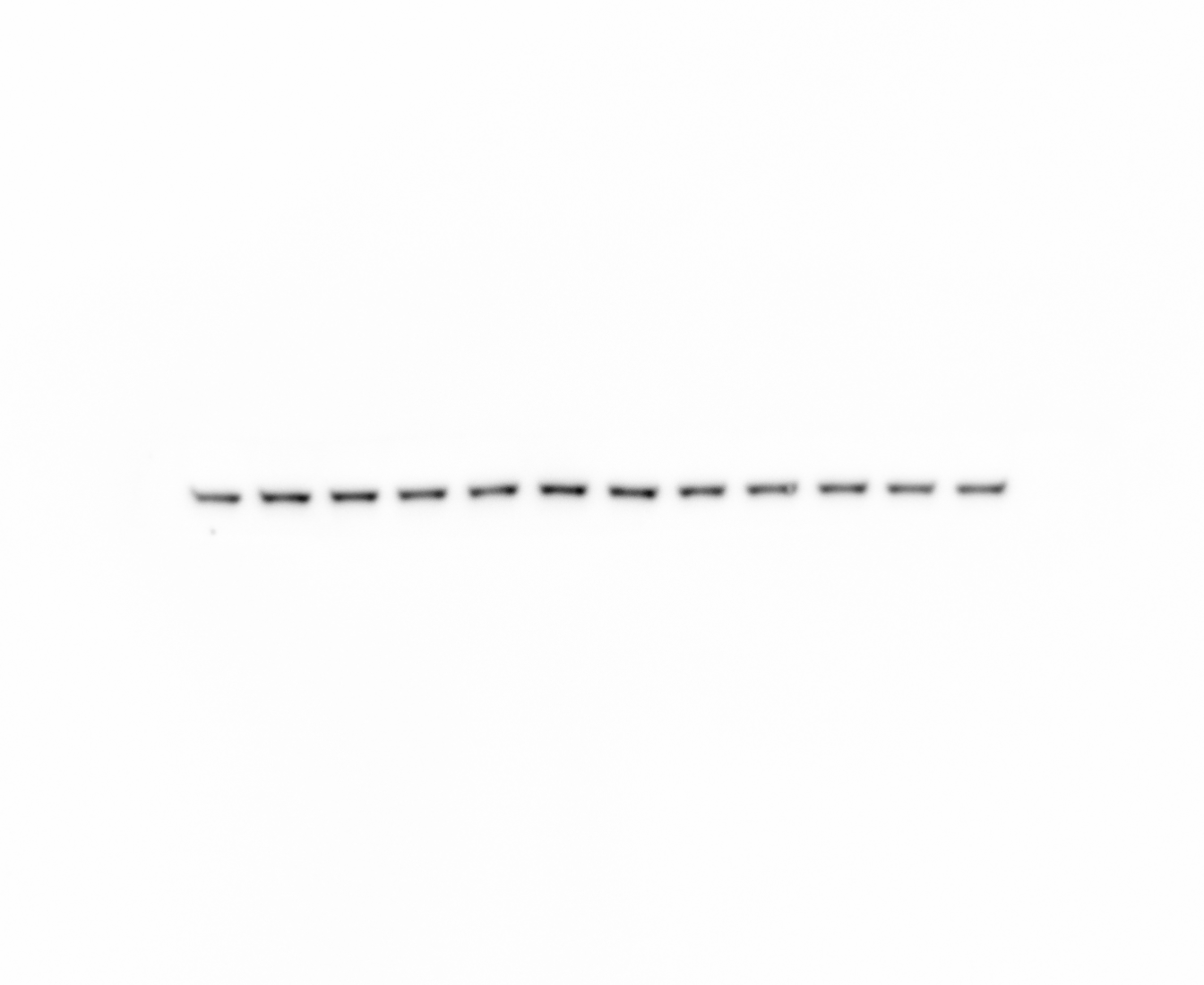

Supplement: Figure 2—source data 1. [file elife-98524-fig2-data1.zip › Fig 2-data1-v1/2B/Tubulin.tif]

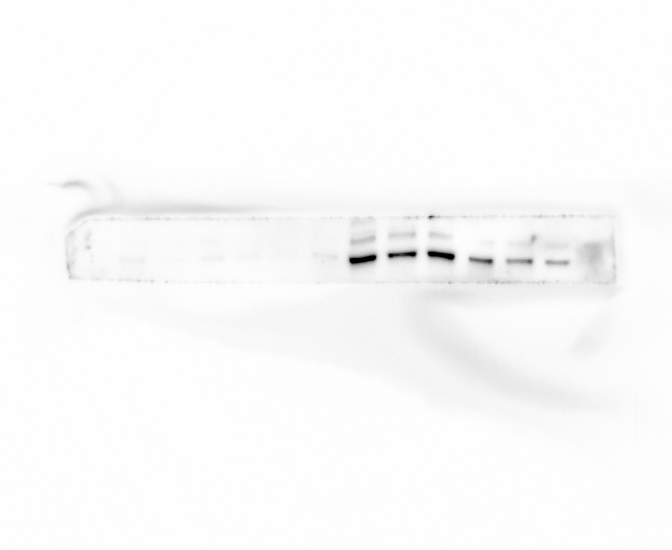

Supplement: Figure 2—source data 1. [file elife-98524-fig2-data1.zip › Fig 2-data1-v1/2B/α-SMA.tif]

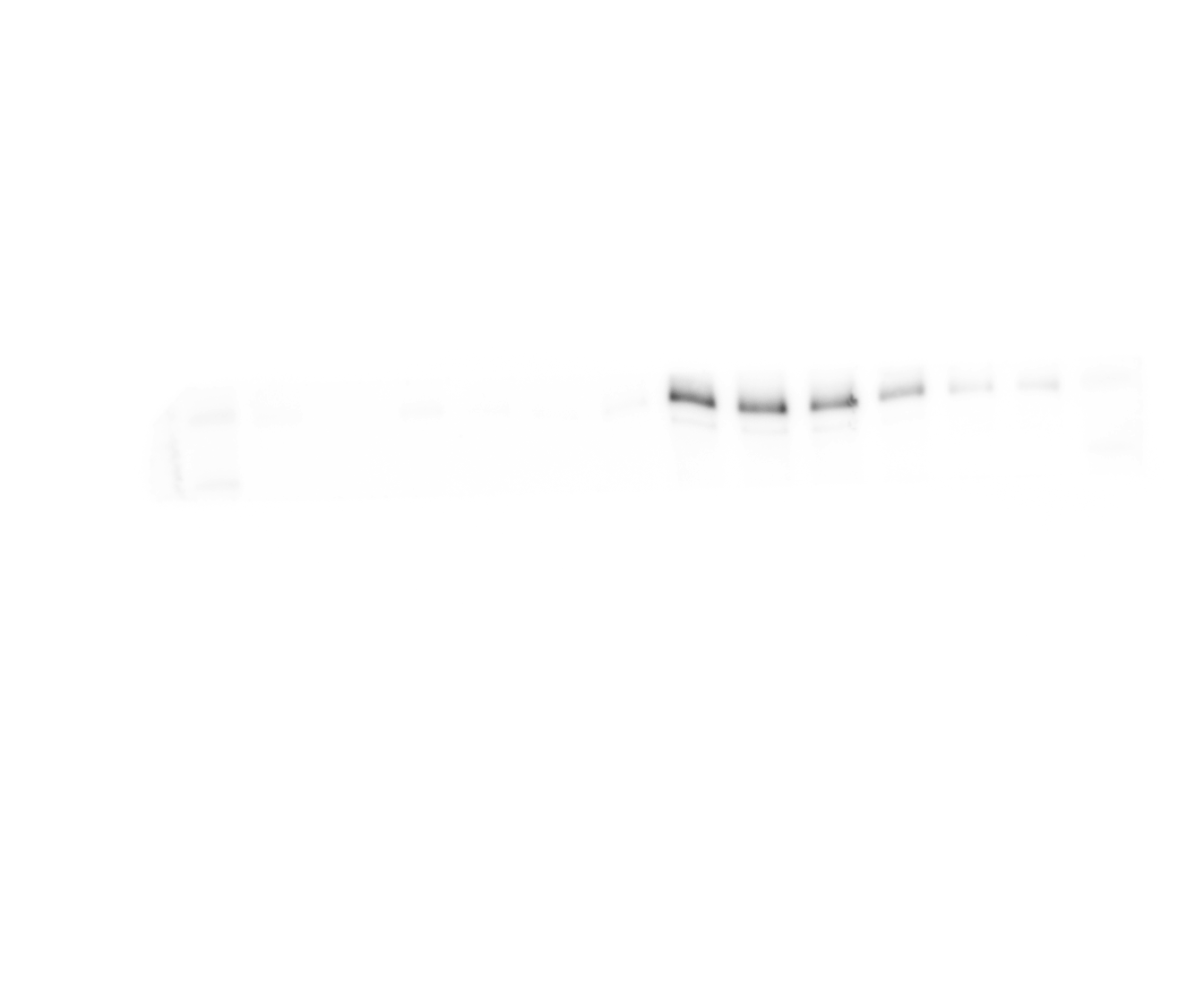

Supplement: Figure 2—source data 1. [file elife-98524-fig2-data1.zip › Fig 2-data1-v1/2F/CCN2.tif]

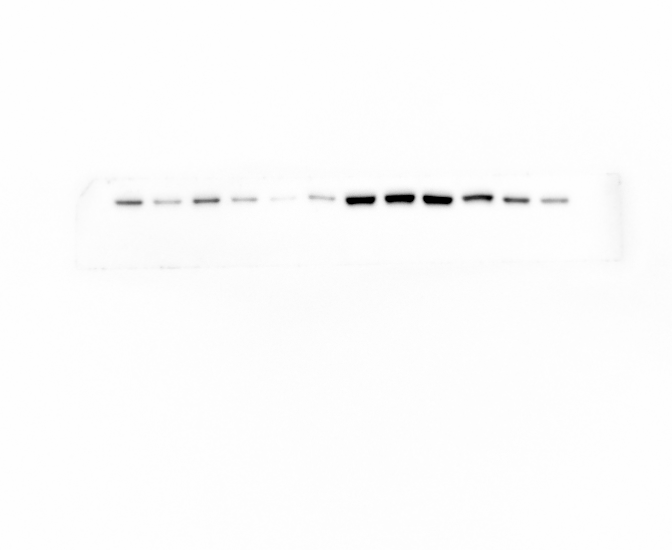

Supplement: Figure 2—source data 1. [file elife-98524-fig2-data1.zip › Fig 2-data1-v1/2F/COL1A1.tif]

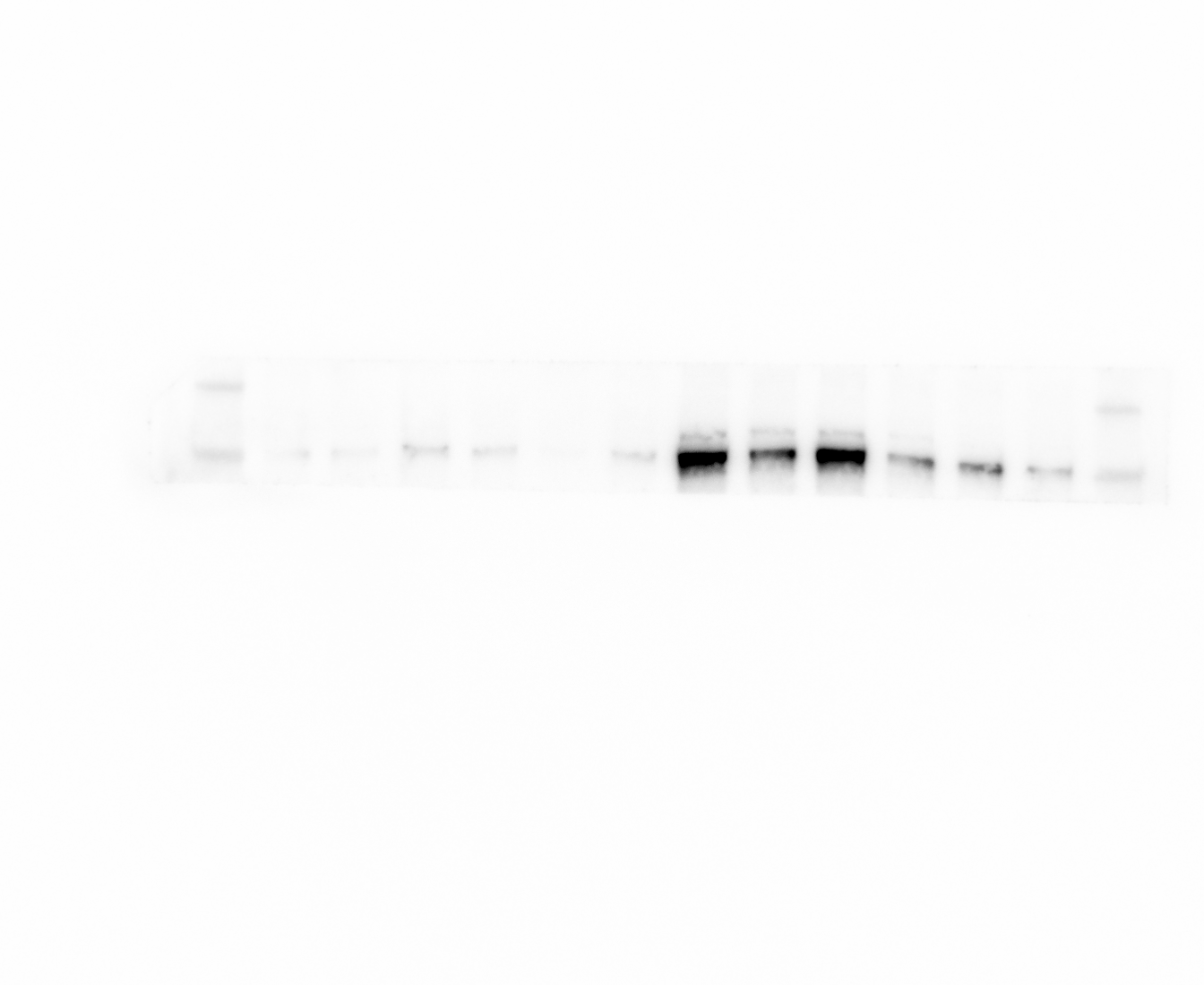

Supplement: Figure 2—source data 1. [file elife-98524-fig2-data1.zip › Fig 2-data1-v1/2F/COL3A1.tif]

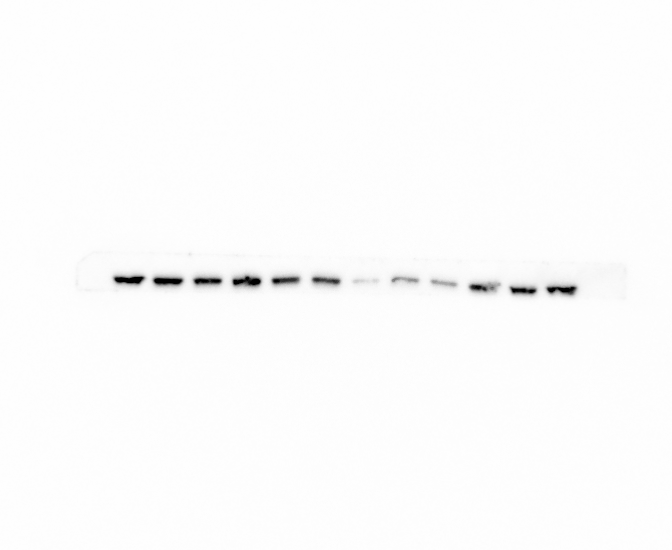

Supplement: Figure 2—source data 1. [file elife-98524-fig2-data1.zip › Fig 2-data1-v1/2F/E-cadherin.tif]

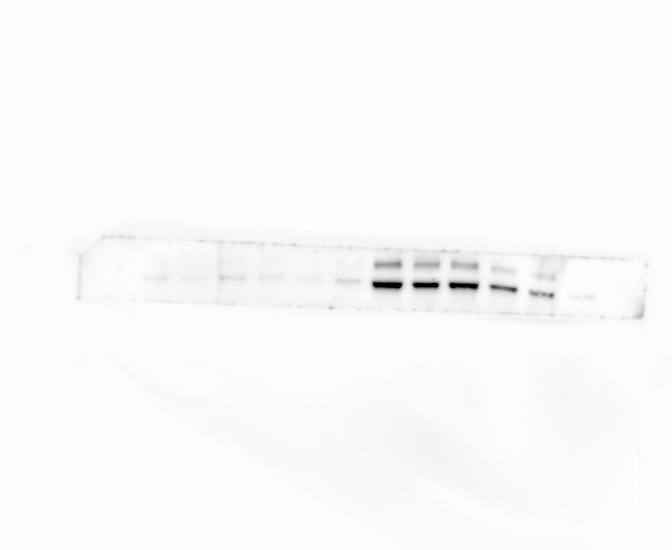

Supplement: Figure 2—source data 1. [file elife-98524-fig2-data1.zip › Fig 2-data1-v1/2F/FN1.tif]

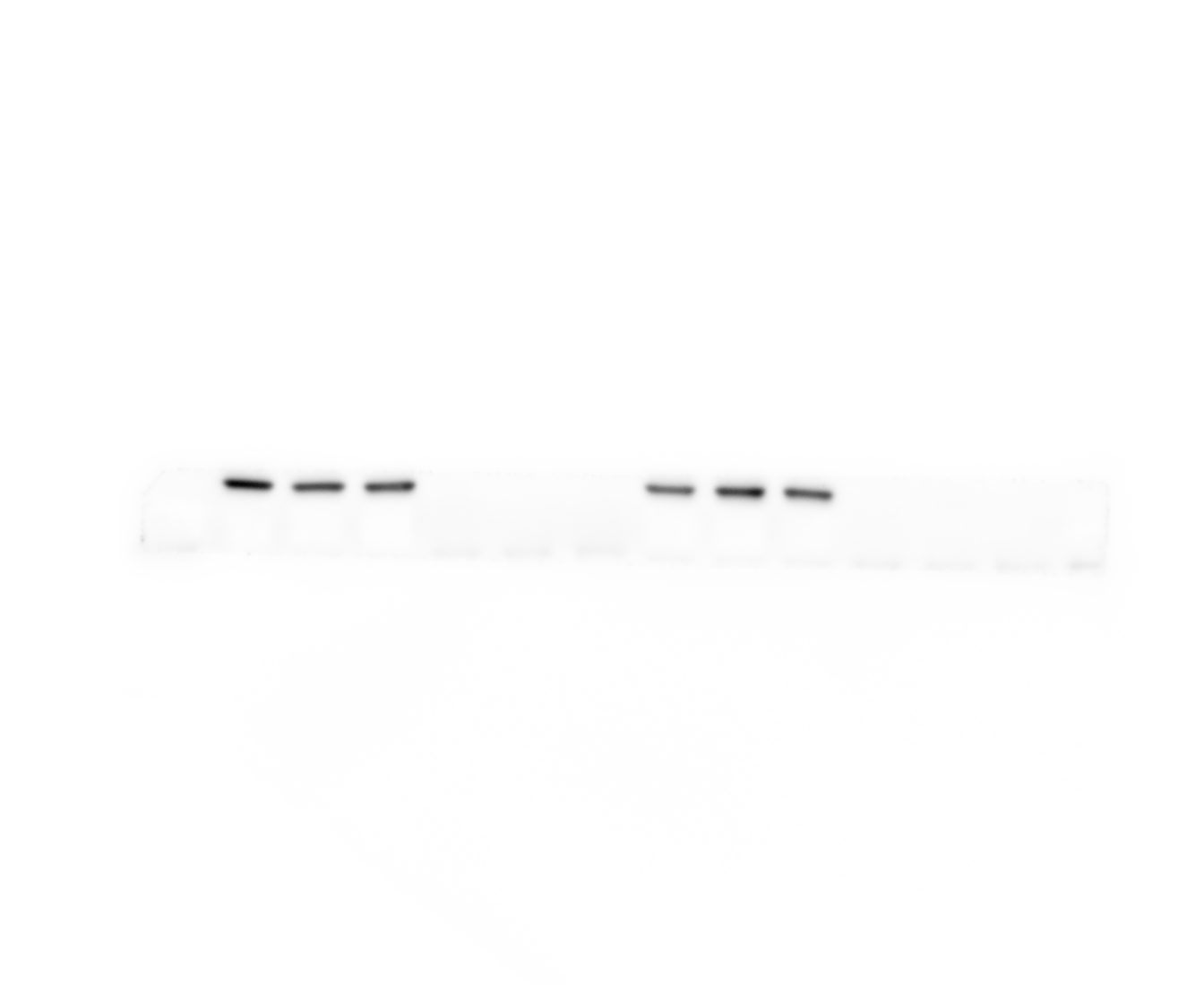

Supplement: Figure 2—source data 1. [file elife-98524-fig2-data1.zip › Fig 2-data1-v1/2F/SIRT4.tif]

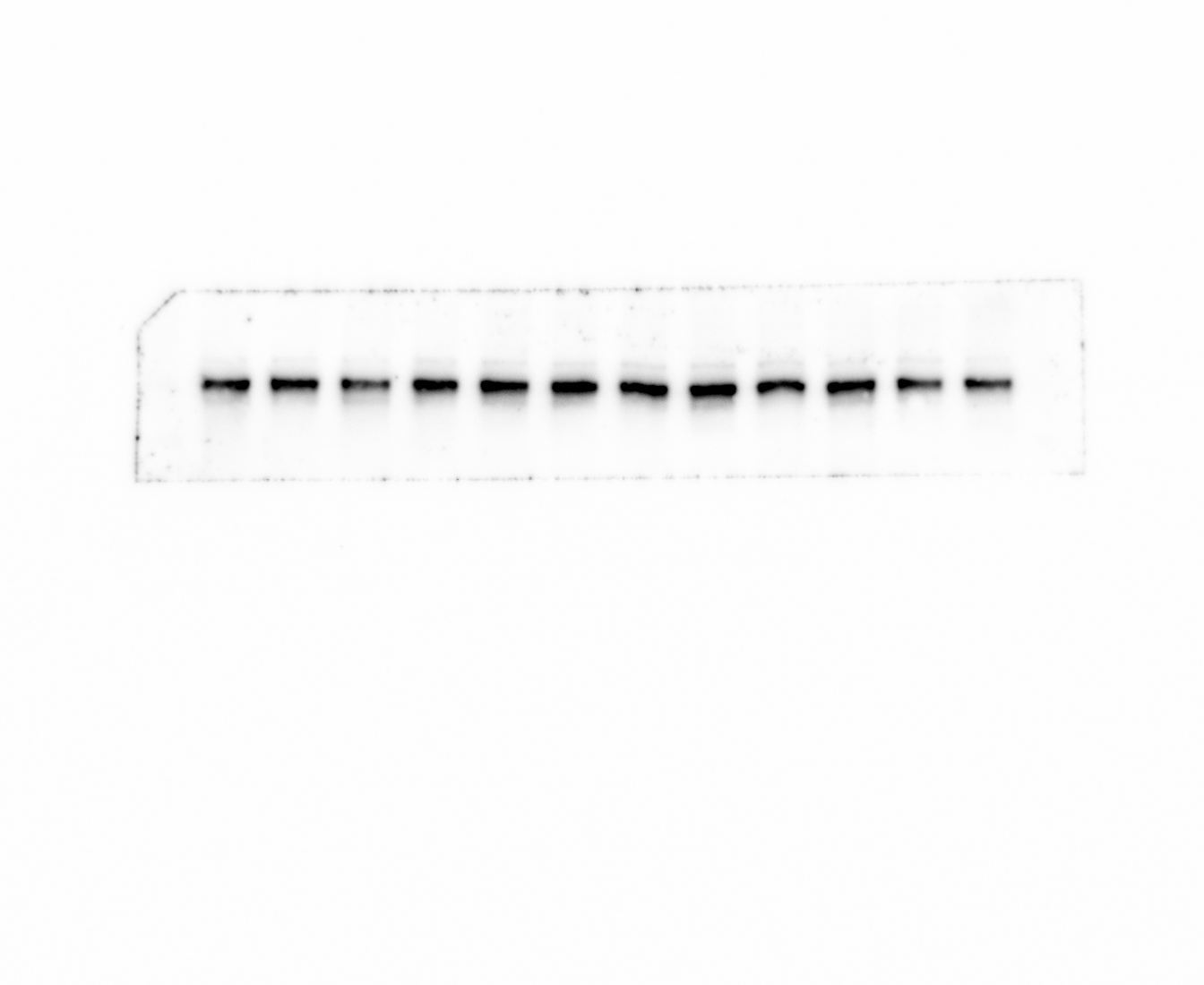

Supplement: Figure 2—source data 1. [file elife-98524-fig2-data1.zip › Fig 2-data1-v1/2F/Tubulin.tif]

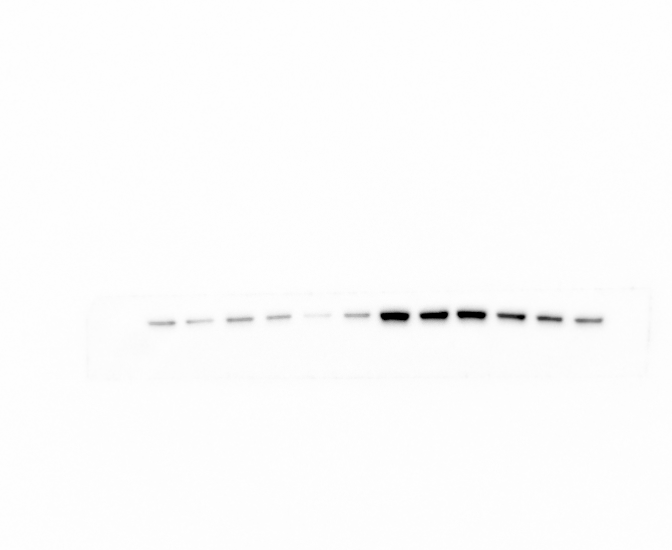

Supplement: Figure 2—source data 1. [file elife-98524-fig2-data1.zip › Fig 2-data1-v1/2F/α-SMA.tif]

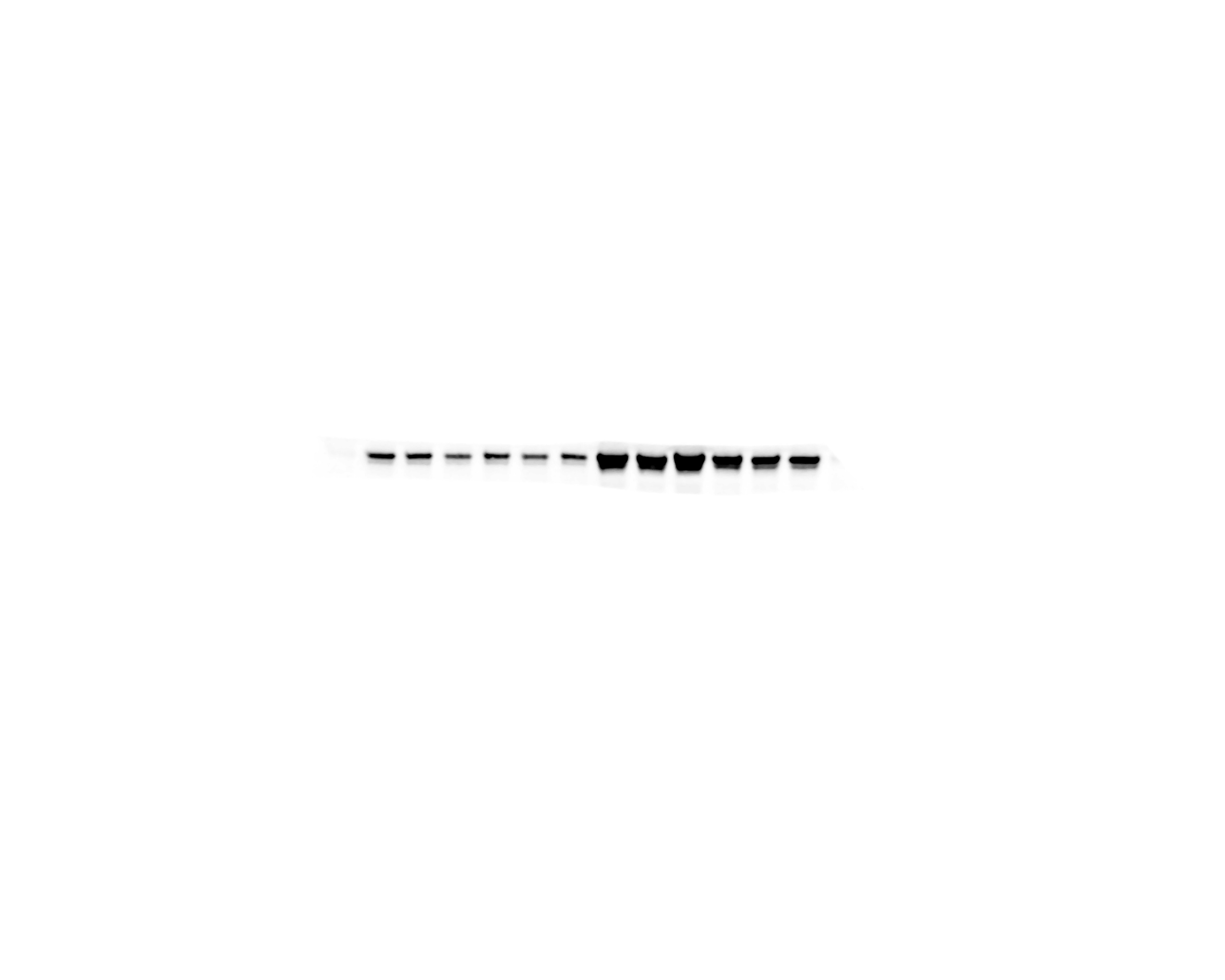

Supplement: Figure 2—source data 1. [file elife-98524-fig2-data1.zip › Fig 2-data1-v1/2J/CCN2.jpg]

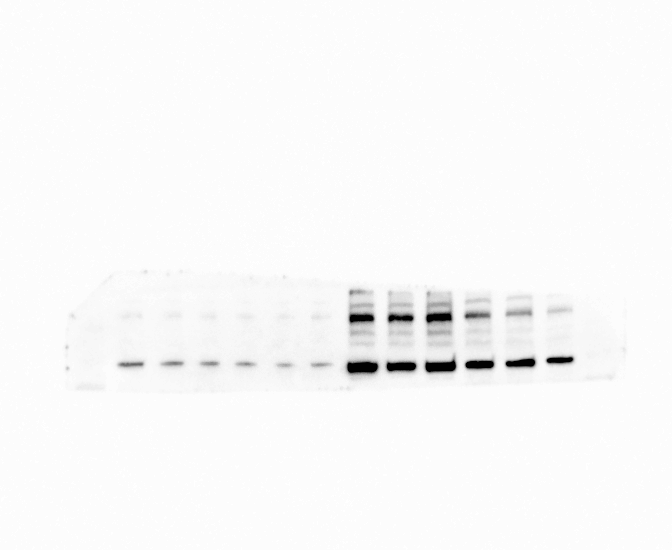

Supplement: Figure 2—source data 1. [file elife-98524-fig2-data1.zip › Fig 2-data1-v1/2J/COL1A1.tif]

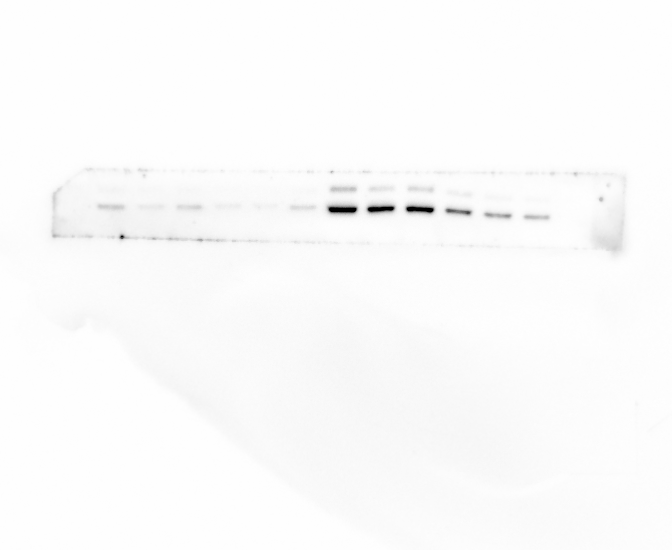

Supplement: Figure 2—source data 1. [file elife-98524-fig2-data1.zip › Fig 2-data1-v1/2J/COL3A1.tif]

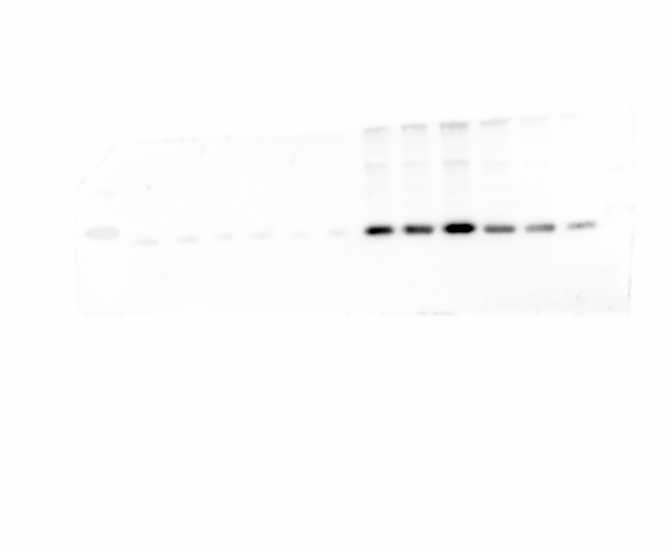

Supplement: Figure 2—source data 1. [file elife-98524-fig2-data1.zip › Fig 2-data1-v1/2J/FN1.tif]

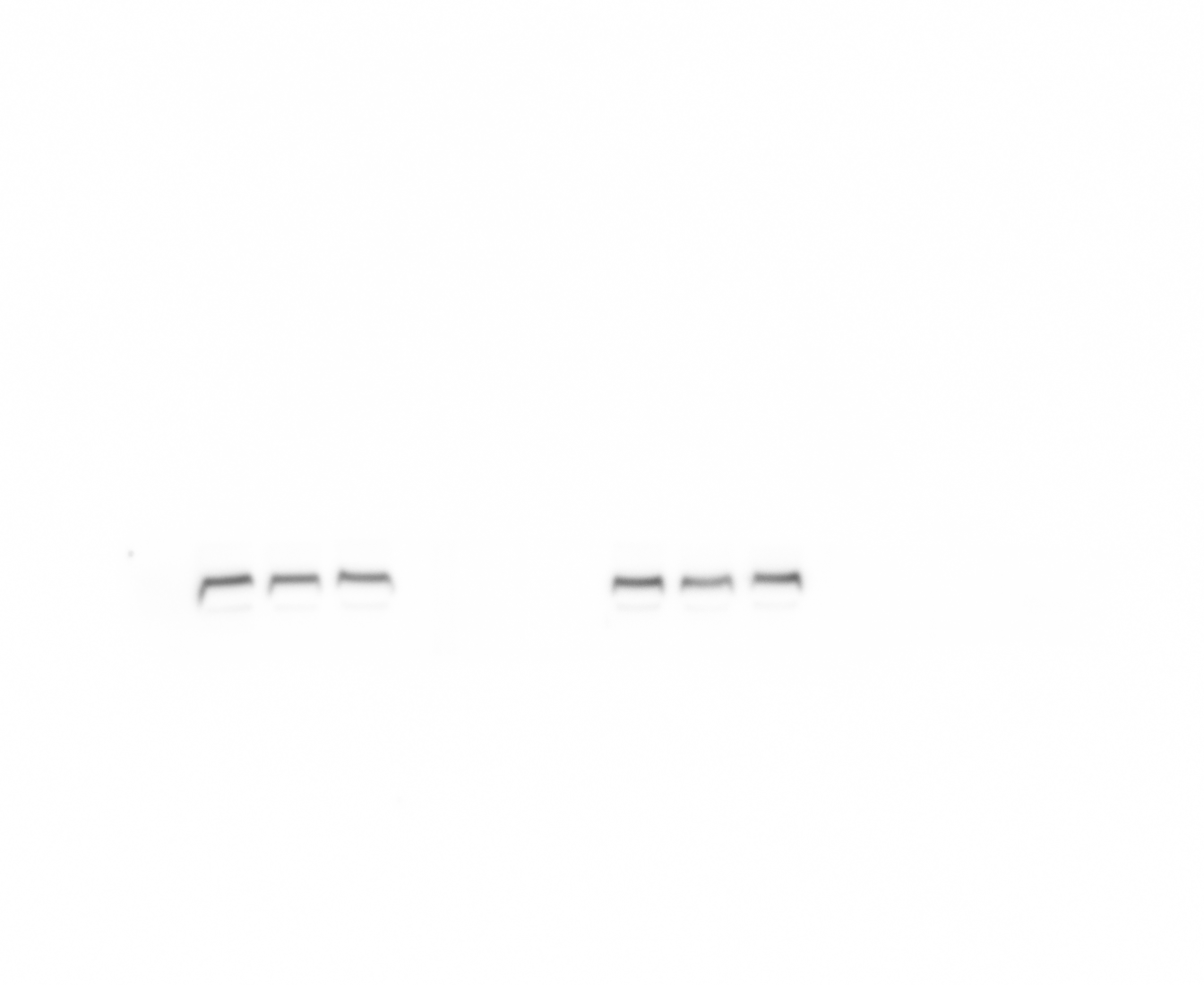

Supplement: Figure 2—source data 1. [file elife-98524-fig2-data1.zip › Fig 2-data1-v1/2J/SIRT4.tif]

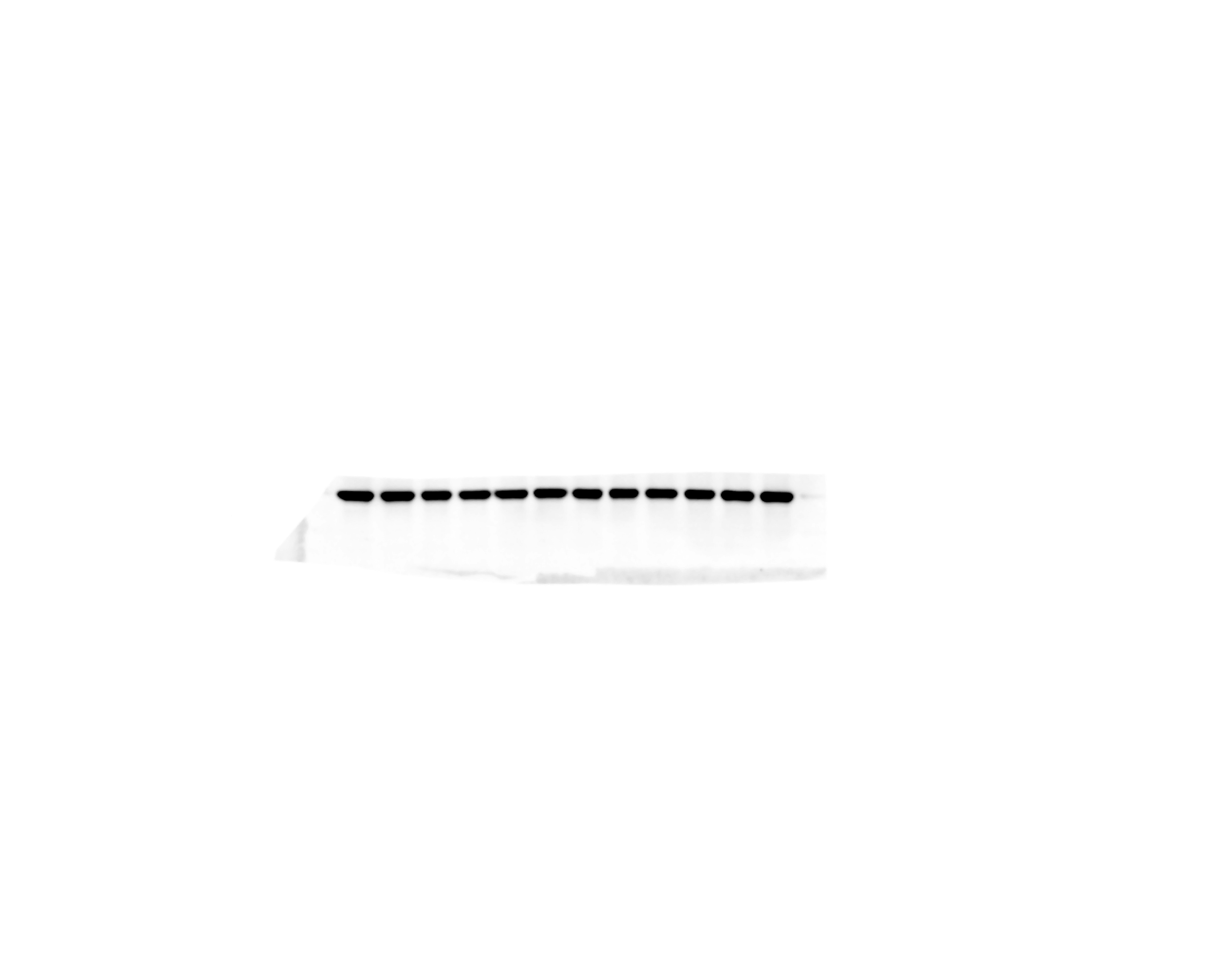

Supplement: Figure 2—source data 1. [file elife-98524-fig2-data1.zip › Fig 2-data1-v1/2J/Tubulin bottom.tiff]

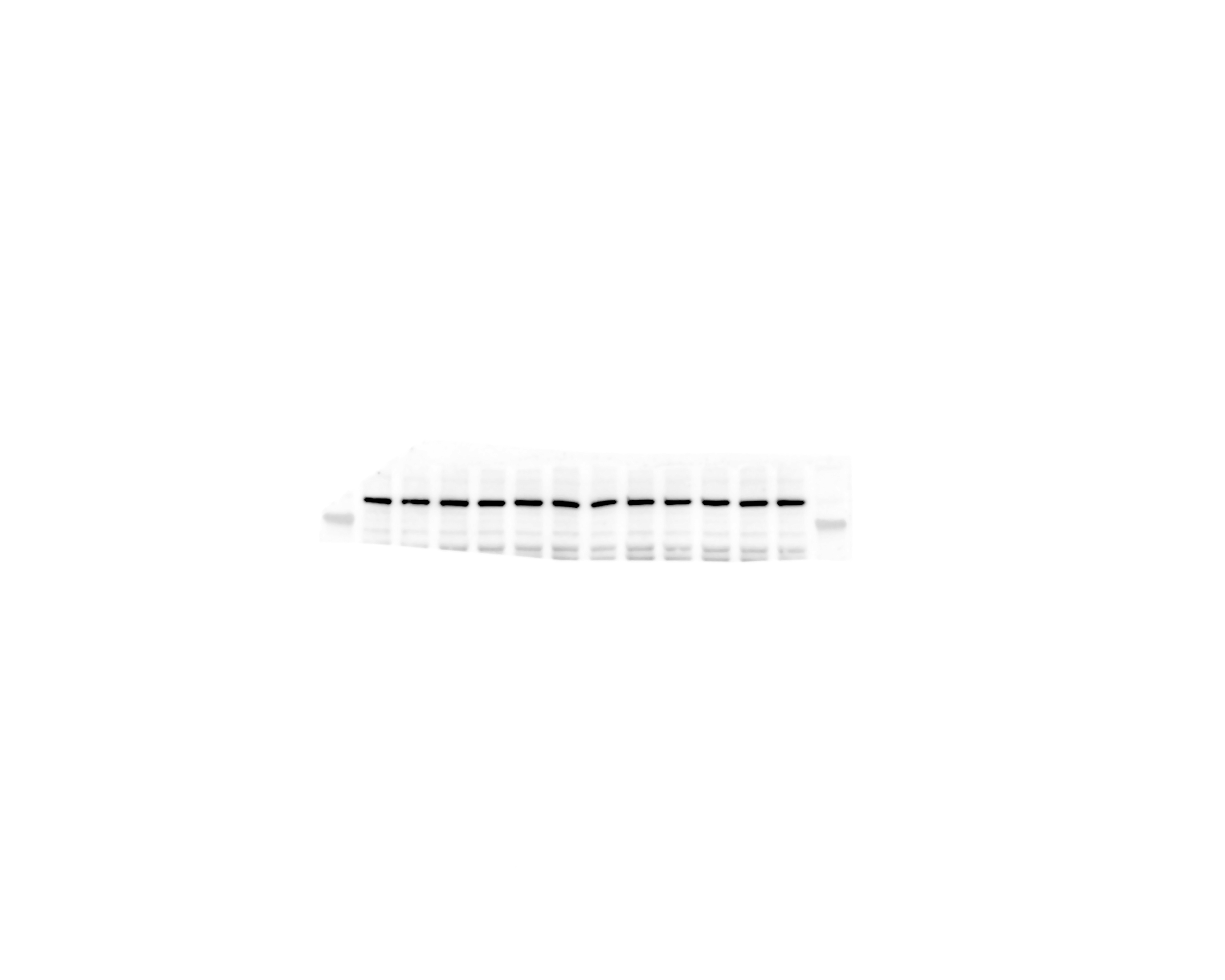

Supplement: Figure 2—source data 1. [file elife-98524-fig2-data1.zip › Fig 2-data1-v1/2J/Tubulin upper.tiff]

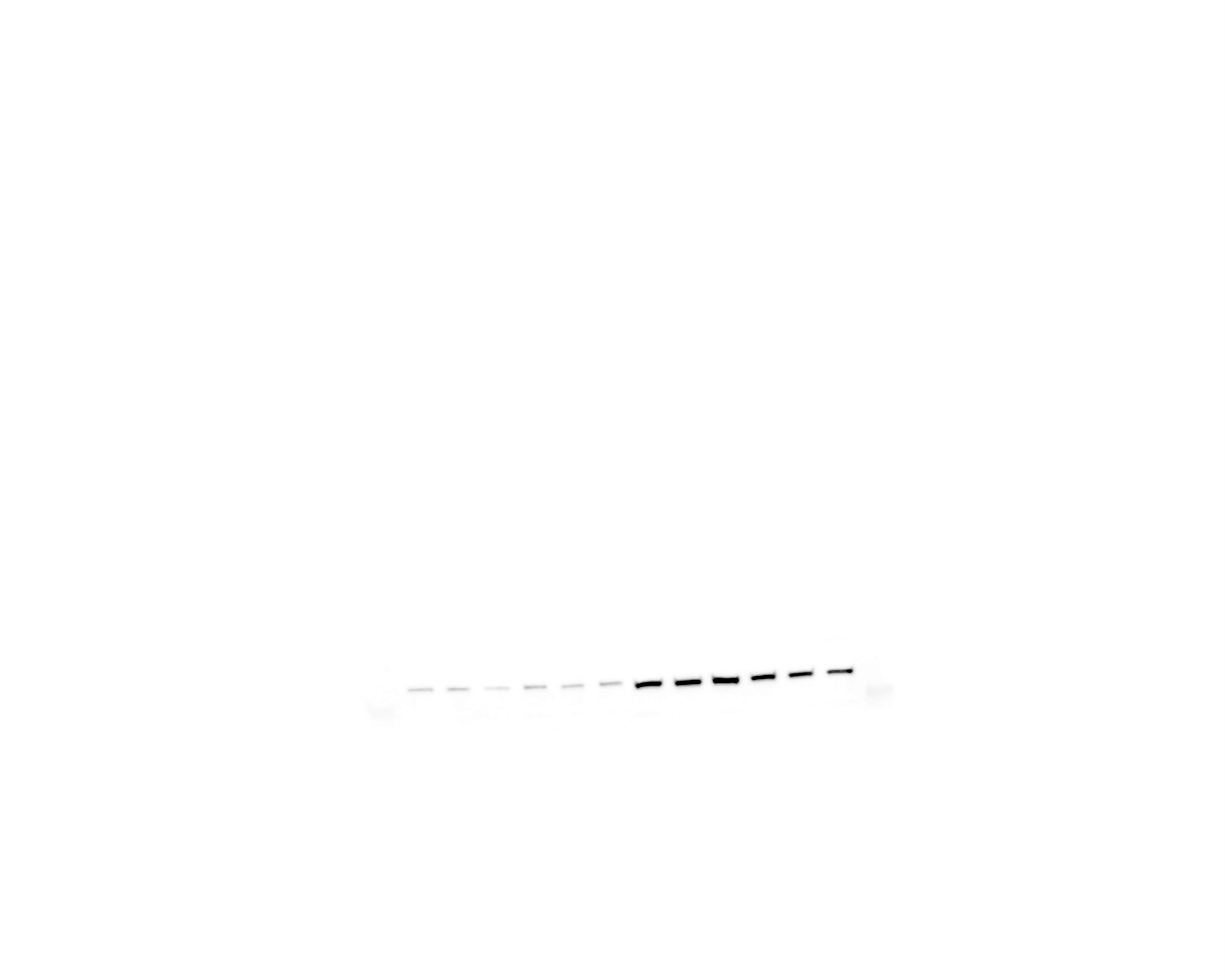

Supplement: Figure 2—source data 1. [file elife-98524-fig2-data1.zip › Fig 2-data1-v1/2J/α-SMA.jpg]

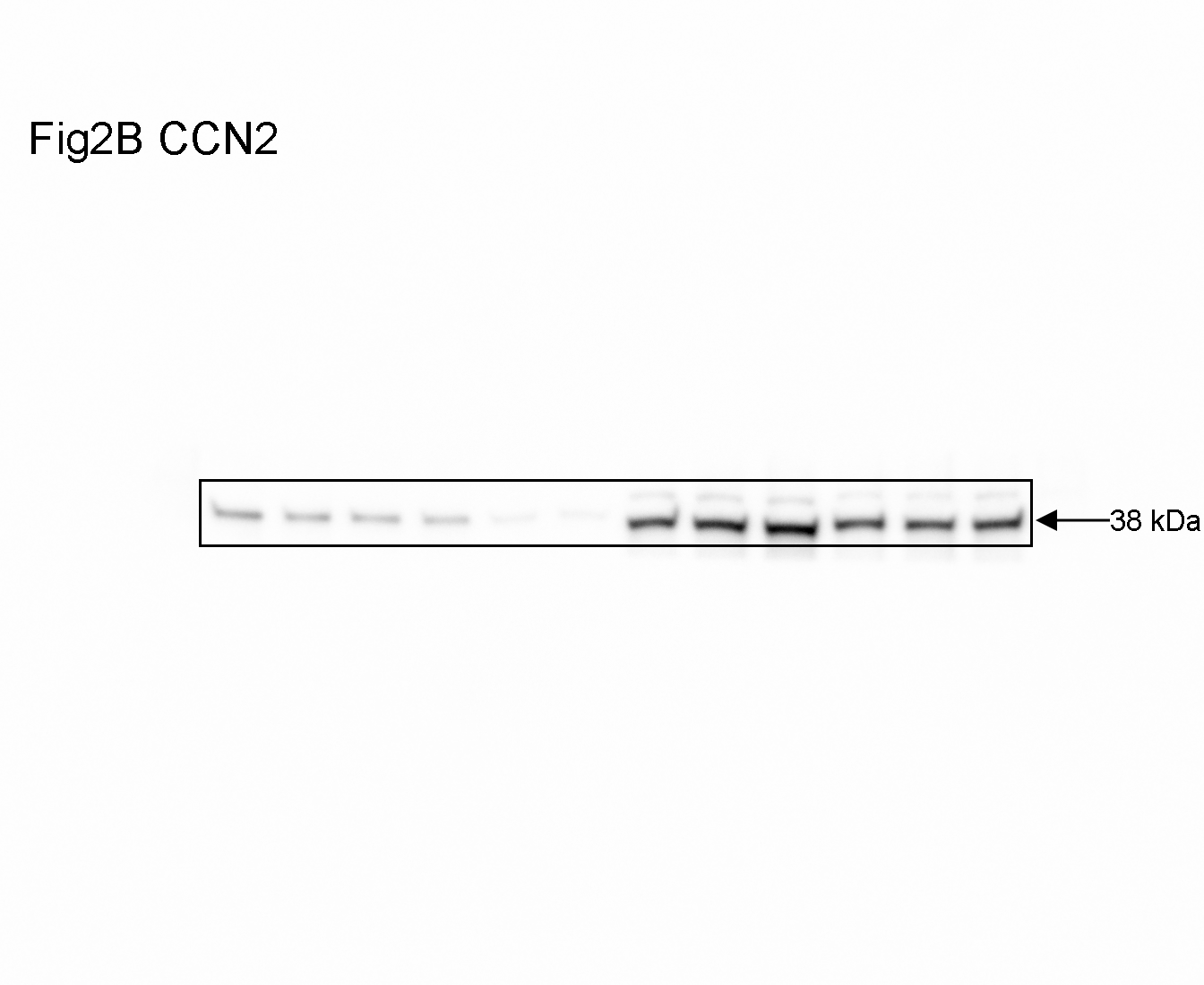

Supplement: Figure 2—source data 2. [file elife-98524-fig2-data2.zip › Fig 2-data2-v1/2B/CCN2.tif]

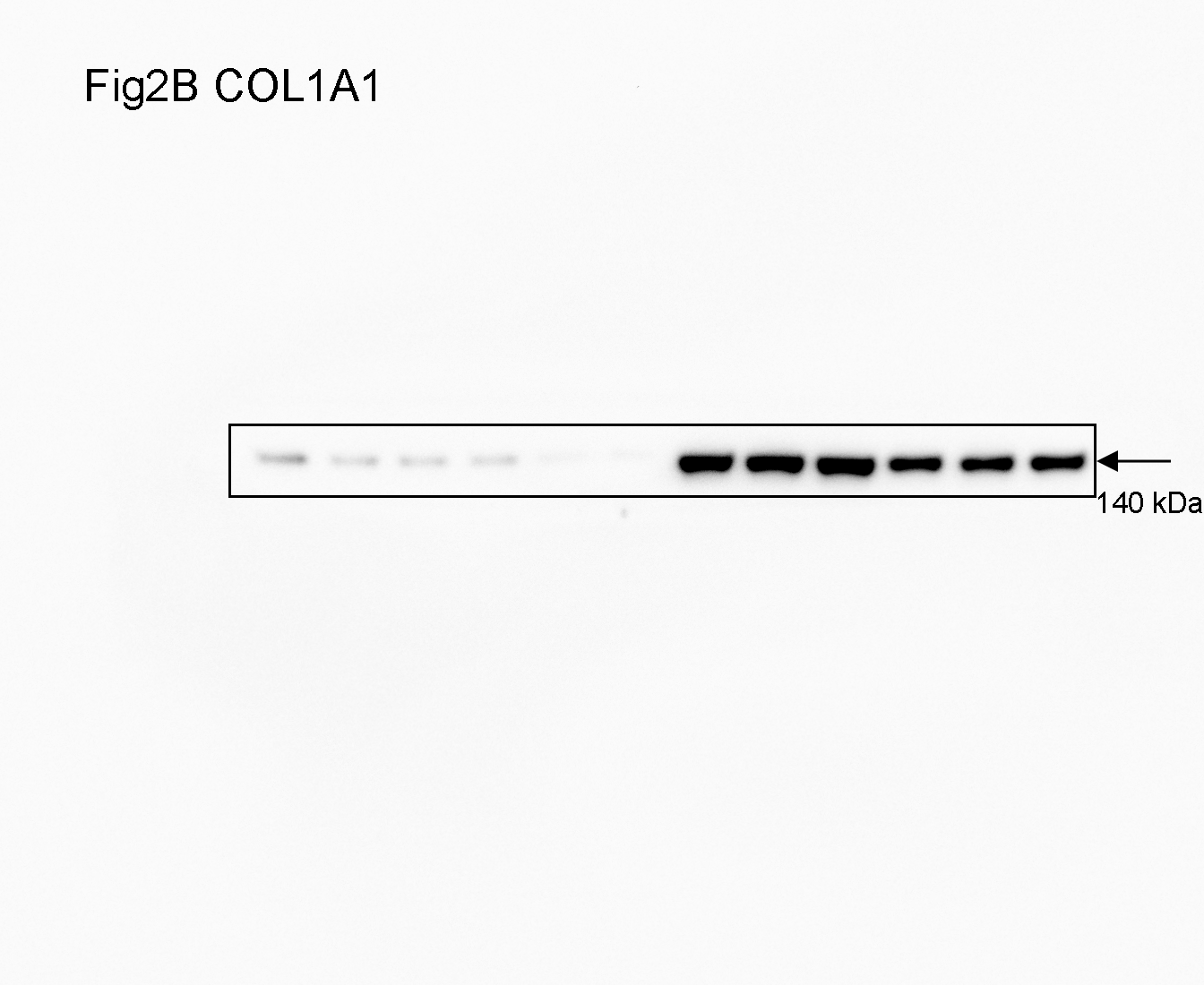

Supplement: Figure 2—source data 2. [file elife-98524-fig2-data2.zip › Fig 2-data2-v1/2B/COL1A1.tif]

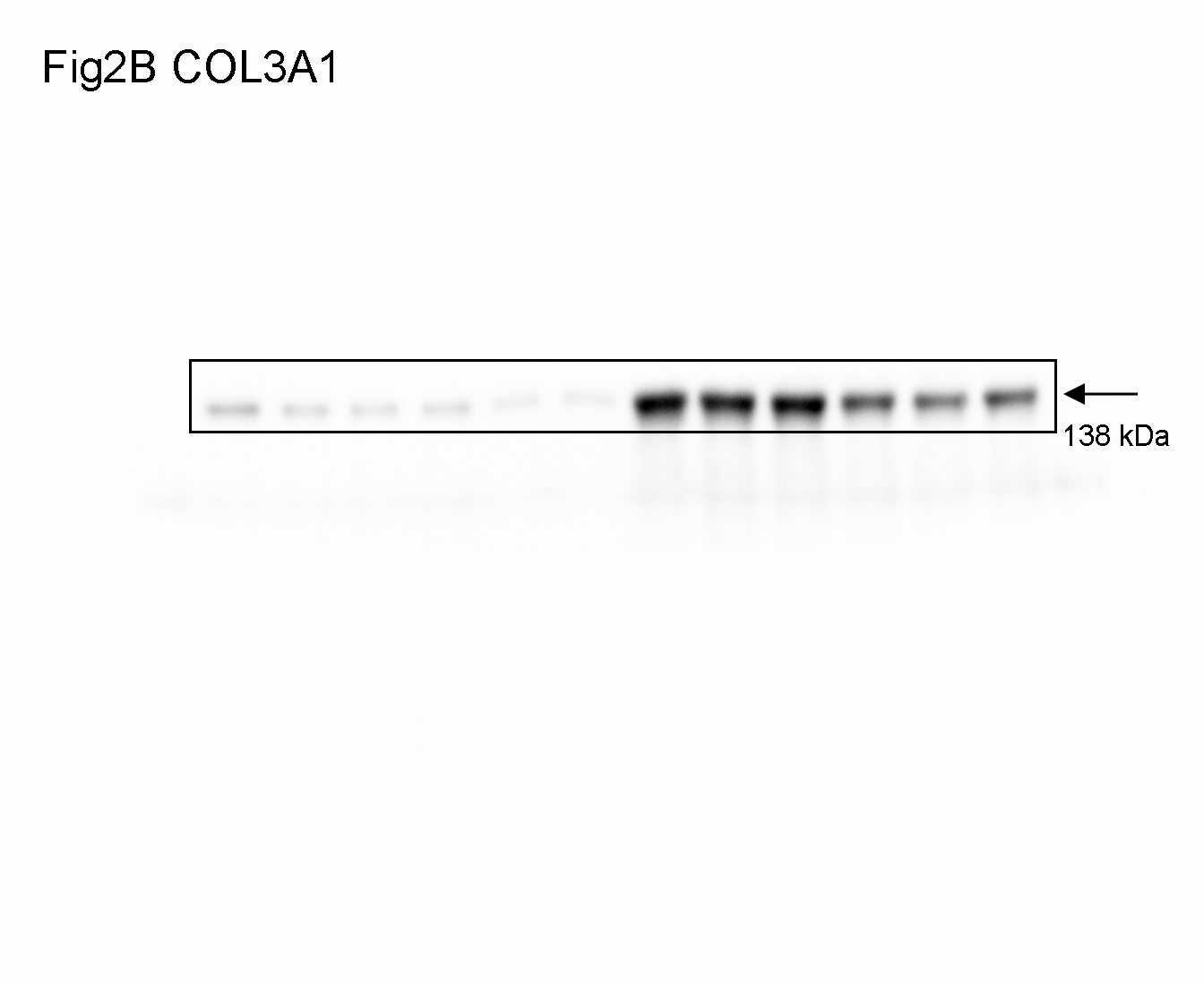

Supplement: Figure 2—source data 2. [file elife-98524-fig2-data2.zip › Fig 2-data2-v1/2B/COL3A1.tif]

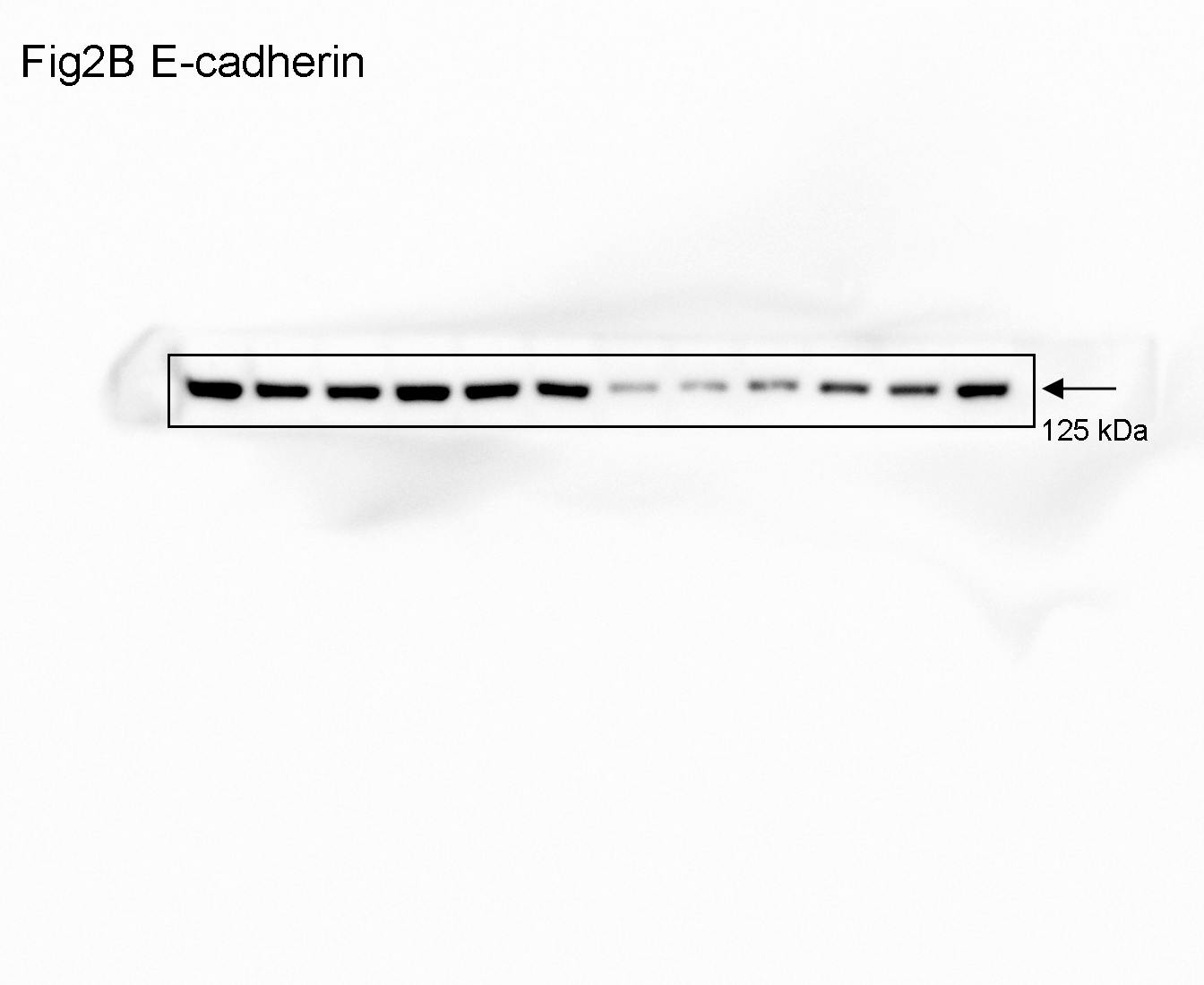

Supplement: Figure 2—source data 2. [file elife-98524-fig2-data2.zip › Fig 2-data2-v1/2B/E-cadherin.tif]

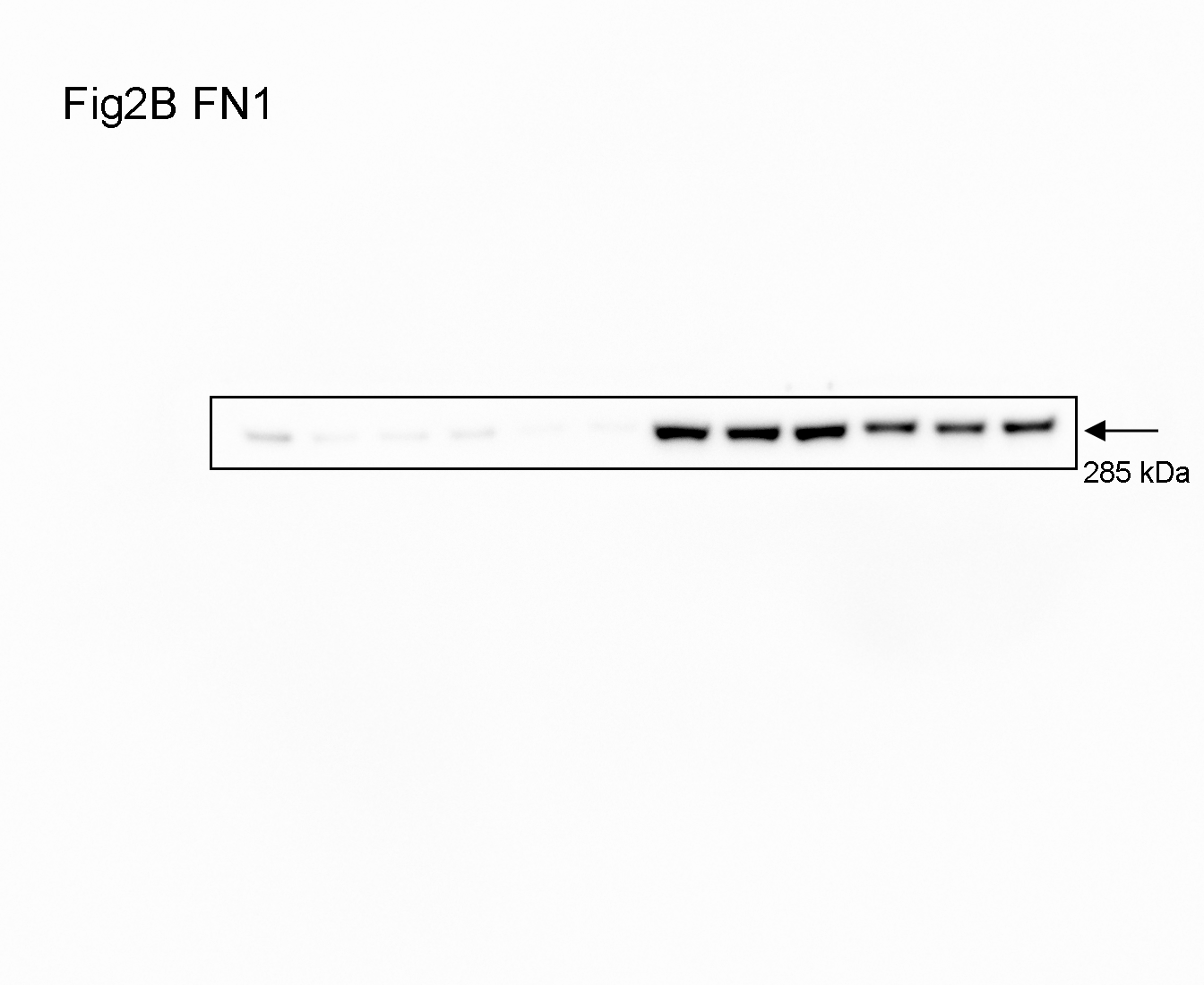

Supplement: Figure 2—source data 2. [file elife-98524-fig2-data2.zip › Fig 2-data2-v1/2B/FN1.tif]

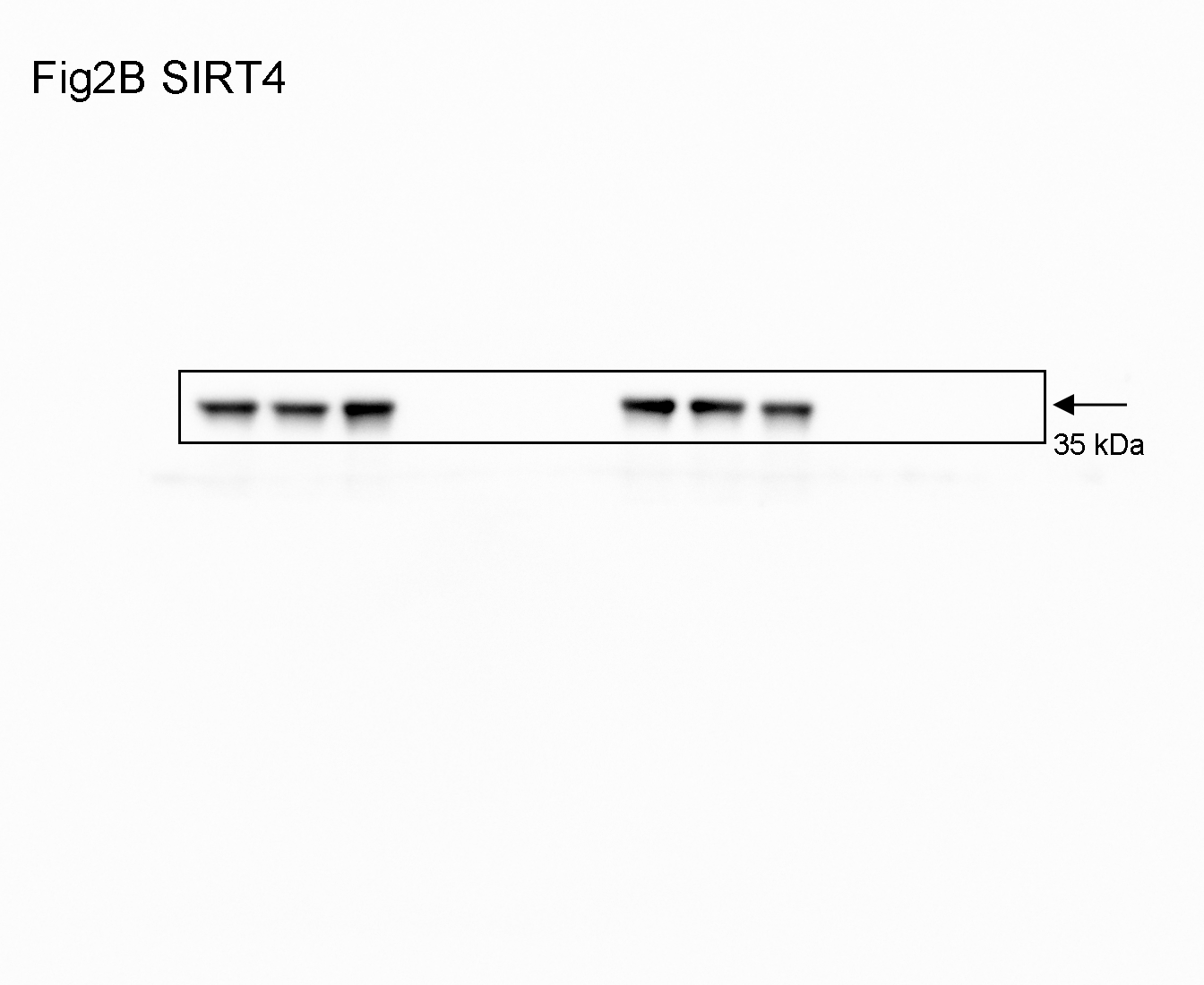

Supplement: Figure 2—source data 2. [file elife-98524-fig2-data2.zip › Fig 2-data2-v1/2B/SIRT4.tif]

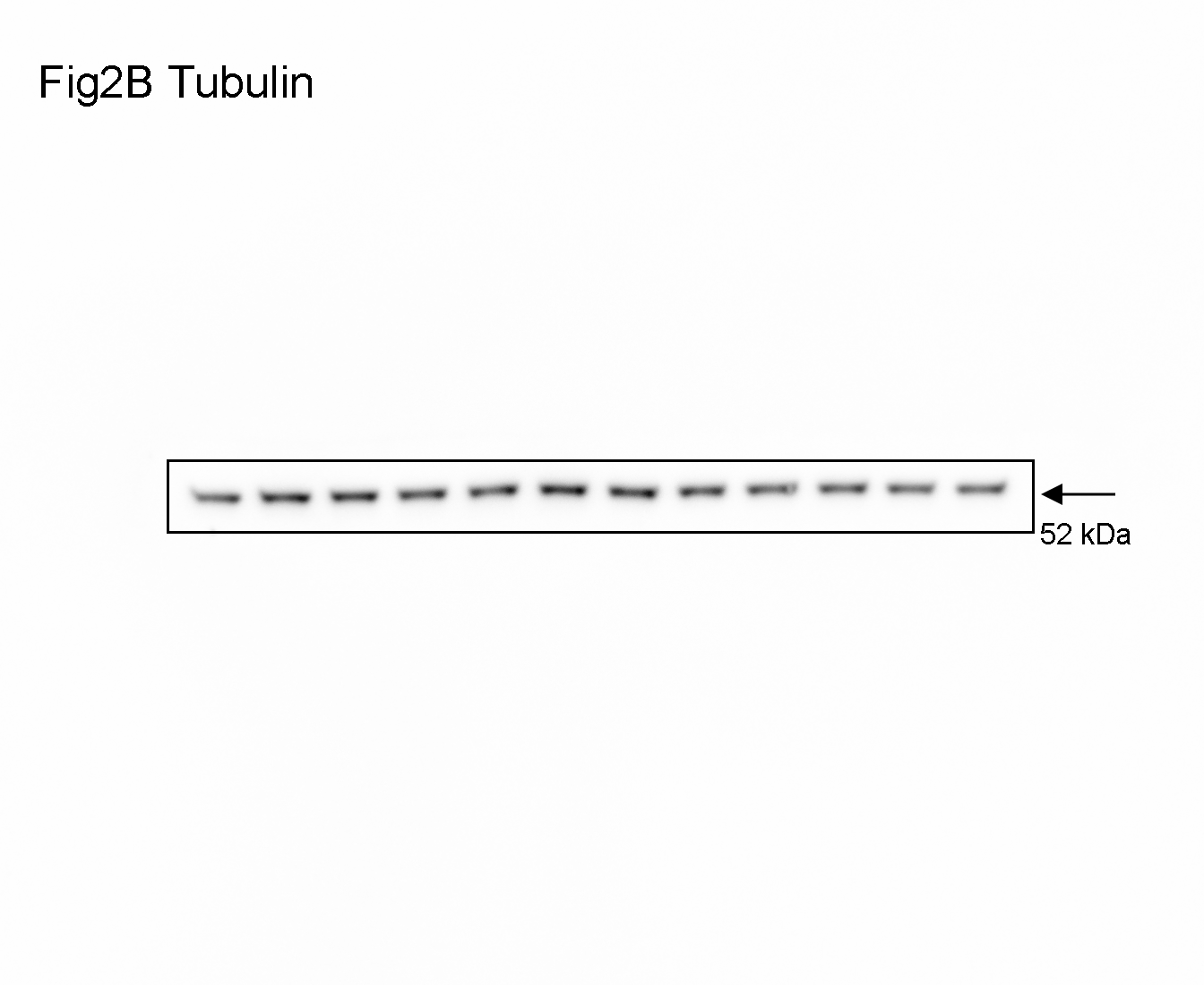

Supplement: Figure 2—source data 2. [file elife-98524-fig2-data2.zip › Fig 2-data2-v1/2B/Tubulin.tif]

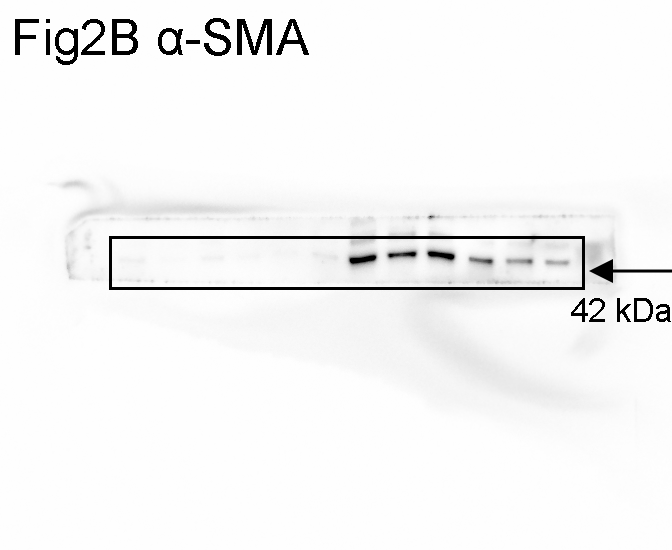

Supplement: Figure 2—source data 2. [file elife-98524-fig2-data2.zip › Fig 2-data2-v1/2B/α-SMA.tif]

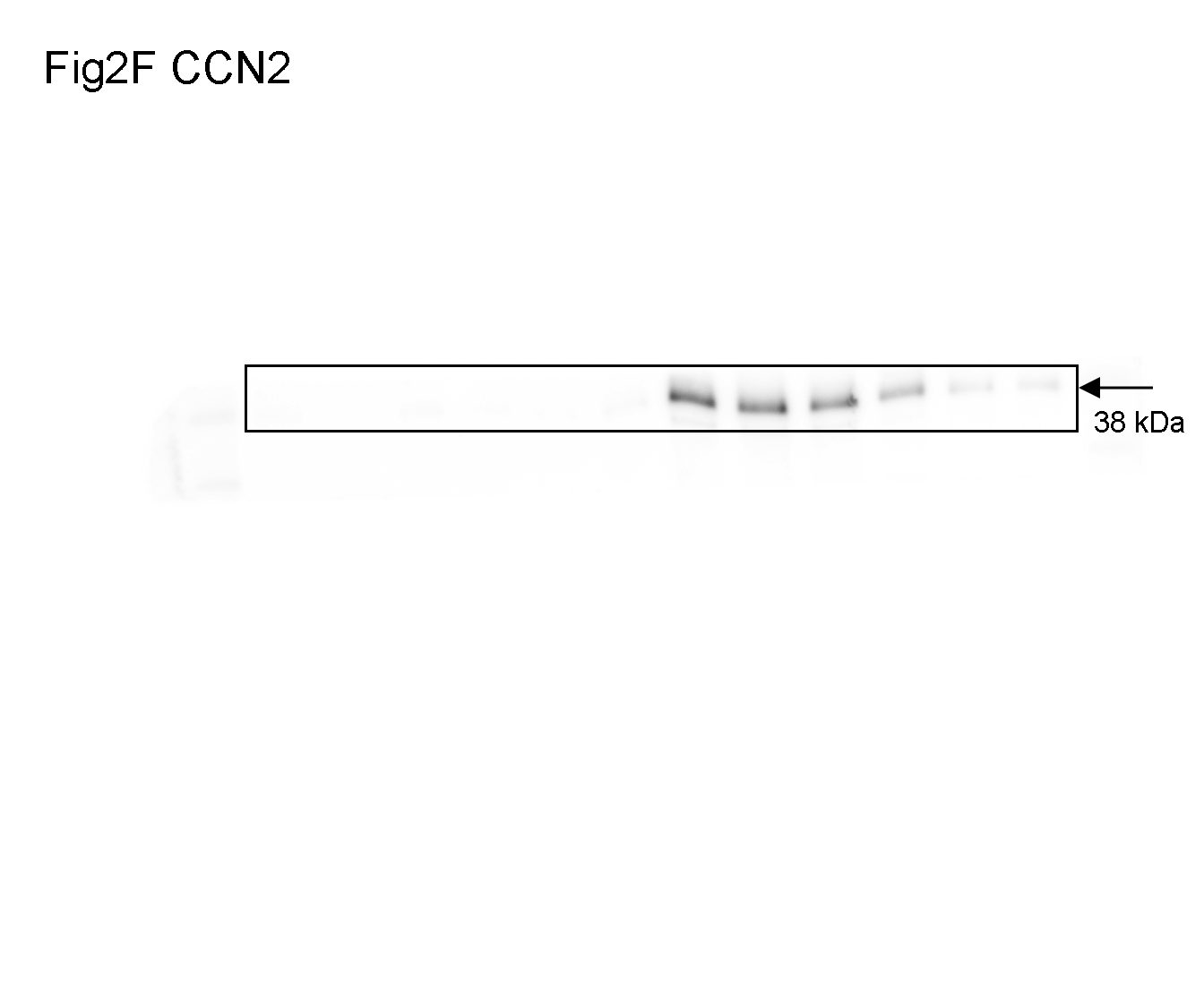

Supplement: Figure 2—source data 2. [file elife-98524-fig2-data2.zip › Fig 2-data2-v1/2F/CCN2.tif]

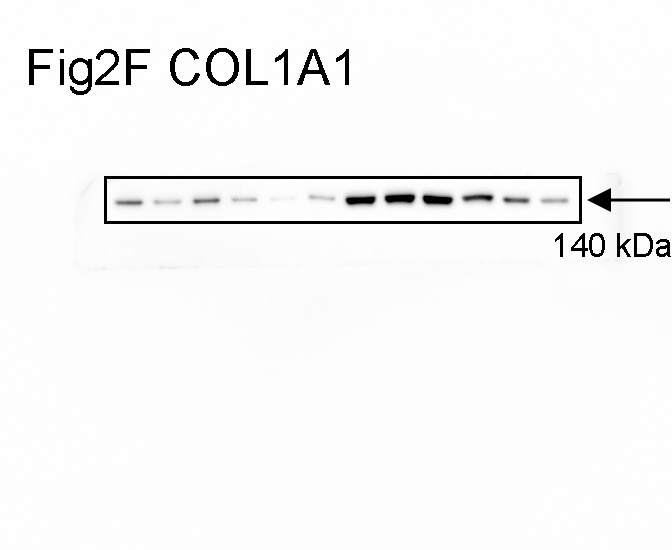

Supplement: Figure 2—source data 2. [file elife-98524-fig2-data2.zip › Fig 2-data2-v1/2F/COL1A1.tif]

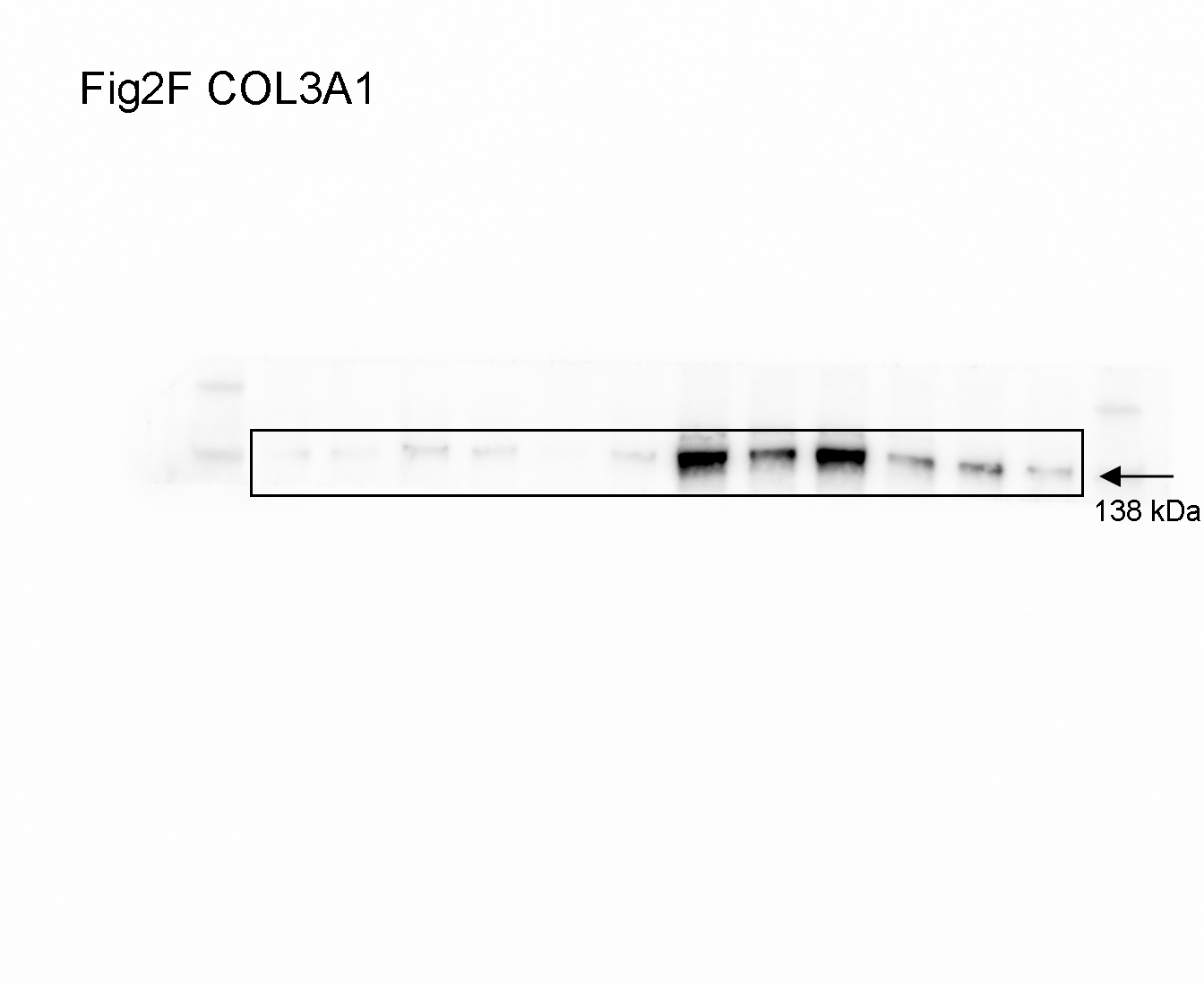

Supplement: Figure 2—source data 2. [file elife-98524-fig2-data2.zip › Fig 2-data2-v1/2F/COL3A1.tif]

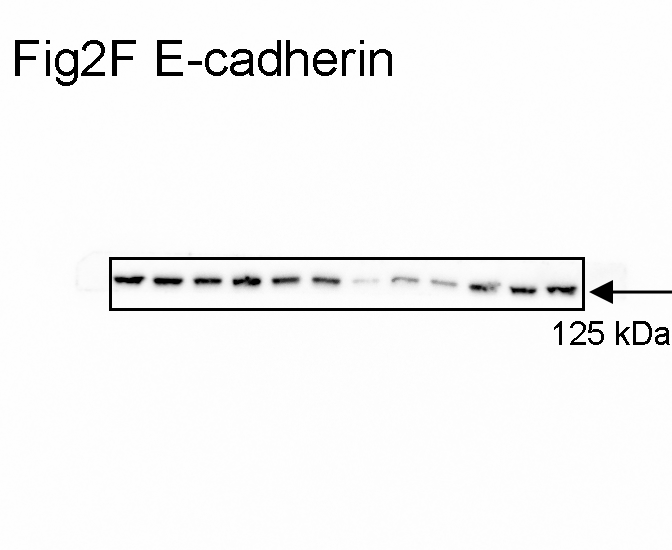

Supplement: Figure 2—source data 2. [file elife-98524-fig2-data2.zip › Fig 2-data2-v1/2F/E-cadherin.tif]

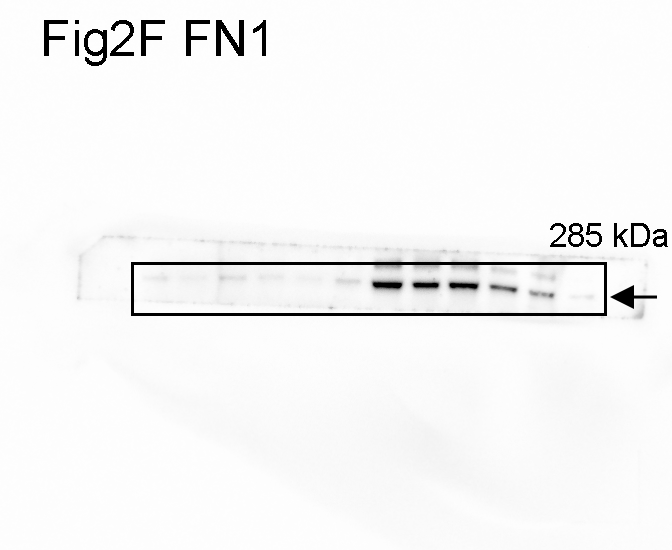

Supplement: Figure 2—source data 2. [file elife-98524-fig2-data2.zip › Fig 2-data2-v1/2F/FN1.tif]

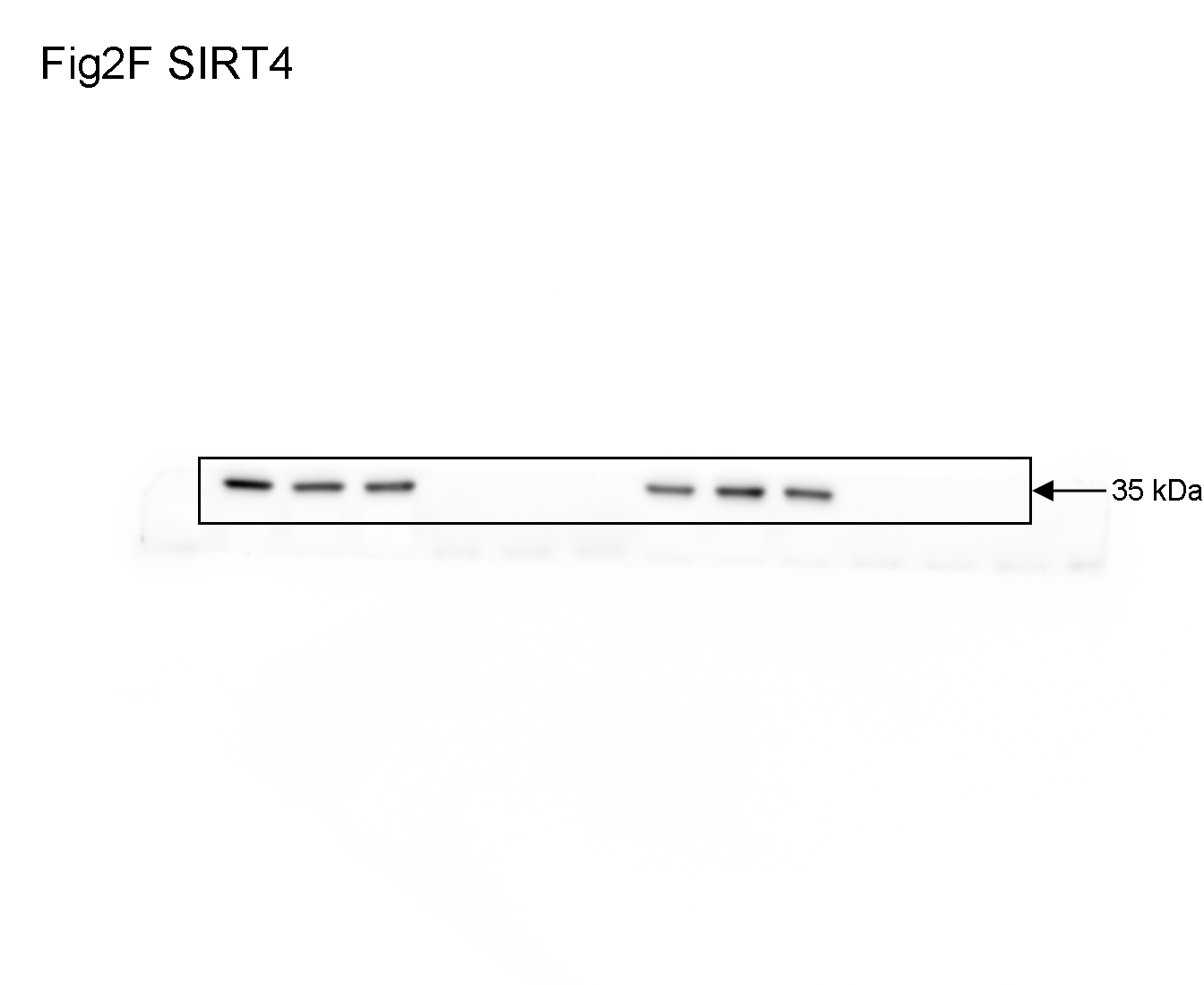

Supplement: Figure 2—source data 2. [file elife-98524-fig2-data2.zip › Fig 2-data2-v1/2F/SIRT4.tif]

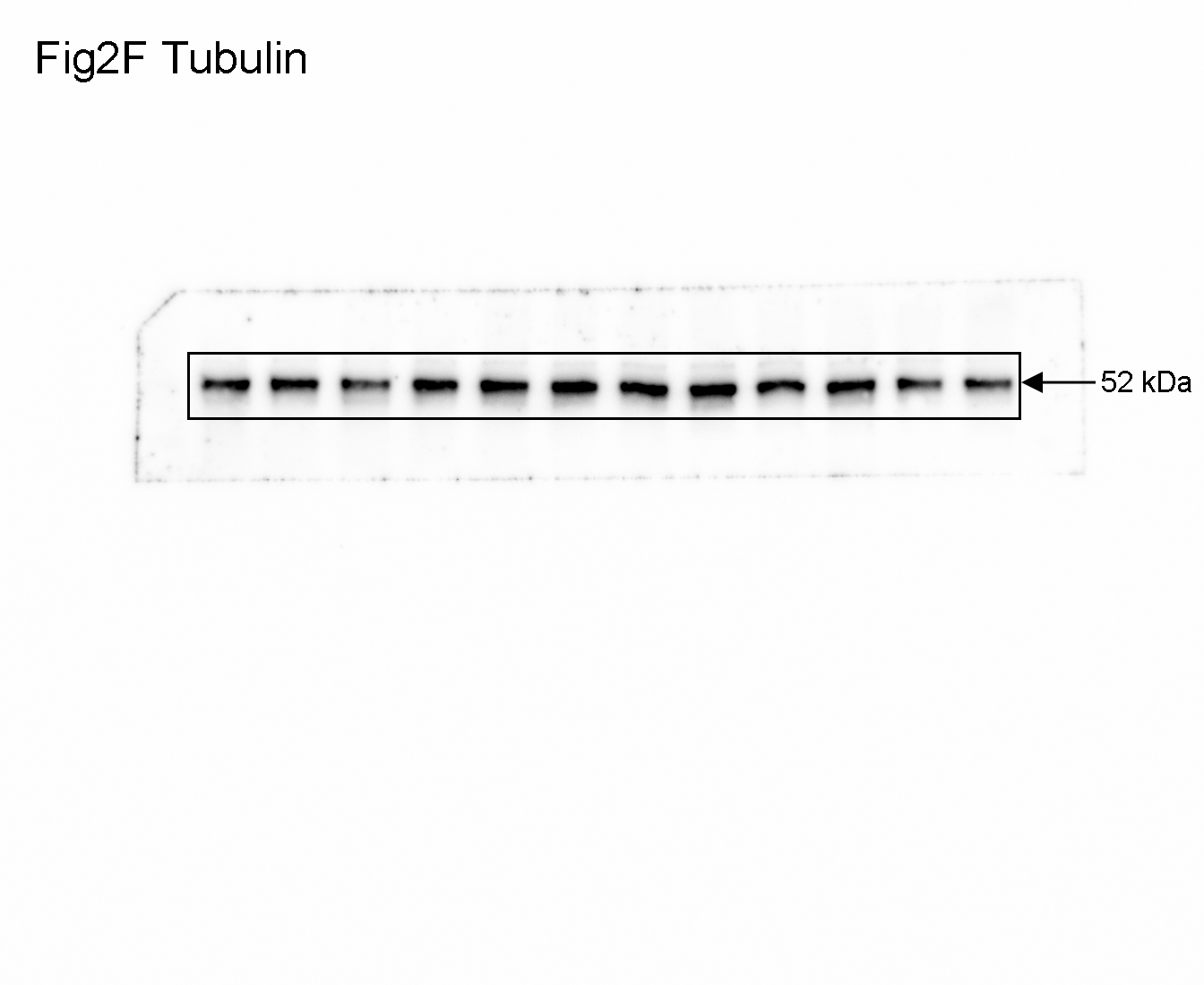

Supplement: Figure 2—source data 2. [file elife-98524-fig2-data2.zip › Fig 2-data2-v1/2F/Tubulin.tif]

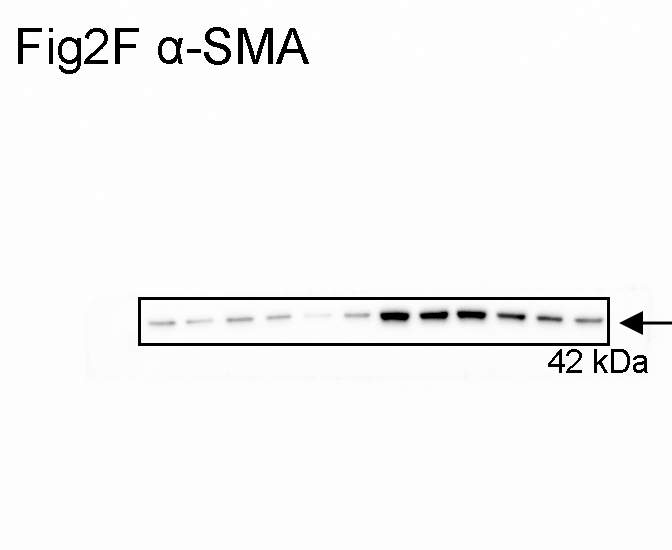

Supplement: Figure 2—source data 2. [file elife-98524-fig2-data2.zip › Fig 2-data2-v1/2F/α-SMA.tif]

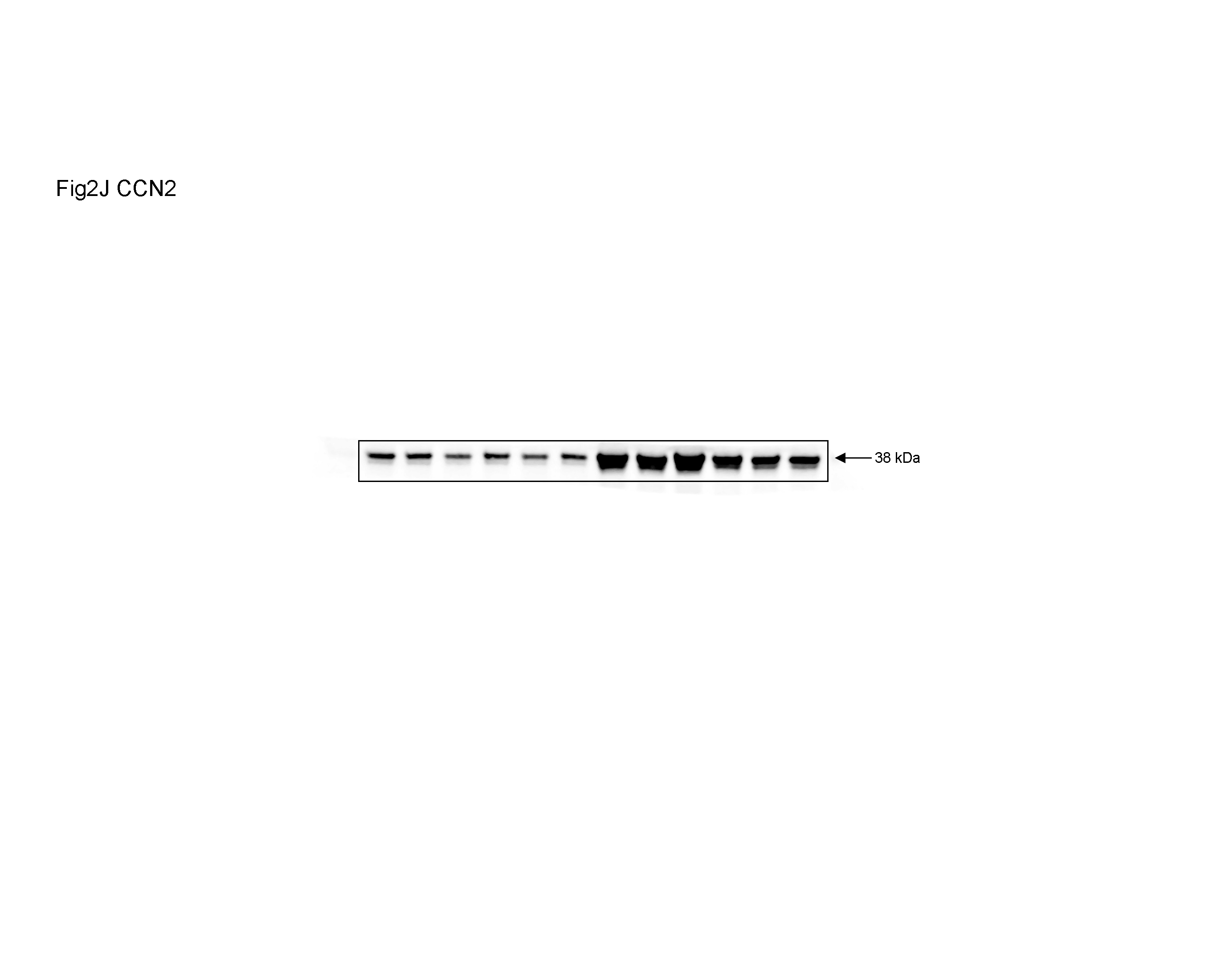

Supplement: Figure 2—source data 2. [file elife-98524-fig2-data2.zip › Fig 2-data2-v1/2J/CCN2.tif]

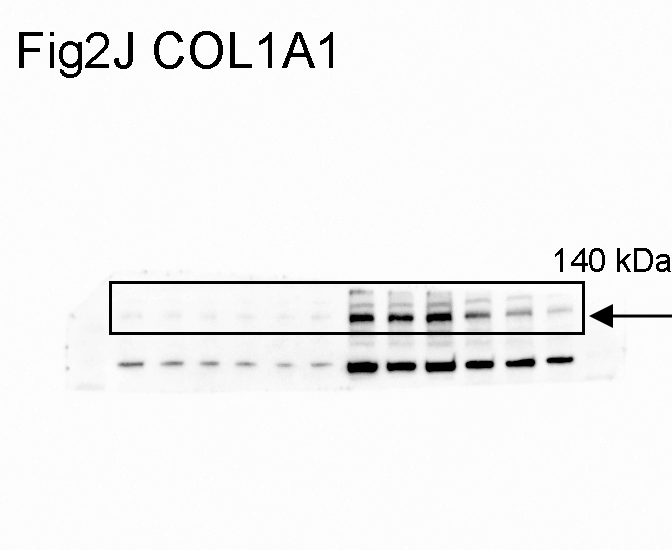

Supplement: Figure 2—source data 2. [file elife-98524-fig2-data2.zip › Fig 2-data2-v1/2J/COL1A1.tif]

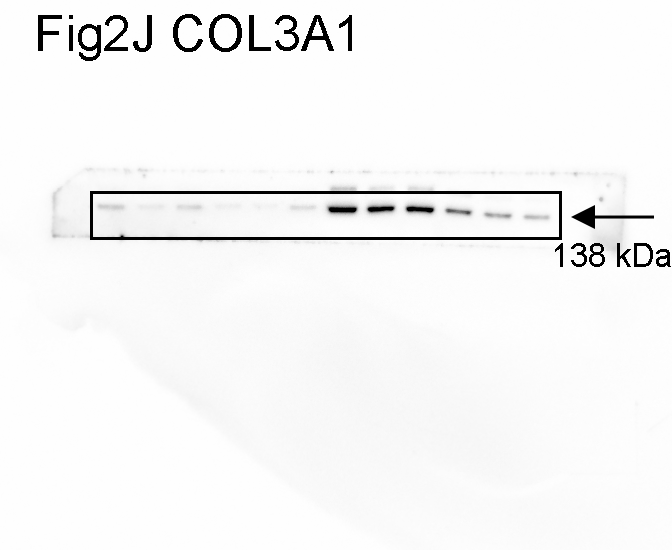

Supplement: Figure 2—source data 2. [file elife-98524-fig2-data2.zip › Fig 2-data2-v1/2J/COL3A1.tif]

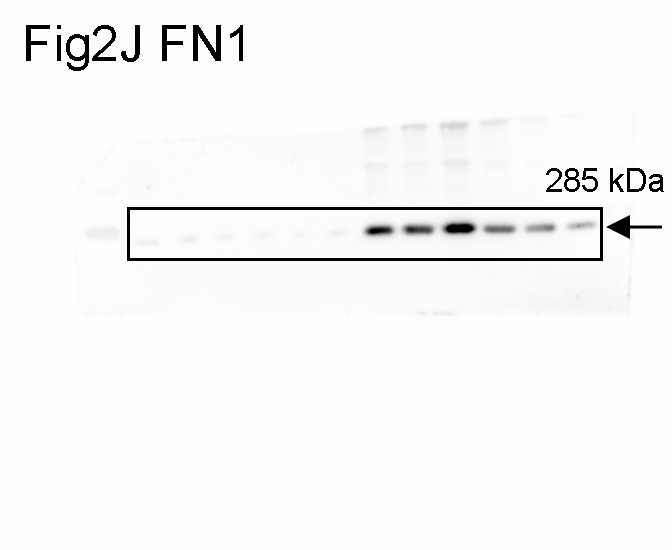

Supplement: Figure 2—source data 2. [file elife-98524-fig2-data2.zip › Fig 2-data2-v1/2J/FN1.tif]

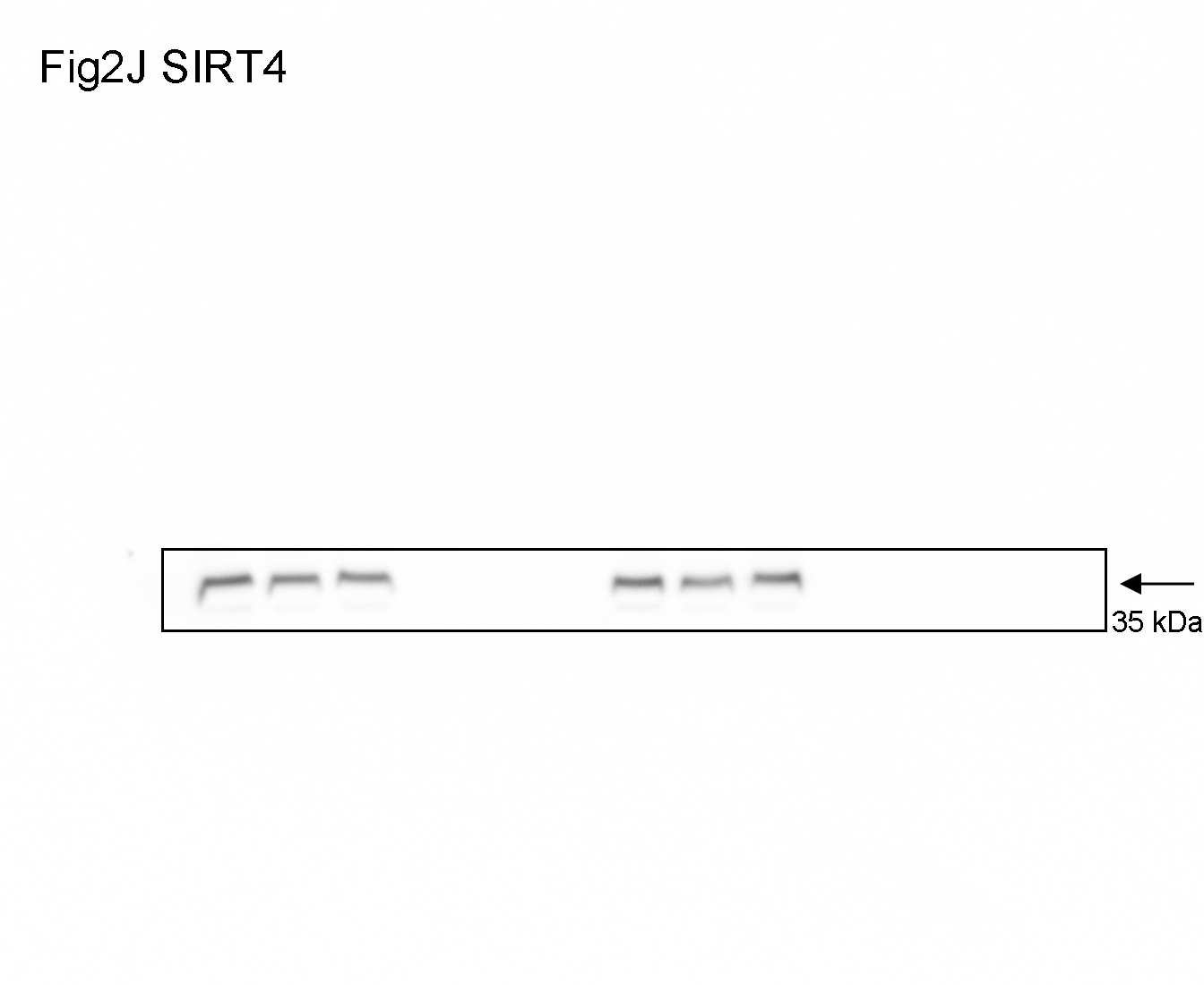

Supplement: Figure 2—source data 2. [file elife-98524-fig2-data2.zip › Fig 2-data2-v1/2J/SIRT4.tif]

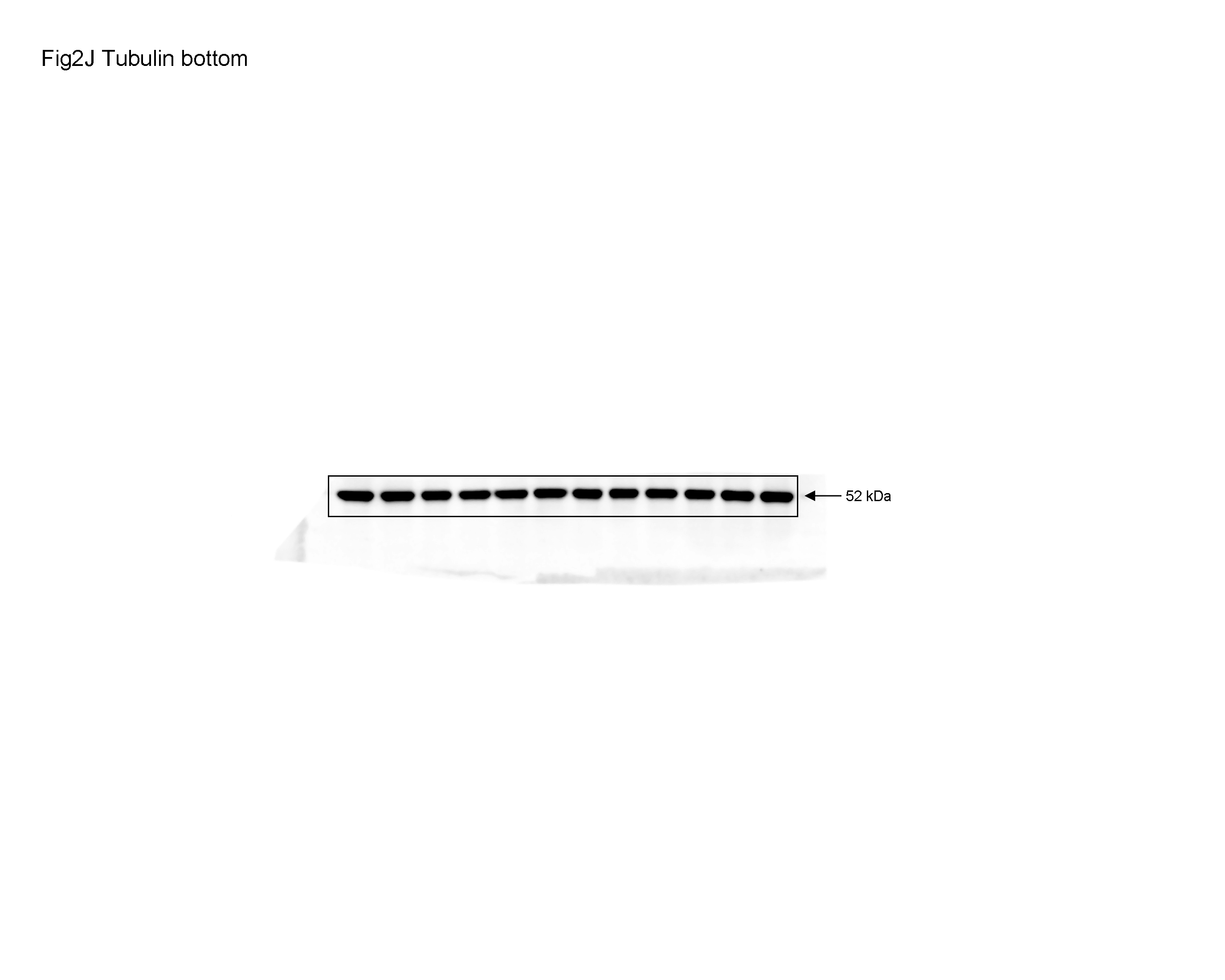

Supplement: Figure 2—source data 2. [file elife-98524-fig2-data2.zip › Fig 2-data2-v1/2J/Tubulin bottom.tif]

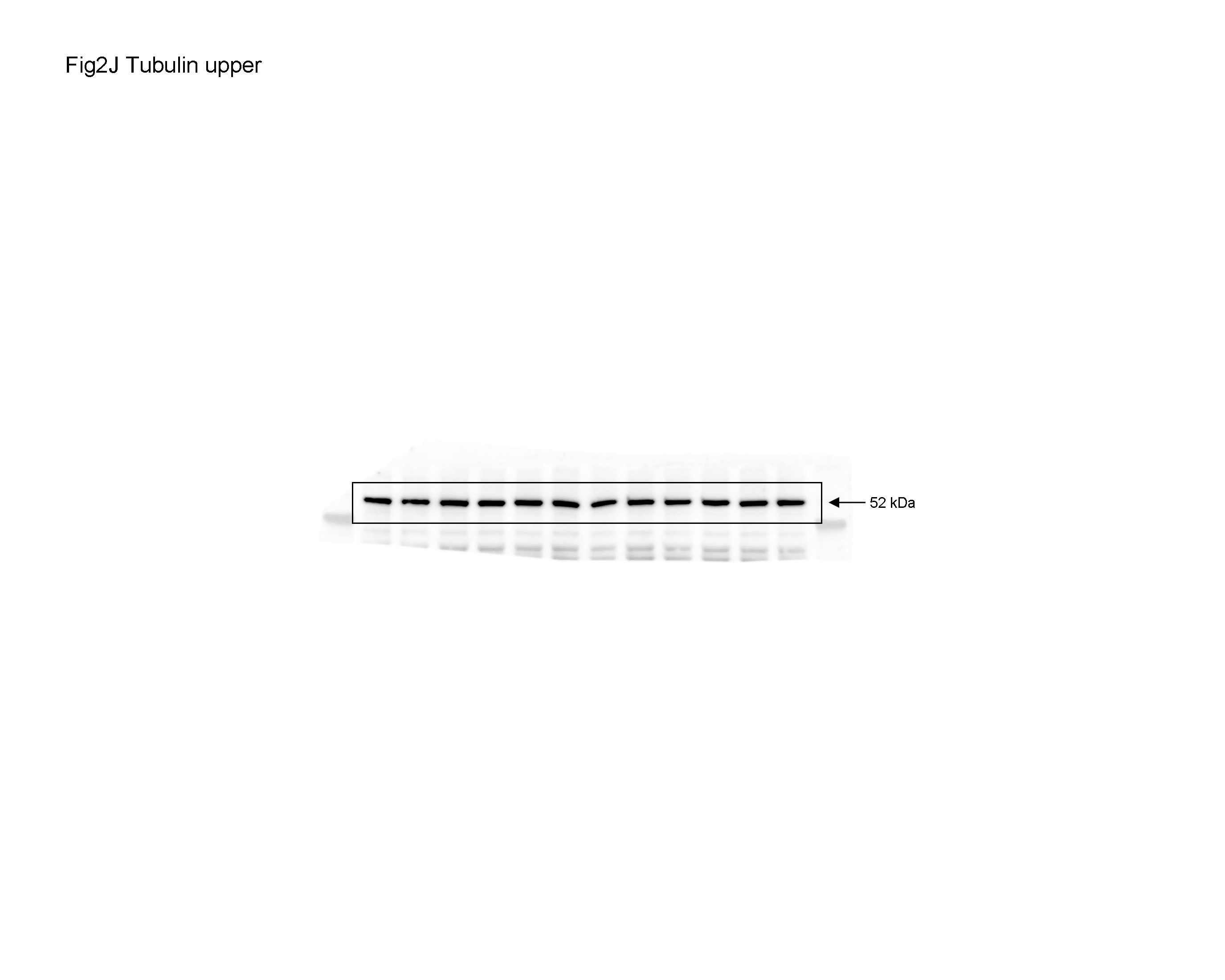

Supplement: Figure 2—source data 2. [file elife-98524-fig2-data2.zip › Fig 2-data2-v1/2J/Tubulin upper.tif]

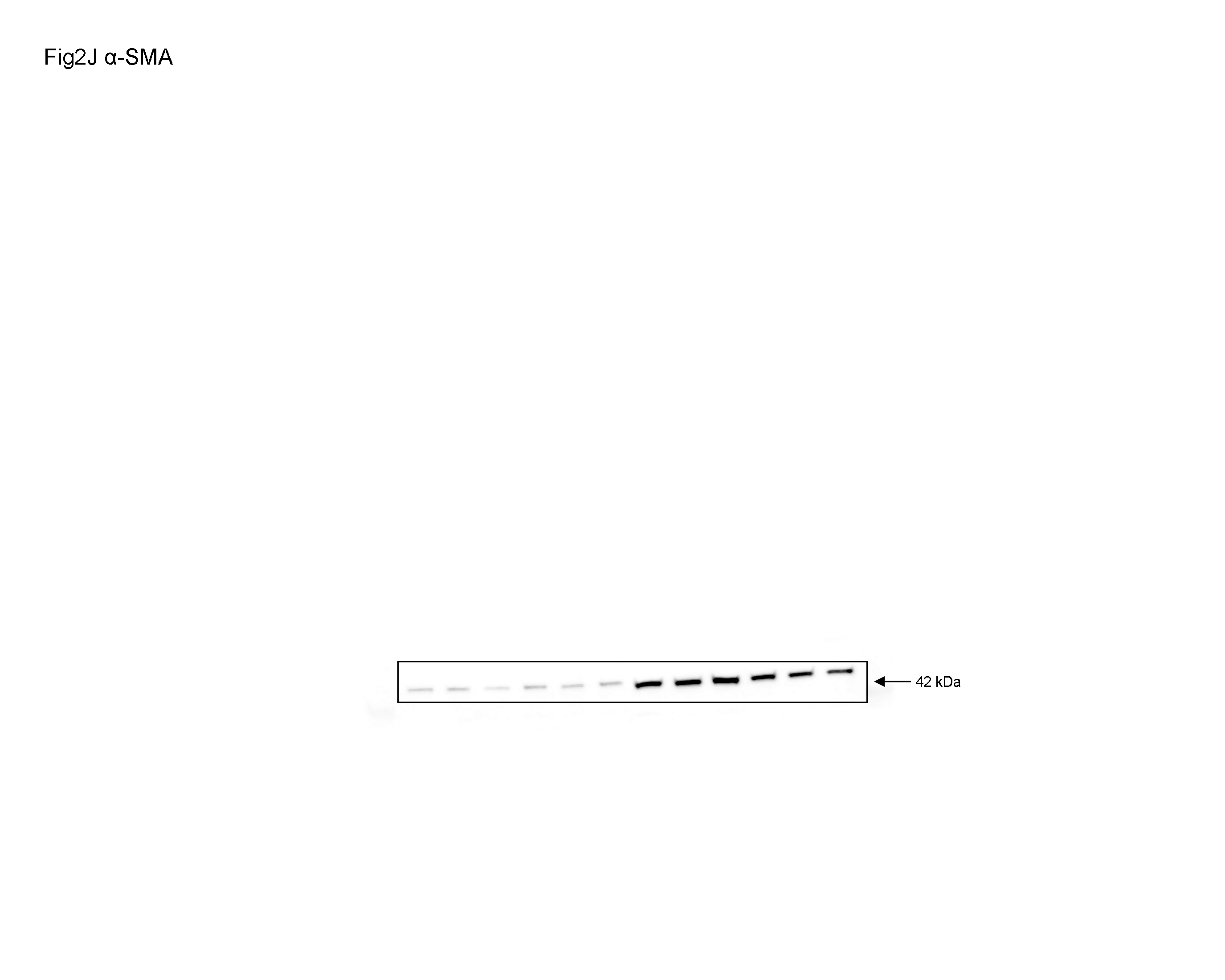

Supplement: Figure 2—source data 2. [file elife-98524-fig2-data2.zip › Fig 2-data2-v1/2J/α-SMA.tif]

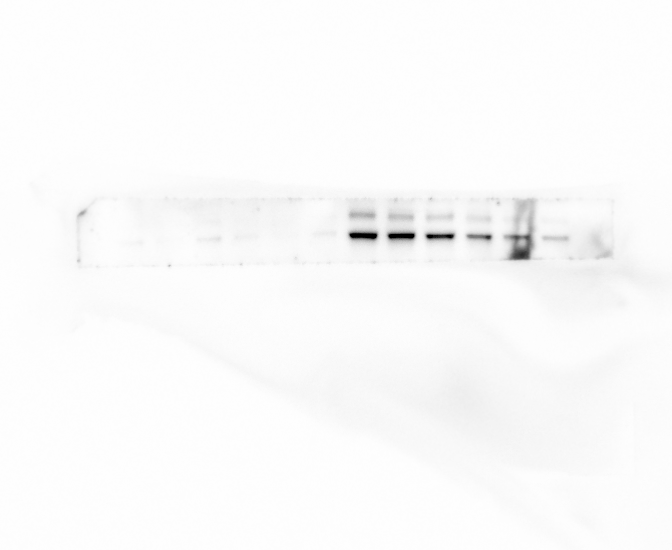

Supplement: Figure 2—figure supplement 1—source data 1. [file elife-98524-fig2-figsupp1-data1.zip › Fig 2-fig S1-data1-v1/Fig S1/S1A/CCN2.tif]

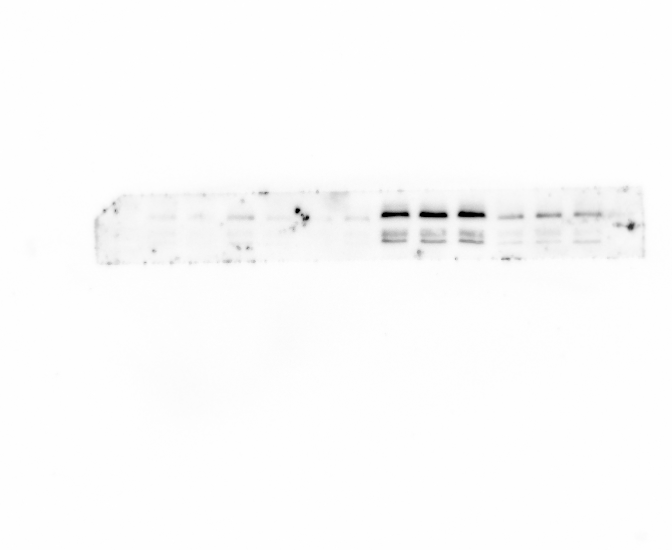

Supplement: Figure 2—figure supplement 1—source data 1. [file elife-98524-fig2-figsupp1-data1.zip › Fig 2-fig S1-data1-v1/Fig S1/S1A/COL1A1.tif]

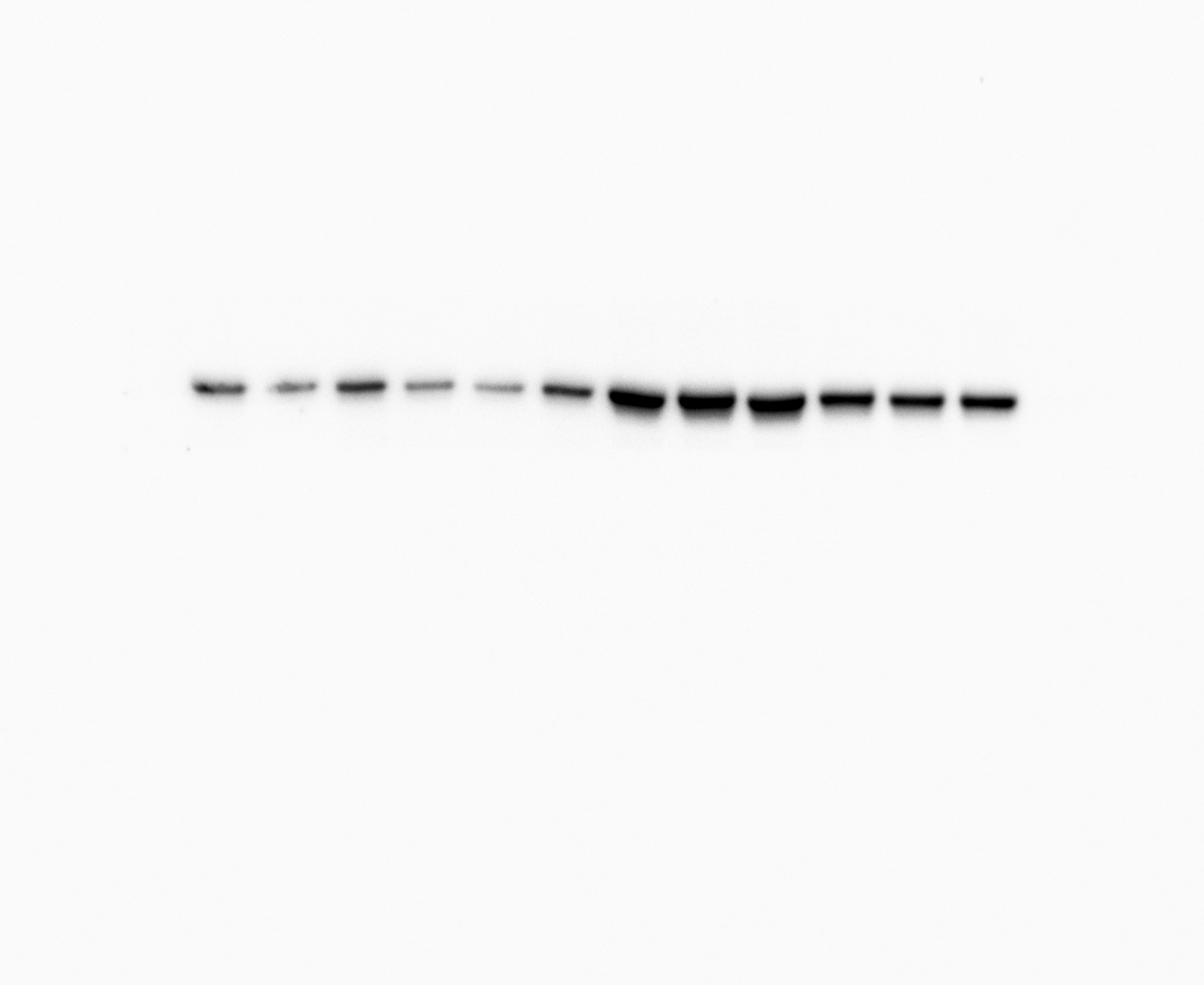

Supplement: Figure 2—figure supplement 1—source data 1. [file elife-98524-fig2-figsupp1-data1.zip › Fig 2-fig S1-data1-v1/Fig S1/S1A/COL3A1.tif]

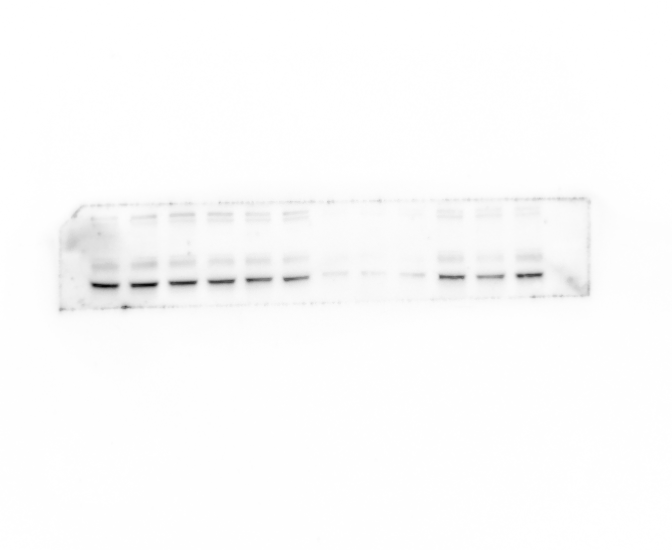

Supplement: Figure 2—figure supplement 1—source data 1. [file elife-98524-fig2-figsupp1-data1.zip › Fig 2-fig S1-data1-v1/Fig S1/S1A/E-cadherin.tif]

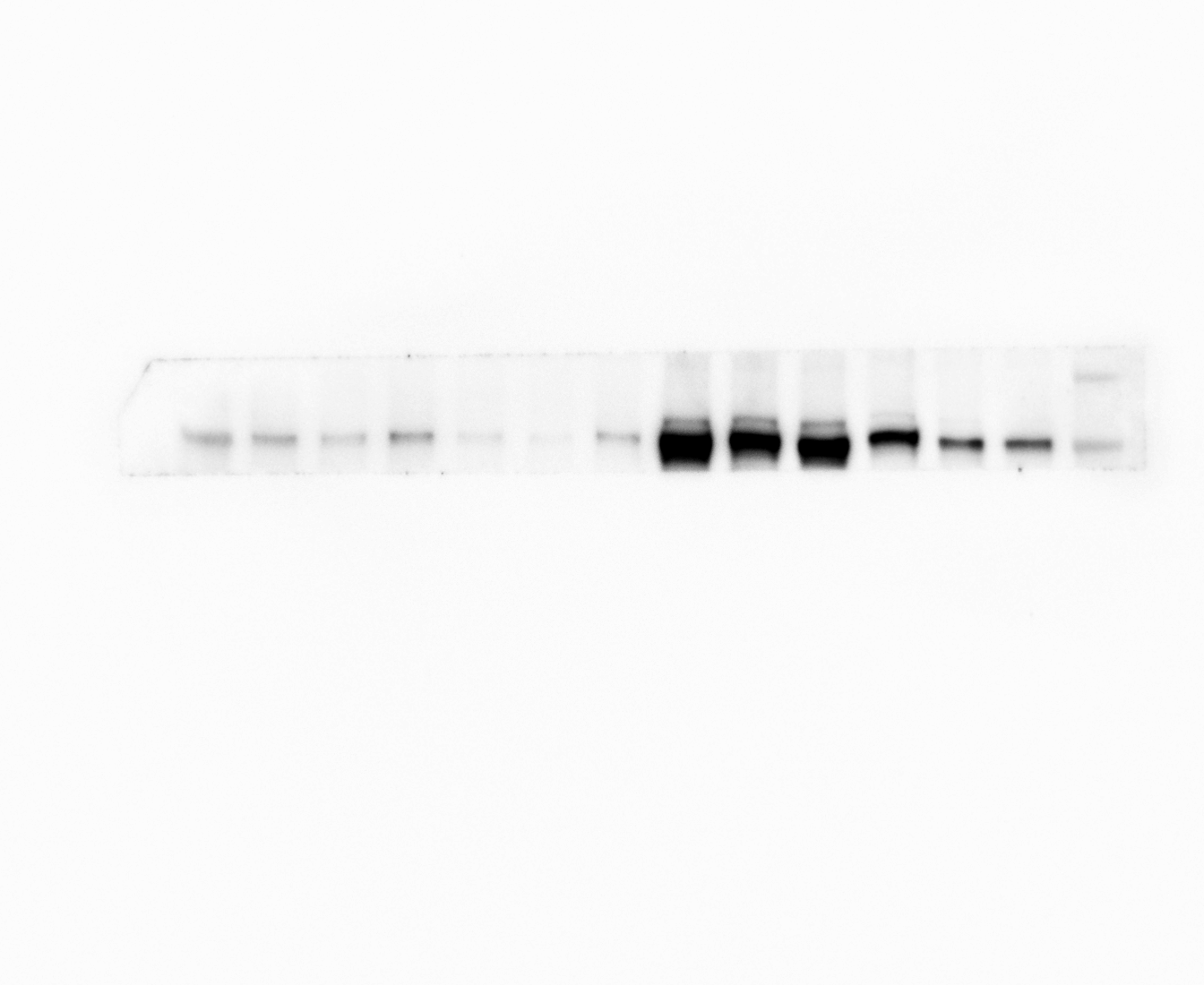

Supplement: Figure 2—figure supplement 1—source data 1. [file elife-98524-fig2-figsupp1-data1.zip › Fig 2-fig S1-data1-v1/Fig S1/S1A/FN1.tif]

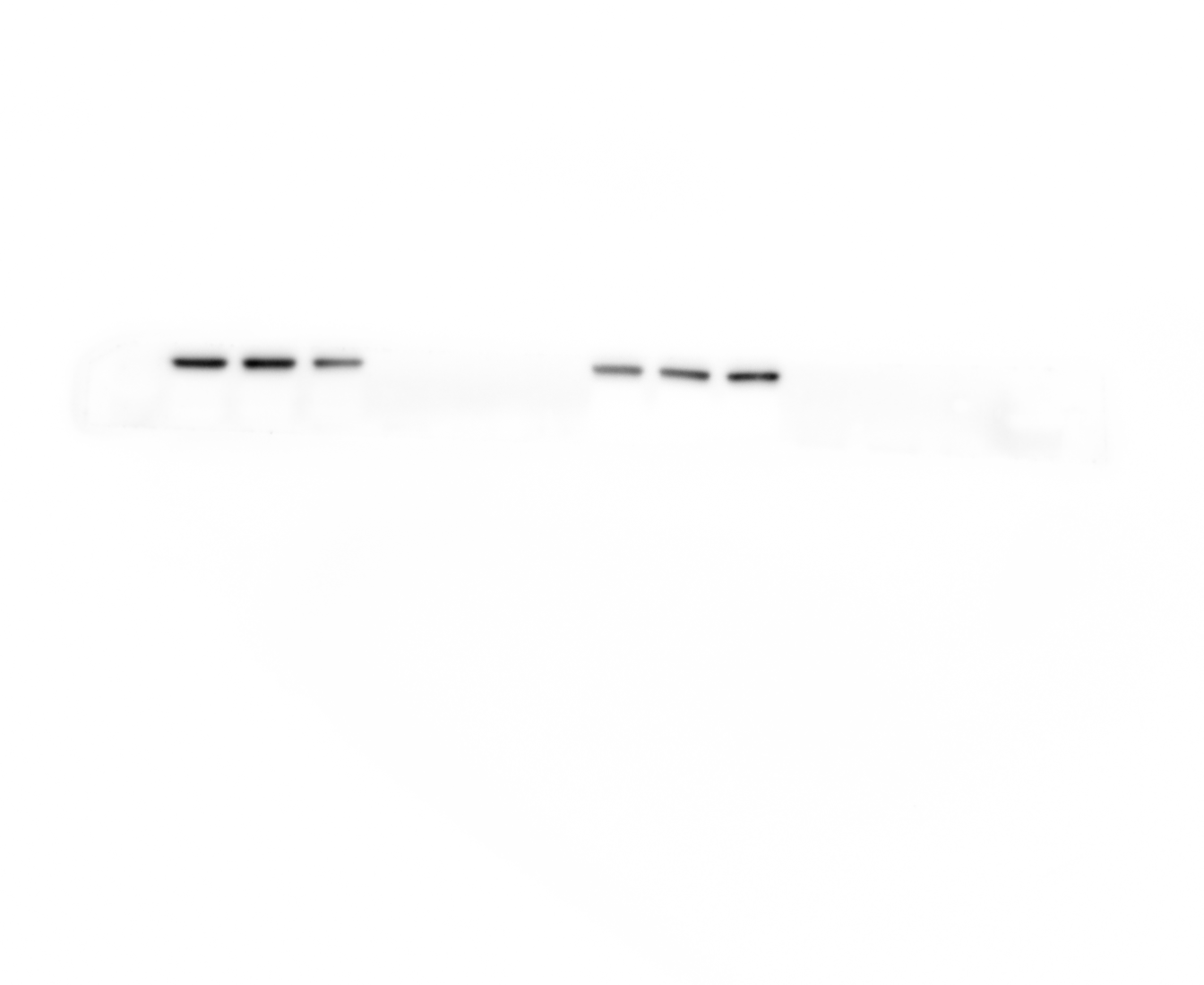

Supplement: Figure 2—figure supplement 1—source data 1. [file elife-98524-fig2-figsupp1-data1.zip › Fig 2-fig S1-data1-v1/Fig S1/S1A/SIRT4.tif]

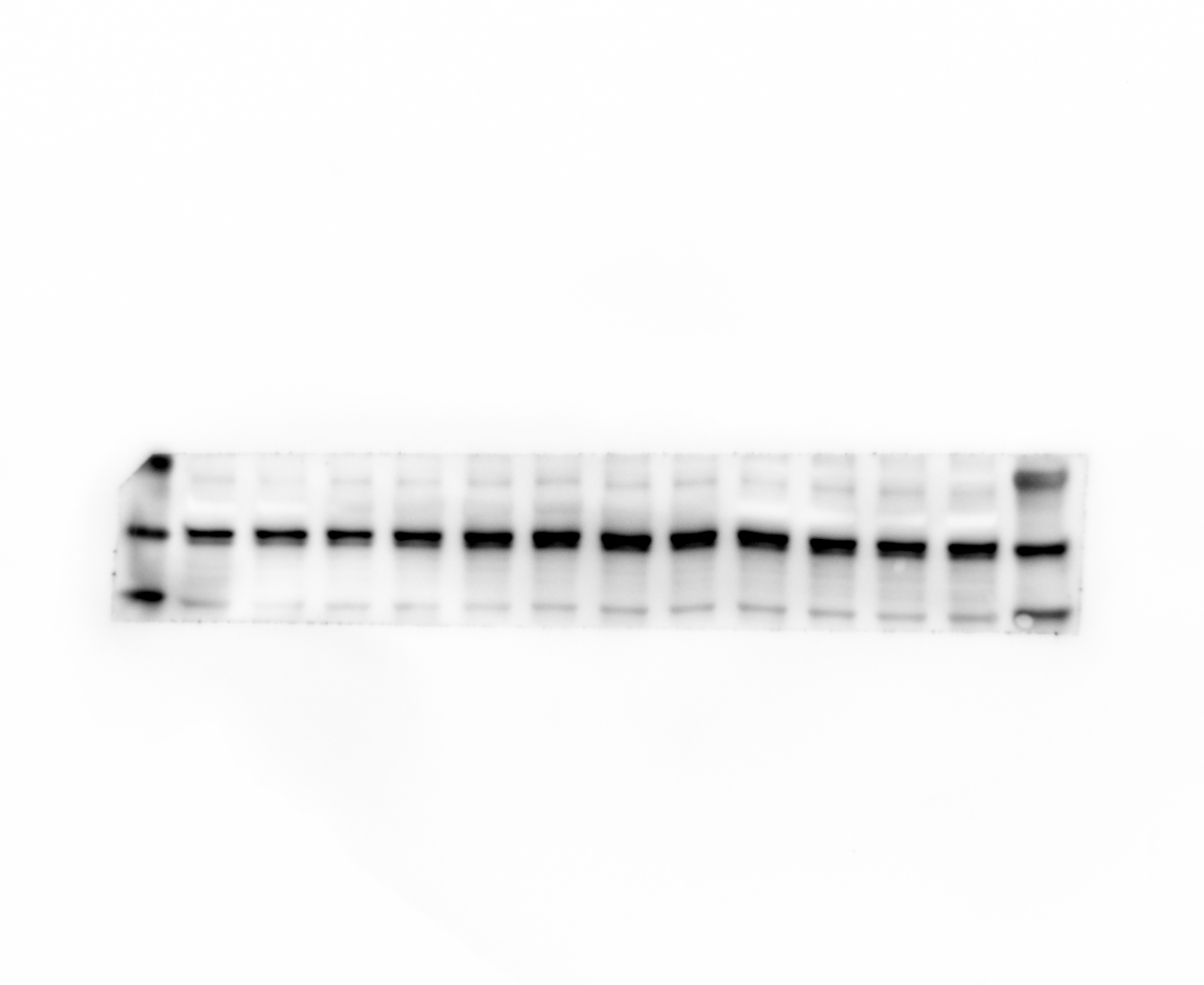

Supplement: Figure 2—figure supplement 1—source data 1. [file elife-98524-fig2-figsupp1-data1.zip › Fig 2-fig S1-data1-v1/Fig S1/S1A/Tubulin.tif]

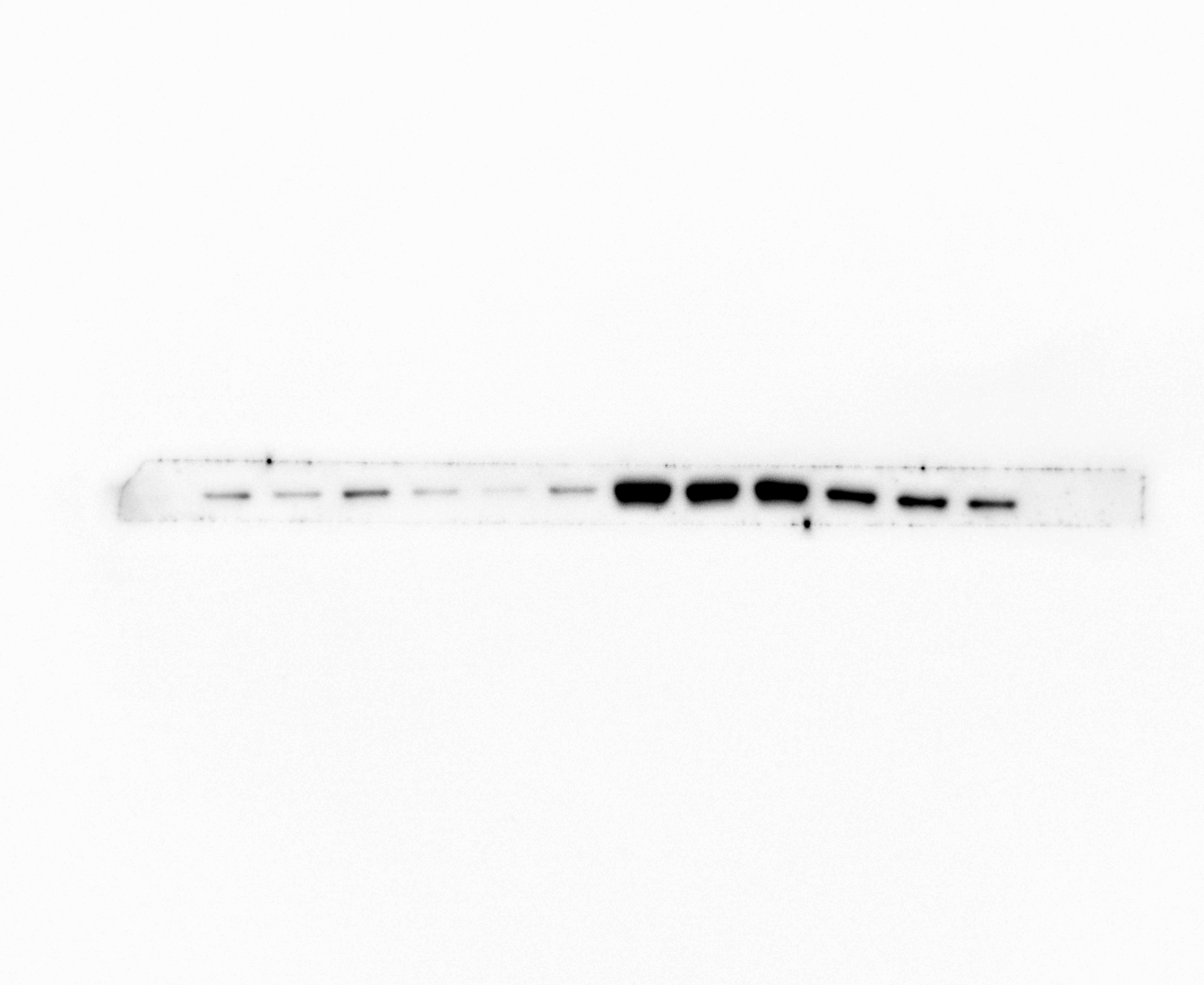

Supplement: Figure 2—figure supplement 1—source data 1. [file elife-98524-fig2-figsupp1-data1.zip › Fig 2-fig S1-data1-v1/Fig S1/S1A/α-SMA.tif]

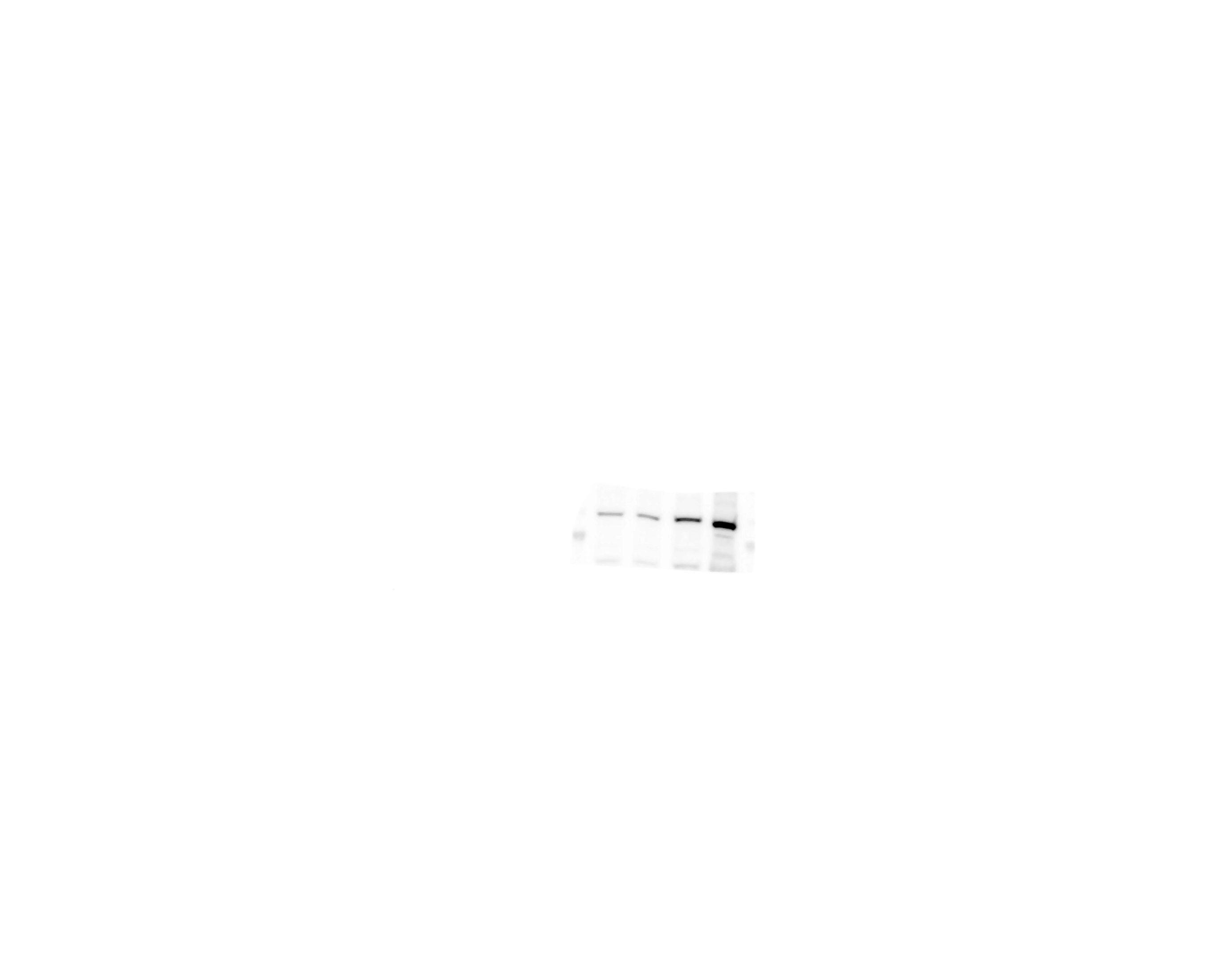

Supplement: Figure 2—figure supplement 1—source data 1. [file elife-98524-fig2-figsupp1-data1.zip › Fig 2-fig S1-data1-v1/Fig S1/S1E/SIRT4.tiff]

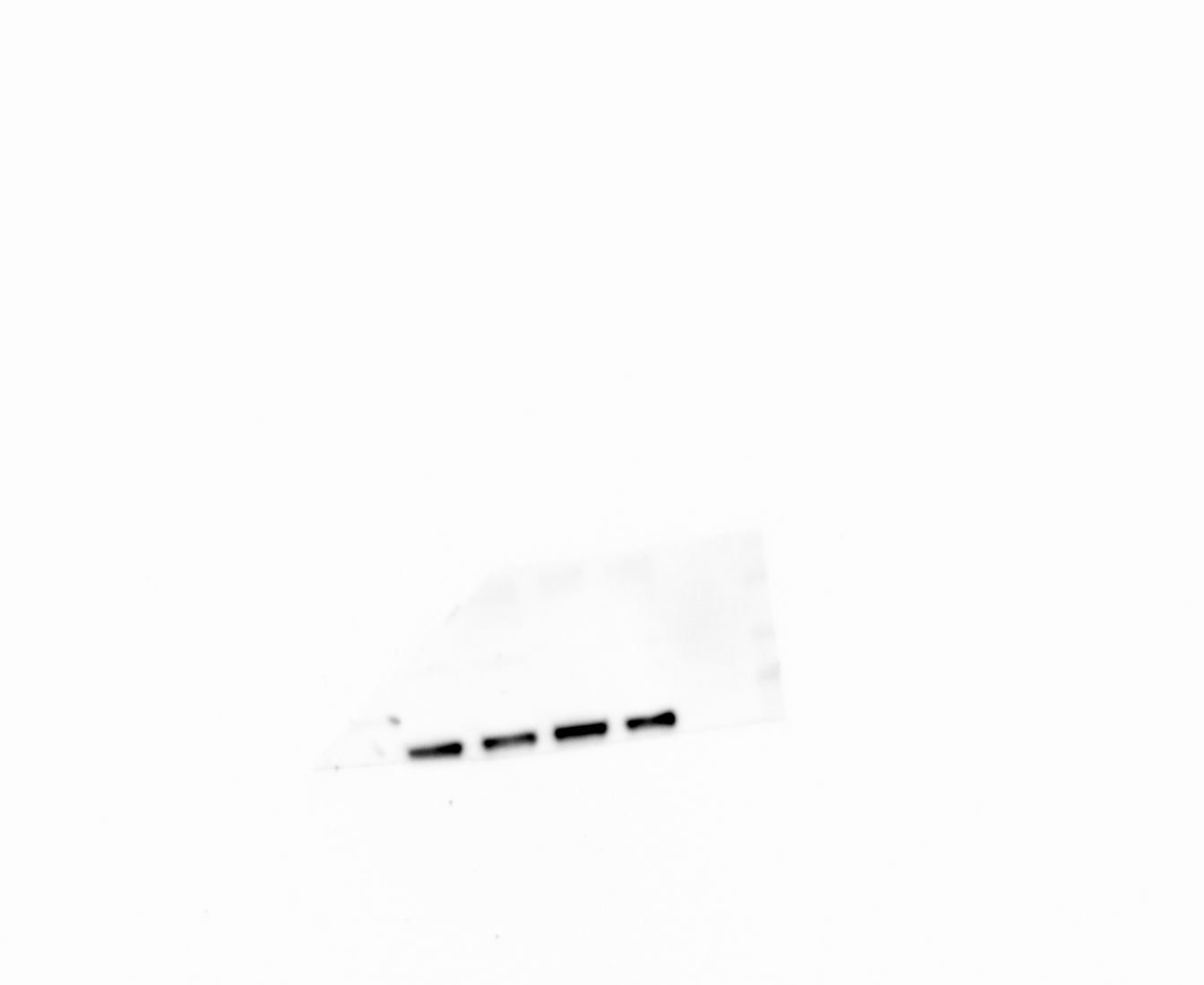

Supplement: Figure 2—figure supplement 1—source data 1. [file elife-98524-fig2-figsupp1-data1.zip › Fig 2-fig S1-data1-v1/Fig S1/S1E/Tubulin.tif]

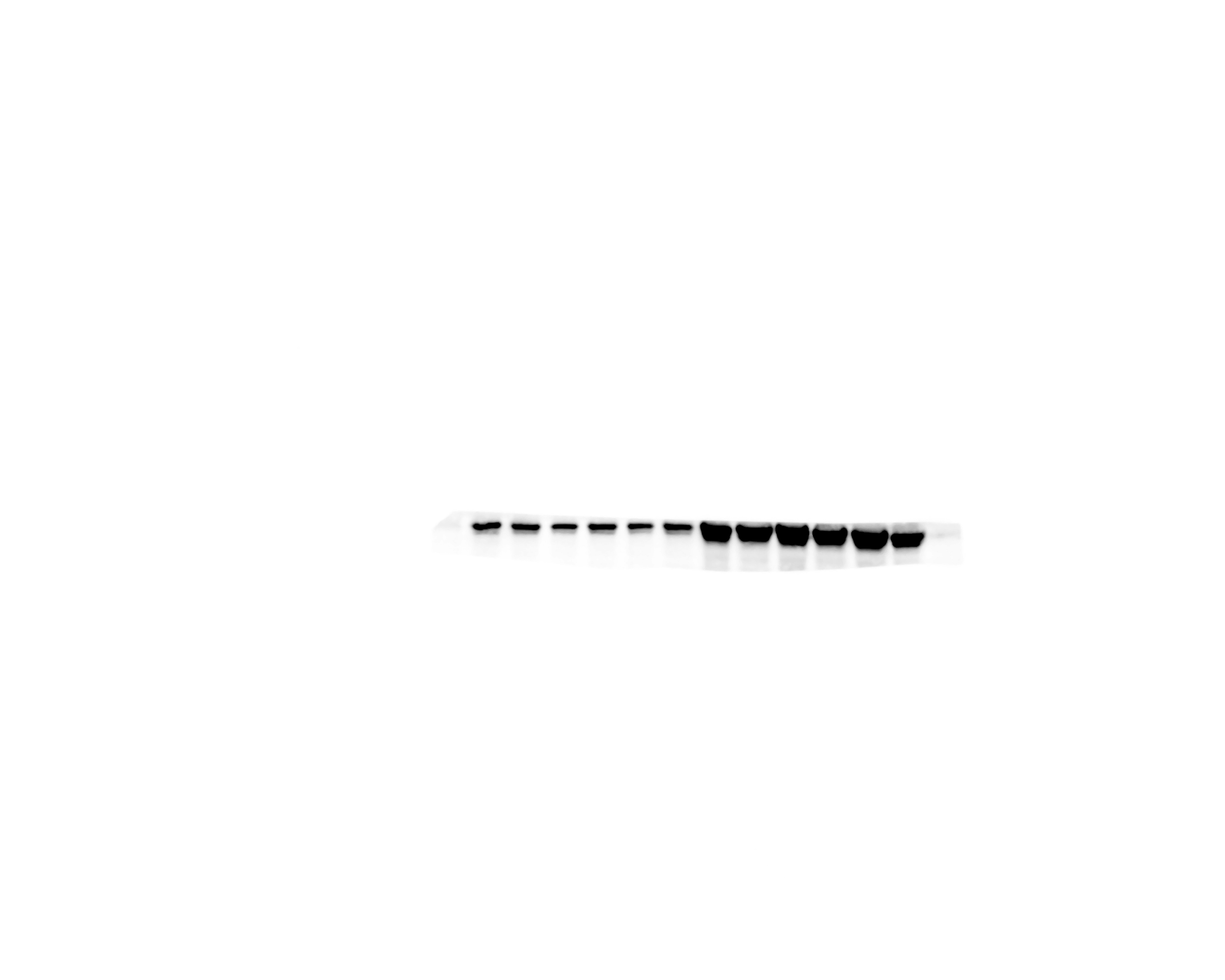

Supplement: Figure 2—figure supplement 1—source data 1. [file elife-98524-fig2-figsupp1-data1.zip › Fig 2-fig S1-data1-v1/Fig S1/S1F/bottom/CCN2.tiff]

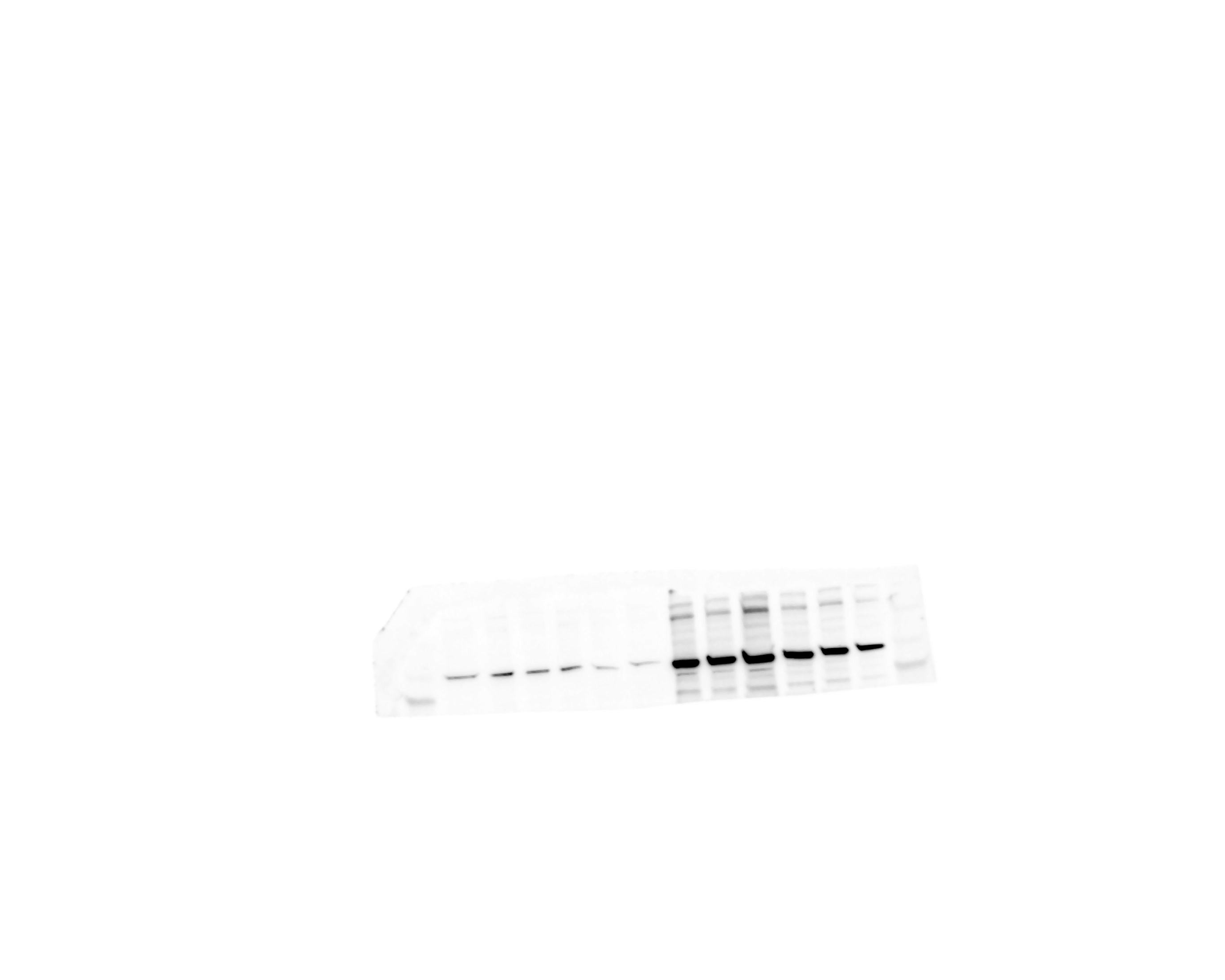

Supplement: Figure 2—figure supplement 1—source data 1. [file elife-98524-fig2-figsupp1-data1.zip › Fig 2-fig S1-data1-v1/Fig S1/S1F/bottom/COL1A1.tiff]

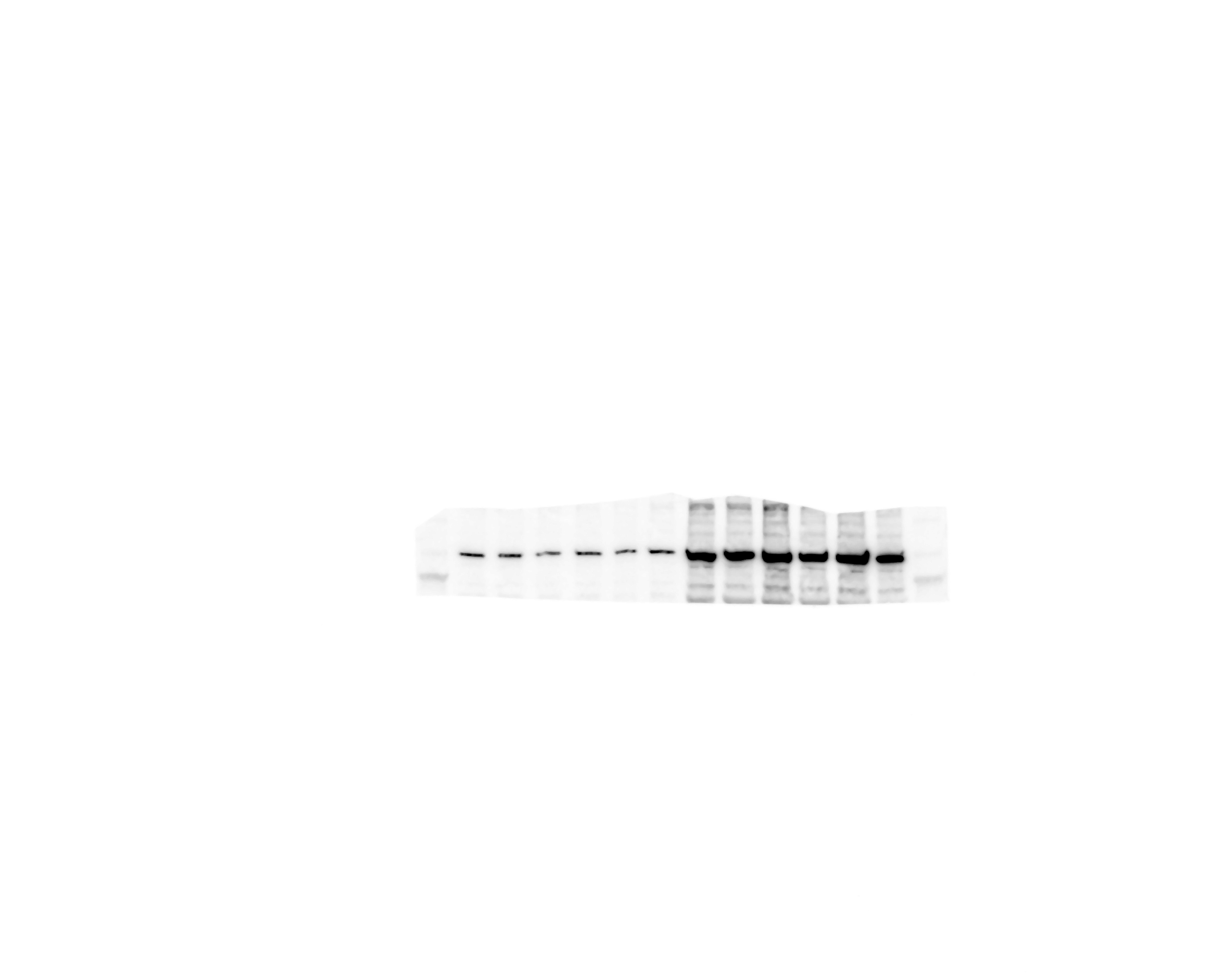

Supplement: Figure 2—figure supplement 1—source data 1. [file elife-98524-fig2-figsupp1-data1.zip › Fig 2-fig S1-data1-v1/Fig S1/S1F/bottom/COL3A1.tiff]

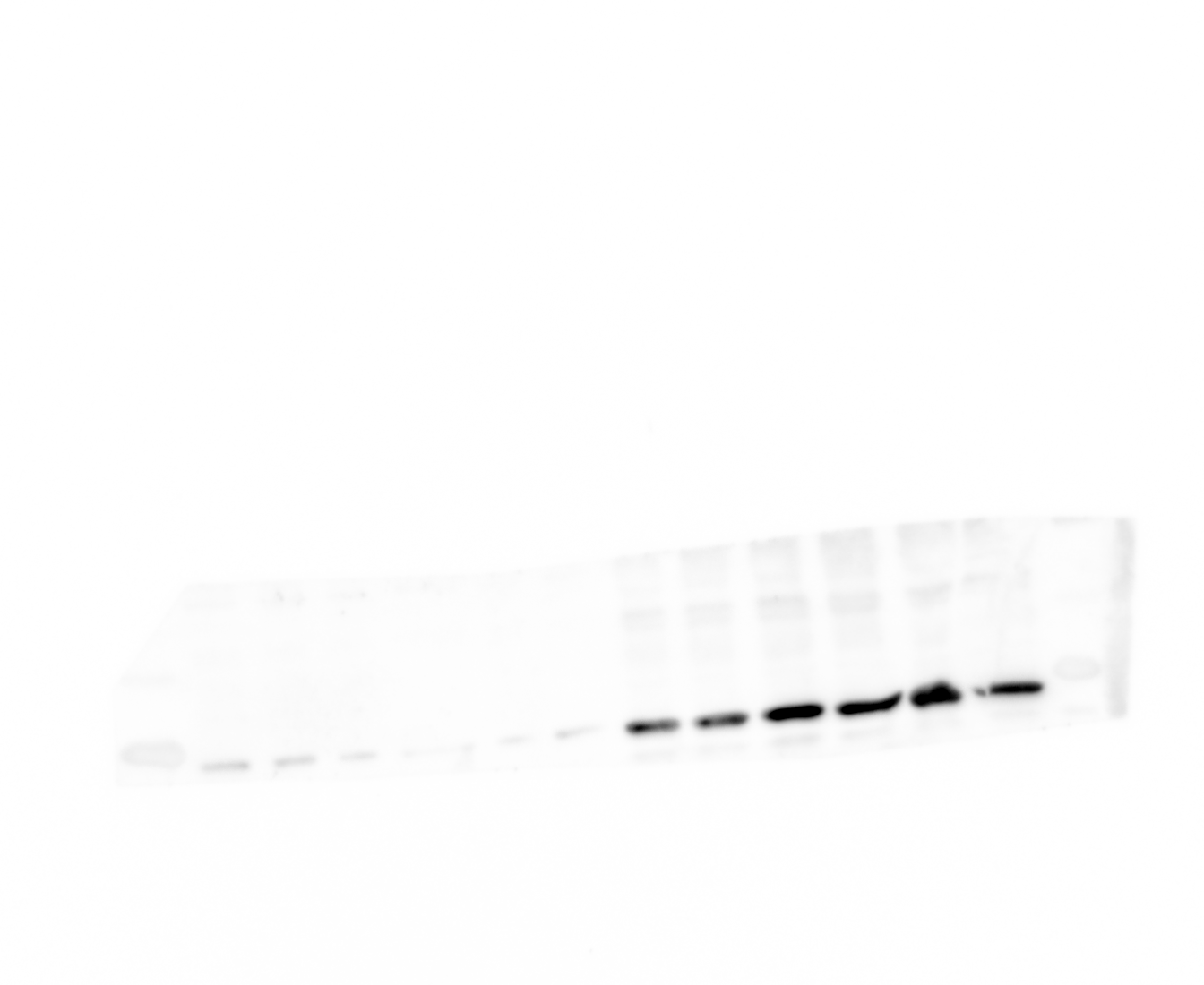

Supplement: Figure 2—figure supplement 1—source data 1. [file elife-98524-fig2-figsupp1-data1.zip › Fig 2-fig S1-data1-v1/Fig S1/S1F/bottom/FN1.tif]

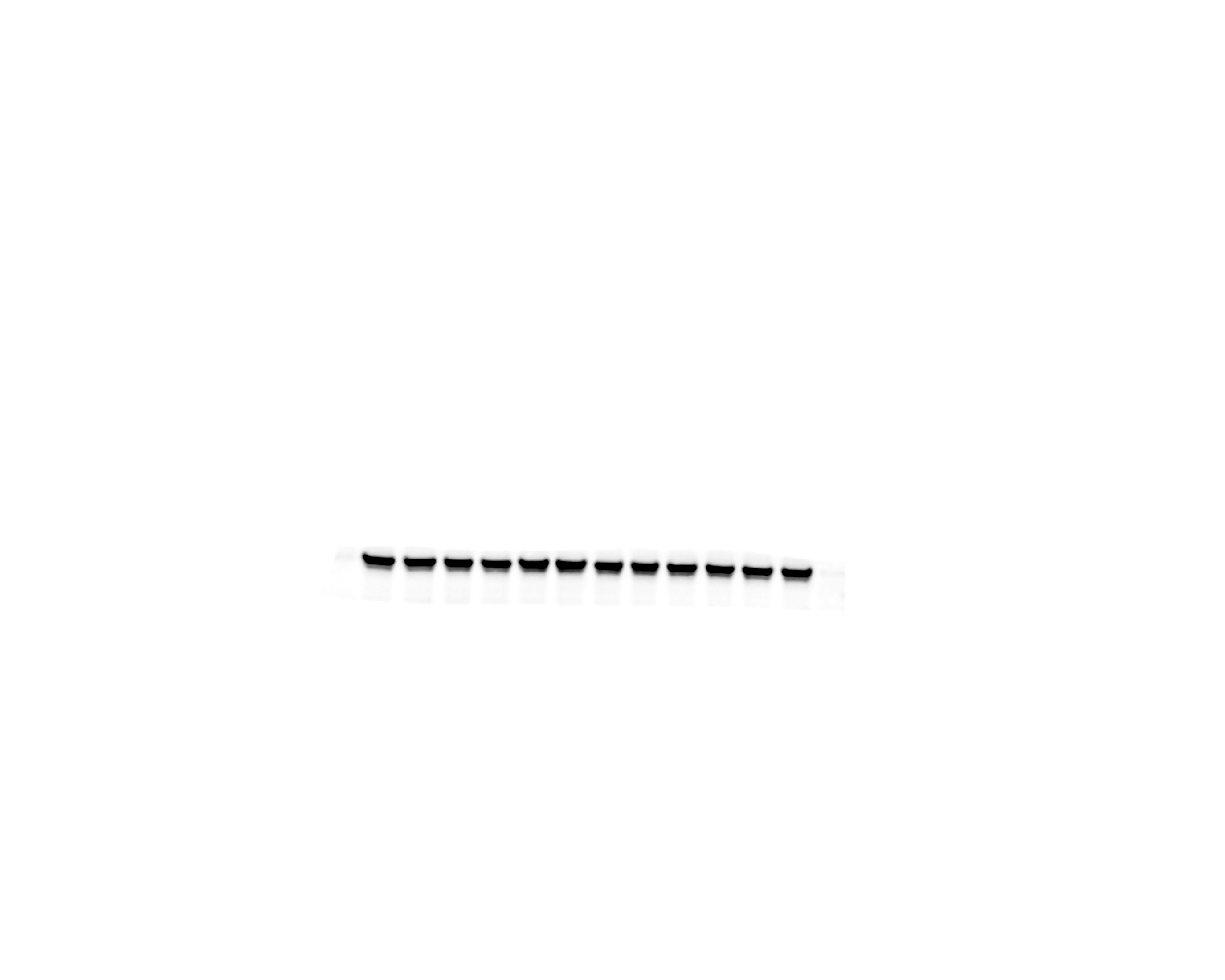

Supplement: Figure 2—figure supplement 1—source data 1. [file elife-98524-fig2-figsupp1-data1.zip › Fig 2-fig S1-data1-v1/Fig S1/S1F/bottom/Tubulin.tiff]

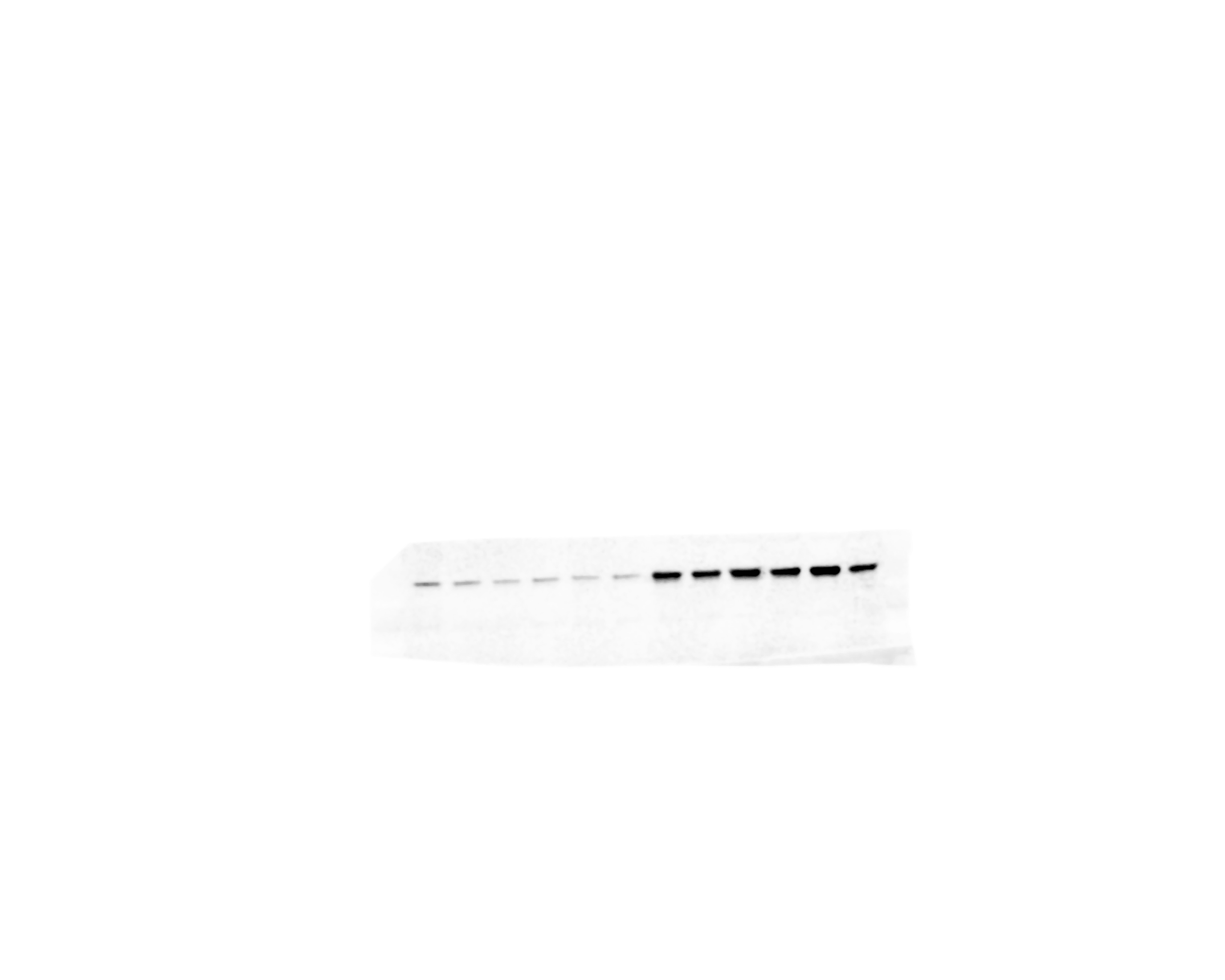

Supplement: Figure 2—figure supplement 1—source data 1. [file elife-98524-fig2-figsupp1-data1.zip › Fig 2-fig S1-data1-v1/Fig S1/S1F/bottom/α-SMA.tiff]

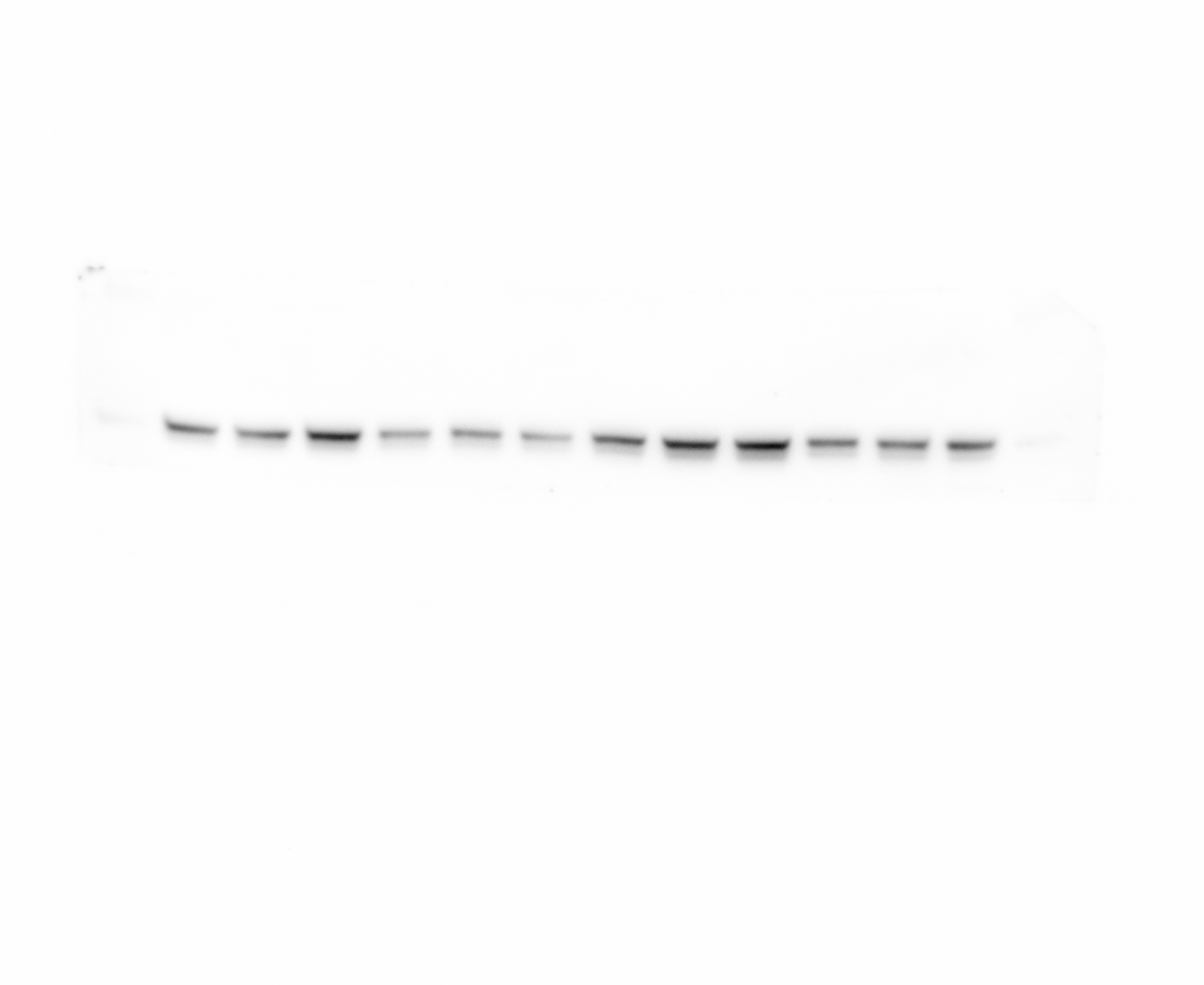

Supplement: Figure 2—figure supplement 1—source data 1. [file elife-98524-fig2-figsupp1-data1.zip › Fig 2-fig S1-data1-v1/Fig S1/S1F/upper/SIRT4.tif]

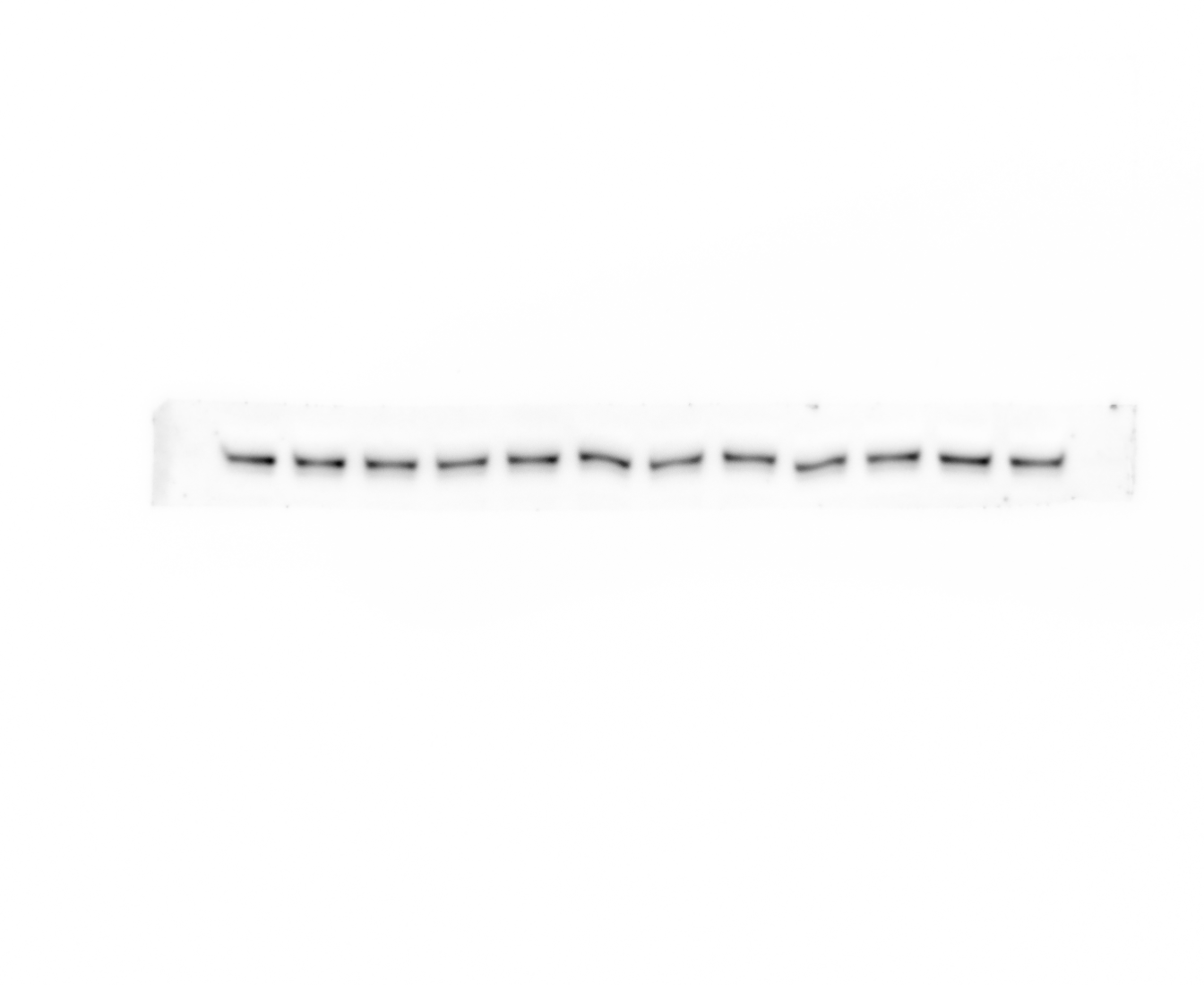

Supplement: Figure 2—figure supplement 1—source data 1. [file elife-98524-fig2-figsupp1-data1.zip › Fig 2-fig S1-data1-v1/Fig S1/S1F/upper/Tubulin.tif]

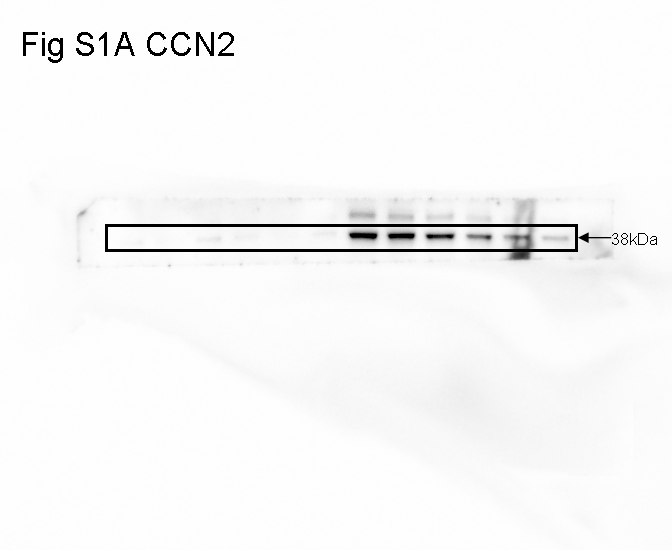

Supplement: Figure 2—figure supplement 1—source data 2. [file elife-98524-fig2-figsupp1-data2.zip › Fig 2-fig S1-data2-v1/S1A/CCN2.tif]

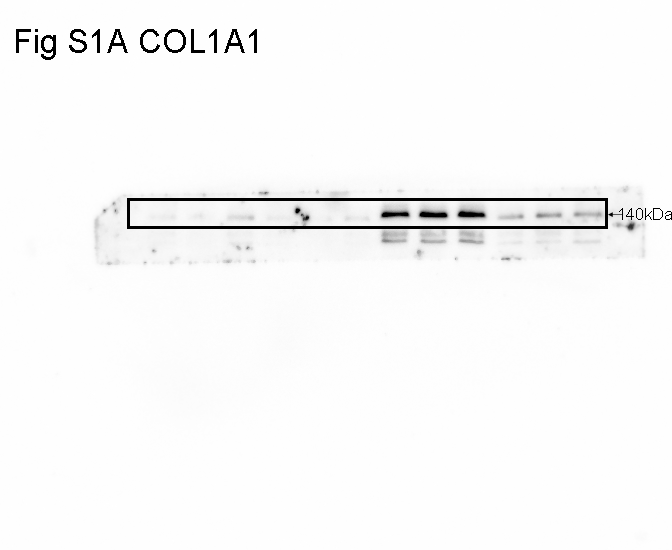

Supplement: Figure 2—figure supplement 1—source data 2. [file elife-98524-fig2-figsupp1-data2.zip › Fig 2-fig S1-data2-v1/S1A/COL1A1.tif]

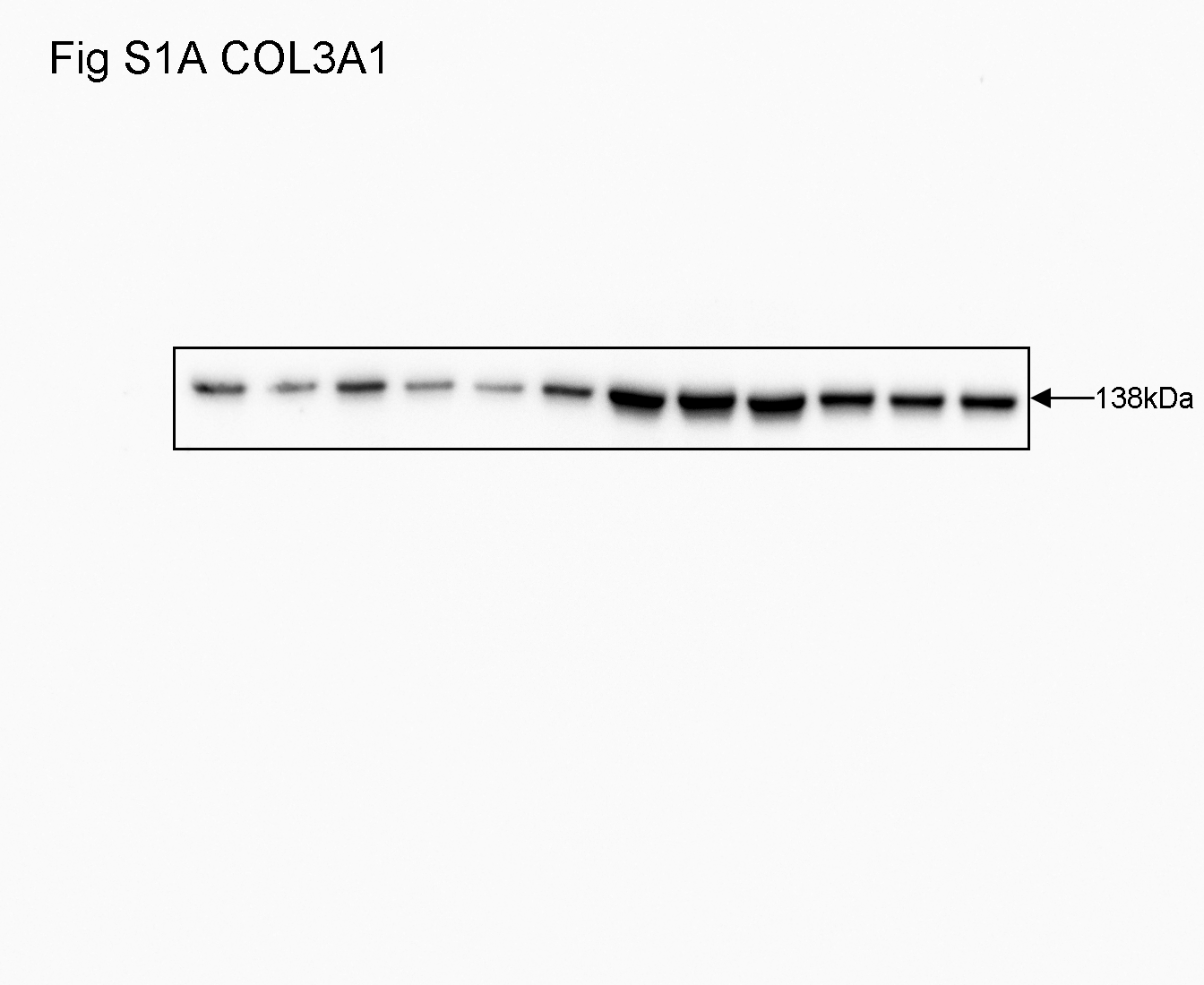

Supplement: Figure 2—figure supplement 1—source data 2. [file elife-98524-fig2-figsupp1-data2.zip › Fig 2-fig S1-data2-v1/S1A/COL3A1.tif]

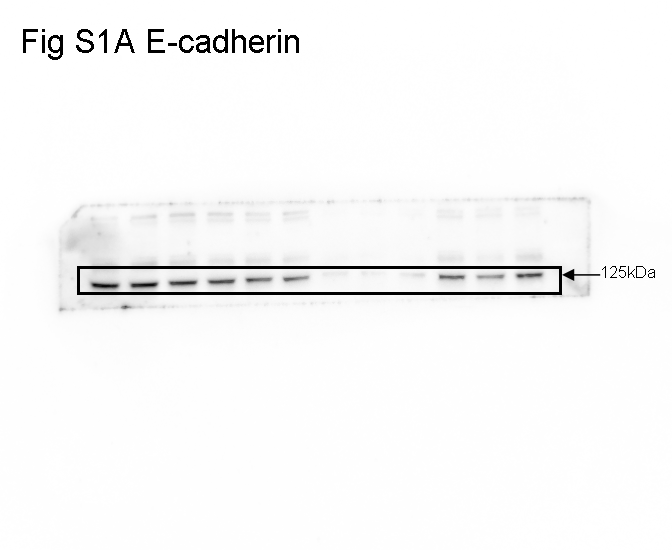

Supplement: Figure 2—figure supplement 1—source data 2. [file elife-98524-fig2-figsupp1-data2.zip › Fig 2-fig S1-data2-v1/S1A/E-cadherin.tif]

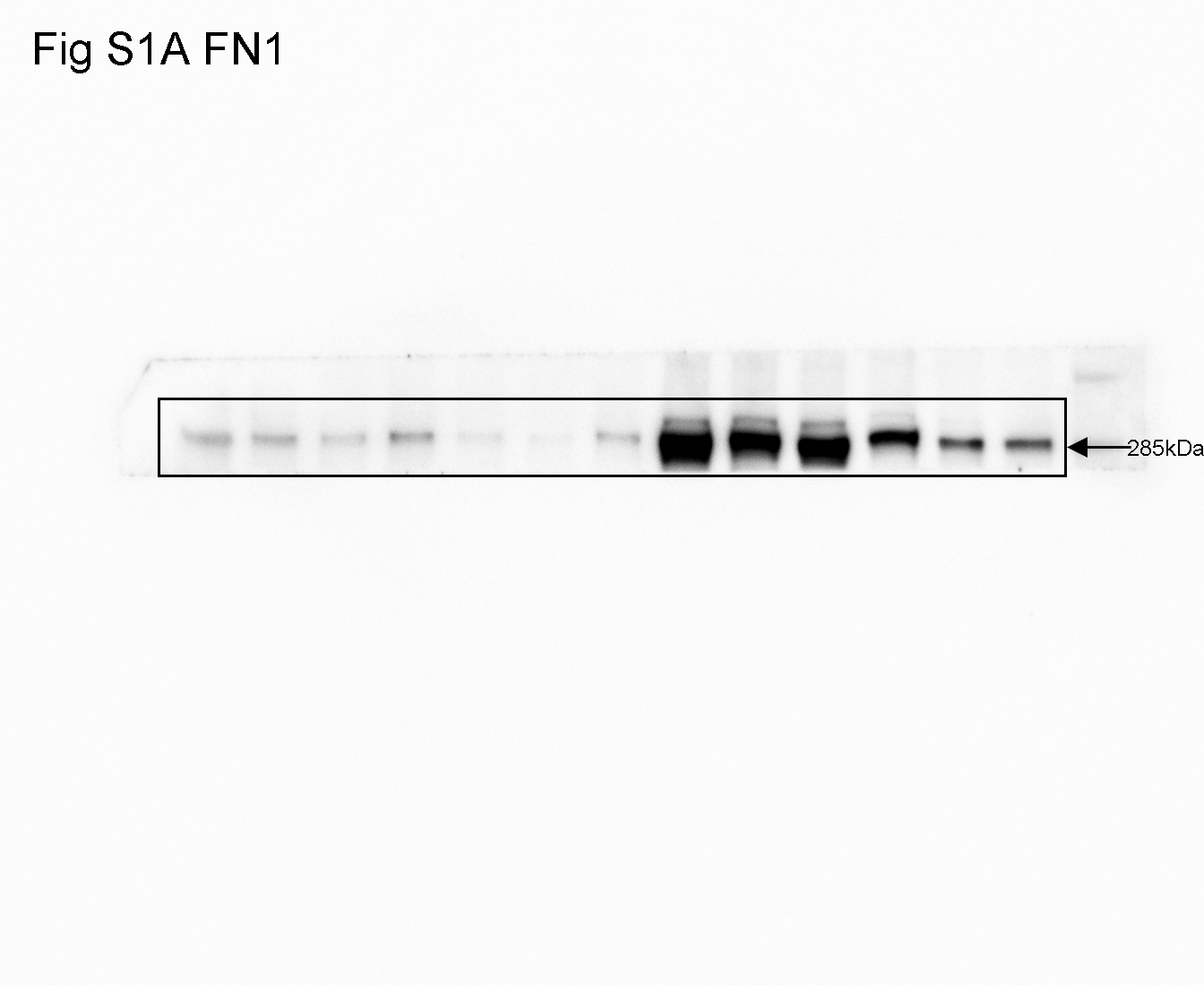

Supplement: Figure 2—figure supplement 1—source data 2. [file elife-98524-fig2-figsupp1-data2.zip › Fig 2-fig S1-data2-v1/S1A/FN1.tif]

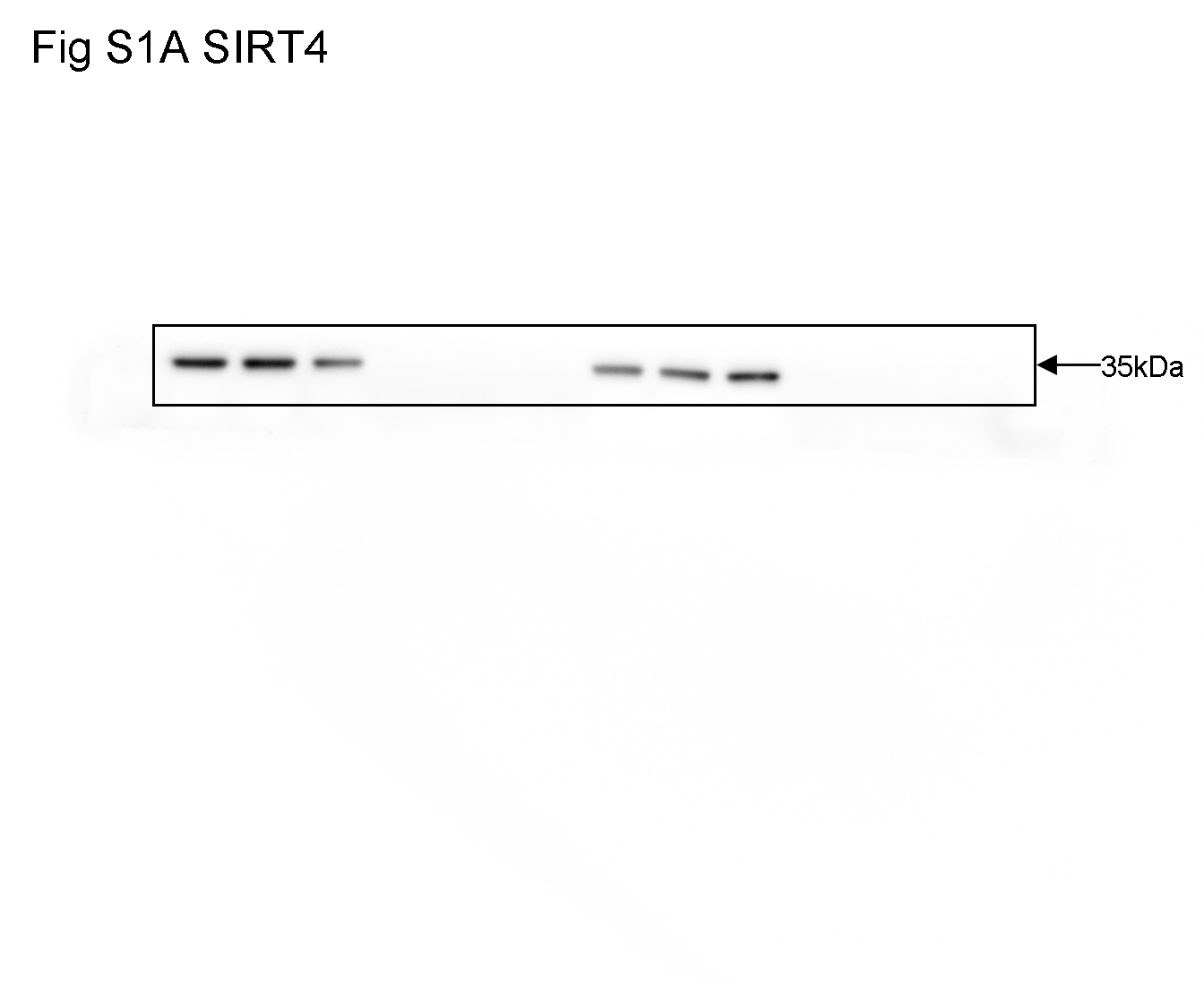

Supplement: Figure 2—figure supplement 1—source data 2. [file elife-98524-fig2-figsupp1-data2.zip › Fig 2-fig S1-data2-v1/S1A/SIRT4.tif]
