# Supplementary figures and images for: Nuclear translocation of SIRT4 mediates deacetylation of U2AF2 to modulate renal fibrosis through alternative splicing-mediated upregulation of CCN2 (part 2 of 9)
Source: eLife. 2024 Nov 4;13:RP98524. doi: 10.7554/eLife.98524 (PMC11534337; doi:10.7554/eLife.98524)

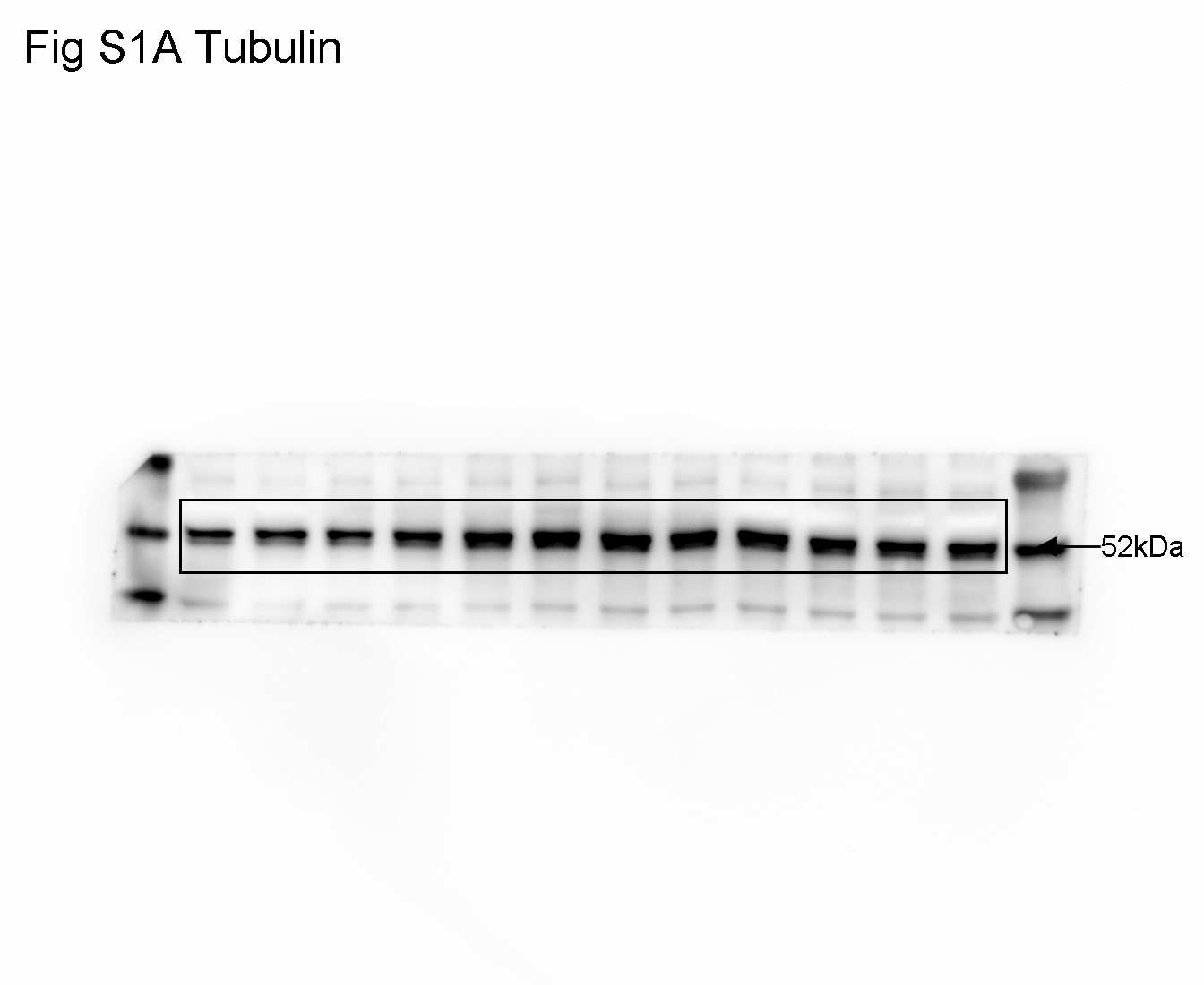

Supplement: Figure 2—figure supplement 1—source data 2. [file elife-98524-fig2-figsupp1-data2.zip › Fig 2-fig S1-data2-v1/S1A/Tubulin.tif]

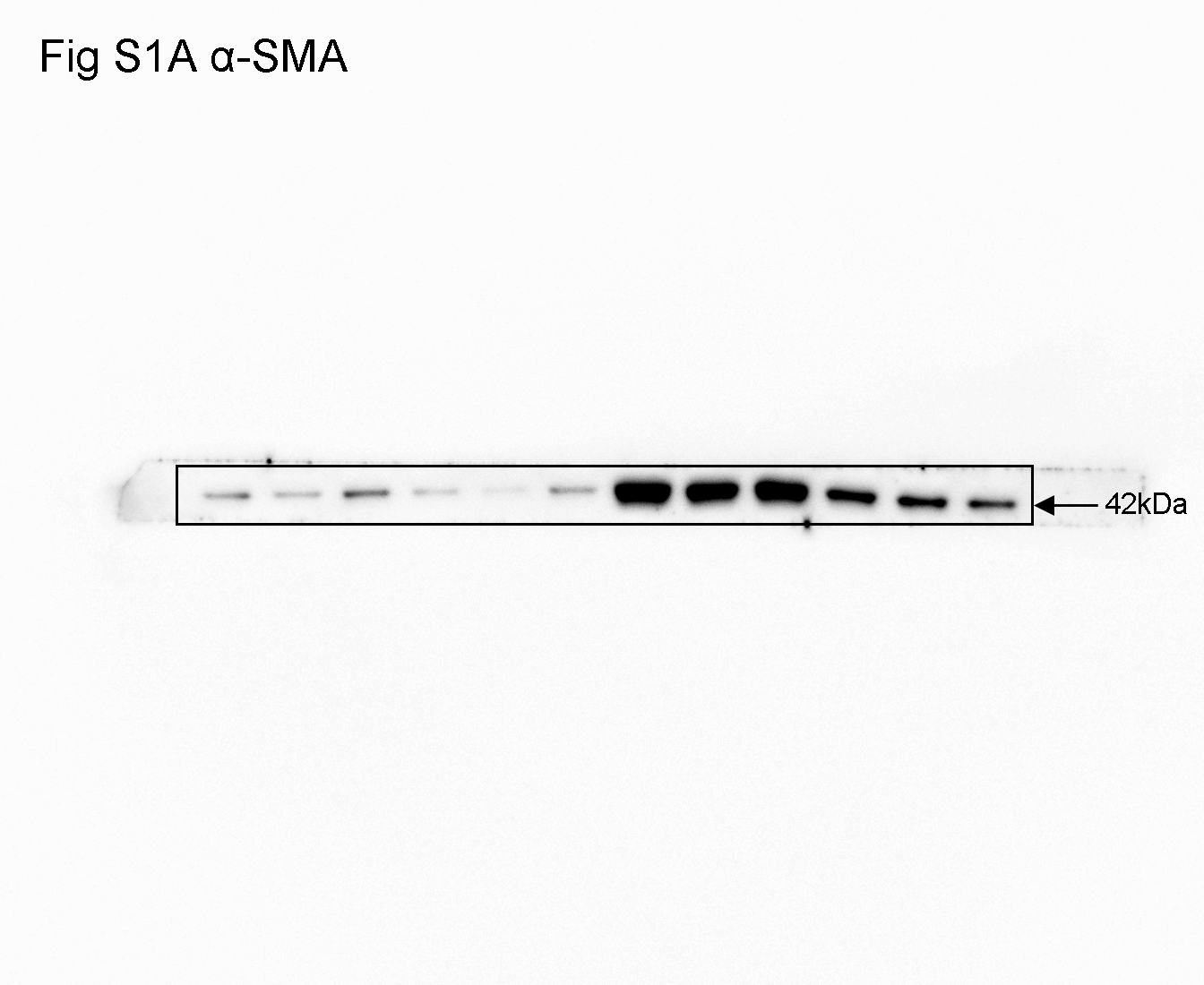

Supplement: Figure 2—figure supplement 1—source data 2. [file elife-98524-fig2-figsupp1-data2.zip › Fig 2-fig S1-data2-v1/S1A/α-SMA.tif]

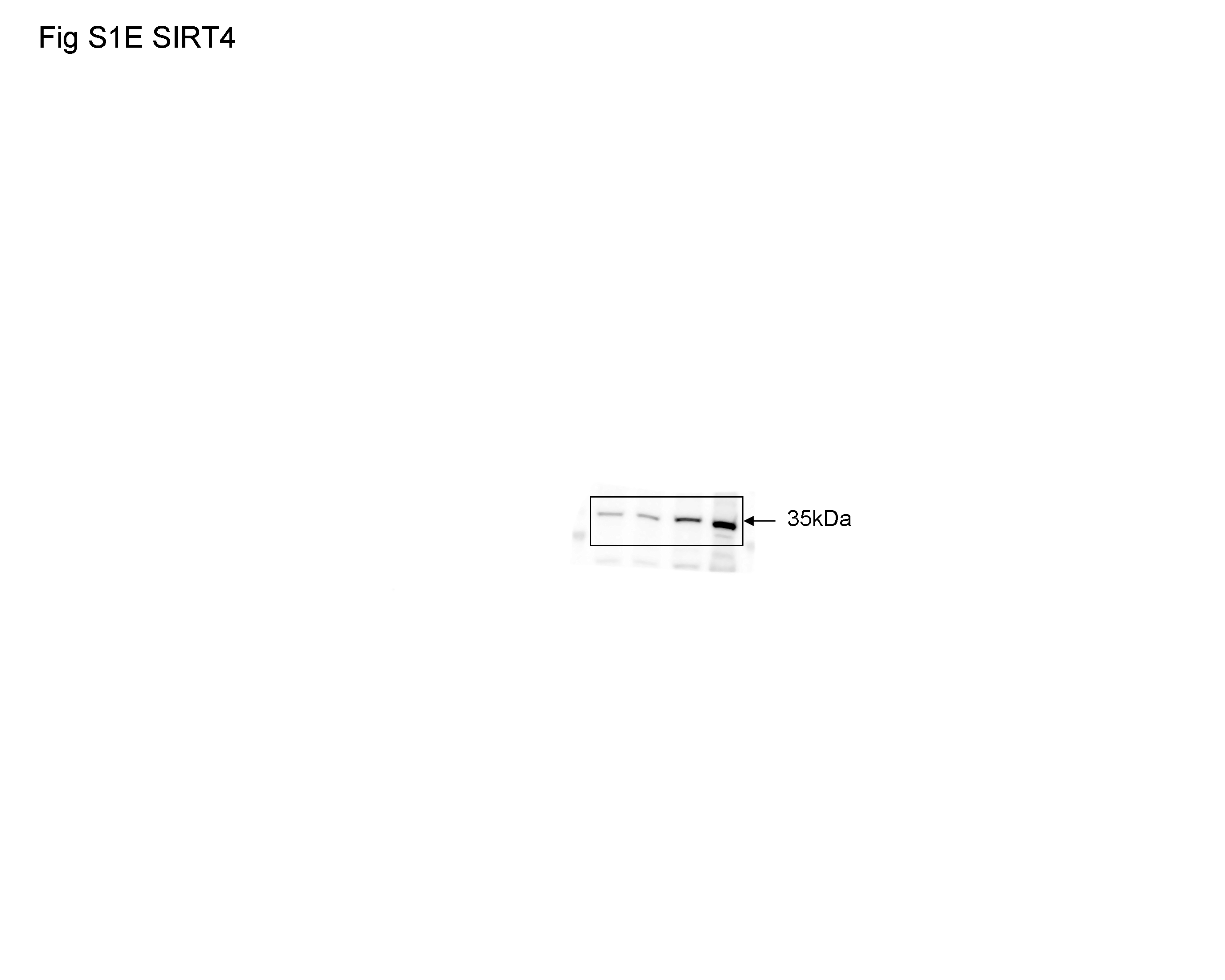

Supplement: Figure 2—figure supplement 1—source data 2. [file elife-98524-fig2-figsupp1-data2.zip › Fig 2-fig S1-data2-v1/S1E/SIRT4.tiff]

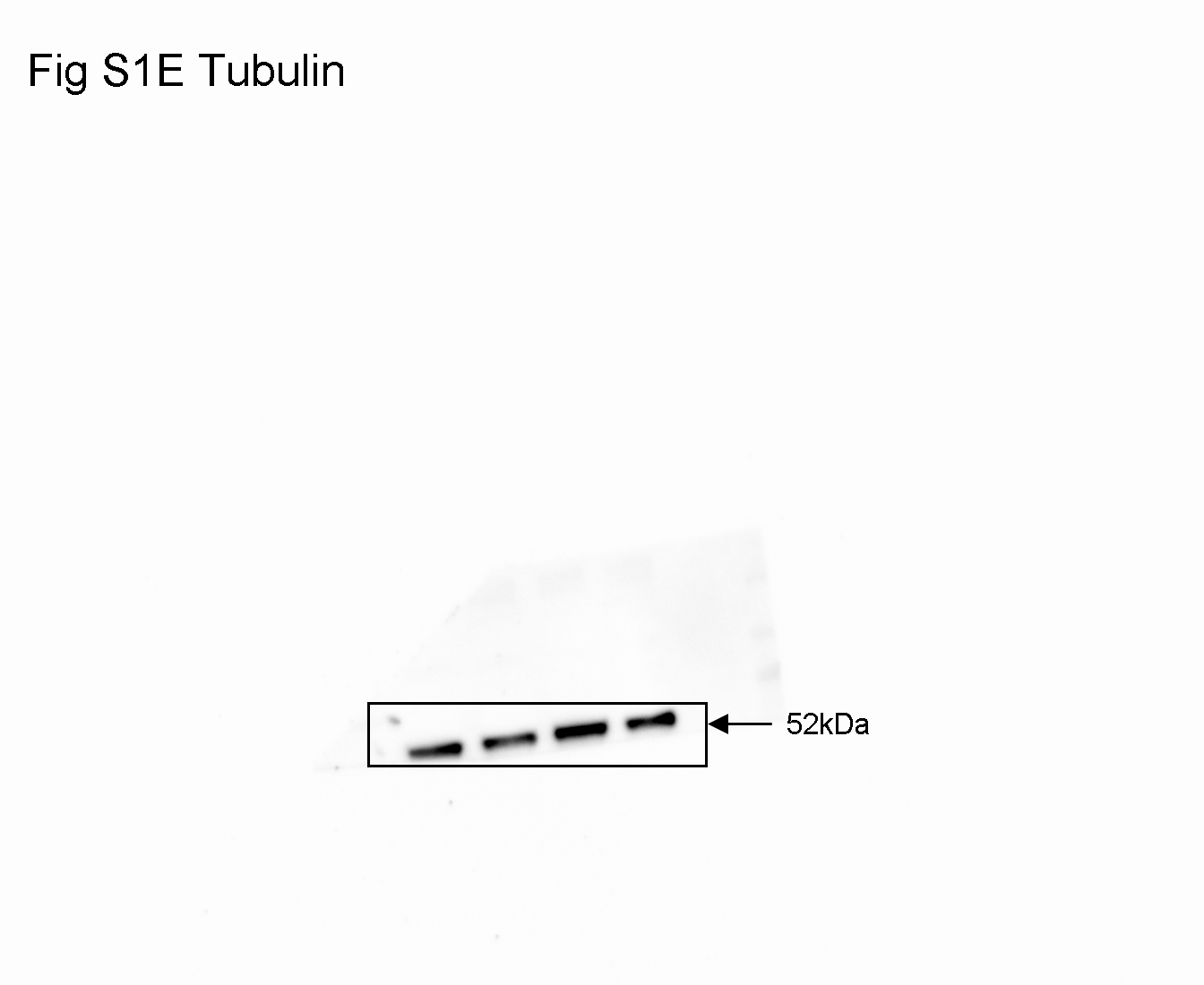

Supplement: Figure 2—figure supplement 1—source data 2. [file elife-98524-fig2-figsupp1-data2.zip › Fig 2-fig S1-data2-v1/S1E/Tubulin.tif]

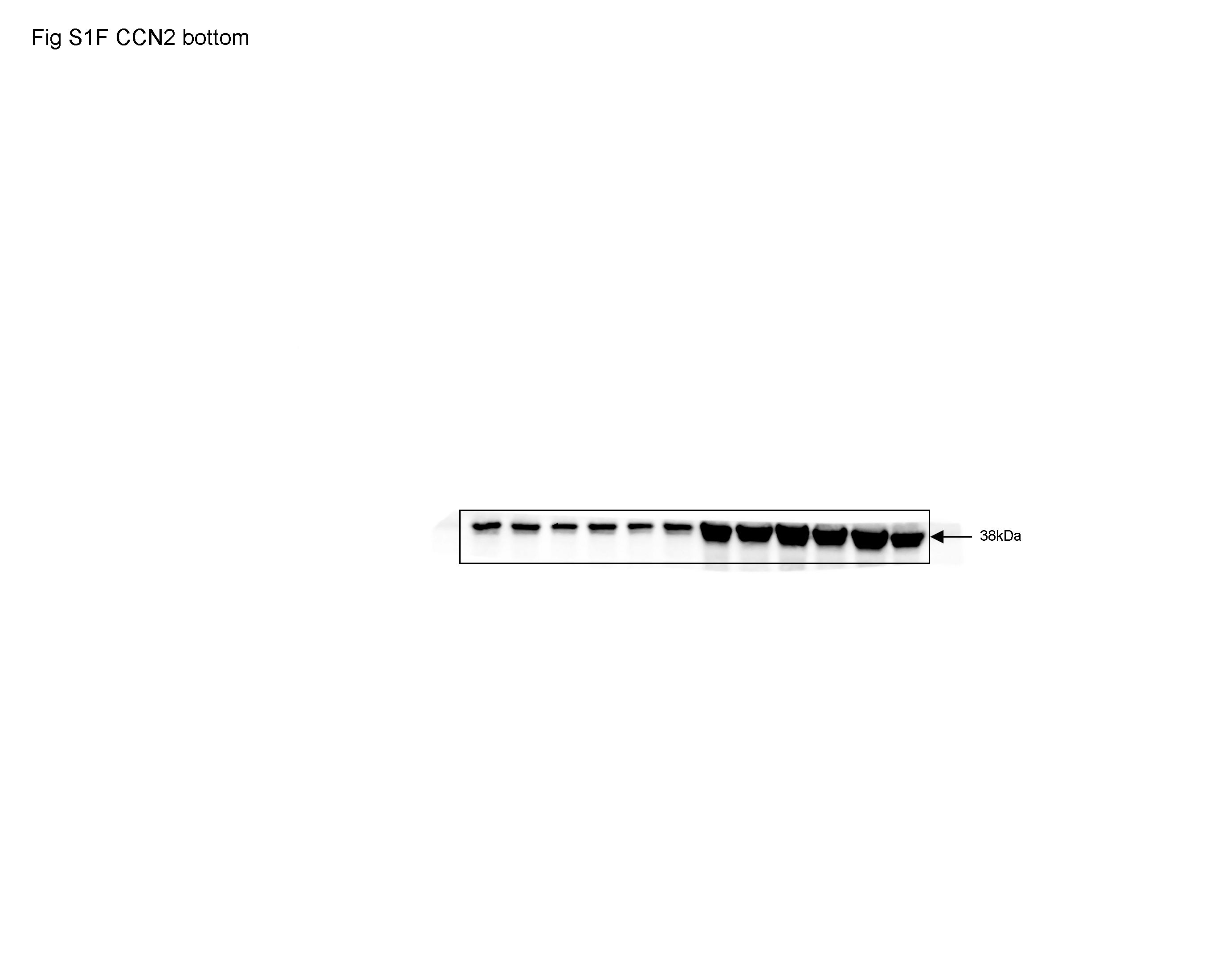

Supplement: Figure 2—figure supplement 1—source data 2. [file elife-98524-fig2-figsupp1-data2.zip › Fig 2-fig S1-data2-v1/S1F/bottom/CCN2.tiff]

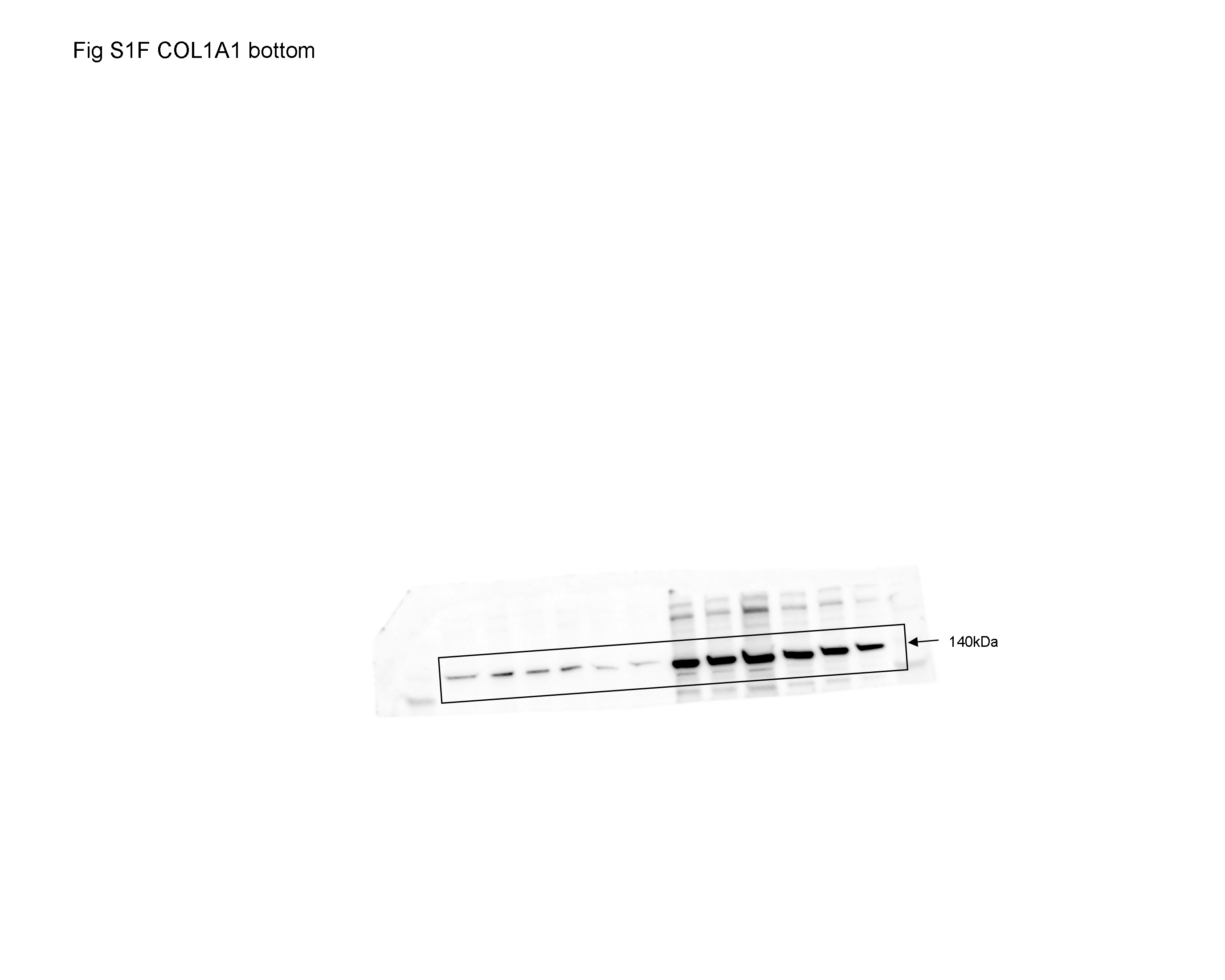

Supplement: Figure 2—figure supplement 1—source data 2. [file elife-98524-fig2-figsupp1-data2.zip › Fig 2-fig S1-data2-v1/S1F/bottom/COL1A1.tiff]

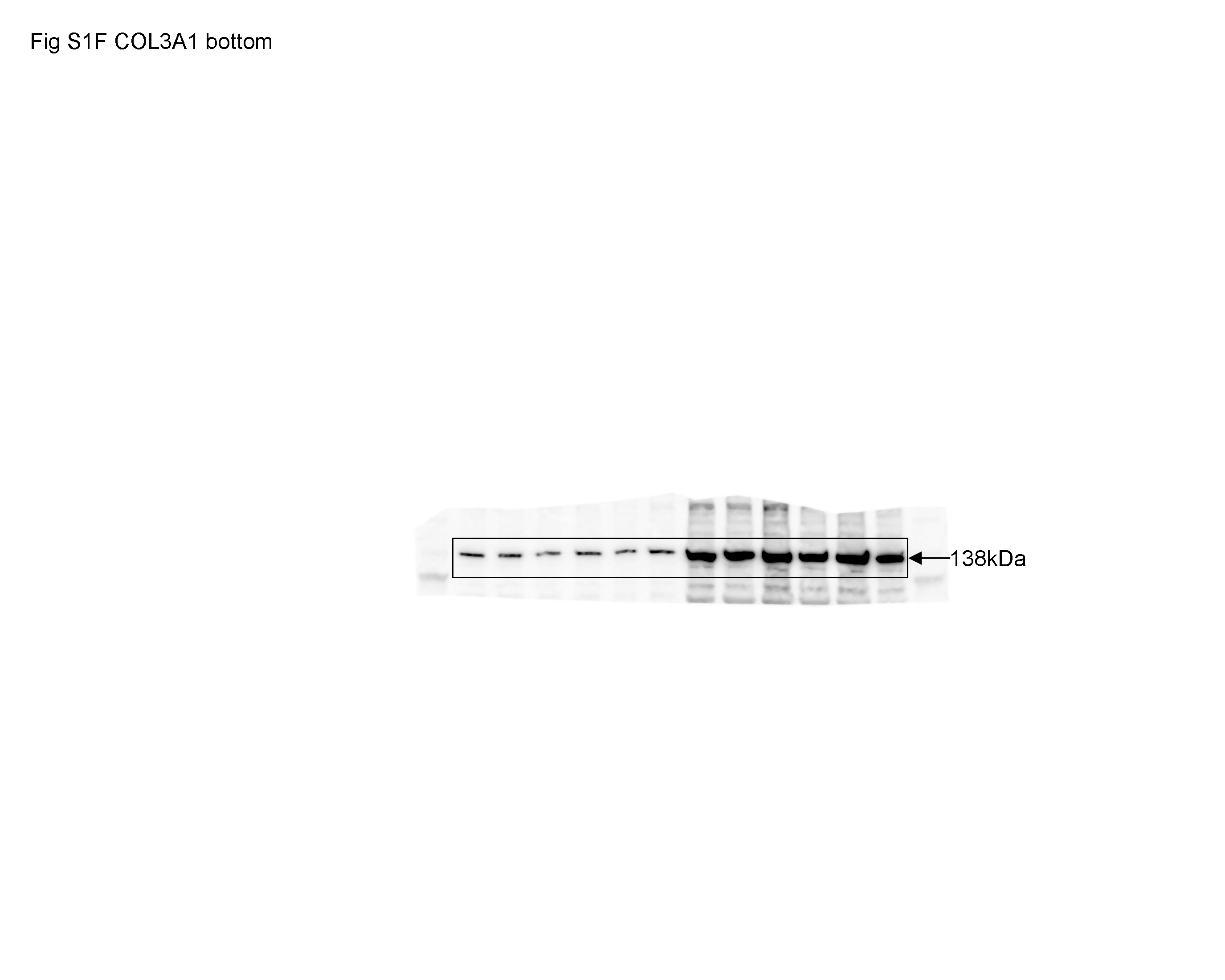

Supplement: Figure 2—figure supplement 1—source data 2. [file elife-98524-fig2-figsupp1-data2.zip › Fig 2-fig S1-data2-v1/S1F/bottom/COL3A1.tiff]

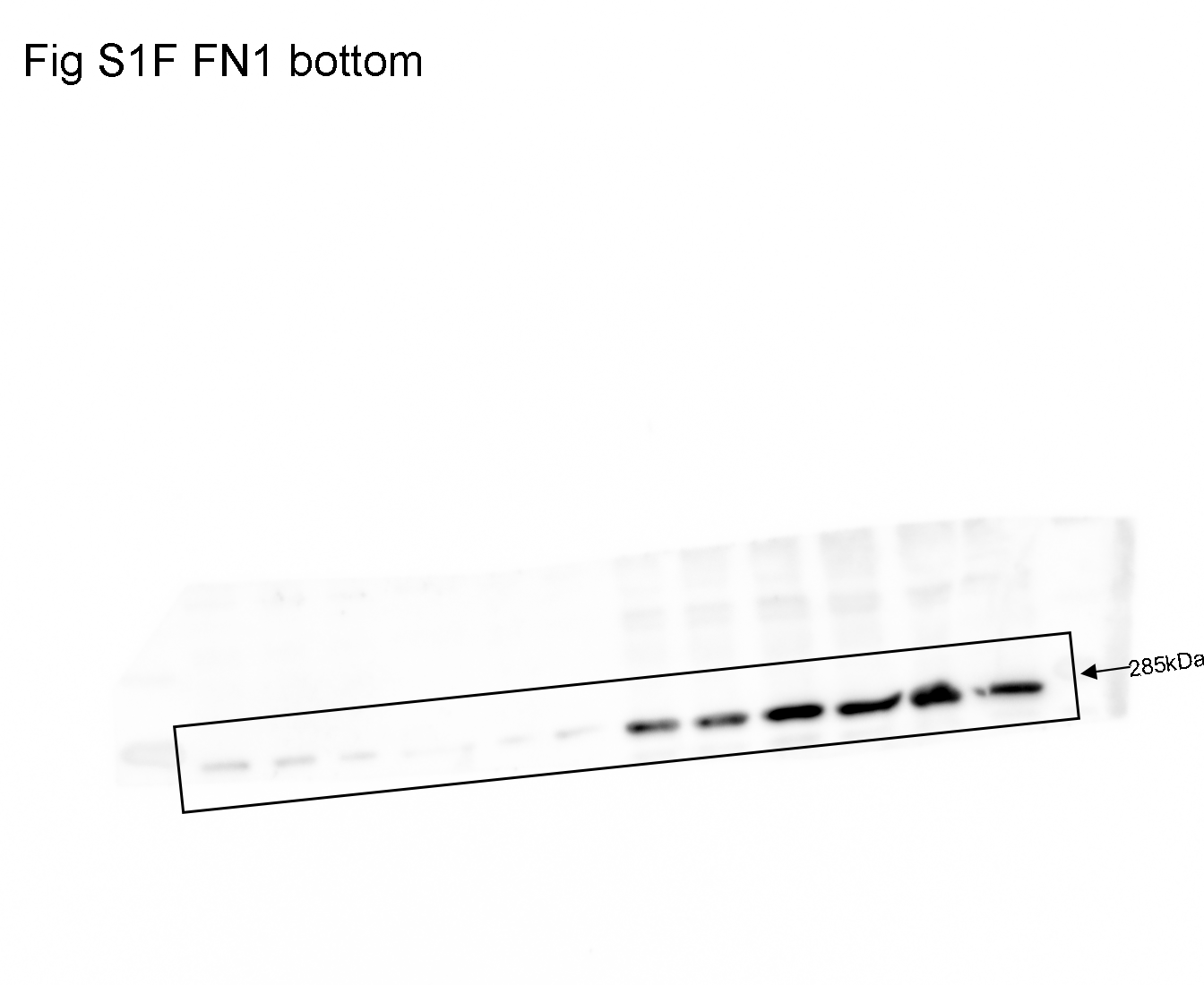

Supplement: Figure 2—figure supplement 1—source data 2. [file elife-98524-fig2-figsupp1-data2.zip › Fig 2-fig S1-data2-v1/S1F/bottom/FN1.tif]

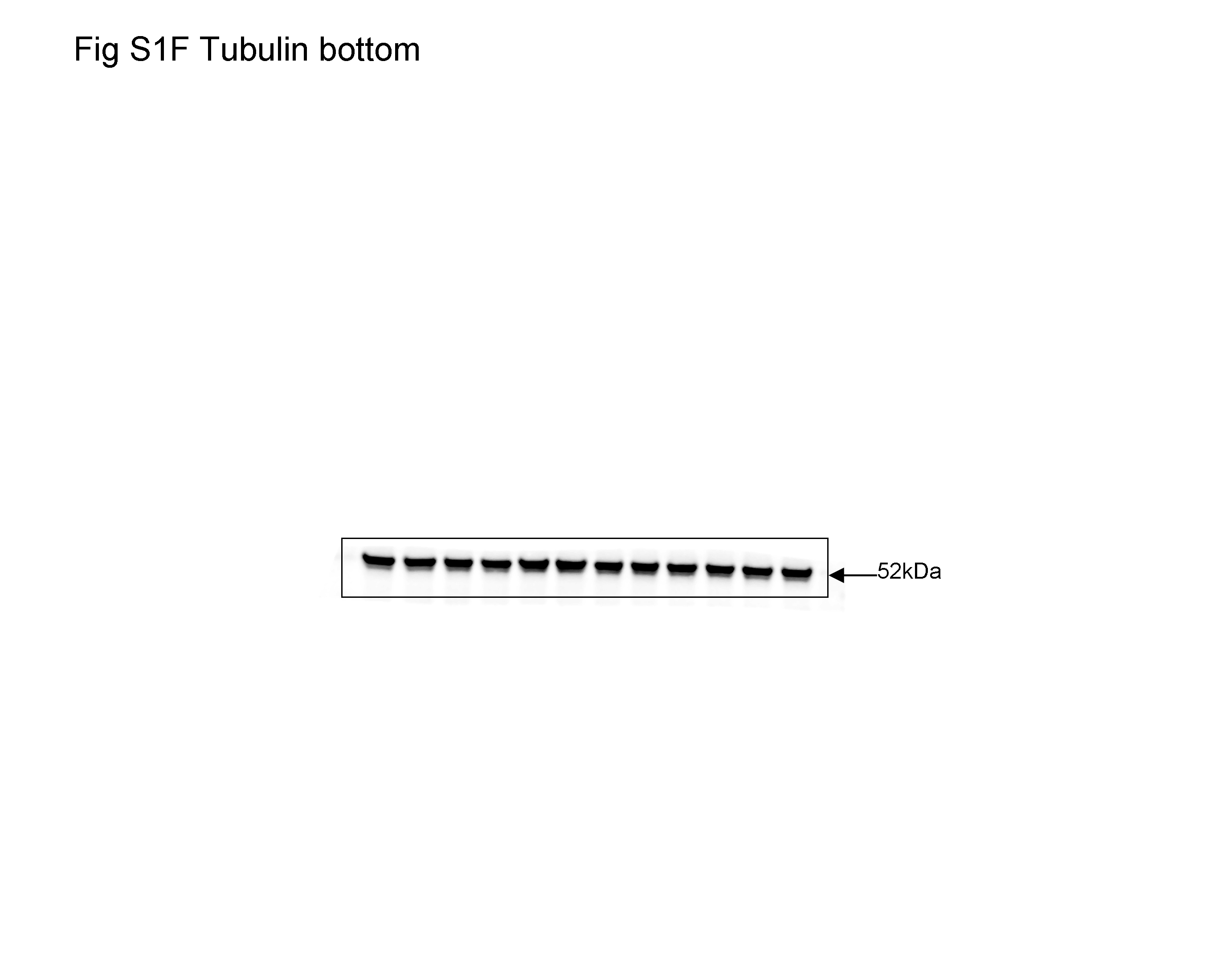

Supplement: Figure 2—figure supplement 1—source data 2. [file elife-98524-fig2-figsupp1-data2.zip › Fig 2-fig S1-data2-v1/S1F/bottom/Tubulin.tiff]

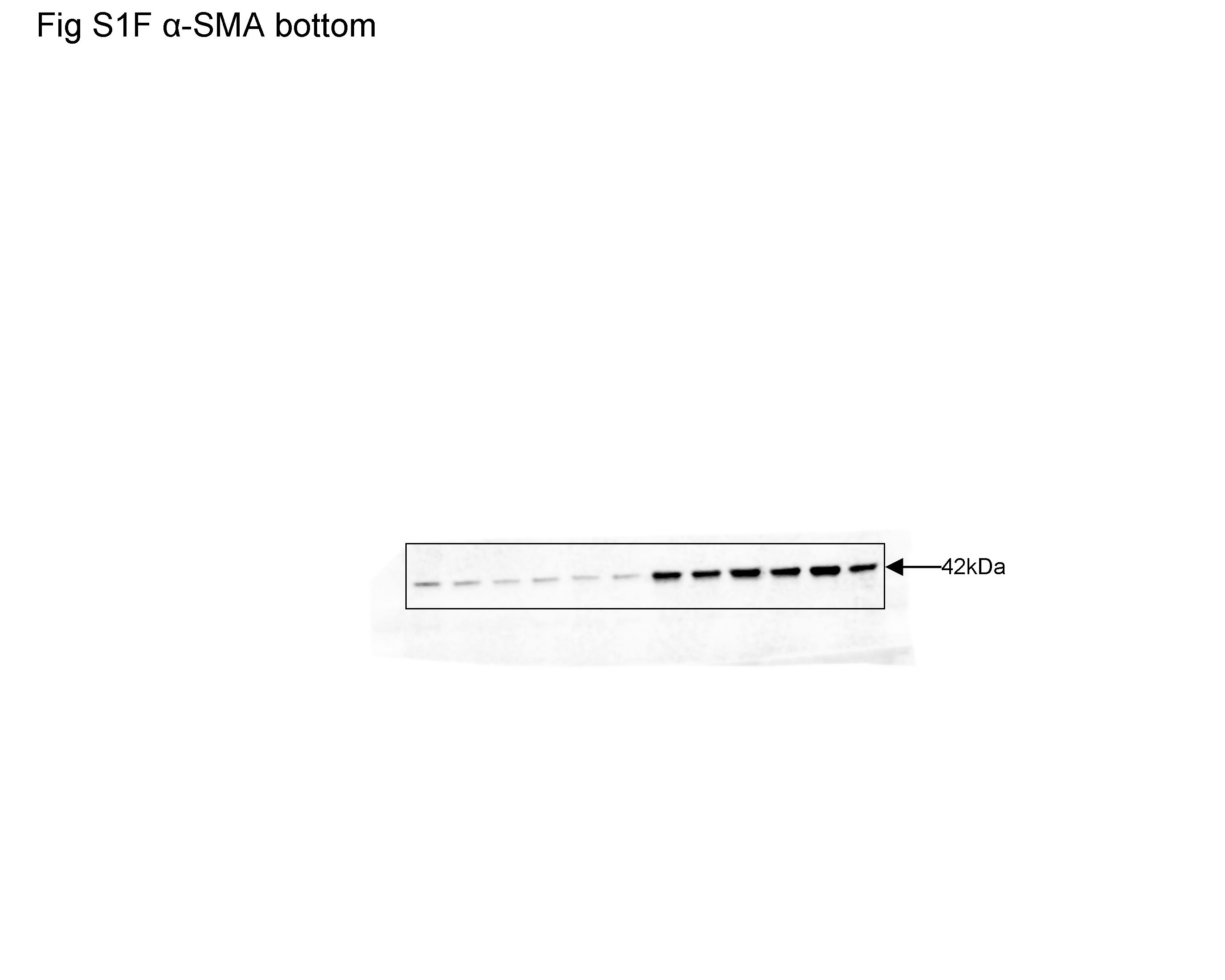

Supplement: Figure 2—figure supplement 1—source data 2. [file elife-98524-fig2-figsupp1-data2.zip › Fig 2-fig S1-data2-v1/S1F/bottom/α-SMA.tiff]

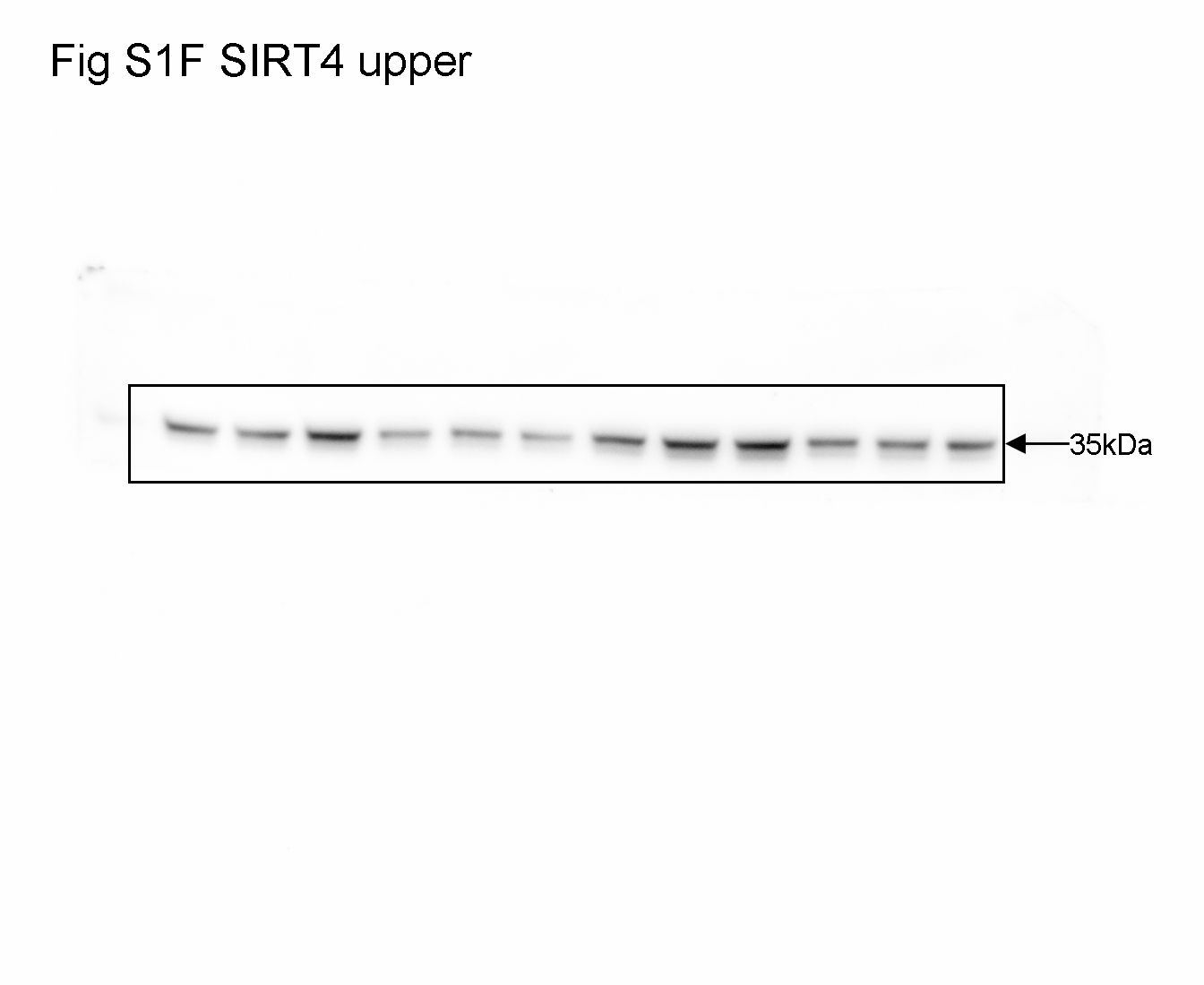

Supplement: Figure 2—figure supplement 1—source data 2. [file elife-98524-fig2-figsupp1-data2.zip › Fig 2-fig S1-data2-v1/S1F/upper/SIRT4.tif]

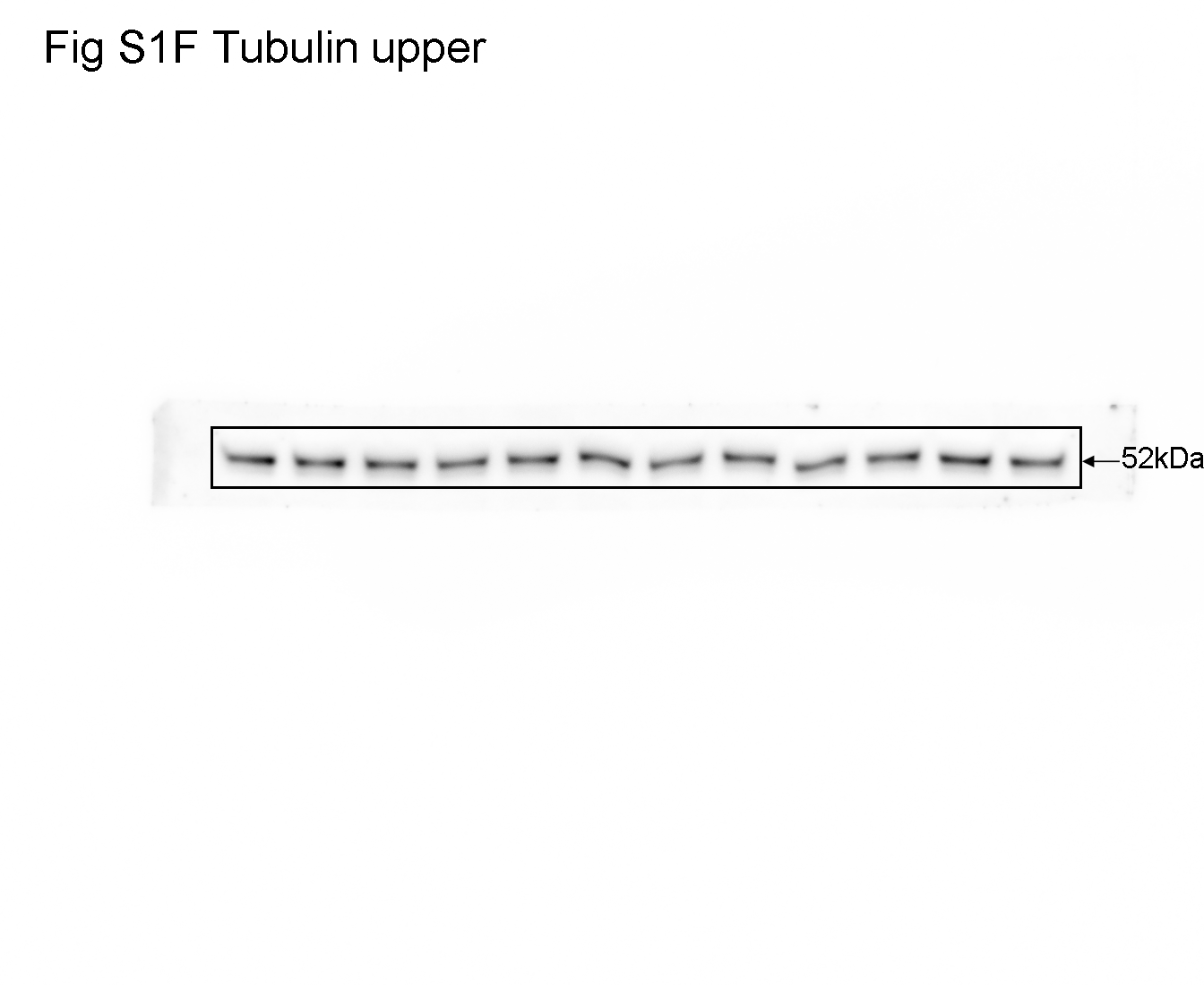

Supplement: Figure 2—figure supplement 1—source data 2. [file elife-98524-fig2-figsupp1-data2.zip › Fig 2-fig S1-data2-v1/S1F/upper/Tubulin.tif]

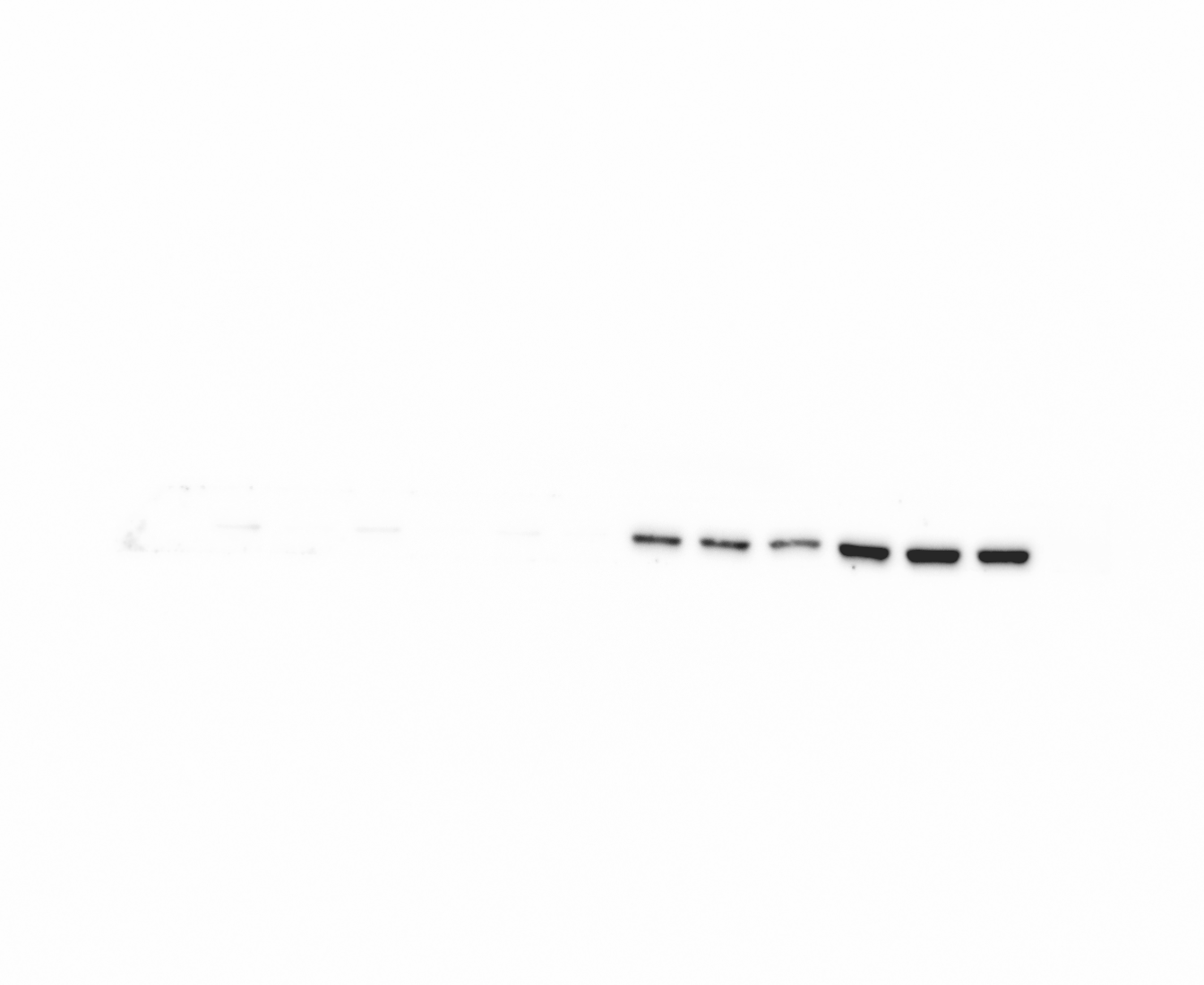

Supplement: Figure 3—source data 1. [file elife-98524-fig3-data1.zip › Fig 3-data1-v1/3B/CCN2.tif]

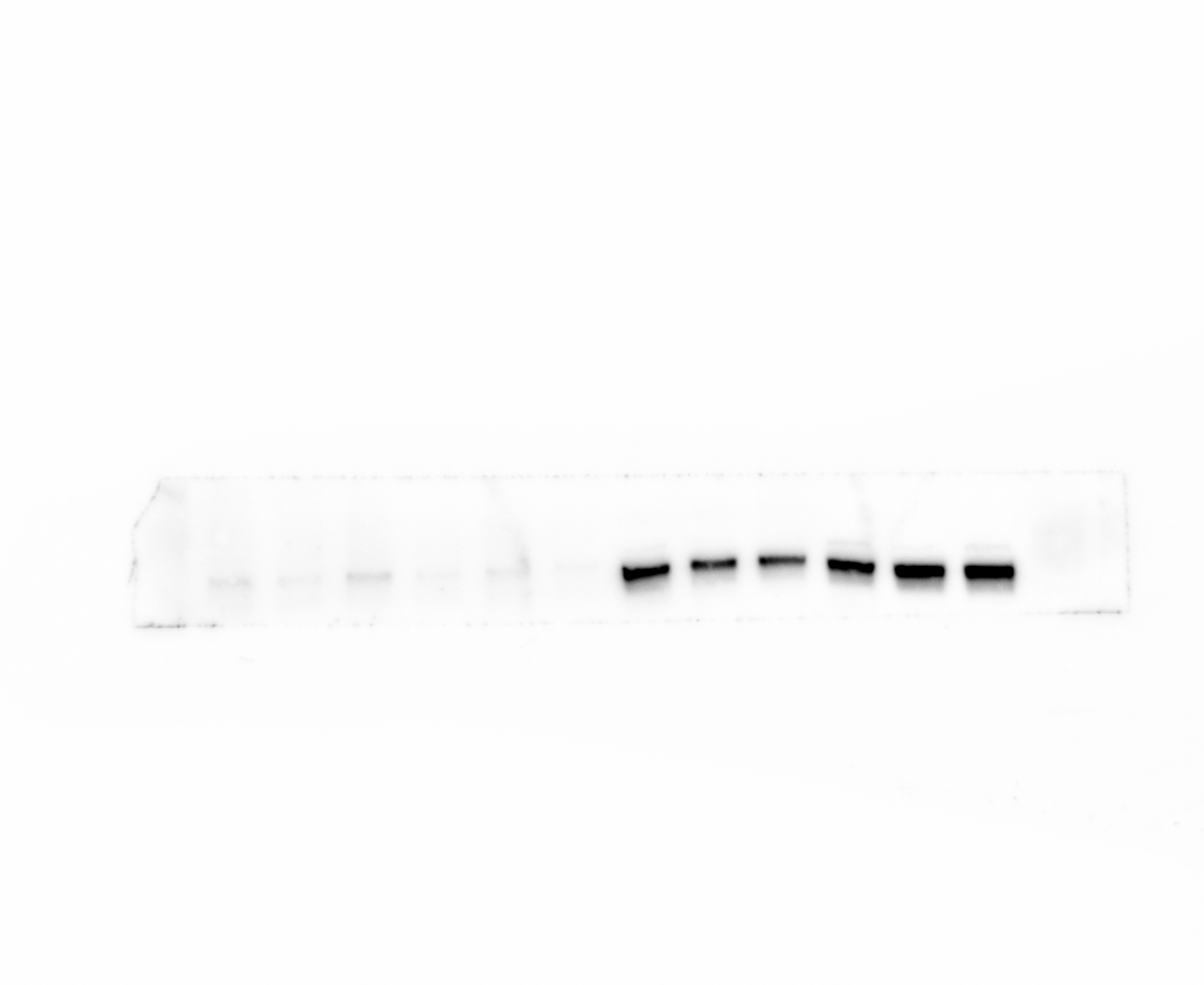

Supplement: Figure 3—source data 1. [file elife-98524-fig3-data1.zip › Fig 3-data1-v1/3B/COL1A1.tif]

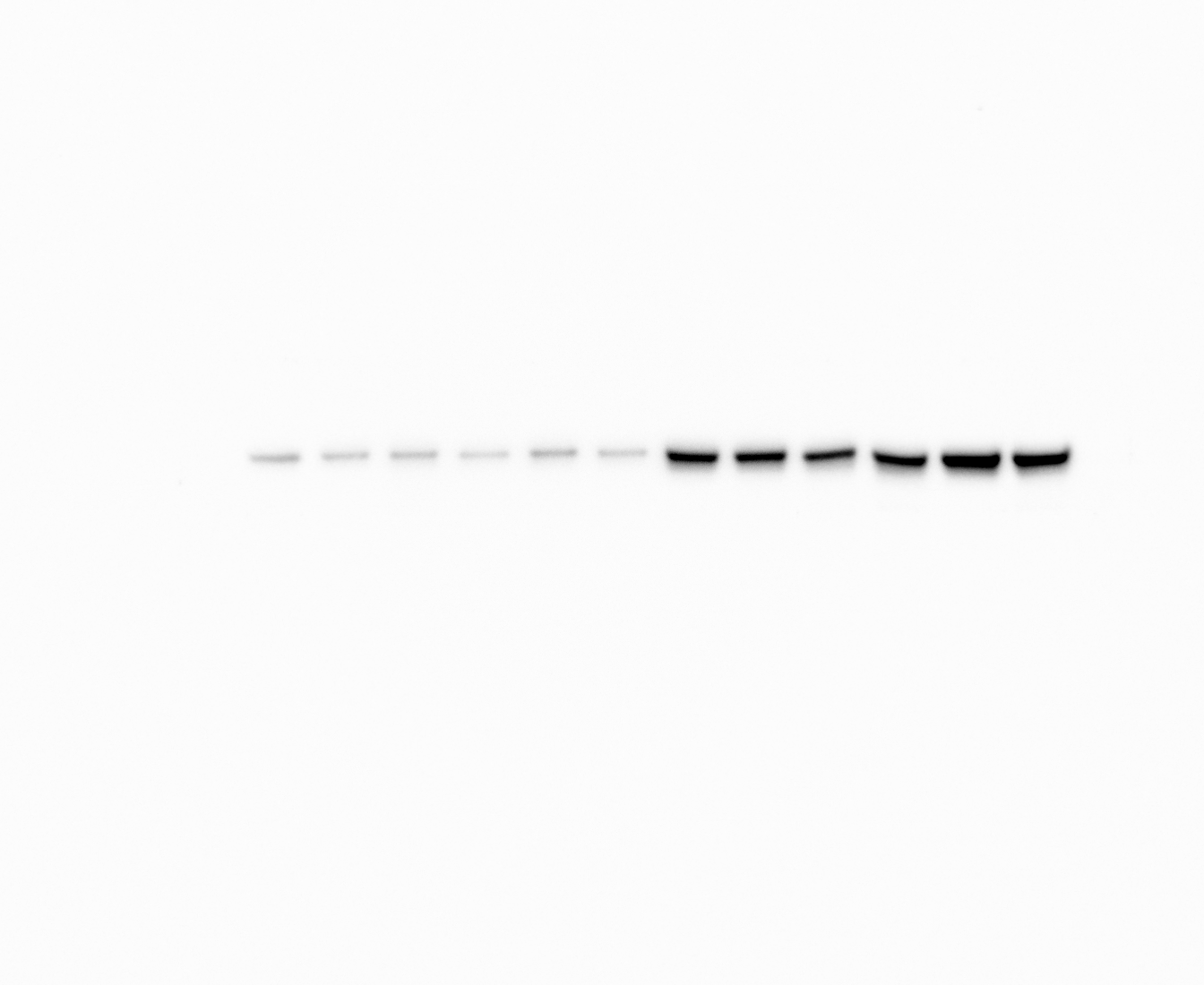

Supplement: Figure 3—source data 1. [file elife-98524-fig3-data1.zip › Fig 3-data1-v1/3B/COL3A1.tif]

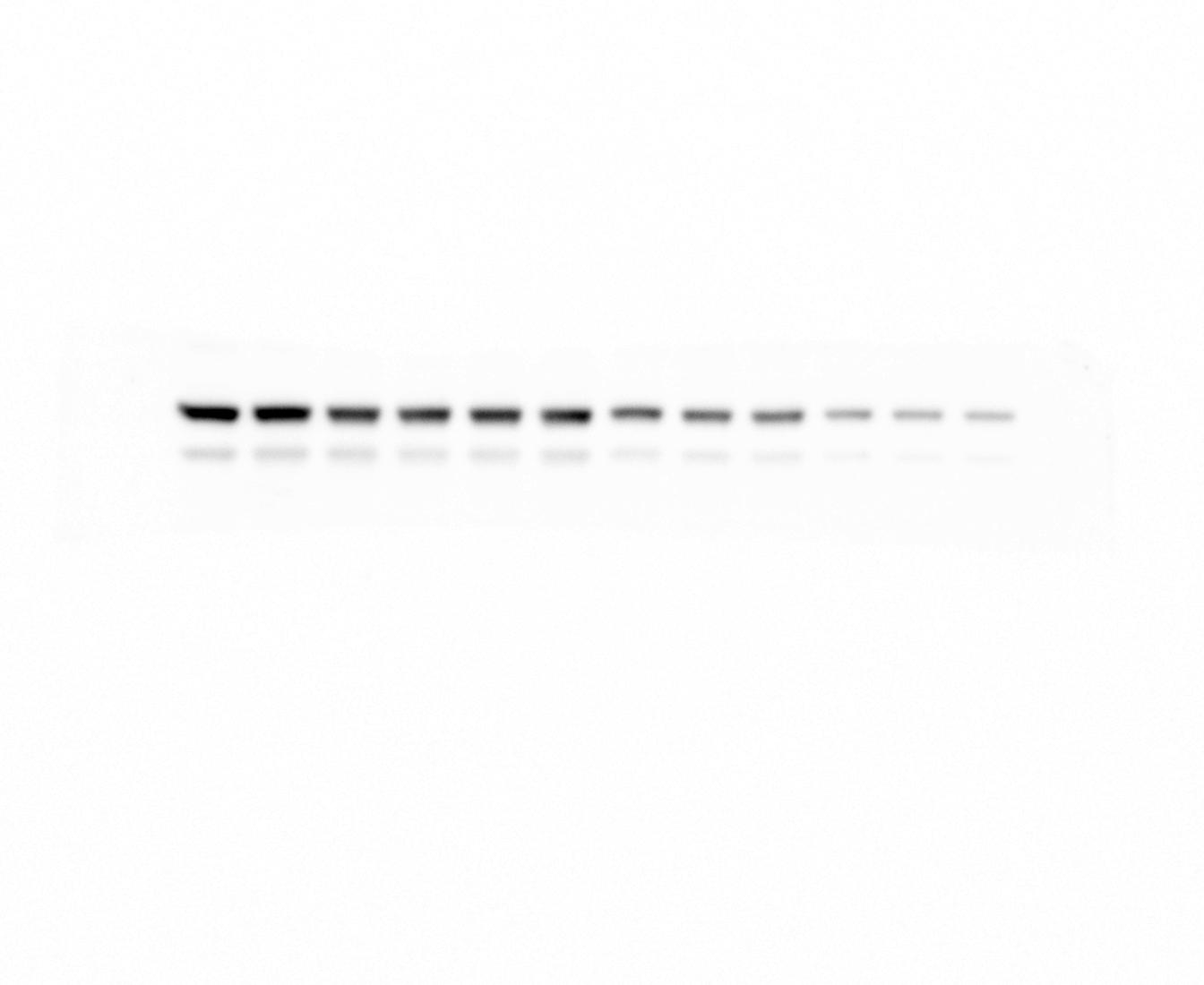

Supplement: Figure 3—source data 1. [file elife-98524-fig3-data1.zip › Fig 3-data1-v1/3B/E-cadherin.tif]

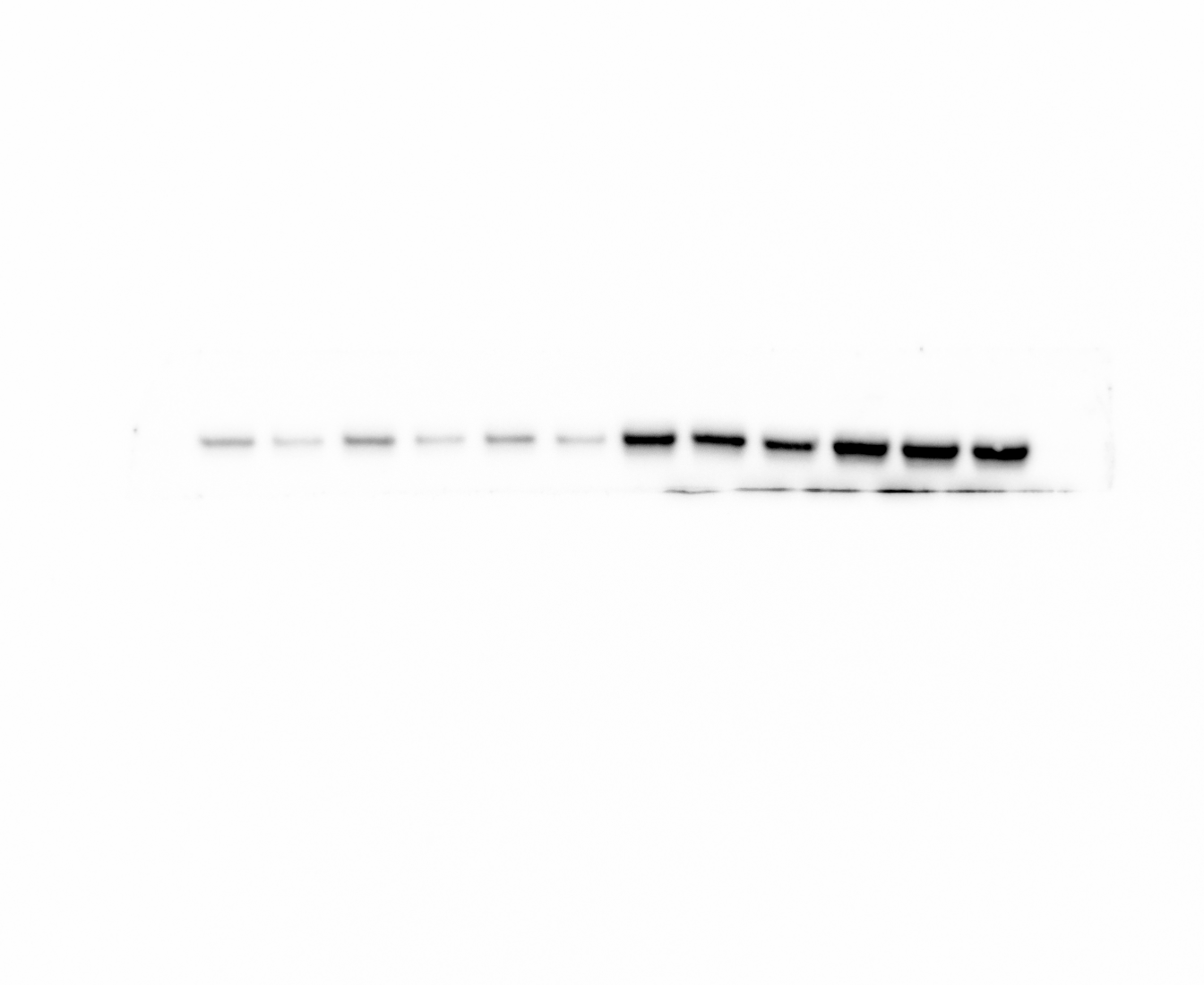

Supplement: Figure 3—source data 1. [file elife-98524-fig3-data1.zip › Fig 3-data1-v1/3B/FN1.tif]

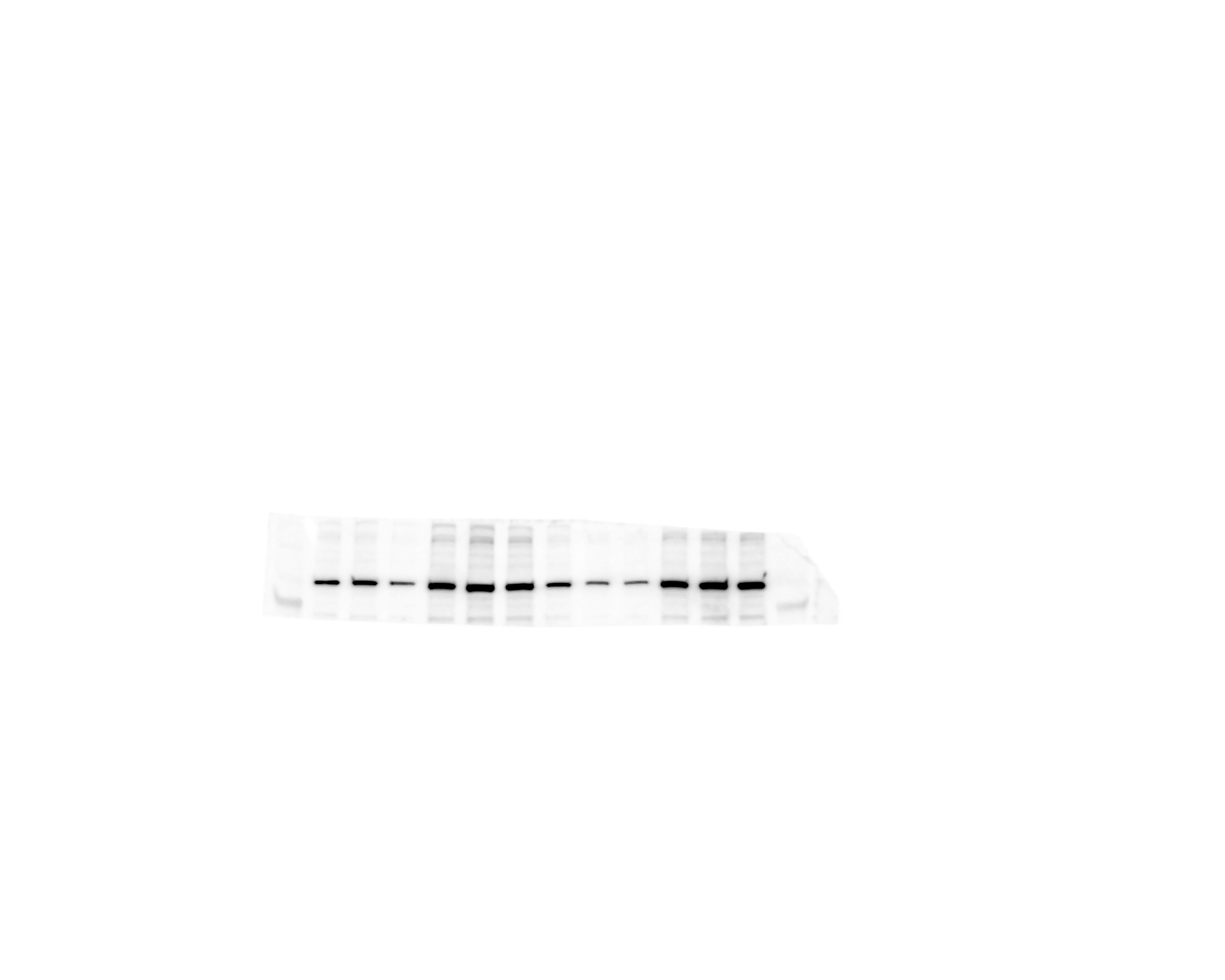

Supplement: Figure 3—source data 1. [file elife-98524-fig3-data1.zip › Fig 3-data1-v1/3B/SIRT4.tiff]

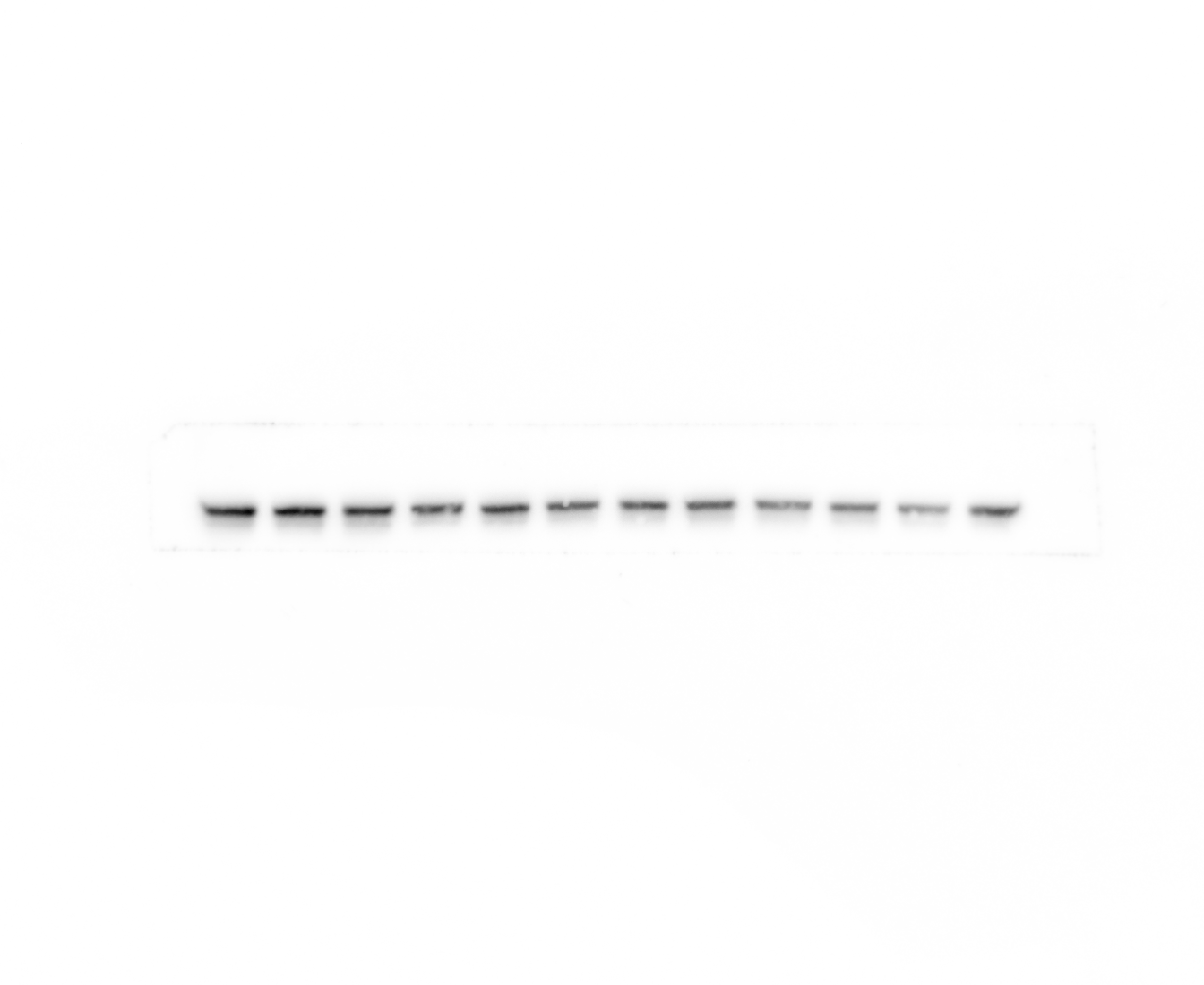

Supplement: Figure 3—source data 1. [file elife-98524-fig3-data1.zip › Fig 3-data1-v1/3B/Tubulin.tif]

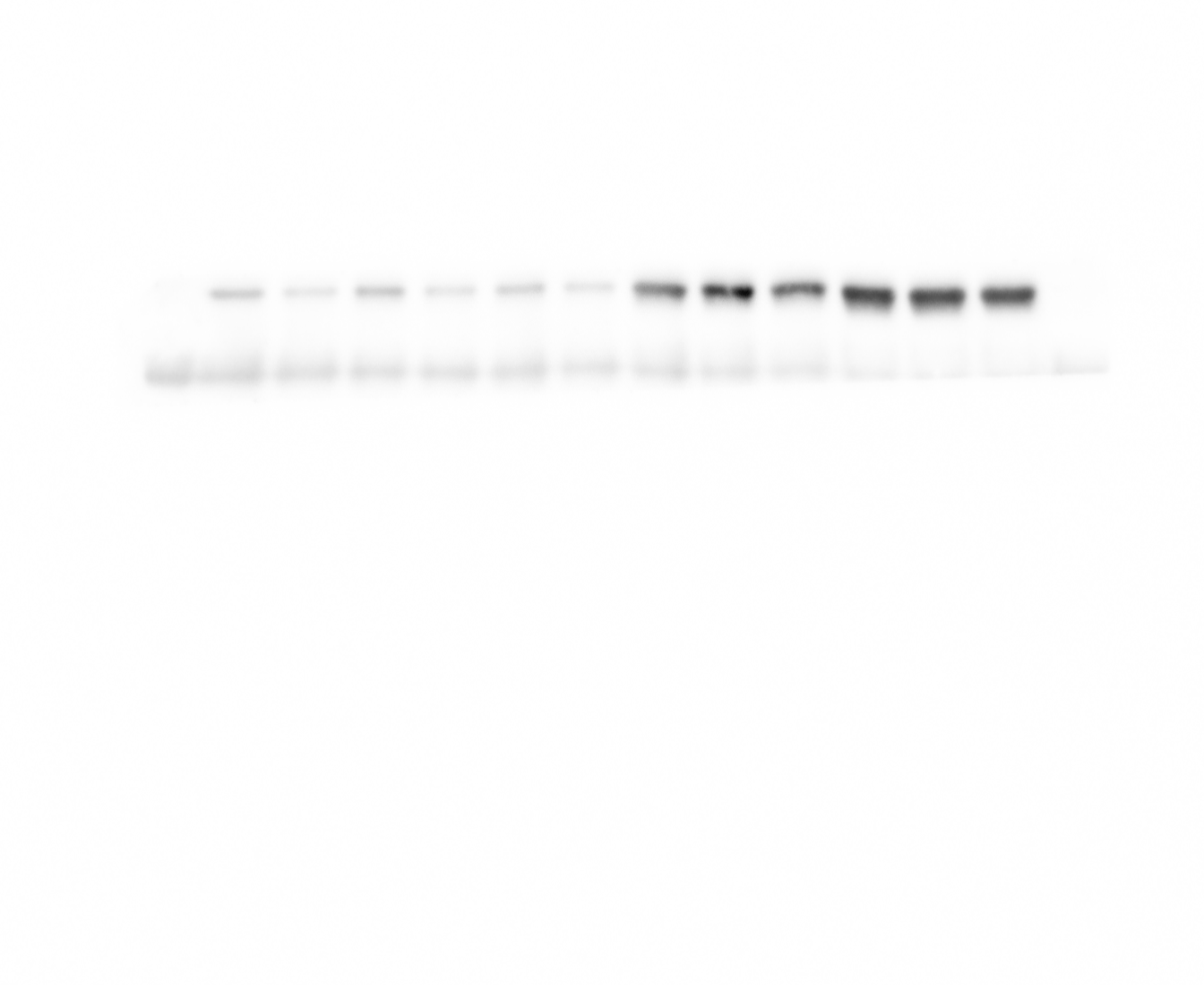

Supplement: Figure 3—source data 1. [file elife-98524-fig3-data1.zip › Fig 3-data1-v1/3B/α-SMA.tif]

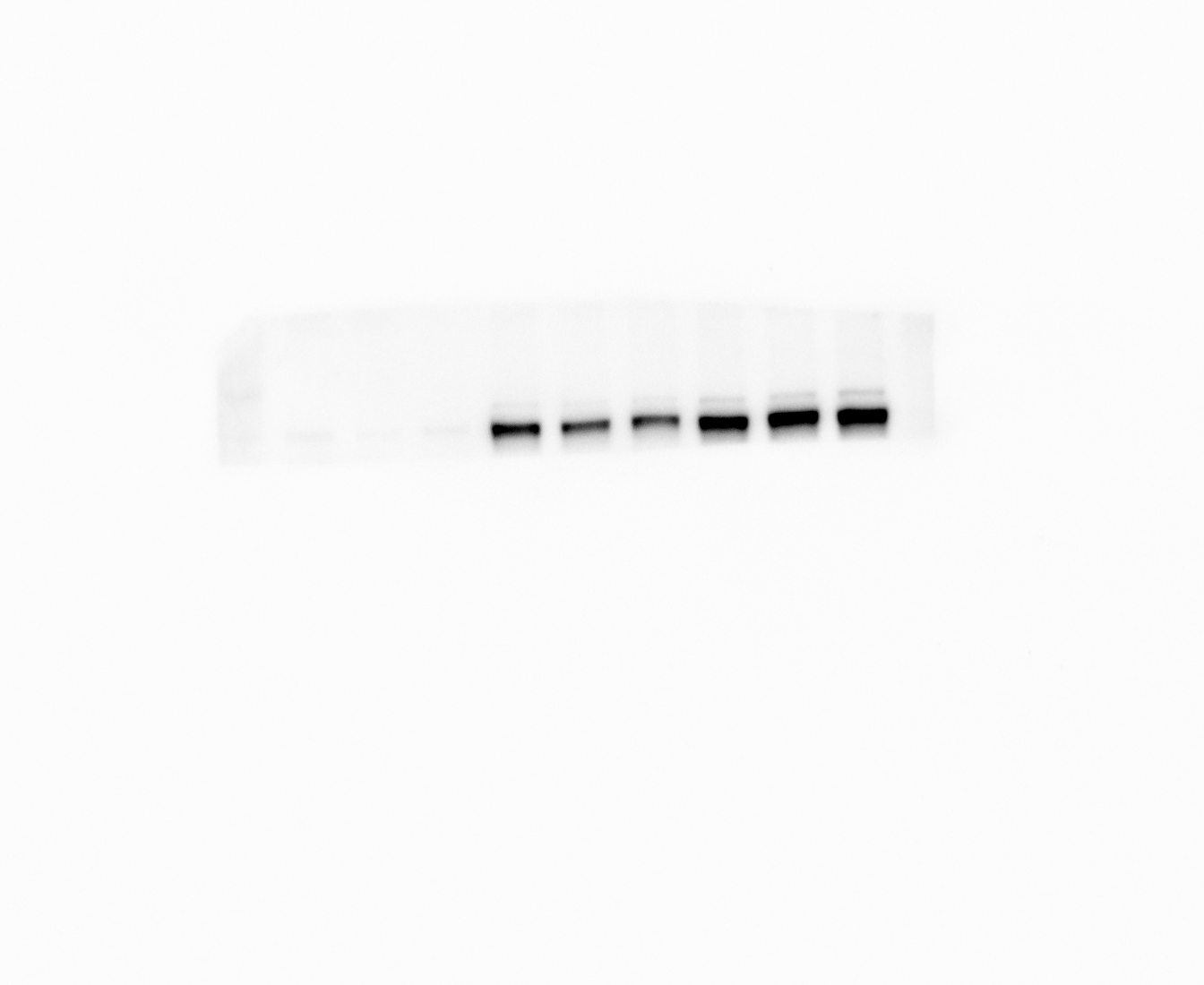

Supplement: Figure 3—source data 1. [file elife-98524-fig3-data1.zip › Fig 3-data1-v1/3E/CCN2.tif]

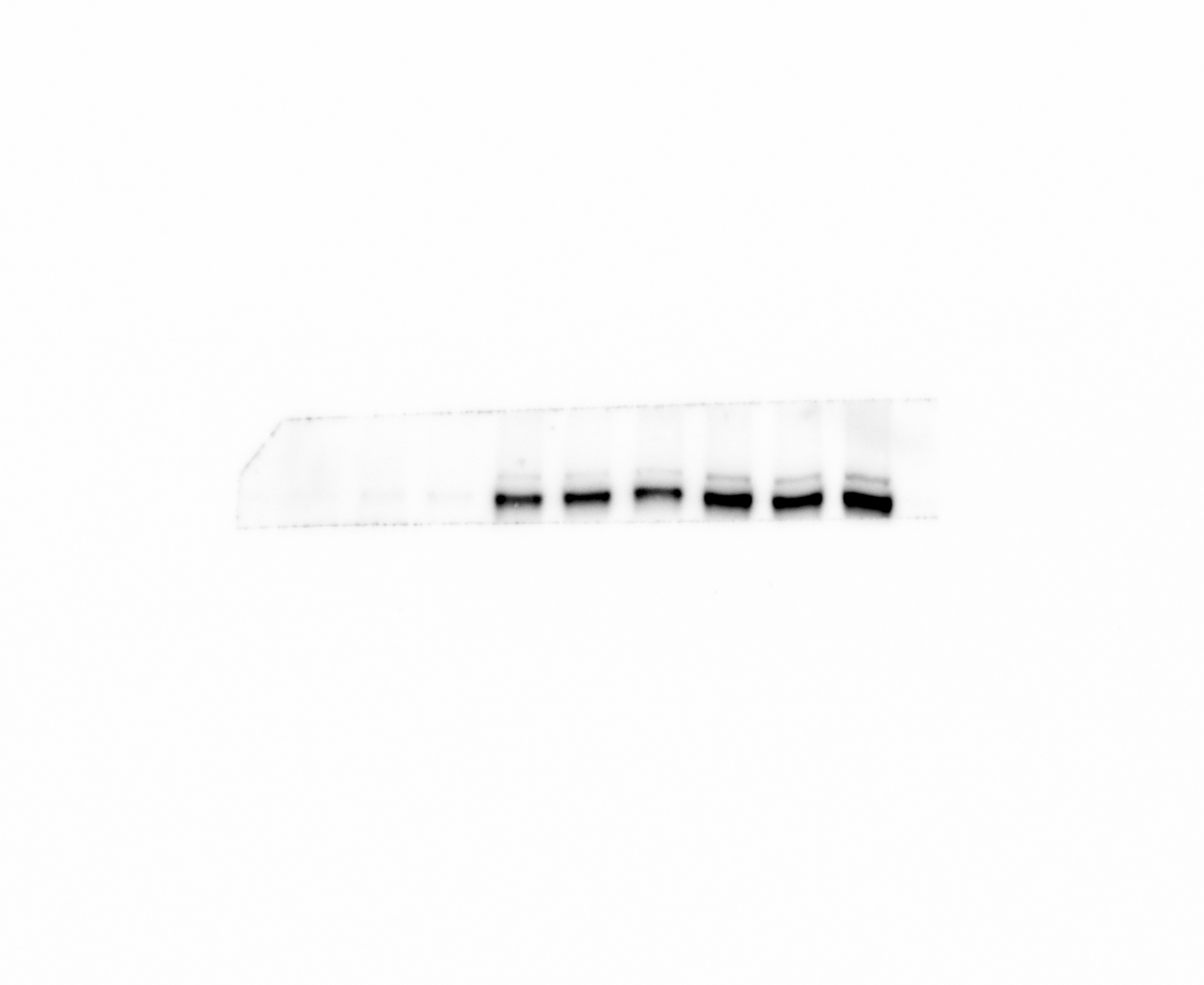

Supplement: Figure 3—source data 1. [file elife-98524-fig3-data1.zip › Fig 3-data1-v1/3E/COL1A1.tif]

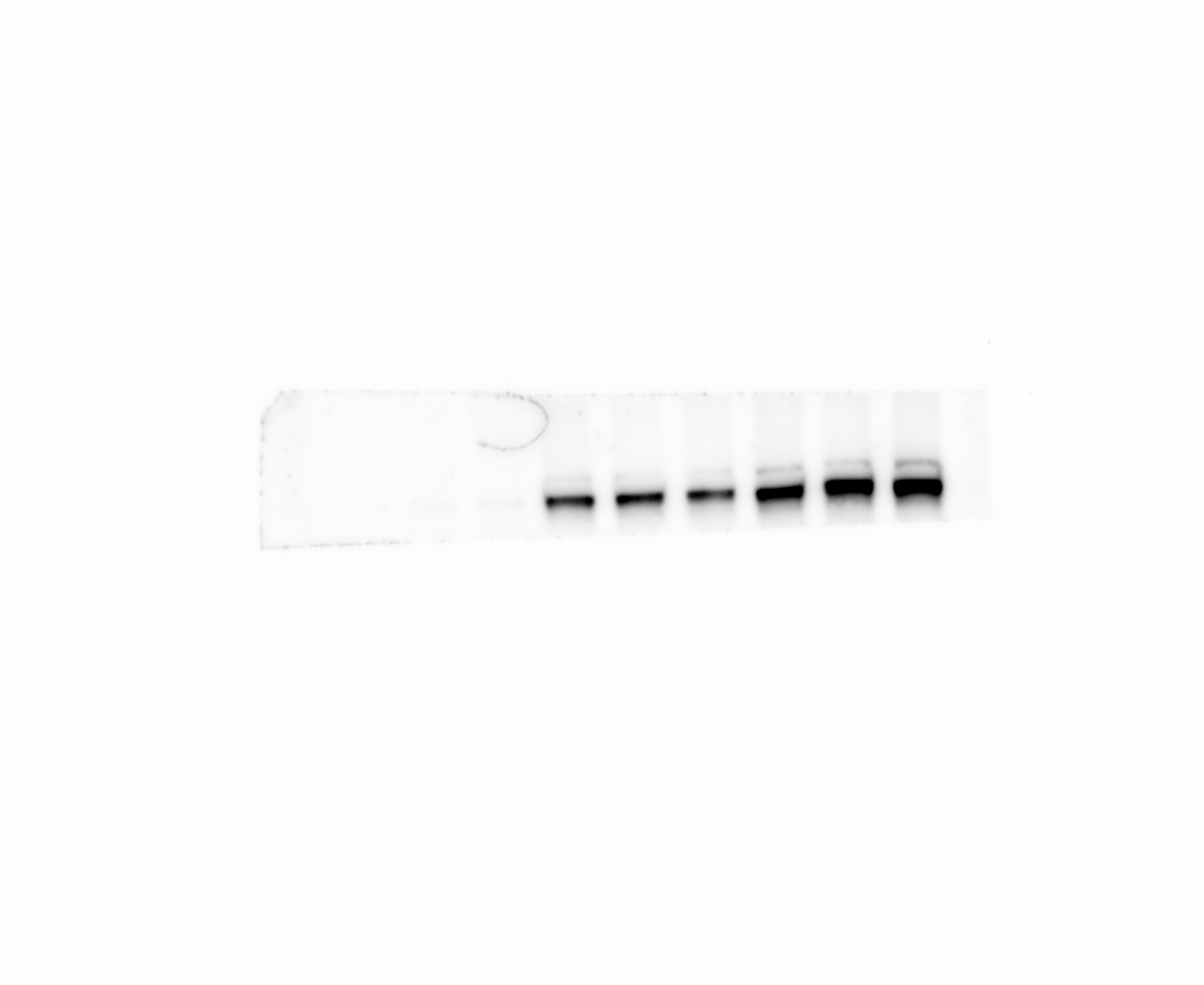

Supplement: Figure 3—source data 1. [file elife-98524-fig3-data1.zip › Fig 3-data1-v1/3E/COL3A1.tif]

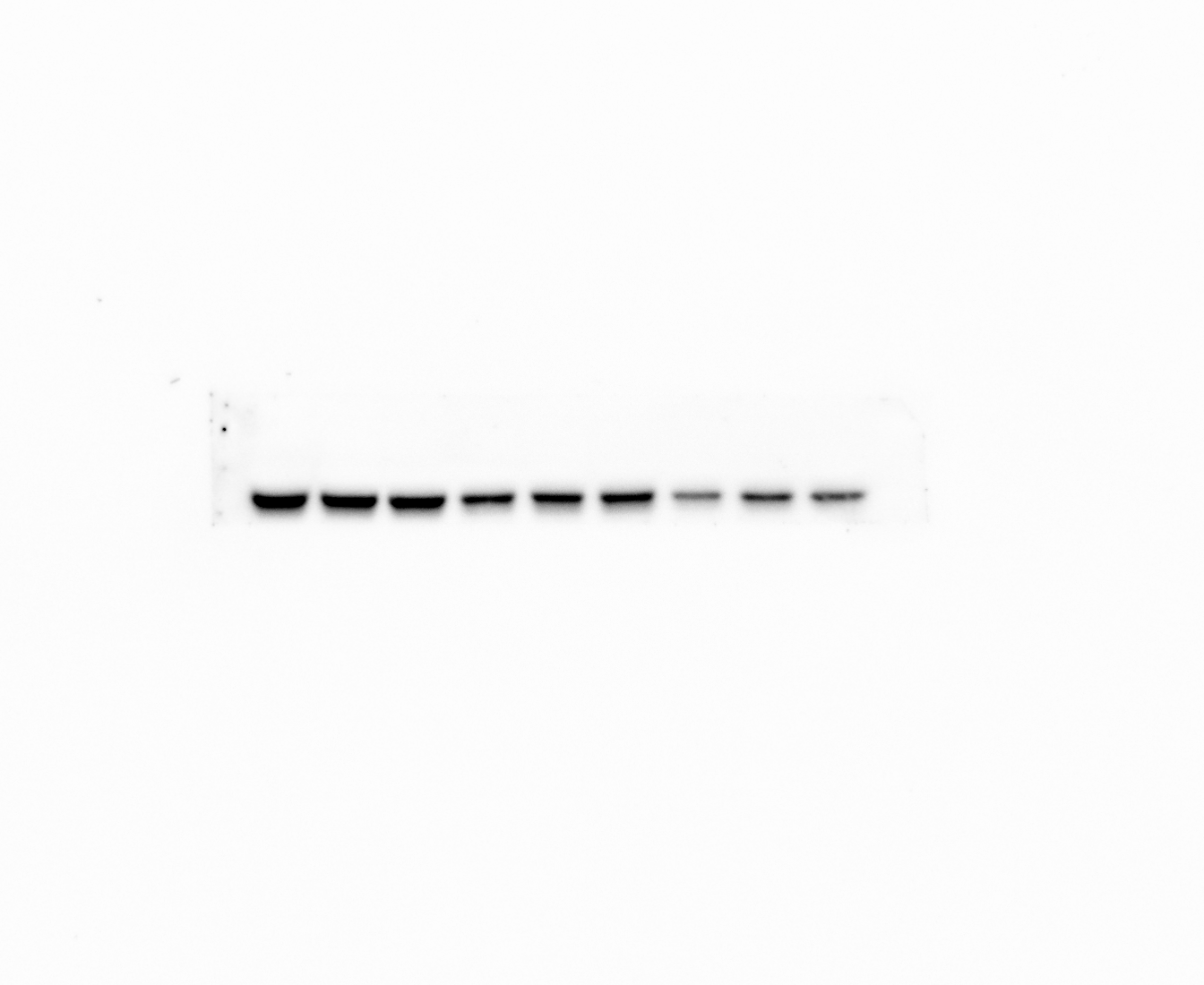

Supplement: Figure 3—source data 1. [file elife-98524-fig3-data1.zip › Fig 3-data1-v1/3E/E-cadherin.tif]

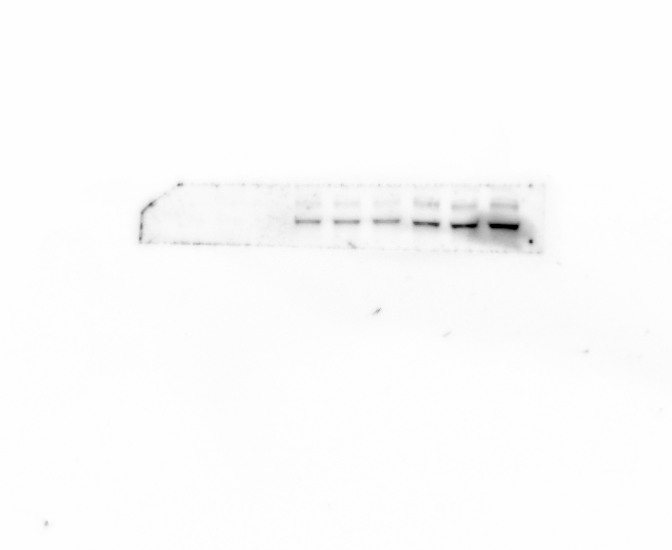

Supplement: Figure 3—source data 1. [file elife-98524-fig3-data1.zip › Fig 3-data1-v1/3E/FN1.tif]

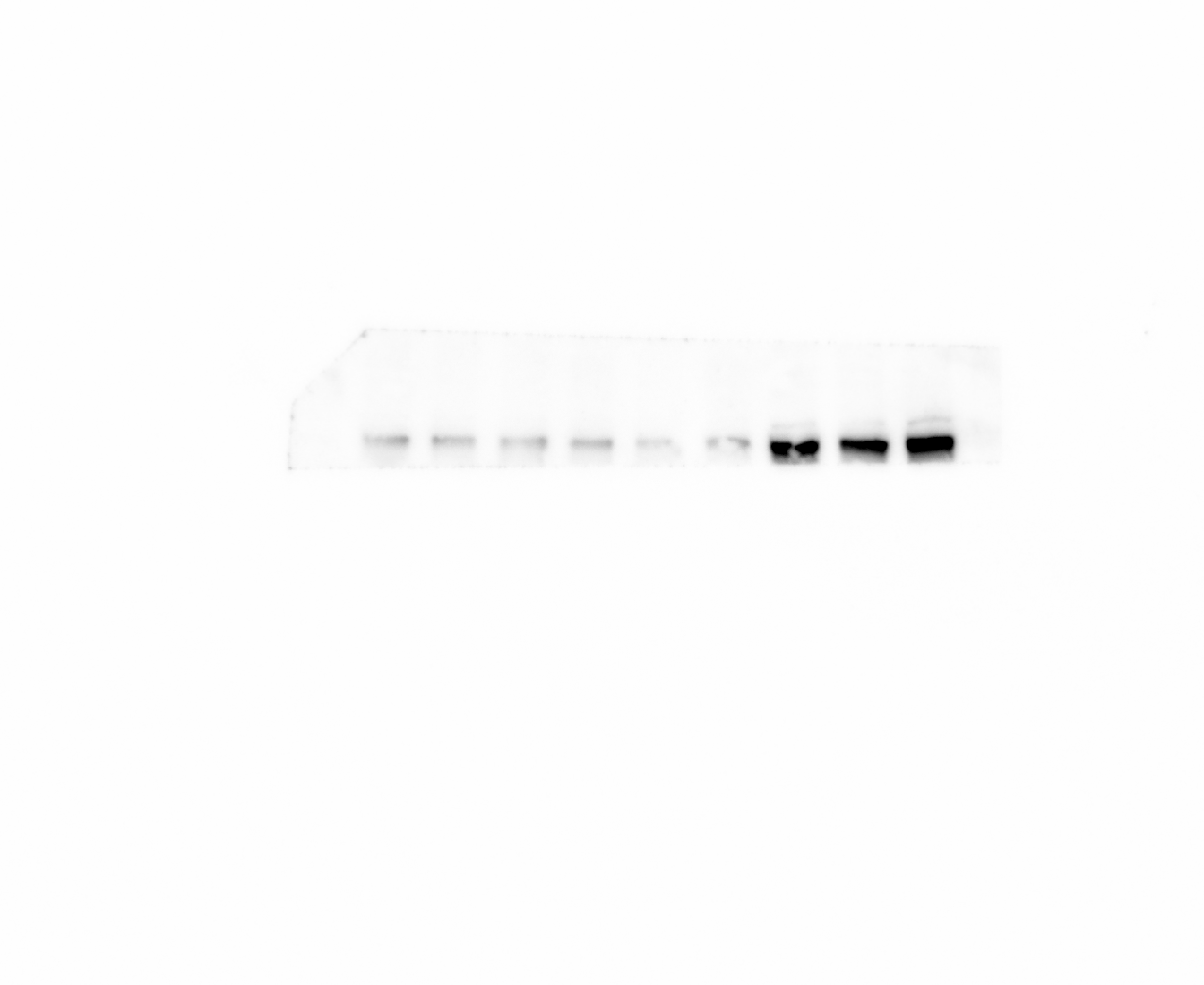

Supplement: Figure 3—source data 1. [file elife-98524-fig3-data1.zip › Fig 3-data1-v1/3E/SIRT4.tif]

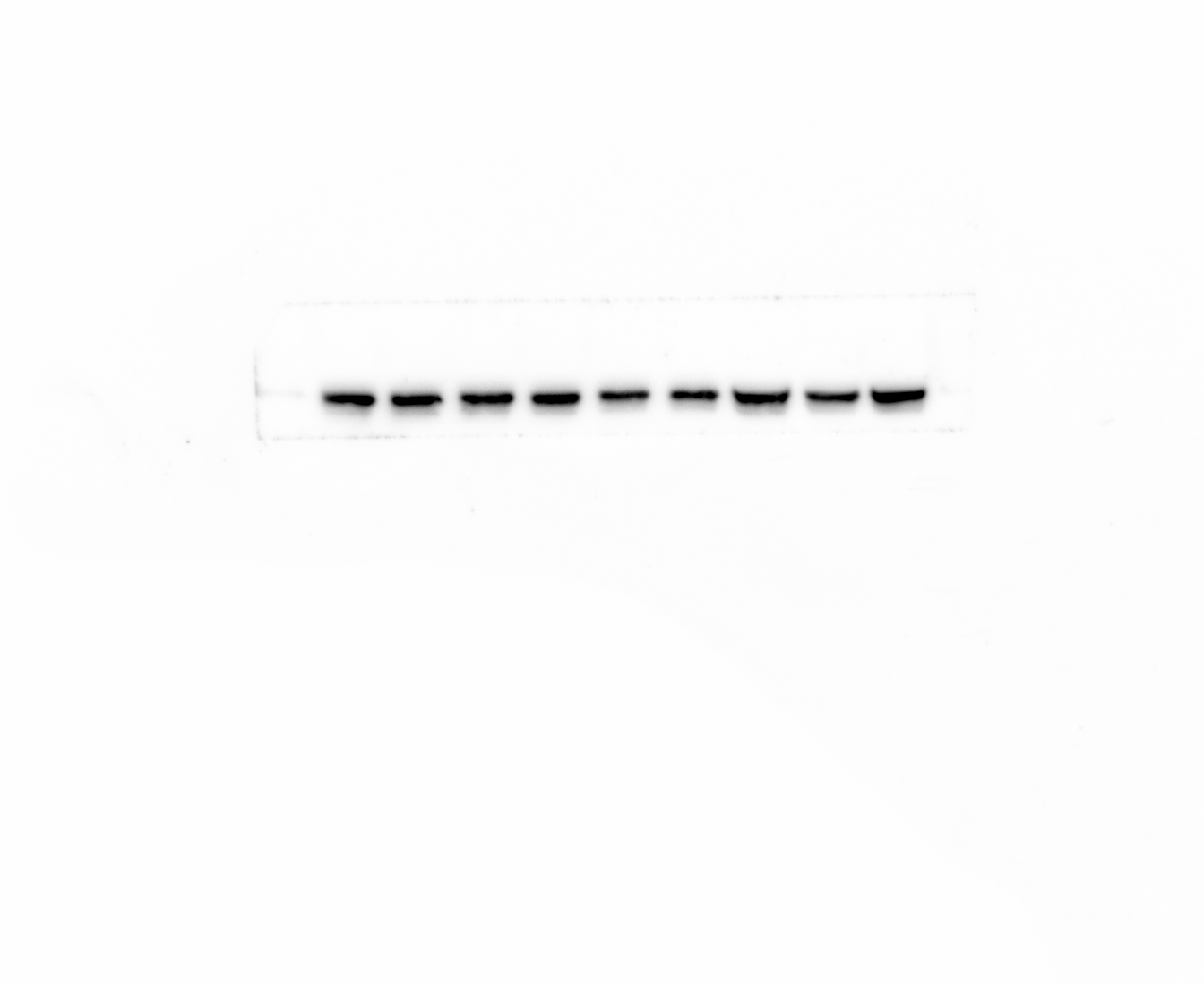

Supplement: Figure 3—source data 1. [file elife-98524-fig3-data1.zip › Fig 3-data1-v1/3E/Tubulin.tif]

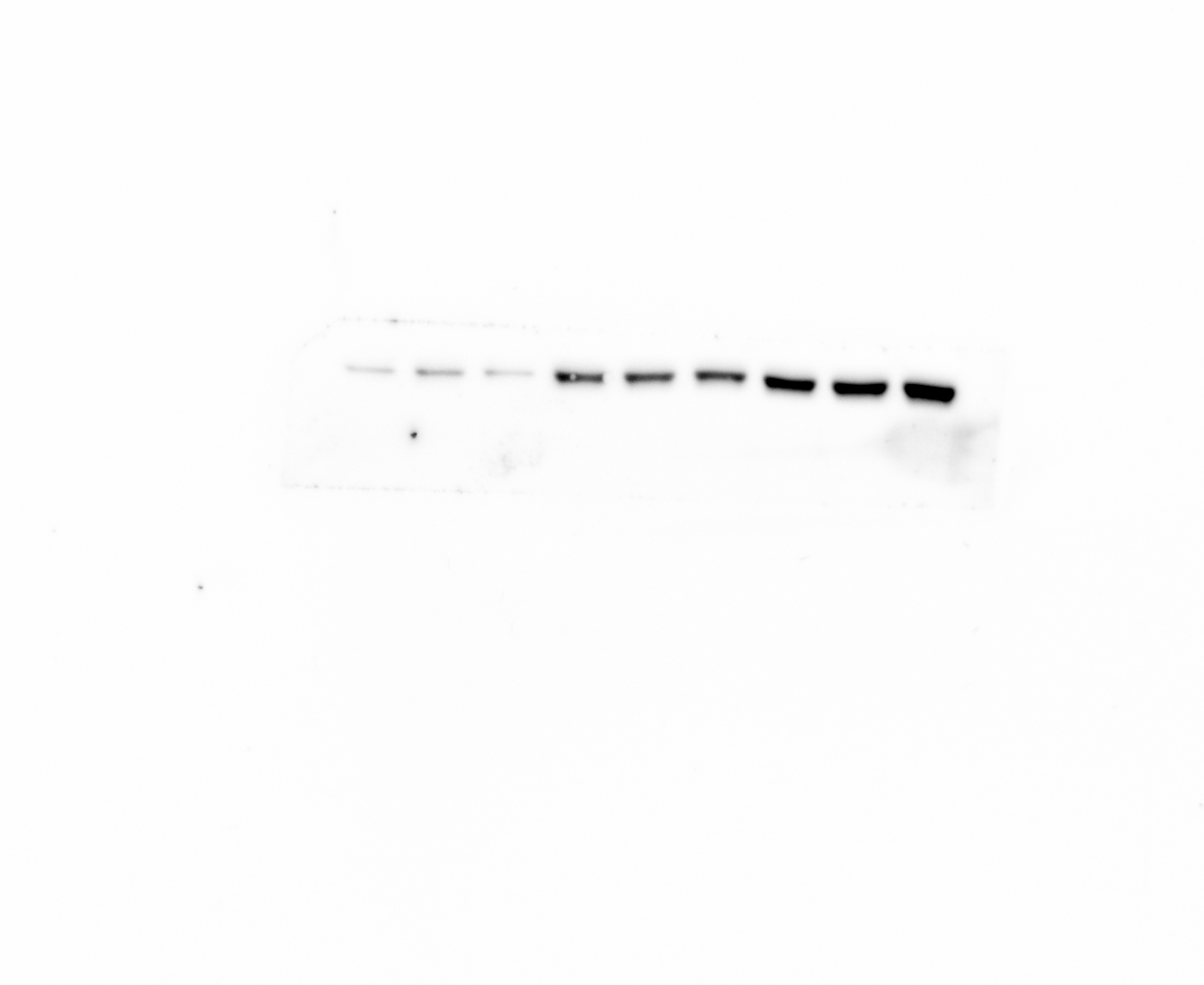

Supplement: Figure 3—source data 1. [file elife-98524-fig3-data1.zip › Fig 3-data1-v1/3E/α-SMA.tif]

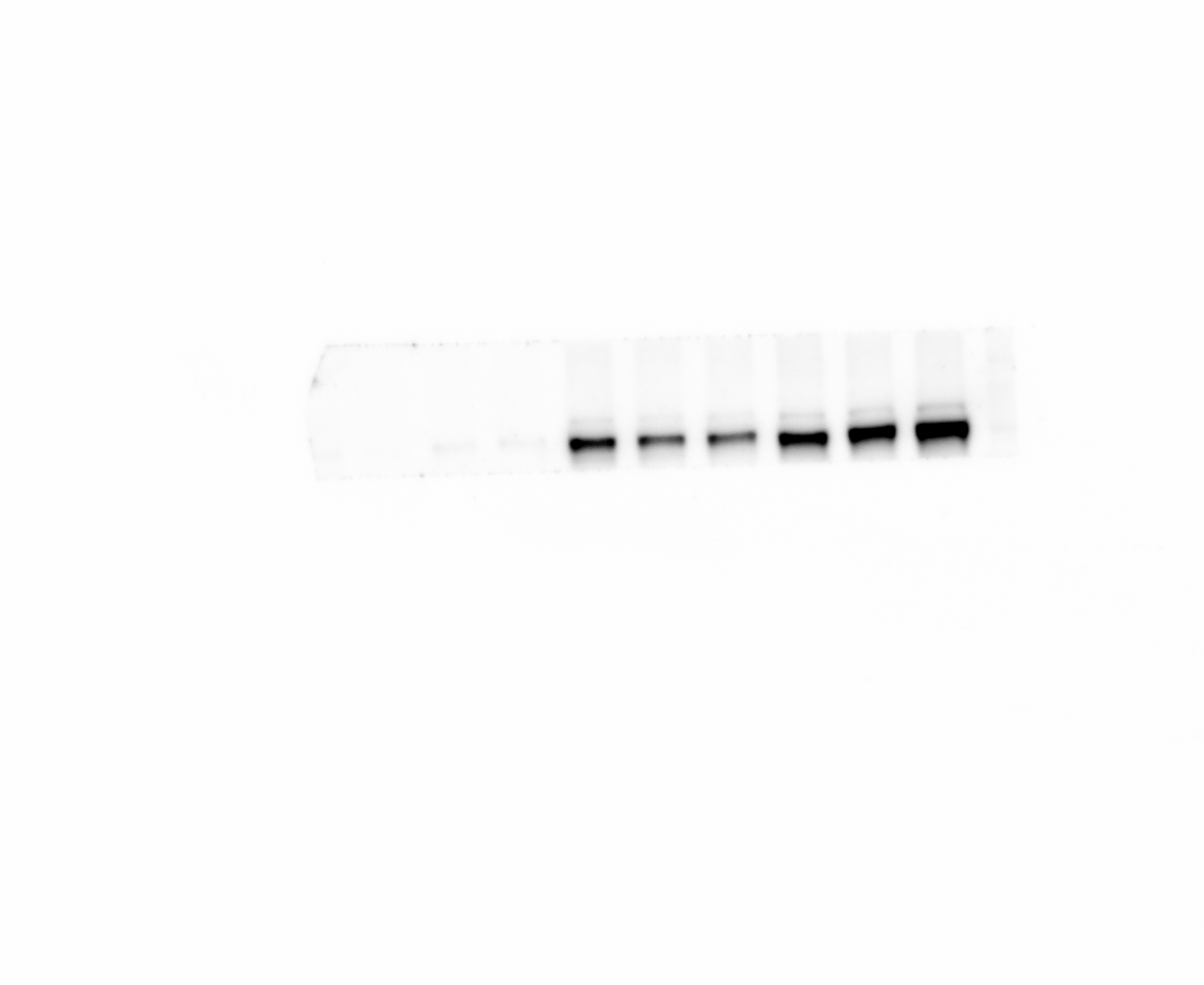

Supplement: Figure 3—source data 1. [file elife-98524-fig3-data1.zip › Fig 3-data1-v1/3H/CCN2.tif]

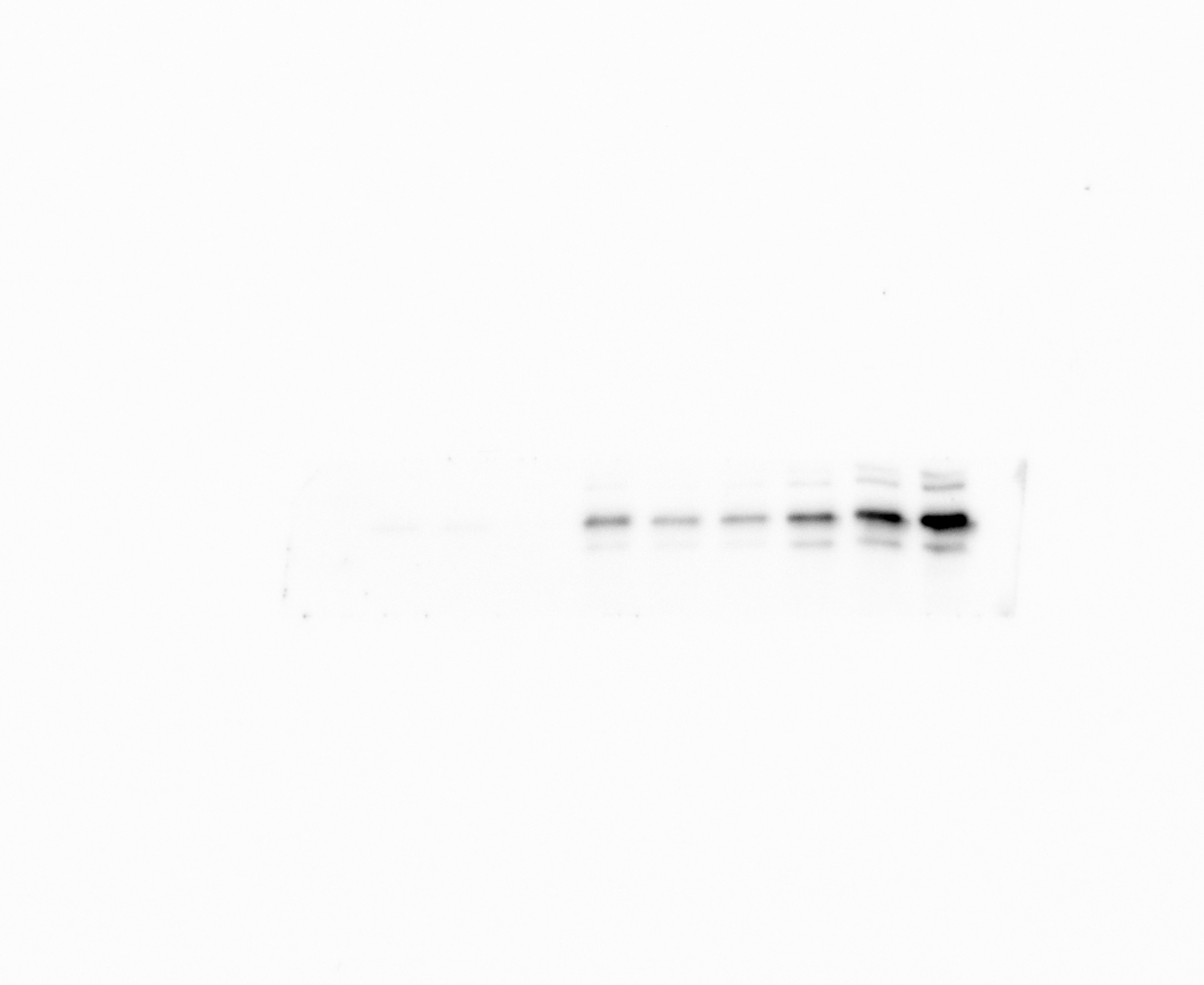

Supplement: Figure 3—source data 1. [file elife-98524-fig3-data1.zip › Fig 3-data1-v1/3H/COL1A1.tif]

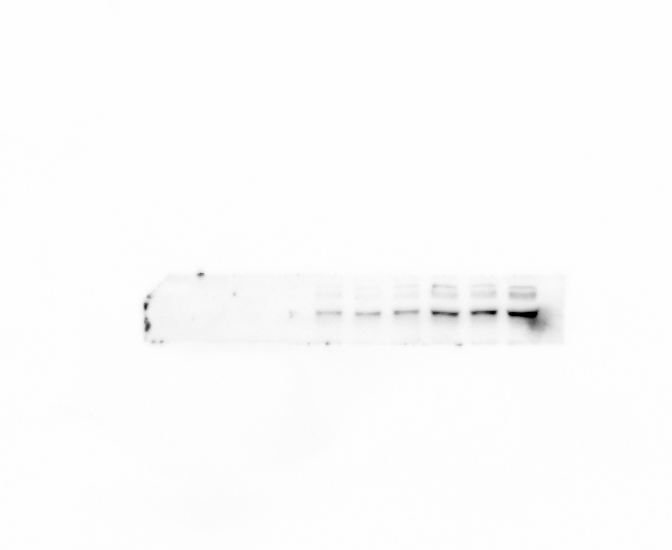

Supplement: Figure 3—source data 1. [file elife-98524-fig3-data1.zip › Fig 3-data1-v1/3H/COL3A1.tif]

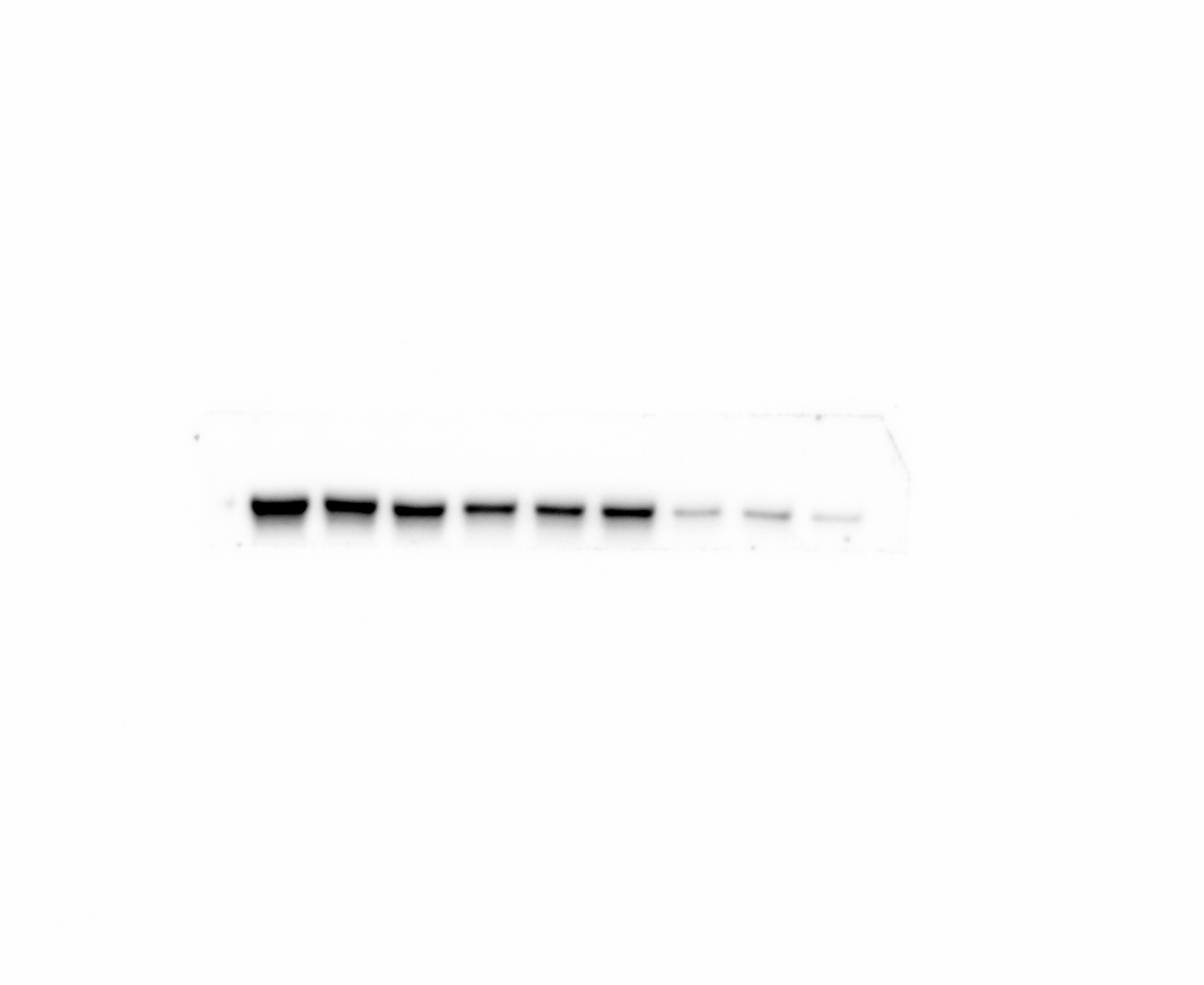

Supplement: Figure 3—source data 1. [file elife-98524-fig3-data1.zip › Fig 3-data1-v1/3H/E-cadherin.tif]

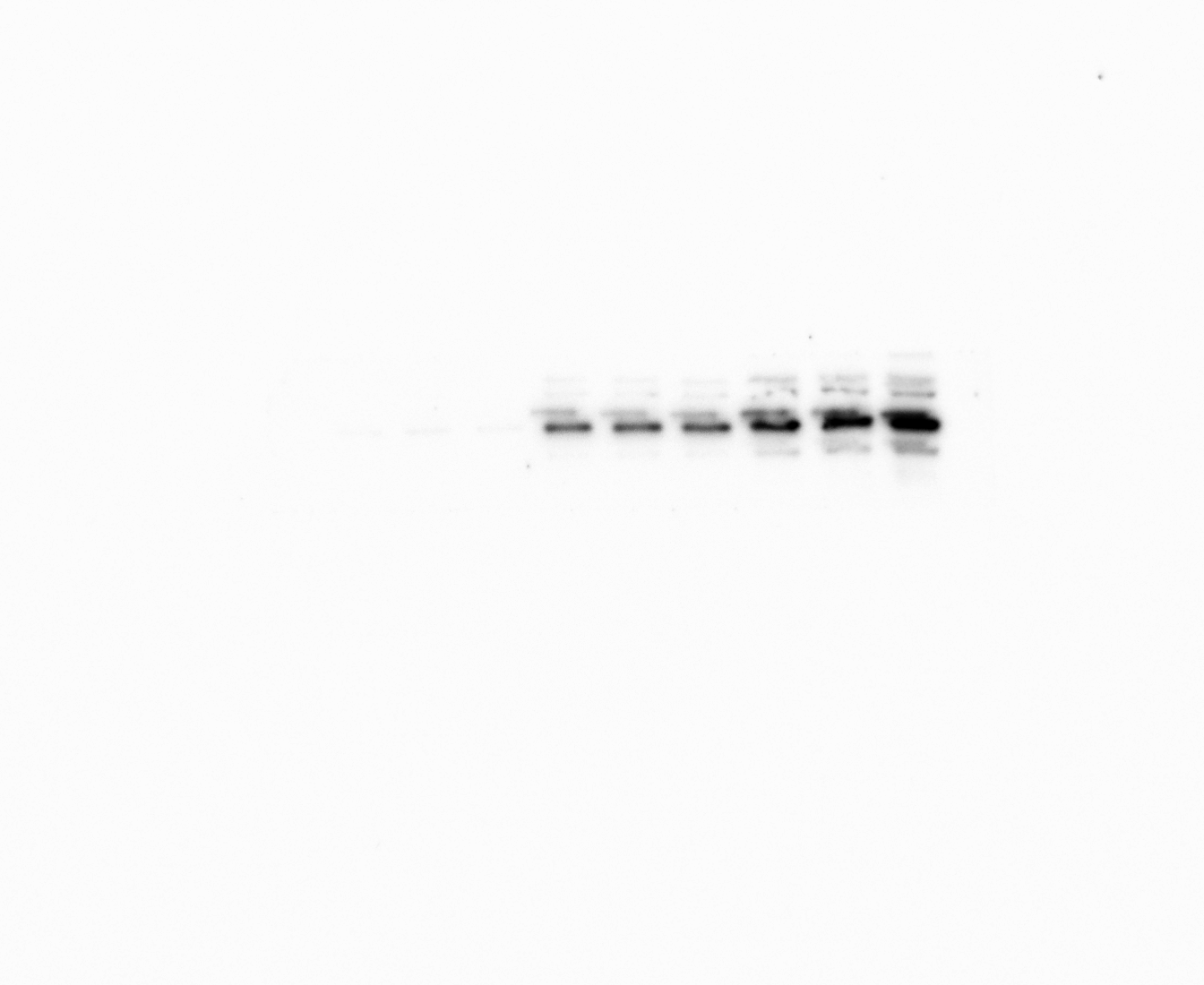

Supplement: Figure 3—source data 1. [file elife-98524-fig3-data1.zip › Fig 3-data1-v1/3H/FN1.tif]

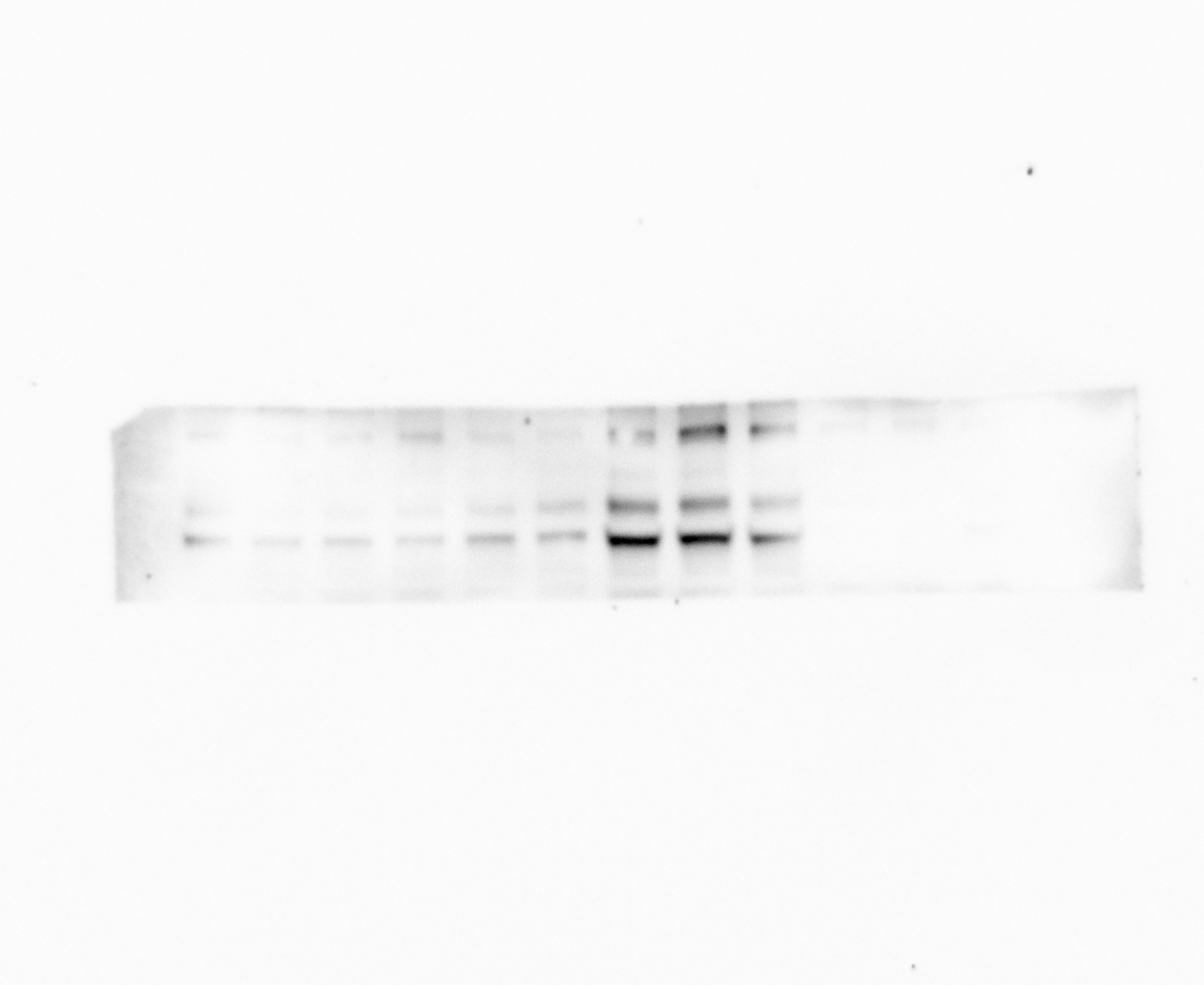

Supplement: Figure 3—source data 1. [file elife-98524-fig3-data1.zip › Fig 3-data1-v1/3H/SRIT4.tif]

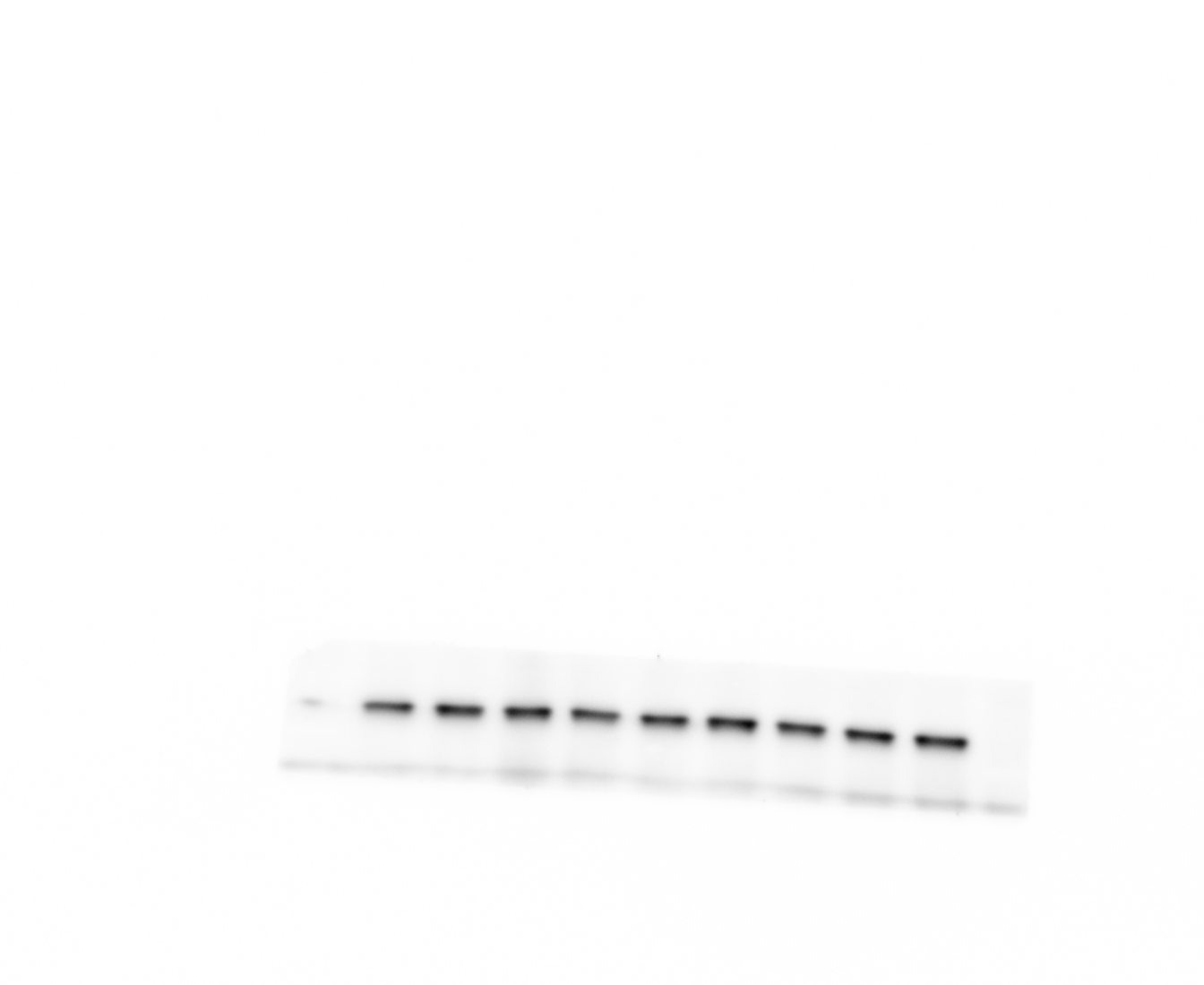

Supplement: Figure 3—source data 1. [file elife-98524-fig3-data1.zip › Fig 3-data1-v1/3H/Tubulin.tif]

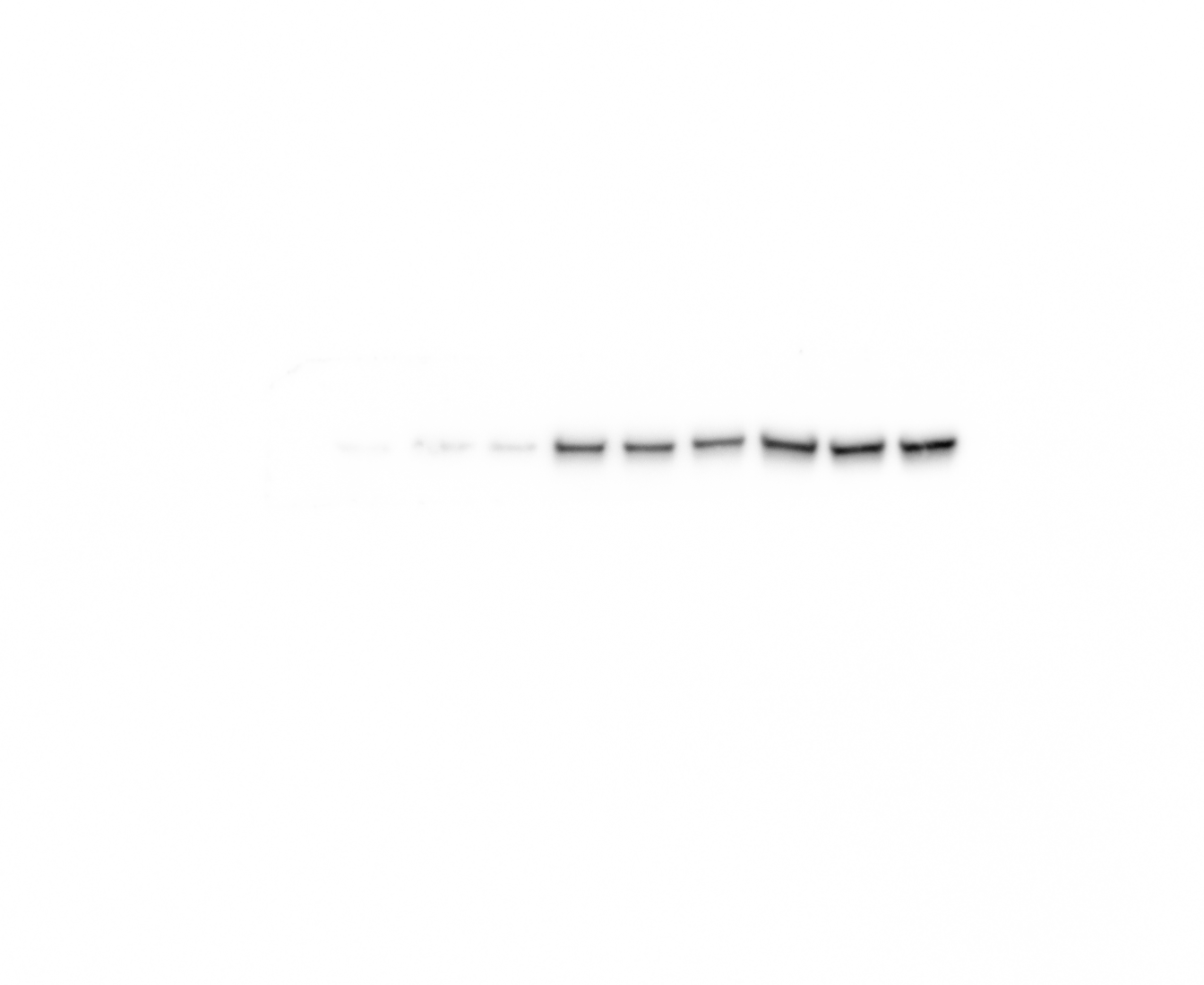

Supplement: Figure 3—source data 1. [file elife-98524-fig3-data1.zip › Fig 3-data1-v1/3H/α-SMA.tif]

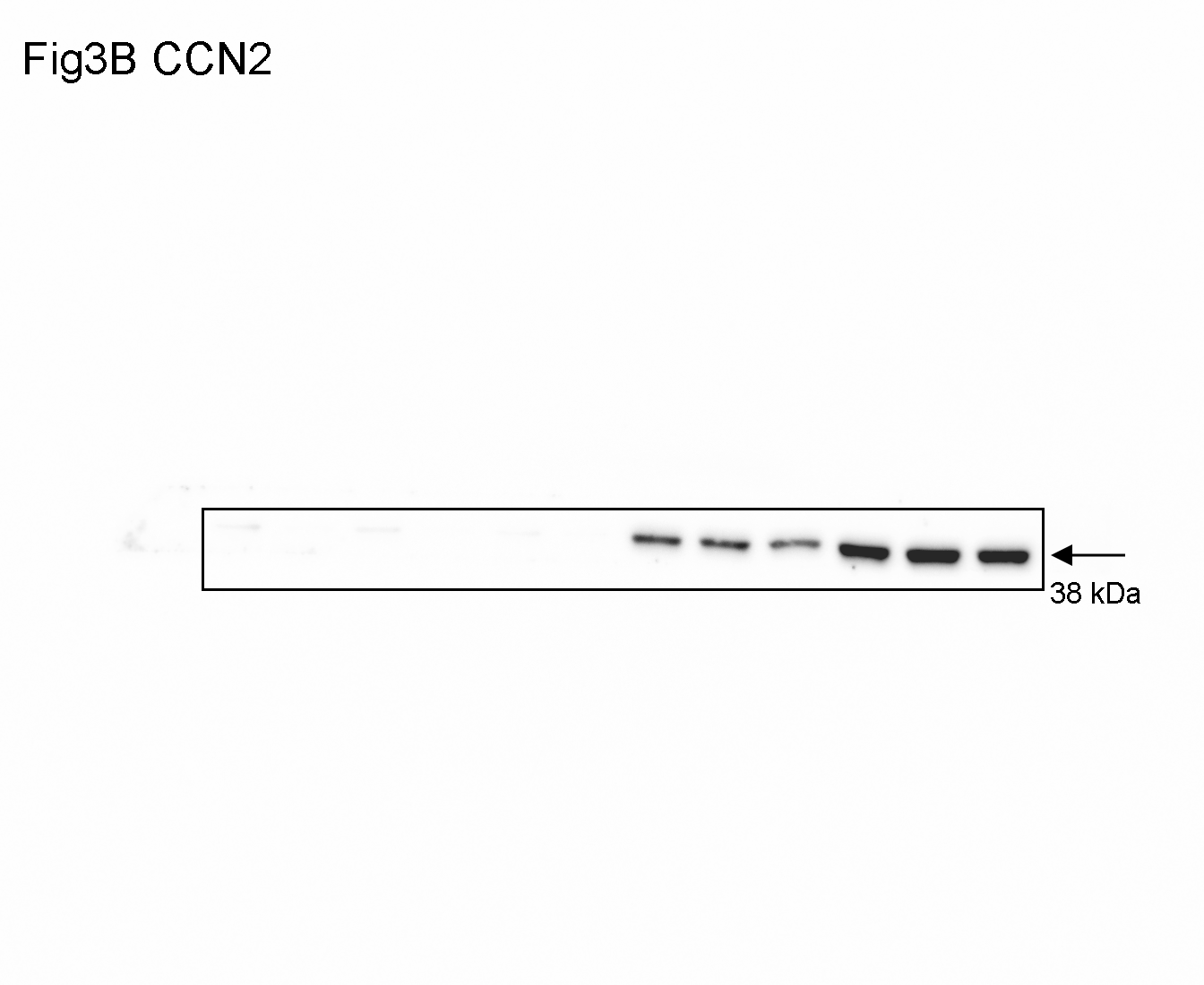

Supplement: Figure 3—source data 2. [file elife-98524-fig3-data2.zip › Fig 3-data2-v1/3B/CCN2.tif]

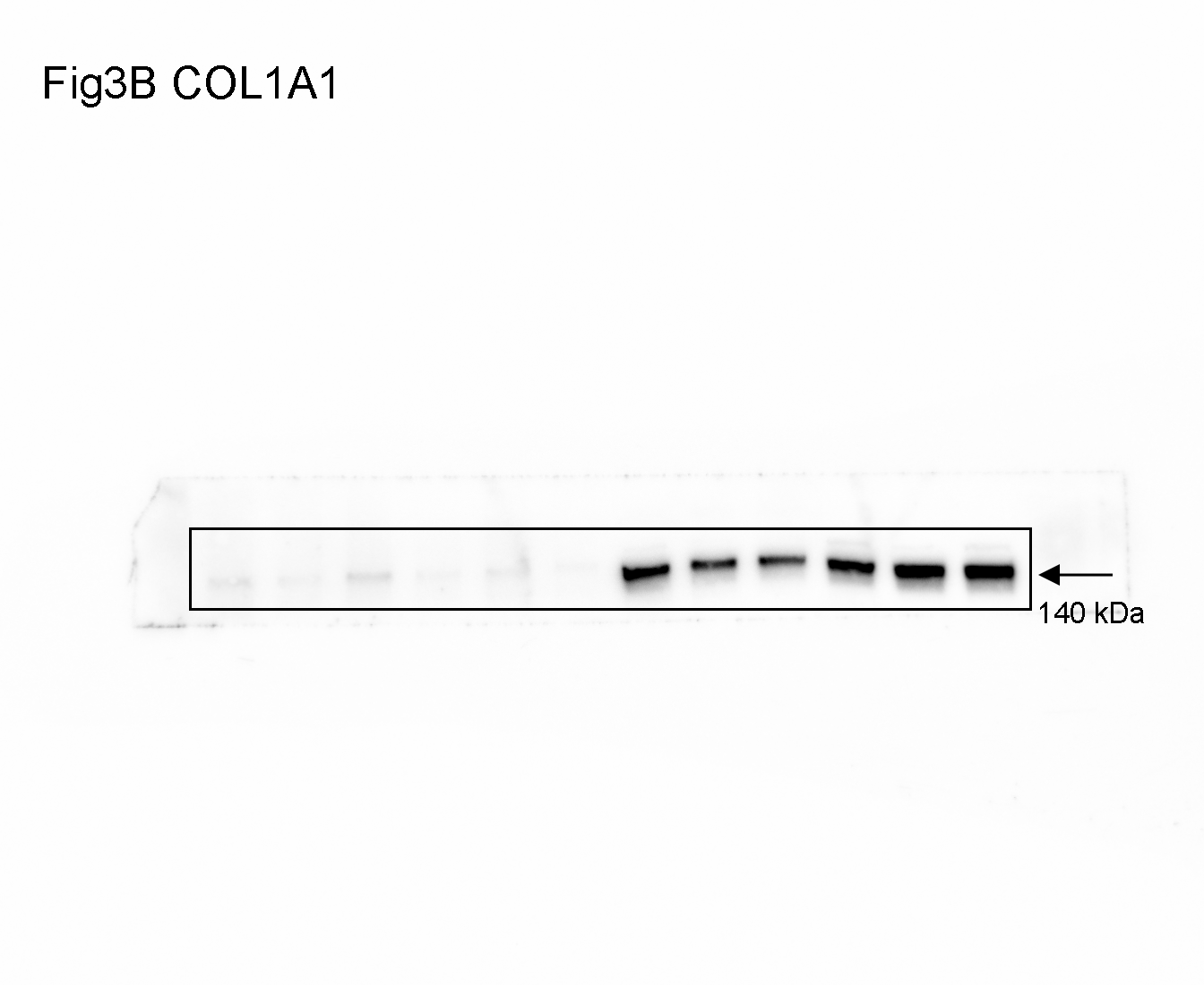

Supplement: Figure 3—source data 2. [file elife-98524-fig3-data2.zip › Fig 3-data2-v1/3B/COL1A1.tif]

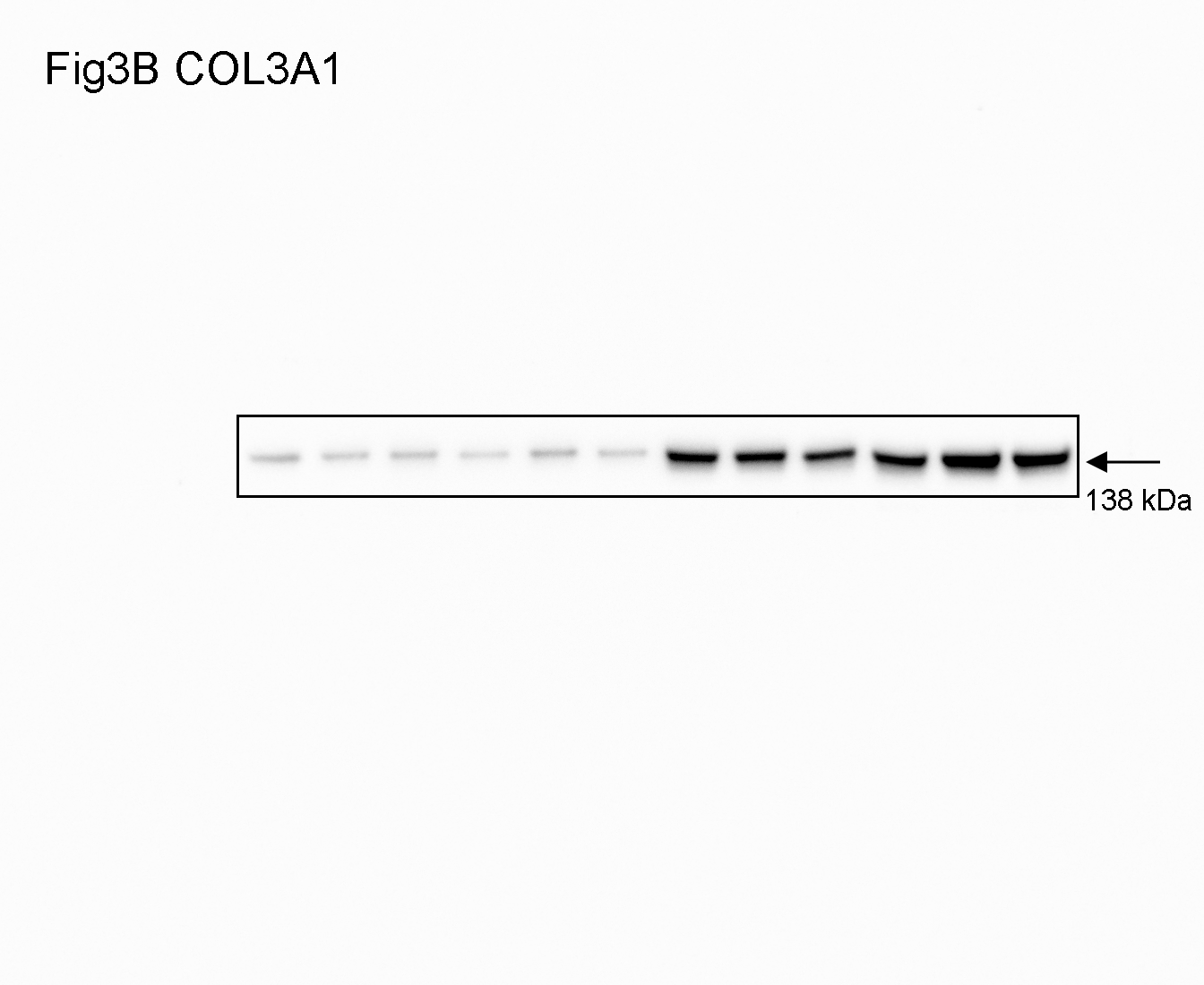

Supplement: Figure 3—source data 2. [file elife-98524-fig3-data2.zip › Fig 3-data2-v1/3B/COL3A1.tif]

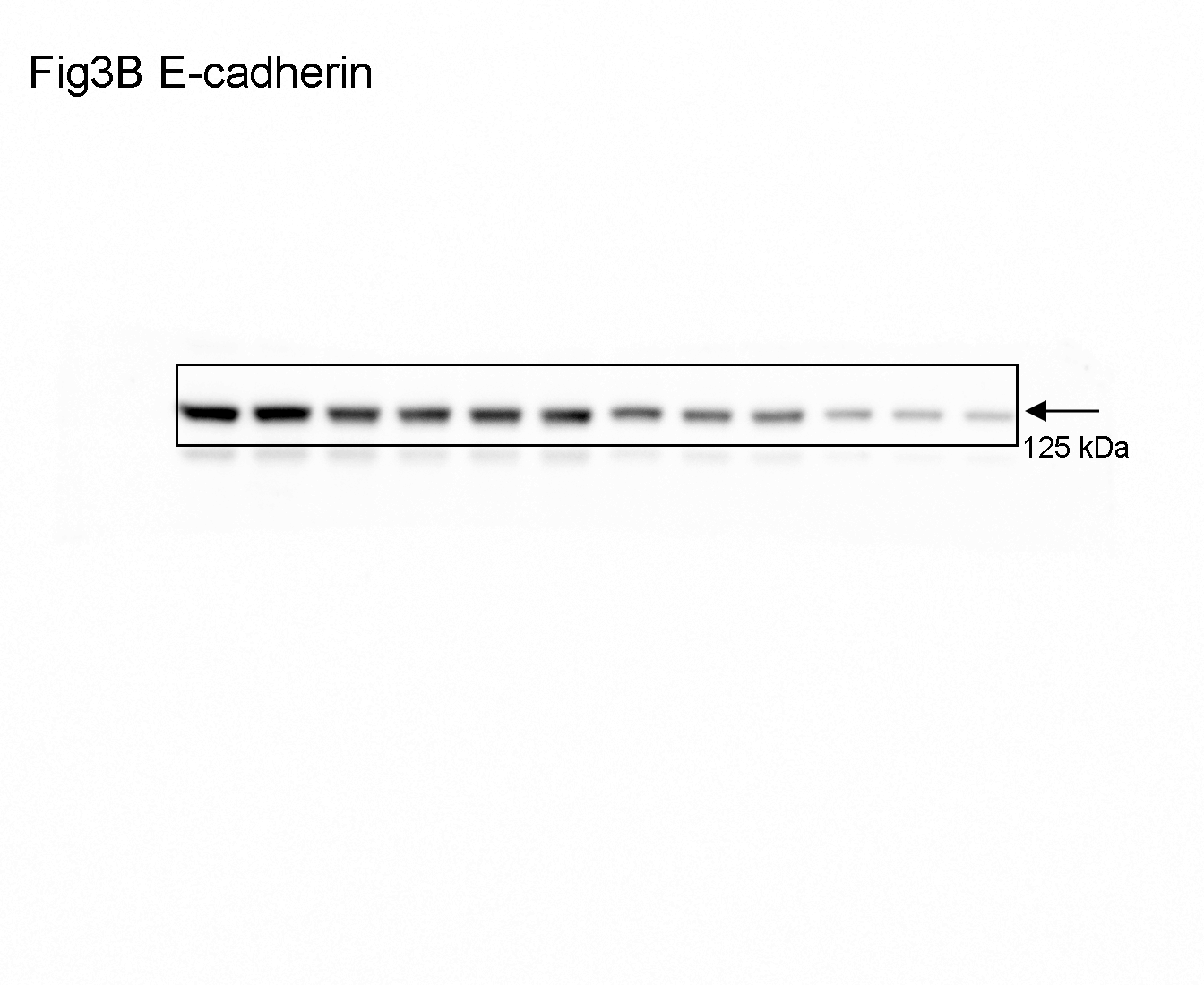

Supplement: Figure 3—source data 2. [file elife-98524-fig3-data2.zip › Fig 3-data2-v1/3B/E-cadherin.tif]

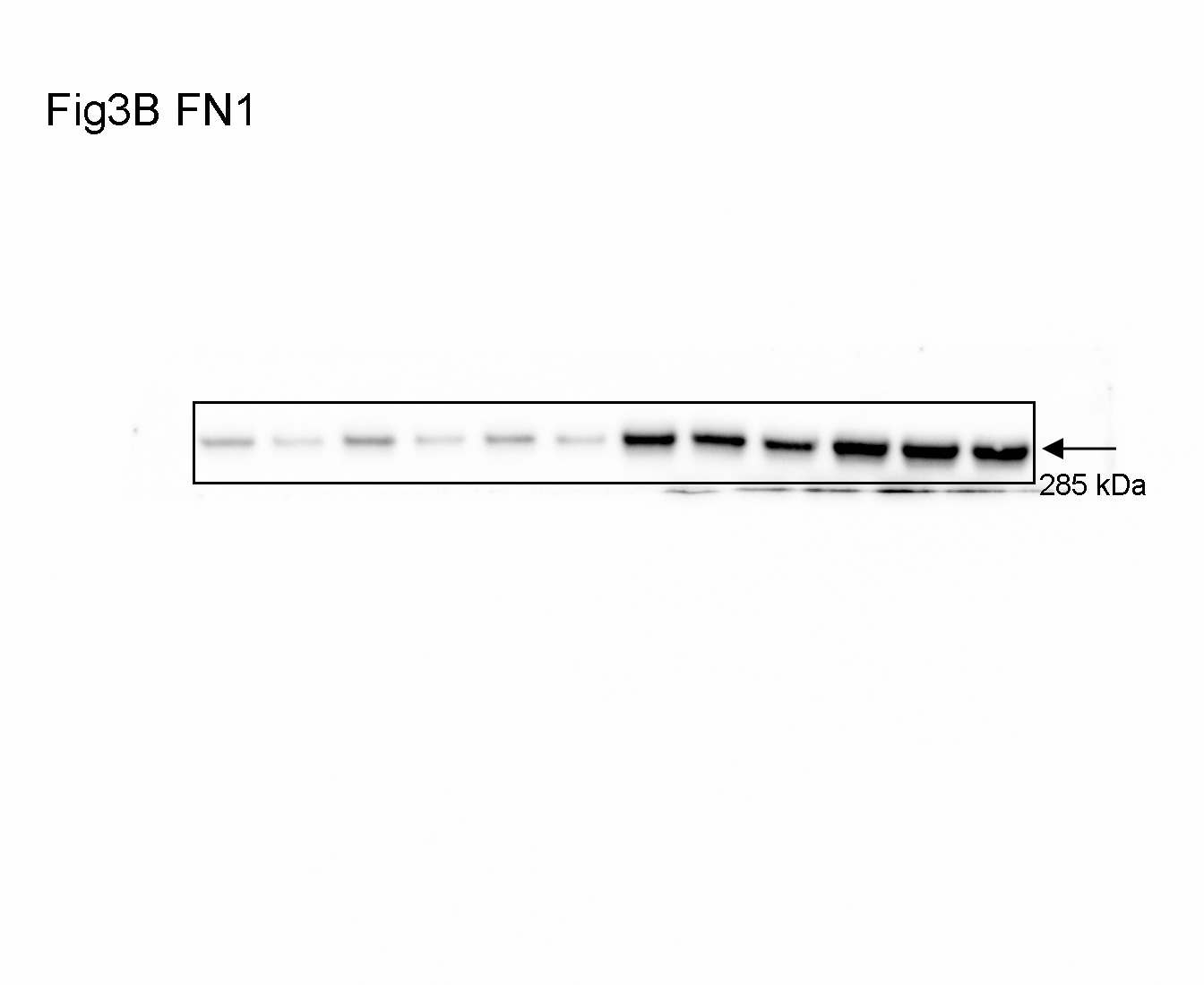

Supplement: Figure 3—source data 2. [file elife-98524-fig3-data2.zip › Fig 3-data2-v1/3B/FN1.tif]

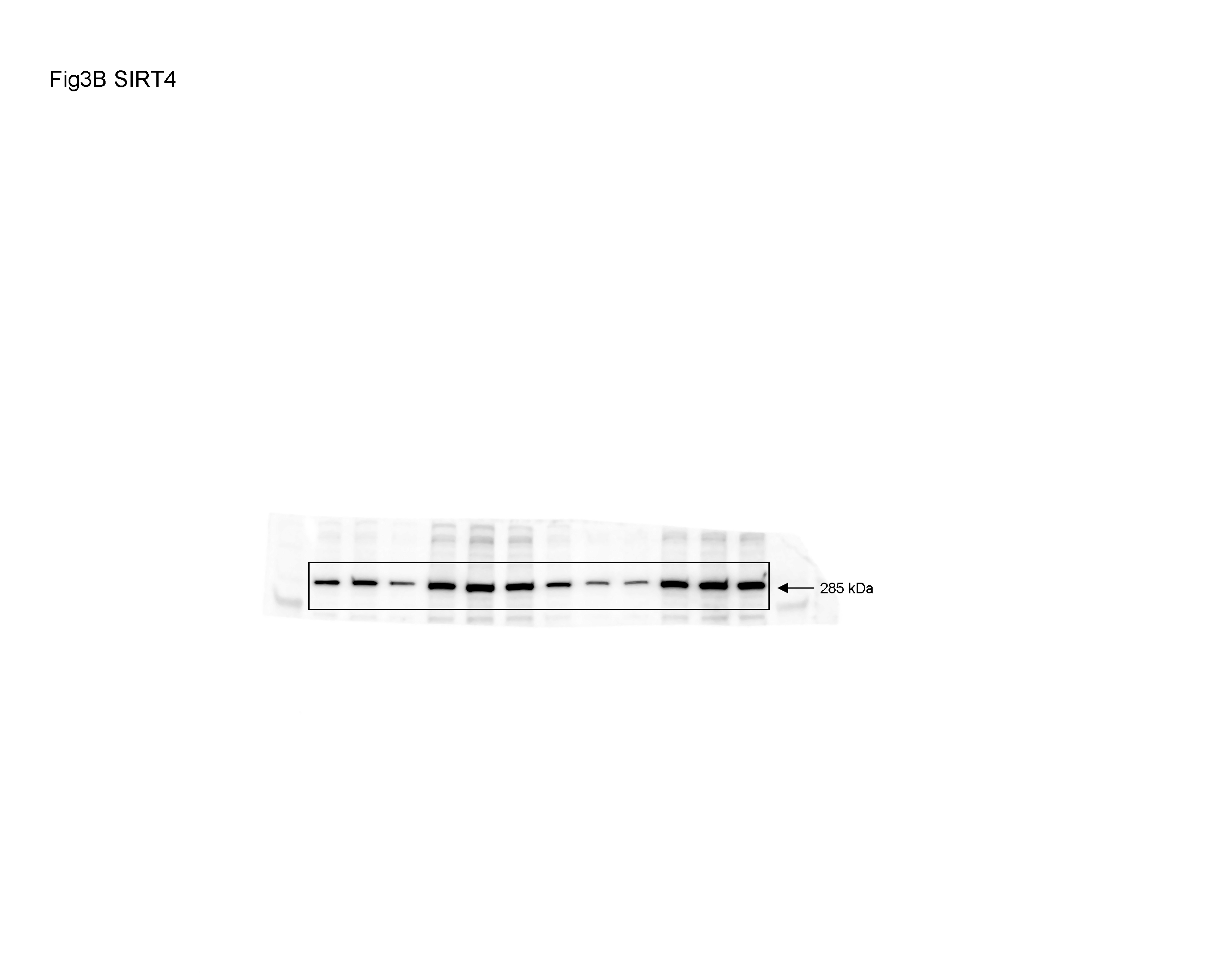

Supplement: Figure 3—source data 2. [file elife-98524-fig3-data2.zip › Fig 3-data2-v1/3B/SIRT4.tif]

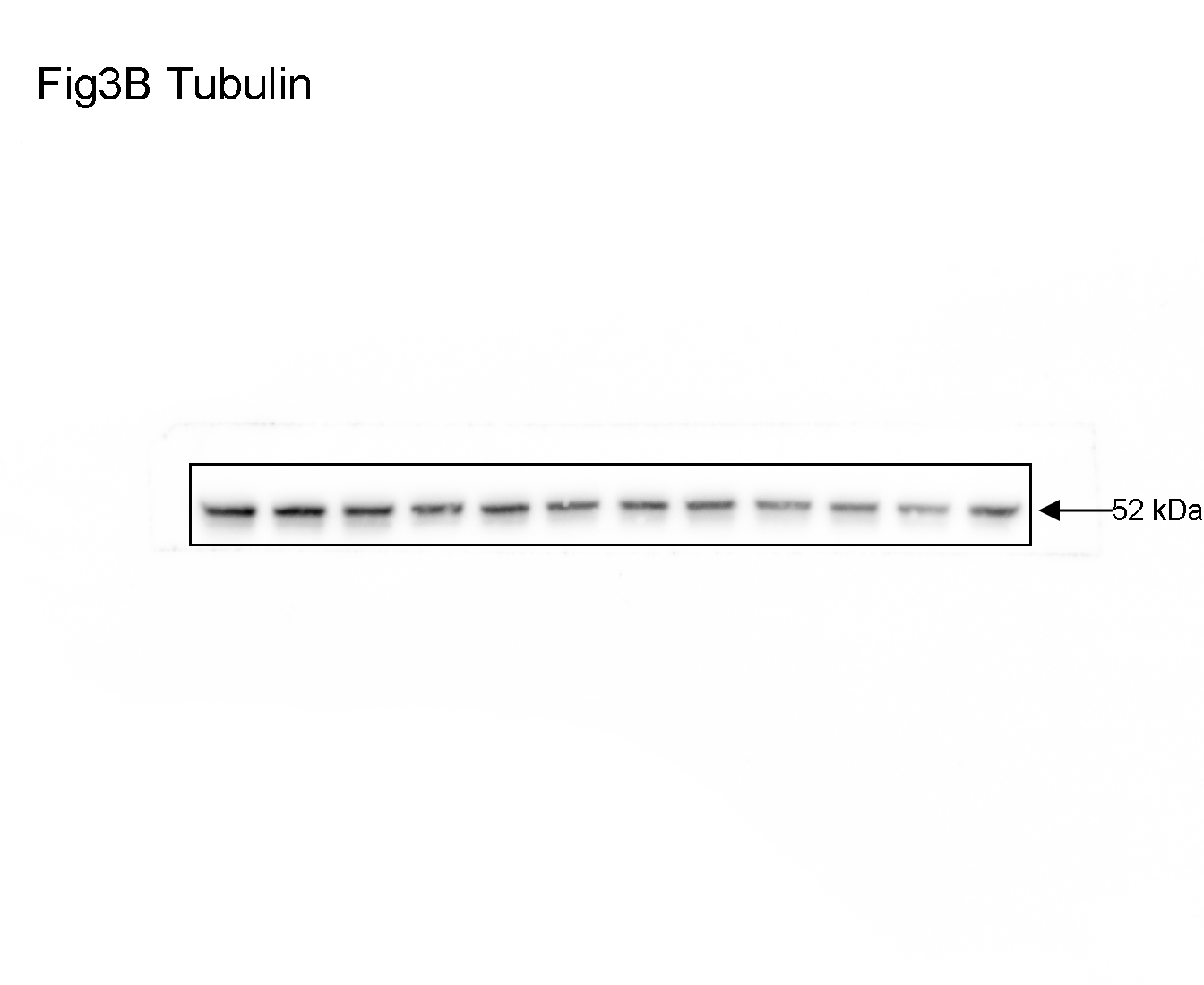

Supplement: Figure 3—source data 2. [file elife-98524-fig3-data2.zip › Fig 3-data2-v1/3B/Tubulin.tif]

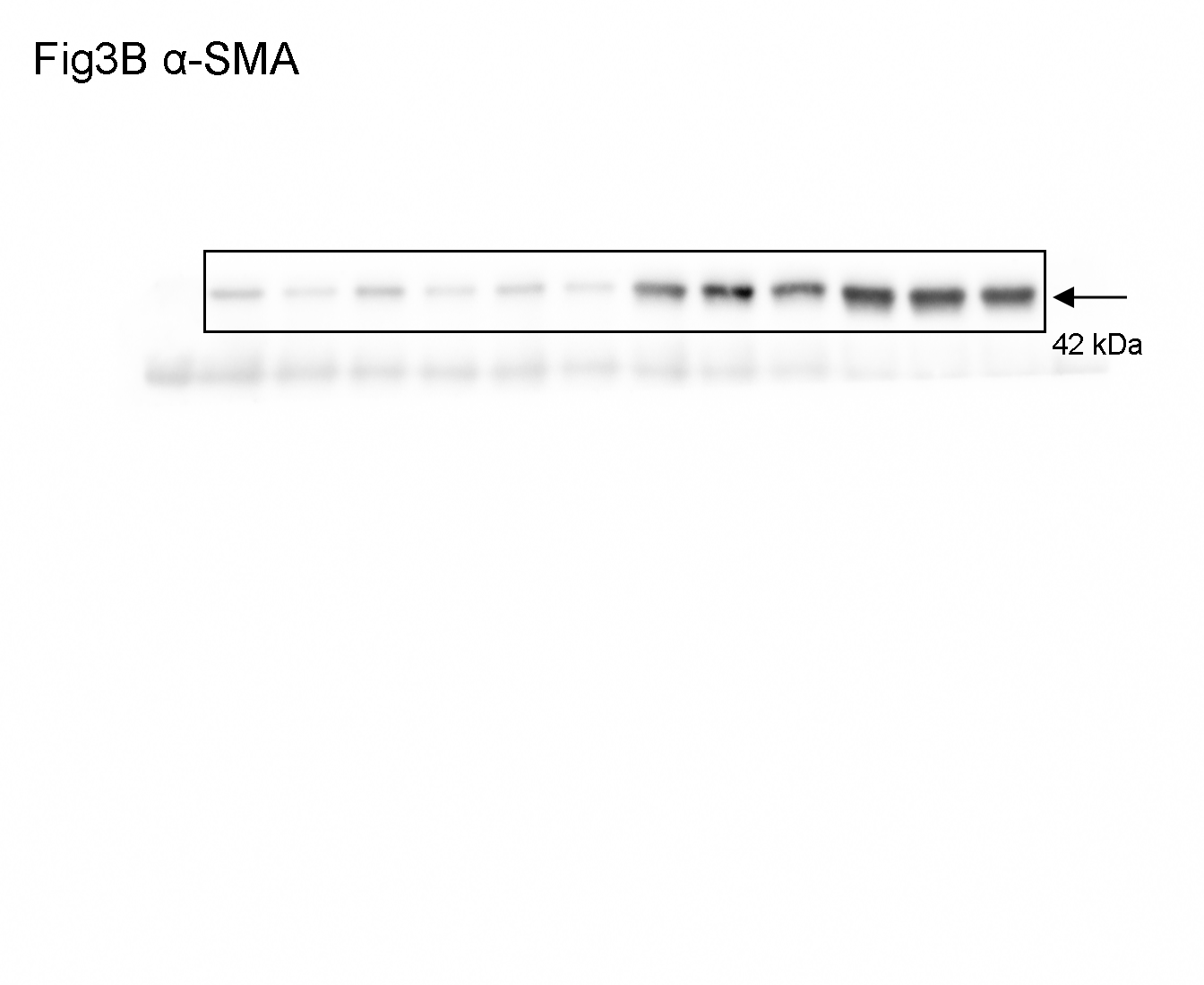

Supplement: Figure 3—source data 2. [file elife-98524-fig3-data2.zip › Fig 3-data2-v1/3B/α-SMA.tif]

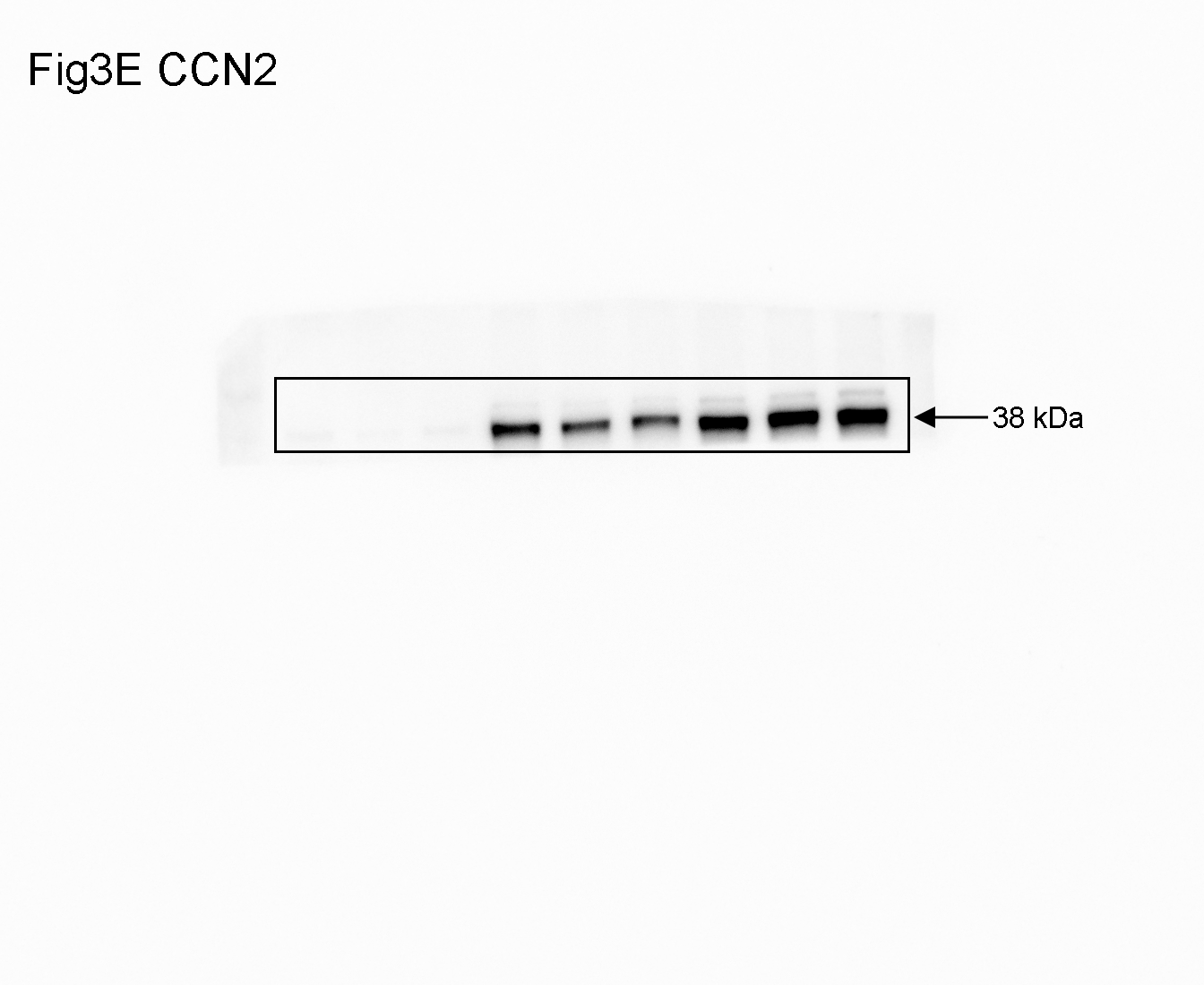

Supplement: Figure 3—source data 2. [file elife-98524-fig3-data2.zip › Fig 3-data2-v1/3E/CCN2.tif]

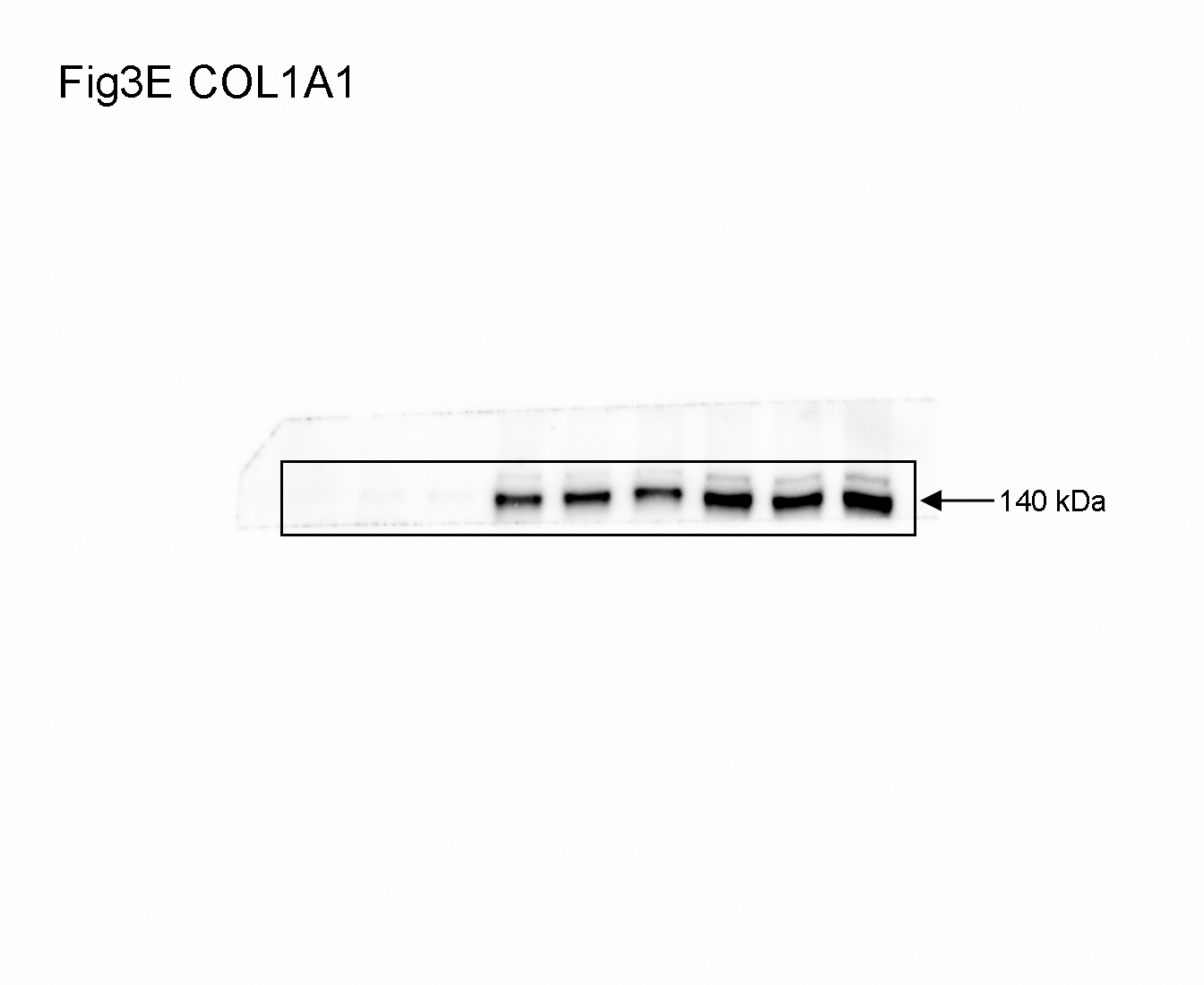

Supplement: Figure 3—source data 2. [file elife-98524-fig3-data2.zip › Fig 3-data2-v1/3E/COL1A1.tif]

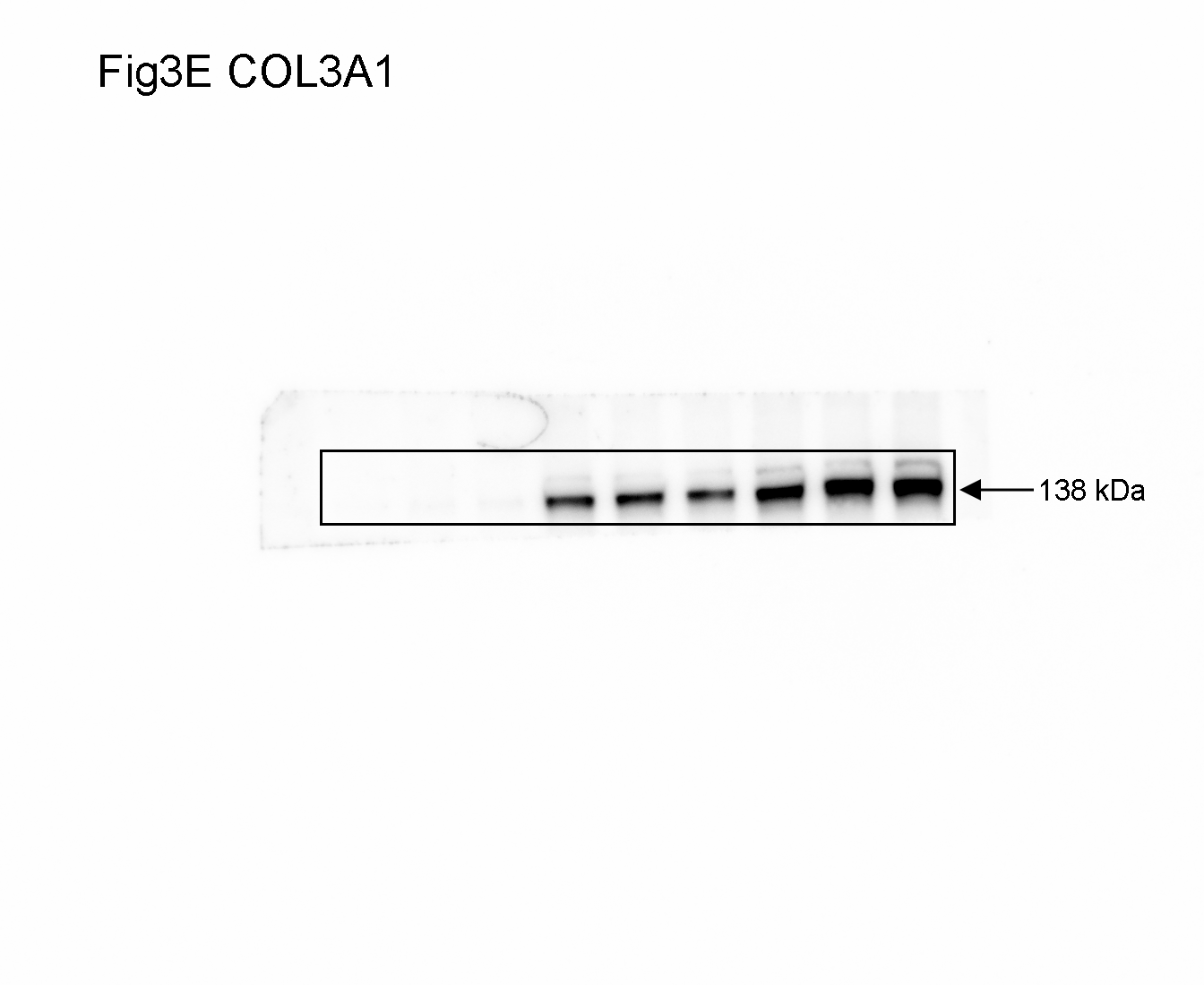

Supplement: Figure 3—source data 2. [file elife-98524-fig3-data2.zip › Fig 3-data2-v1/3E/COL3A1.tif]

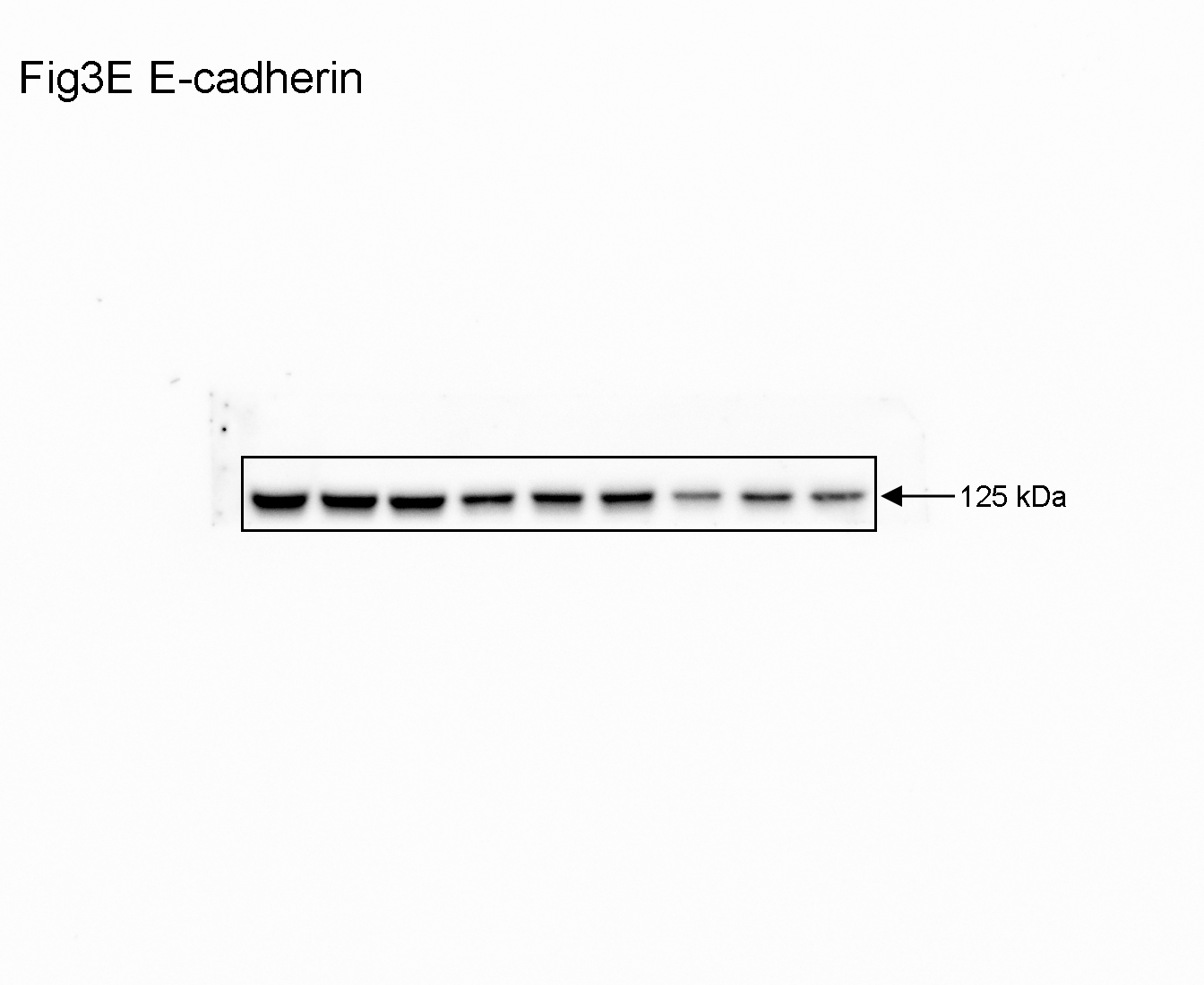

Supplement: Figure 3—source data 2. [file elife-98524-fig3-data2.zip › Fig 3-data2-v1/3E/E-cadherin.tif]

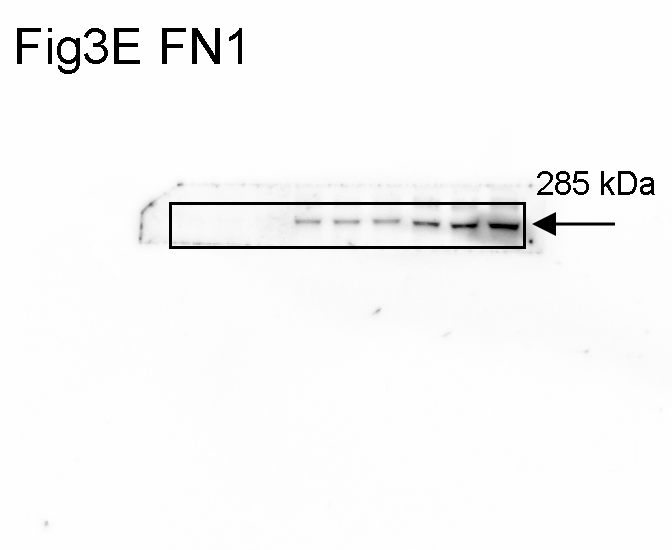

Supplement: Figure 3—source data 2. [file elife-98524-fig3-data2.zip › Fig 3-data2-v1/3E/FN1.tif]

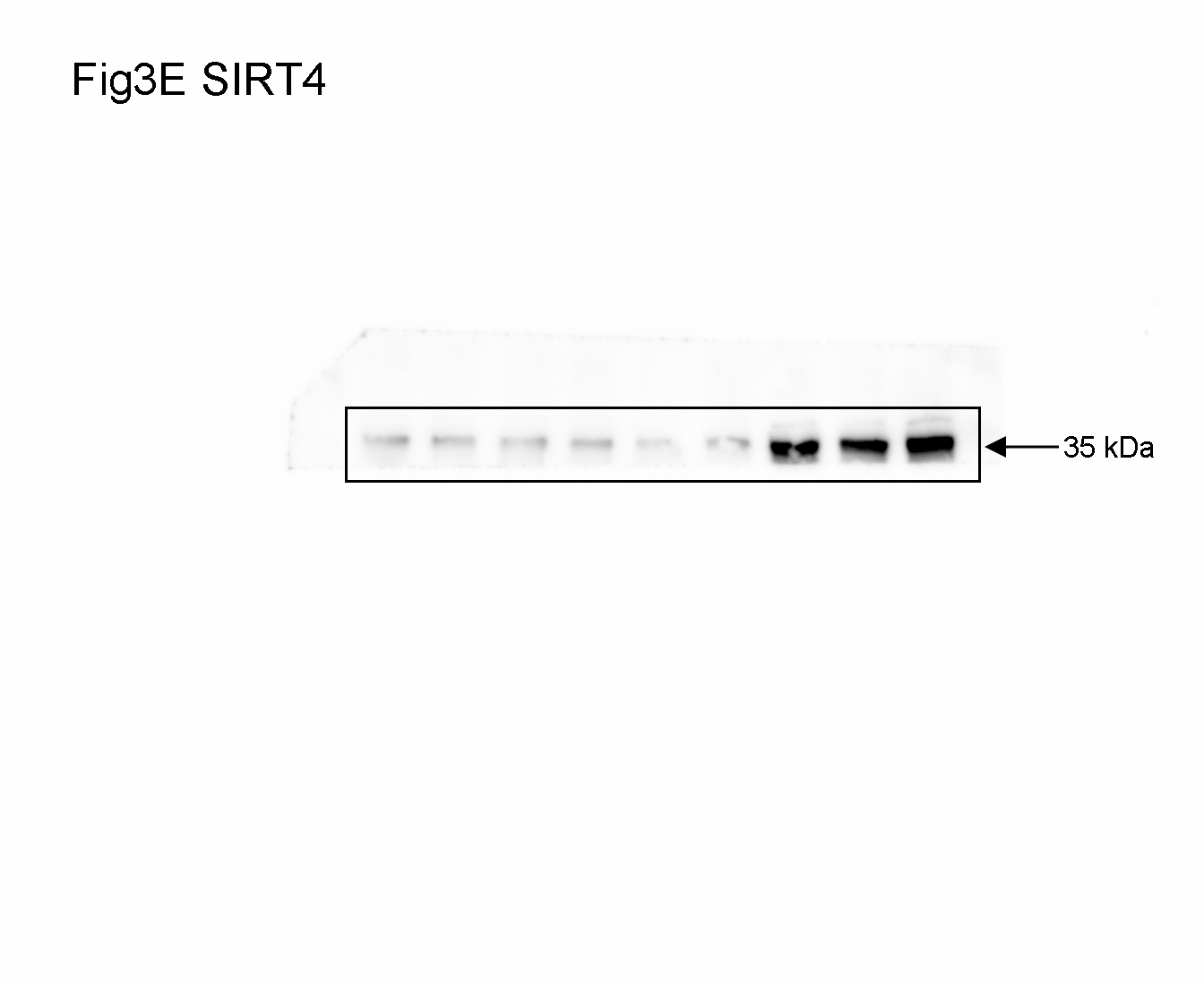

Supplement: Figure 3—source data 2. [file elife-98524-fig3-data2.zip › Fig 3-data2-v1/3E/SIRT4.tif]

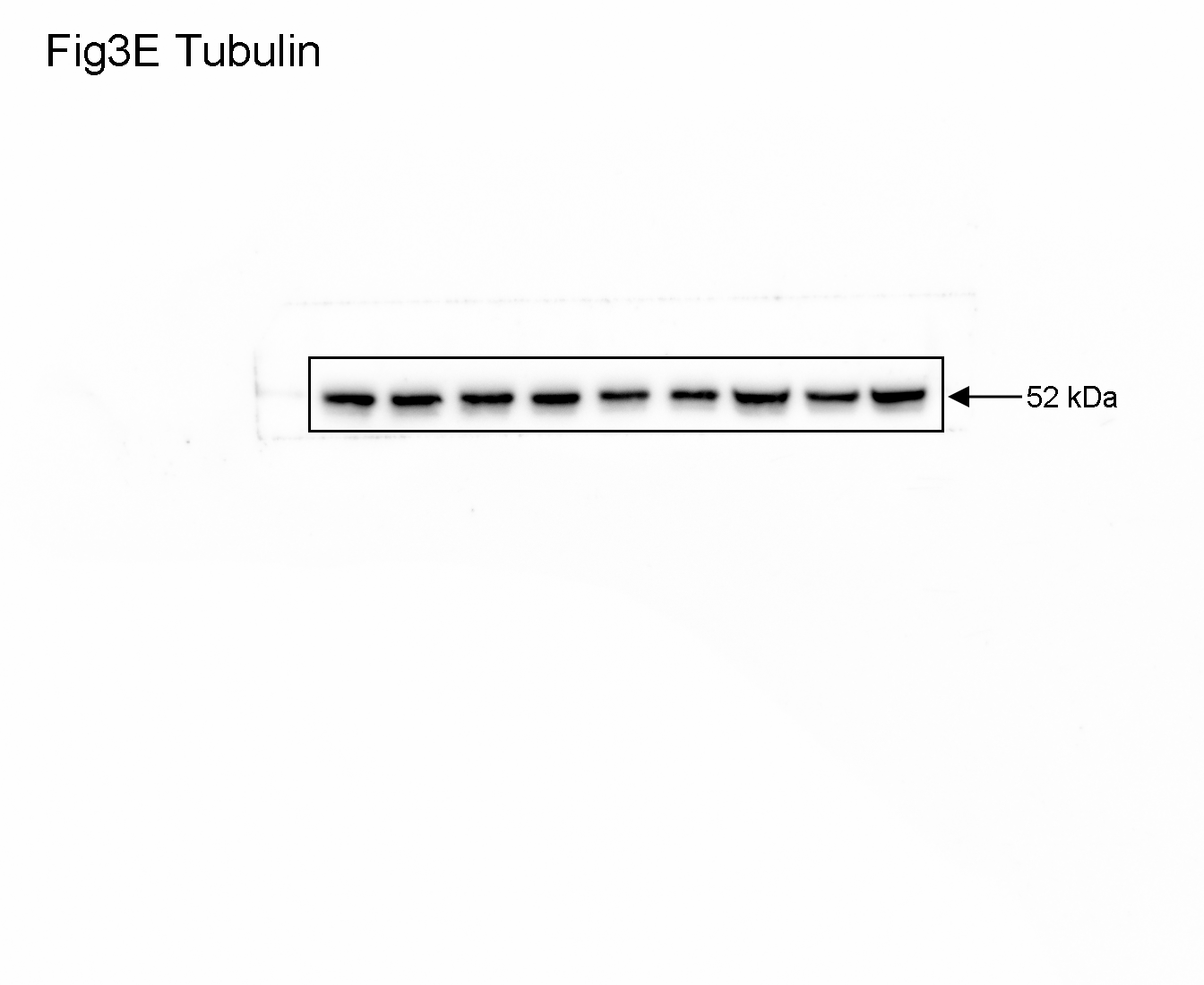

Supplement: Figure 3—source data 2. [file elife-98524-fig3-data2.zip › Fig 3-data2-v1/3E/Tubulin.tif]

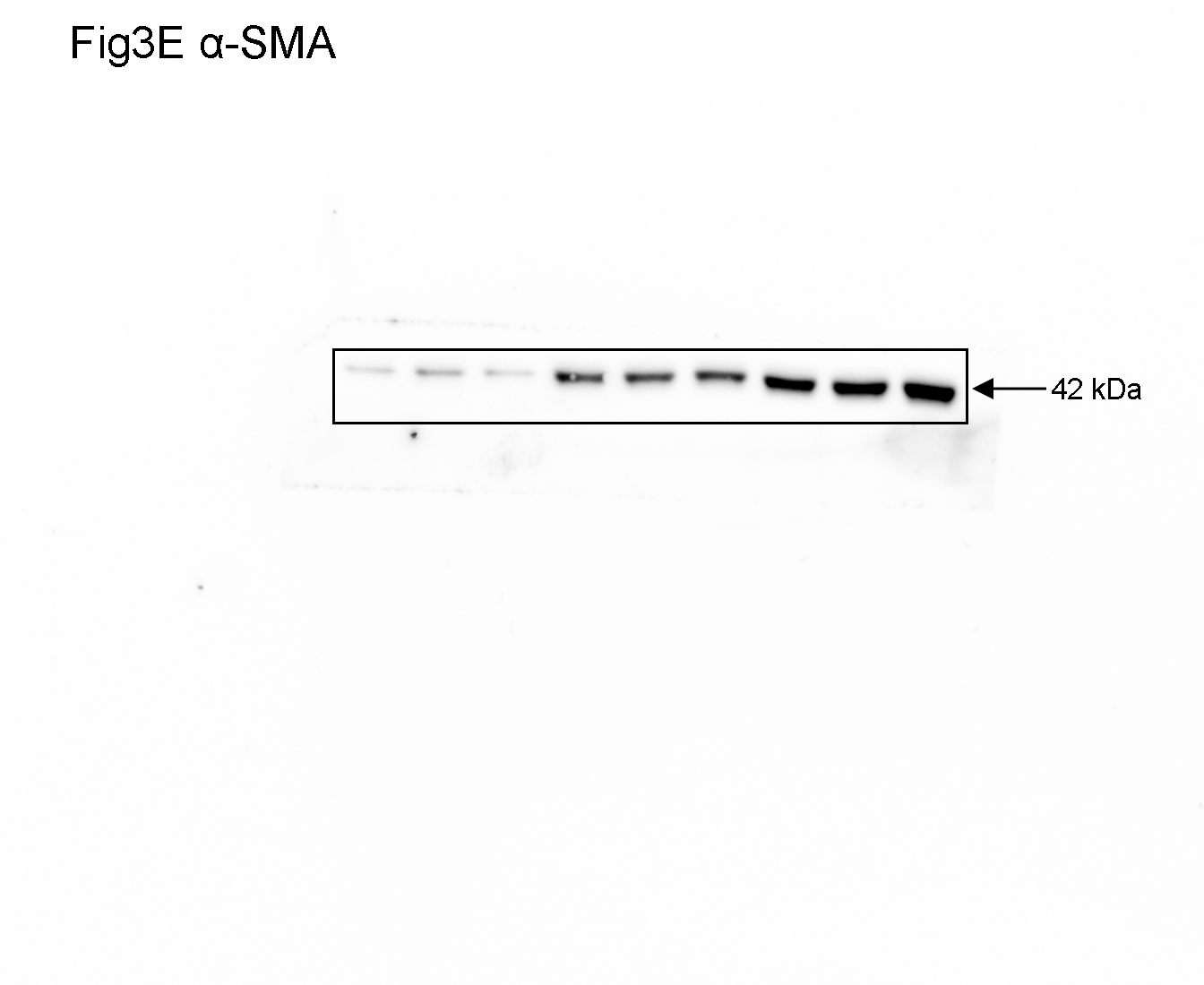

Supplement: Figure 3—source data 2. [file elife-98524-fig3-data2.zip › Fig 3-data2-v1/3E/α-SMA.tif]

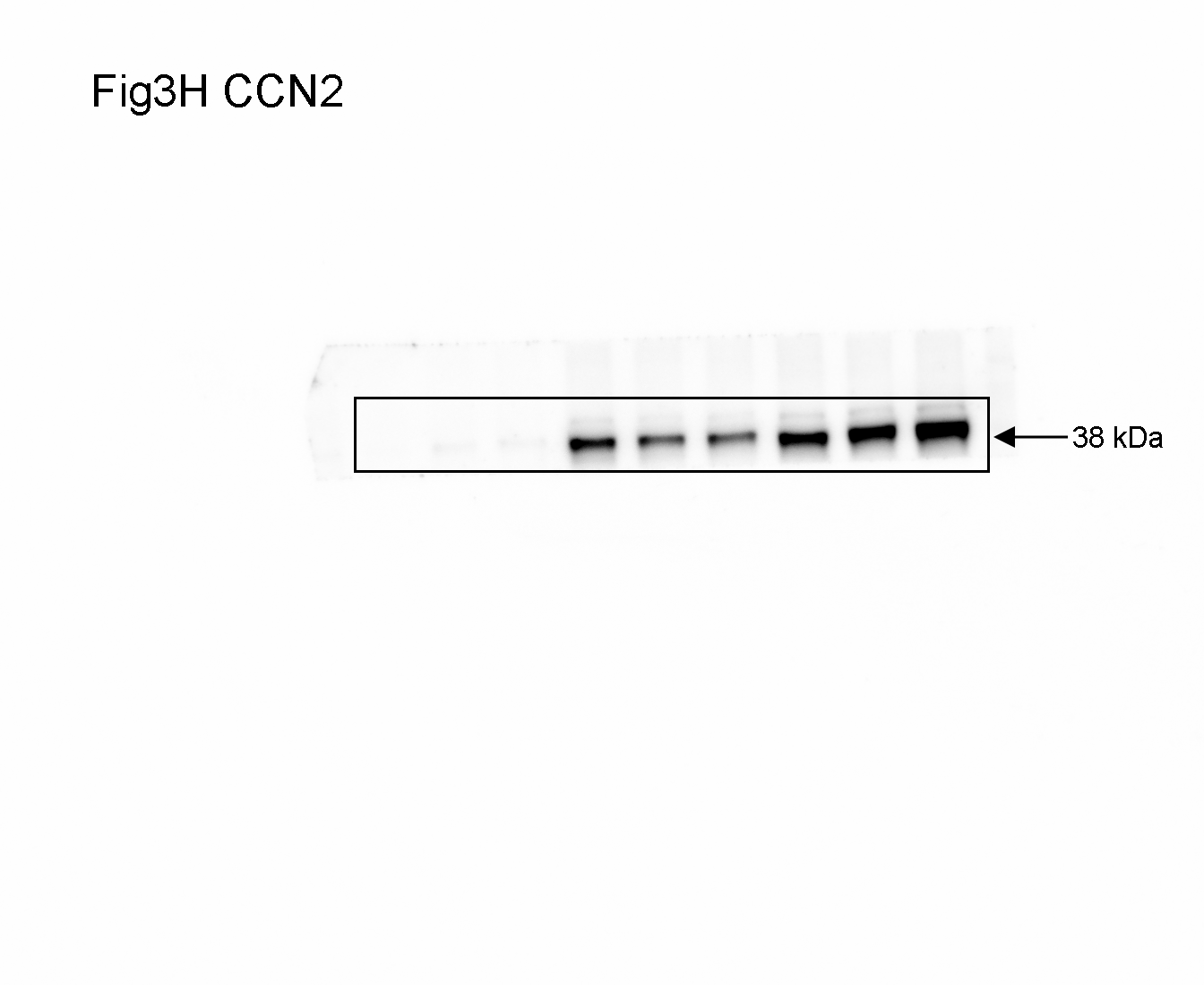

Supplement: Figure 3—source data 2. [file elife-98524-fig3-data2.zip › Fig 3-data2-v1/3H/CCN2.tif]

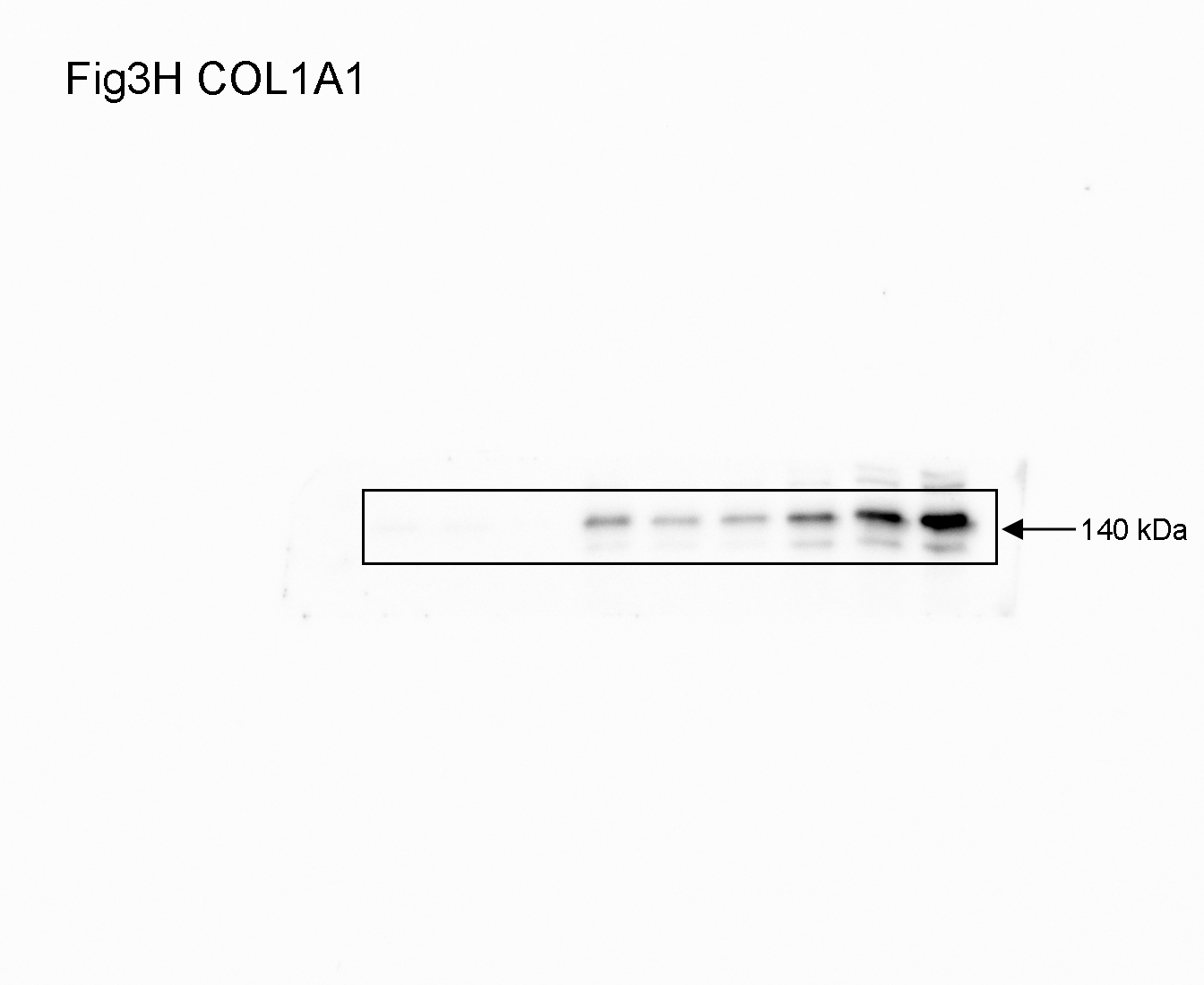

Supplement: Figure 3—source data 2. [file elife-98524-fig3-data2.zip › Fig 3-data2-v1/3H/COL1A1.tif]

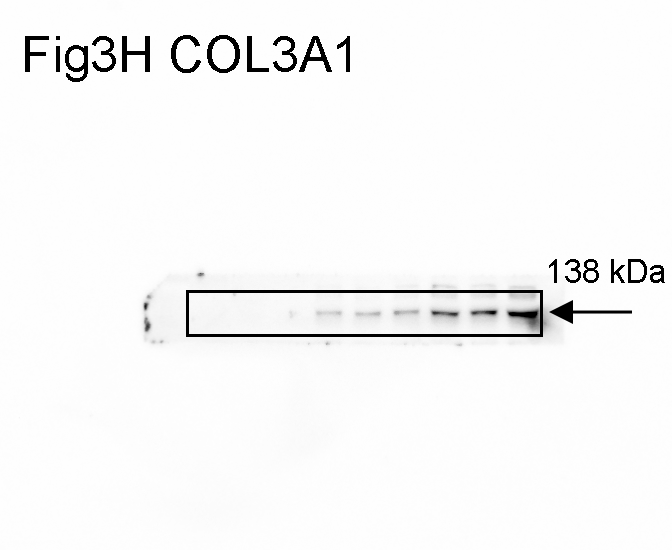

Supplement: Figure 3—source data 2. [file elife-98524-fig3-data2.zip › Fig 3-data2-v1/3H/COL3A1.tif]

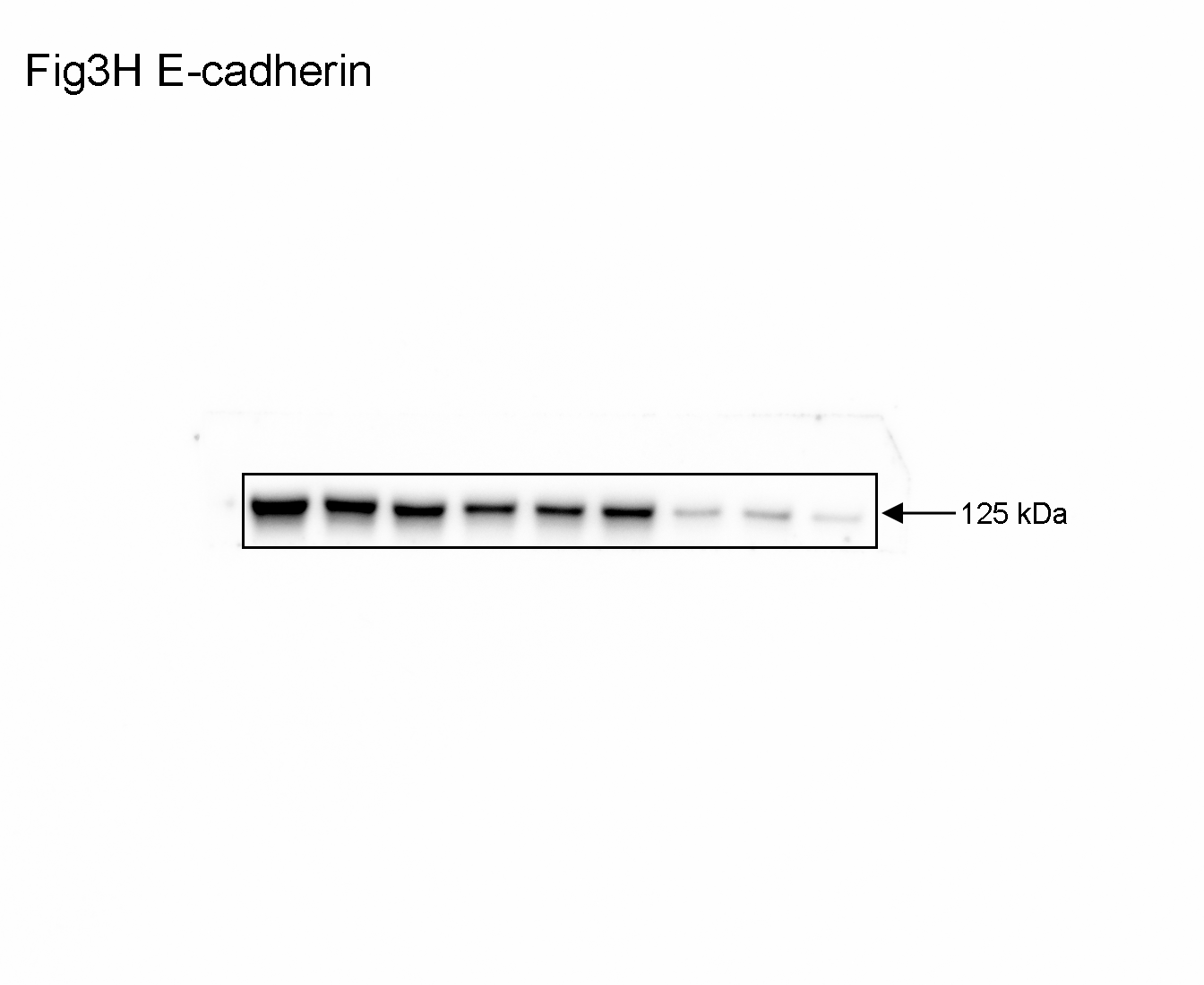

Supplement: Figure 3—source data 2. [file elife-98524-fig3-data2.zip › Fig 3-data2-v1/3H/E-cadherin.tif]

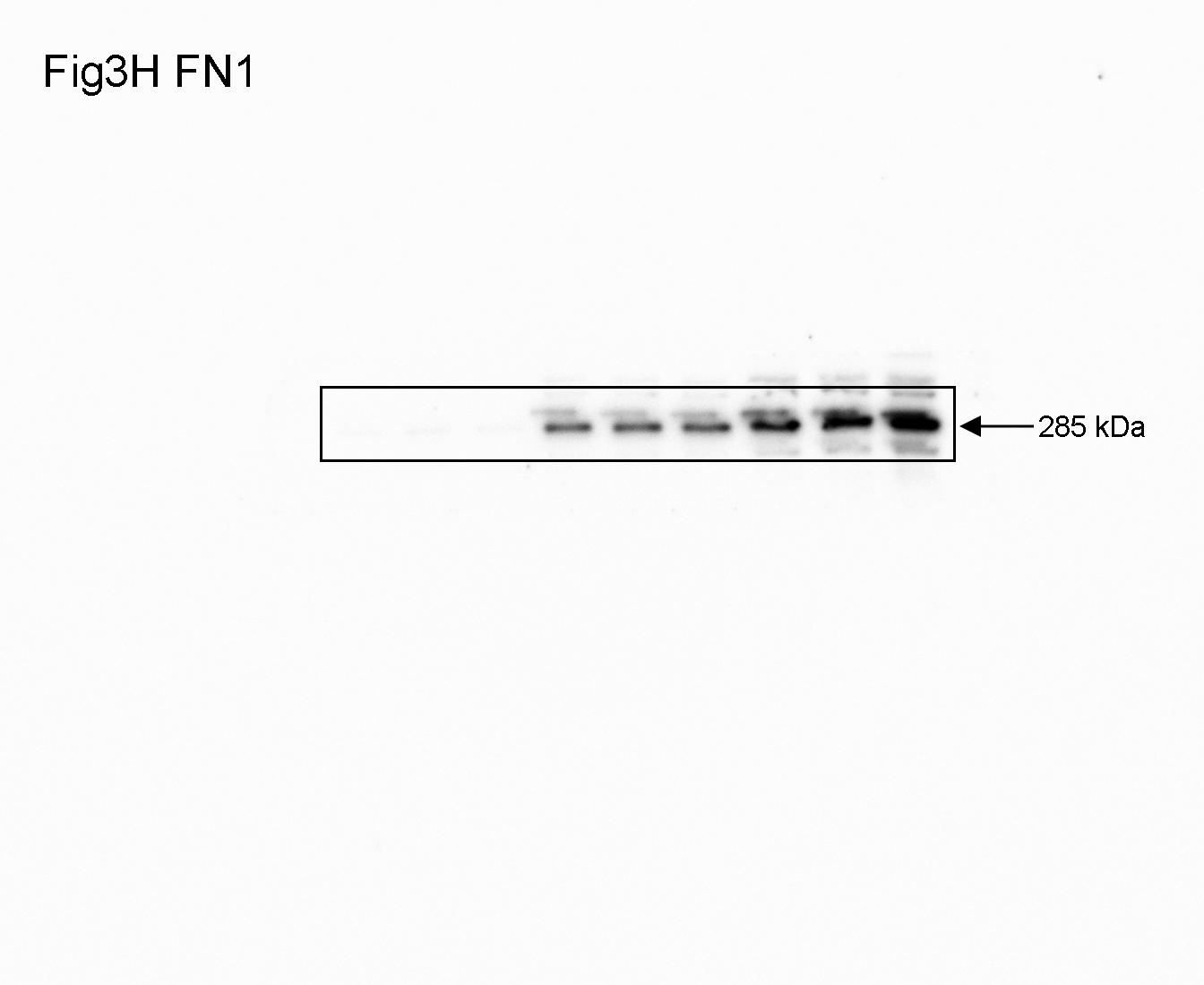

Supplement: Figure 3—source data 2. [file elife-98524-fig3-data2.zip › Fig 3-data2-v1/3H/FN1.tif]

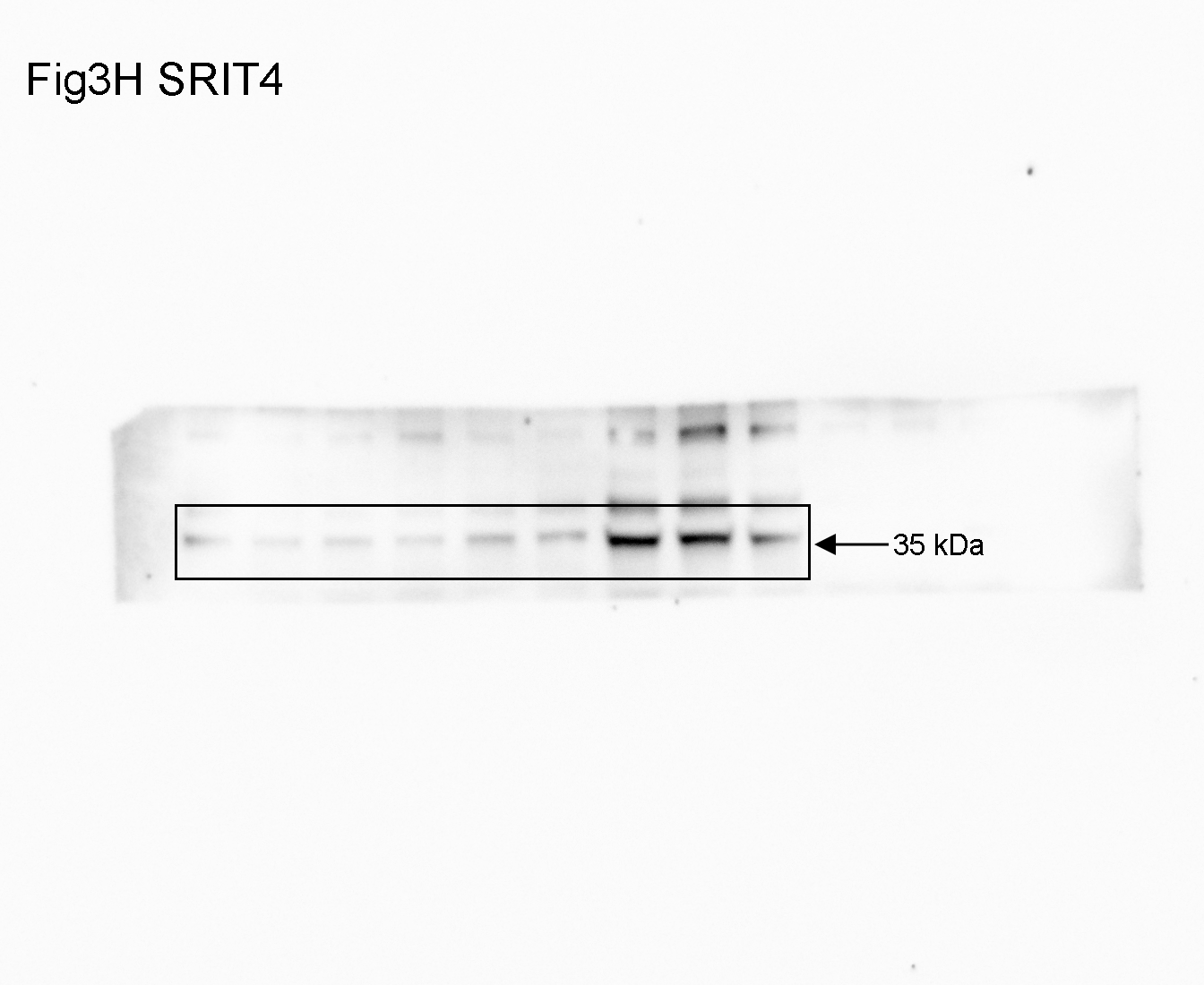

Supplement: Figure 3—source data 2. [file elife-98524-fig3-data2.zip › Fig 3-data2-v1/3H/SRIT4.tif]

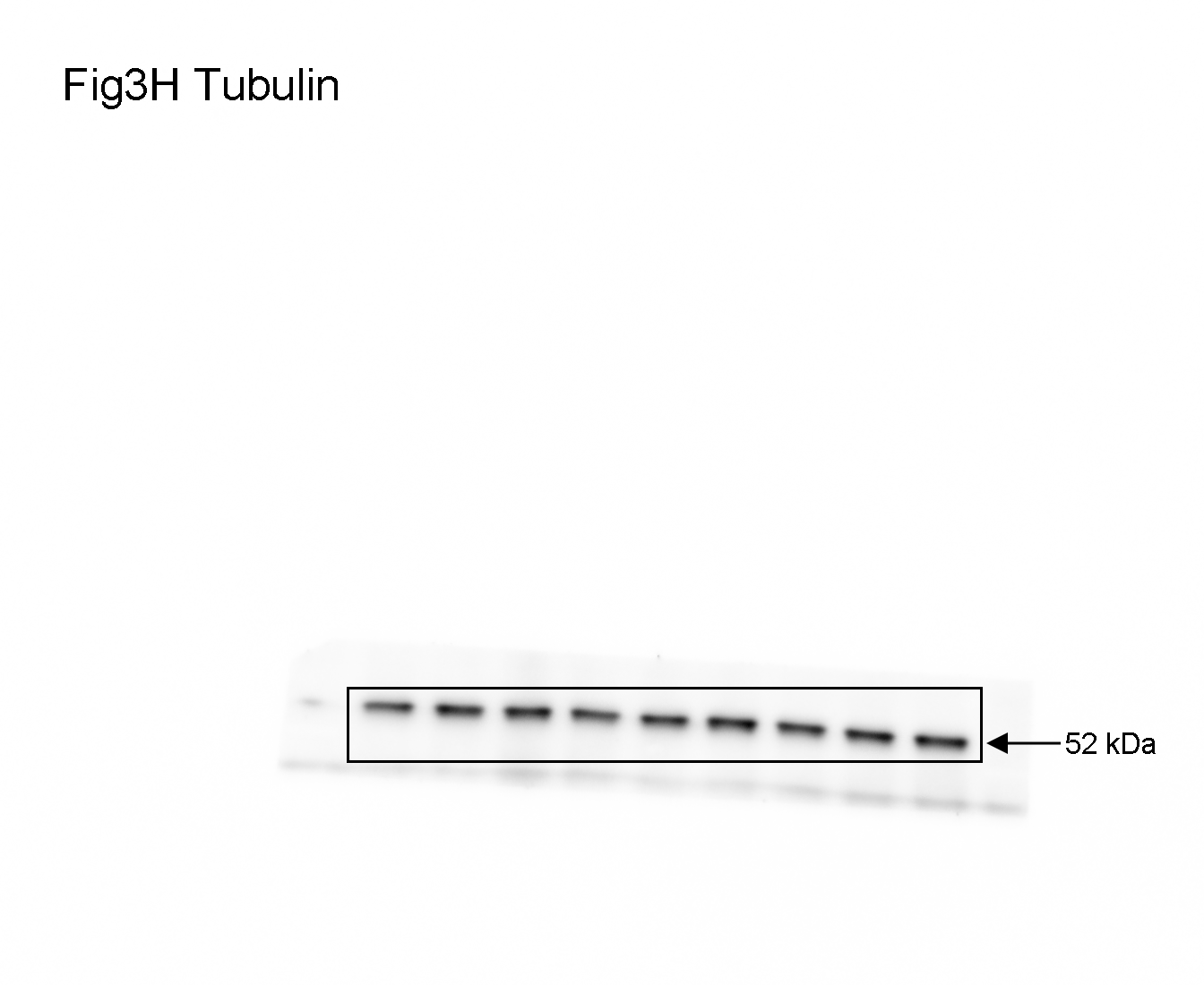

Supplement: Figure 3—source data 2. [file elife-98524-fig3-data2.zip › Fig 3-data2-v1/3H/Tubulin.tif]

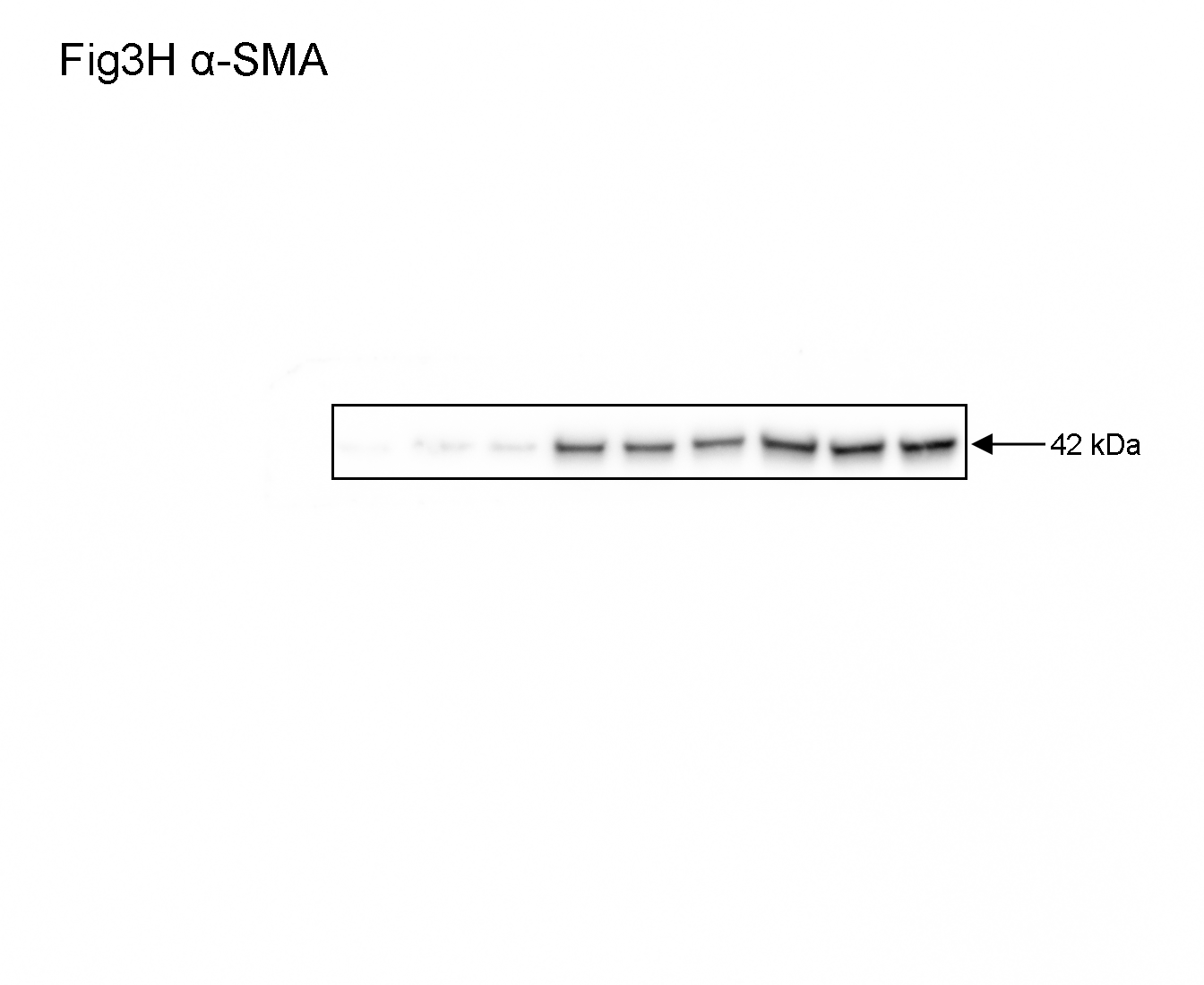

Supplement: Figure 3—source data 2. [file elife-98524-fig3-data2.zip › Fig 3-data2-v1/3H/α-SMA.tif]

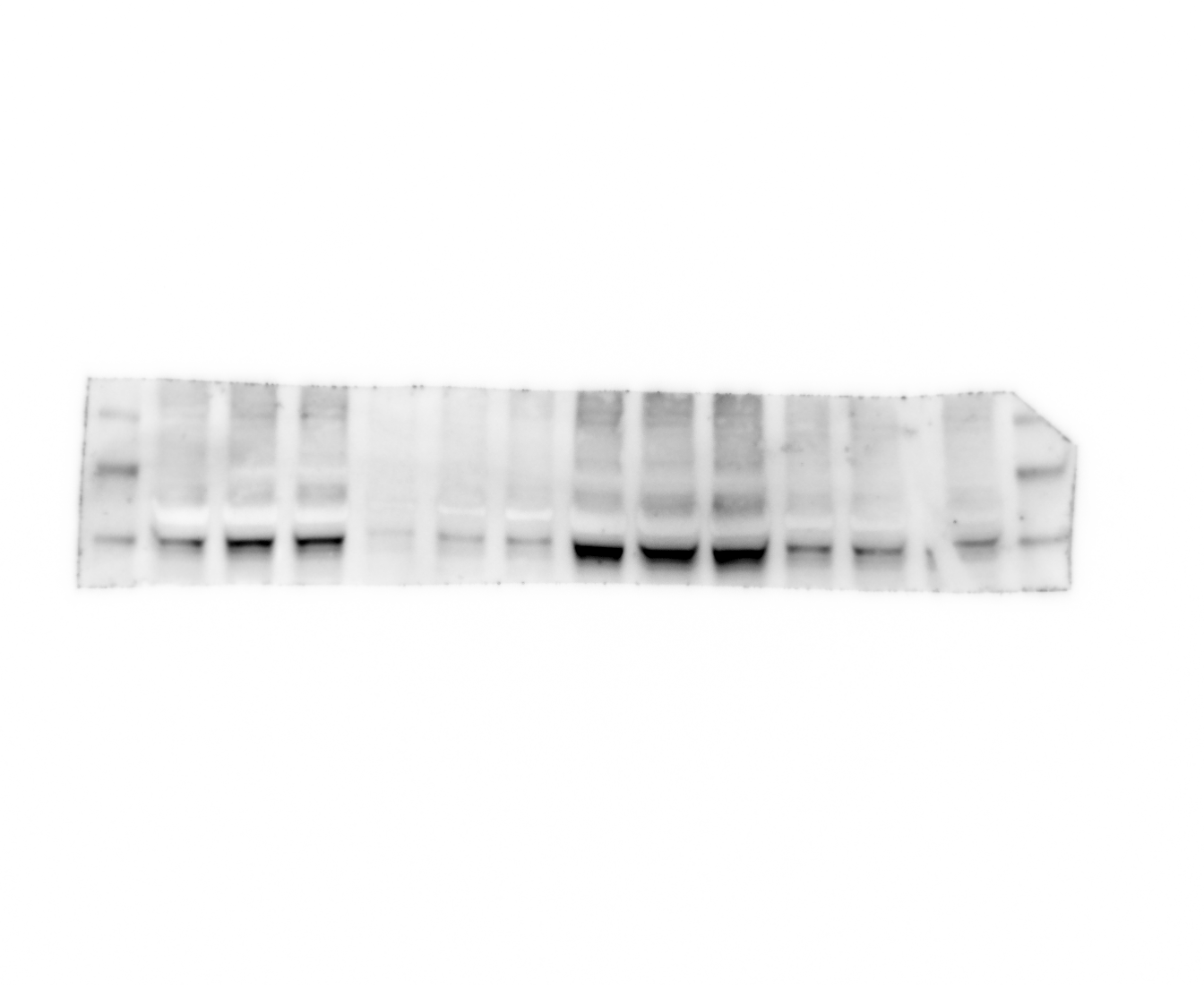

Supplement: Figure 3—figure supplement 1—source data 1. [file elife-98524-fig3-figsupp1-data1.zip › Fig 3-fig S2-data1-v1/S2A/CCN2.tif]

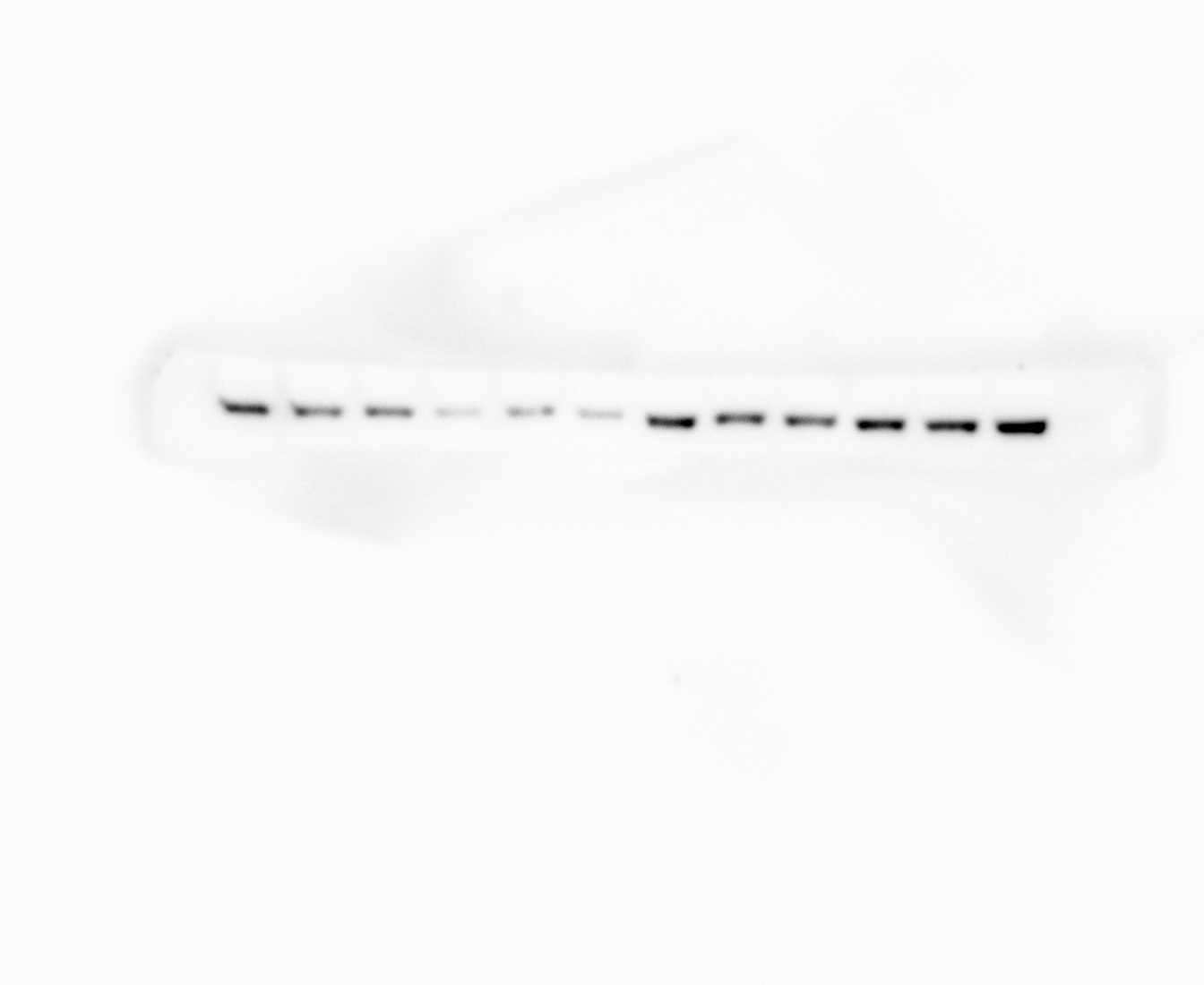

Supplement: Figure 3—figure supplement 1—source data 1. [file elife-98524-fig3-figsupp1-data1.zip › Fig 3-fig S2-data1-v1/S2A/COL1A1.tif]

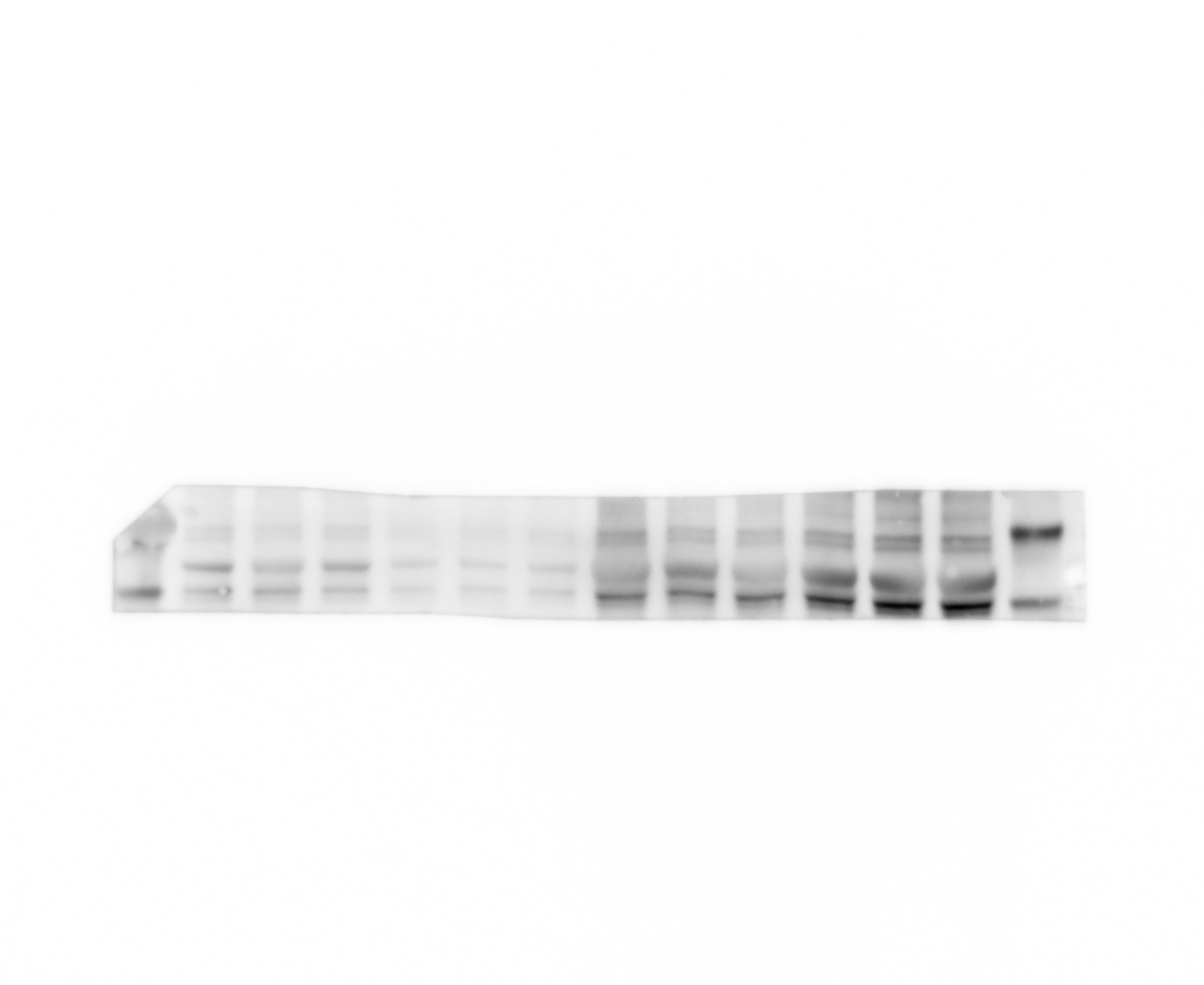

Supplement: Figure 3—figure supplement 1—source data 1. [file elife-98524-fig3-figsupp1-data1.zip › Fig 3-fig S2-data1-v1/S2A/COL3A1.tif]

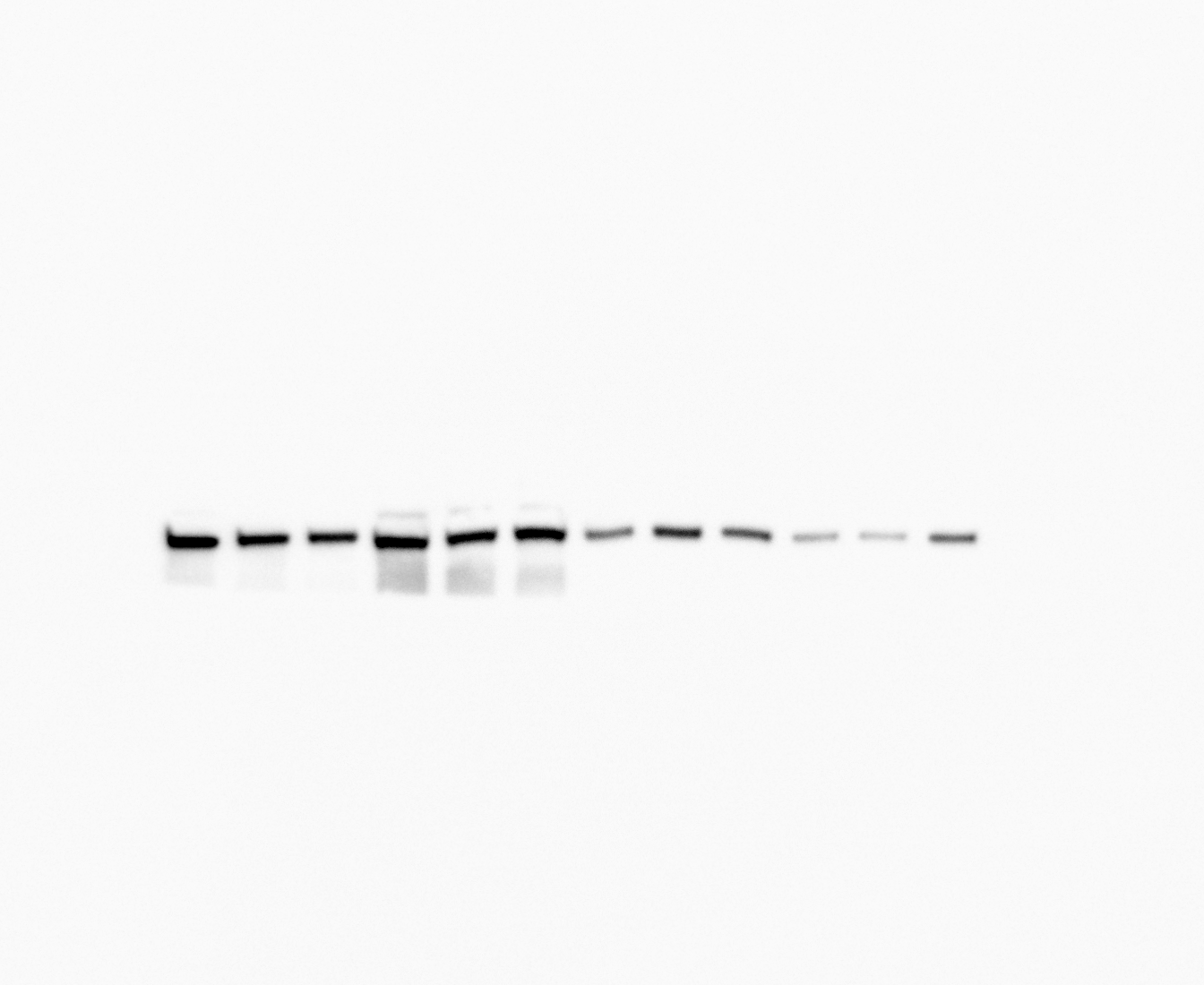

Supplement: Figure 3—figure supplement 1—source data 1. [file elife-98524-fig3-figsupp1-data1.zip › Fig 3-fig S2-data1-v1/S2A/E-cadherin.tif]

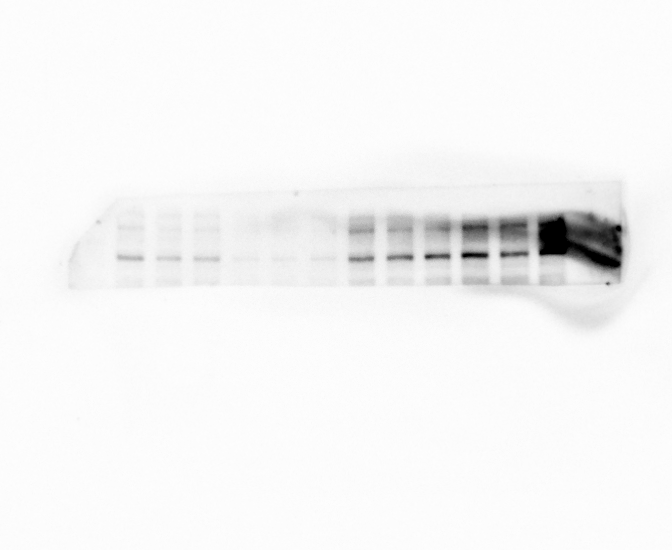

Supplement: Figure 3—figure supplement 1—source data 1. [file elife-98524-fig3-figsupp1-data1.zip › Fig 3-fig S2-data1-v1/S2A/FN1.tif]

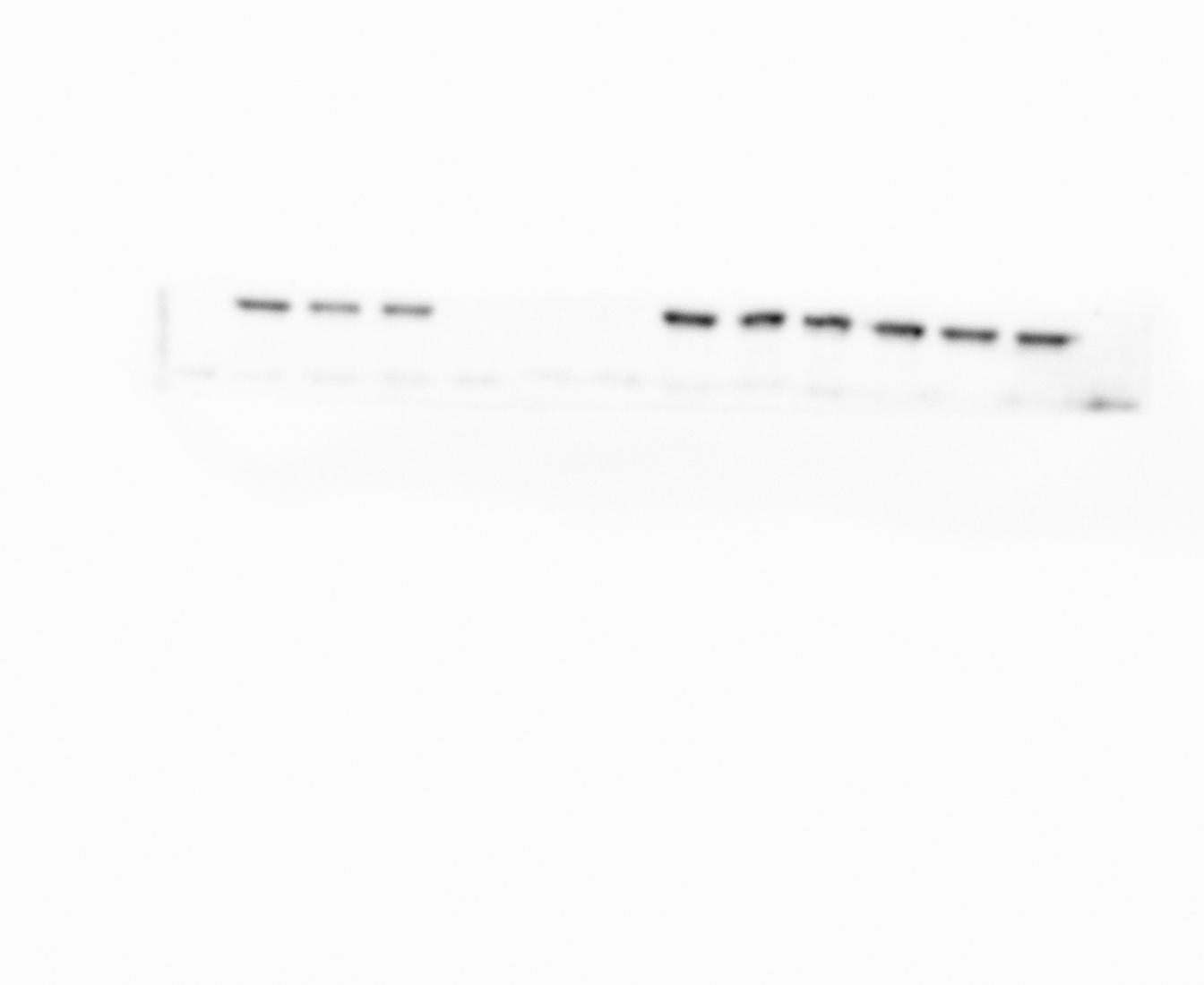

Supplement: Figure 3—figure supplement 1—source data 1. [file elife-98524-fig3-figsupp1-data1.zip › Fig 3-fig S2-data1-v1/S2A/SIRT4.tif]

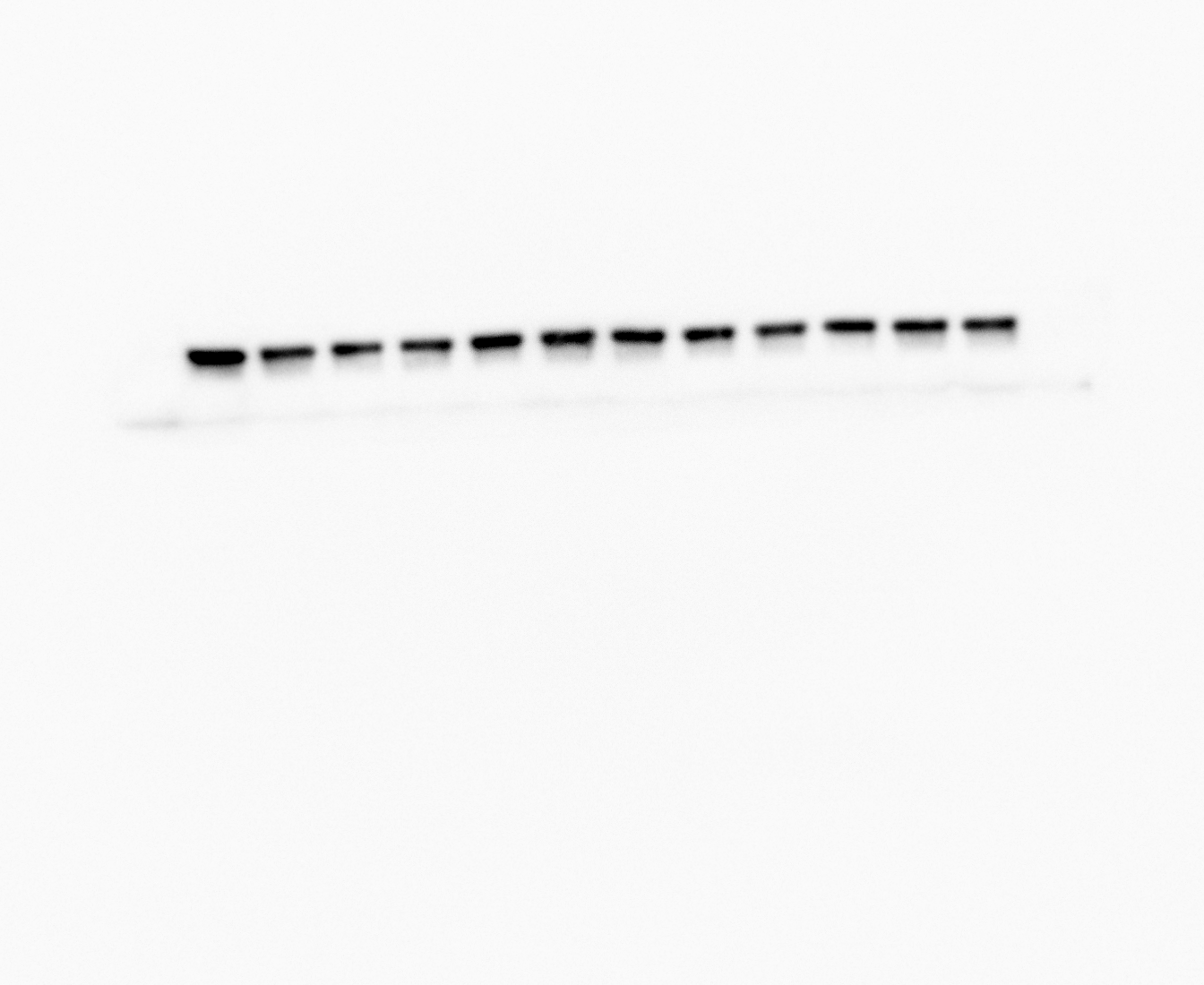

Supplement: Figure 3—figure supplement 1—source data 1. [file elife-98524-fig3-figsupp1-data1.zip › Fig 3-fig S2-data1-v1/S2A/Tubulin.tif]

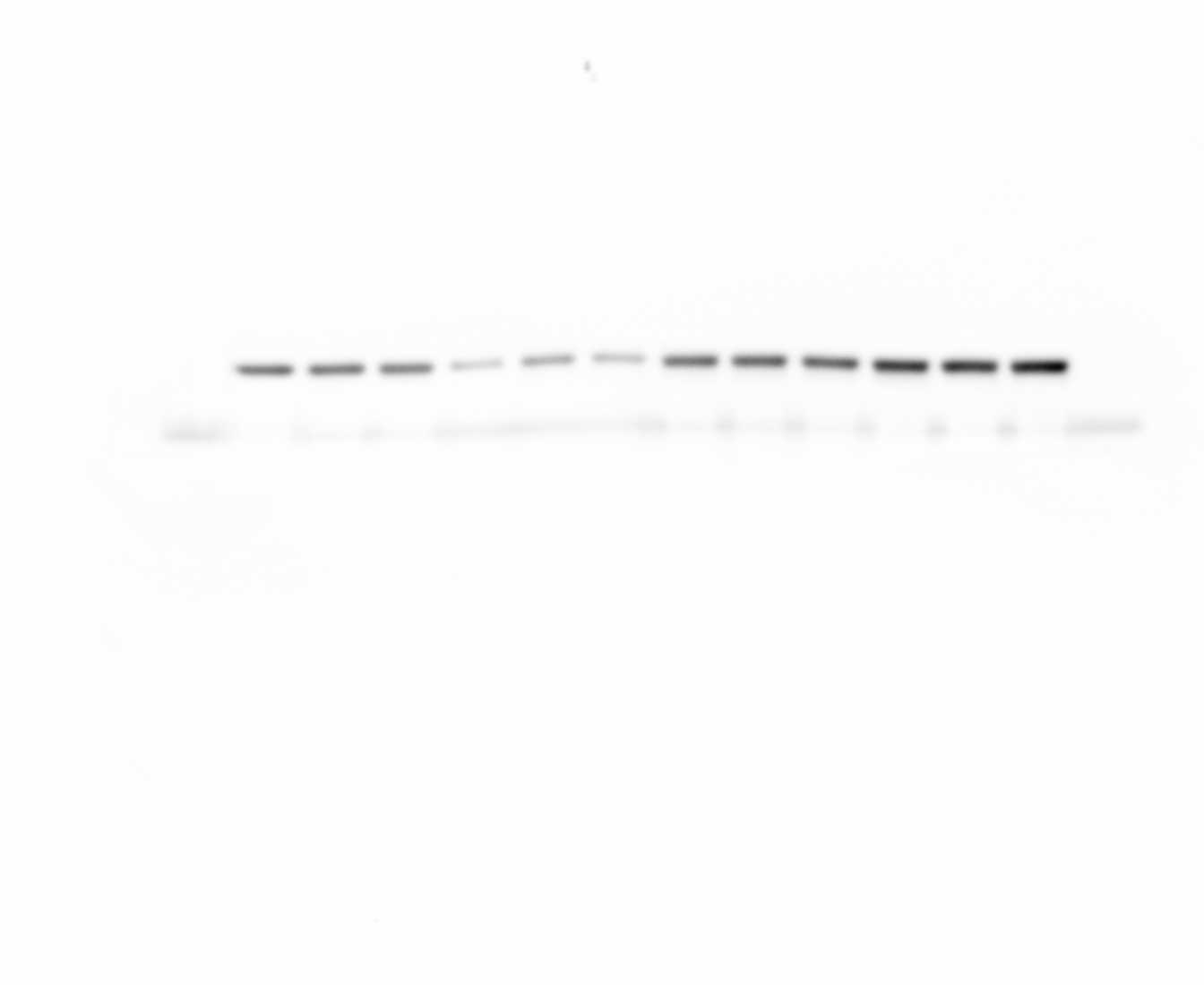

Supplement: Figure 3—figure supplement 1—source data 1. [file elife-98524-fig3-figsupp1-data1.zip › Fig 3-fig S2-data1-v1/S2A/α-SMA.tif]

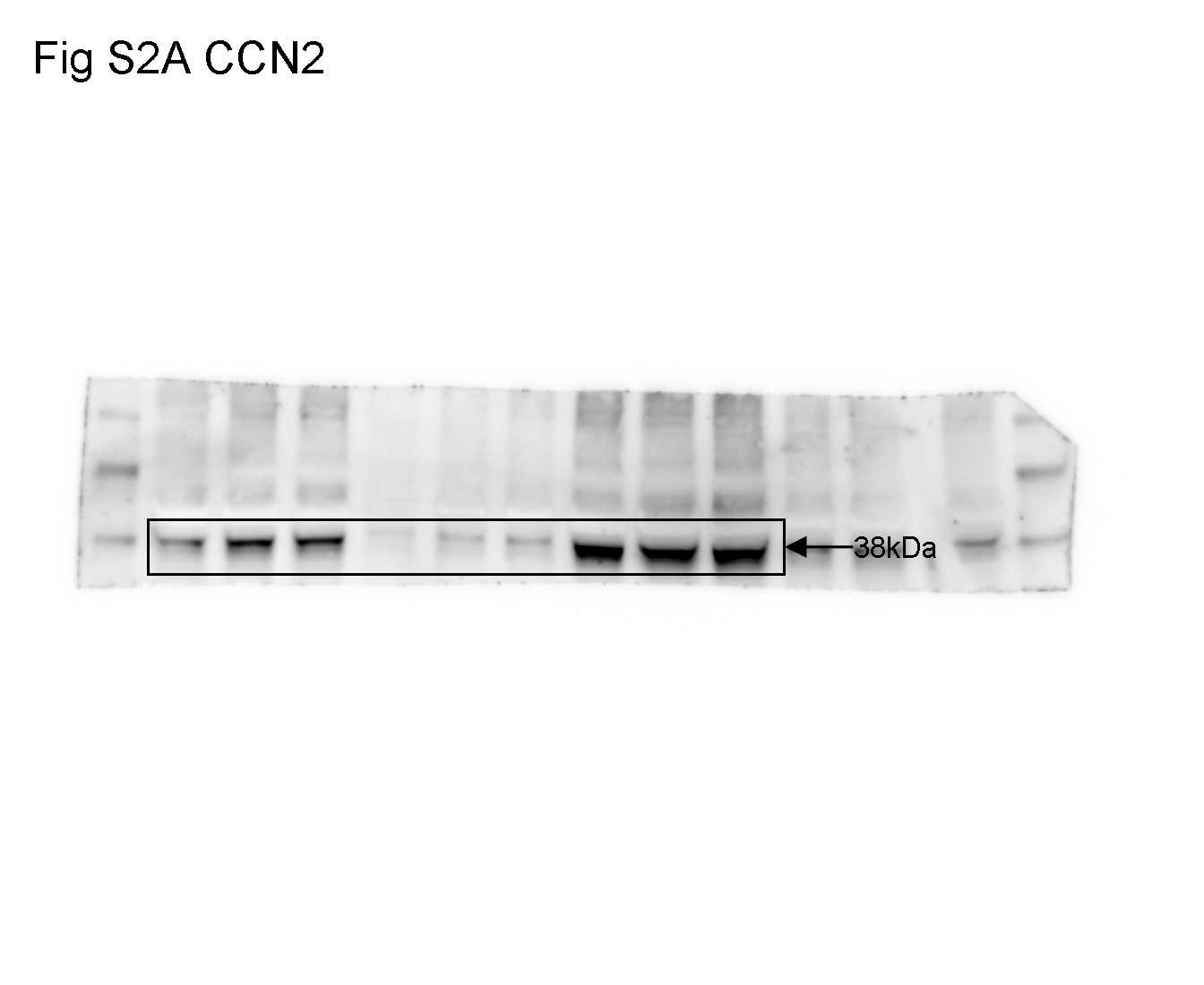

Supplement: Figure 3—figure supplement 1—source data 2. [file elife-98524-fig3-figsupp1-data2.zip › Fig 3-fig S2-data2-v1/S2A/CCN2.tif]

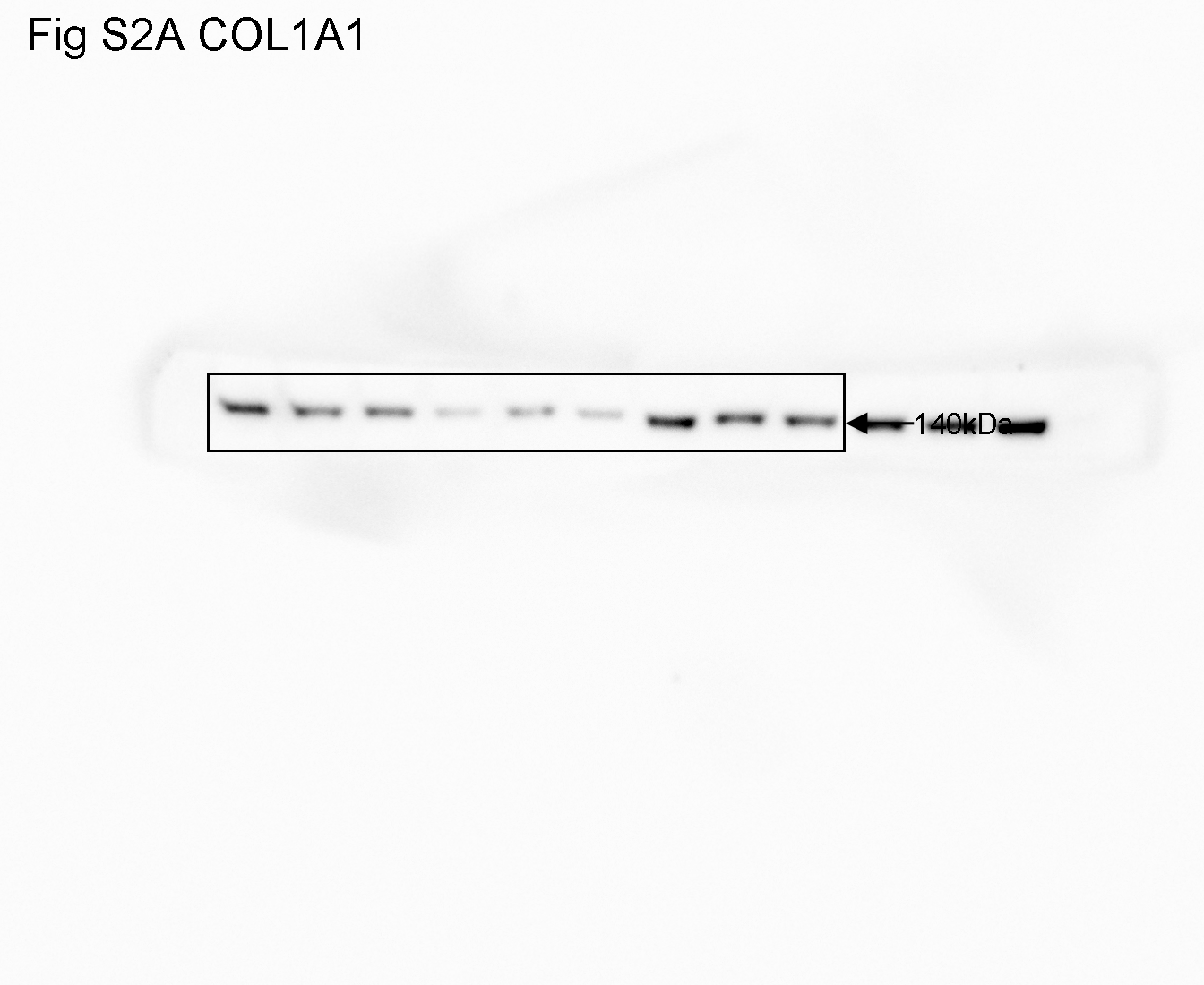

Supplement: Figure 3—figure supplement 1—source data 2. [file elife-98524-fig3-figsupp1-data2.zip › Fig 3-fig S2-data2-v1/S2A/COL1A1.tif]

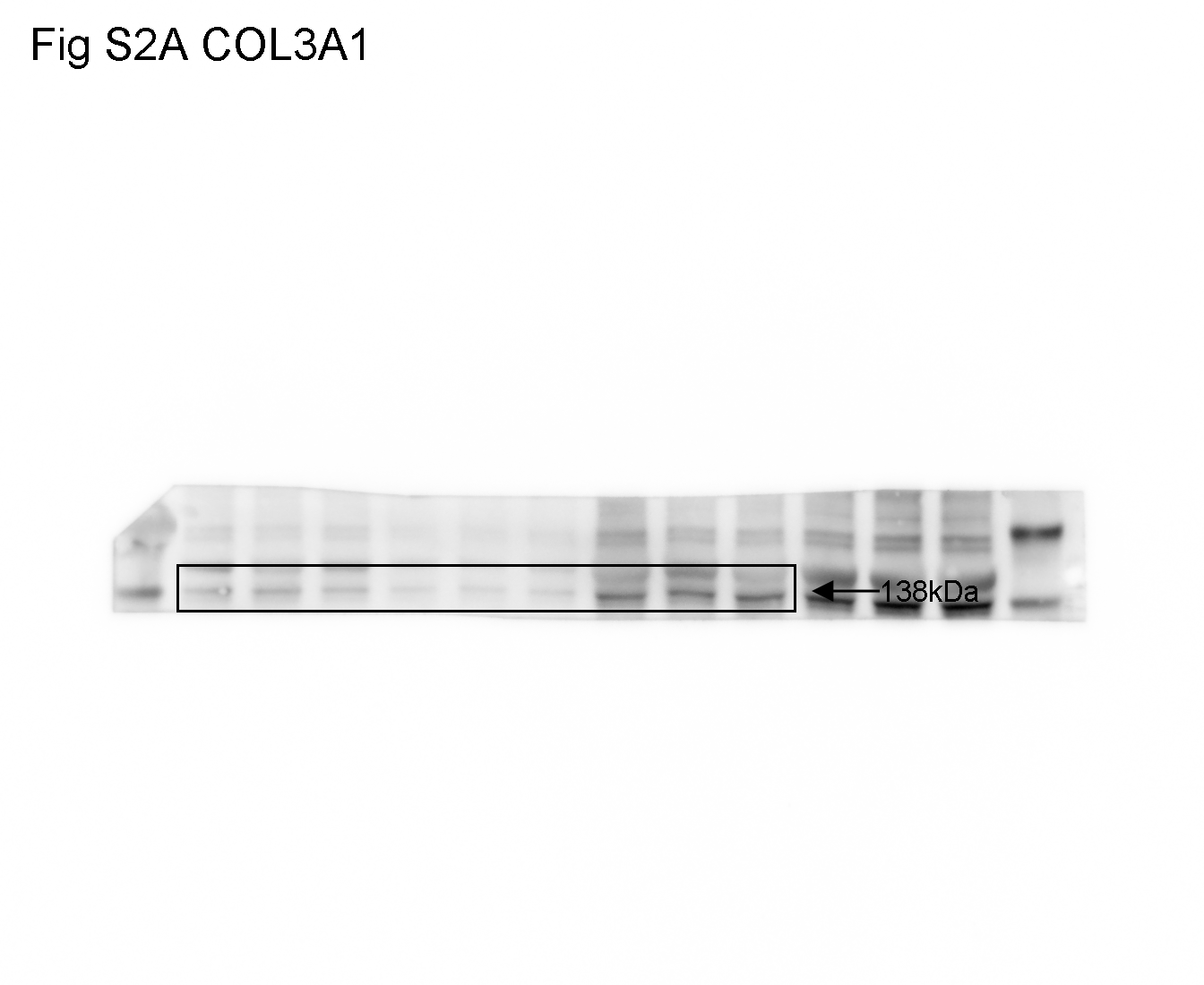

Supplement: Figure 3—figure supplement 1—source data 2. [file elife-98524-fig3-figsupp1-data2.zip › Fig 3-fig S2-data2-v1/S2A/COL3A1.tif]

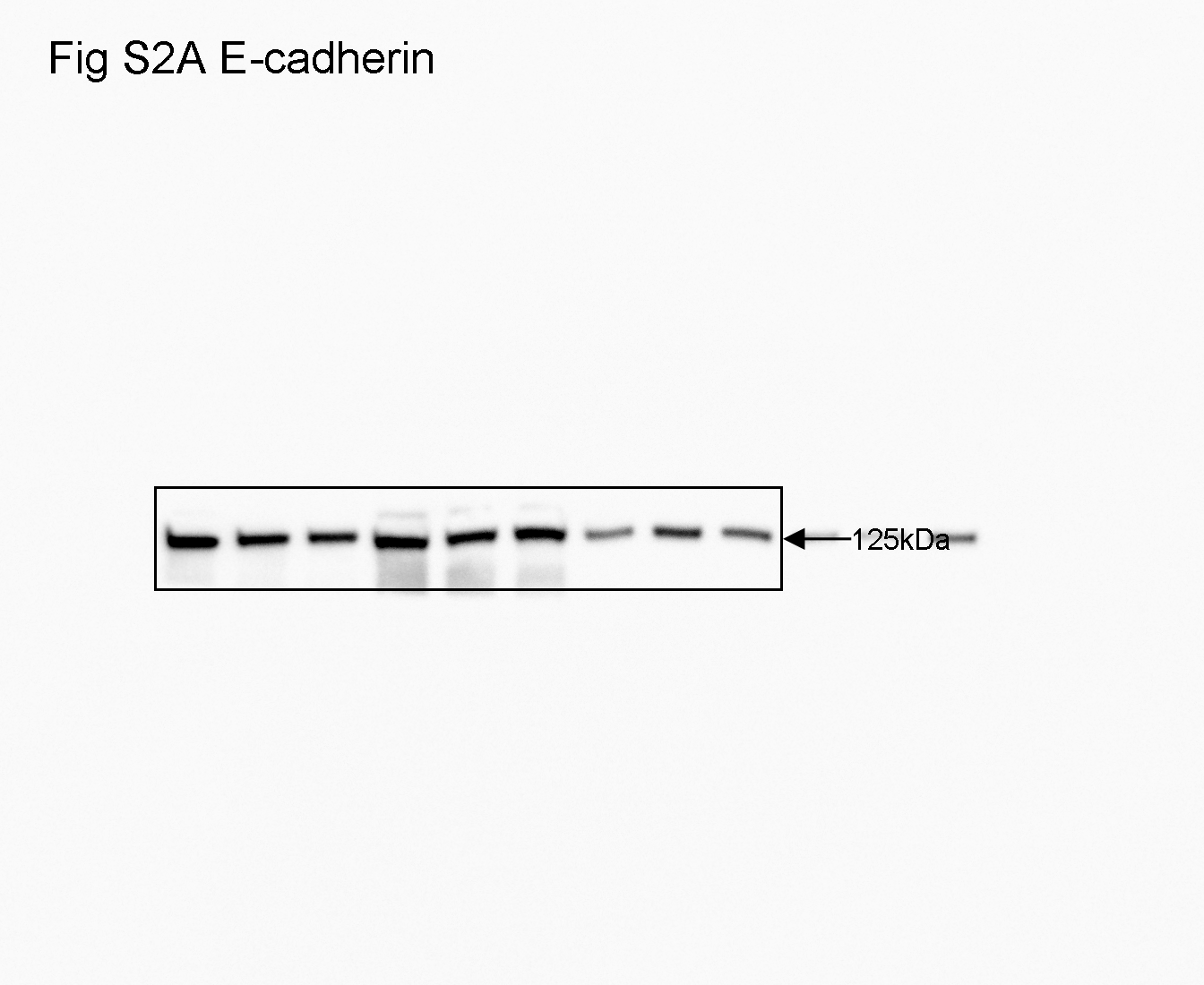

Supplement: Figure 3—figure supplement 1—source data 2. [file elife-98524-fig3-figsupp1-data2.zip › Fig 3-fig S2-data2-v1/S2A/E-cadherin.tif]

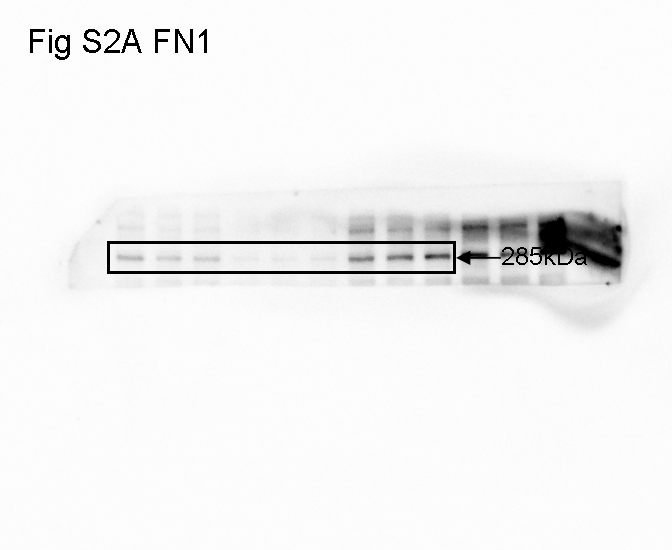

Supplement: Figure 3—figure supplement 1—source data 2. [file elife-98524-fig3-figsupp1-data2.zip › Fig 3-fig S2-data2-v1/S2A/FN1.tif]

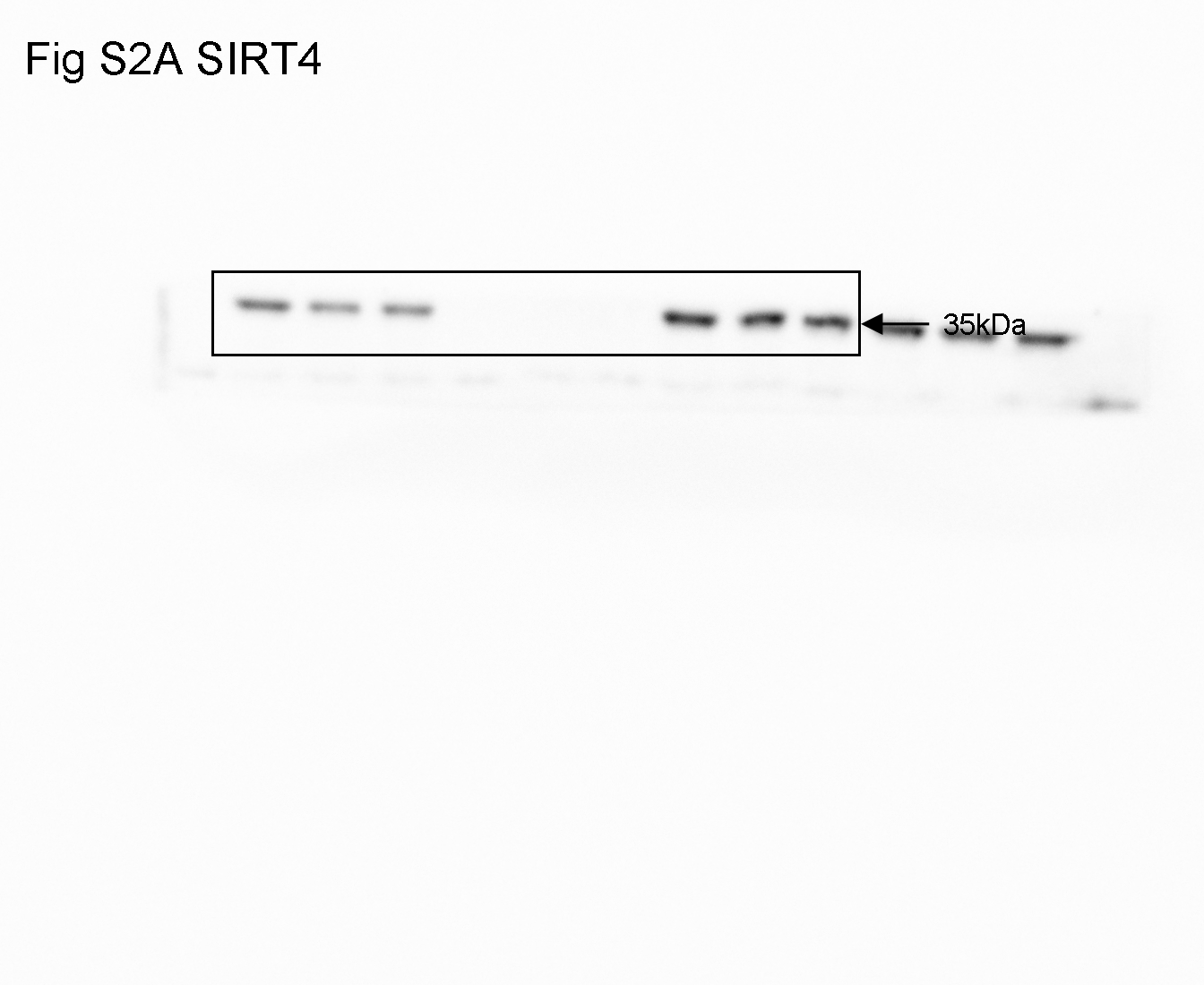

Supplement: Figure 3—figure supplement 1—source data 2. [file elife-98524-fig3-figsupp1-data2.zip › Fig 3-fig S2-data2-v1/S2A/SIRT4.tif]

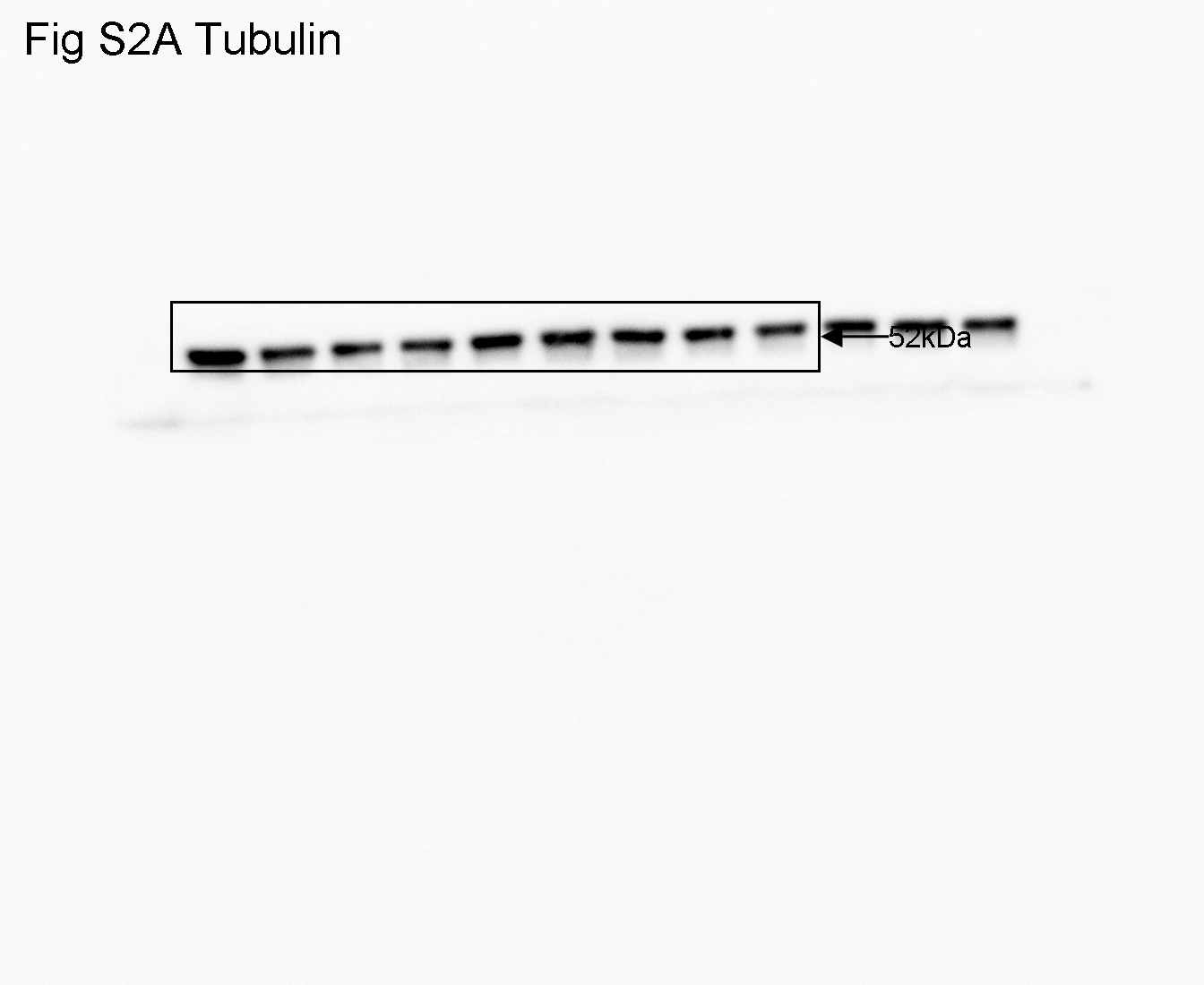

Supplement: Figure 3—figure supplement 1—source data 2. [file elife-98524-fig3-figsupp1-data2.zip › Fig 3-fig S2-data2-v1/S2A/Tubulin.tif]

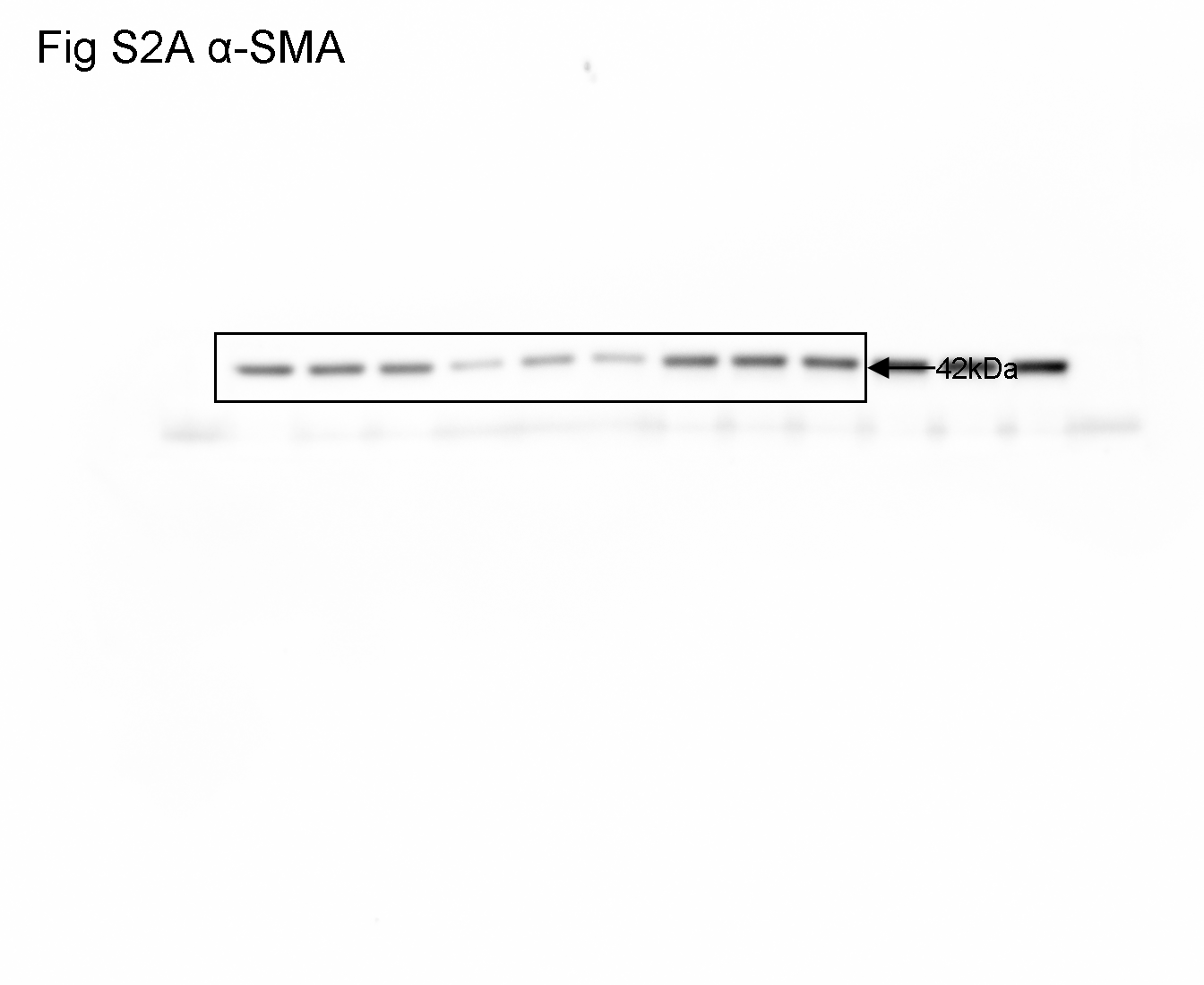

Supplement: Figure 3—figure supplement 1—source data 2. [file elife-98524-fig3-figsupp1-data2.zip › Fig 3-fig S2-data2-v1/S2A/α-SMA.tif]

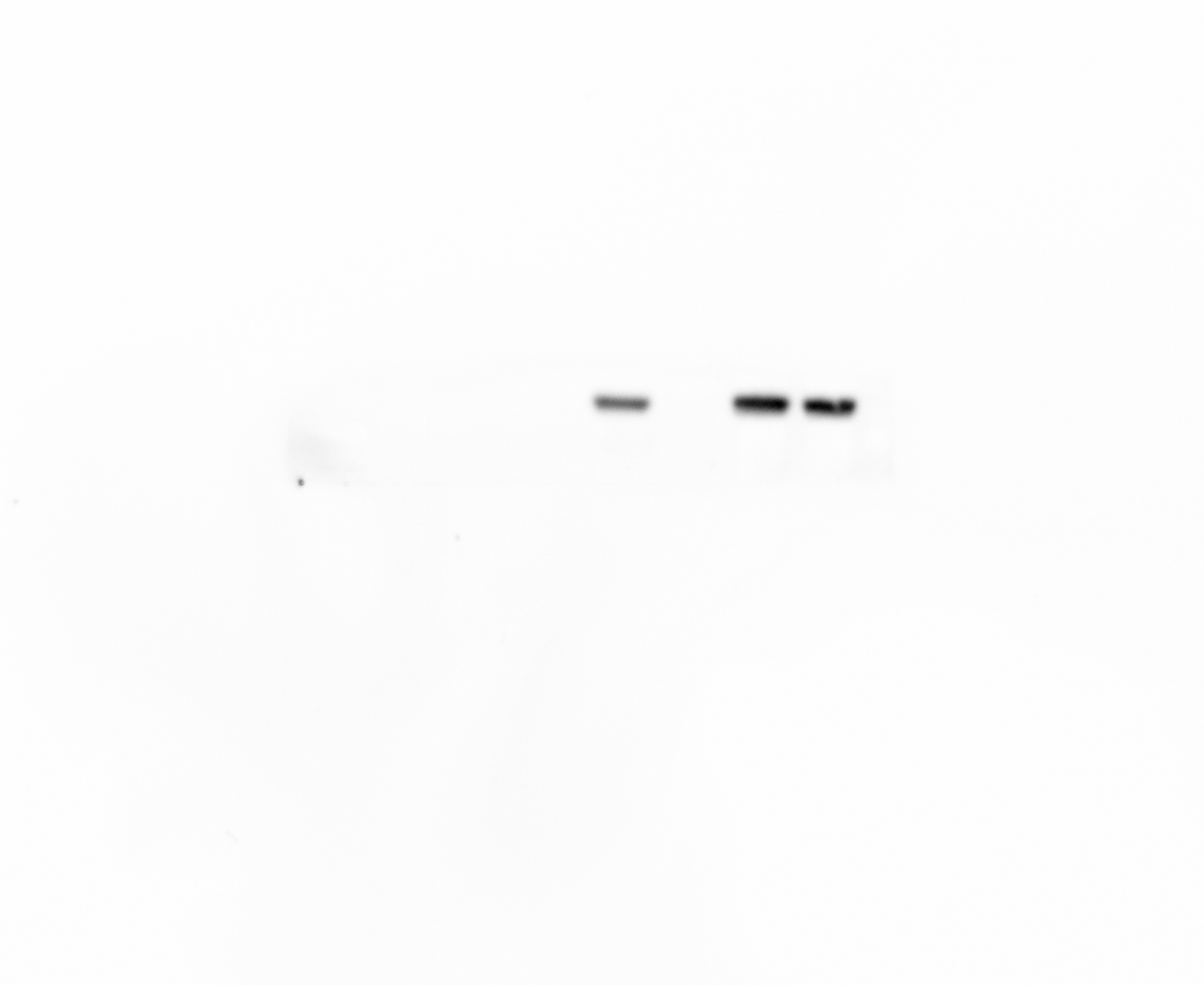

Supplement: Figure 4—source data 1. [file elife-98524-fig4-data1.zip › Fig 4-data1-v1/4C/1/PUF60.tif]

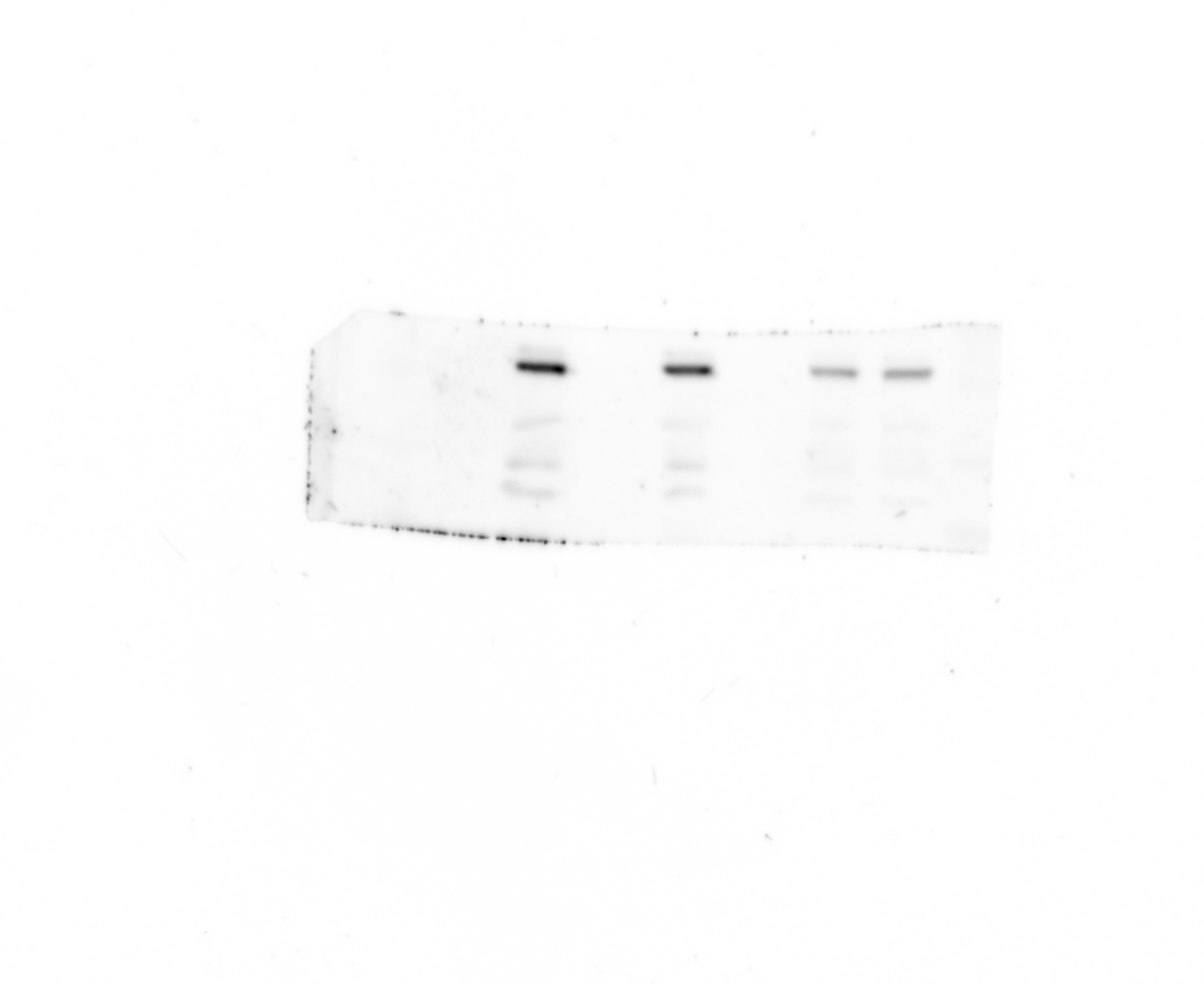

Supplement: Figure 4—source data 1. [file elife-98524-fig4-data1.zip › Fig 4-data1-v1/4C/1/SIRT4 .tif]

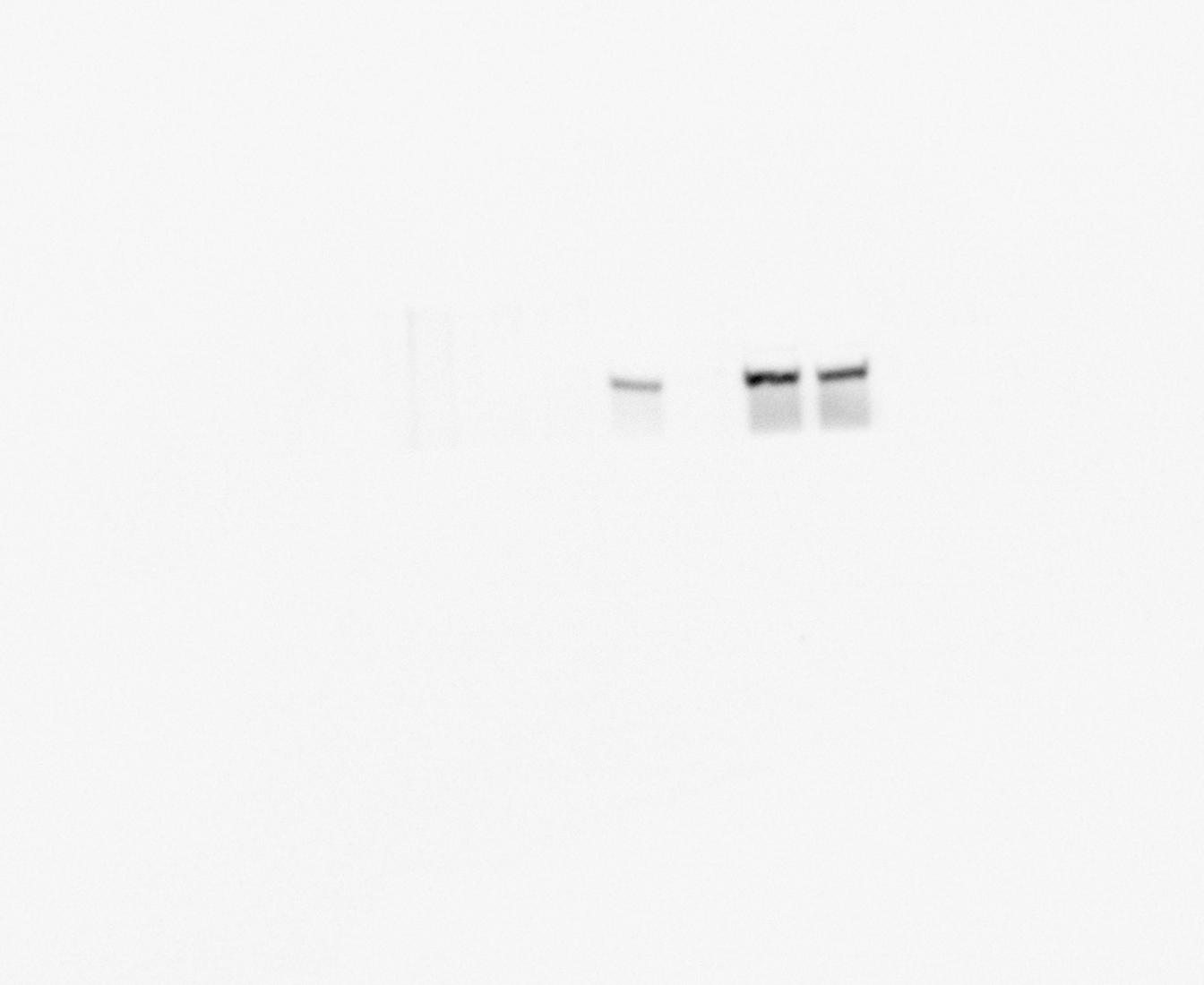

Supplement: Figure 4—source data 1. [file elife-98524-fig4-data1.zip › Fig 4-data1-v1/4C/1/U2AF2.tif]

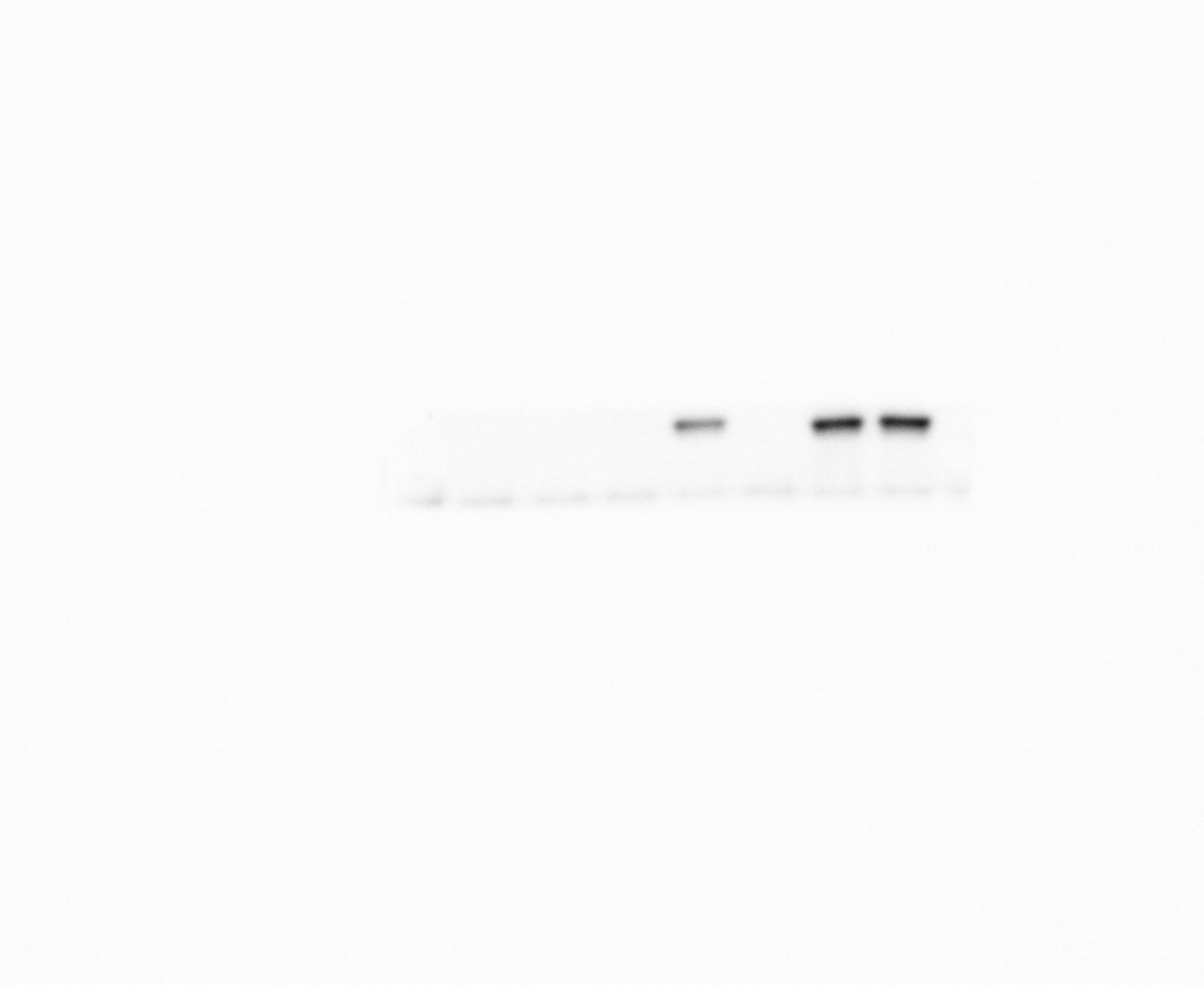

Supplement: Figure 4—source data 1. [file elife-98524-fig4-data1.zip › Fig 4-data1-v1/4C/2/PUF60.tif]

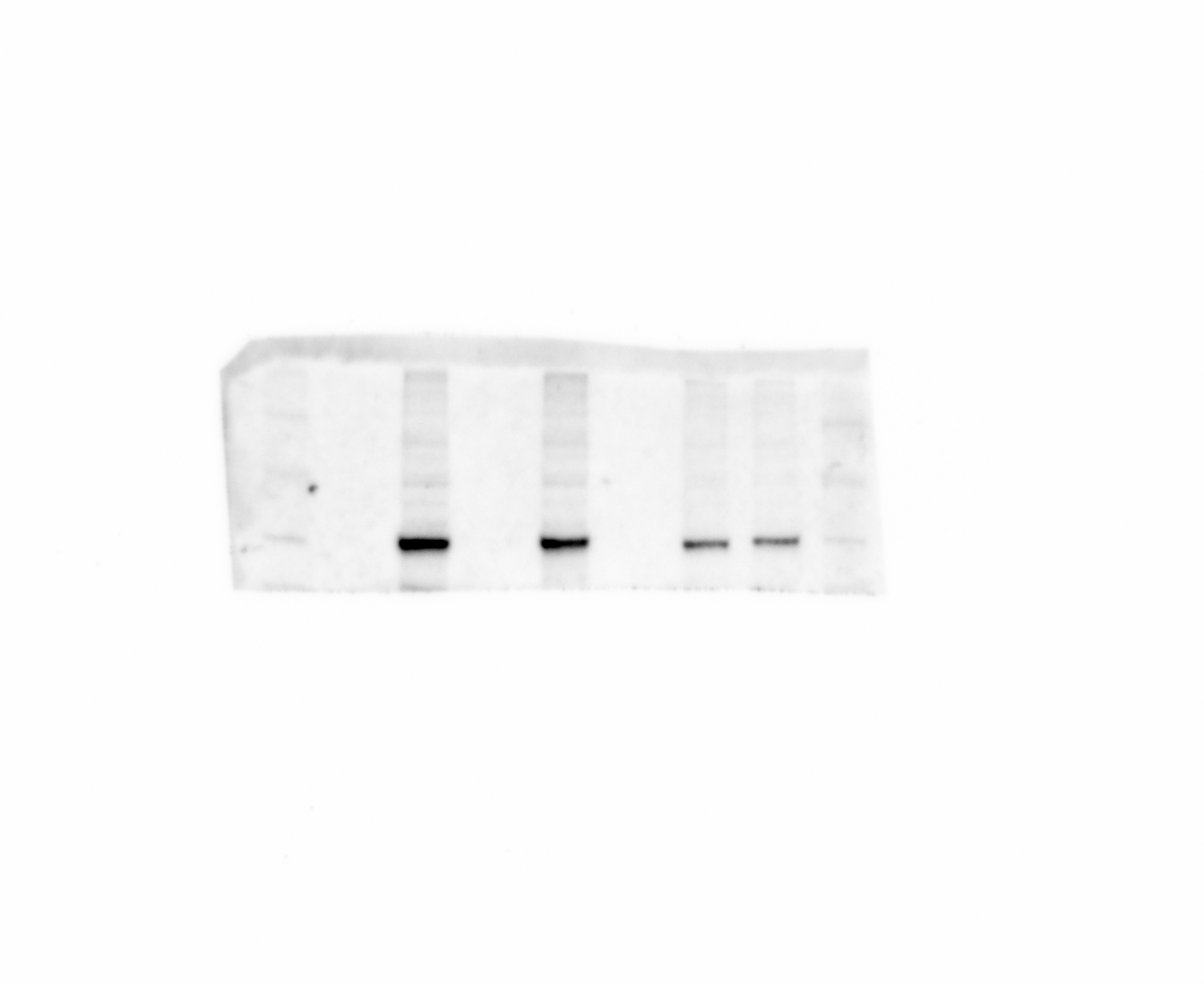

Supplement: Figure 4—source data 1. [file elife-98524-fig4-data1.zip › Fig 4-data1-v1/4C/2/SIRT4.tif]

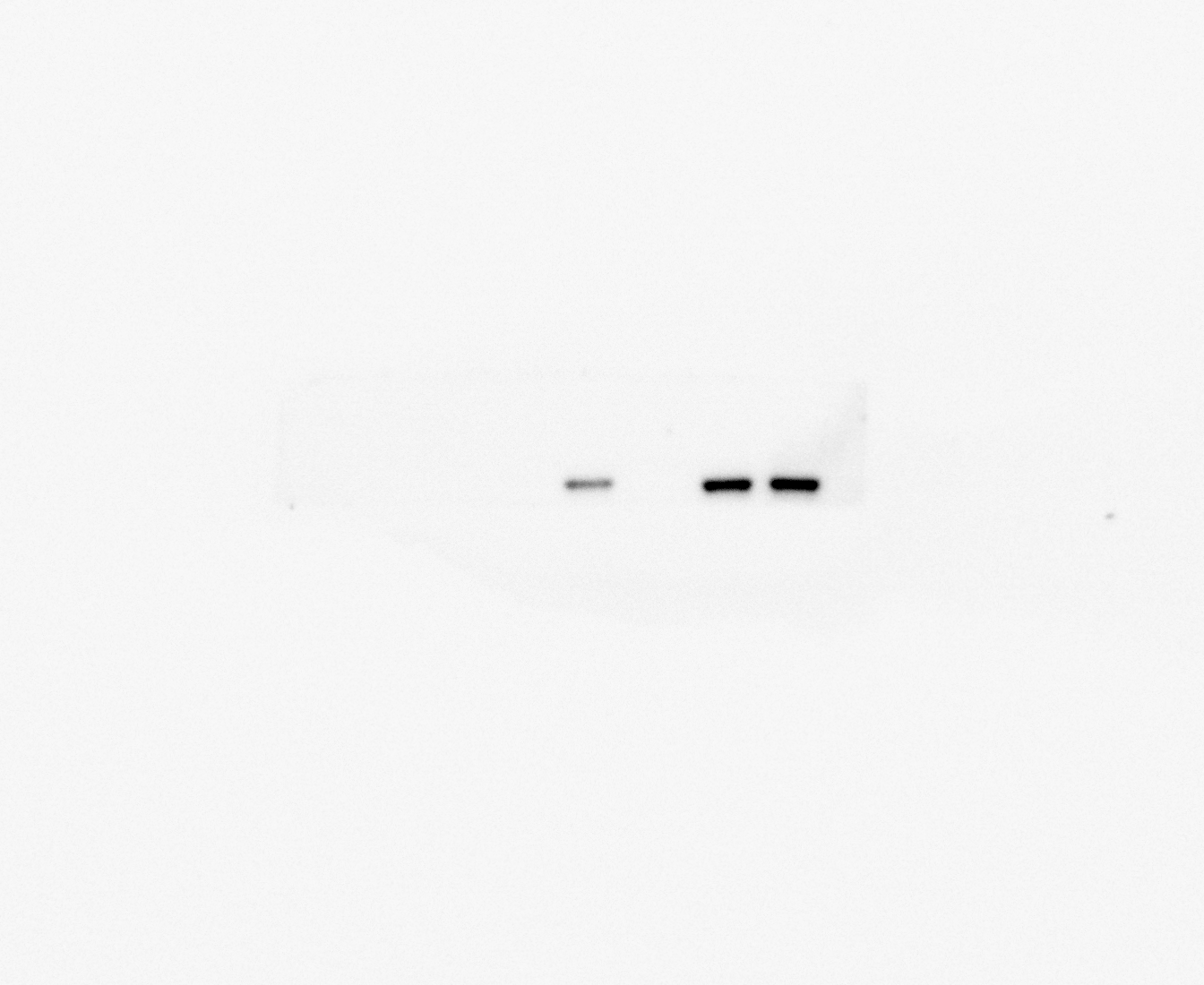

Supplement: Figure 4—source data 1. [file elife-98524-fig4-data1.zip › Fig 4-data1-v1/4C/2/U2AF2.tif]

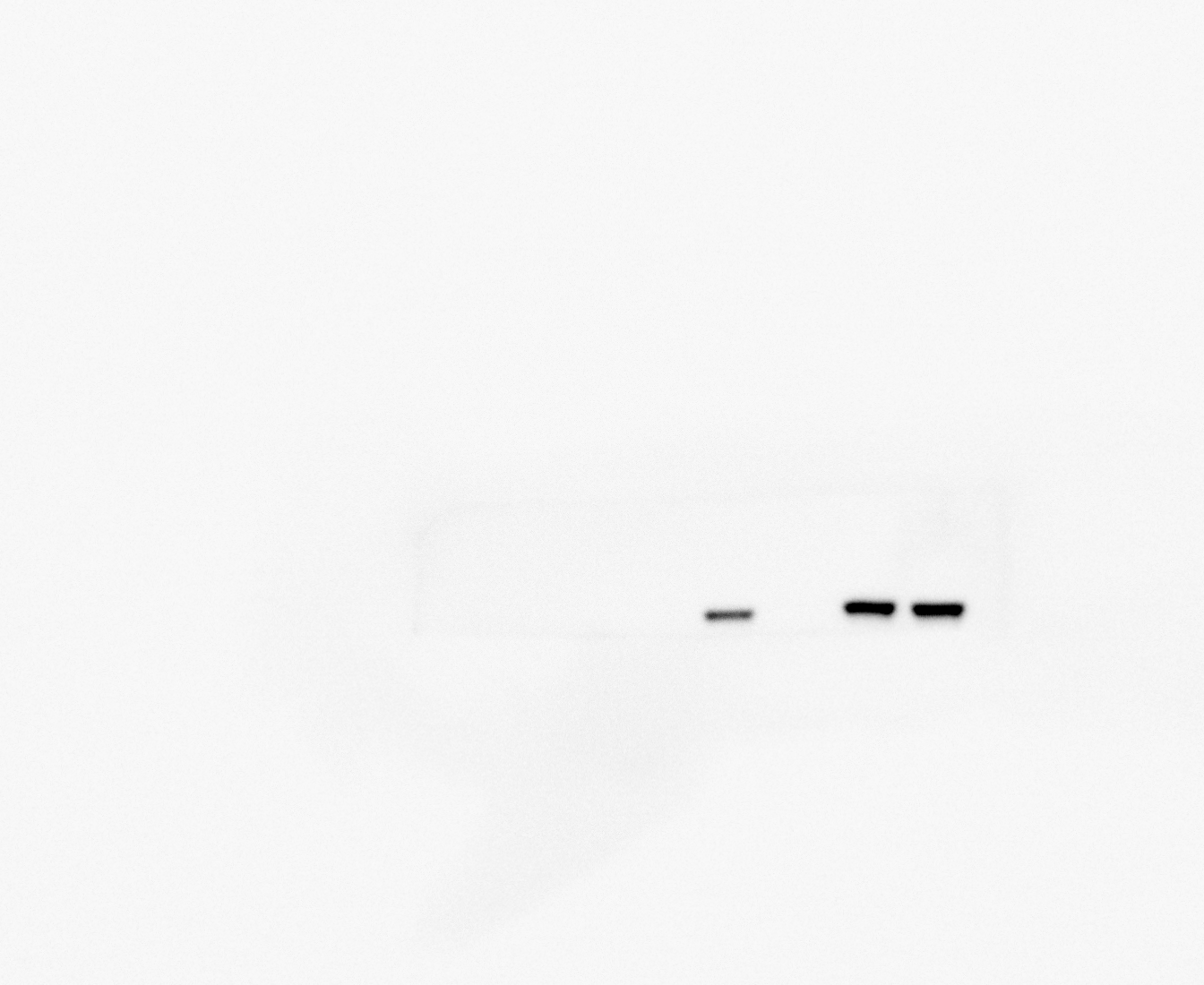

Supplement: Figure 4—source data 1. [file elife-98524-fig4-data1.zip › Fig 4-data1-v1/4C/3/PUF60.tif]

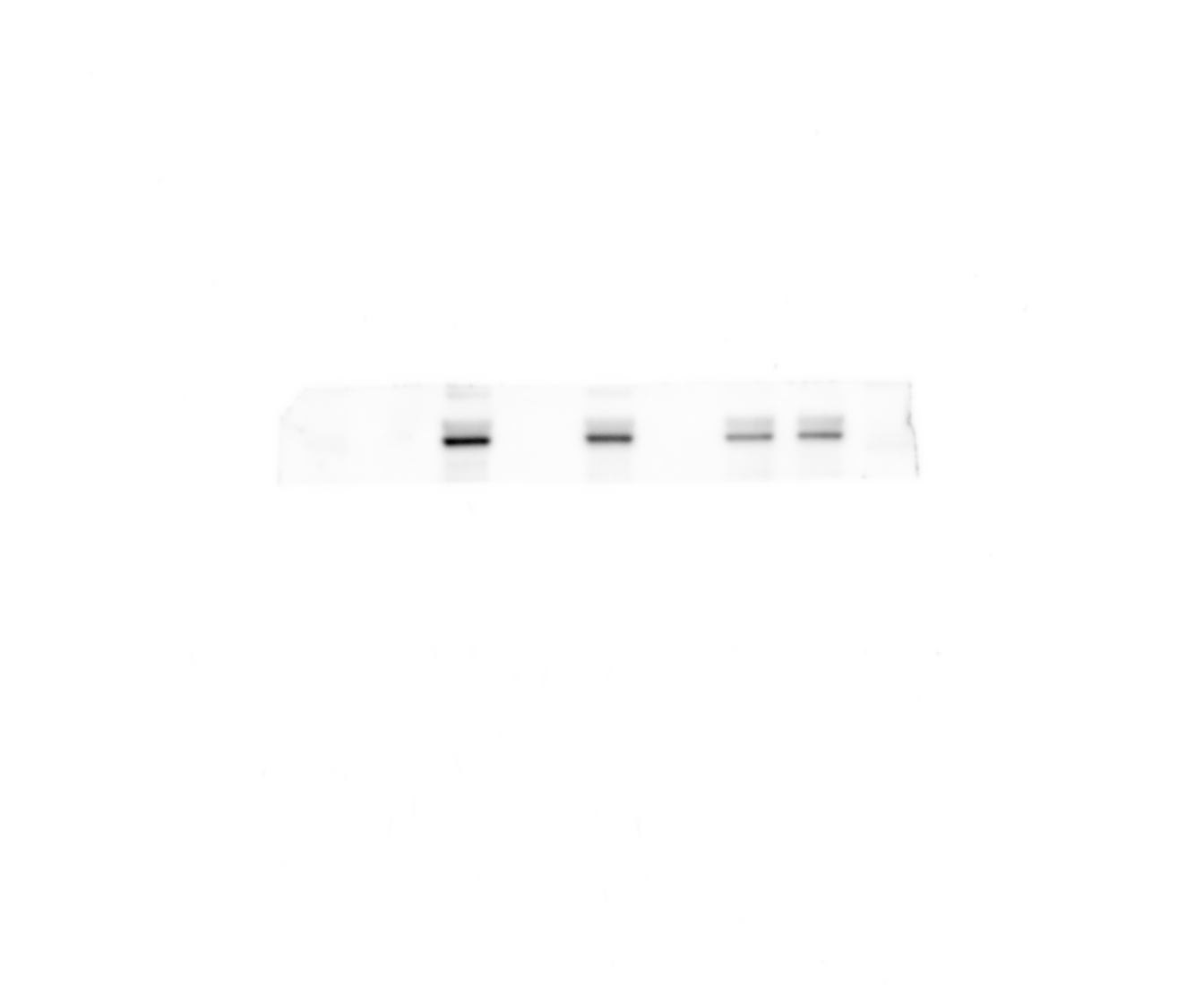

Supplement: Figure 4—source data 1. [file elife-98524-fig4-data1.zip › Fig 4-data1-v1/4C/3/SIRT4.tif]

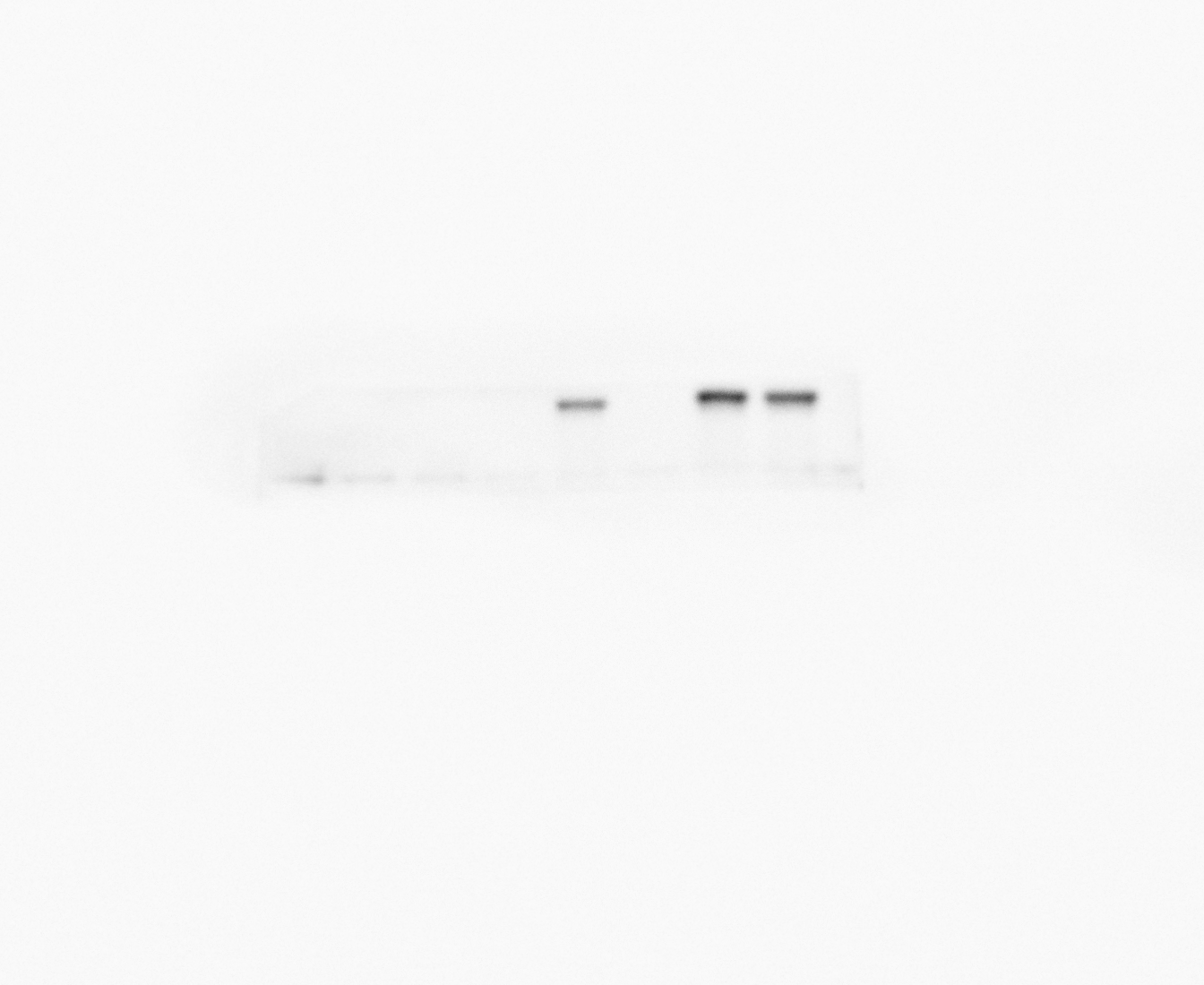

Supplement: Figure 4—source data 1. [file elife-98524-fig4-data1.zip › Fig 4-data1-v1/4C/3/U2AF2.tif]

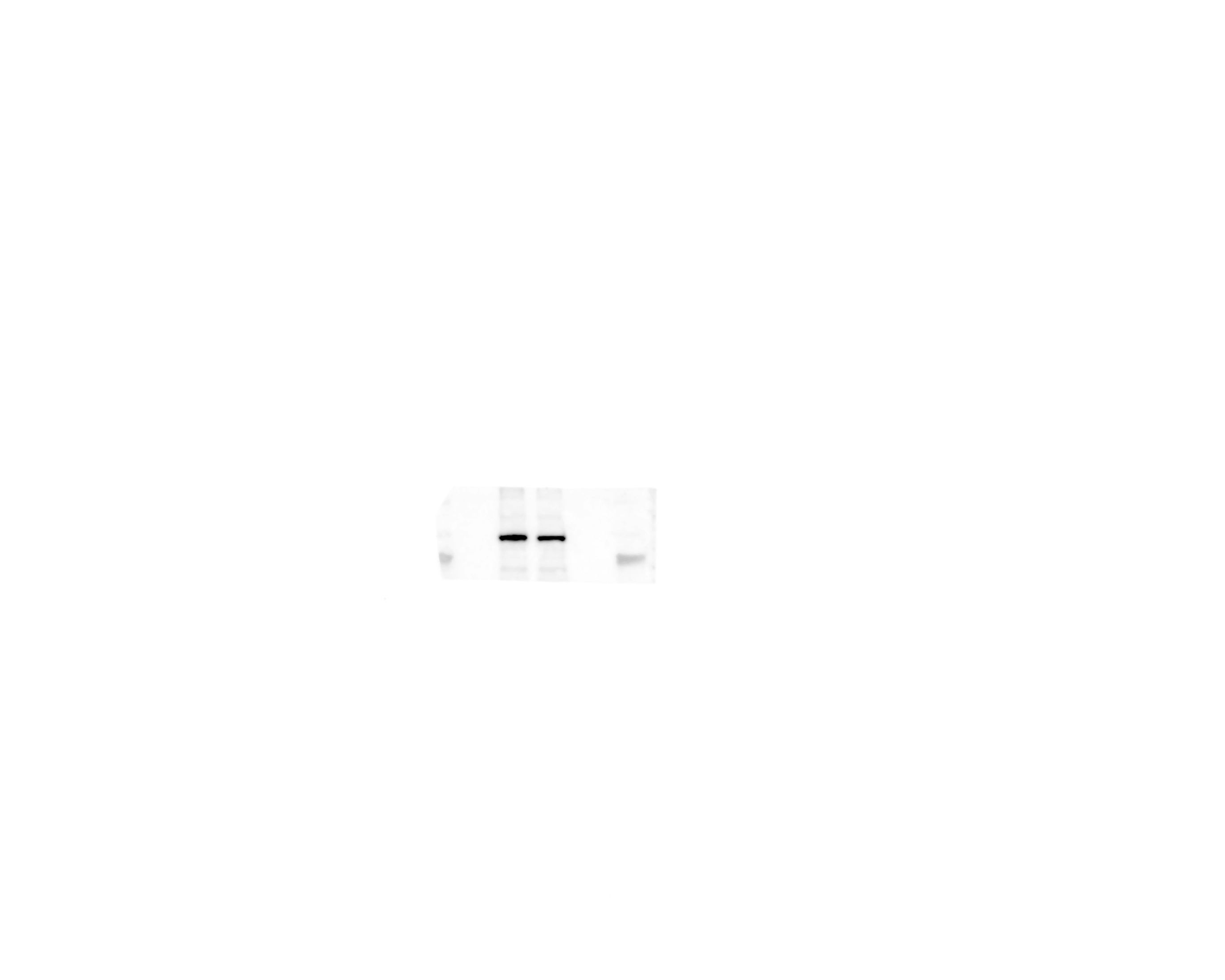

Supplement: Figure 4—source data 1. [file elife-98524-fig4-data1.zip › Fig 4-data1-v1/4C/4/bottom/Flag.tiff]

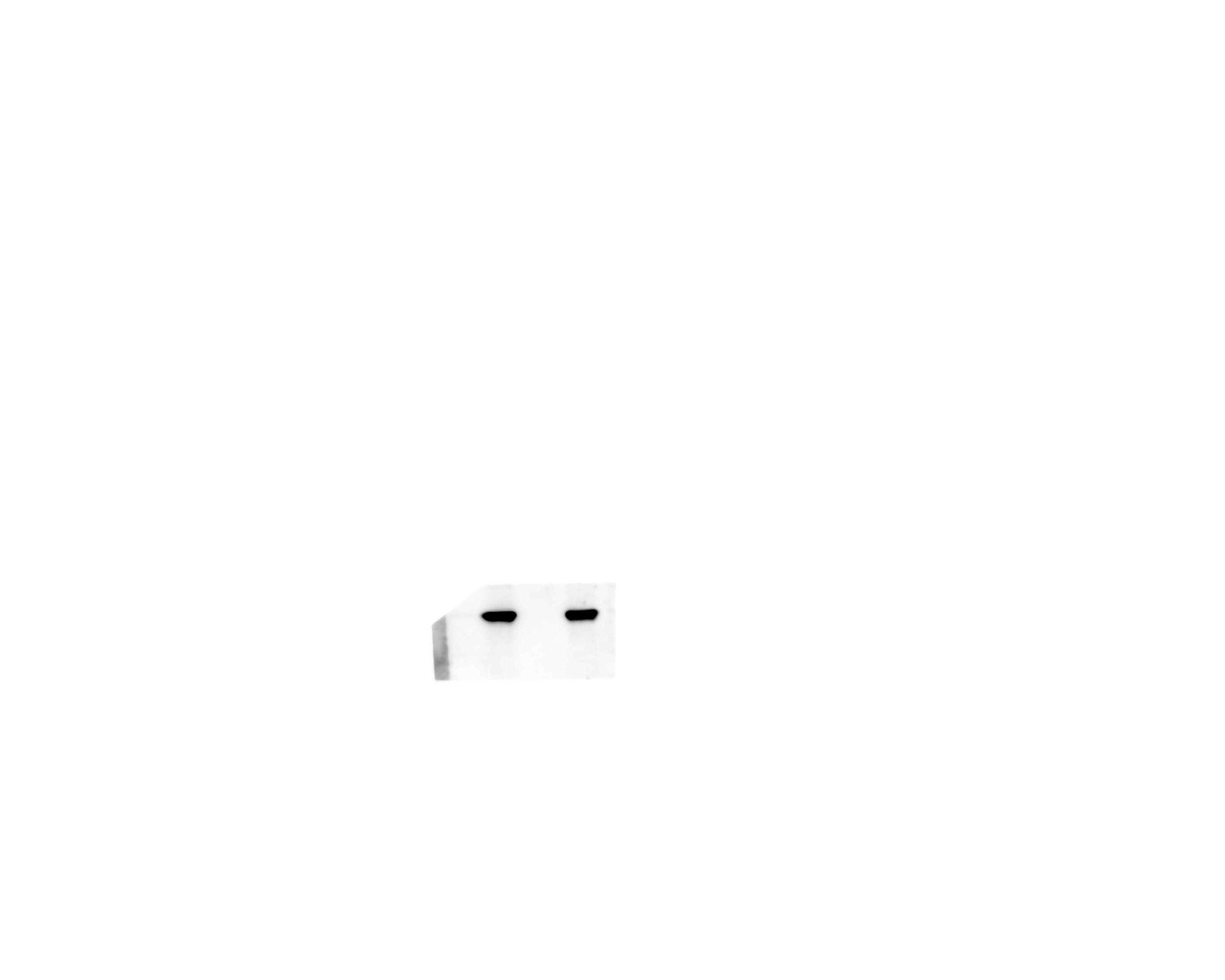

Supplement: Figure 4—source data 1. [file elife-98524-fig4-data1.zip › Fig 4-data1-v1/4C/4/bottom/HA.tiff]

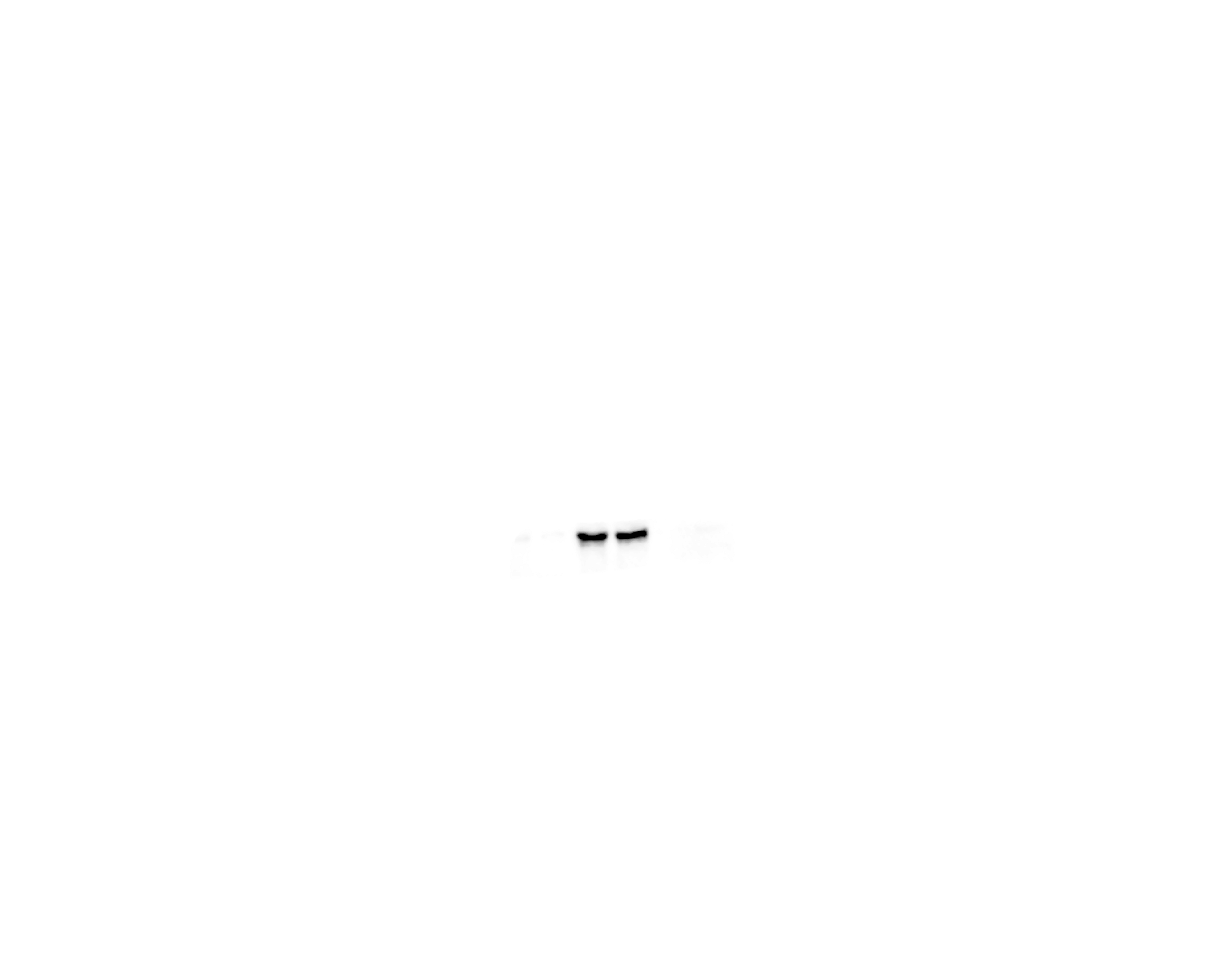

Supplement: Figure 4—source data 1. [file elife-98524-fig4-data1.zip › Fig 4-data1-v1/4C/4/upper/Flag.tiff]

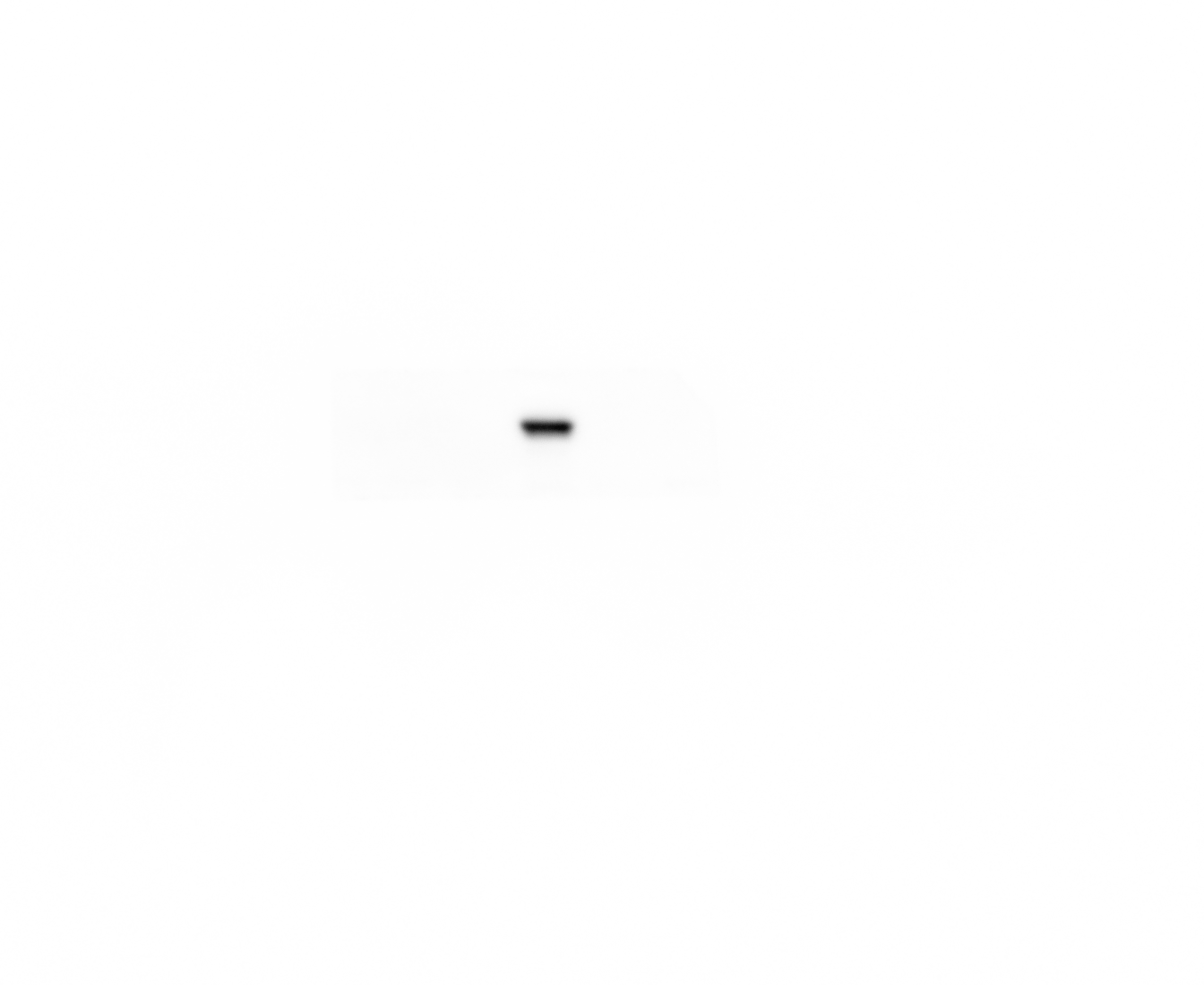

Supplement: Figure 4—source data 1. [file elife-98524-fig4-data1.zip › Fig 4-data1-v1/4C/4/upper/HA.tif]

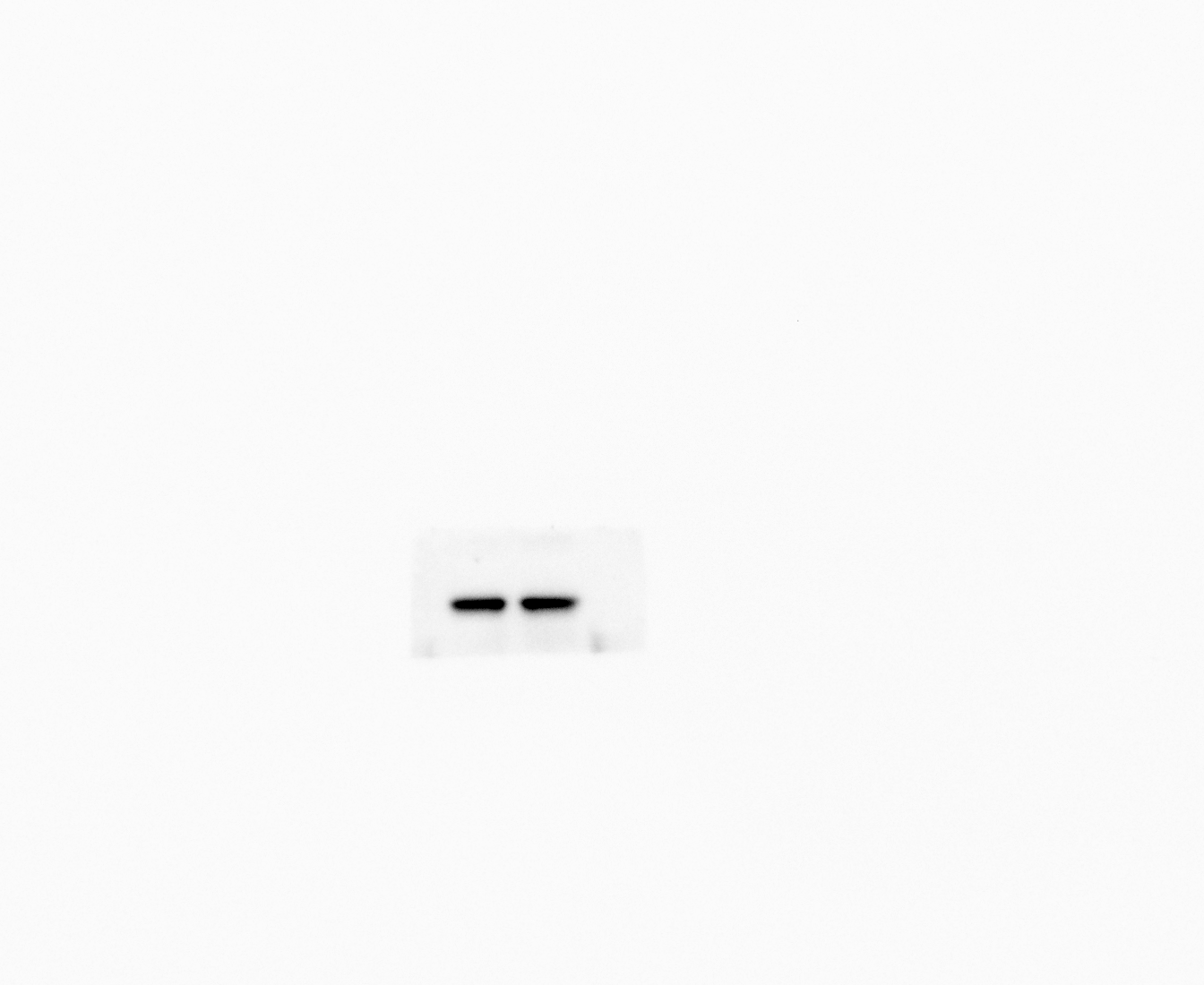

Supplement: Figure 4—source data 1. [file elife-98524-fig4-data1.zip › Fig 4-data1-v1/4D/bottom/PUF60.tif]

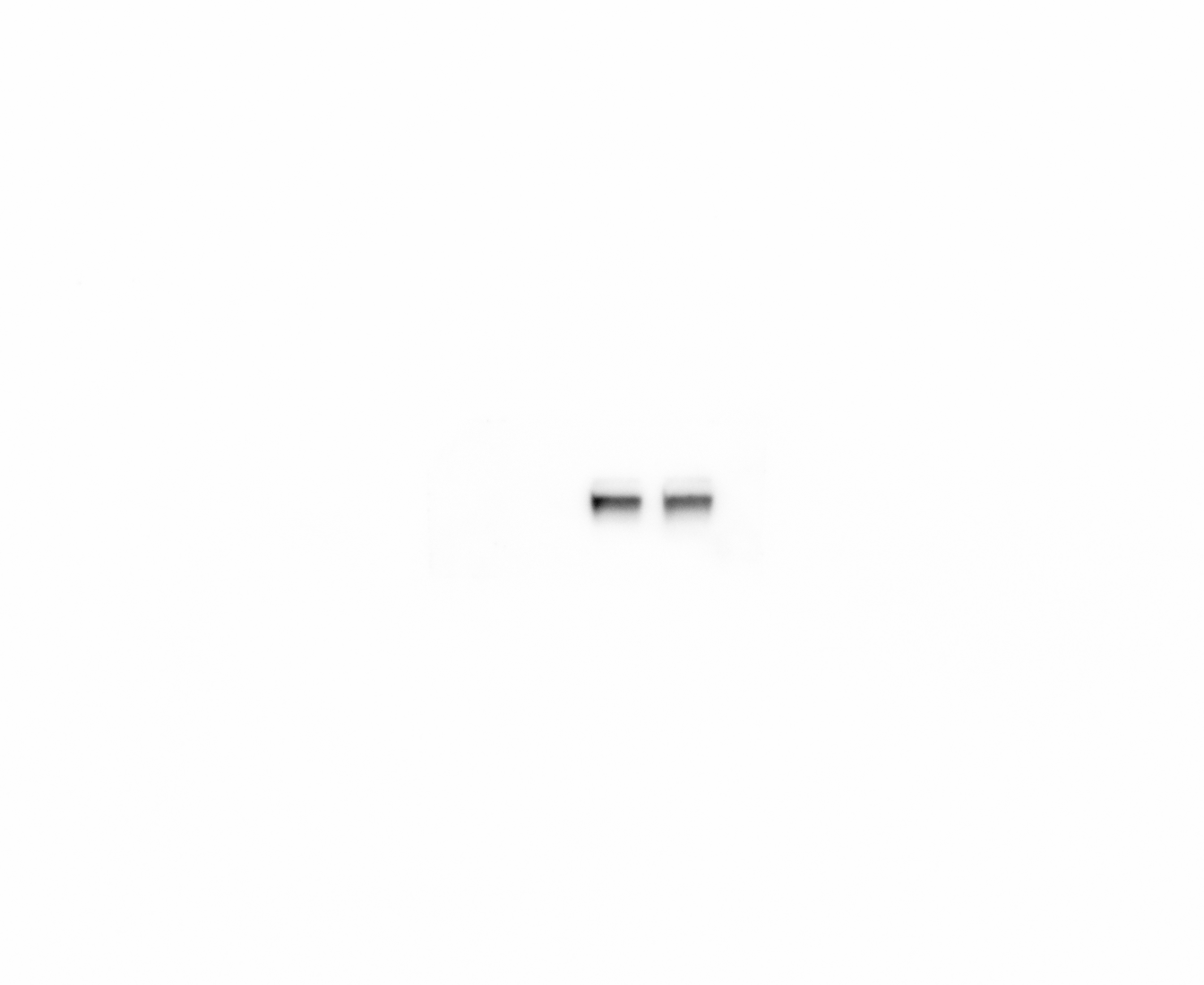

Supplement: Figure 4—source data 1. [file elife-98524-fig4-data1.zip › Fig 4-data1-v1/4D/bottom/SIRT4.tif]

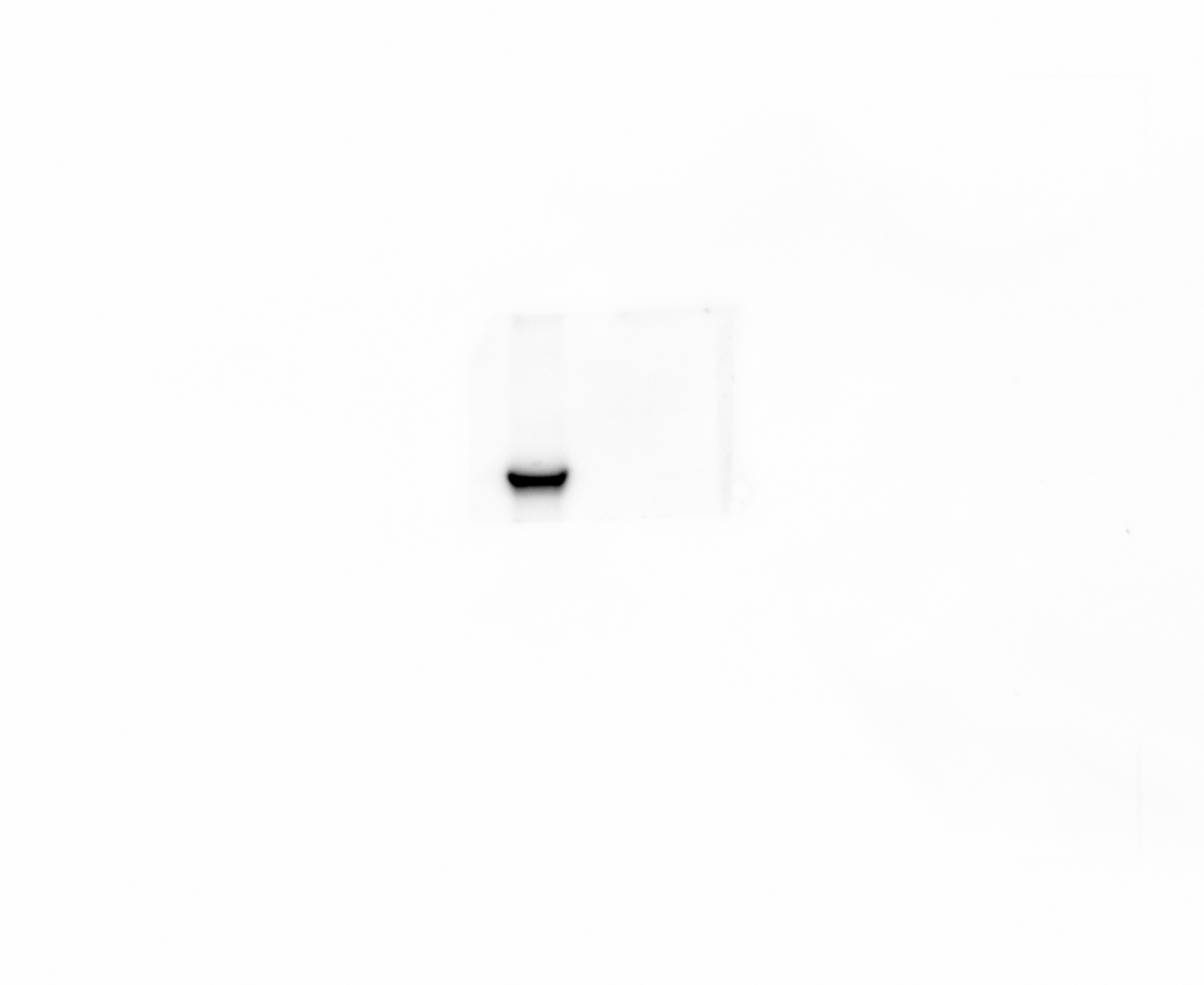

Supplement: Figure 4—source data 1. [file elife-98524-fig4-data1.zip › Fig 4-data1-v1/4D/bottom/U2AF2.tif]

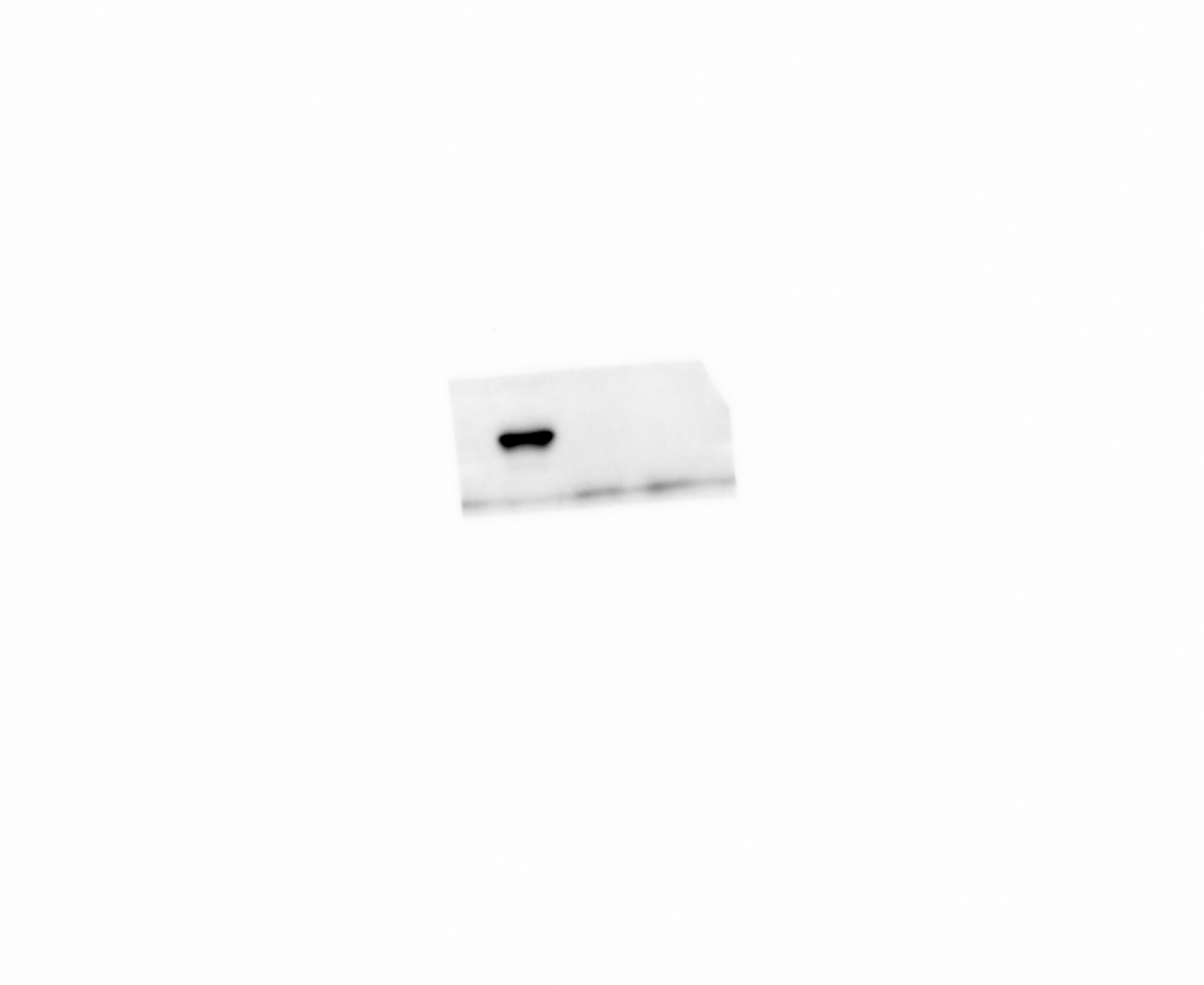

Supplement: Figure 4—source data 1. [file elife-98524-fig4-data1.zip › Fig 4-data1-v1/4D/upper/PUF60.tif]

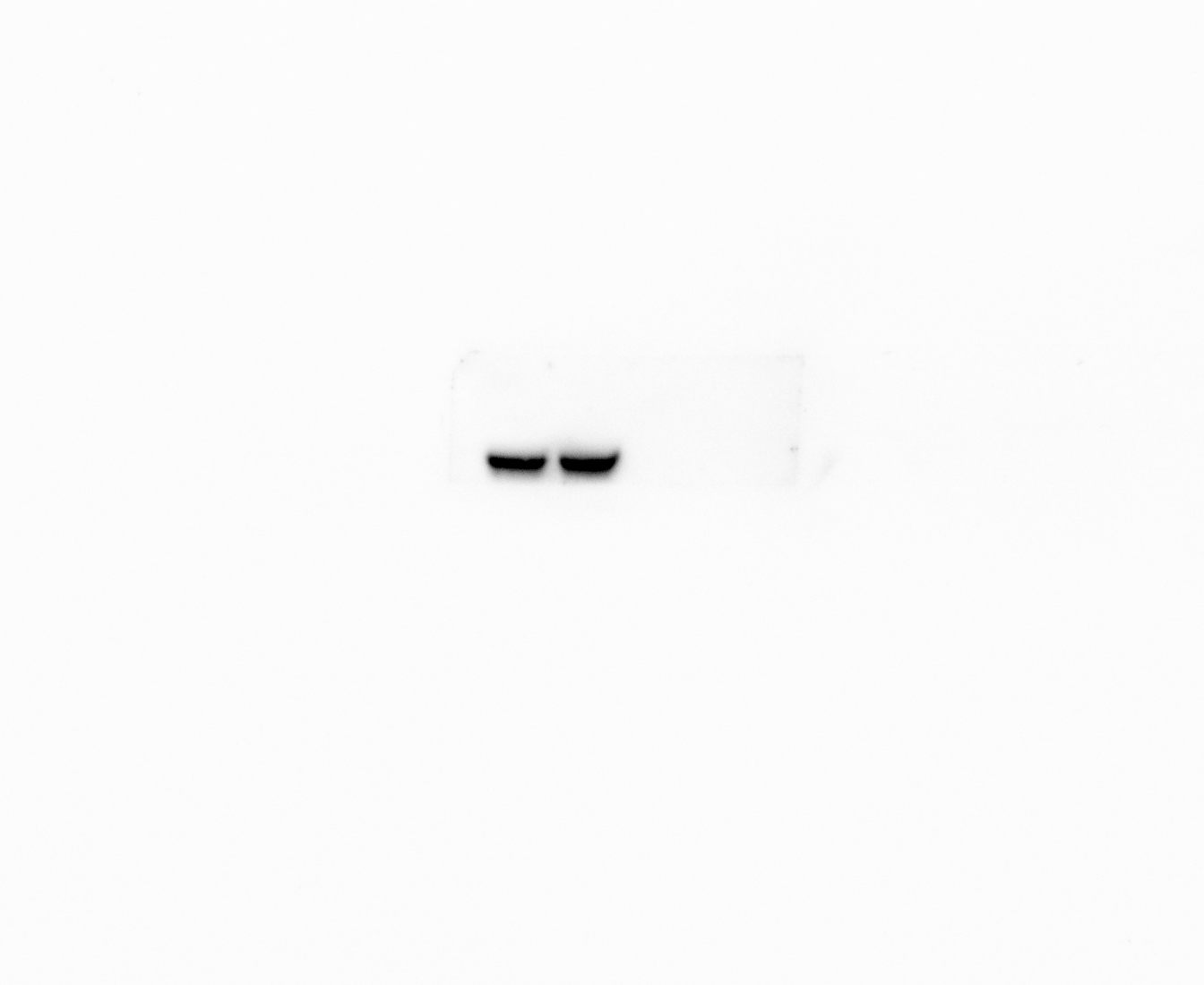

Supplement: Figure 4—source data 1. [file elife-98524-fig4-data1.zip › Fig 4-data1-v1/4D/upper/SITR4.tif]

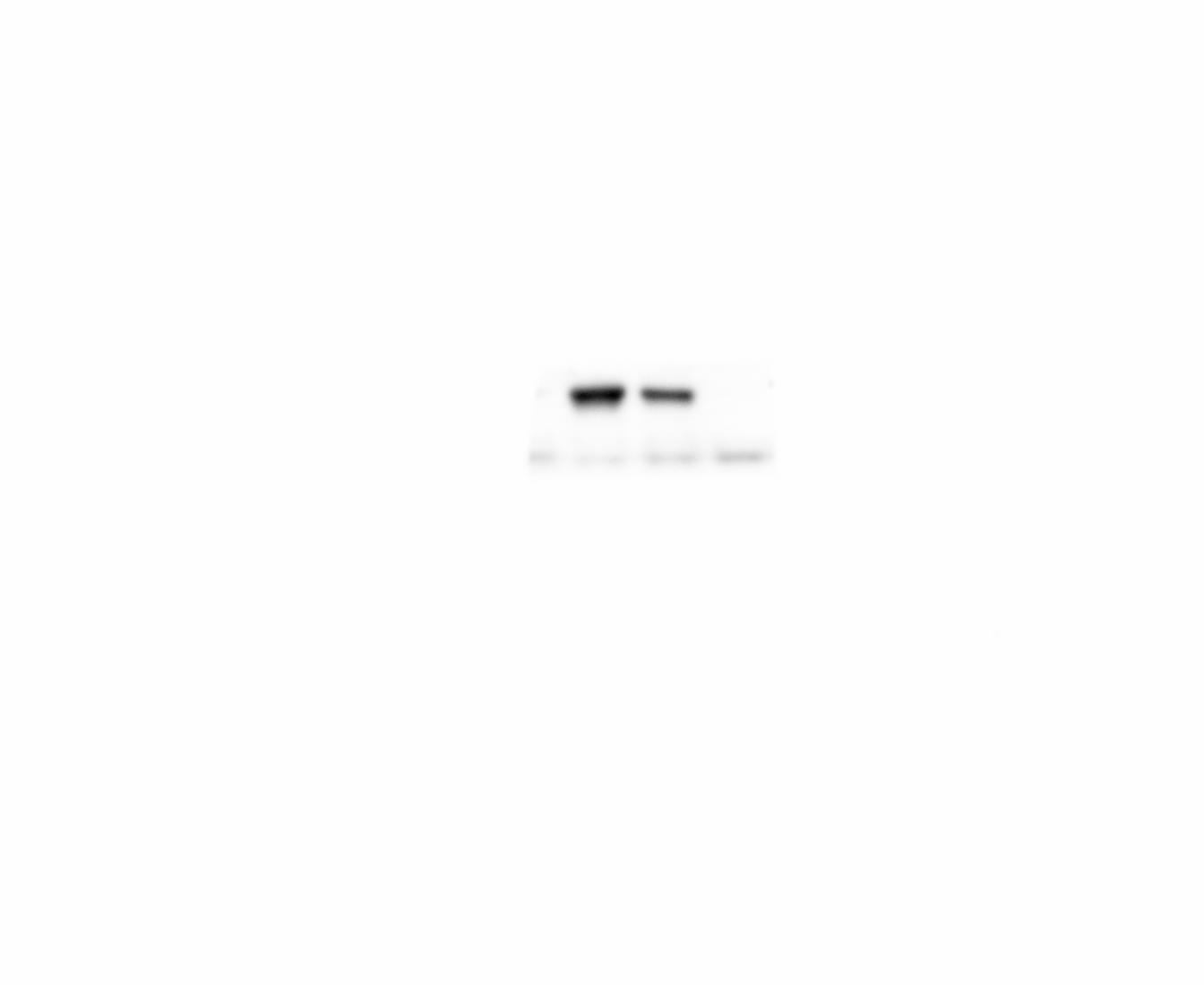

Supplement: Figure 4—source data 1. [file elife-98524-fig4-data1.zip › Fig 4-data1-v1/4E/AcK.tif]

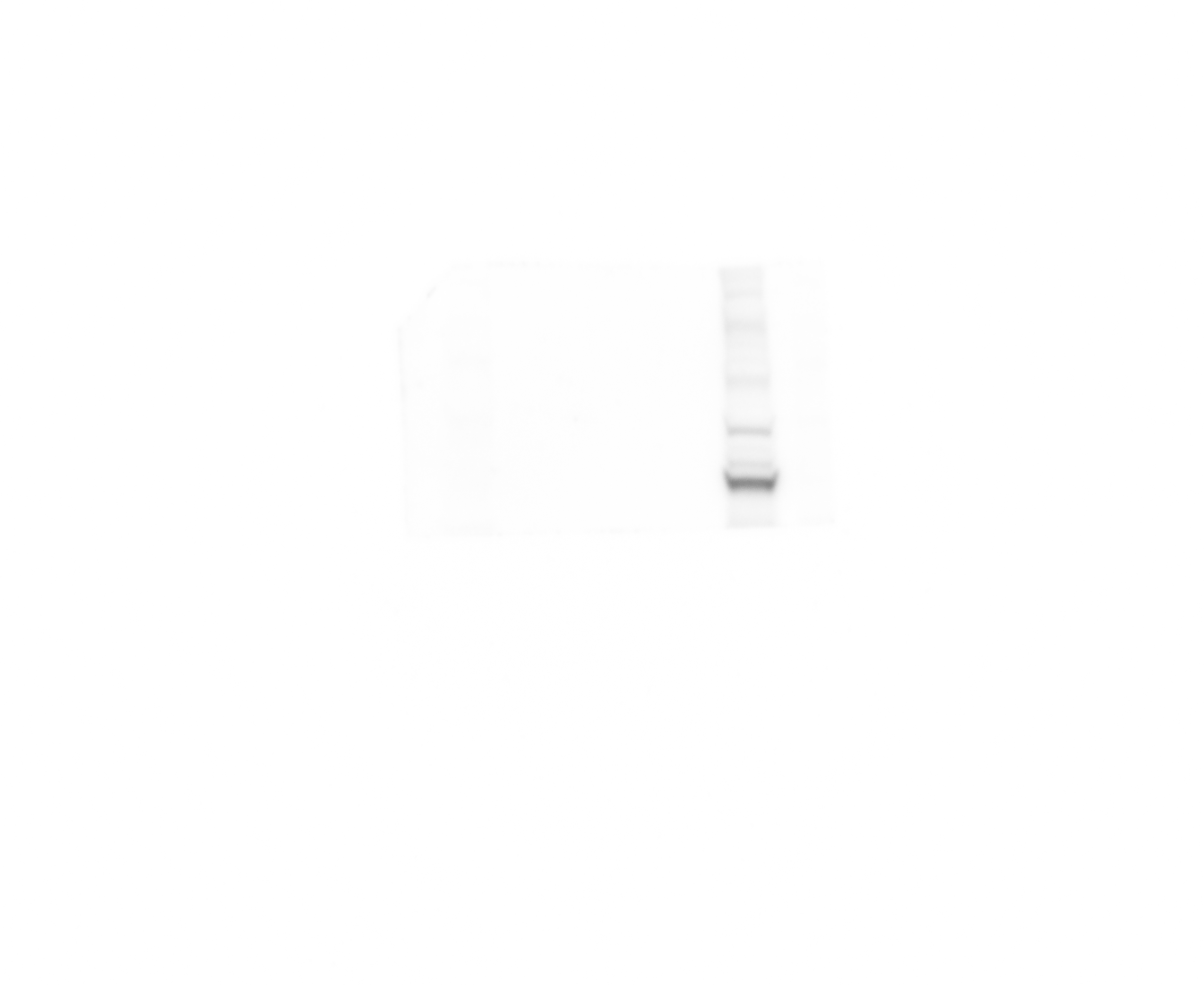

Supplement: Figure 4—source data 1. [file elife-98524-fig4-data1.zip › Fig 4-data1-v1/4E/ADPR.tif]

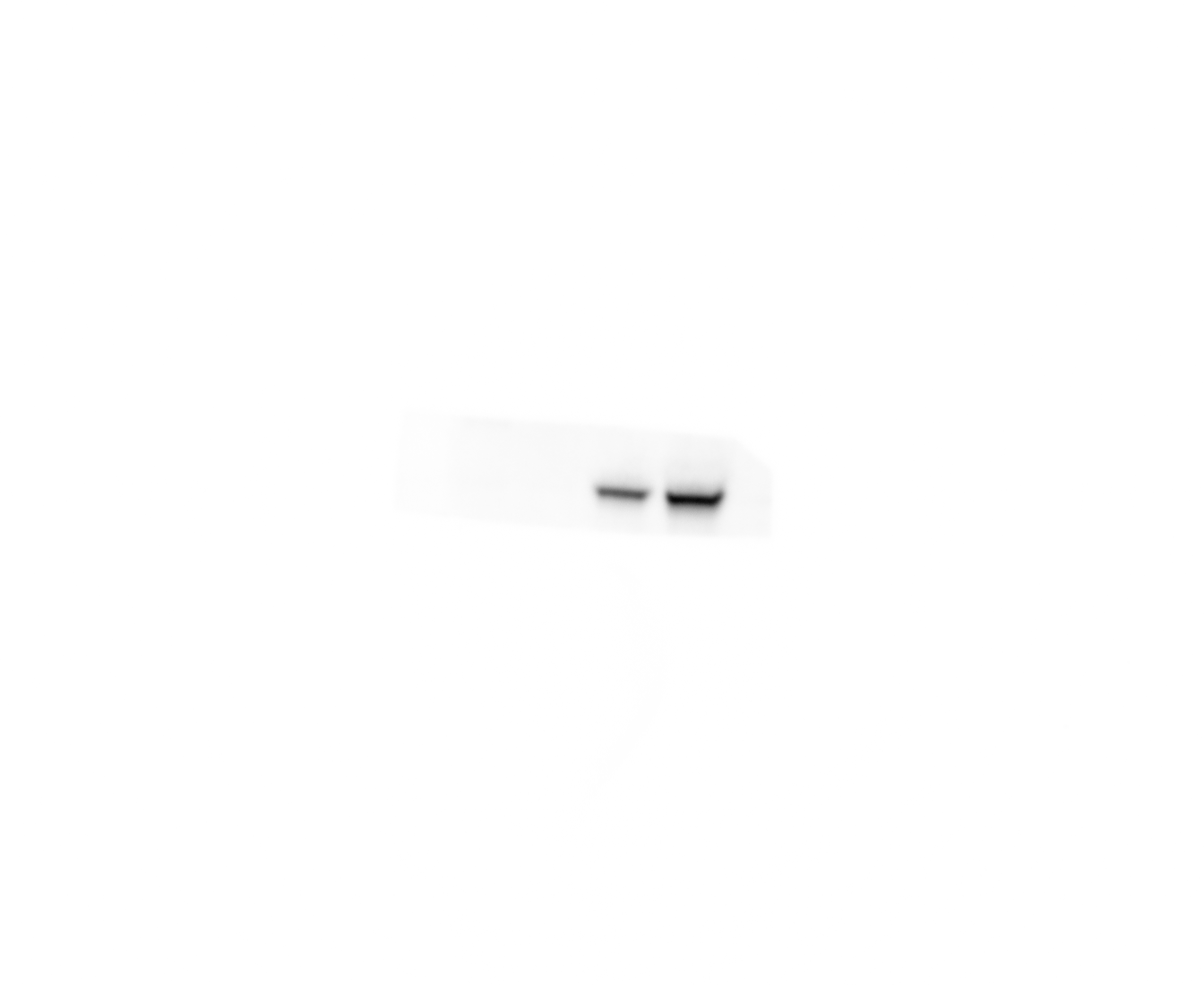

Supplement: Figure 4—source data 1. [file elife-98524-fig4-data1.zip › Fig 4-data1-v1/4E/SIRT4.tif]

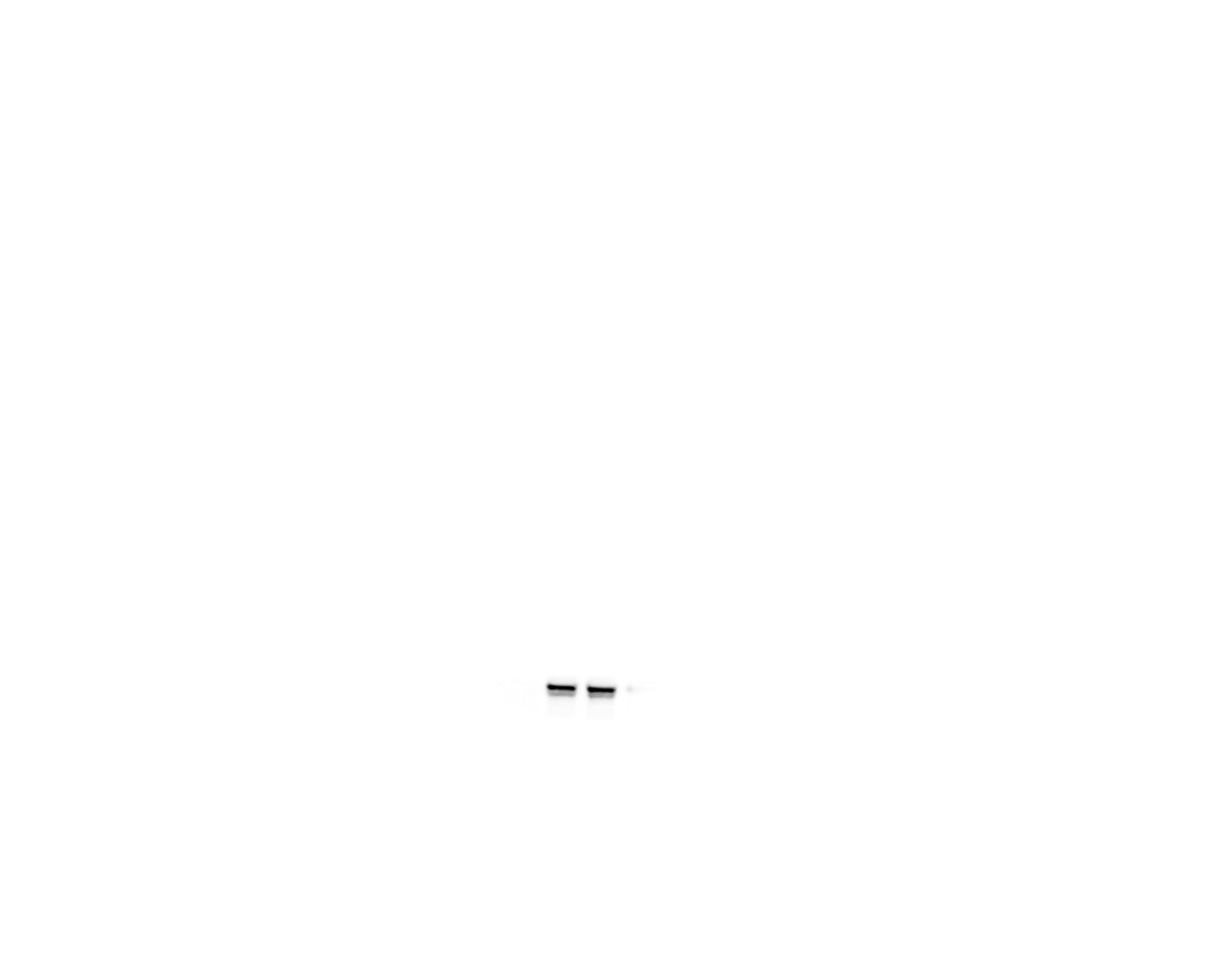

Supplement: Figure 4—source data 1. [file elife-98524-fig4-data1.zip › Fig 4-data1-v1/4E/Tubulin.tiff]

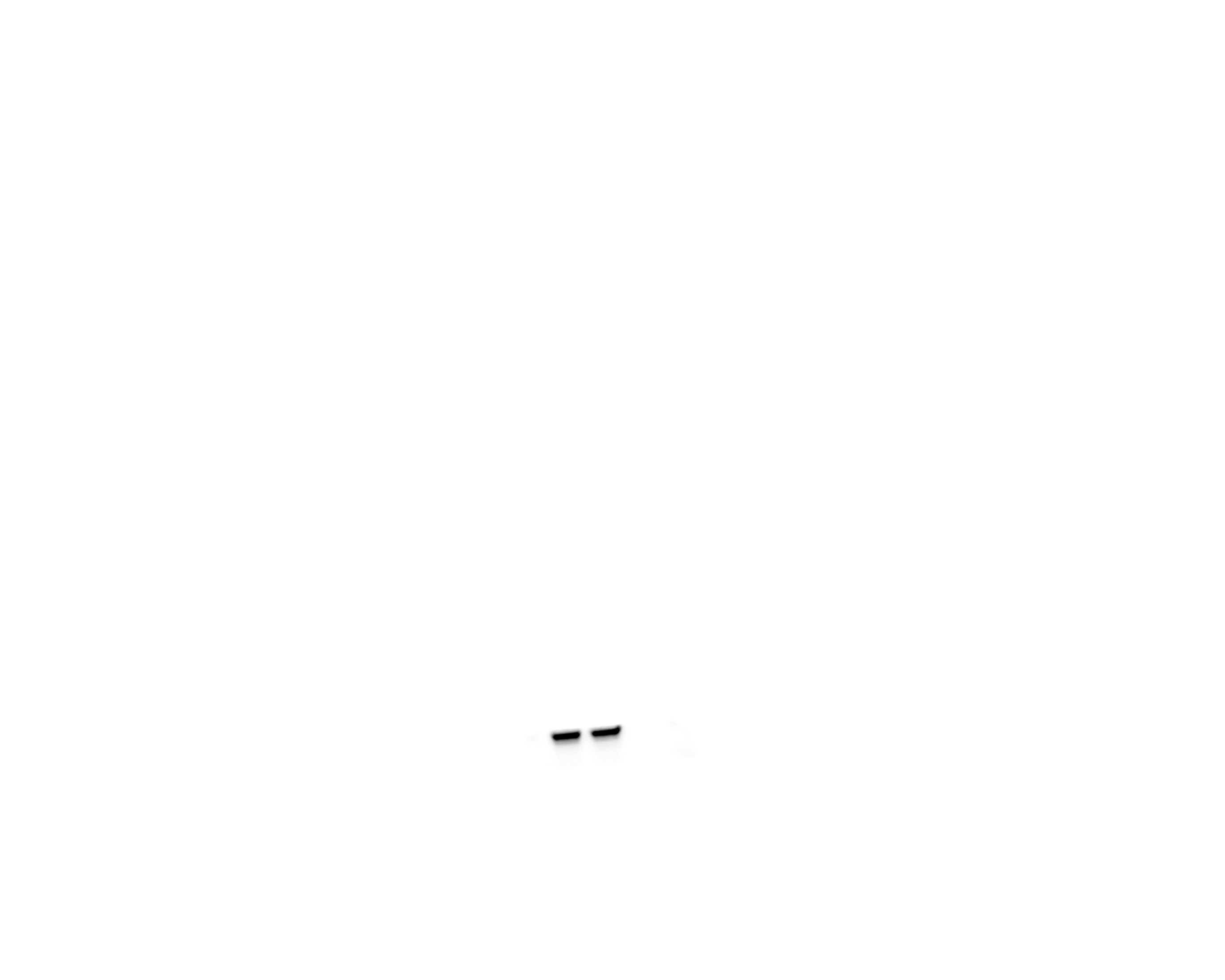

Supplement: Figure 4—source data 1. [file elife-98524-fig4-data1.zip › Fig 4-data1-v1/4E/U2AF2.tiff]

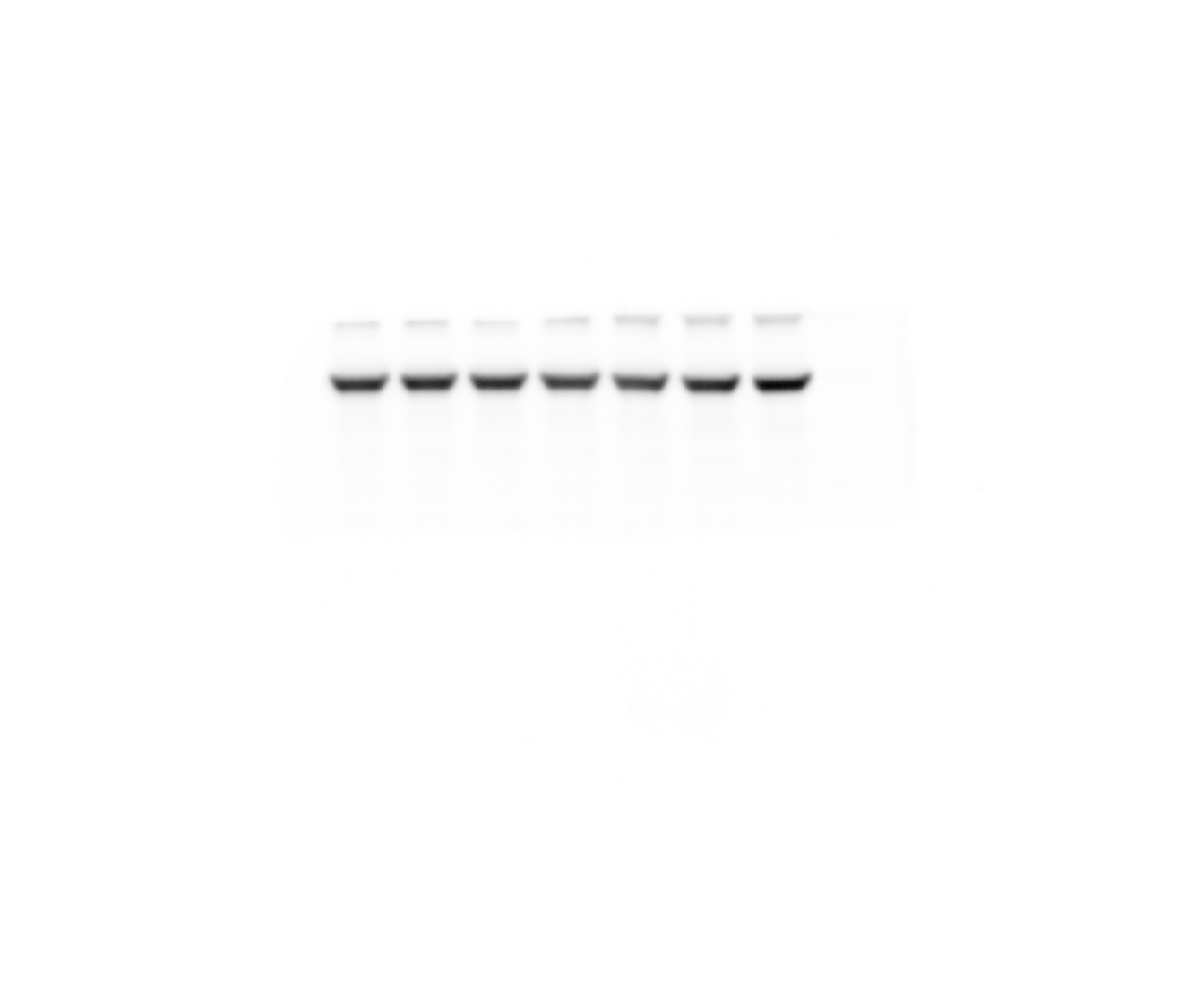

Supplement: Figure 4—source data 1. [file elife-98524-fig4-data1.zip › Fig 4-data1-v1/4F/bottom/Flag.tif]
