# Supplementary figures and images for: Nuclear translocation of SIRT4 mediates deacetylation of U2AF2 to modulate renal fibrosis through alternative splicing-mediated upregulation of CCN2 (part 5 of 9)
Source: eLife. 2024 Nov 4;13:RP98524. doi: 10.7554/eLife.98524 (PMC11534337; doi:10.7554/eLife.98524)

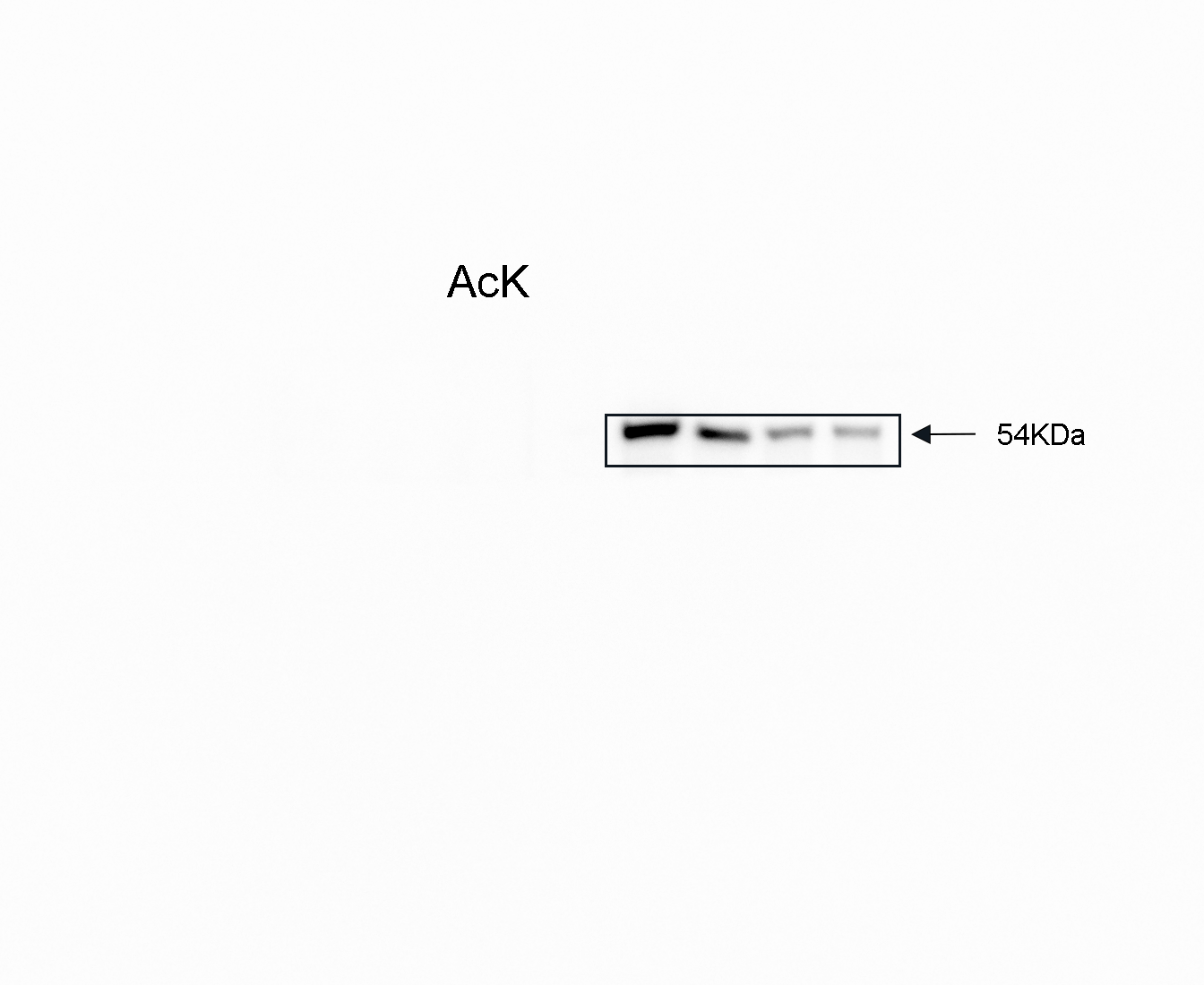

Supplement: Figure 4—source data 2. [file elife-98524-fig4-data2.zip › Fig 4-data2-v1/4Q/upper right/Ack .tif]

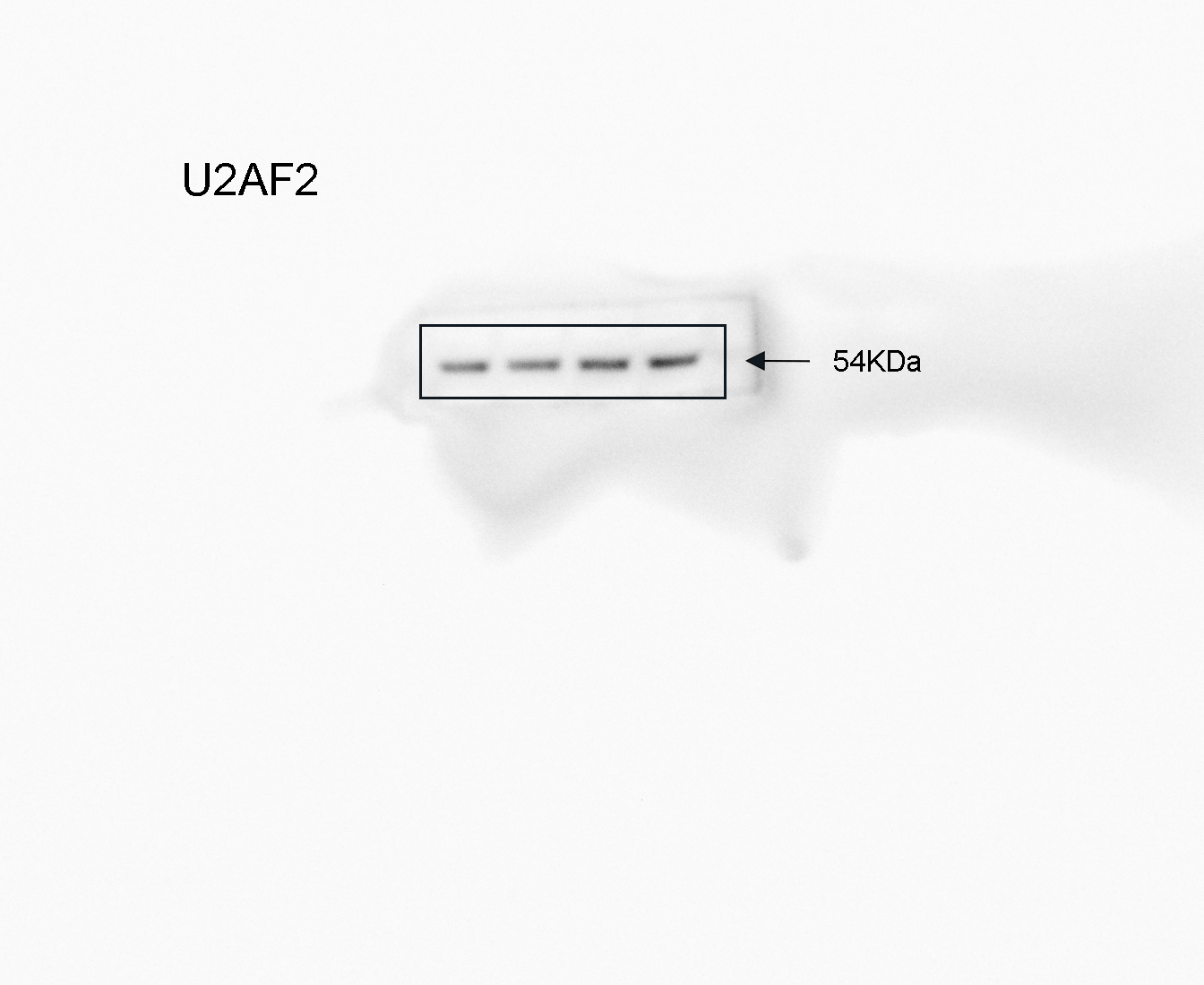

Supplement: Figure 4—source data 2. [file elife-98524-fig4-data2.zip › Fig 4-data2-v1/4Q/upper right/U2AF2 .tif]

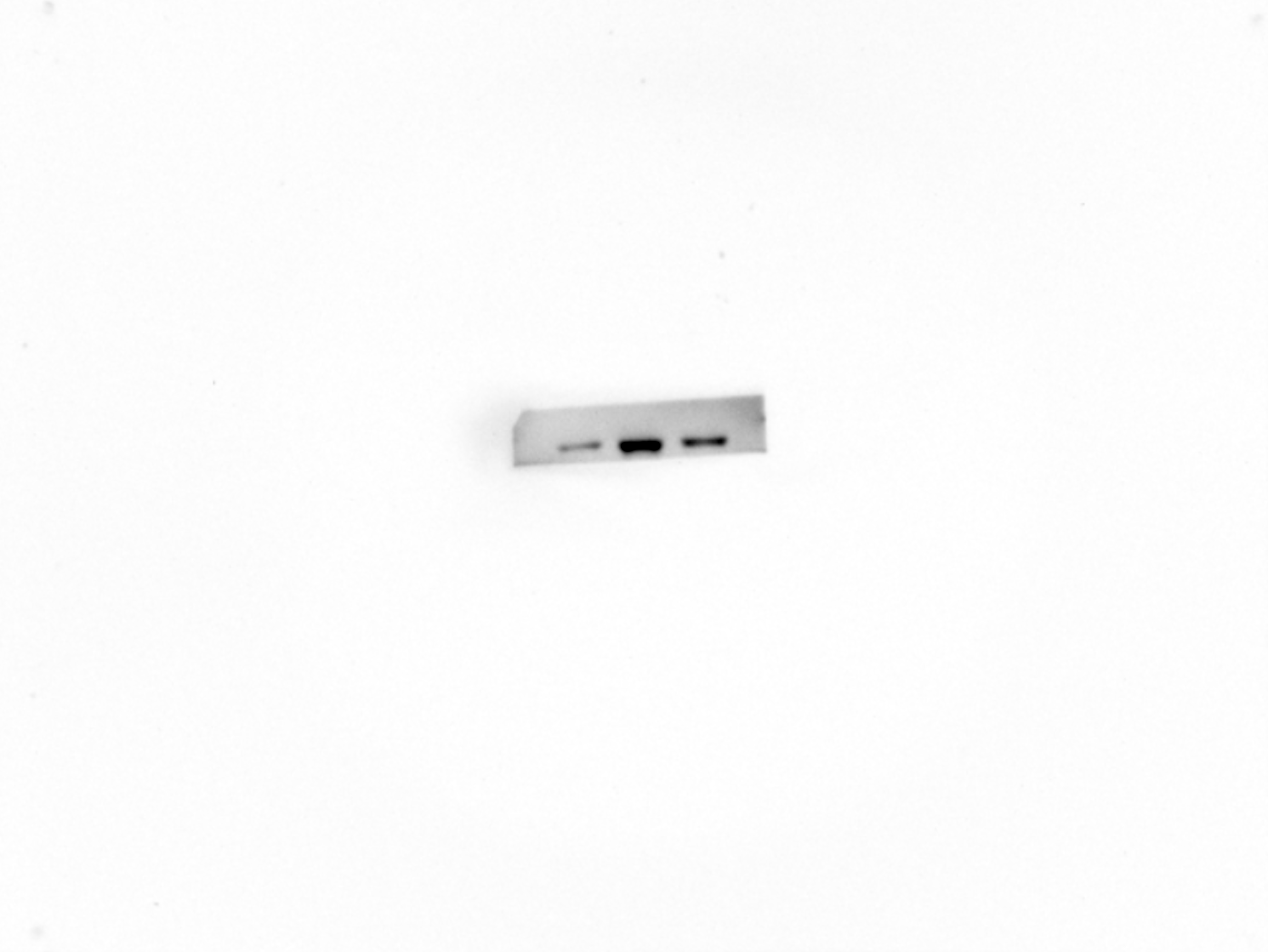

Supplement: Figure 5—source data 1. [file elife-98524-fig5-data1.zip › Fig 5-data1-v1/5A/left/Ac-k left.tif]

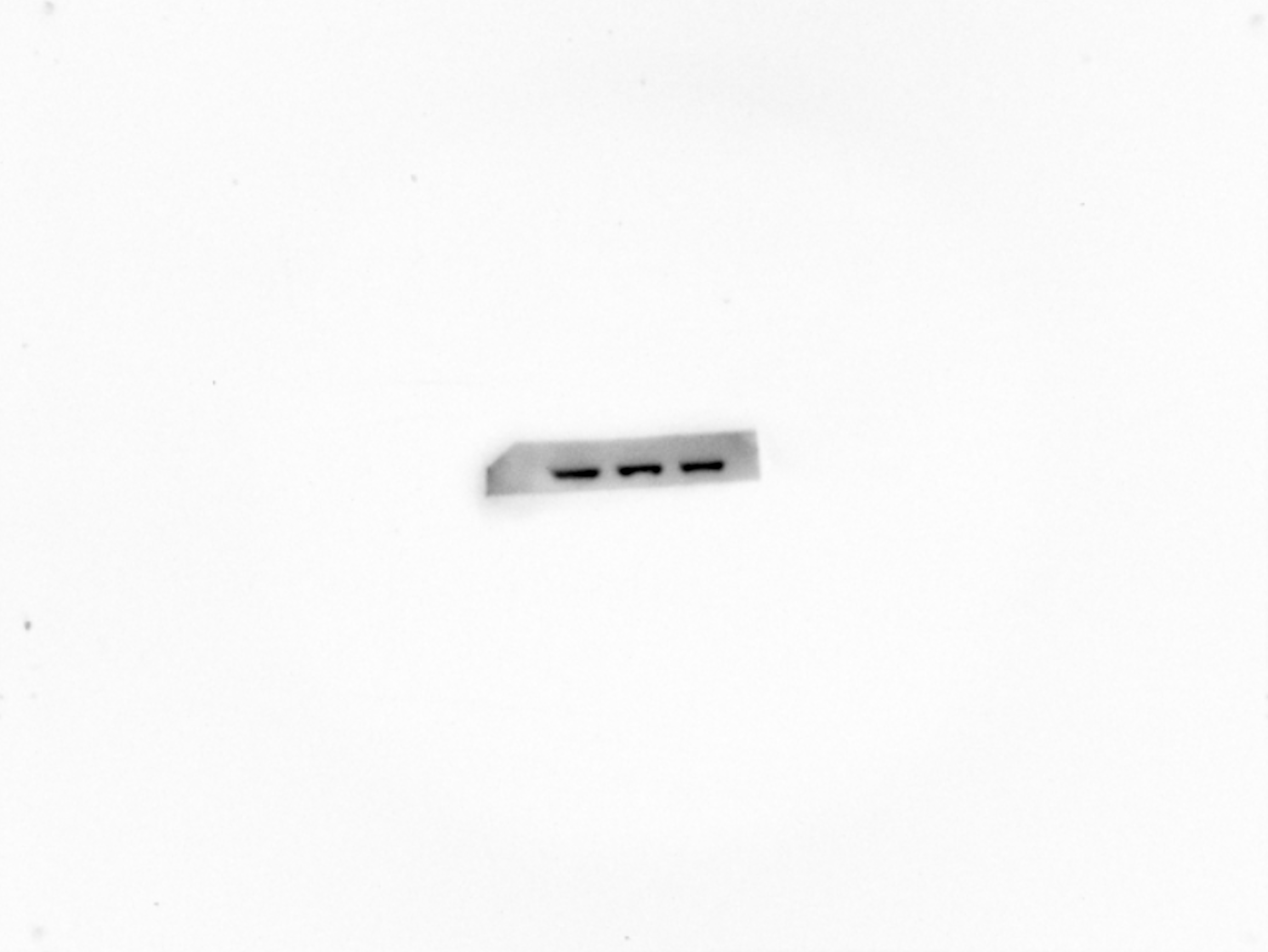

Supplement: Figure 5—source data 1. [file elife-98524-fig5-data1.zip › Fig 5-data1-v1/5A/left/Flag bottom left.tif]

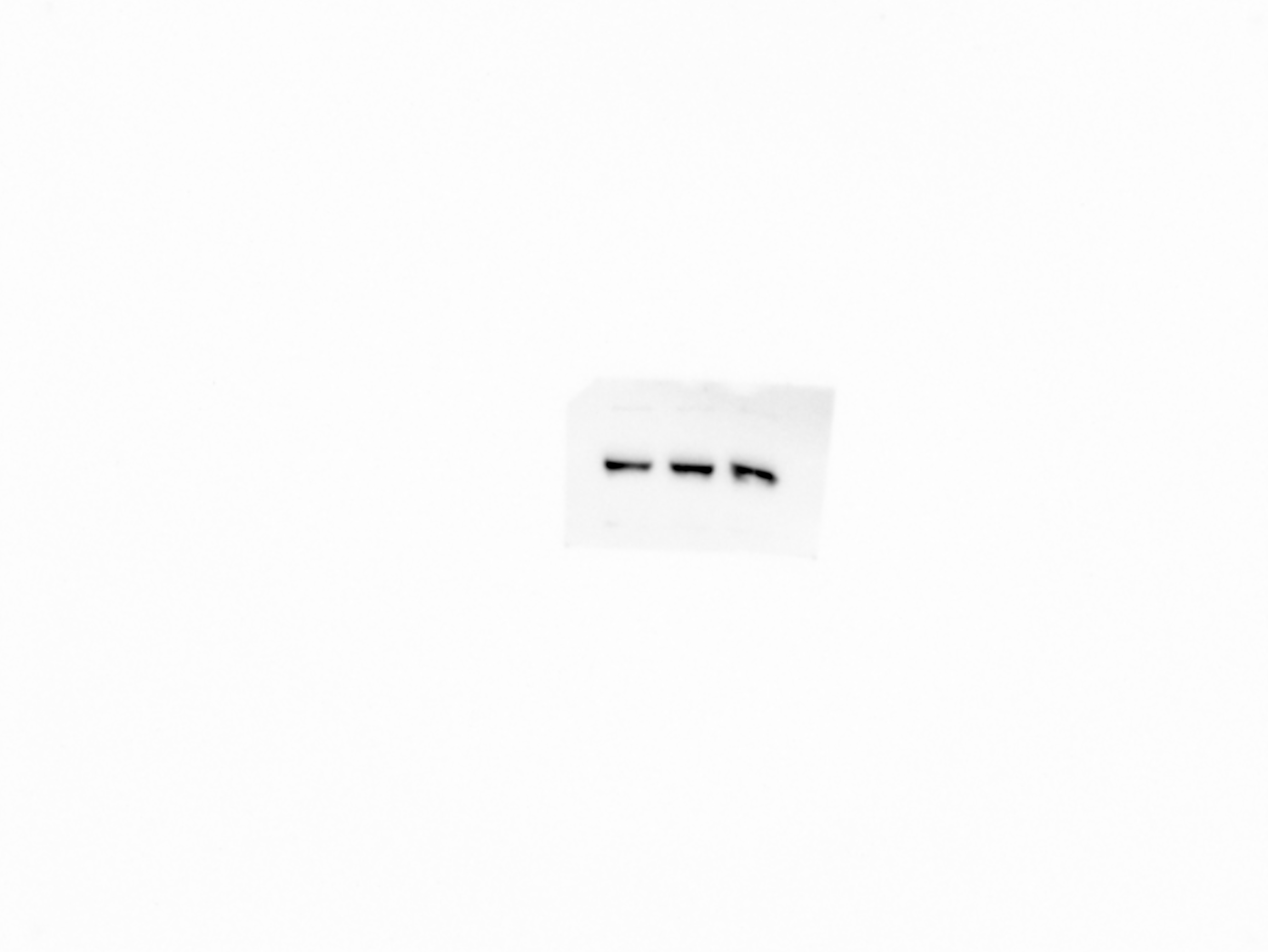

Supplement: Figure 5—source data 1. [file elife-98524-fig5-data1.zip › Fig 5-data1-v1/5A/left/Flag upper left.tif]

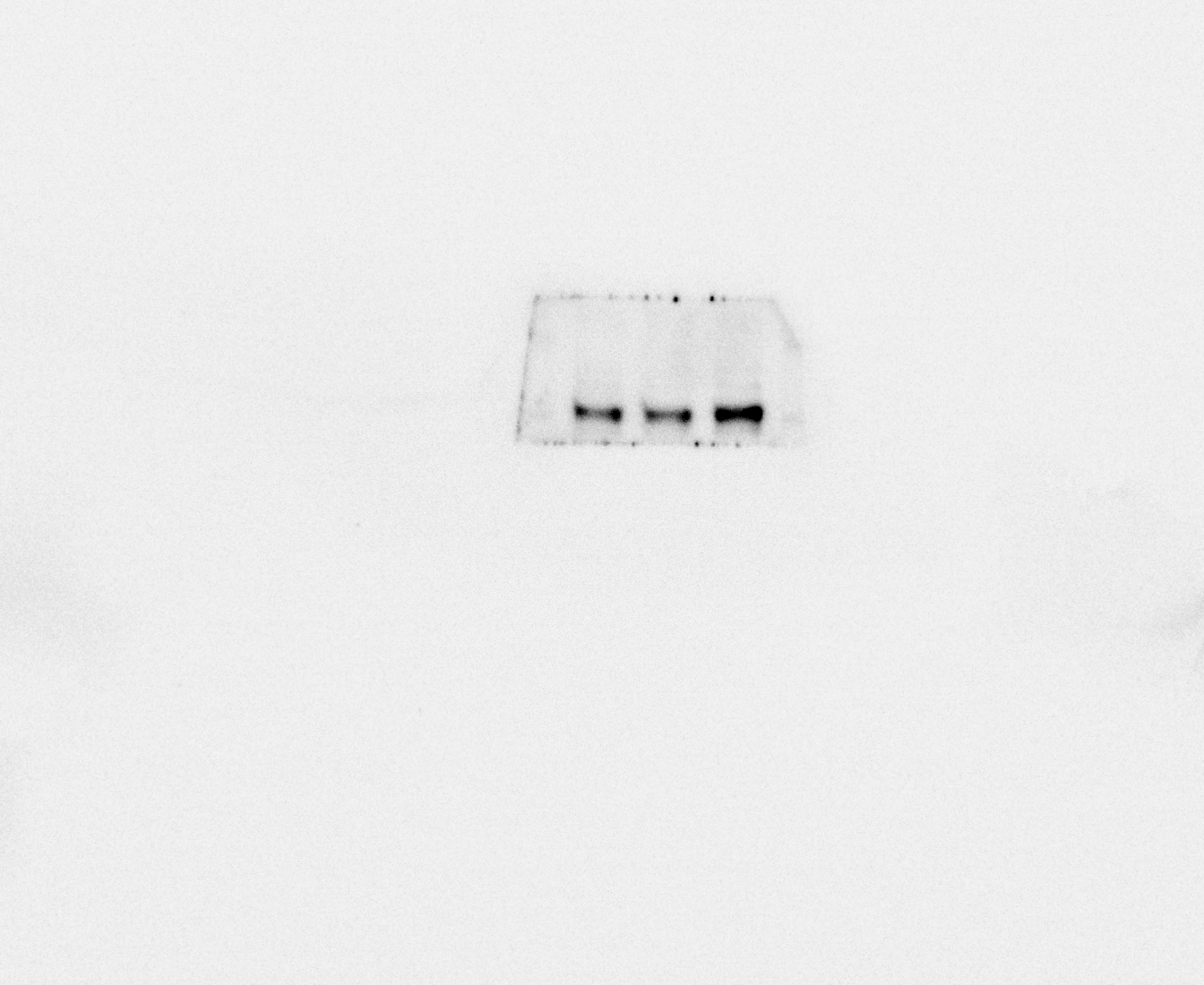

Supplement: Figure 5—source data 1. [file elife-98524-fig5-data1.zip › Fig 5-data1-v1/5A/left/SIRT4 left.tif]

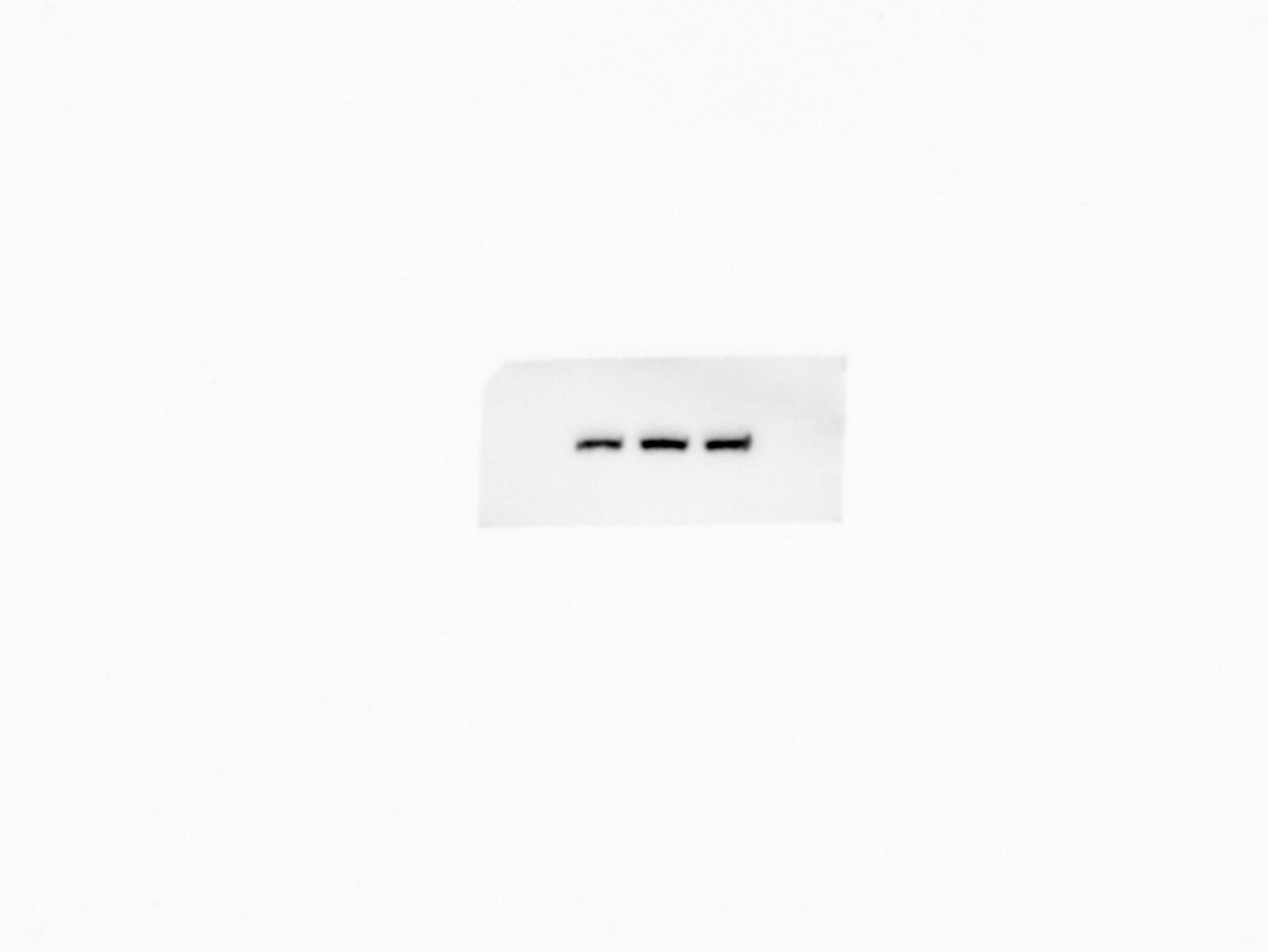

Supplement: Figure 5—source data 1. [file elife-98524-fig5-data1.zip › Fig 5-data1-v1/5A/left/Tubulin left.tif]

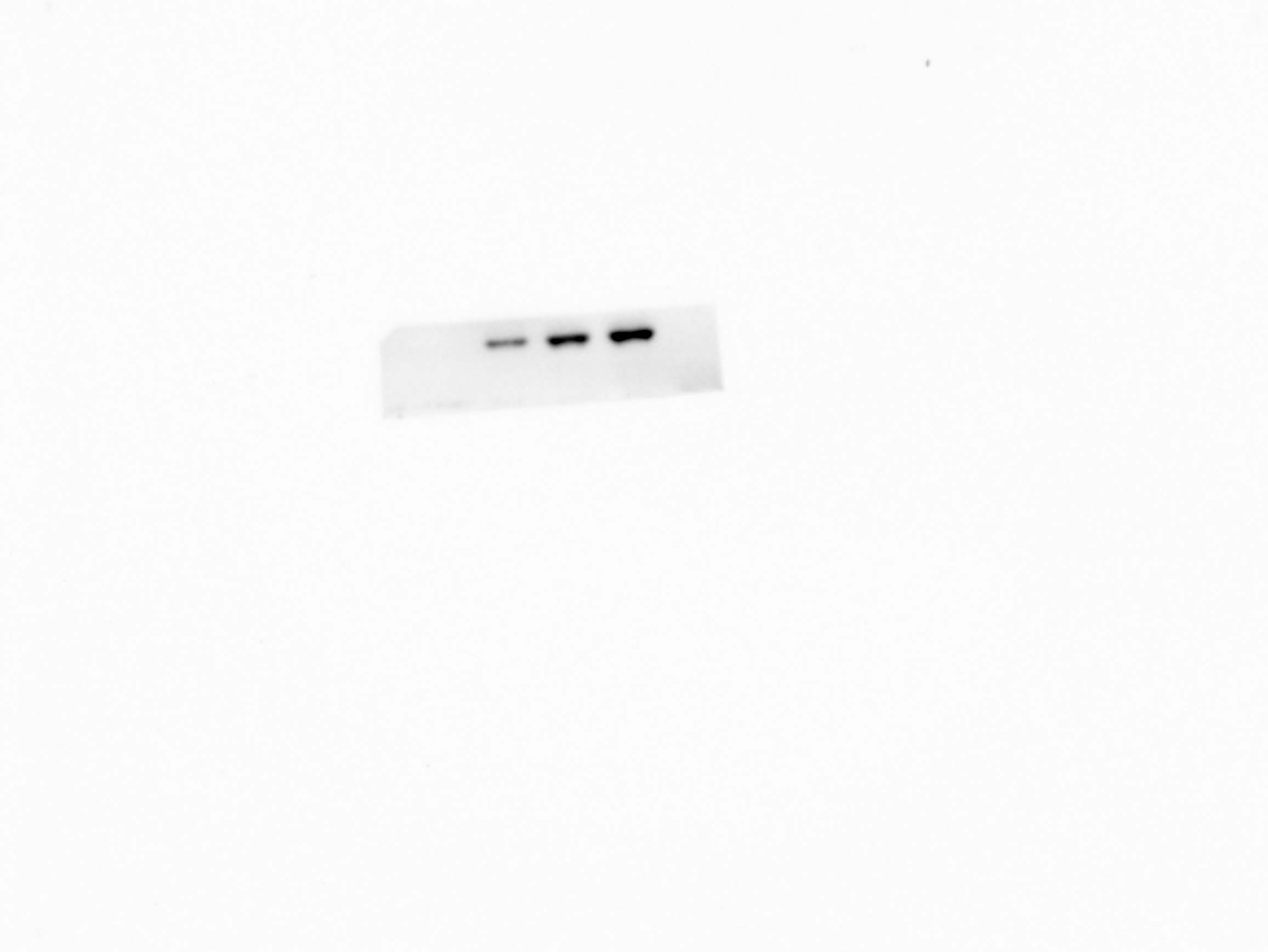

Supplement: Figure 5—source data 1. [file elife-98524-fig5-data1.zip › Fig 5-data1-v1/5A/right/Ac-k right.tif]

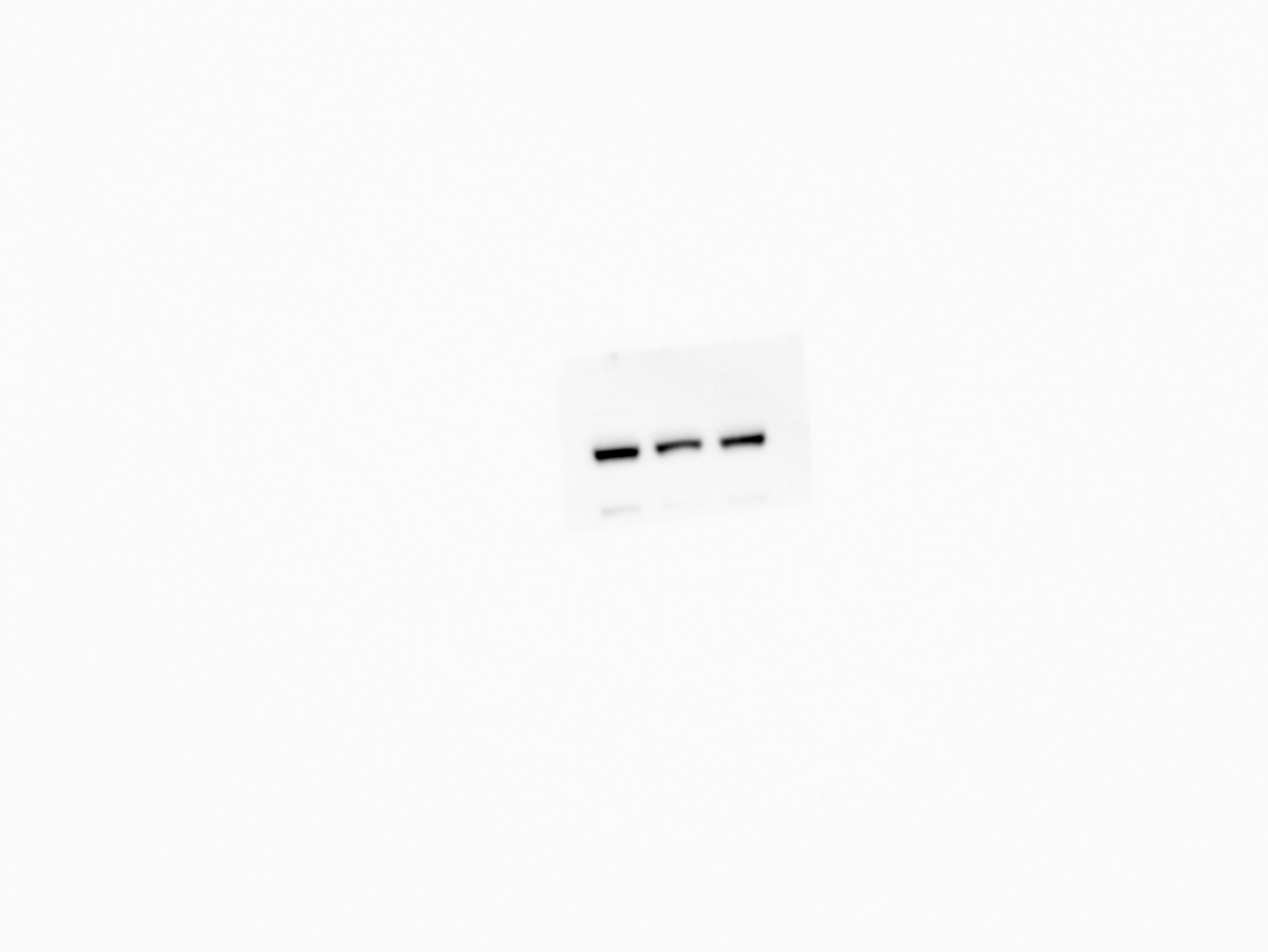

Supplement: Figure 5—source data 1. [file elife-98524-fig5-data1.zip › Fig 5-data1-v1/5A/right/Flag bottom right.tif]

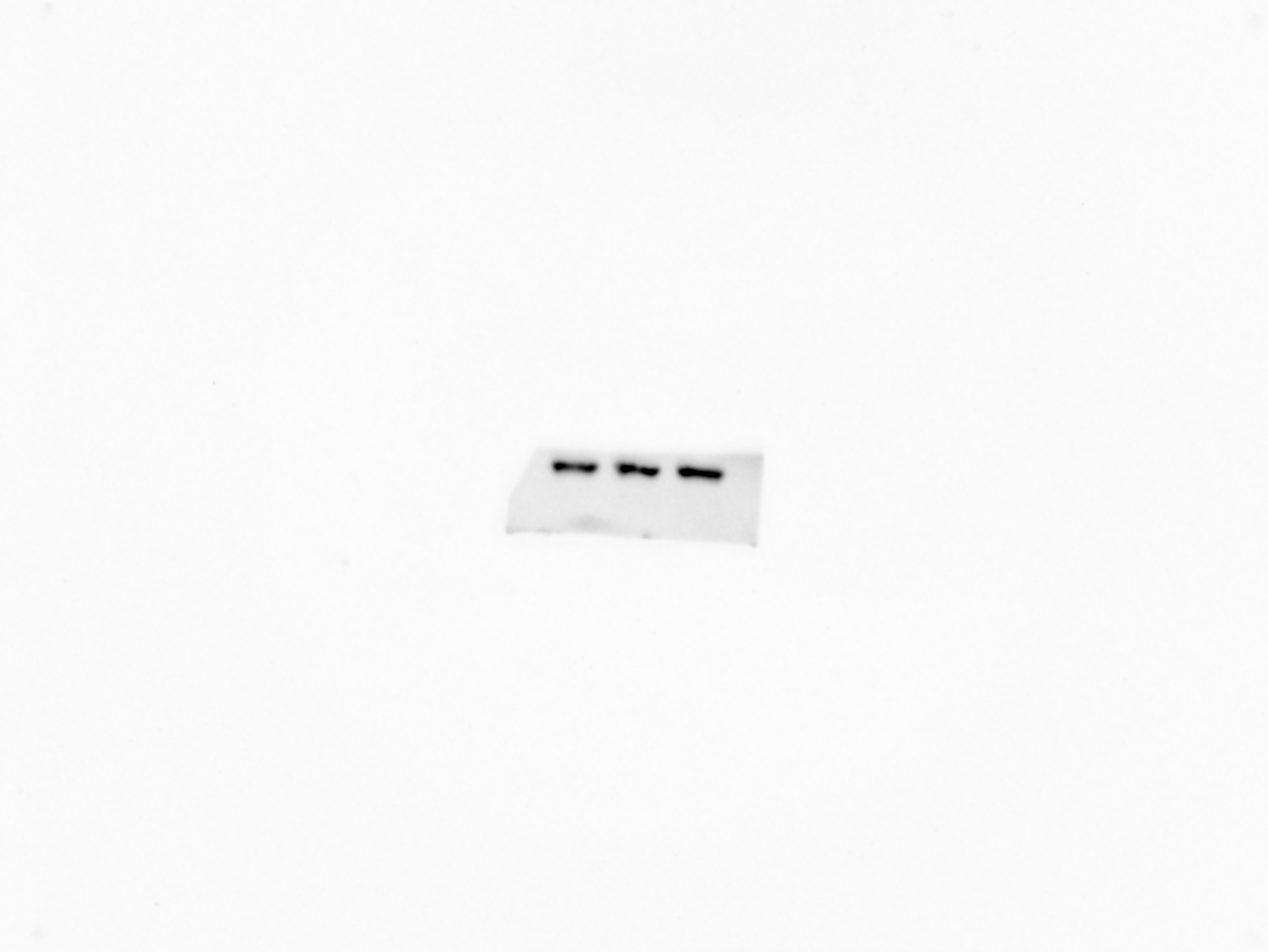

Supplement: Figure 5—source data 1. [file elife-98524-fig5-data1.zip › Fig 5-data1-v1/5A/right/Flag upper right.tif]

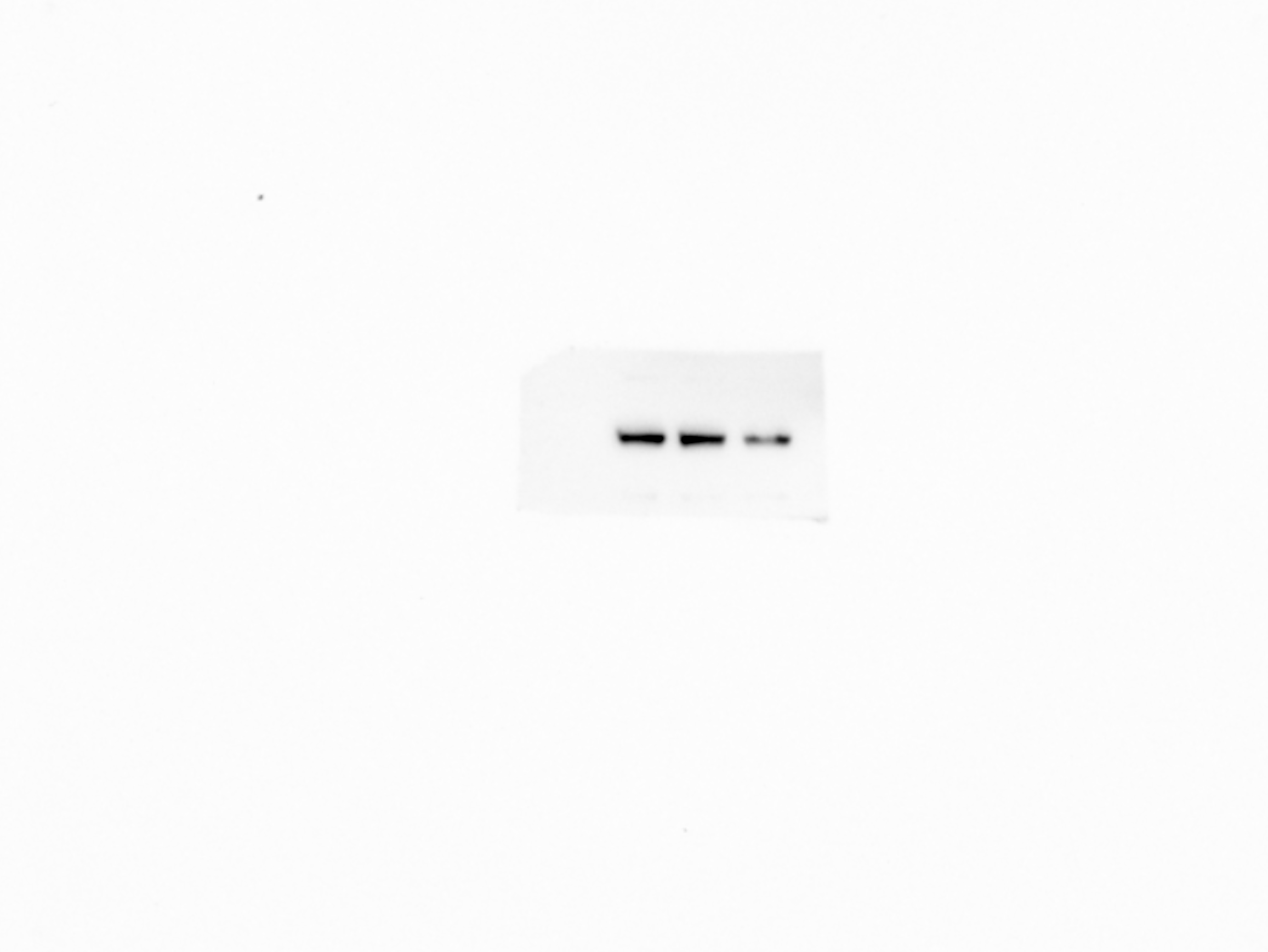

Supplement: Figure 5—source data 1. [file elife-98524-fig5-data1.zip › Fig 5-data1-v1/5A/right/SRIT4 right.tif]

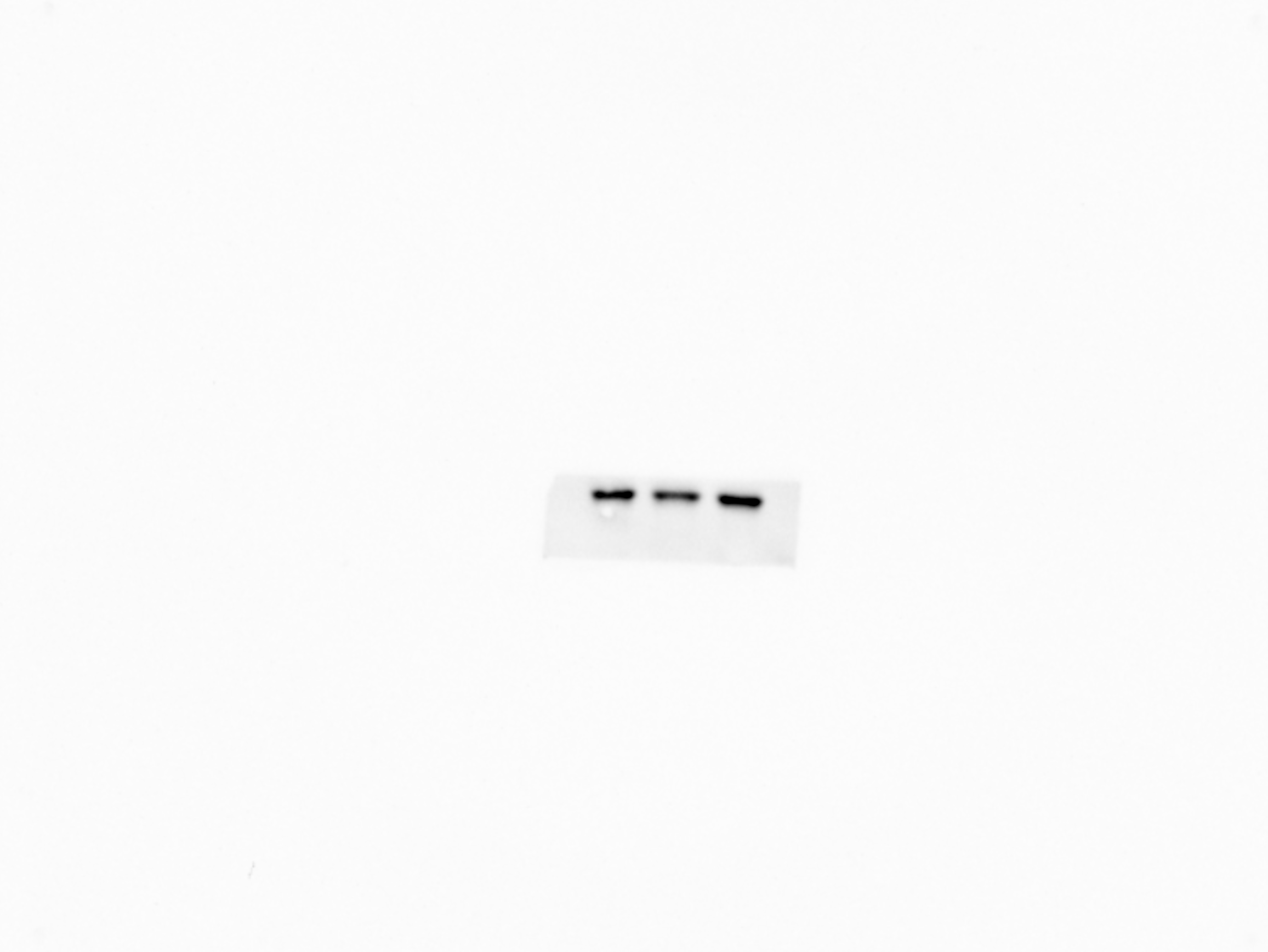

Supplement: Figure 5—source data 1. [file elife-98524-fig5-data1.zip › Fig 5-data1-v1/5A/right/Tubulin right.tif]

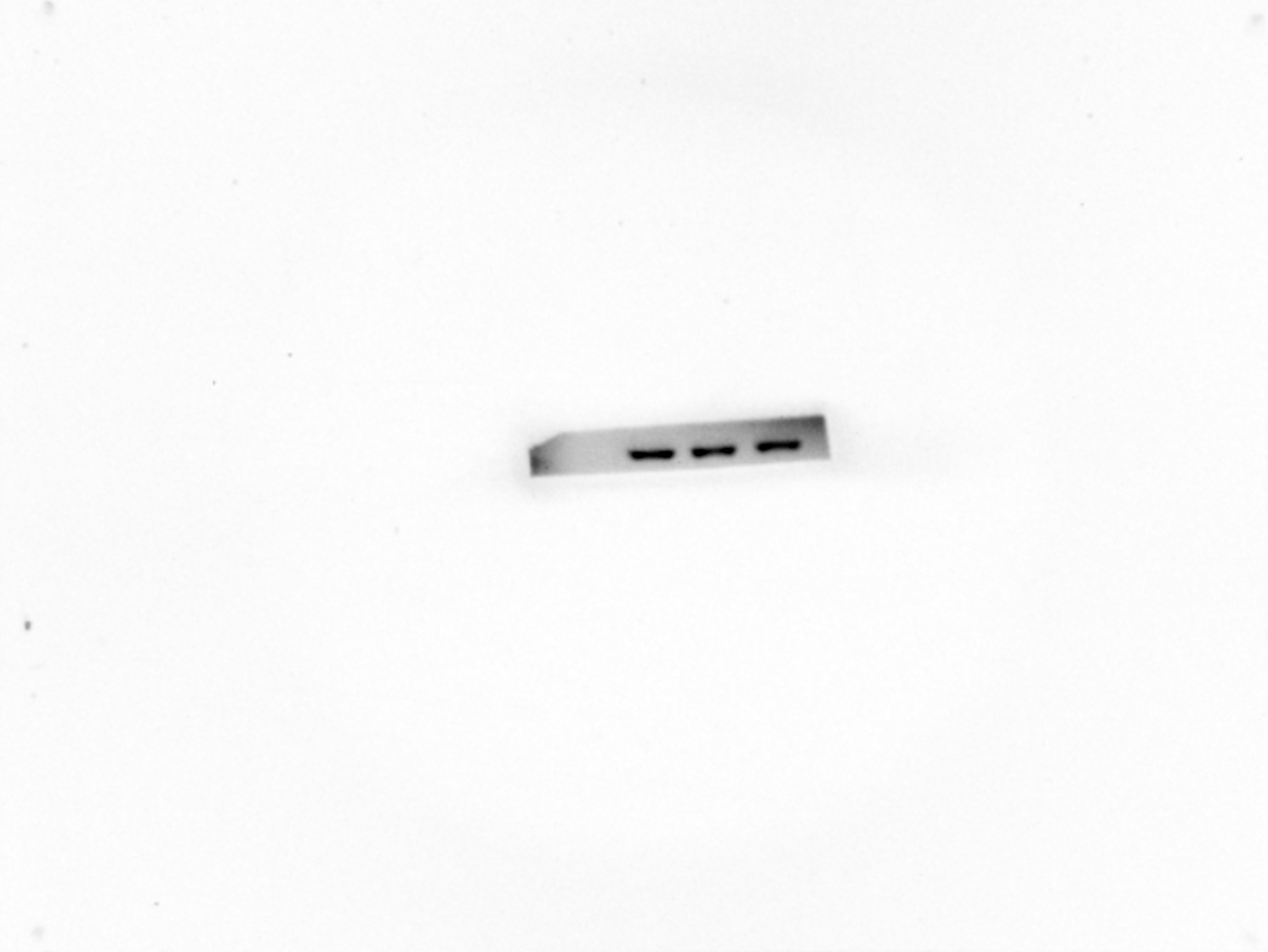

Supplement: Figure 5—source data 1. [file elife-98524-fig5-data1.zip › Fig 5-data1-v1/5B/bottom/Flag bottom.tif]

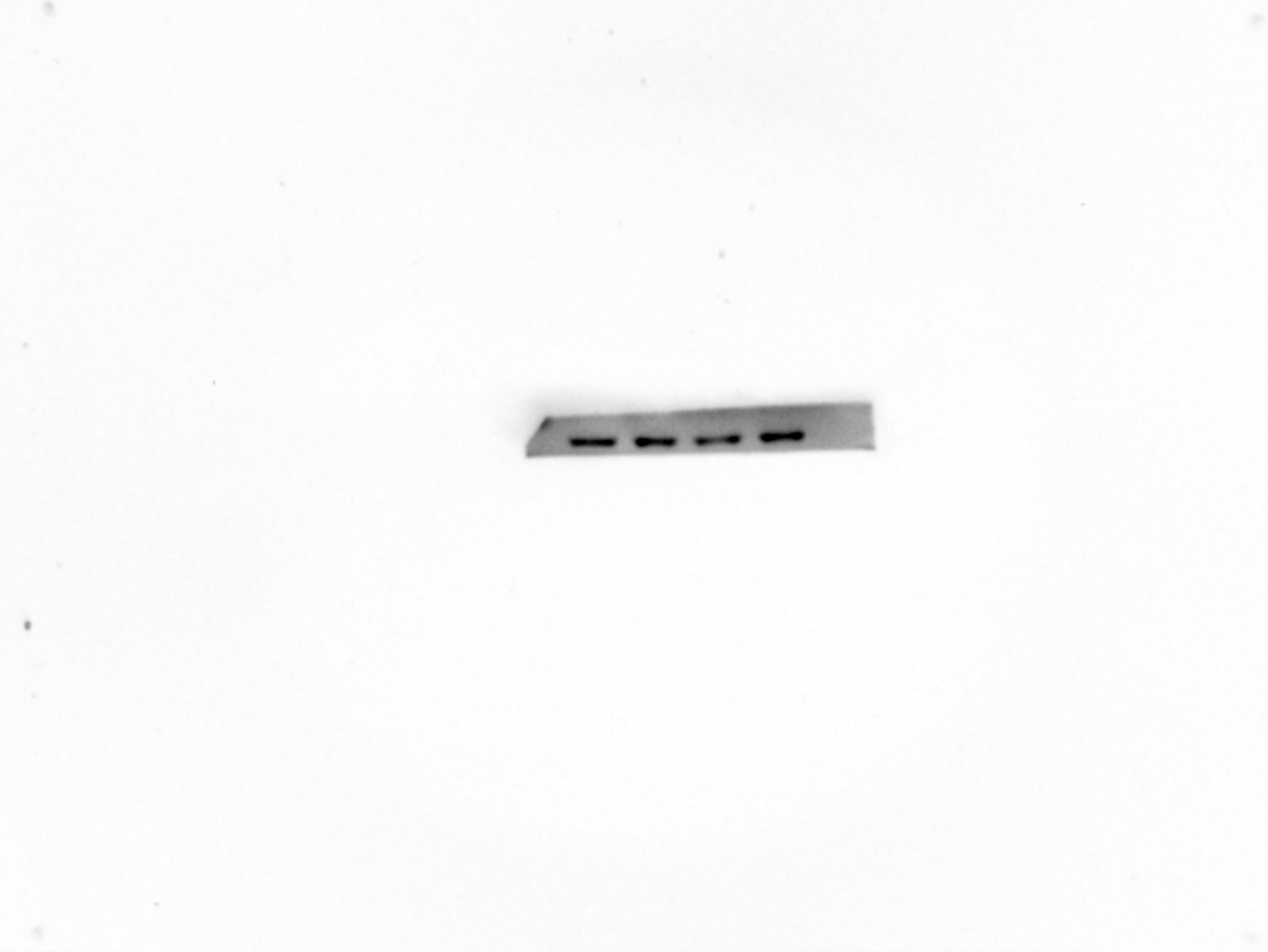

Supplement: Figure 5—source data 1. [file elife-98524-fig5-data1.zip › Fig 5-data1-v1/5B/bottom/HA bottom.tif]

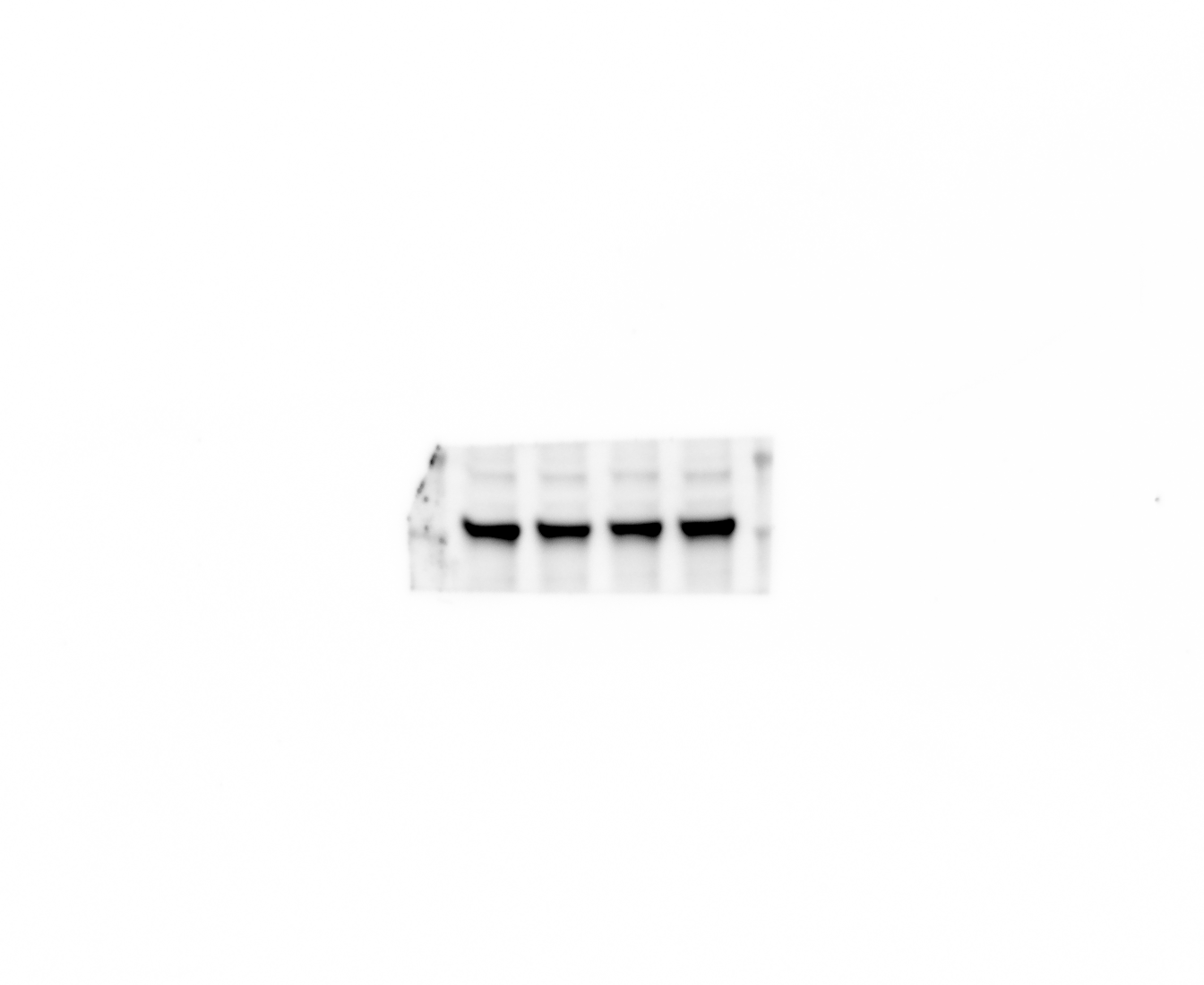

Supplement: Figure 5—source data 1. [file elife-98524-fig5-data1.zip › Fig 5-data1-v1/5B/bottom/Tubulin.tif]

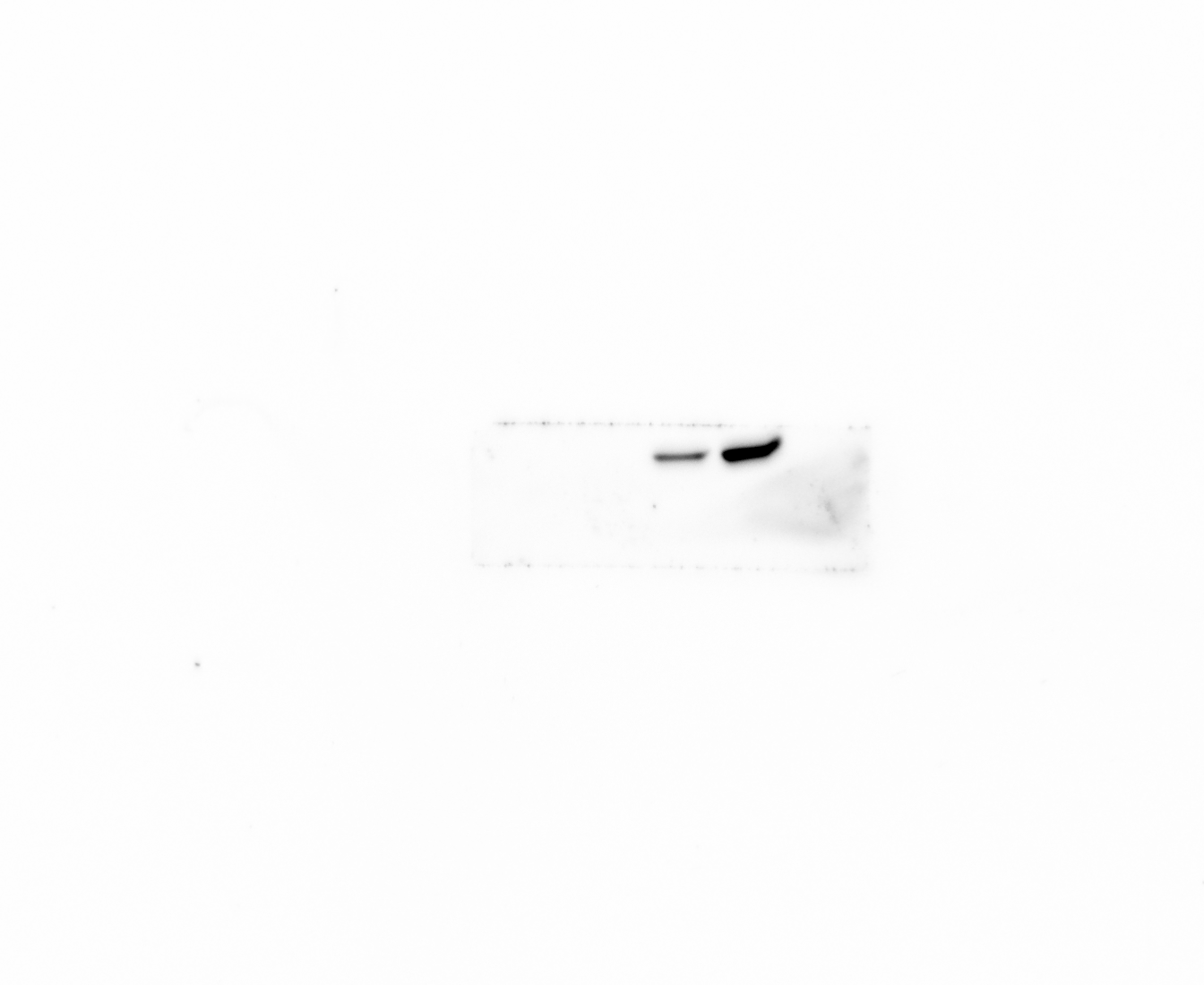

Supplement: Figure 5—source data 1. [file elife-98524-fig5-data1.zip › Fig 5-data1-v1/5B/upper/Flag upper.tif]

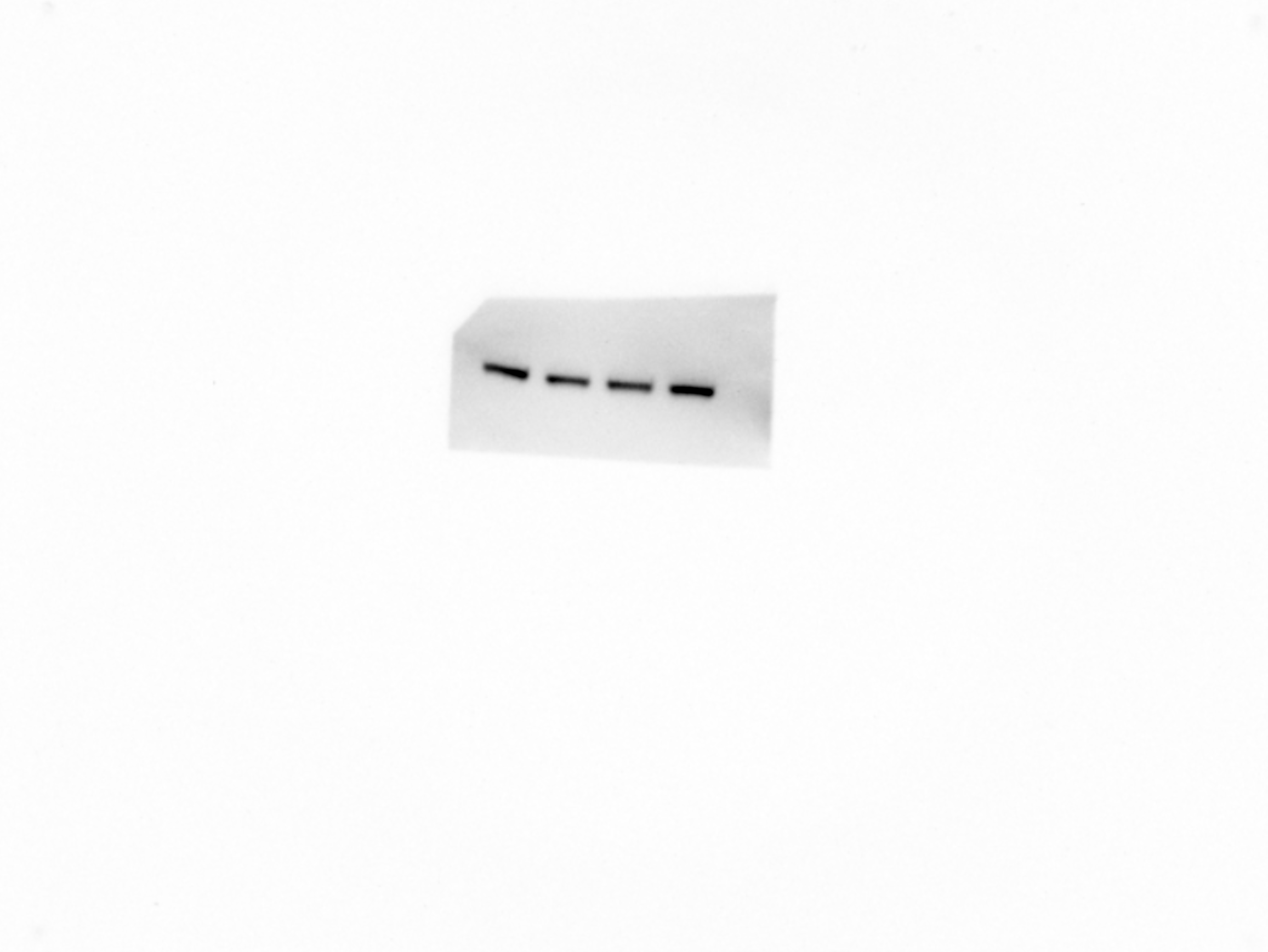

Supplement: Figure 5—source data 1. [file elife-98524-fig5-data1.zip › Fig 5-data1-v1/5B/upper/HA upper.tif]

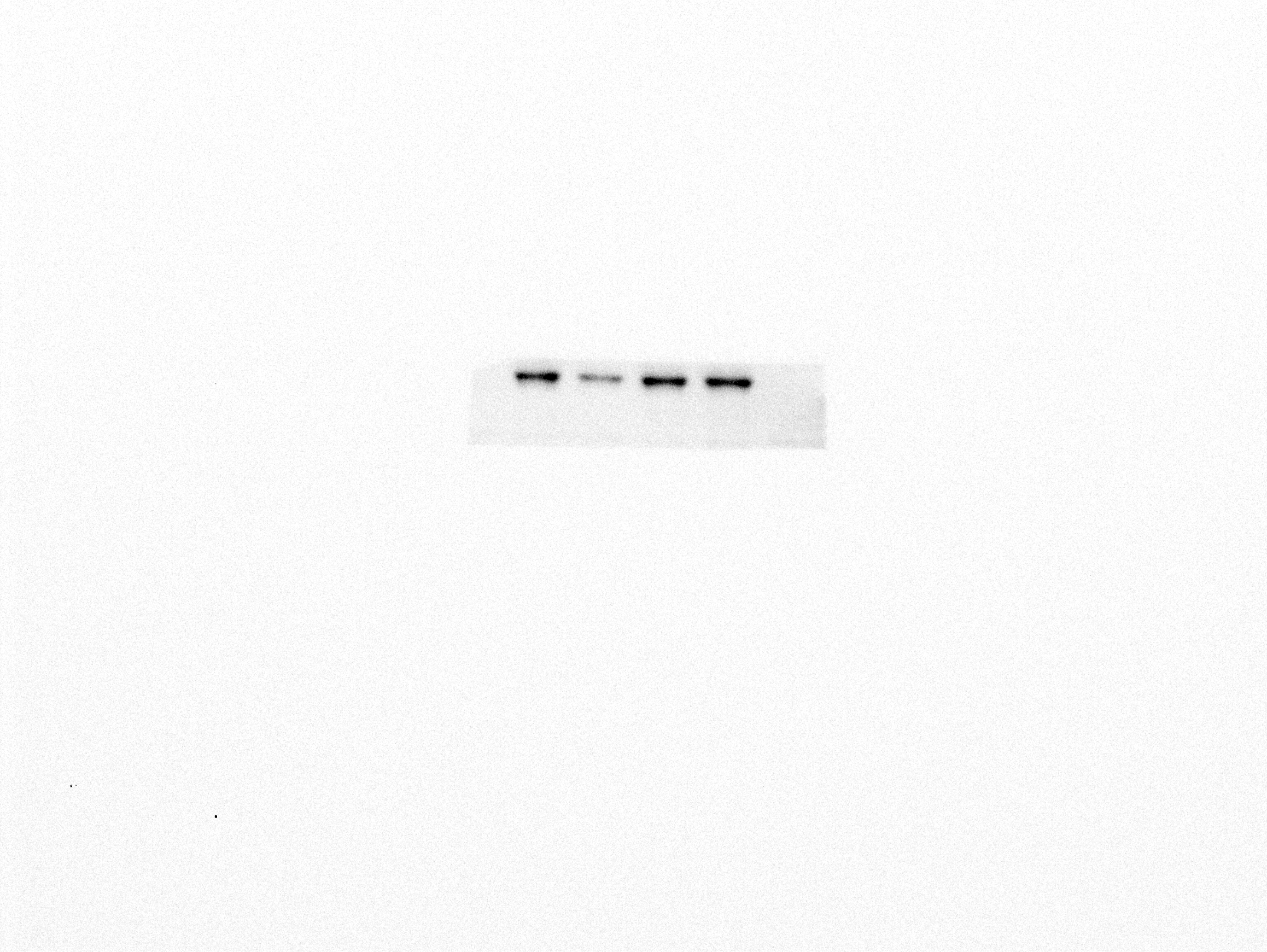

Supplement: Figure 5—source data 1. [file elife-98524-fig5-data1.zip › Fig 5-data1-v1/5C/left/Ac-k left.tif]

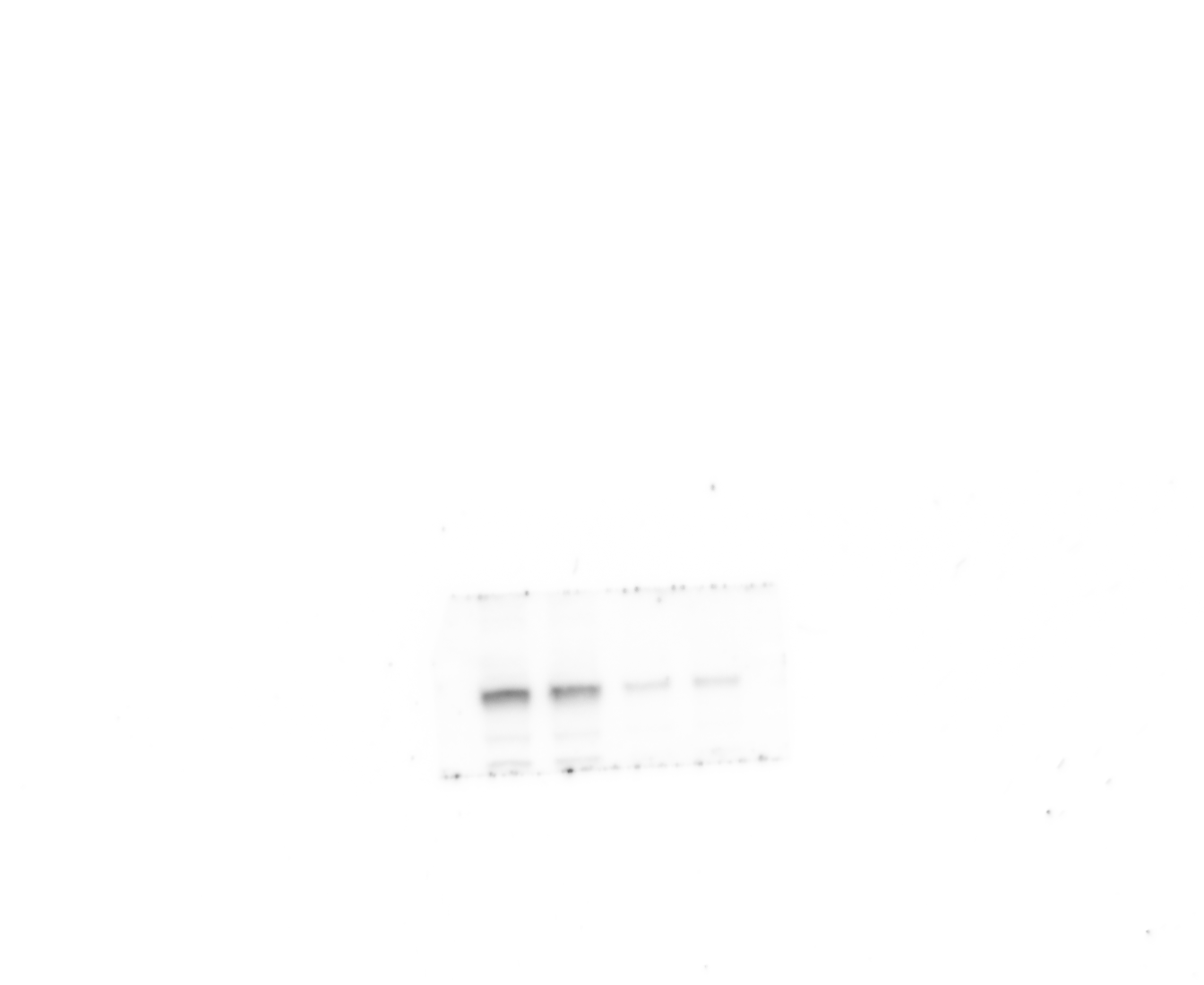

Supplement: Figure 5—source data 1. [file elife-98524-fig5-data1.zip › Fig 5-data1-v1/5C/left/SIRT4 left.tif]

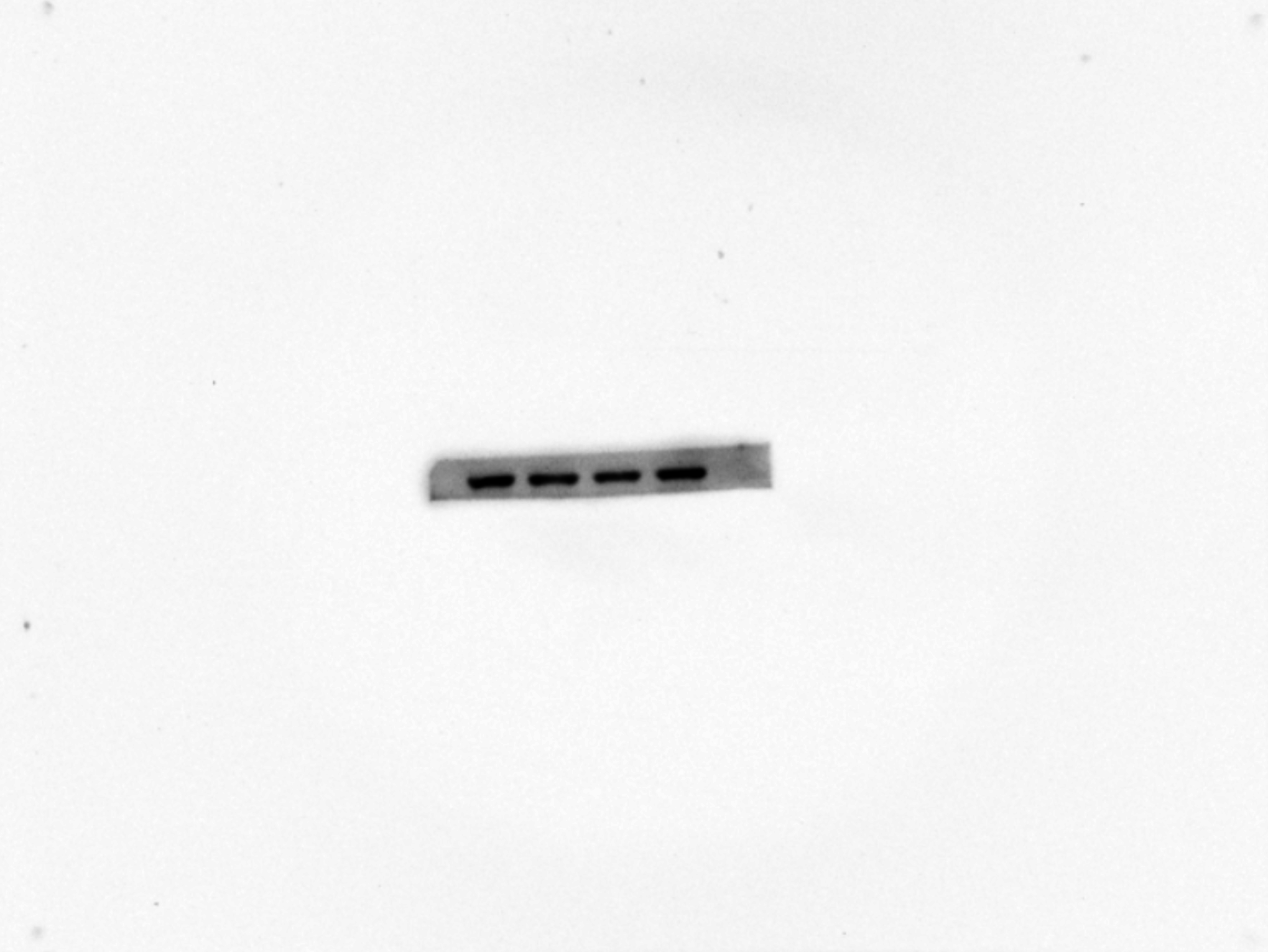

Supplement: Figure 5—source data 1. [file elife-98524-fig5-data1.zip › Fig 5-data1-v1/5C/left/Tubulin left.tif]

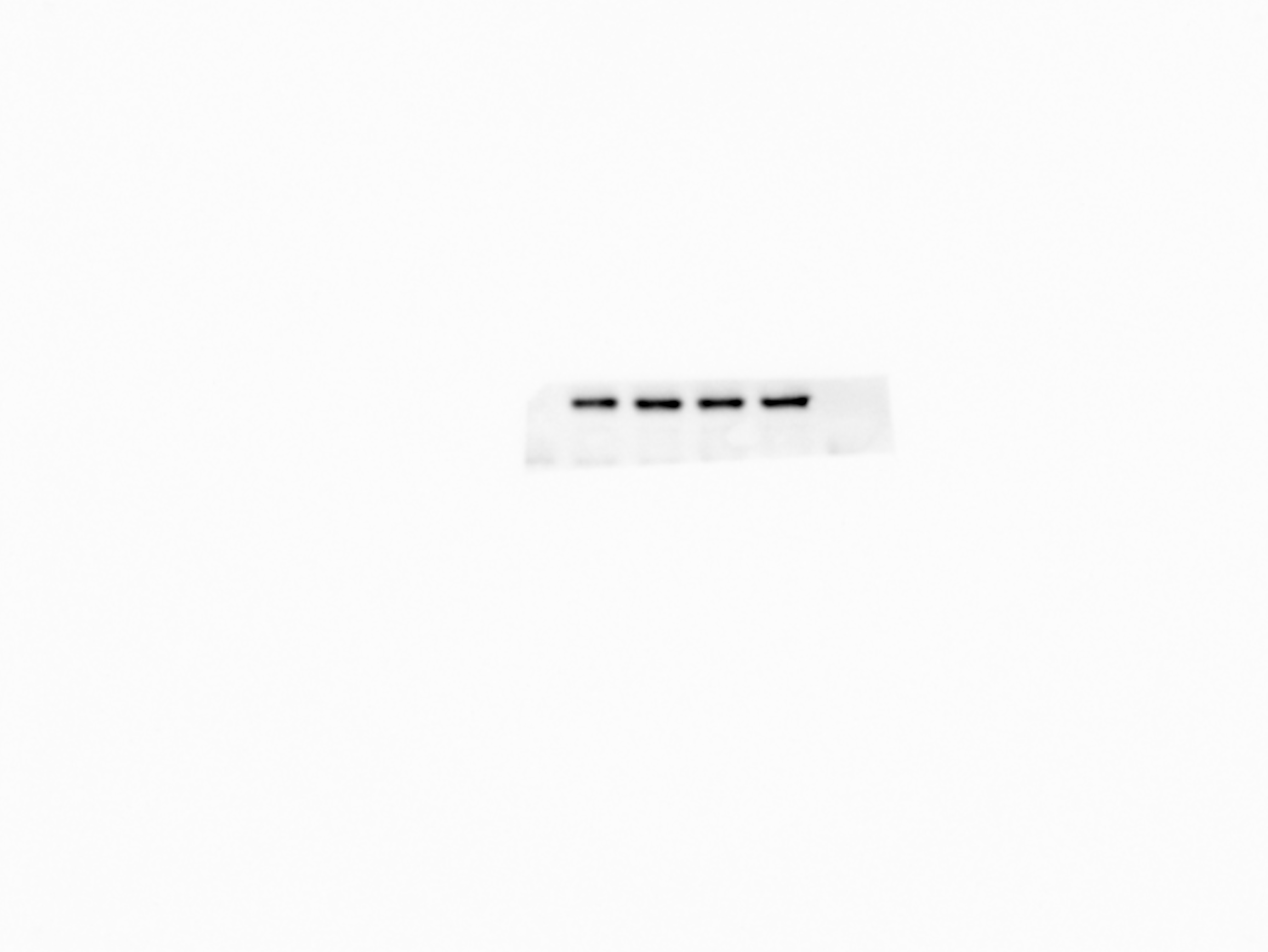

Supplement: Figure 5—source data 1. [file elife-98524-fig5-data1.zip › Fig 5-data1-v1/5C/left/U2AF2 bottom left.tif]

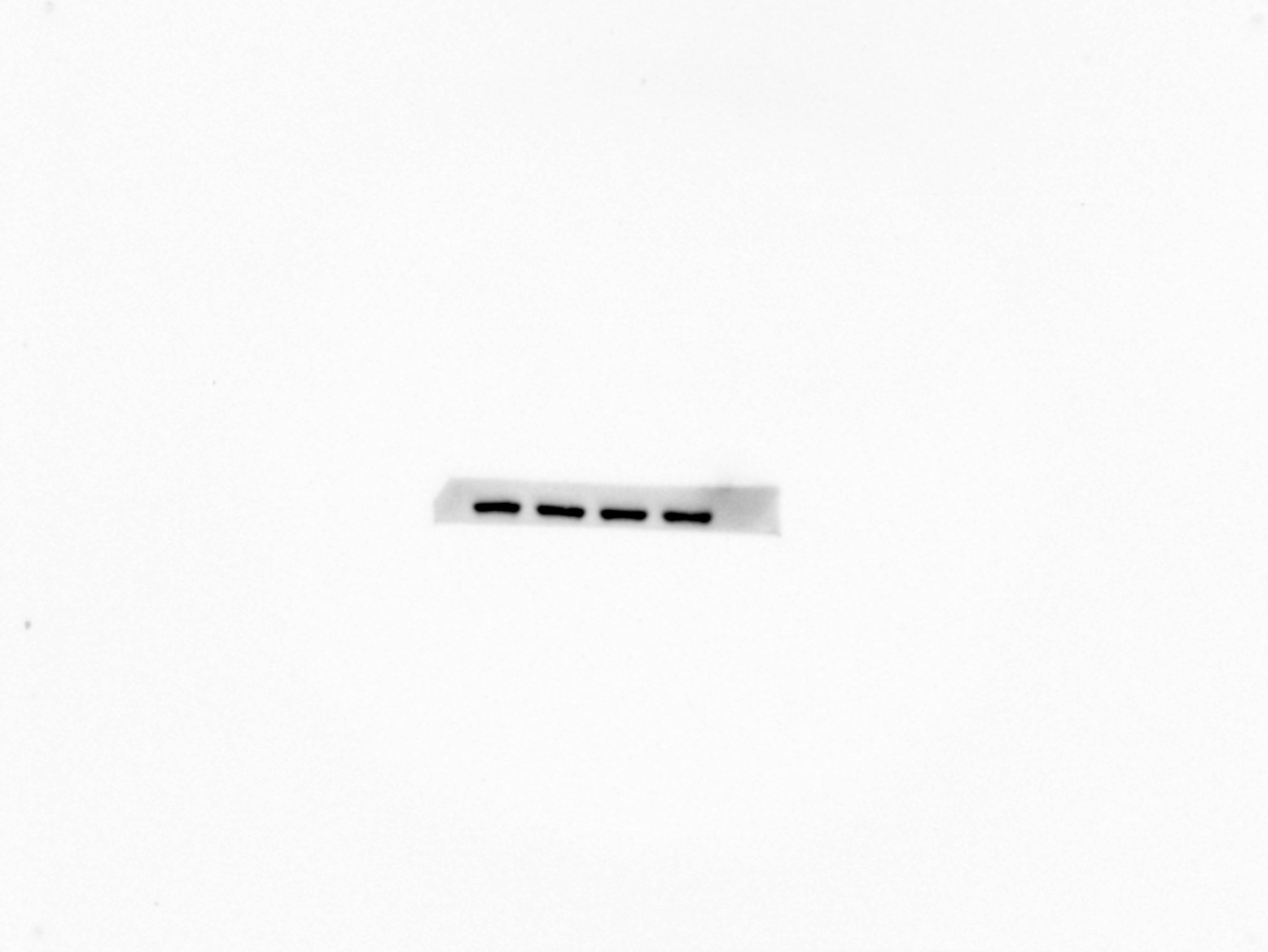

Supplement: Figure 5—source data 1. [file elife-98524-fig5-data1.zip › Fig 5-data1-v1/5C/left/U2AF2 upper left.tif]

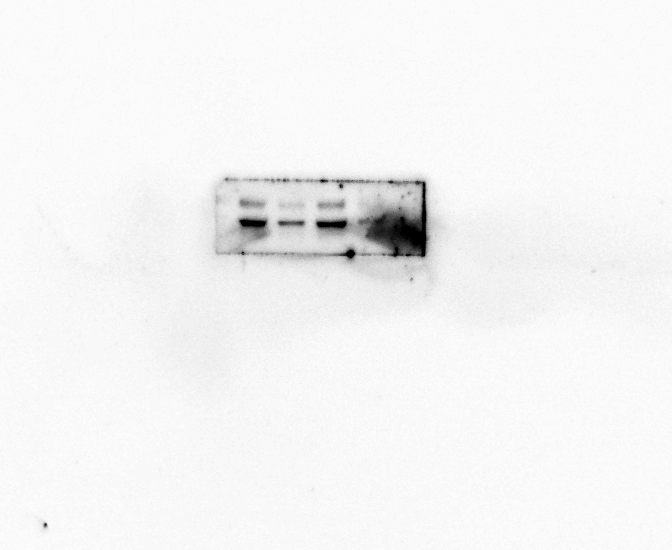

Supplement: Figure 5—source data 1. [file elife-98524-fig5-data1.zip › Fig 5-data1-v1/5C/right/Ac-k right.tif]

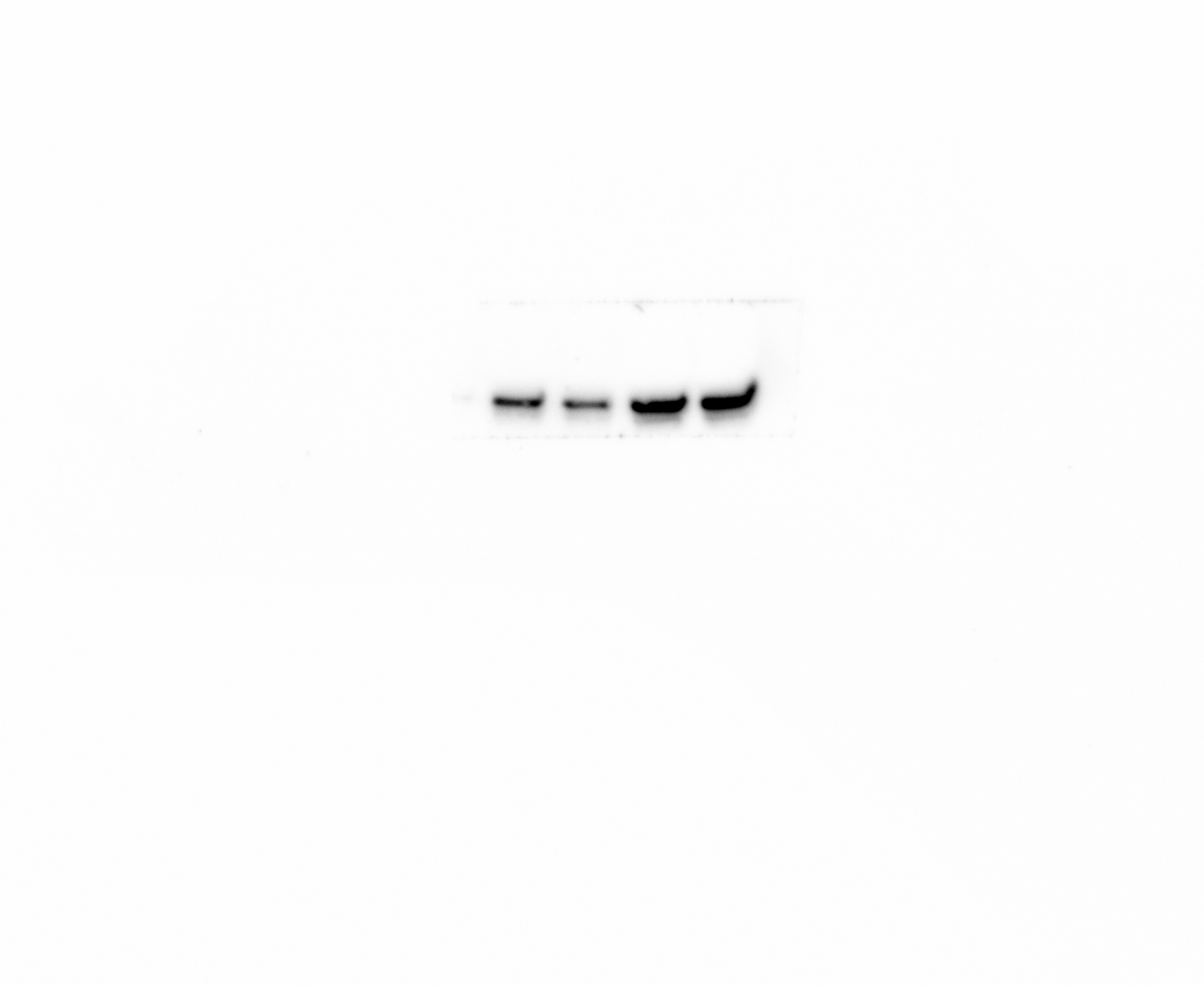

Supplement: Figure 5—source data 1. [file elife-98524-fig5-data1.zip › Fig 5-data1-v1/5C/right/SIRT4 right.tif]

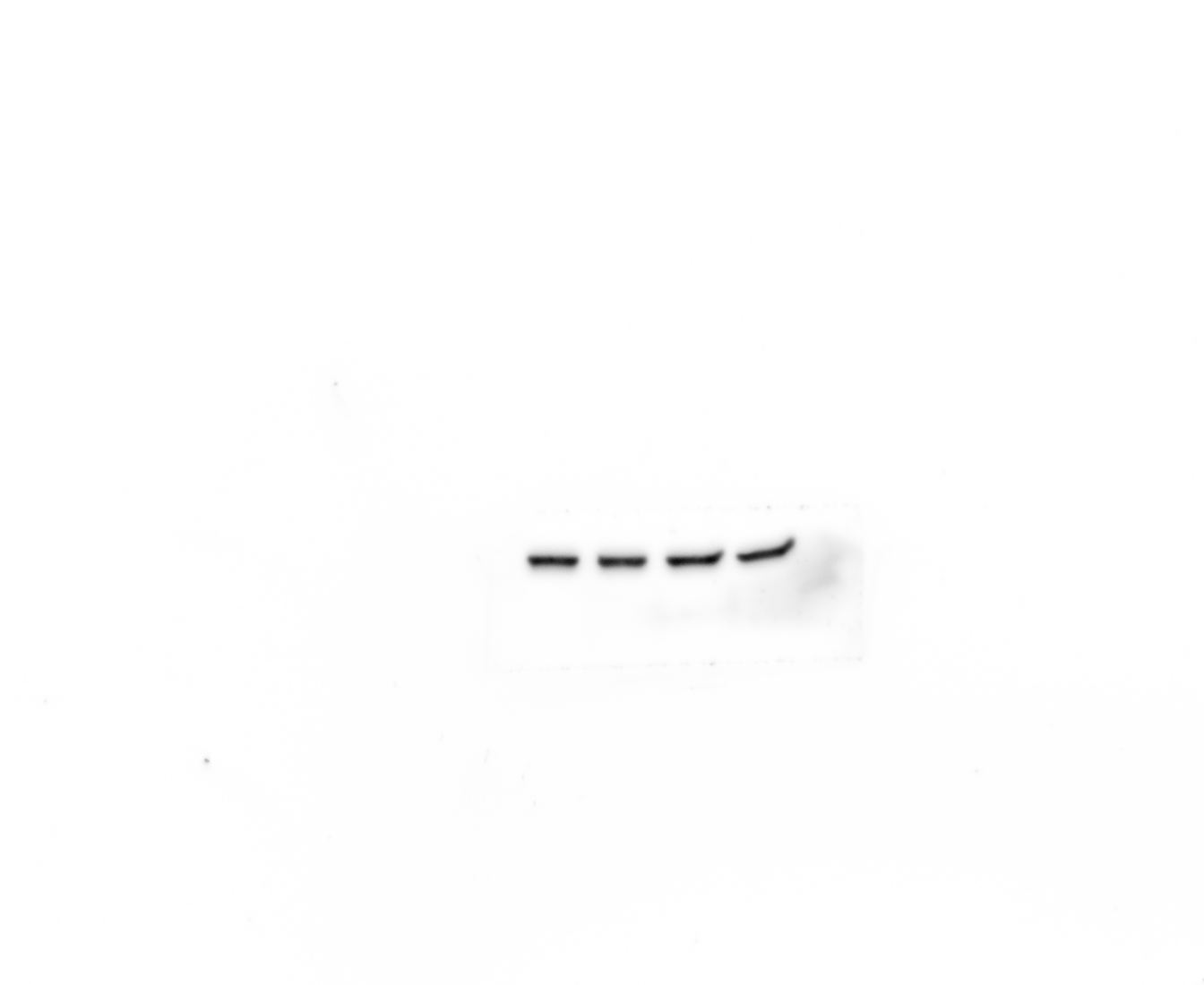

Supplement: Figure 5—source data 1. [file elife-98524-fig5-data1.zip › Fig 5-data1-v1/5C/right/Tubulin right.tif]

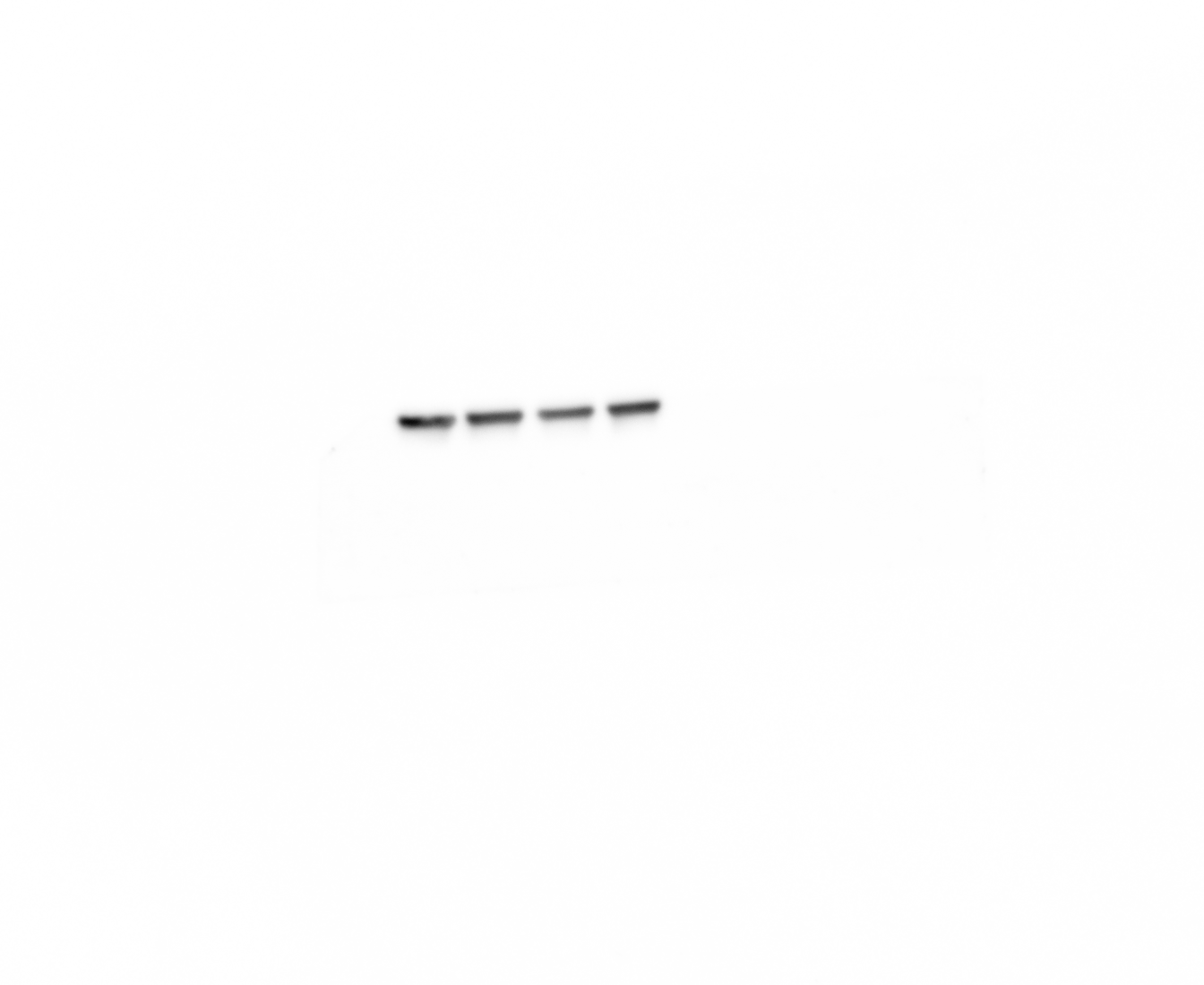

Supplement: Figure 5—source data 1. [file elife-98524-fig5-data1.zip › Fig 5-data1-v1/5C/right/U2AF2 bottom right.tif]

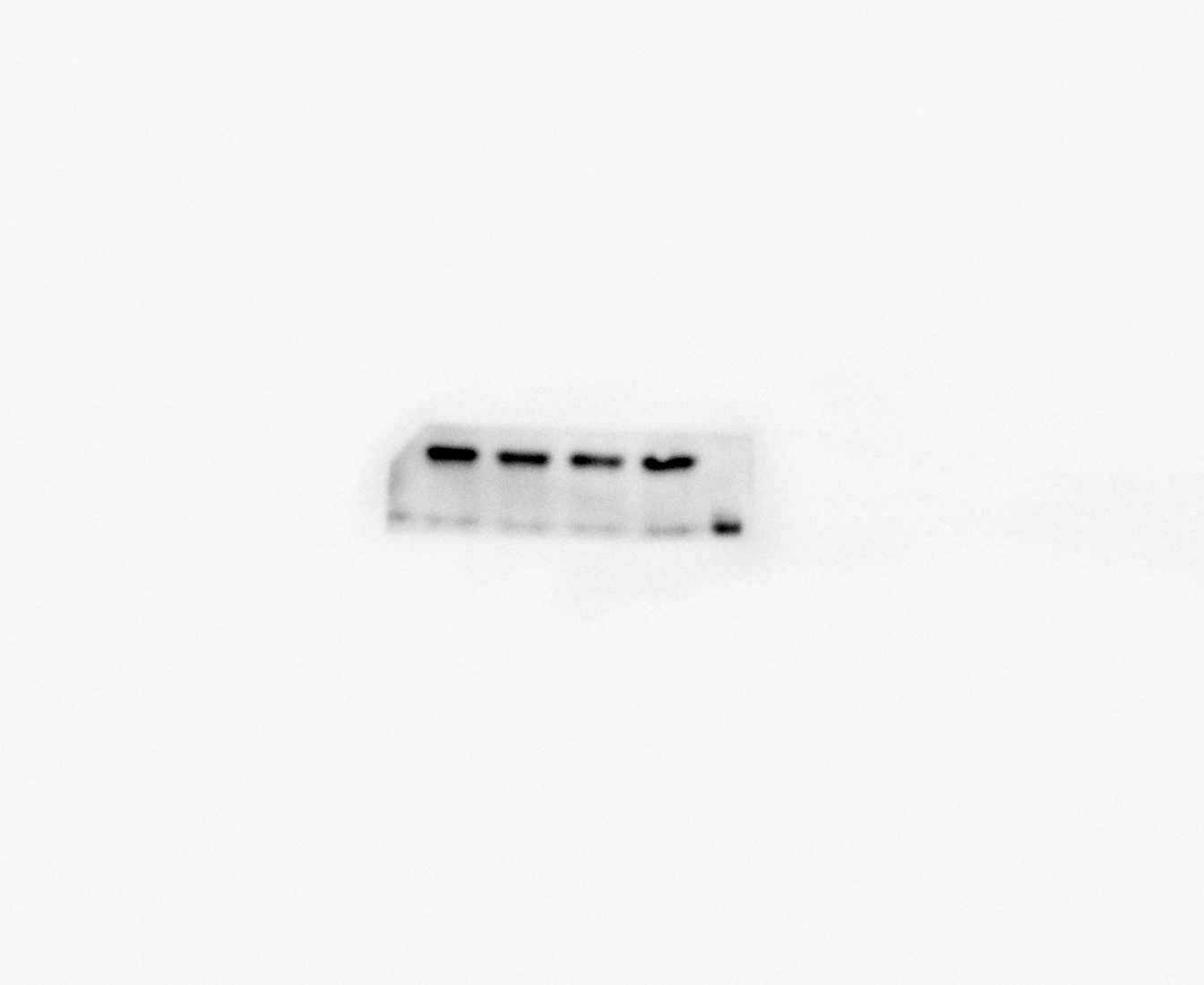

Supplement: Figure 5—source data 1. [file elife-98524-fig5-data1.zip › Fig 5-data1-v1/5C/right/U2AF2 upper right.tif]

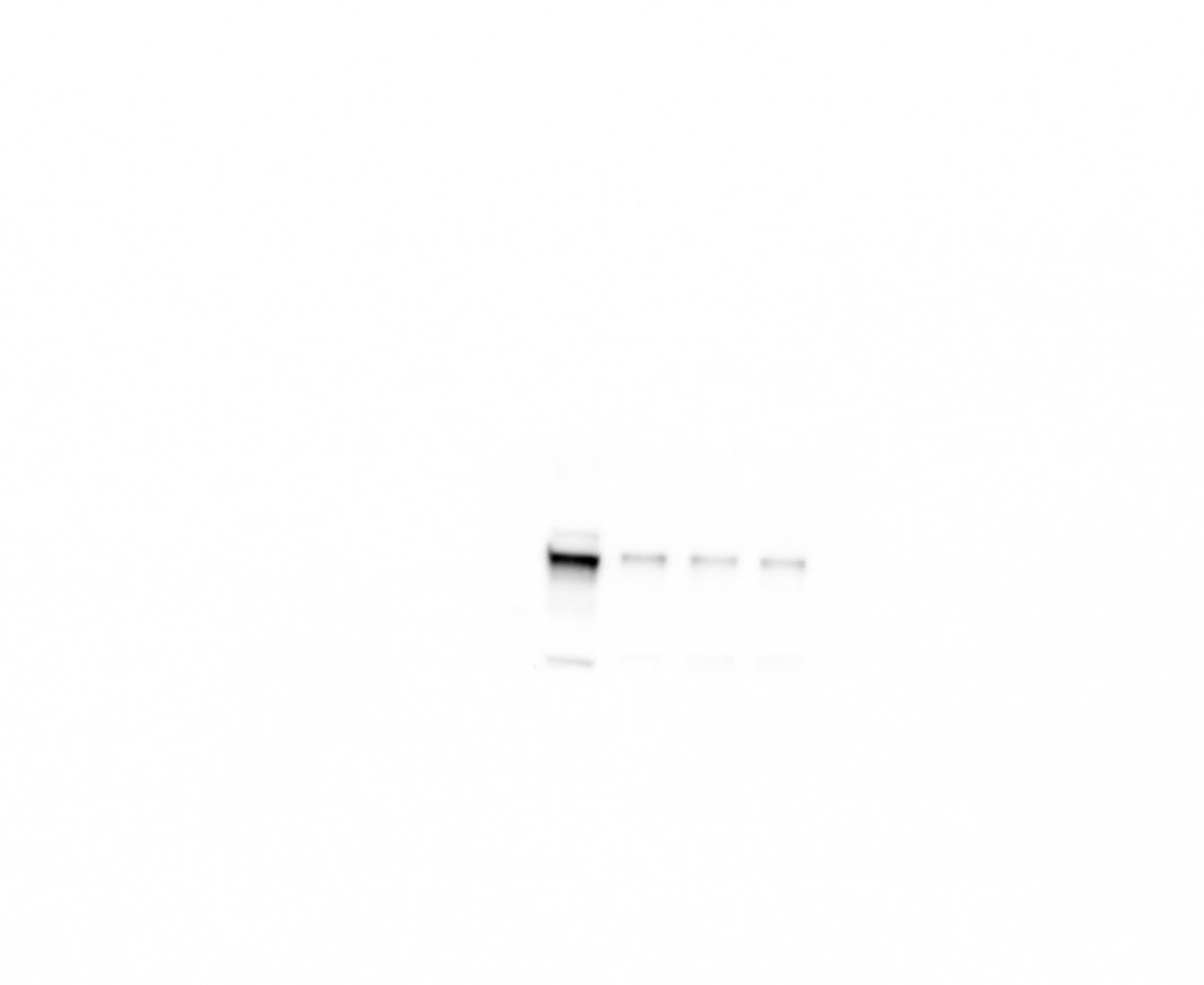

Supplement: Figure 5—source data 1. [file elife-98524-fig5-data1.zip › Fig 5-data1-v1/5D/left/Ac-k.tif]

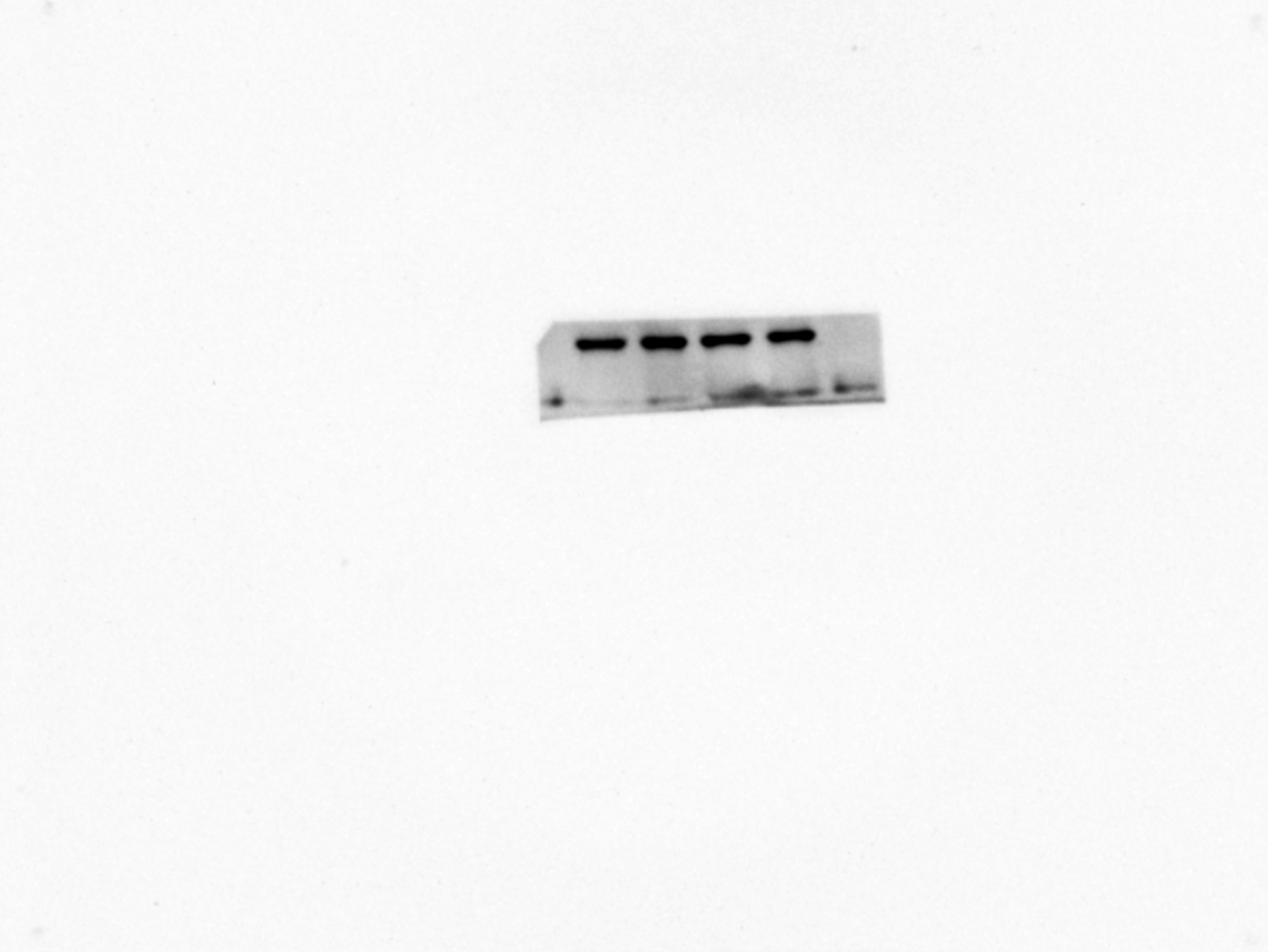

Supplement: Figure 5—source data 1. [file elife-98524-fig5-data1.zip › Fig 5-data1-v1/5D/left/Flag bottom left.tif]

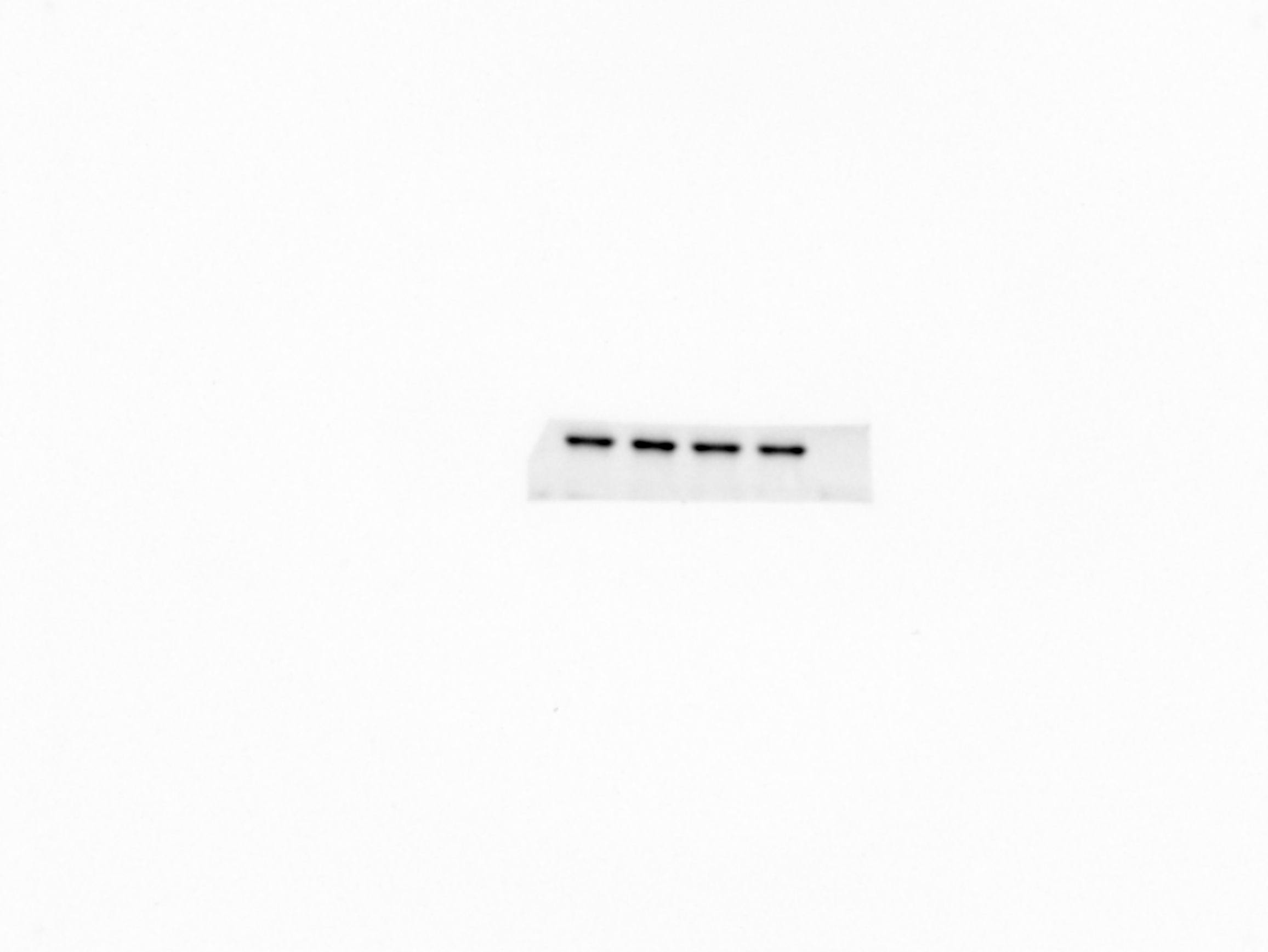

Supplement: Figure 5—source data 1. [file elife-98524-fig5-data1.zip › Fig 5-data1-v1/5D/left/Flag upper left.tif]

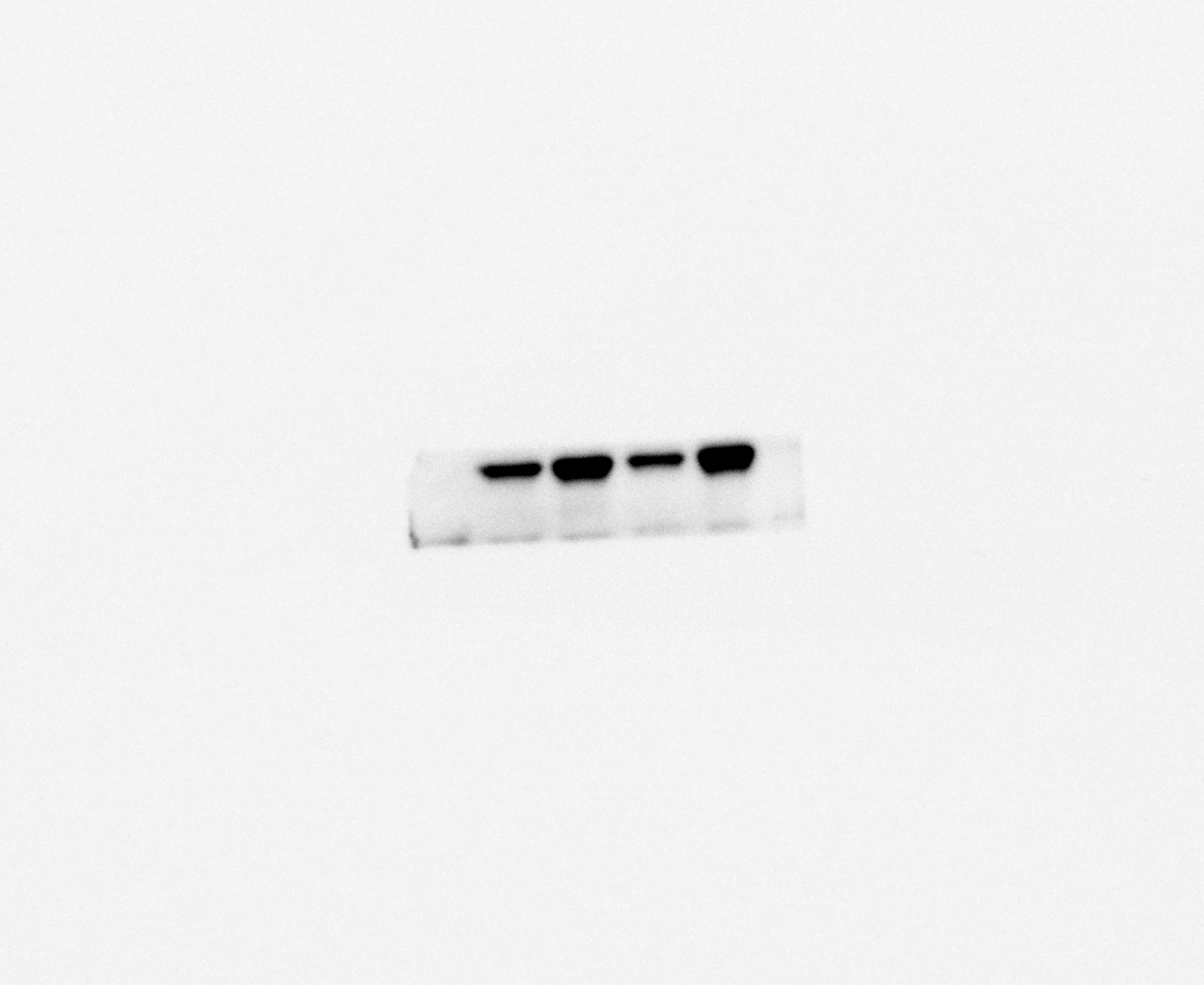

Supplement: Figure 5—source data 1. [file elife-98524-fig5-data1.zip › Fig 5-data1-v1/5D/left/SIRT4.tif]

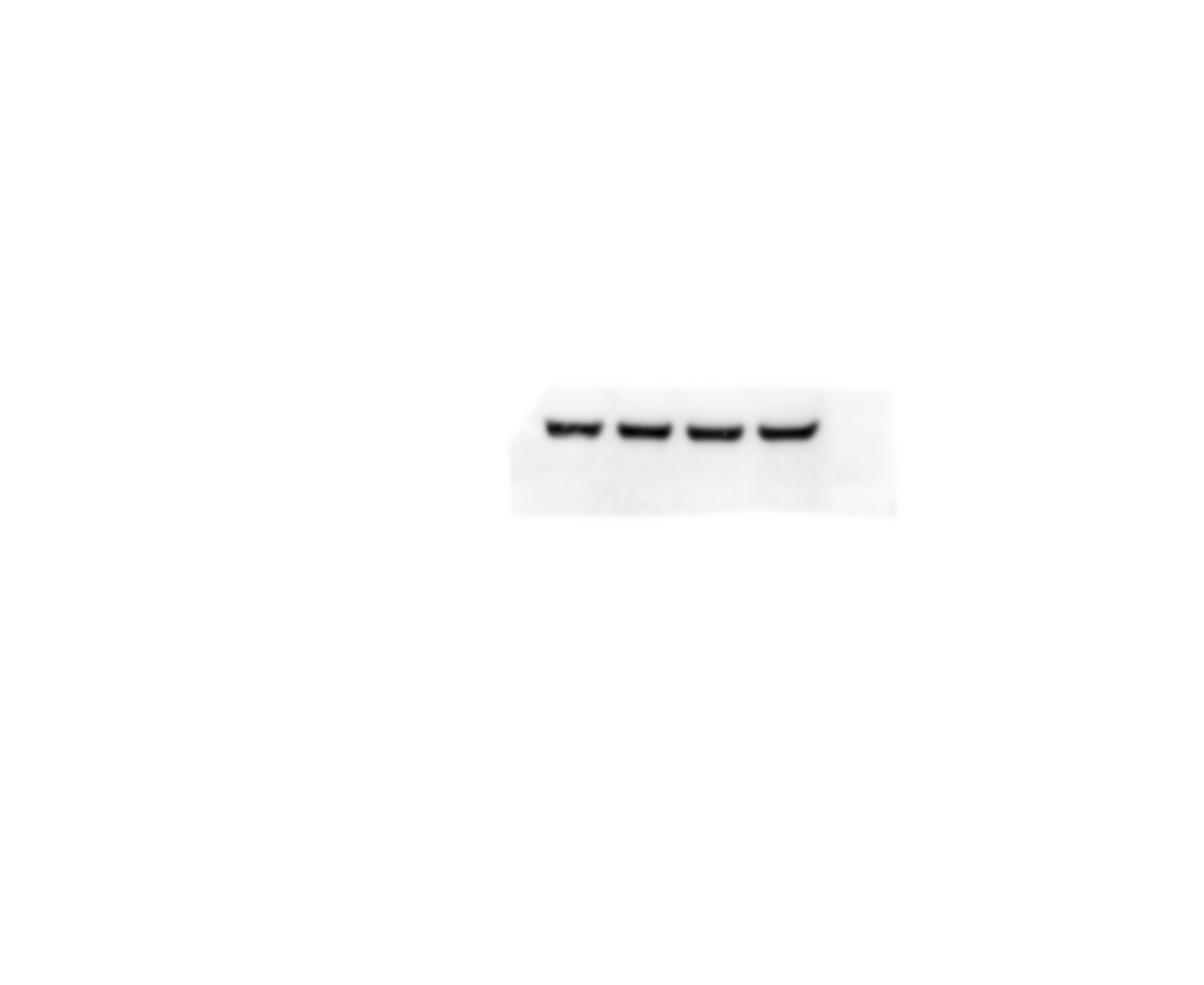

Supplement: Figure 5—source data 1. [file elife-98524-fig5-data1.zip › Fig 5-data1-v1/5D/left/Tubulin.tif]

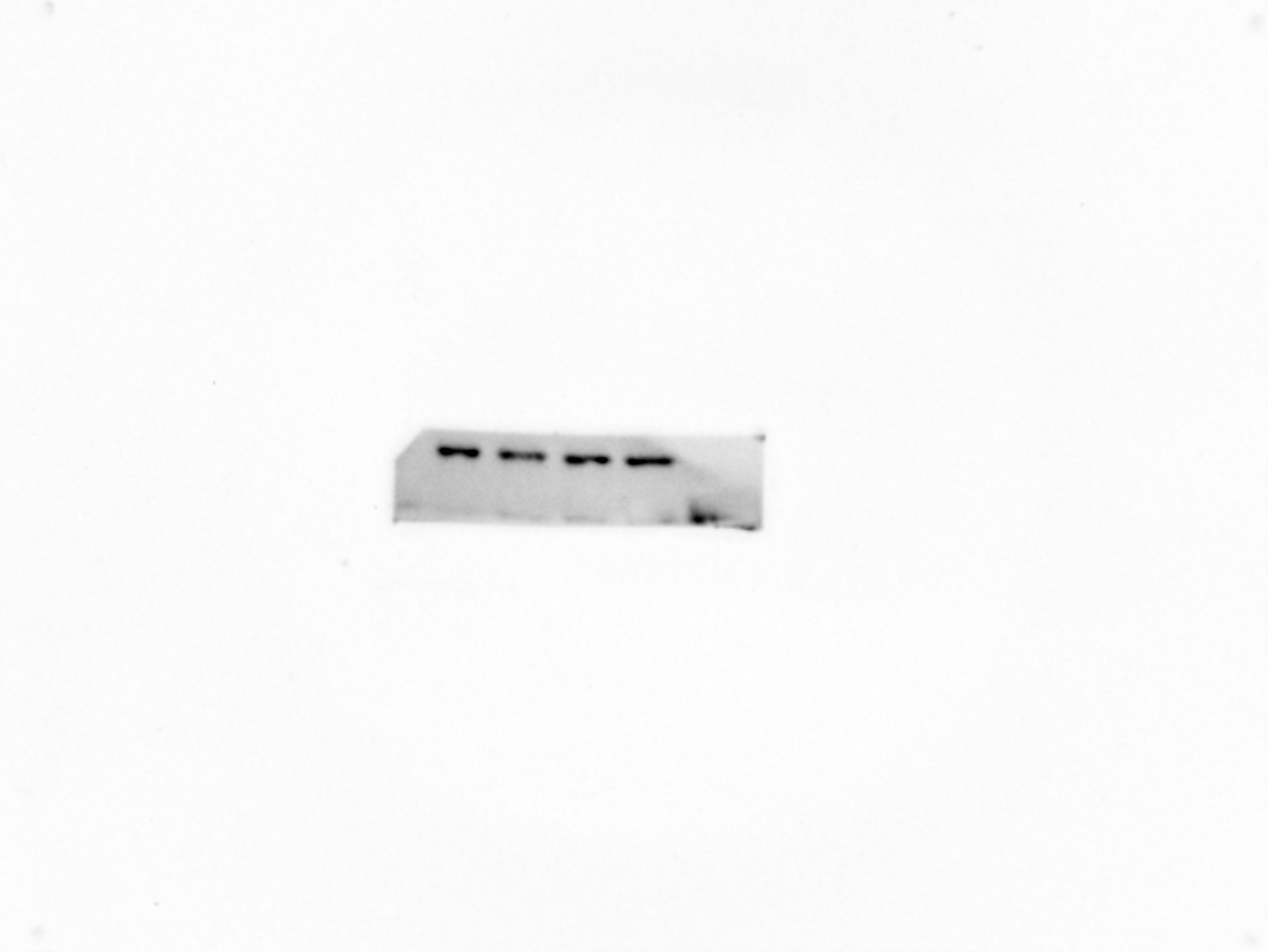

Supplement: Figure 5—source data 1. [file elife-98524-fig5-data1.zip › Fig 5-data1-v1/5D/right/Flag right.tif]

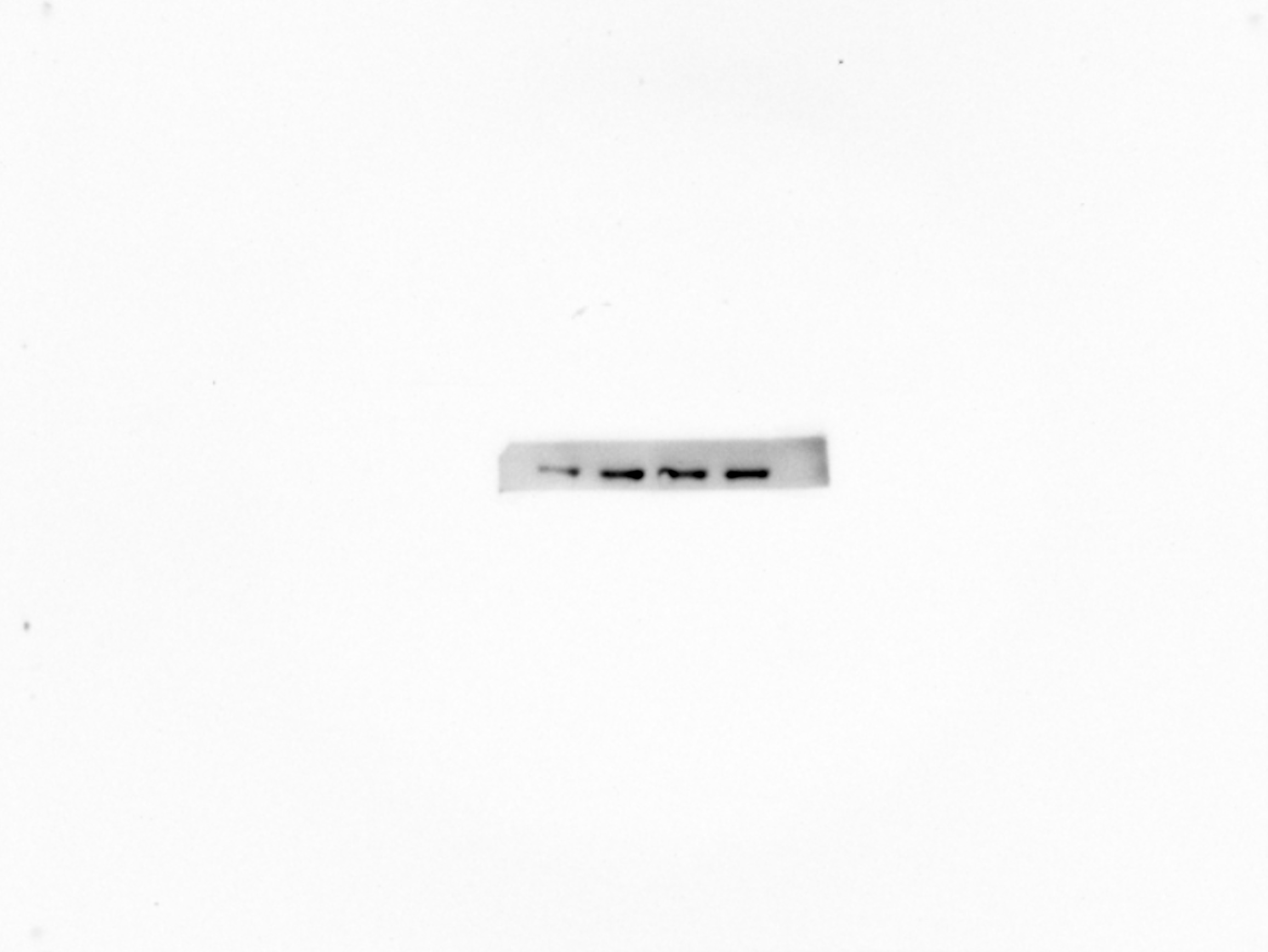

Supplement: Figure 5—source data 1. [file elife-98524-fig5-data1.zip › Fig 5-data1-v1/5D/right/SF3B1.tif]

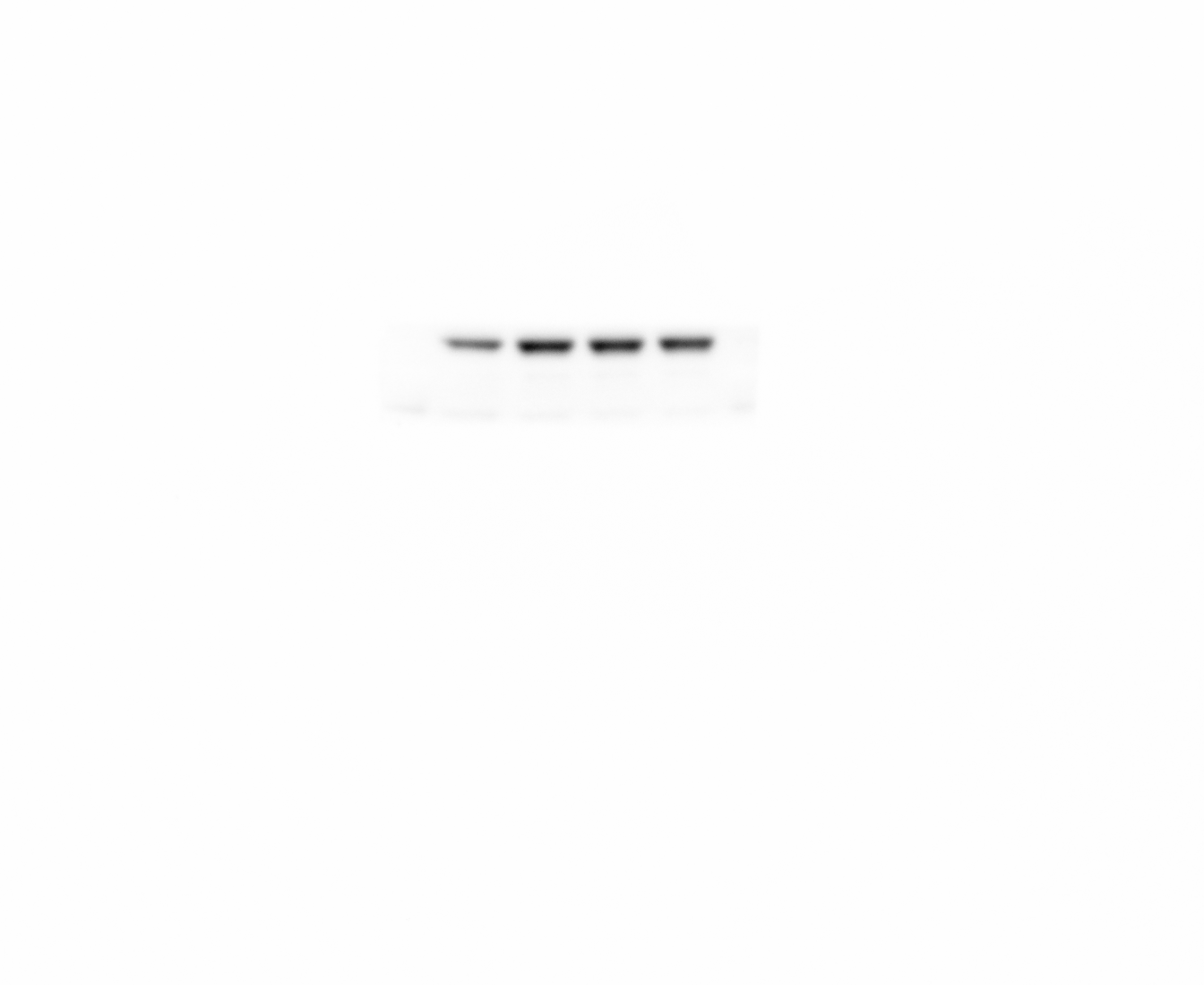

Supplement: Figure 5—source data 1. [file elife-98524-fig5-data1.zip › Fig 5-data1-v1/5D/right/SF3B2.tif]

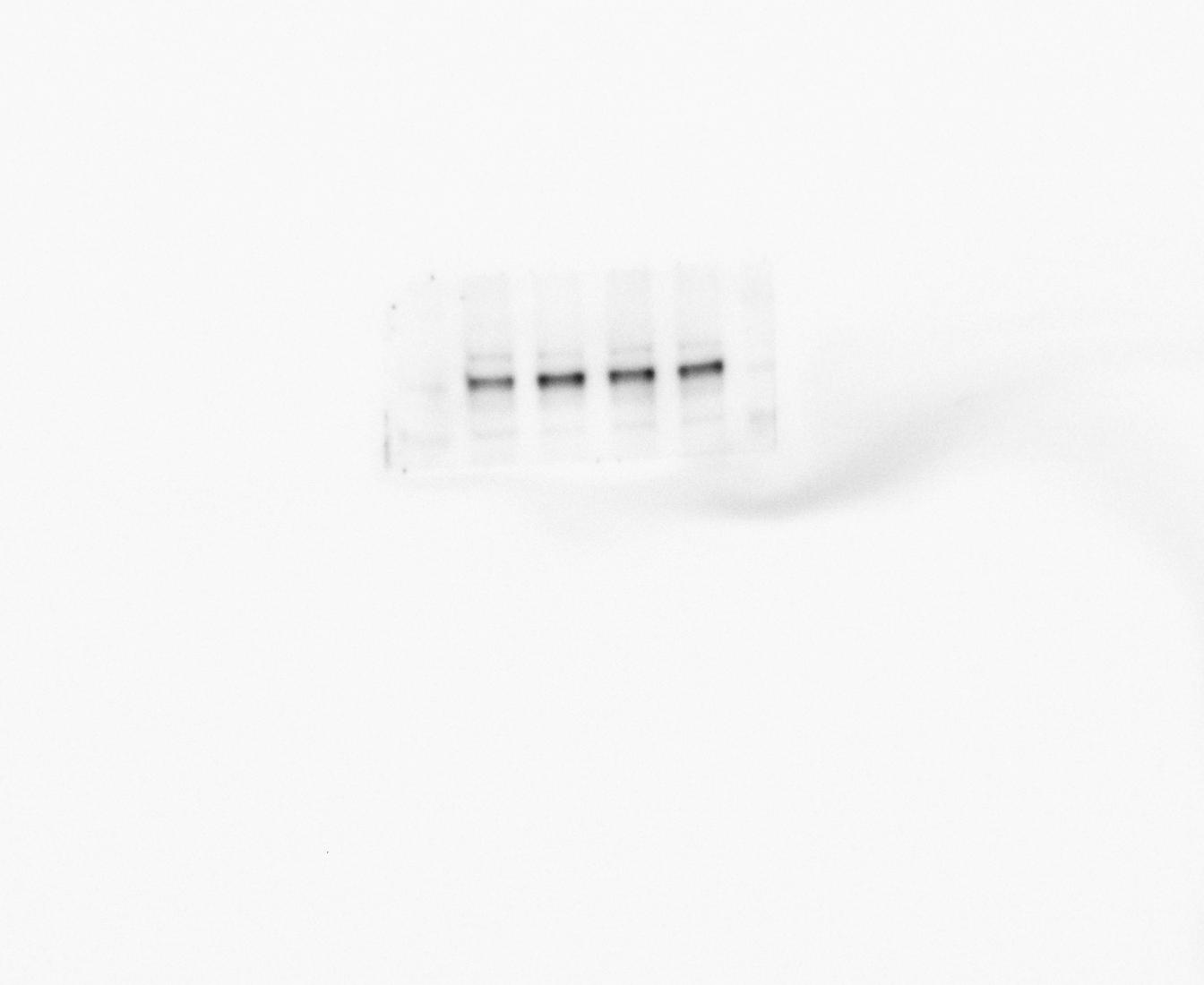

Supplement: Figure 5—source data 1. [file elife-98524-fig5-data1.zip › Fig 5-data1-v1/5D/right/SF3B3.tif]

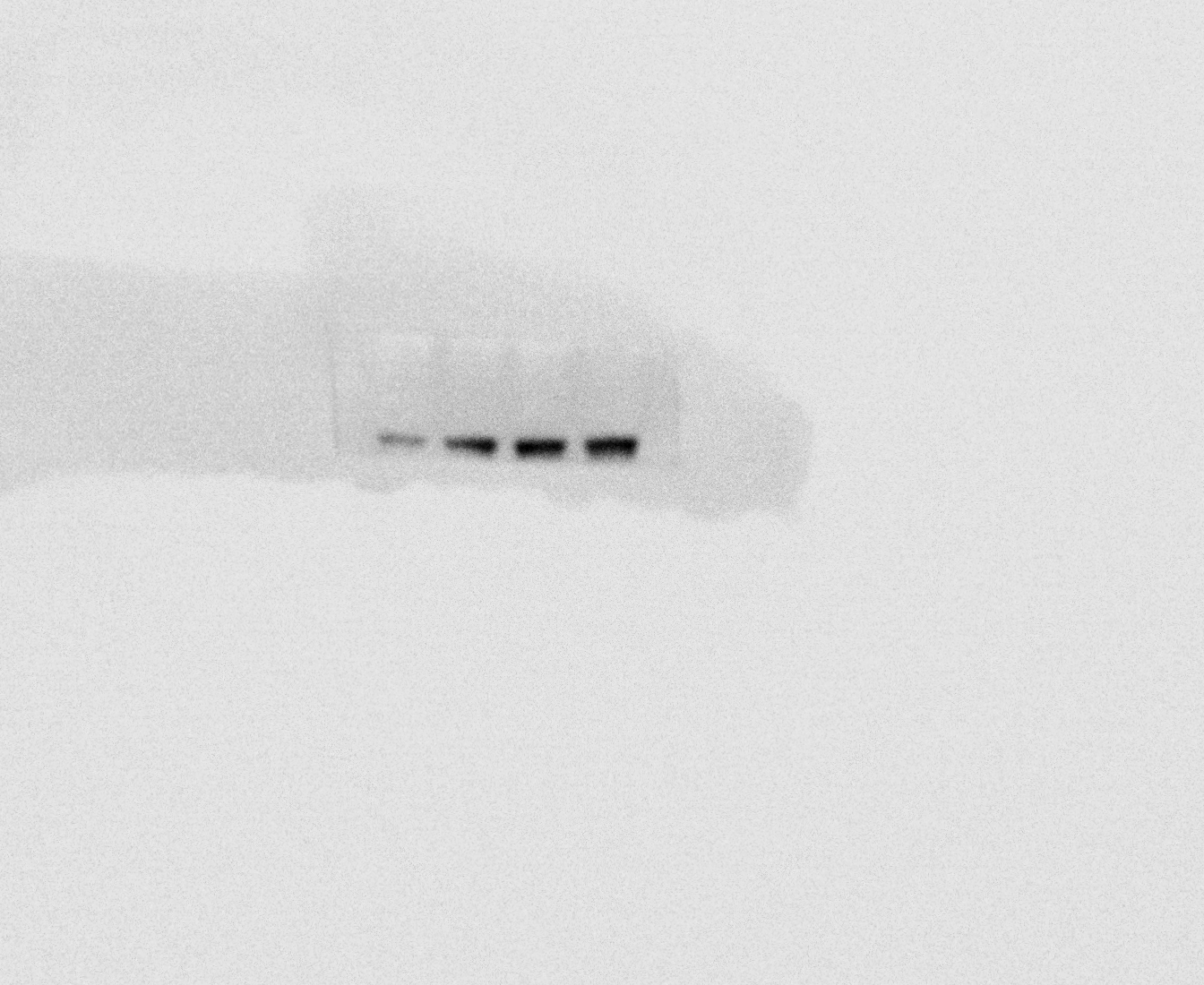

Supplement: Figure 5—source data 1. [file elife-98524-fig5-data1.zip › Fig 5-data1-v1/5D/right/U2AF1.tif]

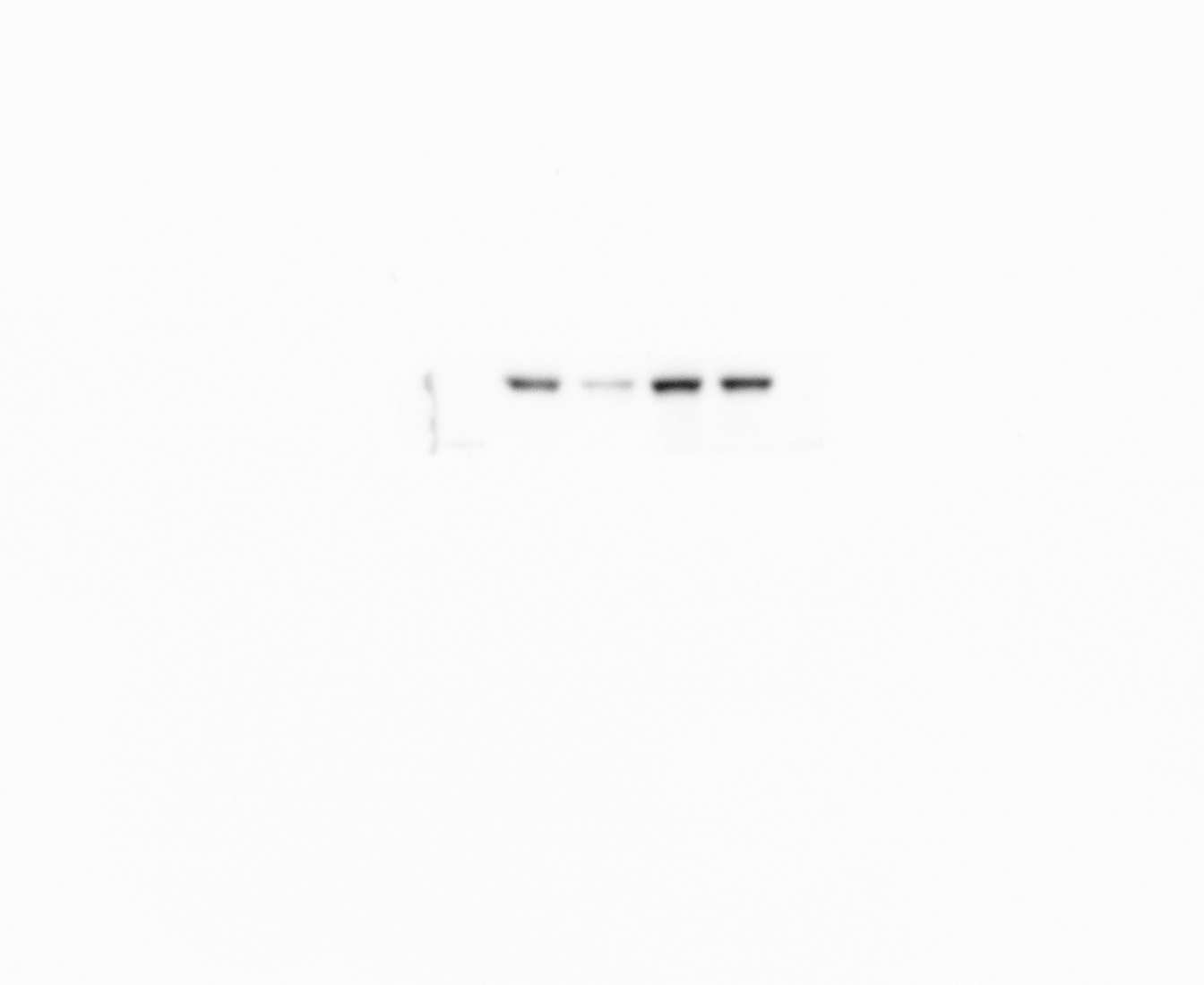

Supplement: Figure 5—source data 1. [file elife-98524-fig5-data1.zip › Fig 5-data1-v1/5E/left/Ac-K.tif]

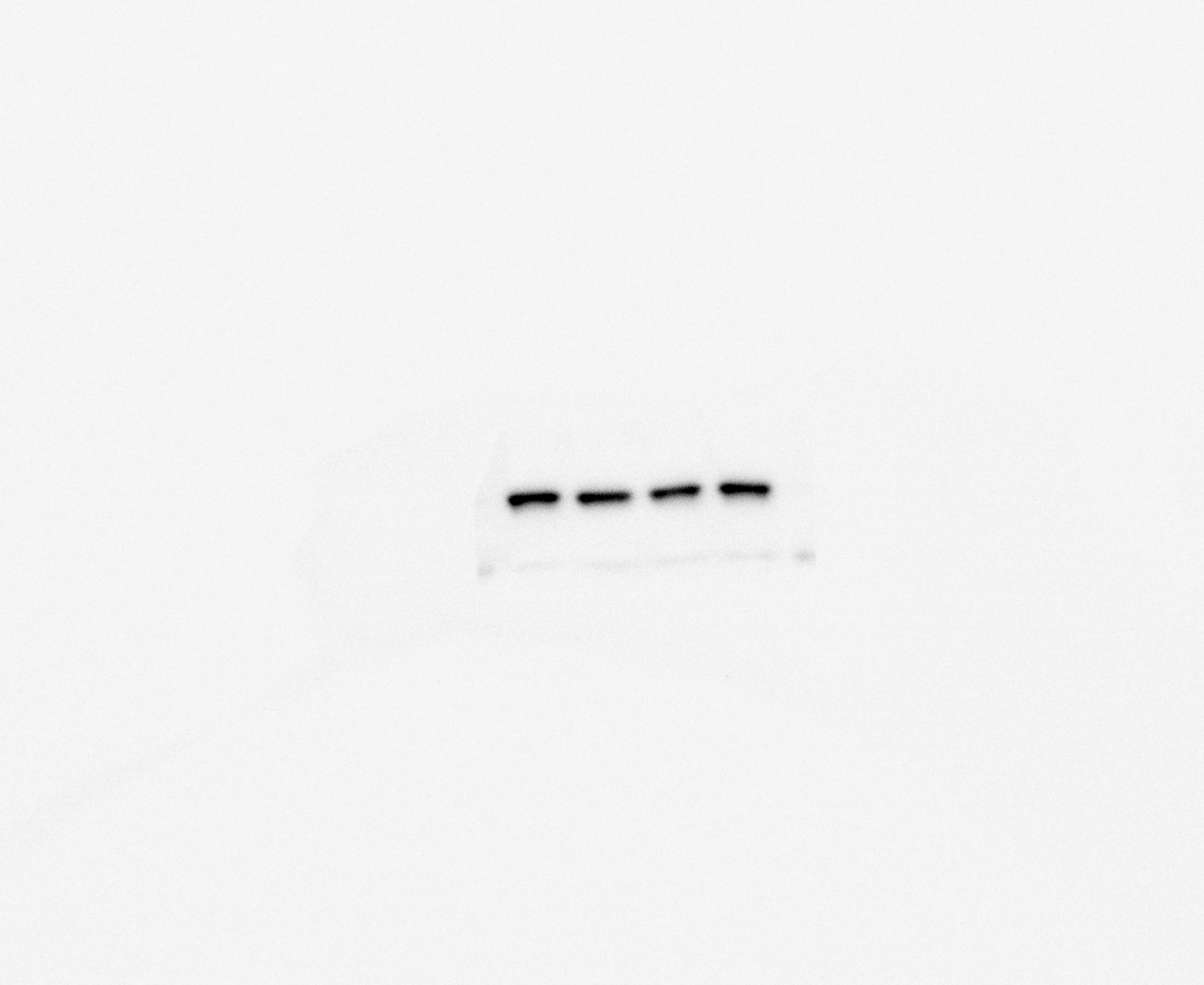

Supplement: Figure 5—source data 1. [file elife-98524-fig5-data1.zip › Fig 5-data1-v1/5E/left/Flag bottom left.tif]

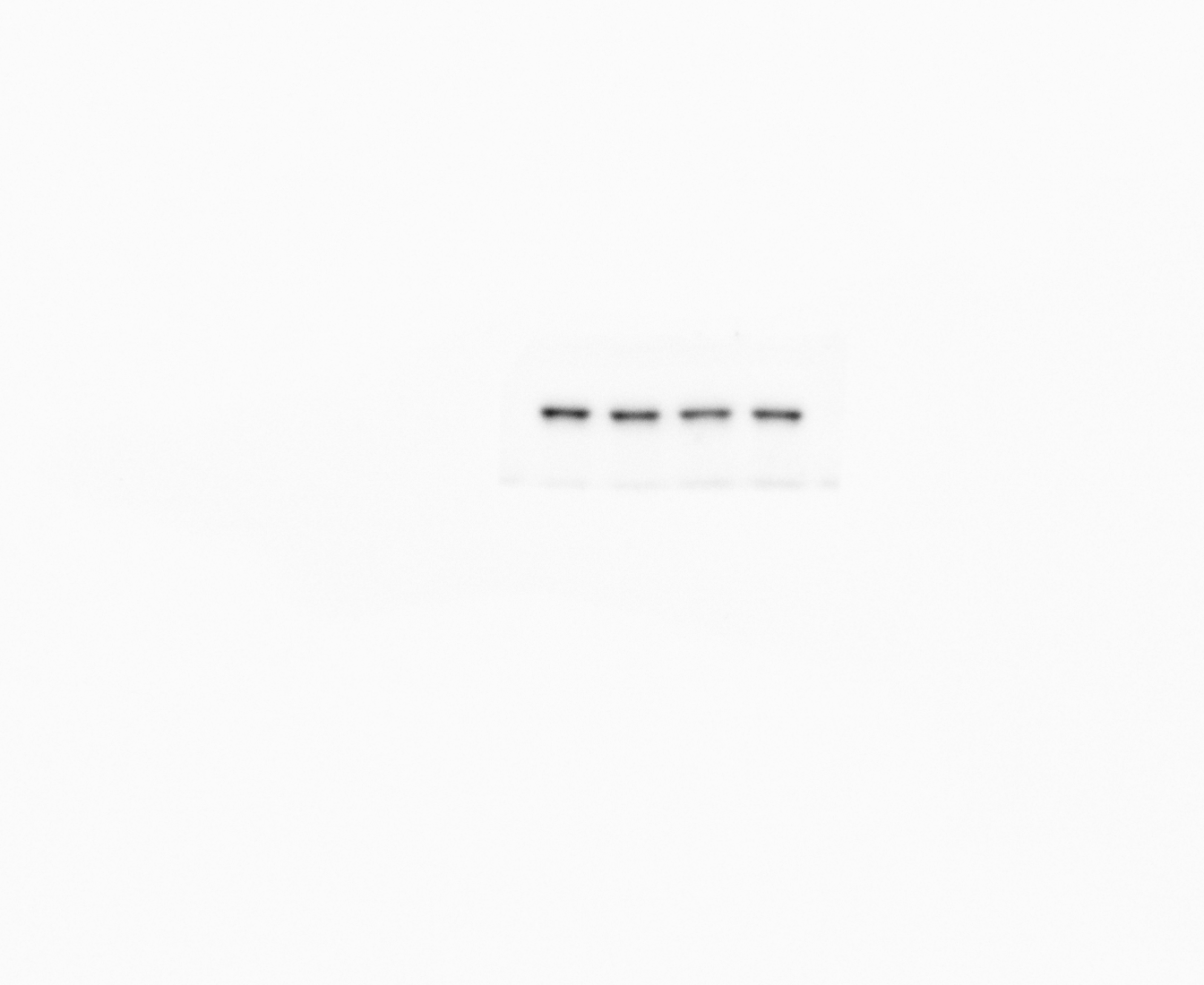

Supplement: Figure 5—source data 1. [file elife-98524-fig5-data1.zip › Fig 5-data1-v1/5E/left/Flag upper left.tif]

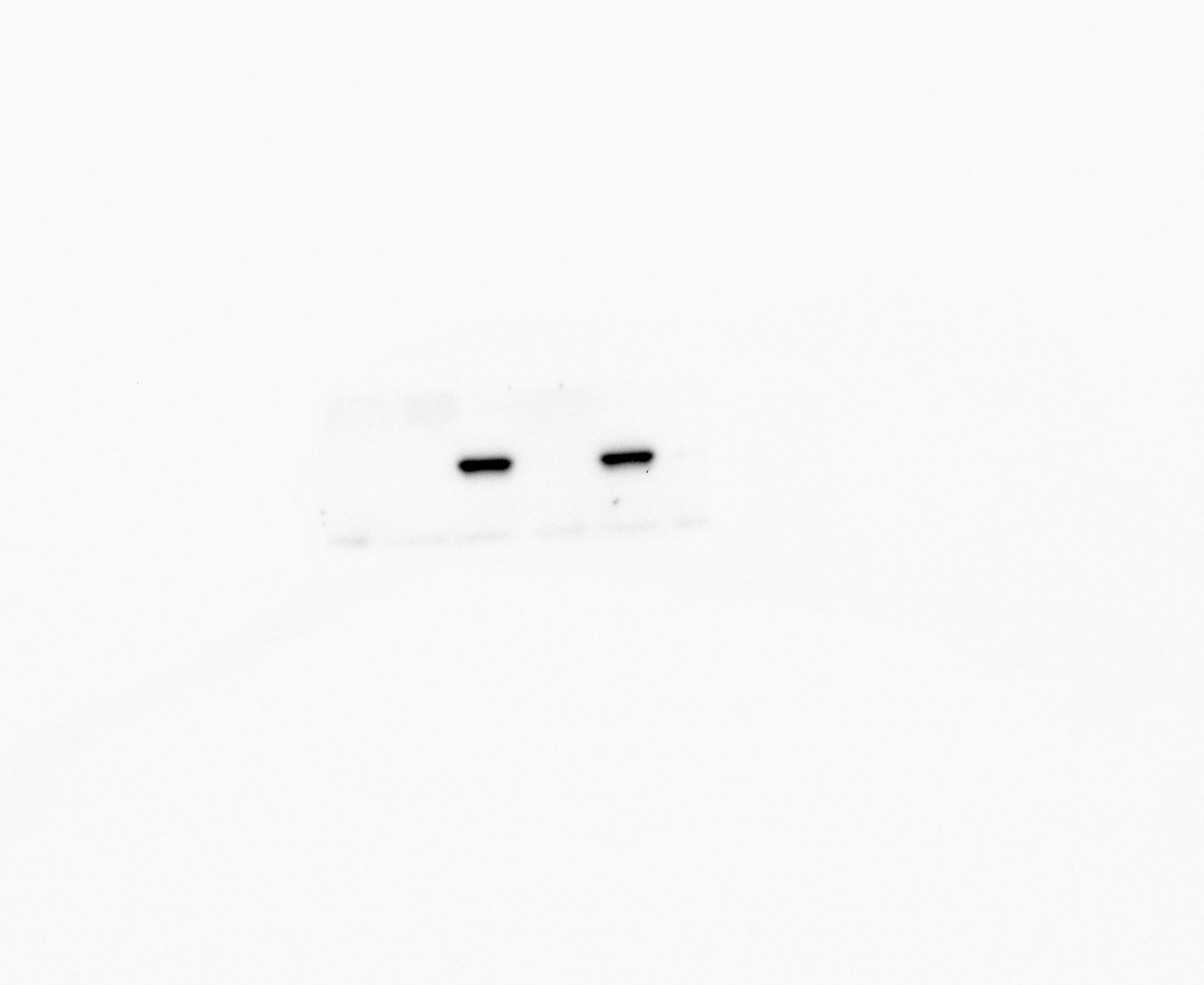

Supplement: Figure 5—source data 1. [file elife-98524-fig5-data1.zip › Fig 5-data1-v1/5E/left/HA.tif]

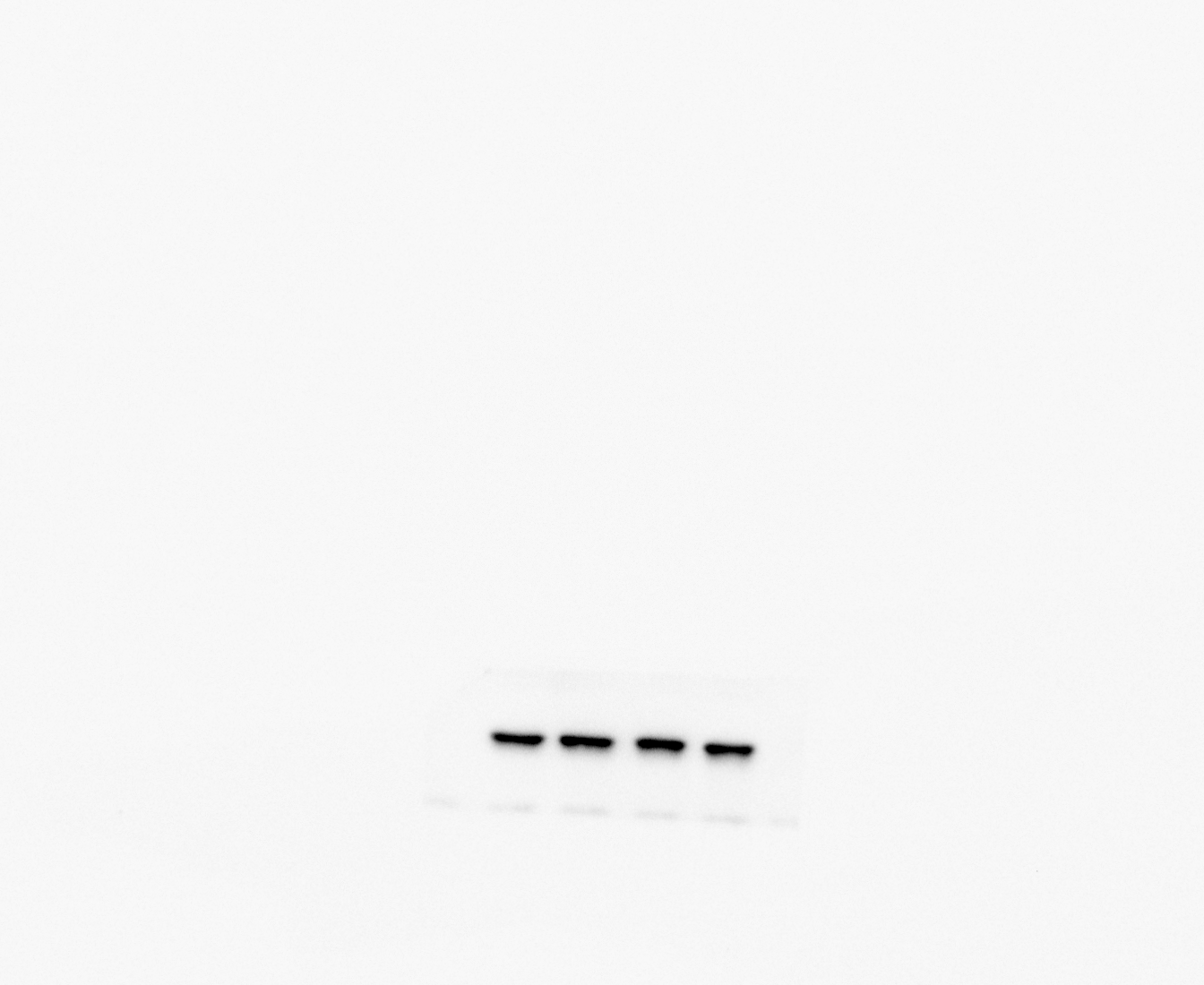

Supplement: Figure 5—source data 1. [file elife-98524-fig5-data1.zip › Fig 5-data1-v1/5E/left/Tubulin.tif]

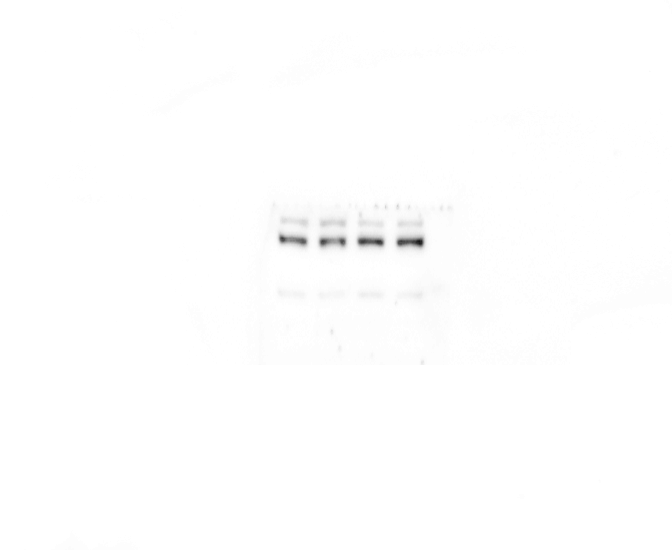

Supplement: Figure 5—source data 1. [file elife-98524-fig5-data1.zip › Fig 5-data1-v1/5E/right/Flag right.tif]

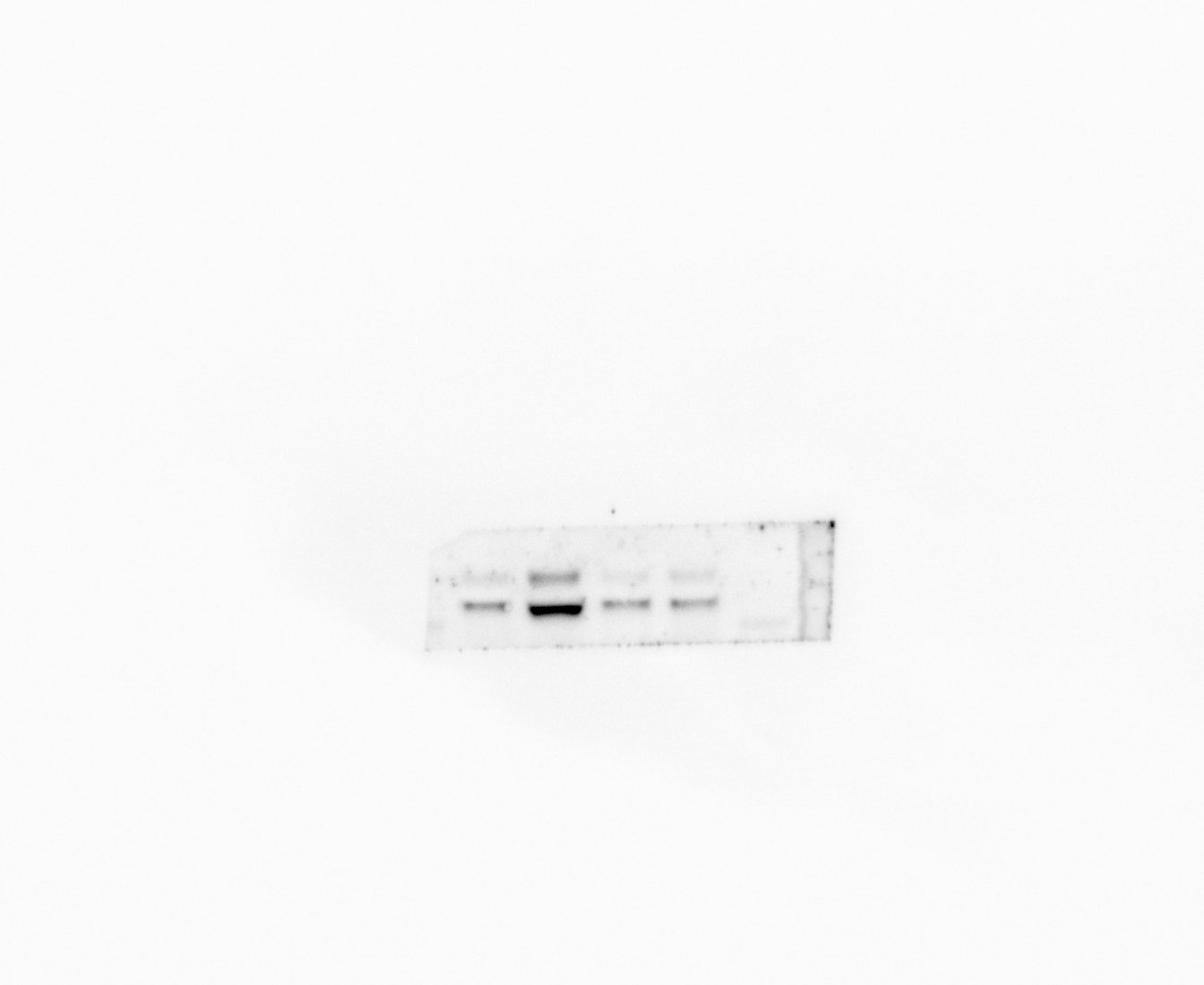

Supplement: Figure 5—source data 1. [file elife-98524-fig5-data1.zip › Fig 5-data1-v1/5E/right/SF3B1.tif]

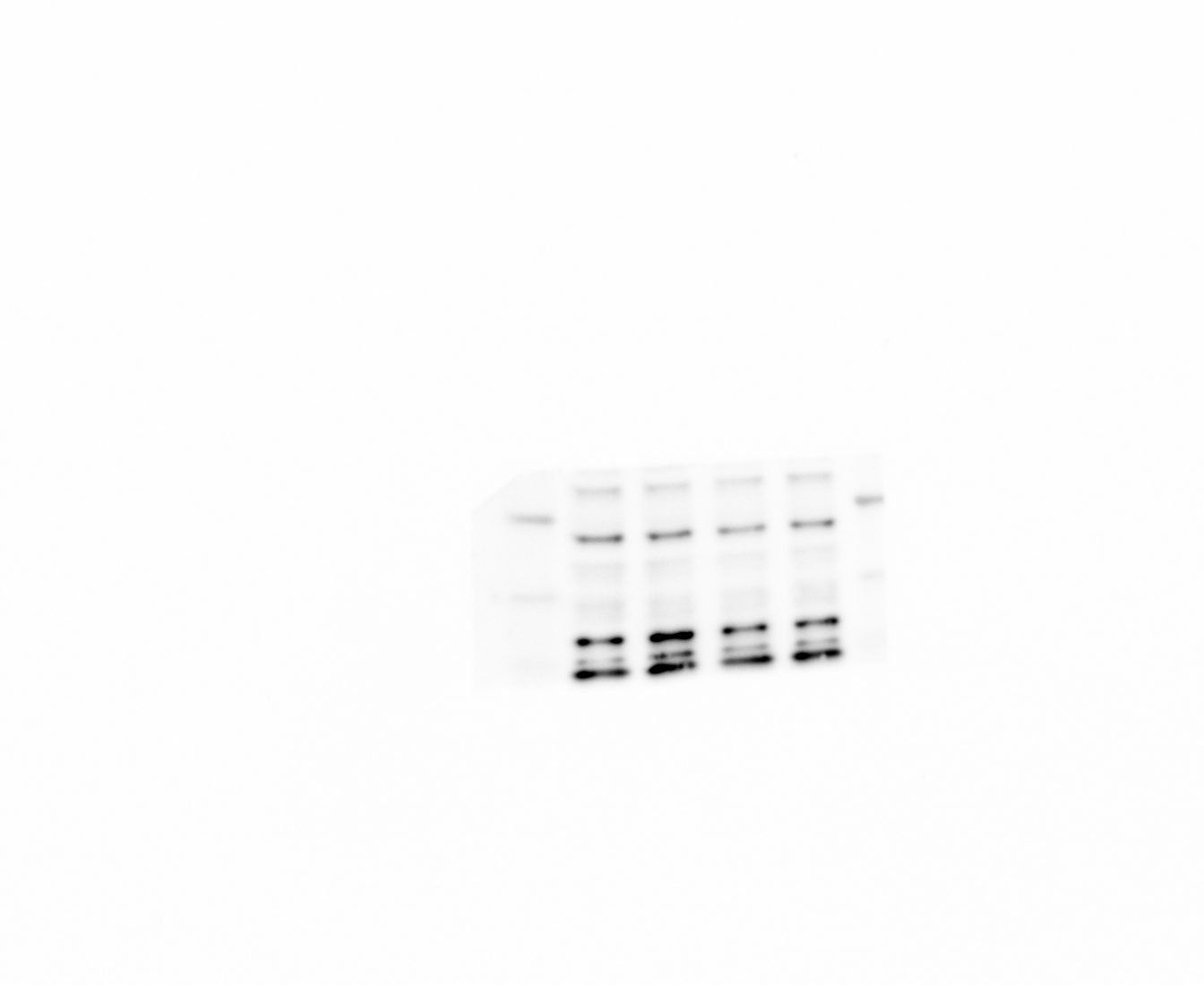

Supplement: Figure 5—source data 1. [file elife-98524-fig5-data1.zip › Fig 5-data1-v1/5E/right/SF3B2.tif]

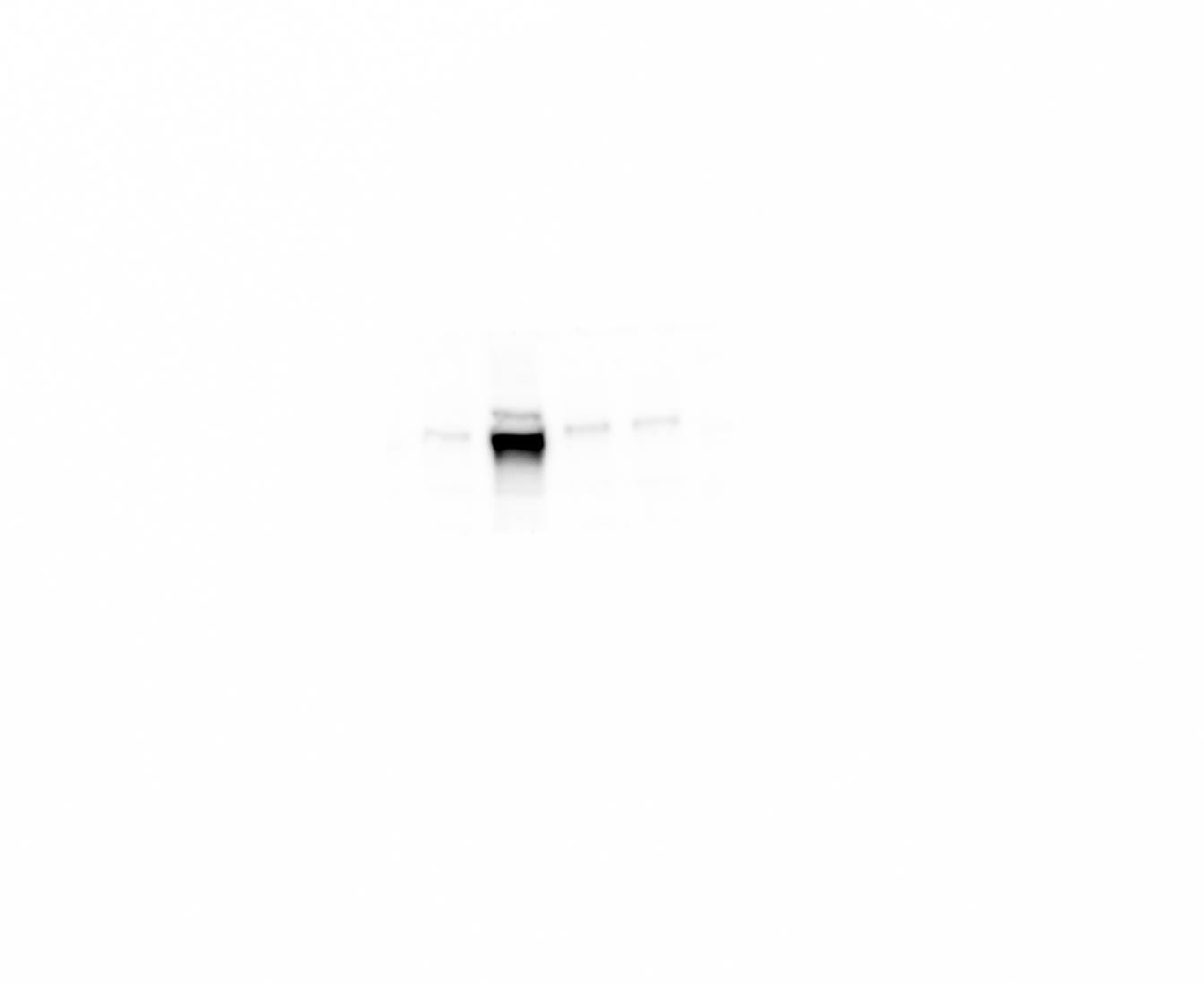

Supplement: Figure 5—source data 1. [file elife-98524-fig5-data1.zip › Fig 5-data1-v1/5E/right/SF3B3.tif]

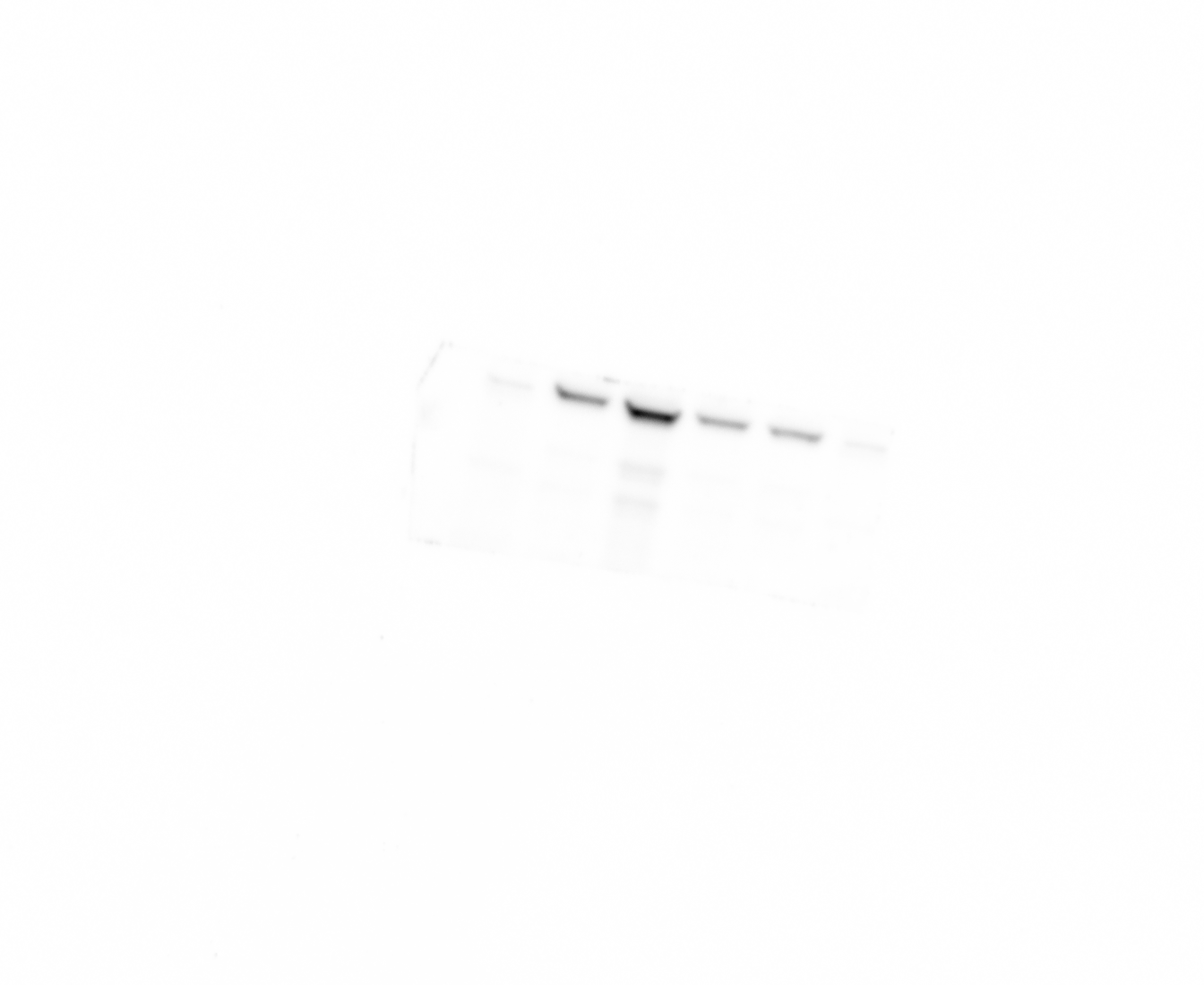

Supplement: Figure 5—source data 1. [file elife-98524-fig5-data1.zip › Fig 5-data1-v1/5E/right/U2AF1.tif]

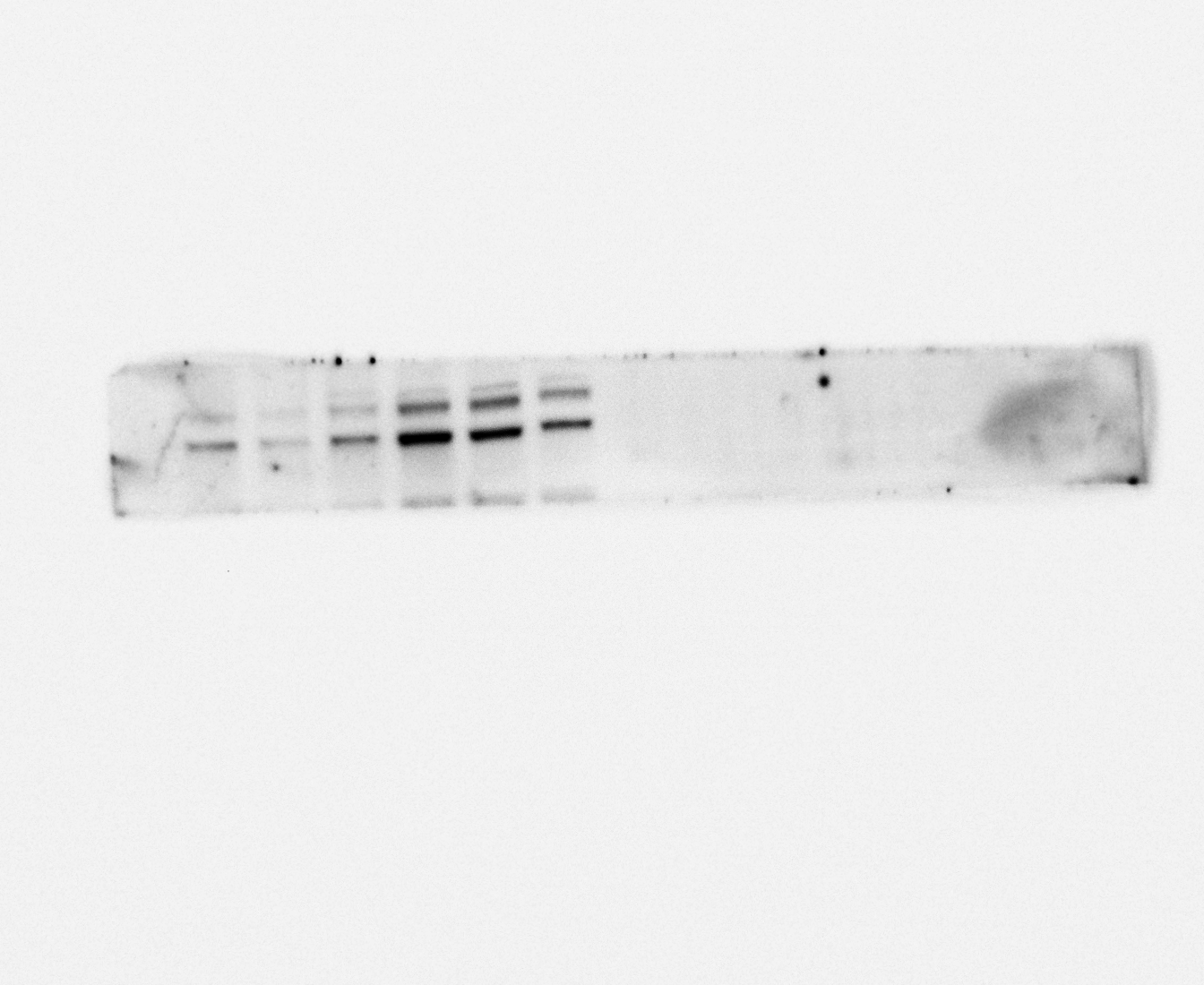

Supplement: Figure 5—source data 1. [file elife-98524-fig5-data1.zip › Fig 5-data1-v1/5G/Ac-k.tif]

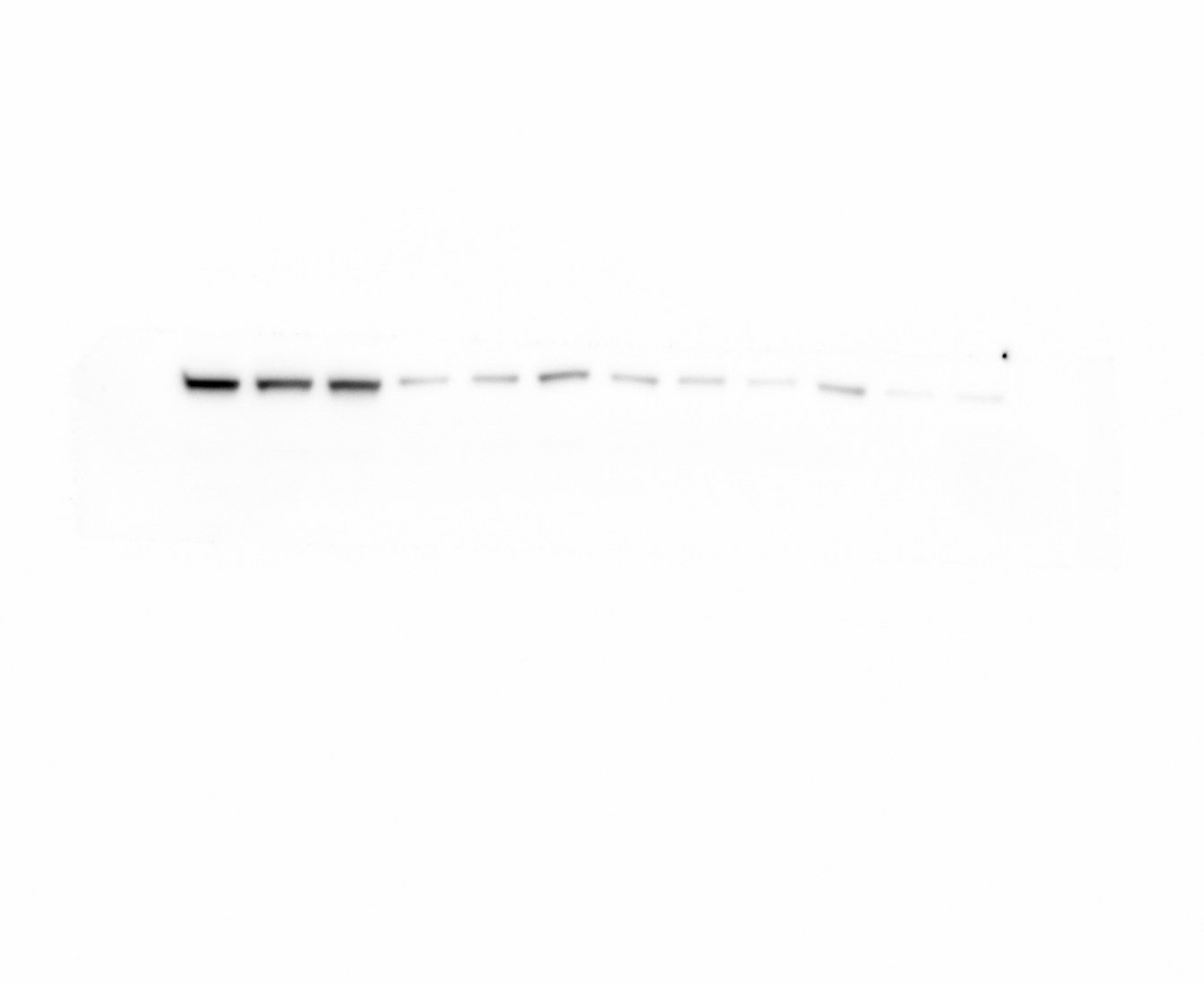

Supplement: Figure 5—source data 1. [file elife-98524-fig5-data1.zip › Fig 5-data1-v1/5G/CCN2.tif]

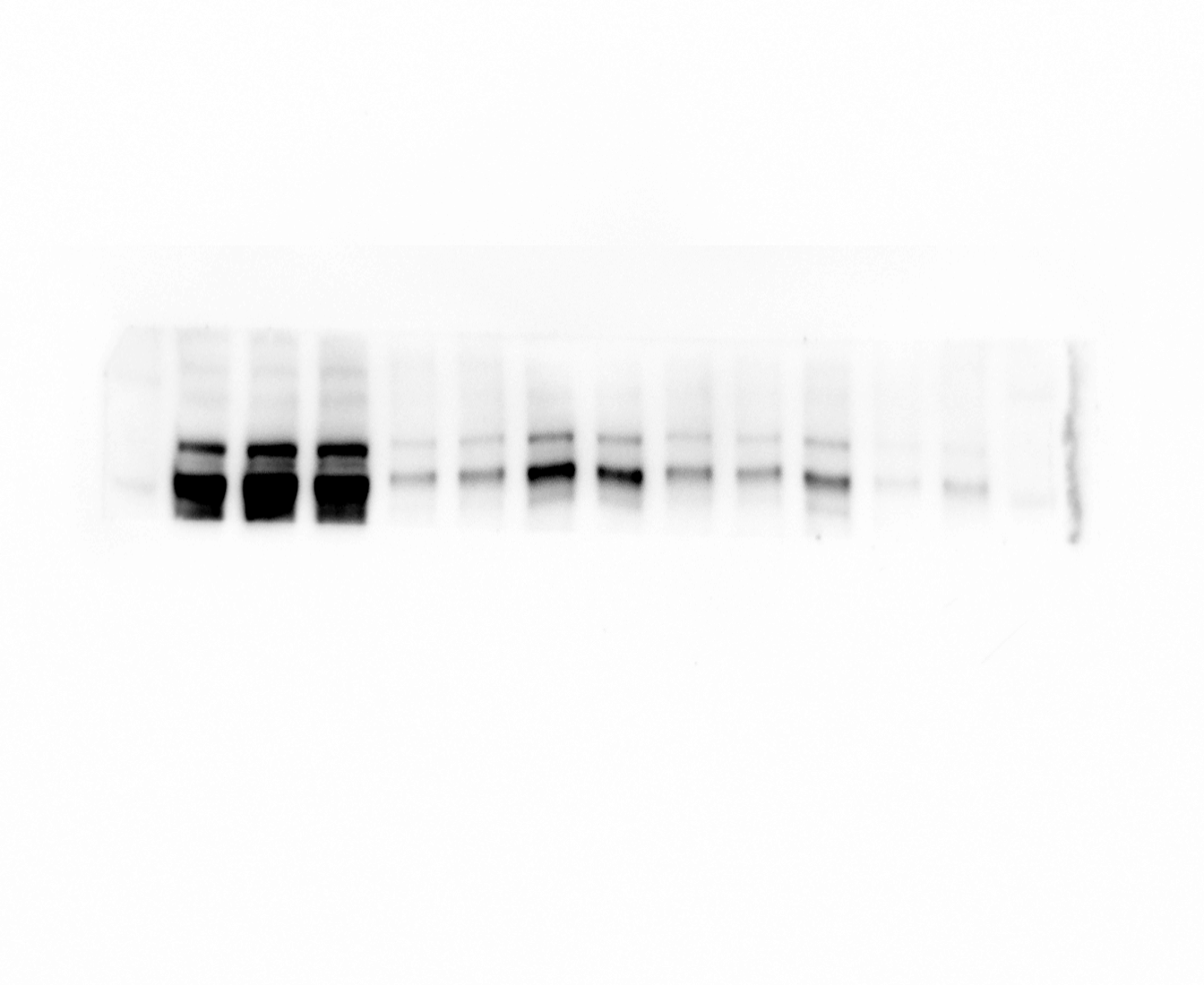

Supplement: Figure 5—source data 1. [file elife-98524-fig5-data1.zip › Fig 5-data1-v1/5G/COL1A1.tif]

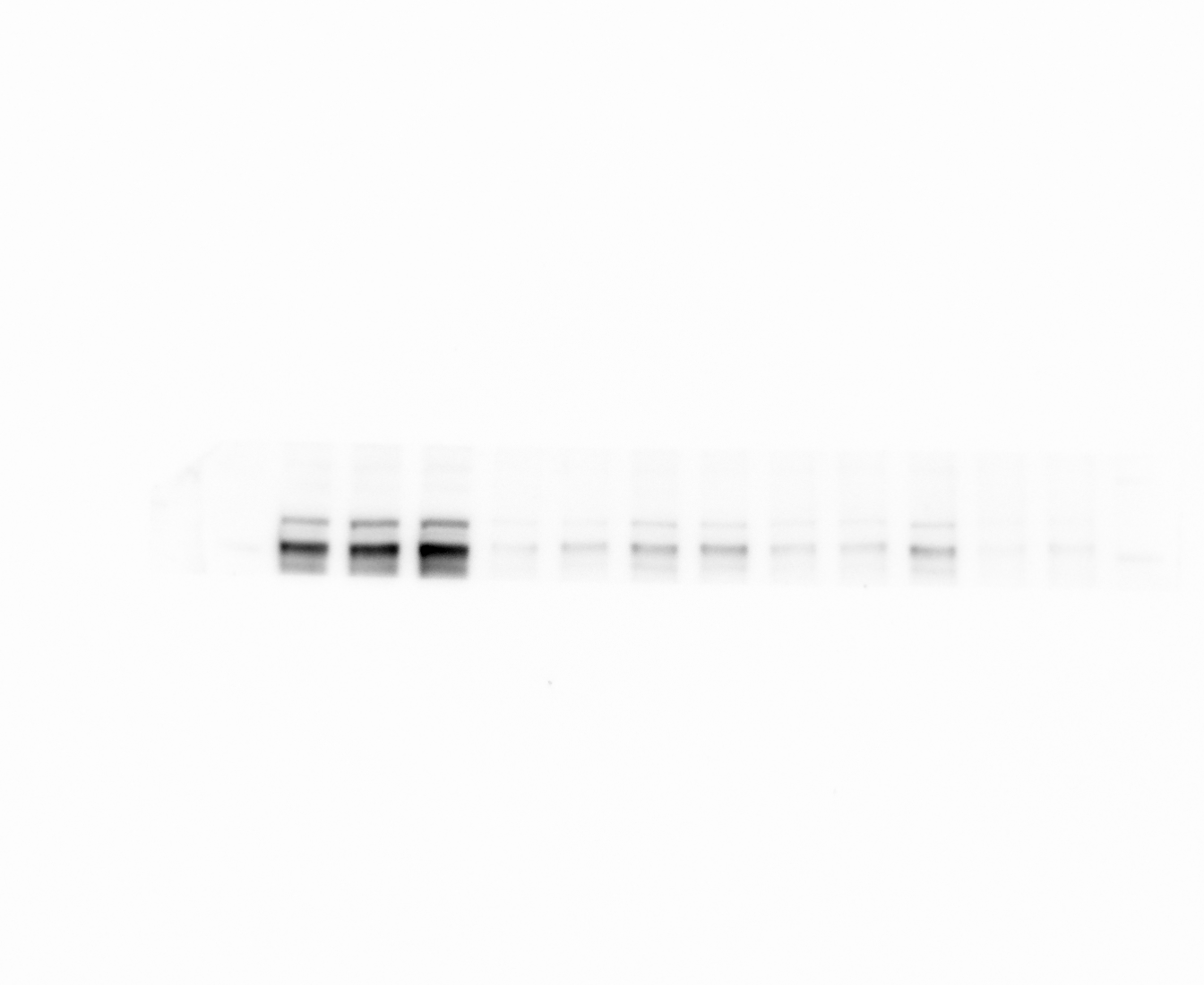

Supplement: Figure 5—source data 1. [file elife-98524-fig5-data1.zip › Fig 5-data1-v1/5G/COL3A1.tif]

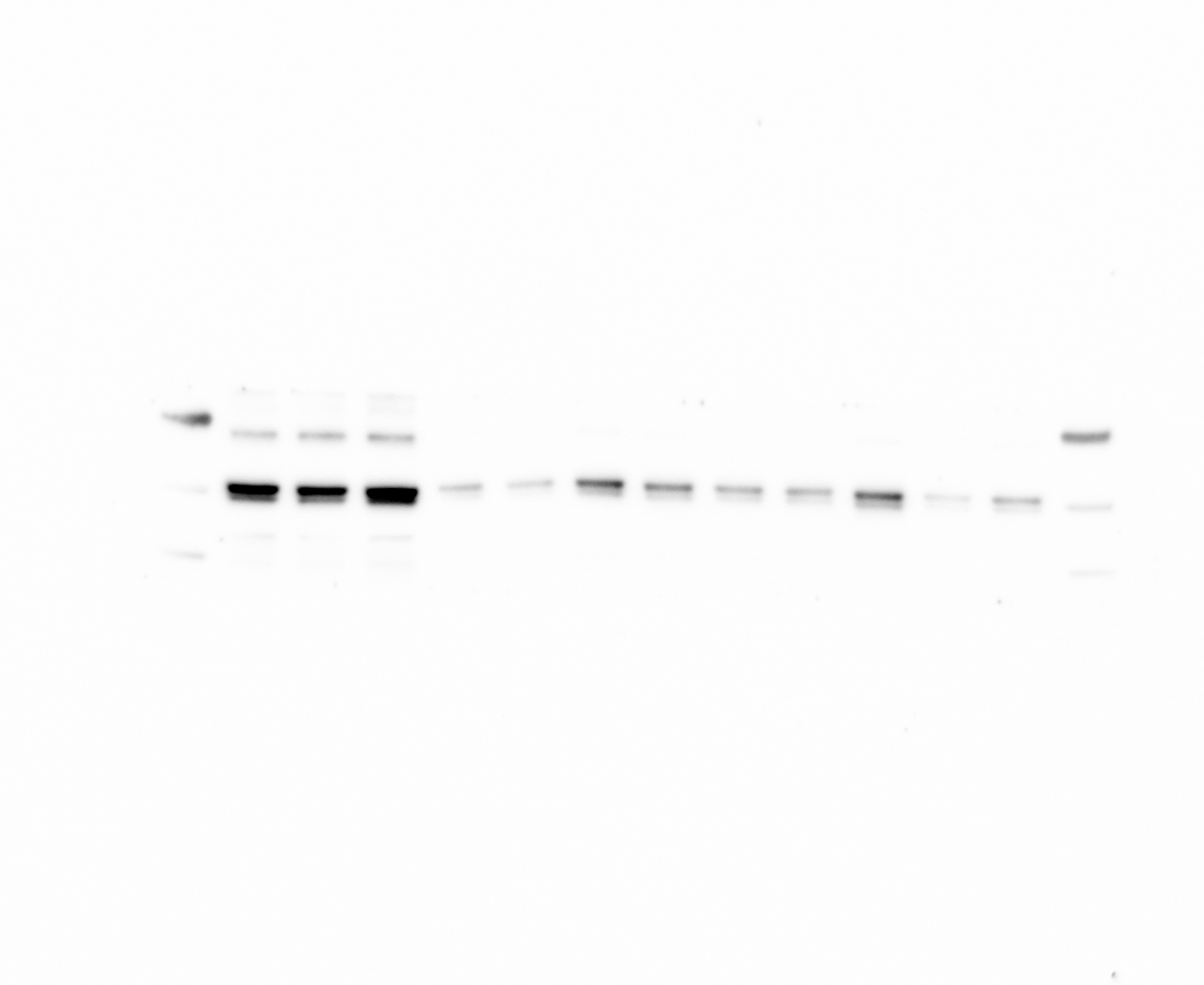

Supplement: Figure 5—source data 1. [file elife-98524-fig5-data1.zip › Fig 5-data1-v1/5G/FN1.tif]

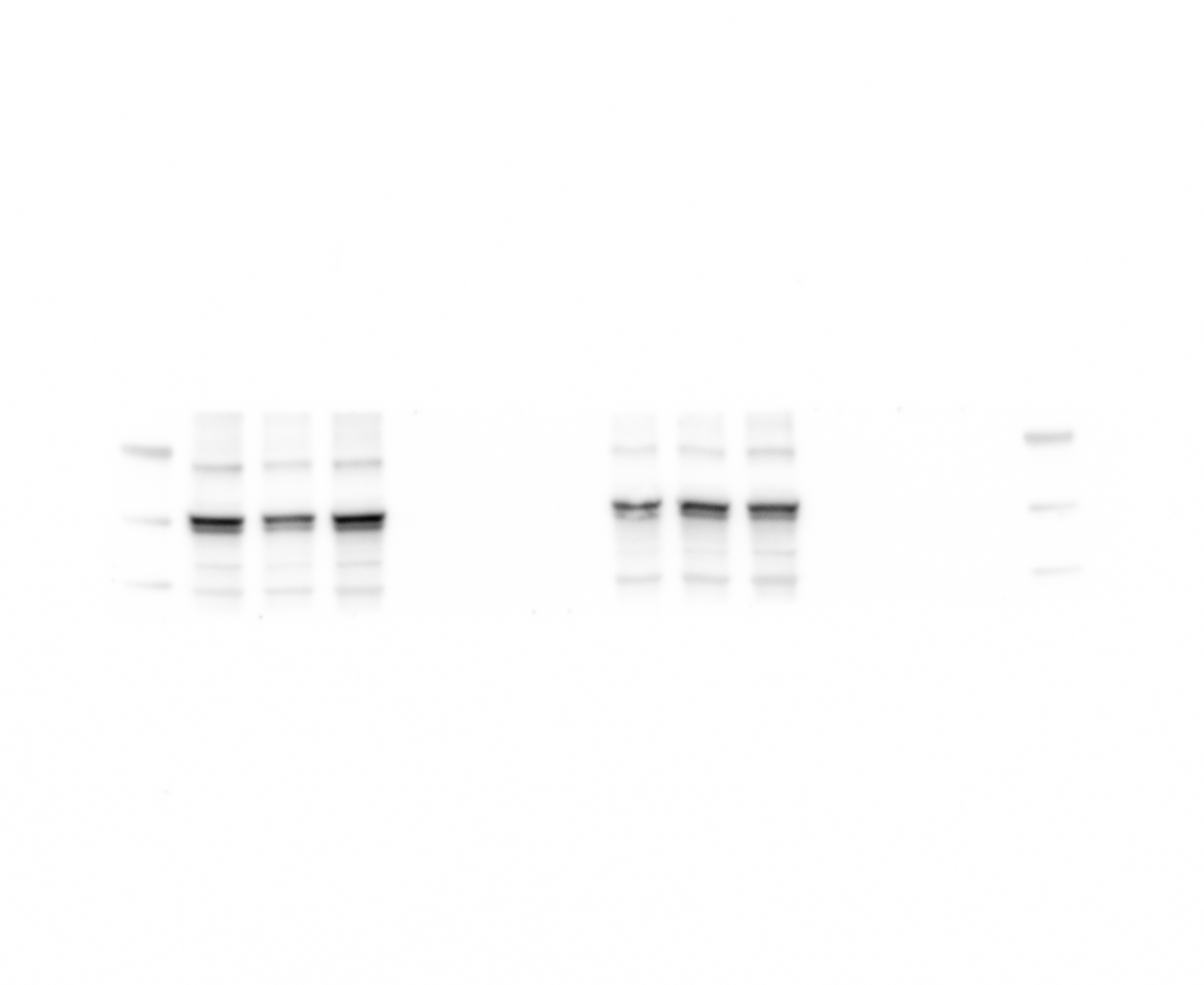

Supplement: Figure 5—source data 1. [file elife-98524-fig5-data1.zip › Fig 5-data1-v1/5G/SIRT4.tif]

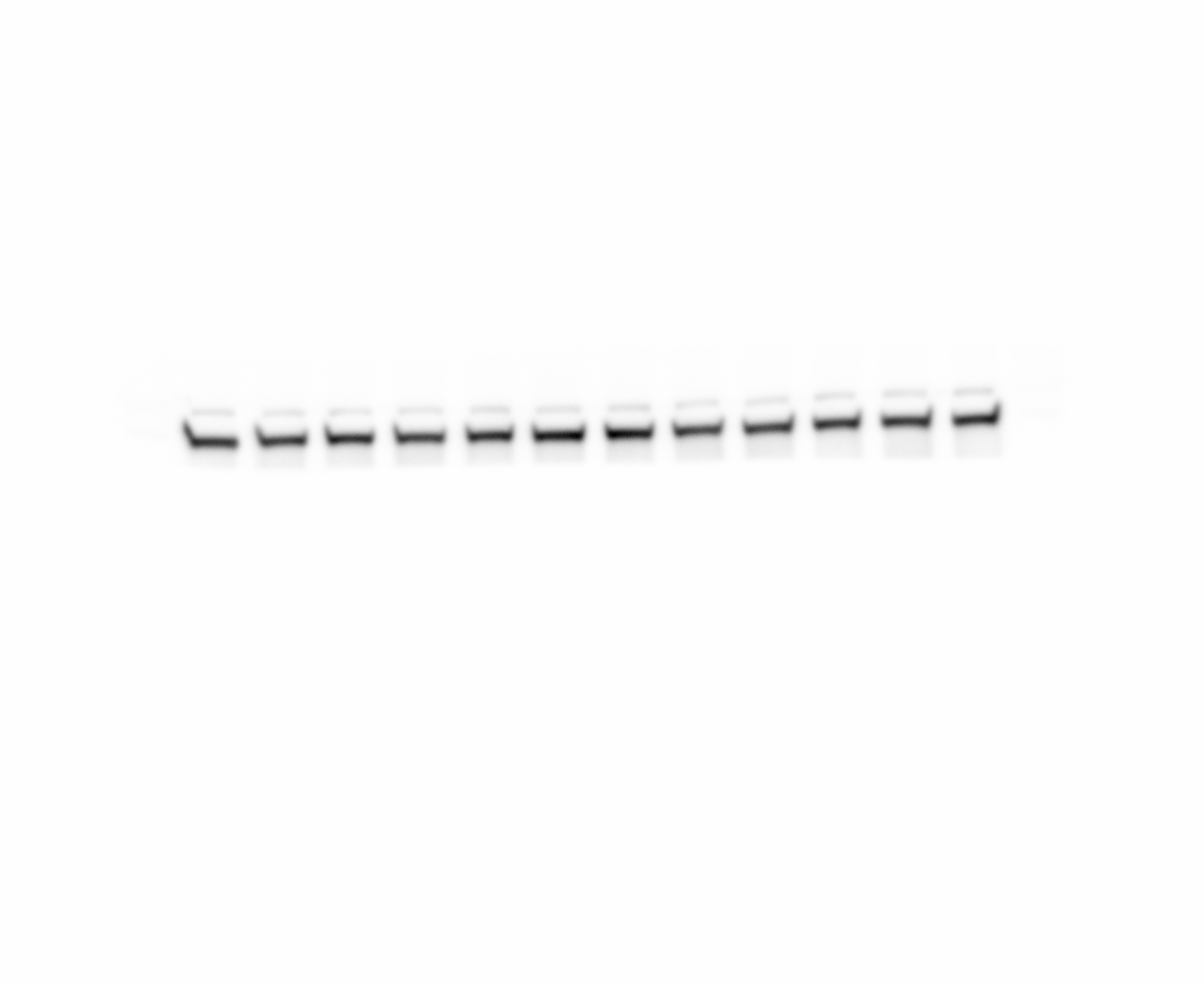

Supplement: Figure 5—source data 1. [file elife-98524-fig5-data1.zip › Fig 5-data1-v1/5G/Tubulin.tif]

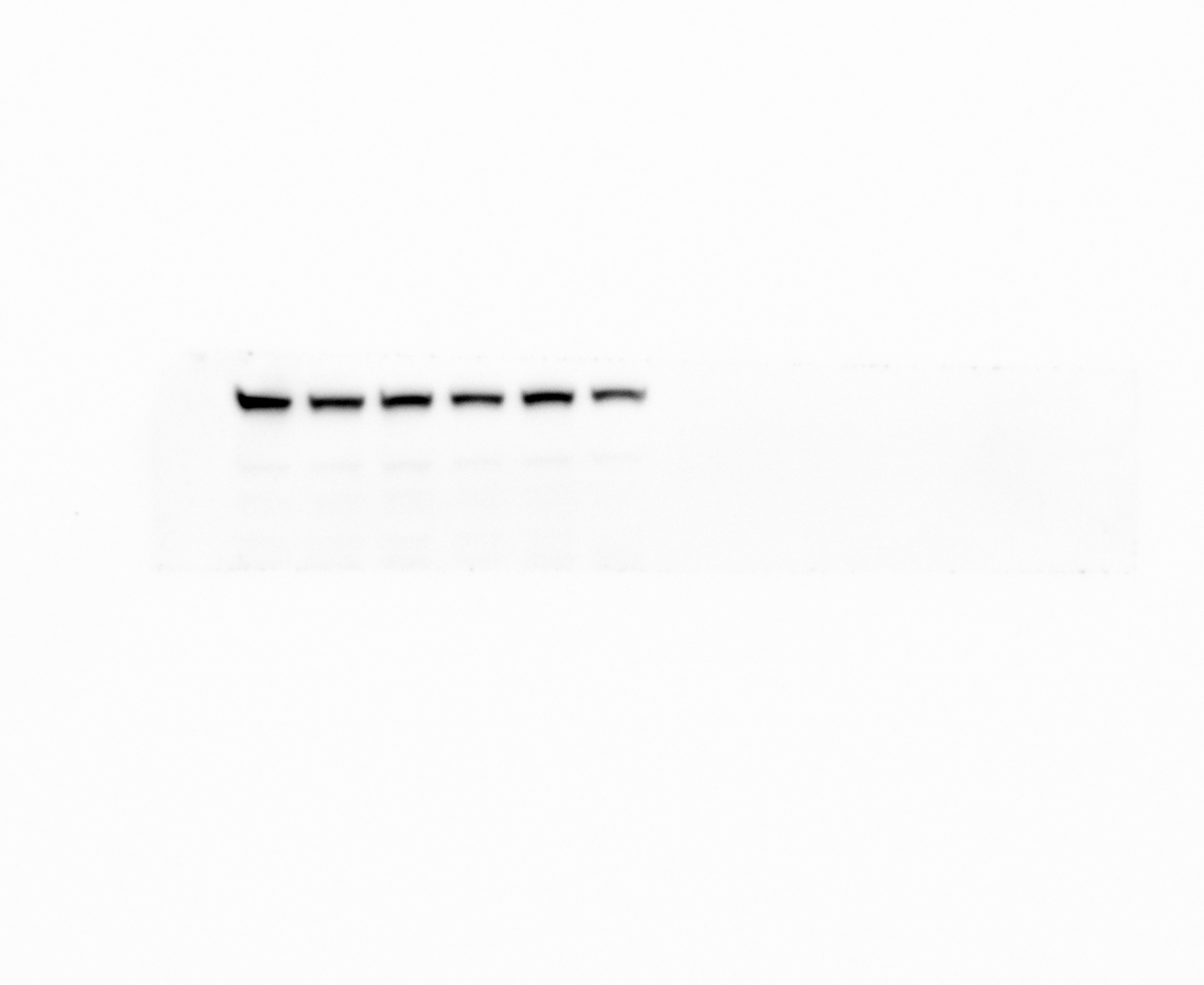

Supplement: Figure 5—source data 1. [file elife-98524-fig5-data1.zip › Fig 5-data1-v1/5G/U2AF2 bottom.tif]

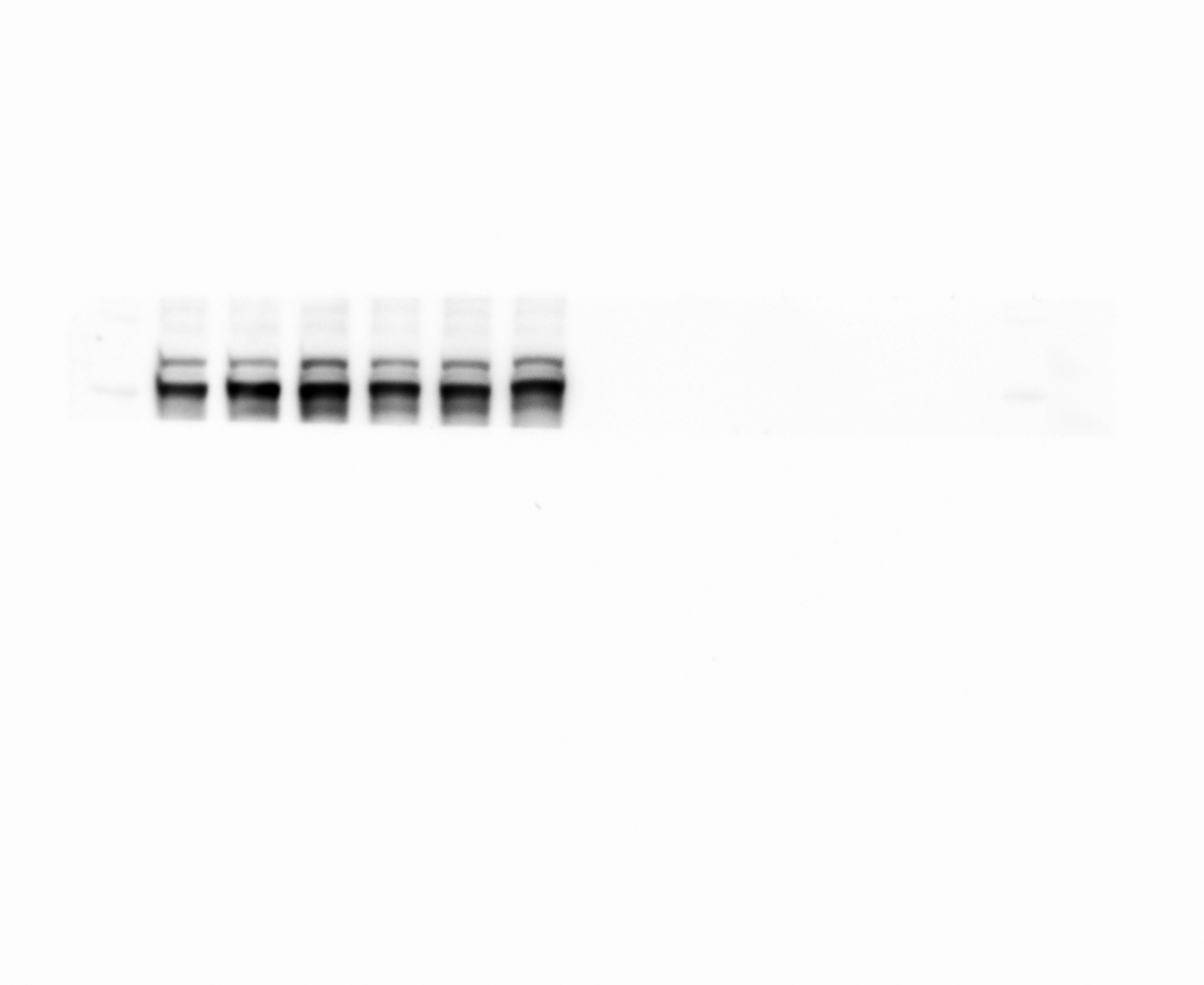

Supplement: Figure 5—source data 1. [file elife-98524-fig5-data1.zip › Fig 5-data1-v1/5G/U2AF2 upper.tif]

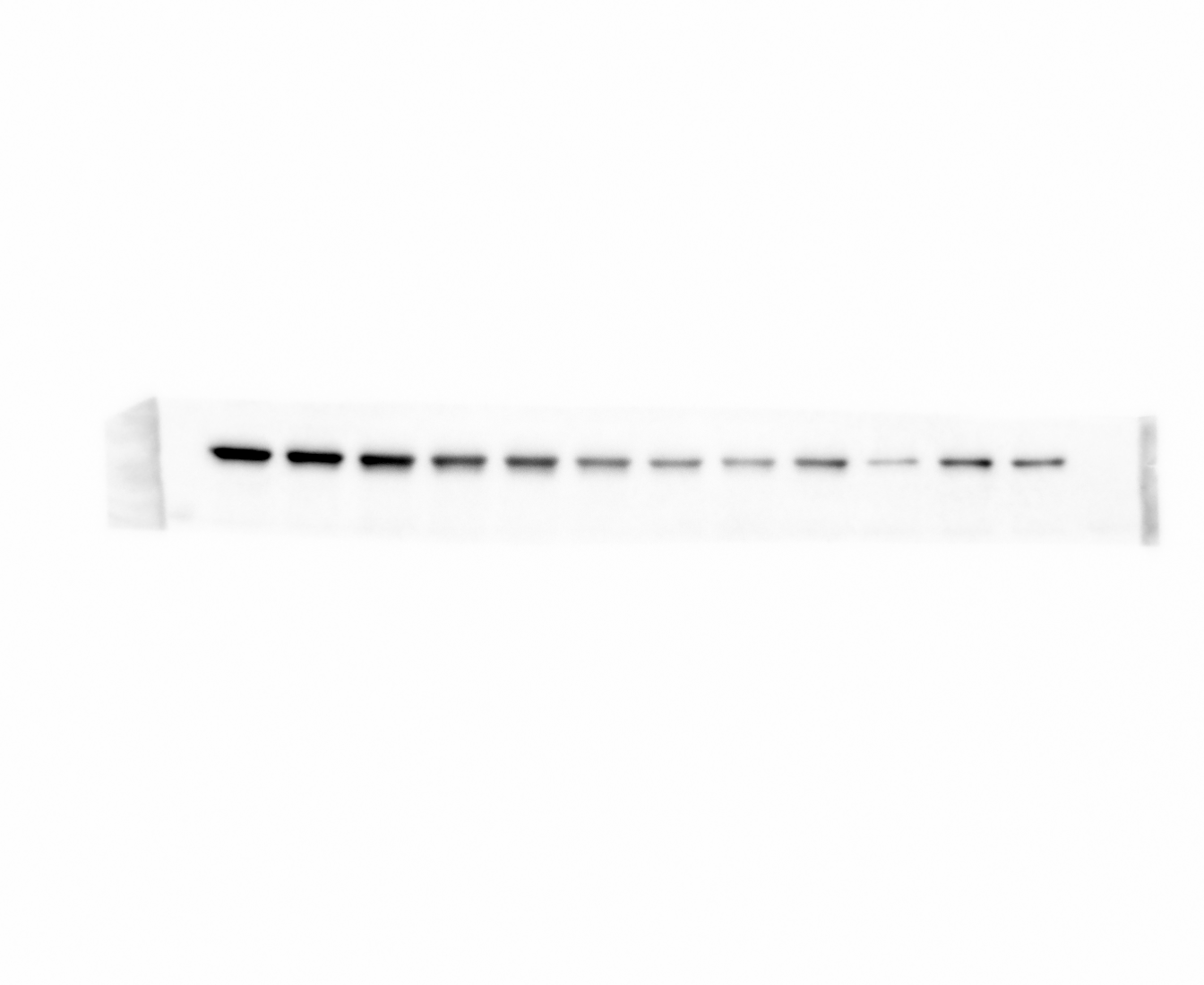

Supplement: Figure 5—source data 1. [file elife-98524-fig5-data1.zip › Fig 5-data1-v1/5I/Ac-k.tif]

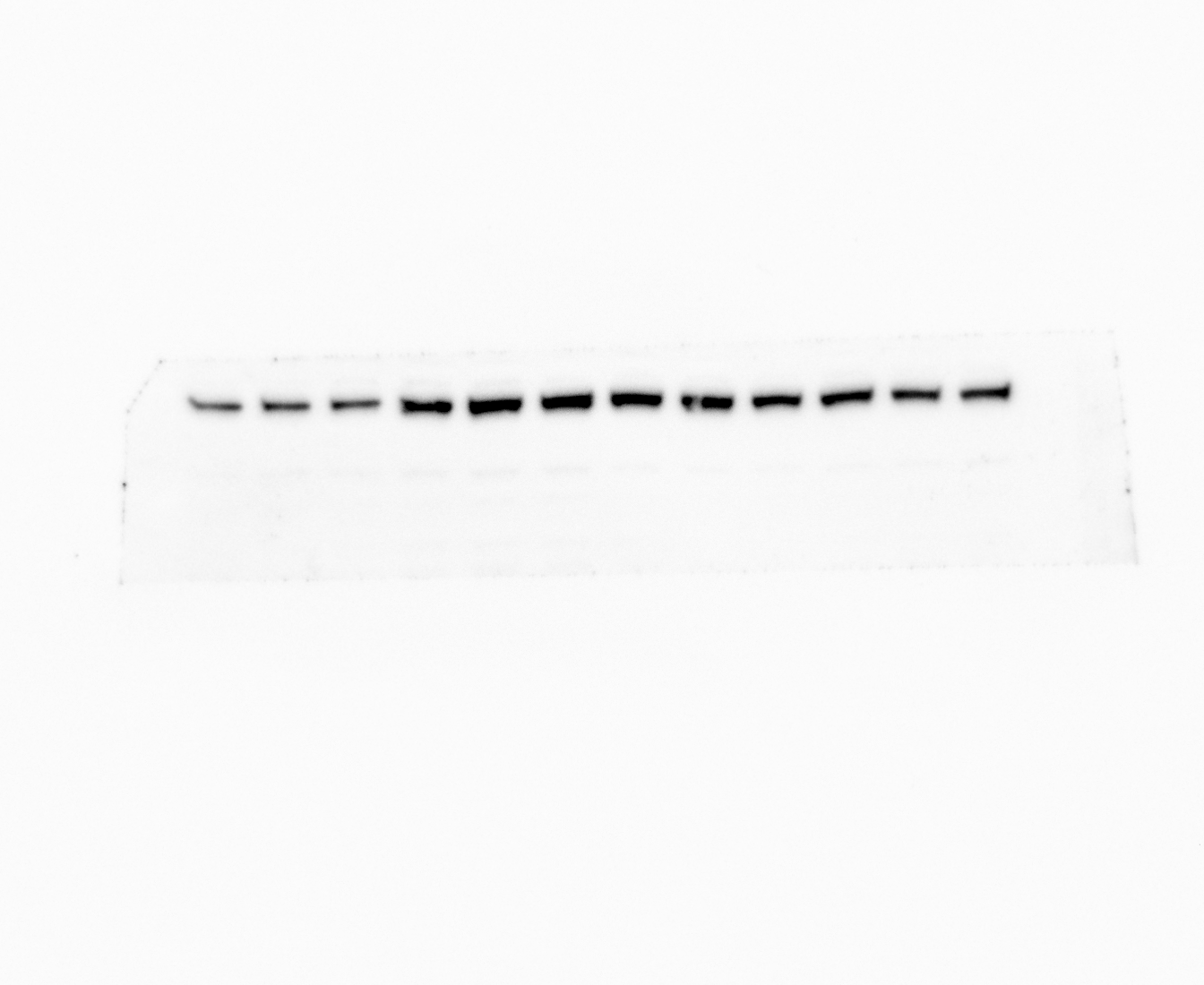

Supplement: Figure 5—source data 1. [file elife-98524-fig5-data1.zip › Fig 5-data1-v1/5I/CCN2.tif]

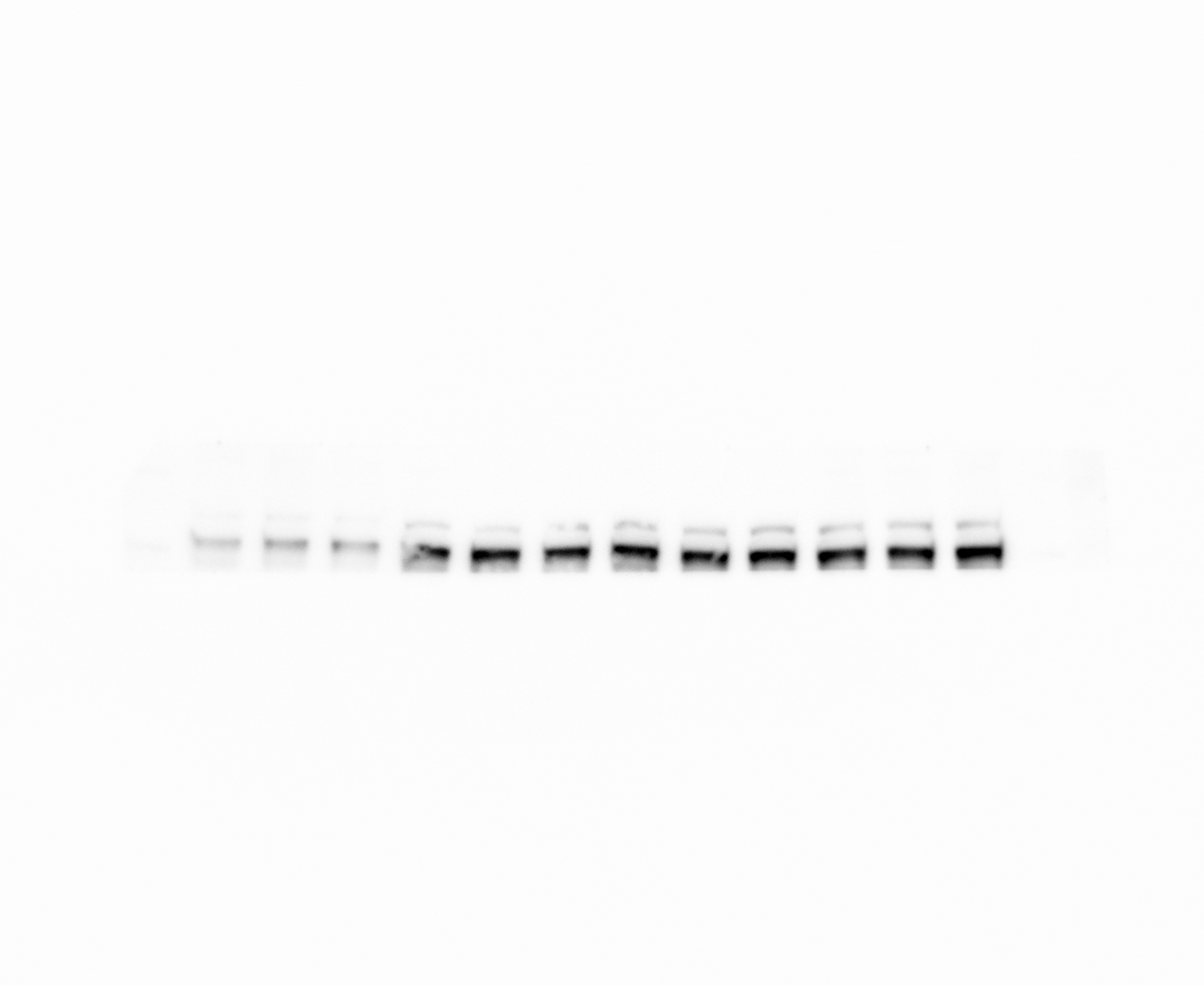

Supplement: Figure 5—source data 1. [file elife-98524-fig5-data1.zip › Fig 5-data1-v1/5I/COL1A1.tif]

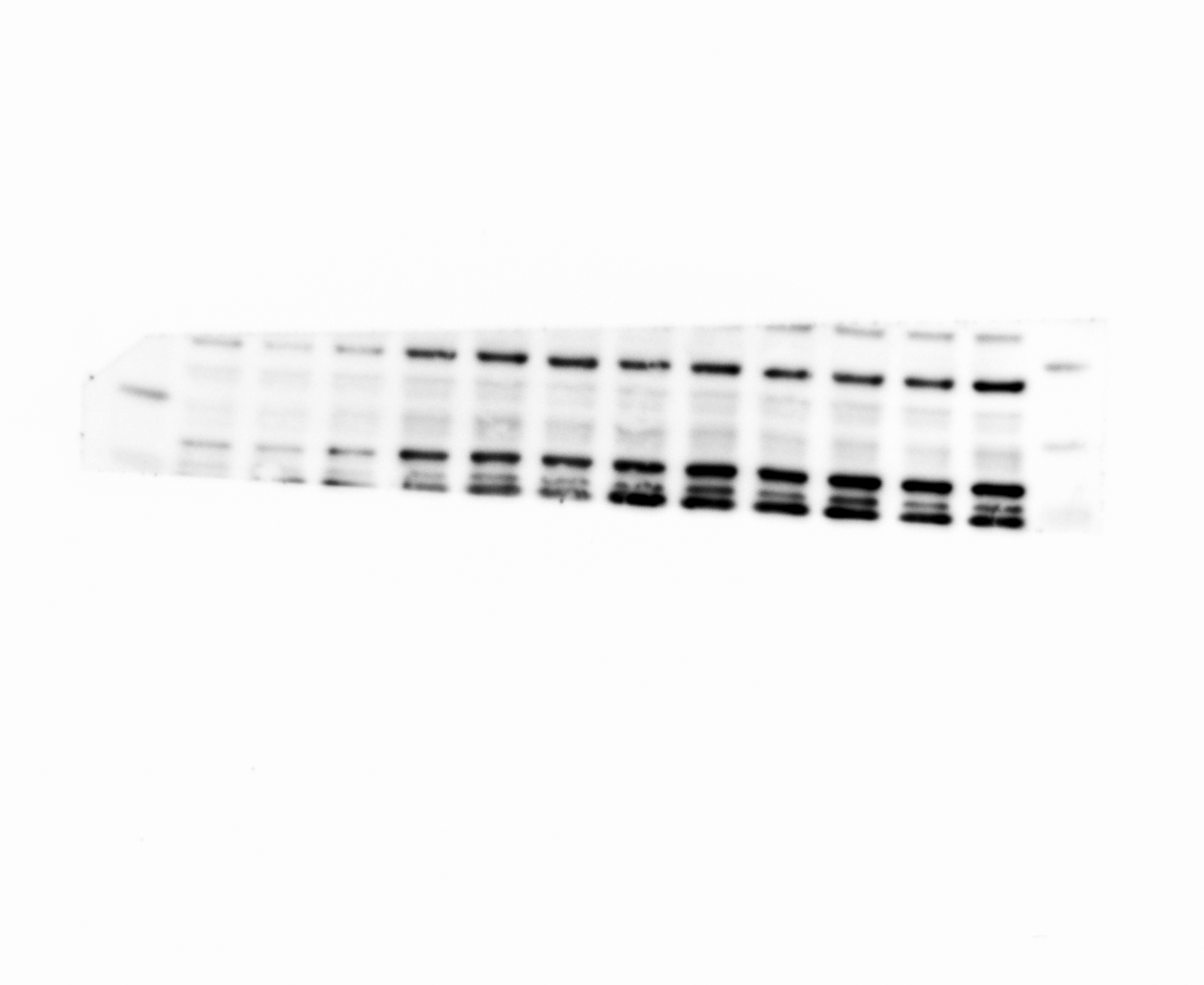

Supplement: Figure 5—source data 1. [file elife-98524-fig5-data1.zip › Fig 5-data1-v1/5I/COL3A1.tif]

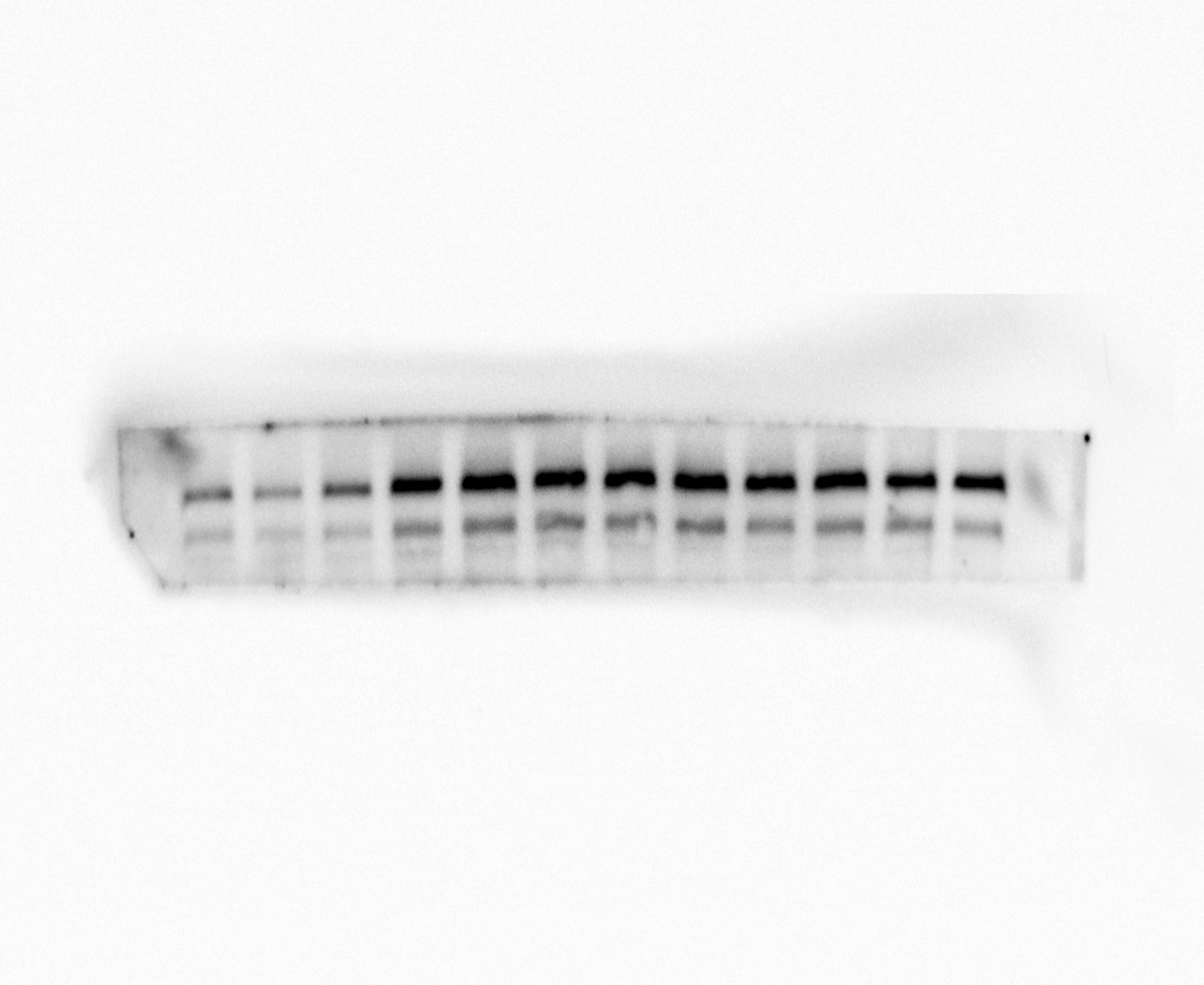

Supplement: Figure 5—source data 1. [file elife-98524-fig5-data1.zip › Fig 5-data1-v1/5I/FN1.tif]

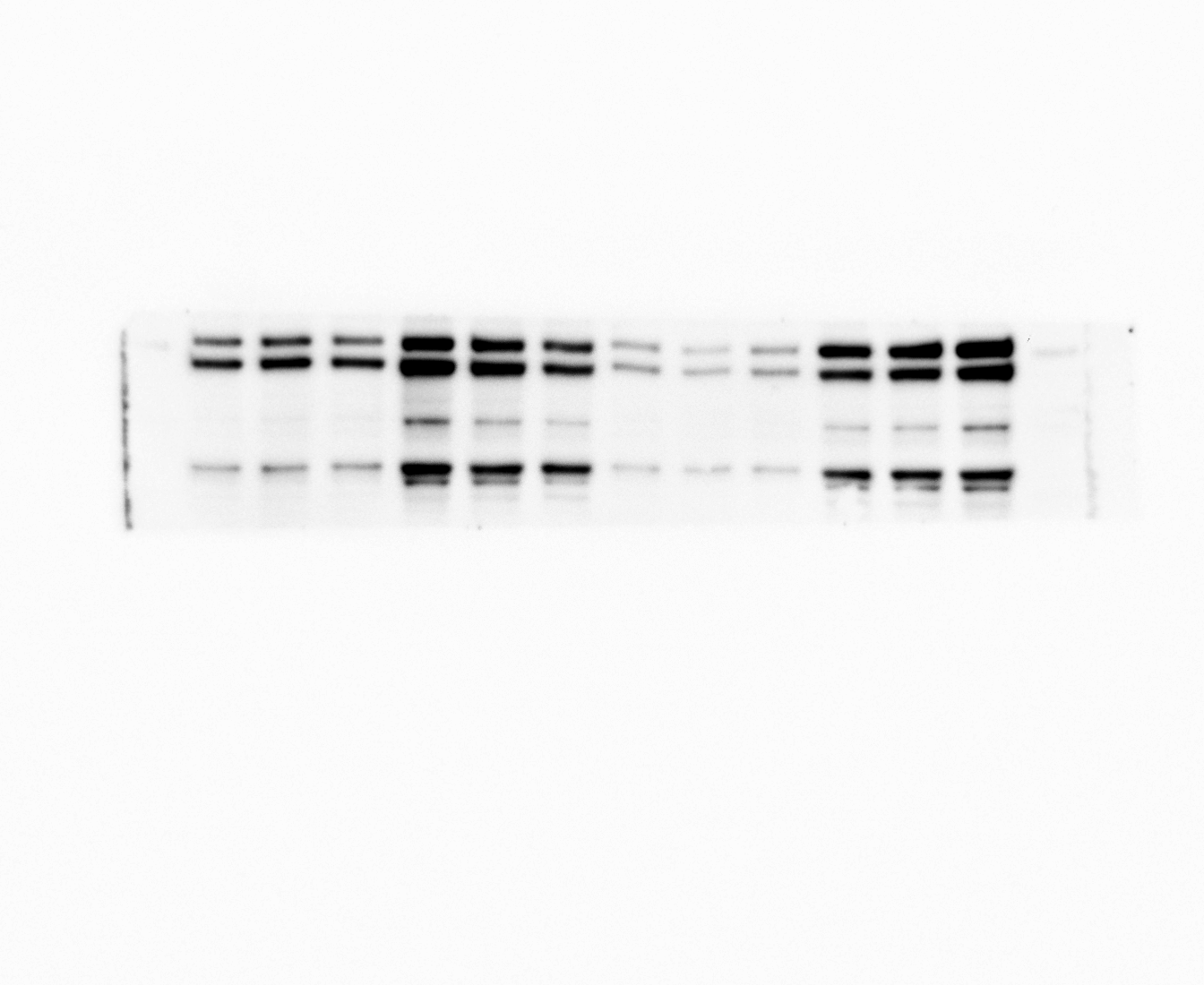

Supplement: Figure 5—source data 1. [file elife-98524-fig5-data1.zip › Fig 5-data1-v1/5I/SIRT4.tif]

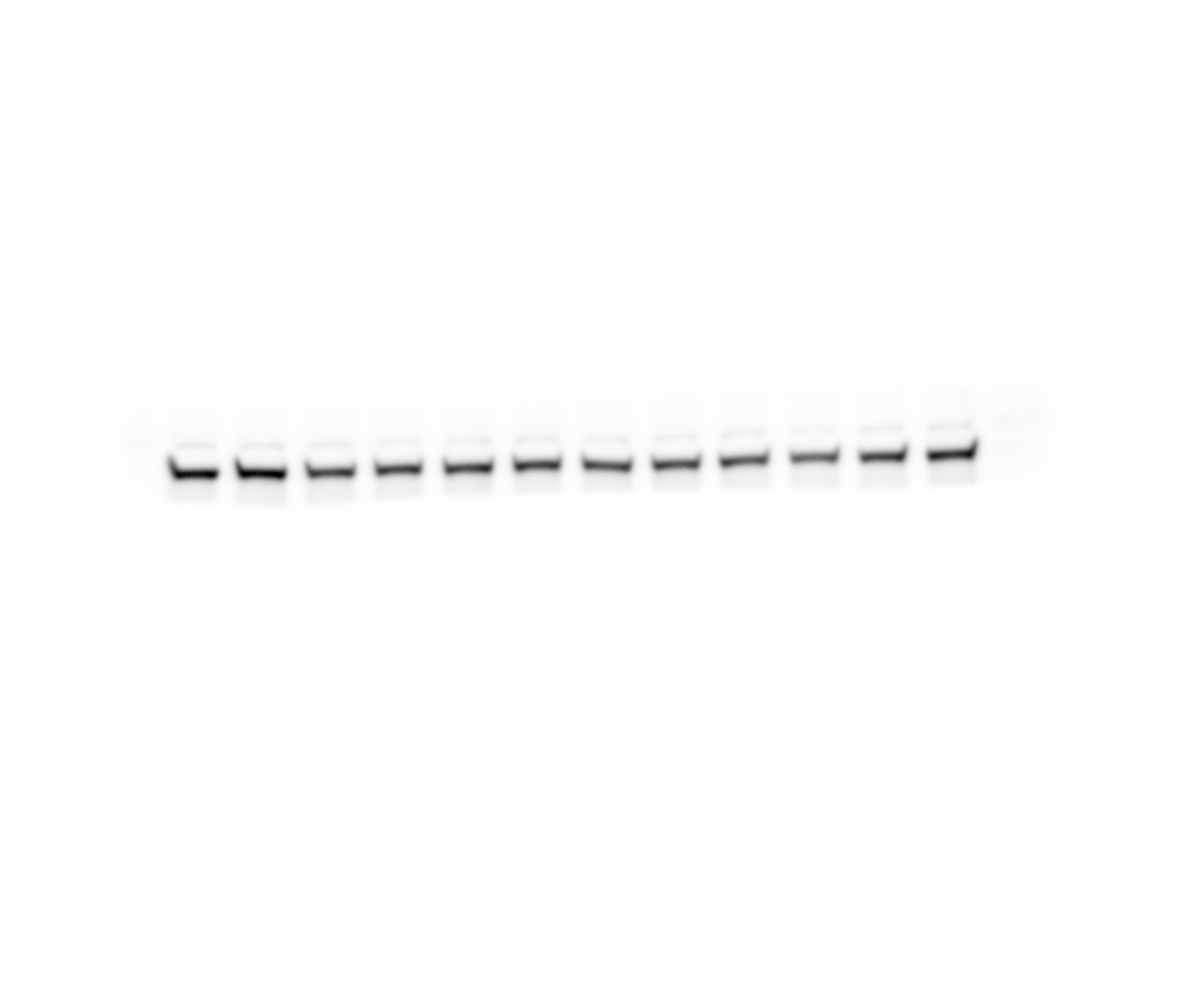

Supplement: Figure 5—source data 1. [file elife-98524-fig5-data1.zip › Fig 5-data1-v1/5I/Tubulin.tif]

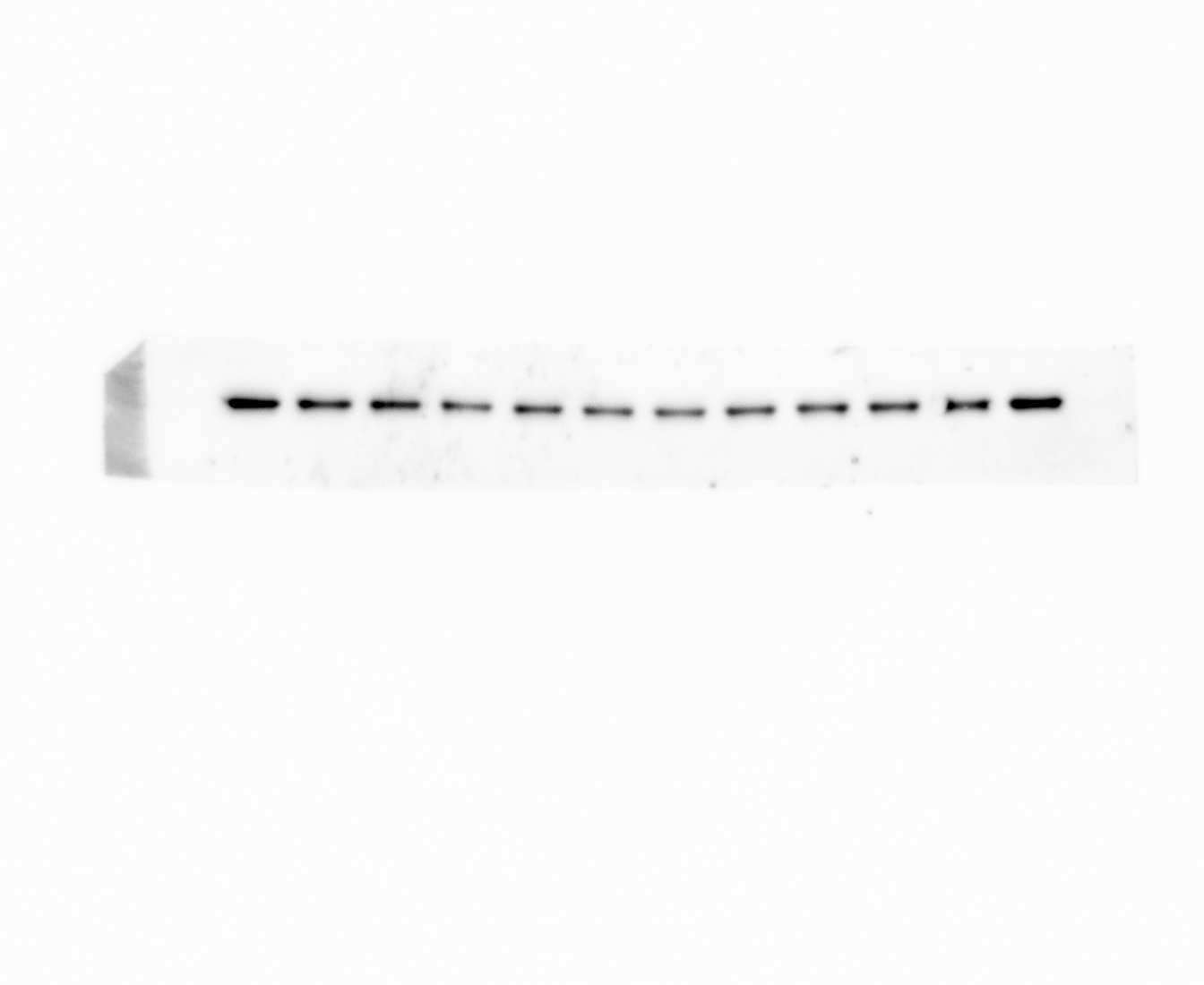

Supplement: Figure 5—source data 1. [file elife-98524-fig5-data1.zip › Fig 5-data1-v1/5I/U2AF2 bottom.tif]

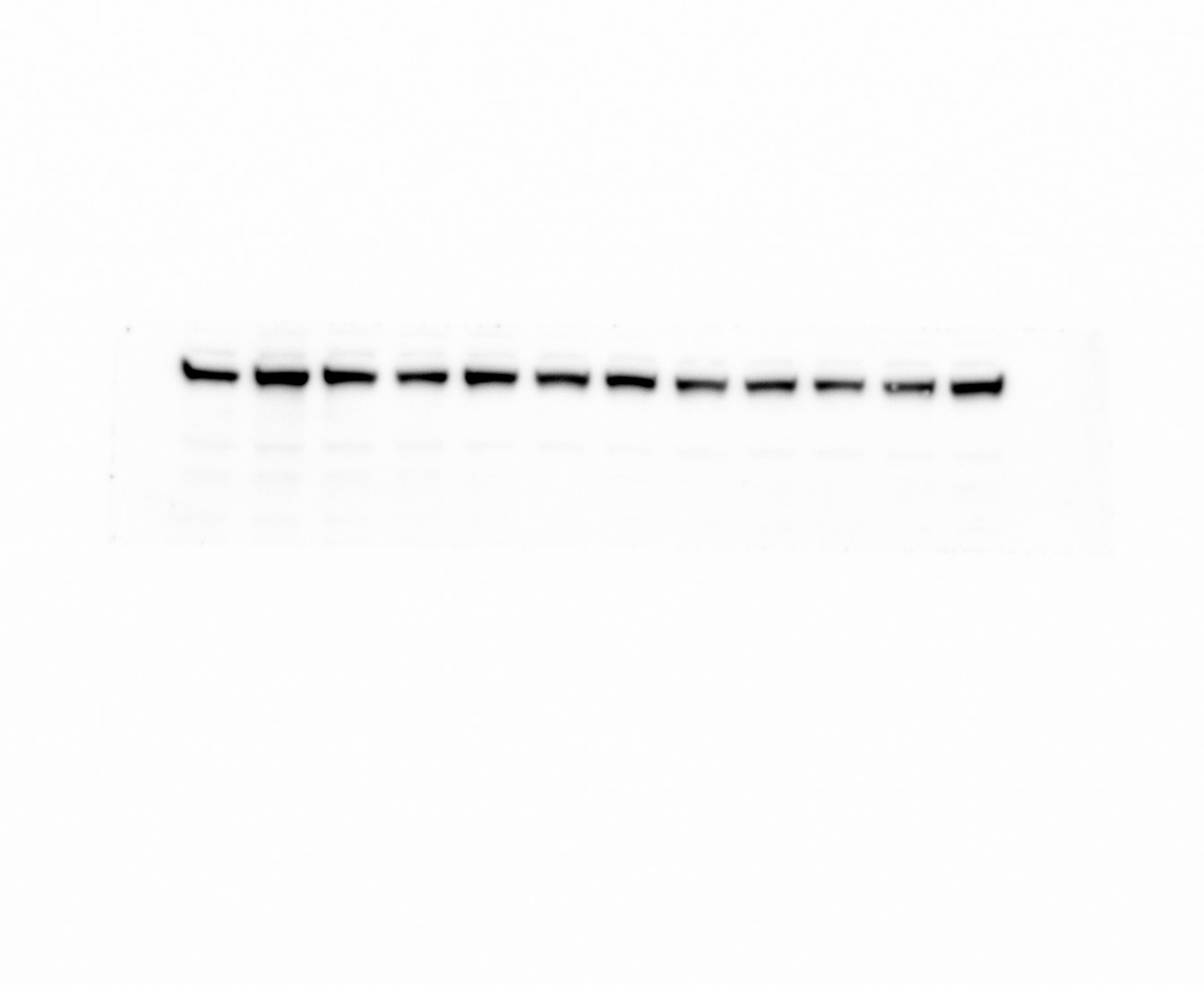

Supplement: Figure 5—source data 1. [file elife-98524-fig5-data1.zip › Fig 5-data1-v1/5I/U2AF2 upper.tif]

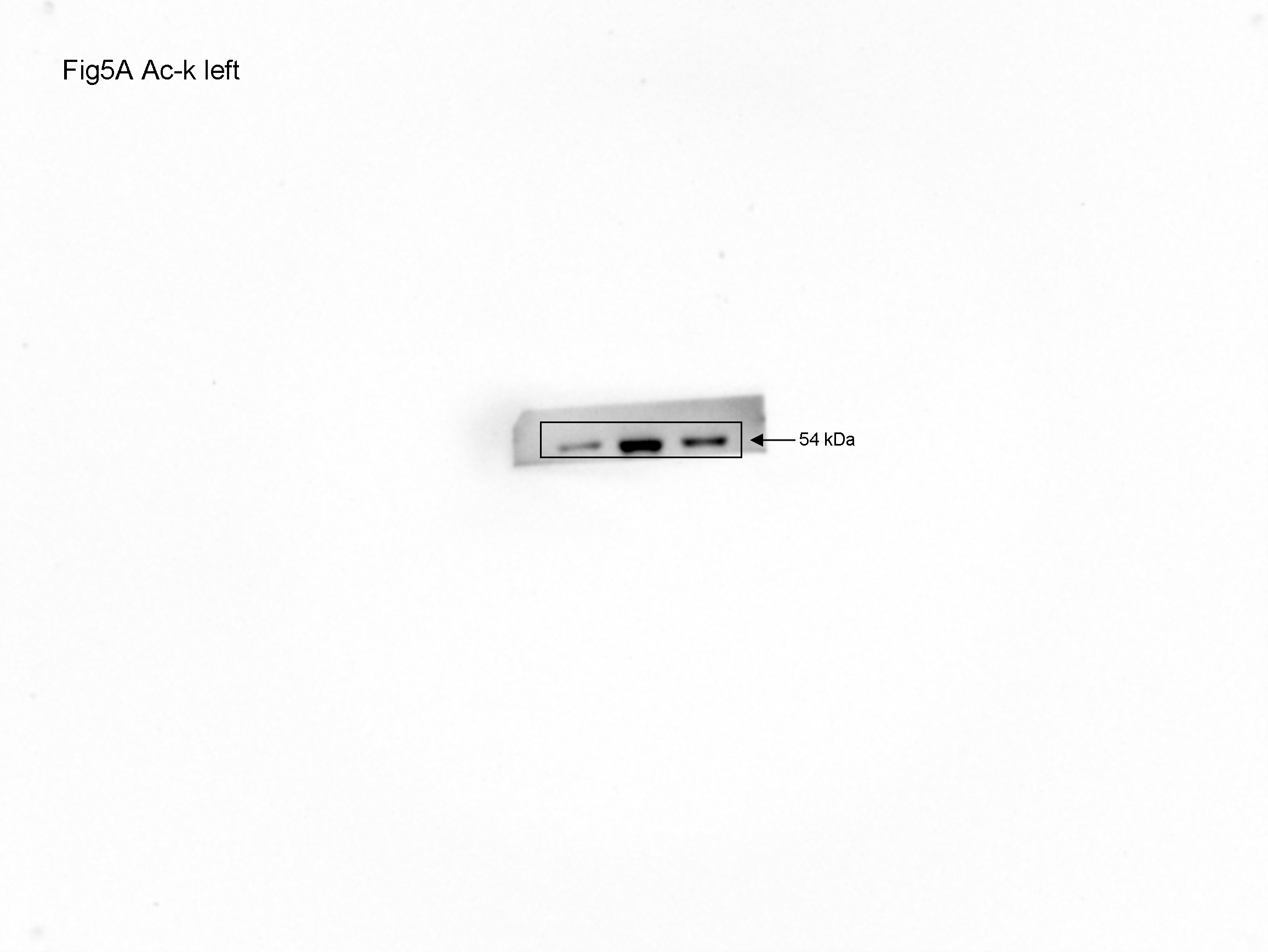

Supplement: Figure 5—source data 2. [file elife-98524-fig5-data2.zip › Fig 5-data2-v1/5A/left/Ac-k left.tif]

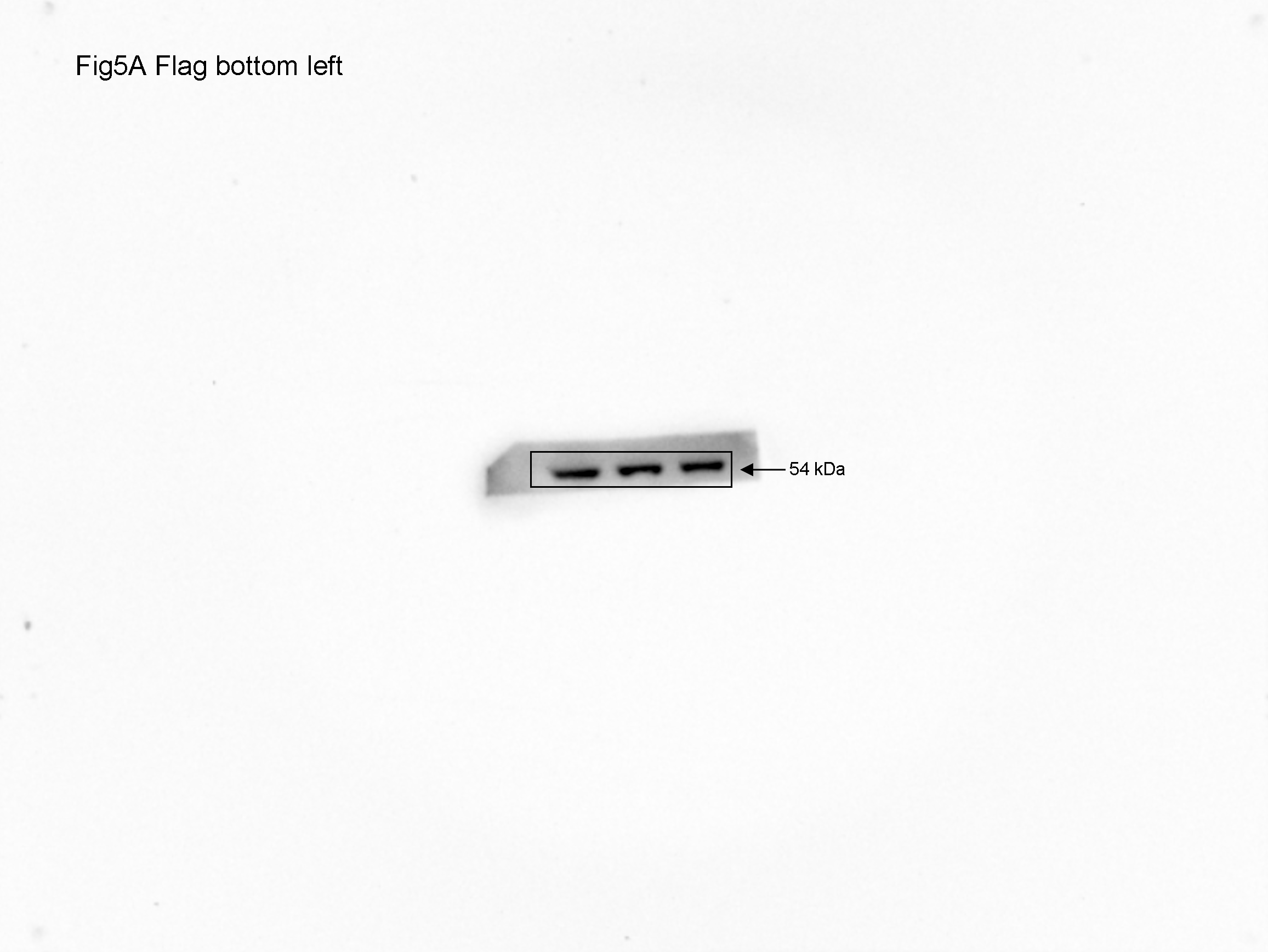

Supplement: Figure 5—source data 2. [file elife-98524-fig5-data2.zip › Fig 5-data2-v1/5A/left/Flag bottom left.tif]

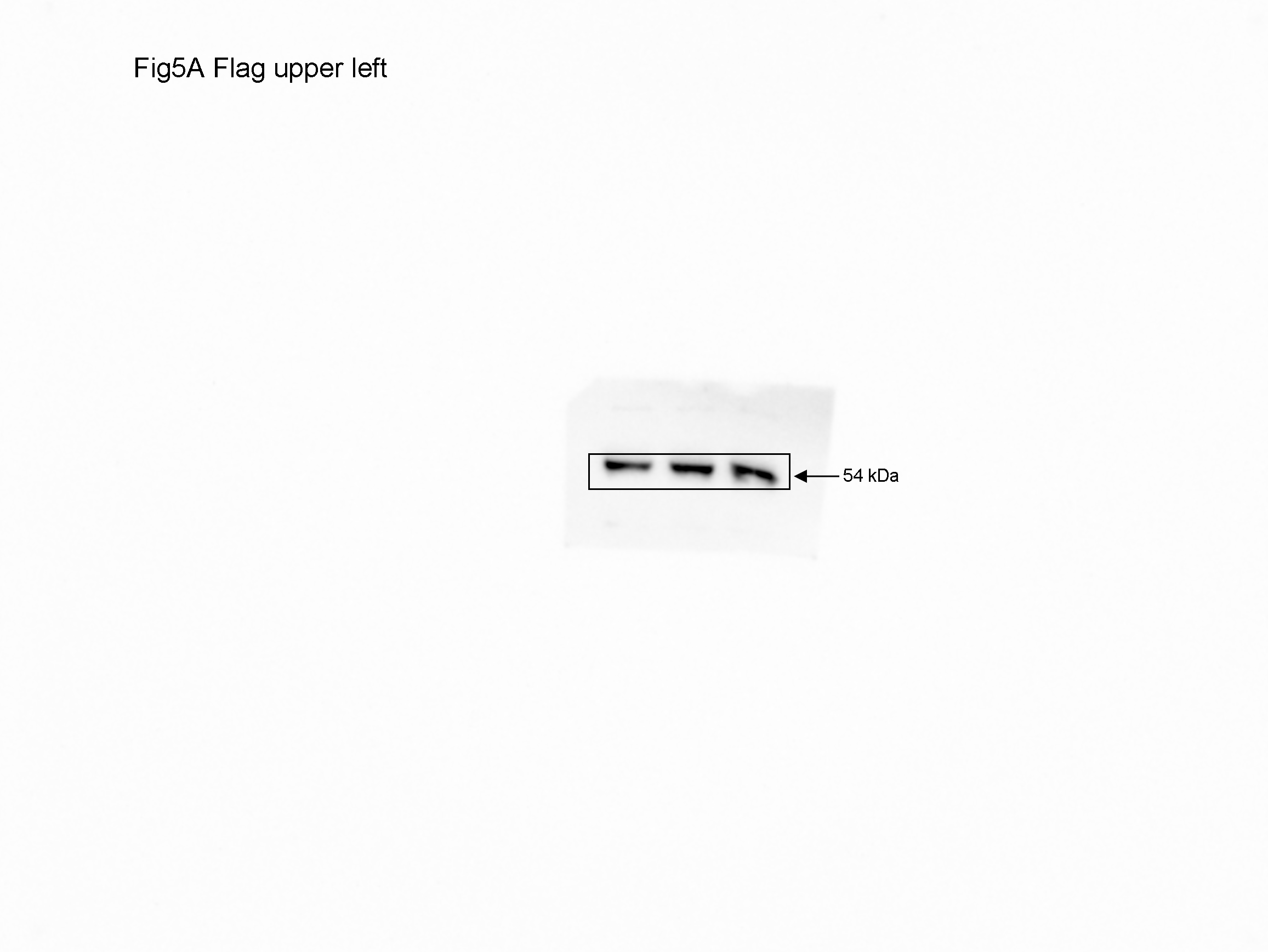

Supplement: Figure 5—source data 2. [file elife-98524-fig5-data2.zip › Fig 5-data2-v1/5A/left/Flag upper left.tif]

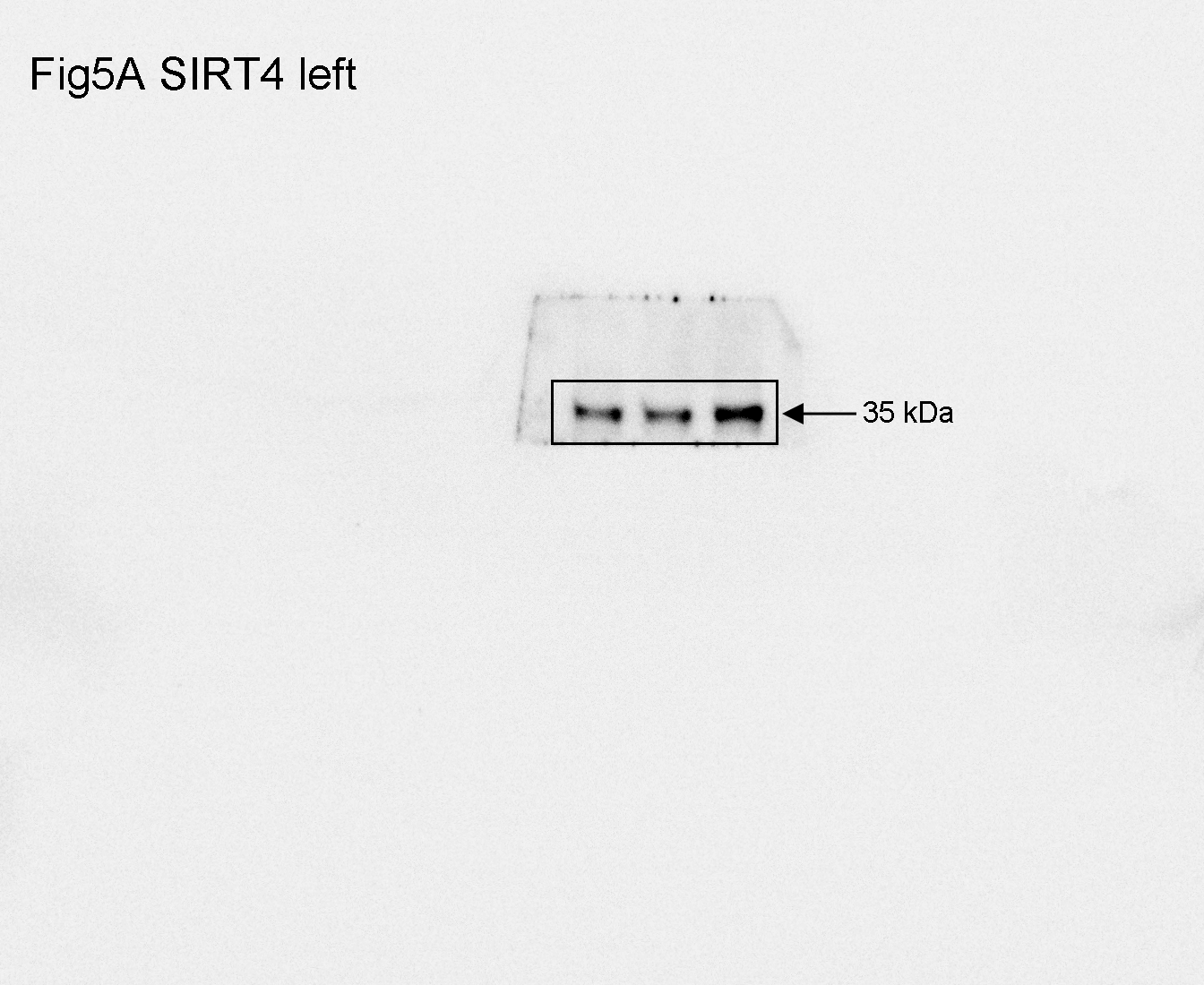

Supplement: Figure 5—source data 2. [file elife-98524-fig5-data2.zip › Fig 5-data2-v1/5A/left/SIRT4 left.tif]

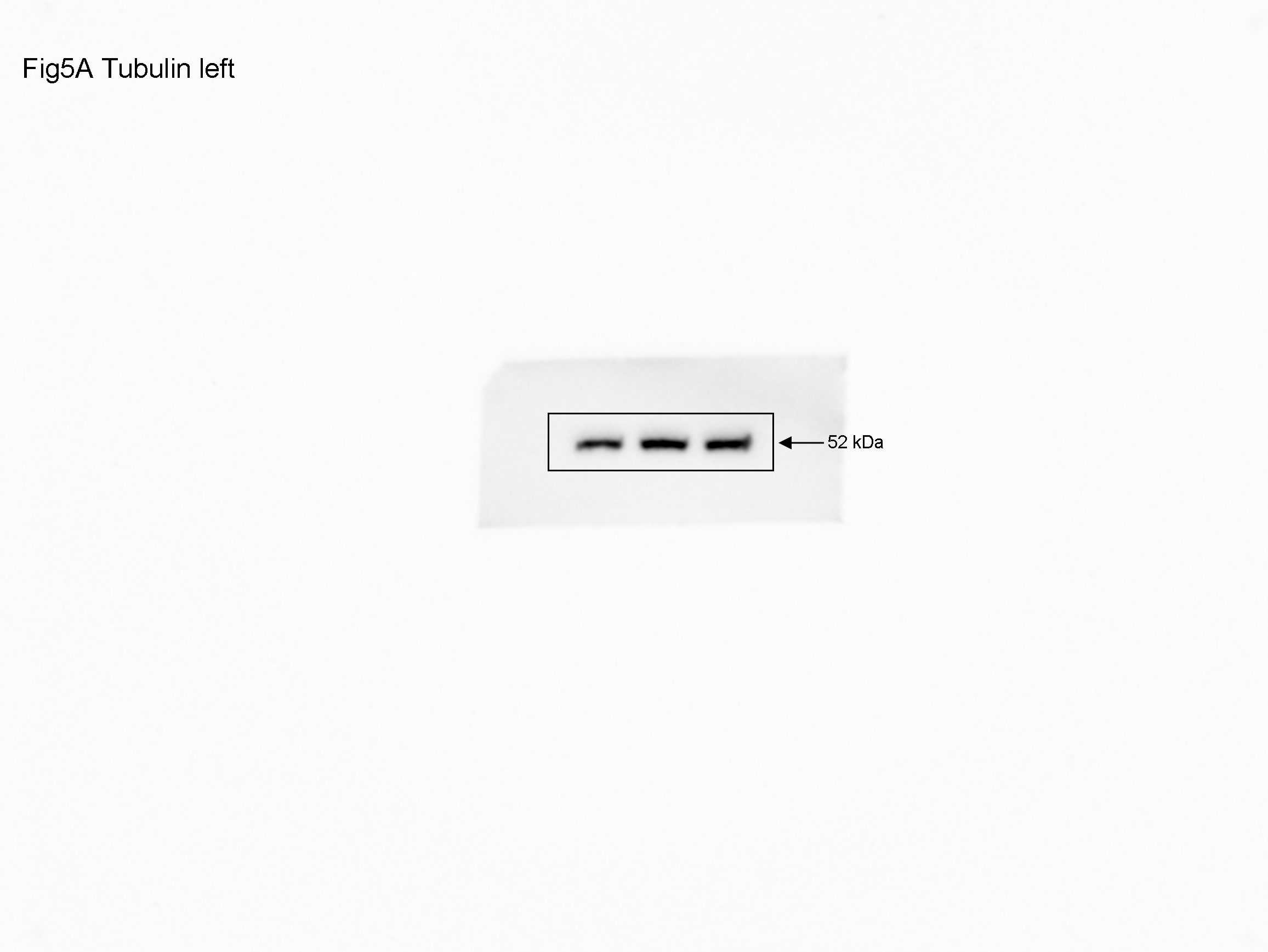

Supplement: Figure 5—source data 2. [file elife-98524-fig5-data2.zip › Fig 5-data2-v1/5A/left/Tubulin left.tif]

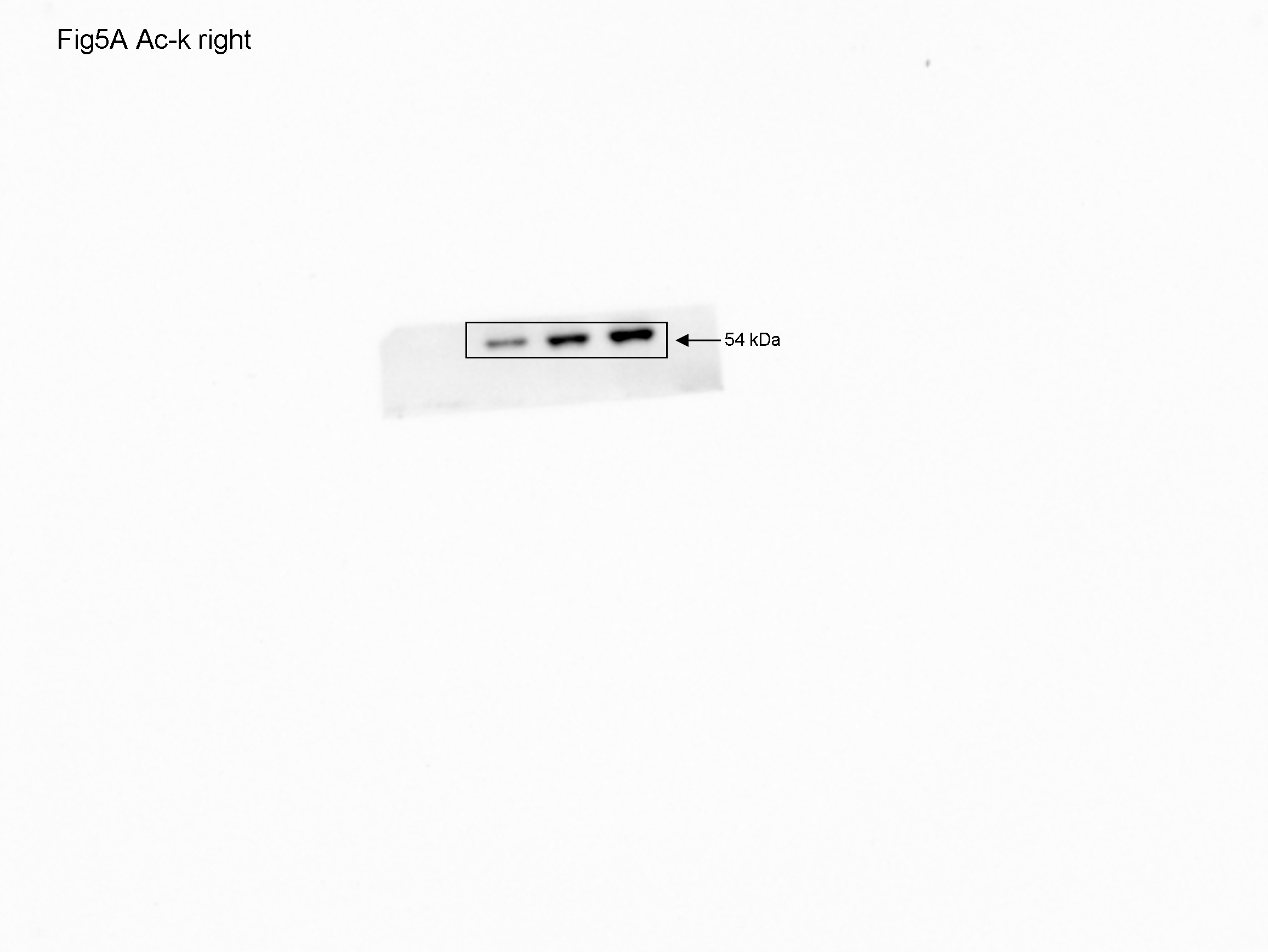

Supplement: Figure 5—source data 2. [file elife-98524-fig5-data2.zip › Fig 5-data2-v1/5A/right/Ac-k right.tif]

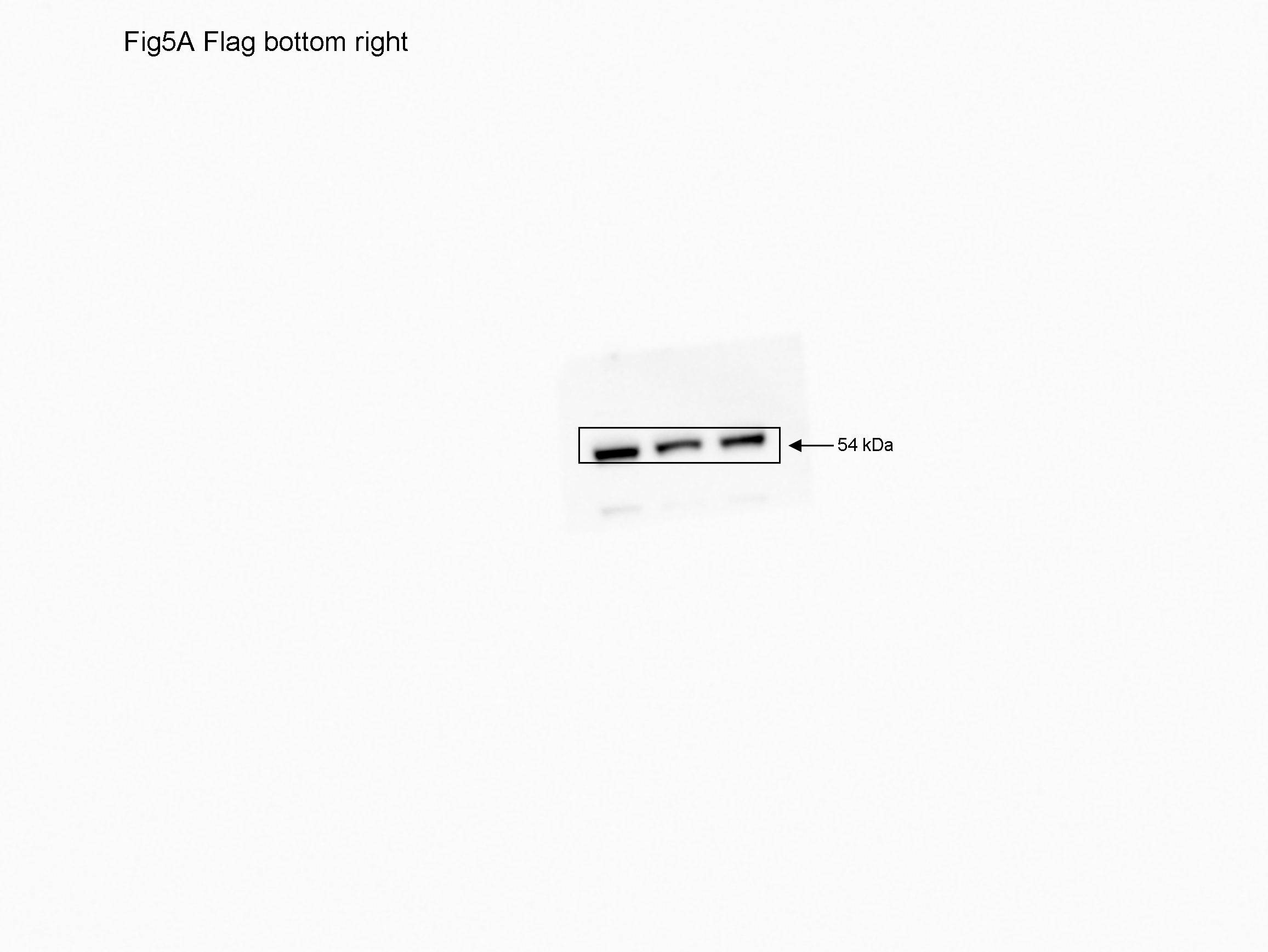

Supplement: Figure 5—source data 2. [file elife-98524-fig5-data2.zip › Fig 5-data2-v1/5A/right/Flag bottom right.tif]

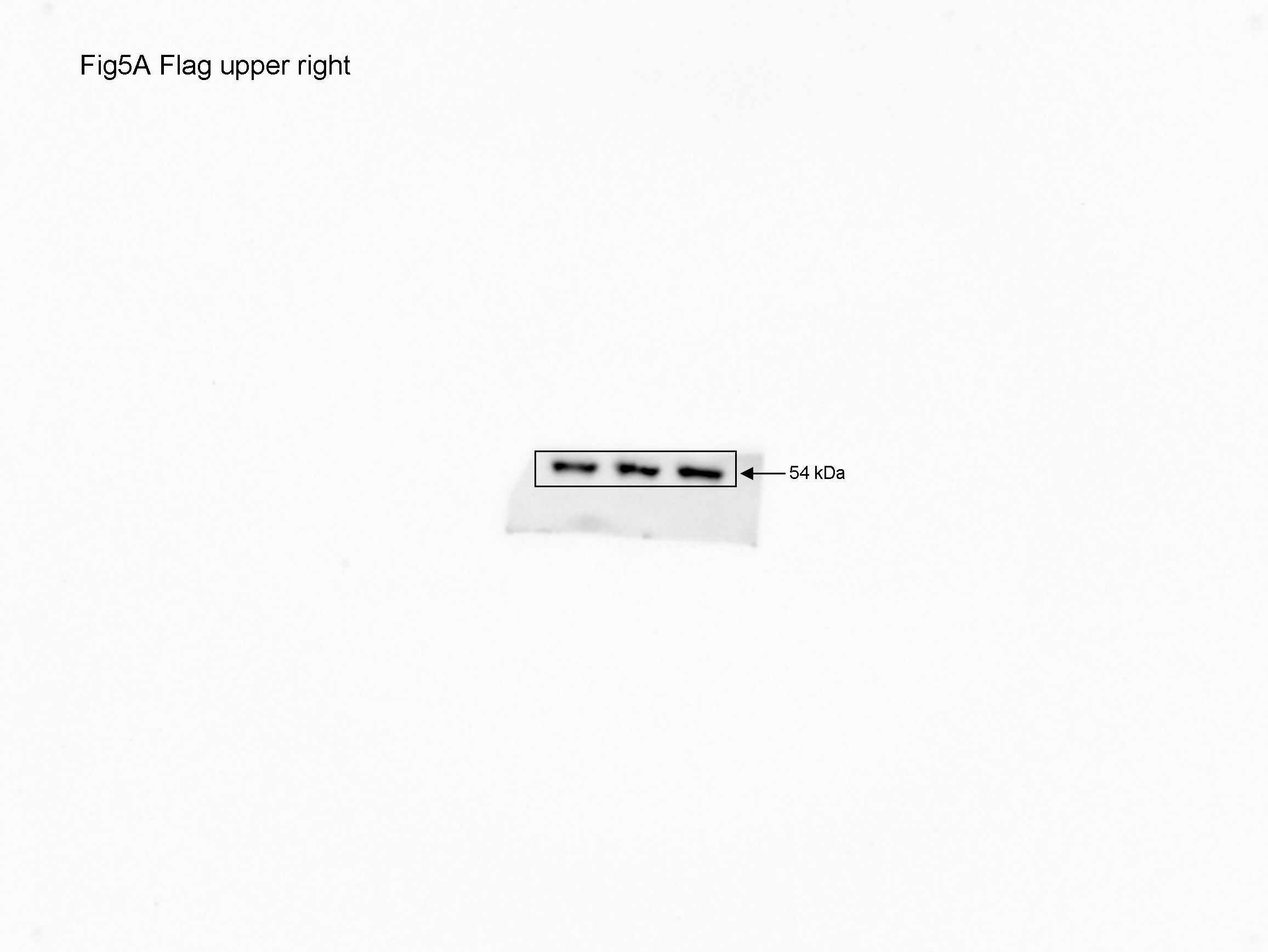

Supplement: Figure 5—source data 2. [file elife-98524-fig5-data2.zip › Fig 5-data2-v1/5A/right/Flag upper right.tif]

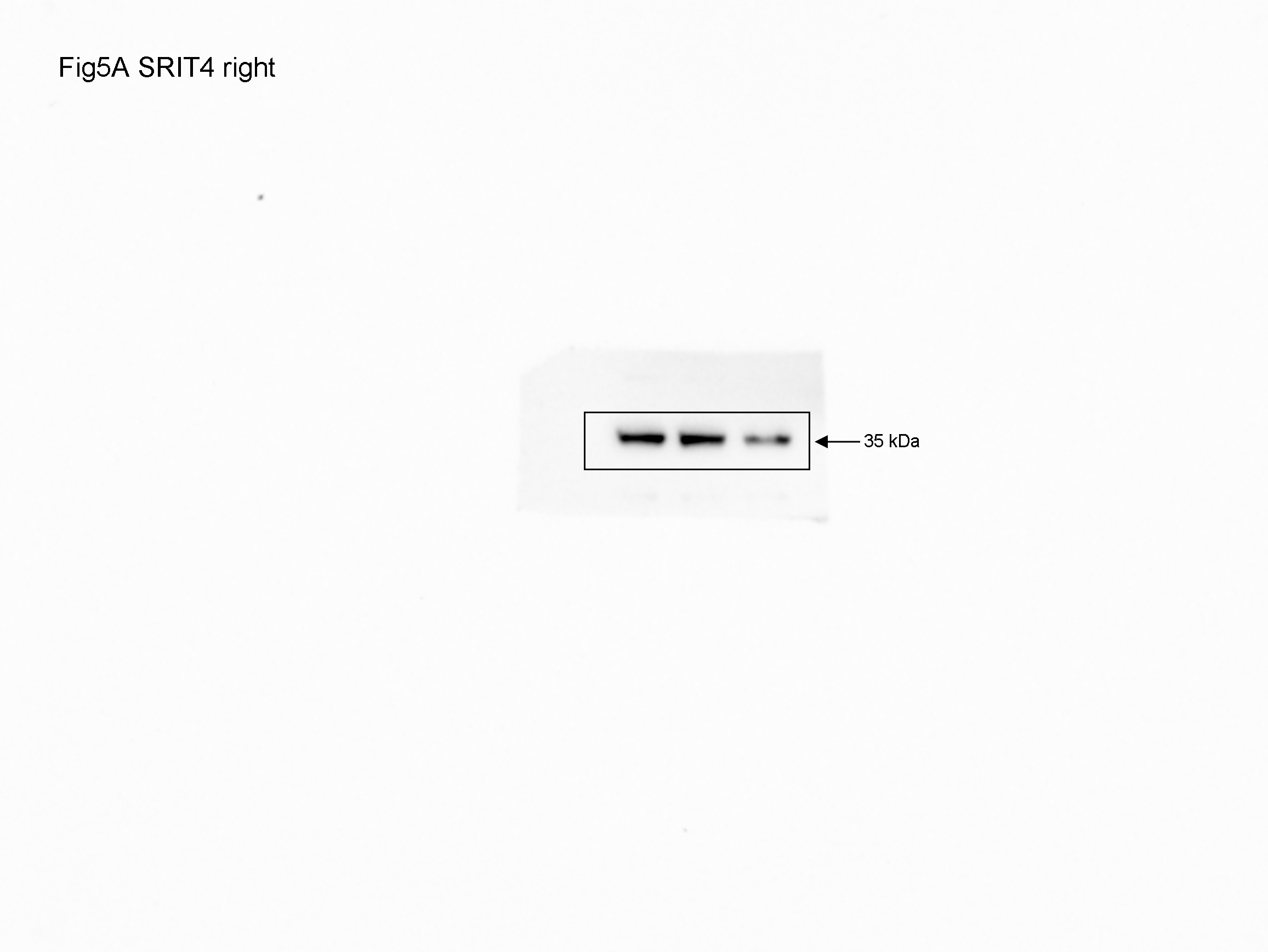

Supplement: Figure 5—source data 2. [file elife-98524-fig5-data2.zip › Fig 5-data2-v1/5A/right/SRIT4 right.tif]

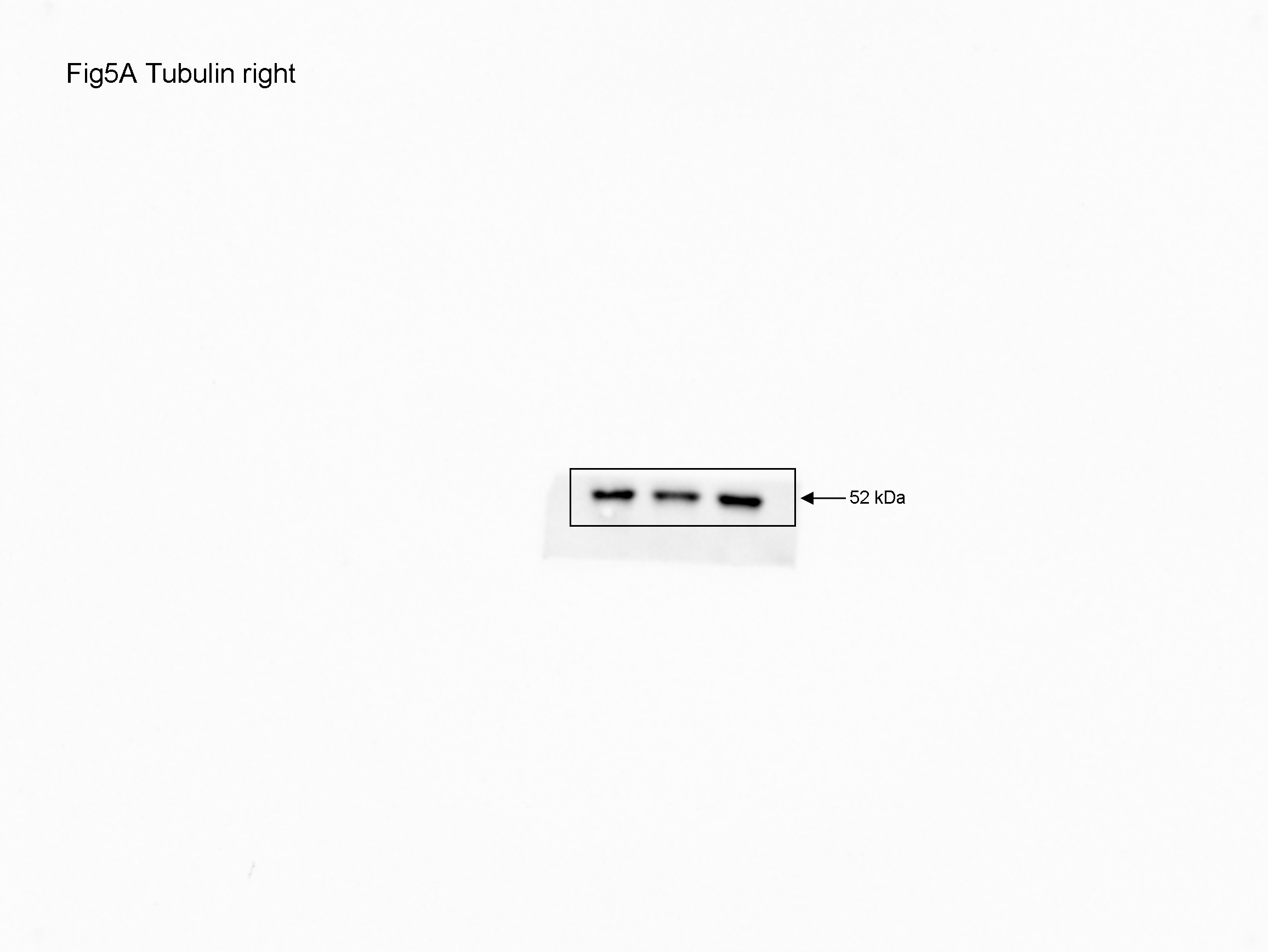

Supplement: Figure 5—source data 2. [file elife-98524-fig5-data2.zip › Fig 5-data2-v1/5A/right/Tubulin right.tif]

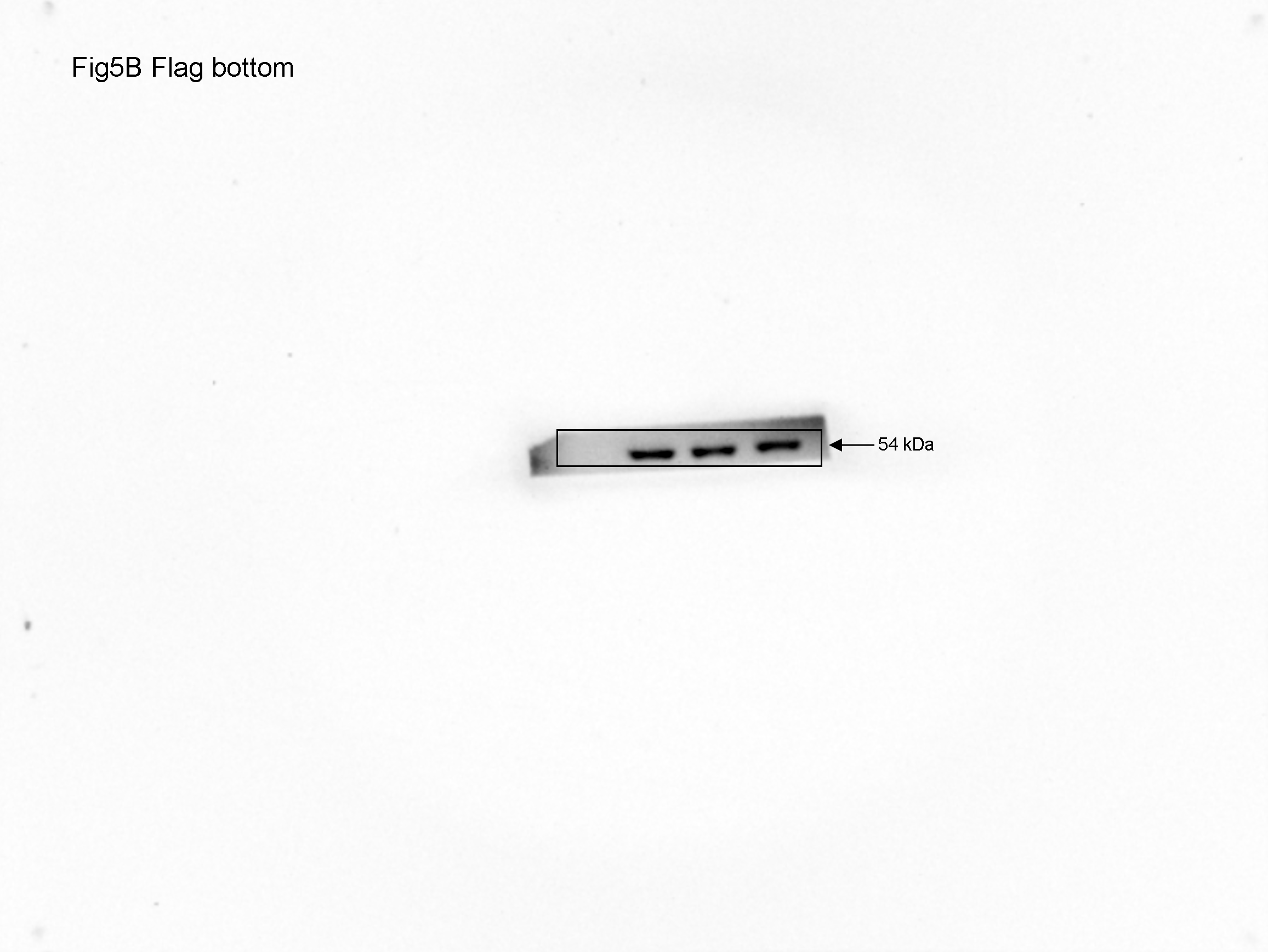

Supplement: Figure 5—source data 2. [file elife-98524-fig5-data2.zip › Fig 5-data2-v1/5B/bottom/Flag bottom.tif]

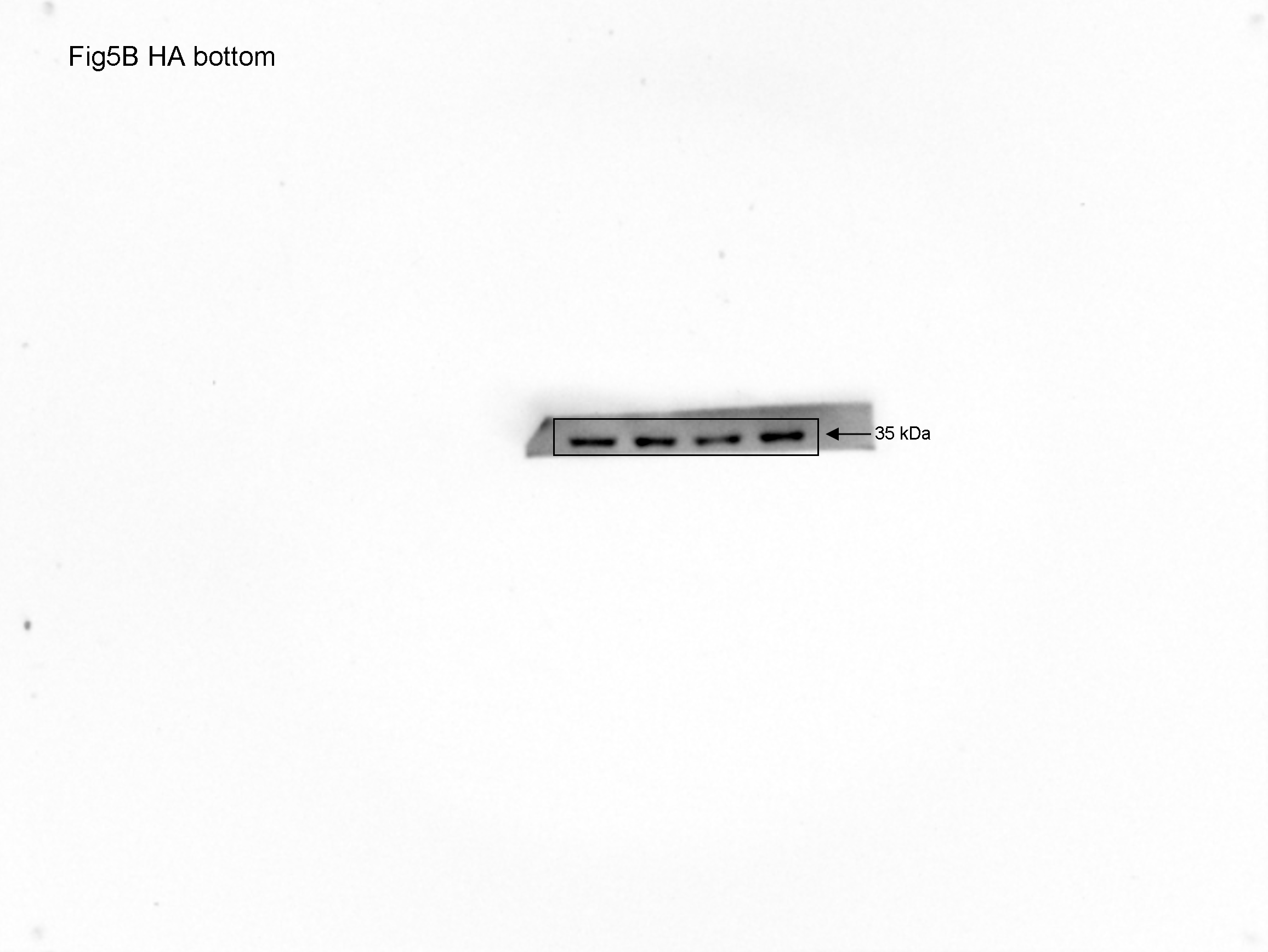

Supplement: Figure 5—source data 2. [file elife-98524-fig5-data2.zip › Fig 5-data2-v1/5B/bottom/HA bottom.tif]

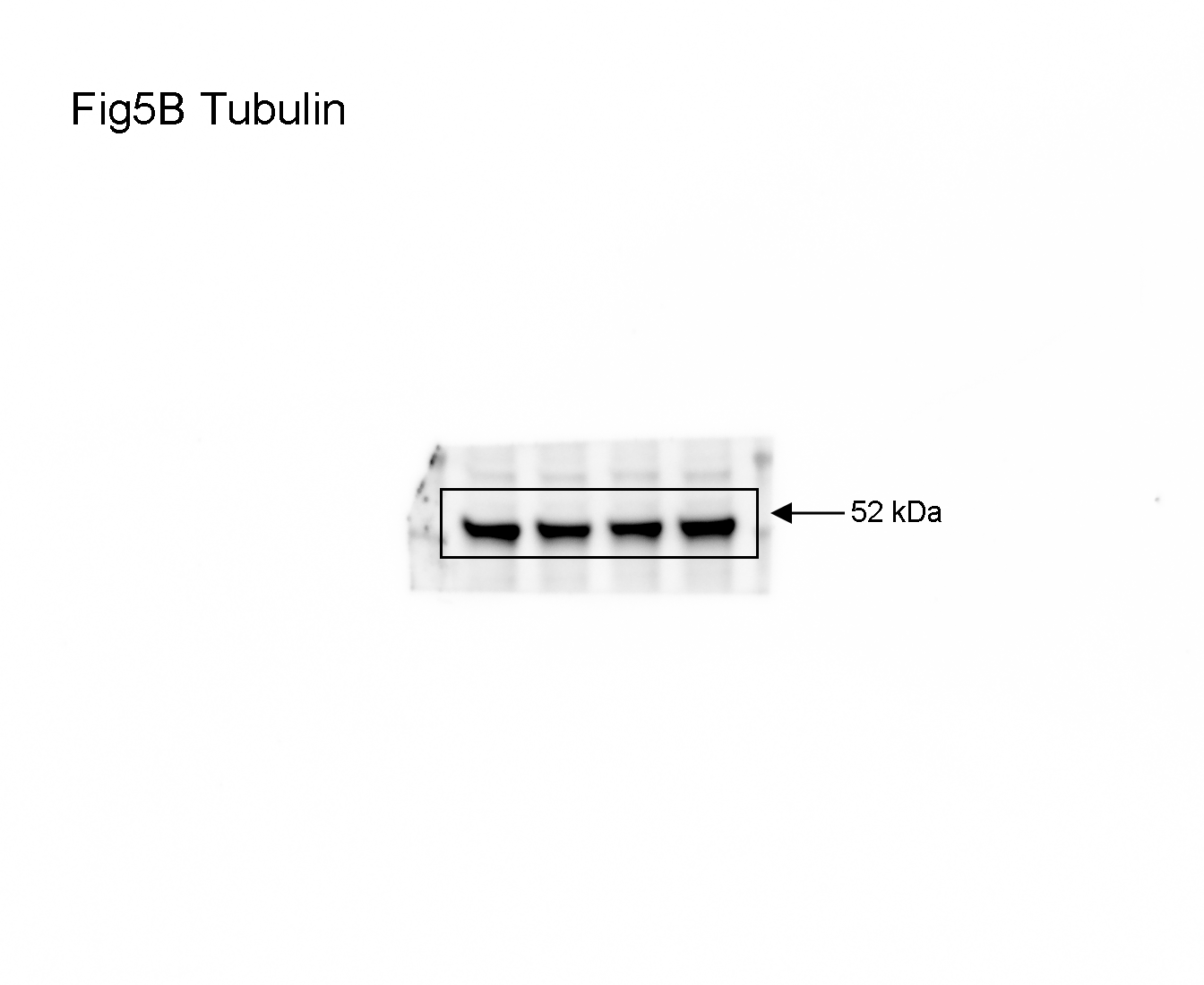

Supplement: Figure 5—source data 2. [file elife-98524-fig5-data2.zip › Fig 5-data2-v1/5B/bottom/Tubulin.tif]

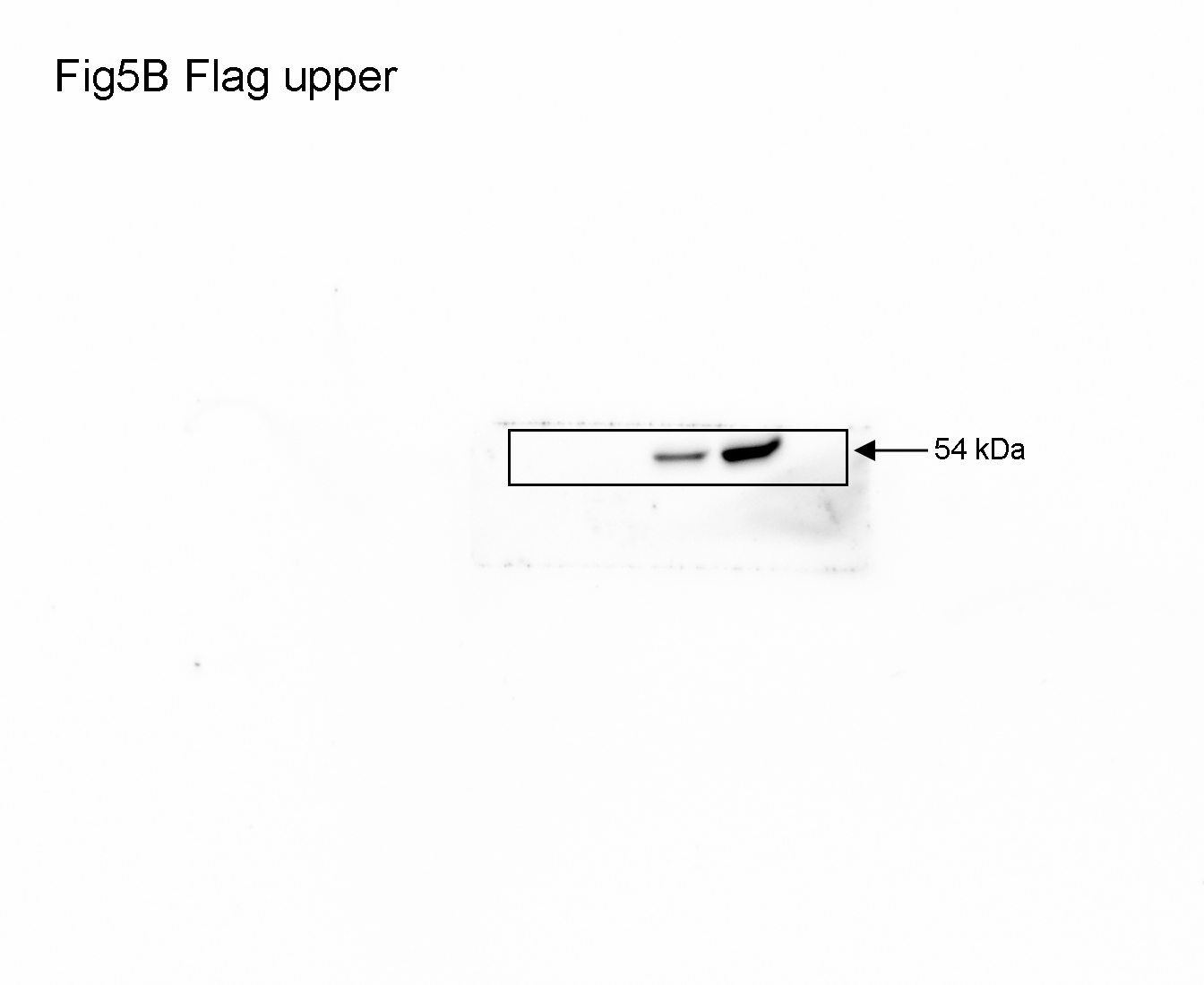

Supplement: Figure 5—source data 2. [file elife-98524-fig5-data2.zip › Fig 5-data2-v1/5B/upper/Flag upper.tif]

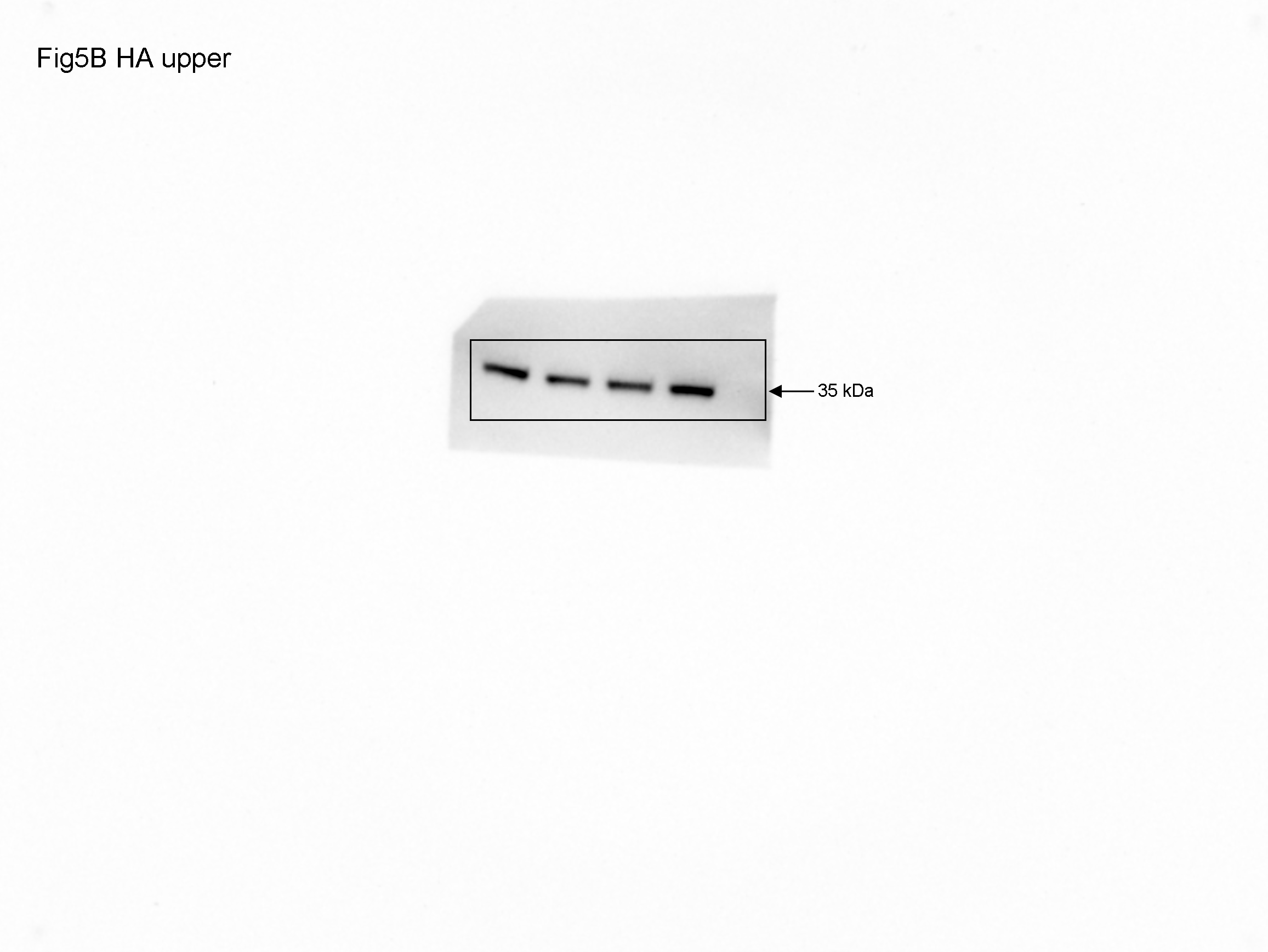

Supplement: Figure 5—source data 2. [file elife-98524-fig5-data2.zip › Fig 5-data2-v1/5B/upper/HA upper.tif]

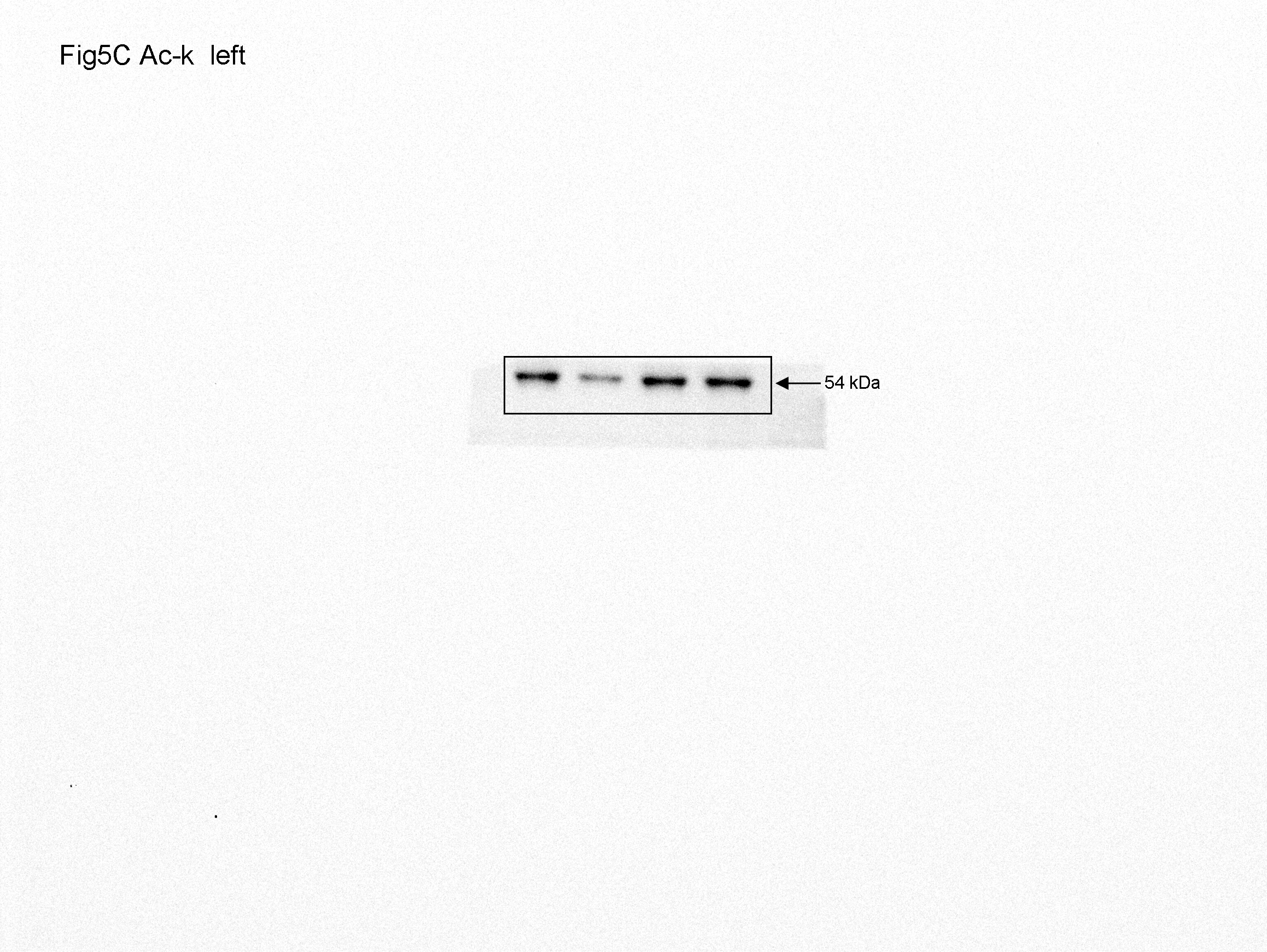

Supplement: Figure 5—source data 2. [file elife-98524-fig5-data2.zip › Fig 5-data2-v1/5C/left/Ac-k left.tif]

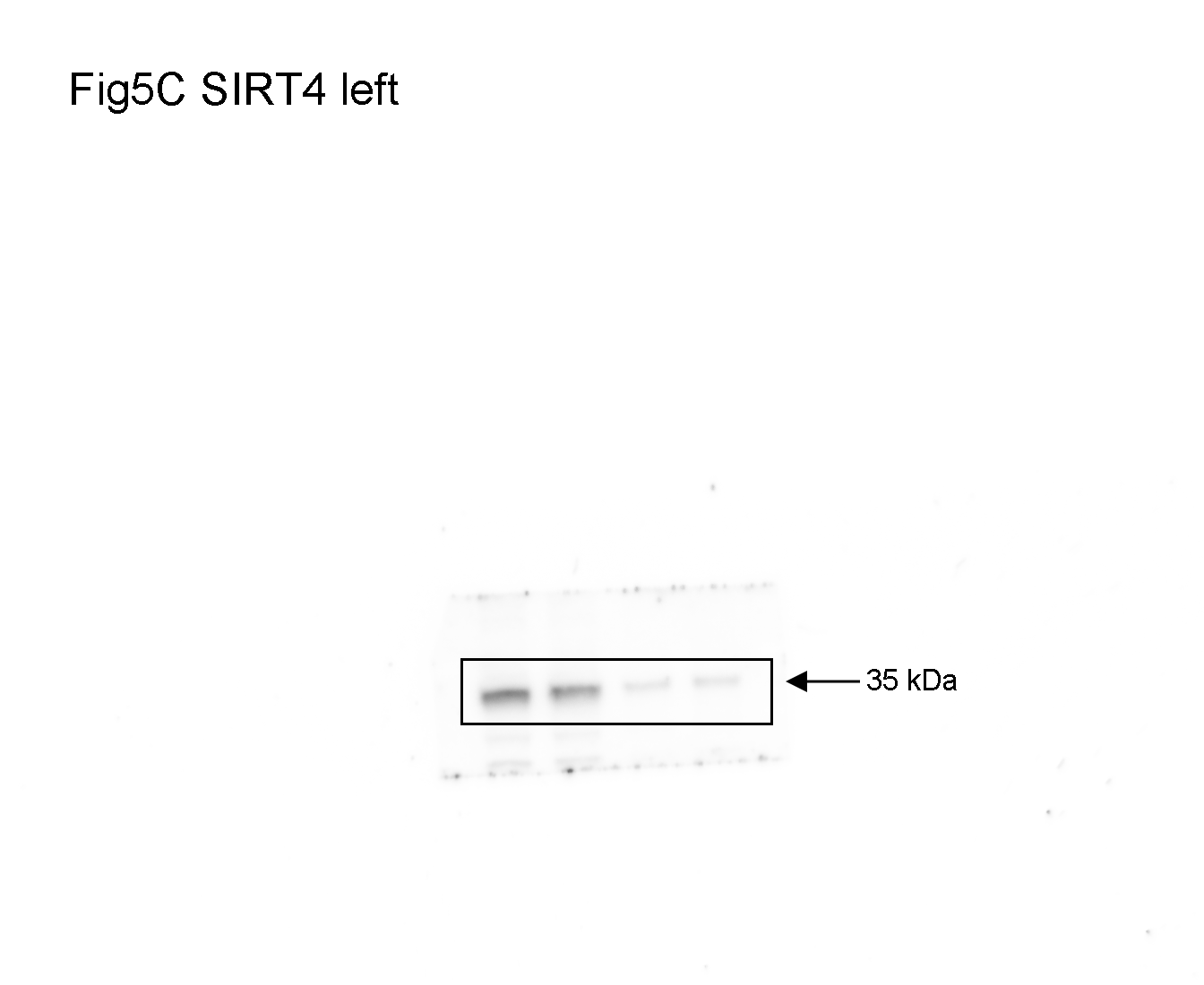

Supplement: Figure 5—source data 2. [file elife-98524-fig5-data2.zip › Fig 5-data2-v1/5C/left/SIRT4 left.tif]

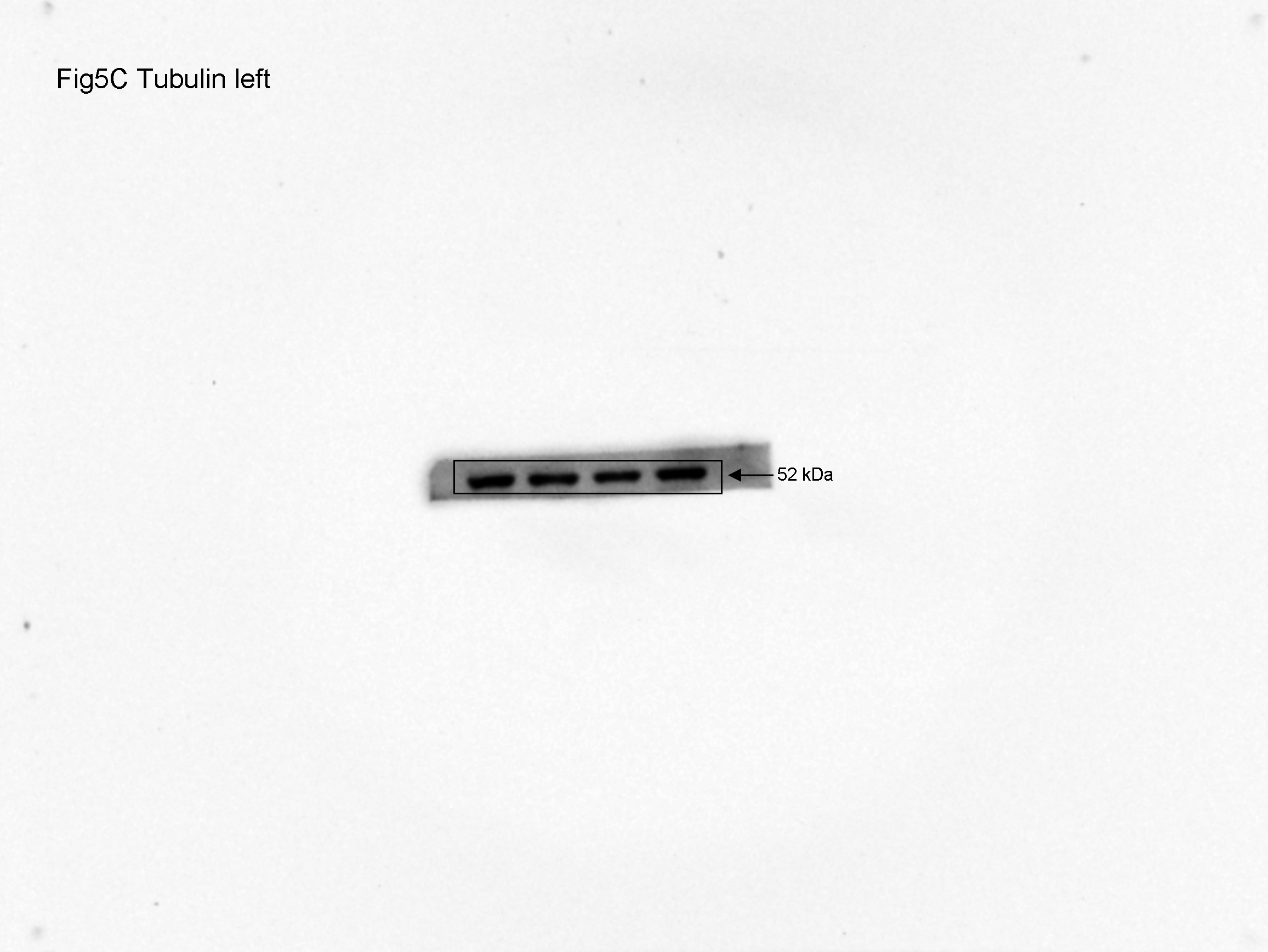

Supplement: Figure 5—source data 2. [file elife-98524-fig5-data2.zip › Fig 5-data2-v1/5C/left/Tubulin left.tif]

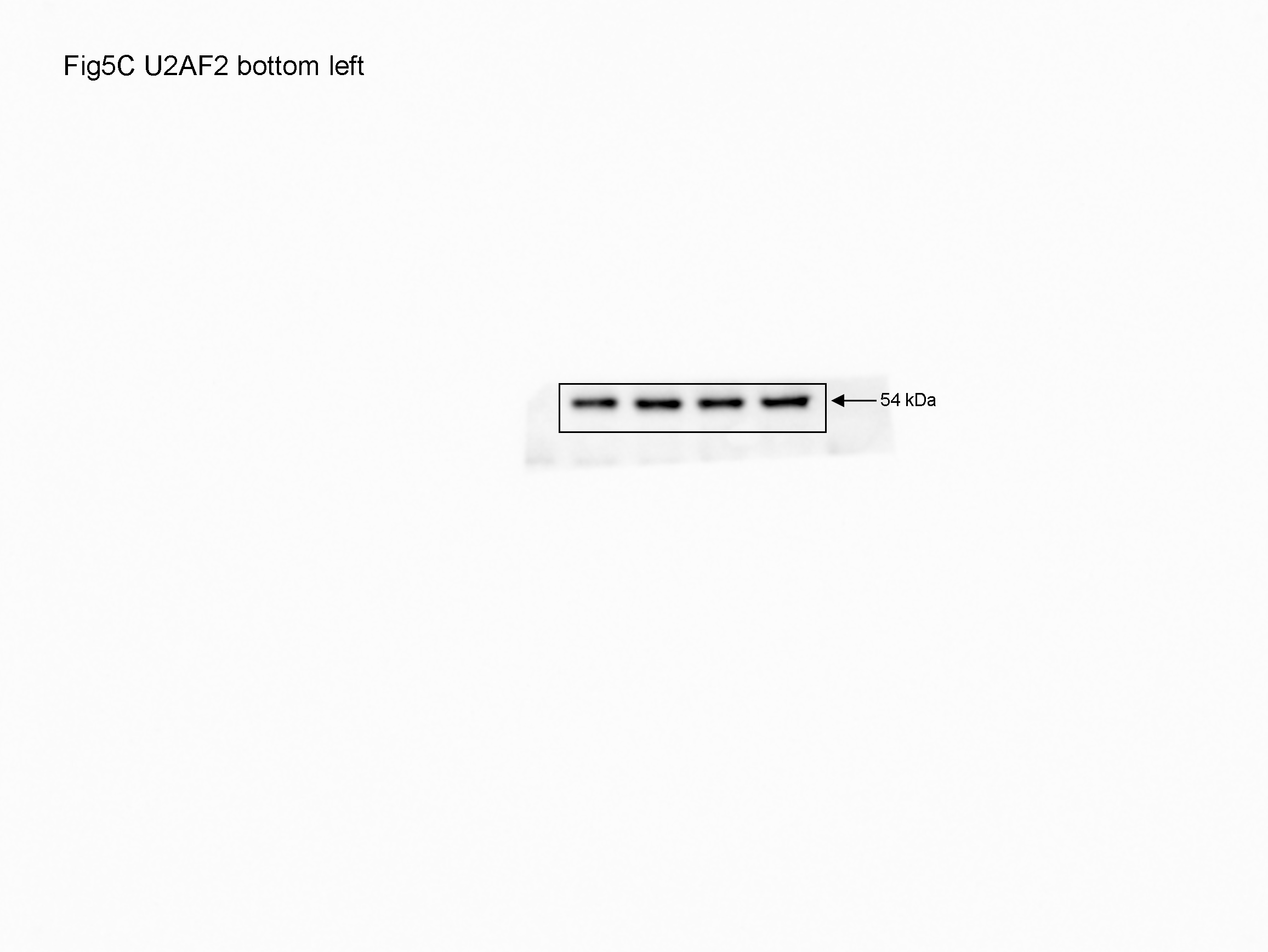

Supplement: Figure 5—source data 2. [file elife-98524-fig5-data2.zip › Fig 5-data2-v1/5C/left/U2AF2 bottom left.tif]

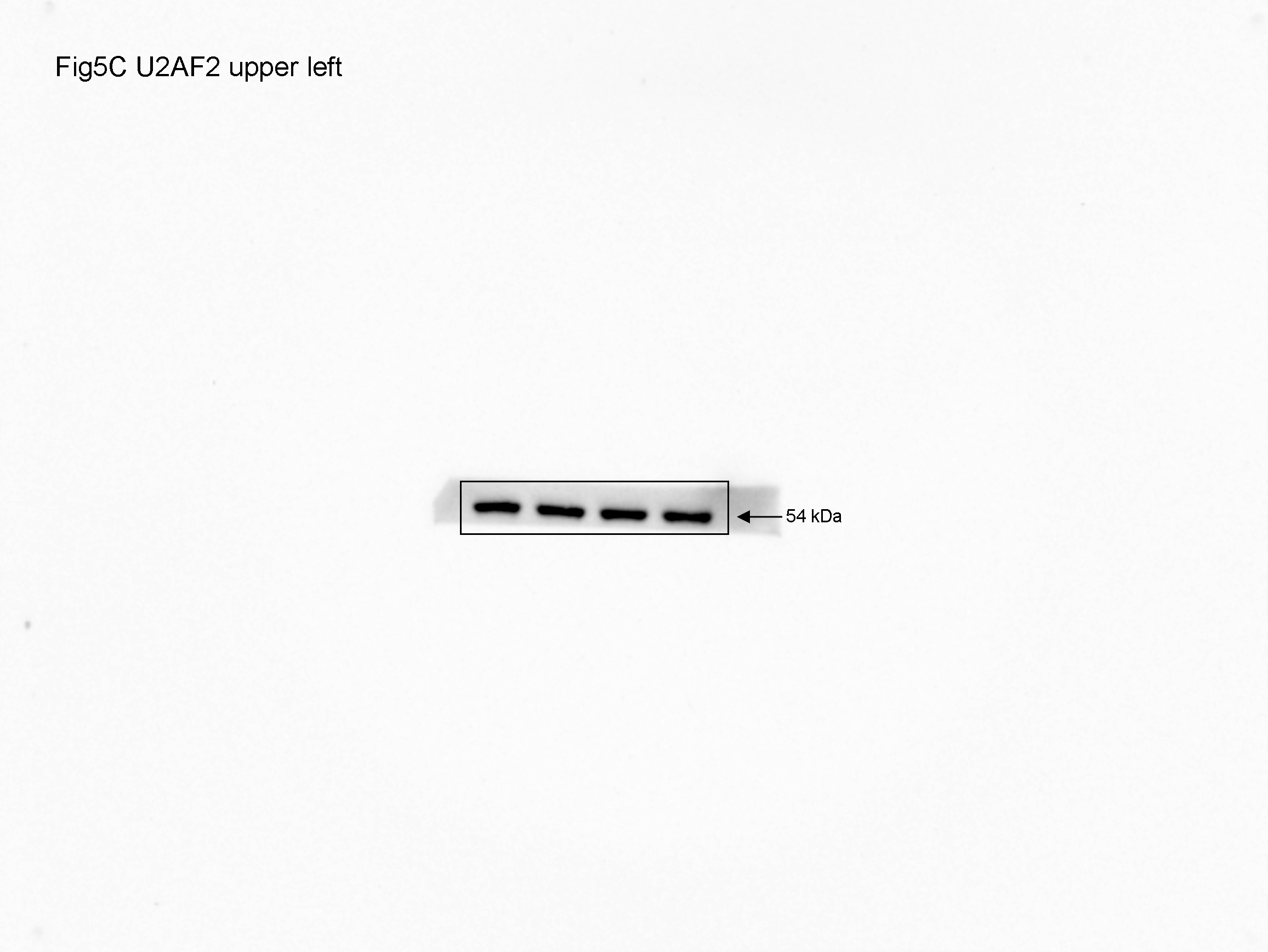

Supplement: Figure 5—source data 2. [file elife-98524-fig5-data2.zip › Fig 5-data2-v1/5C/left/U2AF2 upper left.tif]

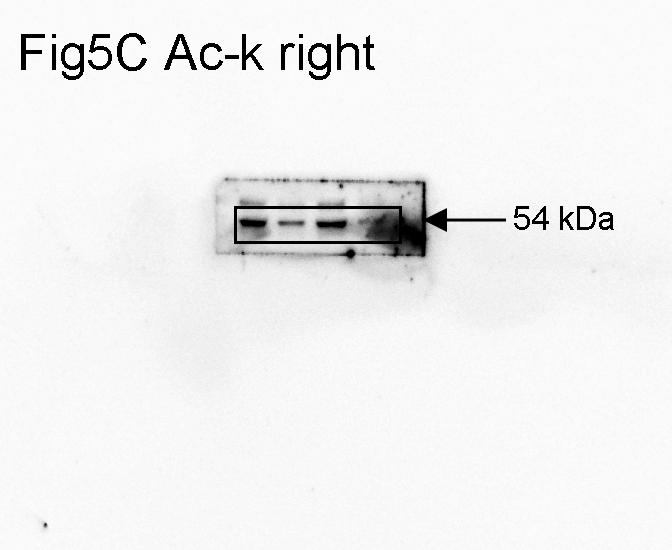

Supplement: Figure 5—source data 2. [file elife-98524-fig5-data2.zip › Fig 5-data2-v1/5C/right/Ac-k right.tif]

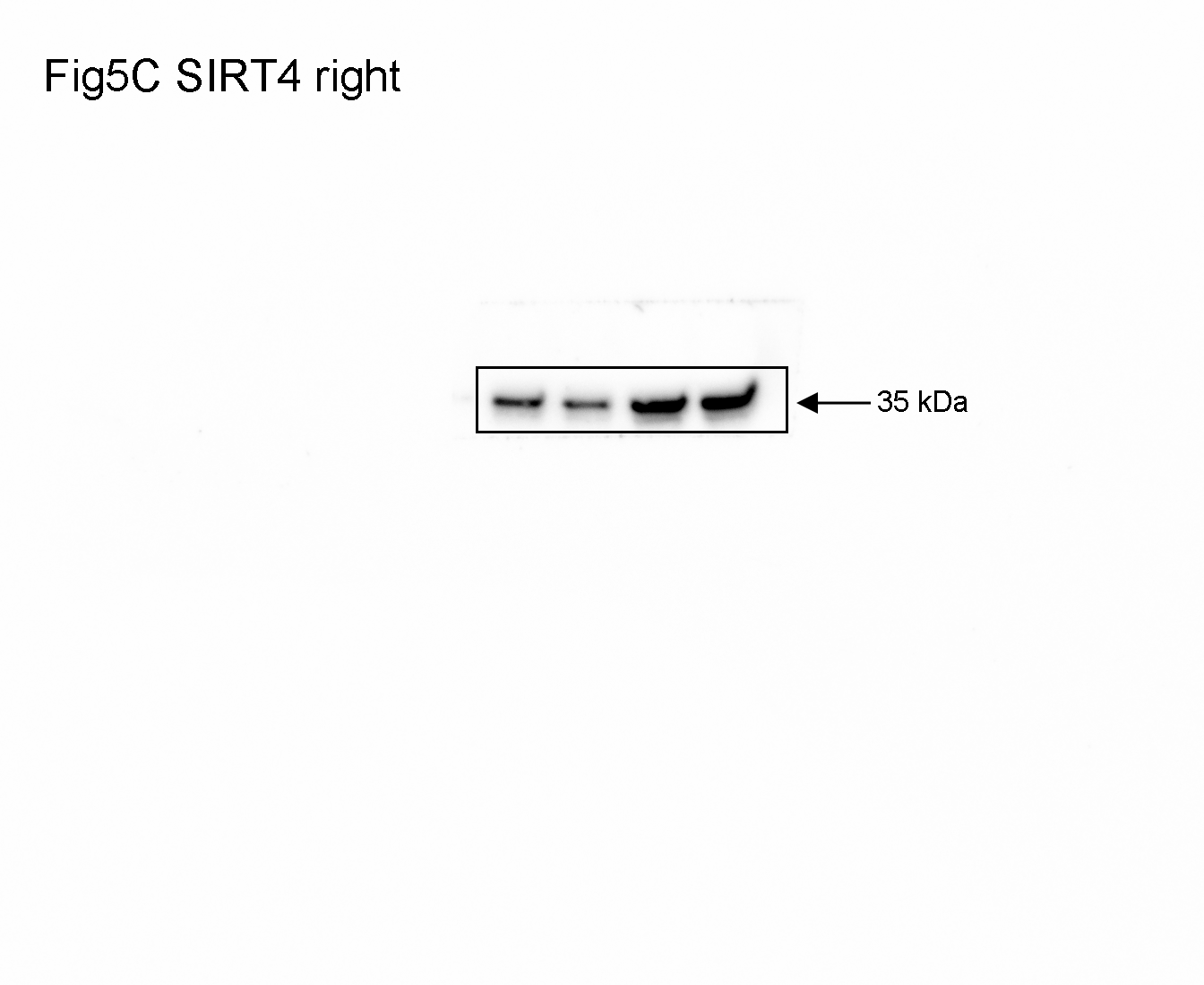

Supplement: Figure 5—source data 2. [file elife-98524-fig5-data2.zip › Fig 5-data2-v1/5C/right/SIRT4 right.tif]

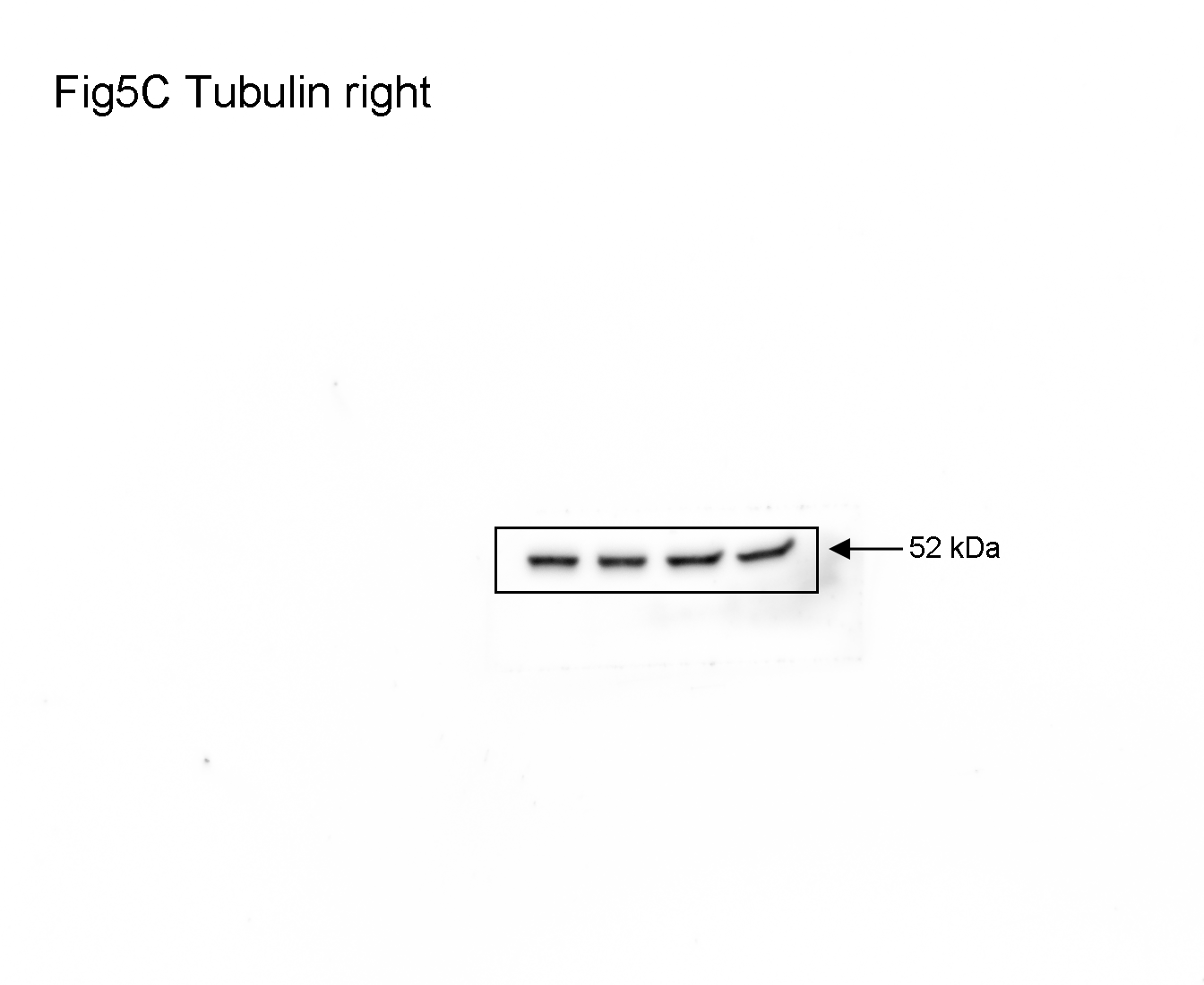

Supplement: Figure 5—source data 2. [file elife-98524-fig5-data2.zip › Fig 5-data2-v1/5C/right/Tubulin right.tif]

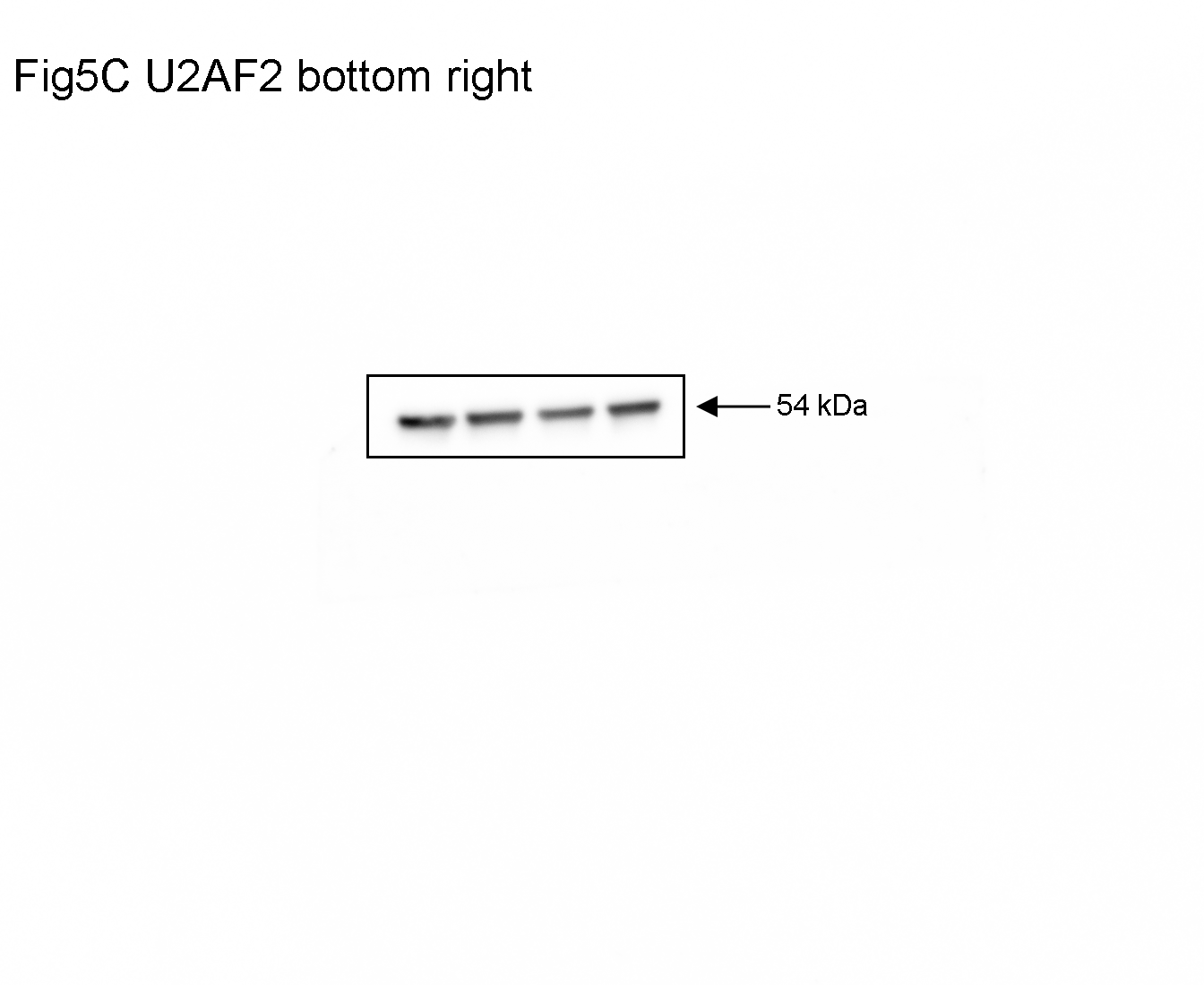

Supplement: Figure 5—source data 2. [file elife-98524-fig5-data2.zip › Fig 5-data2-v1/5C/right/U2AF2 bottom right.tif]

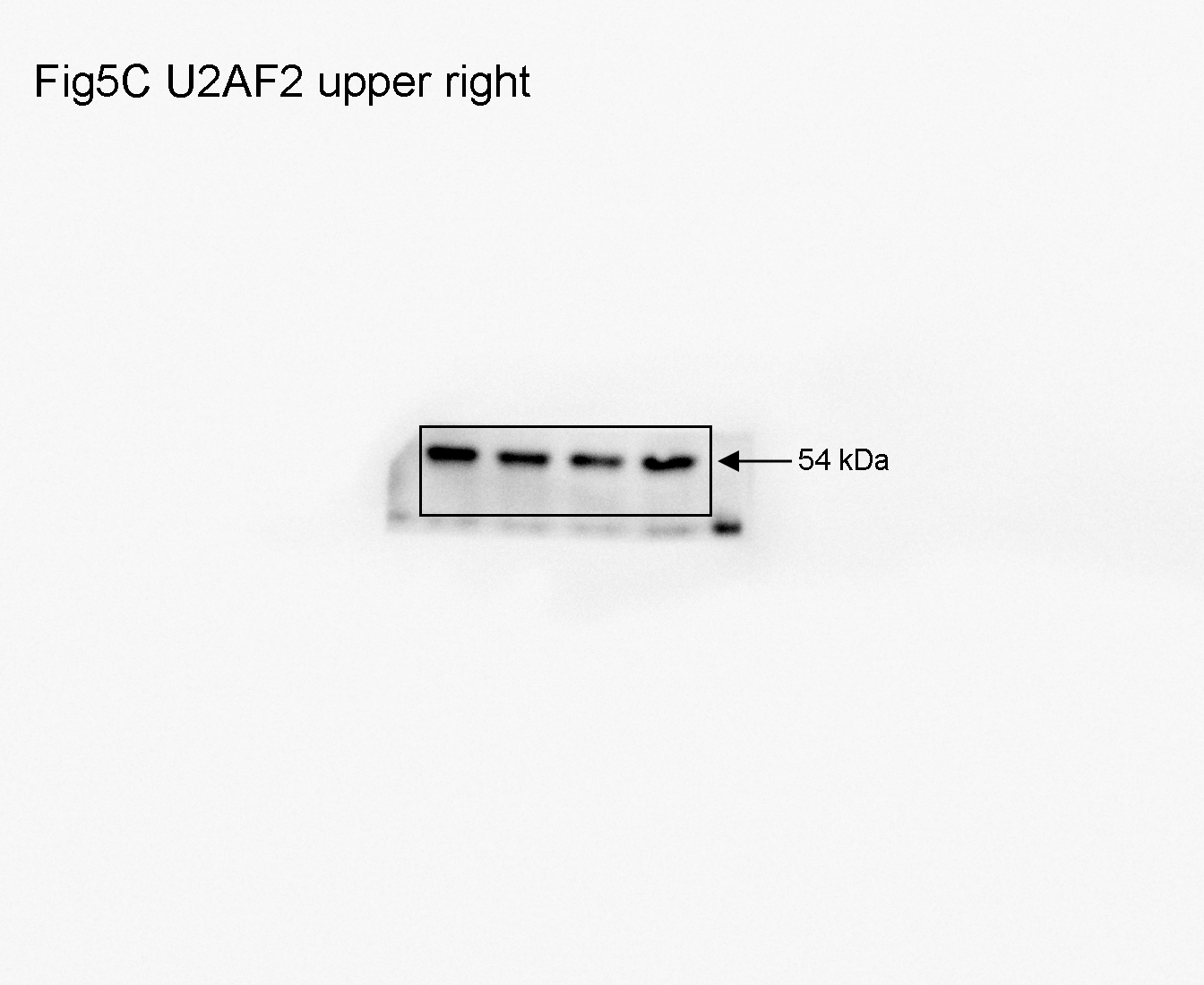

Supplement: Figure 5—source data 2. [file elife-98524-fig5-data2.zip › Fig 5-data2-v1/5C/right/U2AF2 upper right.tif]

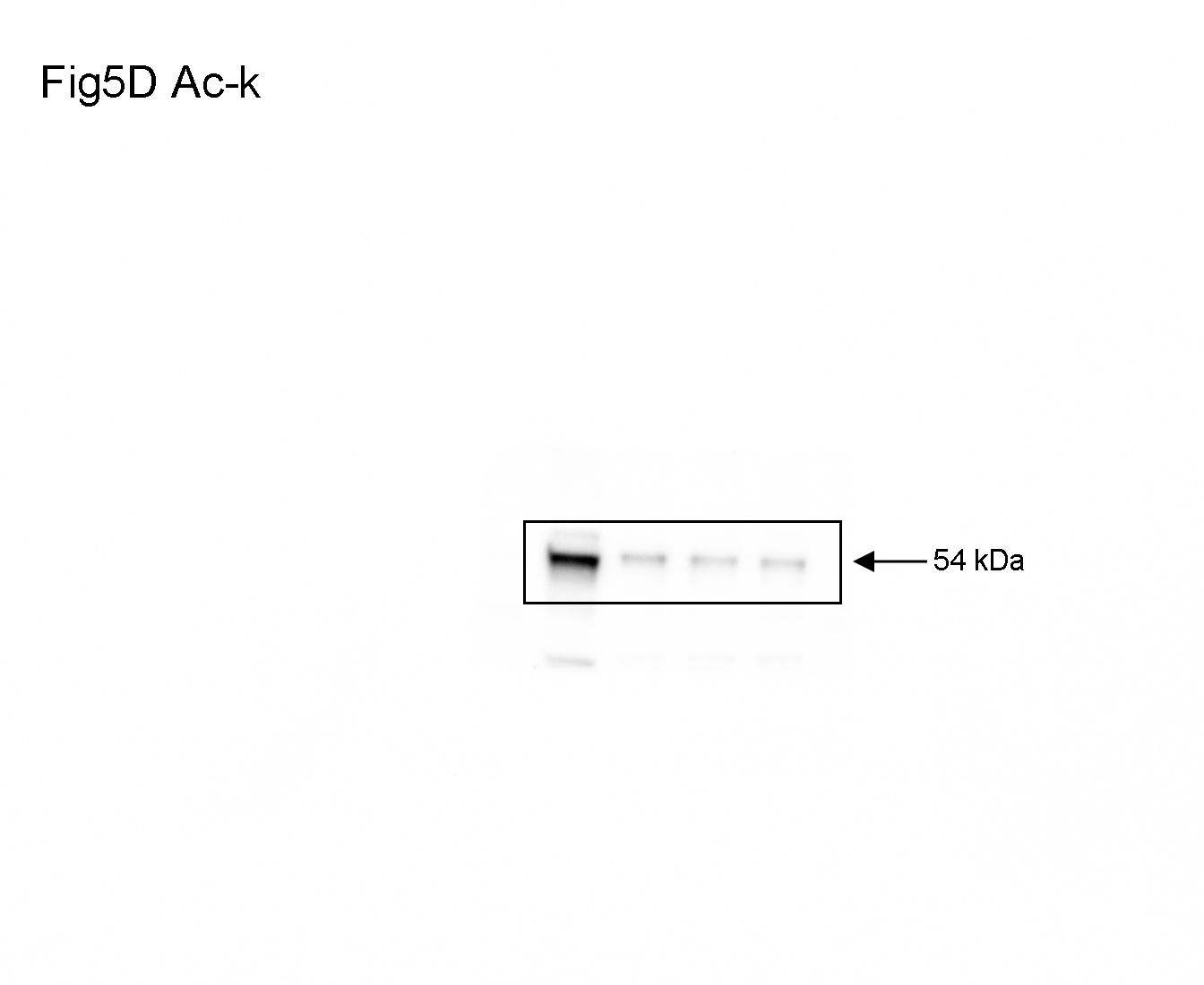

Supplement: Figure 5—source data 2. [file elife-98524-fig5-data2.zip › Fig 5-data2-v1/5D/left/Ac-k.tif]

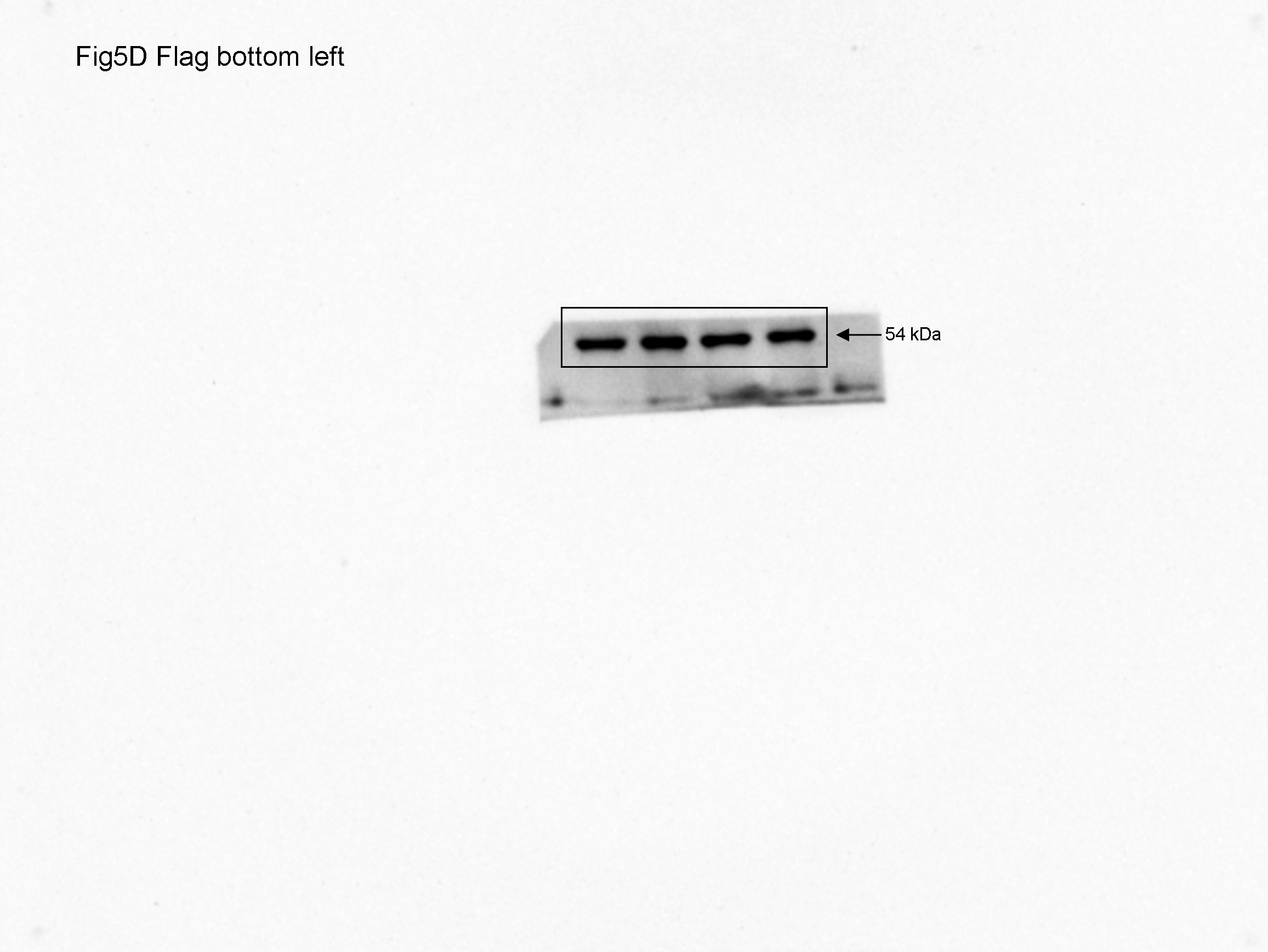

Supplement: Figure 5—source data 2. [file elife-98524-fig5-data2.zip › Fig 5-data2-v1/5D/left/Flag bottom left.tif]

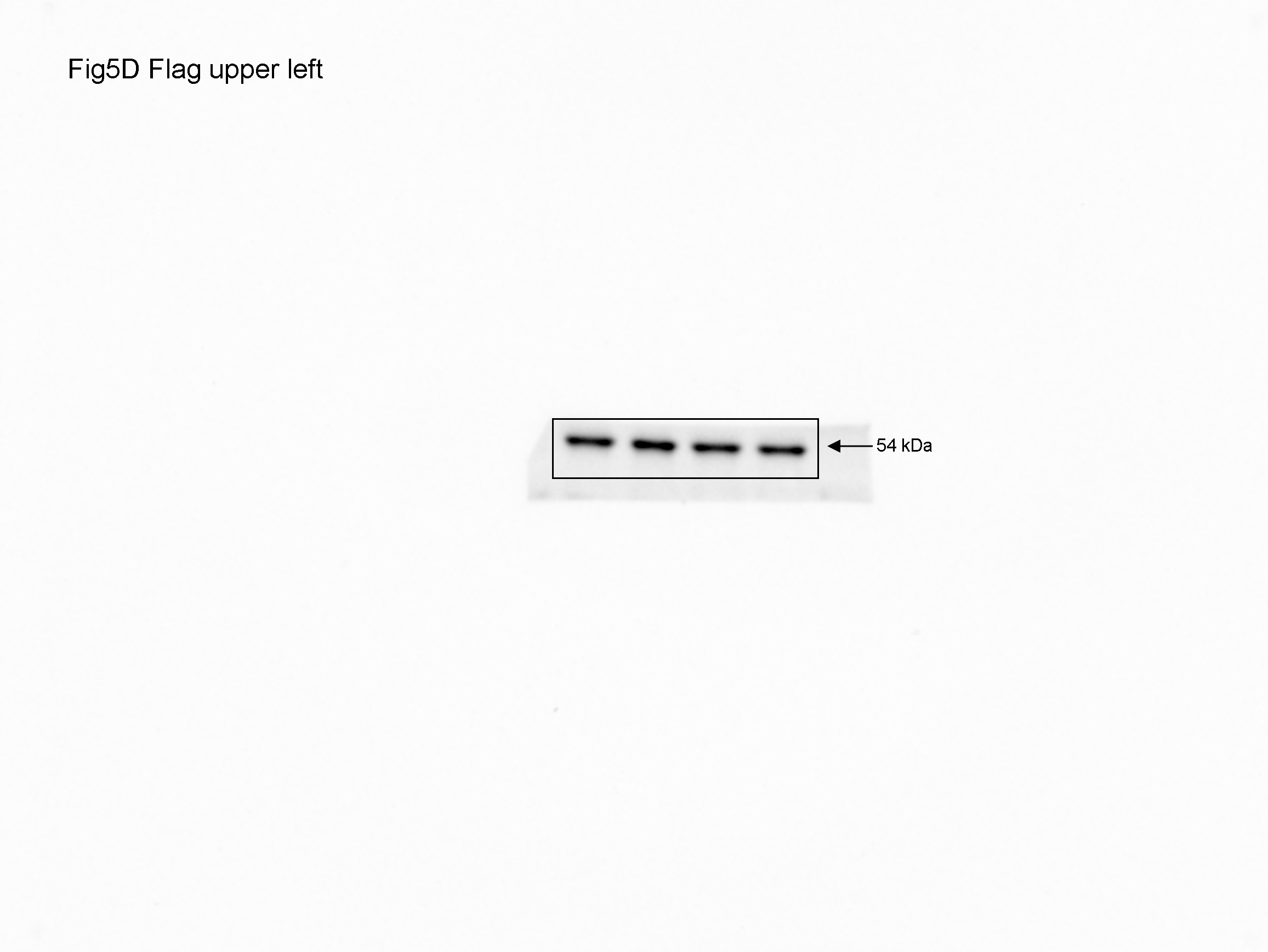

Supplement: Figure 5—source data 2. [file elife-98524-fig5-data2.zip › Fig 5-data2-v1/5D/left/Flag upper left.tif]

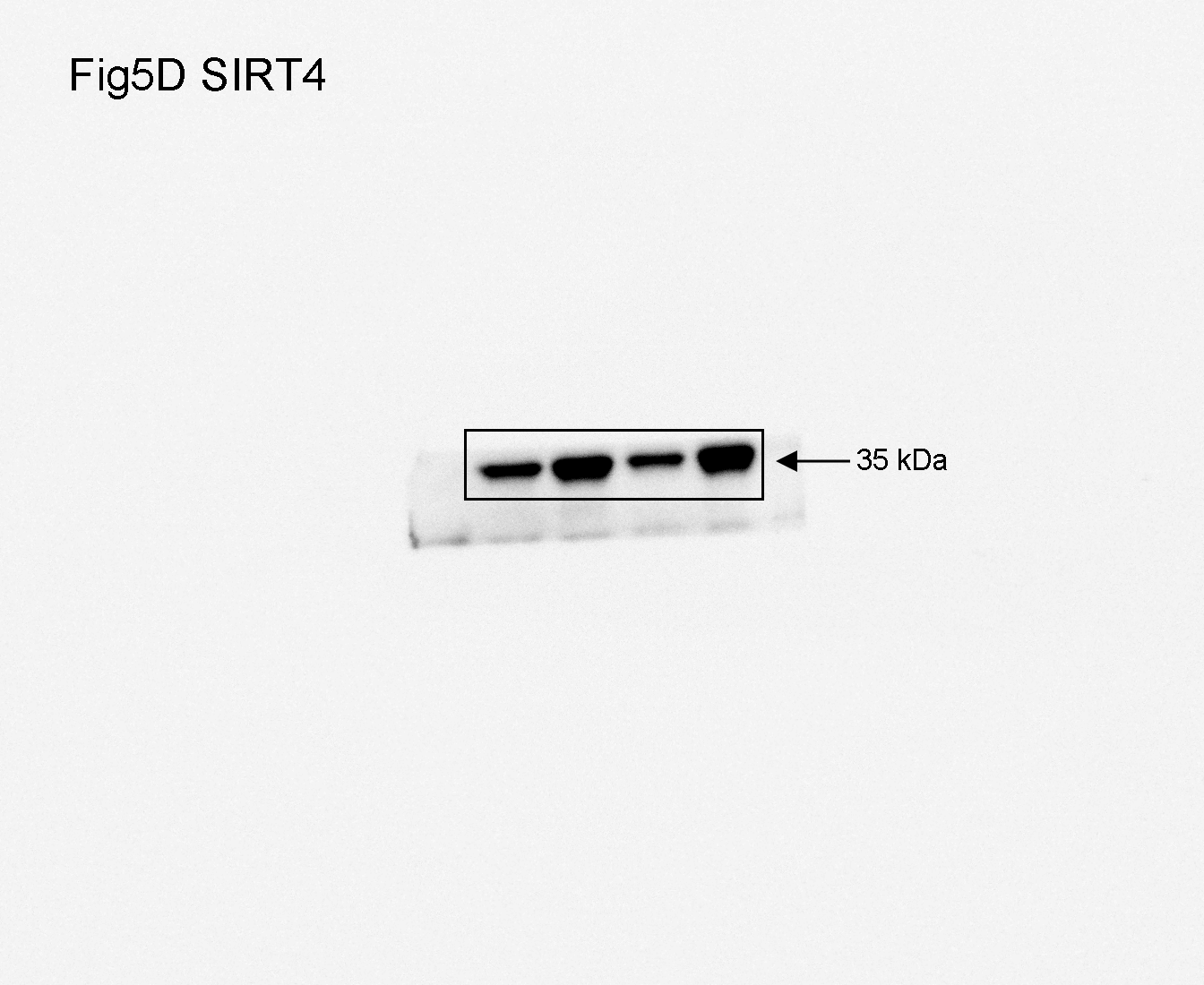

Supplement: Figure 5—source data 2. [file elife-98524-fig5-data2.zip › Fig 5-data2-v1/5D/left/SIRT4.tif]

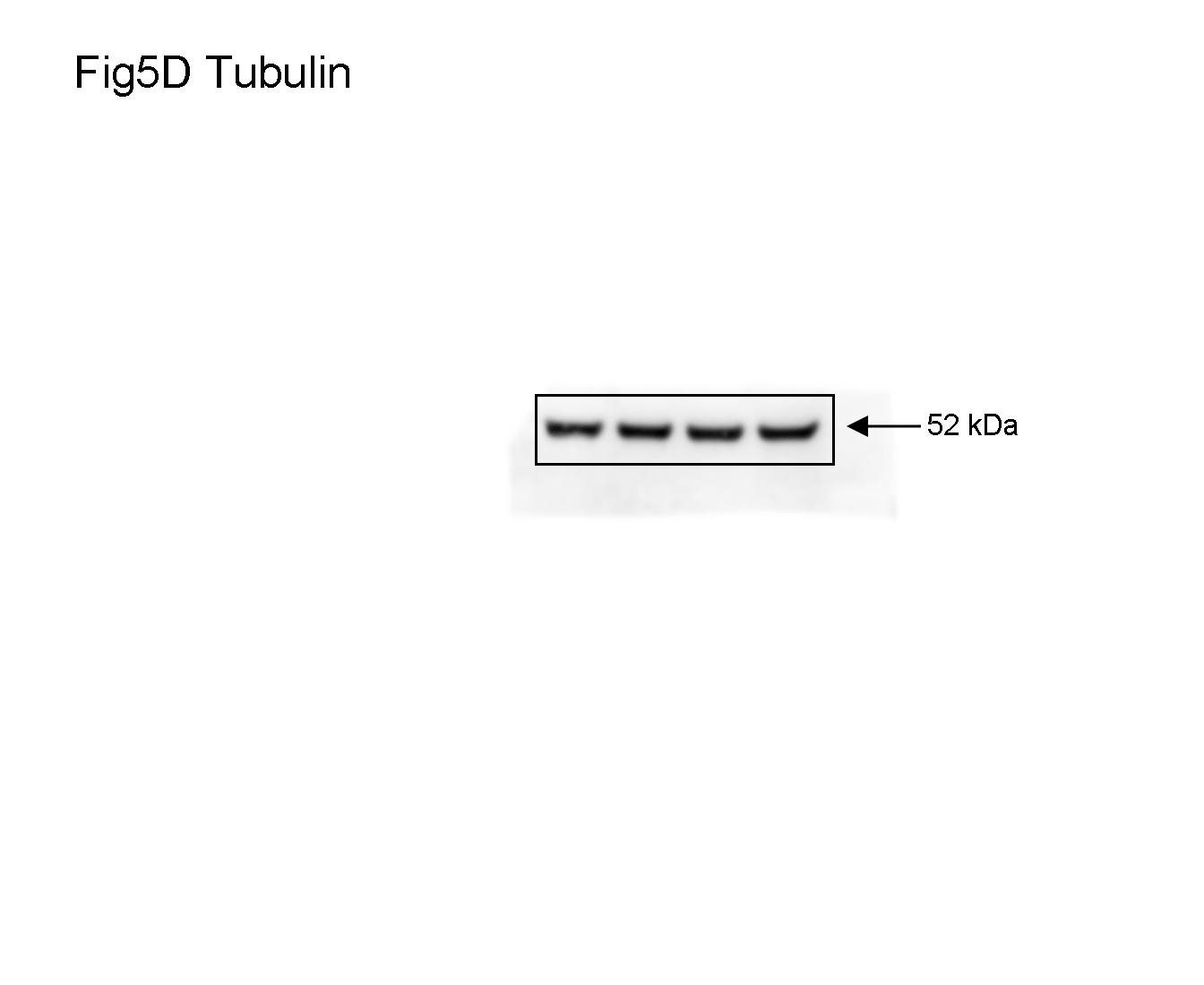

Supplement: Figure 5—source data 2. [file elife-98524-fig5-data2.zip › Fig 5-data2-v1/5D/left/Tubulin.tif]

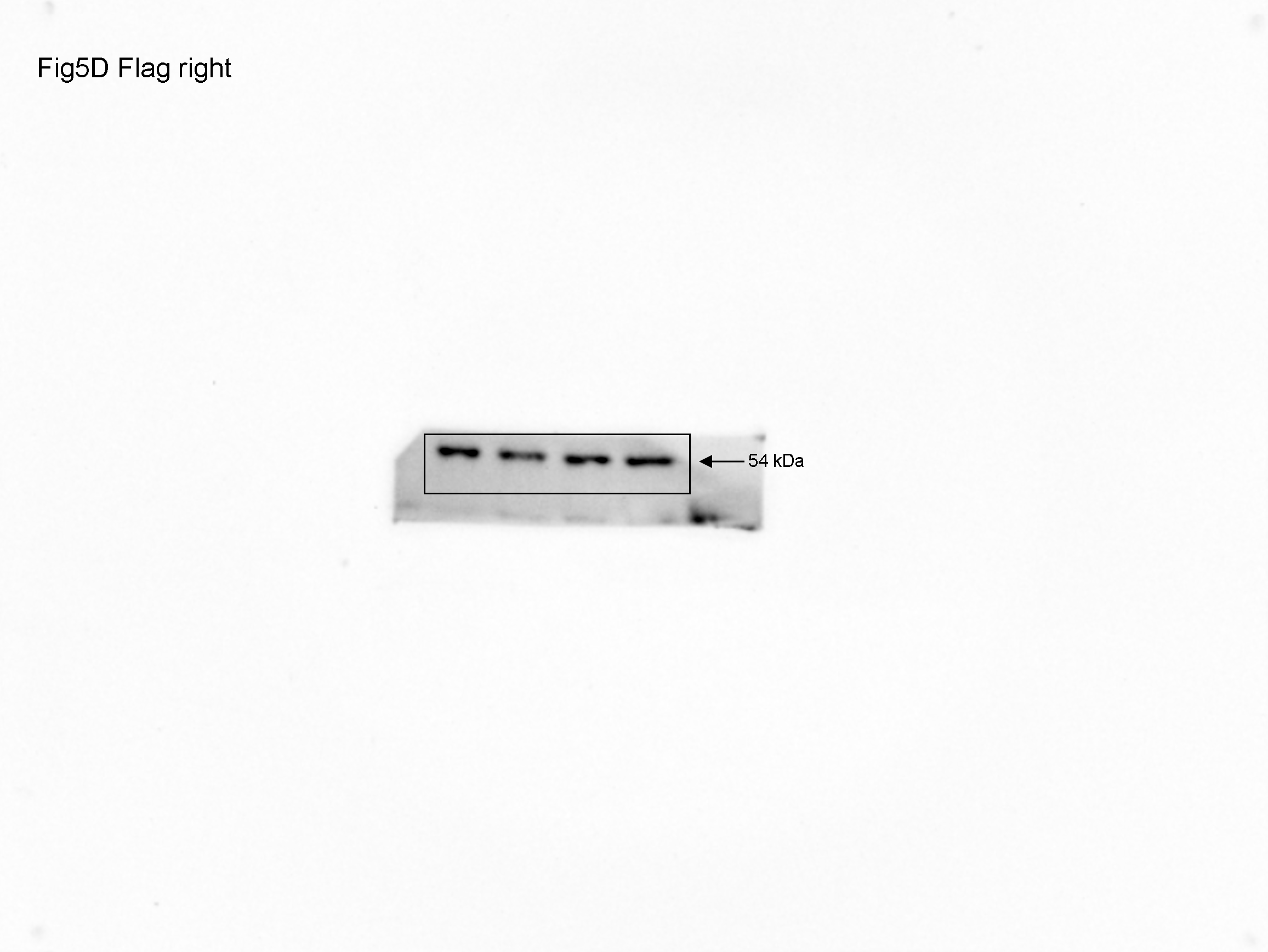

Supplement: Figure 5—source data 2. [file elife-98524-fig5-data2.zip › Fig 5-data2-v1/5D/right/Flag right.tif]

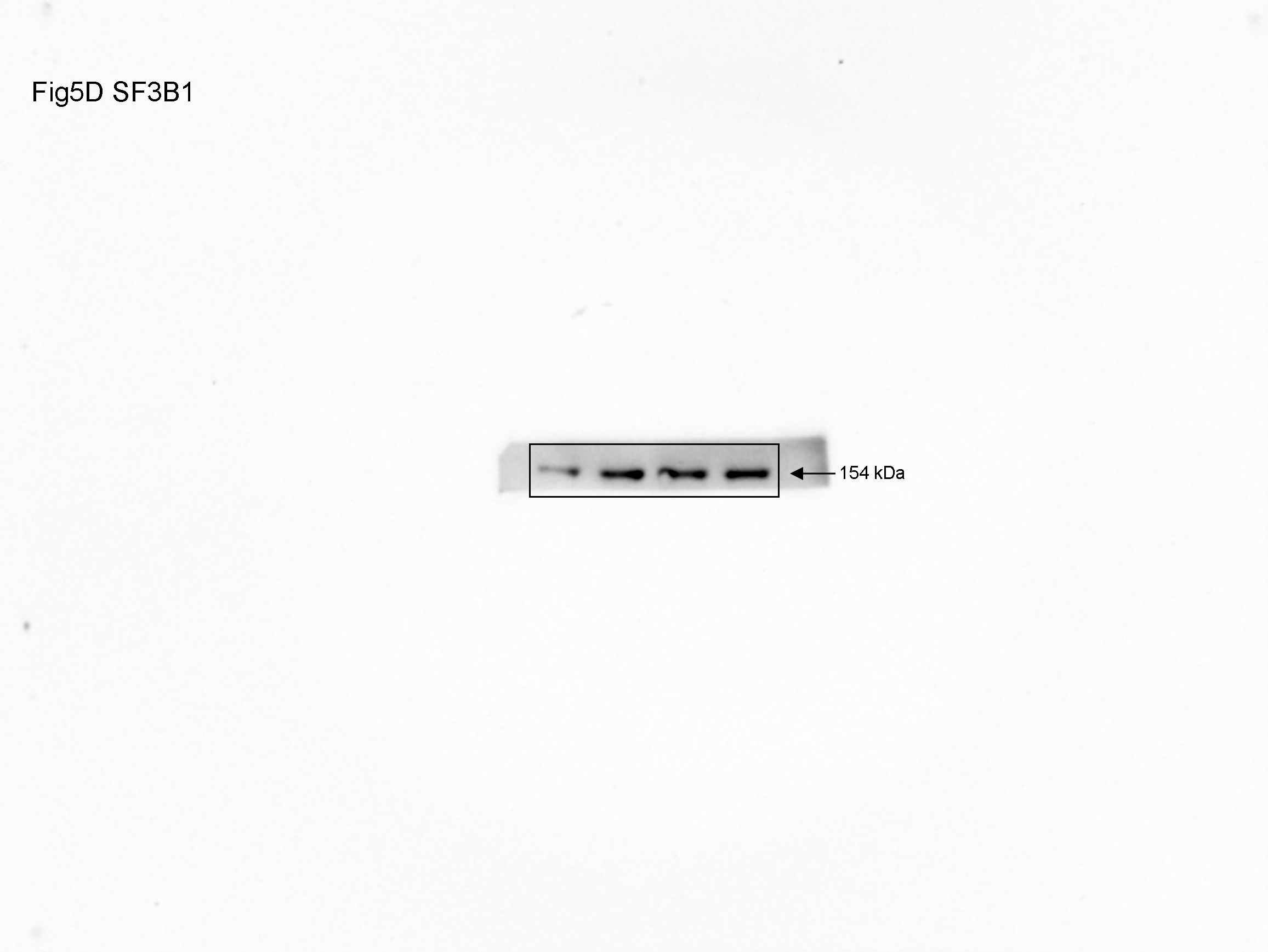

Supplement: Figure 5—source data 2. [file elife-98524-fig5-data2.zip › Fig 5-data2-v1/5D/right/SF3B1.tif]

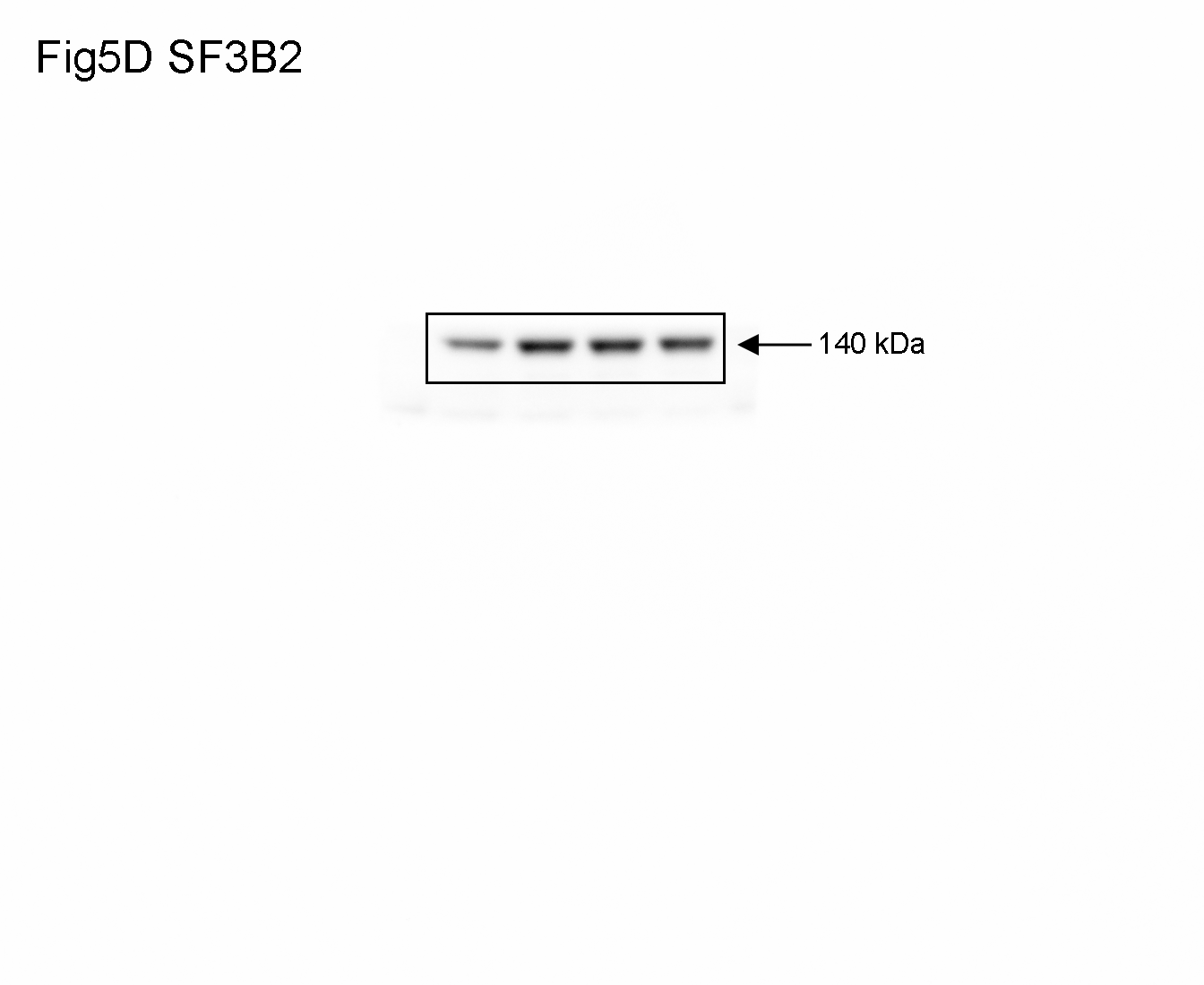

Supplement: Figure 5—source data 2. [file elife-98524-fig5-data2.zip › Fig 5-data2-v1/5D/right/SF3B2.tif]

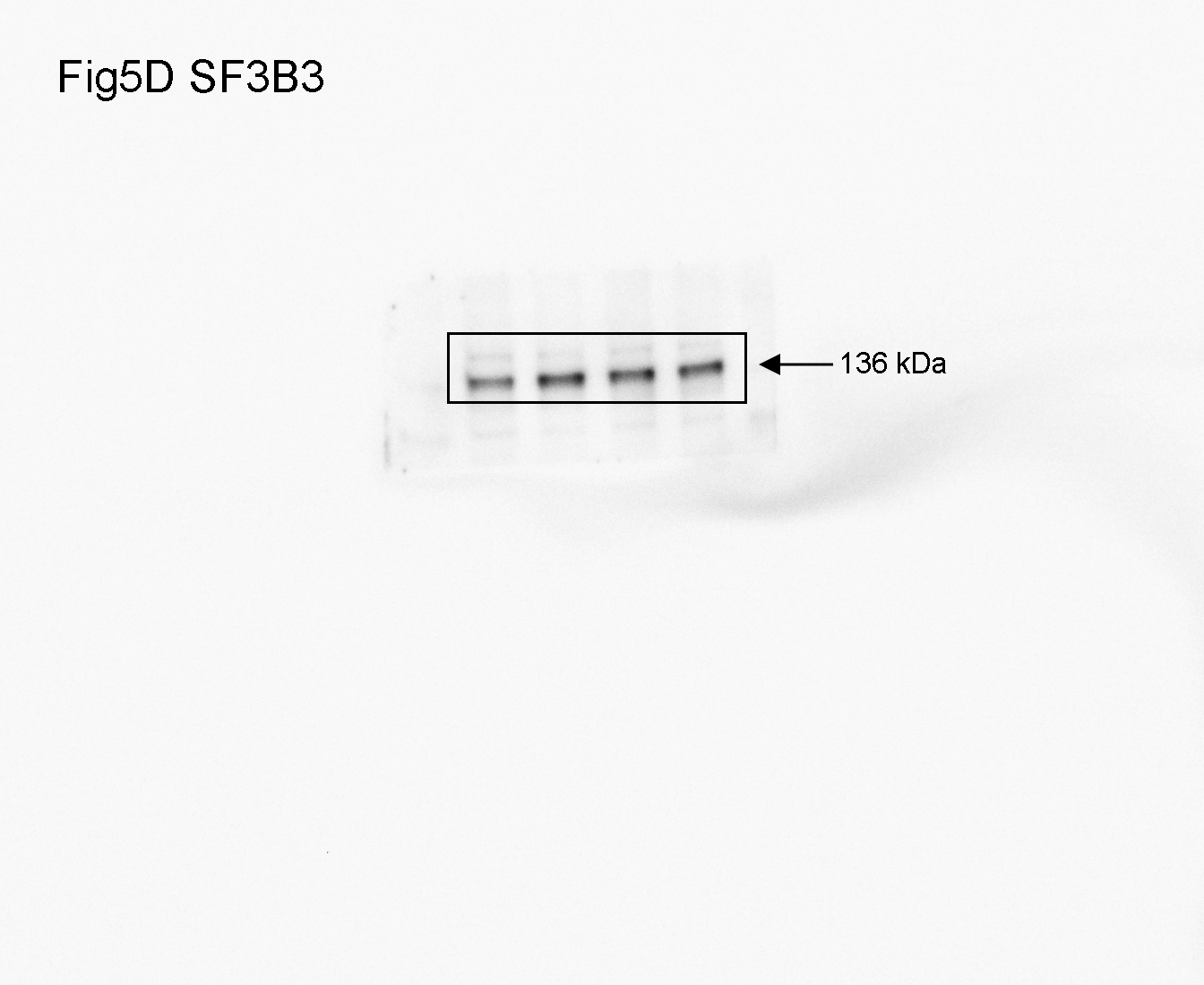

Supplement: Figure 5—source data 2. [file elife-98524-fig5-data2.zip › Fig 5-data2-v1/5D/right/SF3B3.tif]

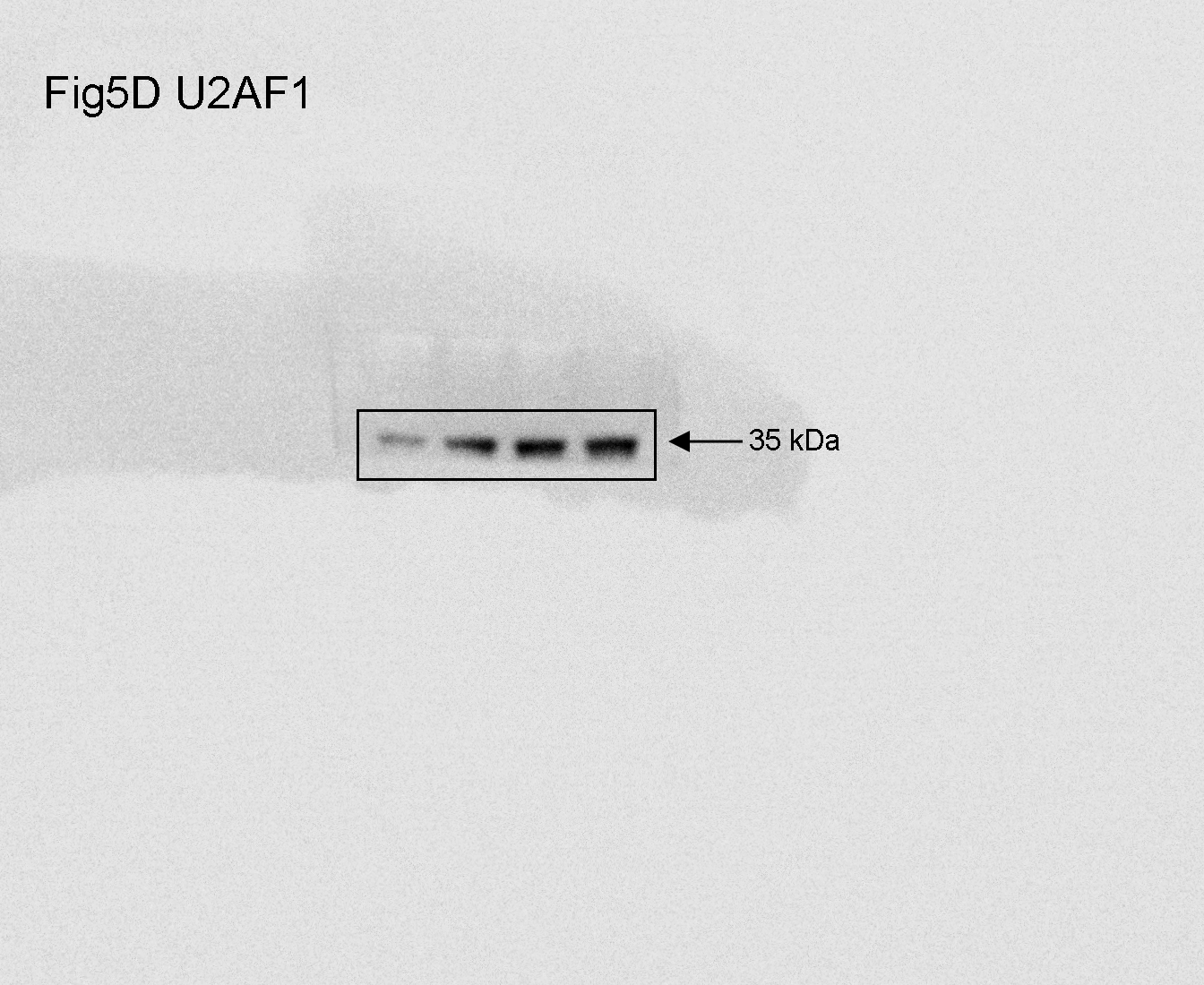

Supplement: Figure 5—source data 2. [file elife-98524-fig5-data2.zip › Fig 5-data2-v1/5D/right/U2AF1.tif]
