# Supplementary figures and images for: Nuclear translocation of SIRT4 mediates deacetylation of U2AF2 to modulate renal fibrosis through alternative splicing-mediated upregulation of CCN2 (part 4 of 9)
Source: eLife. 2024 Nov 4;13:RP98524. doi: 10.7554/eLife.98524 (PMC11534337; doi:10.7554/eLife.98524)

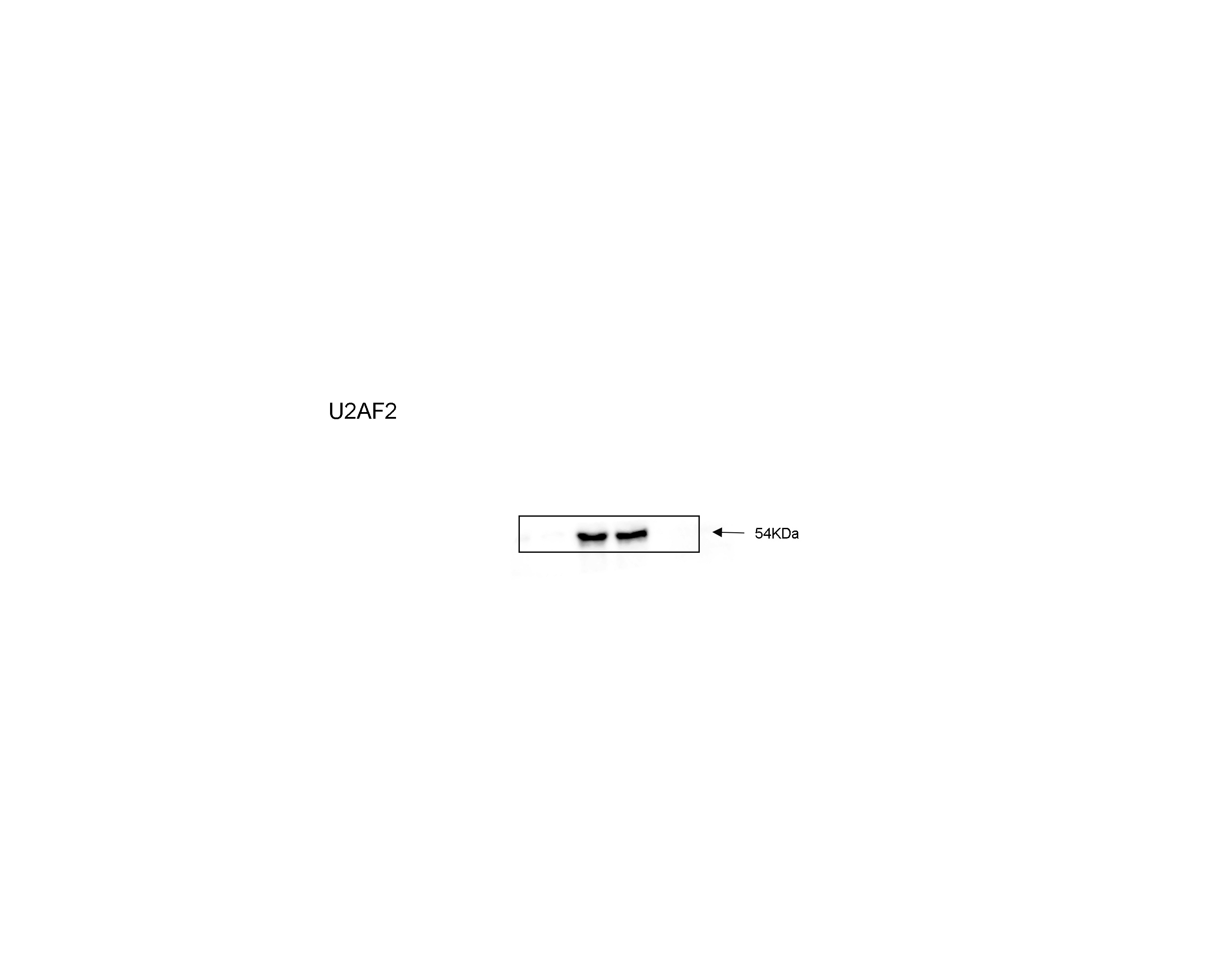

Supplement: Figure 4—source data 2. [file elife-98524-fig4-data2.zip › Fig 4-data2-v1/4C/4/upper/Flag .tif]

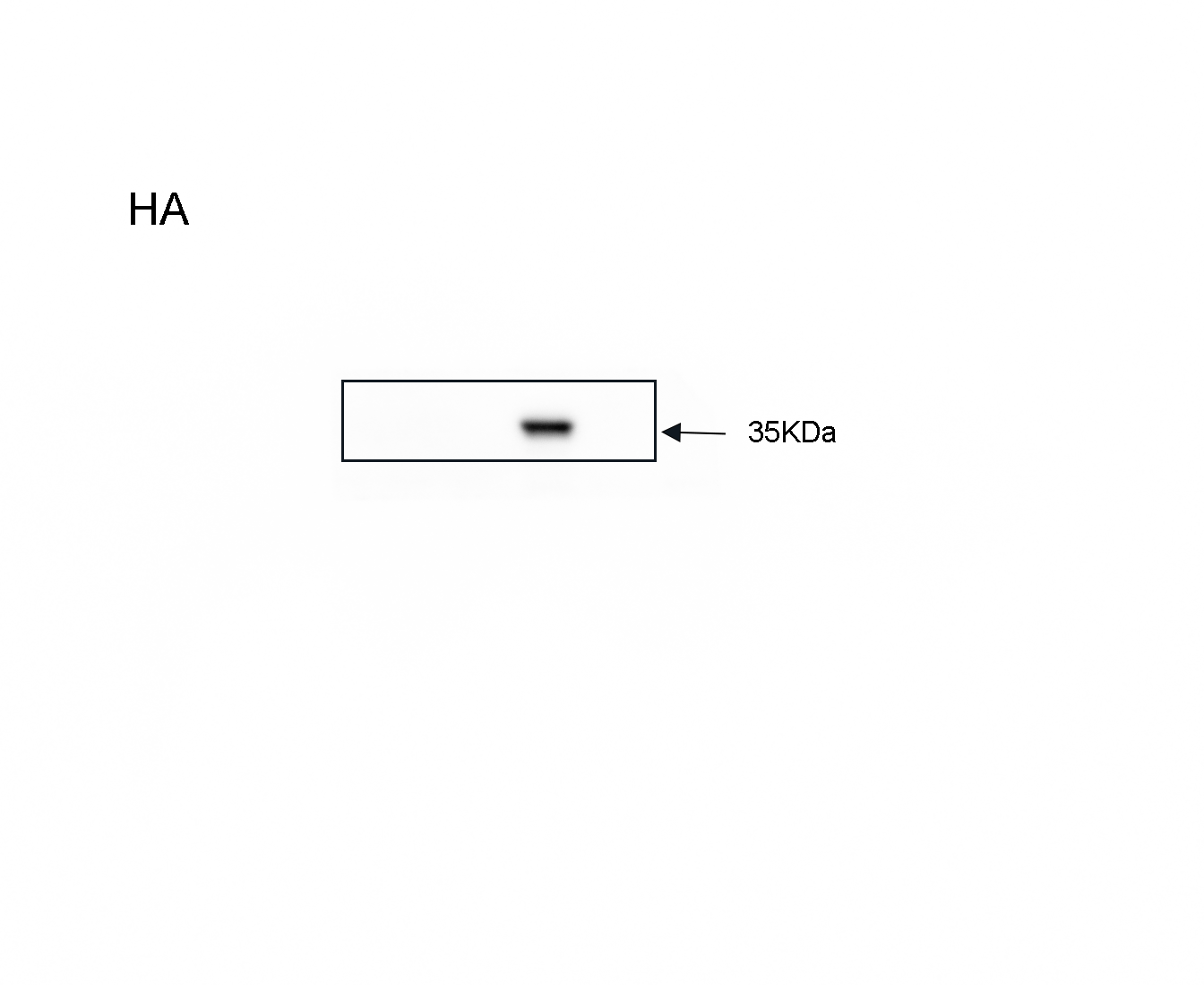

Supplement: Figure 4—source data 2. [file elife-98524-fig4-data2.zip › Fig 4-data2-v1/4C/4/upper/HA .tif]

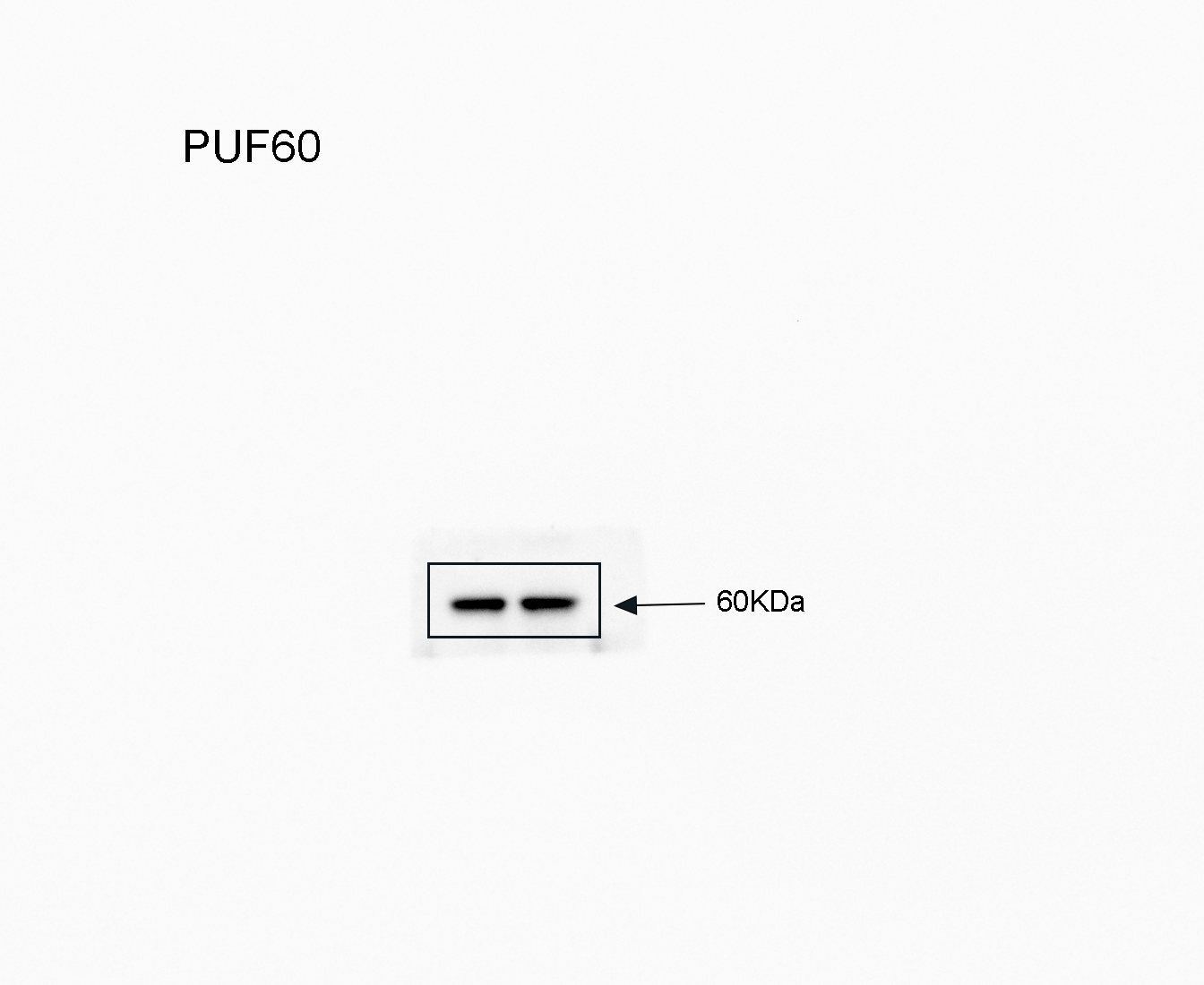

Supplement: Figure 4—source data 2. [file elife-98524-fig4-data2.zip › Fig 4-data2-v1/4D/bottom/PUF60 .tif]

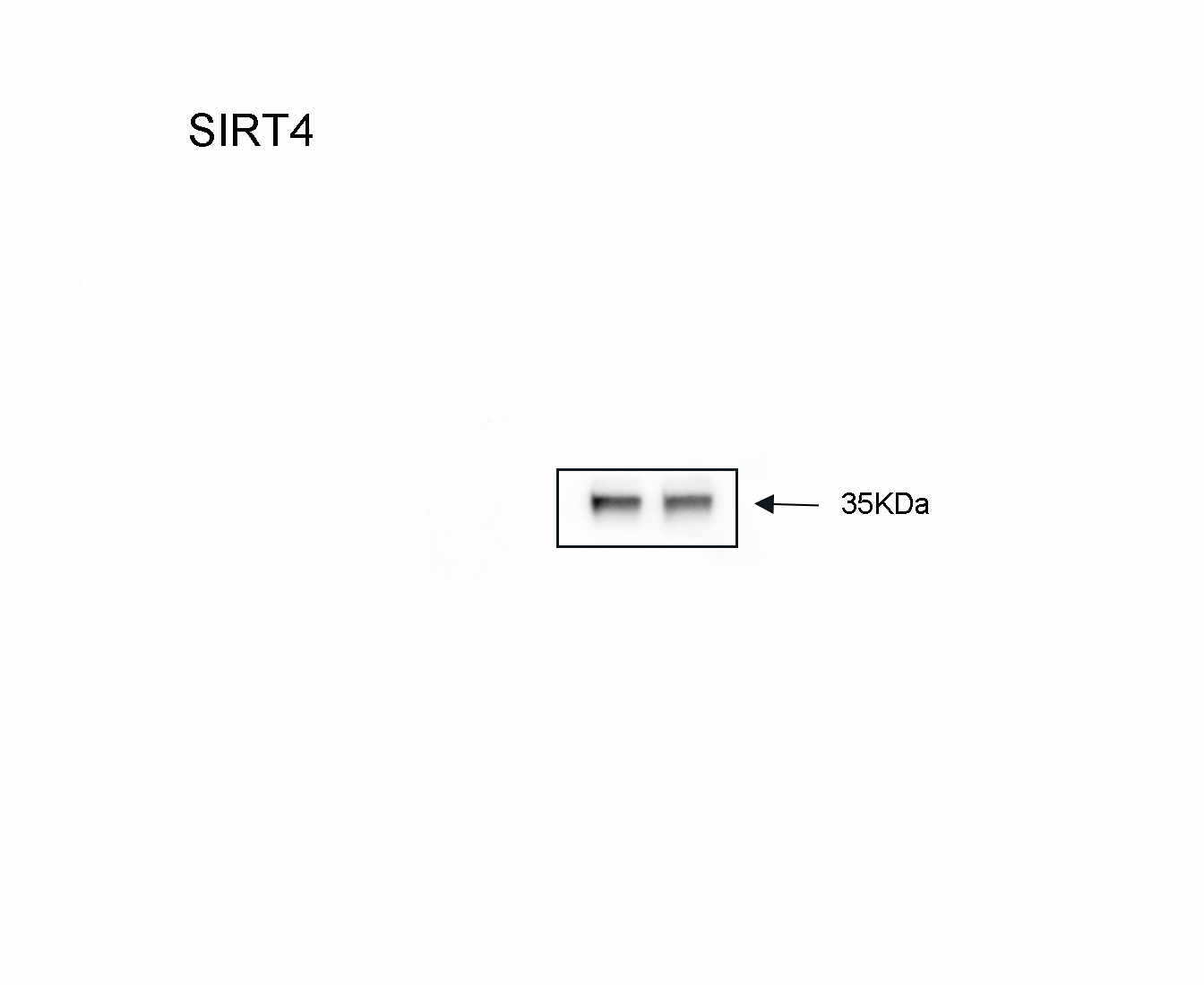

Supplement: Figure 4—source data 2. [file elife-98524-fig4-data2.zip › Fig 4-data2-v1/4D/bottom/SIRT4 .tif]

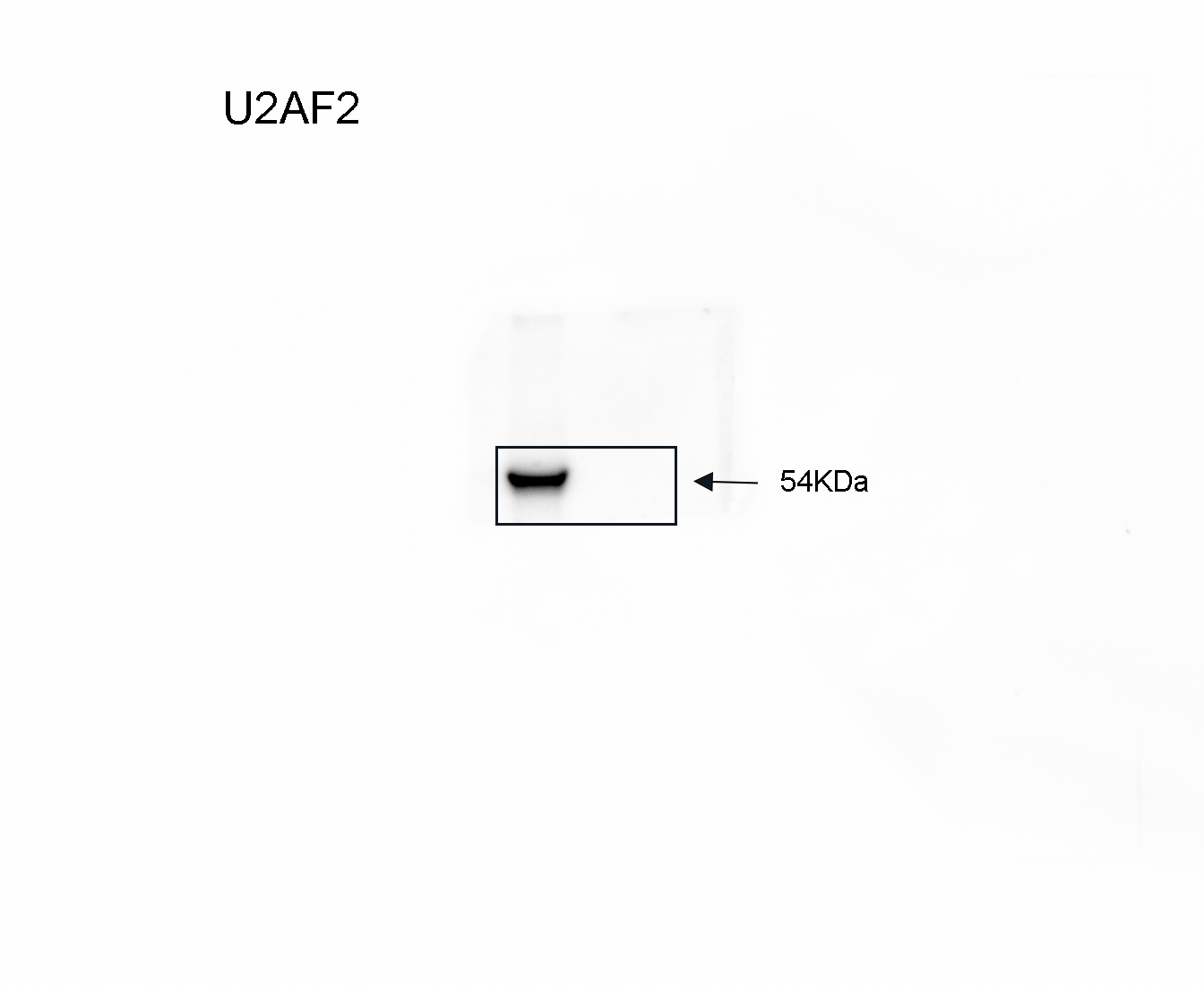

Supplement: Figure 4—source data 2. [file elife-98524-fig4-data2.zip › Fig 4-data2-v1/4D/bottom/U2AF2 .tif]

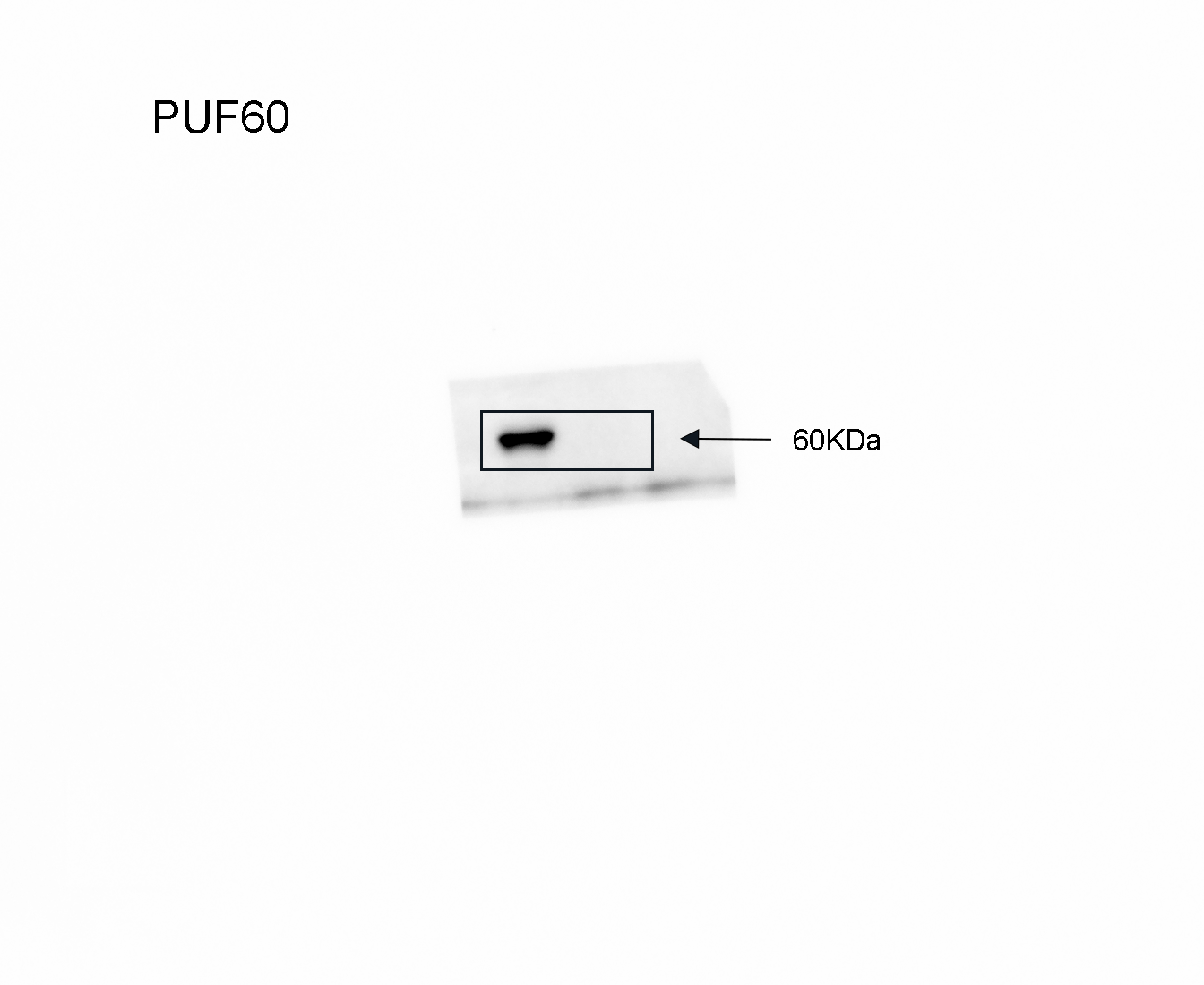

Supplement: Figure 4—source data 2. [file elife-98524-fig4-data2.zip › Fig 4-data2-v1/4D/upper/PUF60 .tif]

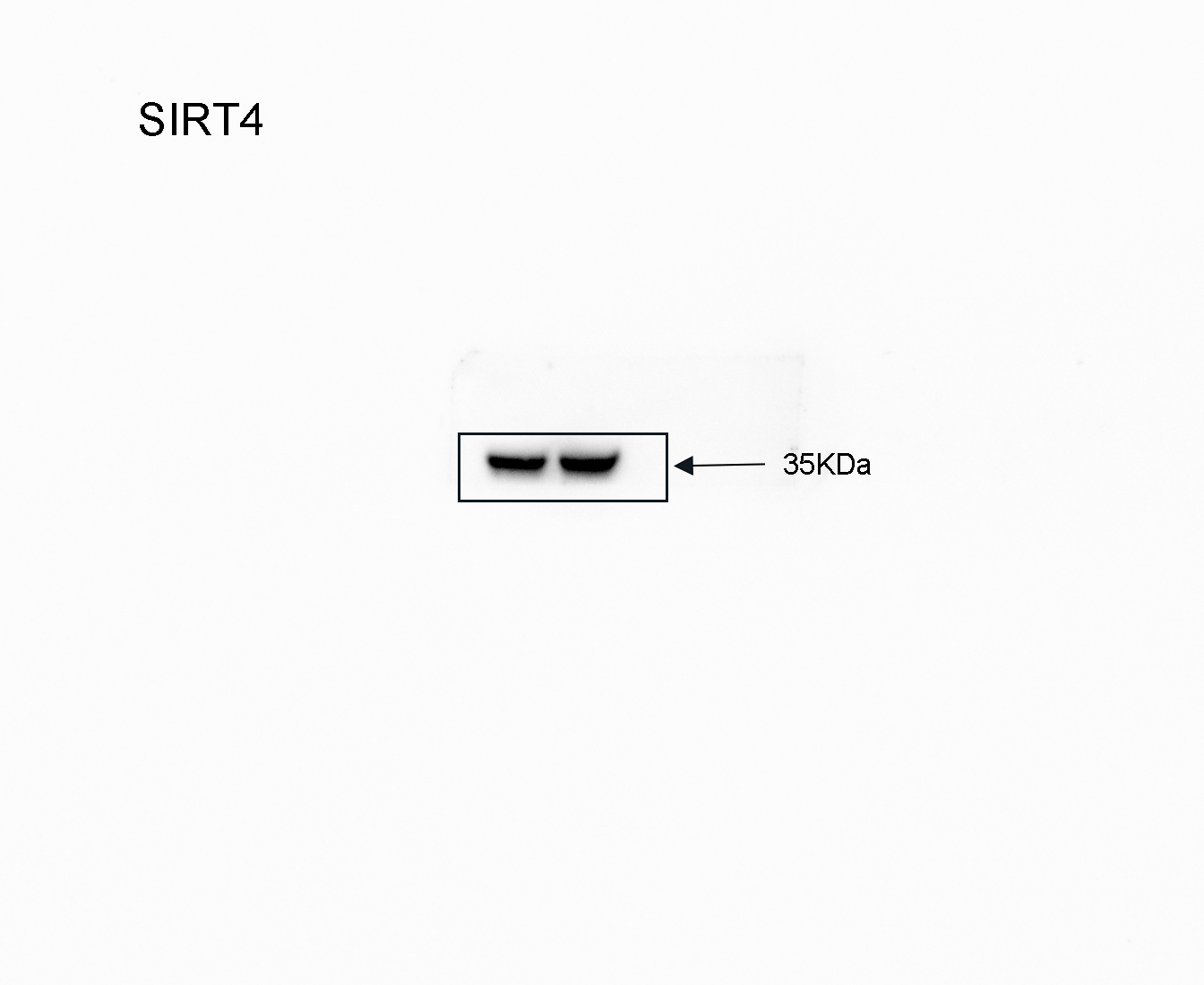

Supplement: Figure 4—source data 2. [file elife-98524-fig4-data2.zip › Fig 4-data2-v1/4D/upper/SITR4 .tif]

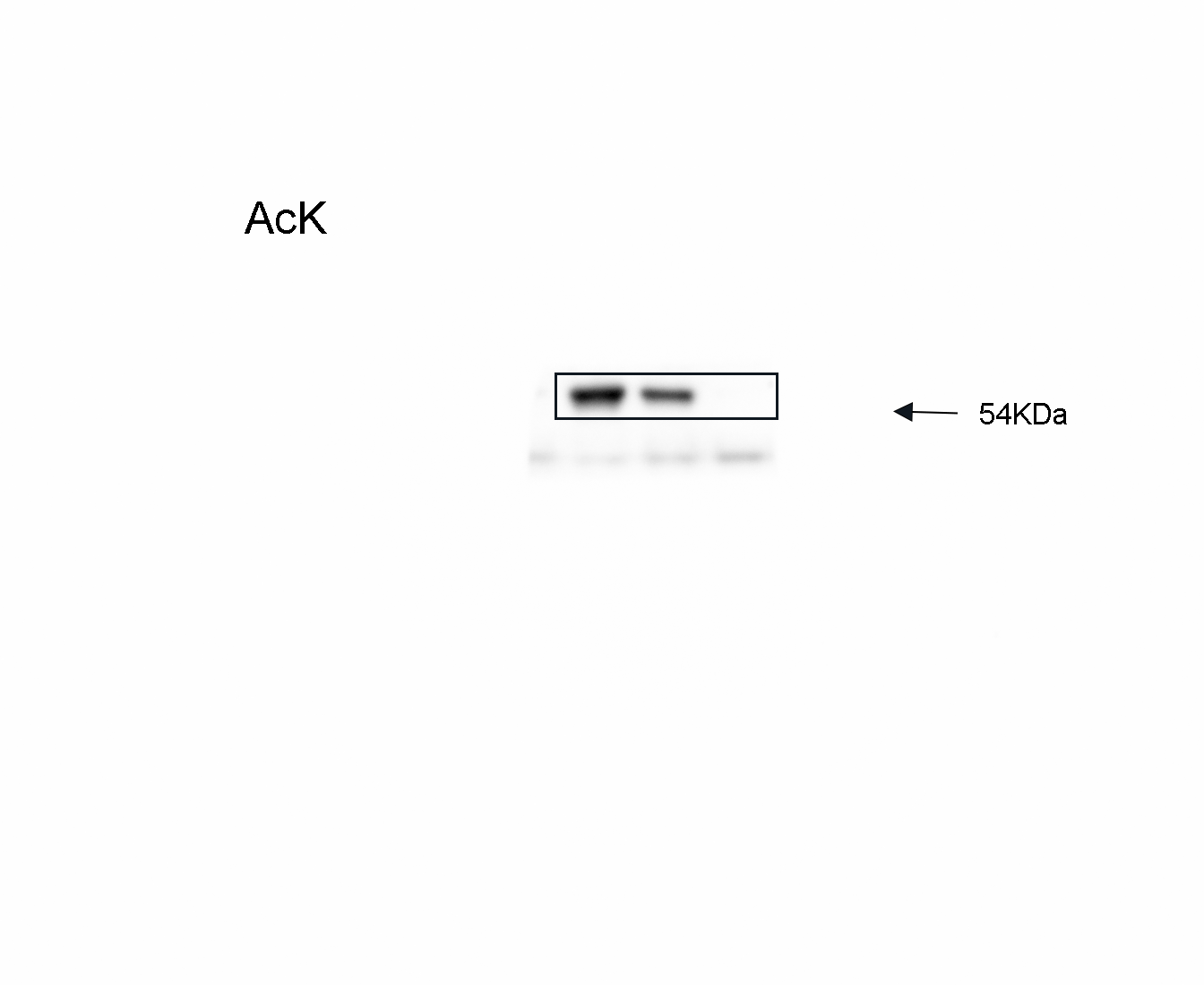

Supplement: Figure 4—source data 2. [file elife-98524-fig4-data2.zip › Fig 4-data2-v1/4E/AcK .tif]

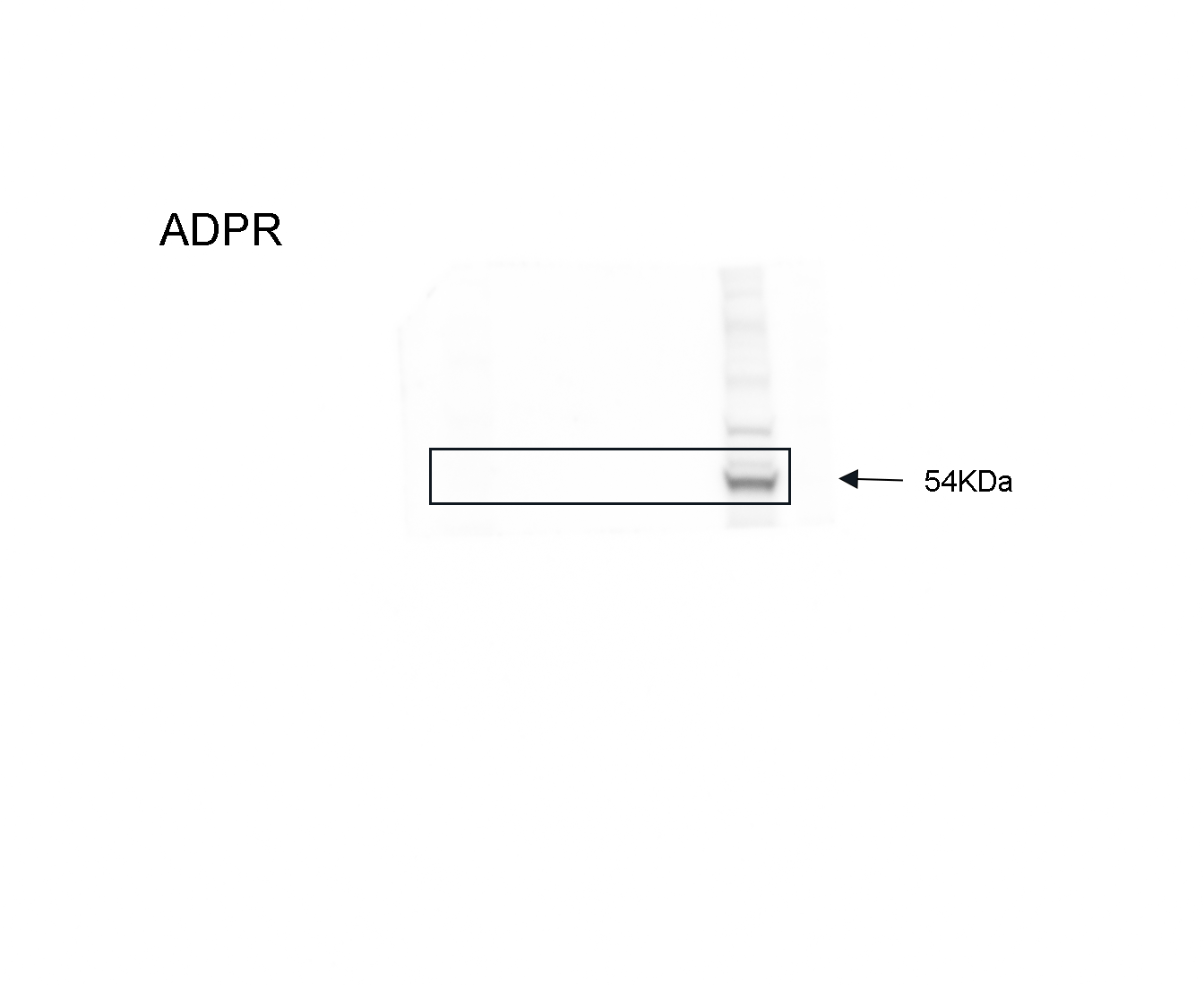

Supplement: Figure 4—source data 2. [file elife-98524-fig4-data2.zip › Fig 4-data2-v1/4E/ADPR .tif]

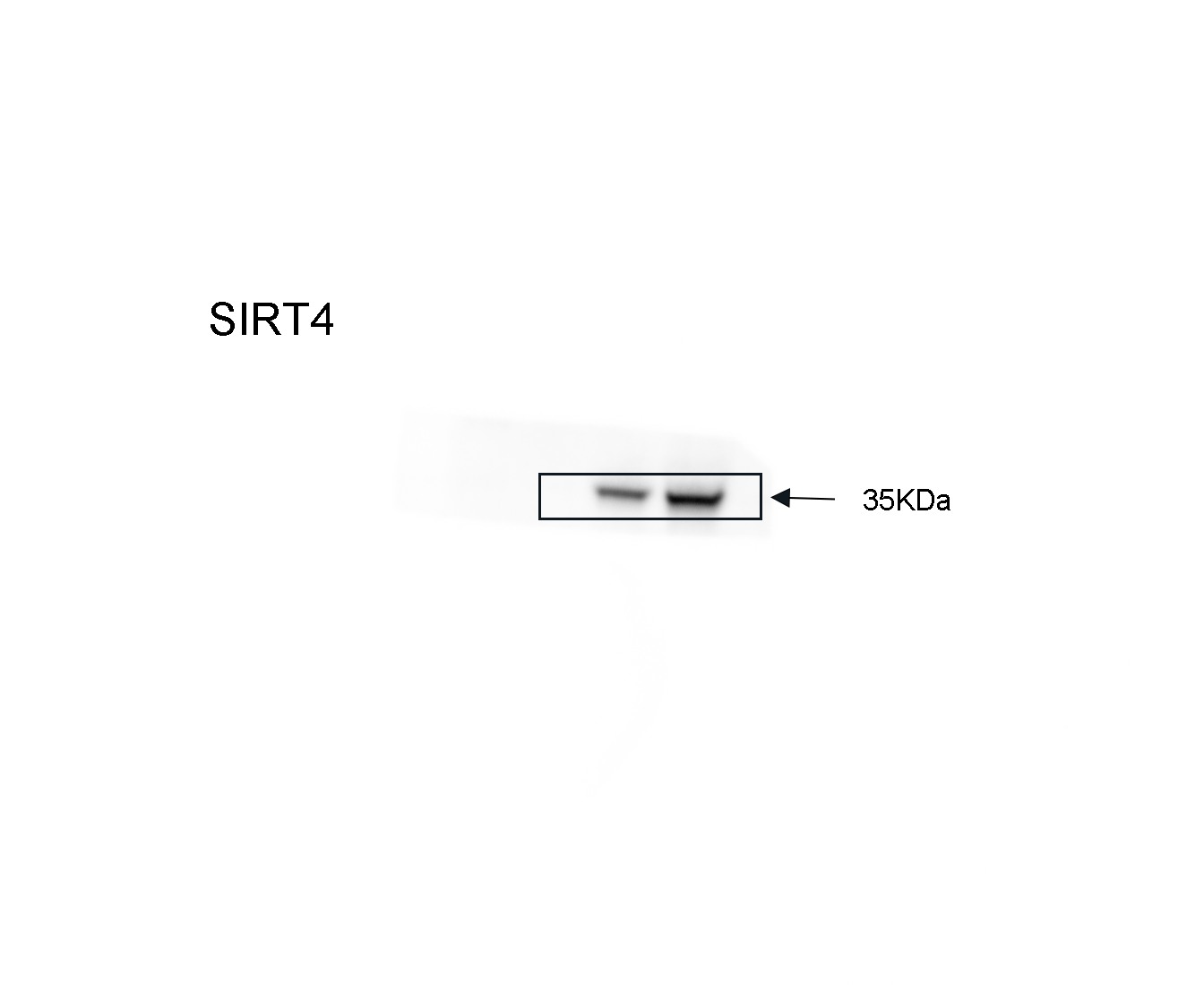

Supplement: Figure 4—source data 2. [file elife-98524-fig4-data2.zip › Fig 4-data2-v1/4E/SIRT4 .tif]

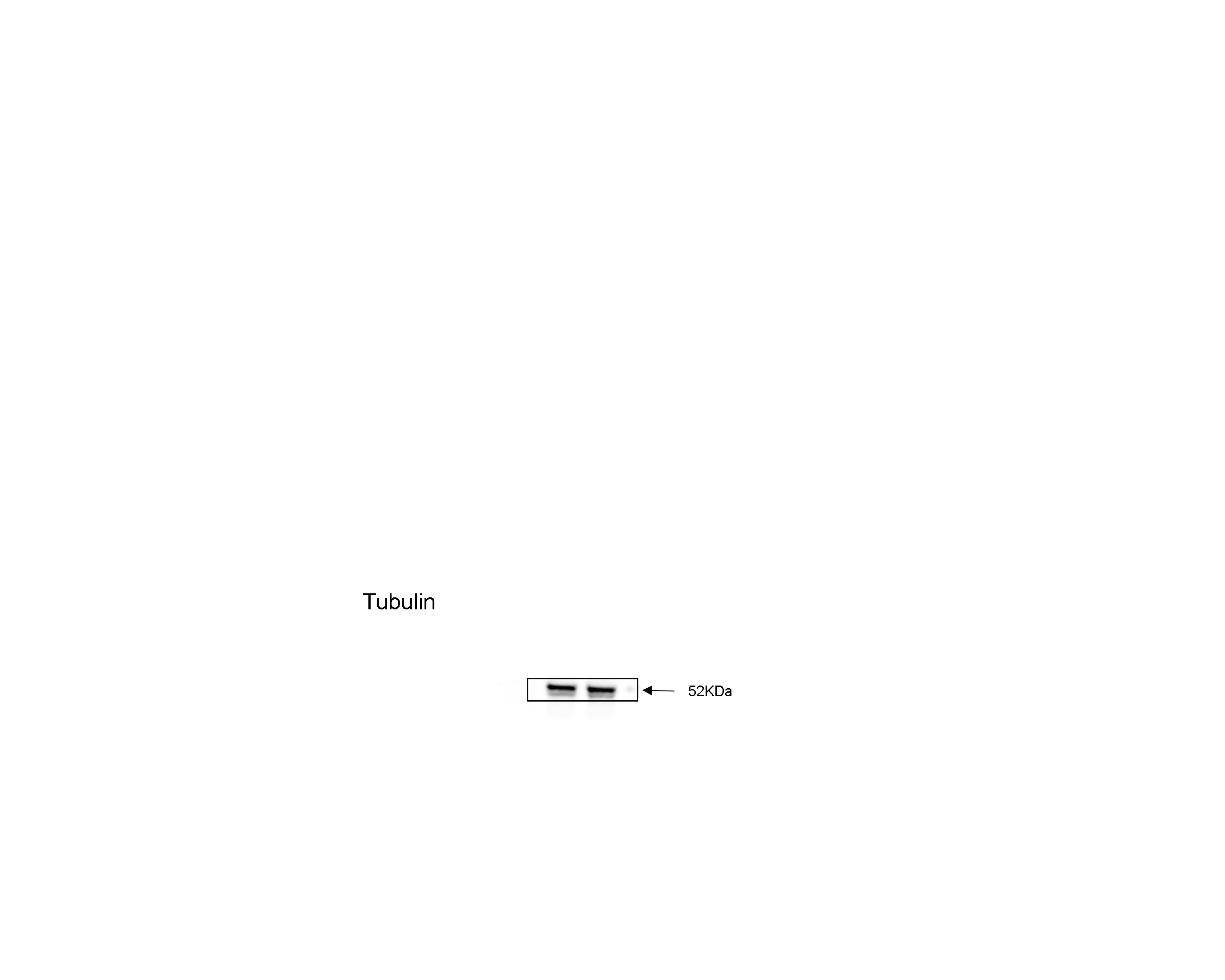

Supplement: Figure 4—source data 2. [file elife-98524-fig4-data2.zip › Fig 4-data2-v1/4E/Tubulin .tif]

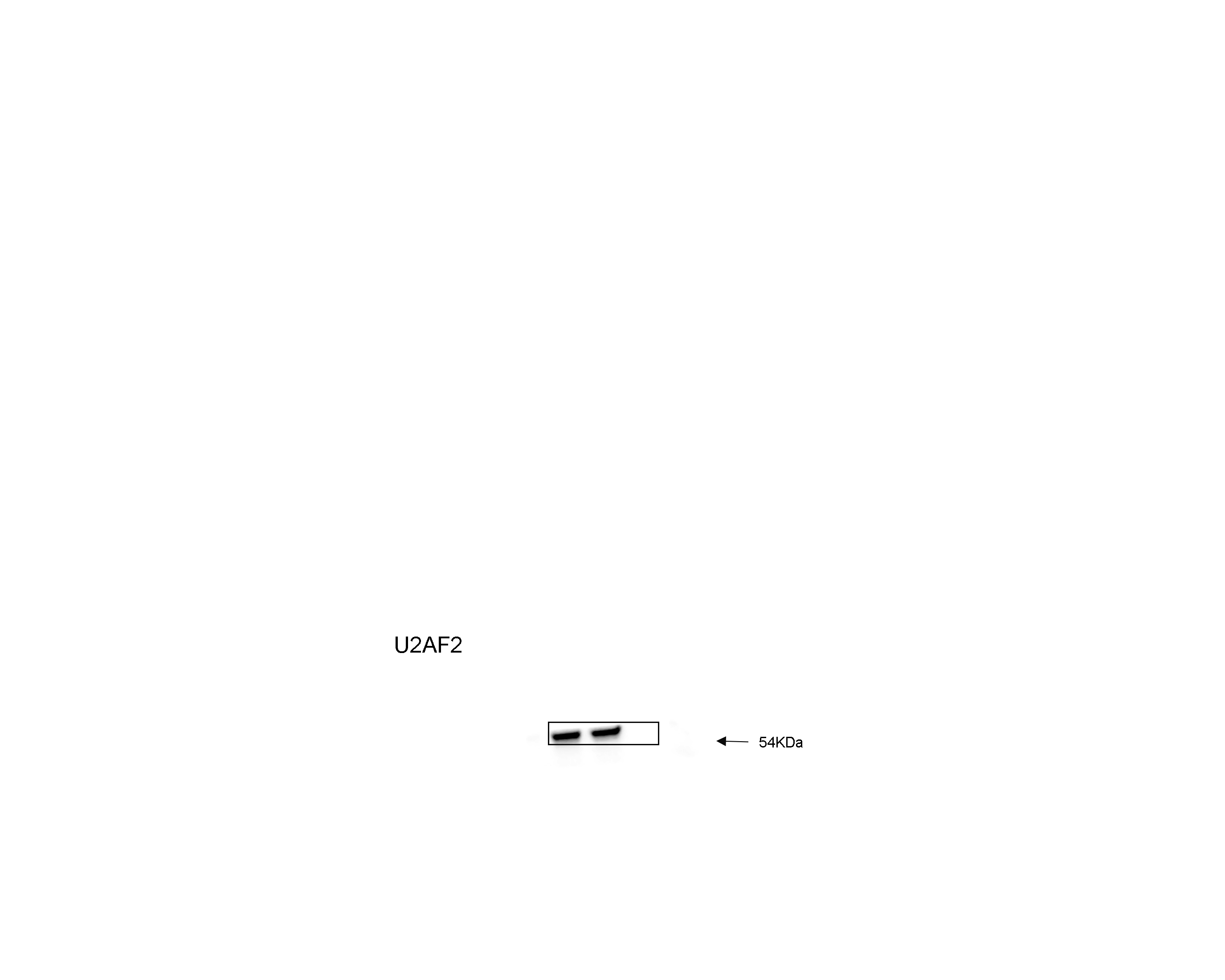

Supplement: Figure 4—source data 2. [file elife-98524-fig4-data2.zip › Fig 4-data2-v1/4E/U2AF2 .tif]

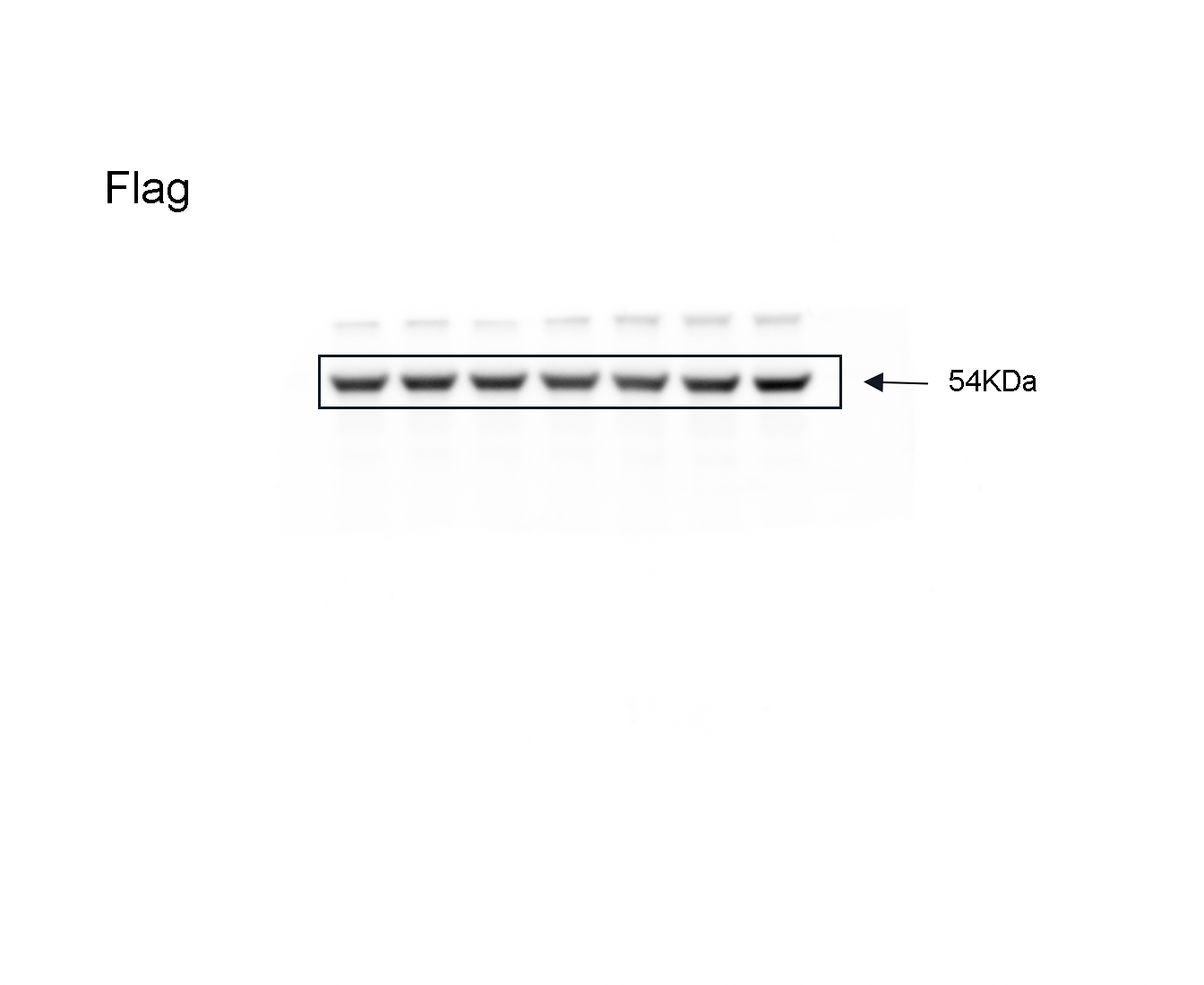

Supplement: Figure 4—source data 2. [file elife-98524-fig4-data2.zip › Fig 4-data2-v1/4F/bottom/Flag .tif]

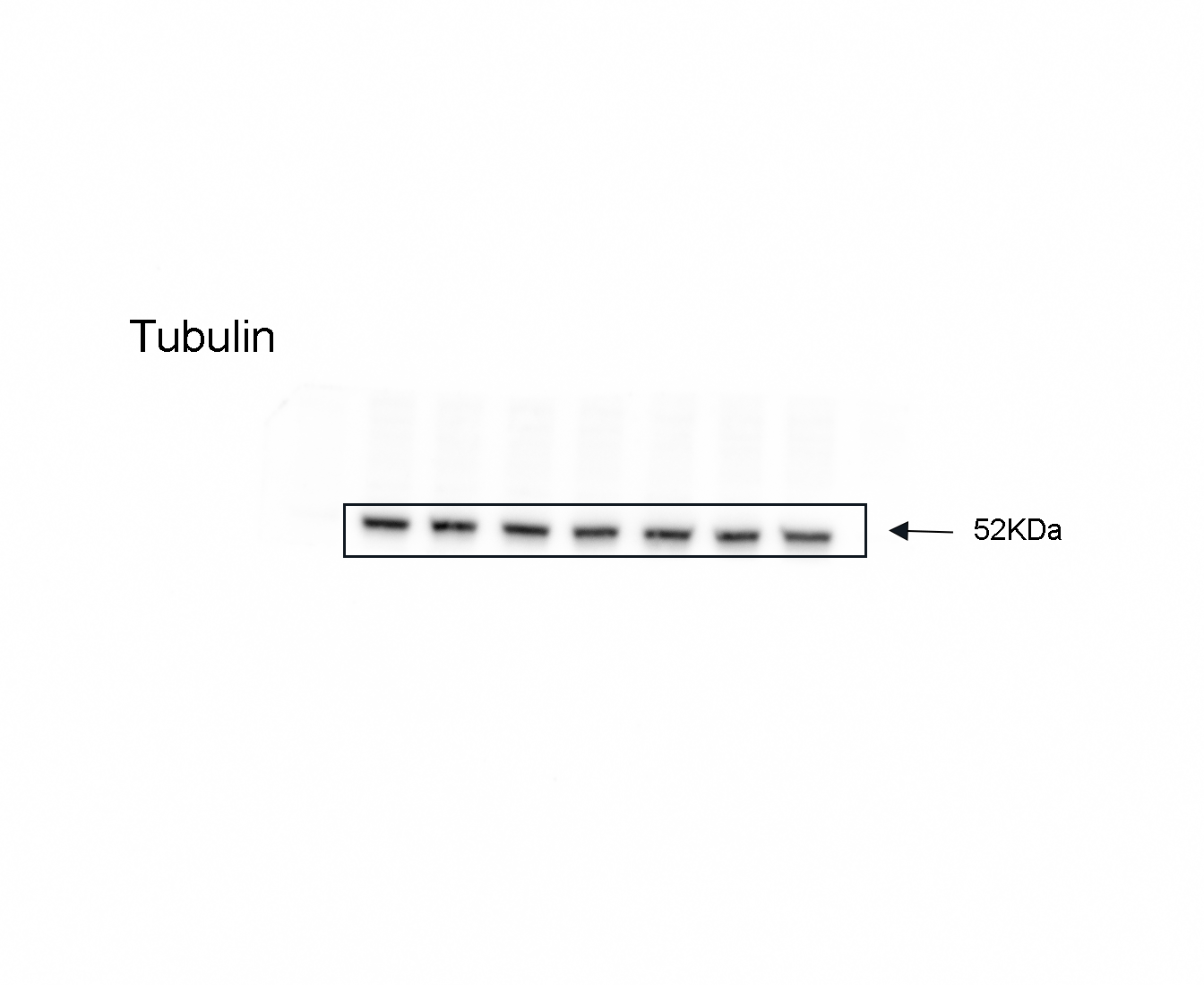

Supplement: Figure 4—source data 2. [file elife-98524-fig4-data2.zip › Fig 4-data2-v1/4F/bottom/Tubulin .tif]

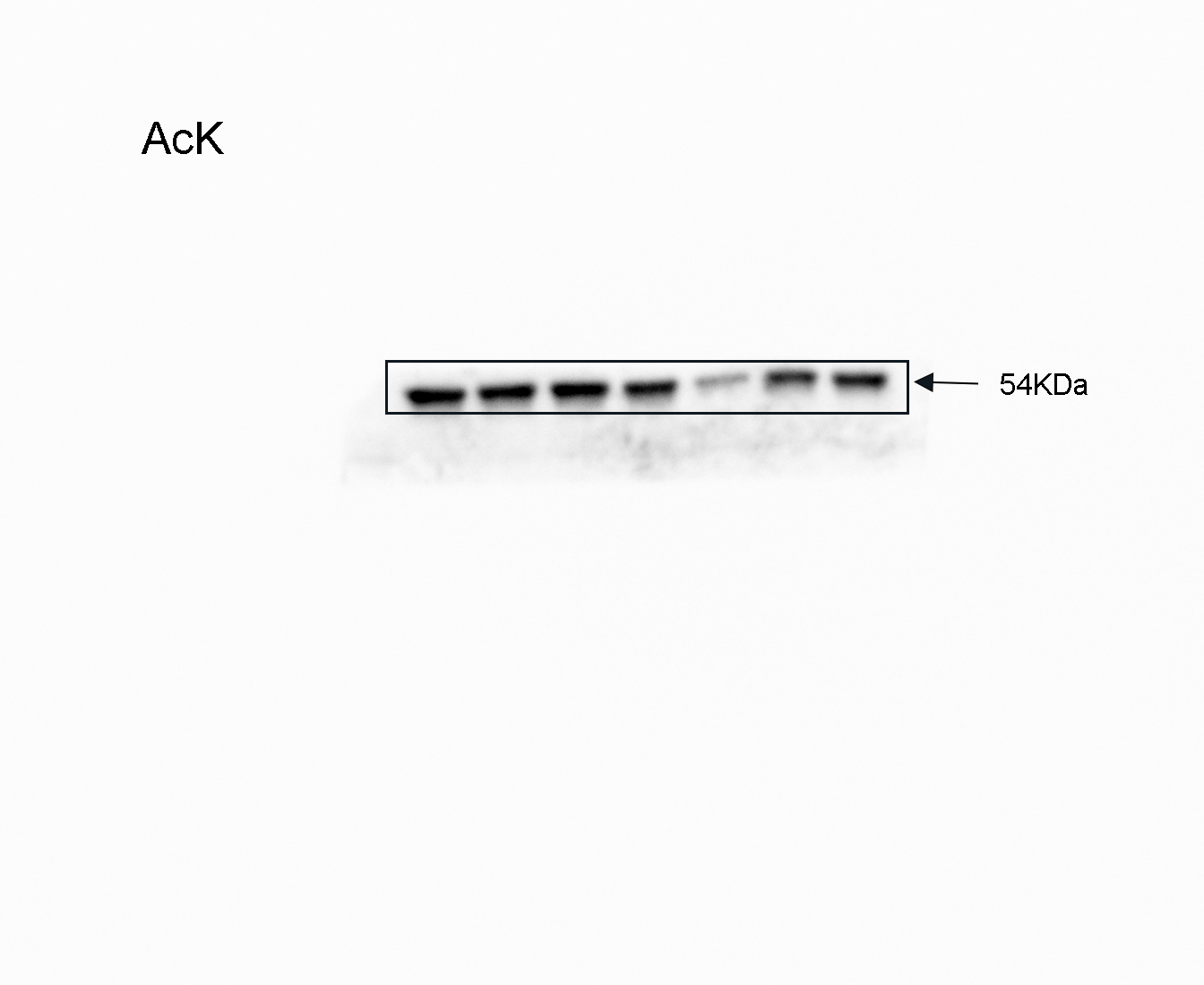

Supplement: Figure 4—source data 2. [file elife-98524-fig4-data2.zip › Fig 4-data2-v1/4F/upper/Ac-k .tif]

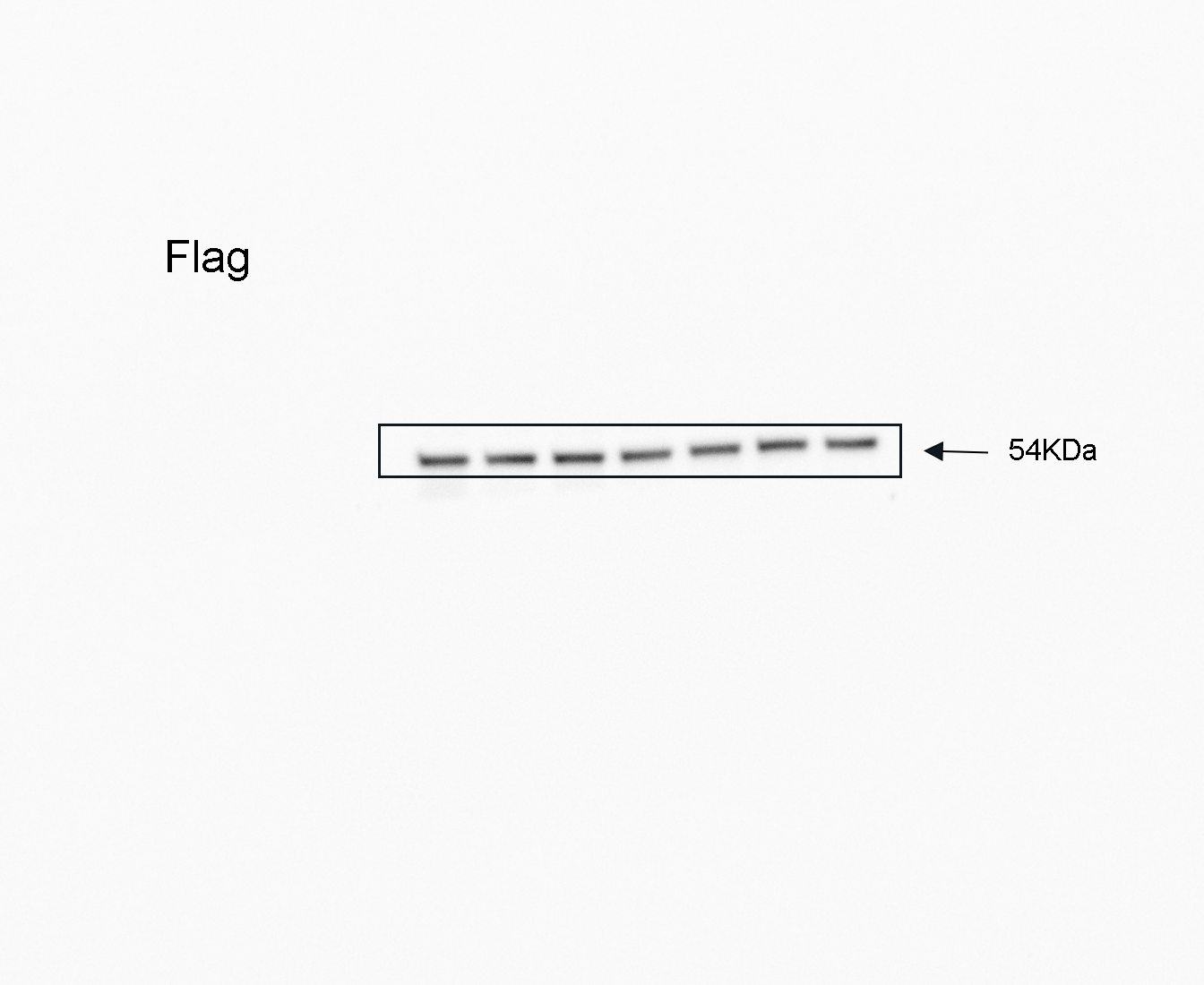

Supplement: Figure 4—source data 2. [file elife-98524-fig4-data2.zip › Fig 4-data2-v1/4F/upper/Flag .tif]

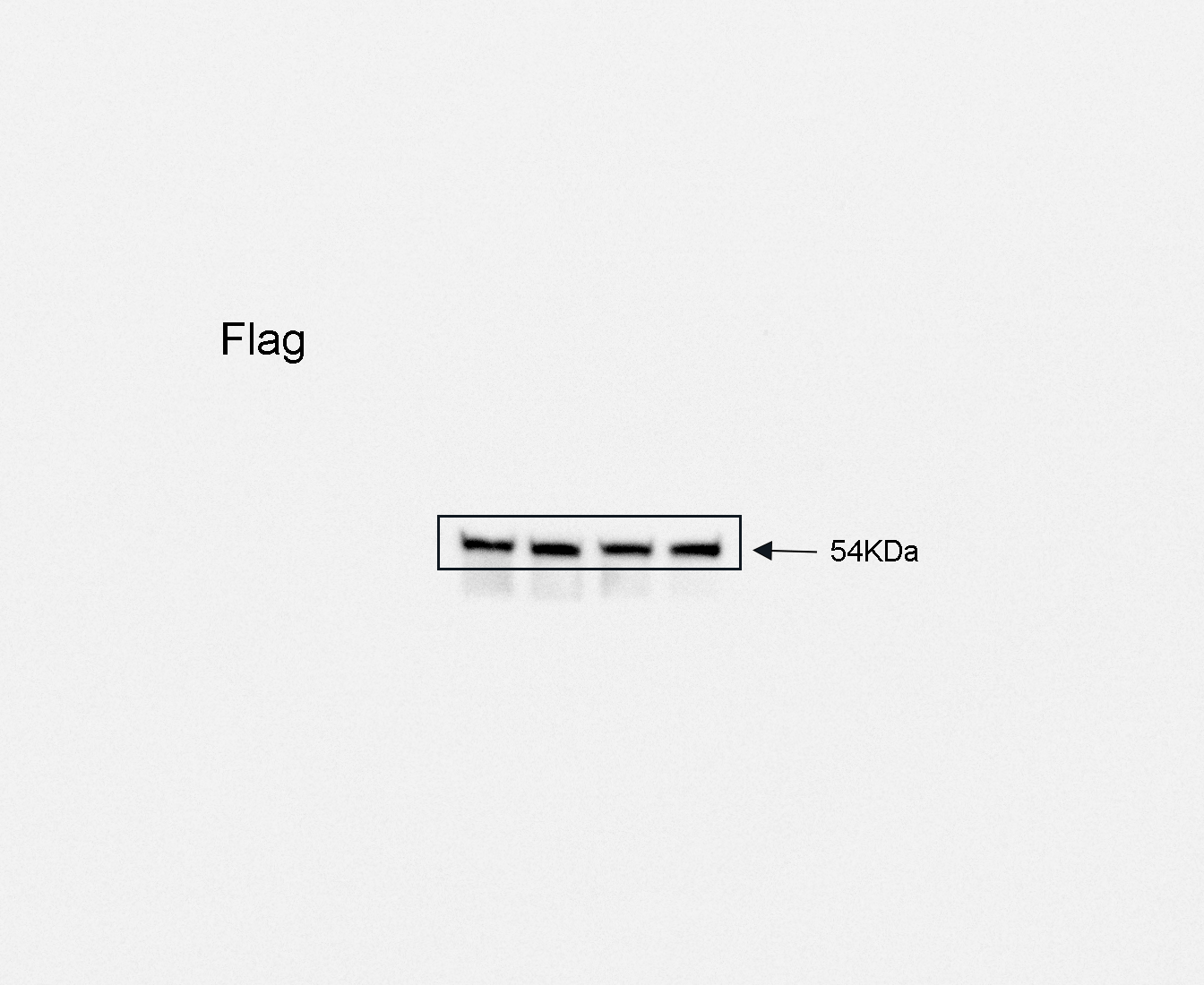

Supplement: Figure 4—source data 2. [file elife-98524-fig4-data2.zip › Fig 4-data2-v1/4H/1/bottom/Flag .tif]

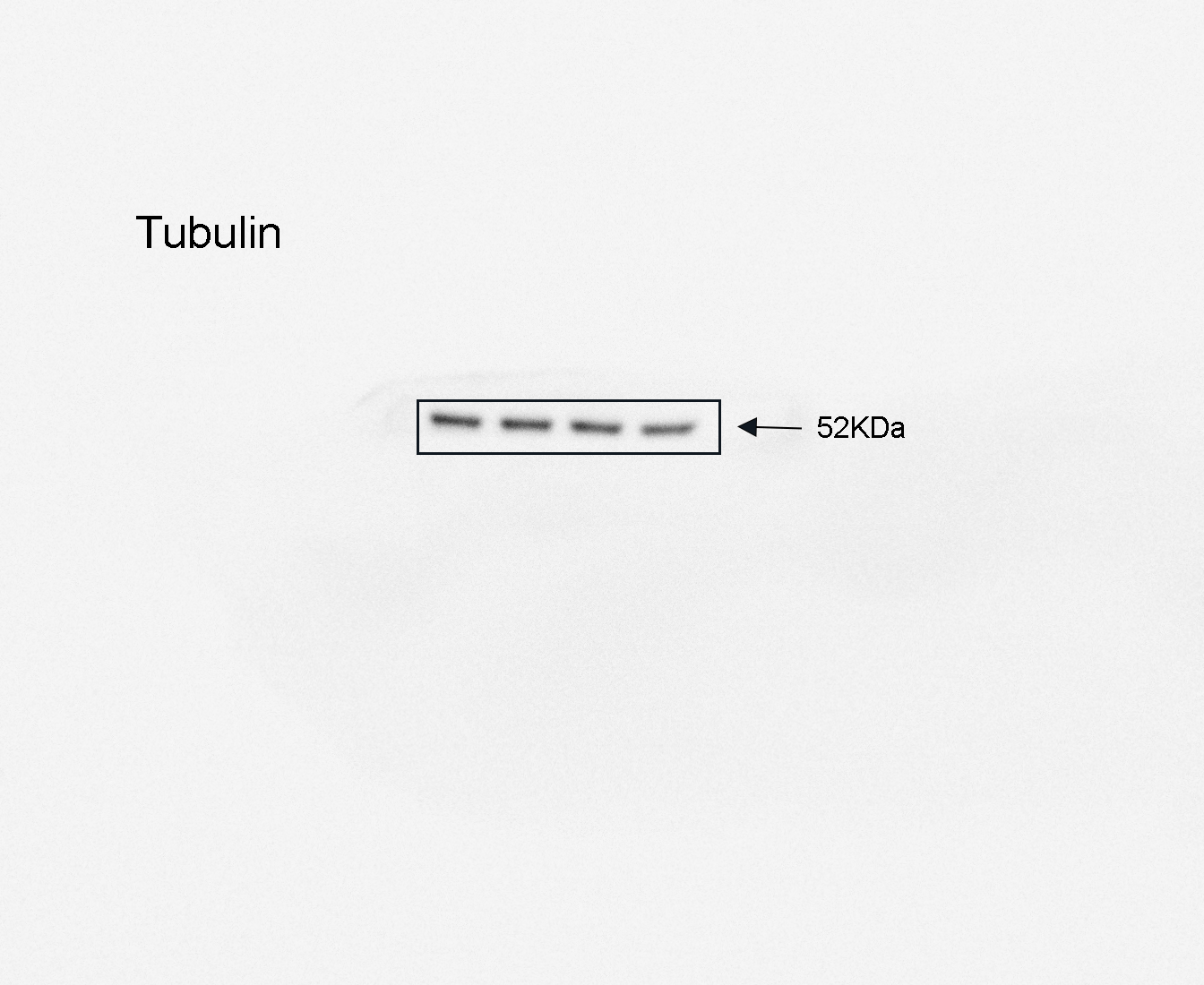

Supplement: Figure 4—source data 2. [file elife-98524-fig4-data2.zip › Fig 4-data2-v1/4H/1/bottom/Tubulin .tif]

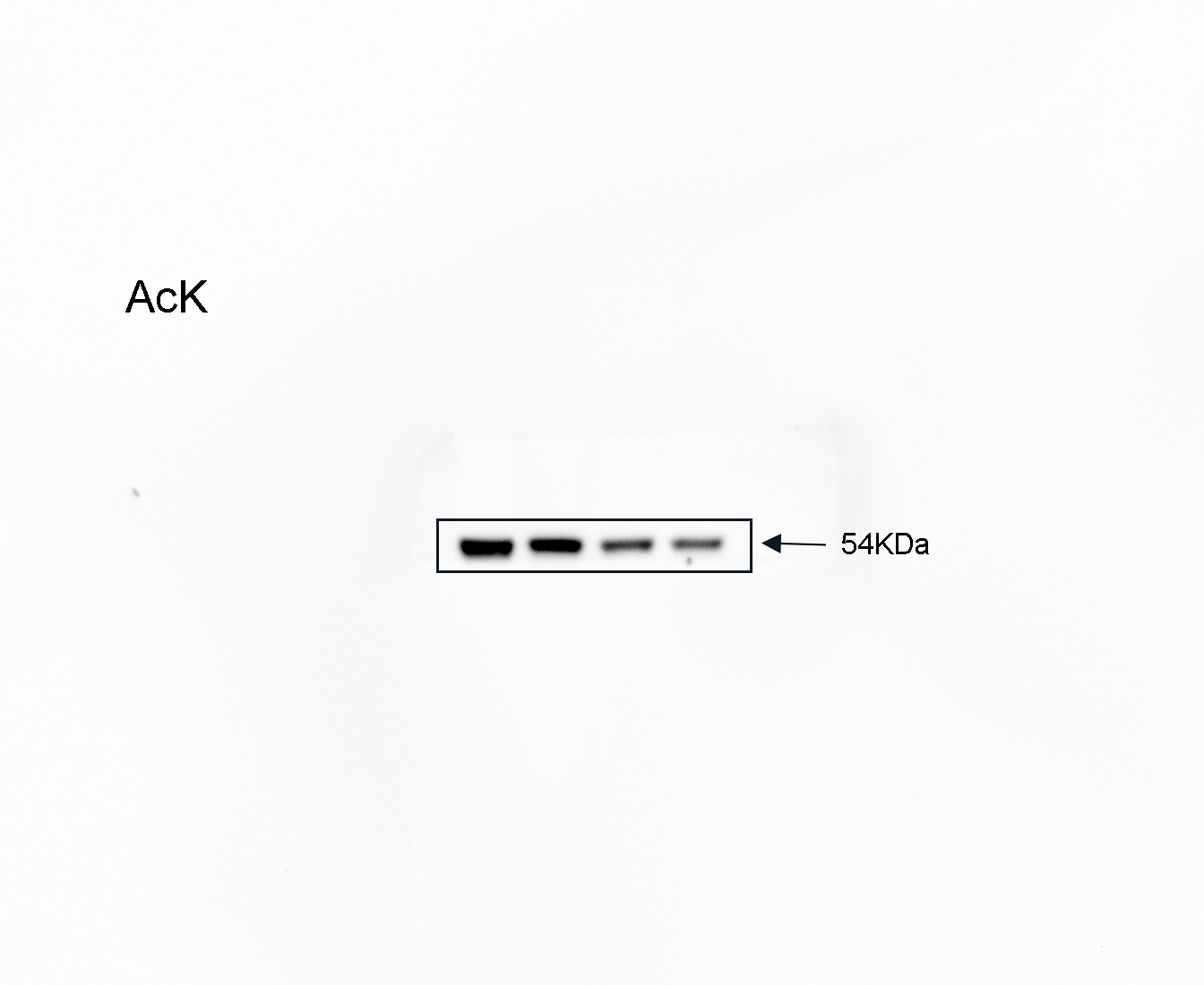

Supplement: Figure 4—source data 2. [file elife-98524-fig4-data2.zip › Fig 4-data2-v1/4H/1/upper/Ac-K .tif]

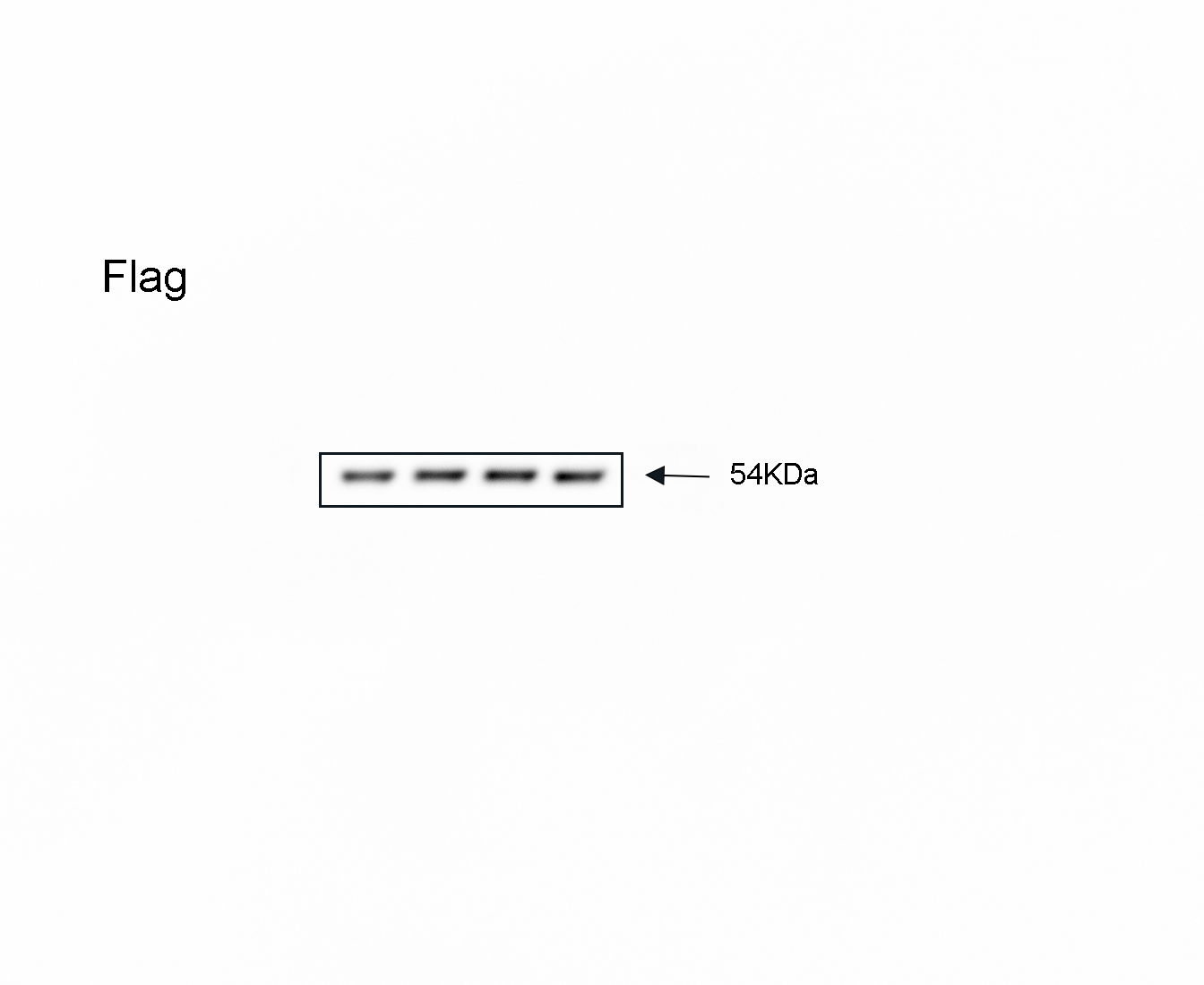

Supplement: Figure 4—source data 2. [file elife-98524-fig4-data2.zip › Fig 4-data2-v1/4H/1/upper/Flag .tif]

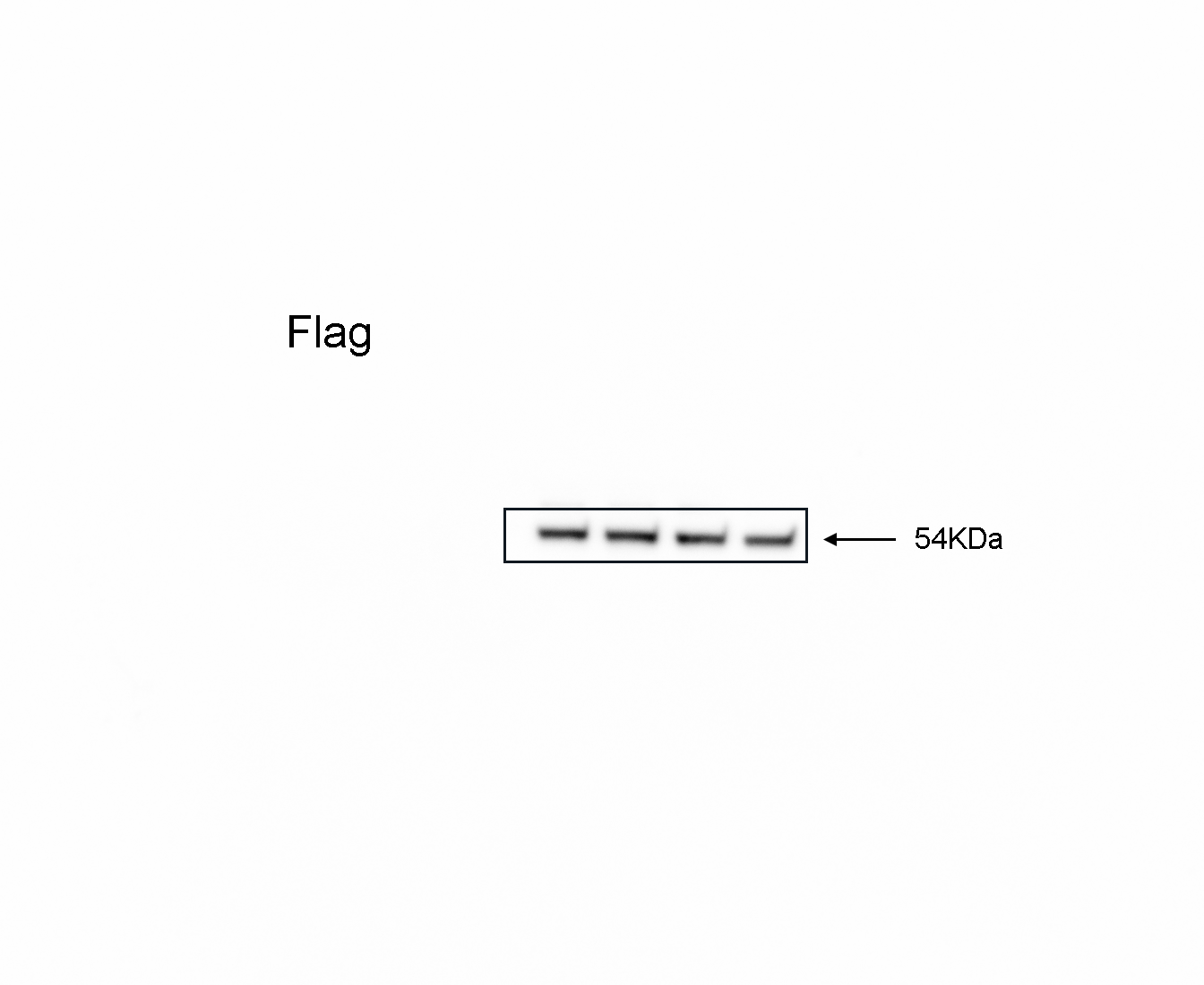

Supplement: Figure 4—source data 2. [file elife-98524-fig4-data2.zip › Fig 4-data2-v1/4H/2/bottom/Flag .tif]

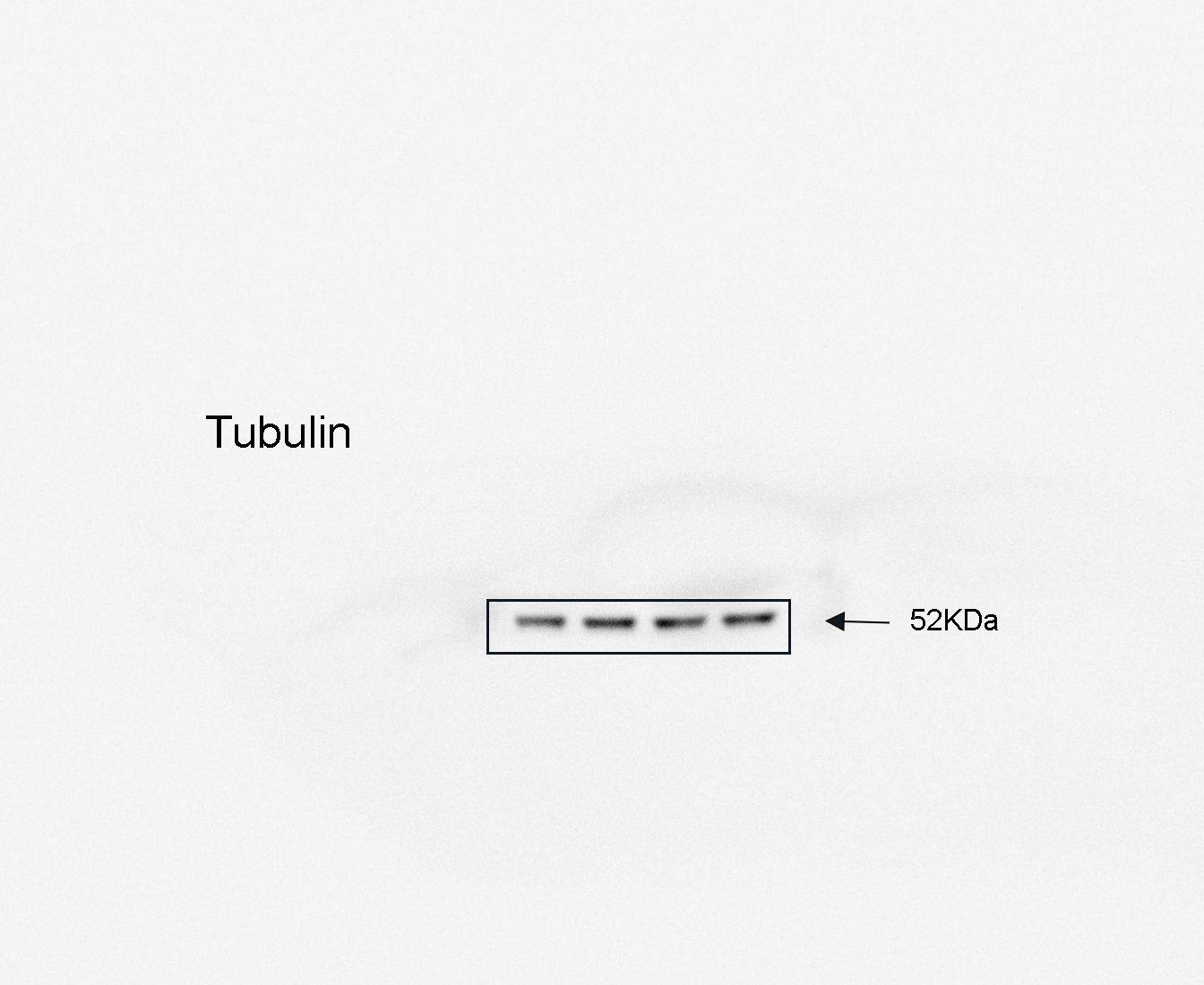

Supplement: Figure 4—source data 2. [file elife-98524-fig4-data2.zip › Fig 4-data2-v1/4H/2/bottom/Tubulin .tif]

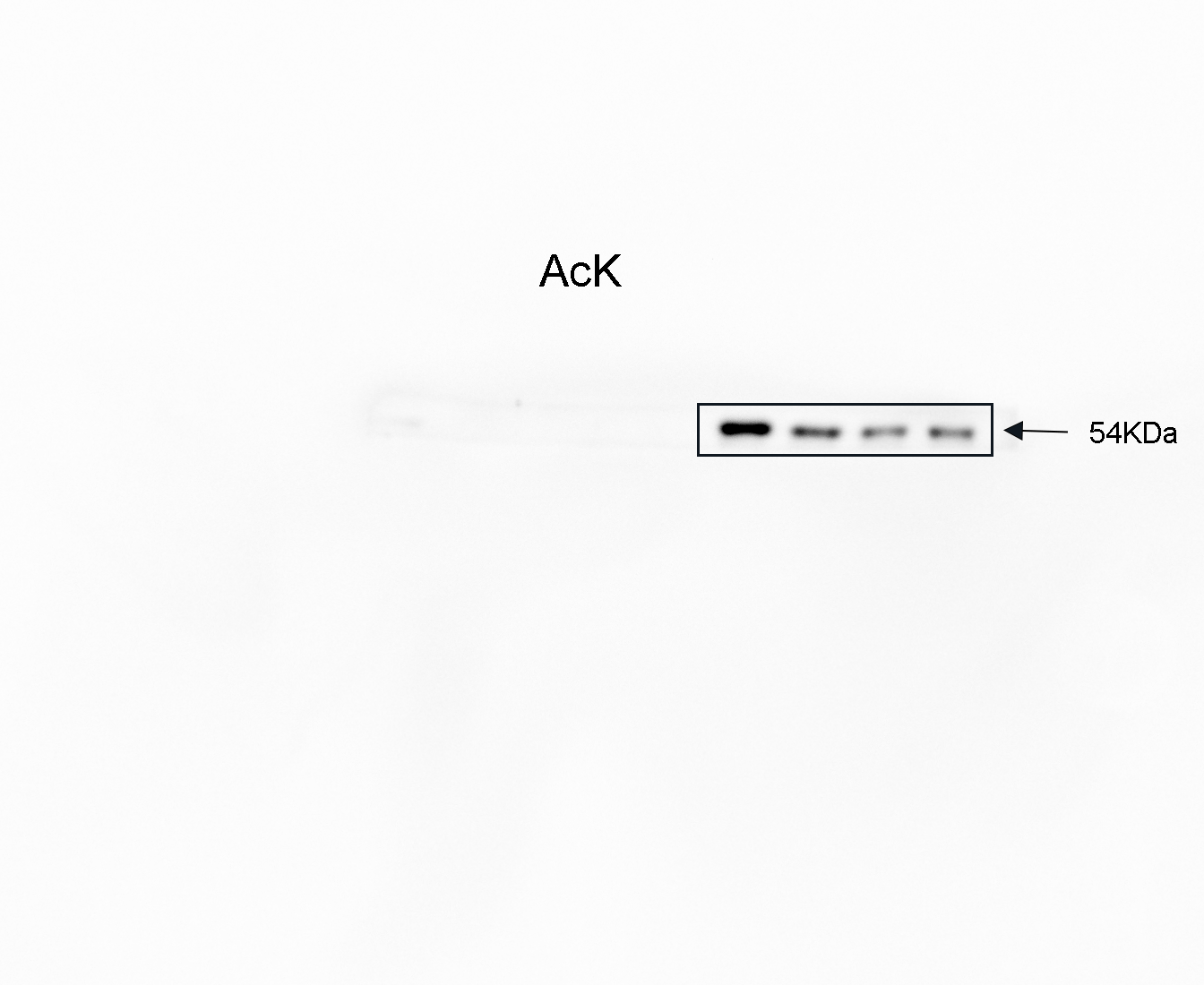

Supplement: Figure 4—source data 2. [file elife-98524-fig4-data2.zip › Fig 4-data2-v1/4H/2/upper/AcK .tif]

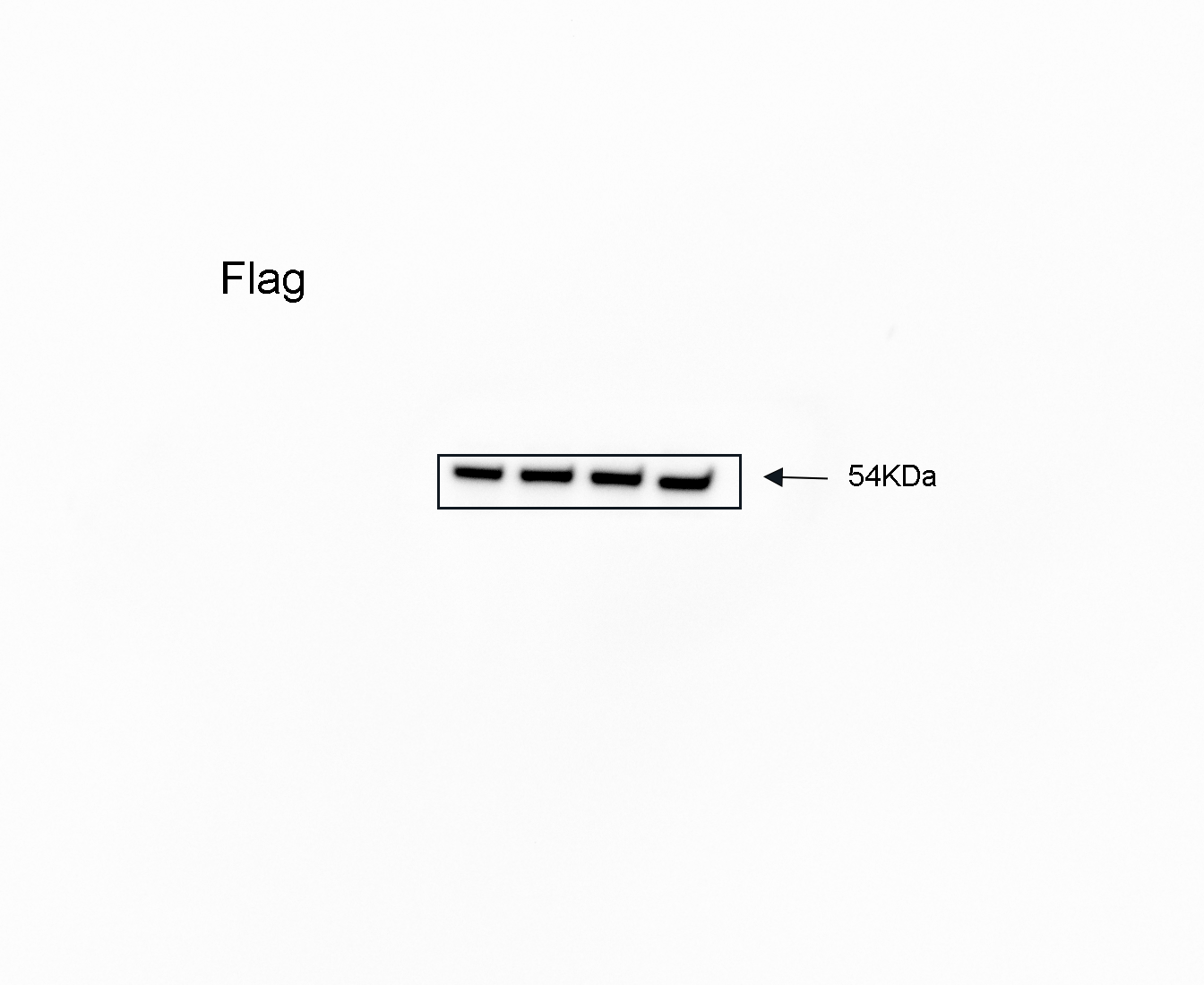

Supplement: Figure 4—source data 2. [file elife-98524-fig4-data2.zip › Fig 4-data2-v1/4H/2/upper/Flag .tif]

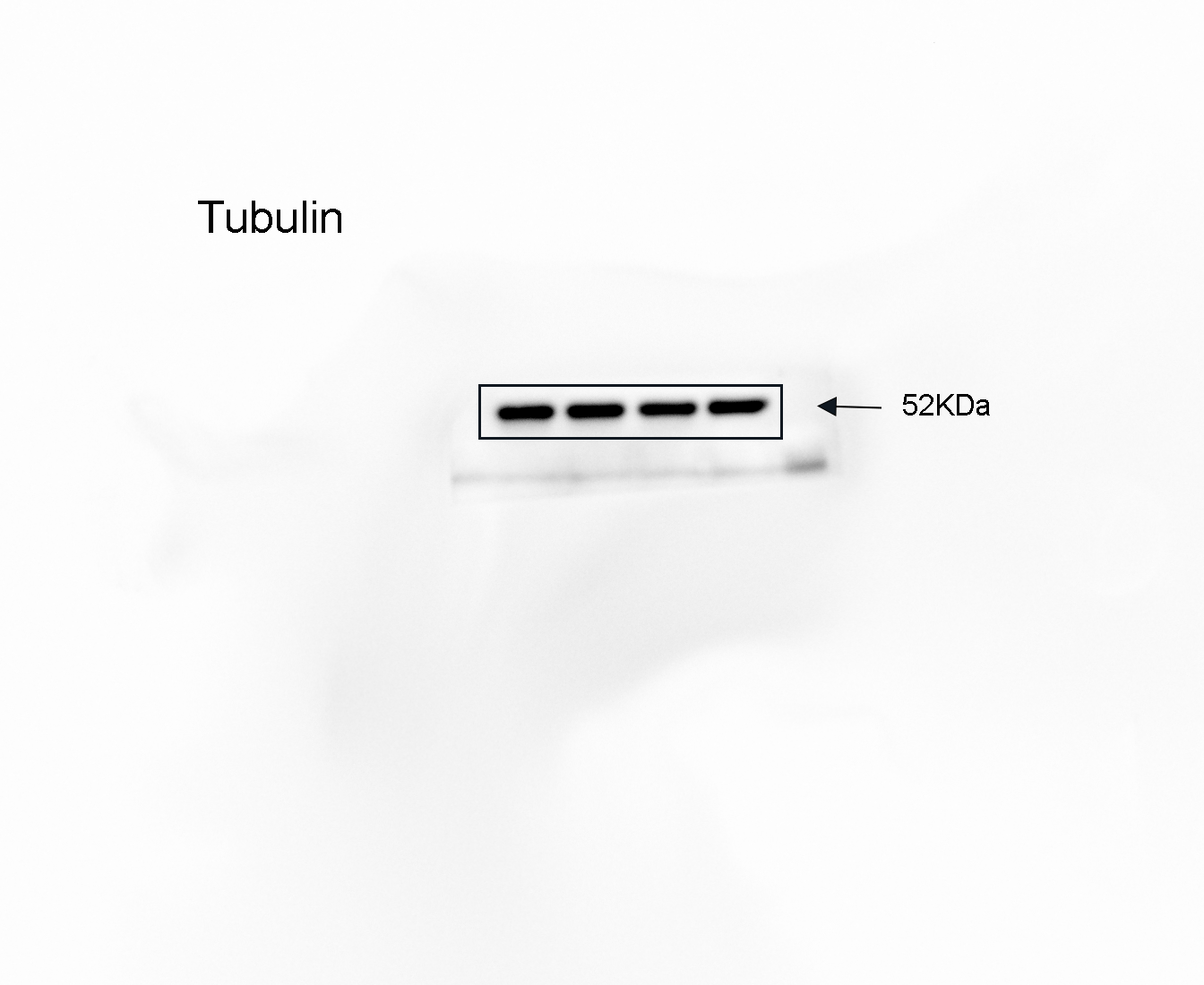

Supplement: Figure 4—source data 2. [file elife-98524-fig4-data2.zip › Fig 4-data2-v1/4I/1/bottom/Tubulin .tif]

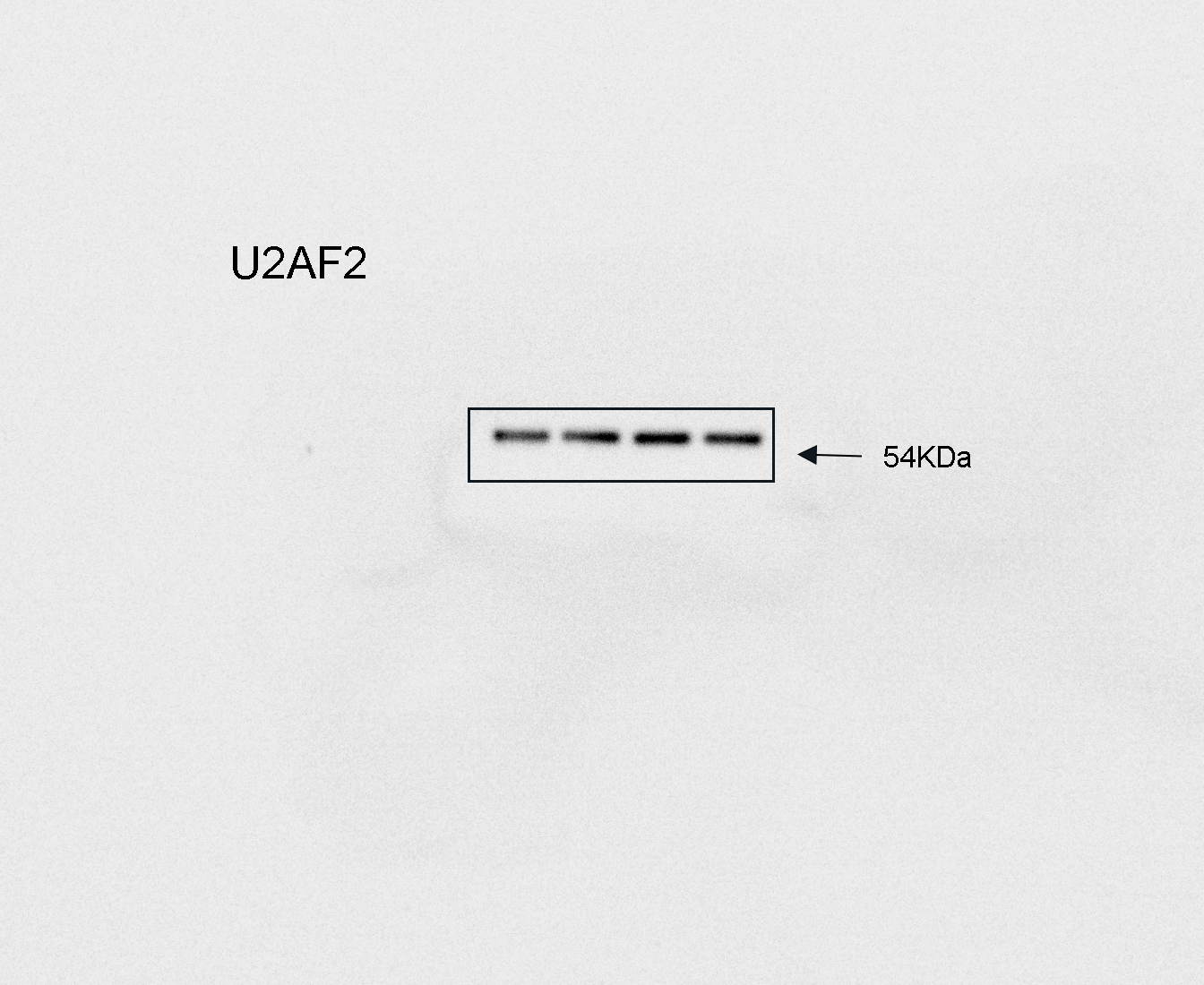

Supplement: Figure 4—source data 2. [file elife-98524-fig4-data2.zip › Fig 4-data2-v1/4I/1/bottom/U2AF2 .tif]

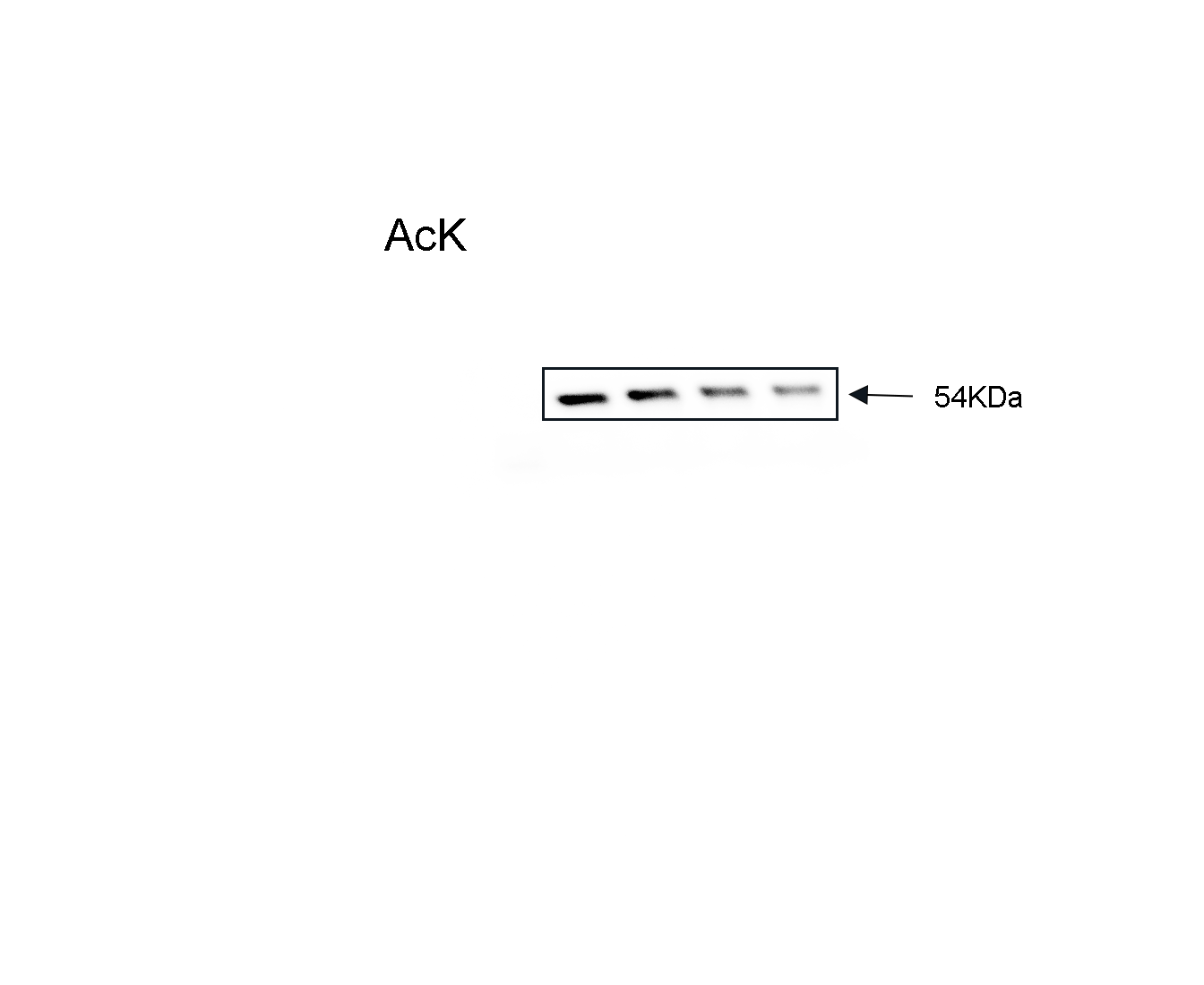

Supplement: Figure 4—source data 2. [file elife-98524-fig4-data2.zip › Fig 4-data2-v1/4I/1/upper/AcK .tif]

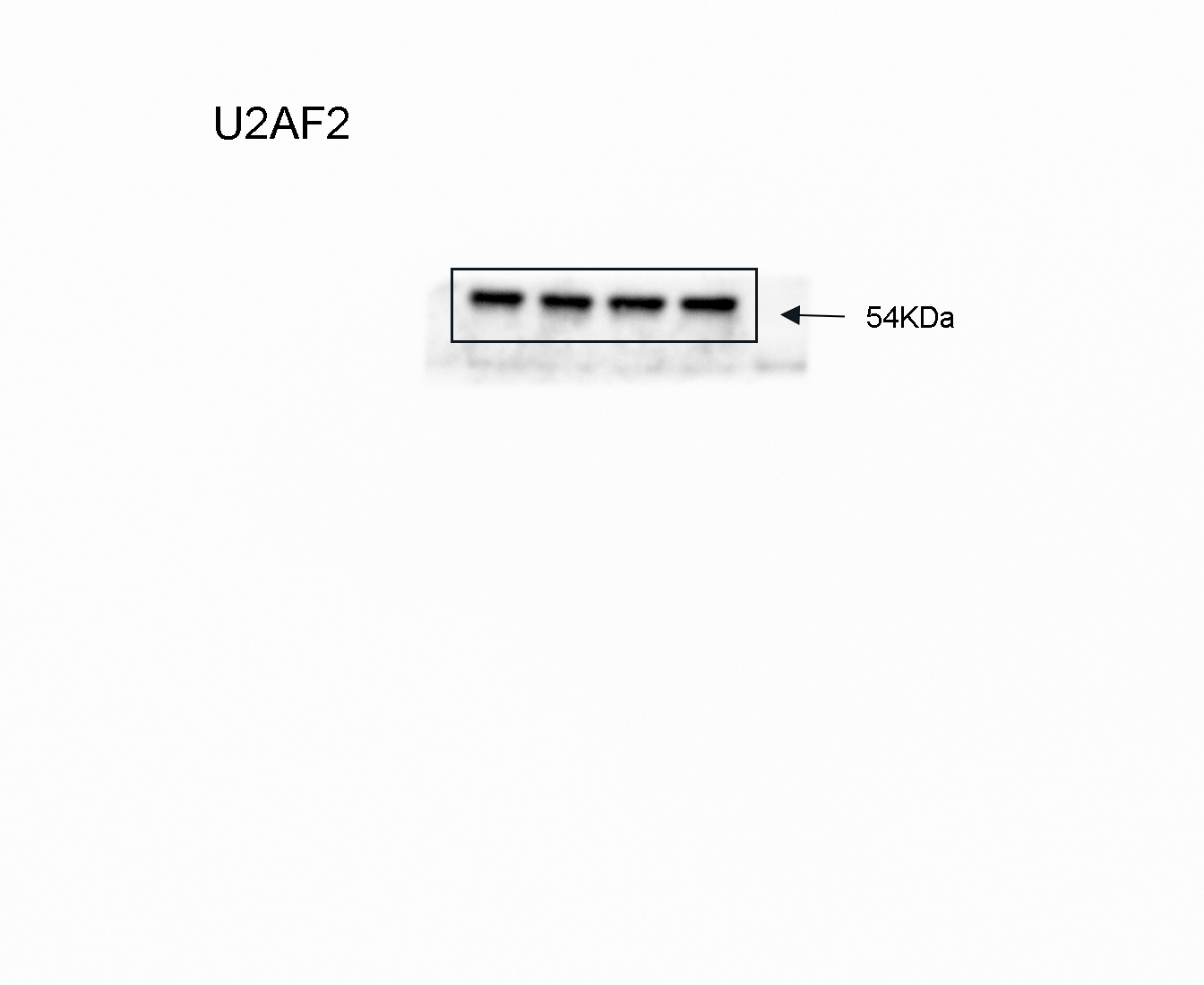

Supplement: Figure 4—source data 2. [file elife-98524-fig4-data2.zip › Fig 4-data2-v1/4I/1/upper/U2AF2 .tif]

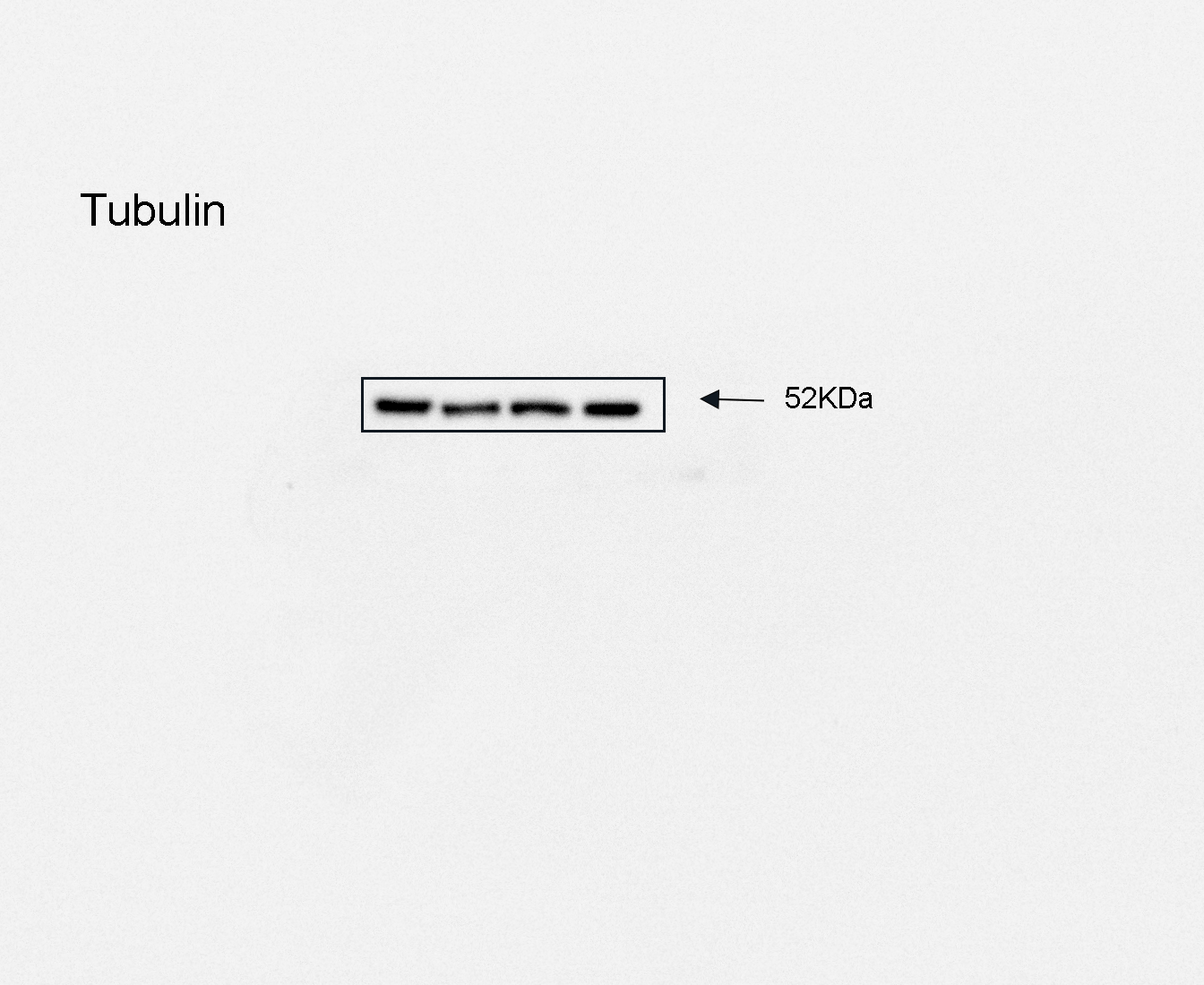

Supplement: Figure 4—source data 2. [file elife-98524-fig4-data2.zip › Fig 4-data2-v1/4I/2/bottom/Tubulin.tif]

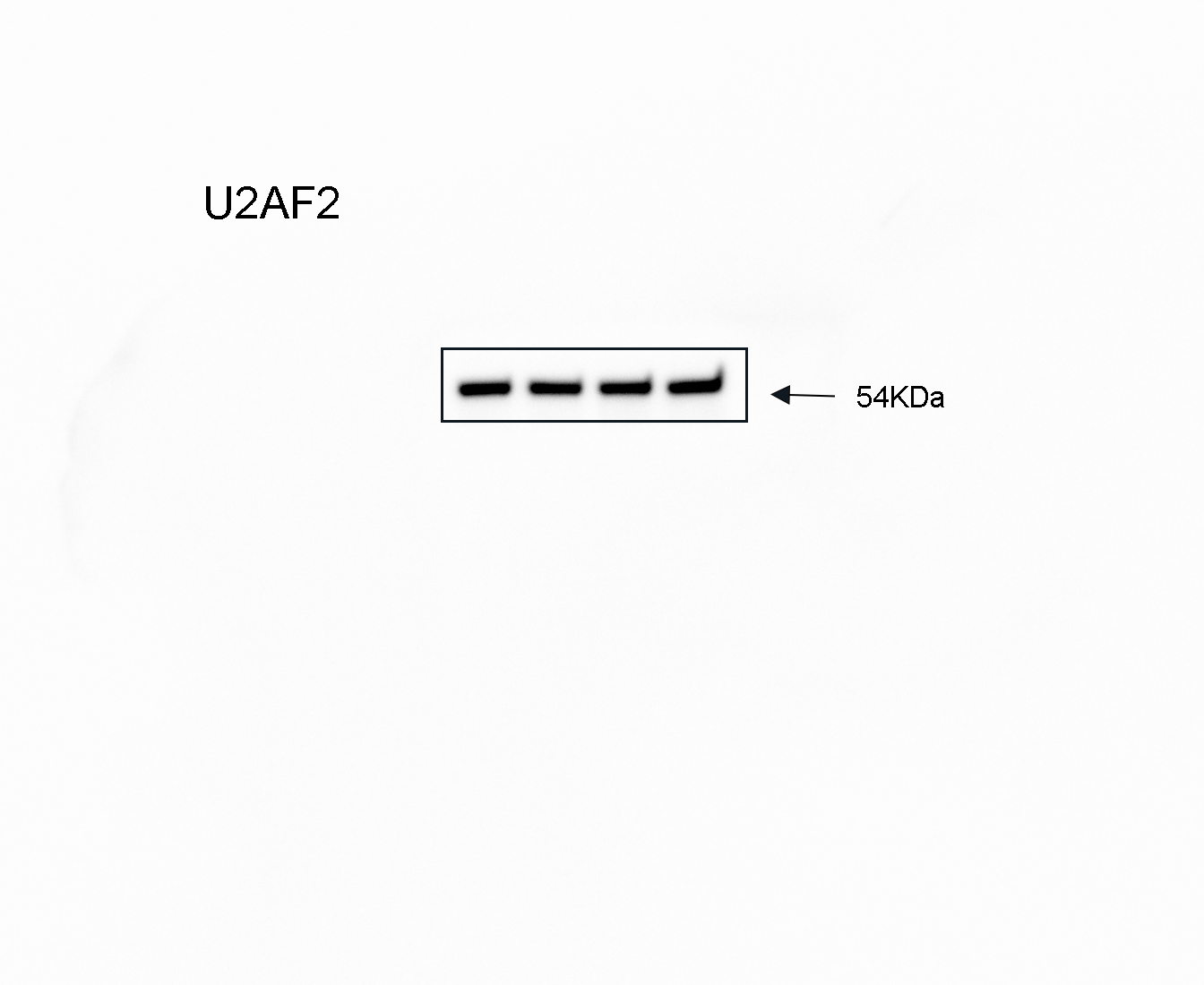

Supplement: Figure 4—source data 2. [file elife-98524-fig4-data2.zip › Fig 4-data2-v1/4I/2/bottom/U2AF2.tif]

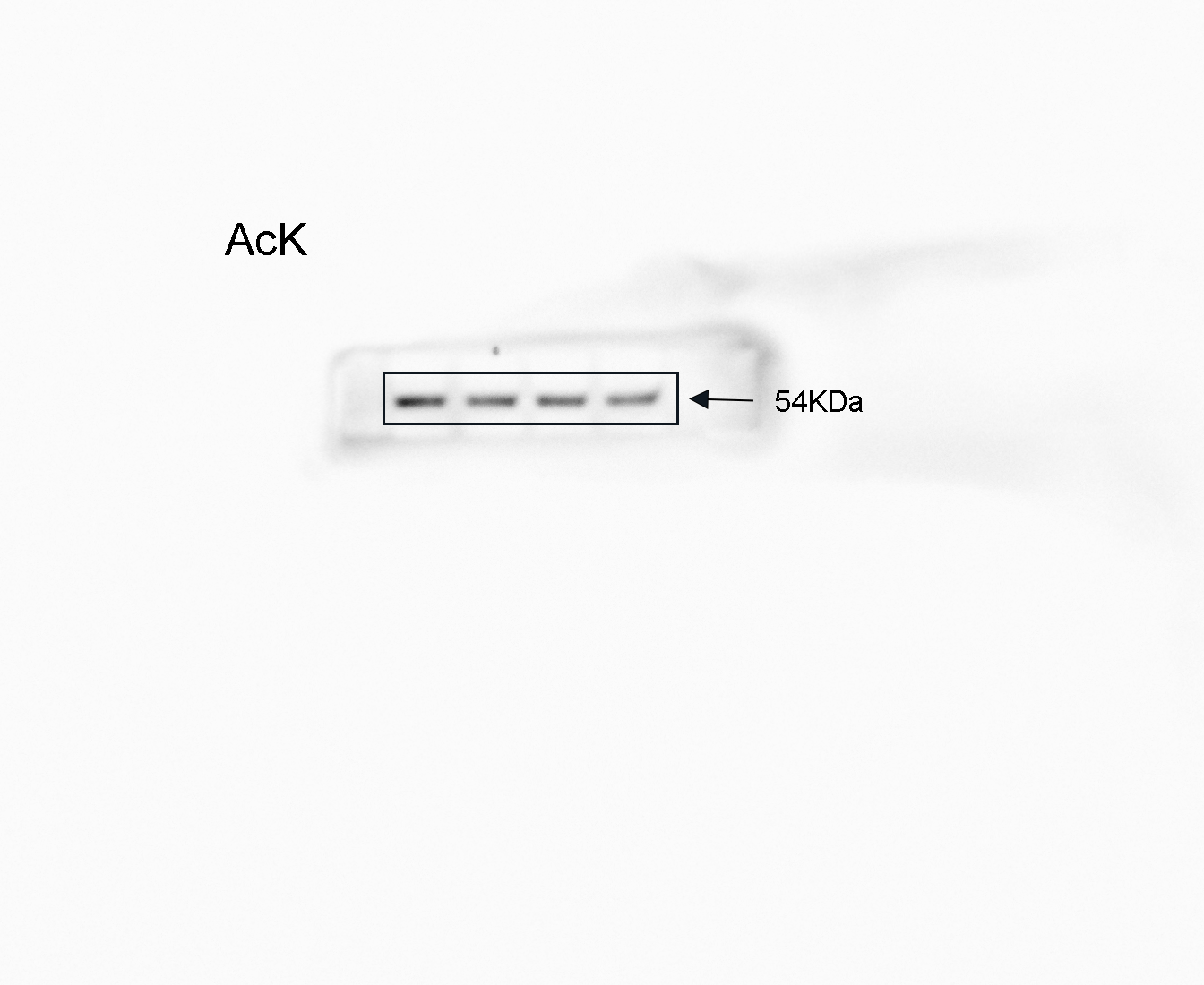

Supplement: Figure 4—source data 2. [file elife-98524-fig4-data2.zip › Fig 4-data2-v1/4I/2/upper/AcK.tif]

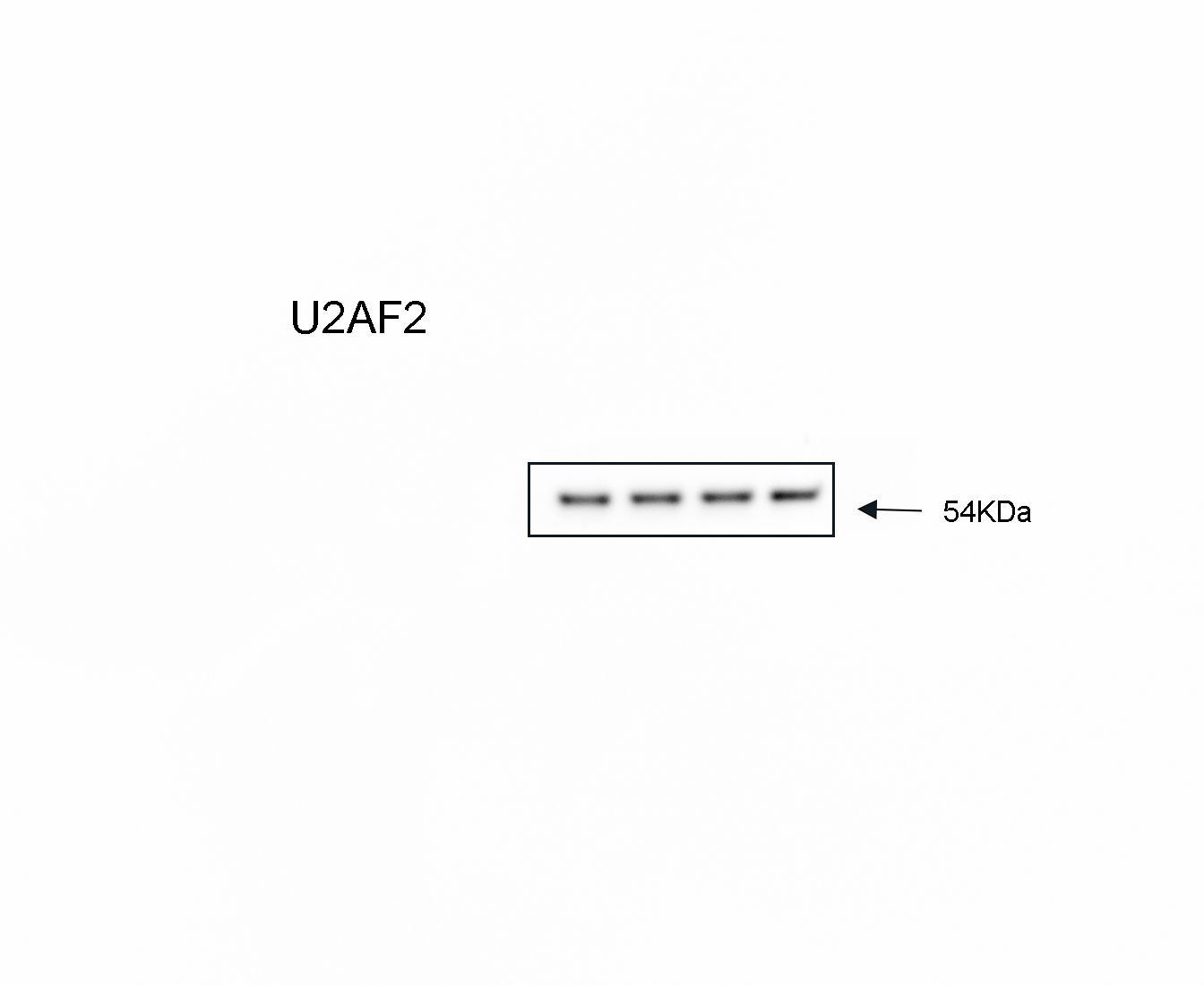

Supplement: Figure 4—source data 2. [file elife-98524-fig4-data2.zip › Fig 4-data2-v1/4I/2/upper/U2AF2.tif]

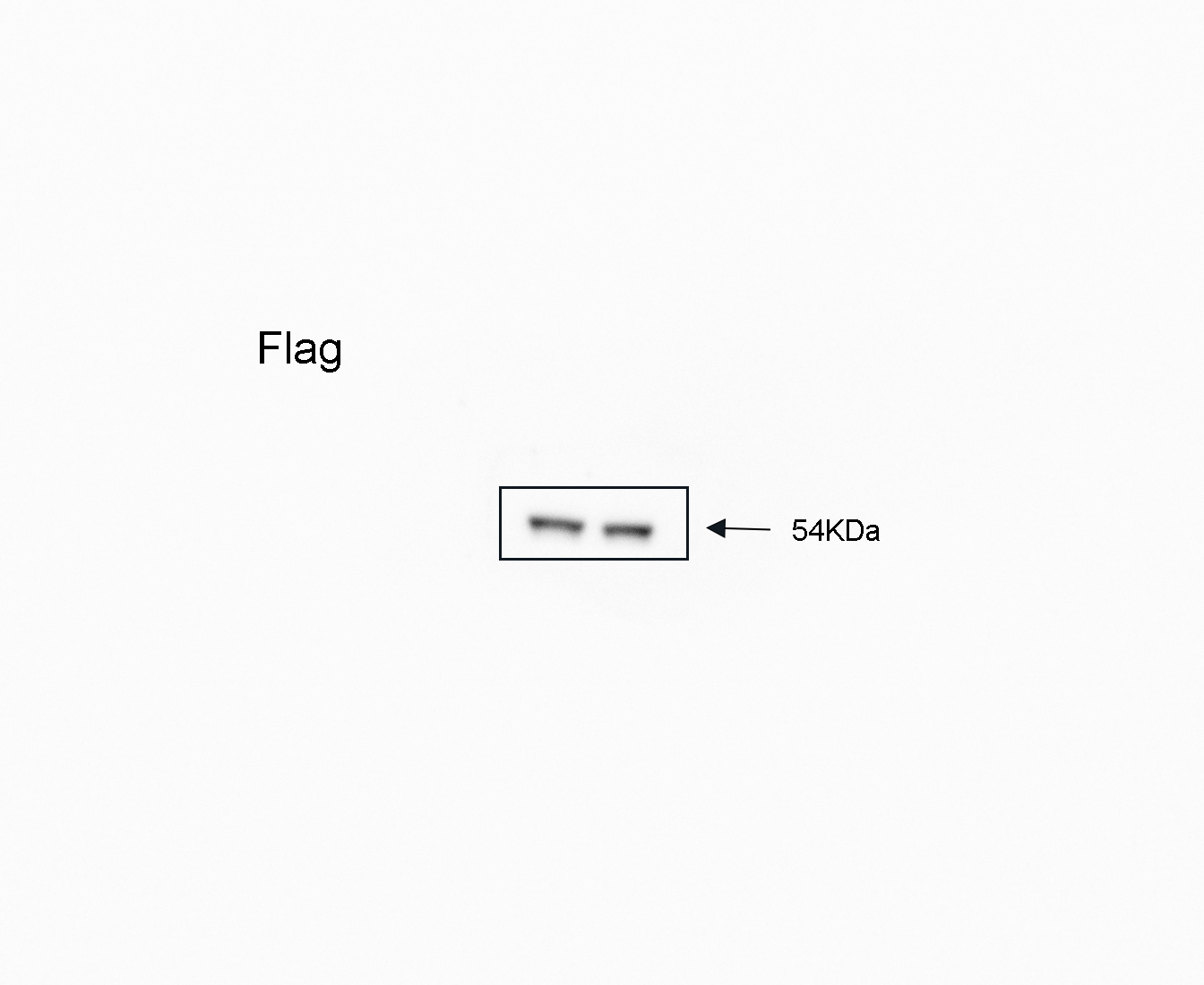

Supplement: Figure 4—source data 2. [file elife-98524-fig4-data2.zip › Fig 4-data2-v1/4J/1-Serum Starv/bottom/Flag .tif]

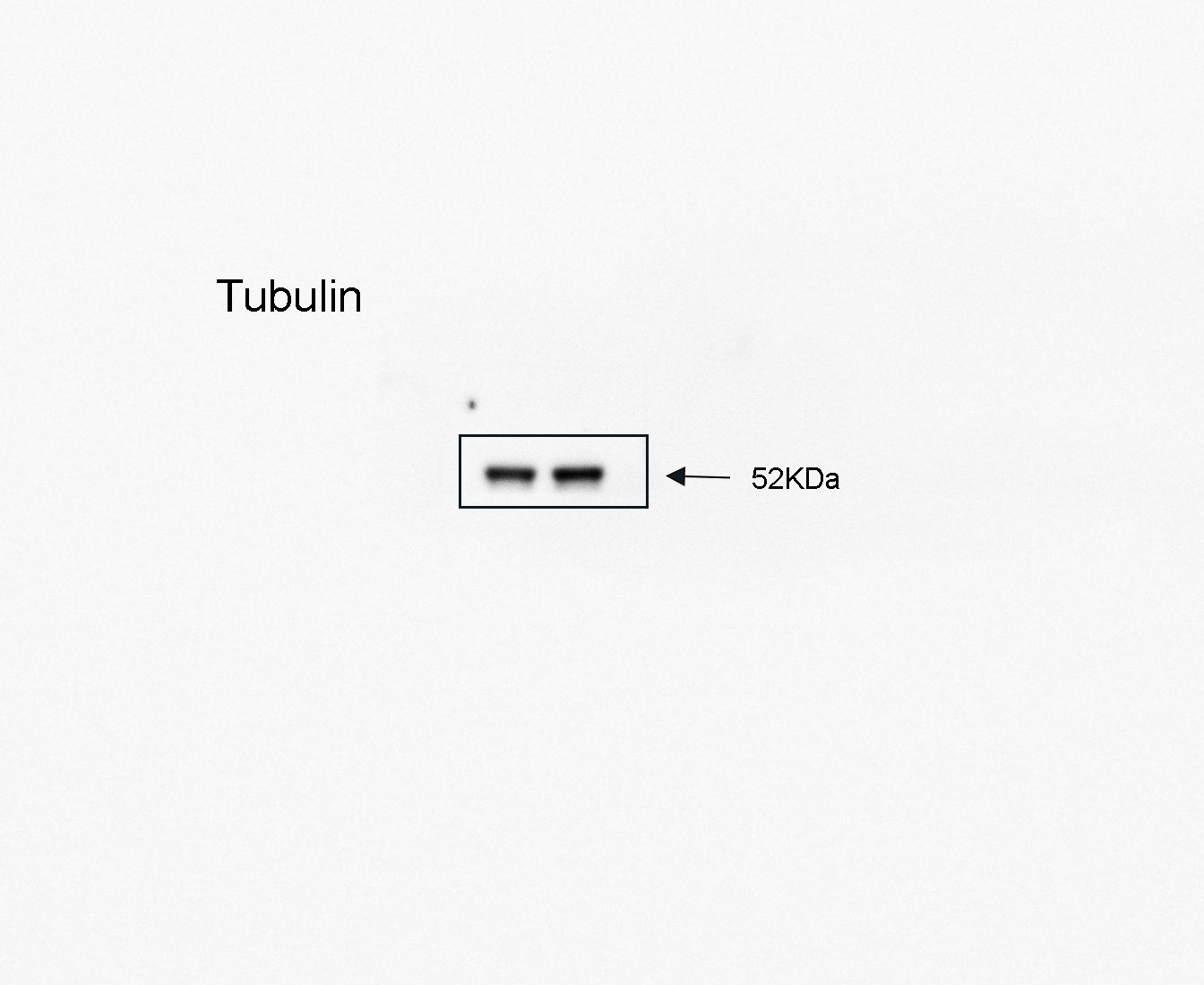

Supplement: Figure 4—source data 2. [file elife-98524-fig4-data2.zip › Fig 4-data2-v1/4J/1-Serum Starv/bottom/Tubulin .tif]

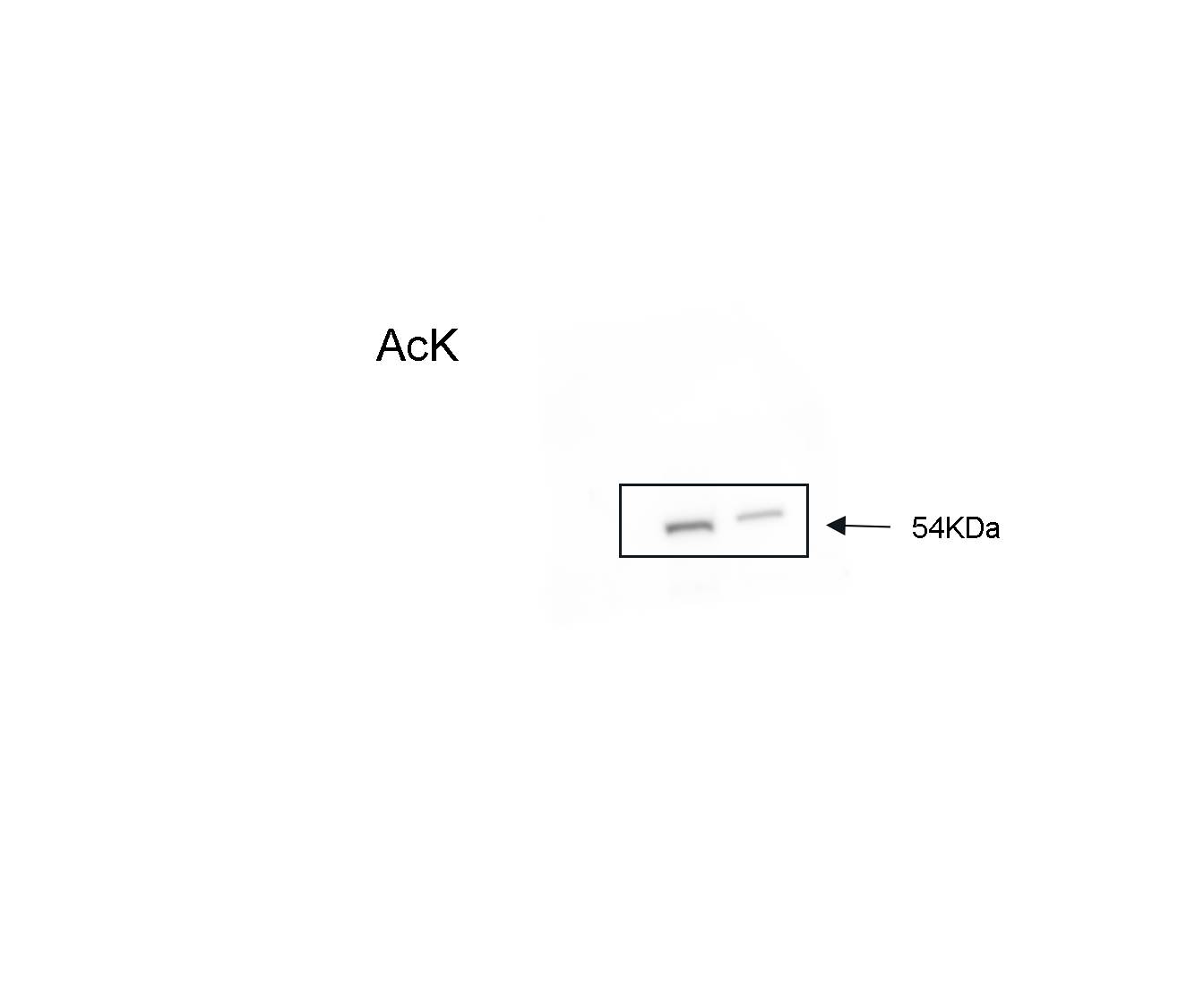

Supplement: Figure 4—source data 2. [file elife-98524-fig4-data2.zip › Fig 4-data2-v1/4J/1-Serum Starv/upper/AcK .tif]

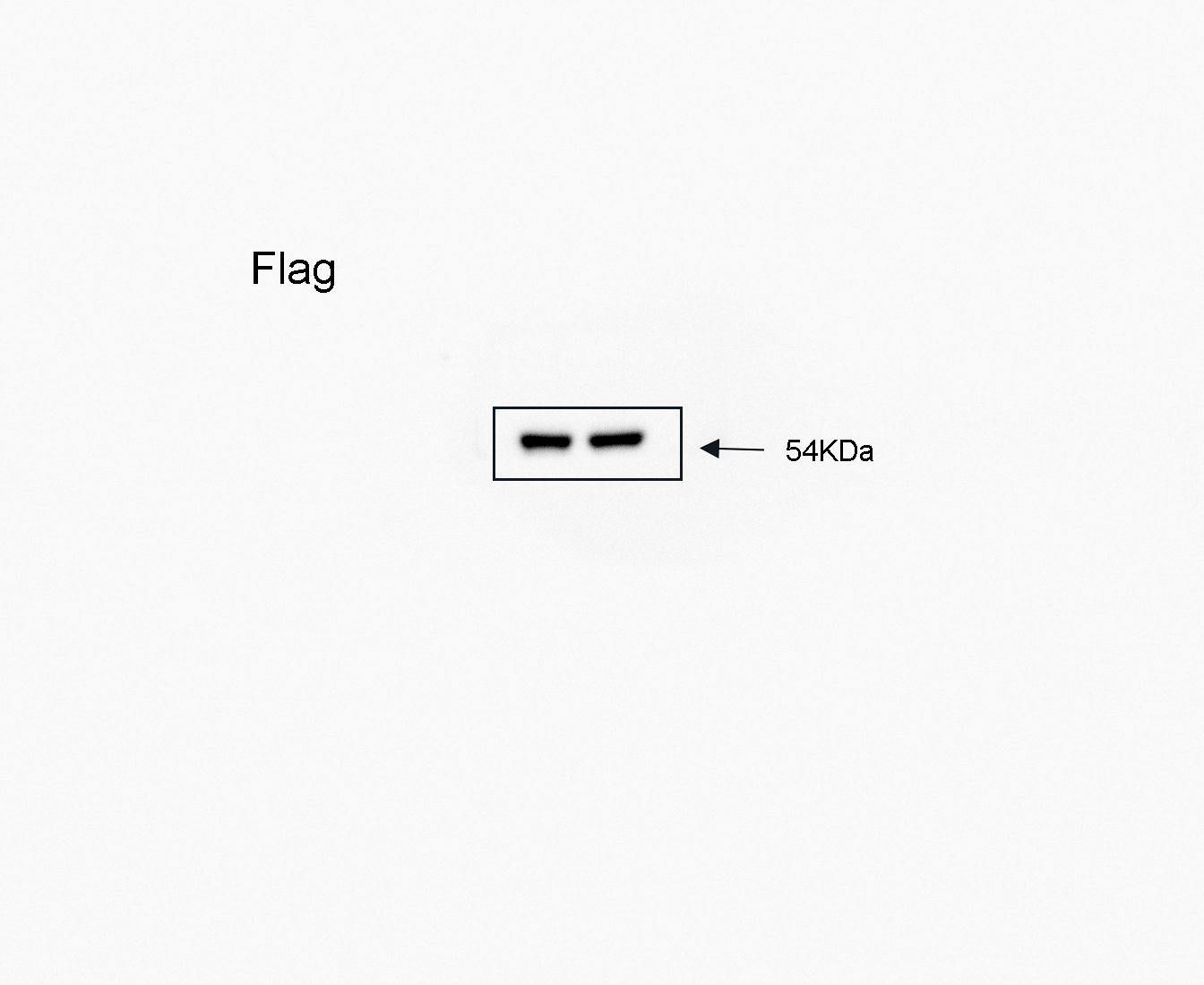

Supplement: Figure 4—source data 2. [file elife-98524-fig4-data2.zip › Fig 4-data2-v1/4J/1-Serum Starv/upper/Flag .tif]

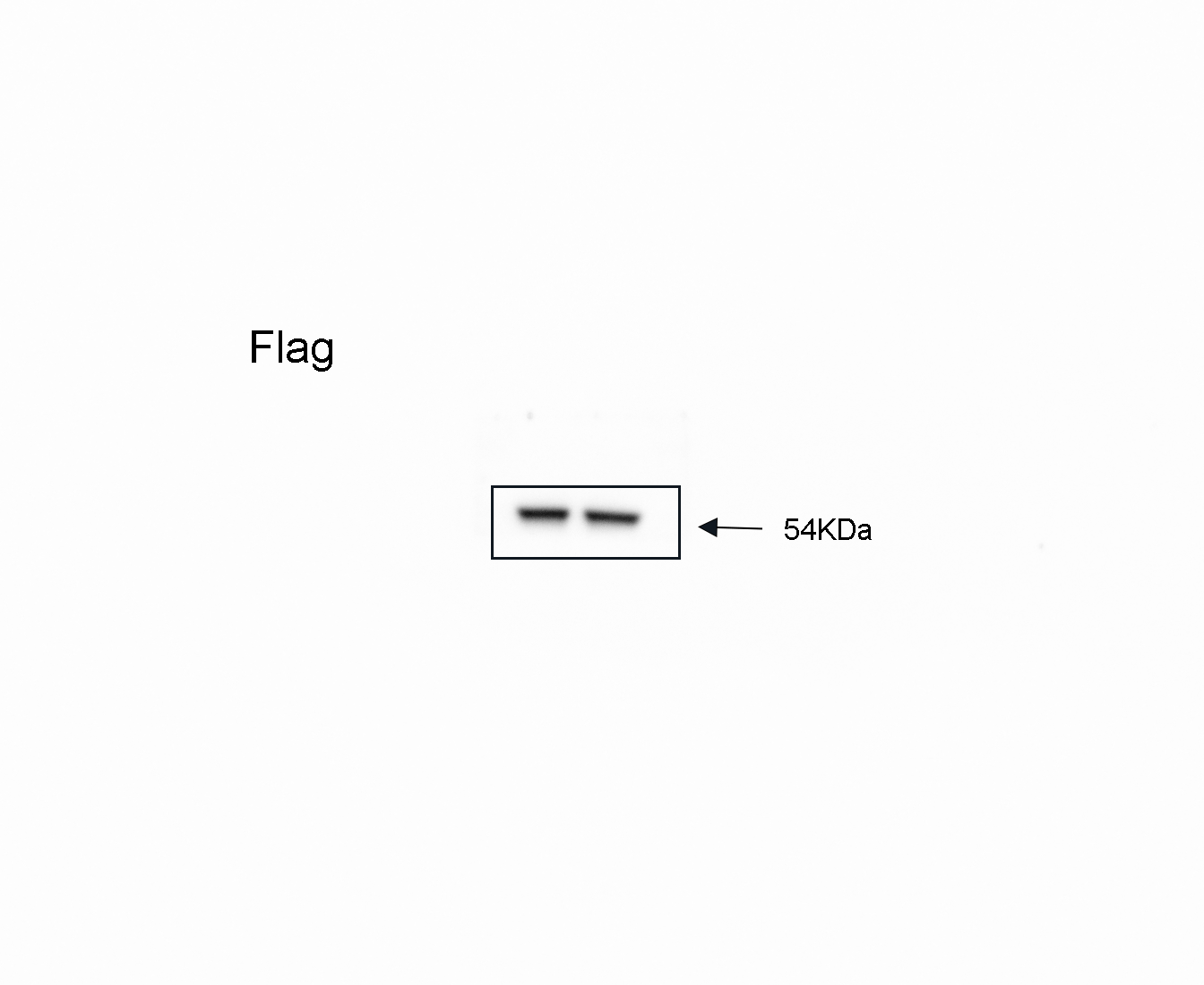

Supplement: Figure 4—source data 2. [file elife-98524-fig4-data2.zip › Fig 4-data2-v1/4J/2-Tunicamycin/bottom/Flag.tif]

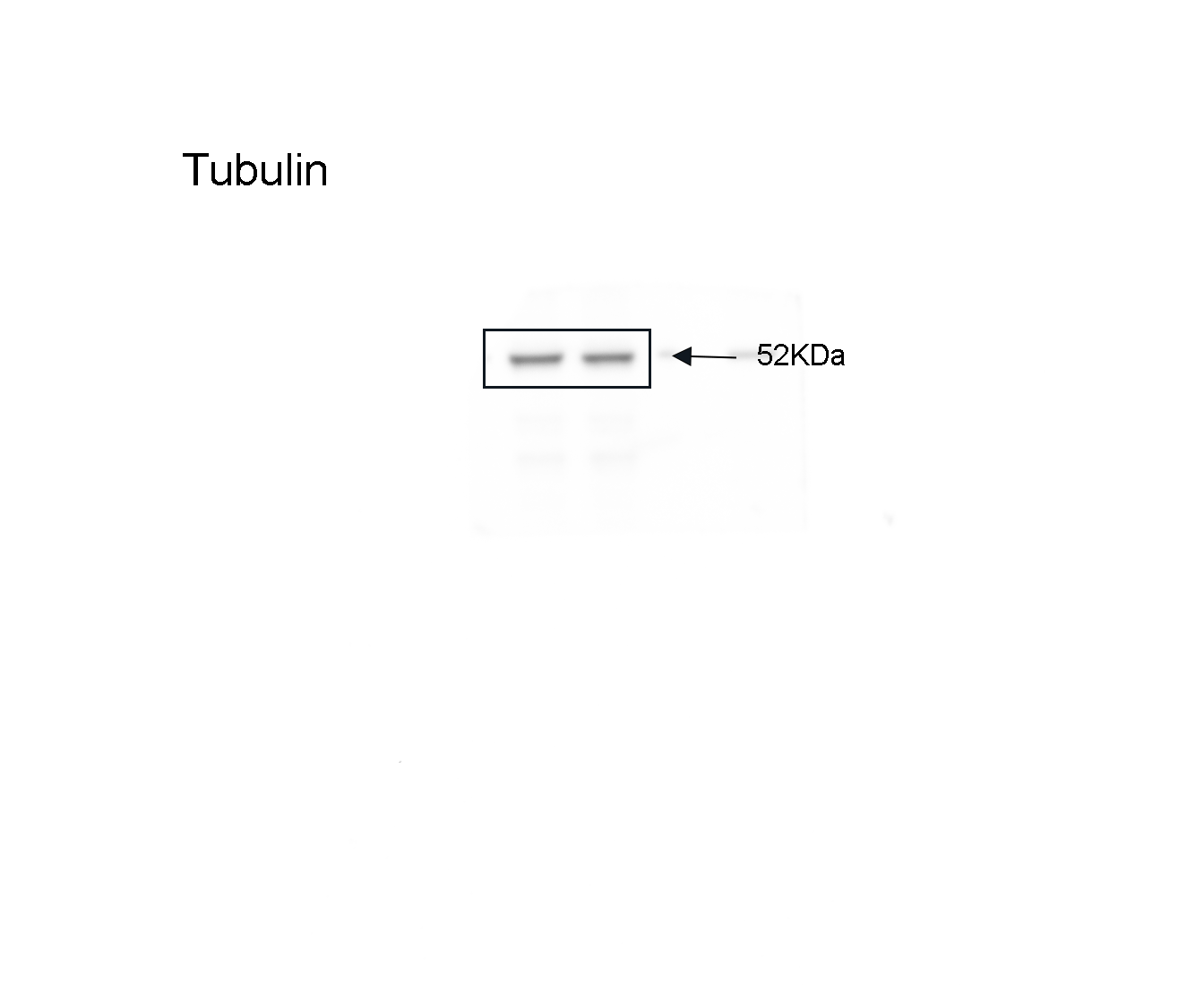

Supplement: Figure 4—source data 2. [file elife-98524-fig4-data2.zip › Fig 4-data2-v1/4J/2-Tunicamycin/bottom/Tubulin.tif]

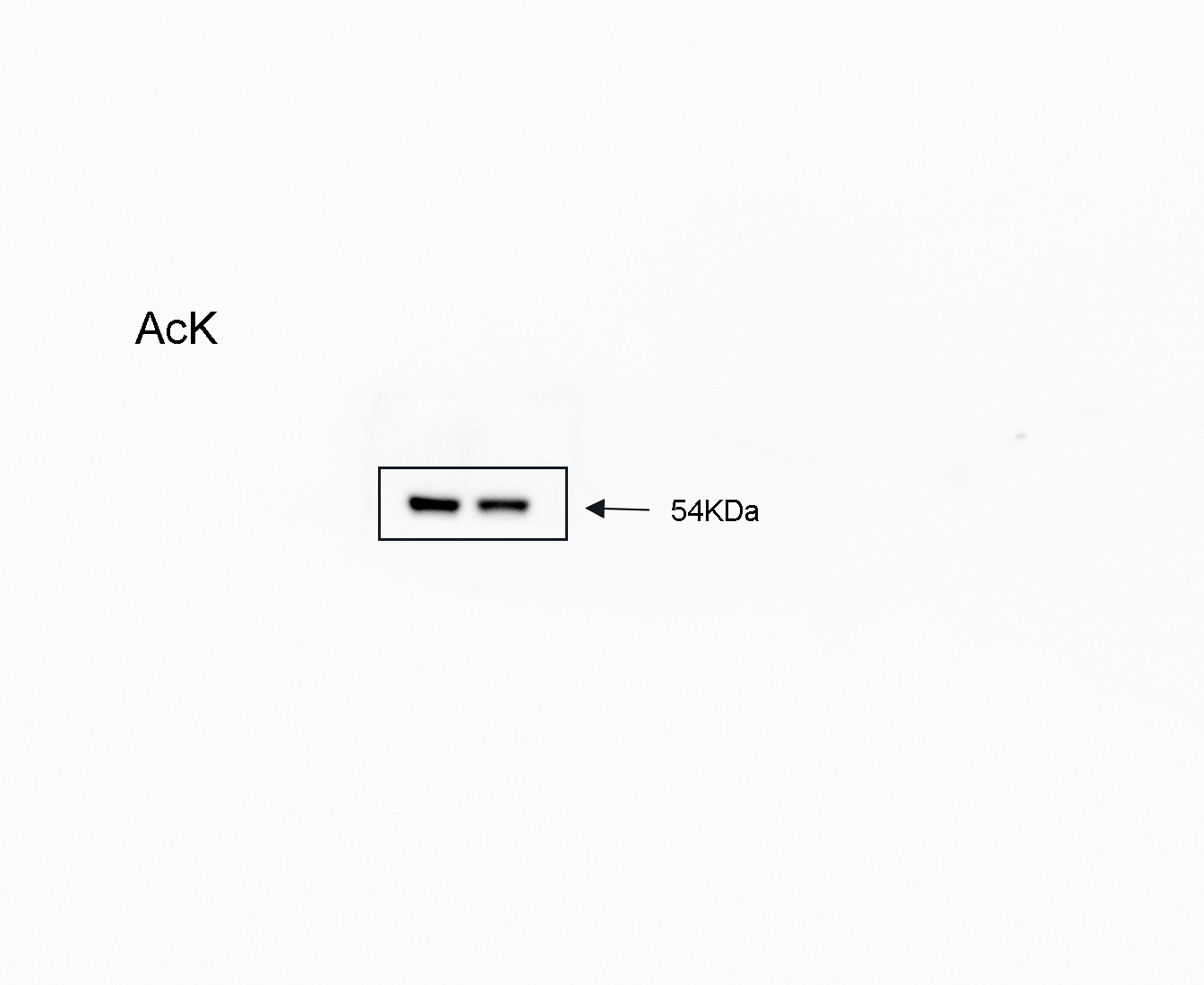

Supplement: Figure 4—source data 2. [file elife-98524-fig4-data2.zip › Fig 4-data2-v1/4J/2-Tunicamycin/upper/AcK.tif]

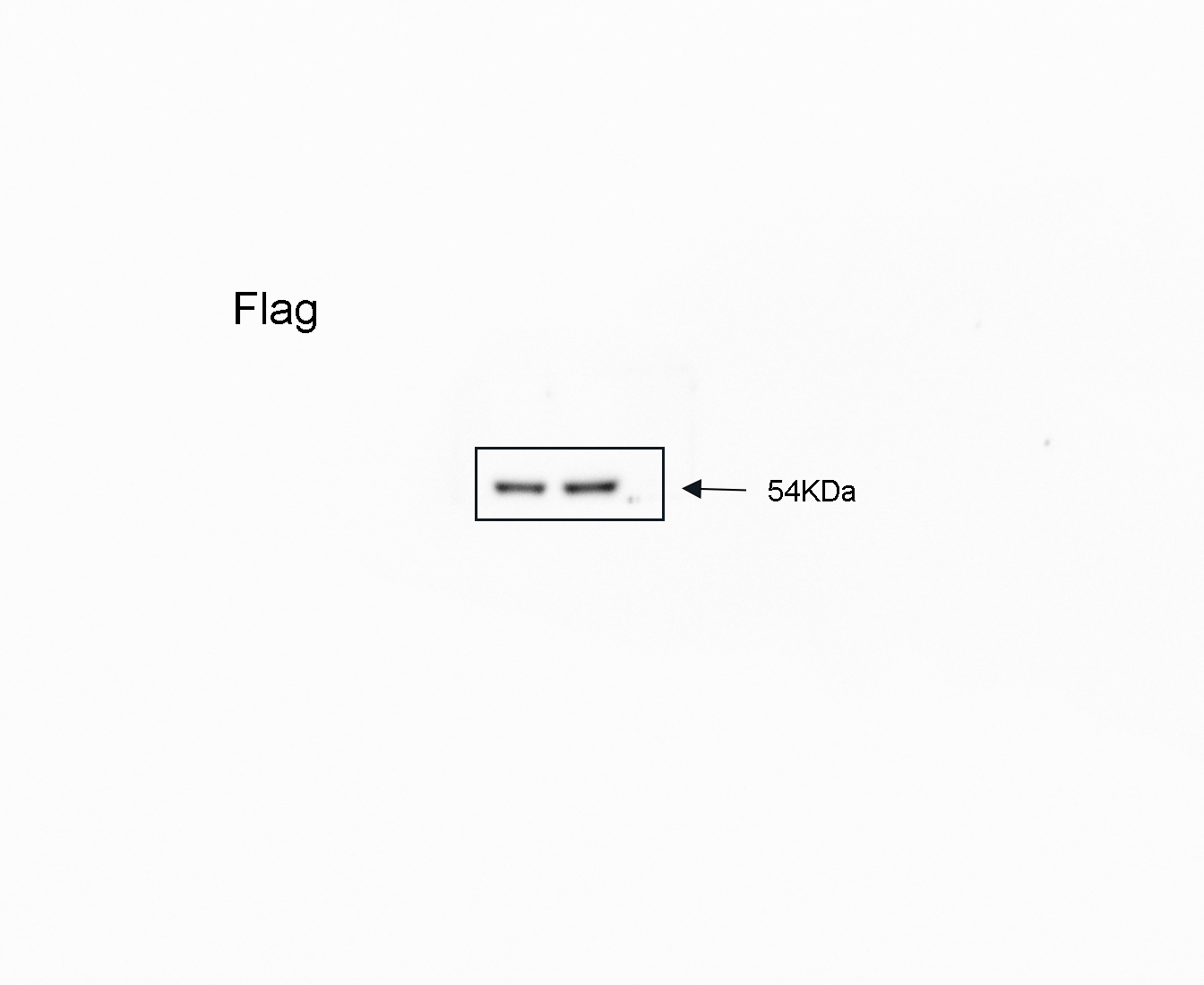

Supplement: Figure 4—source data 2. [file elife-98524-fig4-data2.zip › Fig 4-data2-v1/4J/2-Tunicamycin/upper/Flag.tif]

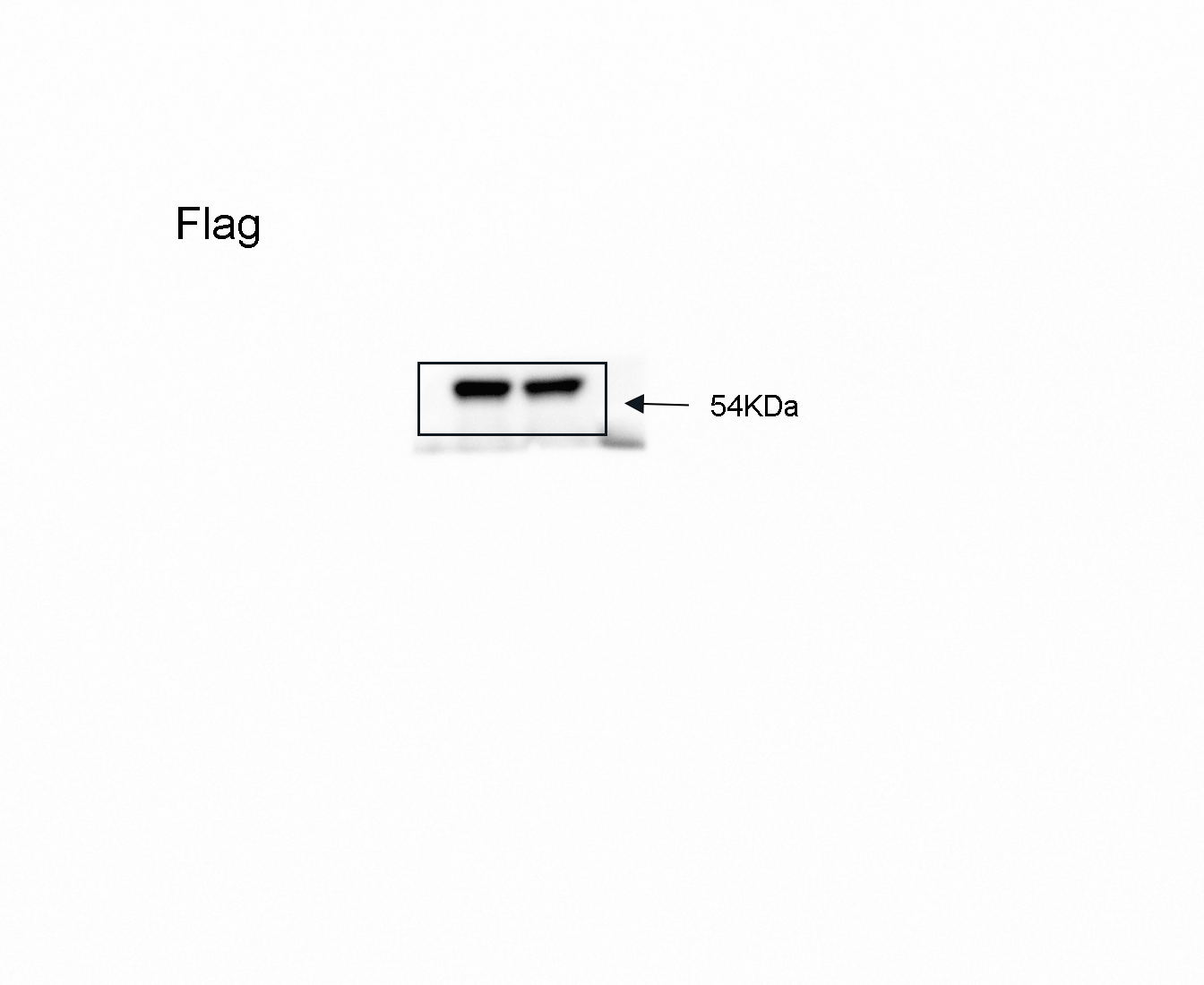

Supplement: Figure 4—source data 2. [file elife-98524-fig4-data2.zip › Fig 4-data2-v1/4J/3-Poly/bottom/Flag .tif]

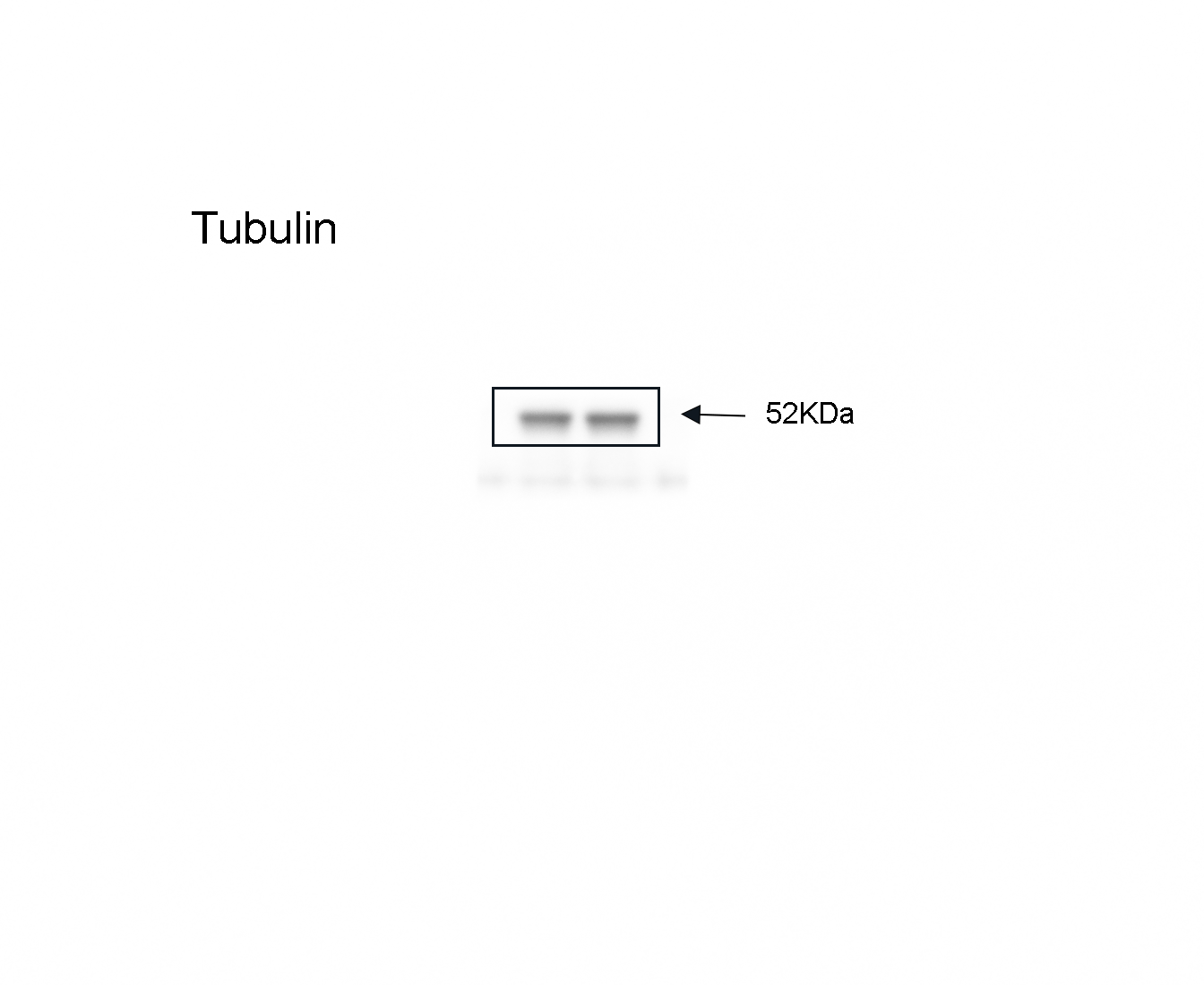

Supplement: Figure 4—source data 2. [file elife-98524-fig4-data2.zip › Fig 4-data2-v1/4J/3-Poly/bottom/Tubulin .tif]

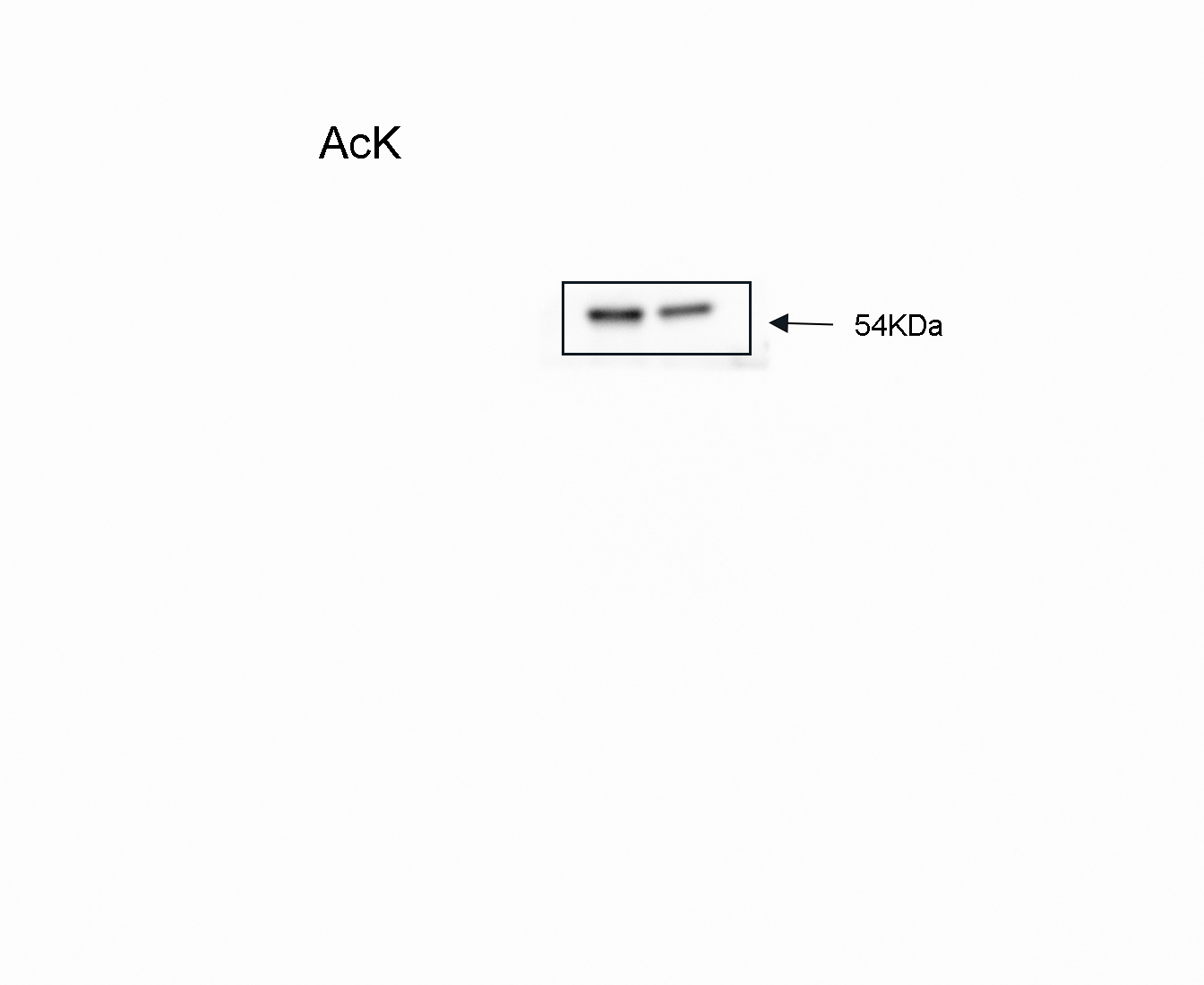

Supplement: Figure 4—source data 2. [file elife-98524-fig4-data2.zip › Fig 4-data2-v1/4J/3-Poly/upper/AcK .tif]

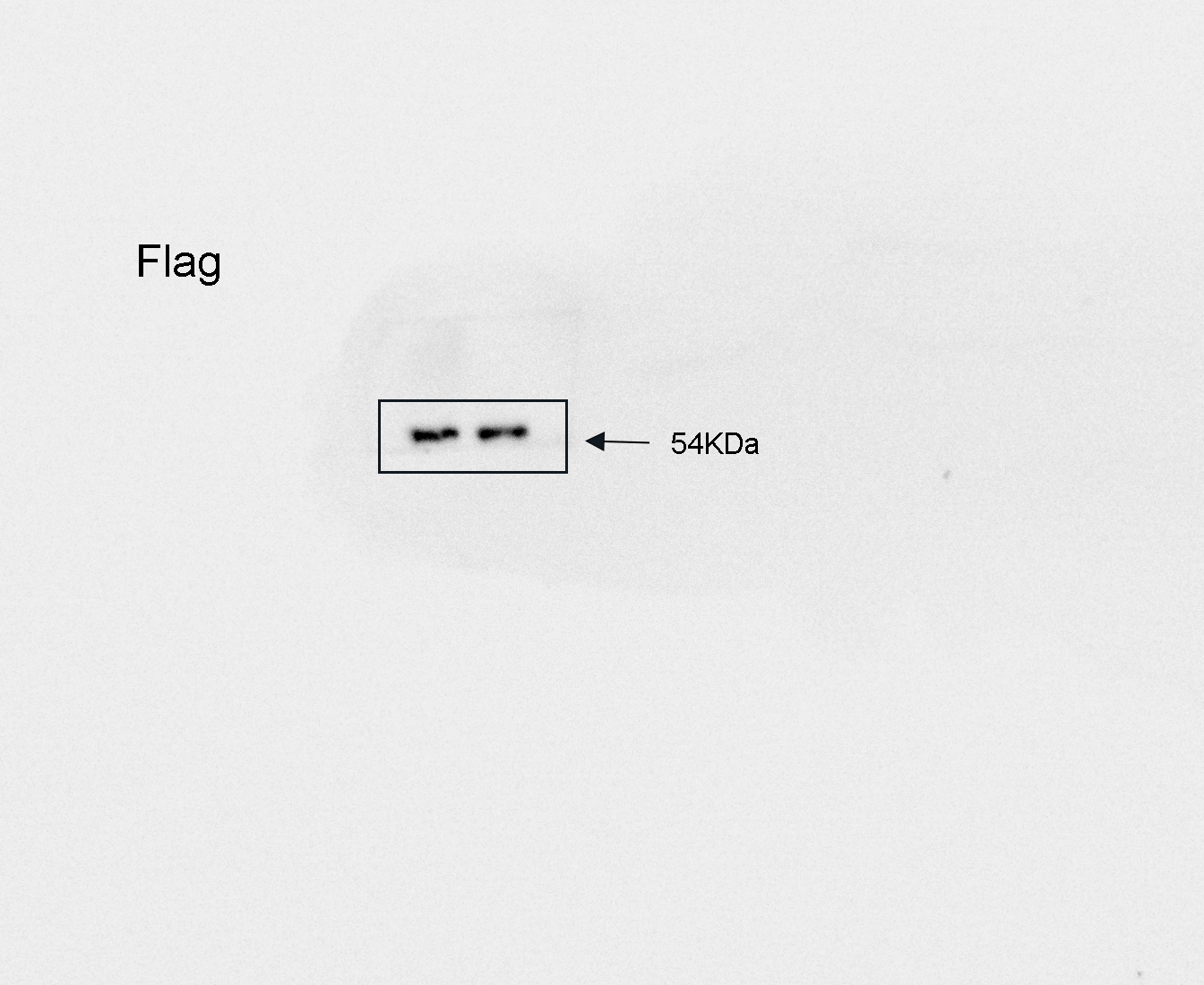

Supplement: Figure 4—source data 2. [file elife-98524-fig4-data2.zip › Fig 4-data2-v1/4J/3-Poly/upper/Flag .tif]

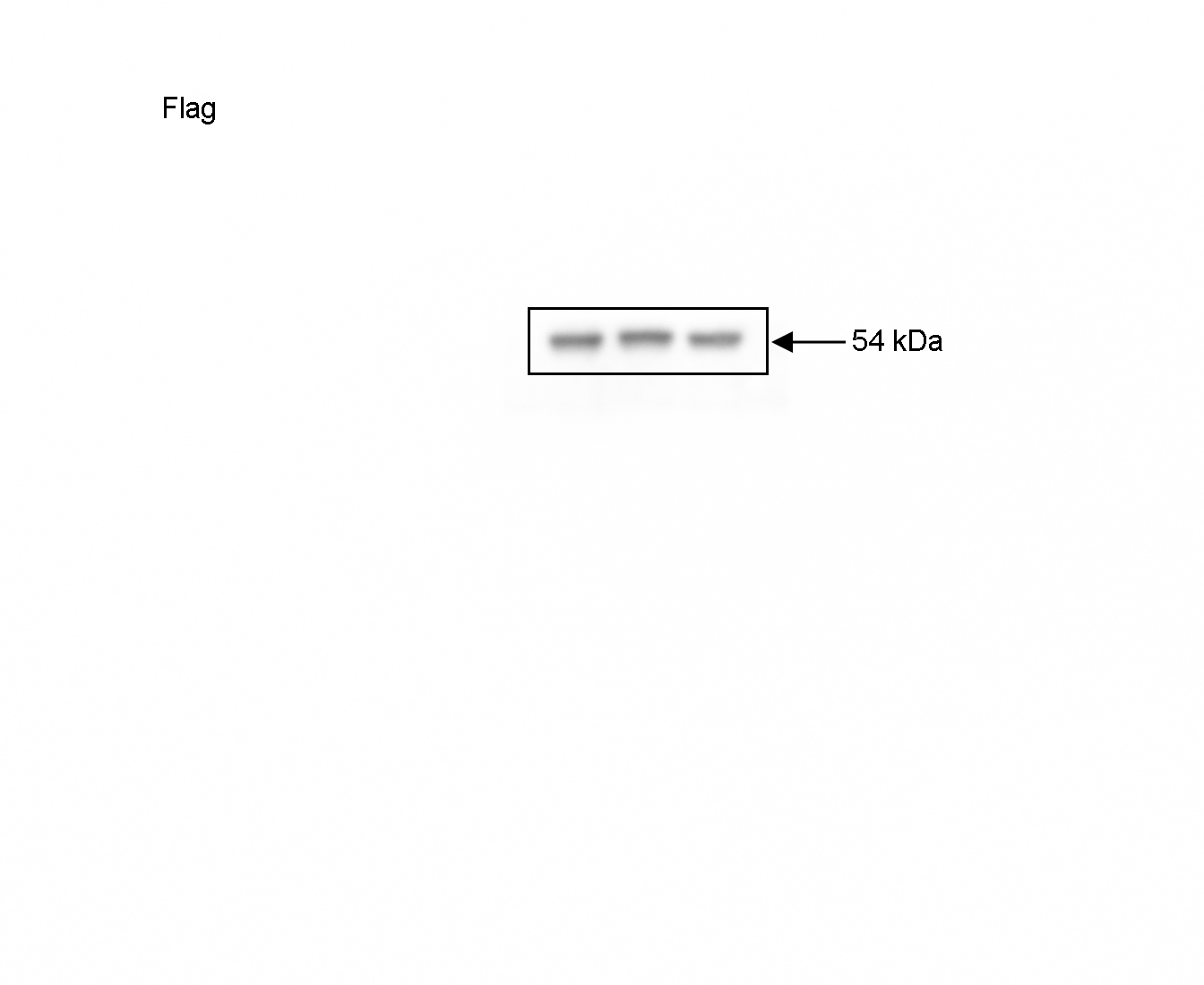

Supplement: Figure 4—source data 2. [file elife-98524-fig4-data2.zip › Fig 4-data2-v1/4J/4-Cisplatin/bottom/Flag.tif]

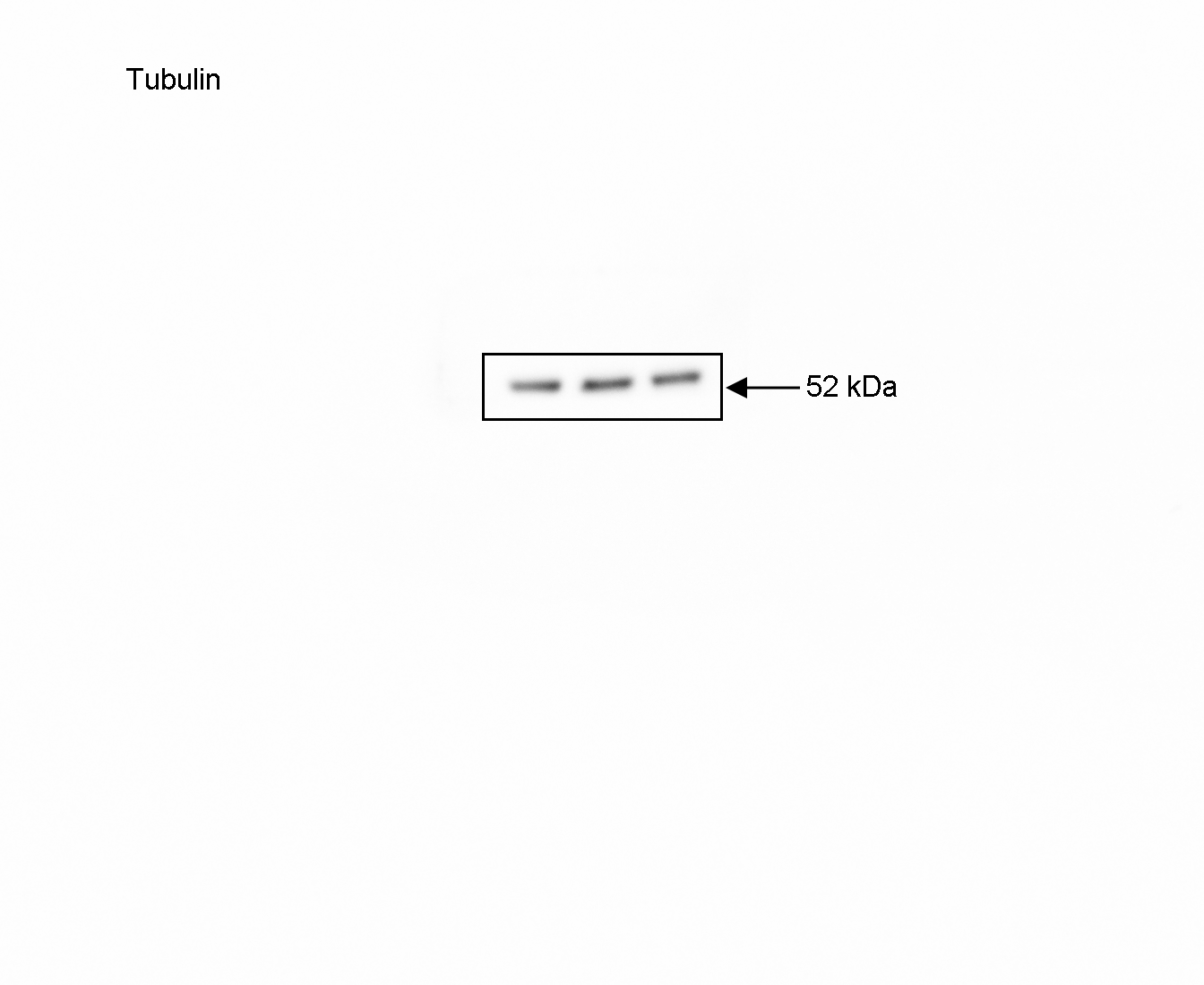

Supplement: Figure 4—source data 2. [file elife-98524-fig4-data2.zip › Fig 4-data2-v1/4J/4-Cisplatin/bottom/Tubulin.tif]

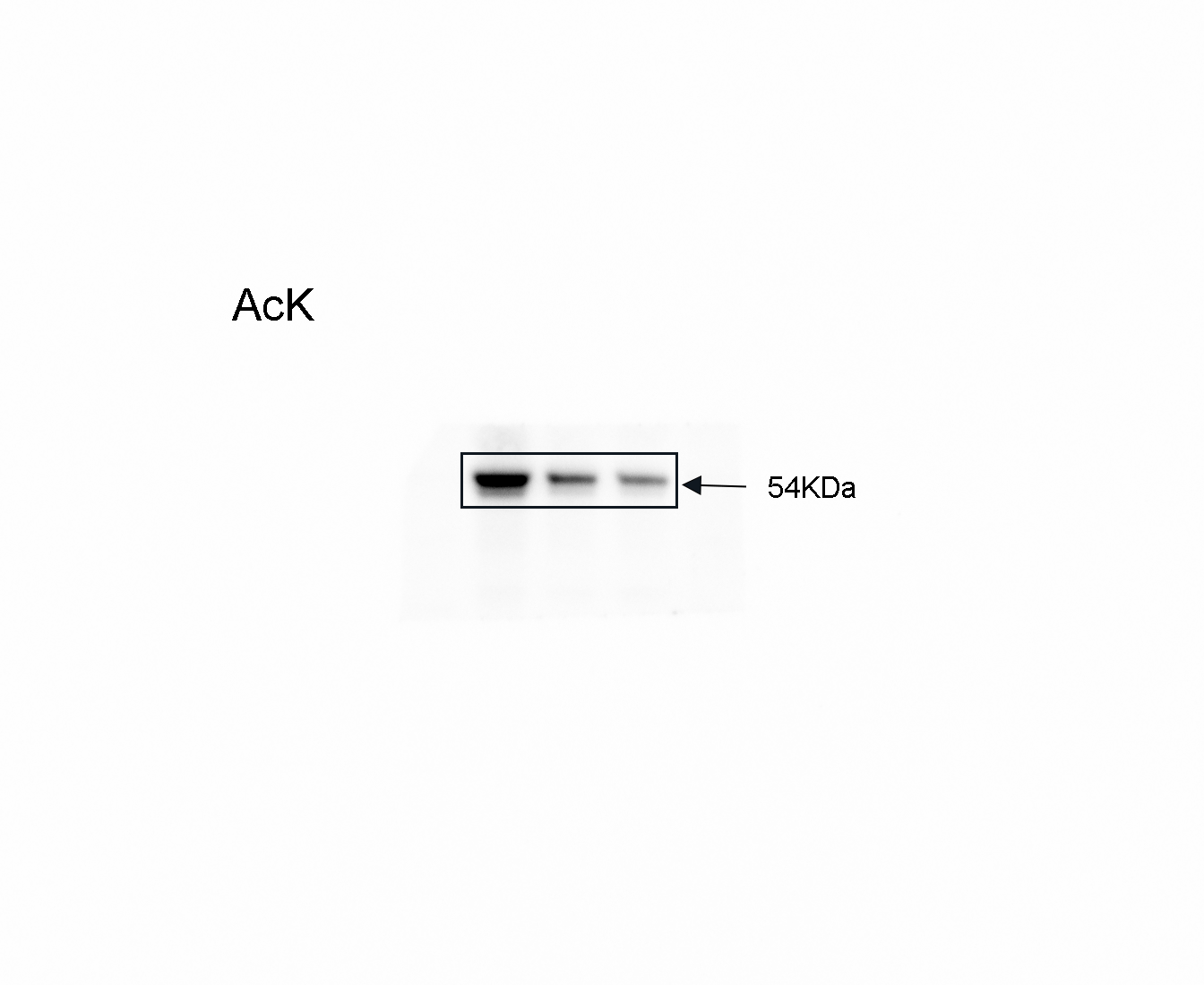

Supplement: Figure 4—source data 2. [file elife-98524-fig4-data2.zip › Fig 4-data2-v1/4J/4-Cisplatin/upper/Ack .tif]

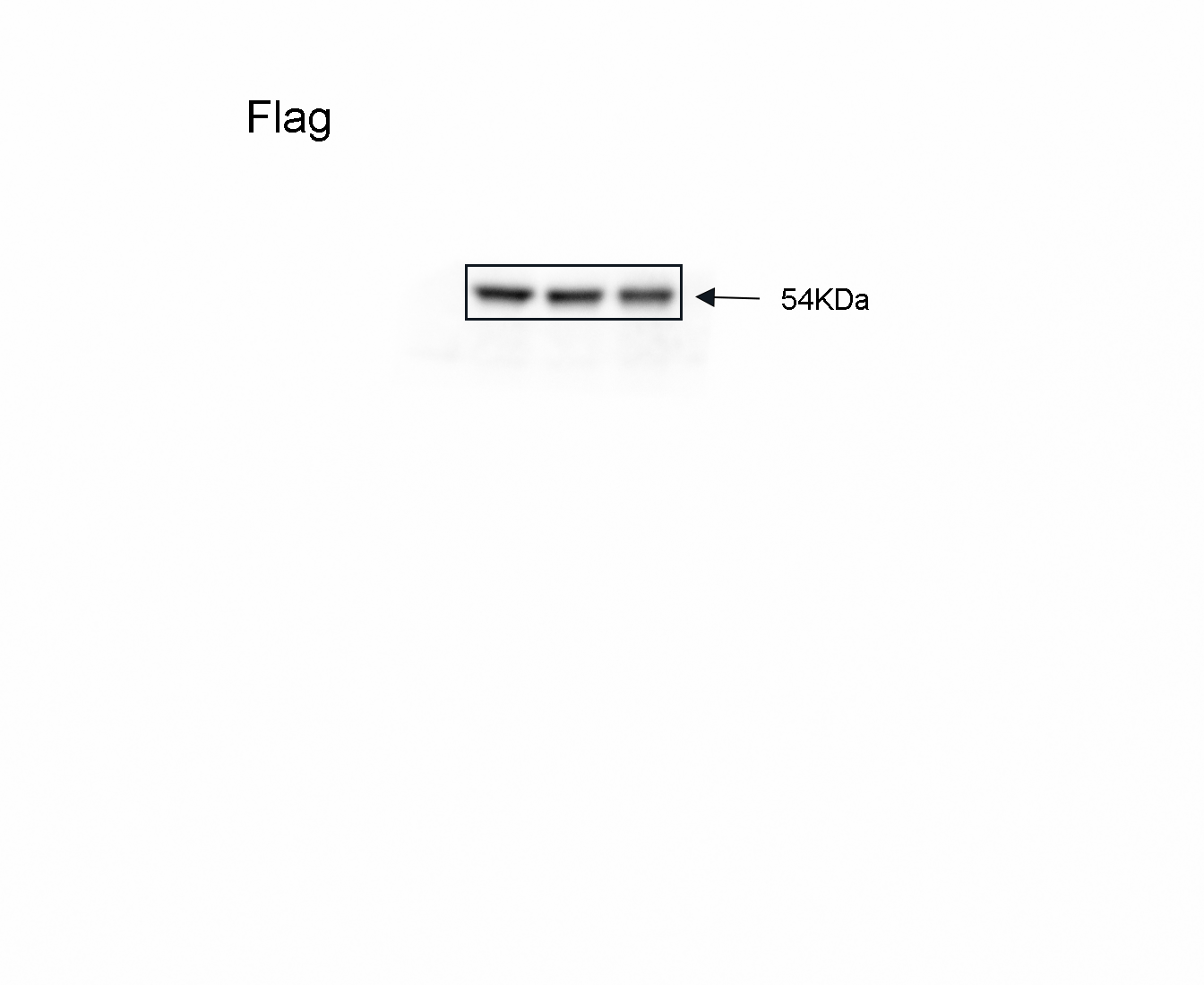

Supplement: Figure 4—source data 2. [file elife-98524-fig4-data2.zip › Fig 4-data2-v1/4J/4-Cisplatin/upper/Flag .tif]

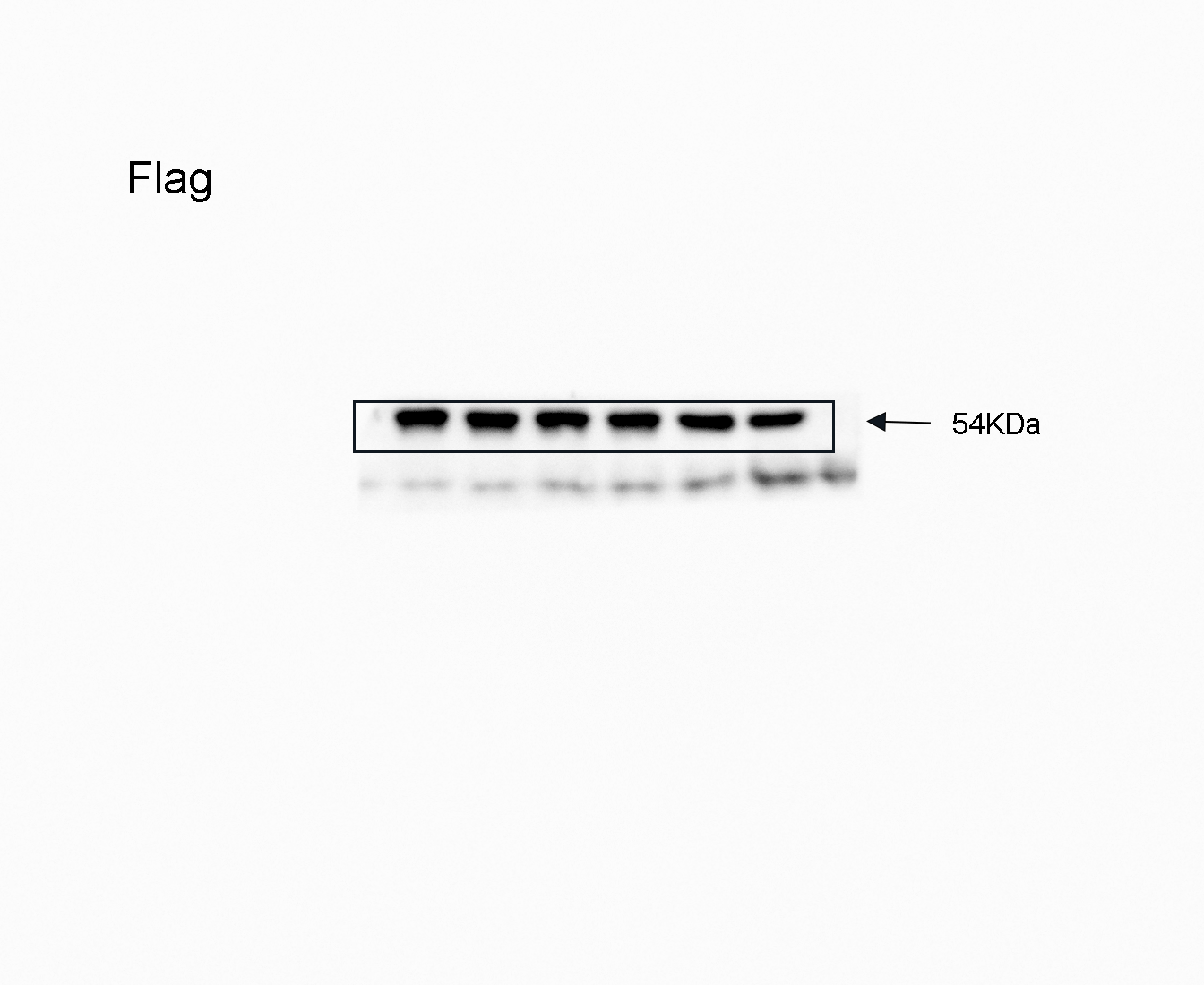

Supplement: Figure 4—source data 2. [file elife-98524-fig4-data2.zip › Fig 4-data2-v1/4K/left/Flag .tif]

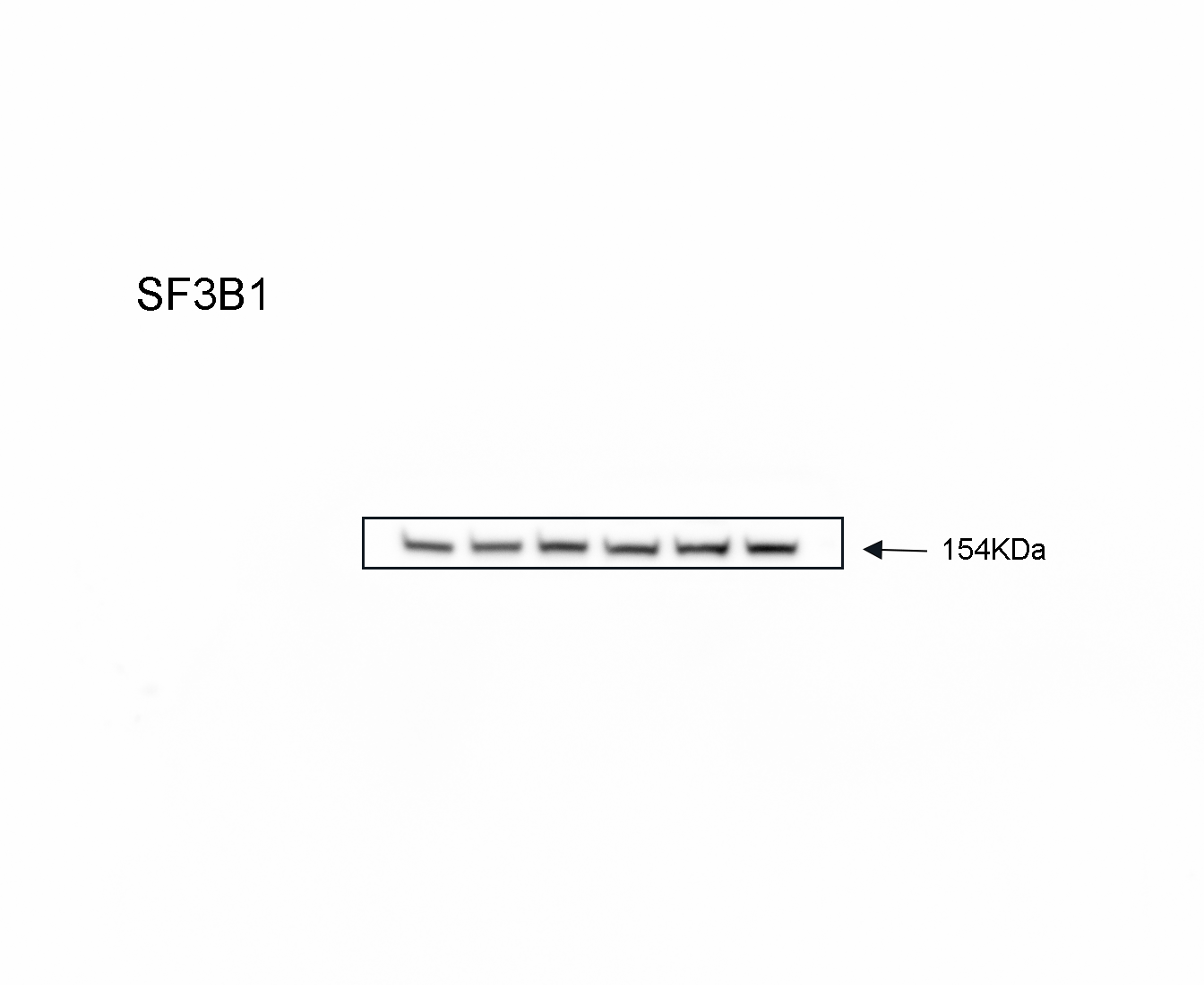

Supplement: Figure 4—source data 2. [file elife-98524-fig4-data2.zip › Fig 4-data2-v1/4K/left/SF3B1 .tif]

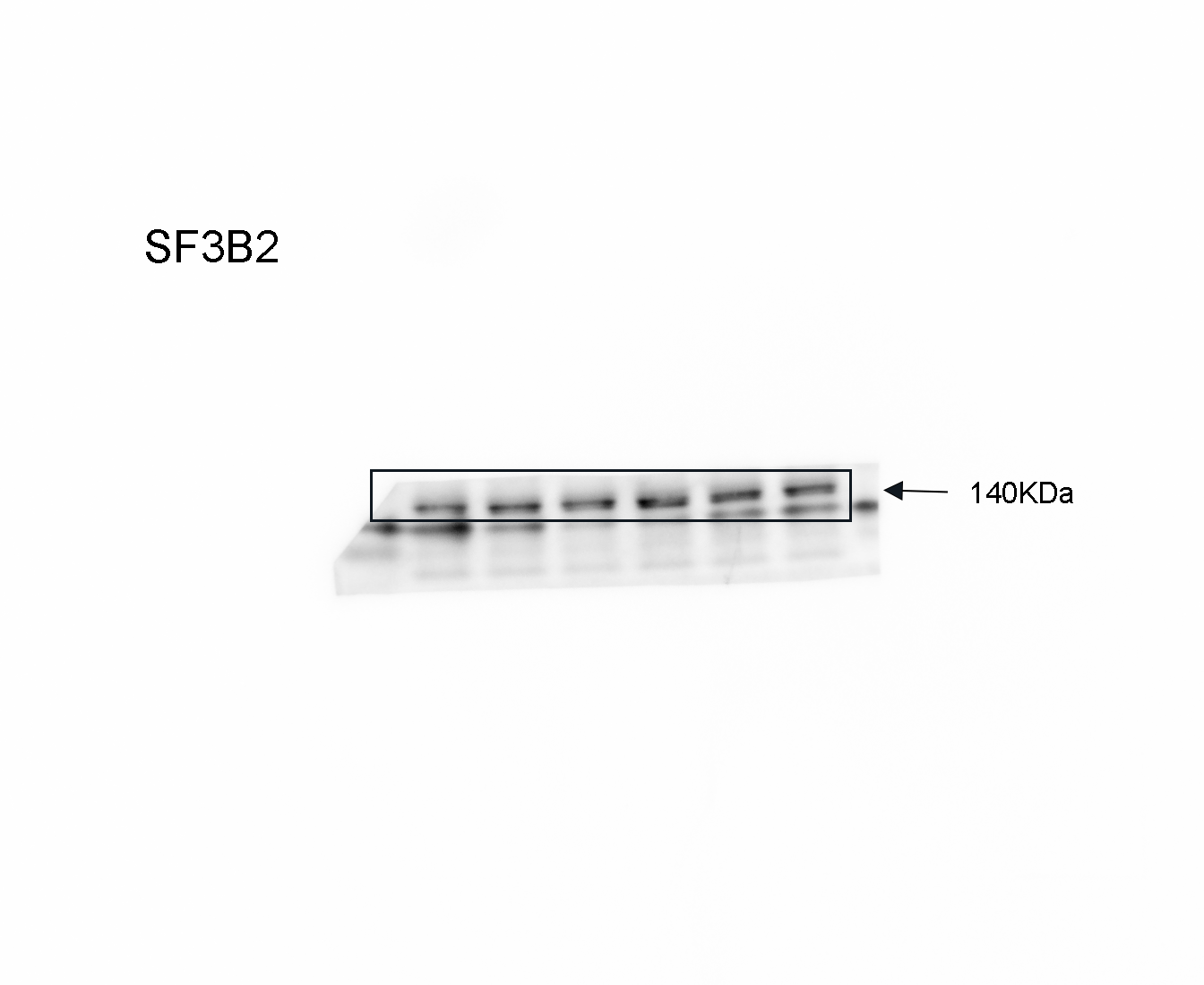

Supplement: Figure 4—source data 2. [file elife-98524-fig4-data2.zip › Fig 4-data2-v1/4K/left/SF3B2 .tif]

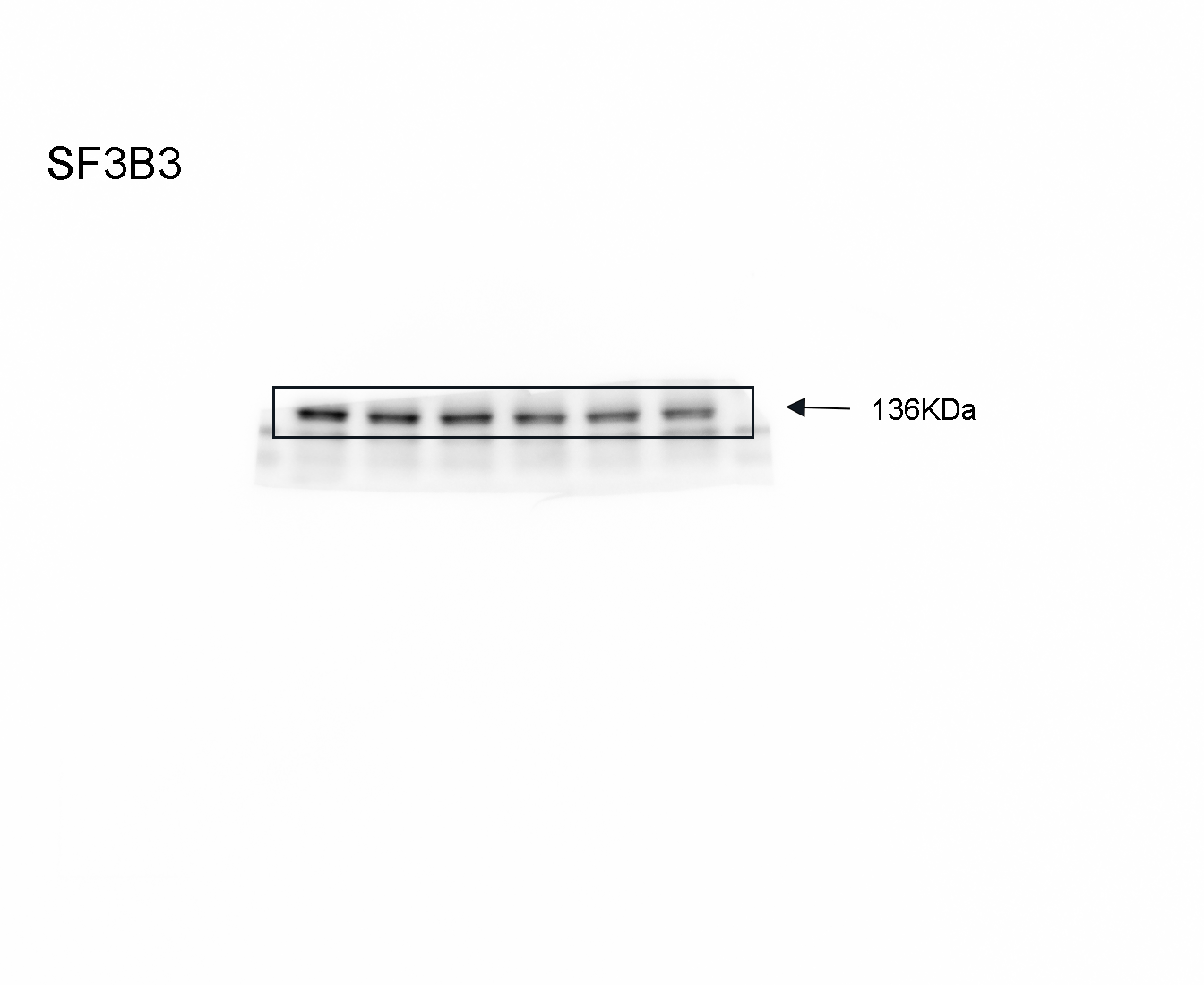

Supplement: Figure 4—source data 2. [file elife-98524-fig4-data2.zip › Fig 4-data2-v1/4K/left/SF3B3 .tif]

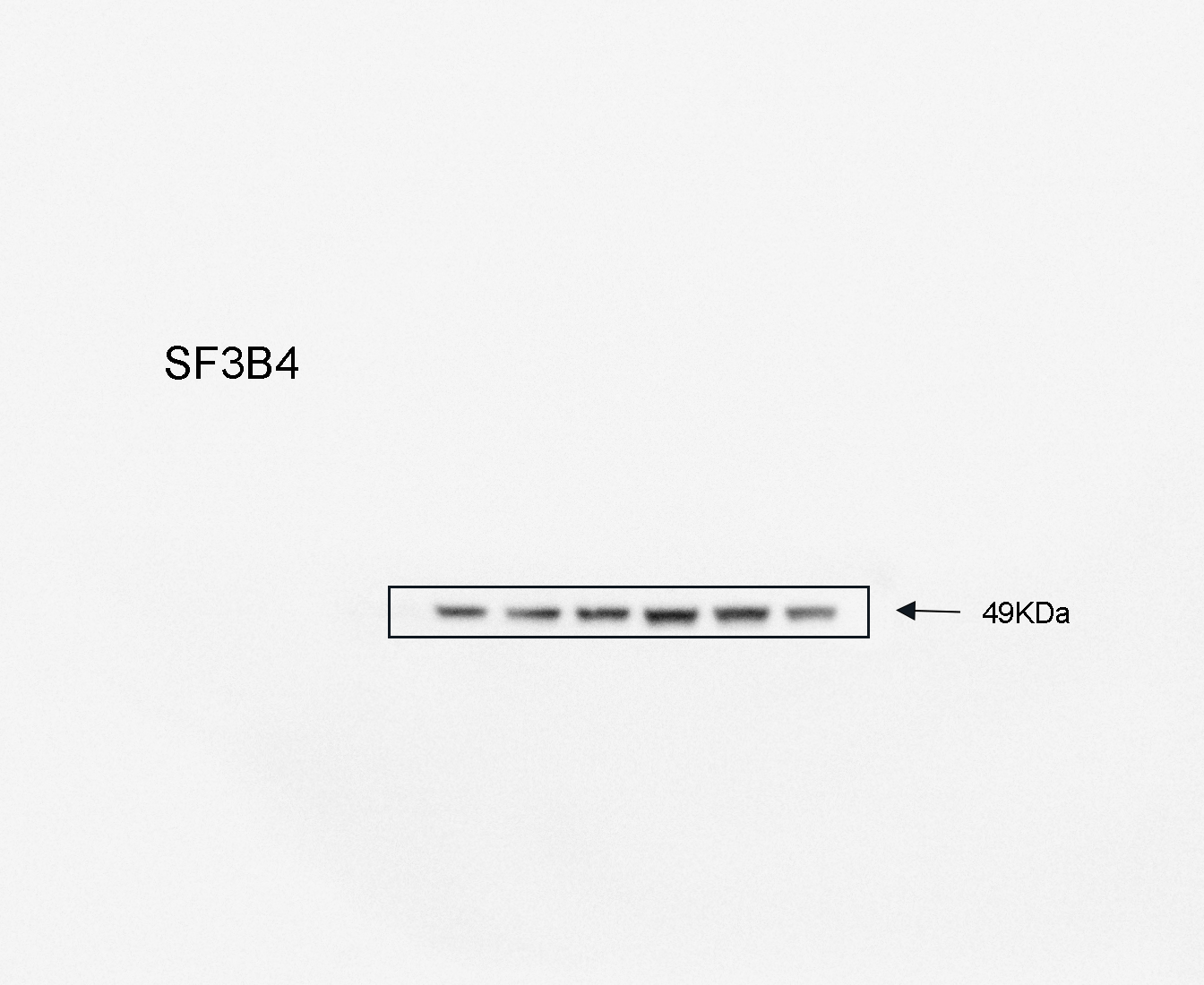

Supplement: Figure 4—source data 2. [file elife-98524-fig4-data2.zip › Fig 4-data2-v1/4K/left/SF3B4 .tif]

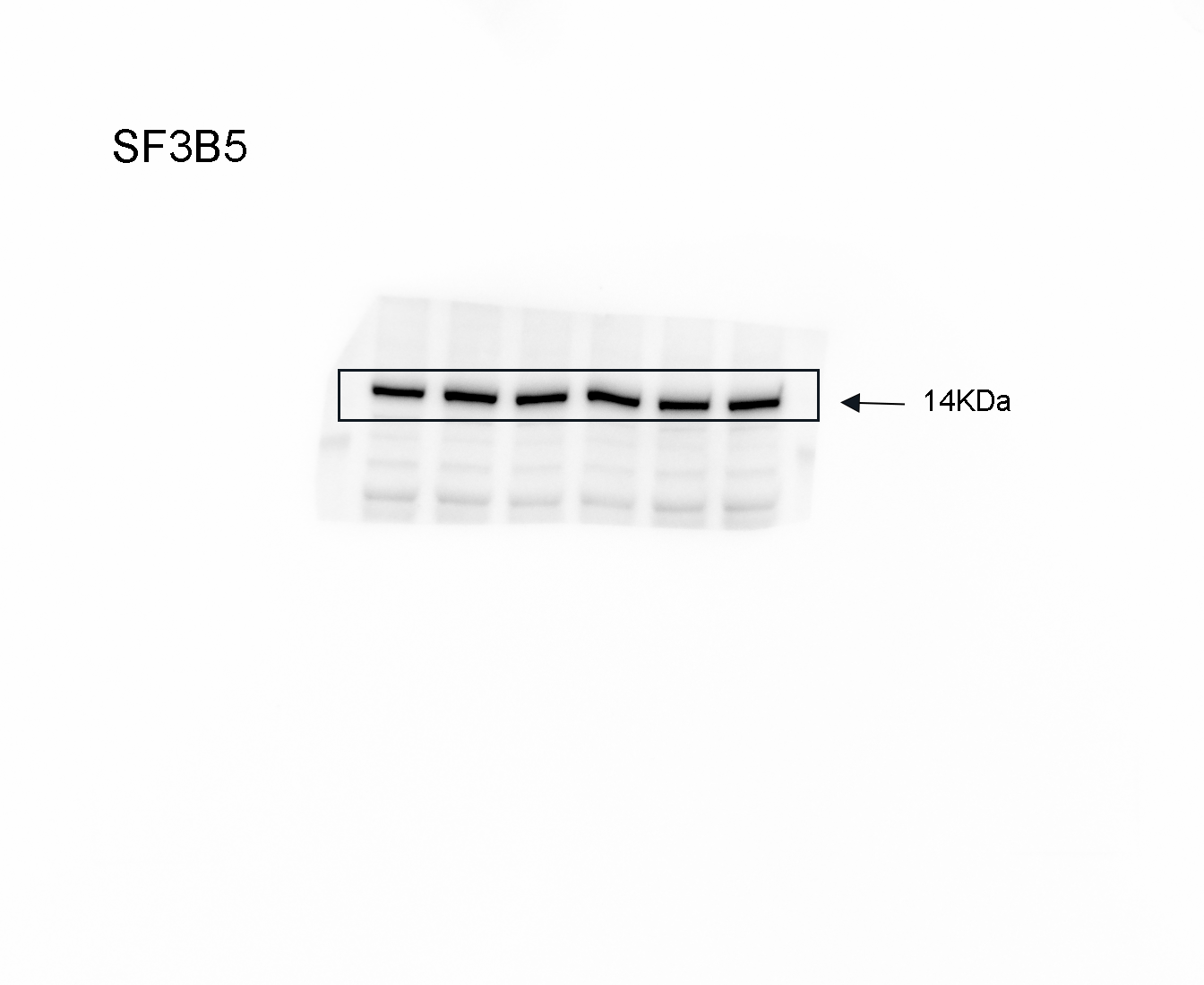

Supplement: Figure 4—source data 2. [file elife-98524-fig4-data2.zip › Fig 4-data2-v1/4K/left/SF3B5 .tif]

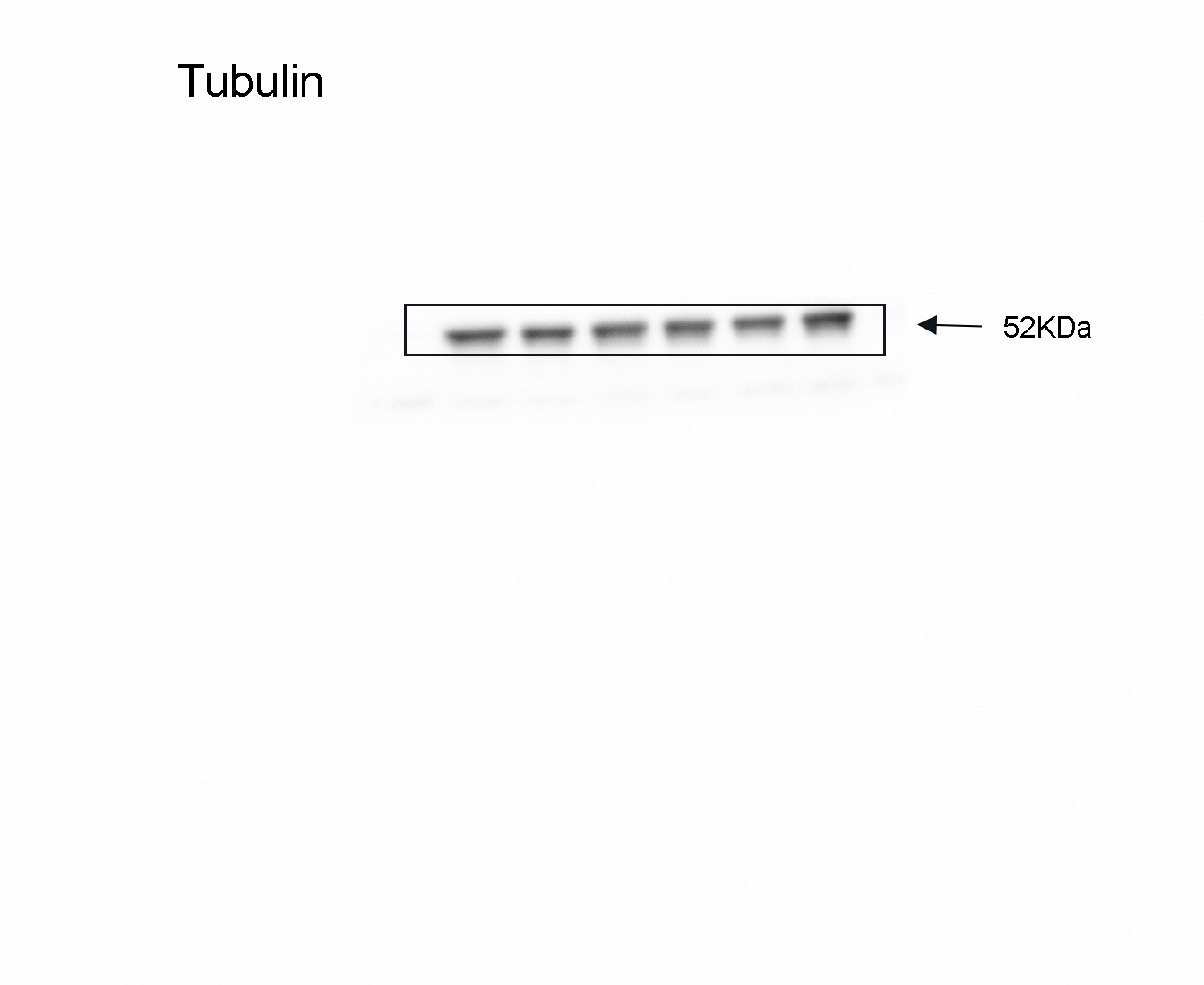

Supplement: Figure 4—source data 2. [file elife-98524-fig4-data2.zip › Fig 4-data2-v1/4K/left/Tubulin .tif]

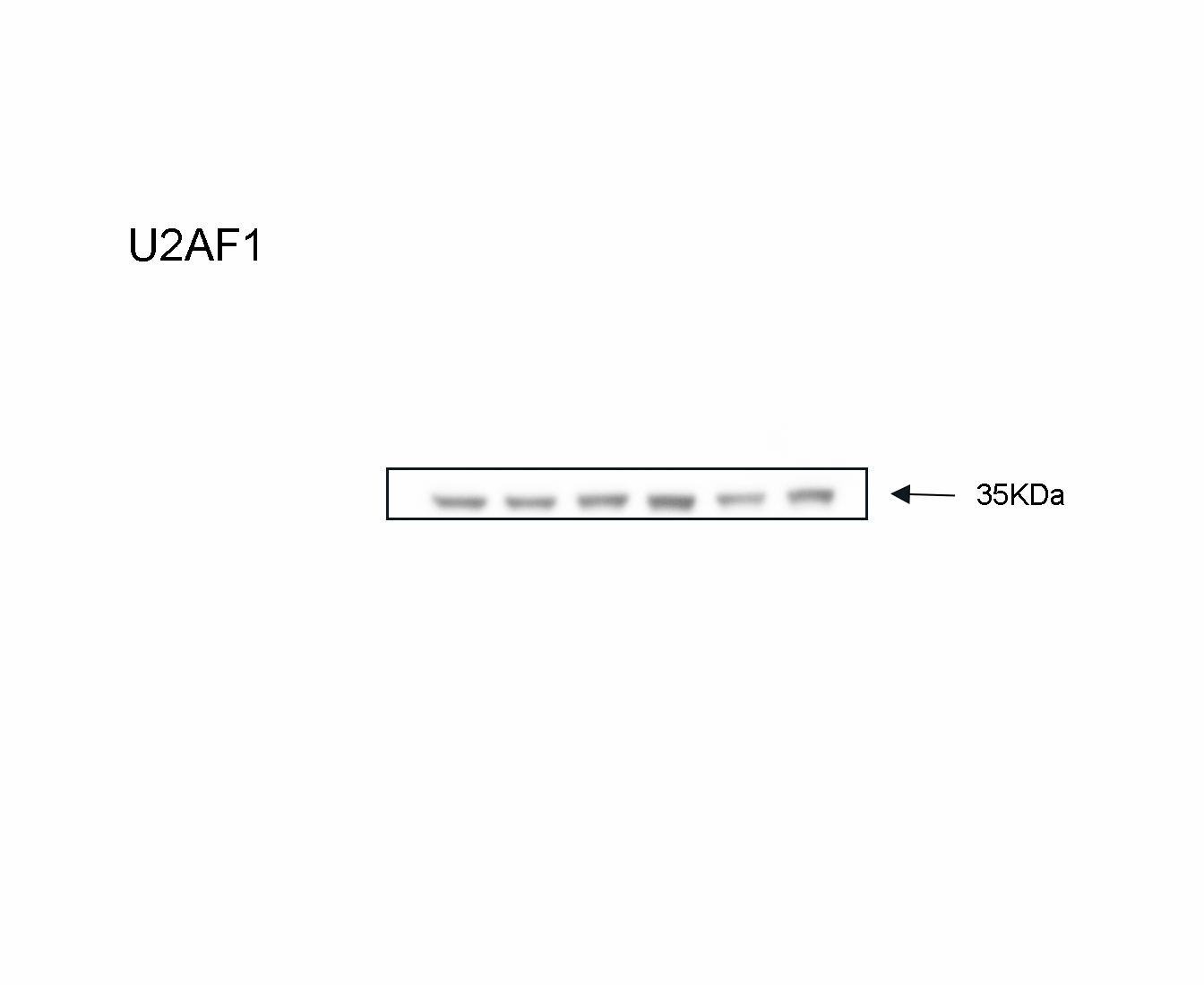

Supplement: Figure 4—source data 2. [file elife-98524-fig4-data2.zip › Fig 4-data2-v1/4K/left/U2AF1 .tif]

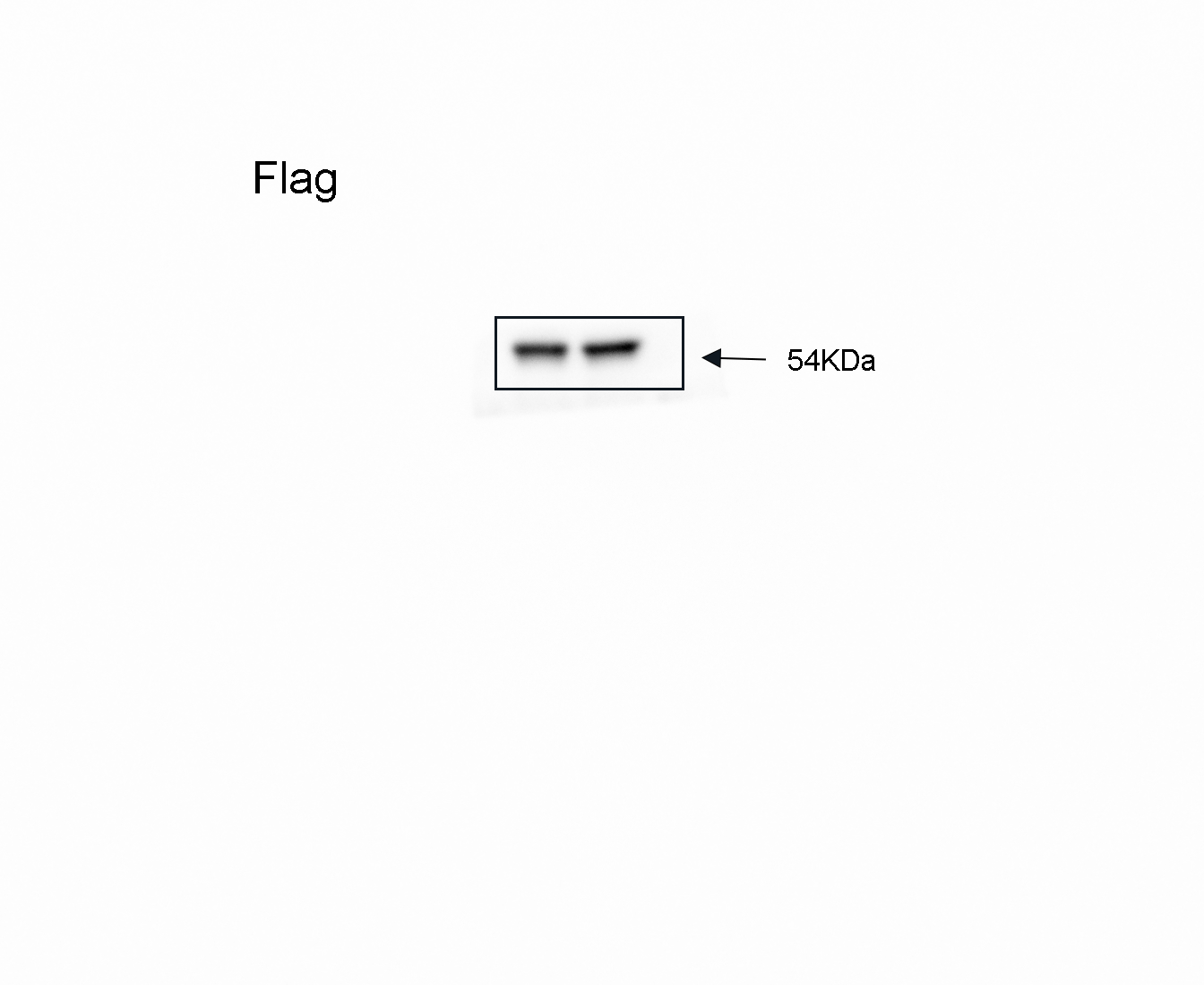

Supplement: Figure 4—source data 2. [file elife-98524-fig4-data2.zip › Fig 4-data2-v1/4K/middle/Flag .tif]

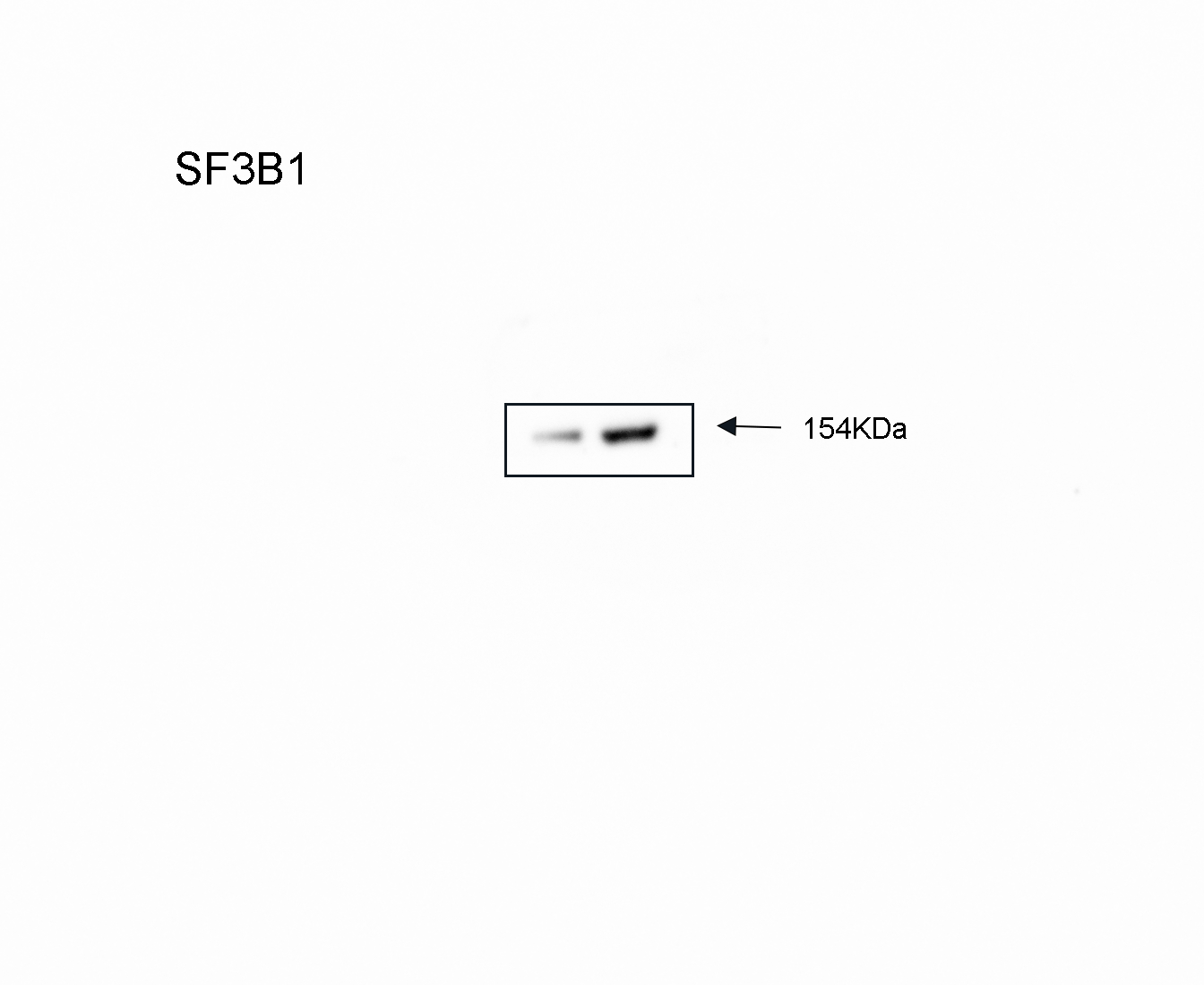

Supplement: Figure 4—source data 2. [file elife-98524-fig4-data2.zip › Fig 4-data2-v1/4K/middle/SF3B1 .tif]

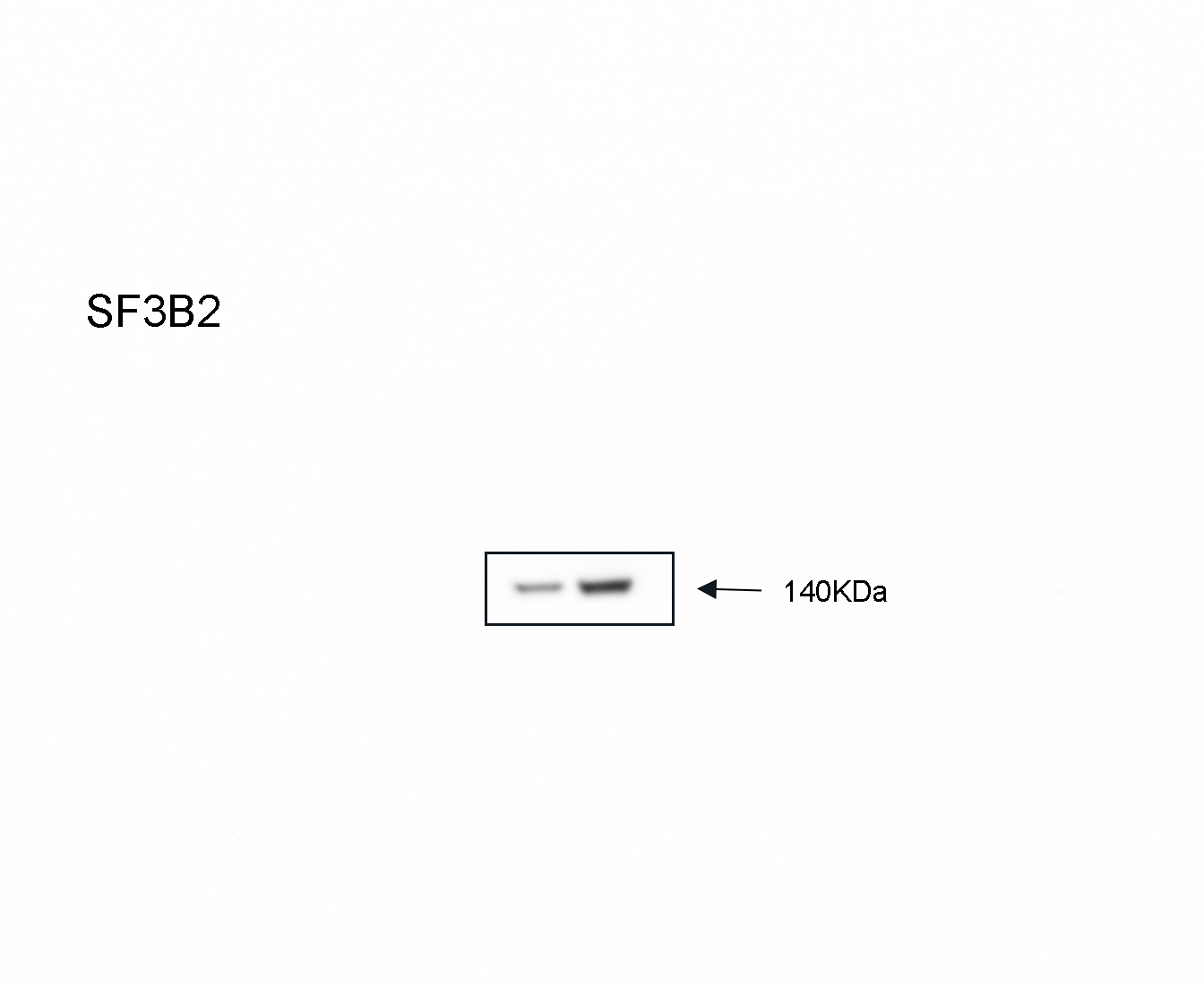

Supplement: Figure 4—source data 2. [file elife-98524-fig4-data2.zip › Fig 4-data2-v1/4K/middle/SF3B2 .tif]

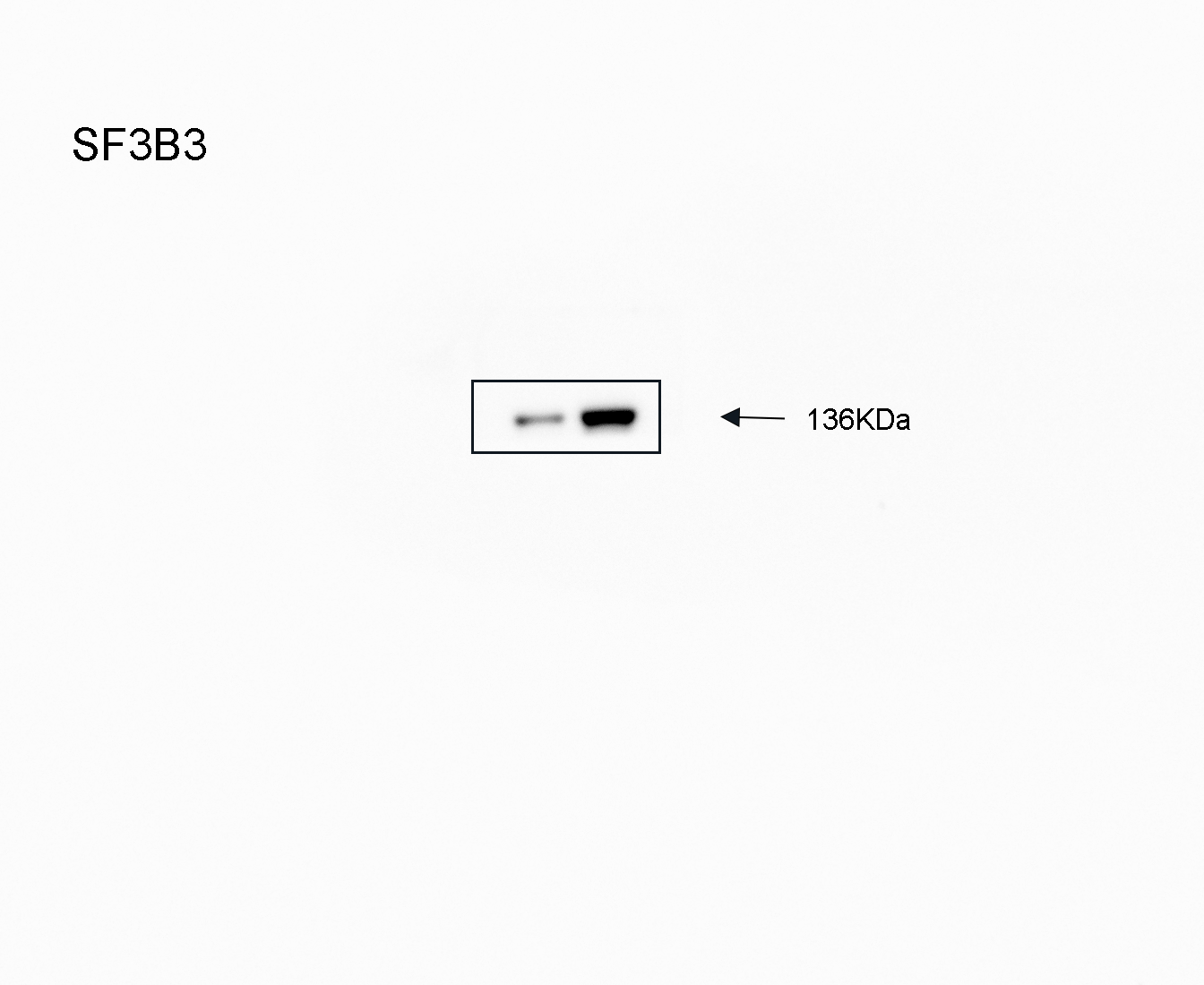

Supplement: Figure 4—source data 2. [file elife-98524-fig4-data2.zip › Fig 4-data2-v1/4K/middle/SF3B3 .tif]

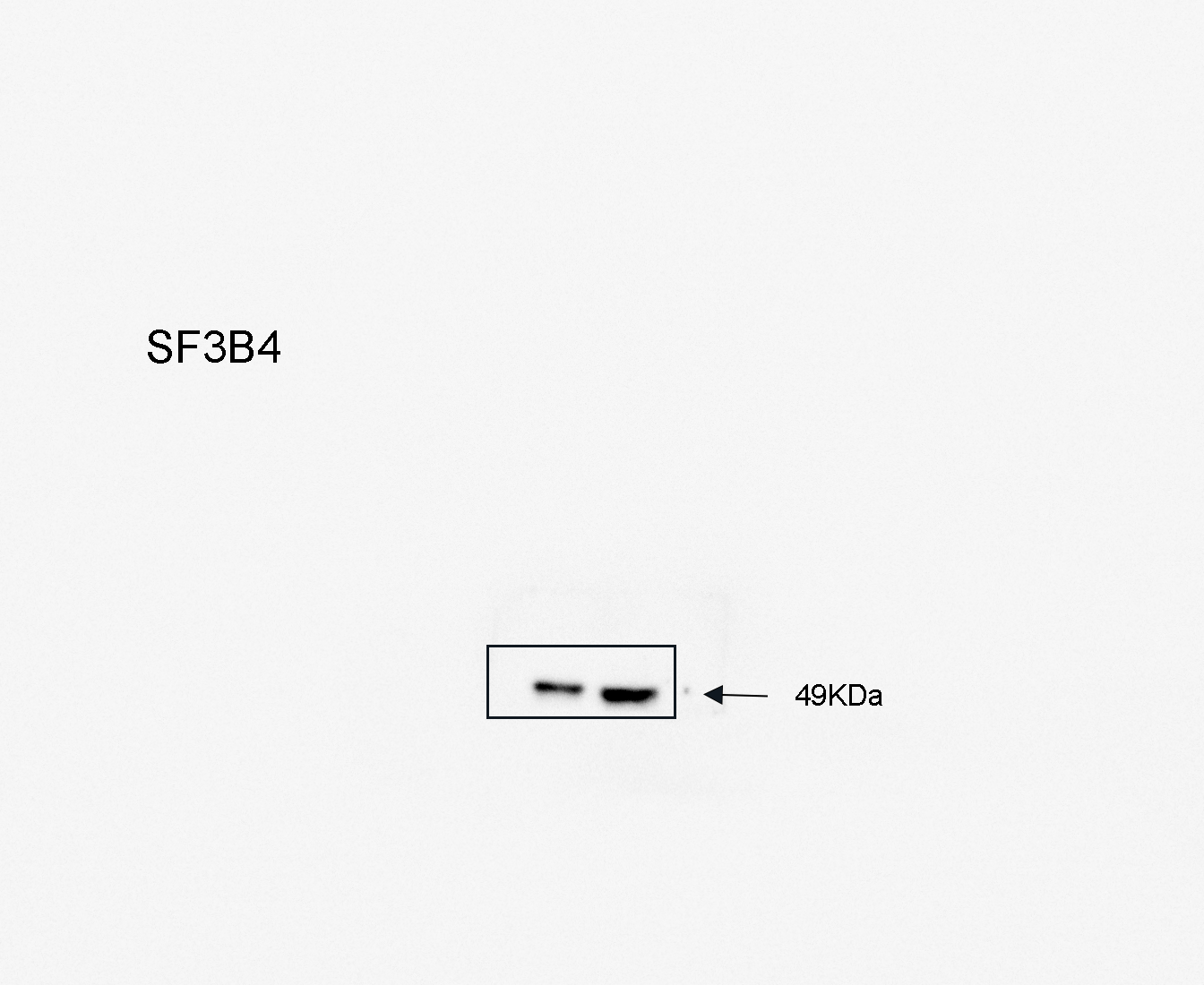

Supplement: Figure 4—source data 2. [file elife-98524-fig4-data2.zip › Fig 4-data2-v1/4K/middle/SF3B4 .tif]

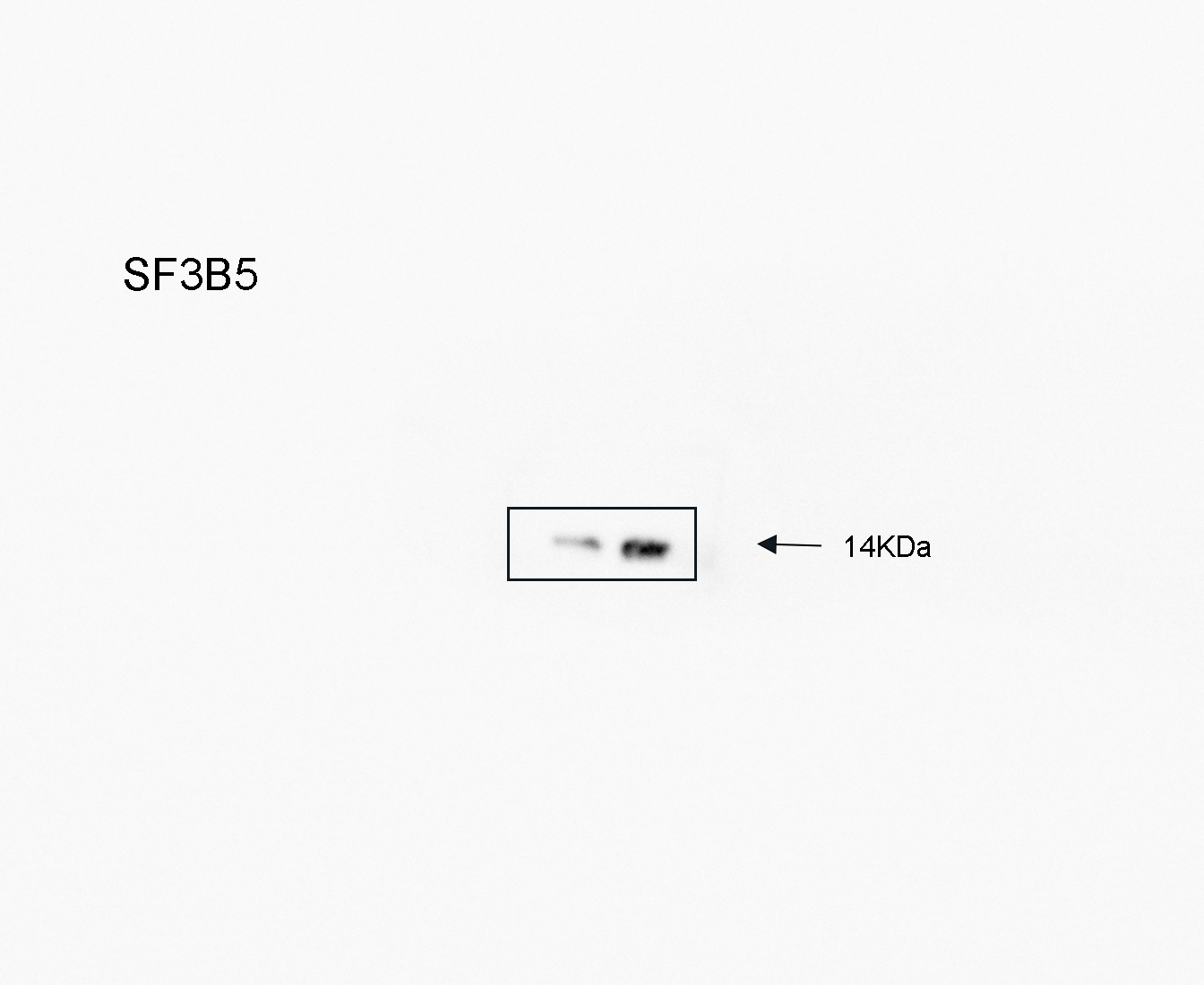

Supplement: Figure 4—source data 2. [file elife-98524-fig4-data2.zip › Fig 4-data2-v1/4K/middle/SF3B5 .tif]

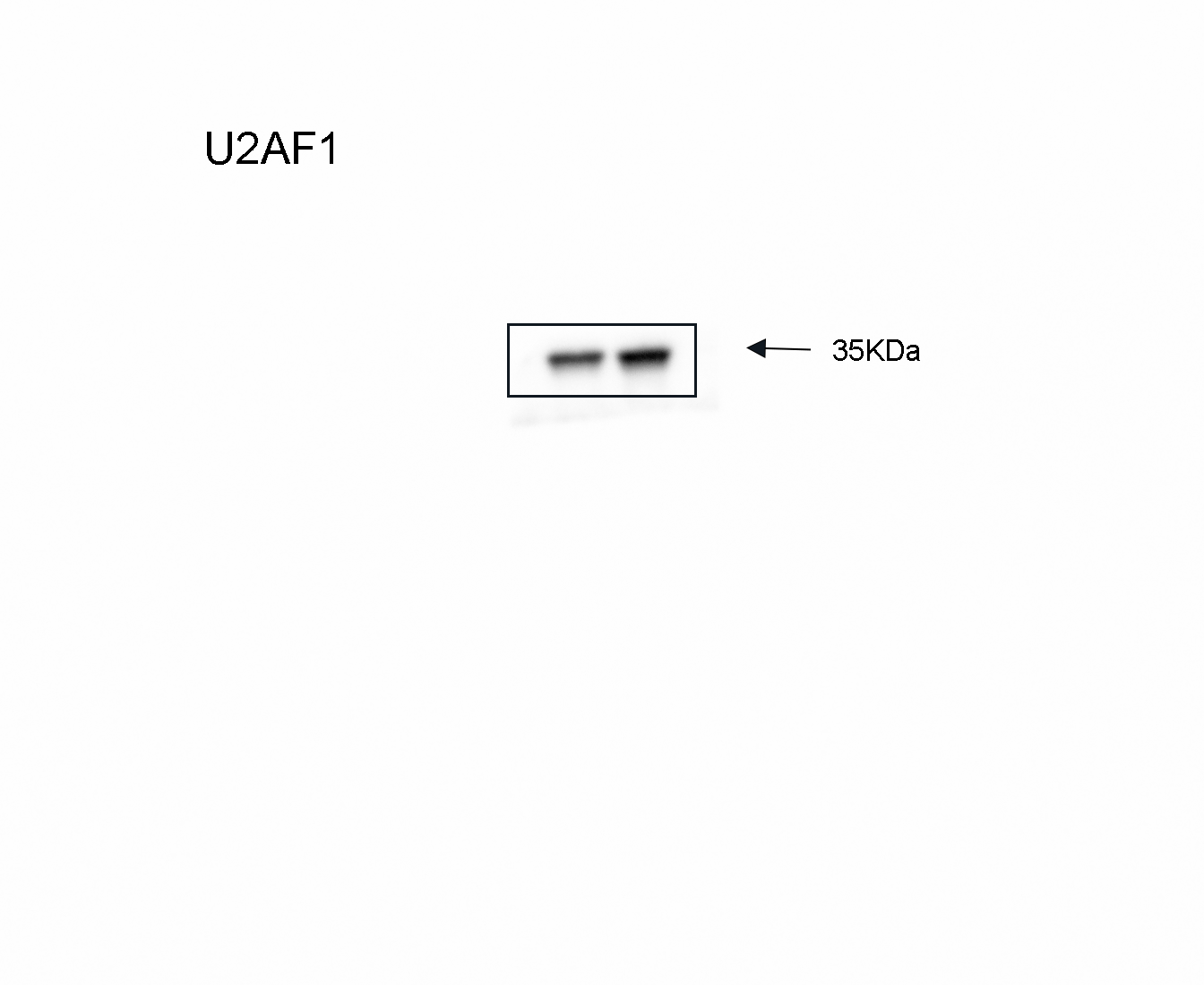

Supplement: Figure 4—source data 2. [file elife-98524-fig4-data2.zip › Fig 4-data2-v1/4K/middle/U2AF1 .tif]

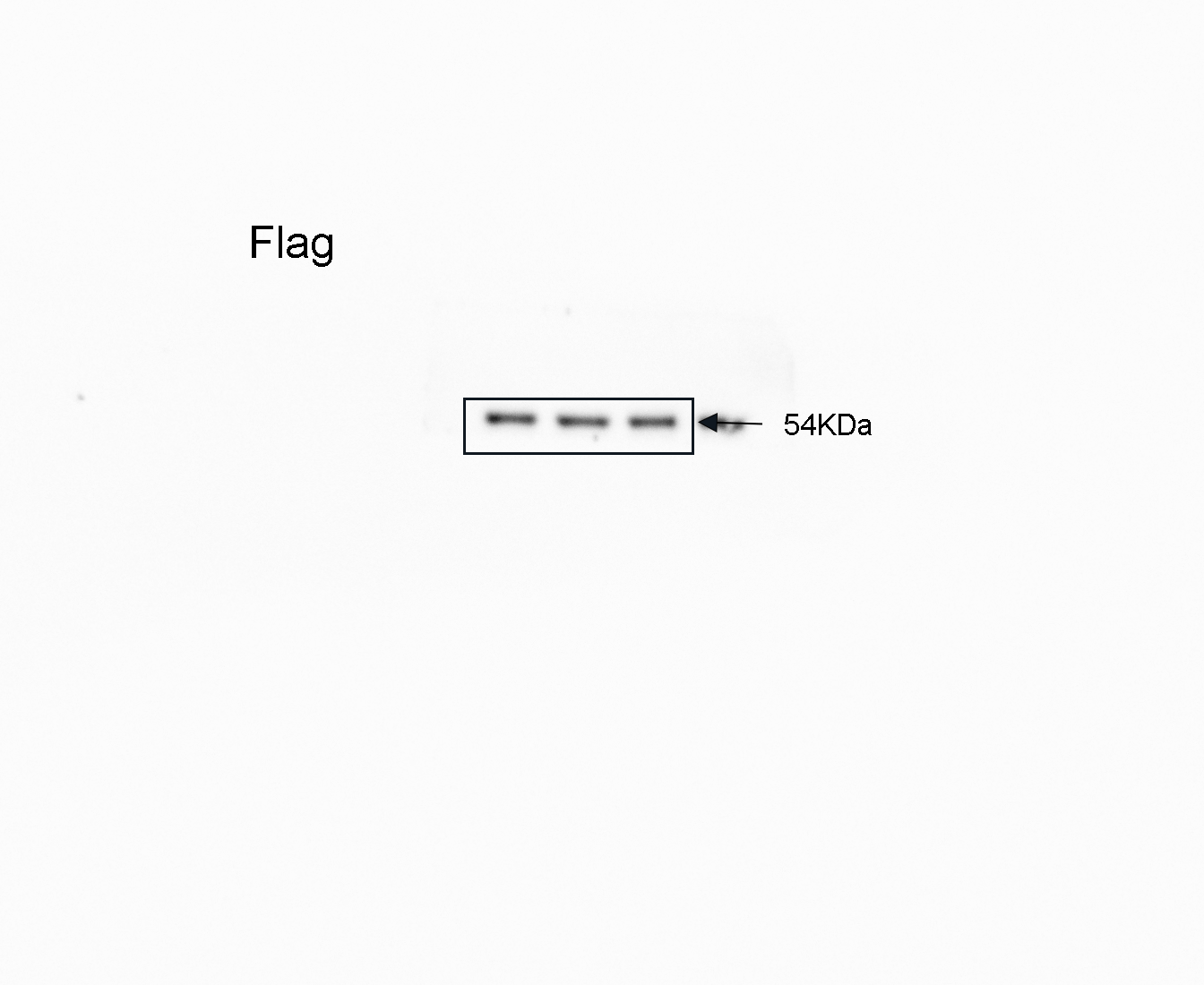

Supplement: Figure 4—source data 2. [file elife-98524-fig4-data2.zip › Fig 4-data2-v1/4K/right/Flag .tif]

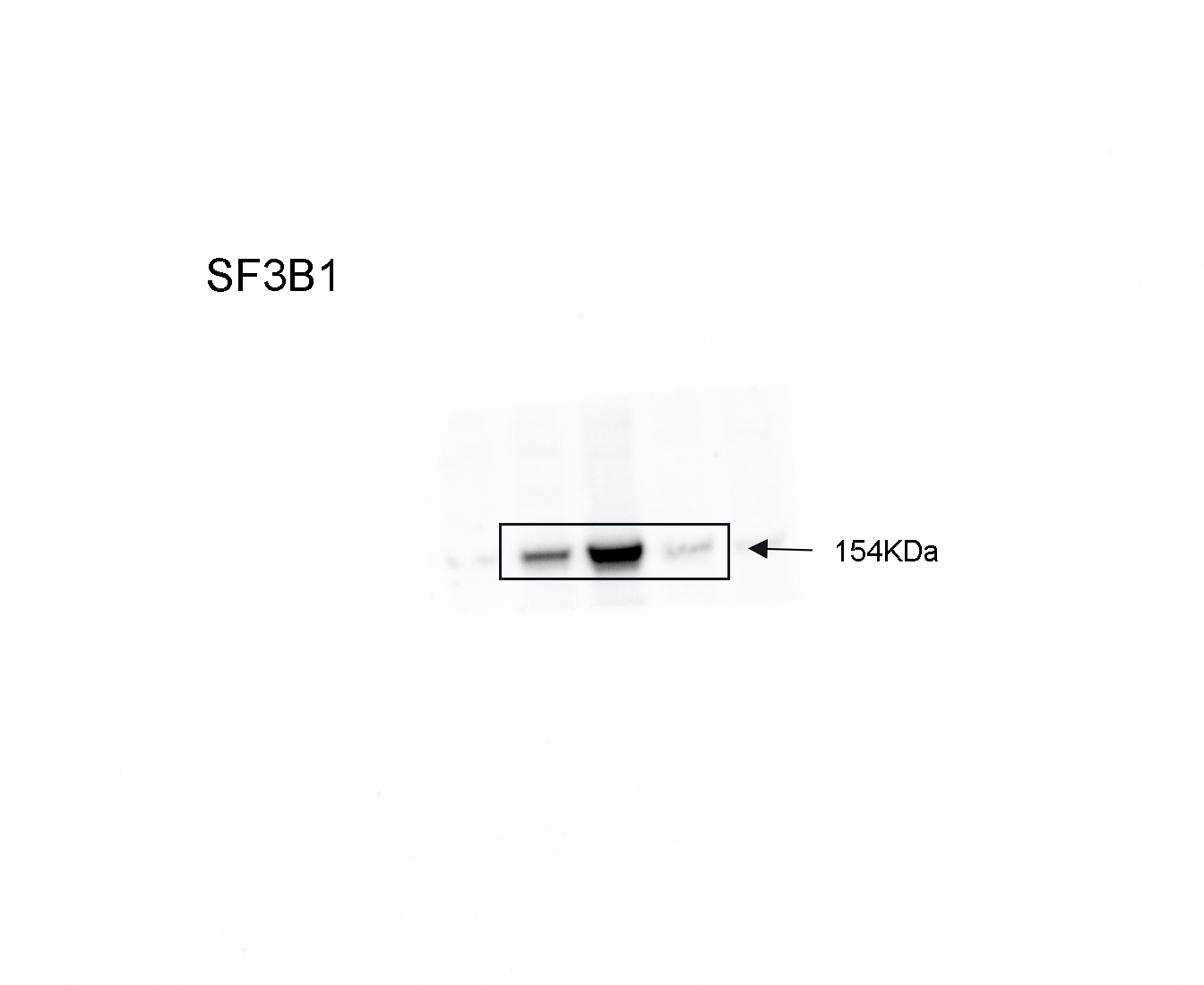

Supplement: Figure 4—source data 2. [file elife-98524-fig4-data2.zip › Fig 4-data2-v1/4K/right/SF3B1 .tif]

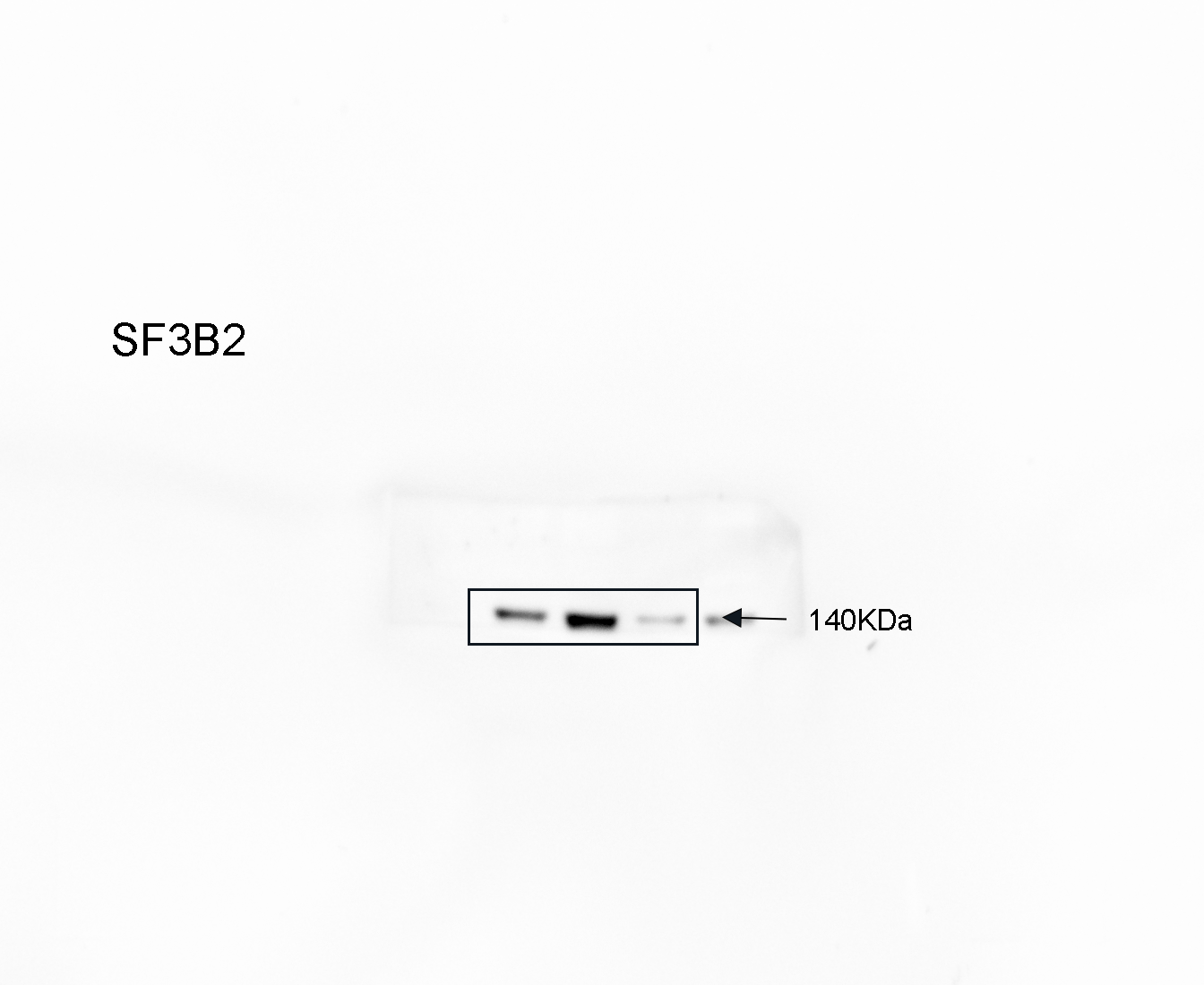

Supplement: Figure 4—source data 2. [file elife-98524-fig4-data2.zip › Fig 4-data2-v1/4K/right/SF3B2 .tif]

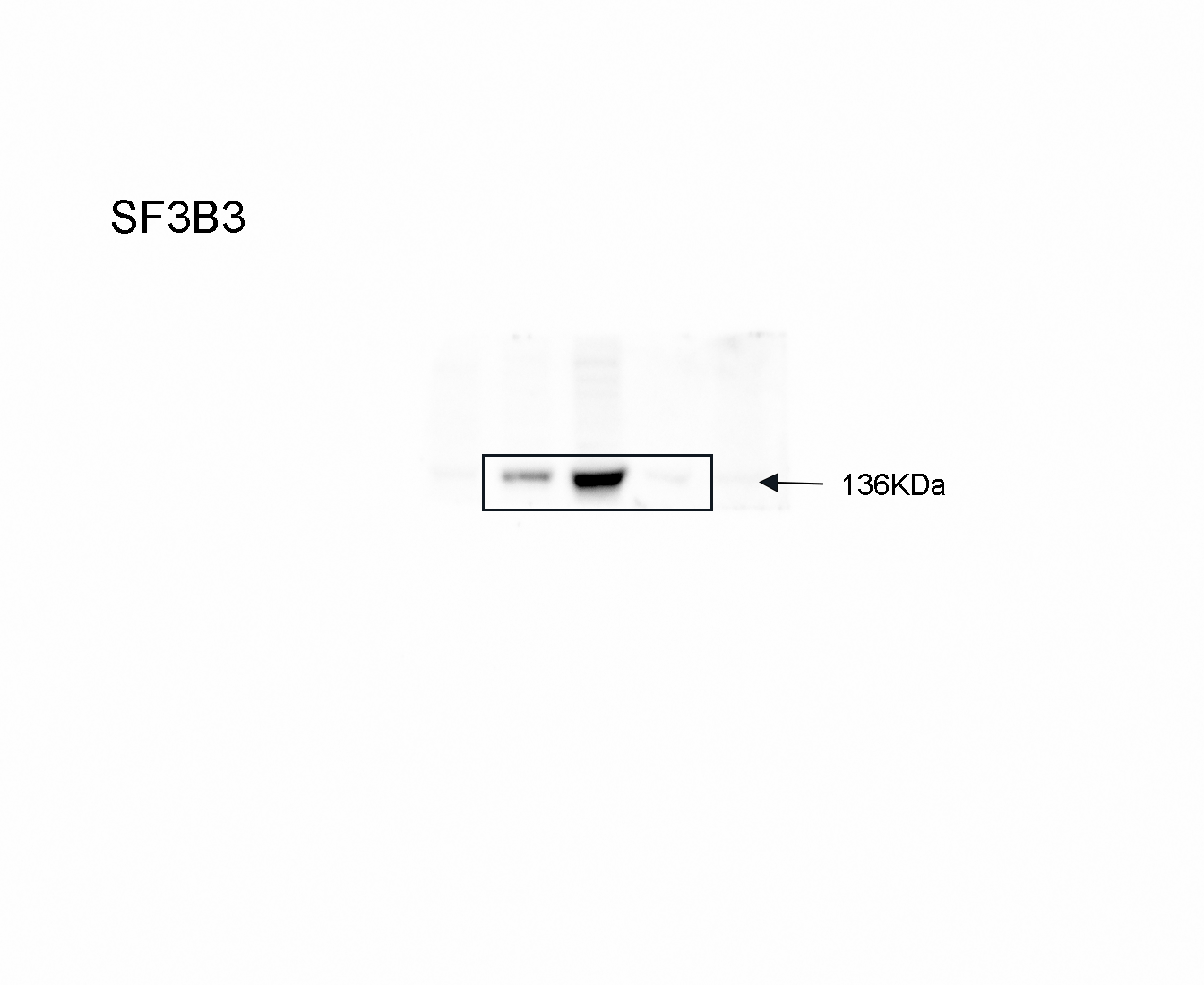

Supplement: Figure 4—source data 2. [file elife-98524-fig4-data2.zip › Fig 4-data2-v1/4K/right/SF3B3 .tif]

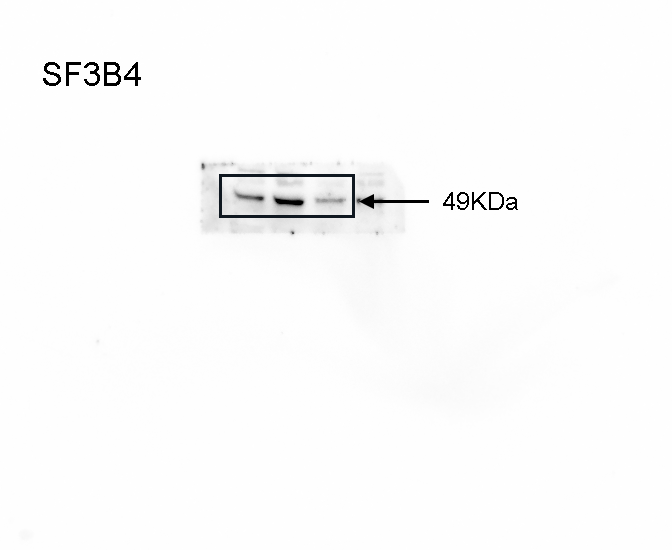

Supplement: Figure 4—source data 2. [file elife-98524-fig4-data2.zip › Fig 4-data2-v1/4K/right/SF3B4 .tif]

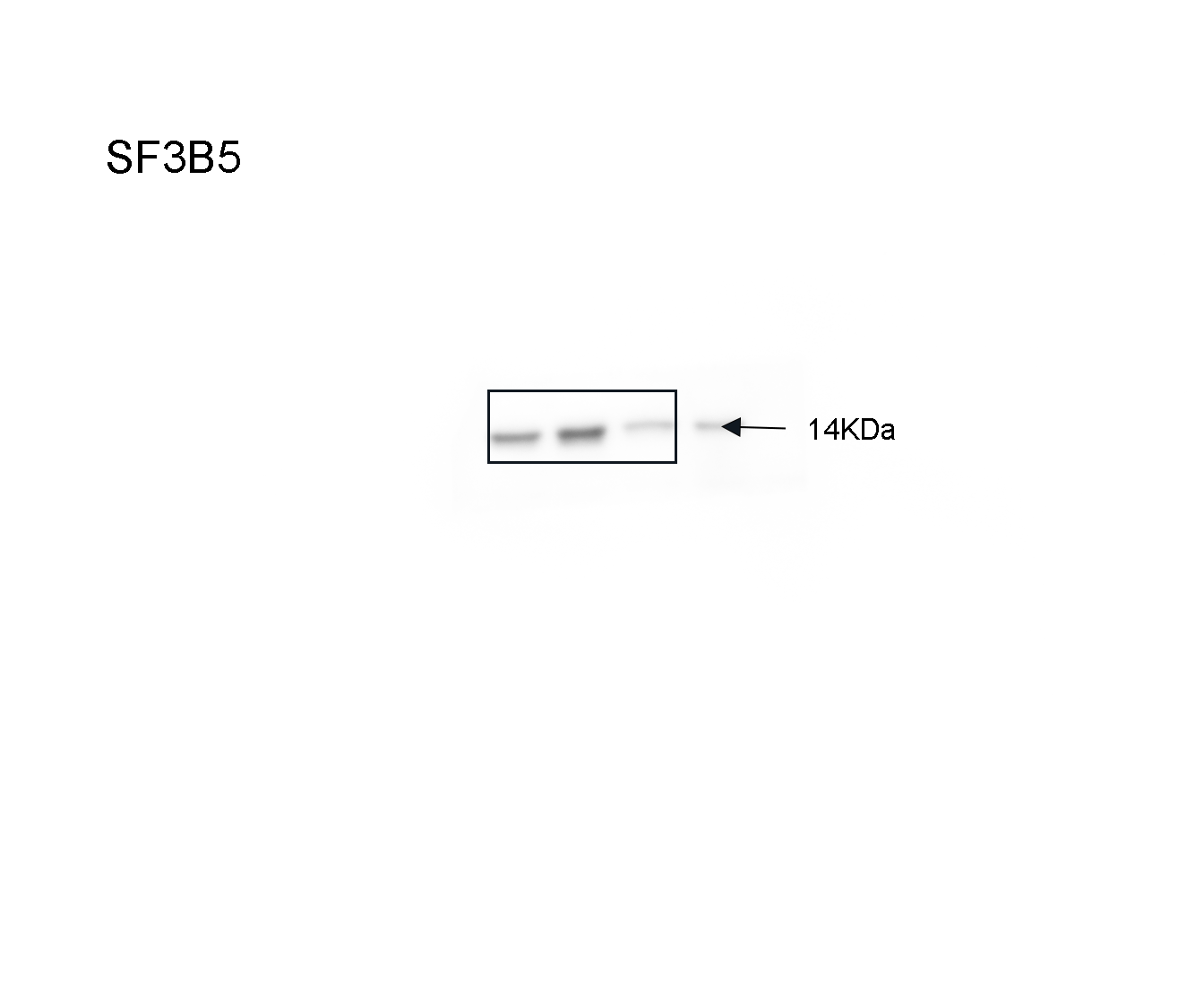

Supplement: Figure 4—source data 2. [file elife-98524-fig4-data2.zip › Fig 4-data2-v1/4K/right/SF3B5 .tif]

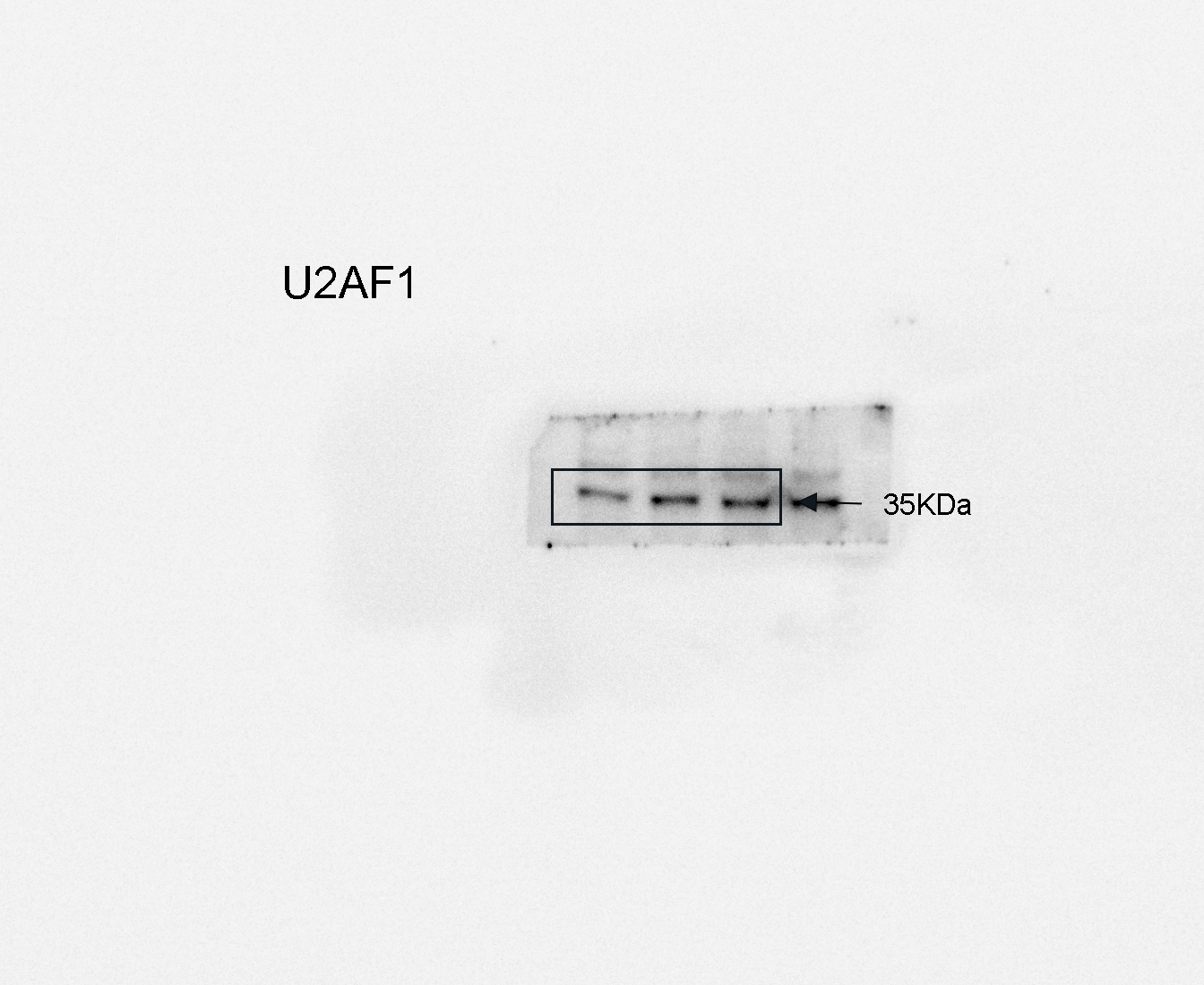

Supplement: Figure 4—source data 2. [file elife-98524-fig4-data2.zip › Fig 4-data2-v1/4K/right/U2AF1 .tif]

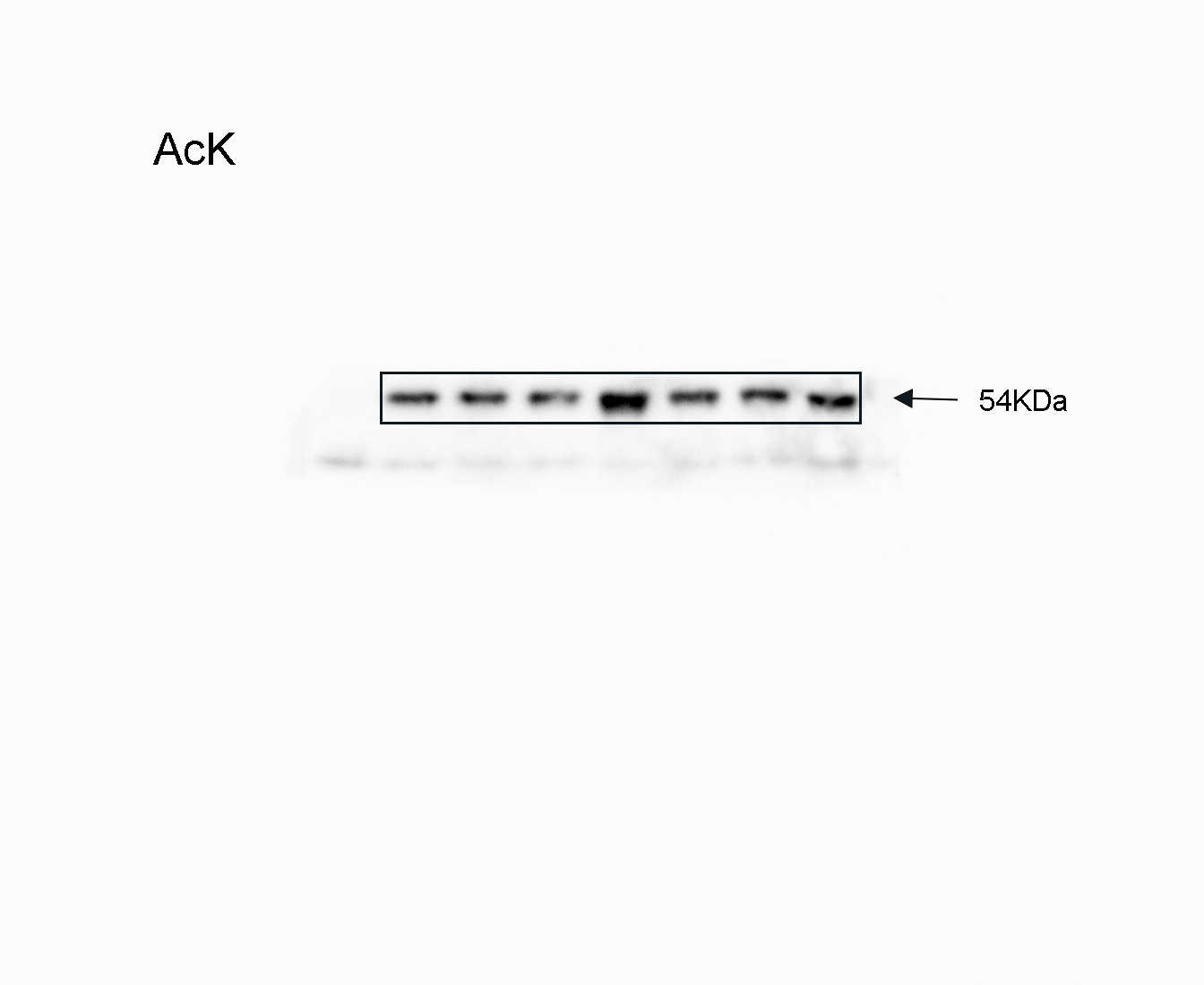

Supplement: Figure 4—source data 2. [file elife-98524-fig4-data2.zip › Fig 4-data2-v1/4M/AcK .tif]

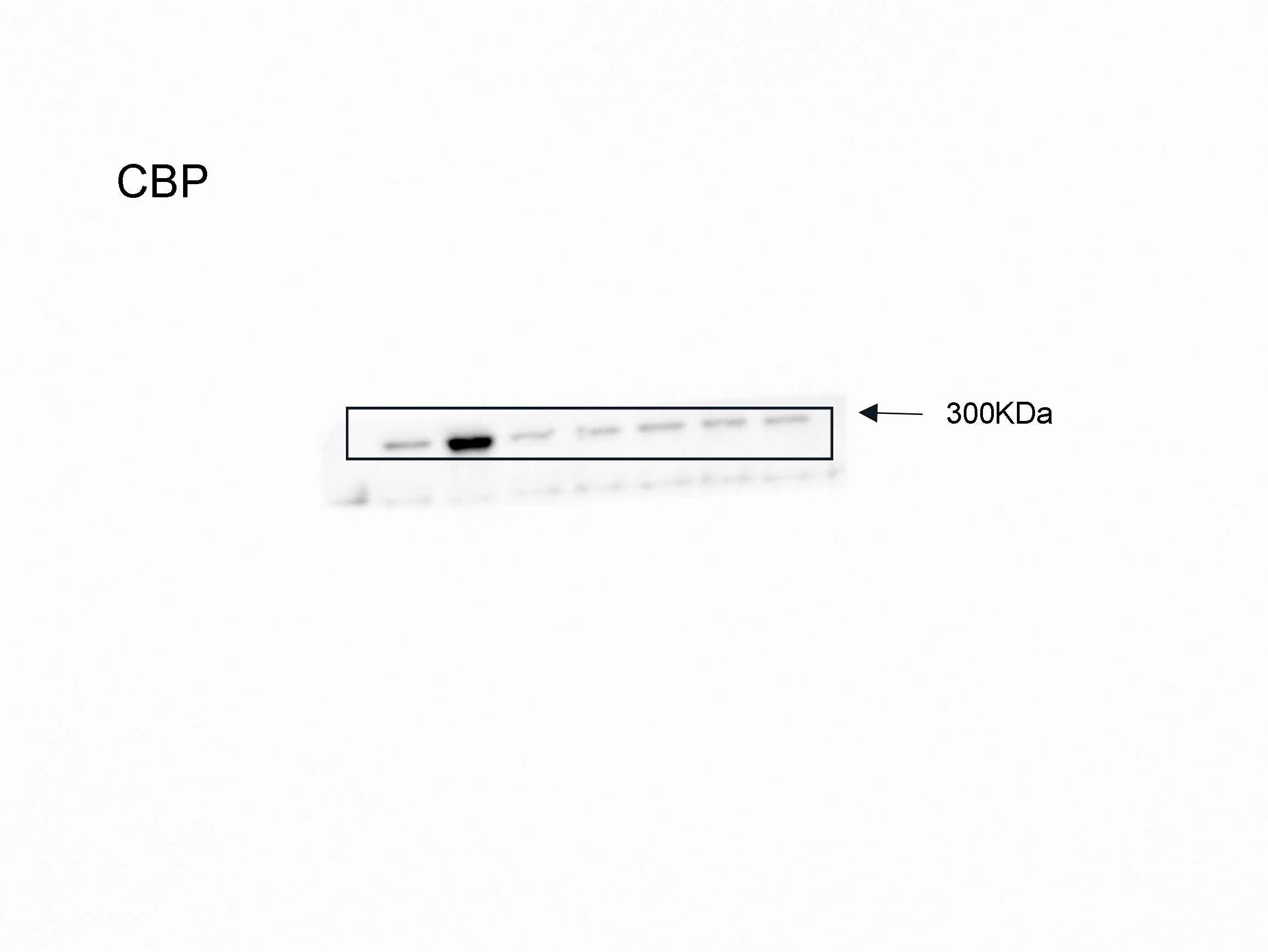

Supplement: Figure 4—source data 2. [file elife-98524-fig4-data2.zip › Fig 4-data2-v1/4M/CBP .tif]

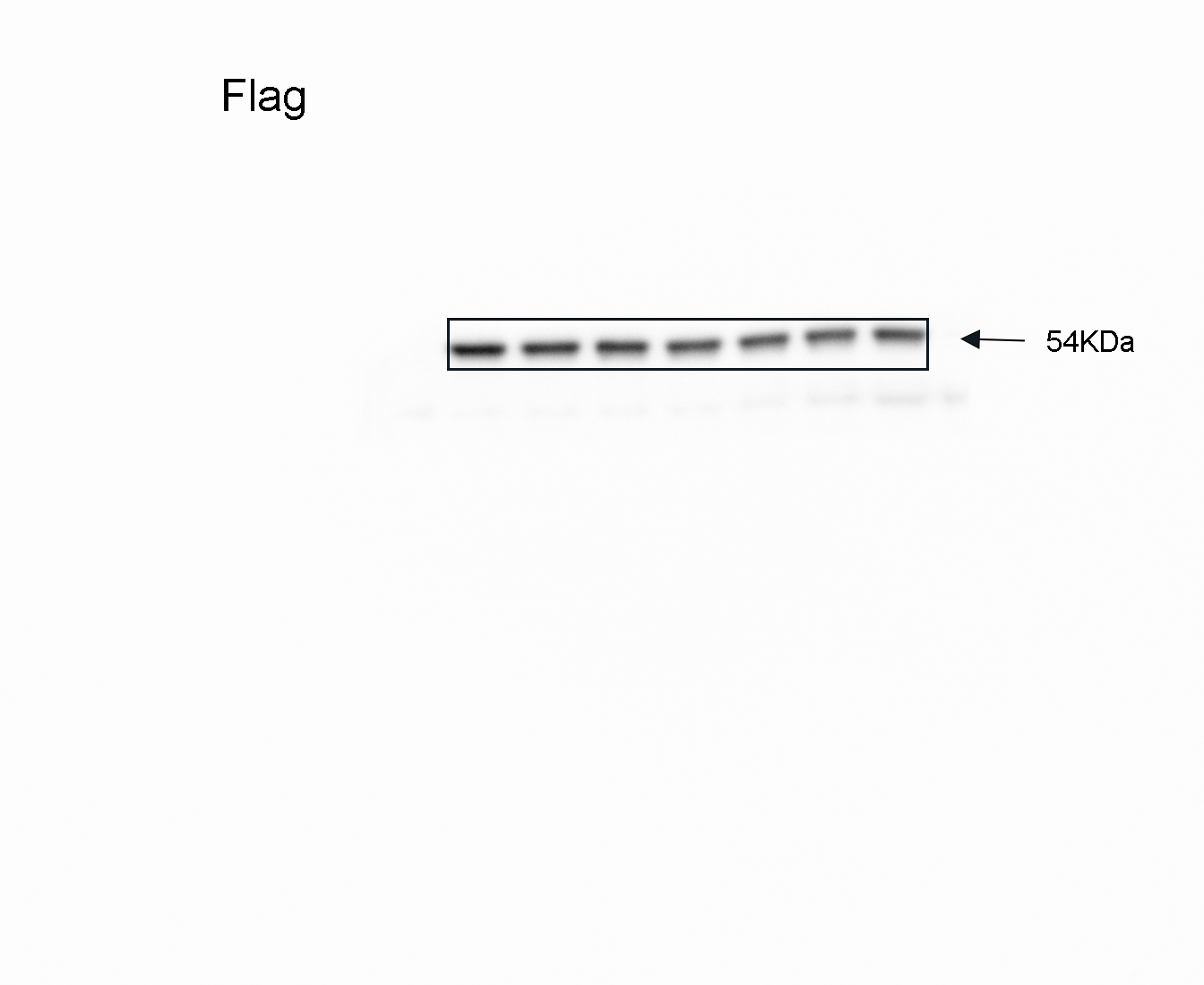

Supplement: Figure 4—source data 2. [file elife-98524-fig4-data2.zip › Fig 4-data2-v1/4M/Flag .tif]

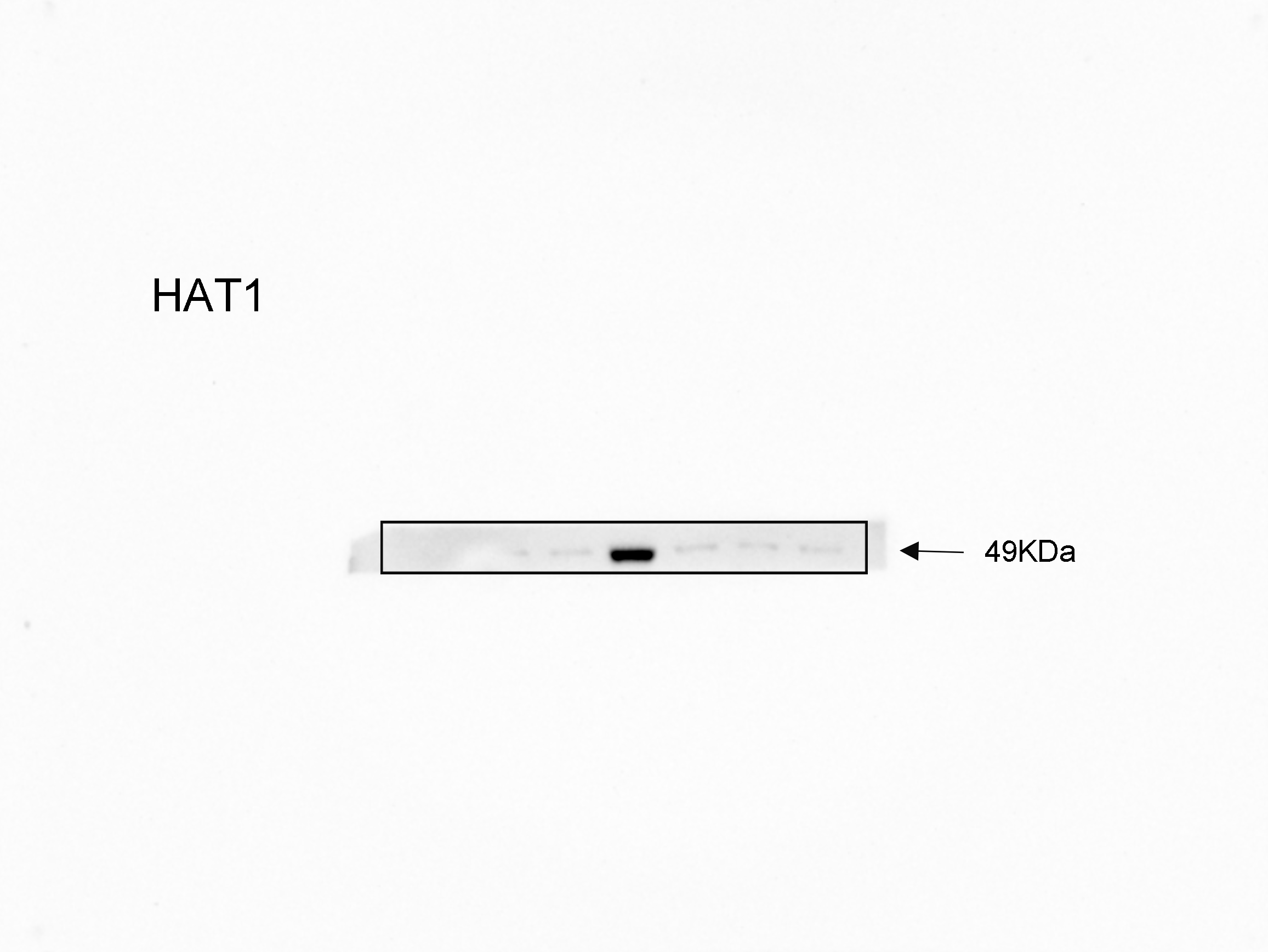

Supplement: Figure 4—source data 2. [file elife-98524-fig4-data2.zip › Fig 4-data2-v1/4M/HAT1 .tif]

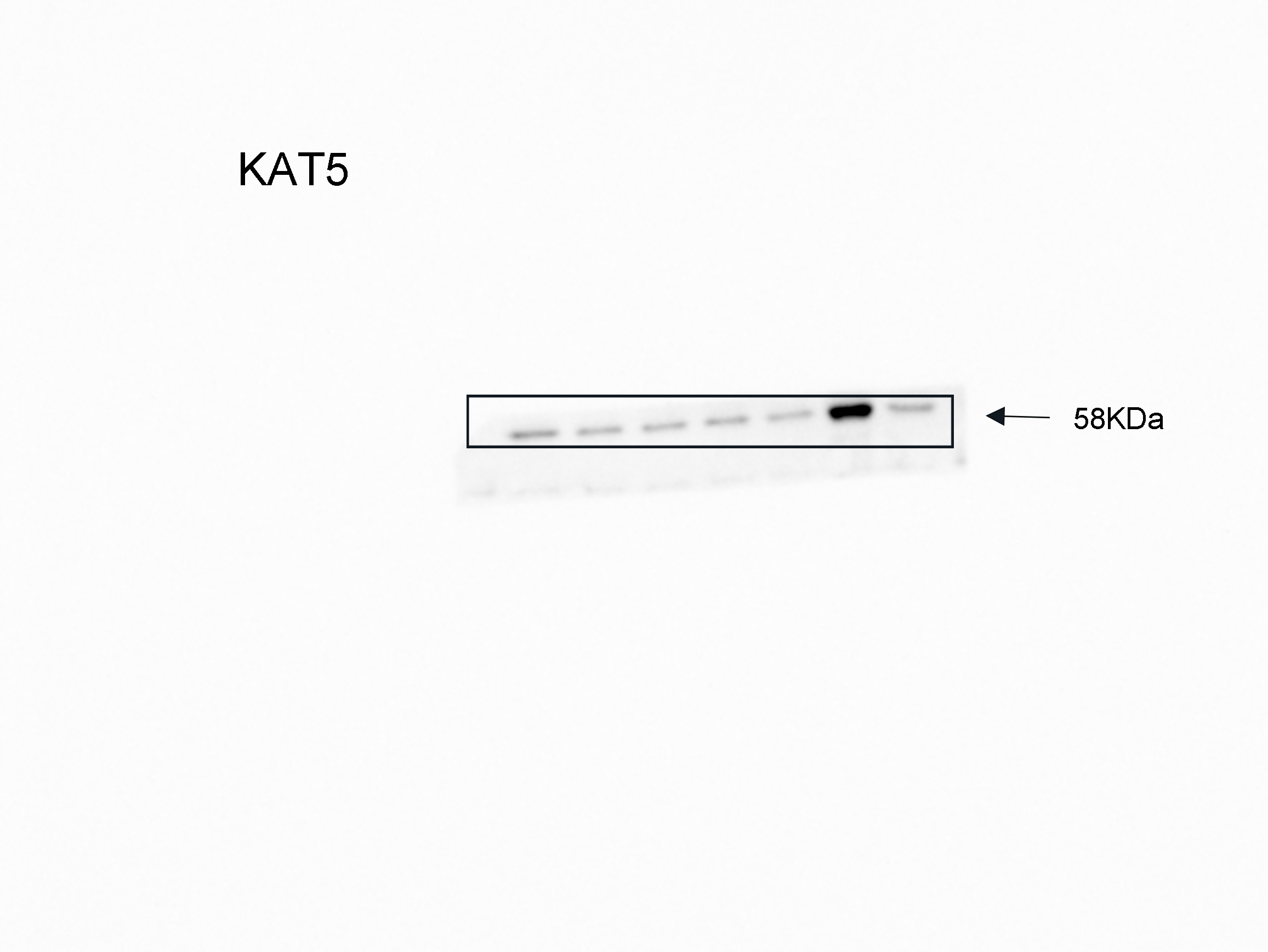

Supplement: Figure 4—source data 2. [file elife-98524-fig4-data2.zip › Fig 4-data2-v1/4M/KAT5 .tif]

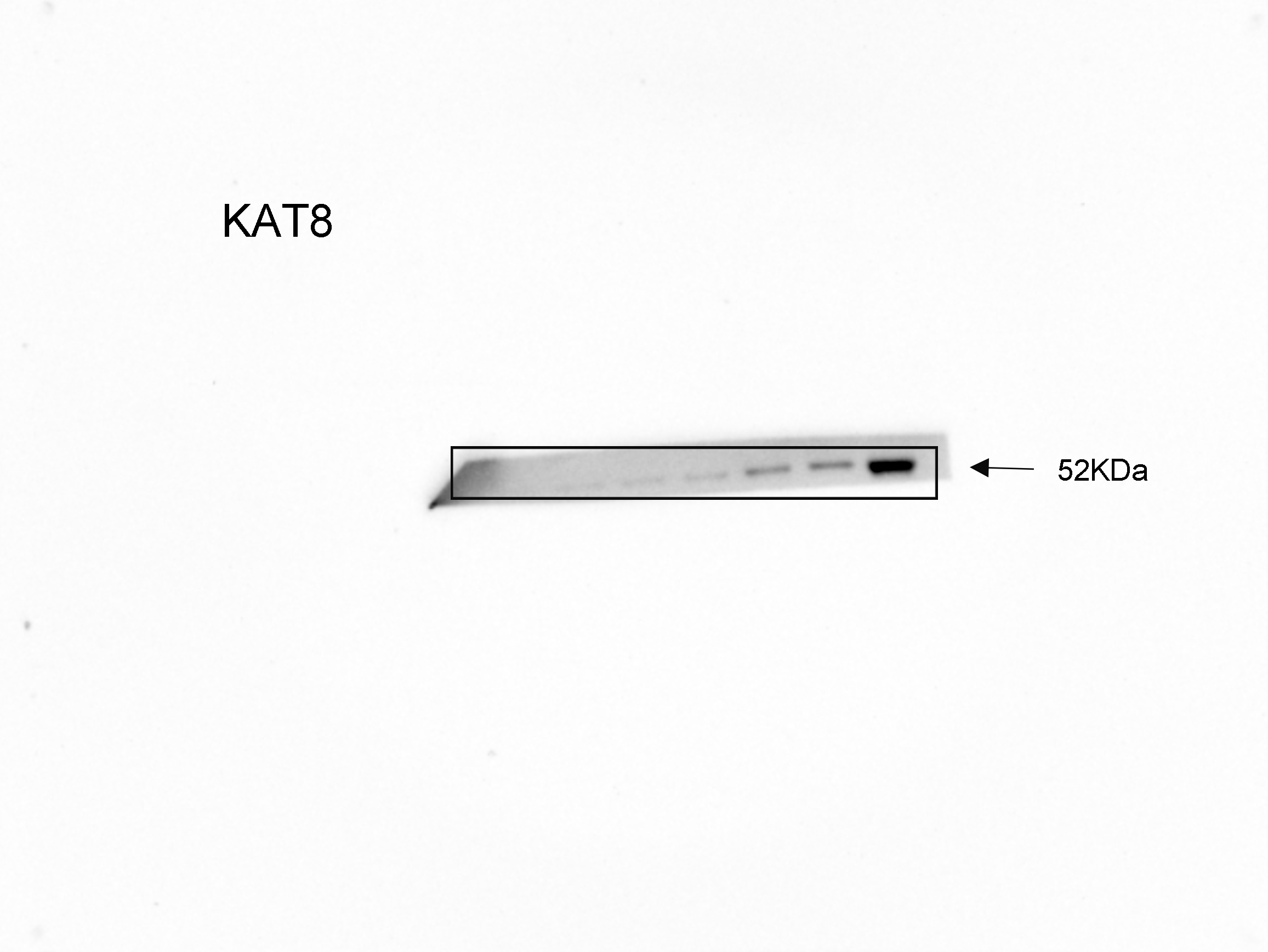

Supplement: Figure 4—source data 2. [file elife-98524-fig4-data2.zip › Fig 4-data2-v1/4M/KAT8 .tif]

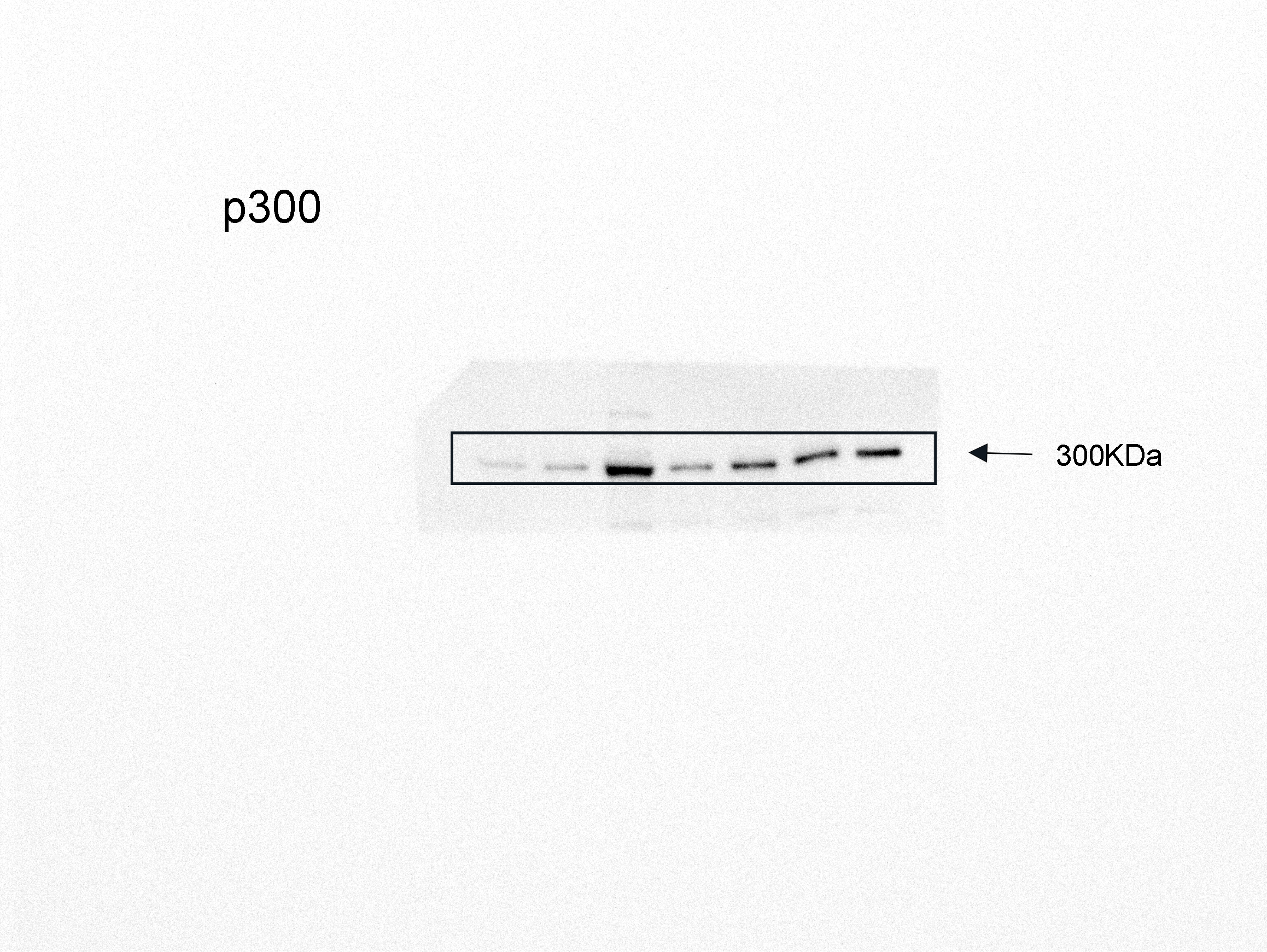

Supplement: Figure 4—source data 2. [file elife-98524-fig4-data2.zip › Fig 4-data2-v1/4M/p300 .tif]

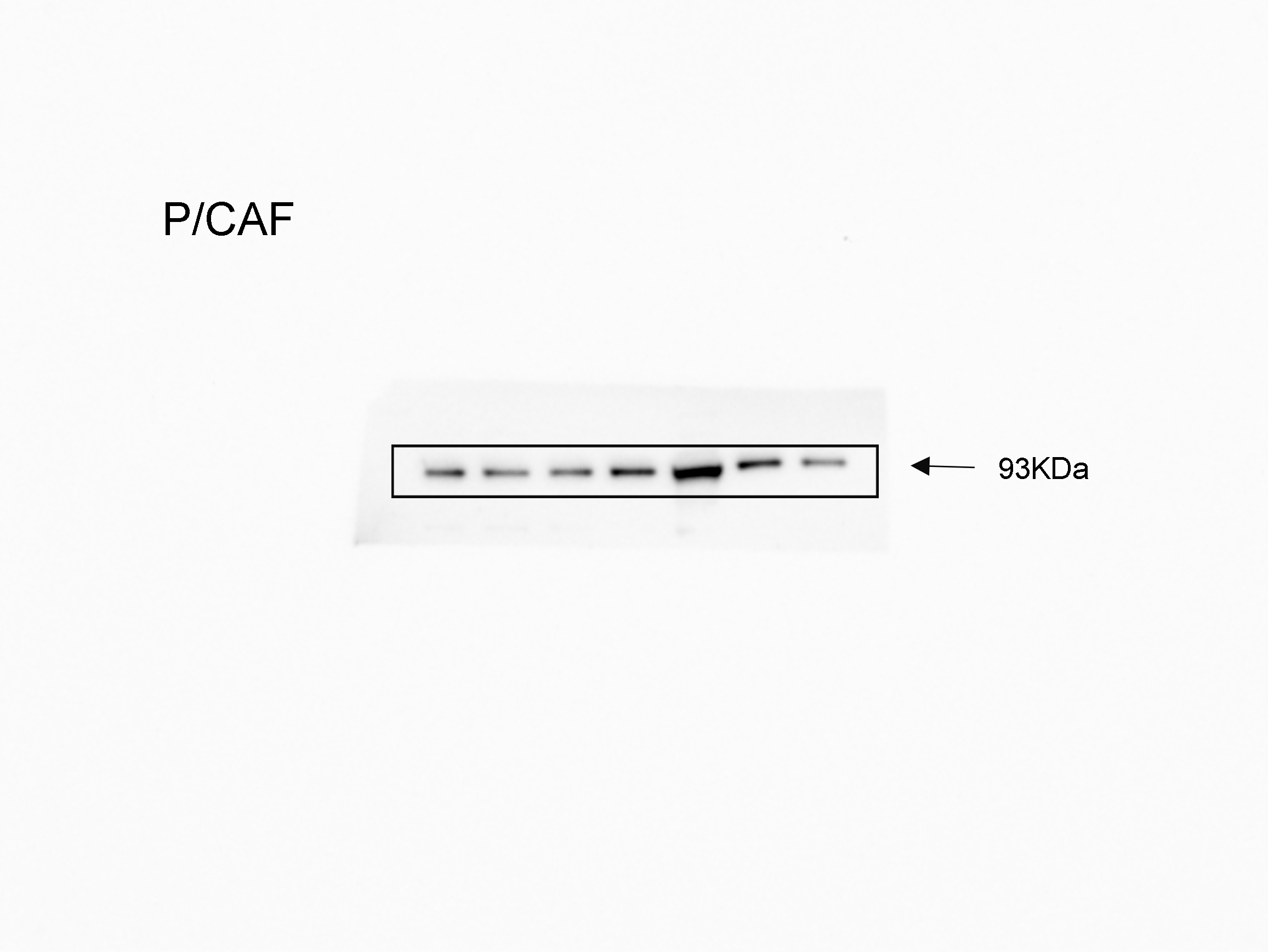

Supplement: Figure 4—source data 2. [file elife-98524-fig4-data2.zip › Fig 4-data2-v1/4M/P_CAF .tif]

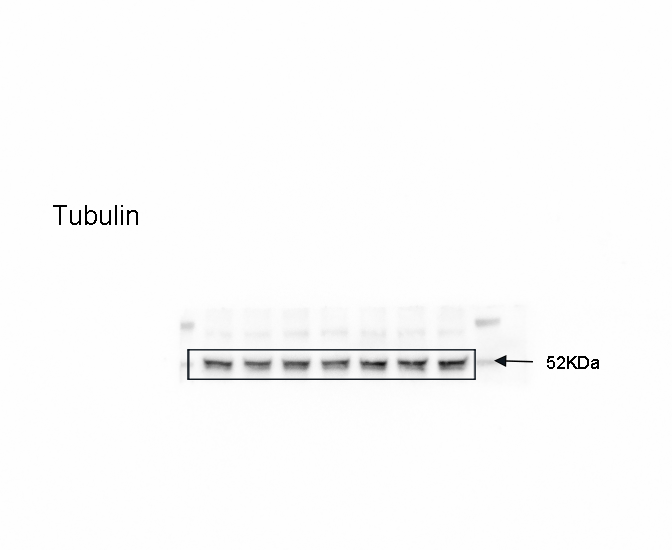

Supplement: Figure 4—source data 2. [file elife-98524-fig4-data2.zip › Fig 4-data2-v1/4M/Tubulin .tif]

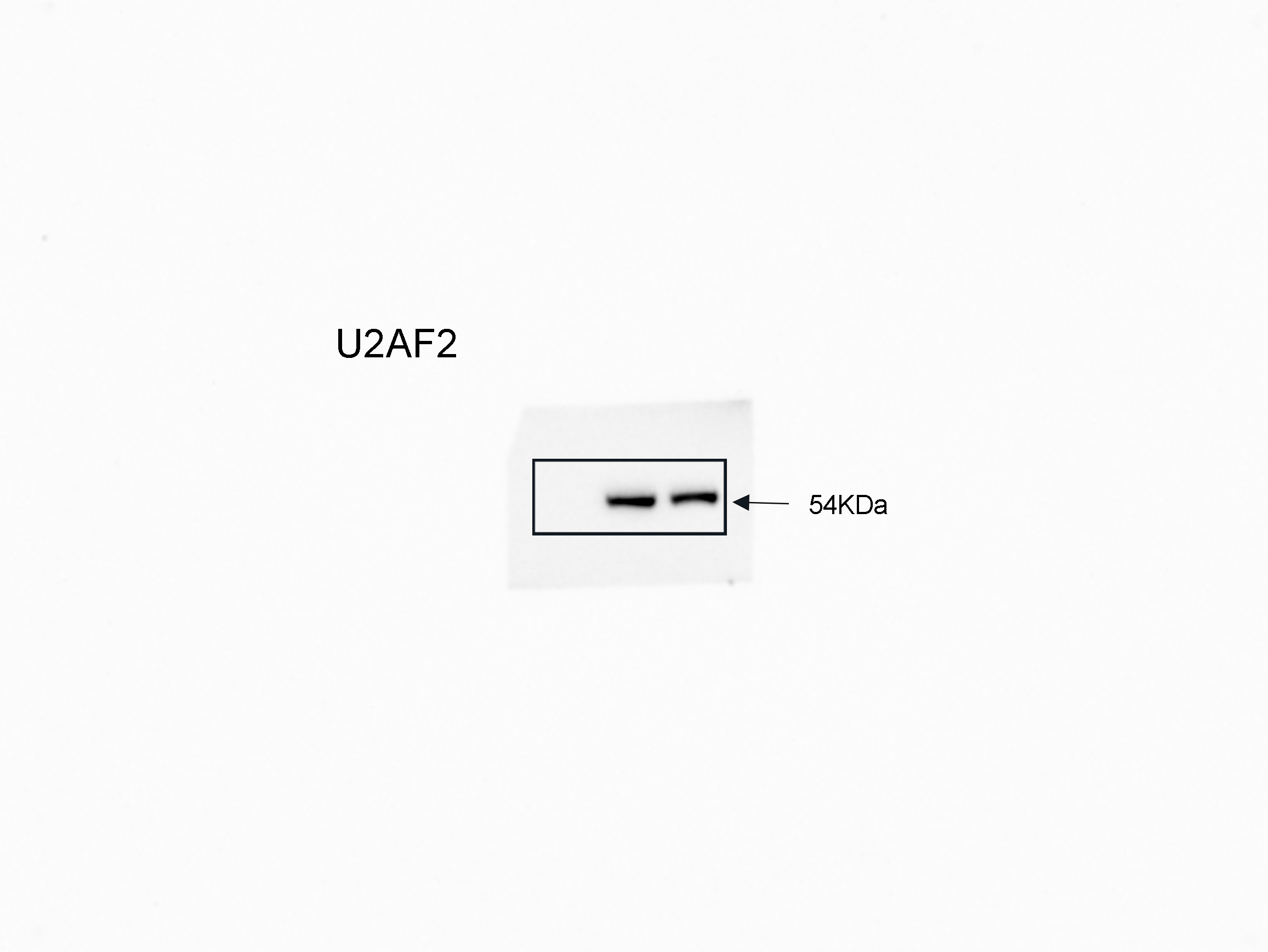

Supplement: Figure 4—source data 2. [file elife-98524-fig4-data2.zip › Fig 4-data2-v1/4N/bottom/Flag .tif]

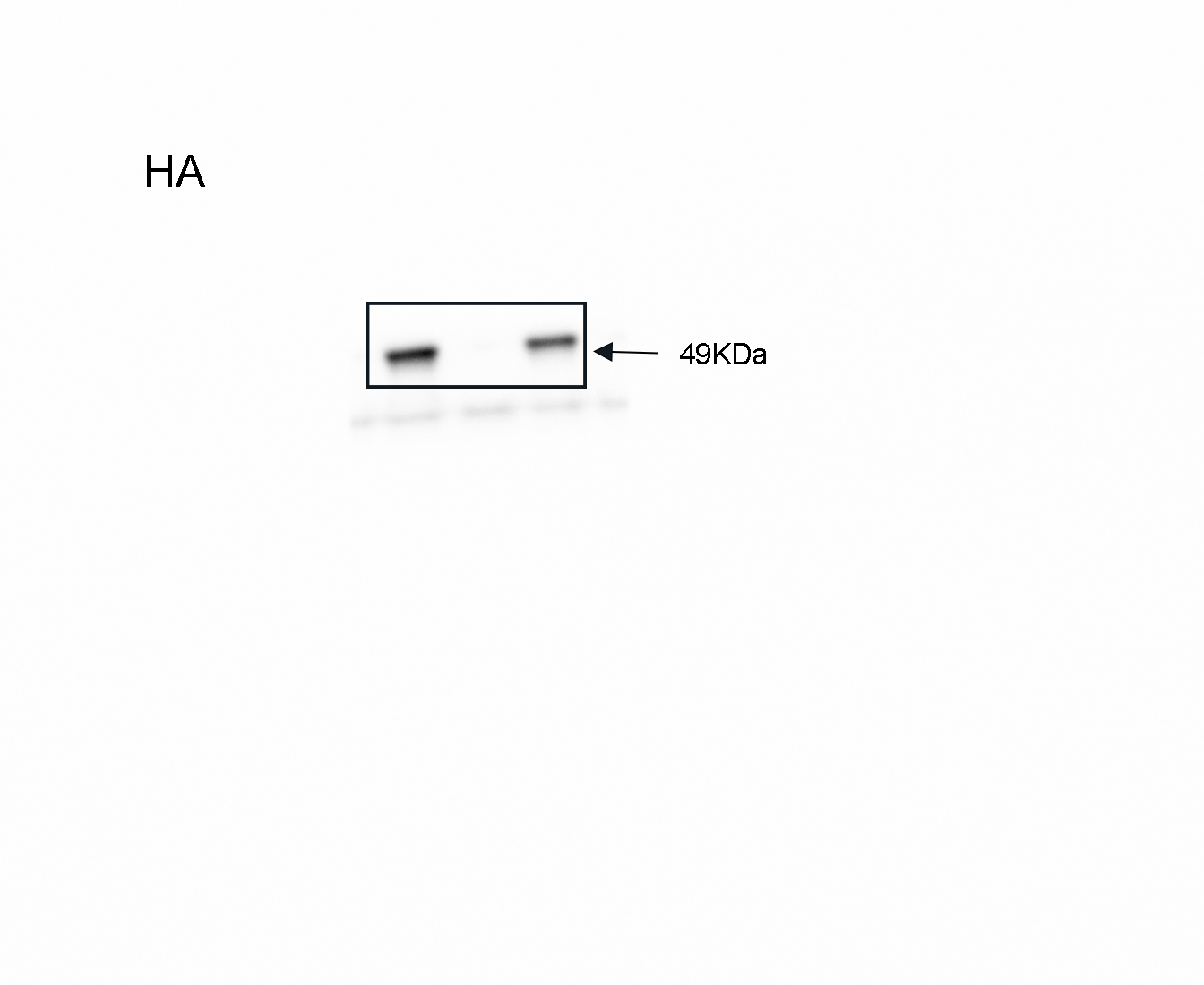

Supplement: Figure 4—source data 2. [file elife-98524-fig4-data2.zip › Fig 4-data2-v1/4N/bottom/HA .tif]

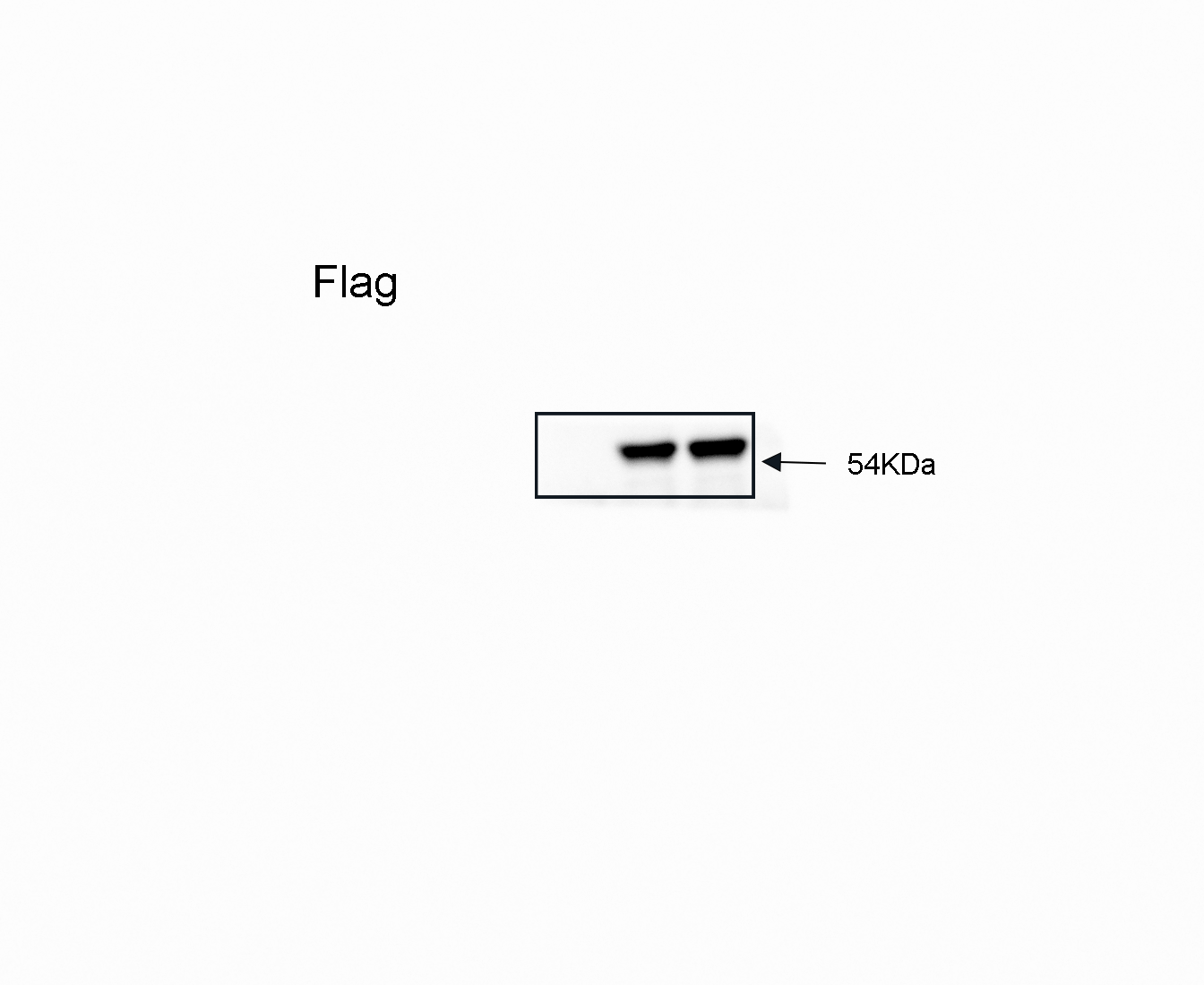

Supplement: Figure 4—source data 2. [file elife-98524-fig4-data2.zip › Fig 4-data2-v1/4N/middle/Flag .tif]

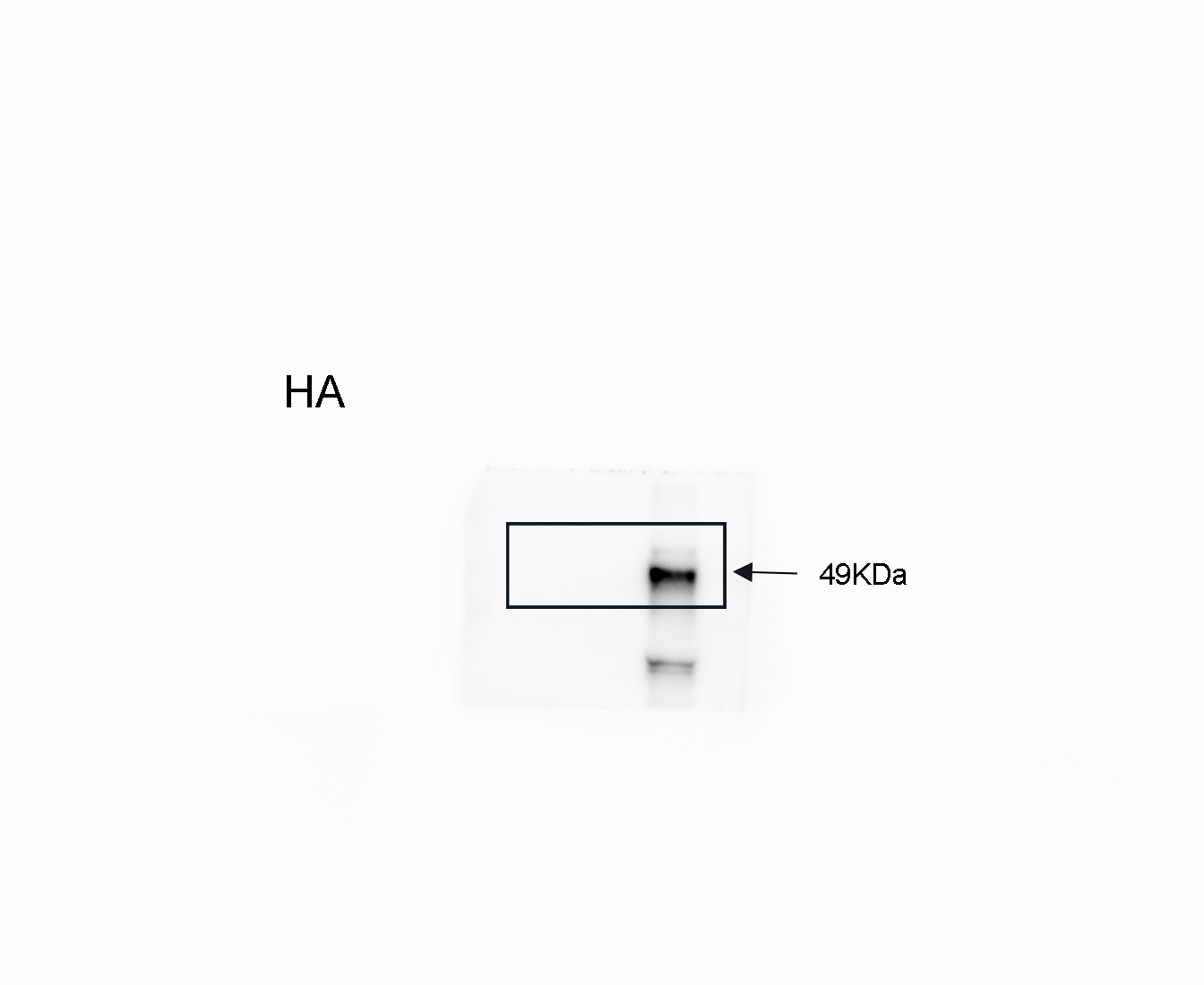

Supplement: Figure 4—source data 2. [file elife-98524-fig4-data2.zip › Fig 4-data2-v1/4N/middle/HA .tif]

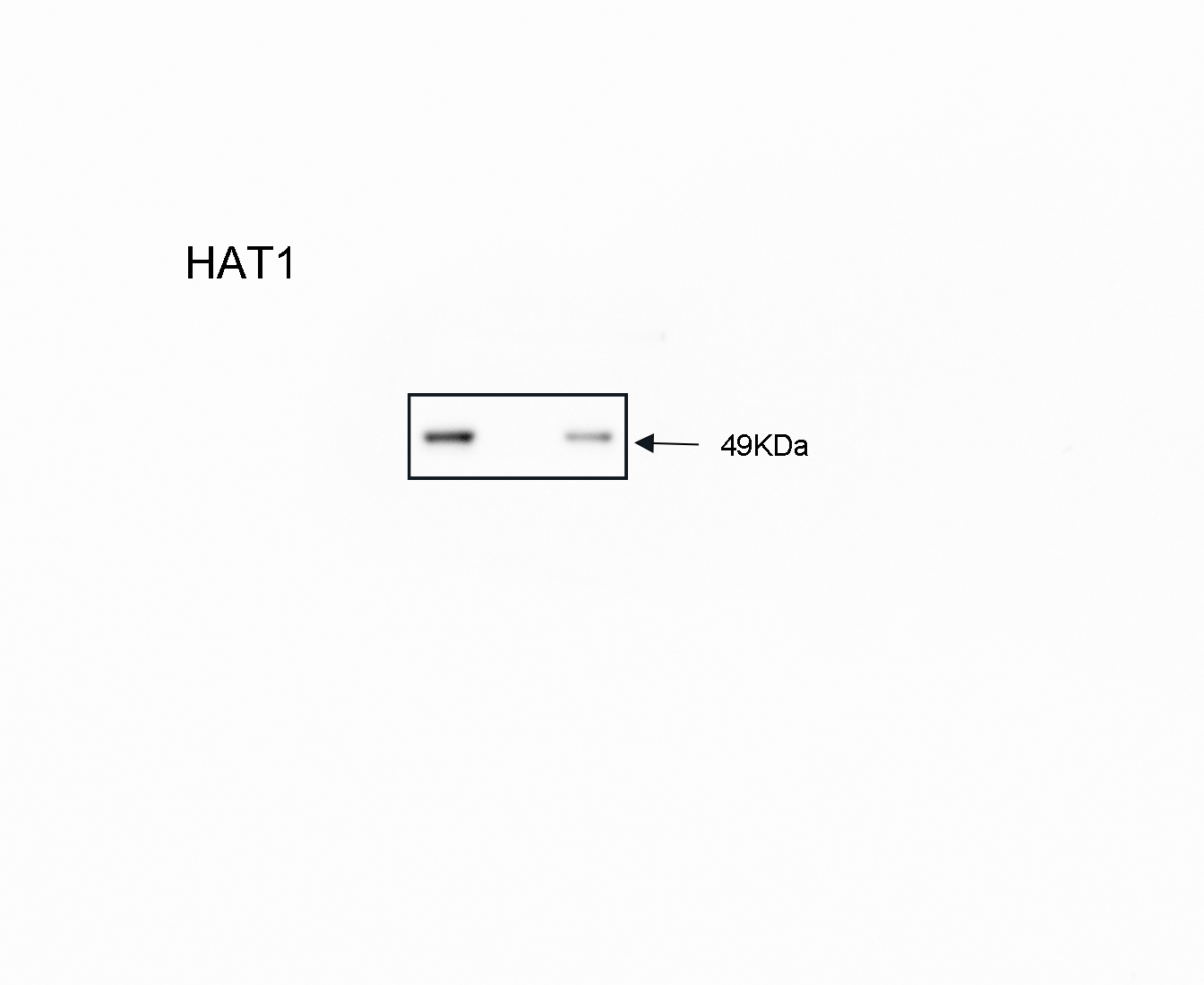

Supplement: Figure 4—source data 2. [file elife-98524-fig4-data2.zip › Fig 4-data2-v1/4N/upper/HAT1 .tif]

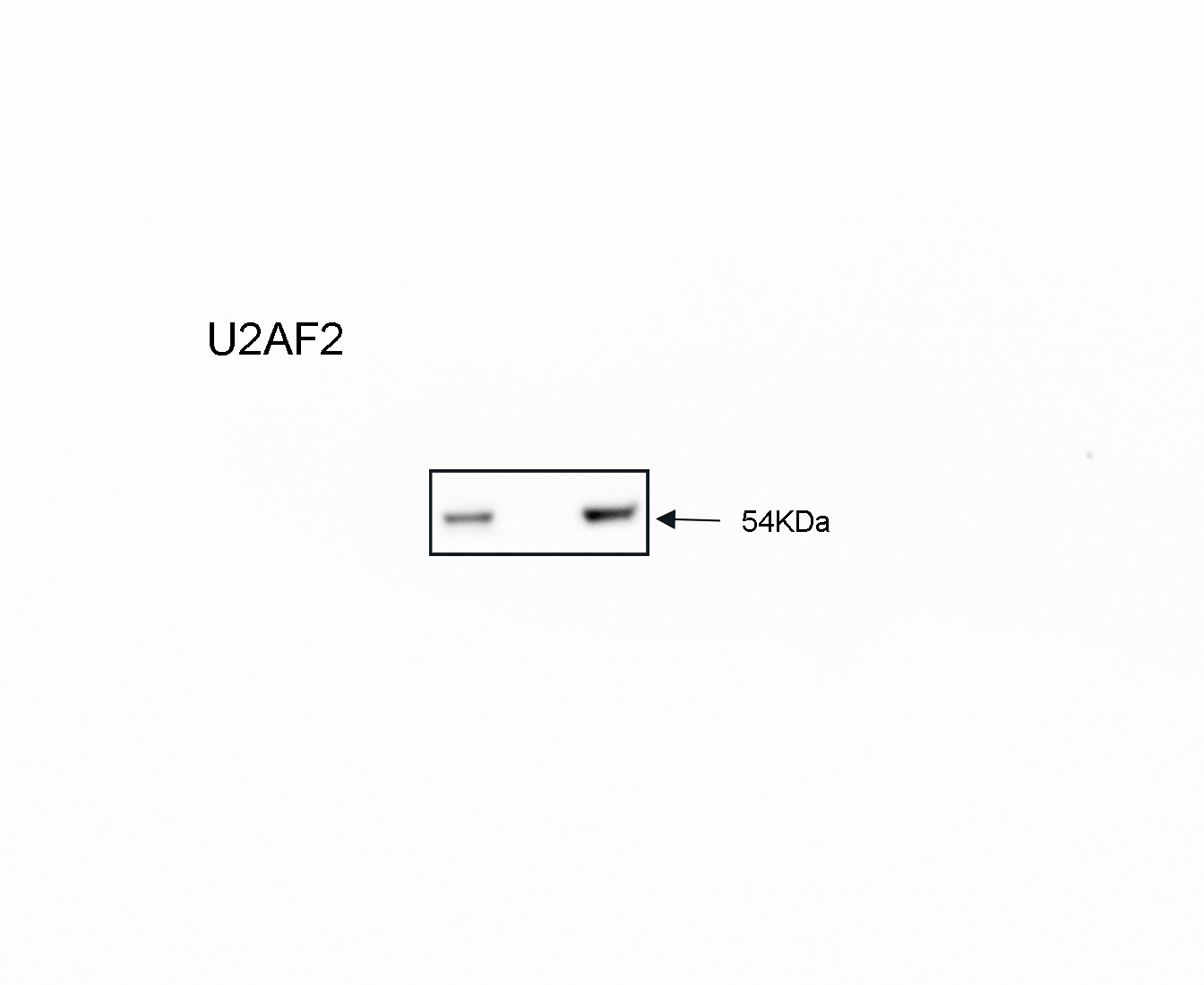

Supplement: Figure 4—source data 2. [file elife-98524-fig4-data2.zip › Fig 4-data2-v1/4N/upper/U2AF2.tif]

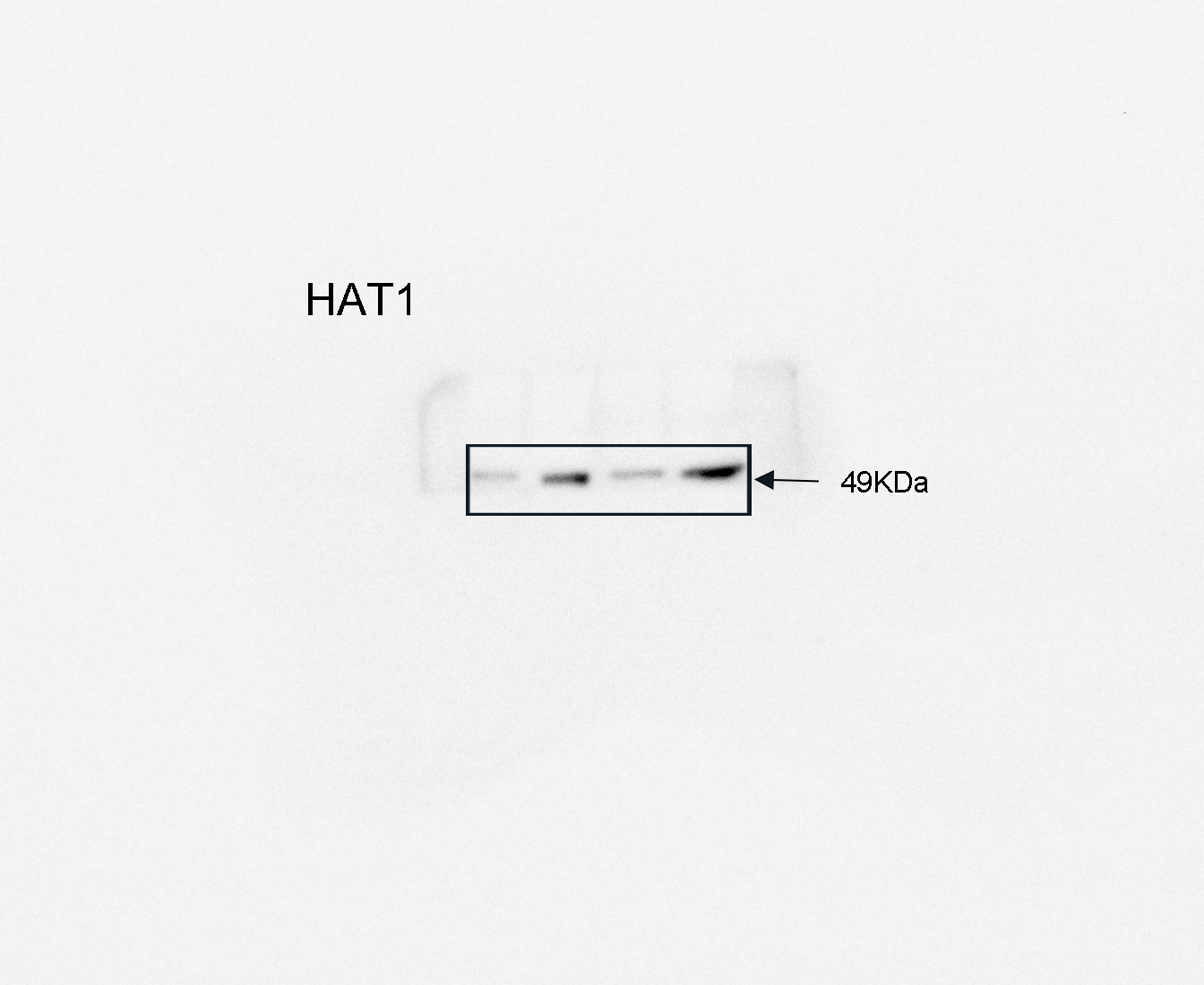

Supplement: Figure 4—source data 2. [file elife-98524-fig4-data2.zip › Fig 4-data2-v1/4P/bottom/HAT1 .tif]

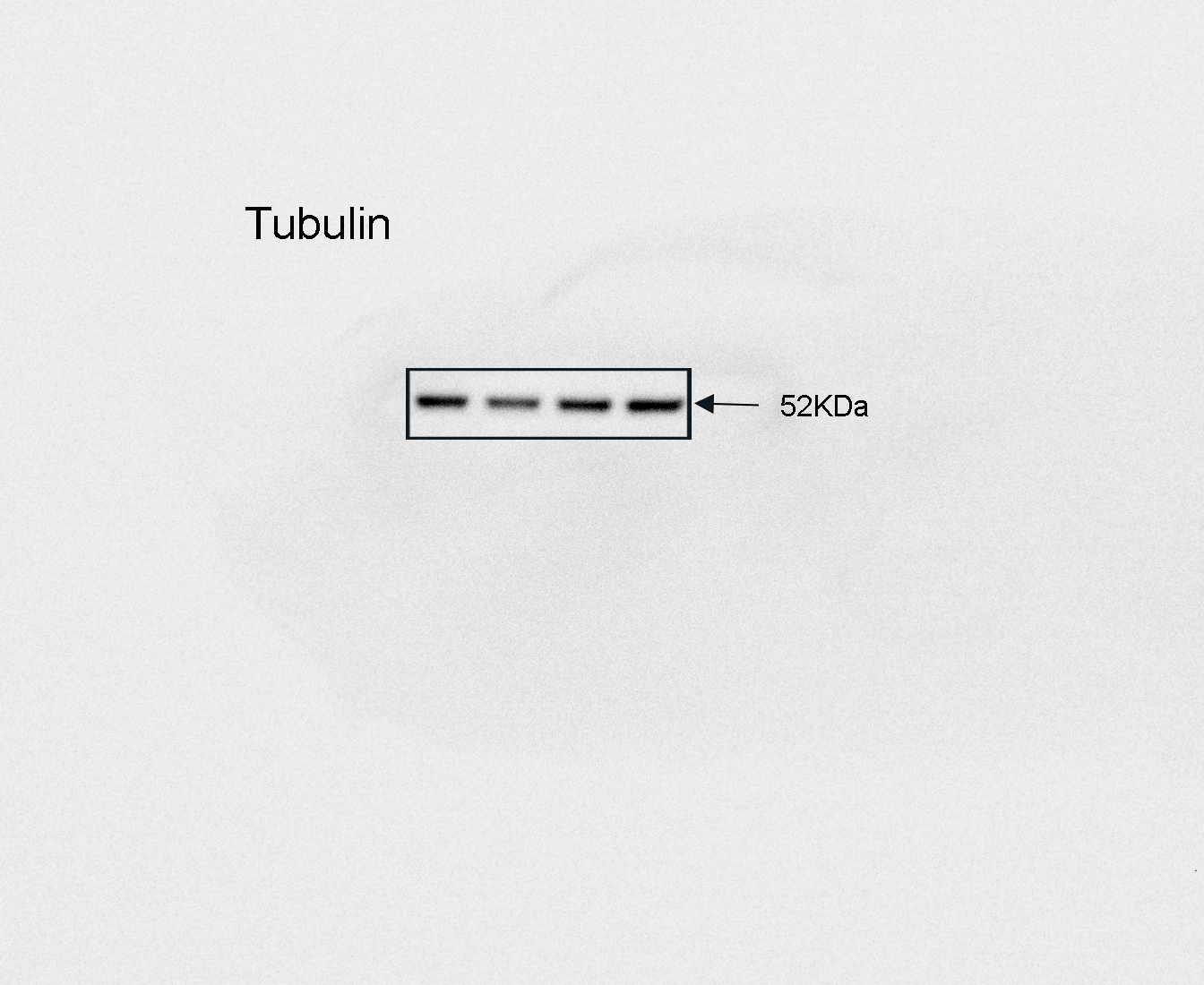

Supplement: Figure 4—source data 2. [file elife-98524-fig4-data2.zip › Fig 4-data2-v1/4P/bottom/Tubulin .tif]

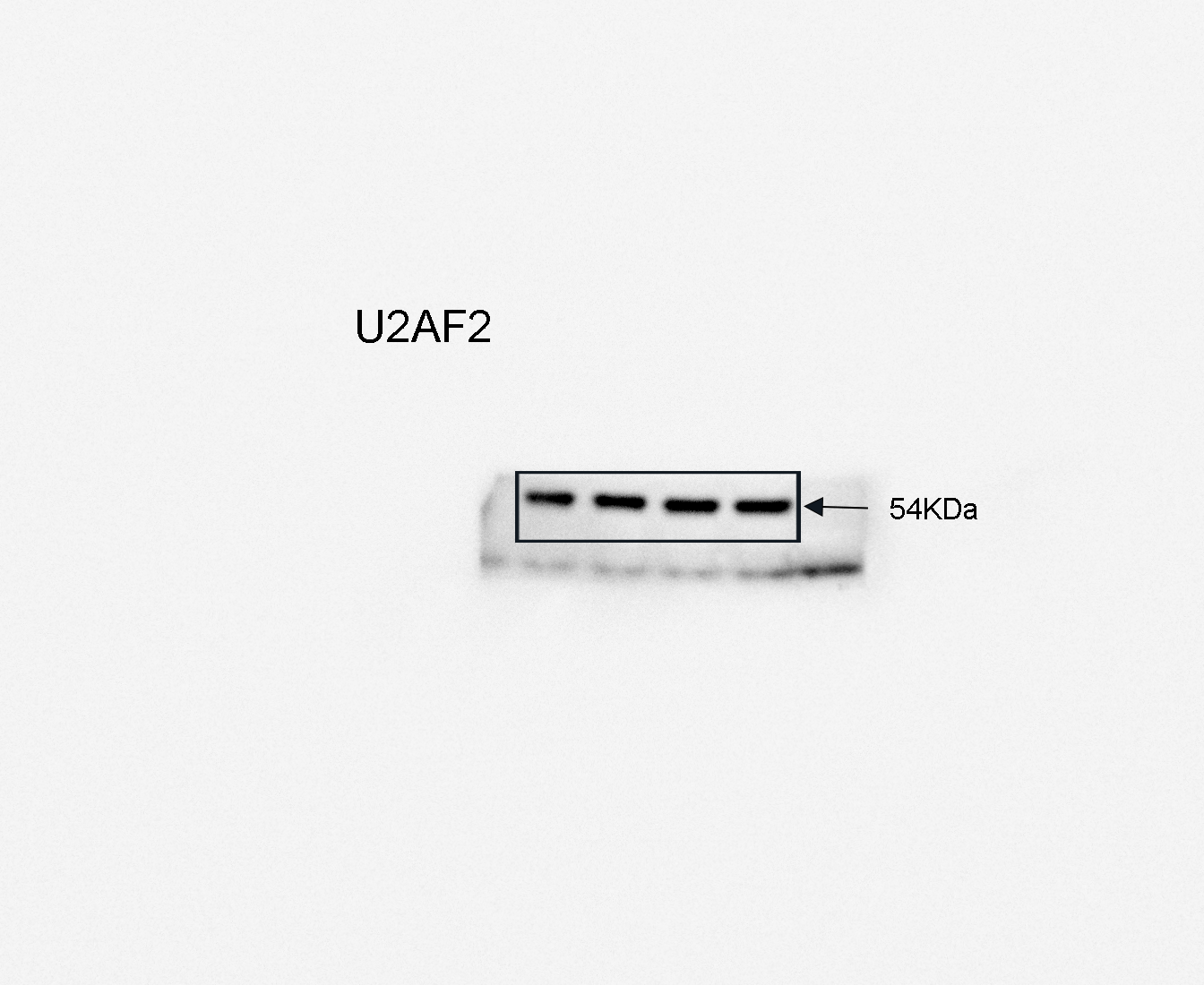

Supplement: Figure 4—source data 2. [file elife-98524-fig4-data2.zip › Fig 4-data2-v1/4P/bottom/U2AF2 .tif]

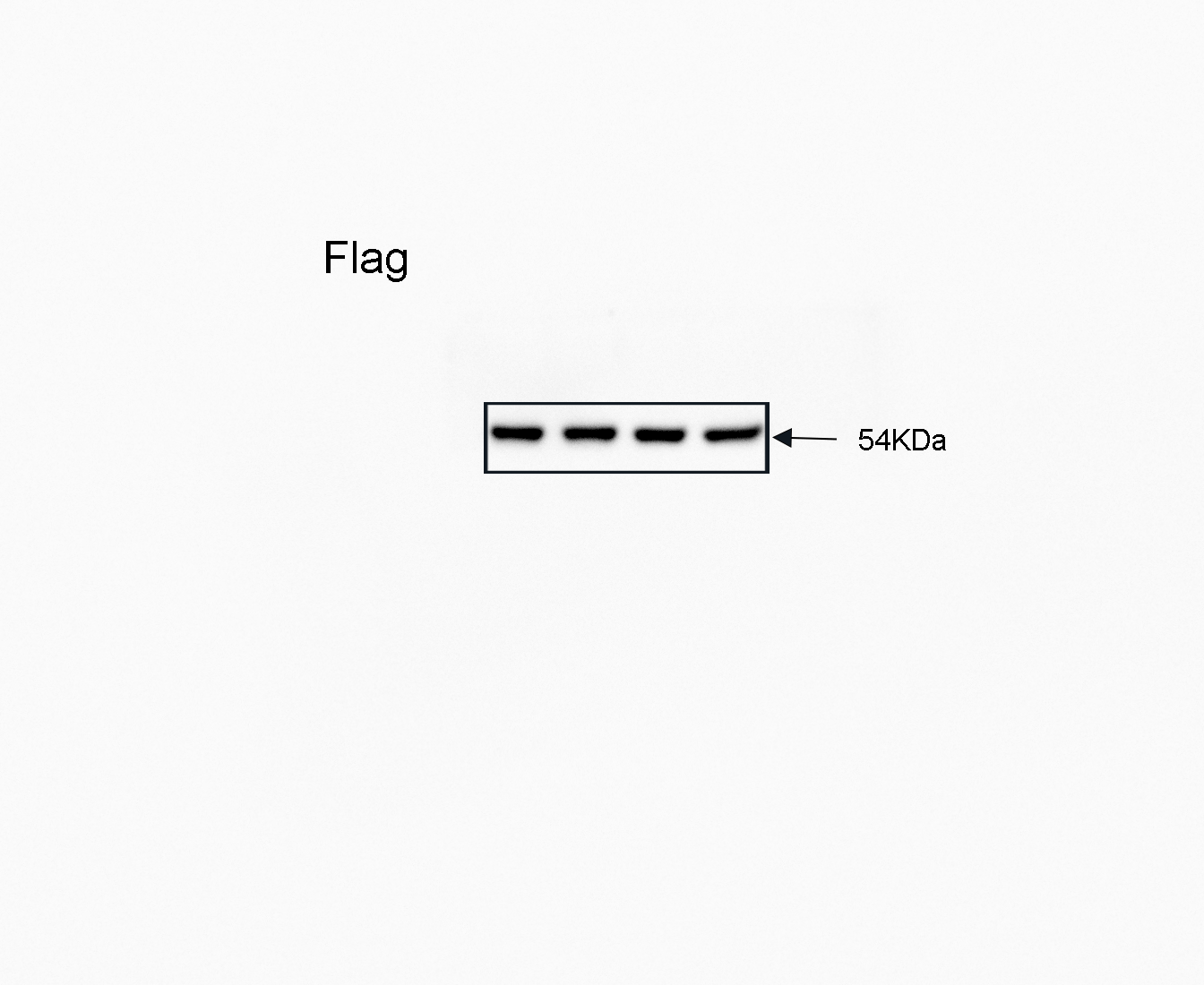

Supplement: Figure 4—source data 2. [file elife-98524-fig4-data2.zip › Fig 4-data2-v1/4P/middle/Flag .tif]

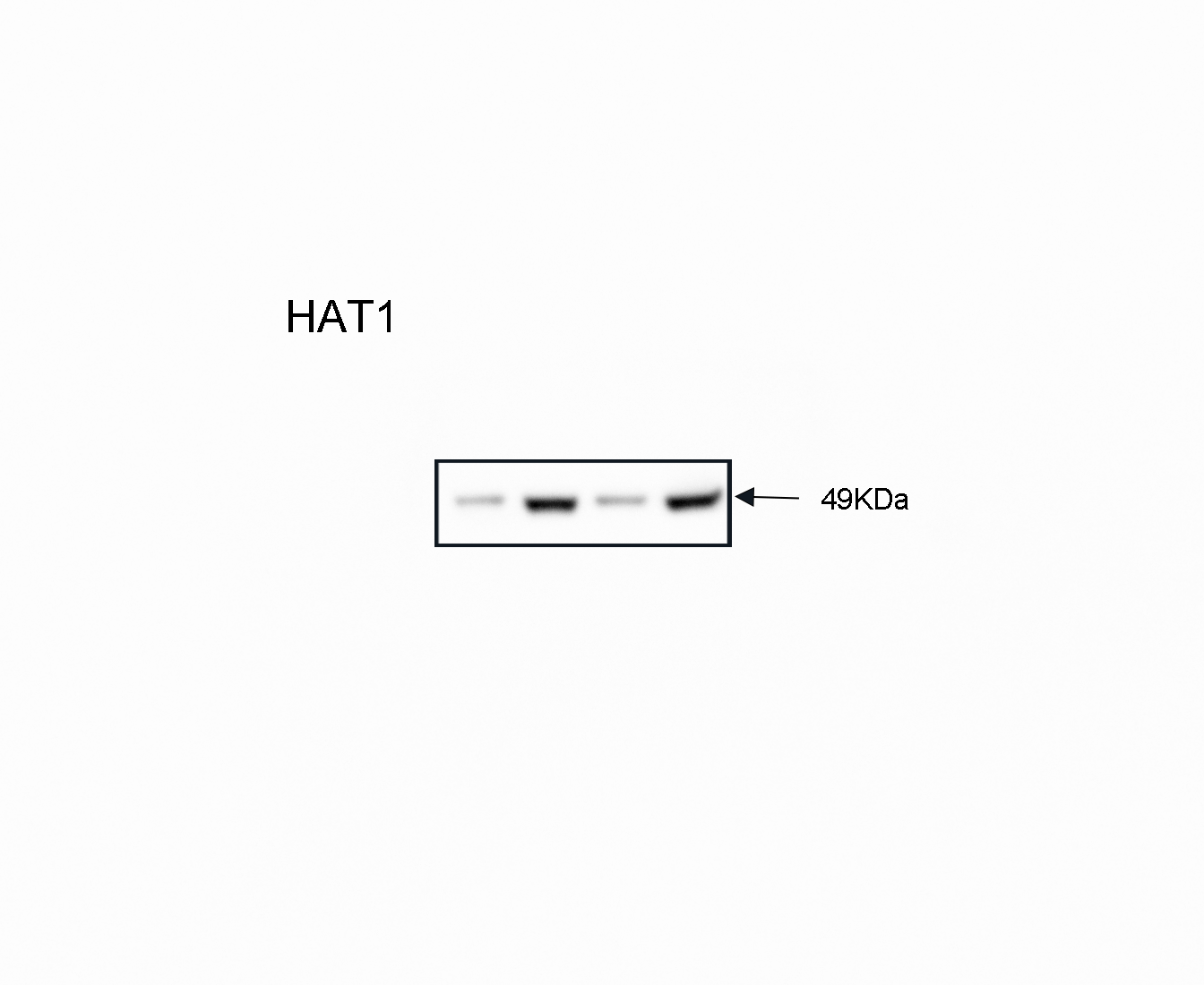

Supplement: Figure 4—source data 2. [file elife-98524-fig4-data2.zip › Fig 4-data2-v1/4P/middle/HAT1 .tif]

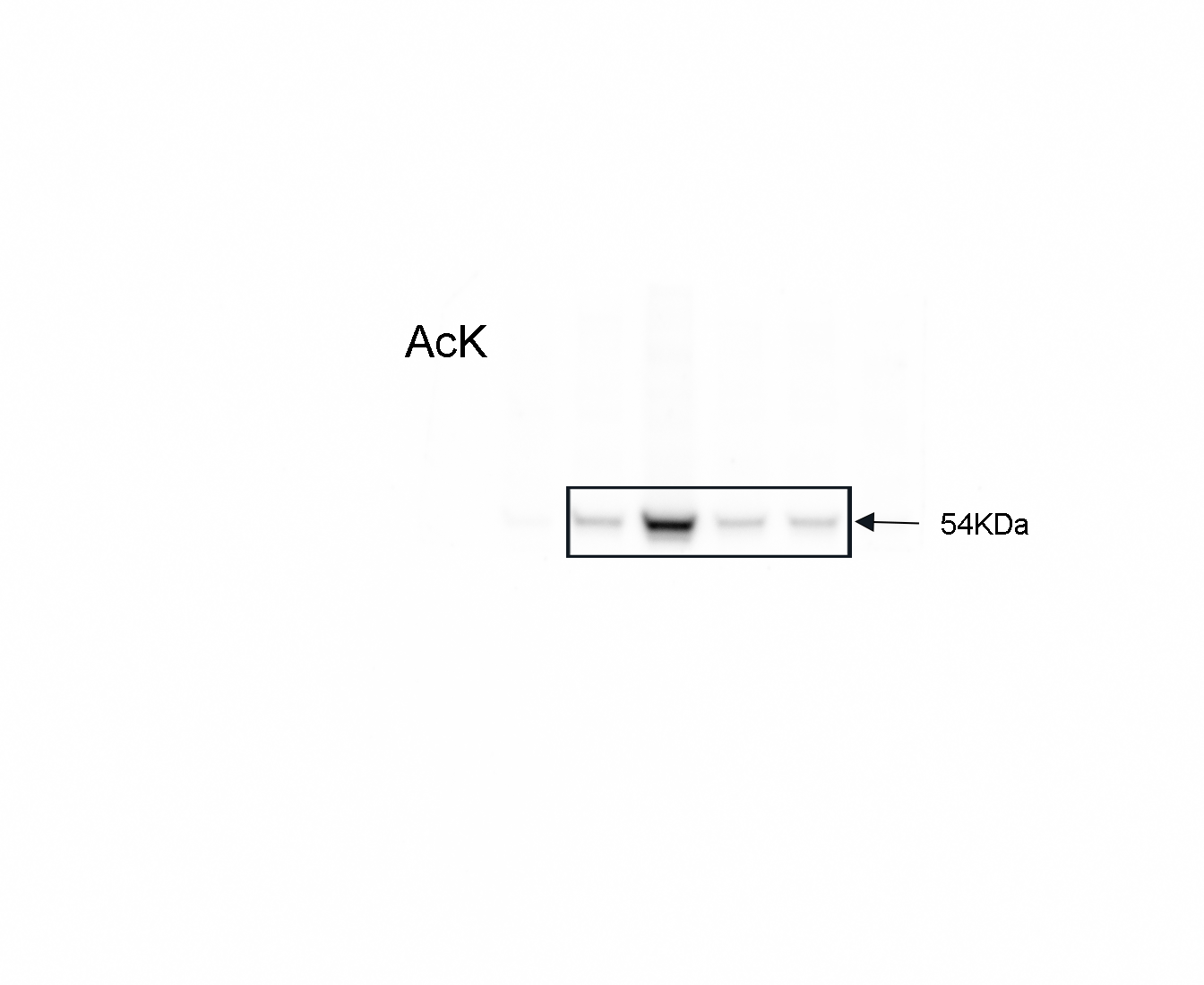

Supplement: Figure 4—source data 2. [file elife-98524-fig4-data2.zip › Fig 4-data2-v1/4P/upper/AcK .tif]

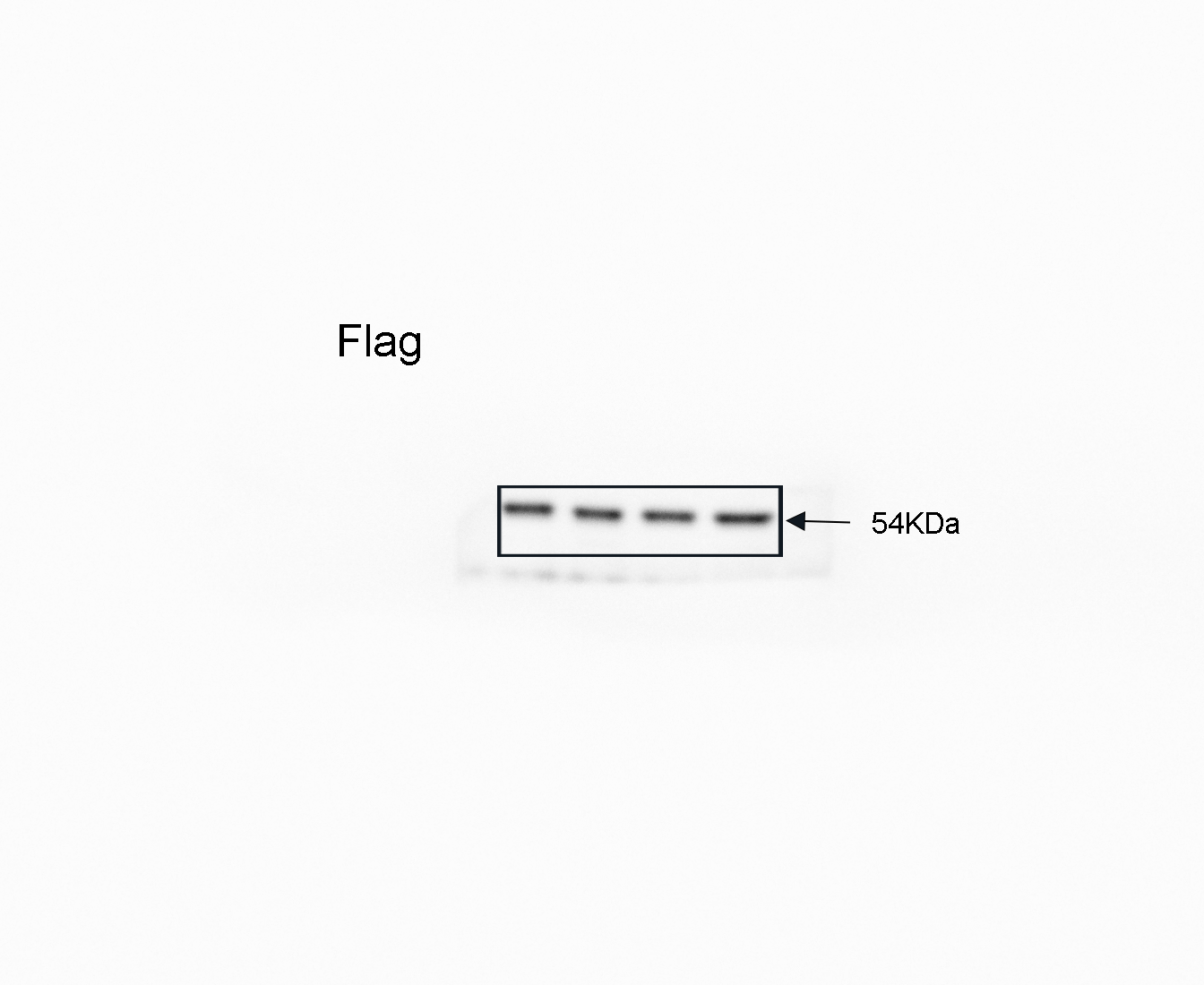

Supplement: Figure 4—source data 2. [file elife-98524-fig4-data2.zip › Fig 4-data2-v1/4P/upper/Flag .tif]

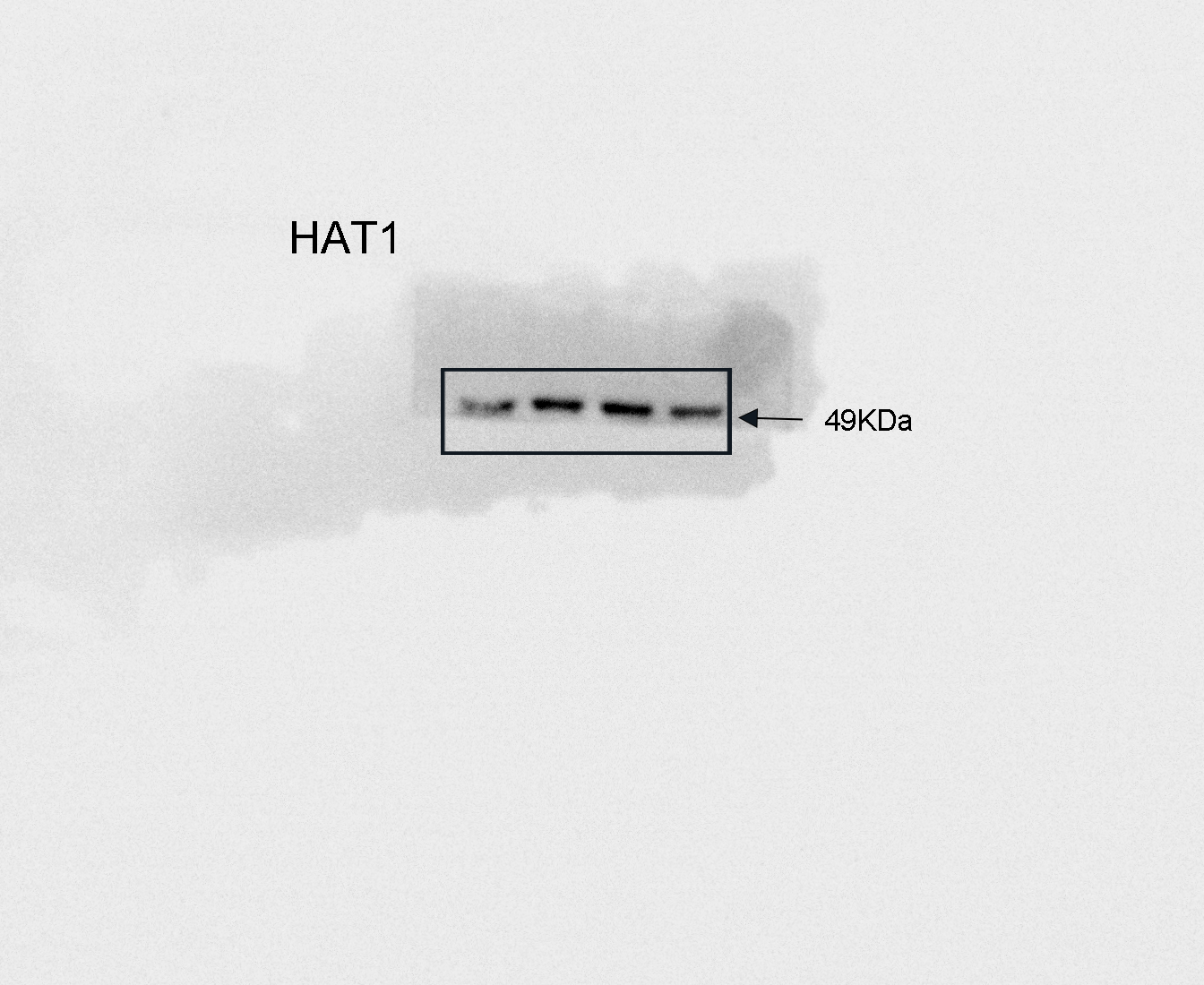

Supplement: Figure 4—source data 2. [file elife-98524-fig4-data2.zip › Fig 4-data2-v1/4Q/bottom left/HAT1 .tif]

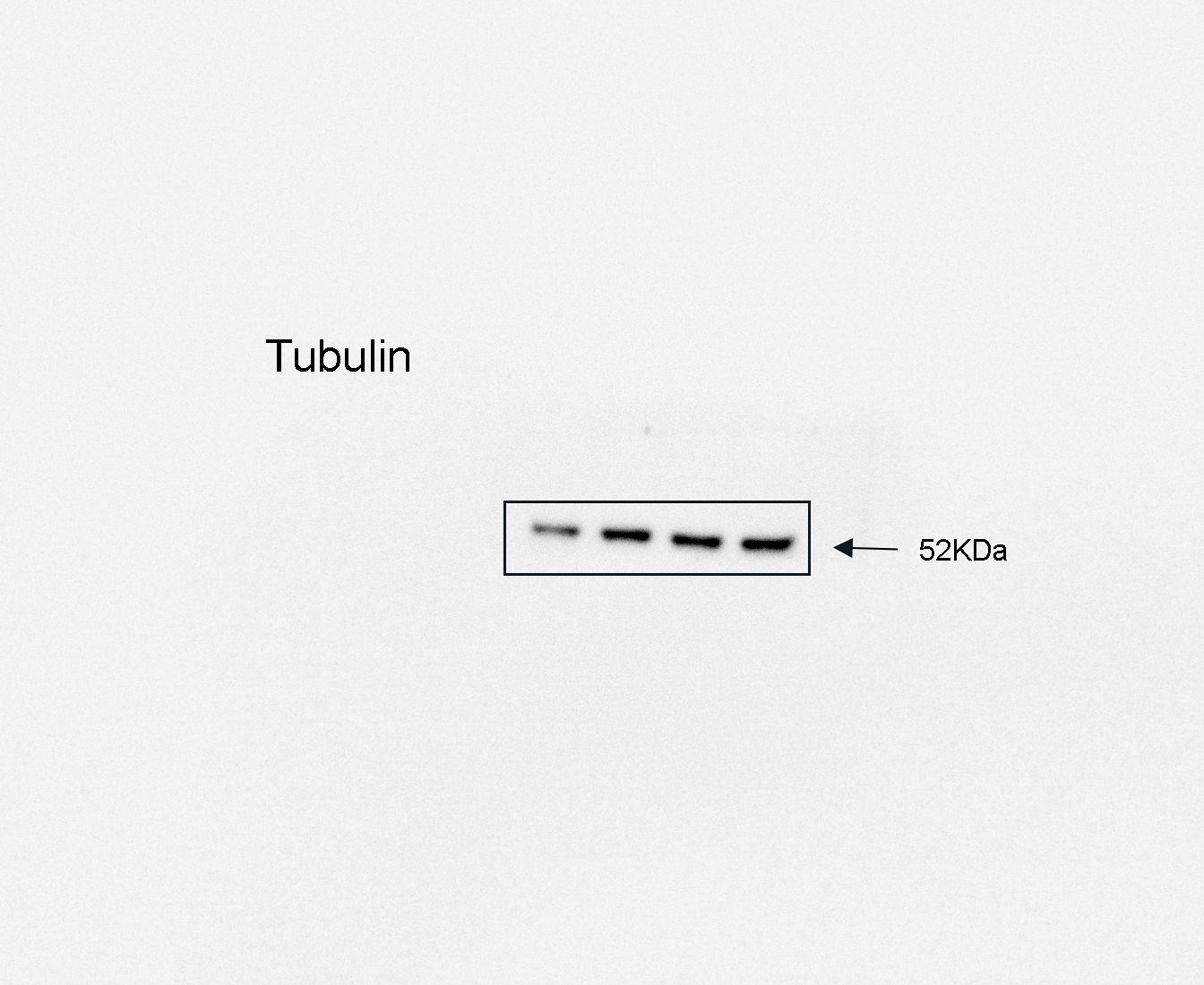

Supplement: Figure 4—source data 2. [file elife-98524-fig4-data2.zip › Fig 4-data2-v1/4Q/bottom left/Tubulin .tif]

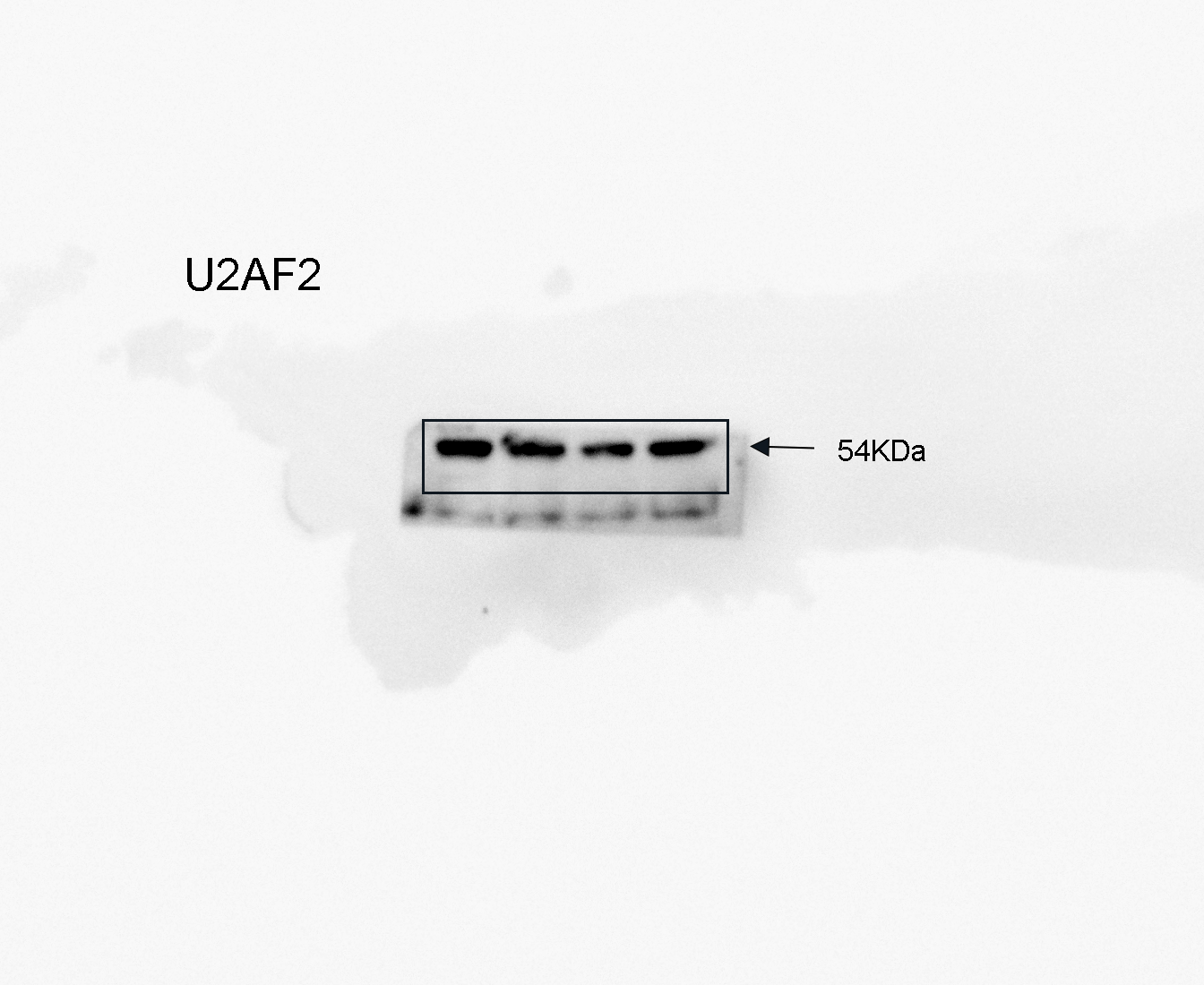

Supplement: Figure 4—source data 2. [file elife-98524-fig4-data2.zip › Fig 4-data2-v1/4Q/bottom left/U2AF2 .tif]

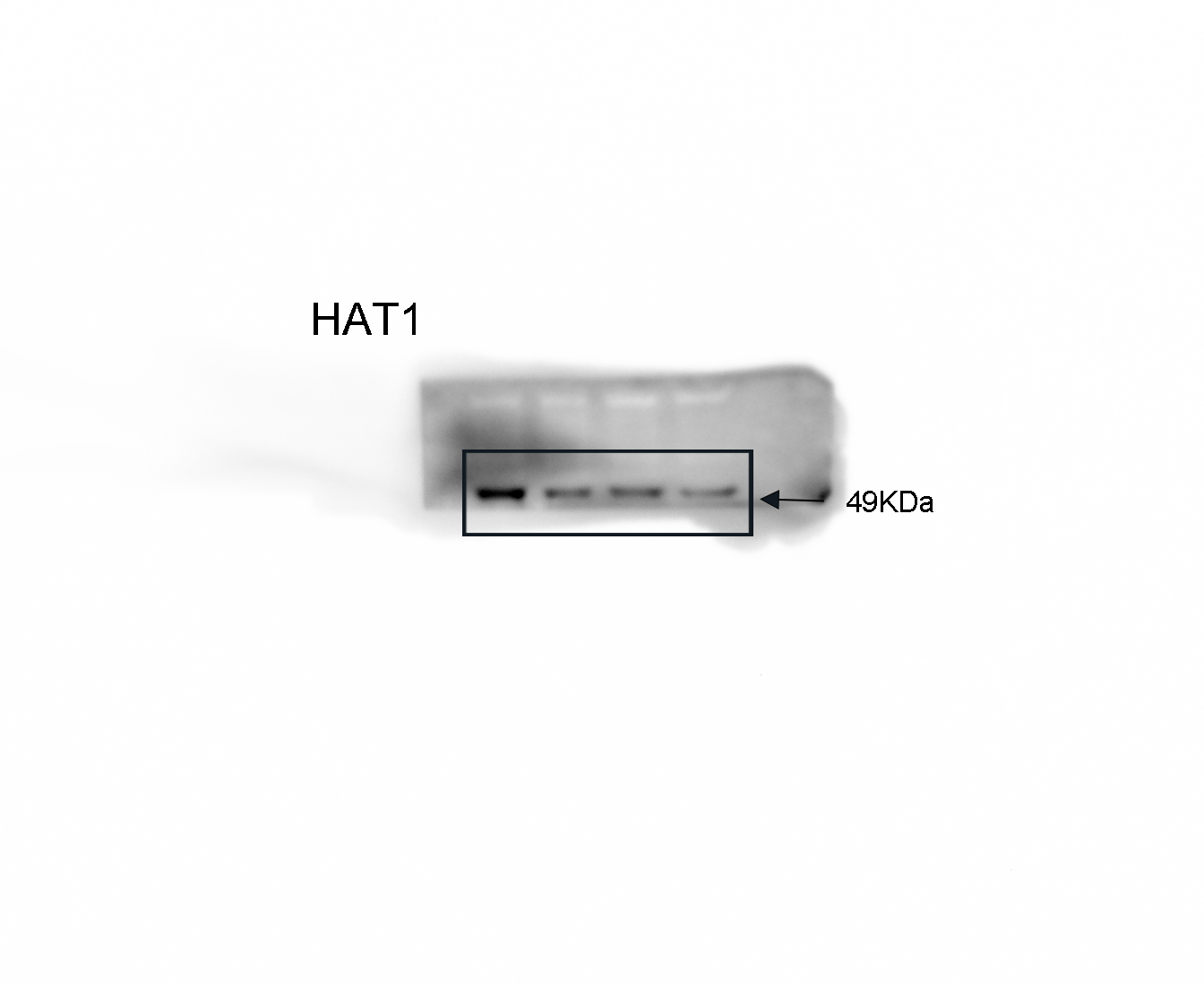

Supplement: Figure 4—source data 2. [file elife-98524-fig4-data2.zip › Fig 4-data2-v1/4Q/bottom right/HAT1 .tif]

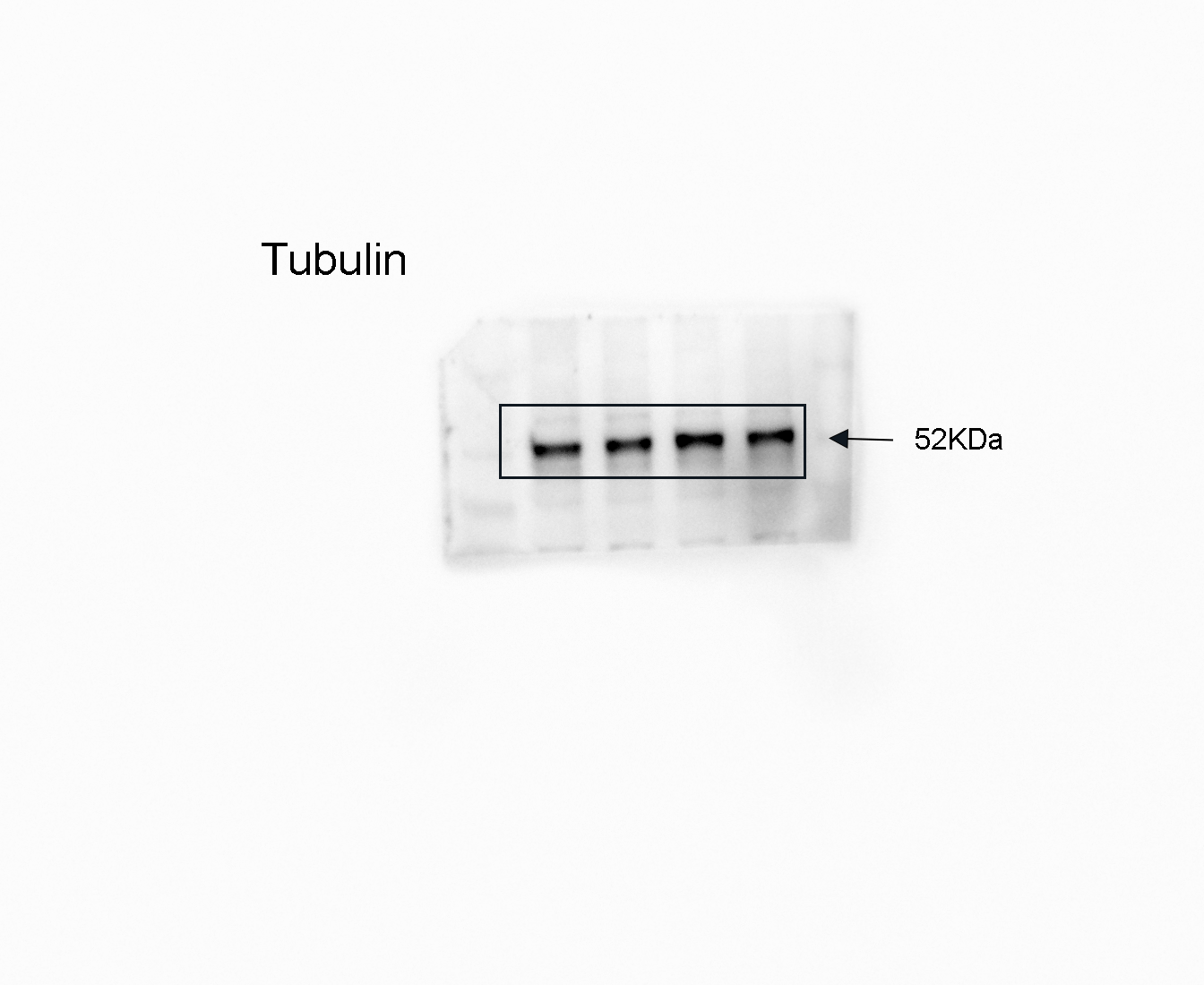

Supplement: Figure 4—source data 2. [file elife-98524-fig4-data2.zip › Fig 4-data2-v1/4Q/bottom right/Tubulin .tif]

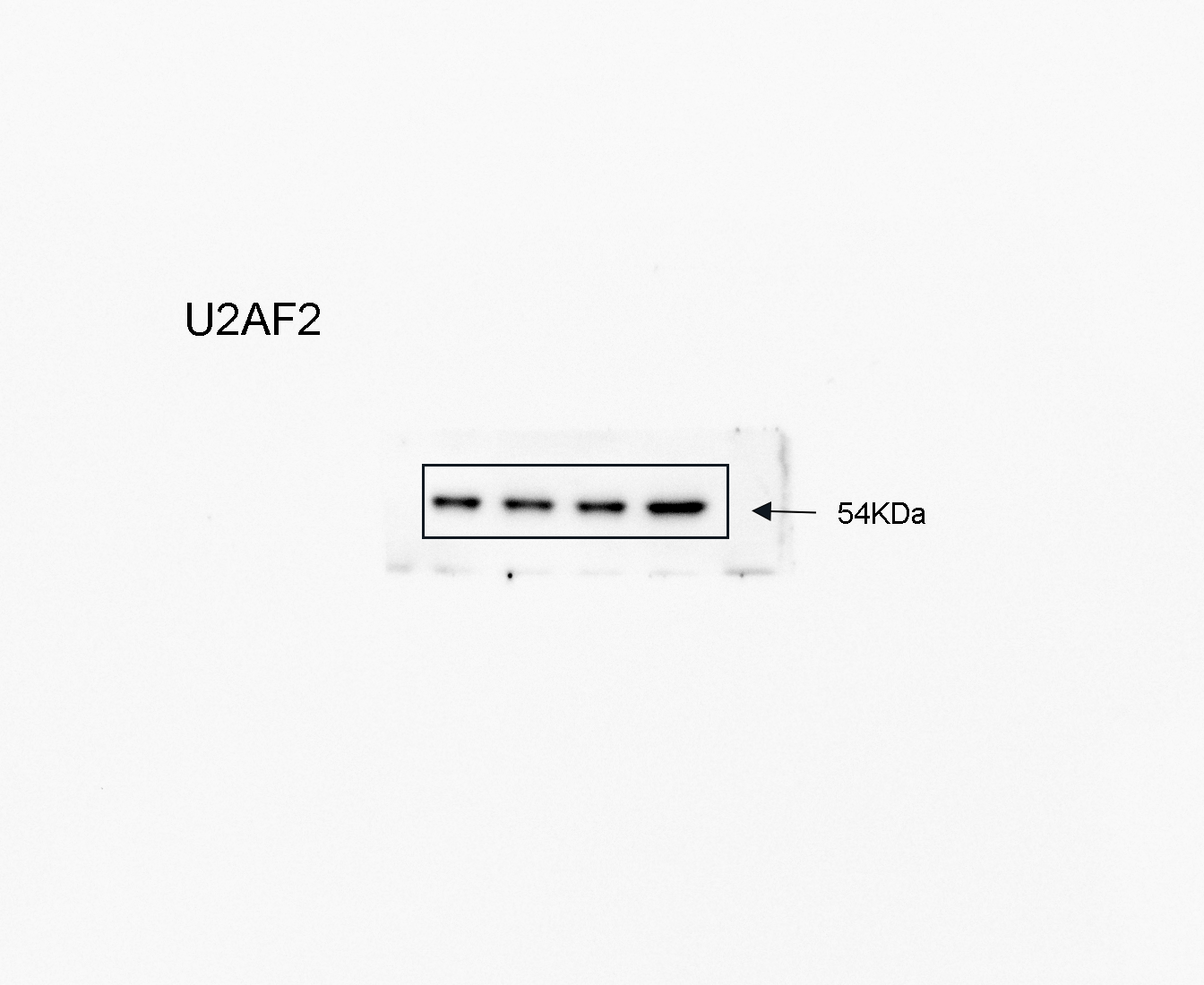

Supplement: Figure 4—source data 2. [file elife-98524-fig4-data2.zip › Fig 4-data2-v1/4Q/bottom right/U2AF2 .tif]

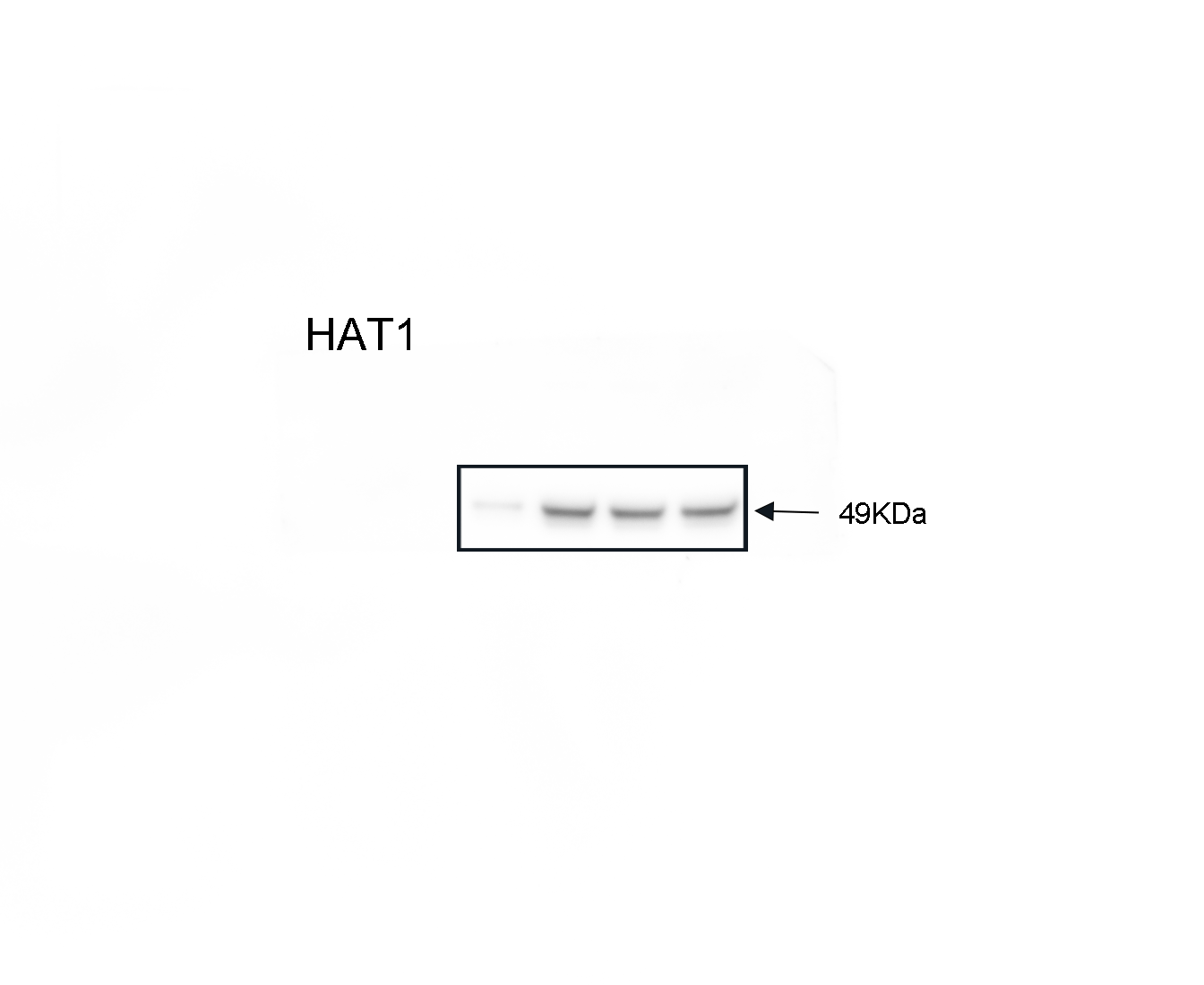

Supplement: Figure 4—source data 2. [file elife-98524-fig4-data2.zip › Fig 4-data2-v1/4Q/upper left/HAT1 .tif]

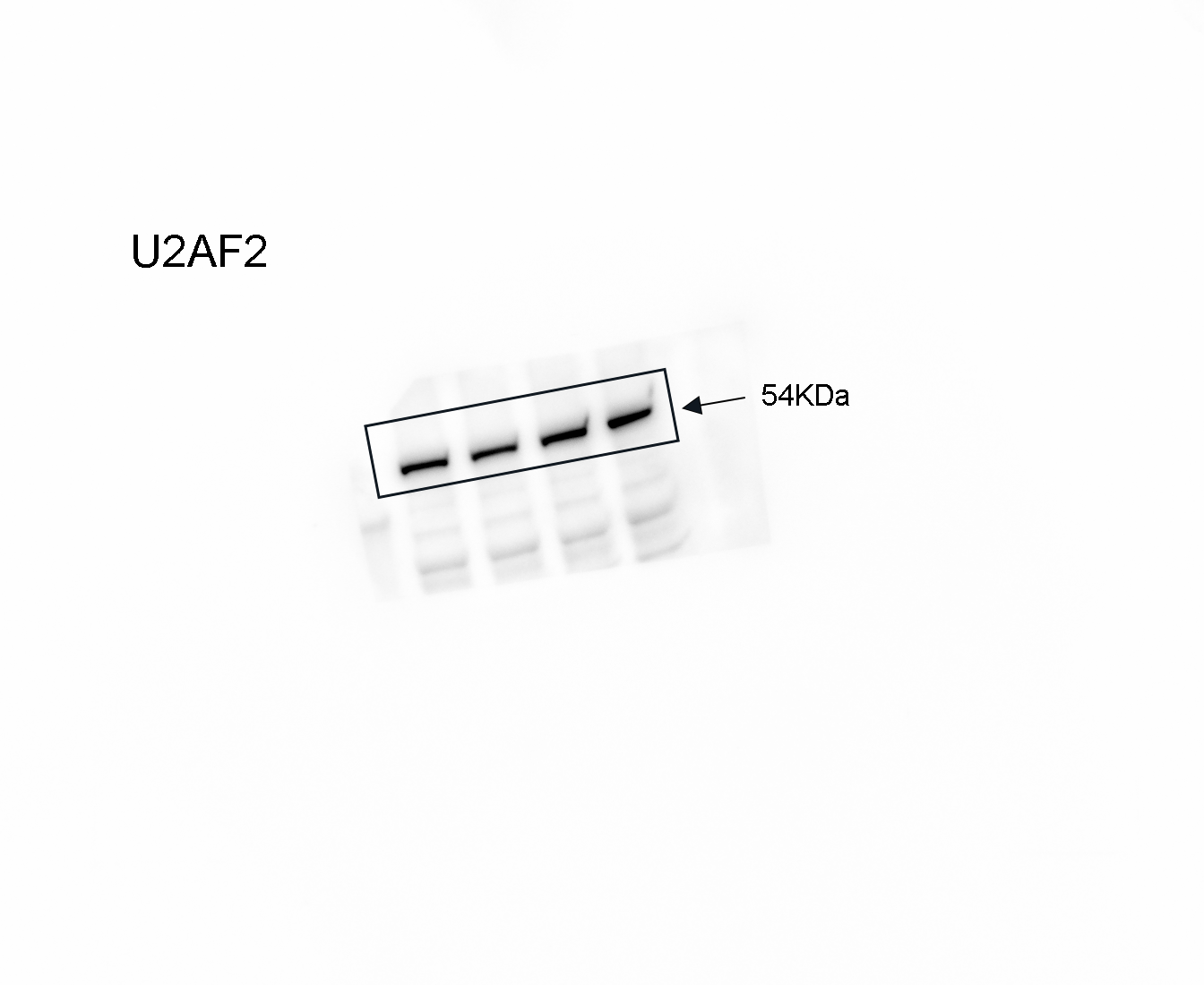

Supplement: Figure 4—source data 2. [file elife-98524-fig4-data2.zip › Fig 4-data2-v1/4Q/upper left/U2AF2 .tif]
