# Supplementary figures and images for: Nuclear translocation of SIRT4 mediates deacetylation of U2AF2 to modulate renal fibrosis through alternative splicing-mediated upregulation of CCN2 (part 6 of 9)
Source: eLife. 2024 Nov 4;13:RP98524. doi: 10.7554/eLife.98524 (PMC11534337; doi:10.7554/eLife.98524)

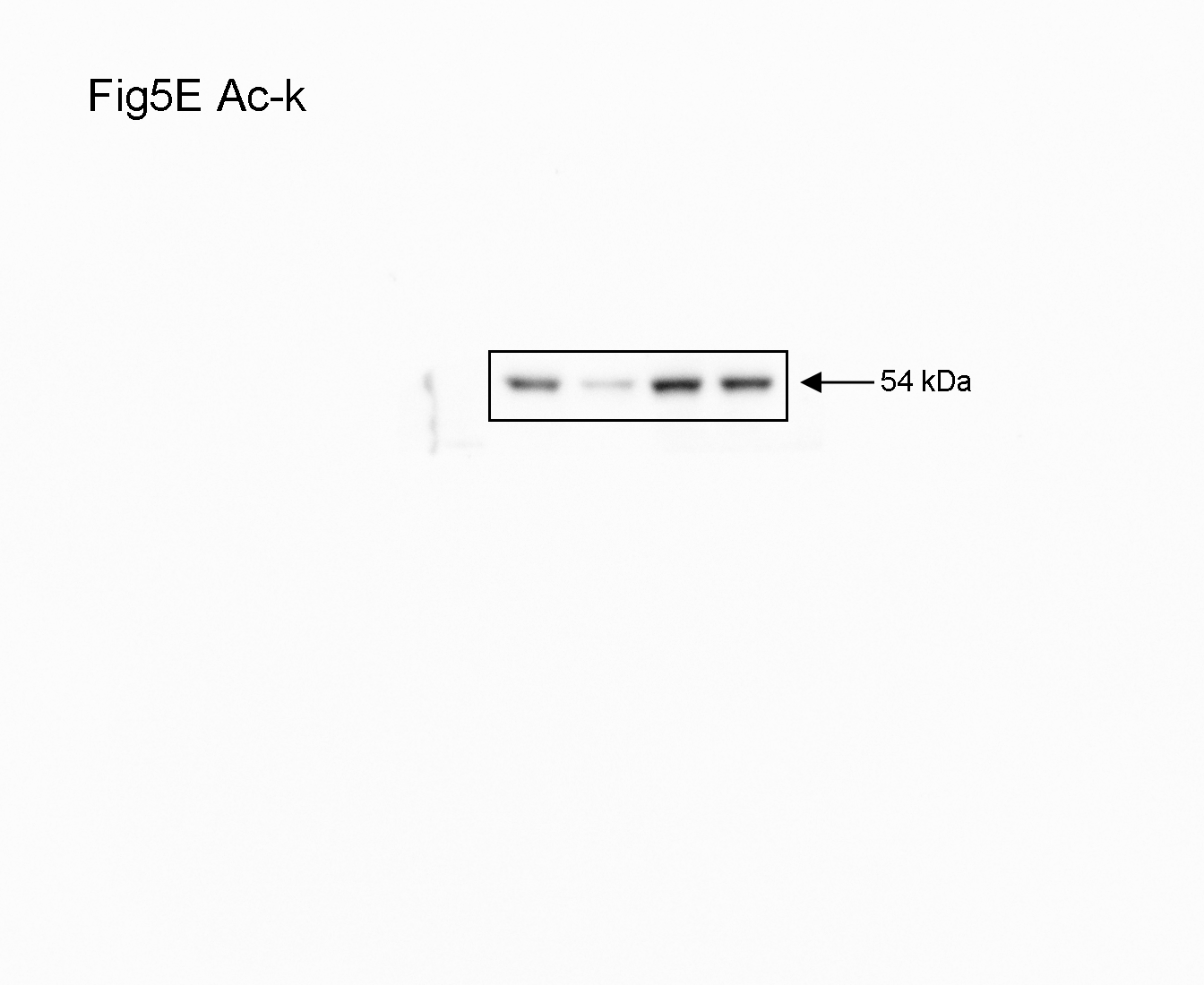

Supplement: Figure 5—source data 2. [file elife-98524-fig5-data2.zip › Fig 5-data2-v1/5E/left/Ac-K.tif]

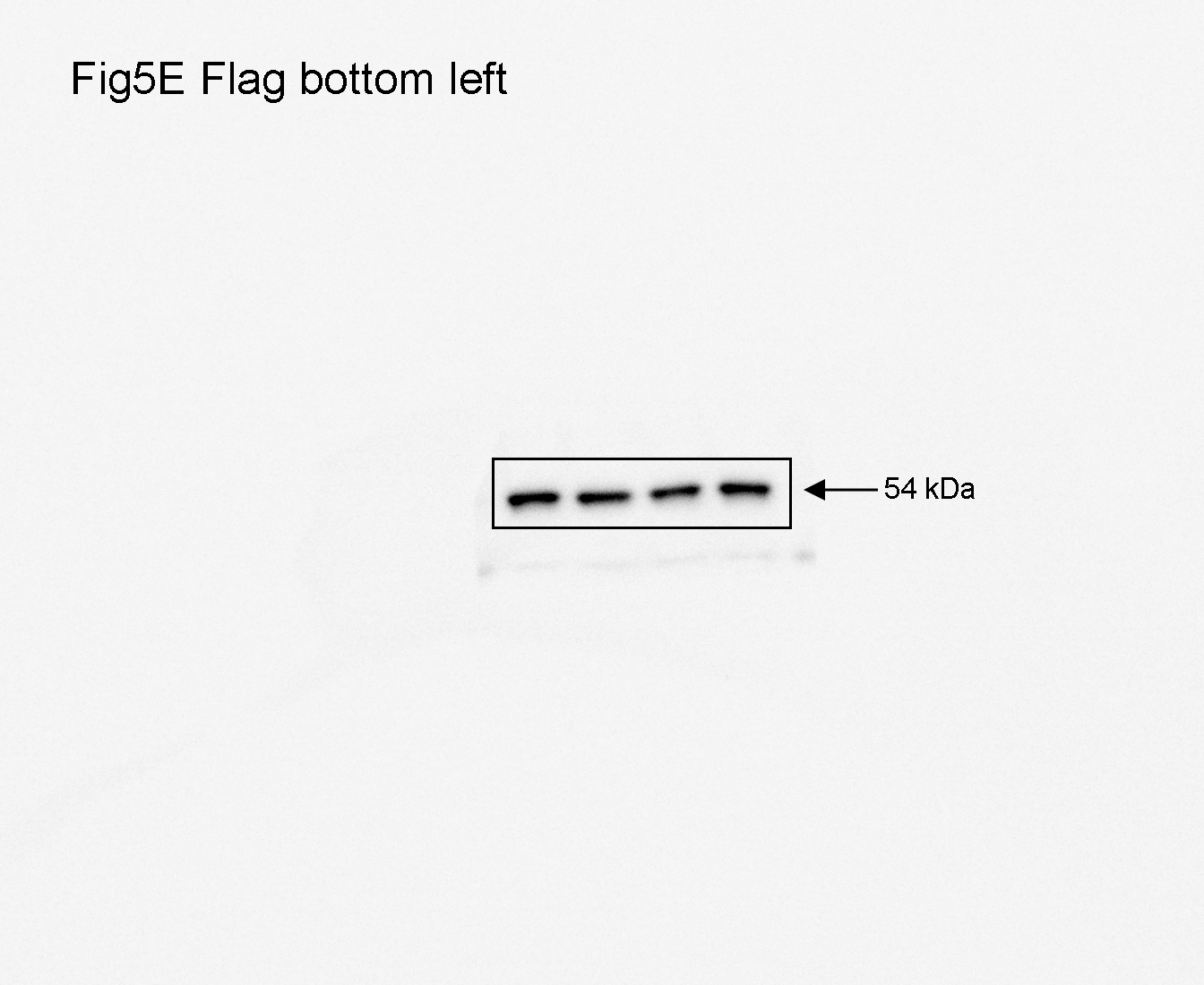

Supplement: Figure 5—source data 2. [file elife-98524-fig5-data2.zip › Fig 5-data2-v1/5E/left/Flag bottom left.tif]

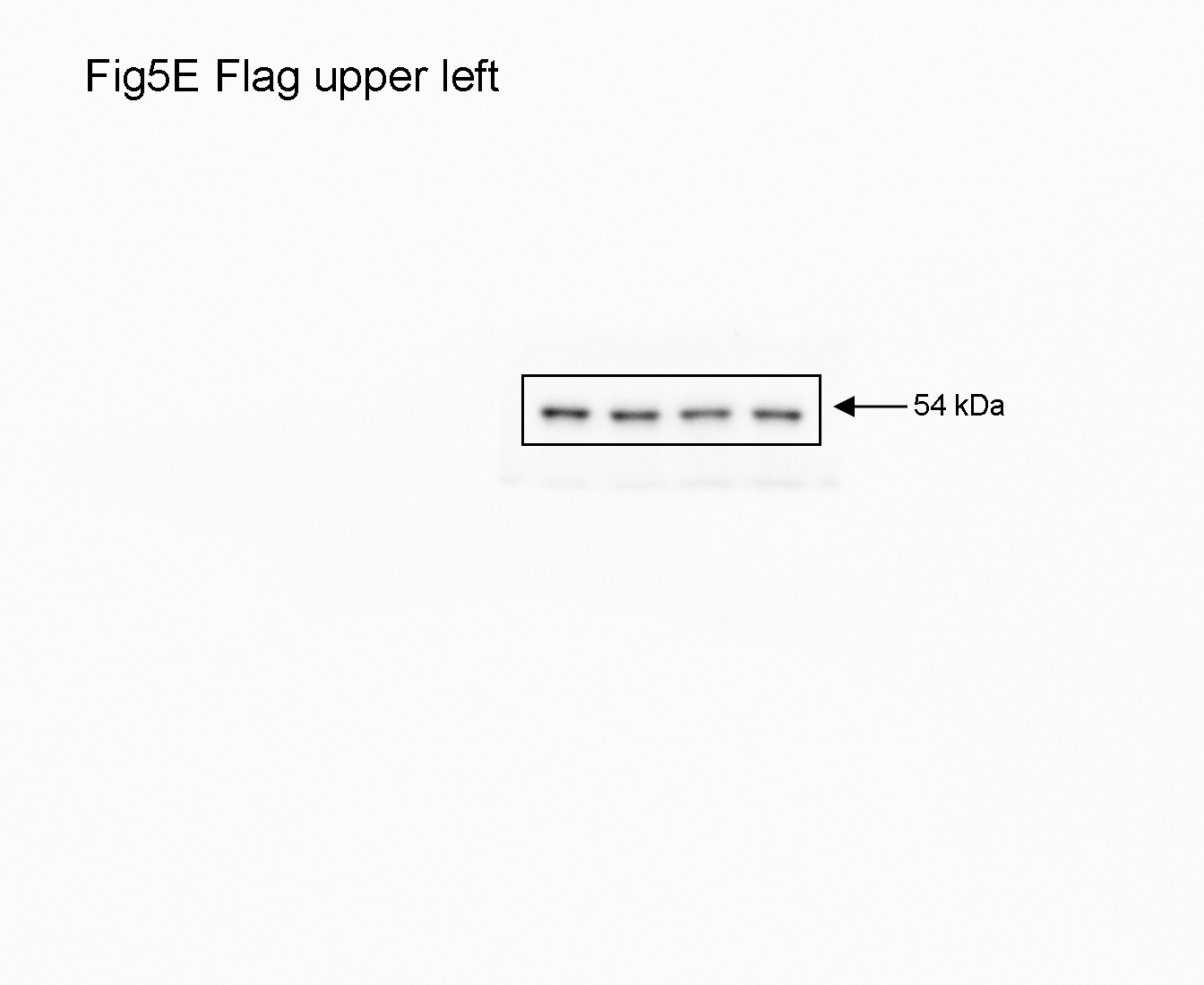

Supplement: Figure 5—source data 2. [file elife-98524-fig5-data2.zip › Fig 5-data2-v1/5E/left/Flag upper left.tif]

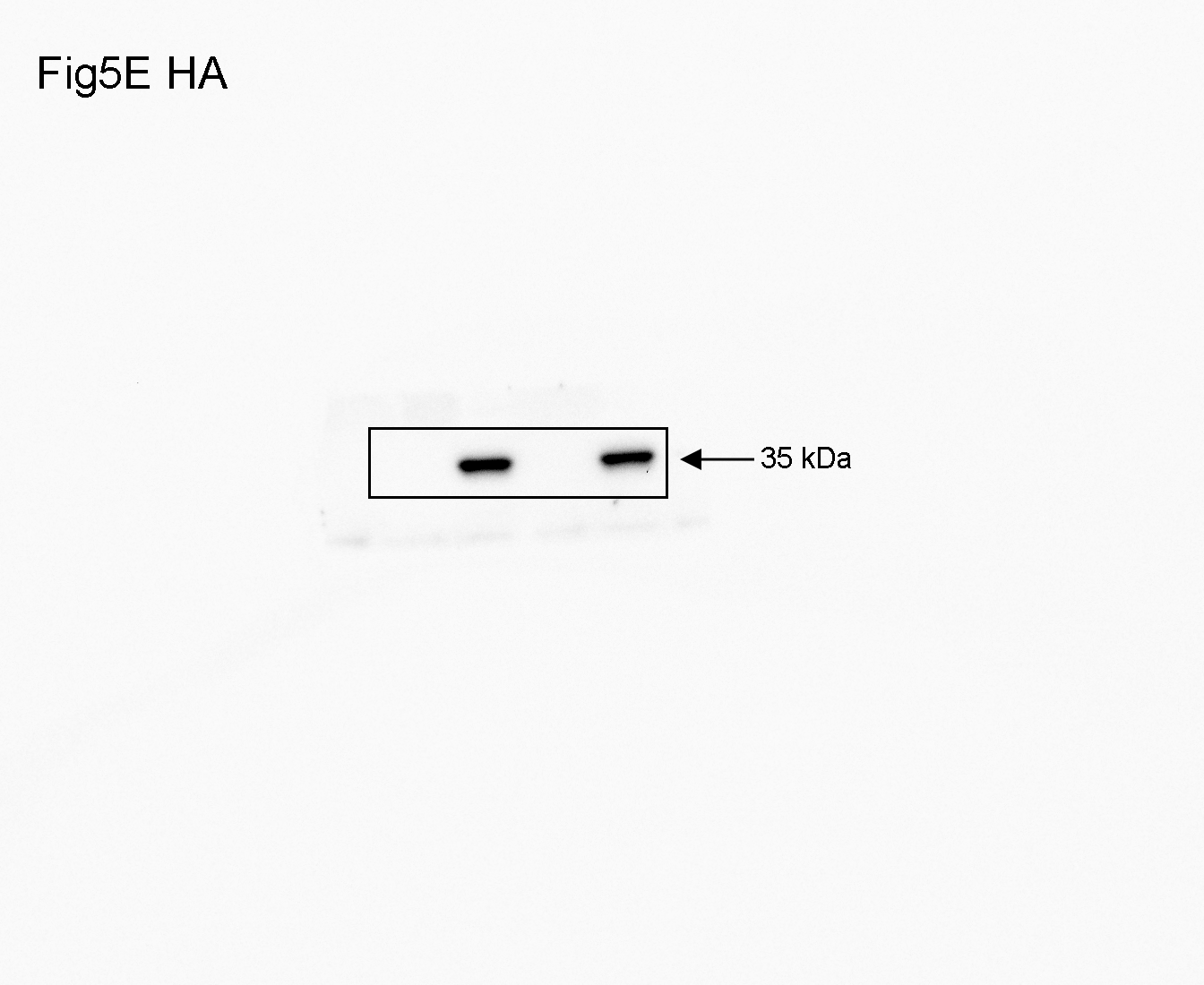

Supplement: Figure 5—source data 2. [file elife-98524-fig5-data2.zip › Fig 5-data2-v1/5E/left/HA.tif]

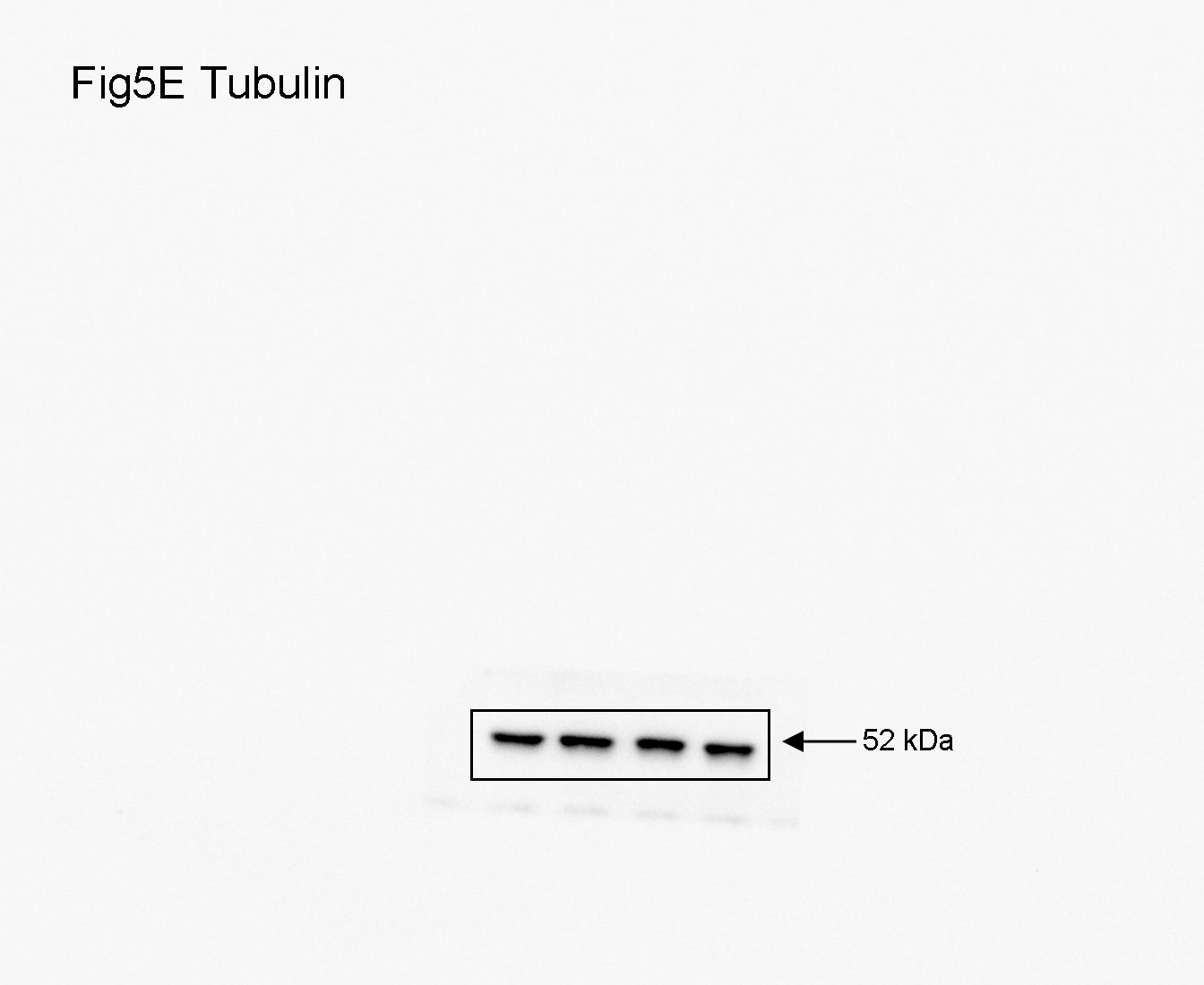

Supplement: Figure 5—source data 2. [file elife-98524-fig5-data2.zip › Fig 5-data2-v1/5E/left/Tubulin.tif]

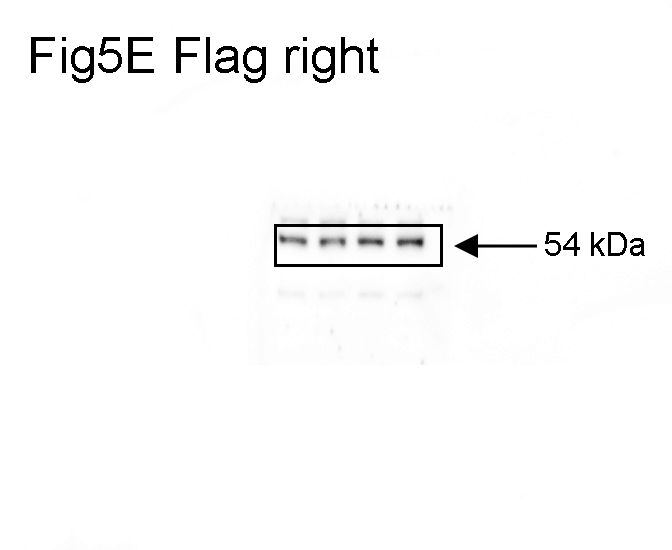

Supplement: Figure 5—source data 2. [file elife-98524-fig5-data2.zip › Fig 5-data2-v1/5E/right/Flag right.tif]

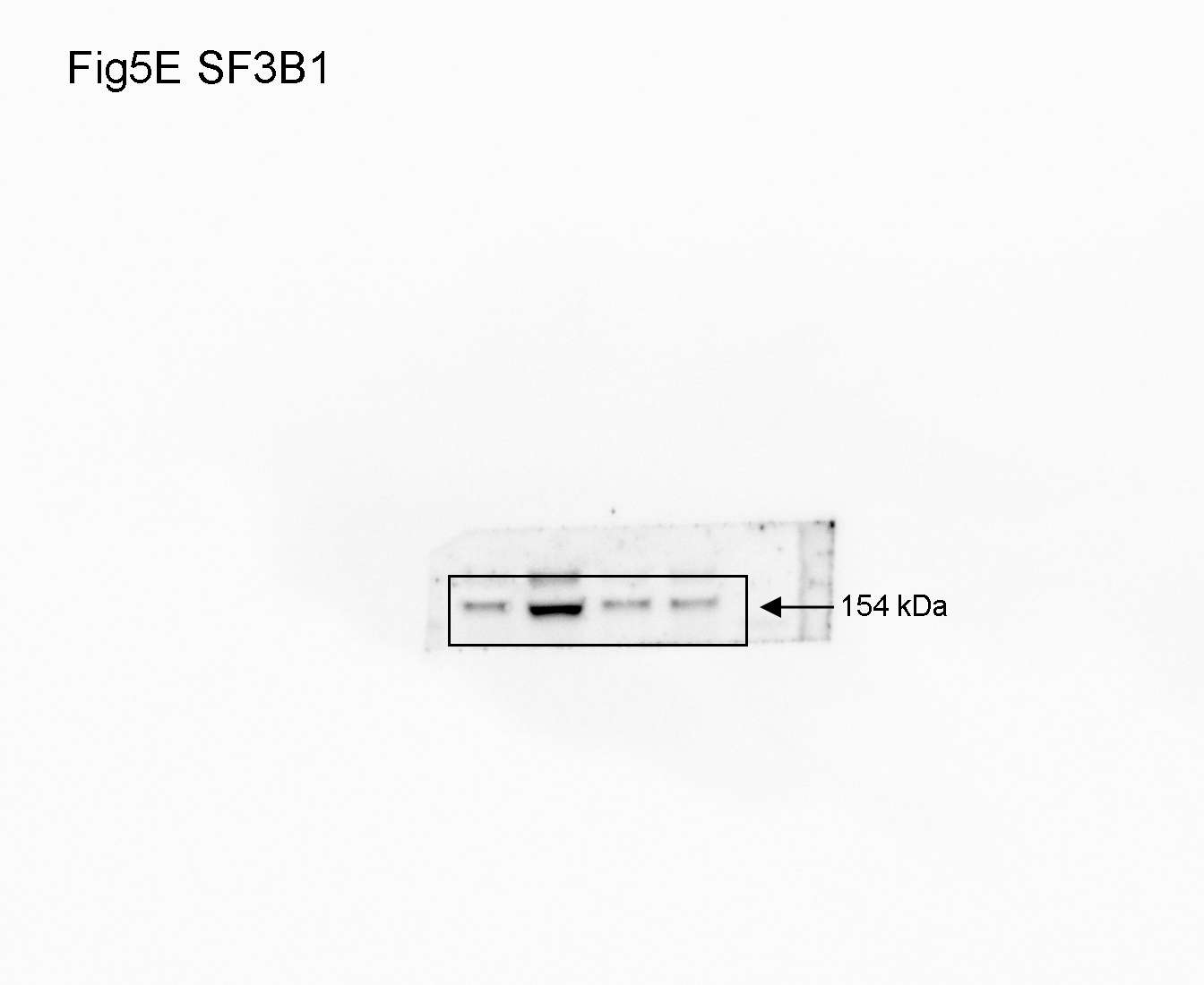

Supplement: Figure 5—source data 2. [file elife-98524-fig5-data2.zip › Fig 5-data2-v1/5E/right/SF3B1.tif]

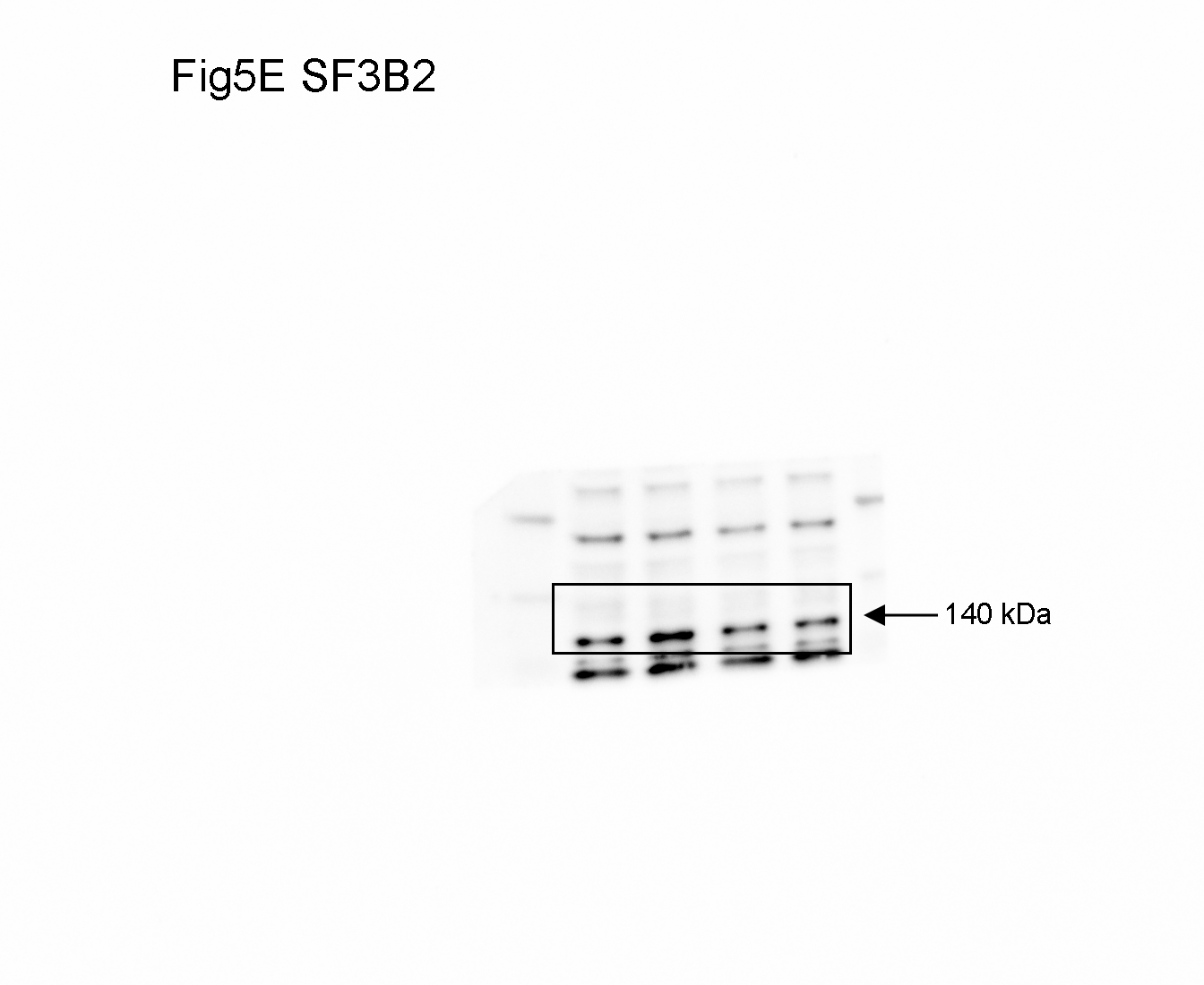

Supplement: Figure 5—source data 2. [file elife-98524-fig5-data2.zip › Fig 5-data2-v1/5E/right/SF3B2.tif]

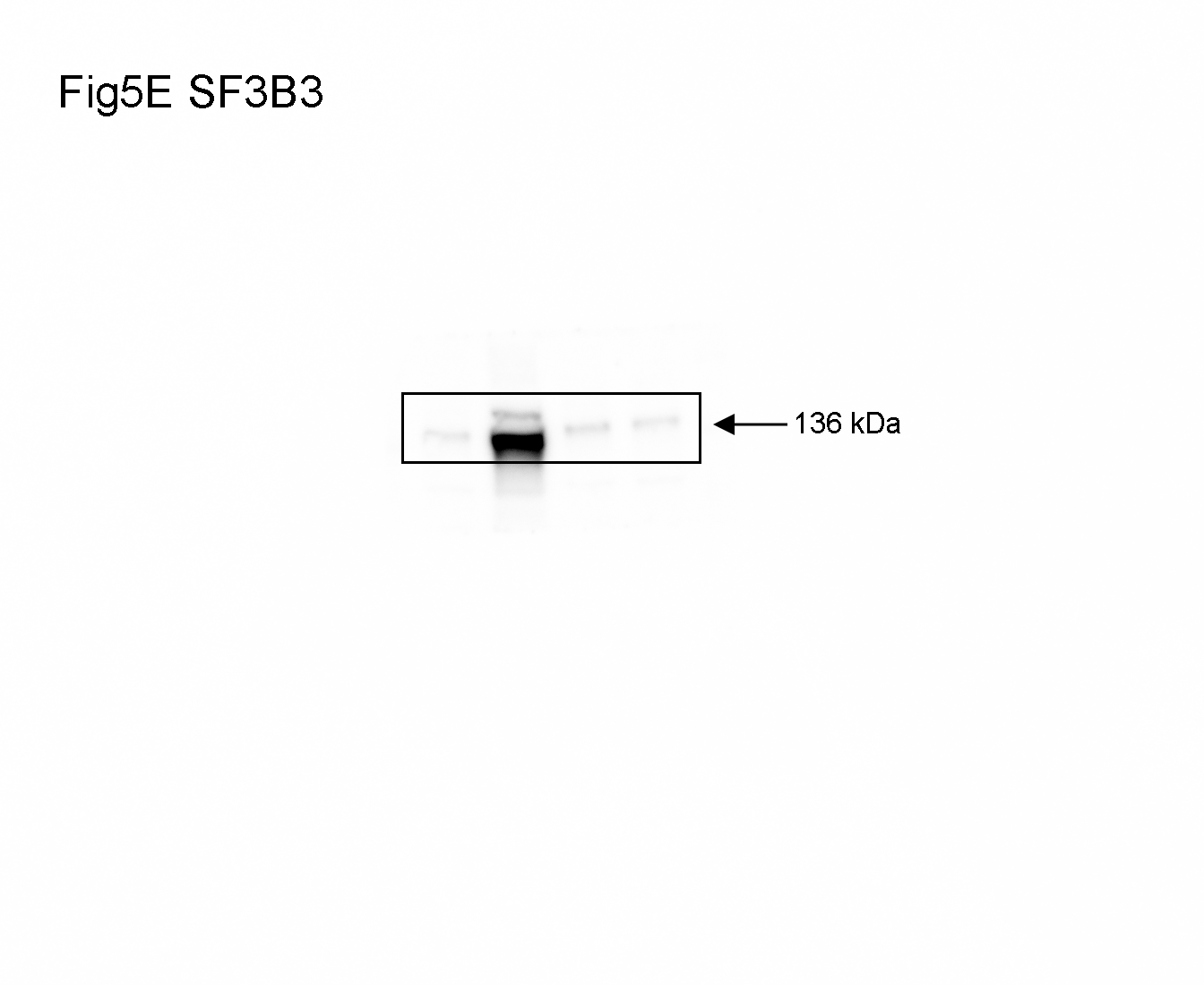

Supplement: Figure 5—source data 2. [file elife-98524-fig5-data2.zip › Fig 5-data2-v1/5E/right/SF3B3.tif]

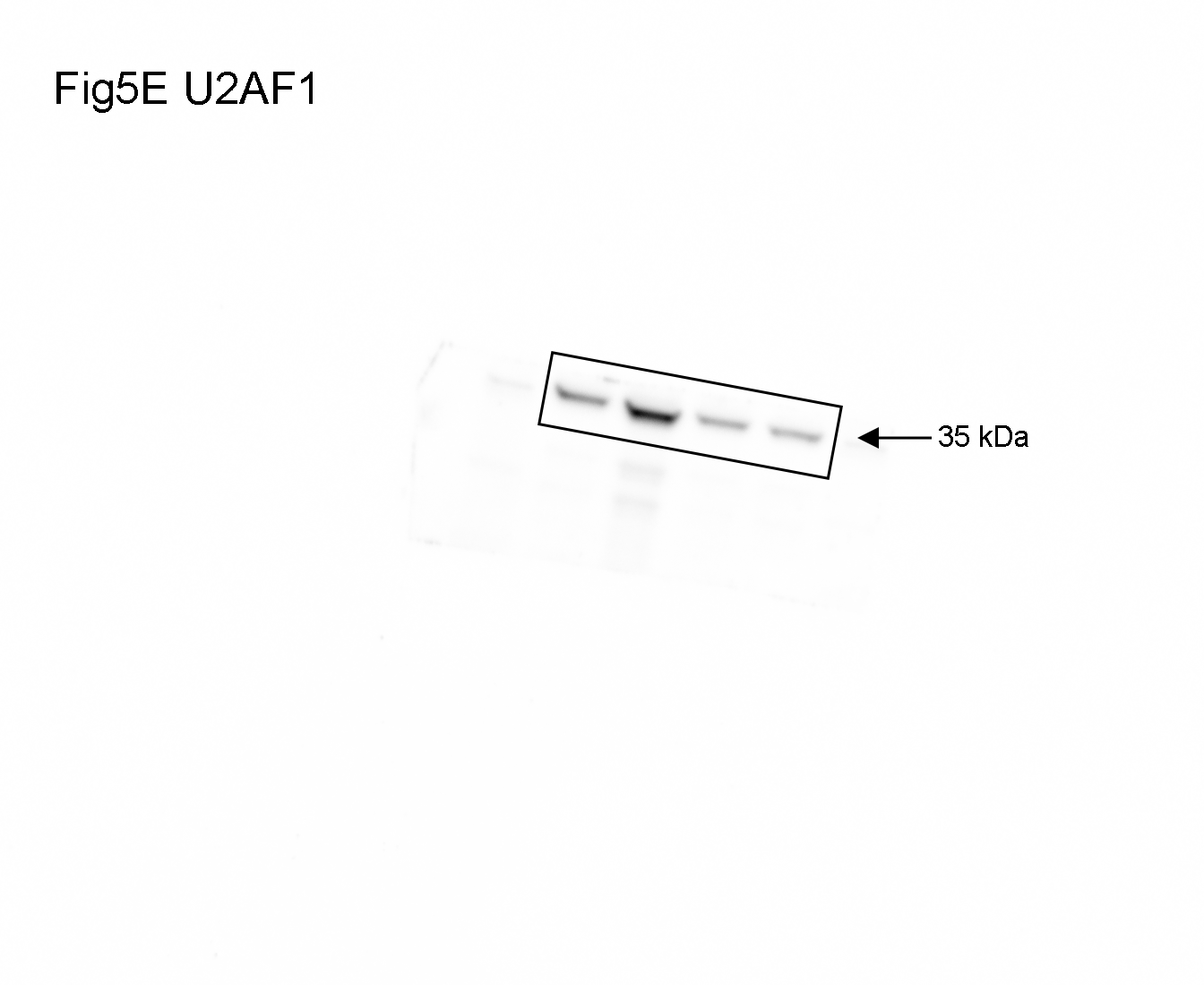

Supplement: Figure 5—source data 2. [file elife-98524-fig5-data2.zip › Fig 5-data2-v1/5E/right/U2AF1.tif]

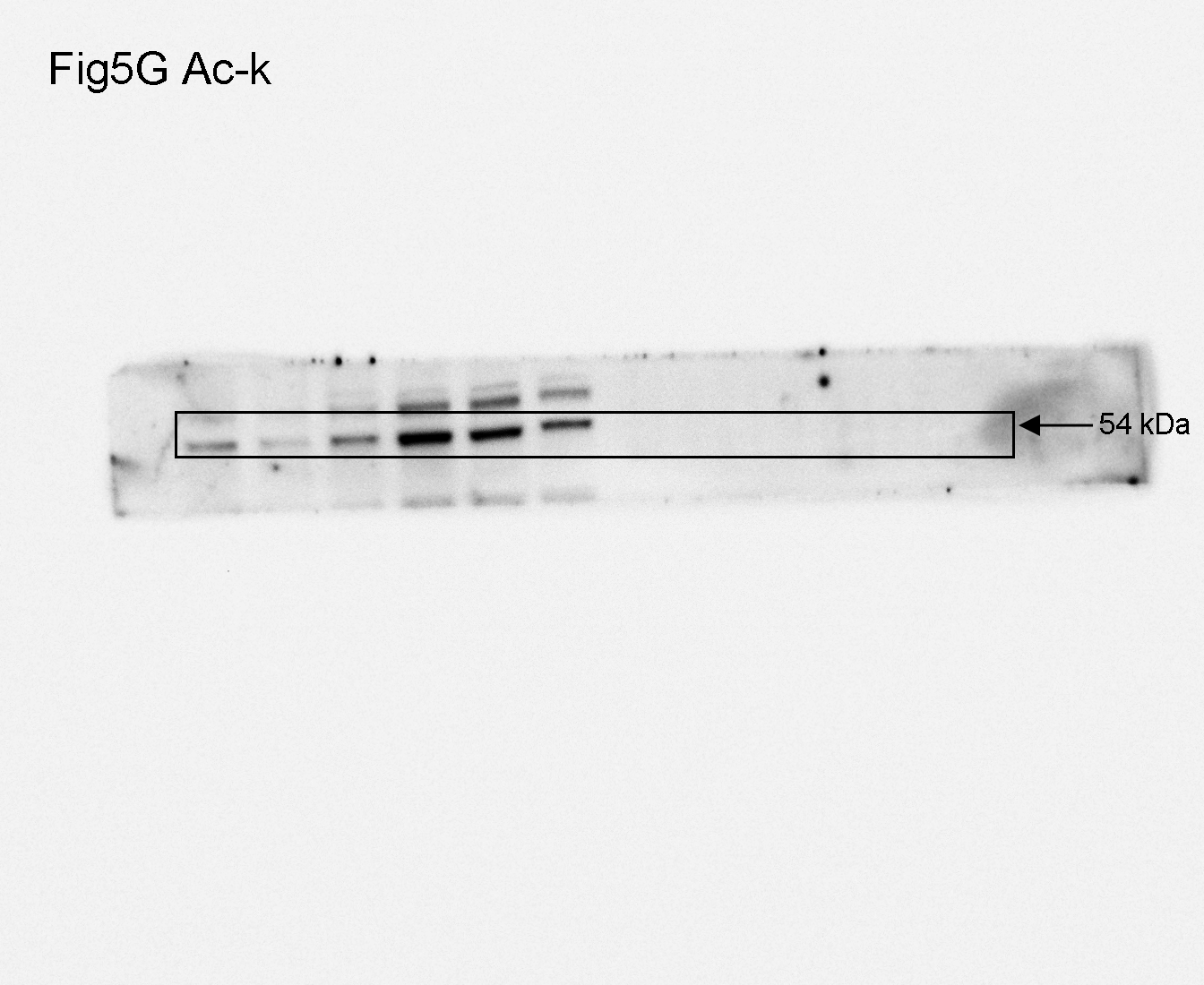

Supplement: Figure 5—source data 2. [file elife-98524-fig5-data2.zip › Fig 5-data2-v1/5G/Ac-k.tif]

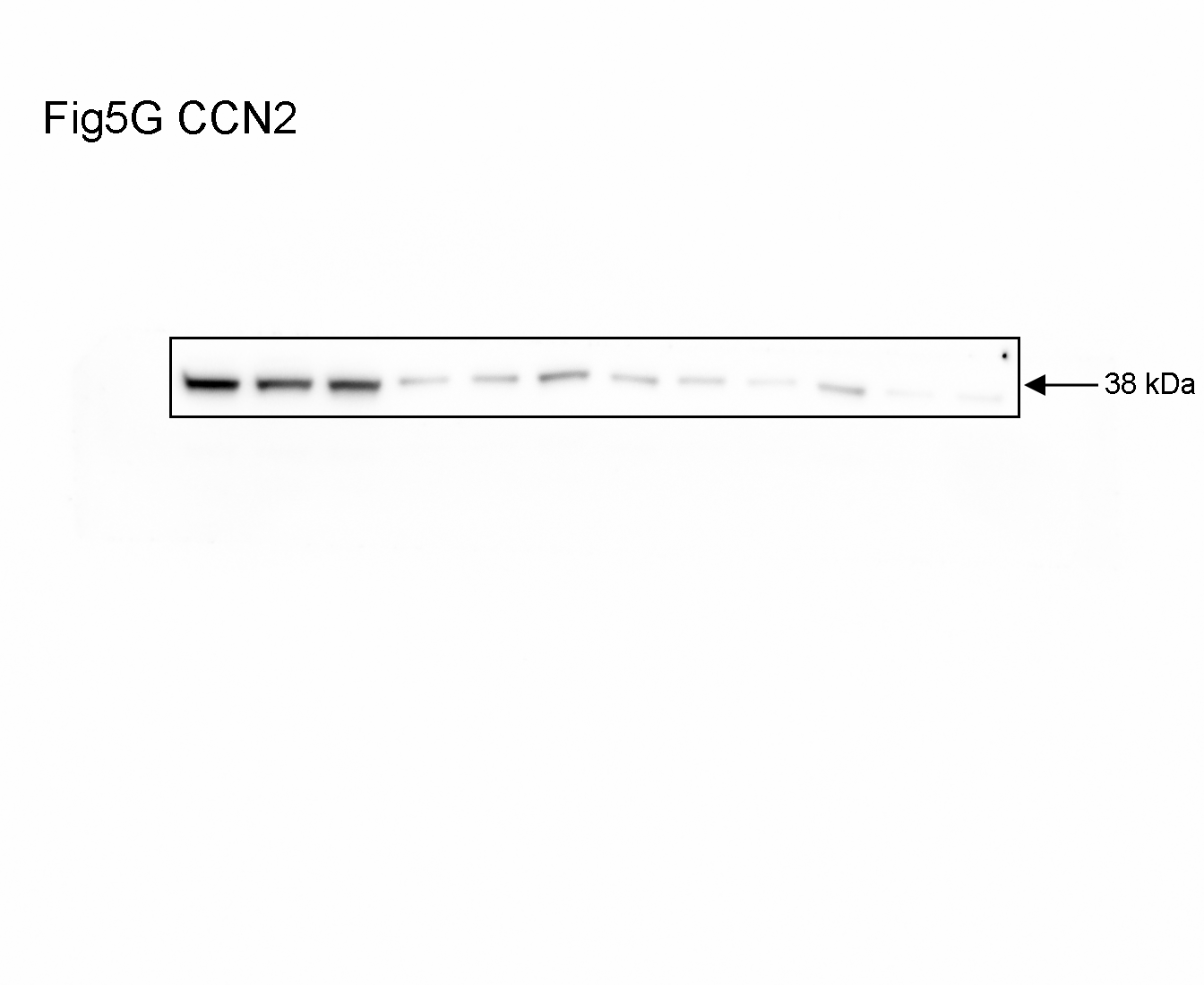

Supplement: Figure 5—source data 2. [file elife-98524-fig5-data2.zip › Fig 5-data2-v1/5G/CCN2.tif]

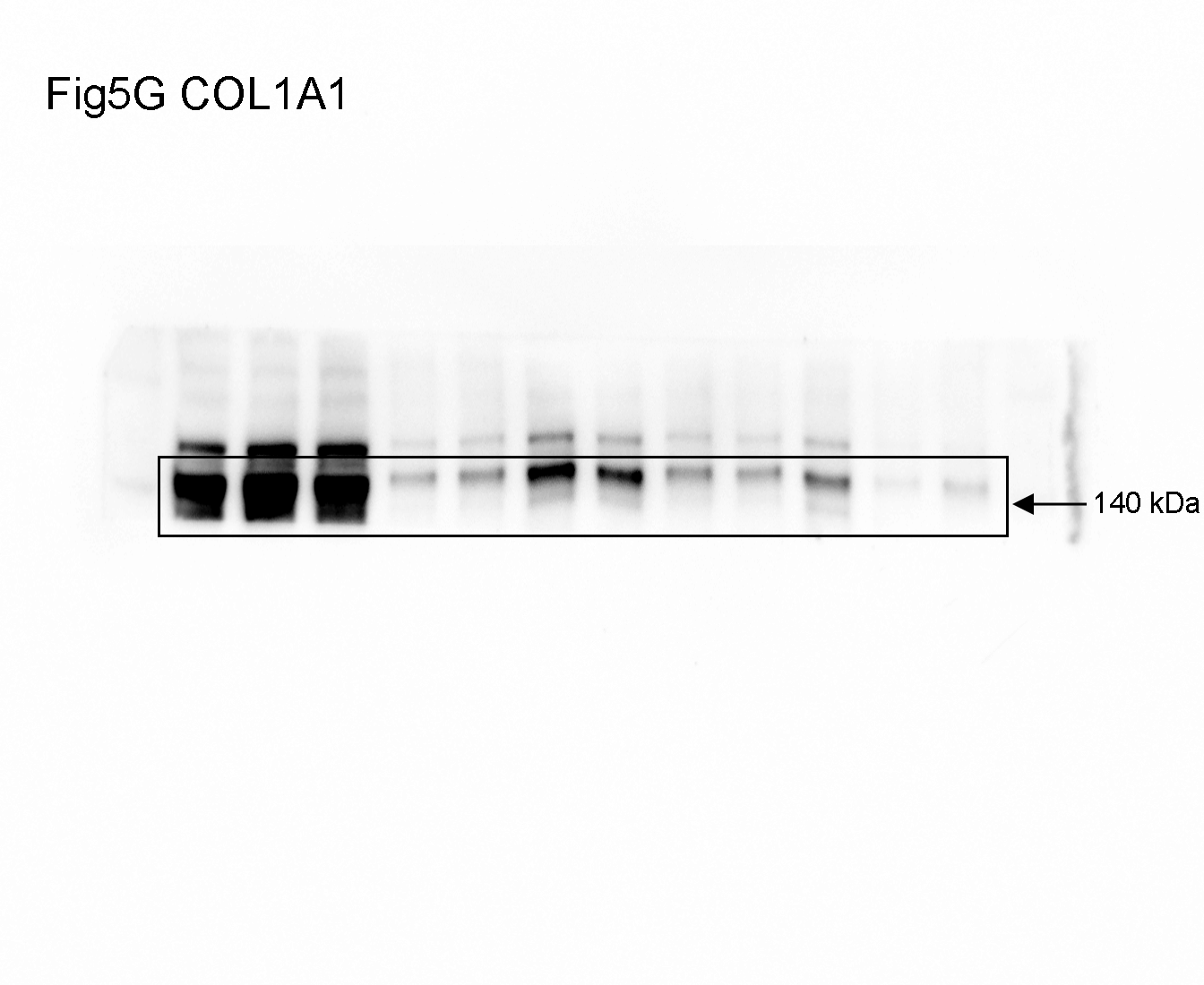

Supplement: Figure 5—source data 2. [file elife-98524-fig5-data2.zip › Fig 5-data2-v1/5G/COL1A1.tif]

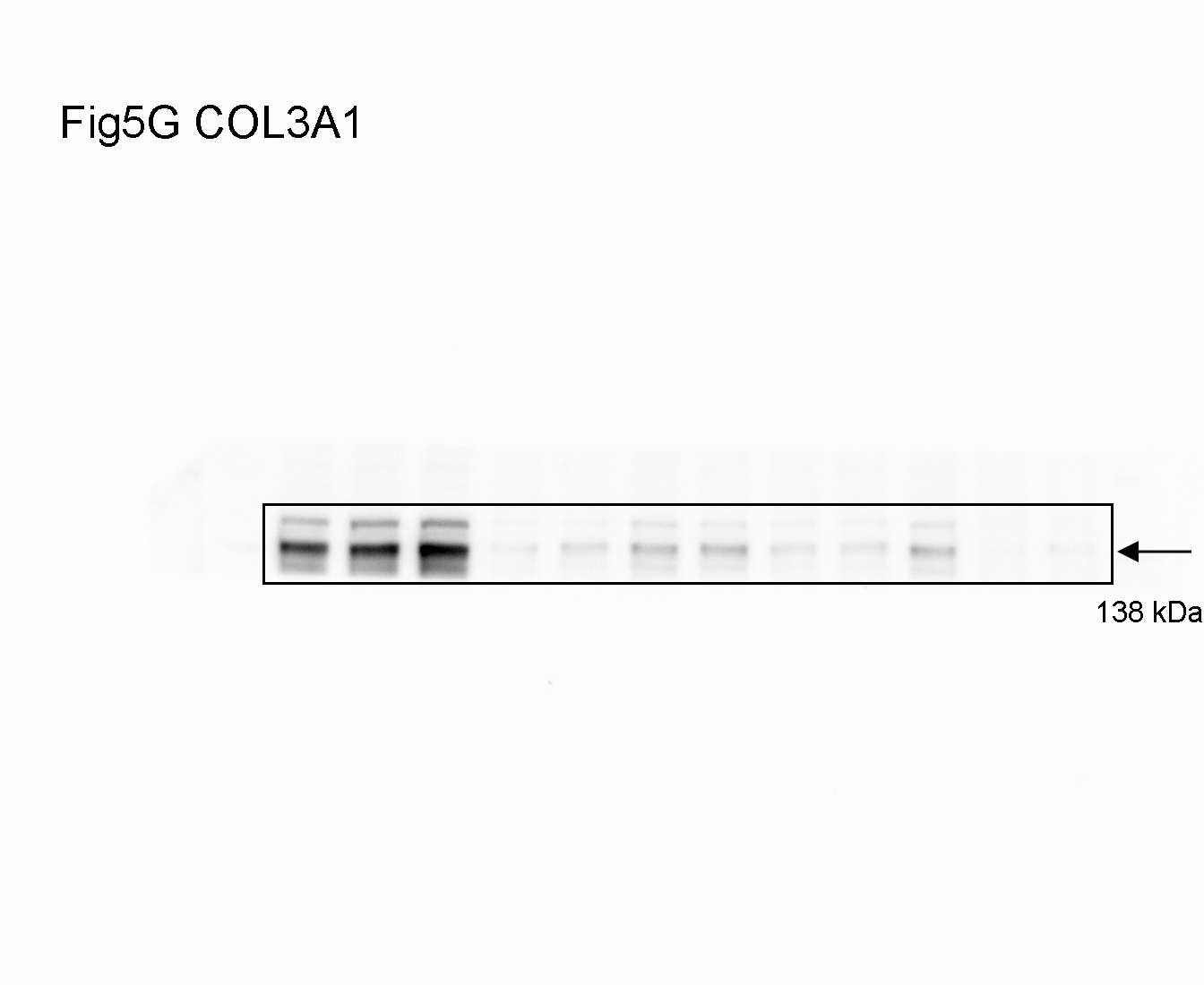

Supplement: Figure 5—source data 2. [file elife-98524-fig5-data2.zip › Fig 5-data2-v1/5G/COL3A1.tif]

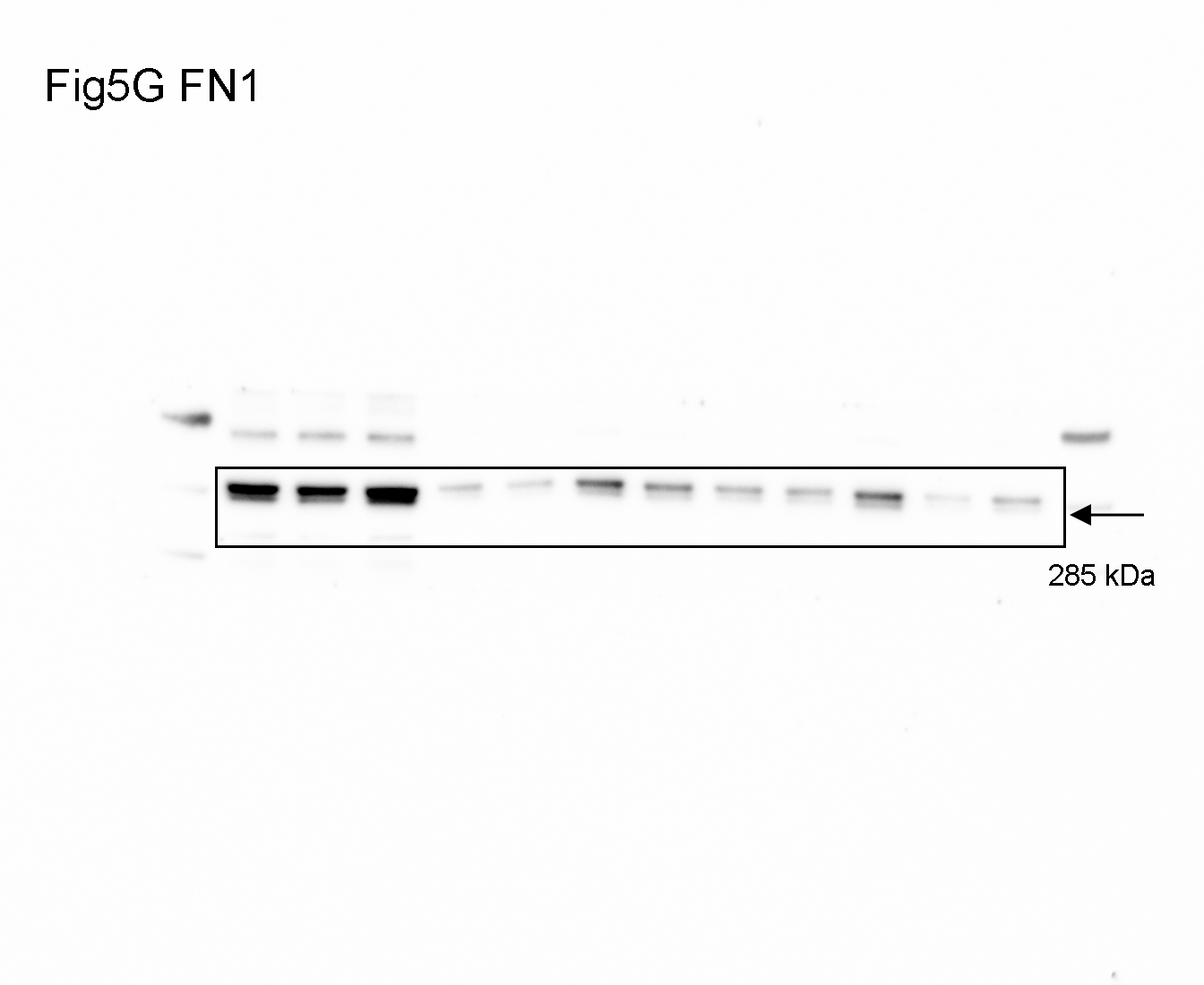

Supplement: Figure 5—source data 2. [file elife-98524-fig5-data2.zip › Fig 5-data2-v1/5G/FN1.tif]

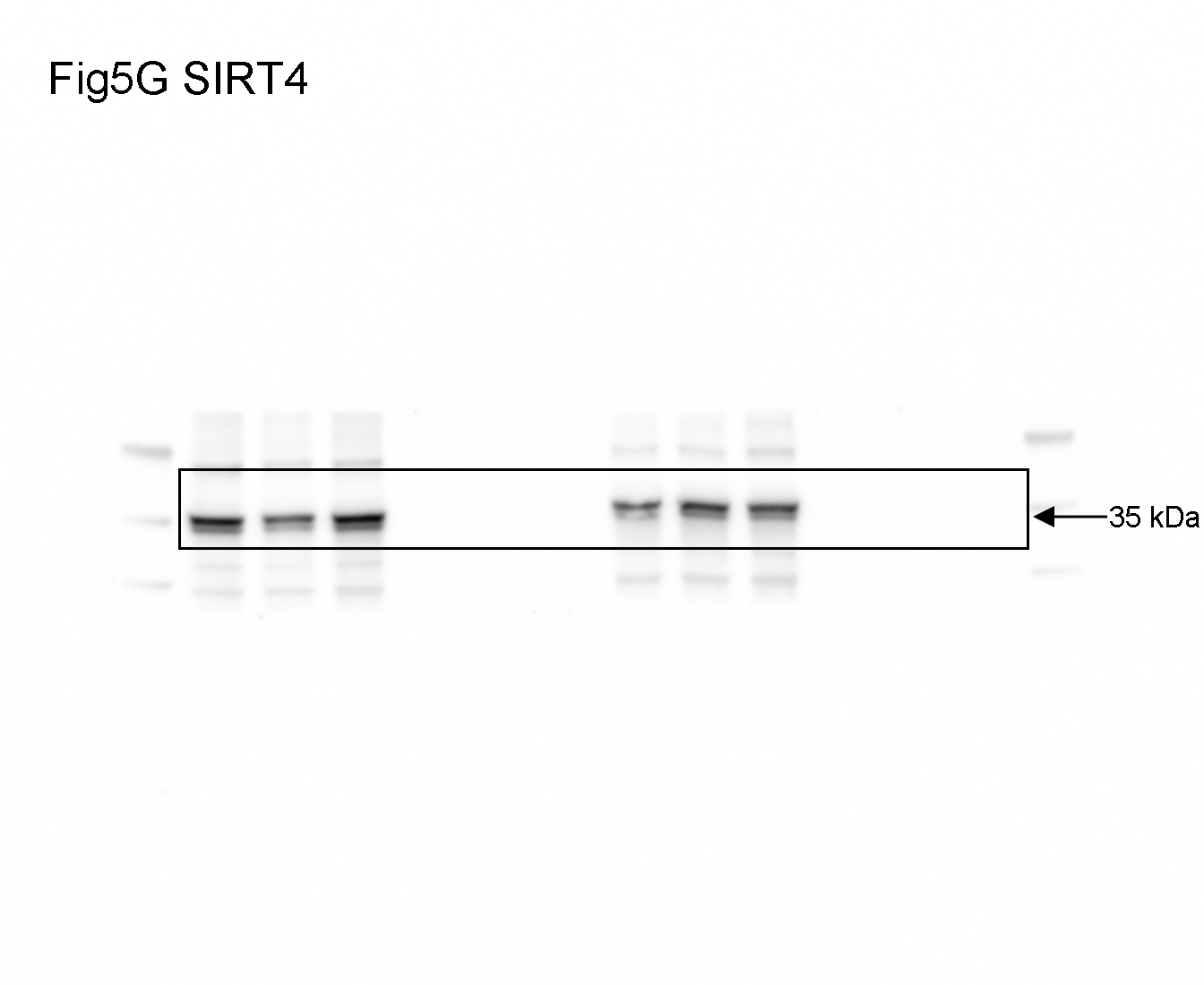

Supplement: Figure 5—source data 2. [file elife-98524-fig5-data2.zip › Fig 5-data2-v1/5G/SIRT4.tif]

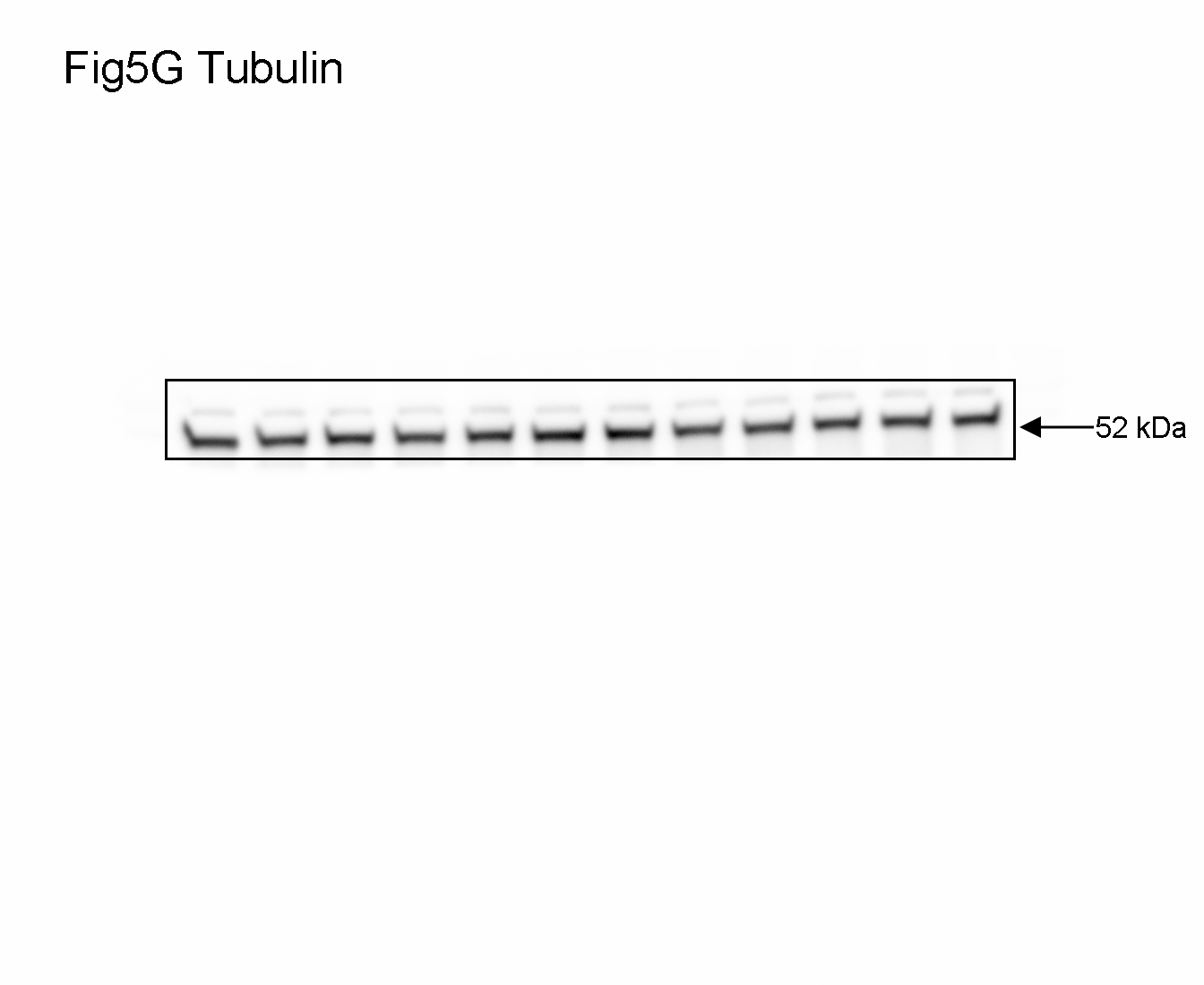

Supplement: Figure 5—source data 2. [file elife-98524-fig5-data2.zip › Fig 5-data2-v1/5G/Tubulin.tif]

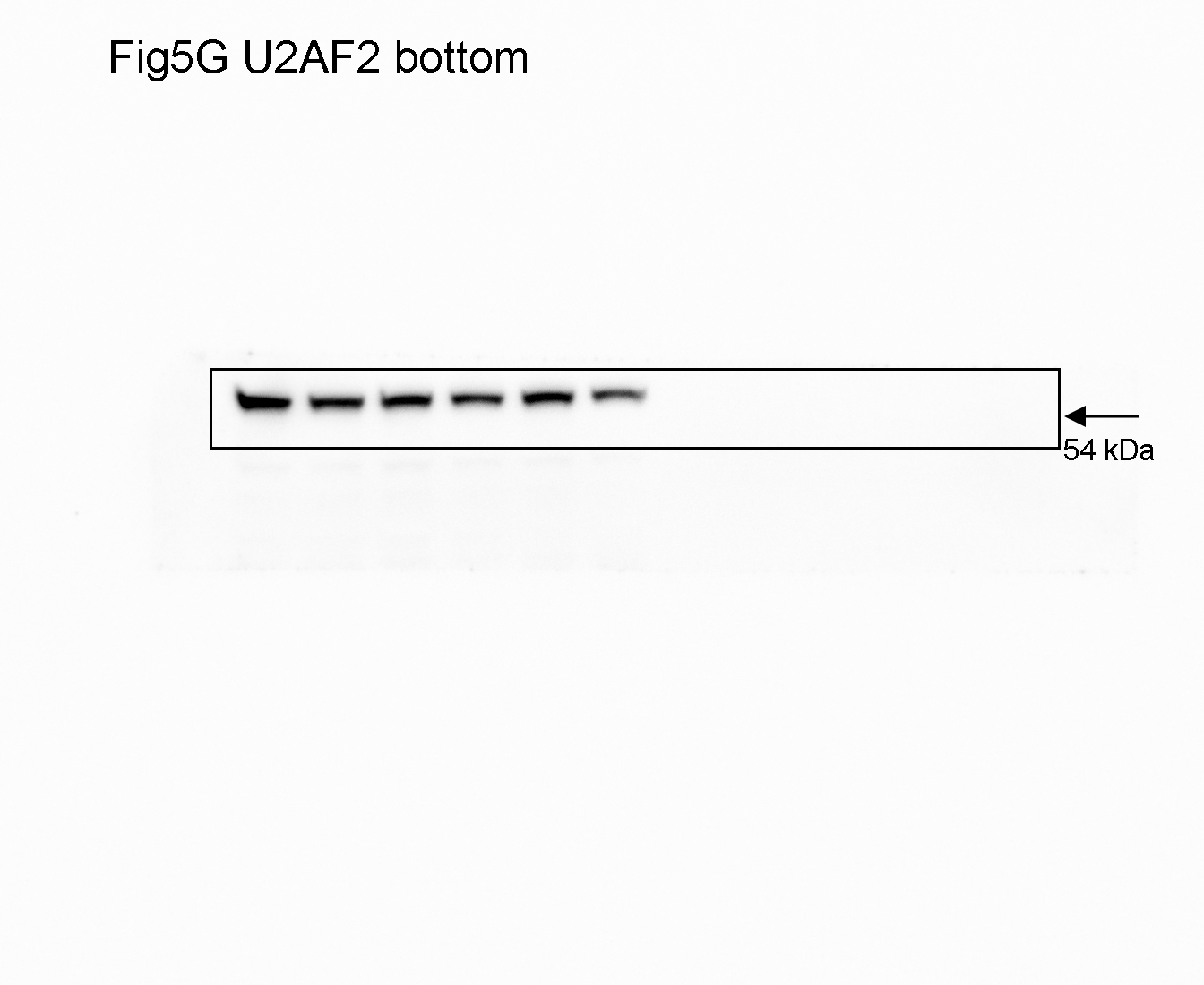

Supplement: Figure 5—source data 2. [file elife-98524-fig5-data2.zip › Fig 5-data2-v1/5G/U2AF2 bottom.tif]

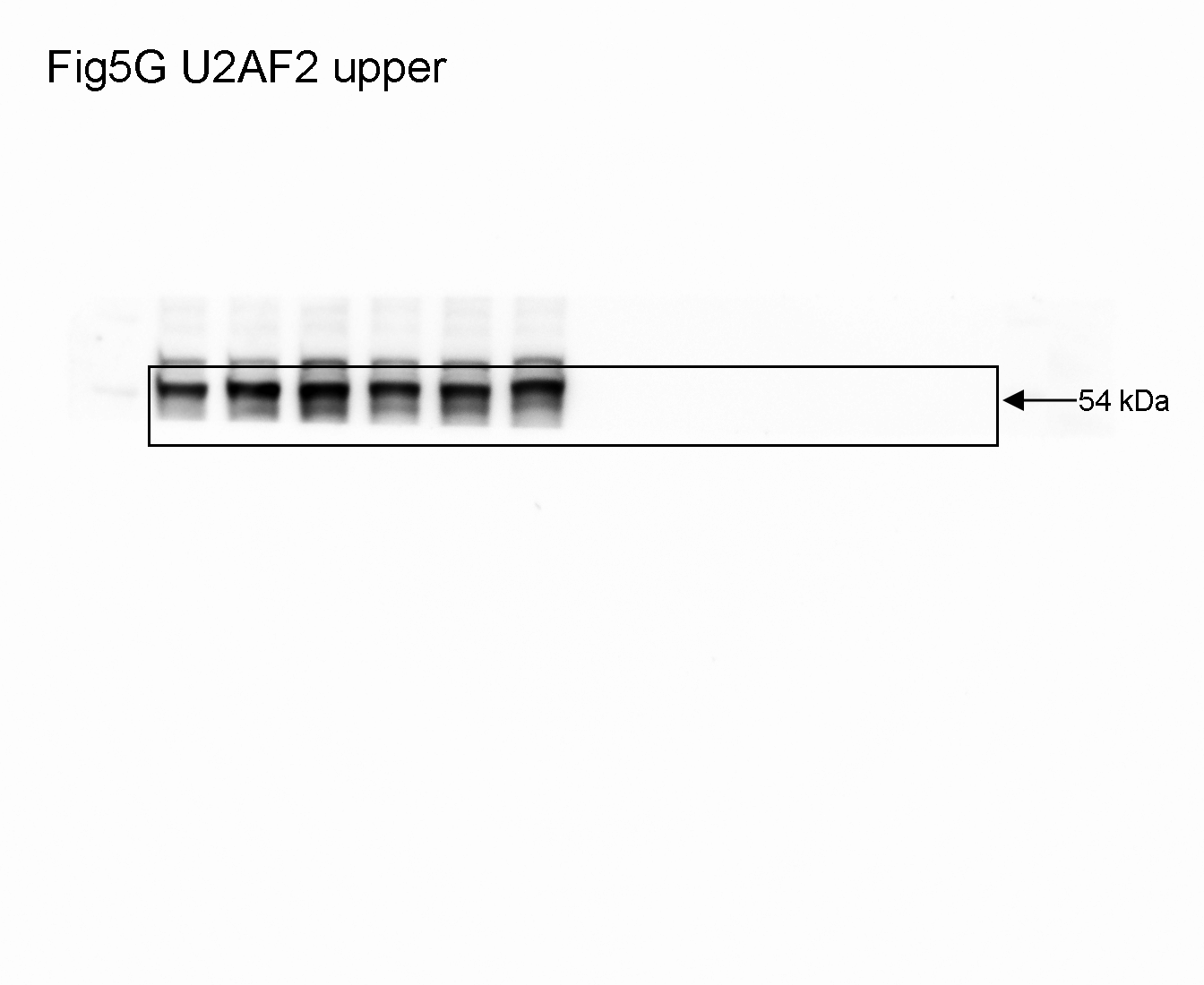

Supplement: Figure 5—source data 2. [file elife-98524-fig5-data2.zip › Fig 5-data2-v1/5G/U2AF2 upper.tif]

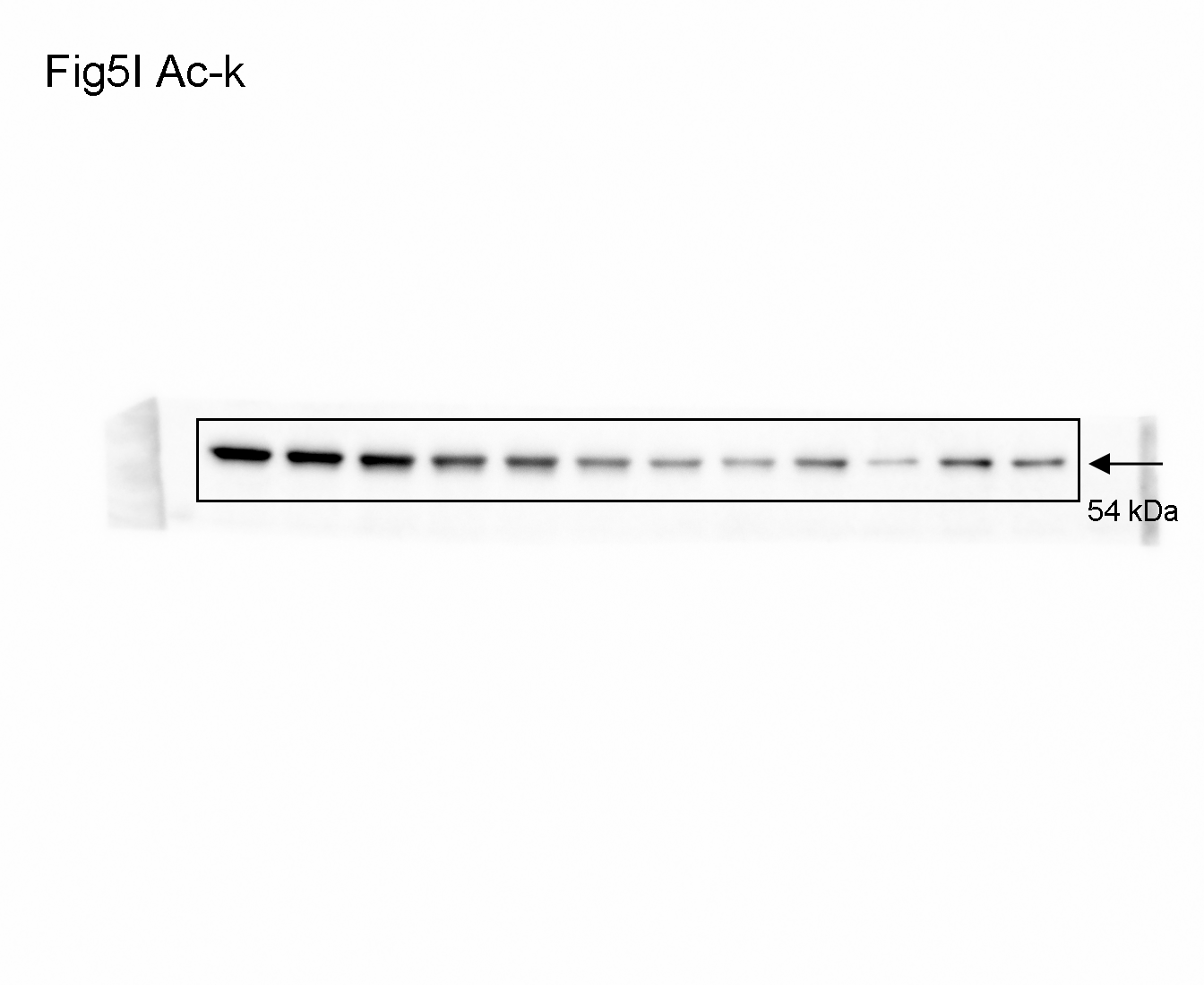

Supplement: Figure 5—source data 2. [file elife-98524-fig5-data2.zip › Fig 5-data2-v1/5I/Ac-k.tif]

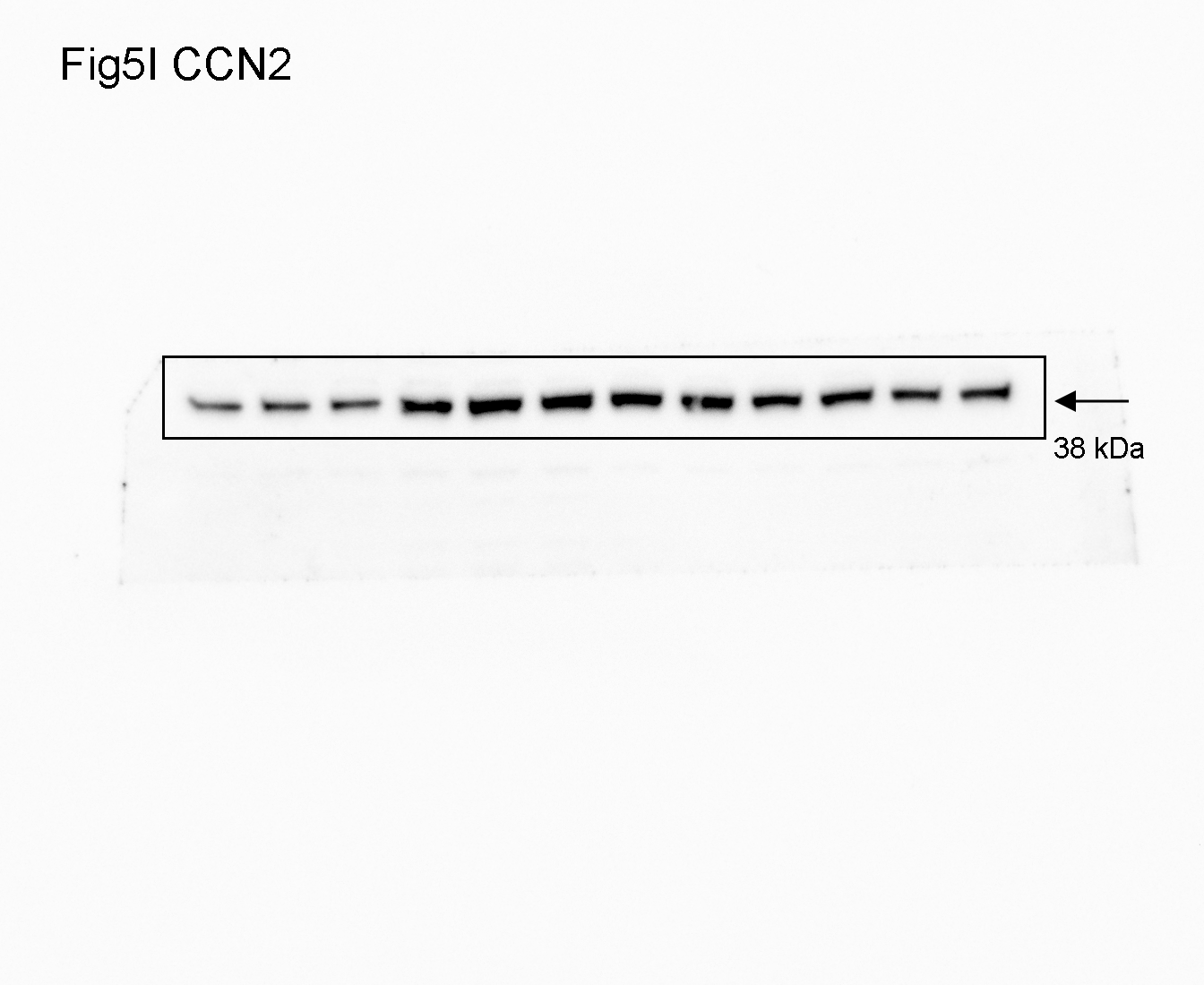

Supplement: Figure 5—source data 2. [file elife-98524-fig5-data2.zip › Fig 5-data2-v1/5I/CCN2.tif]

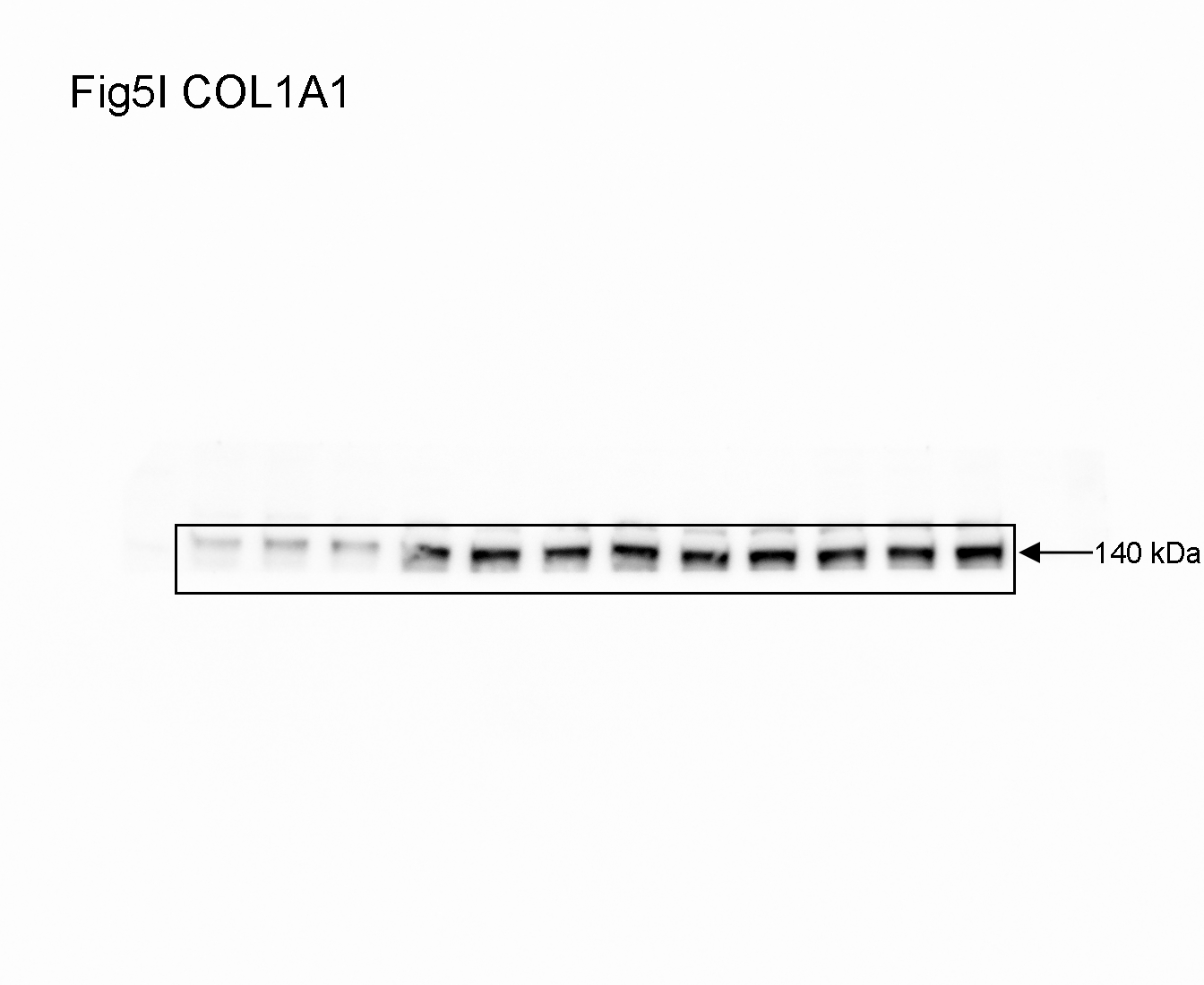

Supplement: Figure 5—source data 2. [file elife-98524-fig5-data2.zip › Fig 5-data2-v1/5I/COL1A1.tif]

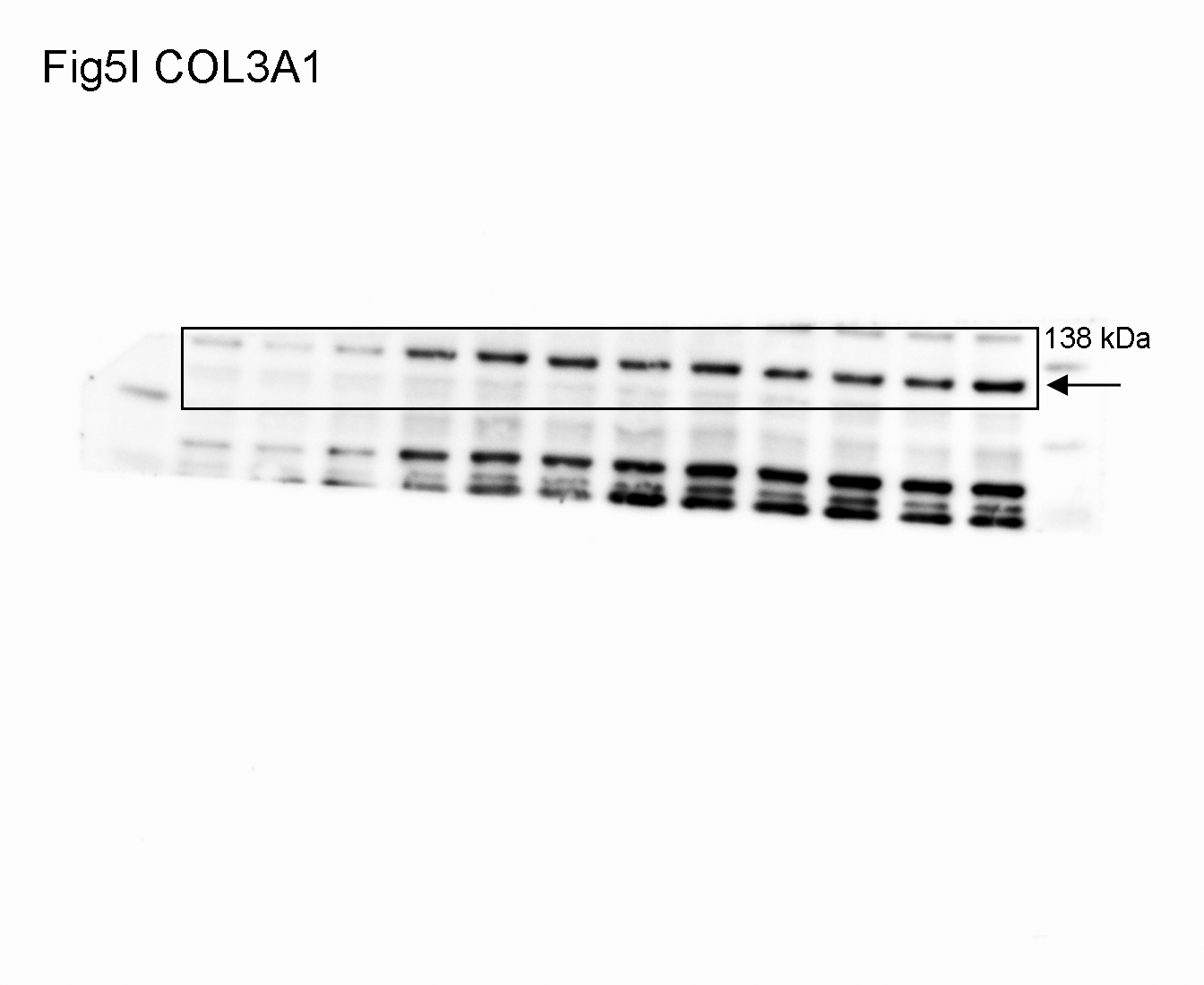

Supplement: Figure 5—source data 2. [file elife-98524-fig5-data2.zip › Fig 5-data2-v1/5I/COL3A1.tif]

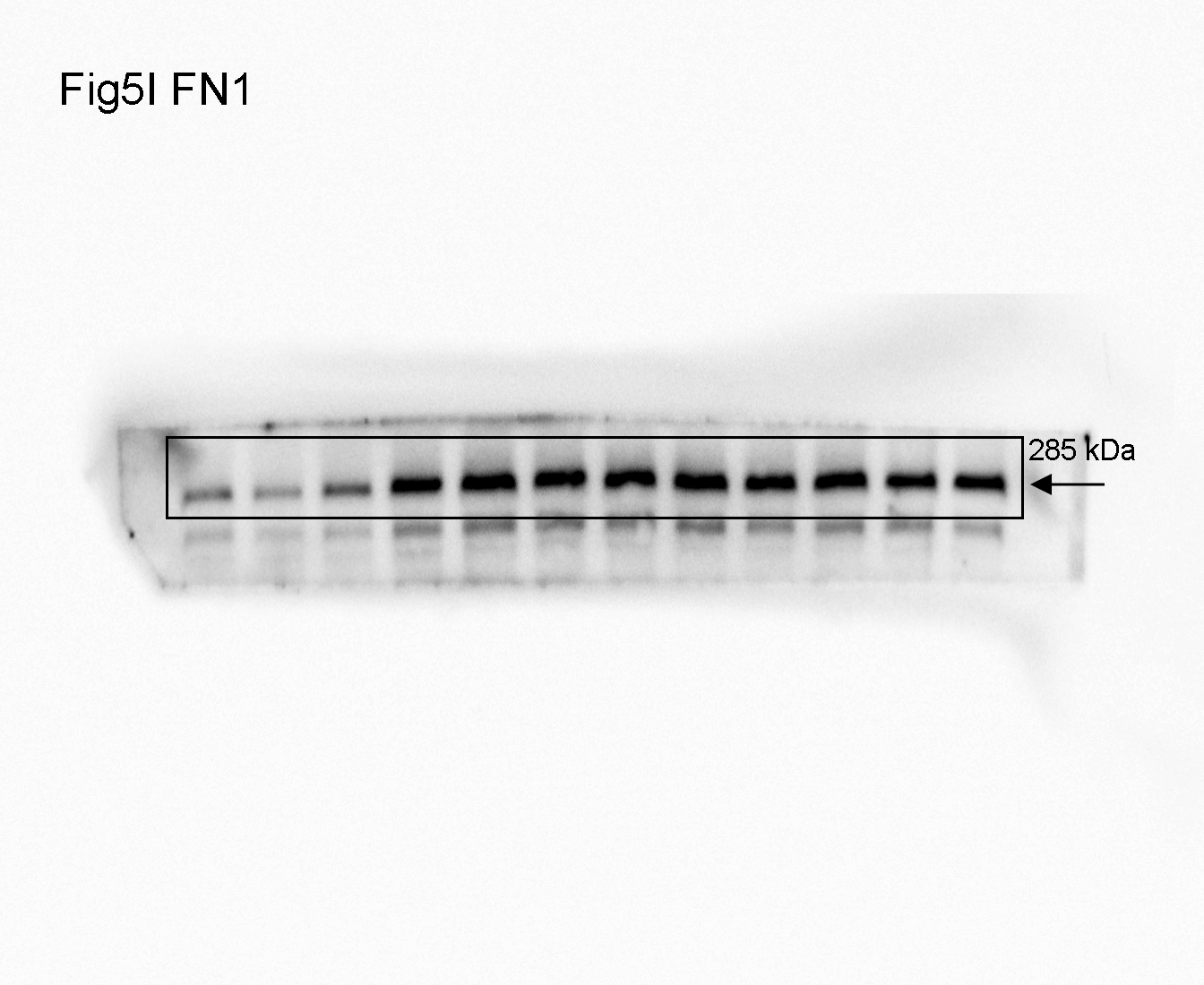

Supplement: Figure 5—source data 2. [file elife-98524-fig5-data2.zip › Fig 5-data2-v1/5I/FN1.tif]

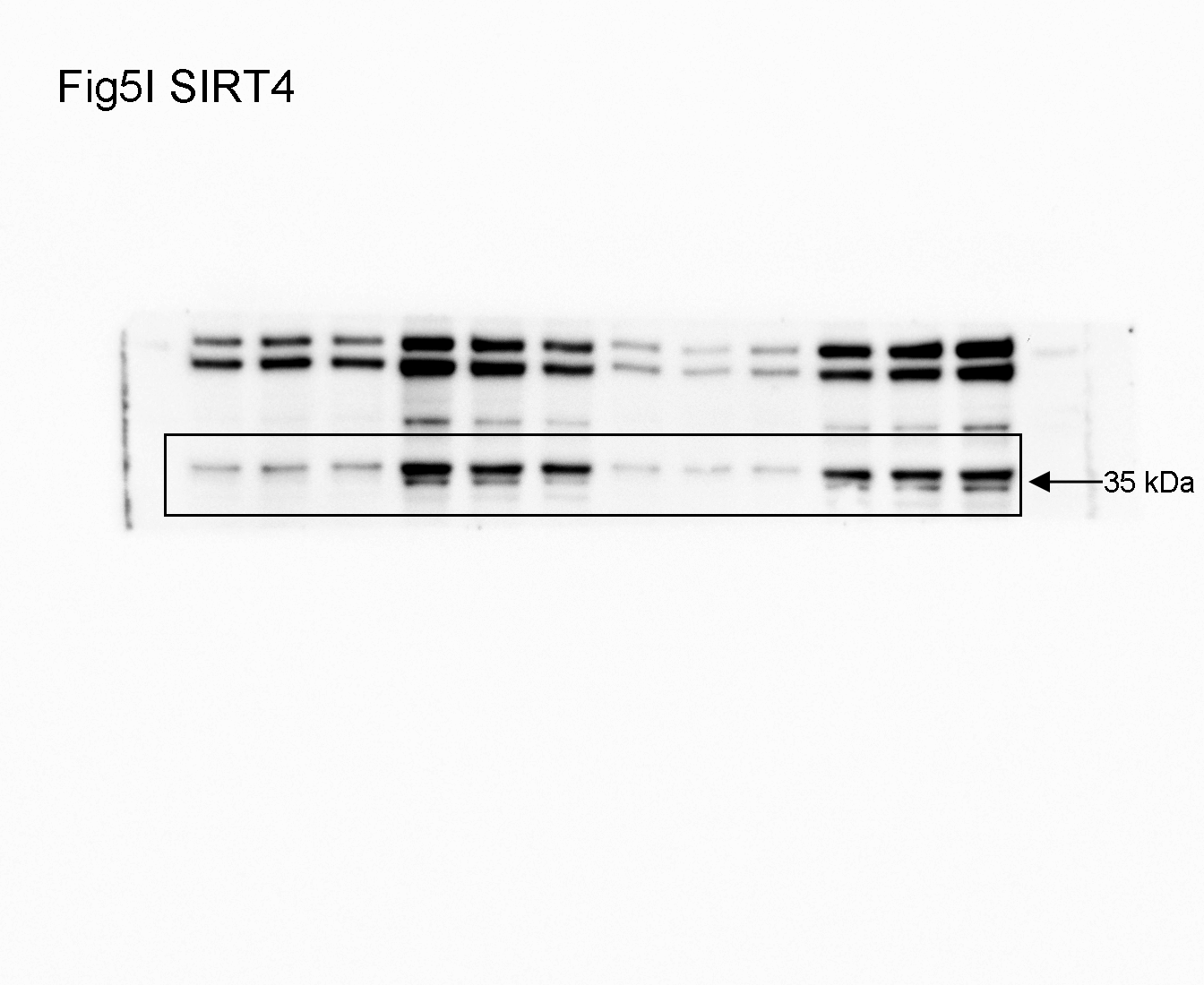

Supplement: Figure 5—source data 2. [file elife-98524-fig5-data2.zip › Fig 5-data2-v1/5I/SIRT4.tif]

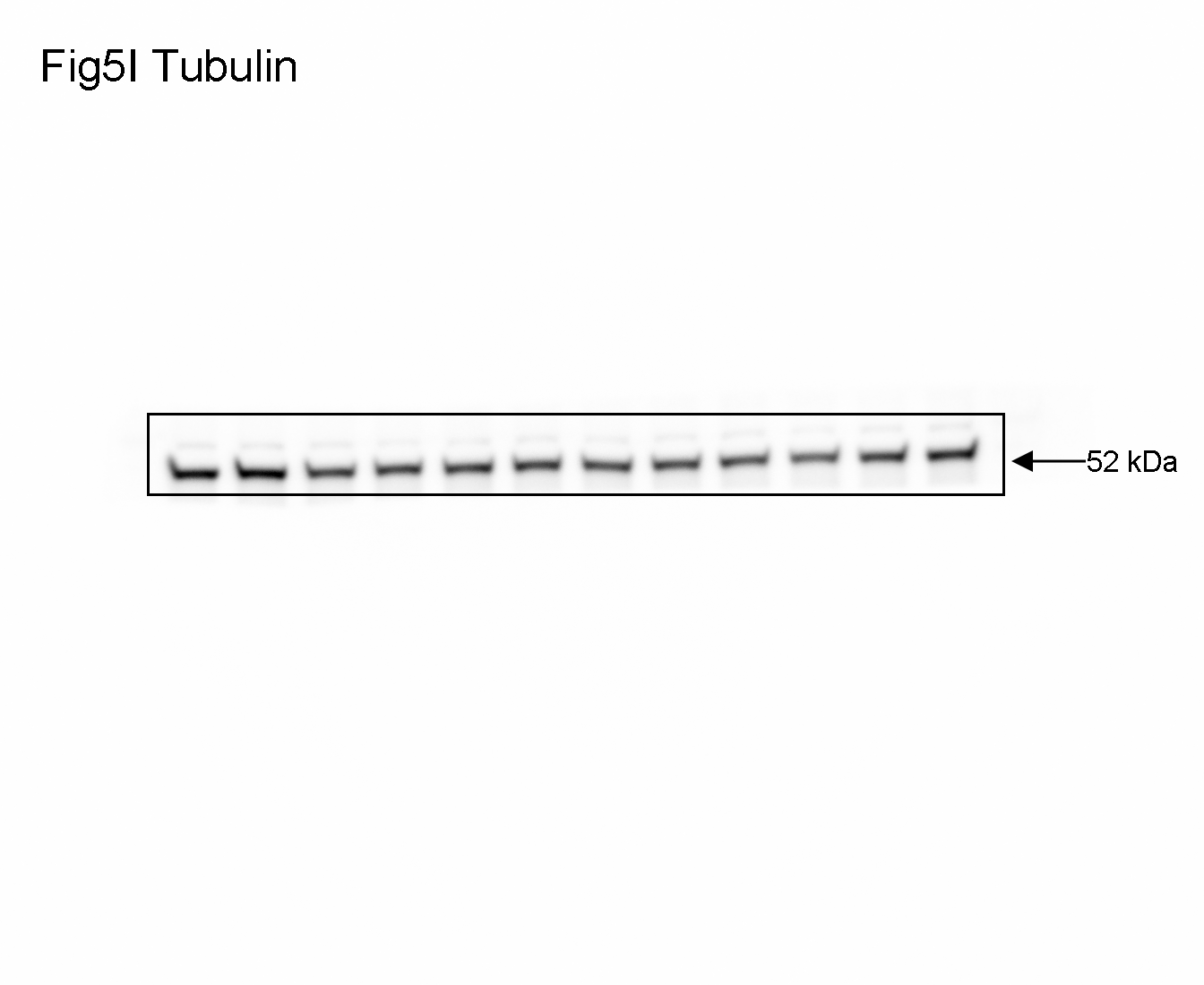

Supplement: Figure 5—source data 2. [file elife-98524-fig5-data2.zip › Fig 5-data2-v1/5I/Tubulin.tif]

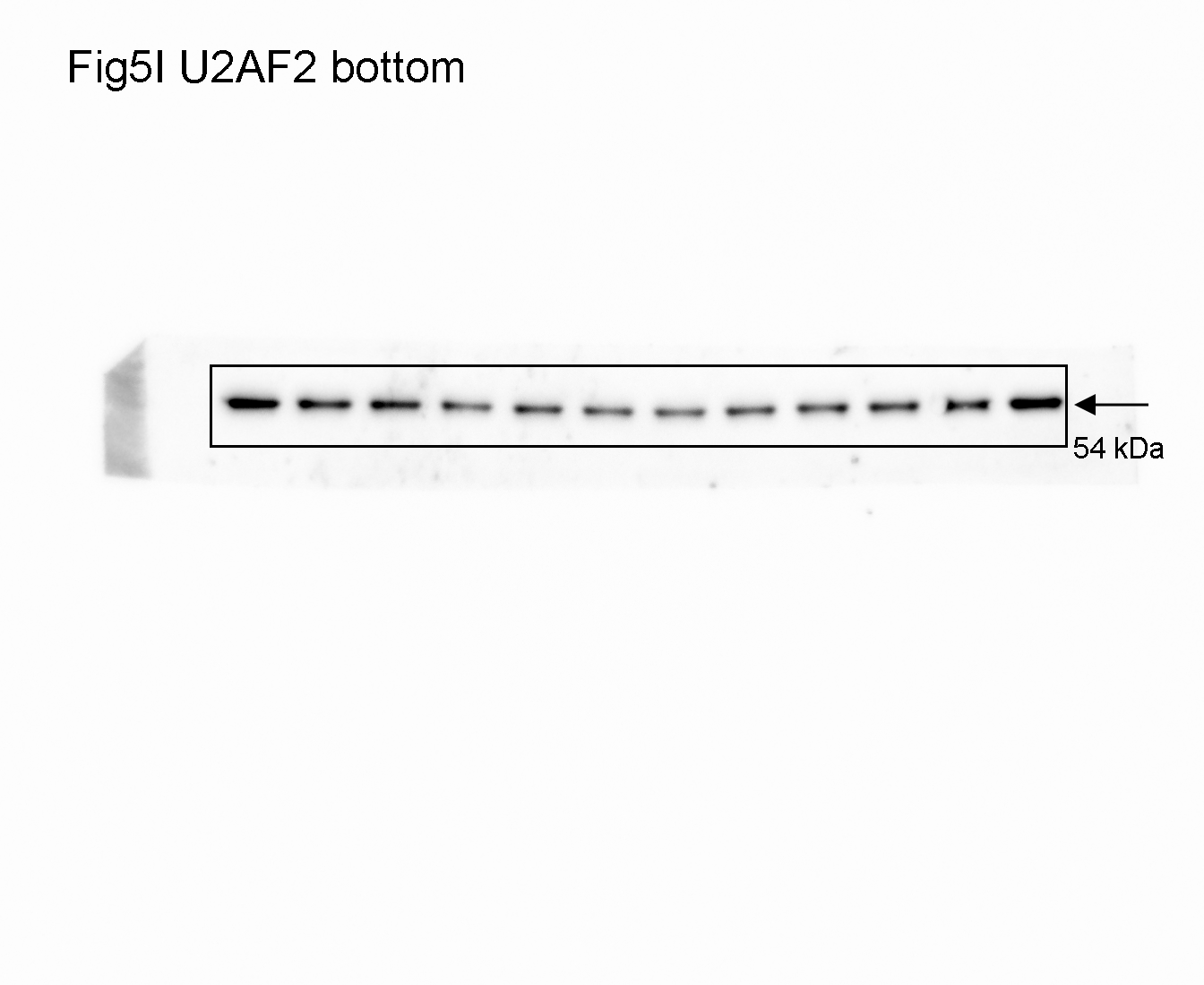

Supplement: Figure 5—source data 2. [file elife-98524-fig5-data2.zip › Fig 5-data2-v1/5I/U2AF2 bottom.tif]

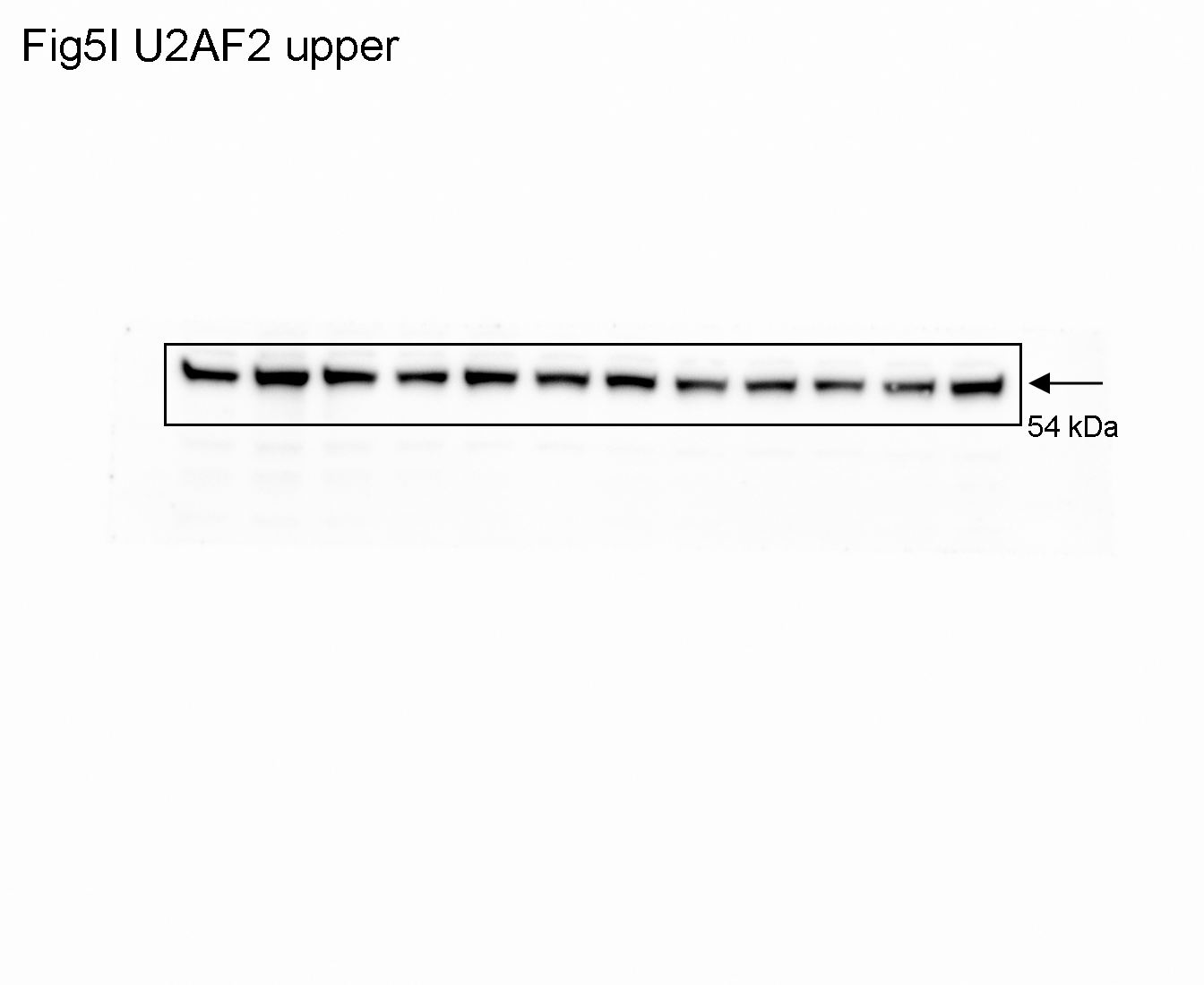

Supplement: Figure 5—source data 2. [file elife-98524-fig5-data2.zip › Fig 5-data2-v1/5I/U2AF2 upper.tif]

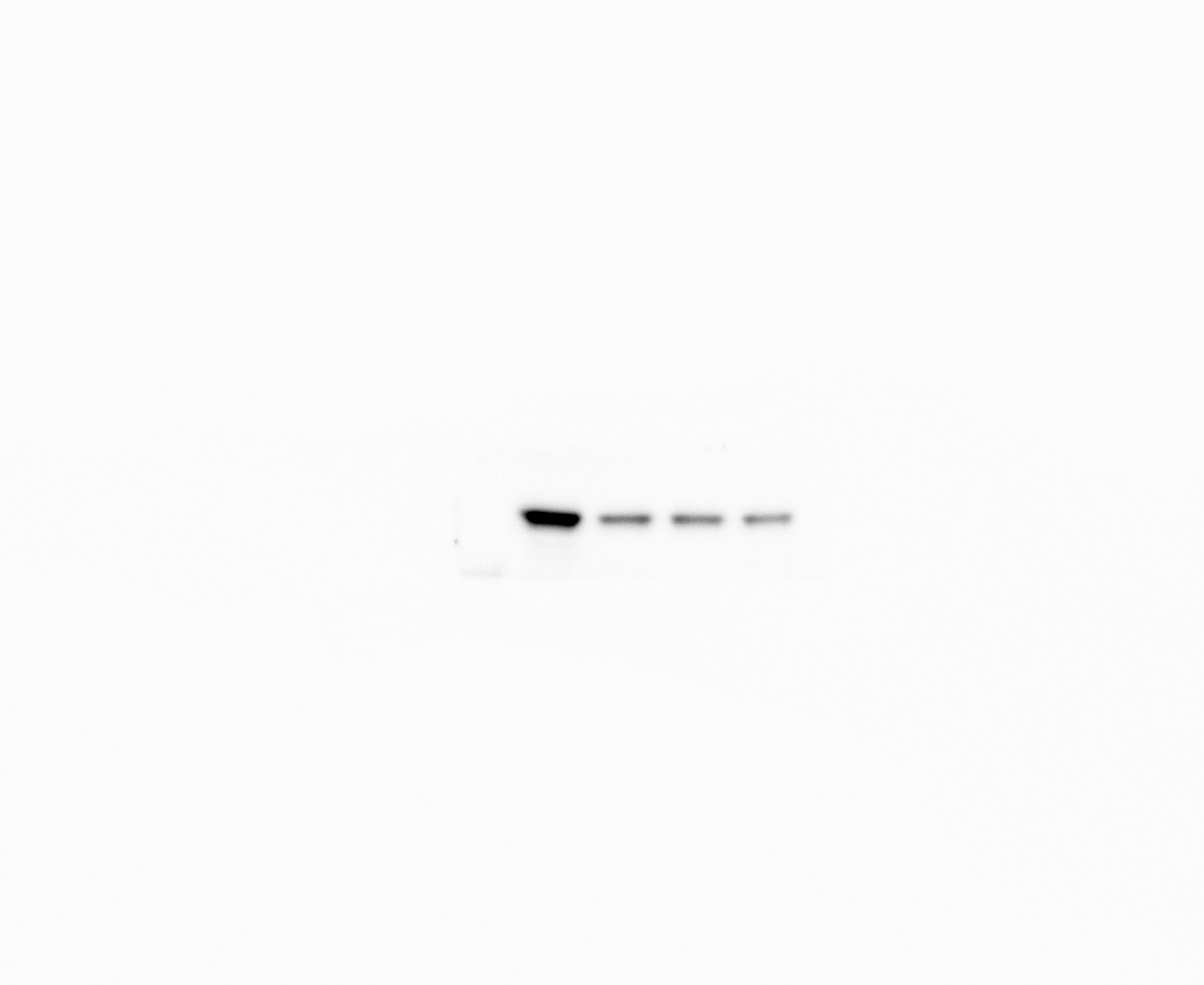

Supplement: Figure 6—source data 1. [file elife-98524-fig6-data1.zip › Fig 6-data1-v1/6C/Ac-k.tif]

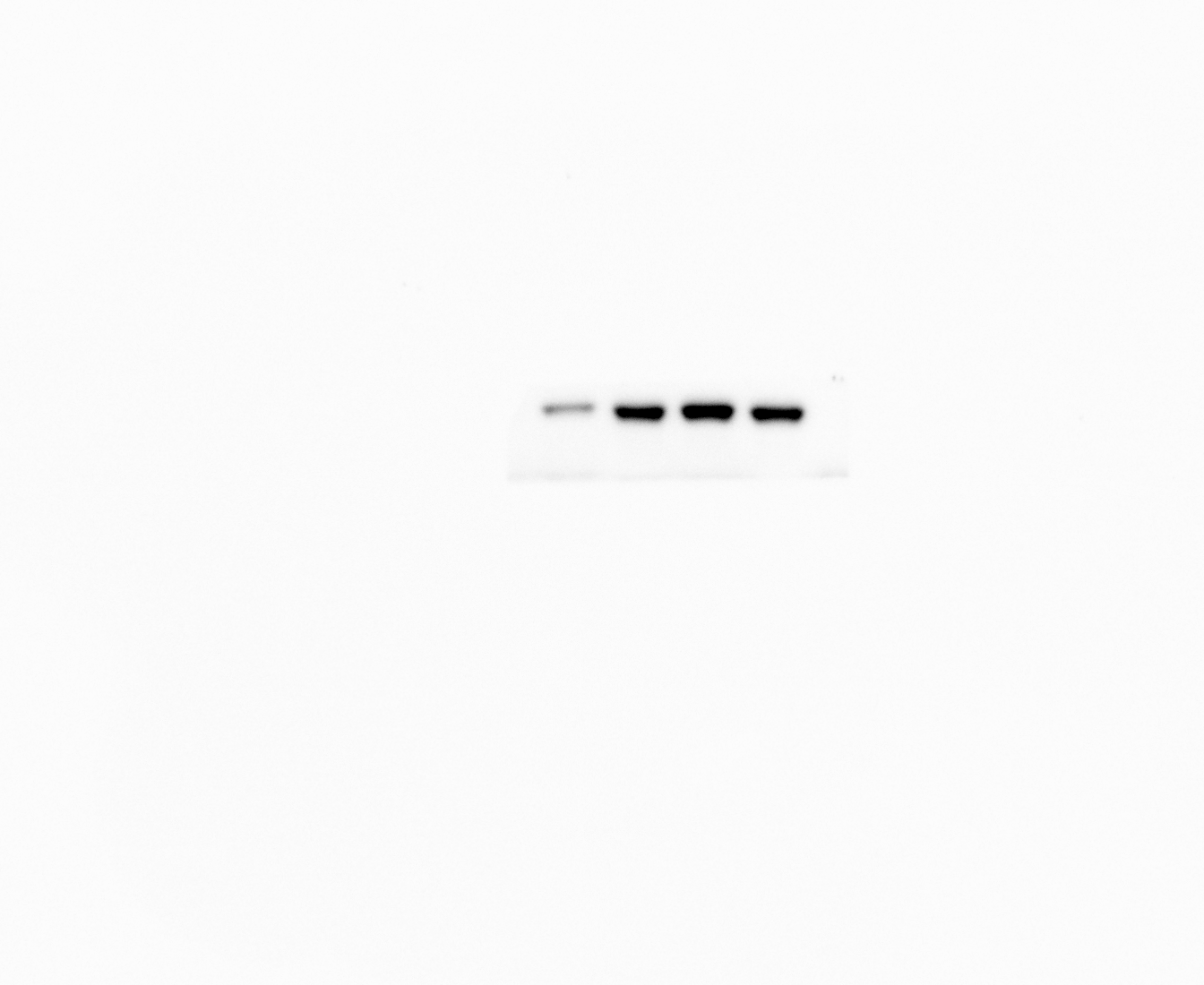

Supplement: Figure 6—source data 1. [file elife-98524-fig6-data1.zip › Fig 6-data1-v1/6C/CCN2.tif]

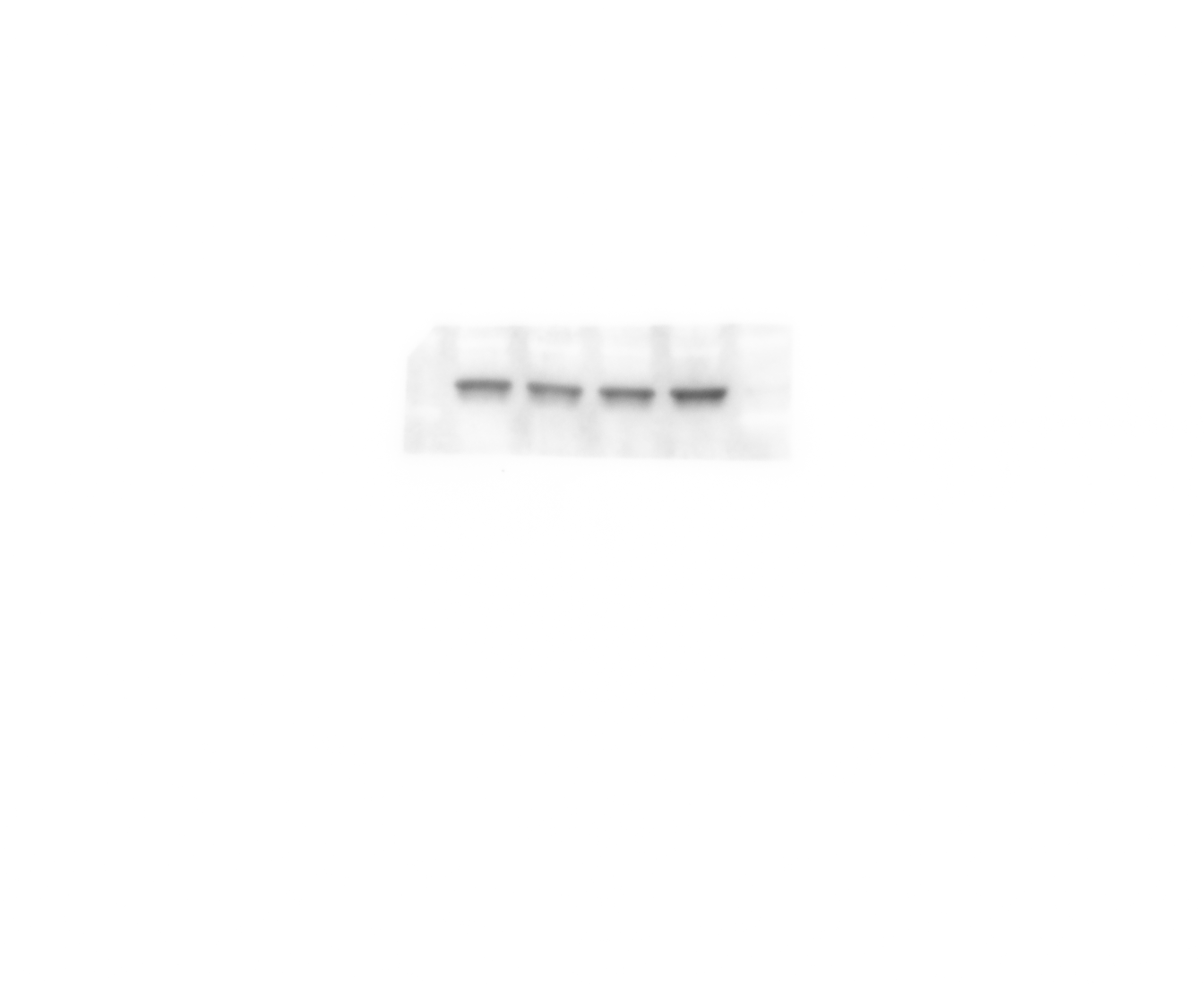

Supplement: Figure 6—source data 1. [file elife-98524-fig6-data1.zip › Fig 6-data1-v1/6C/Flag bottom.tif]

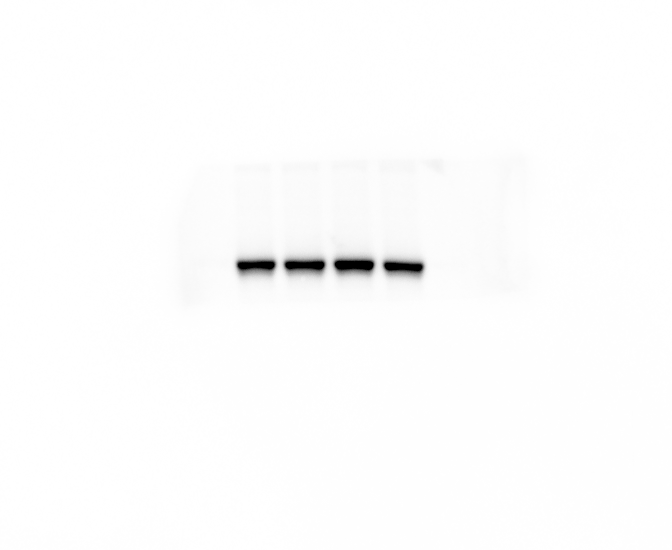

Supplement: Figure 6—source data 1. [file elife-98524-fig6-data1.zip › Fig 6-data1-v1/6C/Flag upper.tif]

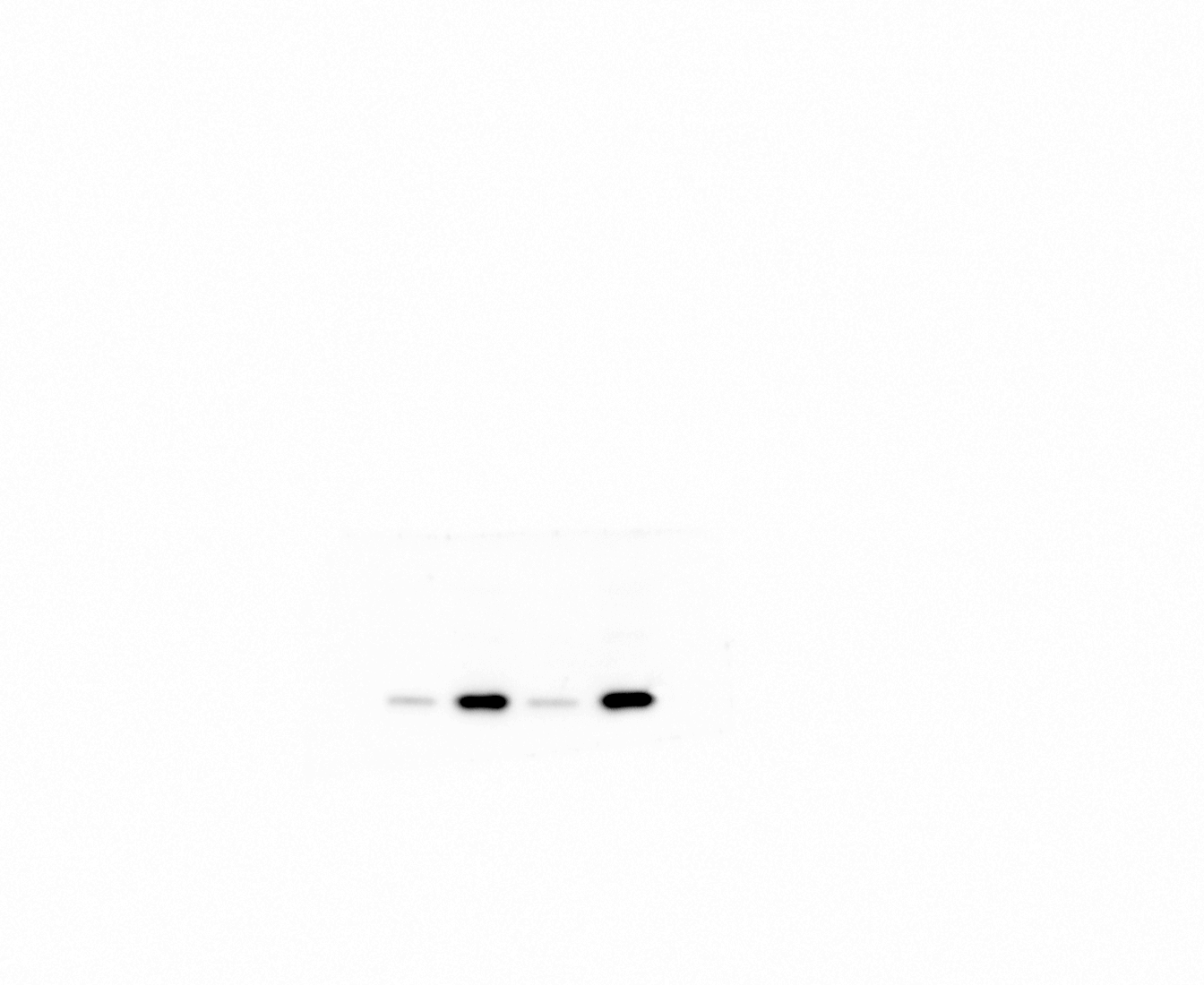

Supplement: Figure 6—source data 1. [file elife-98524-fig6-data1.zip › Fig 6-data1-v1/6C/SIRT4.tif]

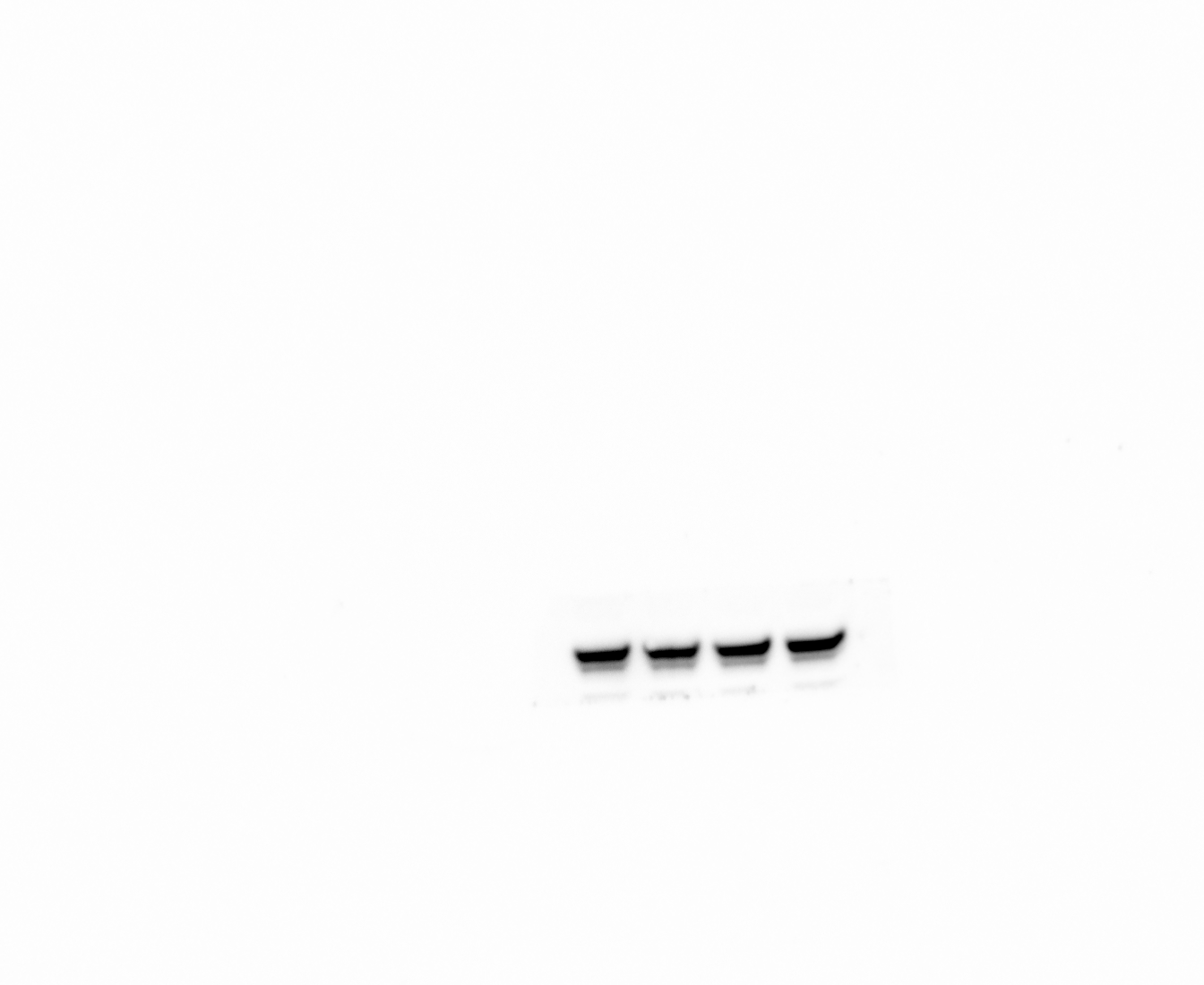

Supplement: Figure 6—source data 1. [file elife-98524-fig6-data1.zip › Fig 6-data1-v1/6C/Tubulin.tif]

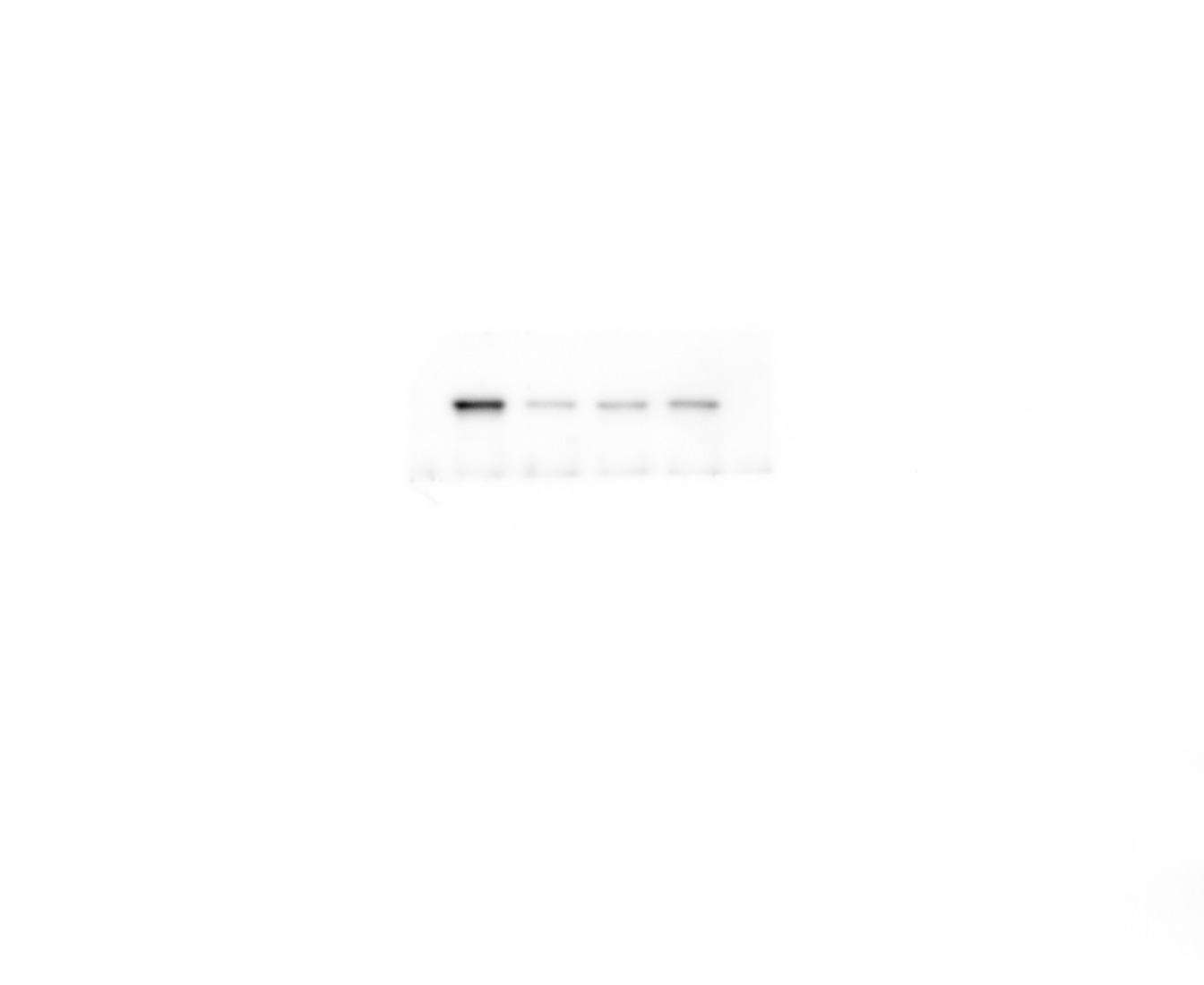

Supplement: Figure 6—source data 1. [file elife-98524-fig6-data1.zip › Fig 6-data1-v1/6D/Ac-k.tif]

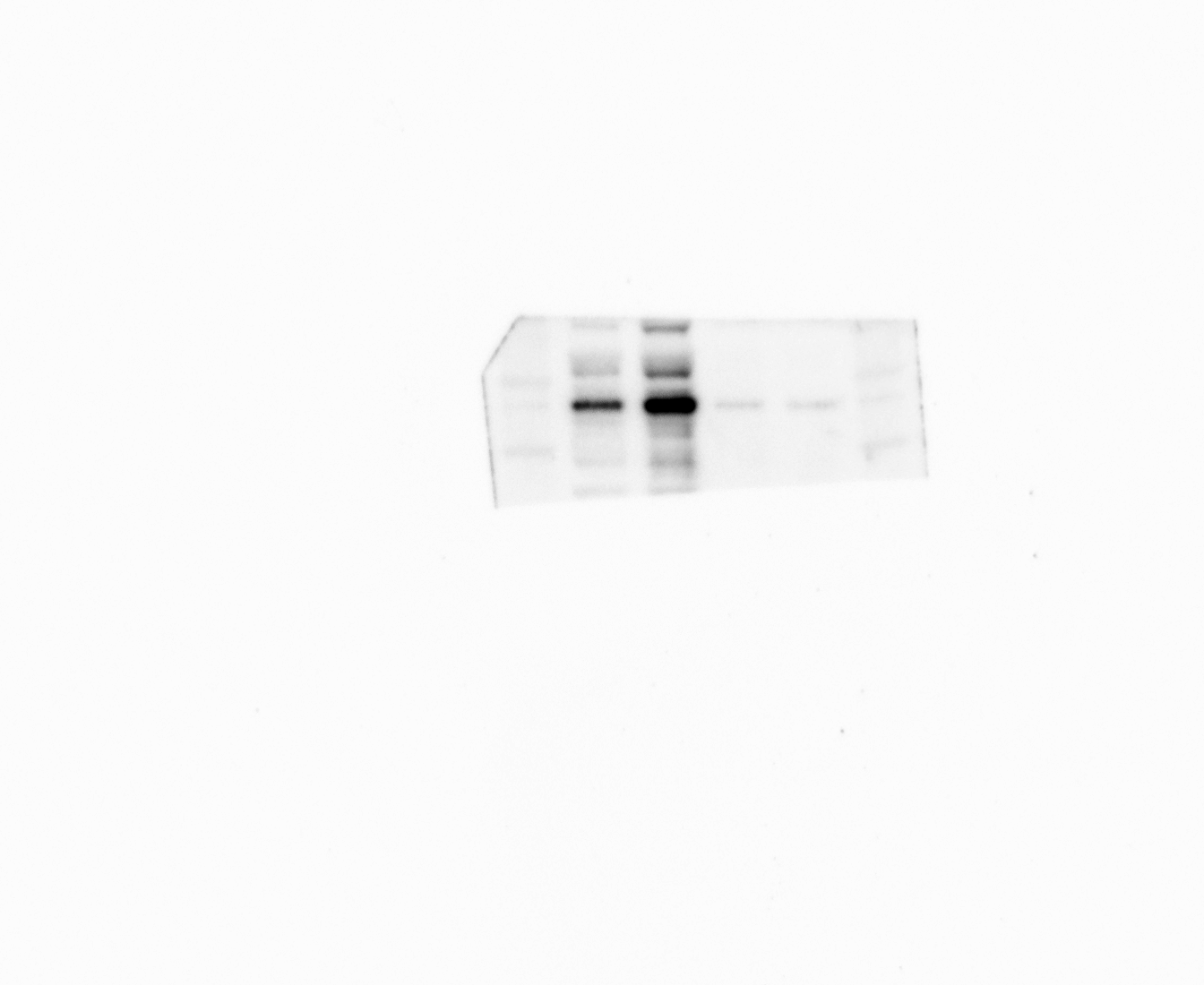

Supplement: Figure 6—source data 1. [file elife-98524-fig6-data1.zip › Fig 6-data1-v1/6D/CCN2.tif]

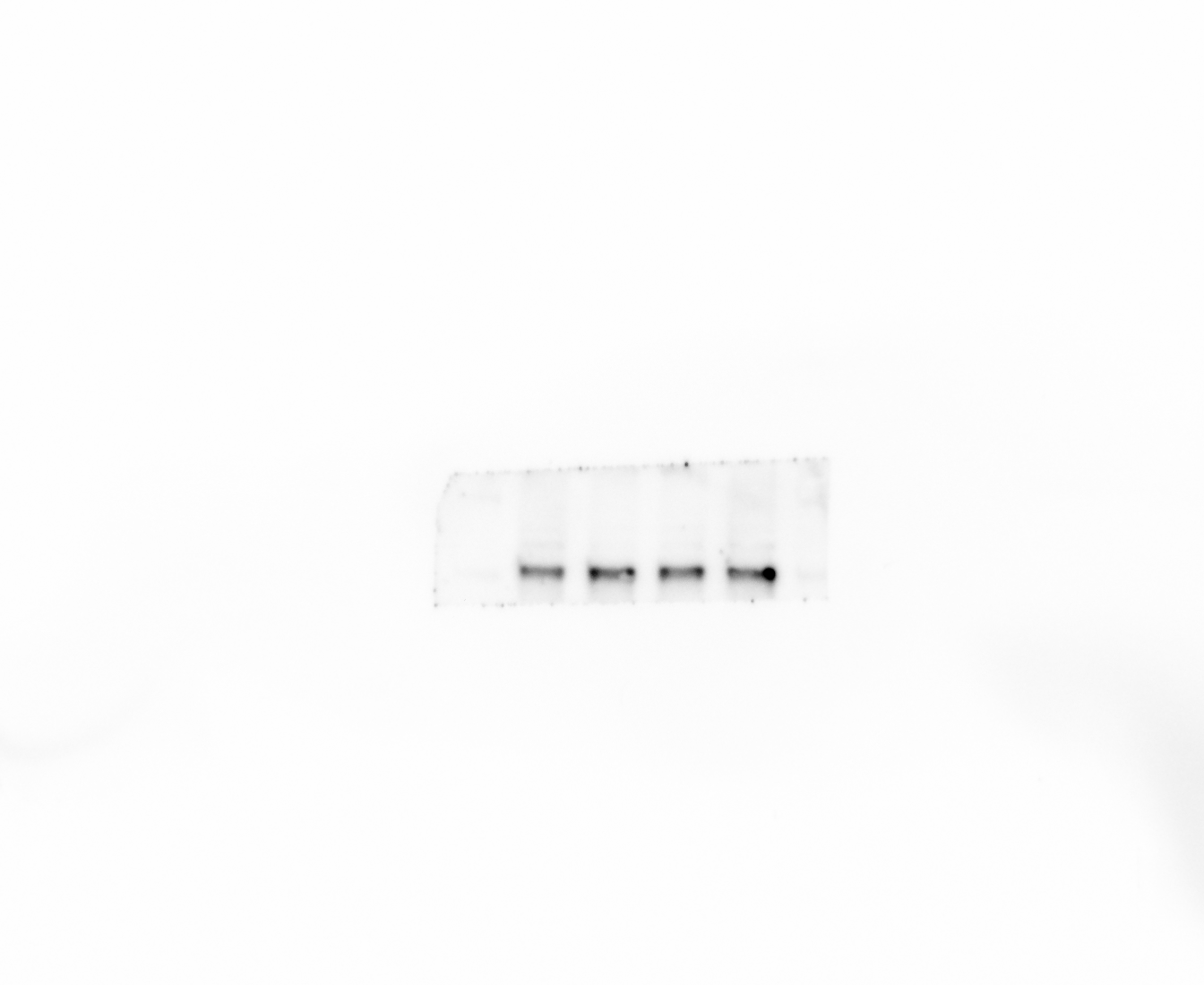

Supplement: Figure 6—source data 1. [file elife-98524-fig6-data1.zip › Fig 6-data1-v1/6D/Flag bottom.tif]

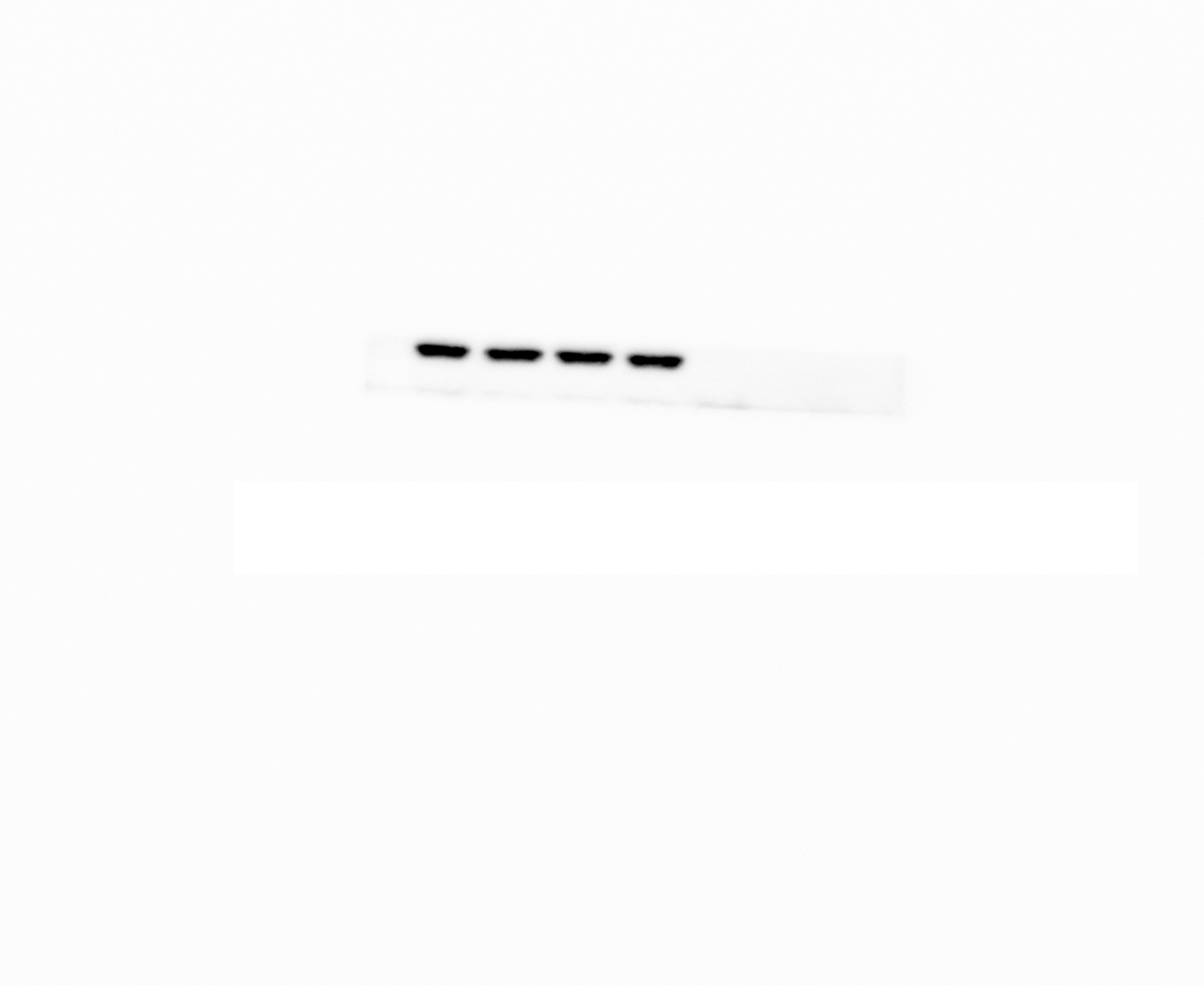

Supplement: Figure 6—source data 1. [file elife-98524-fig6-data1.zip › Fig 6-data1-v1/6D/Flag upper.tif]

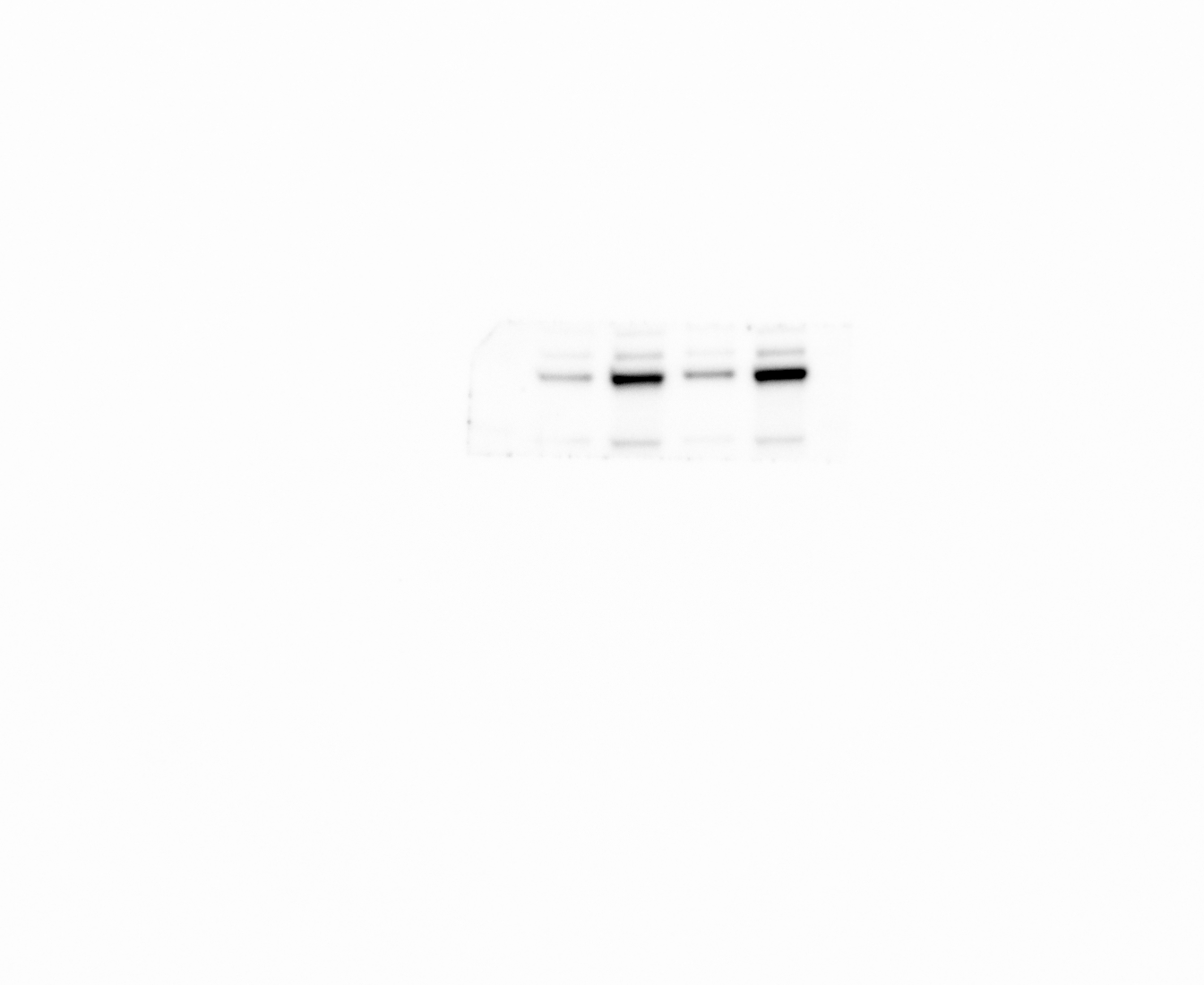

Supplement: Figure 6—source data 1. [file elife-98524-fig6-data1.zip › Fig 6-data1-v1/6D/SIRT4.tif]

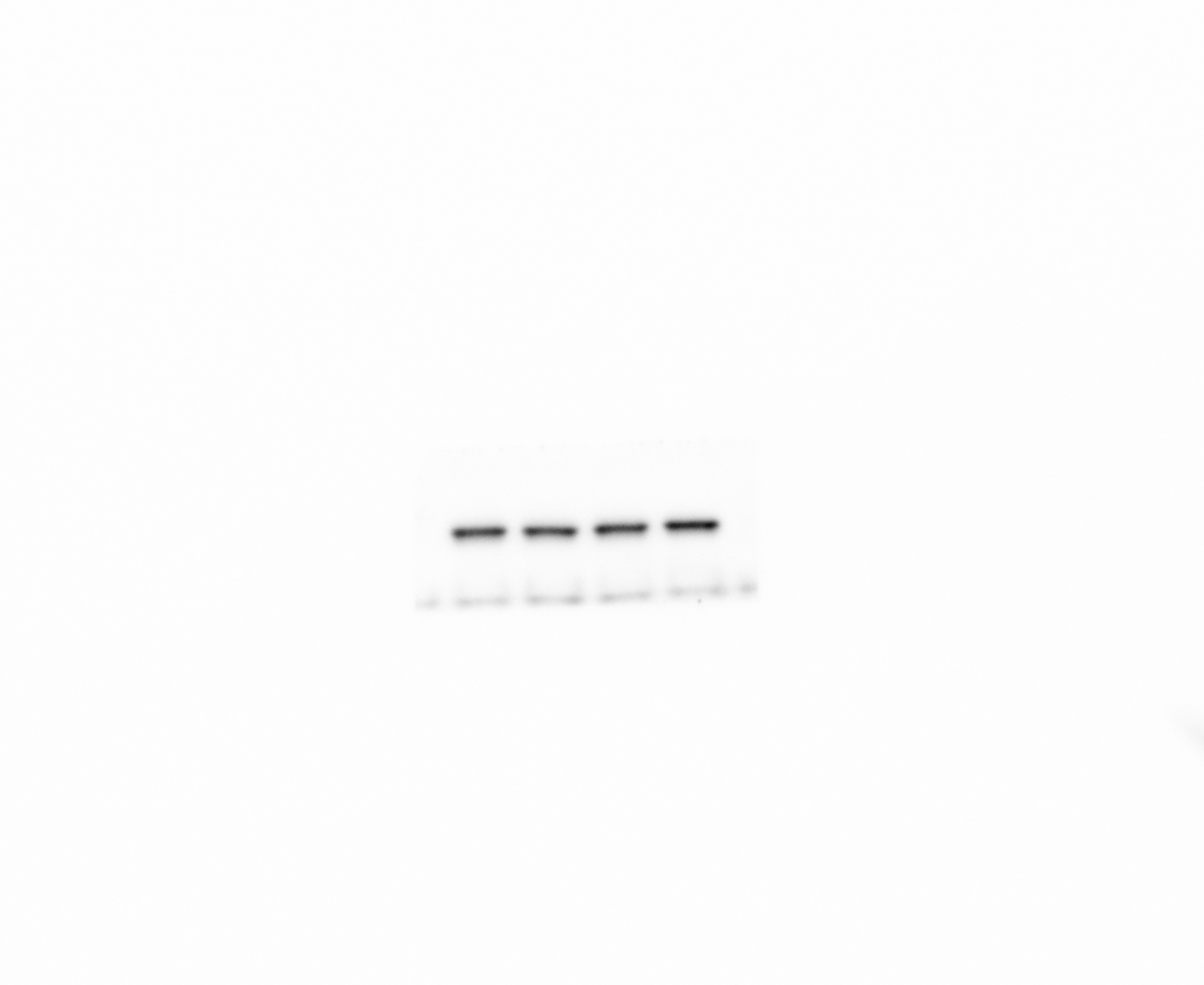

Supplement: Figure 6—source data 1. [file elife-98524-fig6-data1.zip › Fig 6-data1-v1/6D/Tubulin.tif]

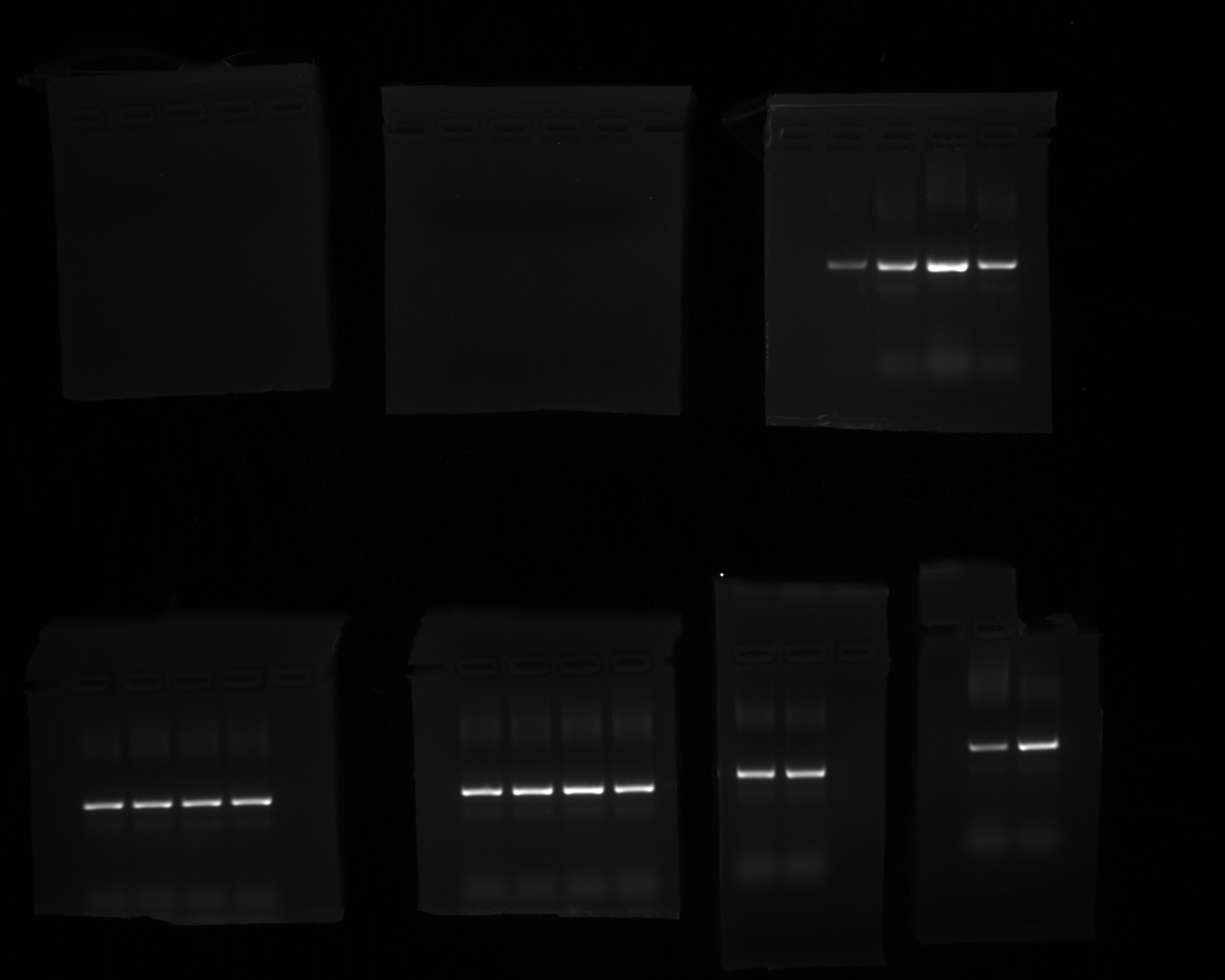

Supplement: Figure 6—source data 1. [file elife-98524-fig6-data1.zip › Fig 6-data1-v1/6J/4J-1 Input IgG+4J-2 IgG.tif]

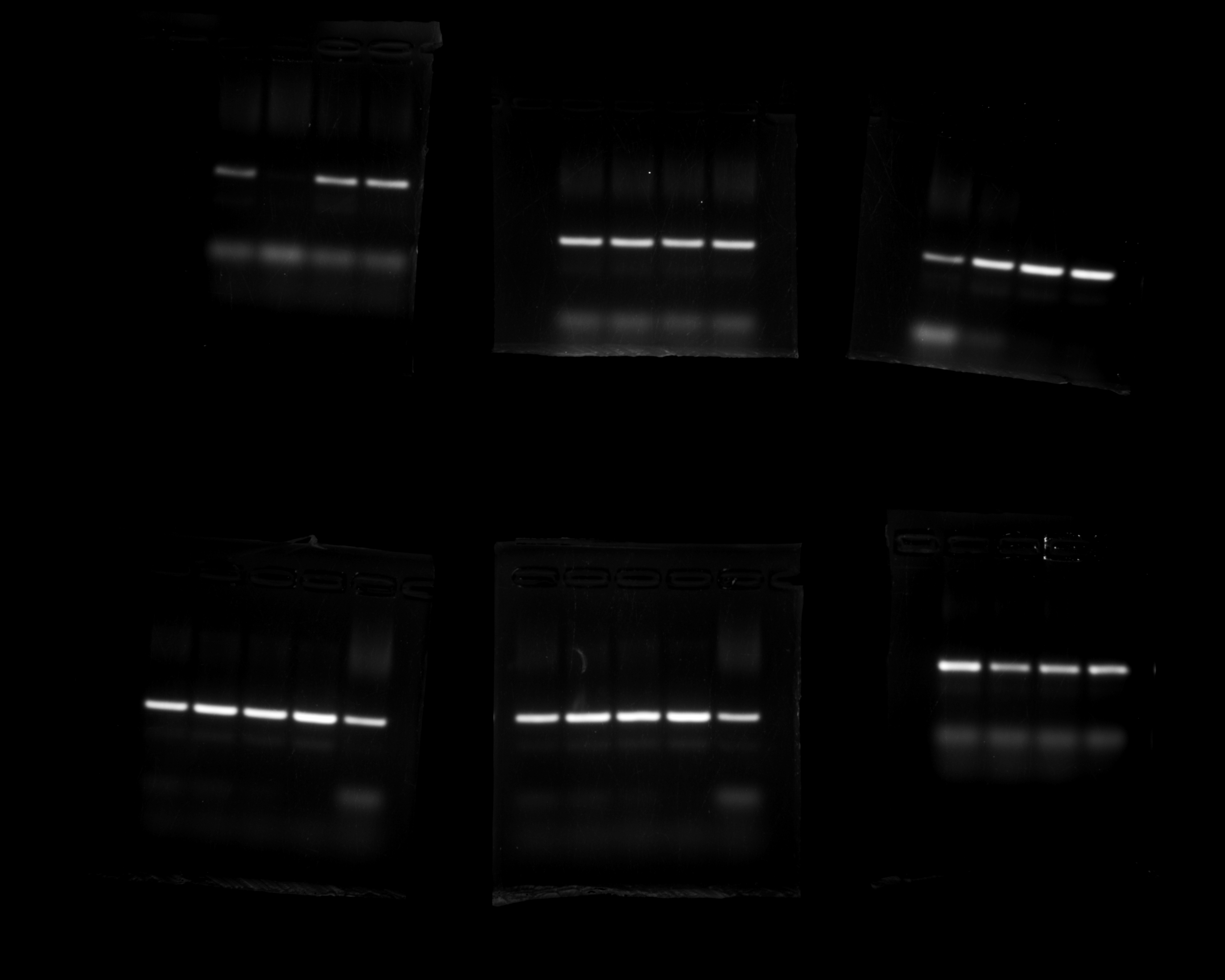

Supplement: Figure 6—source data 1. [file elife-98524-fig6-data1.zip › Fig 6-data1-v1/6J/4J-1 U2AF2.tif]

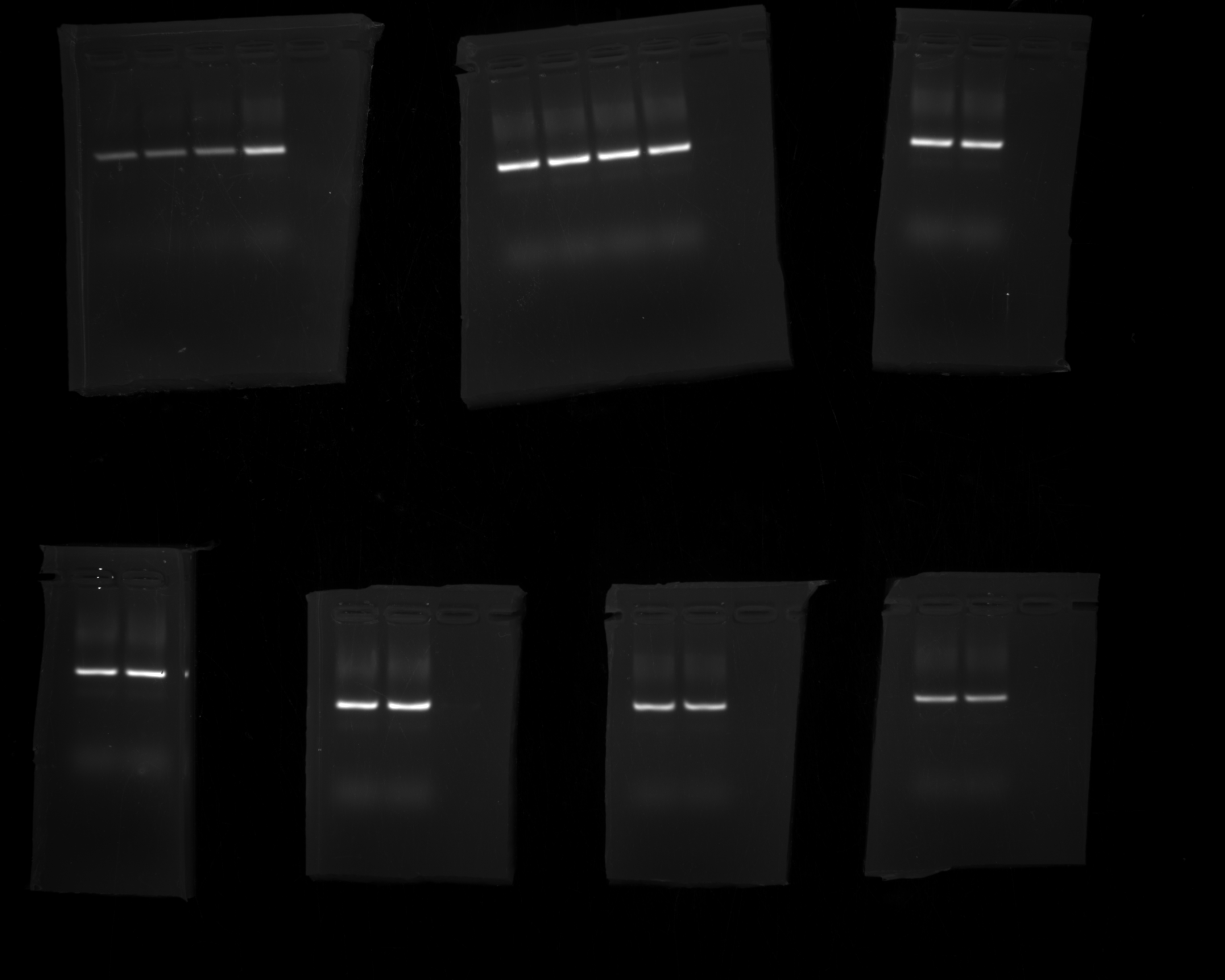

Supplement: Figure 6—source data 1. [file elife-98524-fig6-data1.zip › Fig 6-data1-v1/6J/4J-2 Input.tif]

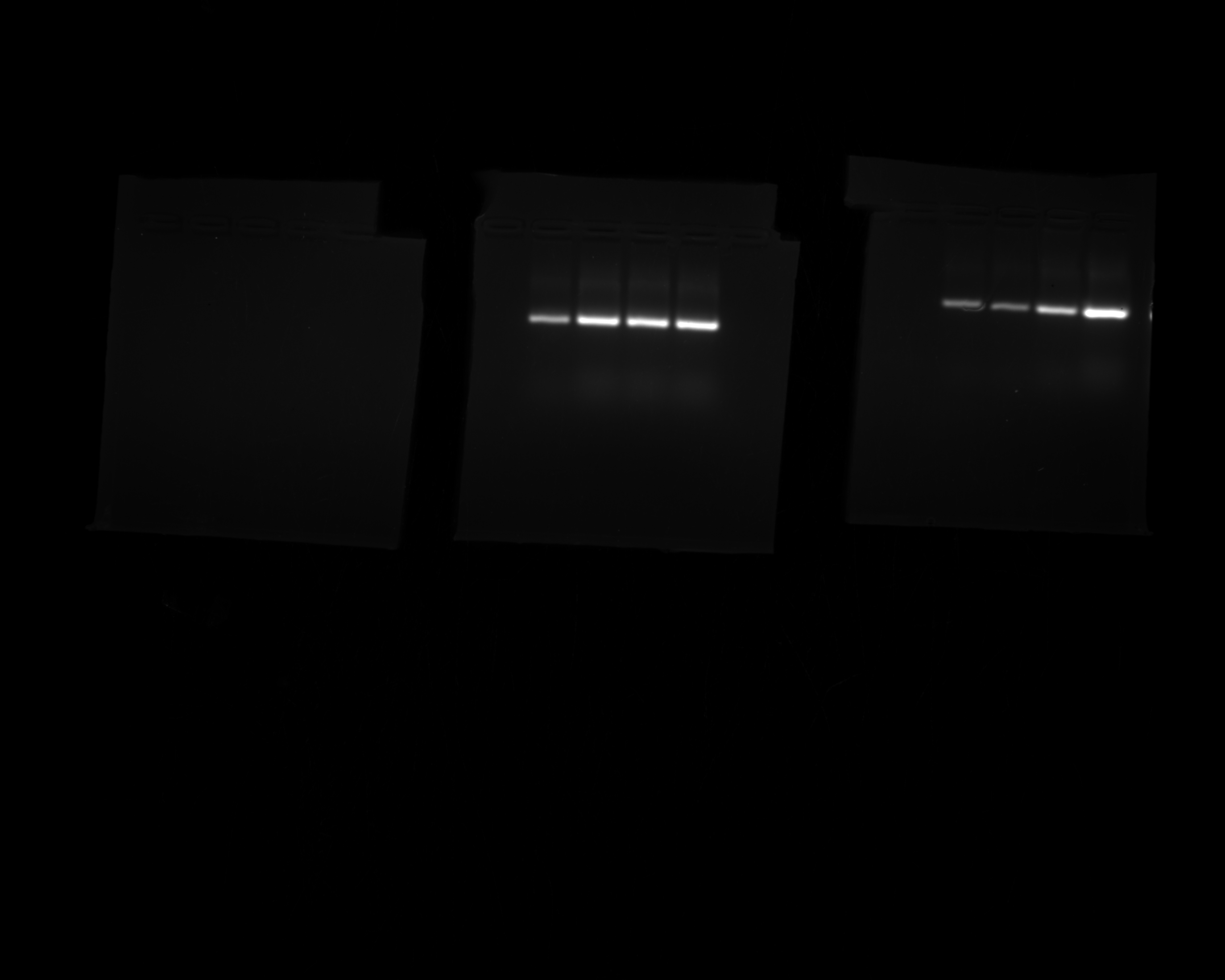

Supplement: Figure 6—source data 1. [file elife-98524-fig6-data1.zip › Fig 6-data1-v1/6J/4J-2 U2AF2.tif]

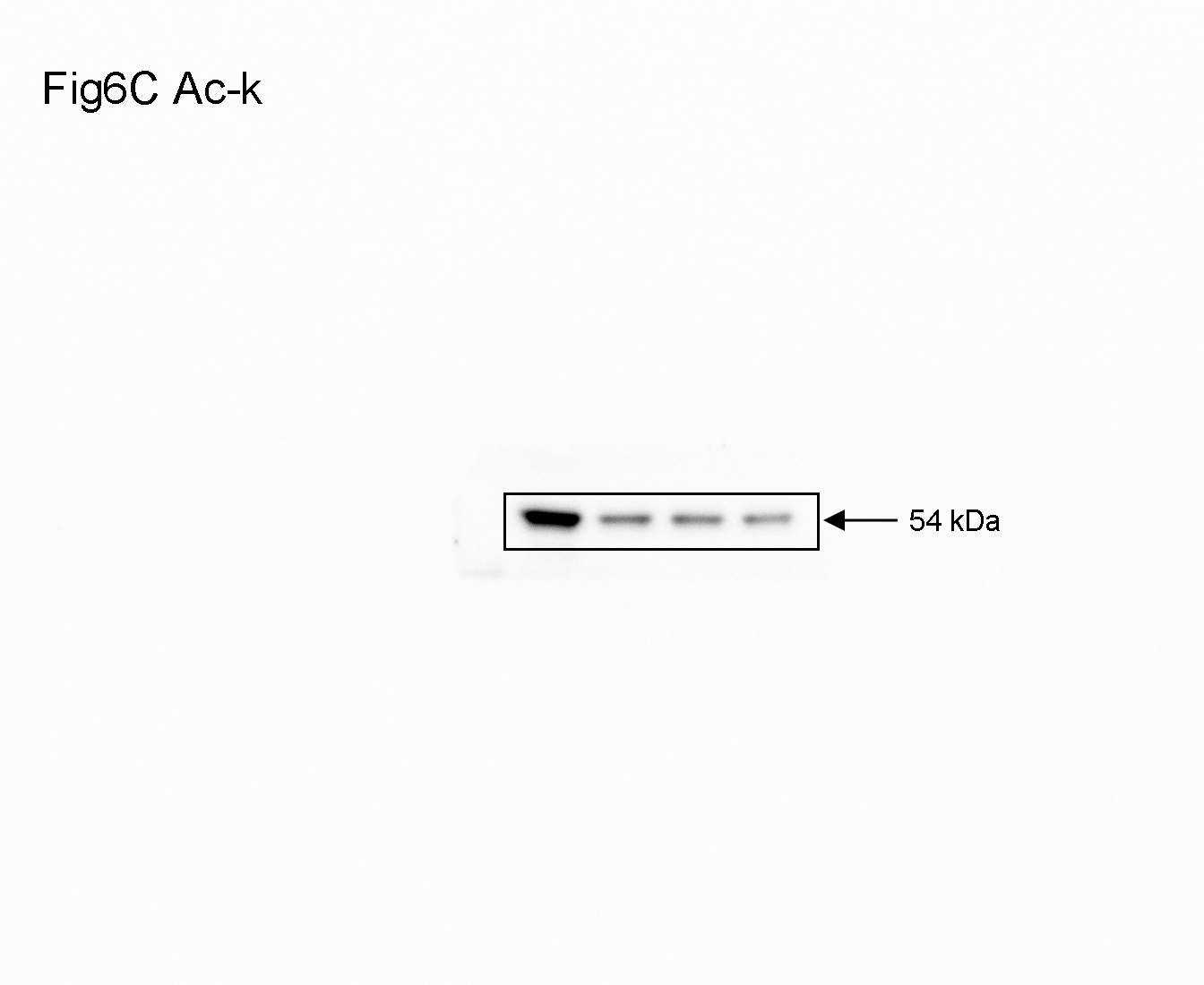

Supplement: Figure 6—source data 2. [file elife-98524-fig6-data2.zip › Fig 6-data2-v1/6C/Ac-k.tif]

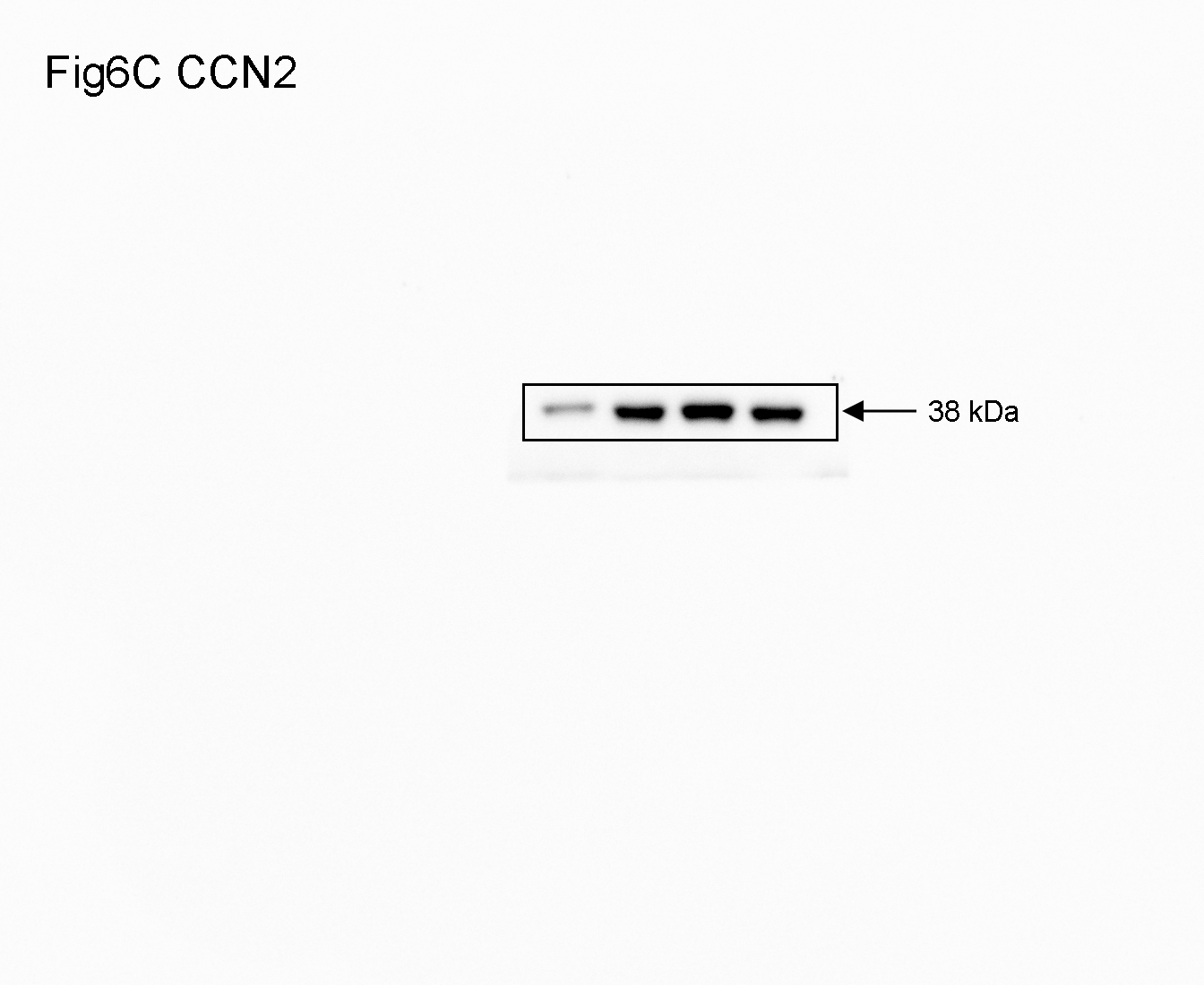

Supplement: Figure 6—source data 2. [file elife-98524-fig6-data2.zip › Fig 6-data2-v1/6C/CCN2.tif]

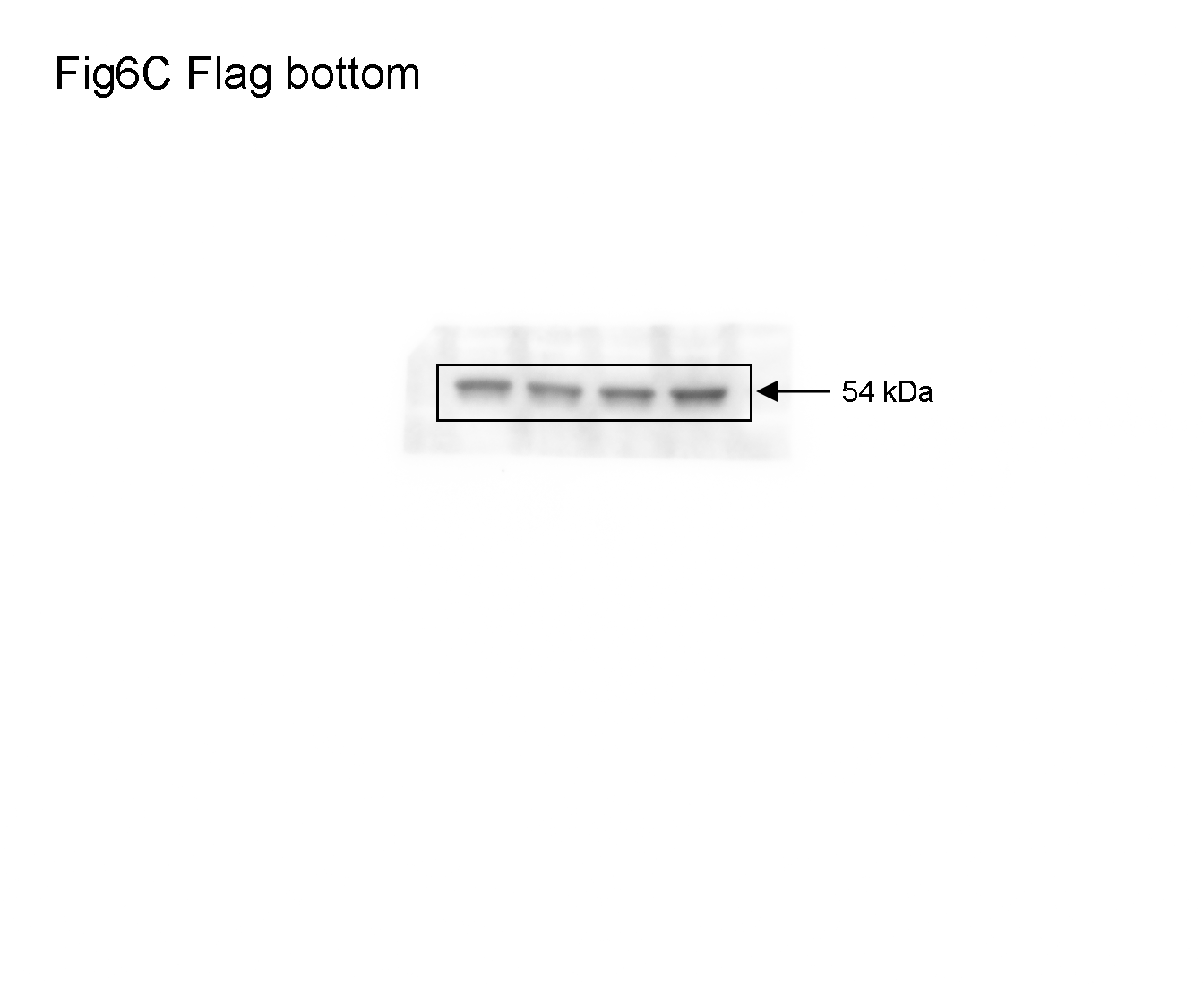

Supplement: Figure 6—source data 2. [file elife-98524-fig6-data2.zip › Fig 6-data2-v1/6C/Flag bottom.tif]

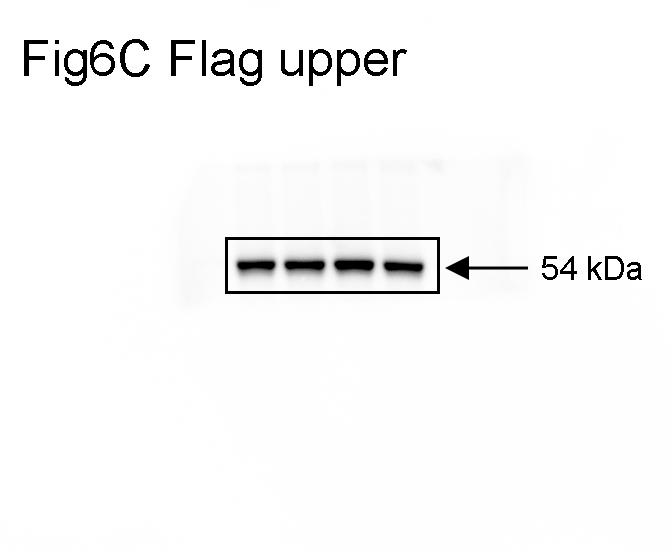

Supplement: Figure 6—source data 2. [file elife-98524-fig6-data2.zip › Fig 6-data2-v1/6C/Flag upper.tif]

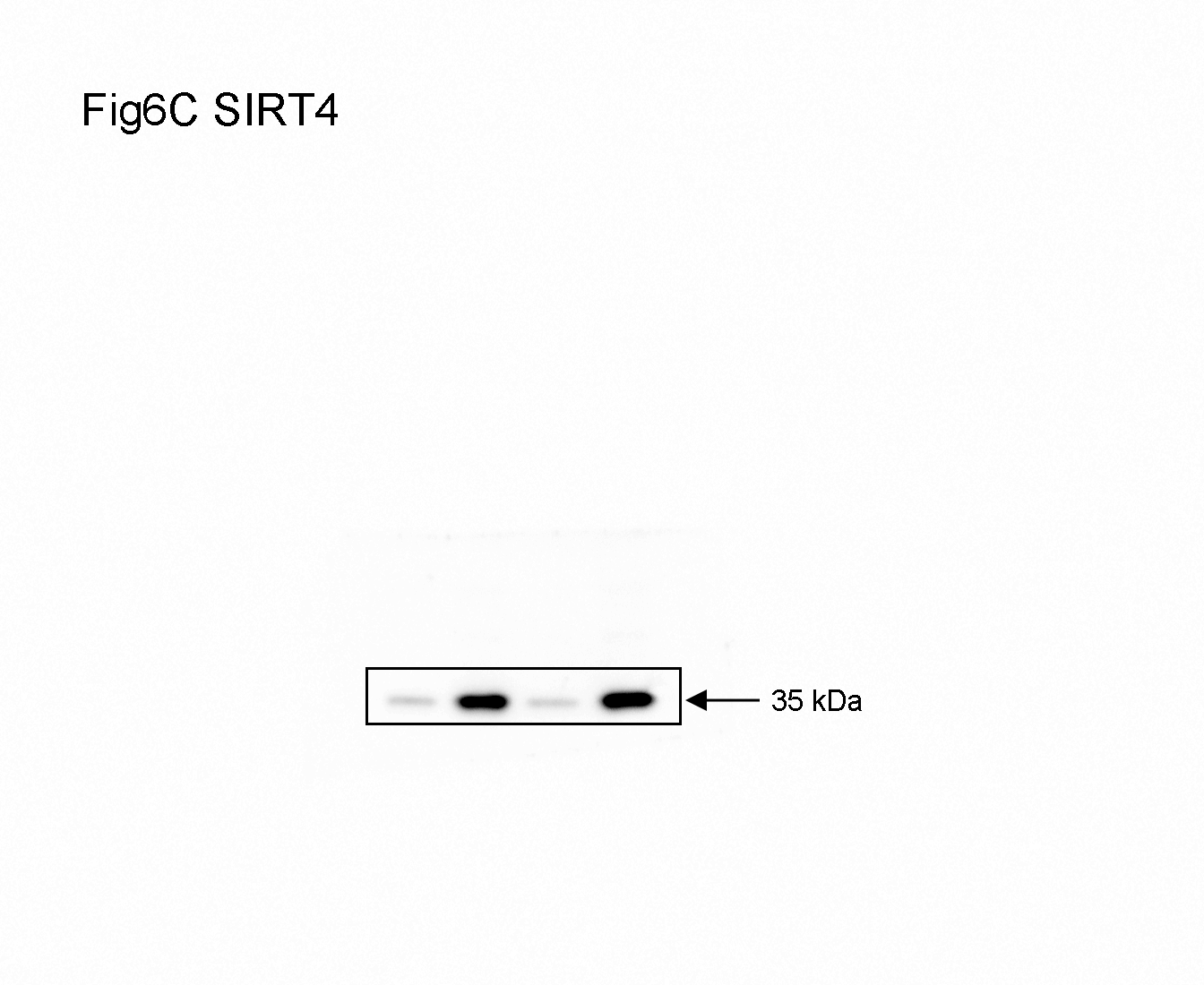

Supplement: Figure 6—source data 2. [file elife-98524-fig6-data2.zip › Fig 6-data2-v1/6C/SIRT4.tif]

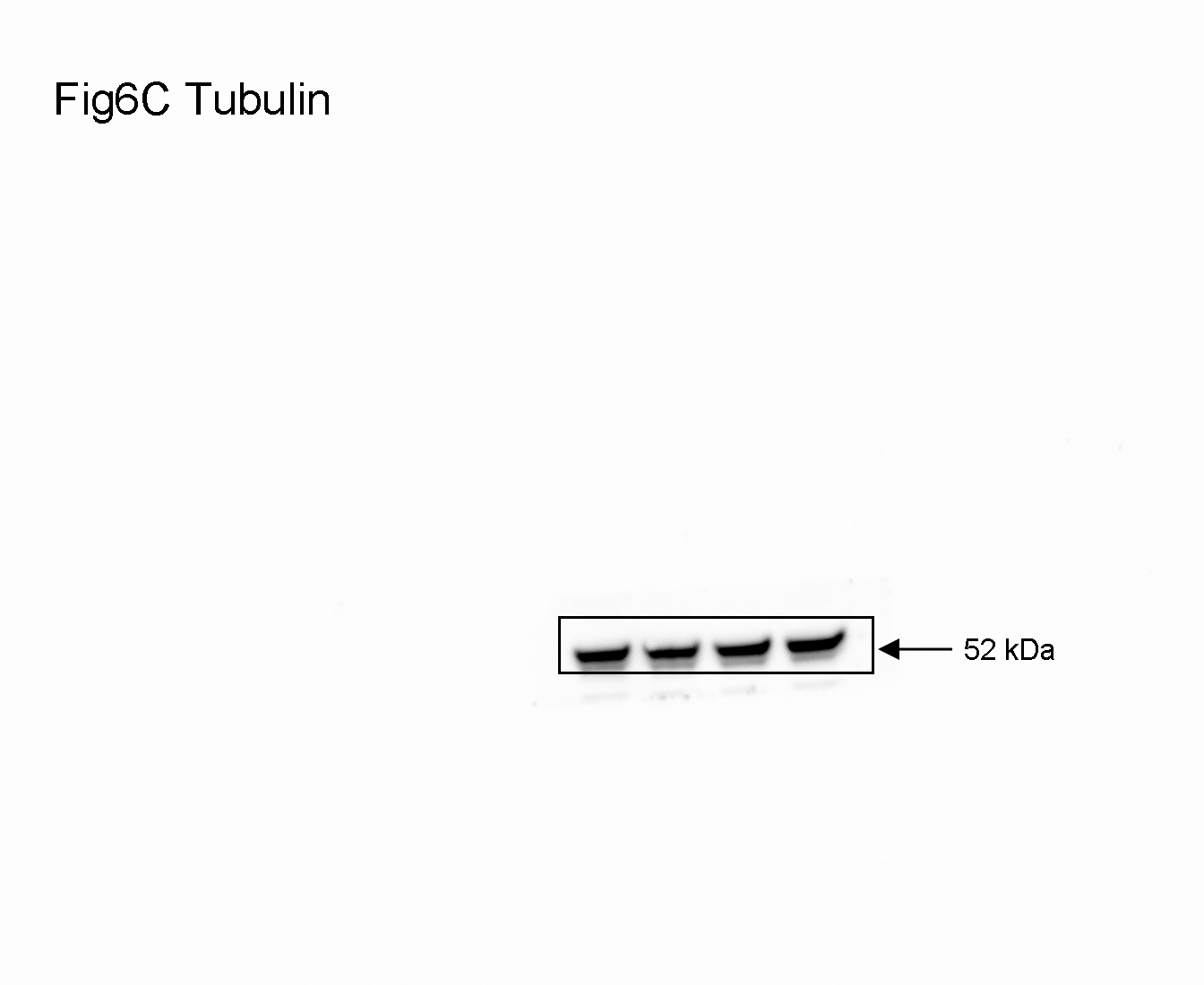

Supplement: Figure 6—source data 2. [file elife-98524-fig6-data2.zip › Fig 6-data2-v1/6C/Tubulin.tif]

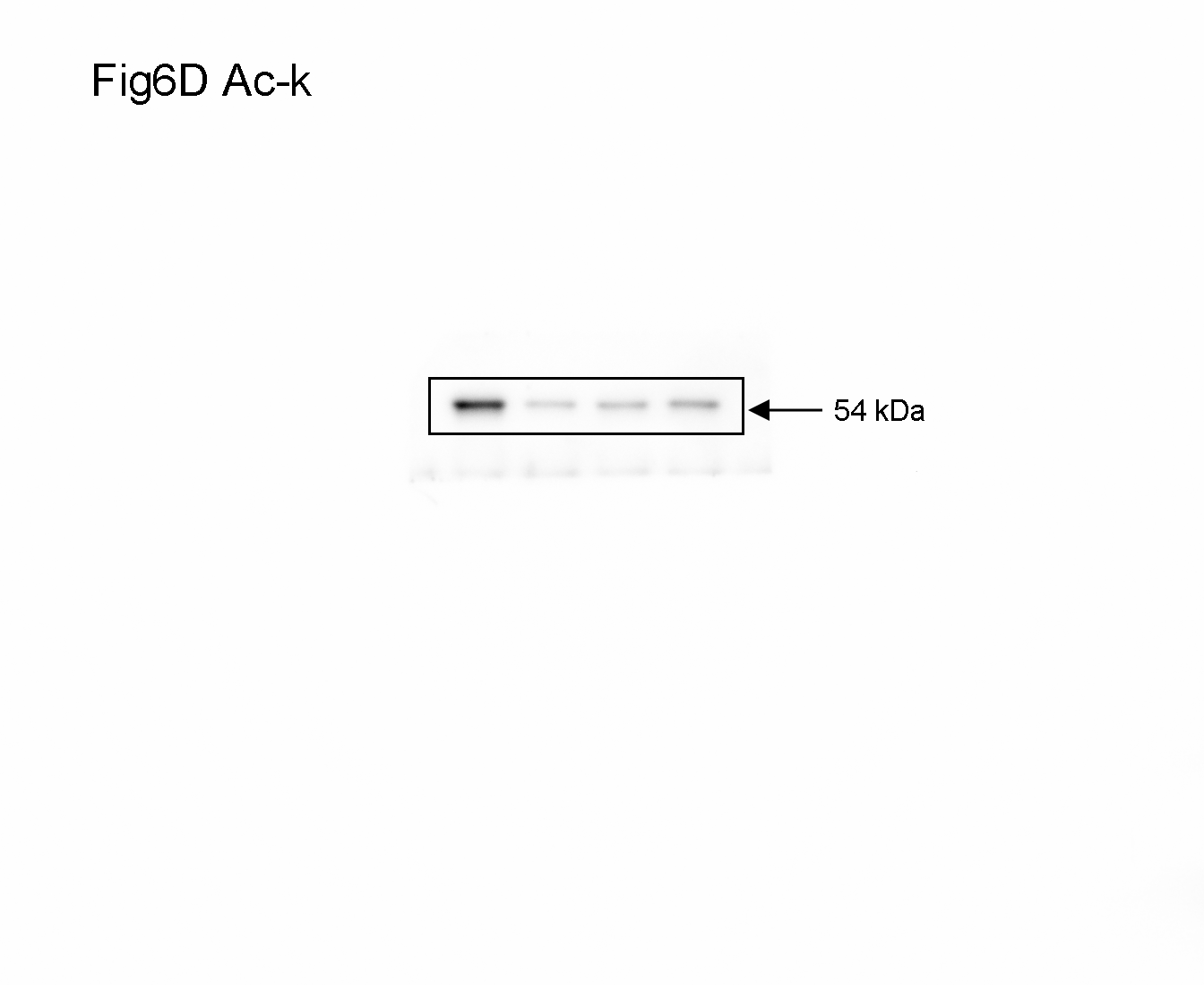

Supplement: Figure 6—source data 2. [file elife-98524-fig6-data2.zip › Fig 6-data2-v1/6D/Ac-k.tif]

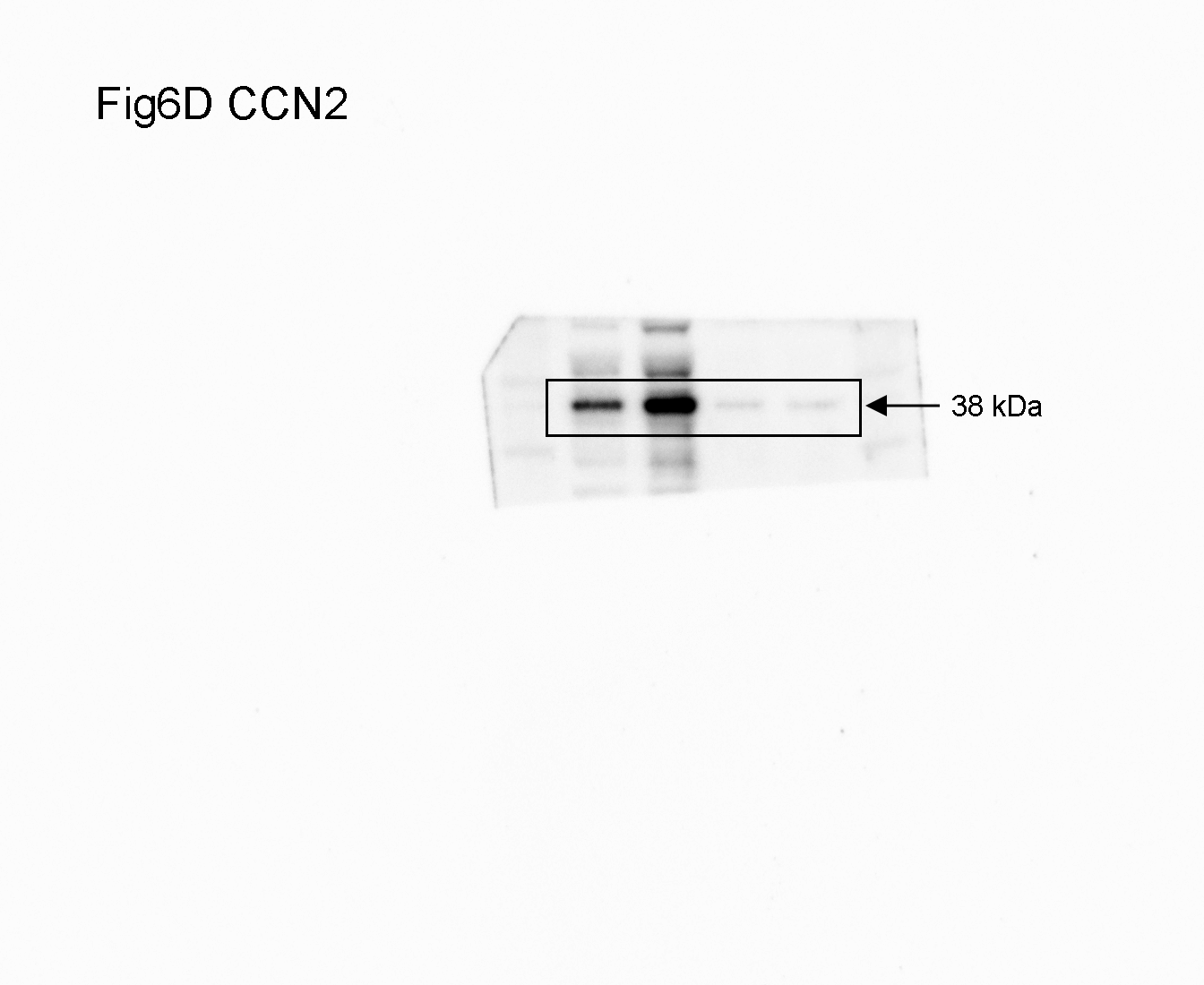

Supplement: Figure 6—source data 2. [file elife-98524-fig6-data2.zip › Fig 6-data2-v1/6D/CCN2.tif]

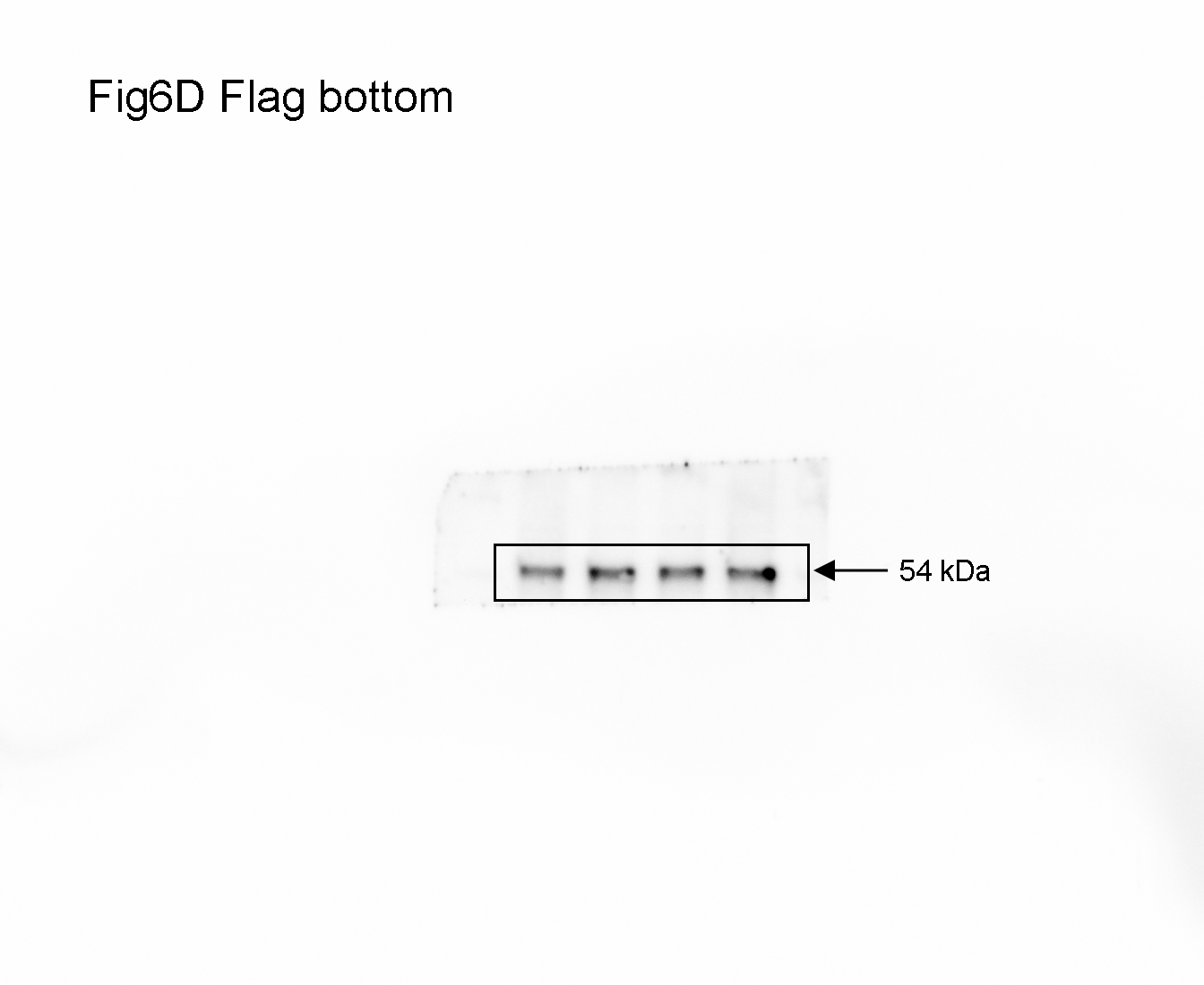

Supplement: Figure 6—source data 2. [file elife-98524-fig6-data2.zip › Fig 6-data2-v1/6D/Flag bottom.tif]

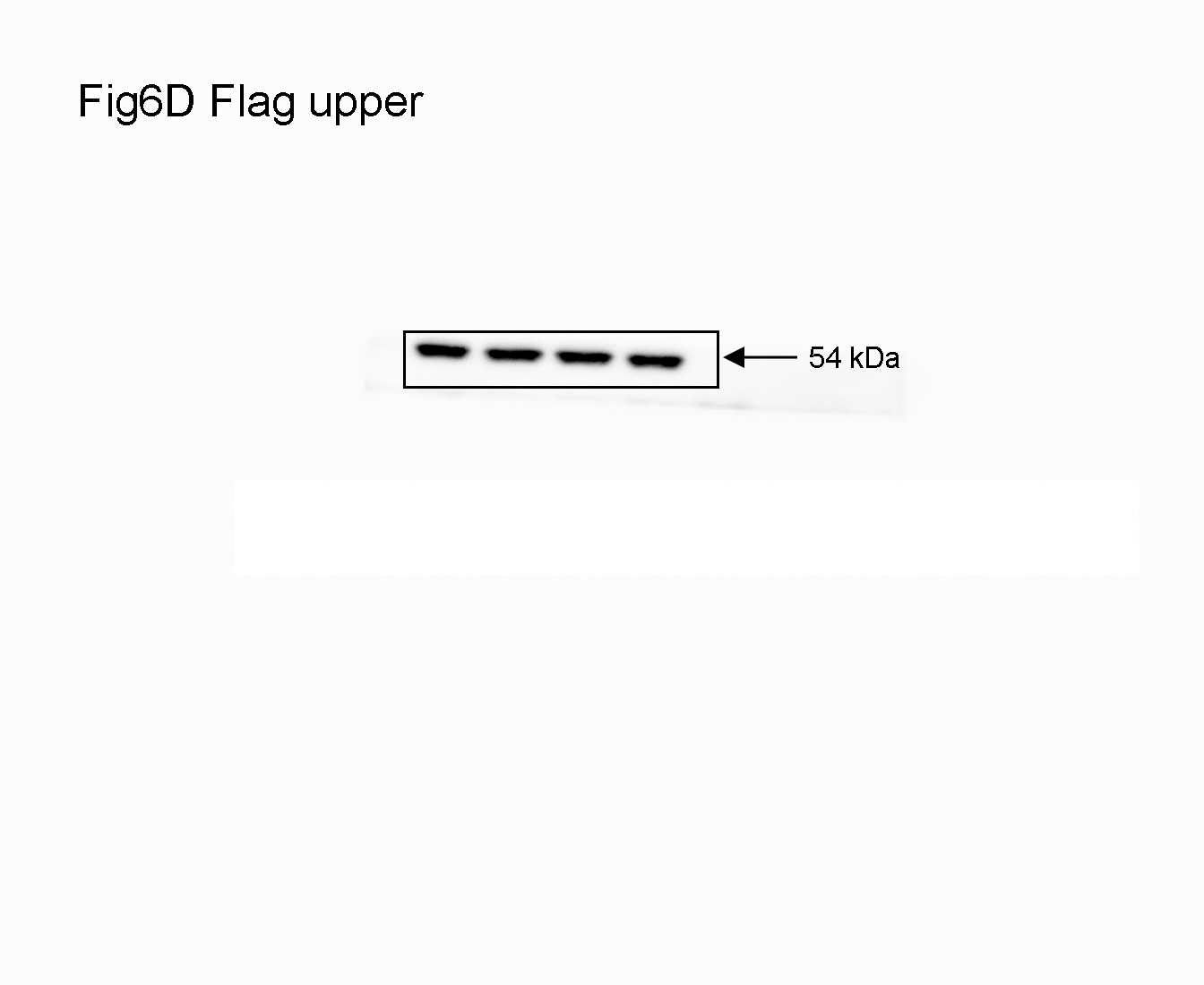

Supplement: Figure 6—source data 2. [file elife-98524-fig6-data2.zip › Fig 6-data2-v1/6D/Flag upper.tif]

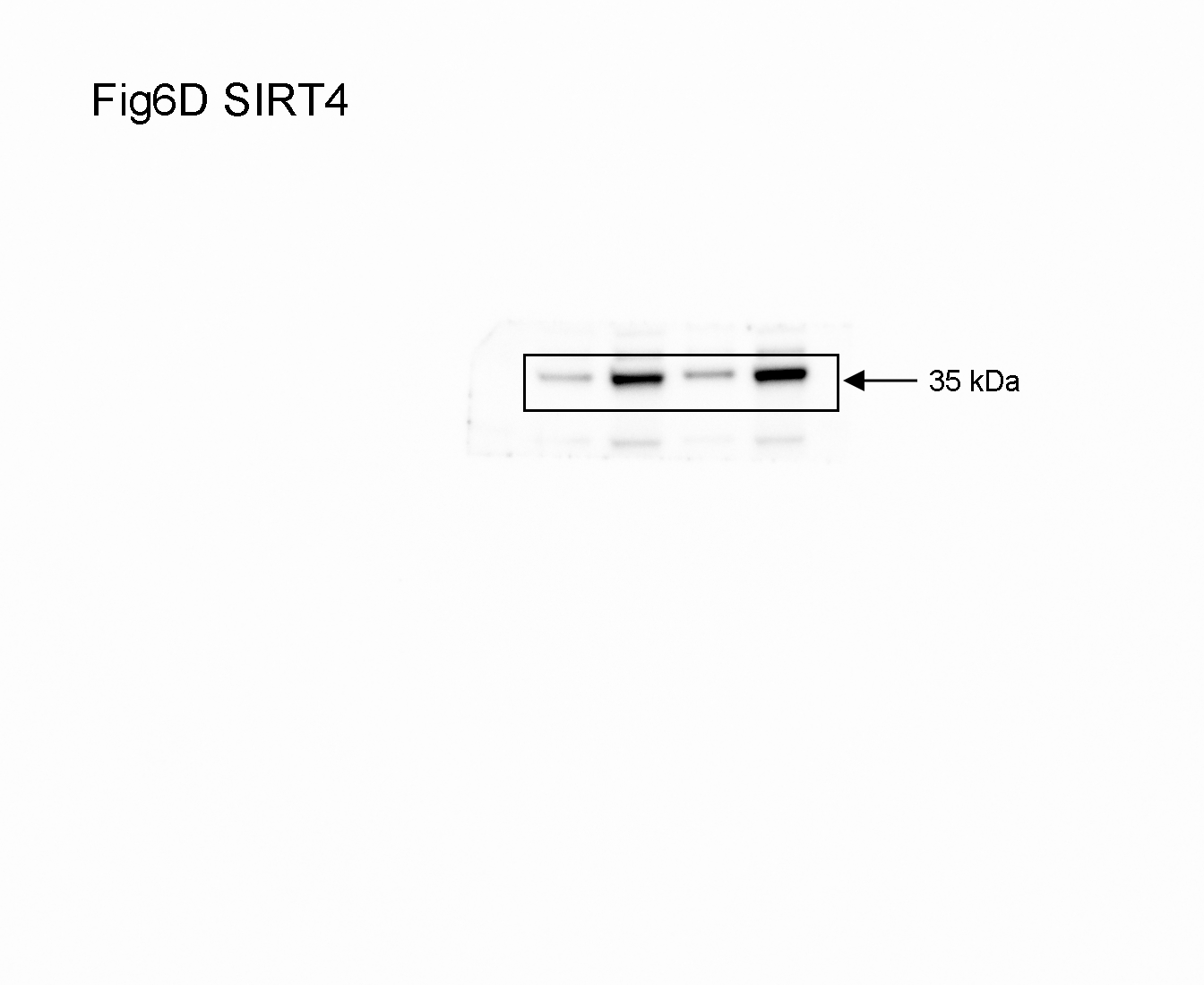

Supplement: Figure 6—source data 2. [file elife-98524-fig6-data2.zip › Fig 6-data2-v1/6D/SIRT4.tif]

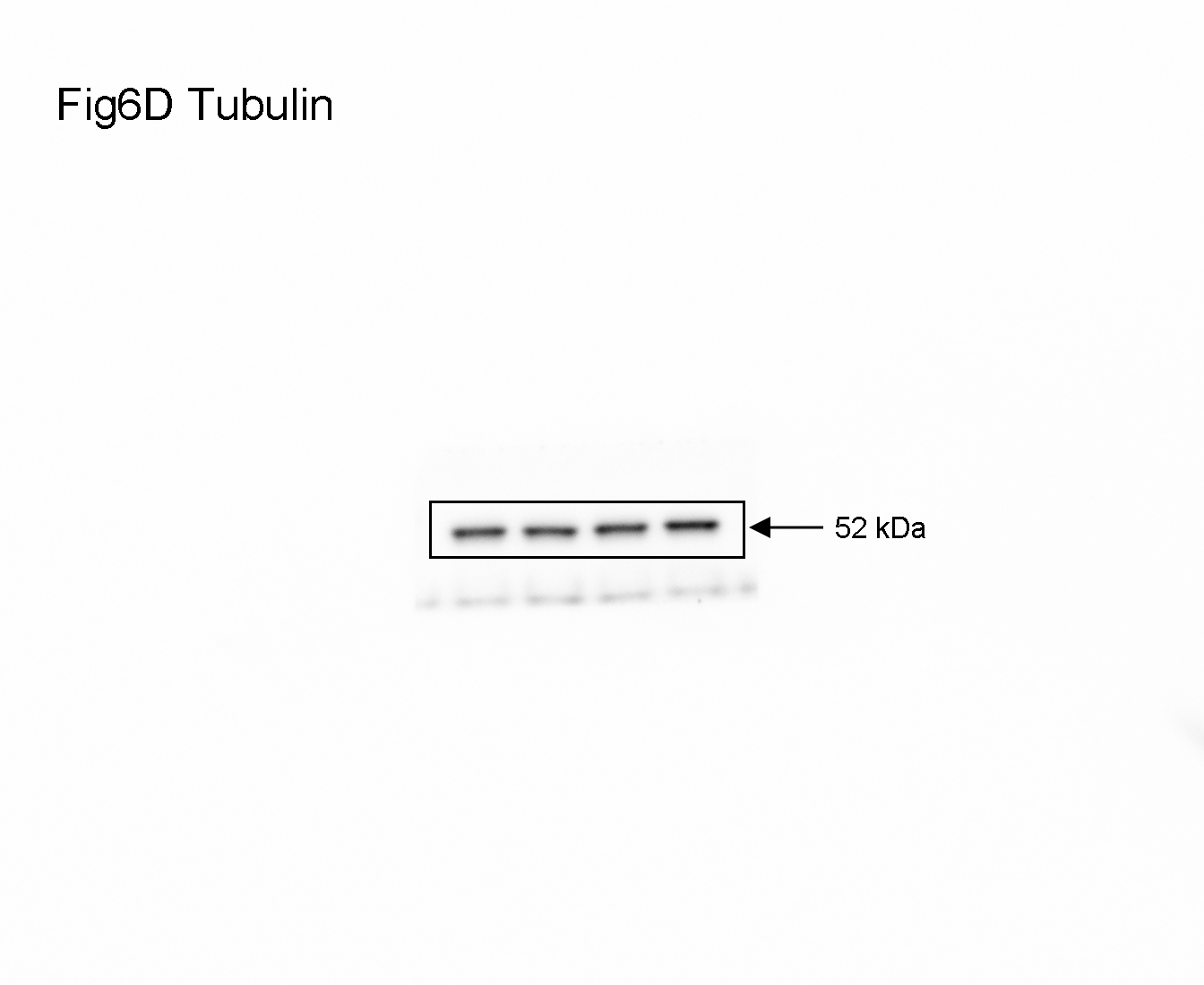

Supplement: Figure 6—source data 2. [file elife-98524-fig6-data2.zip › Fig 6-data2-v1/6D/Tubulin.tif]

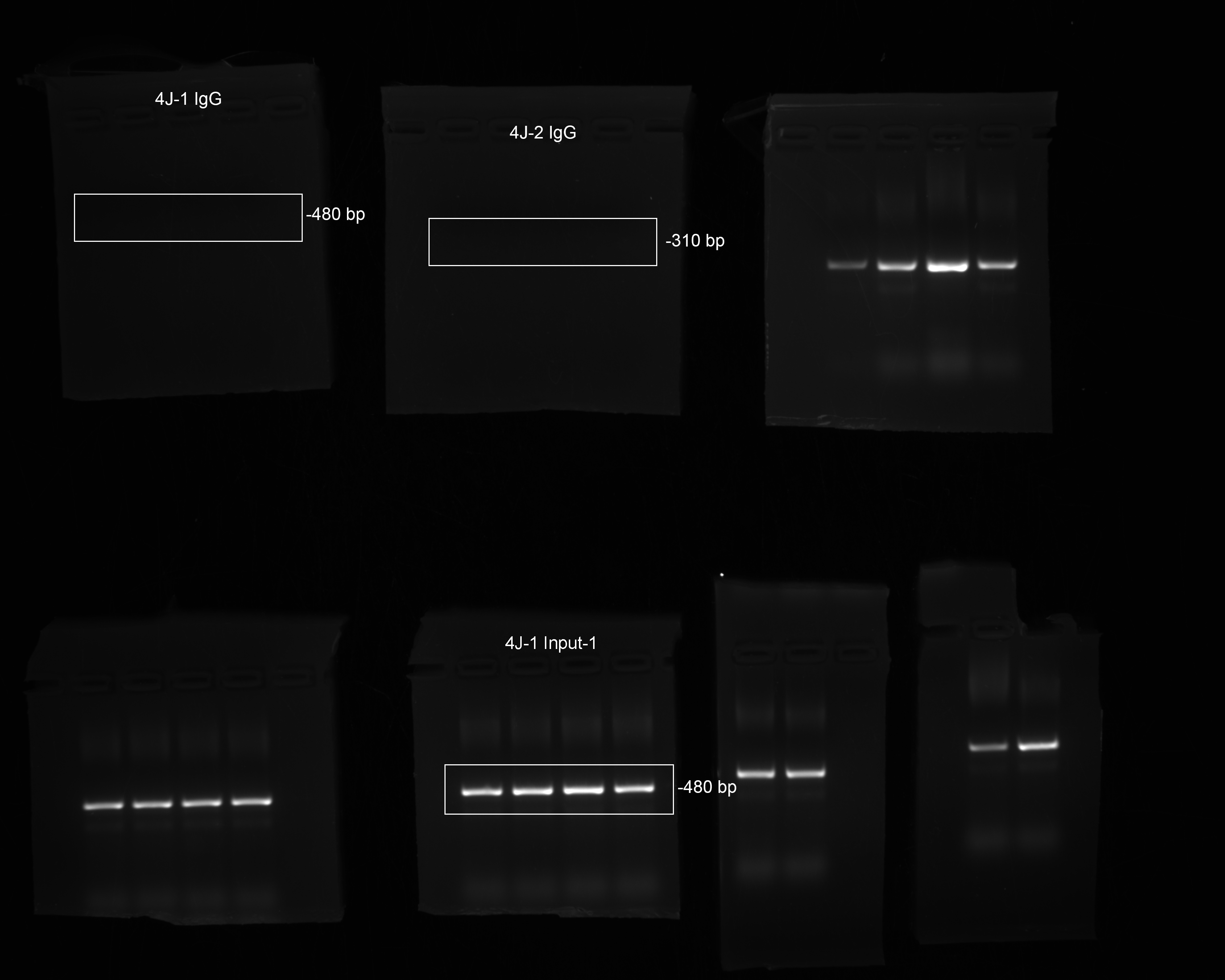

Supplement: Figure 6—source data 2. [file elife-98524-fig6-data2.zip › Fig 6-data2-v1/6J/4J-1 Input IgG+4J-2 IgG.tif]

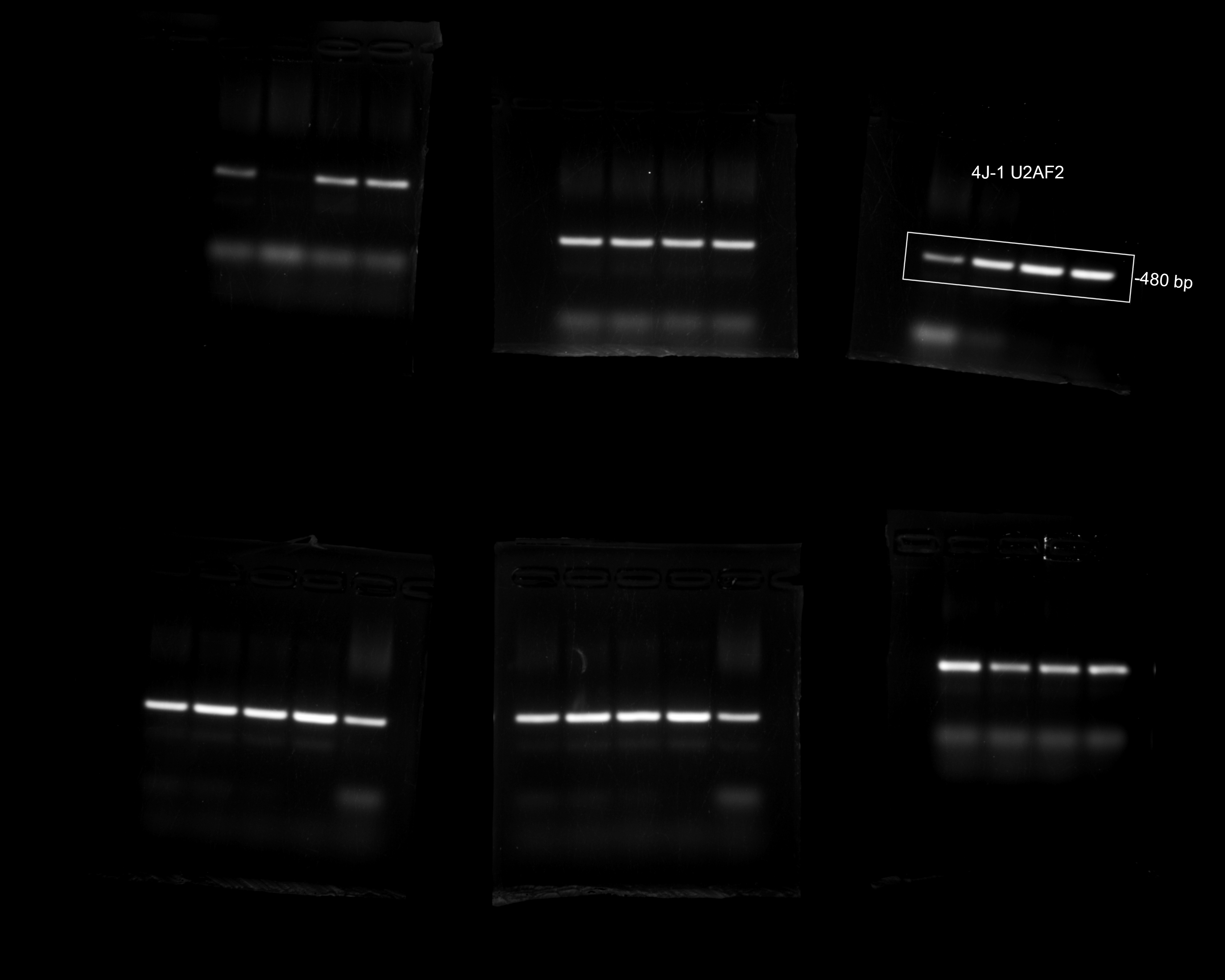

Supplement: Figure 6—source data 2. [file elife-98524-fig6-data2.zip › Fig 6-data2-v1/6J/4J-1 U2AF2.tif]

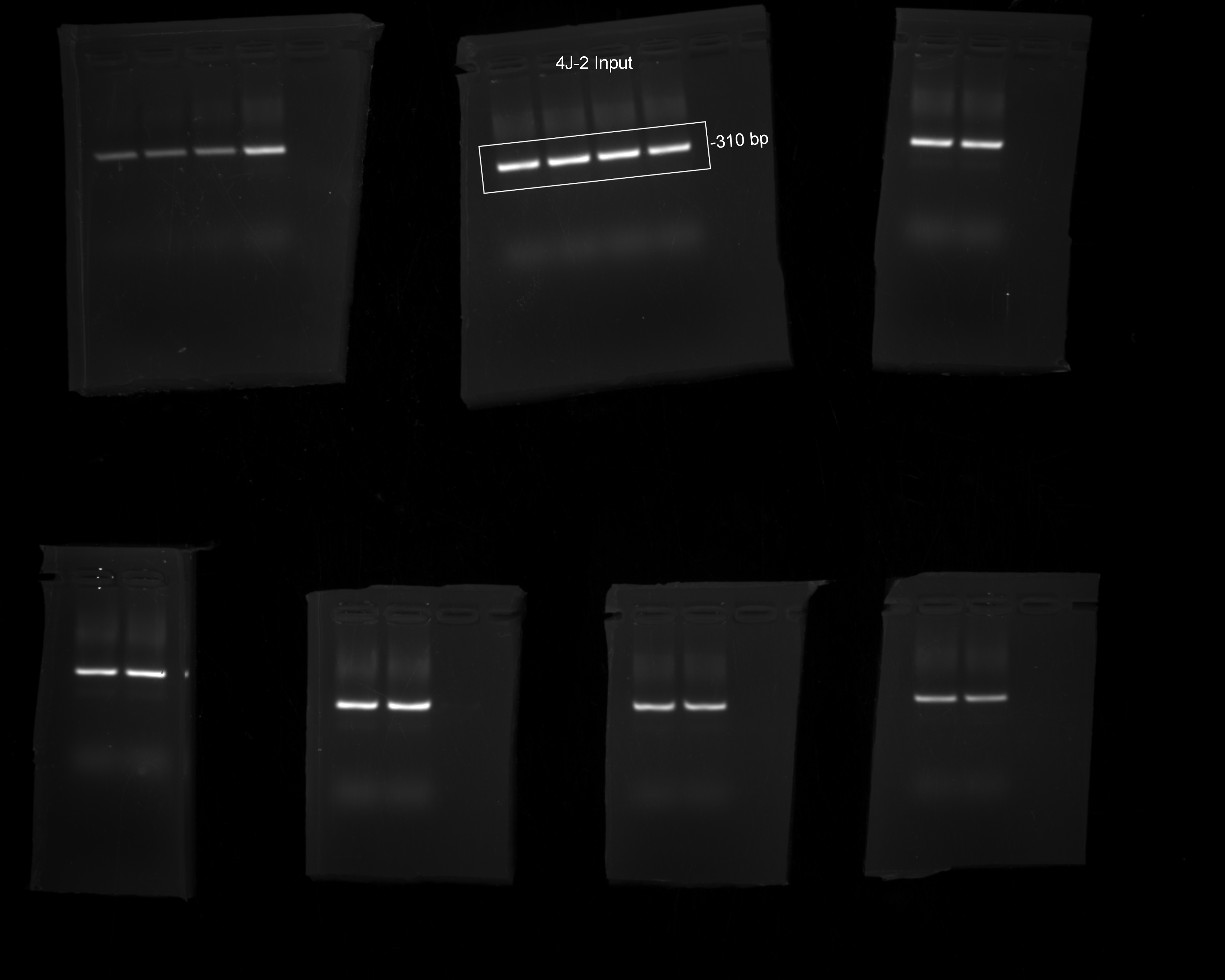

Supplement: Figure 6—source data 2. [file elife-98524-fig6-data2.zip › Fig 6-data2-v1/6J/4J-2 Input.tif]

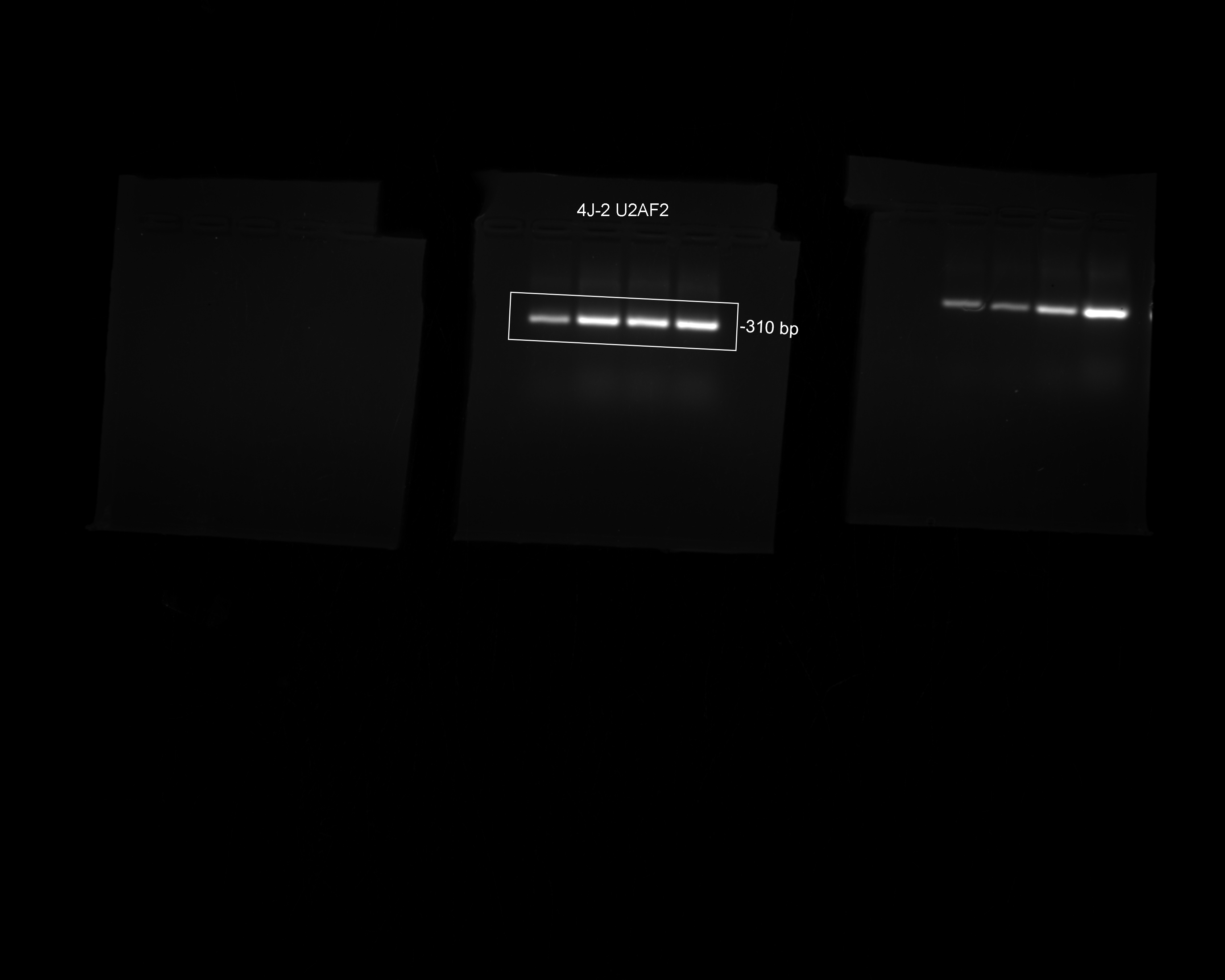

Supplement: Figure 6—source data 2. [file elife-98524-fig6-data2.zip › Fig 6-data2-v1/6J/4J-2 U2AF2.tif]

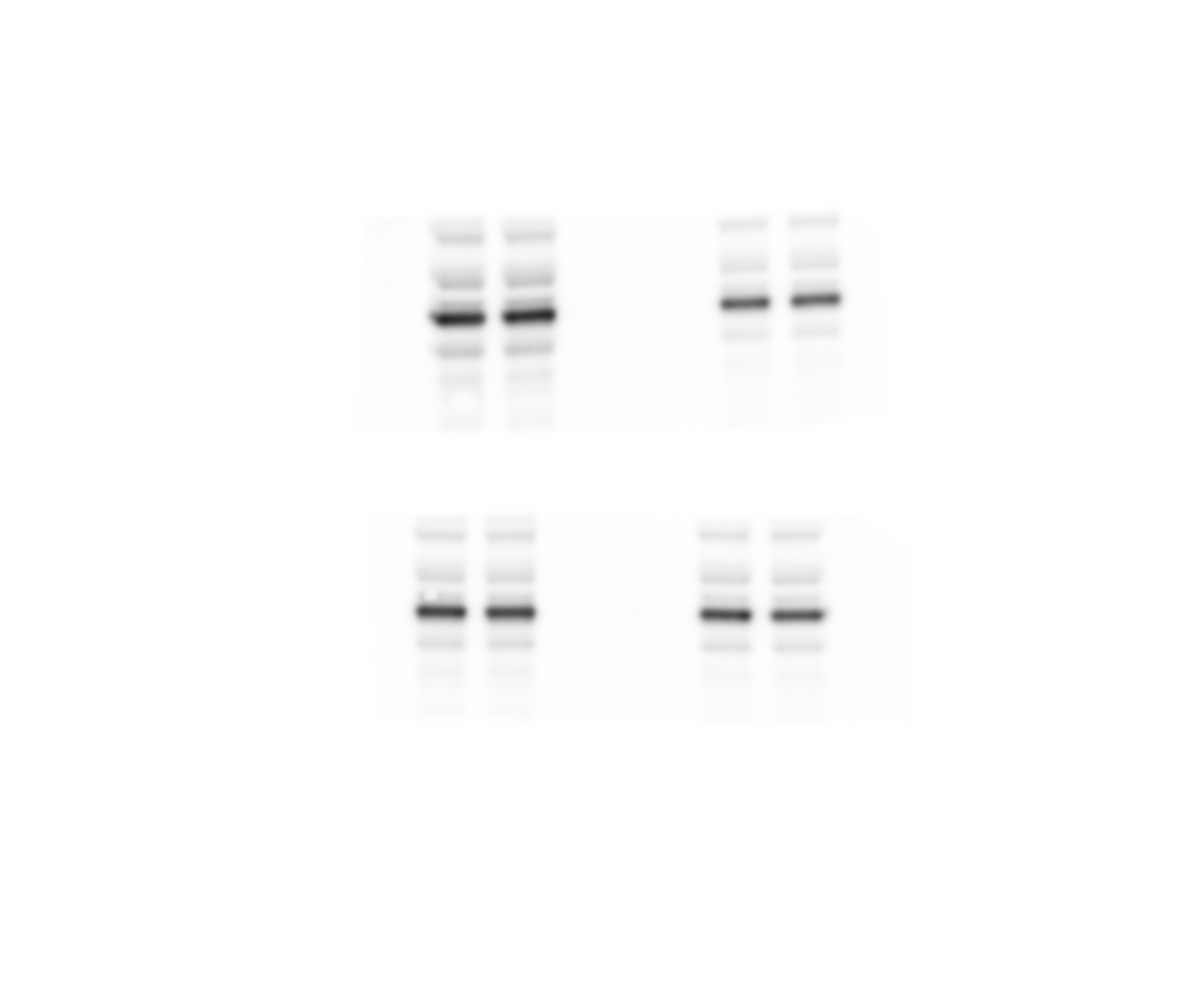

Supplement: Figure 7—source data 1. [file elife-98524-fig7-data1.zip › Fig 7-data1-v1/7A/COX IV.tif]

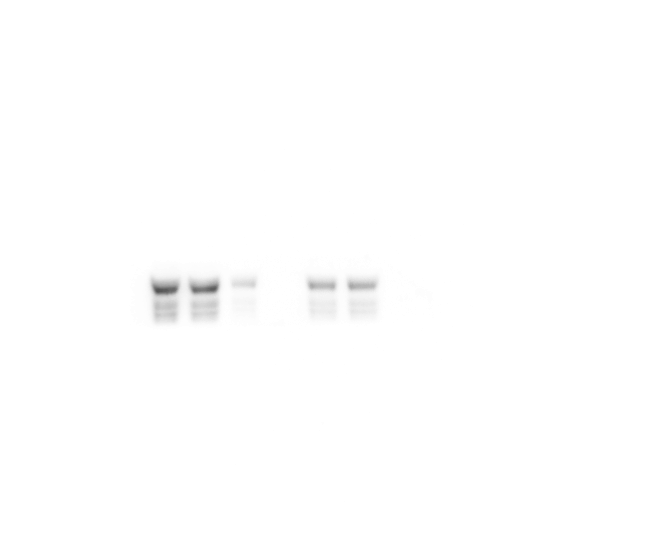

Supplement: Figure 7—source data 1. [file elife-98524-fig7-data1.zip › Fig 7-data1-v1/7A/SIRT4.tif]

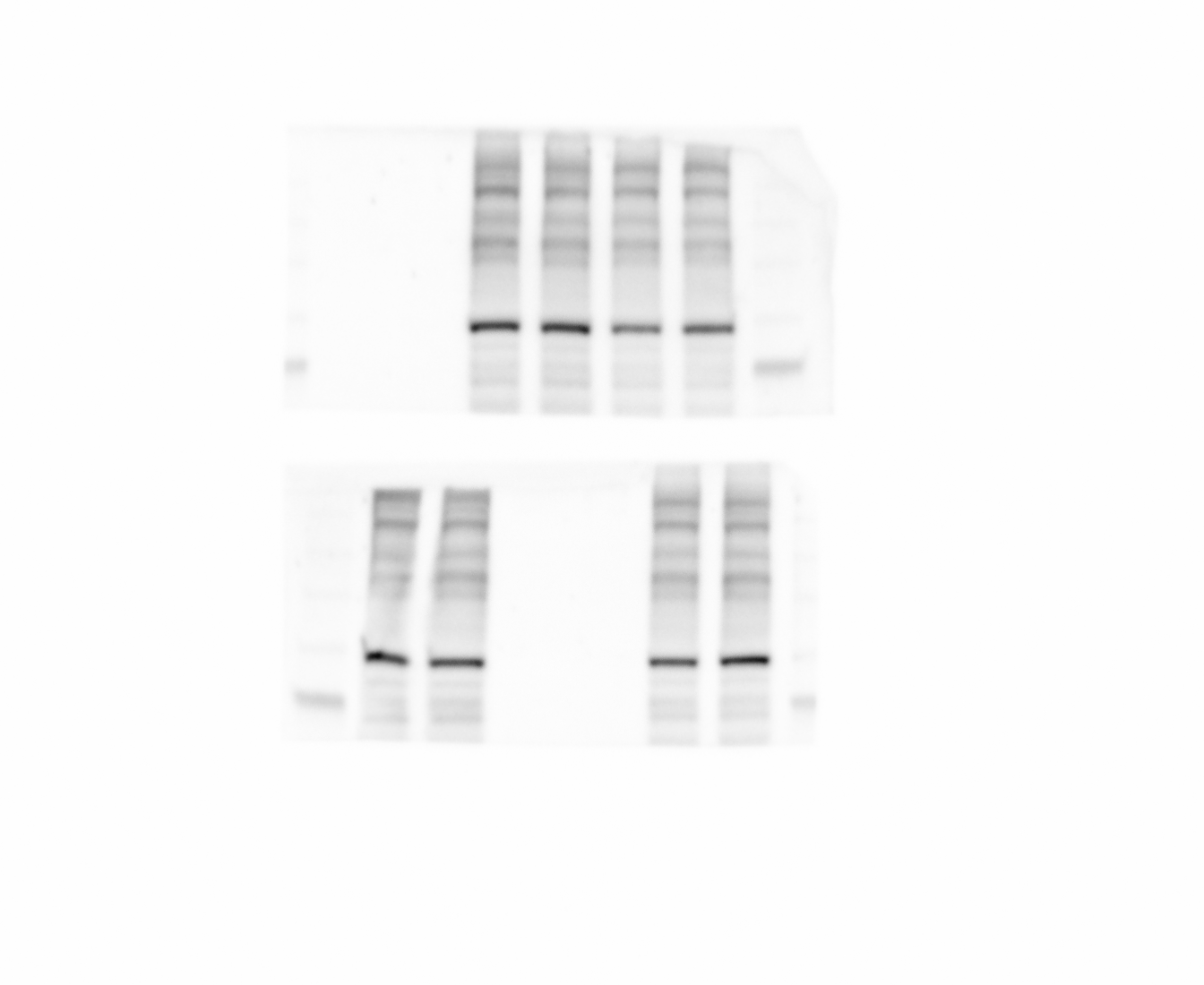

Supplement: Figure 7—source data 1. [file elife-98524-fig7-data1.zip › Fig 7-data1-v1/7A/Tubulin.tif]

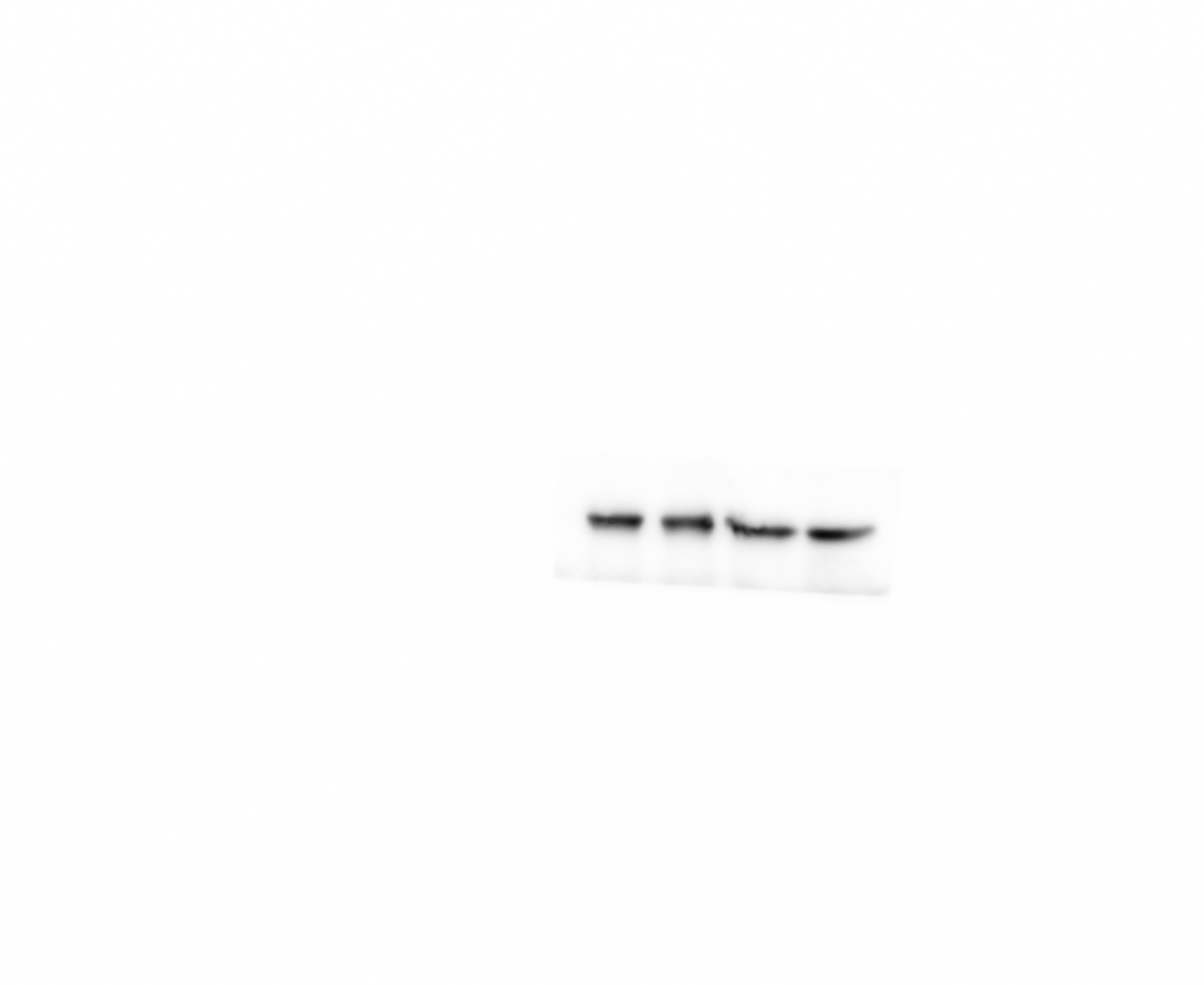

Supplement: Figure 7—source data 1. [file elife-98524-fig7-data1.zip › Fig 7-data1-v1/7B/left/COX IV.tif]

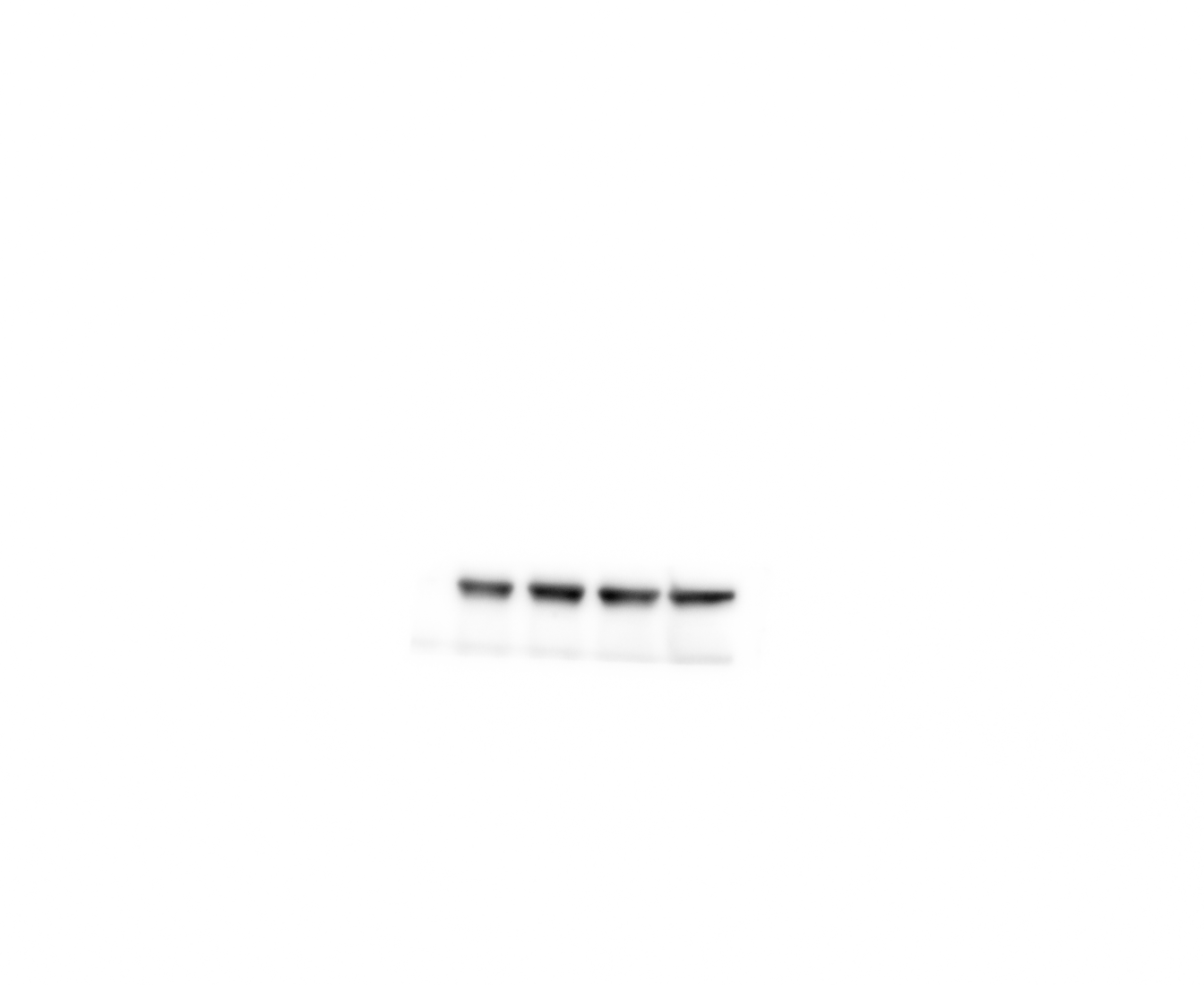

Supplement: Figure 7—source data 1. [file elife-98524-fig7-data1.zip › Fig 7-data1-v1/7B/left/SIRT4 left.tif]

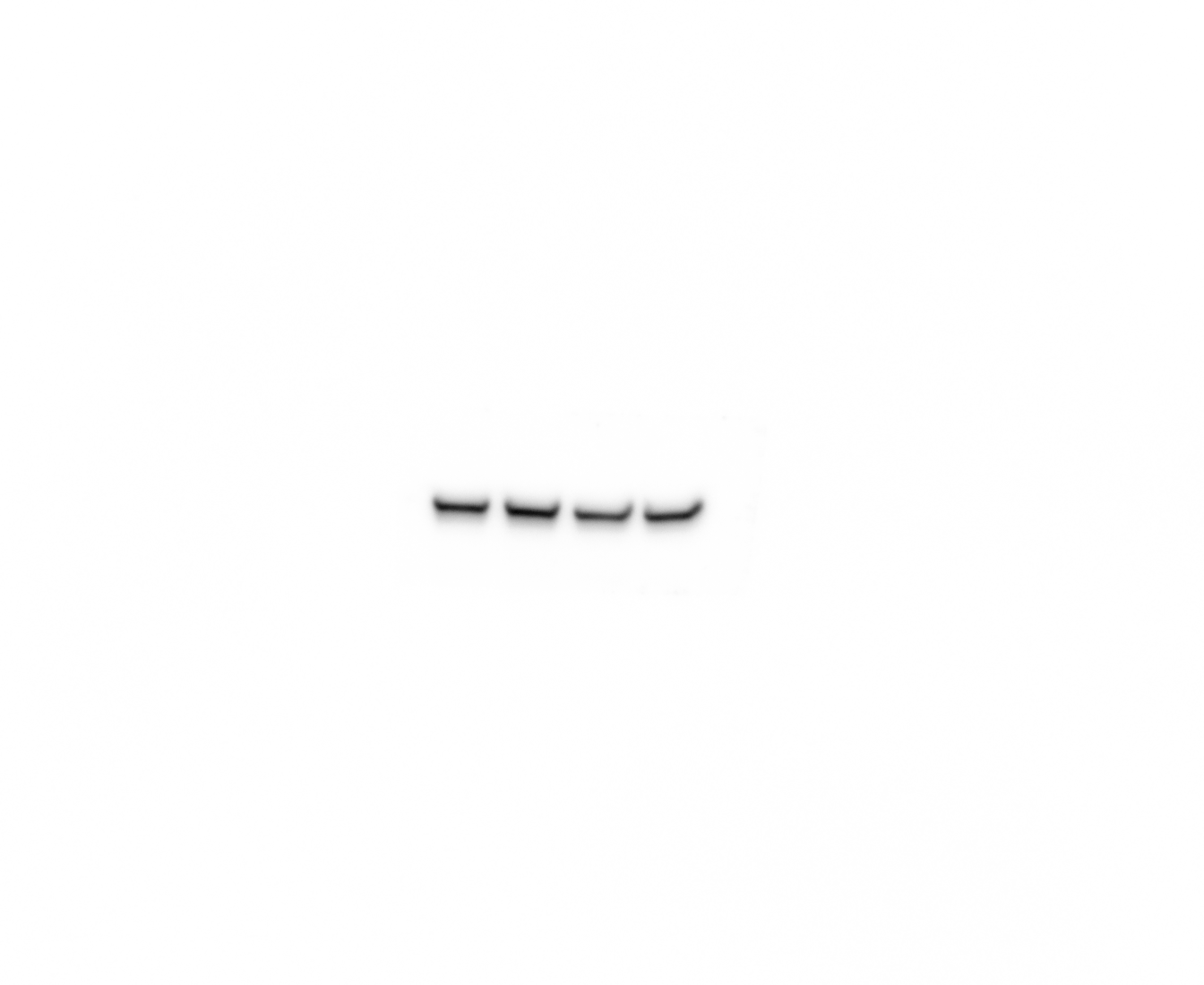

Supplement: Figure 7—source data 1. [file elife-98524-fig7-data1.zip › Fig 7-data1-v1/7B/left/Tubulin left.tif]

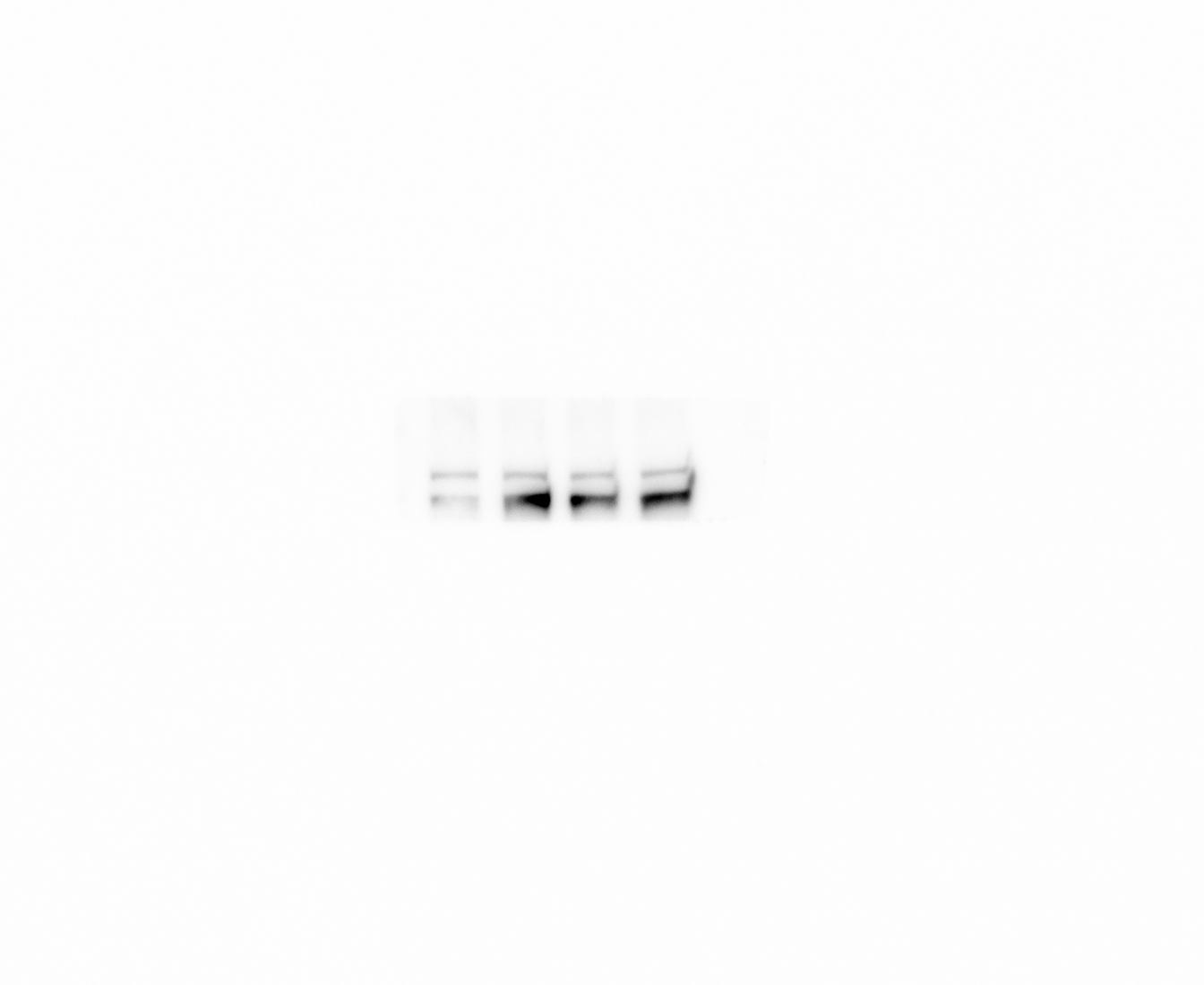

Supplement: Figure 7—source data 1. [file elife-98524-fig7-data1.zip › Fig 7-data1-v1/7B/right/SIRT4 right.tif]

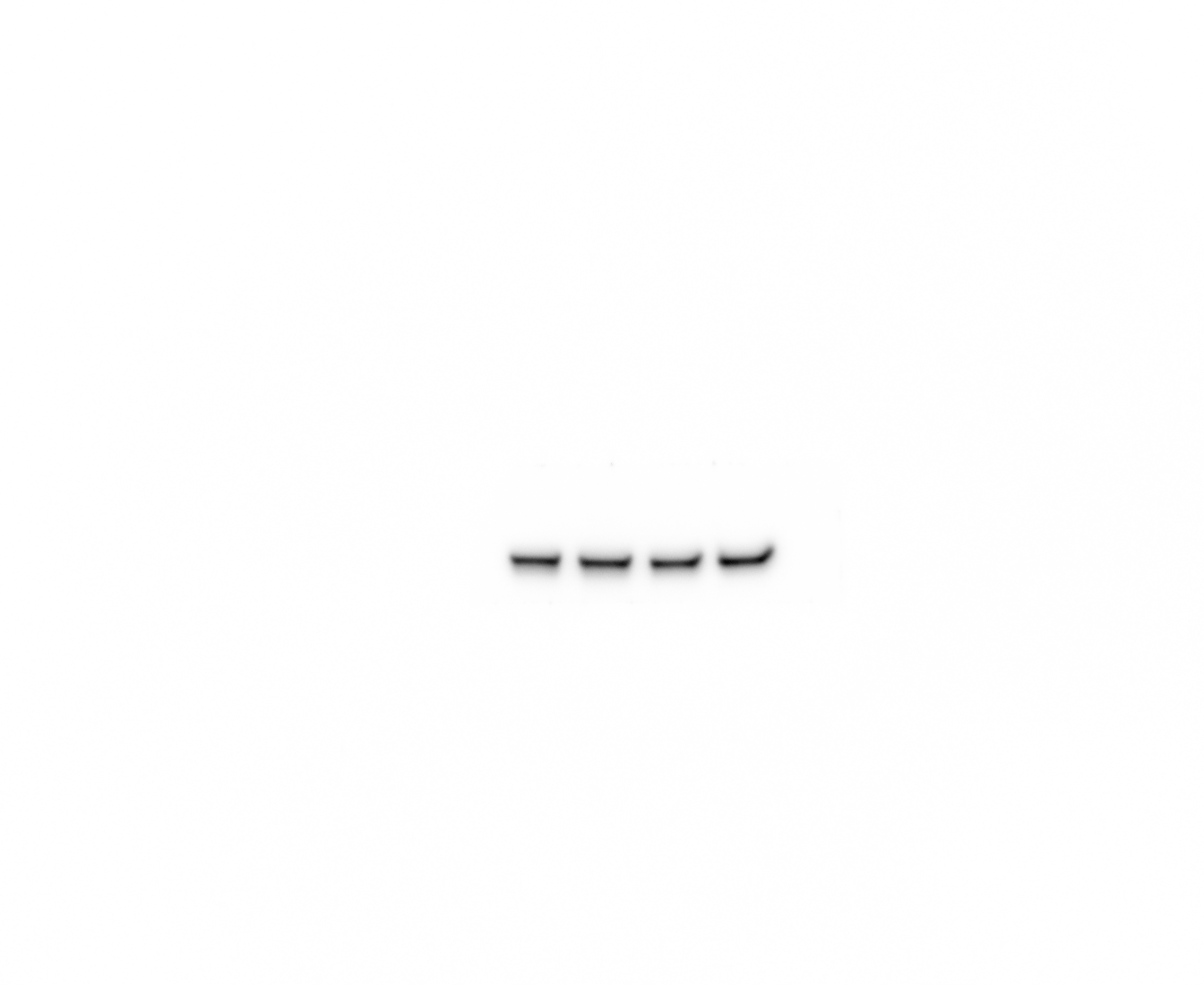

Supplement: Figure 7—source data 1. [file elife-98524-fig7-data1.zip › Fig 7-data1-v1/7B/right/Tubulin right.tif]

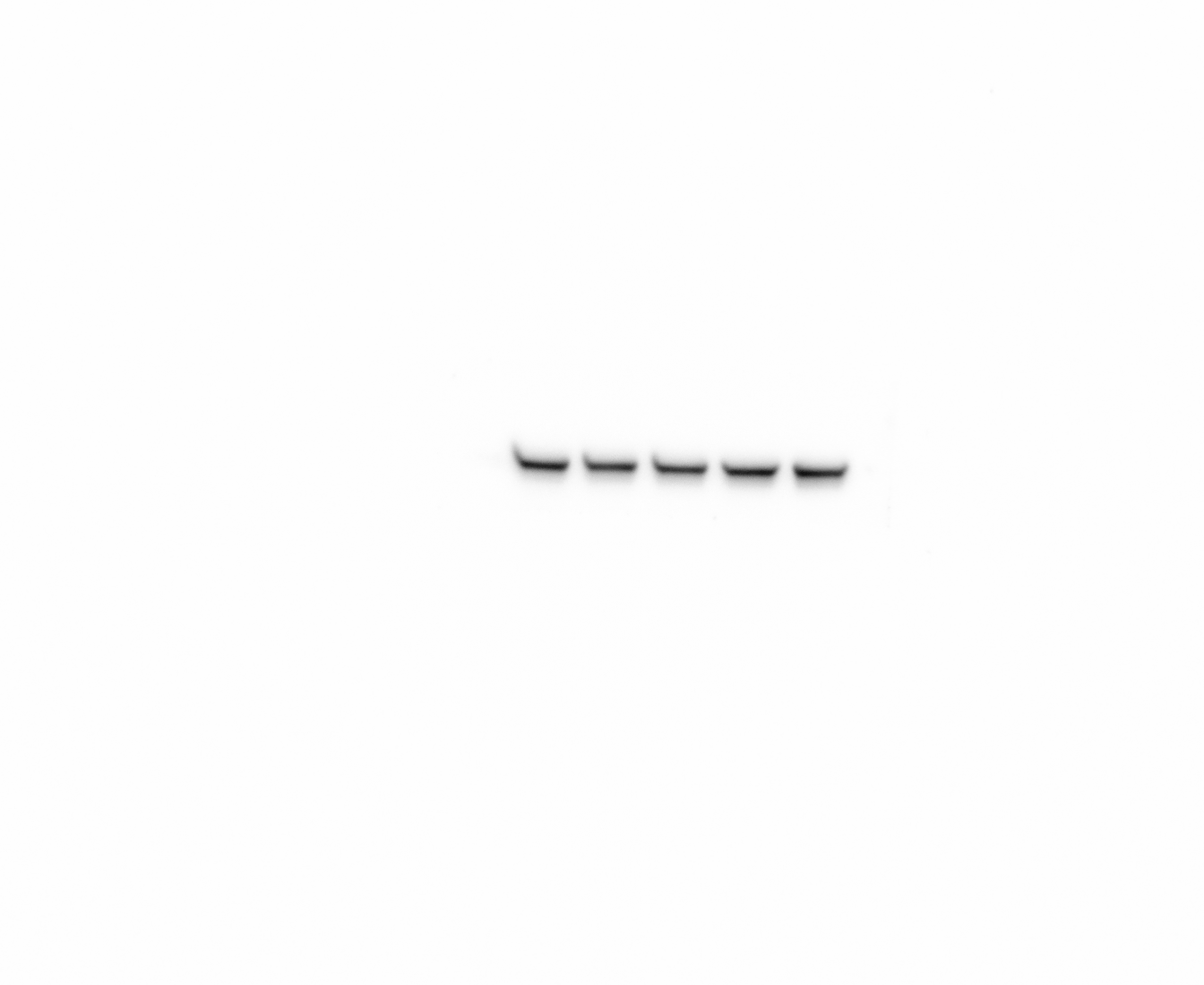

Supplement: Figure 7—source data 1. [file elife-98524-fig7-data1.zip › Fig 7-data1-v1/7D/bottom/COX IV bottom.tif]

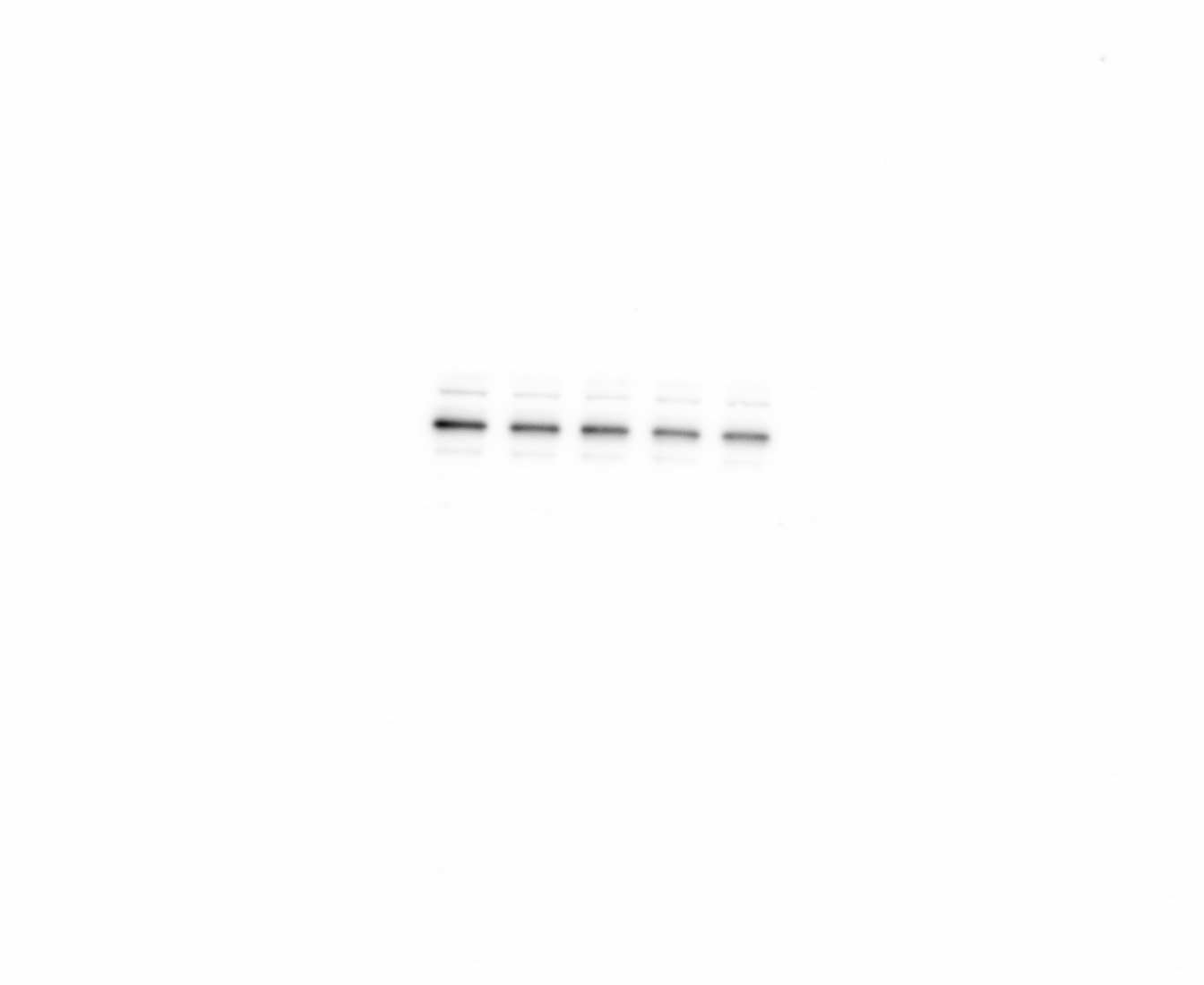

Supplement: Figure 7—source data 1. [file elife-98524-fig7-data1.zip › Fig 7-data1-v1/7D/bottom/SIRT4 bottom.tif]

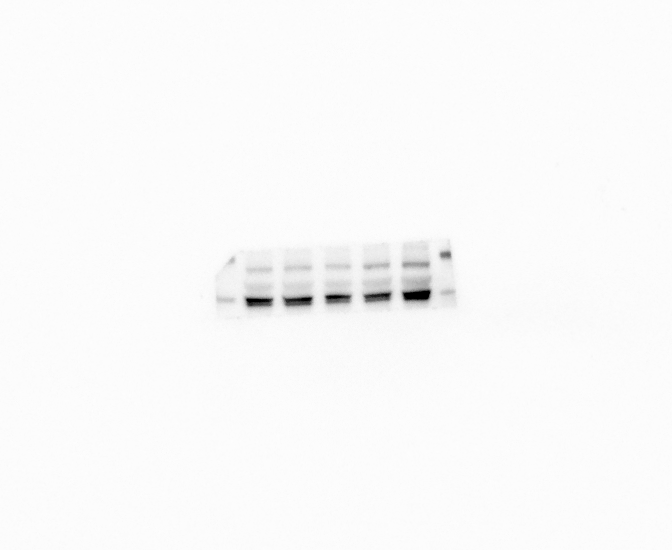

Supplement: Figure 7—source data 1. [file elife-98524-fig7-data1.zip › Fig 7-data1-v1/7D/middle/COX IV middle.tif]

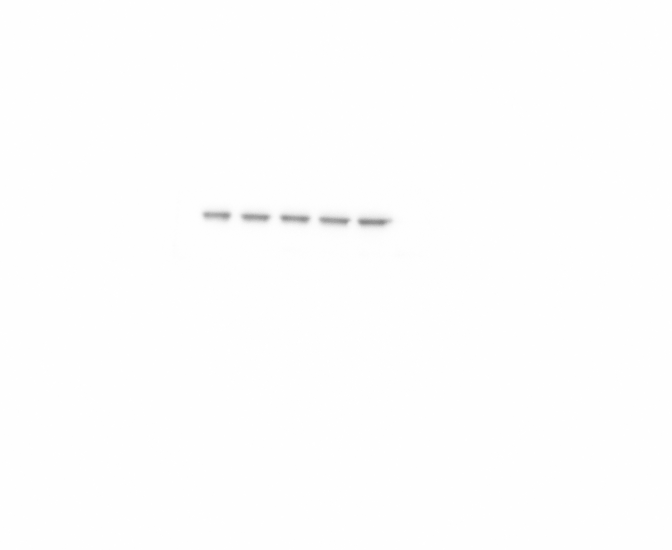

Supplement: Figure 7—source data 1. [file elife-98524-fig7-data1.zip › Fig 7-data1-v1/7D/middle/SIRT4 middle.tif]

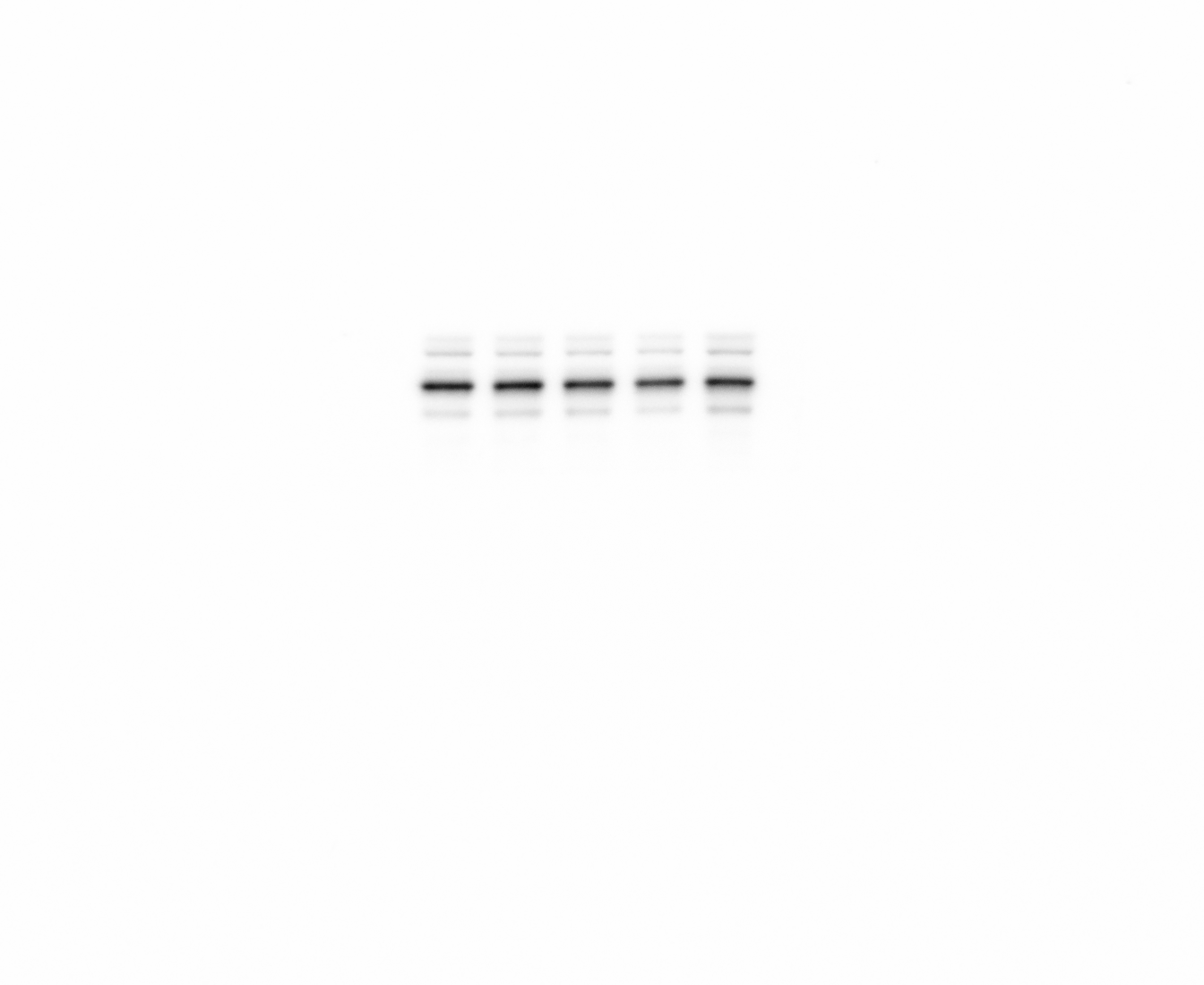

Supplement: Figure 7—source data 1. [file elife-98524-fig7-data1.zip › Fig 7-data1-v1/7D/middle/Tubulin middle.tif]

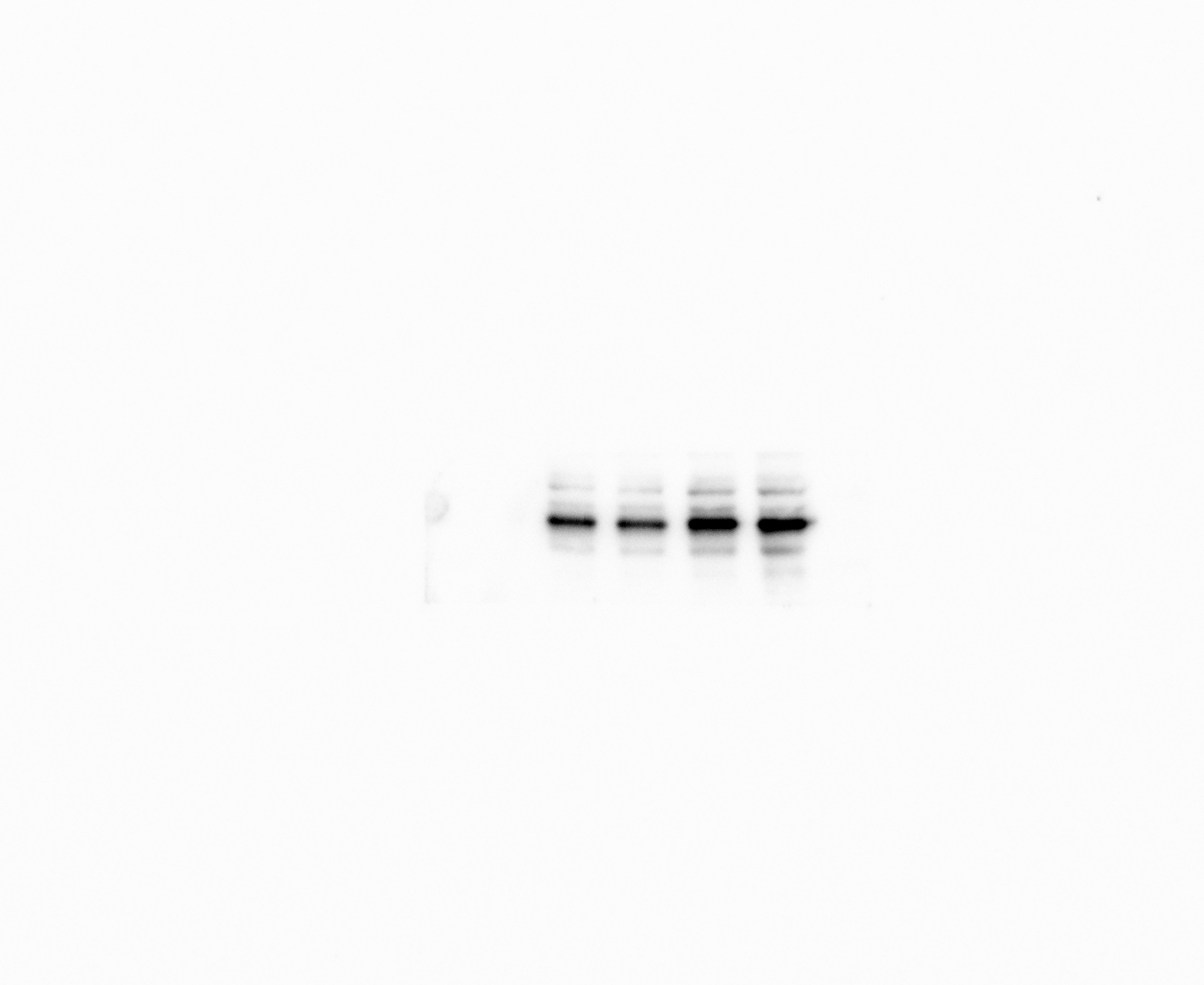

Supplement: Figure 7—source data 1. [file elife-98524-fig7-data1.zip › Fig 7-data1-v1/7D/upper/SIRT4 upper.tif]

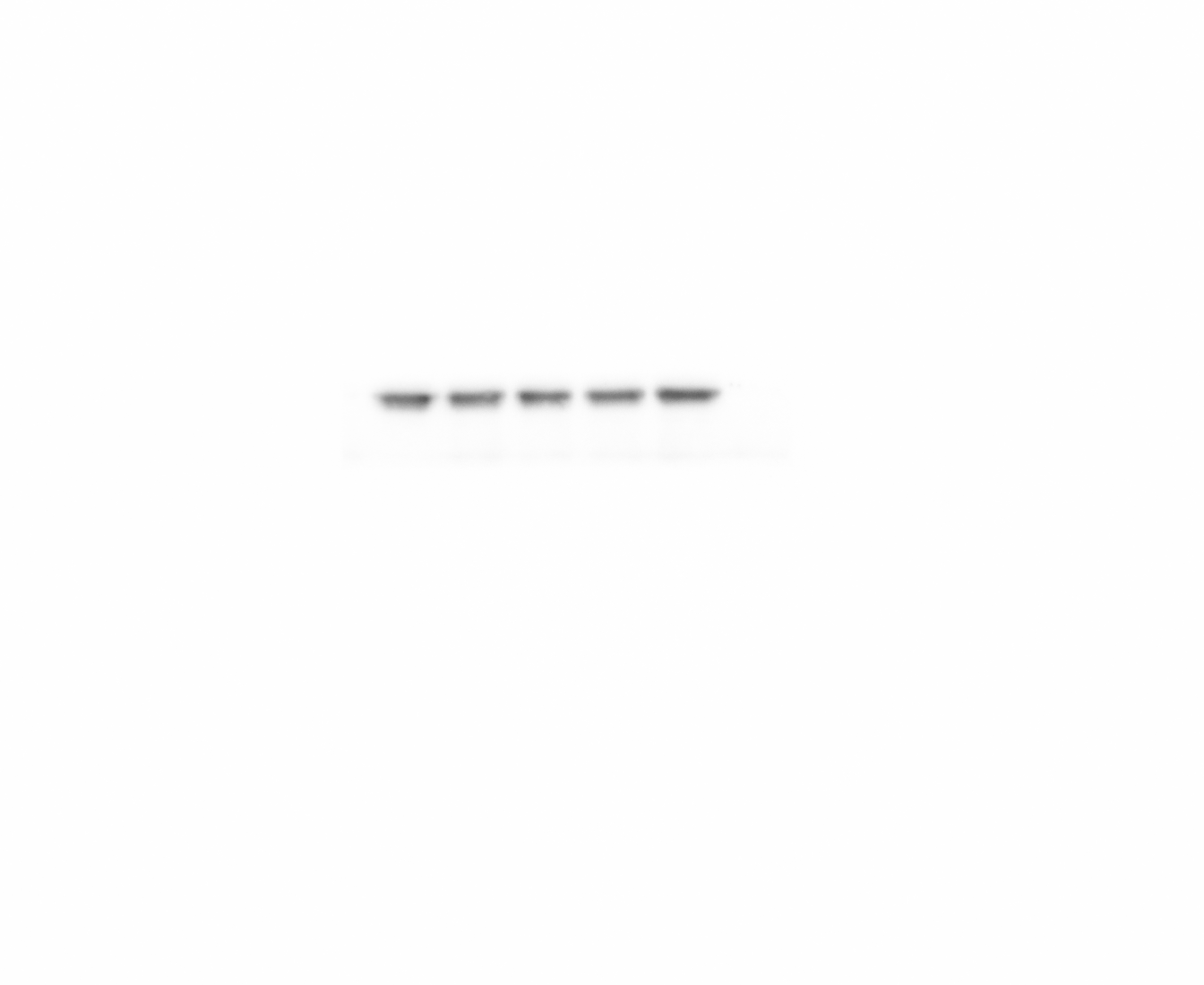

Supplement: Figure 7—source data 1. [file elife-98524-fig7-data1.zip › Fig 7-data1-v1/7D/upper/Tubulin upper.tif]

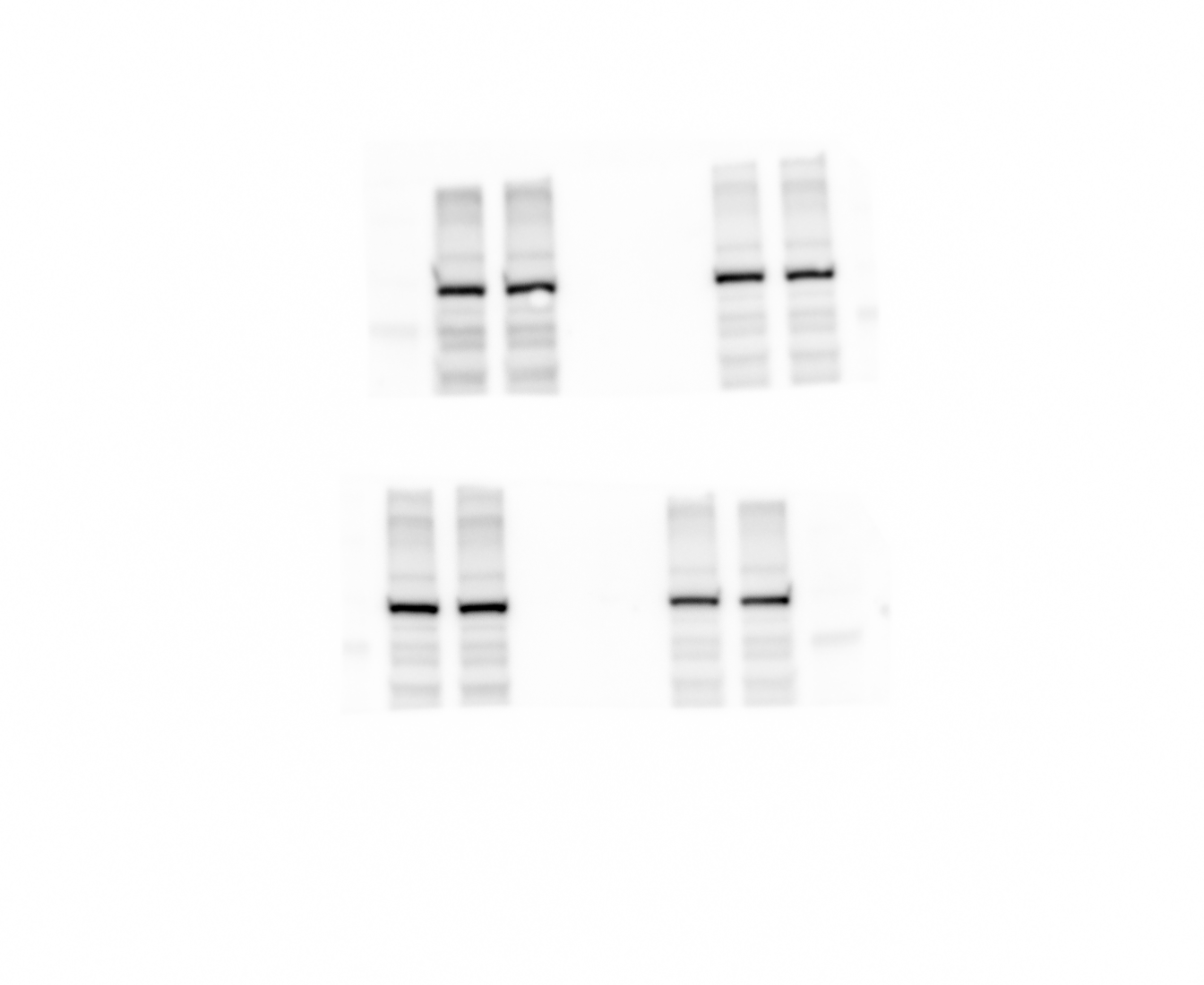

Supplement: Figure 7—source data 1. [file elife-98524-fig7-data1.zip › Fig 7-data1-v1/7E/left/COX IV bottom.tif]

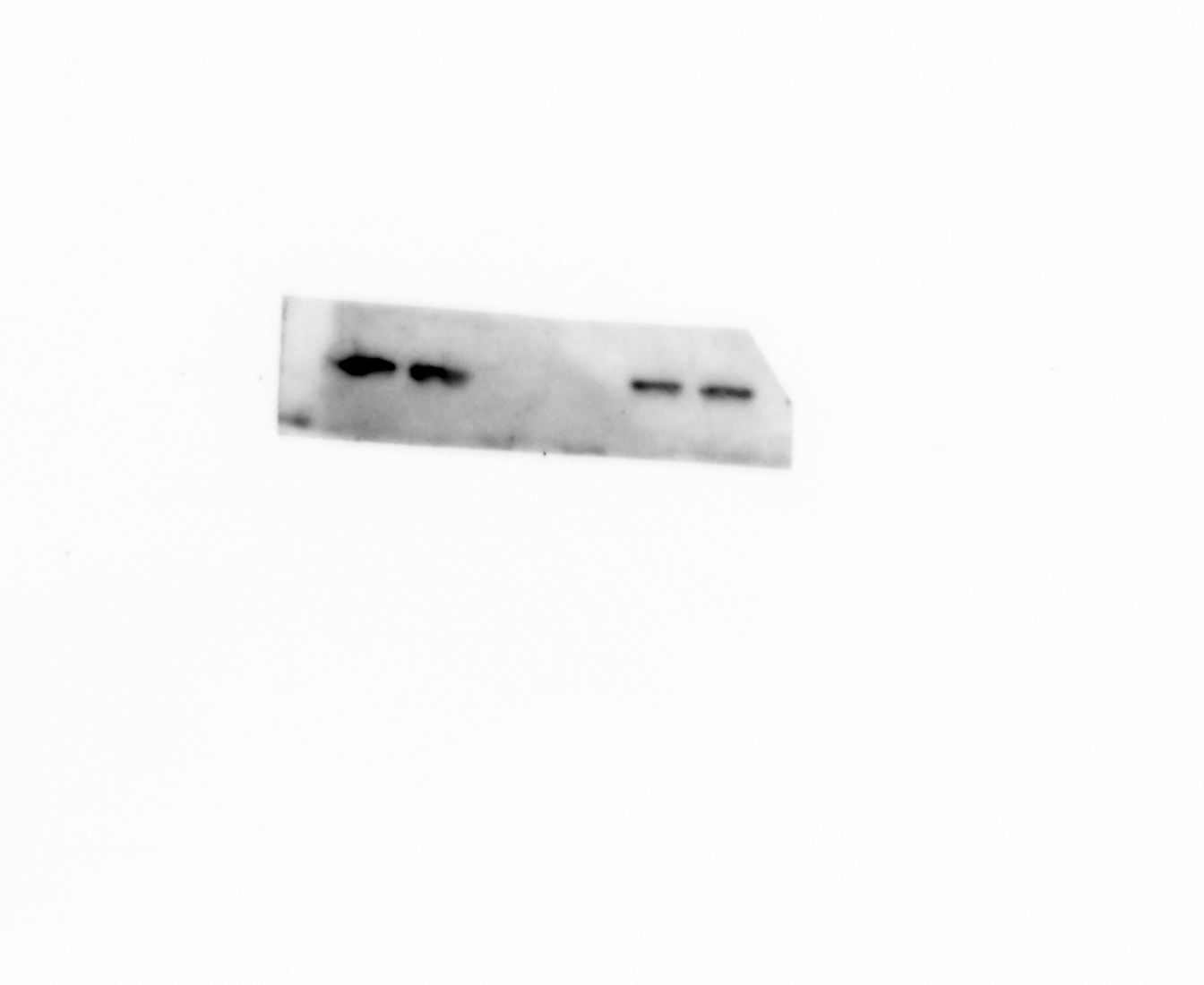

Supplement: Figure 7—source data 1. [file elife-98524-fig7-data1.zip › Fig 7-data1-v1/7E/left/COX IV upper.tif]

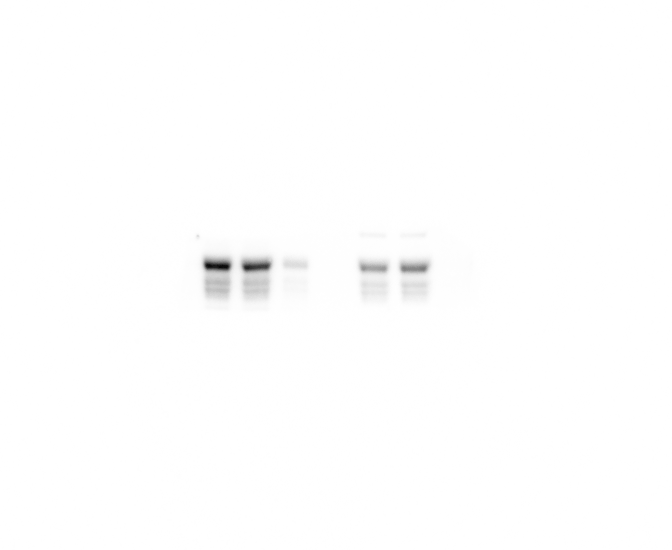

Supplement: Figure 7—source data 1. [file elife-98524-fig7-data1.zip › Fig 7-data1-v1/7E/left/SIRT4 bottom.tif]

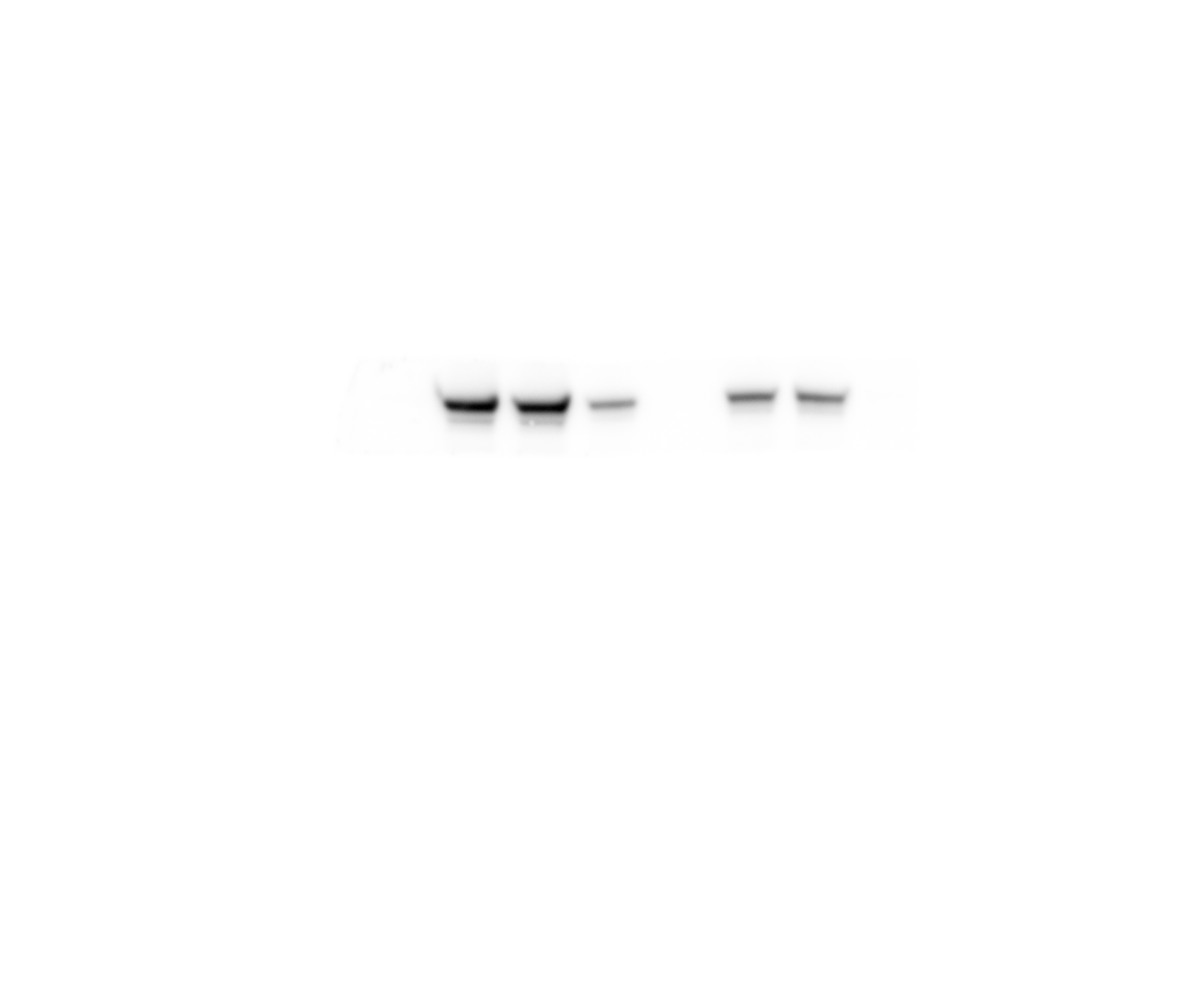

Supplement: Figure 7—source data 1. [file elife-98524-fig7-data1.zip › Fig 7-data1-v1/7E/left/SIRT4 upper.tif]

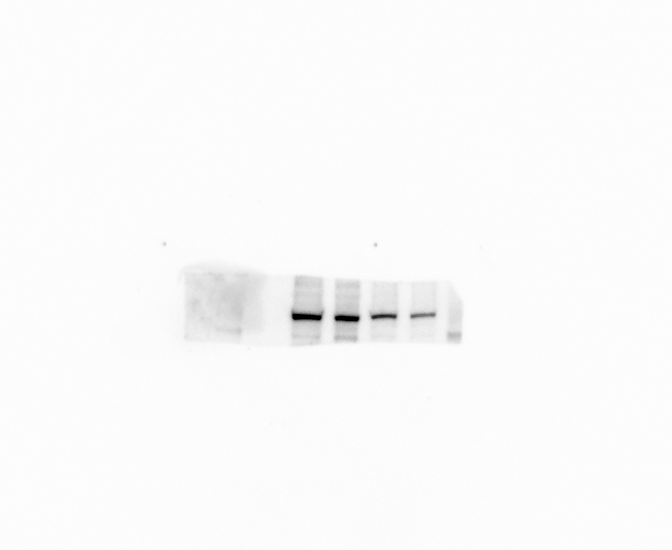

Supplement: Figure 7—source data 1. [file elife-98524-fig7-data1.zip › Fig 7-data1-v1/7E/left/Tubulin bottom left.tif]

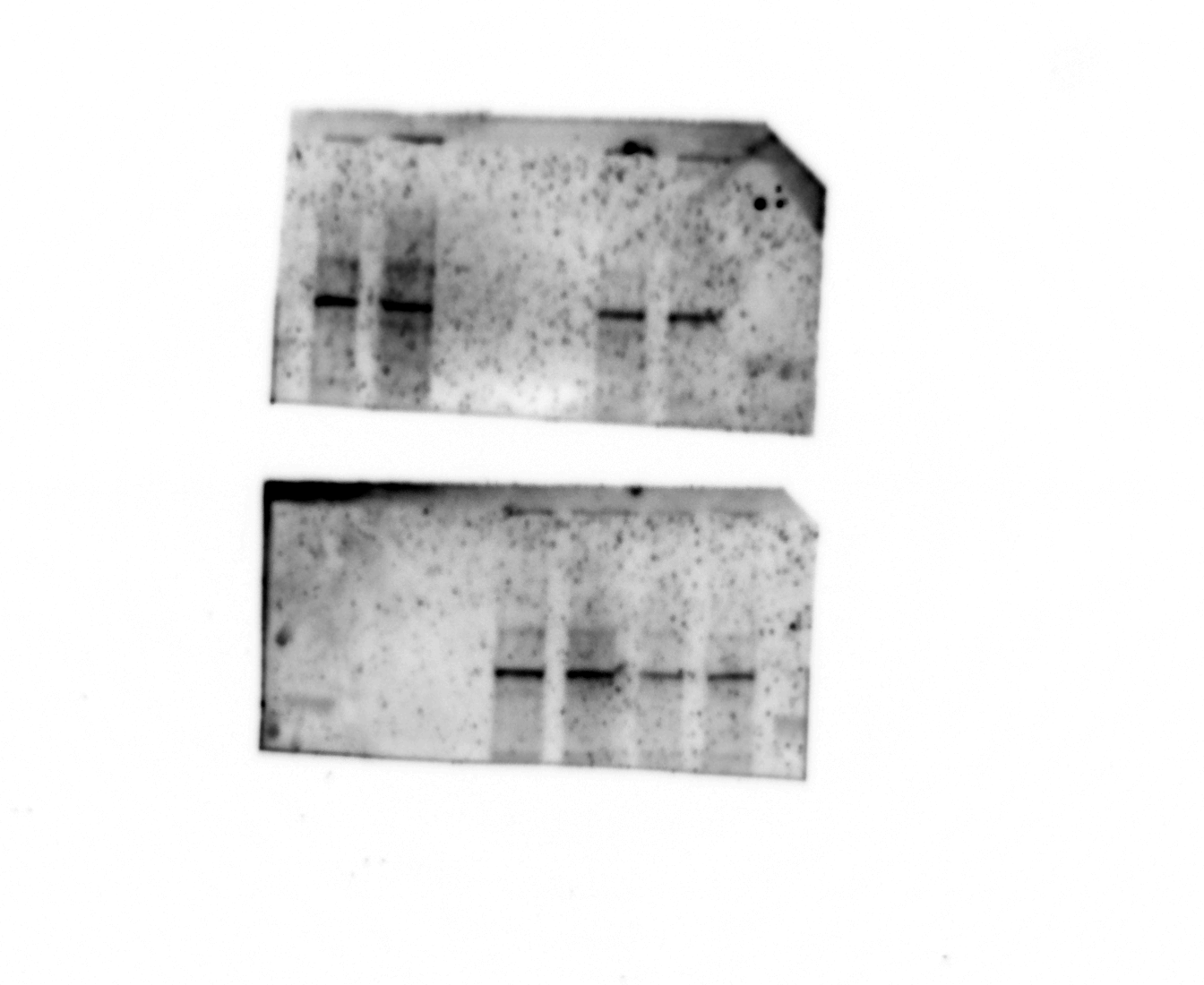

Supplement: Figure 7—source data 1. [file elife-98524-fig7-data1.zip › Fig 7-data1-v1/7E/left/Tubulin upper left.tif]

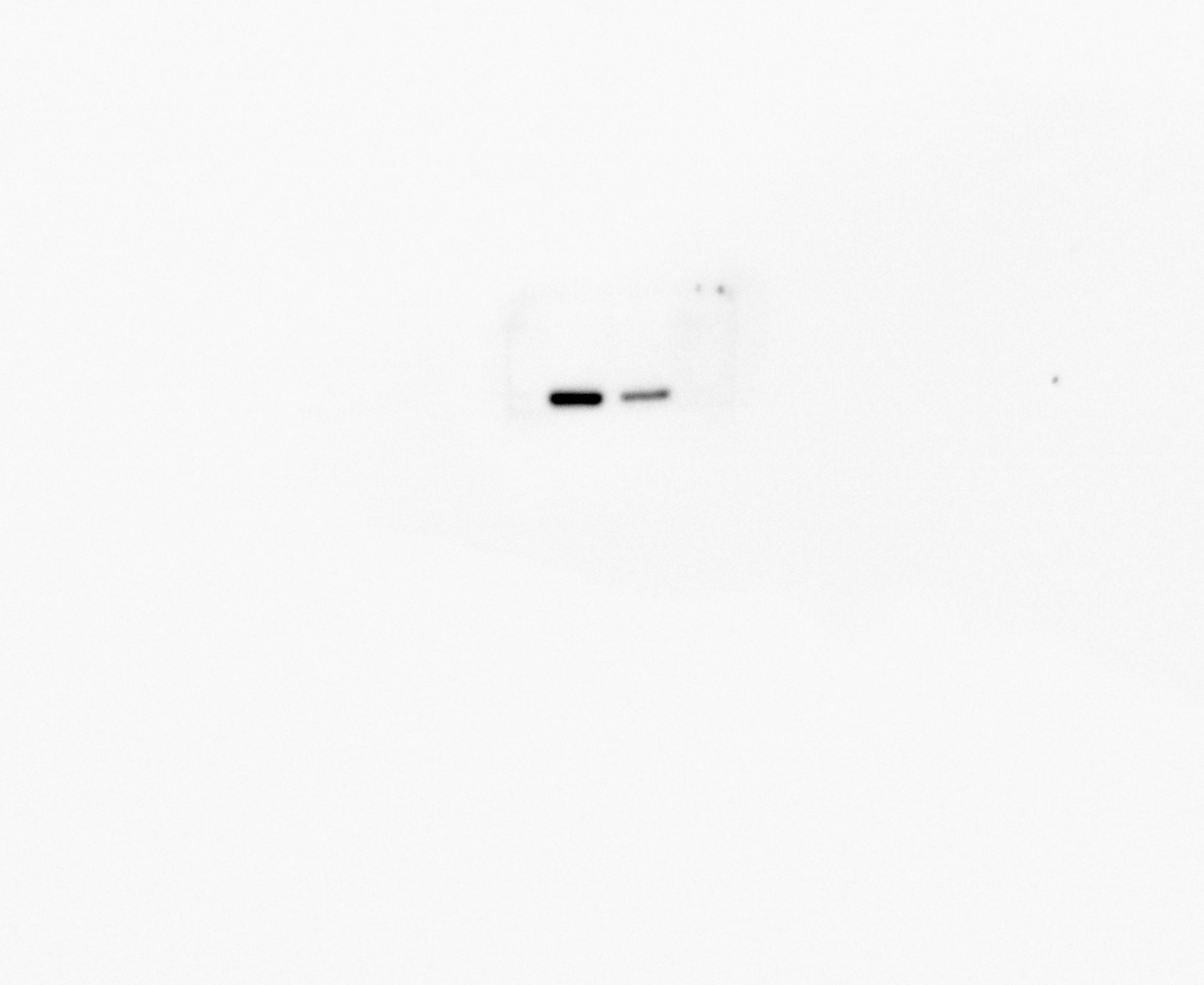

Supplement: Figure 7—source data 1. [file elife-98524-fig7-data1.zip › Fig 7-data1-v1/7E/right/BAK.tif]

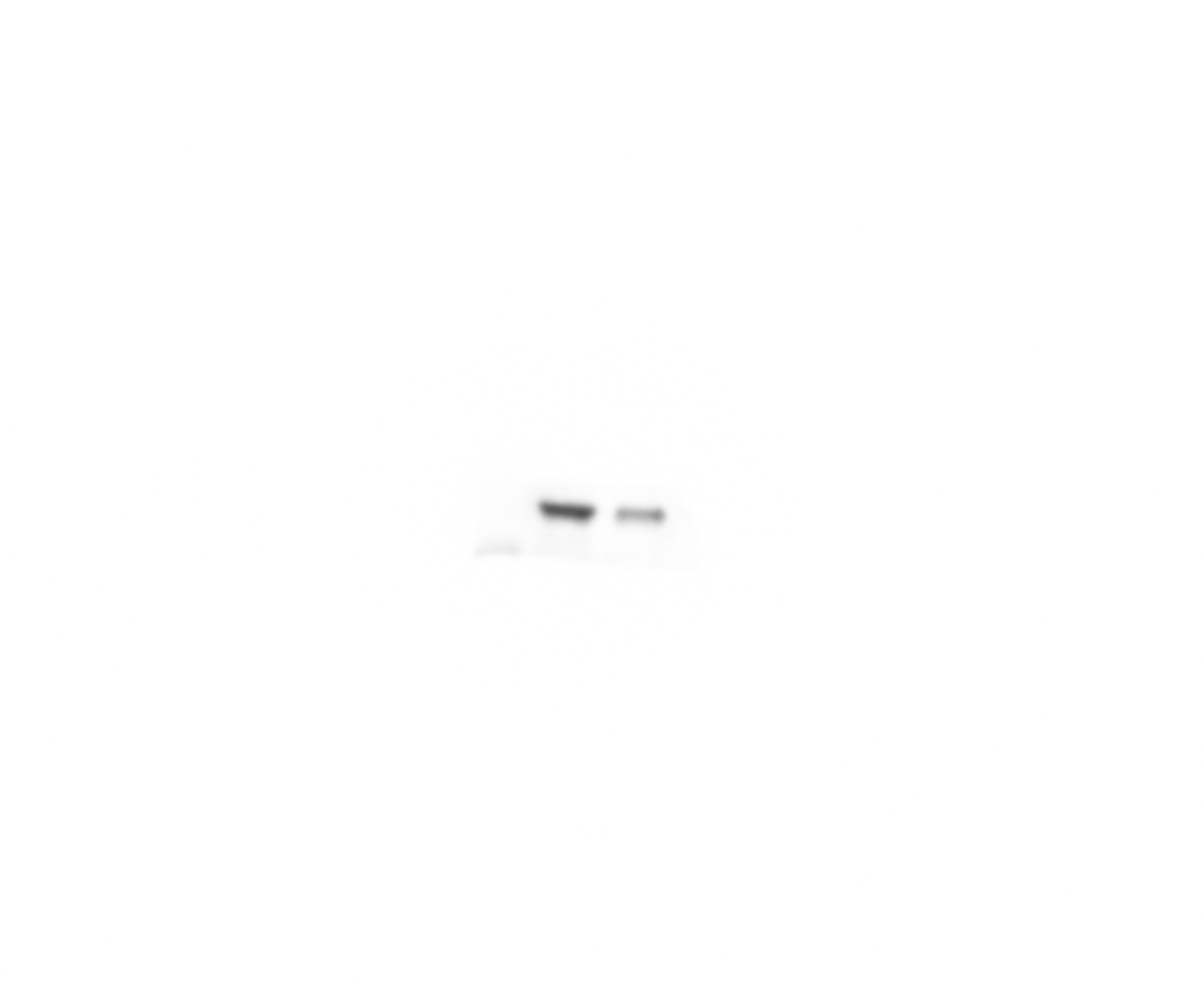

Supplement: Figure 7—source data 1. [file elife-98524-fig7-data1.zip › Fig 7-data1-v1/7E/right/BAX.tif]

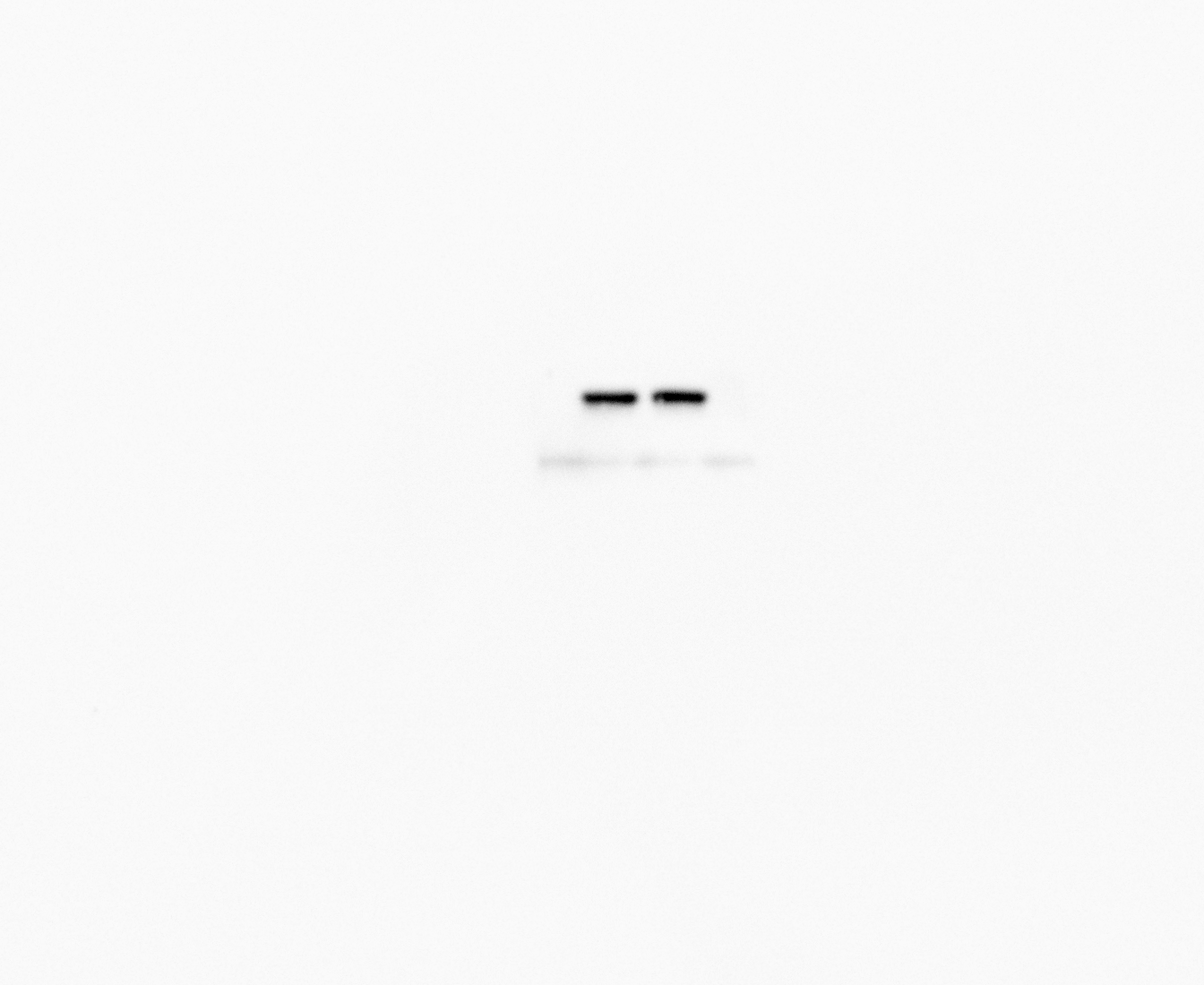

Supplement: Figure 7—source data 1. [file elife-98524-fig7-data1.zip › Fig 7-data1-v1/7E/right/Tubulin bottom right.tif]

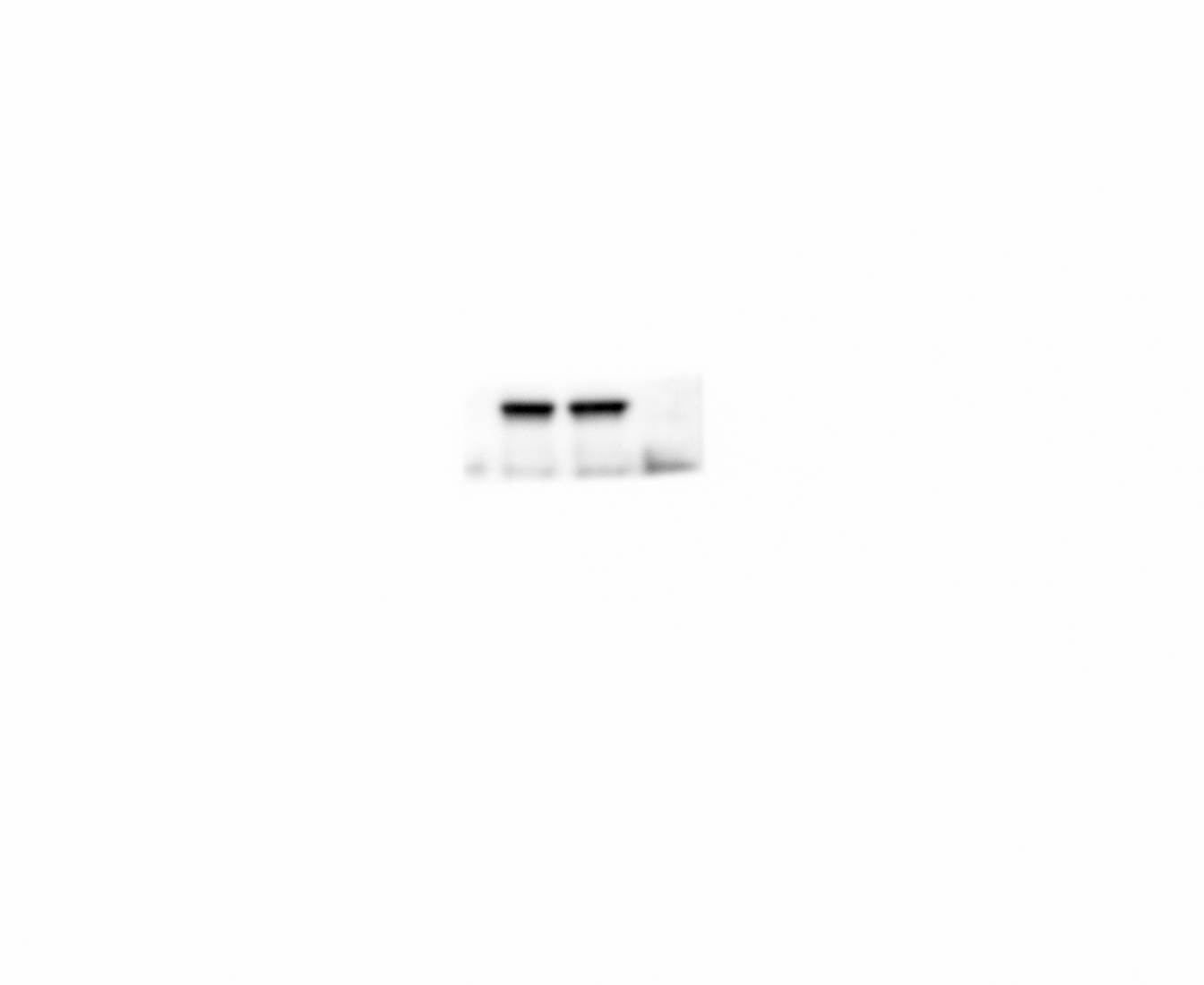

Supplement: Figure 7—source data 1. [file elife-98524-fig7-data1.zip › Fig 7-data1-v1/7E/right/Tubulin upper right.tif]

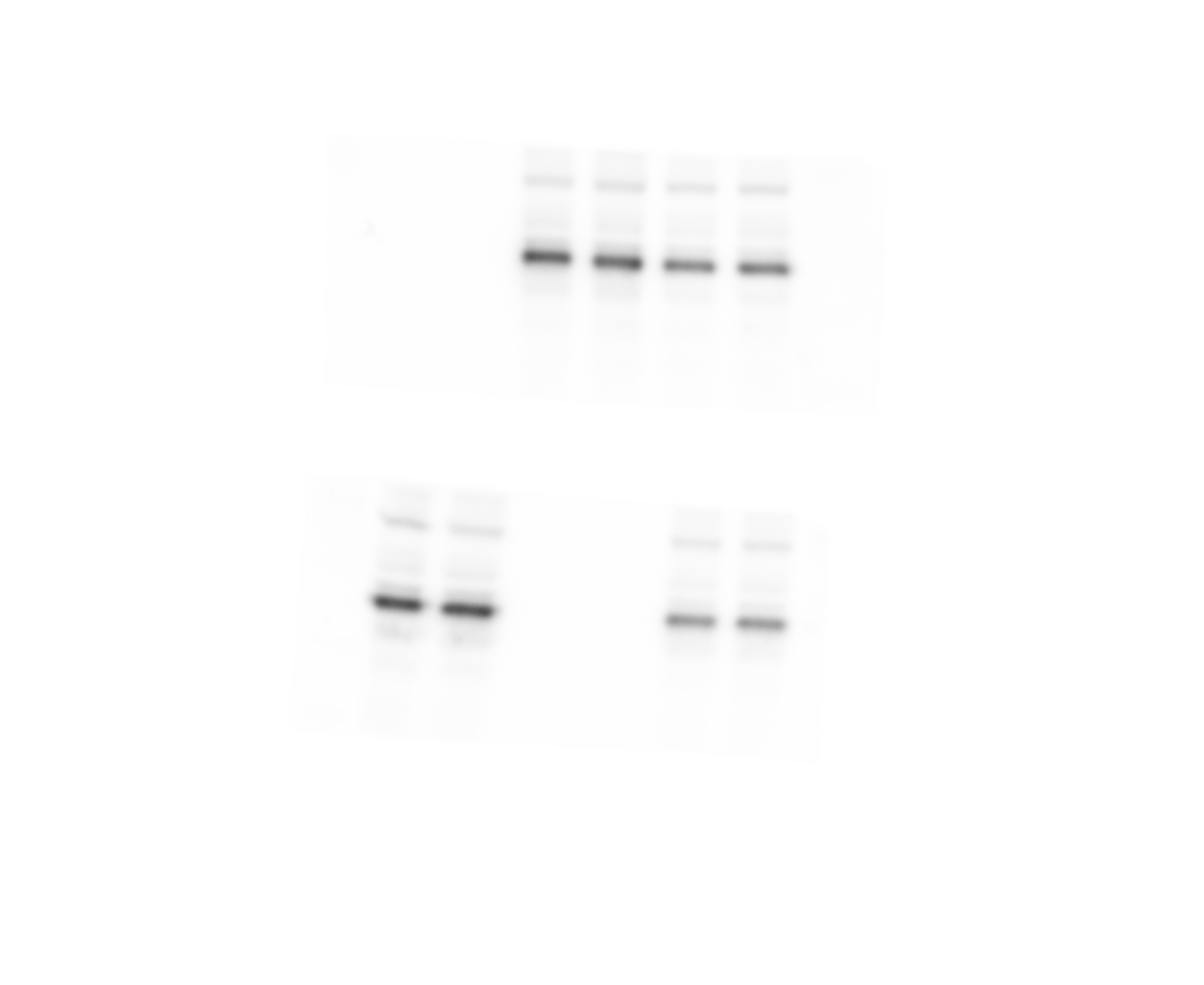

Supplement: Figure 7—source data 1. [file elife-98524-fig7-data1.zip › Fig 7-data1-v1/7F/COX IV.tif]

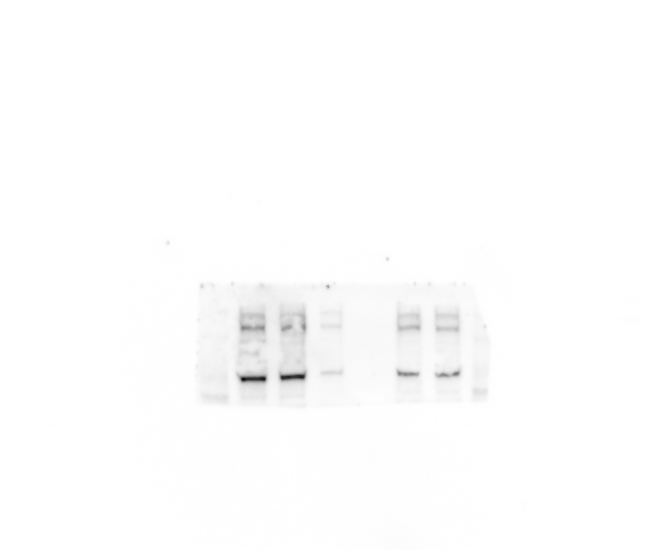

Supplement: Figure 7—source data 1. [file elife-98524-fig7-data1.zip › Fig 7-data1-v1/7F/SIRT4.tif]

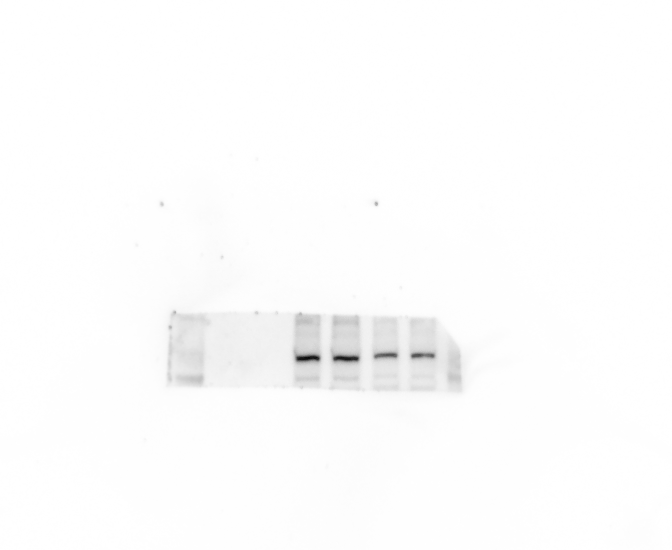

Supplement: Figure 7—source data 1. [file elife-98524-fig7-data1.zip › Fig 7-data1-v1/7F/Tubulin.tif]

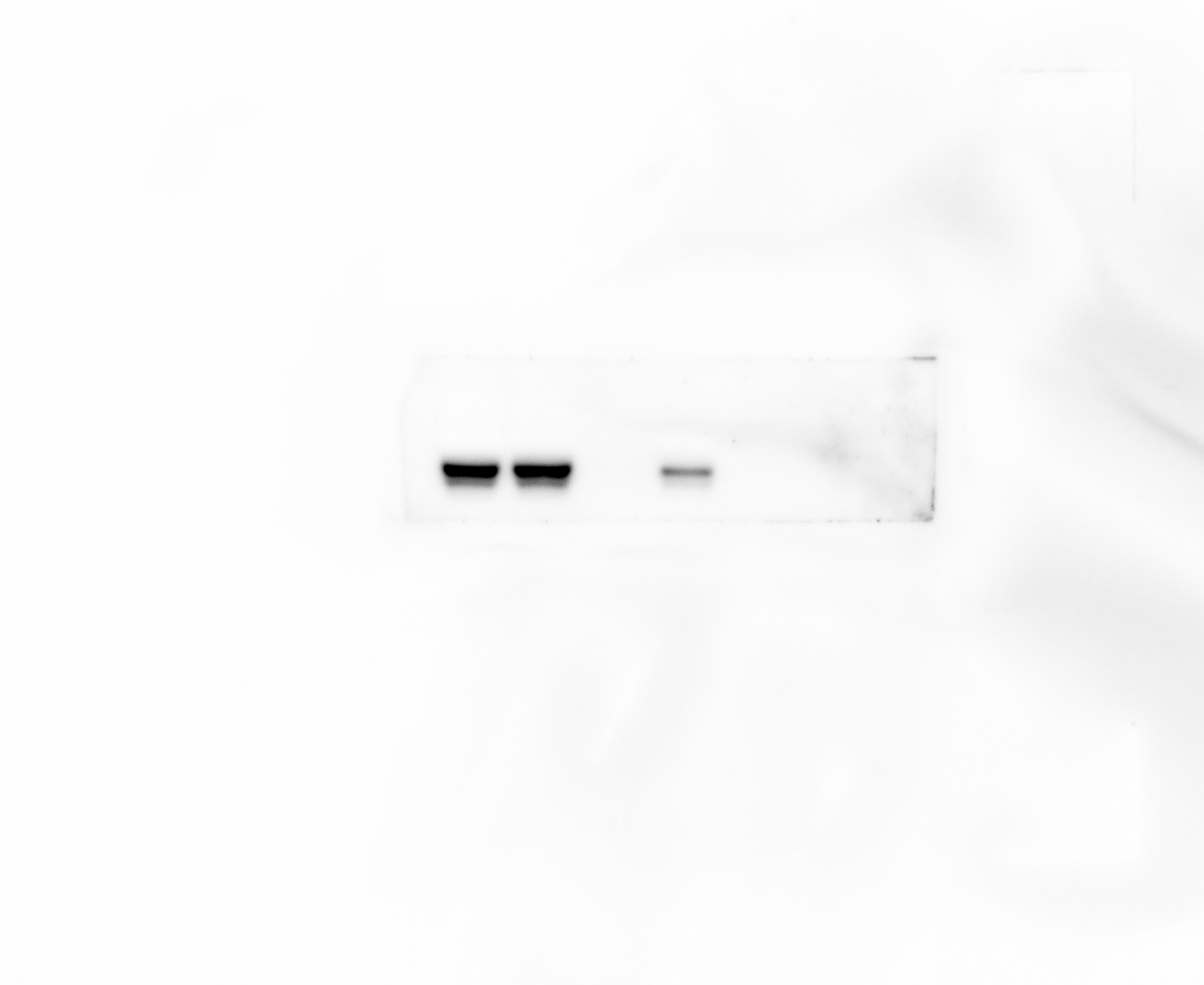

Supplement: Figure 7—source data 1. [file elife-98524-fig7-data1.zip › Fig 7-data1-v1/7G/SIRT4.tif]

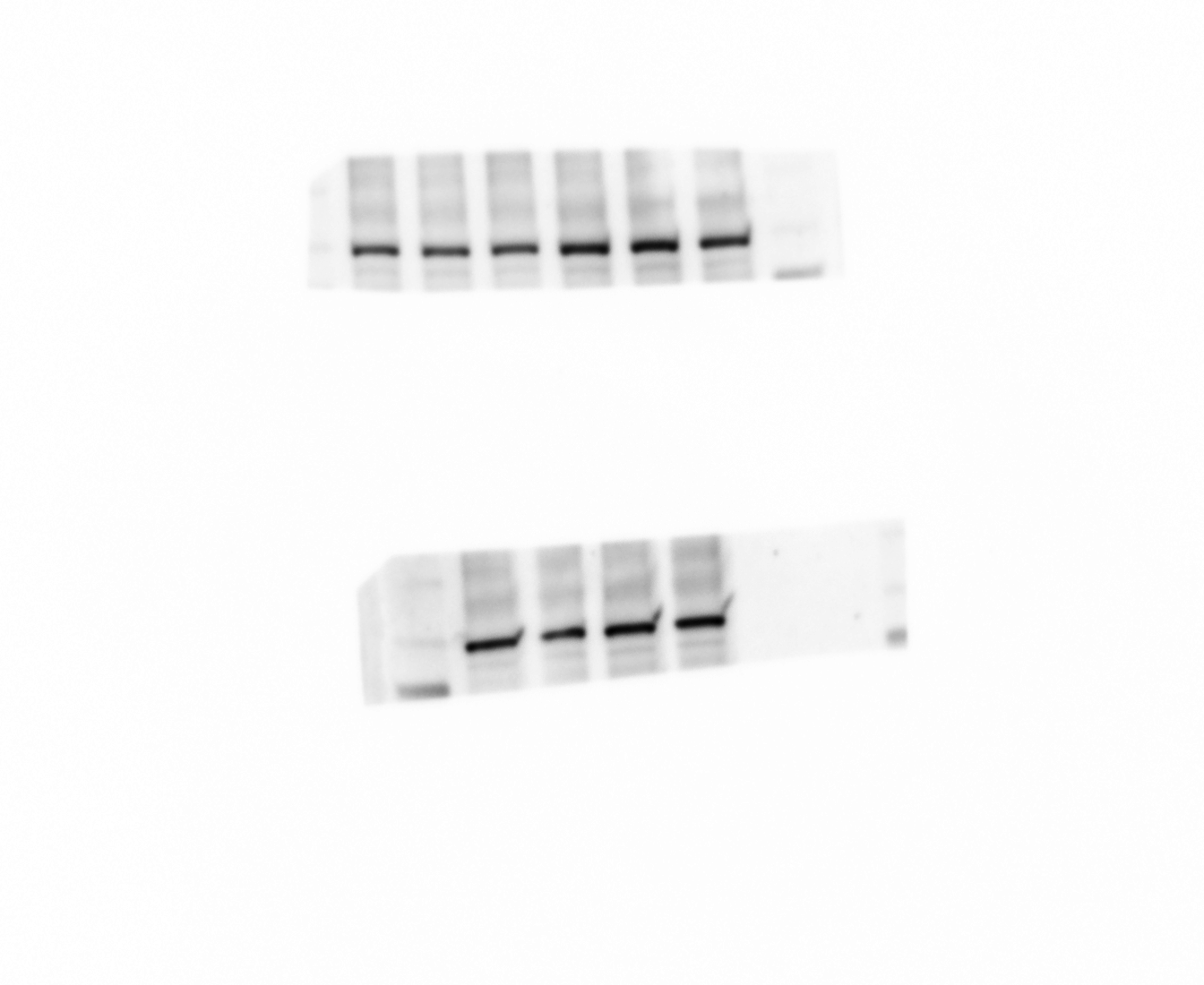

Supplement: Figure 7—source data 1. [file elife-98524-fig7-data1.zip › Fig 7-data1-v1/7G/Tubulin.tif]

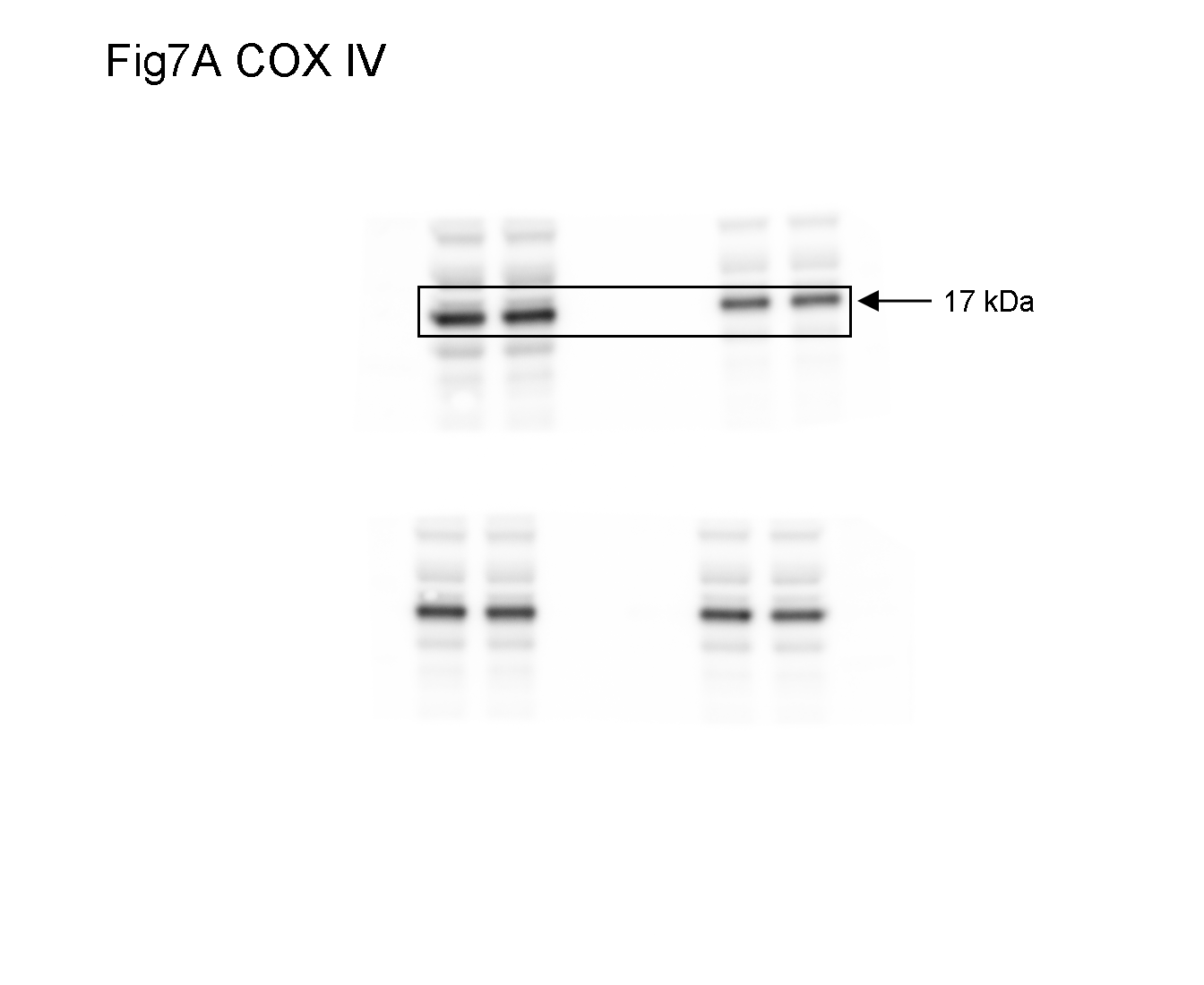

Supplement: Figure 7—source data 2. [file elife-98524-fig7-data2.zip › Fig 7-data2-v1/7A/COX IV.tif]

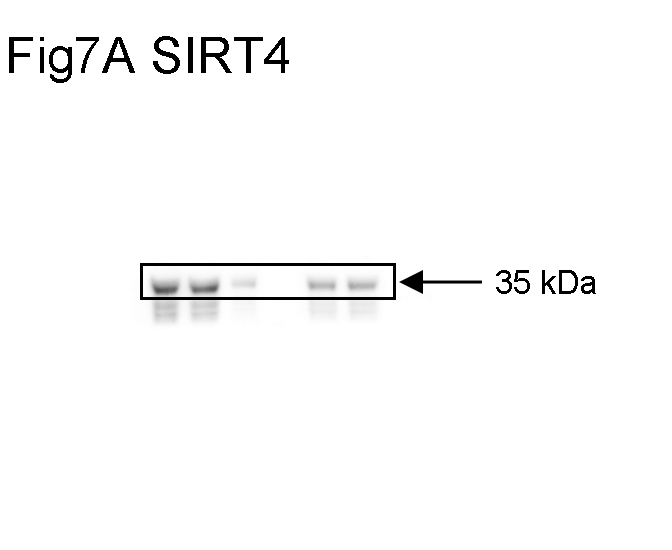

Supplement: Figure 7—source data 2. [file elife-98524-fig7-data2.zip › Fig 7-data2-v1/7A/SIRT4.tif]

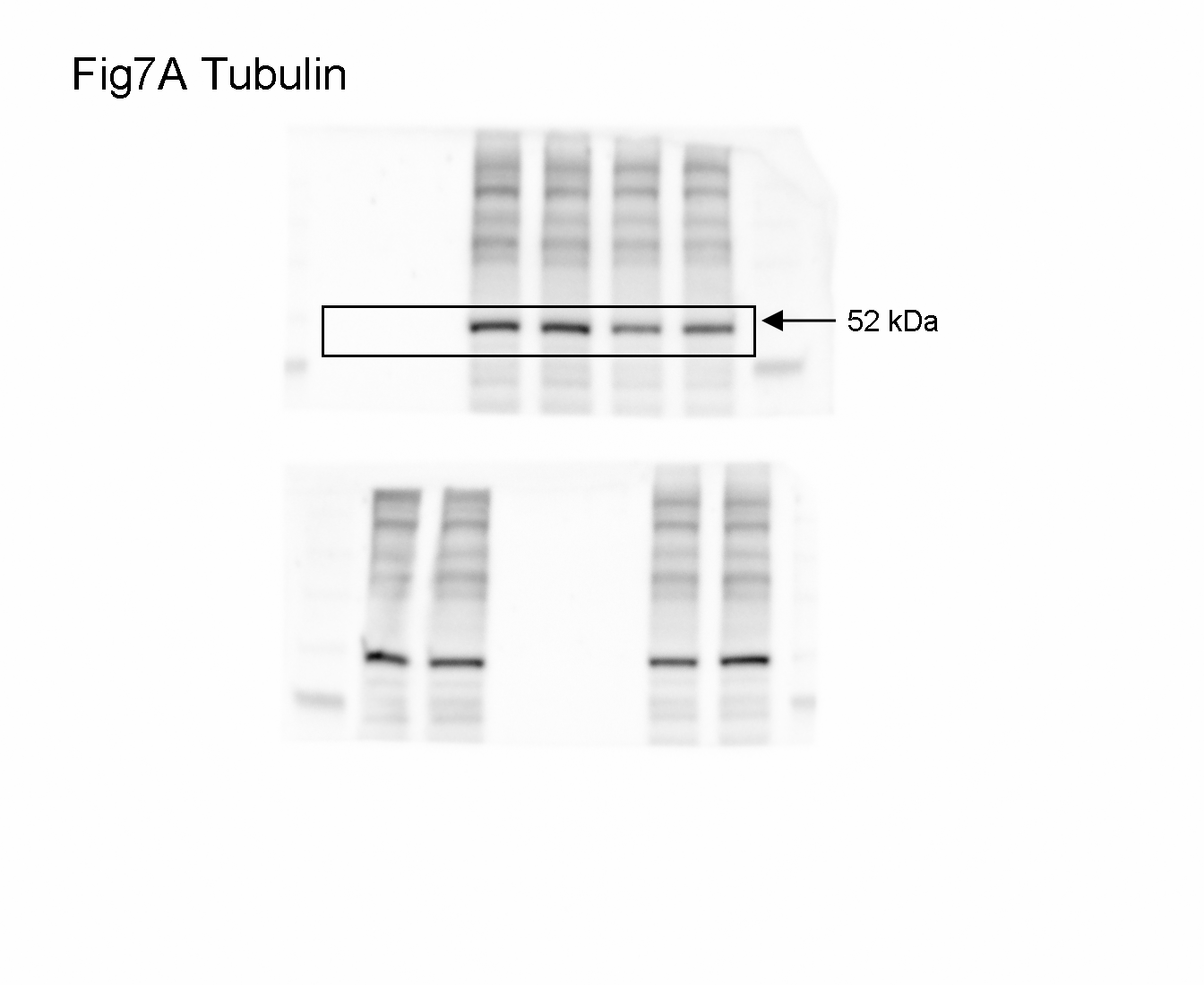

Supplement: Figure 7—source data 2. [file elife-98524-fig7-data2.zip › Fig 7-data2-v1/7A/Tubulin.tif]

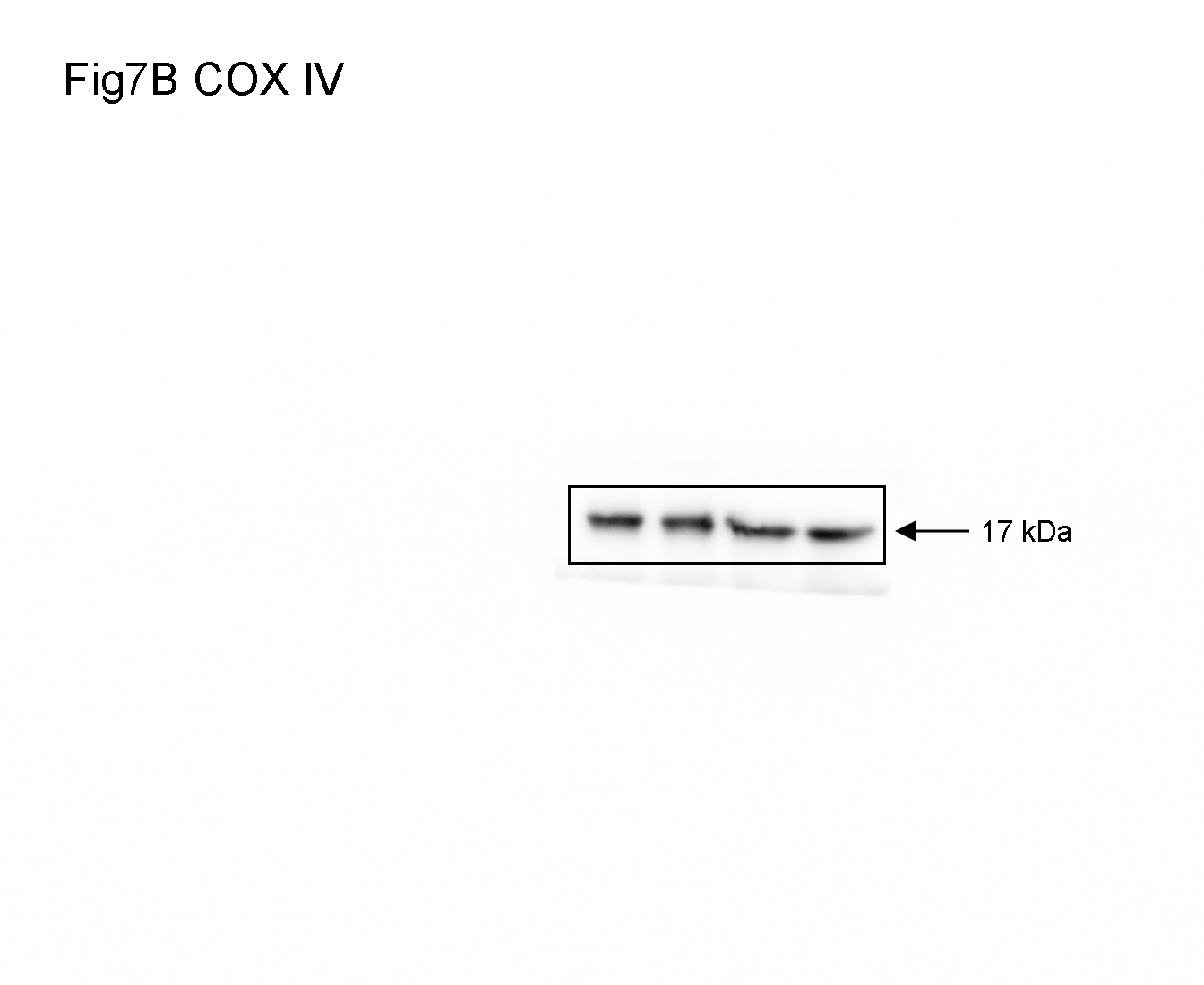

Supplement: Figure 7—source data 2. [file elife-98524-fig7-data2.zip › Fig 7-data2-v1/7B/left/COX IV.tif]

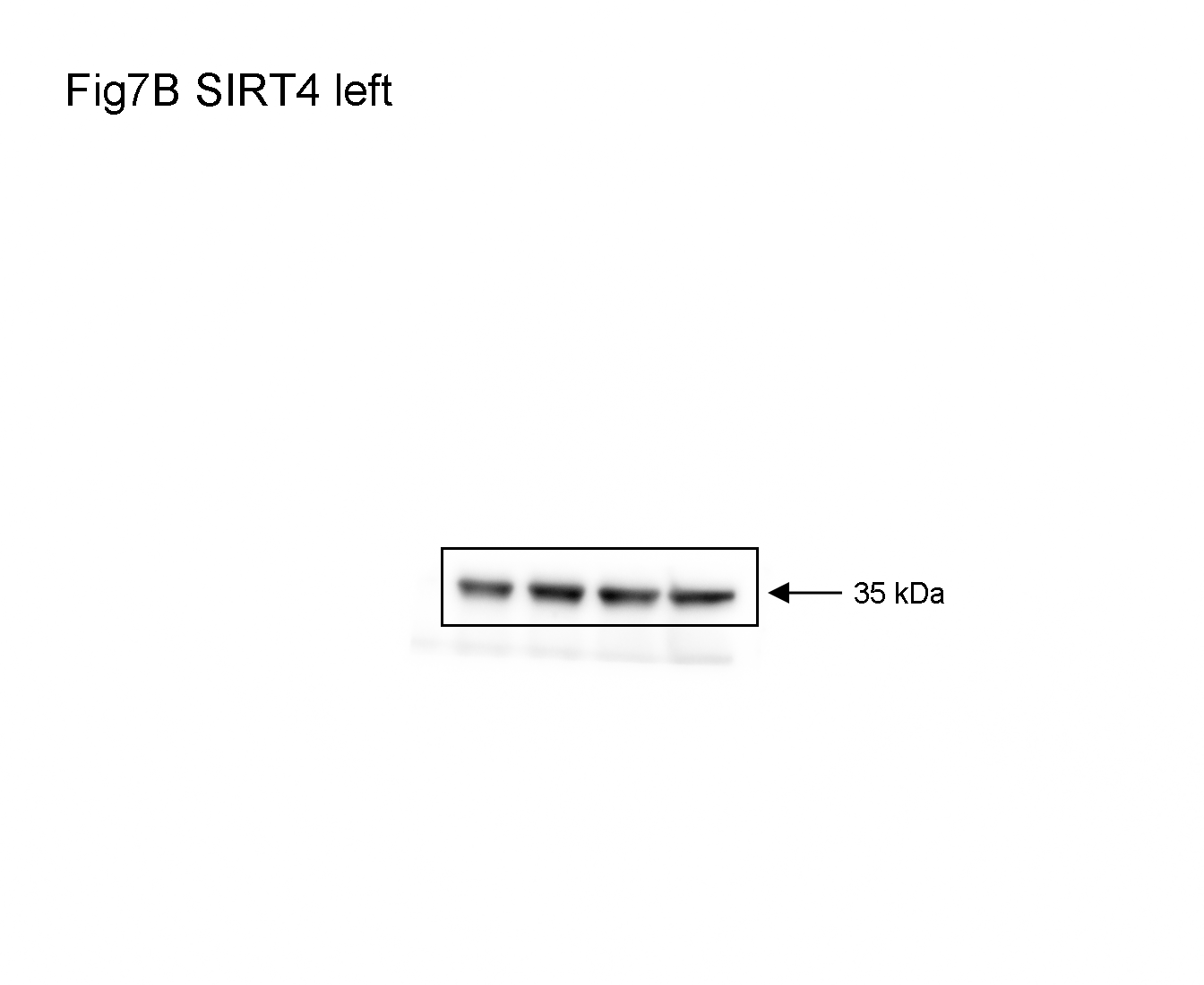

Supplement: Figure 7—source data 2. [file elife-98524-fig7-data2.zip › Fig 7-data2-v1/7B/left/SIRT4 left.tif]

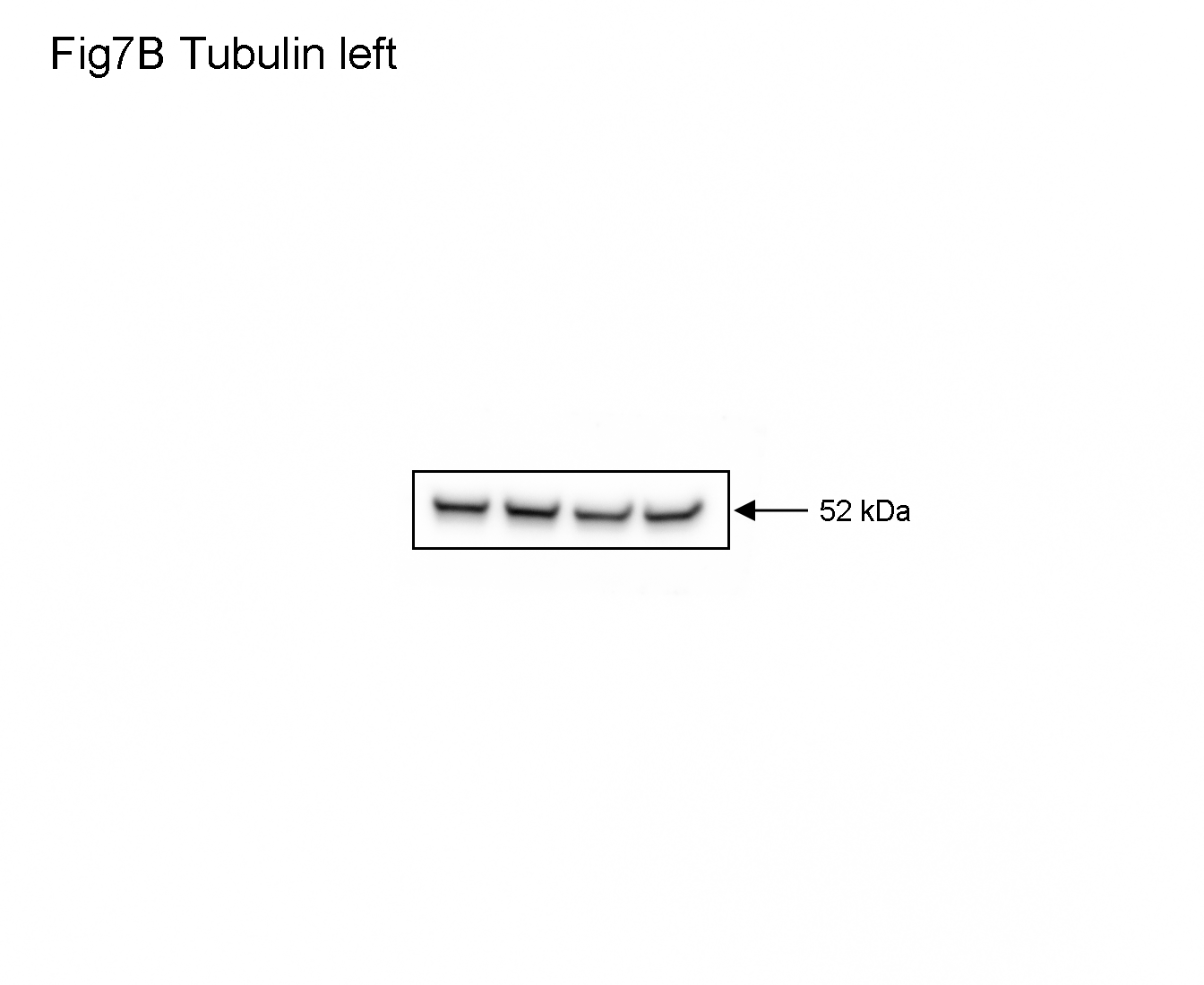

Supplement: Figure 7—source data 2. [file elife-98524-fig7-data2.zip › Fig 7-data2-v1/7B/left/Tubulin left.tif]

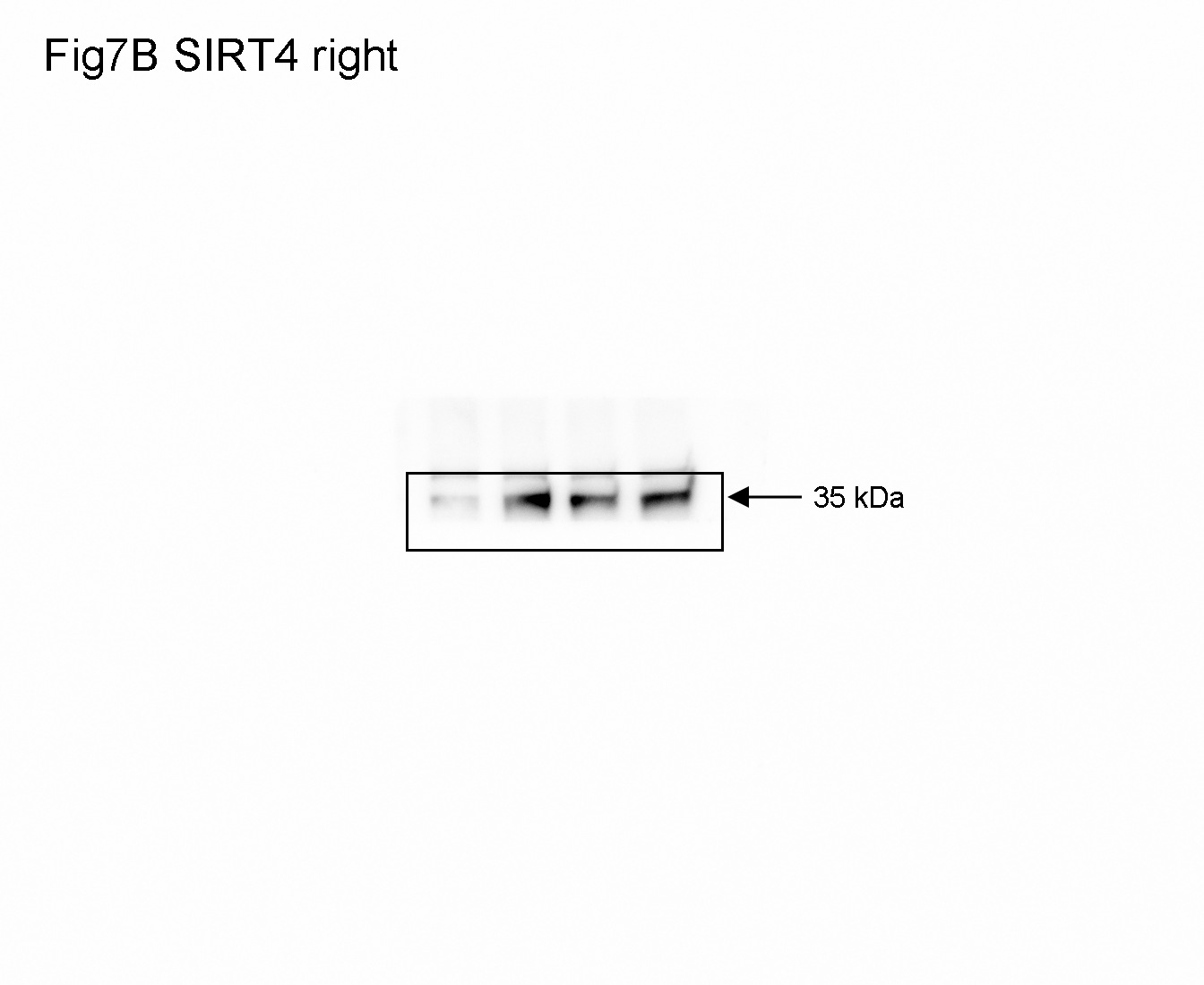

Supplement: Figure 7—source data 2. [file elife-98524-fig7-data2.zip › Fig 7-data2-v1/7B/right/SIRT4 right.tif]

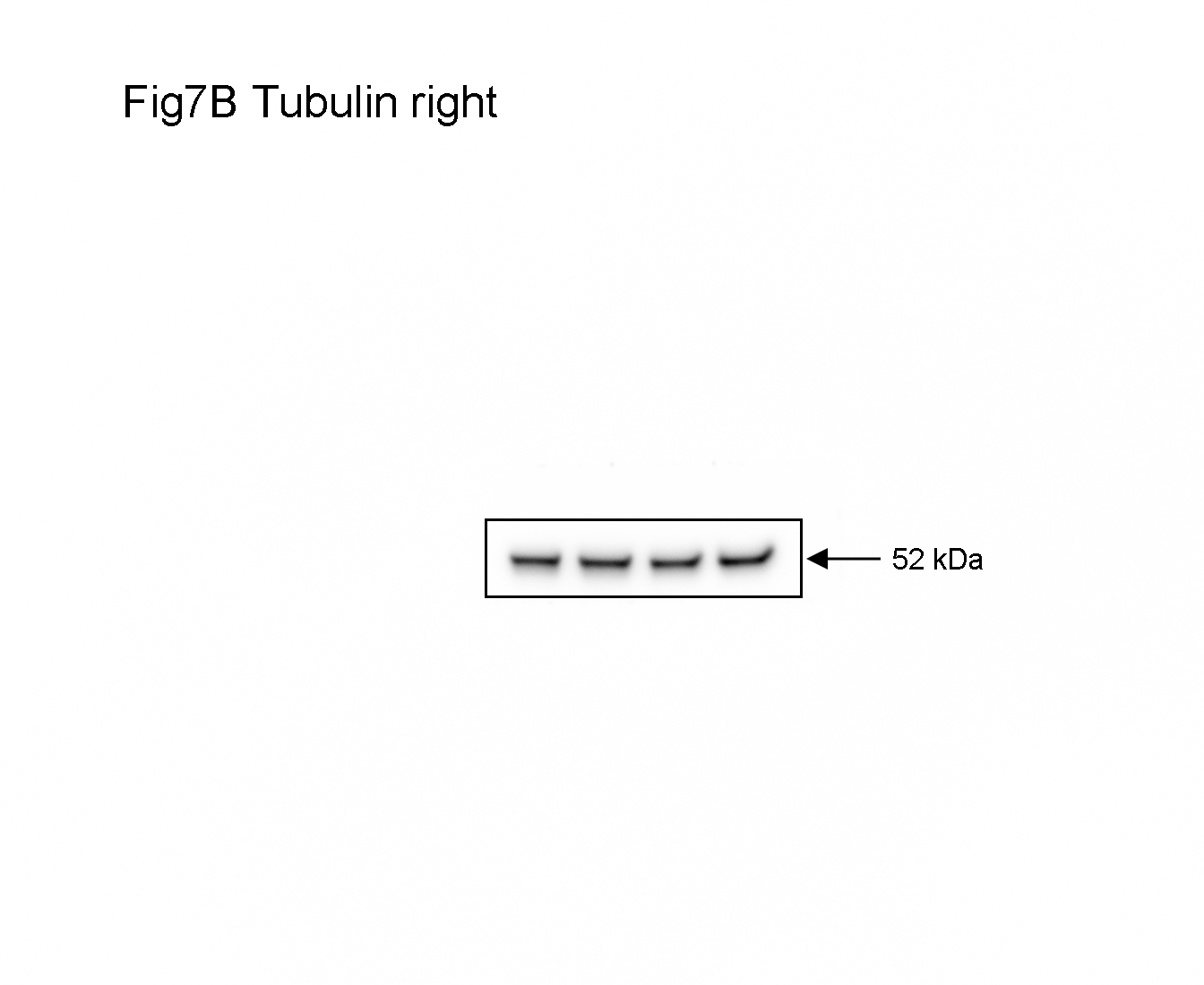

Supplement: Figure 7—source data 2. [file elife-98524-fig7-data2.zip › Fig 7-data2-v1/7B/right/Tubulin right.tif]

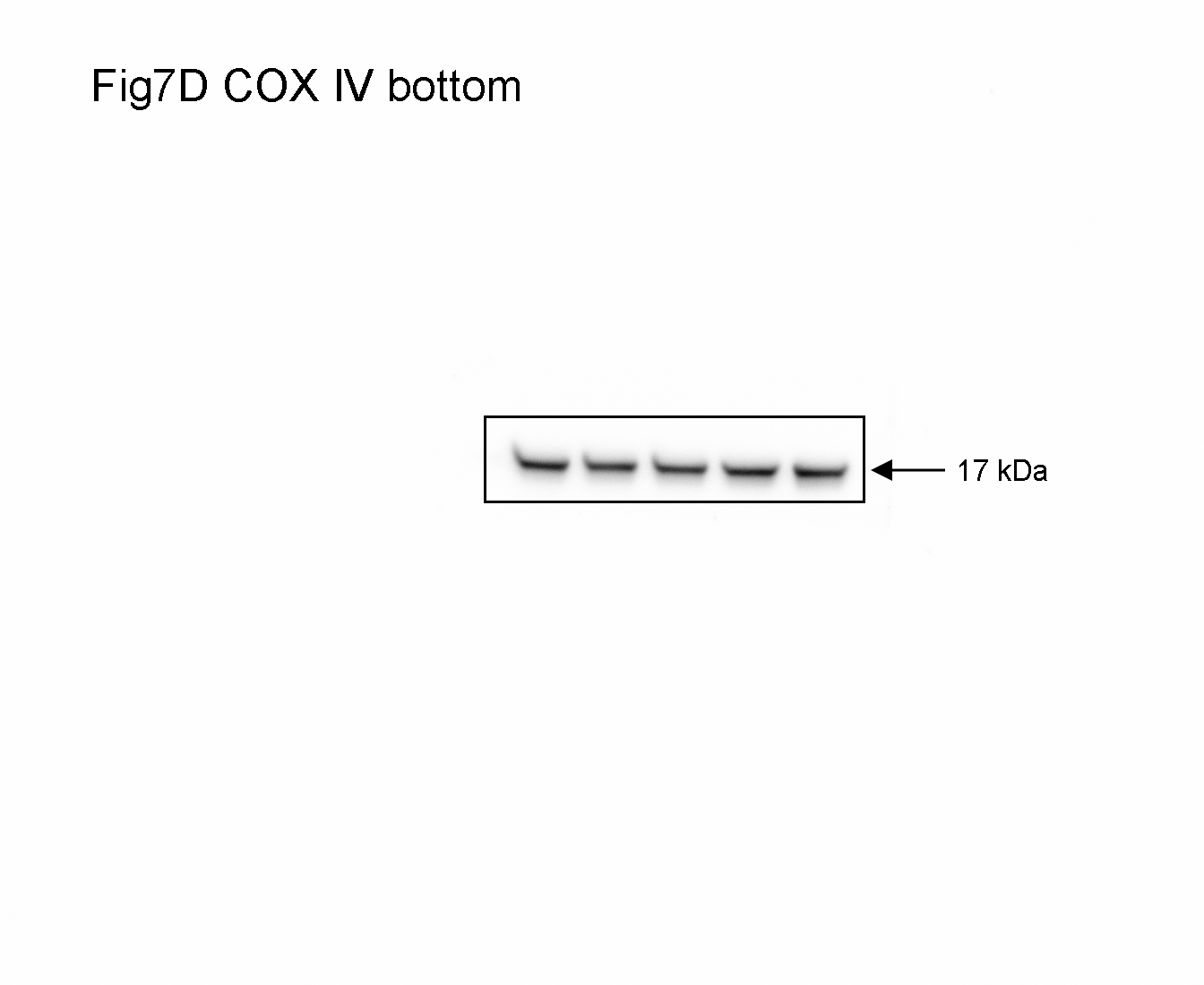

Supplement: Figure 7—source data 2. [file elife-98524-fig7-data2.zip › Fig 7-data2-v1/7D/bottom/COX IV bottom.tif]

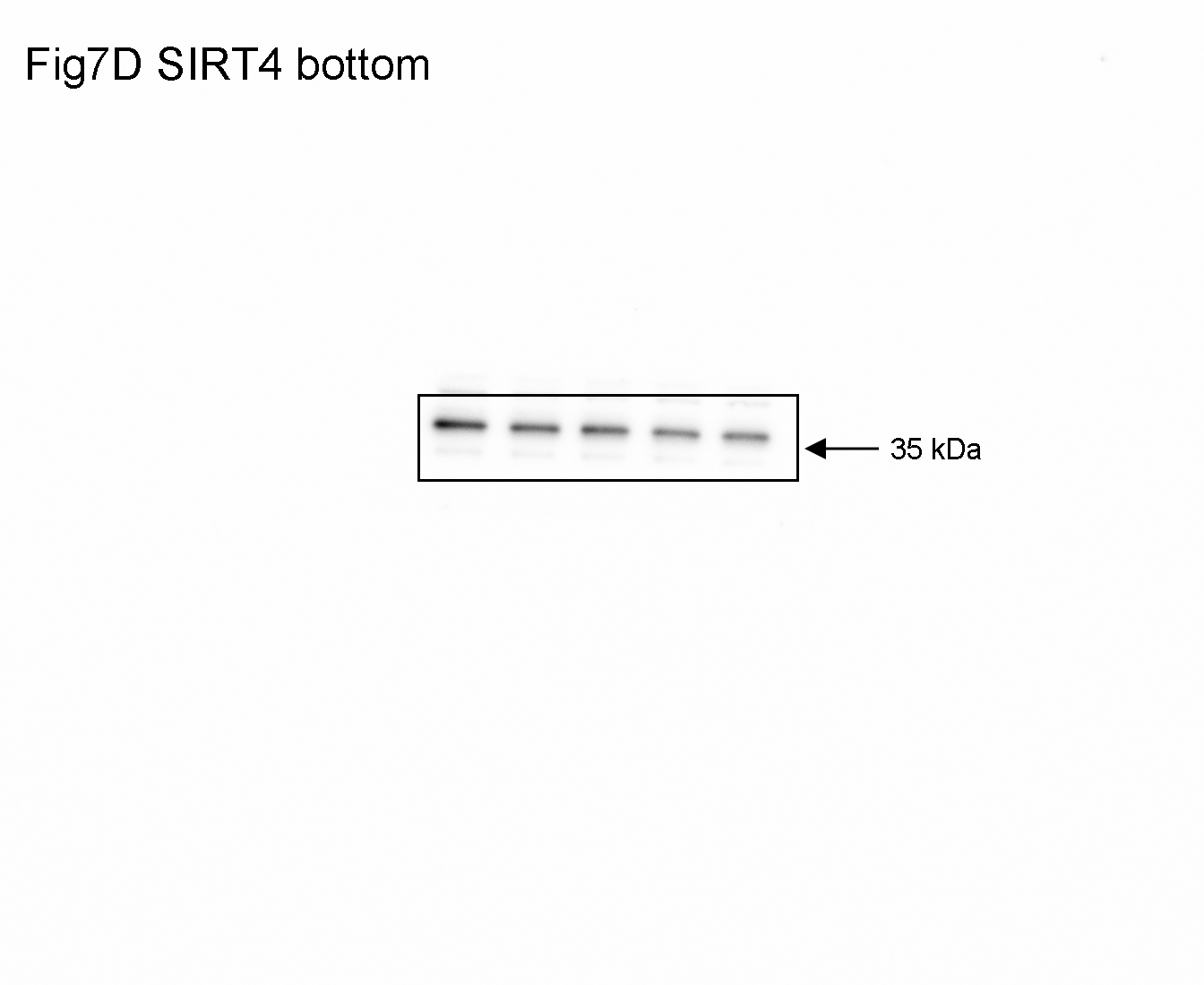

Supplement: Figure 7—source data 2. [file elife-98524-fig7-data2.zip › Fig 7-data2-v1/7D/bottom/SIRT4 bottom.tif]
