# Supplementary figures and images for: Nuclear translocation of SIRT4 mediates deacetylation of U2AF2 to modulate renal fibrosis through alternative splicing-mediated upregulation of CCN2 (part 7 of 9)
Source: eLife. 2024 Nov 4;13:RP98524. doi: 10.7554/eLife.98524 (PMC11534337; doi:10.7554/eLife.98524)

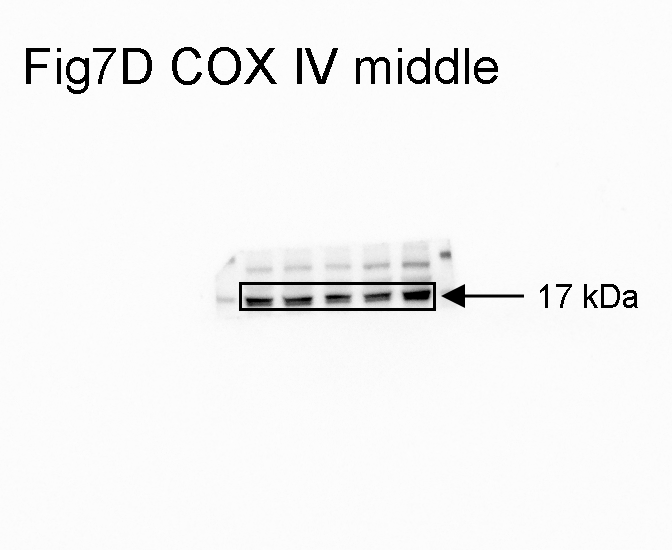

Supplement: Figure 7—source data 2. [file elife-98524-fig7-data2.zip › Fig 7-data2-v1/7D/middle/COX IV middle.tif]

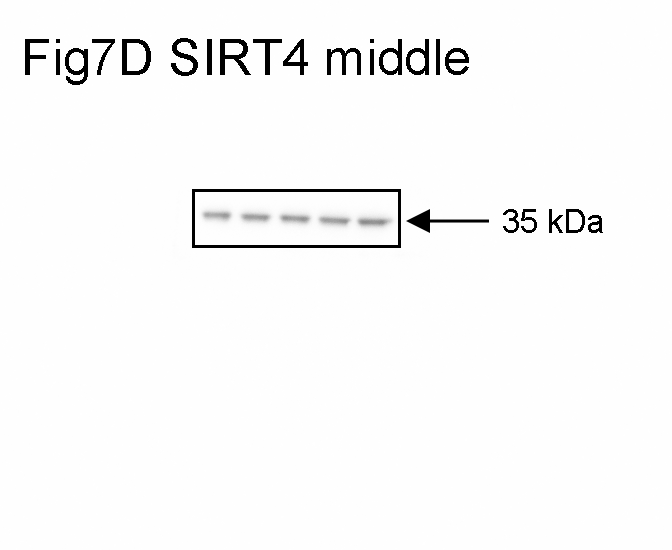

Supplement: Figure 7—source data 2. [file elife-98524-fig7-data2.zip › Fig 7-data2-v1/7D/middle/SIRT4 middle.tif]

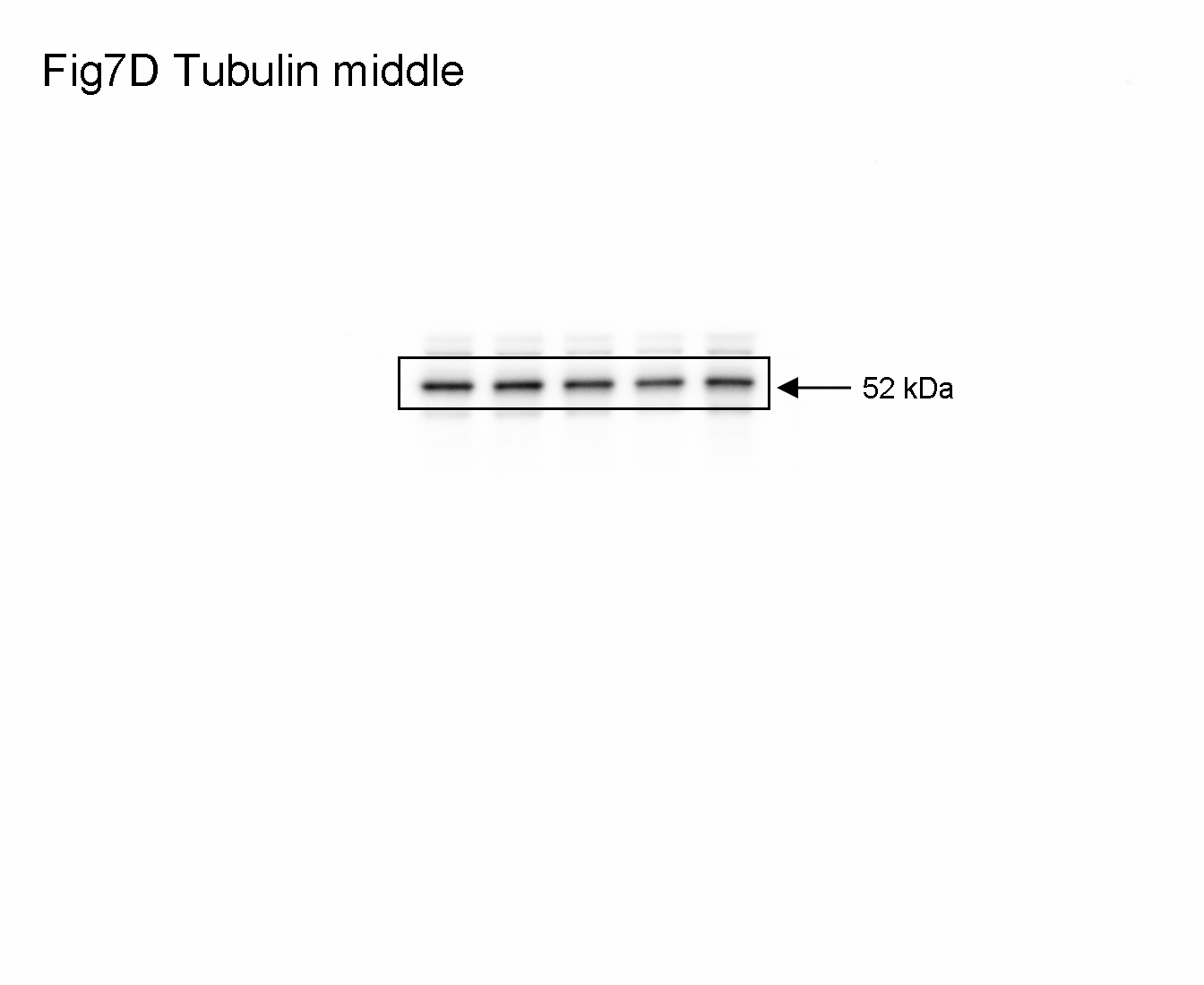

Supplement: Figure 7—source data 2. [file elife-98524-fig7-data2.zip › Fig 7-data2-v1/7D/middle/Tubulin middle.tif]

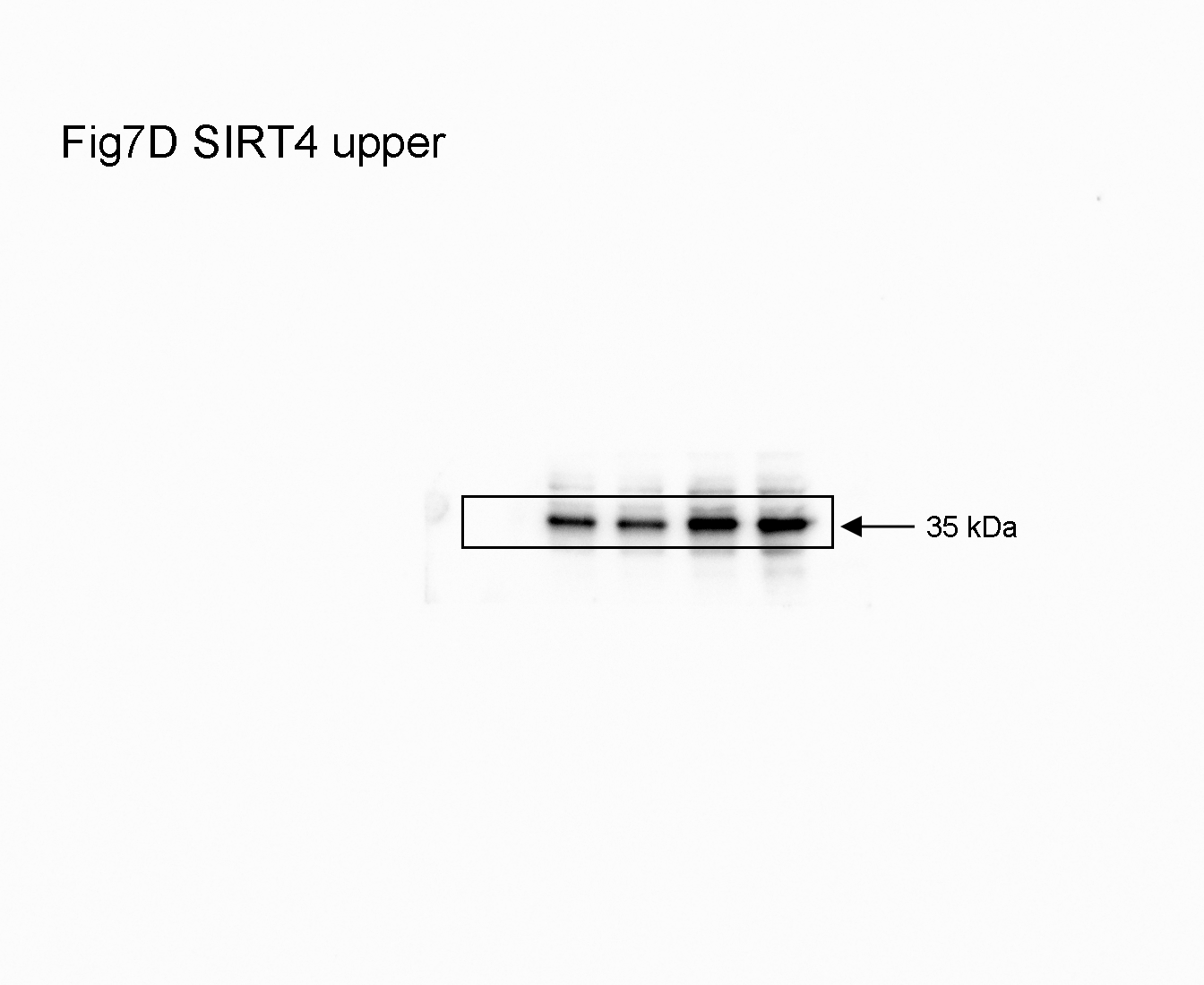

Supplement: Figure 7—source data 2. [file elife-98524-fig7-data2.zip › Fig 7-data2-v1/7D/upper/SIRT4 upper.tif]

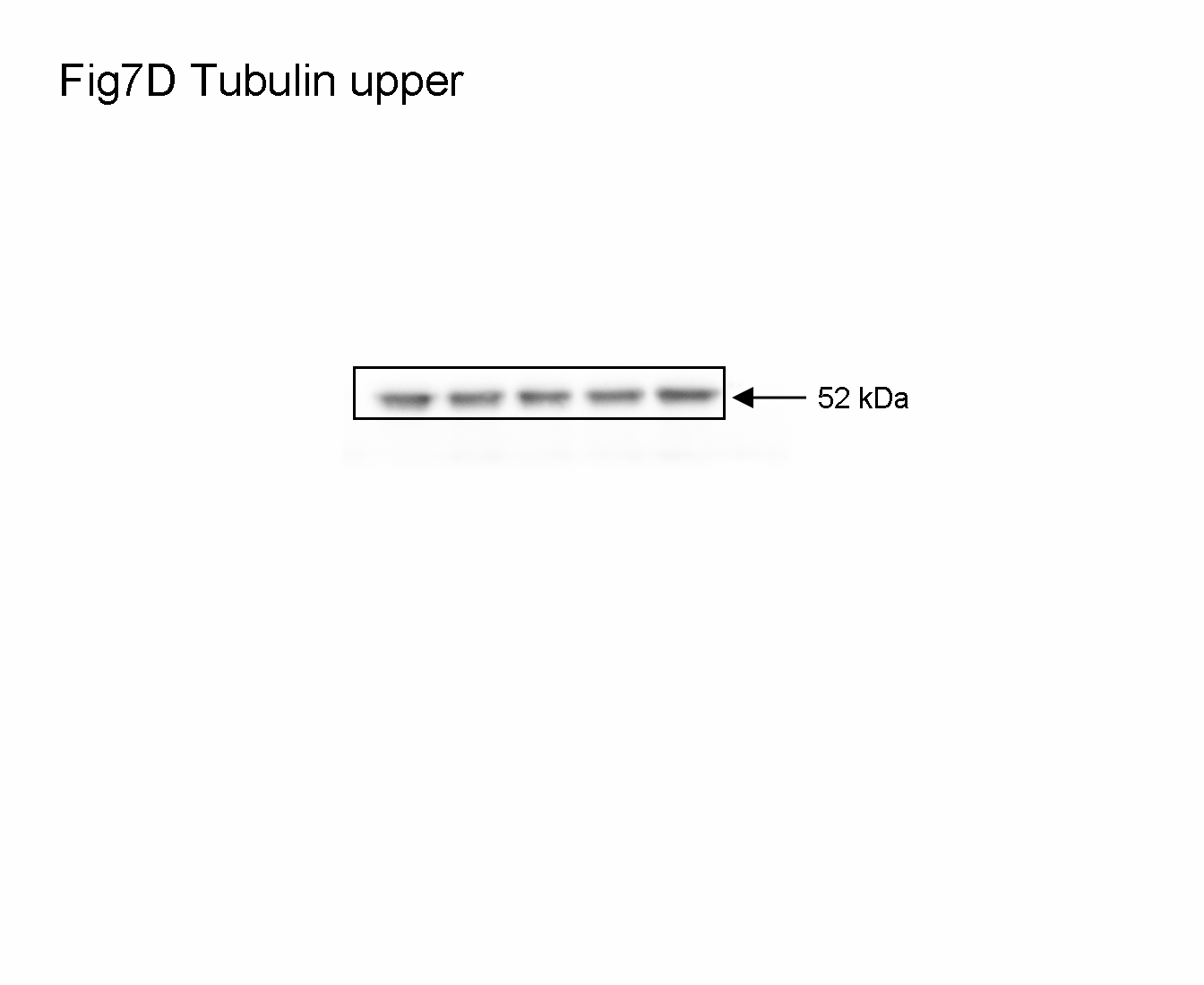

Supplement: Figure 7—source data 2. [file elife-98524-fig7-data2.zip › Fig 7-data2-v1/7D/upper/Tubulin upper.tif]

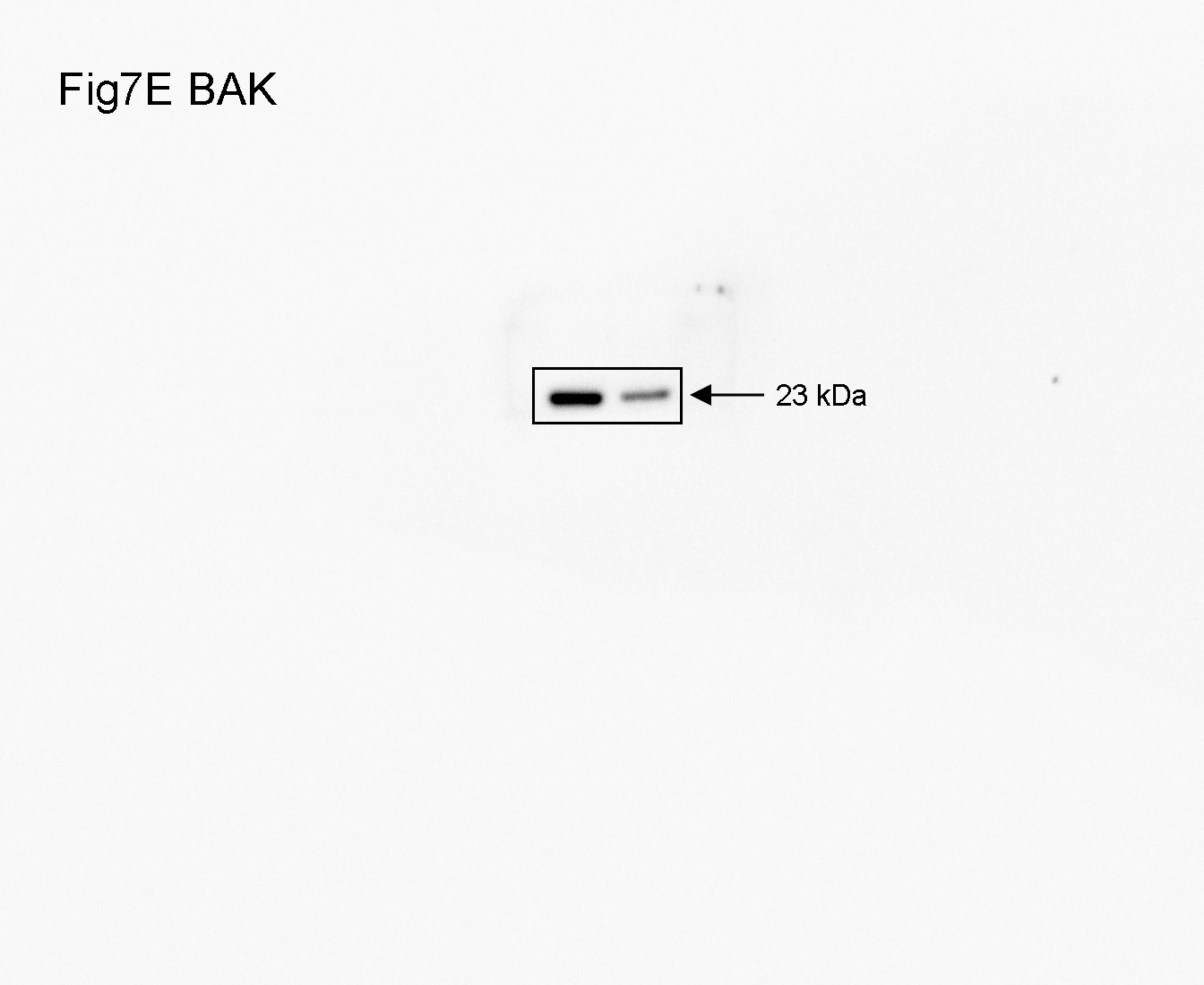

Supplement: Figure 7—source data 2. [file elife-98524-fig7-data2.zip › Fig 7-data2-v1/7E/BAK.tif]

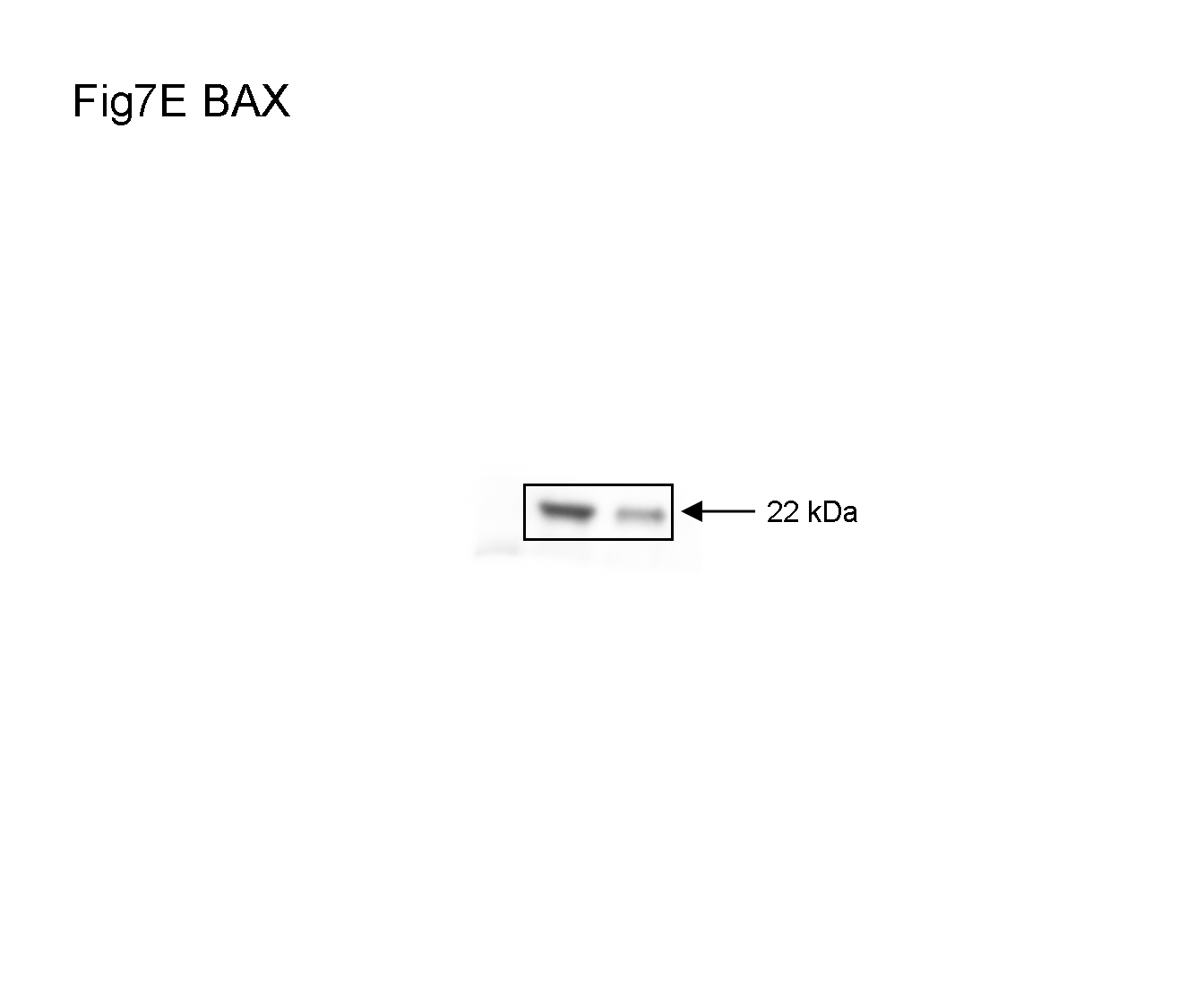

Supplement: Figure 7—source data 2. [file elife-98524-fig7-data2.zip › Fig 7-data2-v1/7E/BAX.tif]

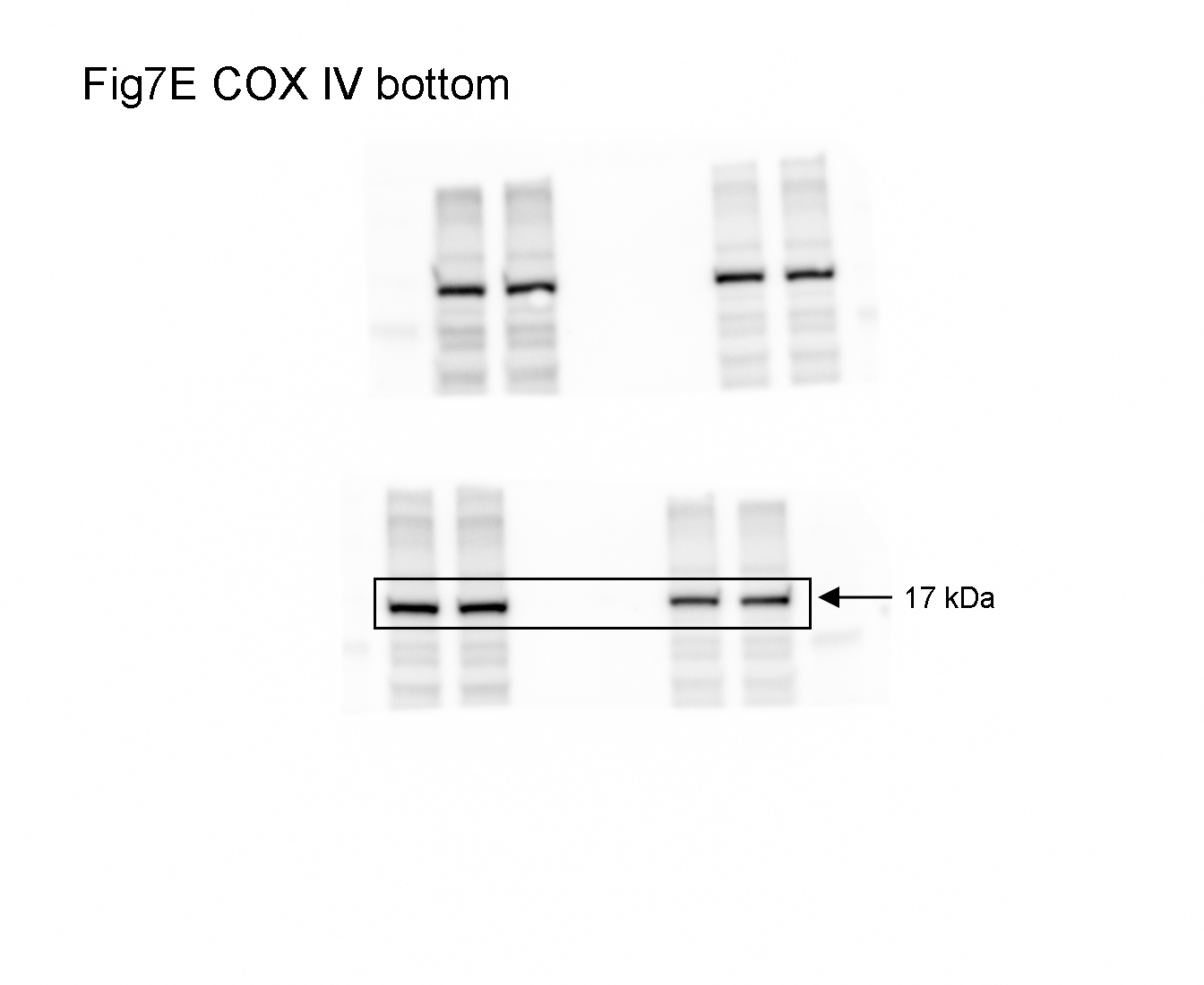

Supplement: Figure 7—source data 2. [file elife-98524-fig7-data2.zip › Fig 7-data2-v1/7E/COX IV bottom.tif]

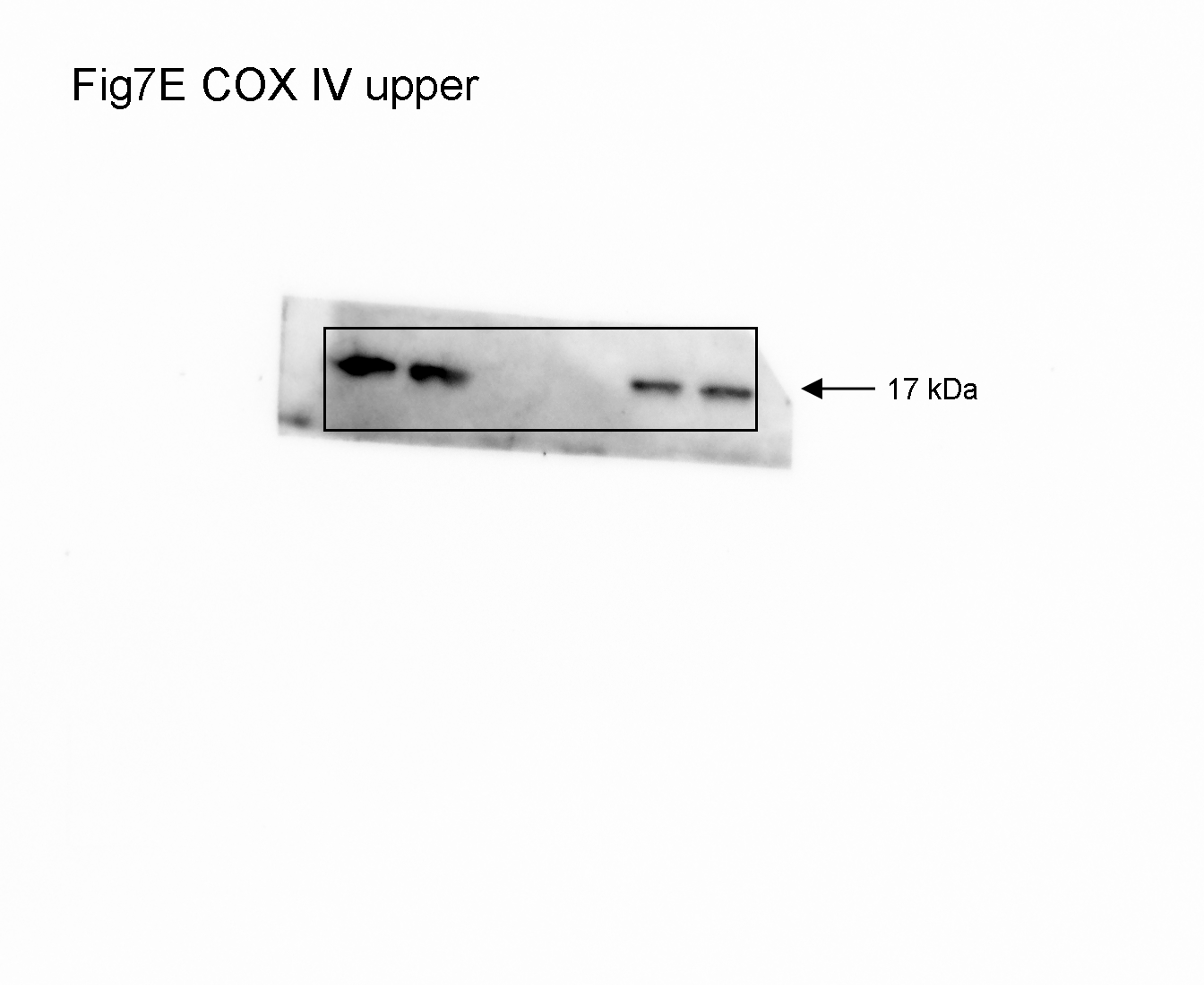

Supplement: Figure 7—source data 2. [file elife-98524-fig7-data2.zip › Fig 7-data2-v1/7E/COX IV upper.tif]

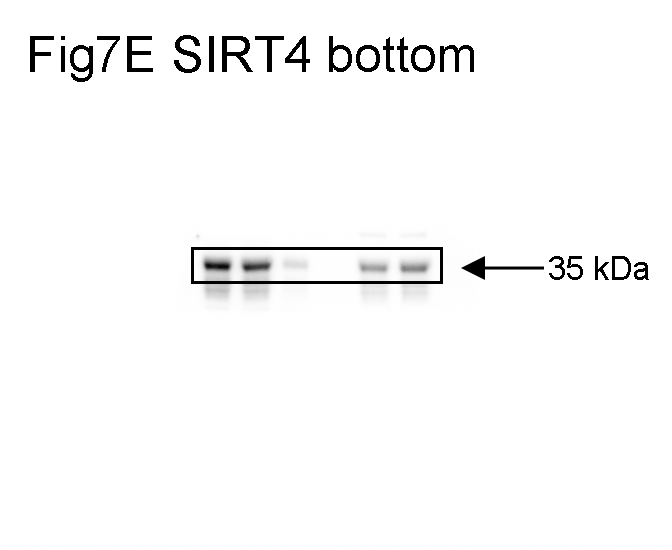

Supplement: Figure 7—source data 2. [file elife-98524-fig7-data2.zip › Fig 7-data2-v1/7E/SIRT4 bottom.tif]

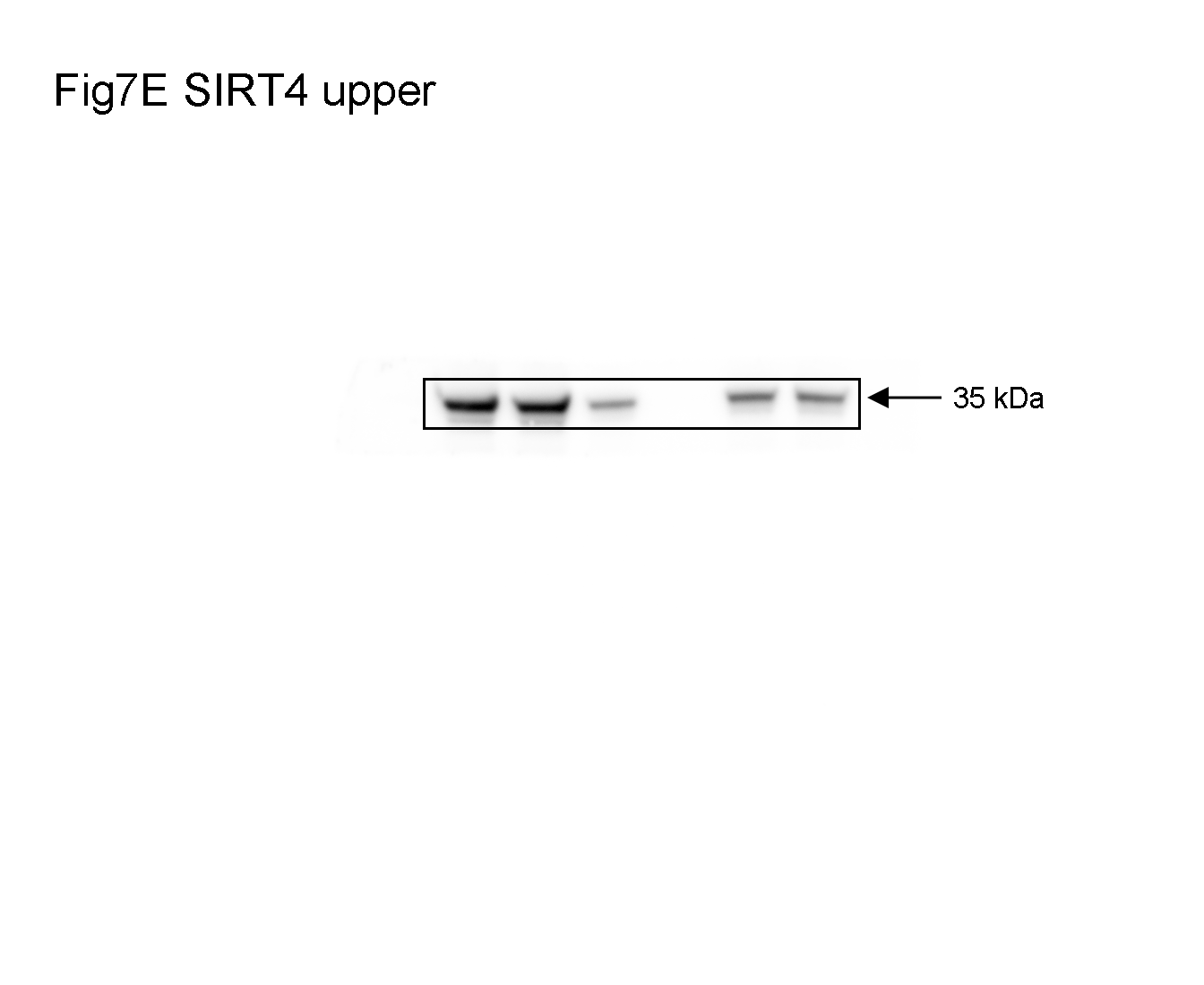

Supplement: Figure 7—source data 2. [file elife-98524-fig7-data2.zip › Fig 7-data2-v1/7E/SIRT4 upper.tif]

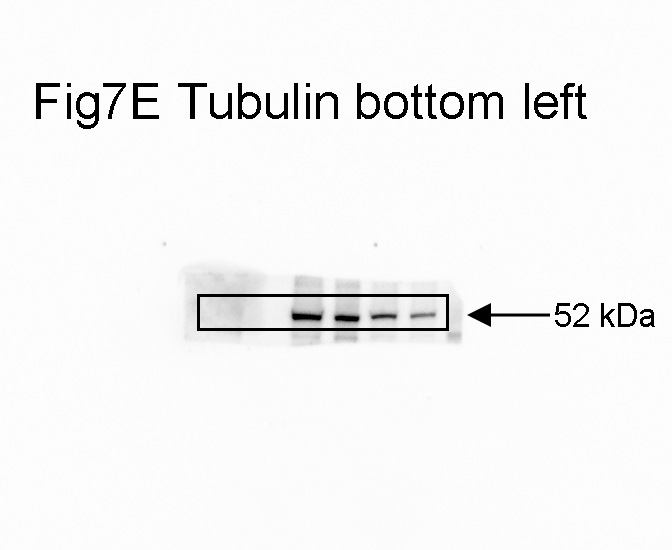

Supplement: Figure 7—source data 2. [file elife-98524-fig7-data2.zip › Fig 7-data2-v1/7E/Tubulin bottom left.tif]

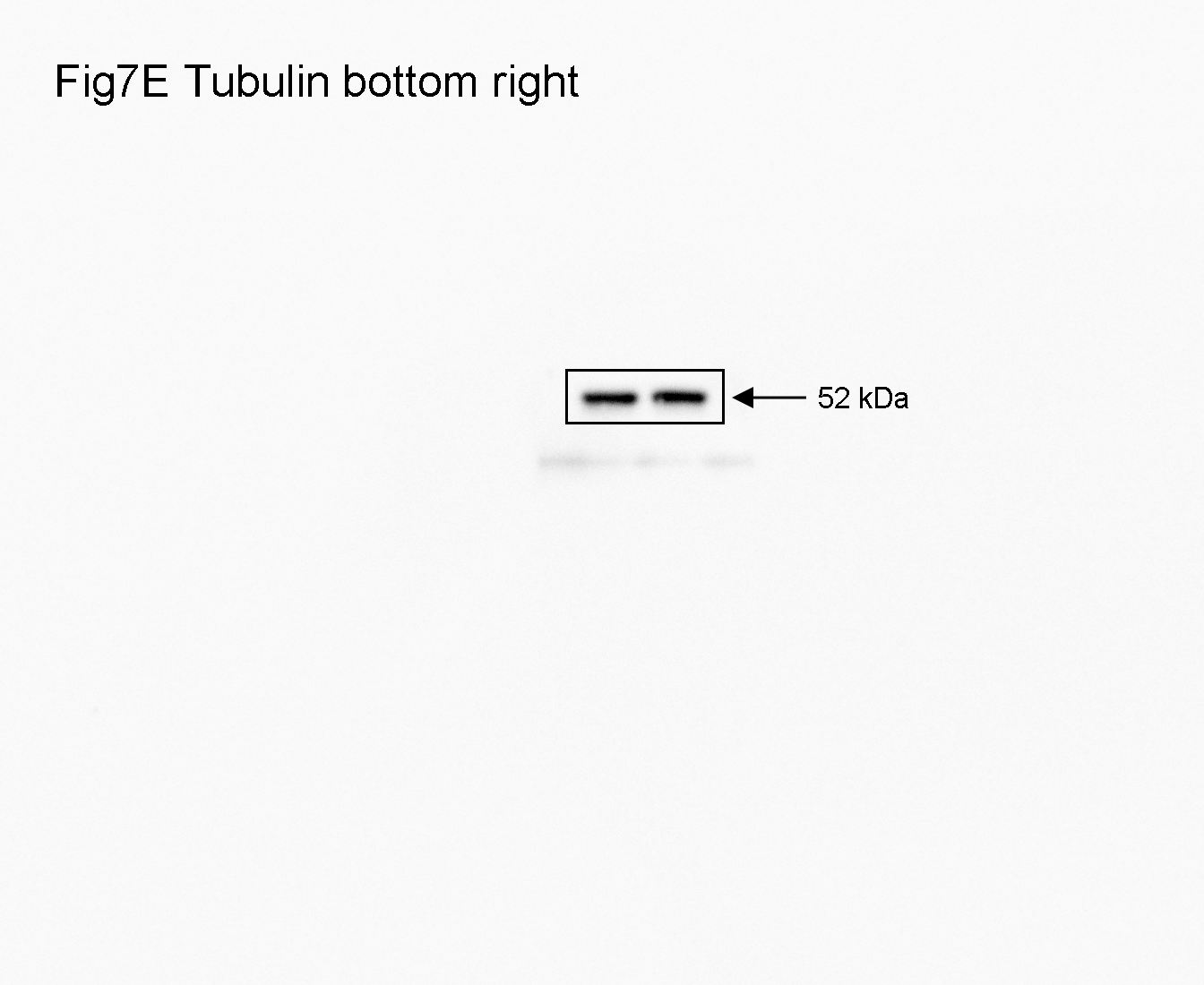

Supplement: Figure 7—source data 2. [file elife-98524-fig7-data2.zip › Fig 7-data2-v1/7E/Tubulin bottom right.tif]

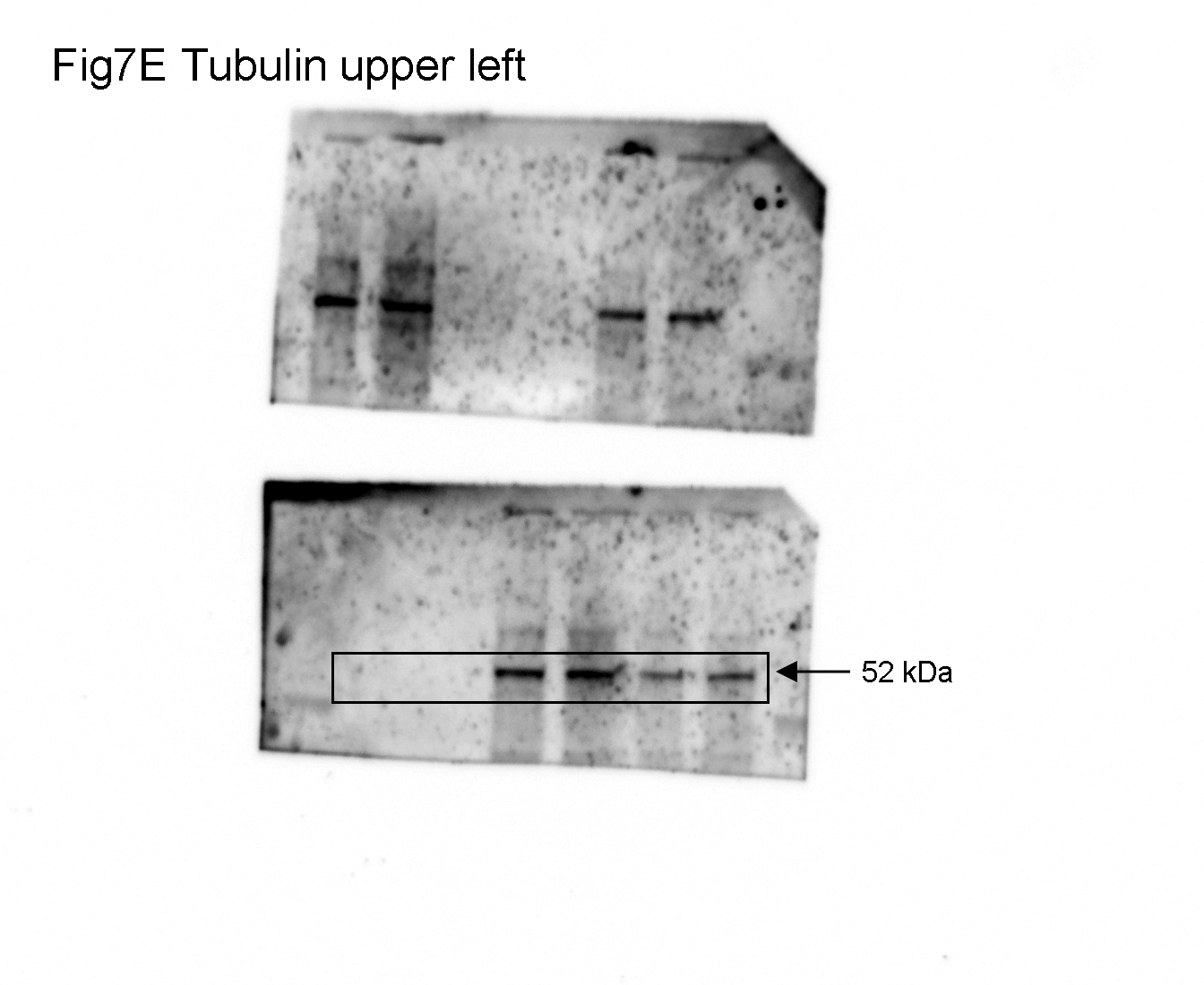

Supplement: Figure 7—source data 2. [file elife-98524-fig7-data2.zip › Fig 7-data2-v1/7E/Tubulin upper left.tif]

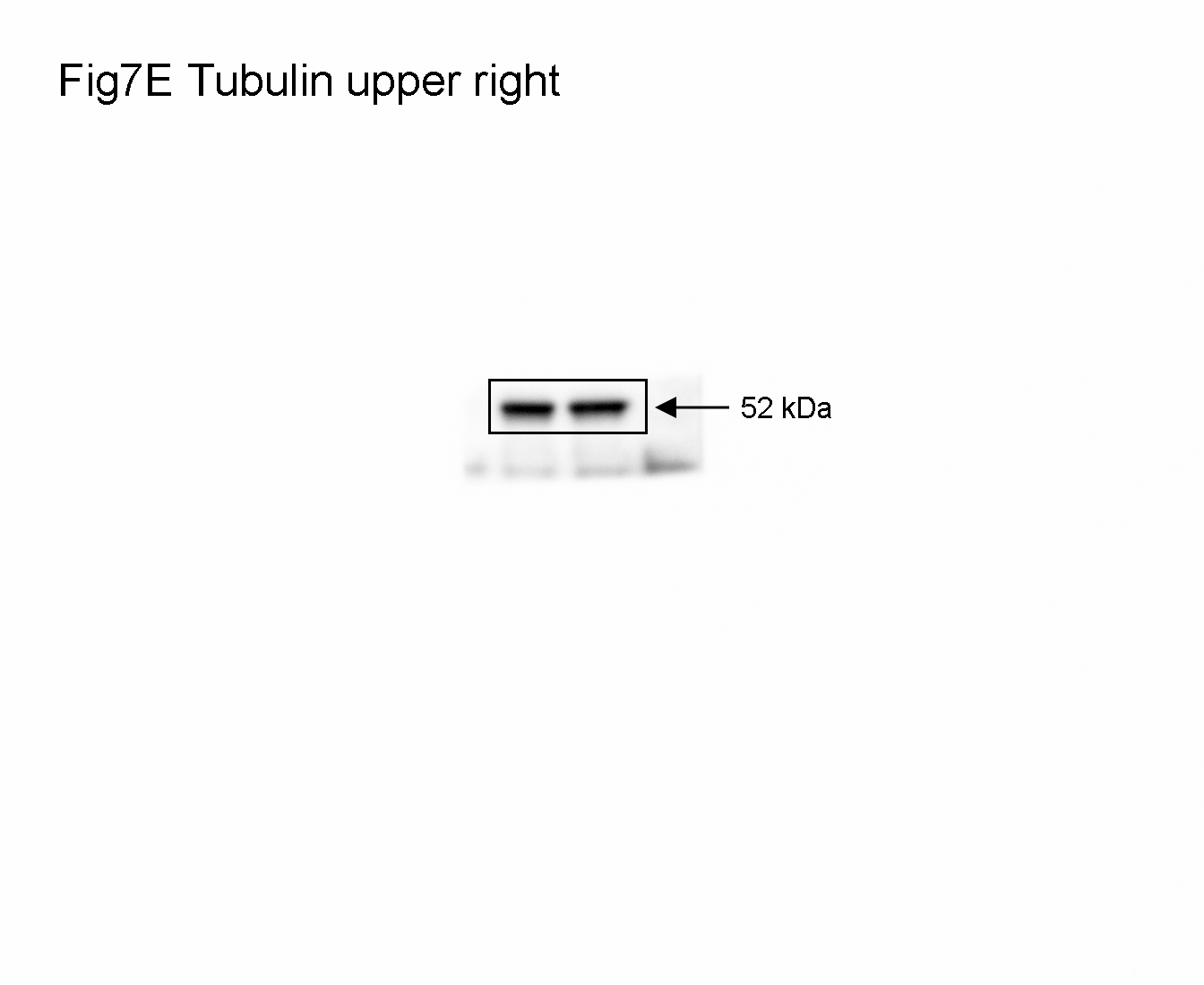

Supplement: Figure 7—source data 2. [file elife-98524-fig7-data2.zip › Fig 7-data2-v1/7E/Tubulin upper right.tif]

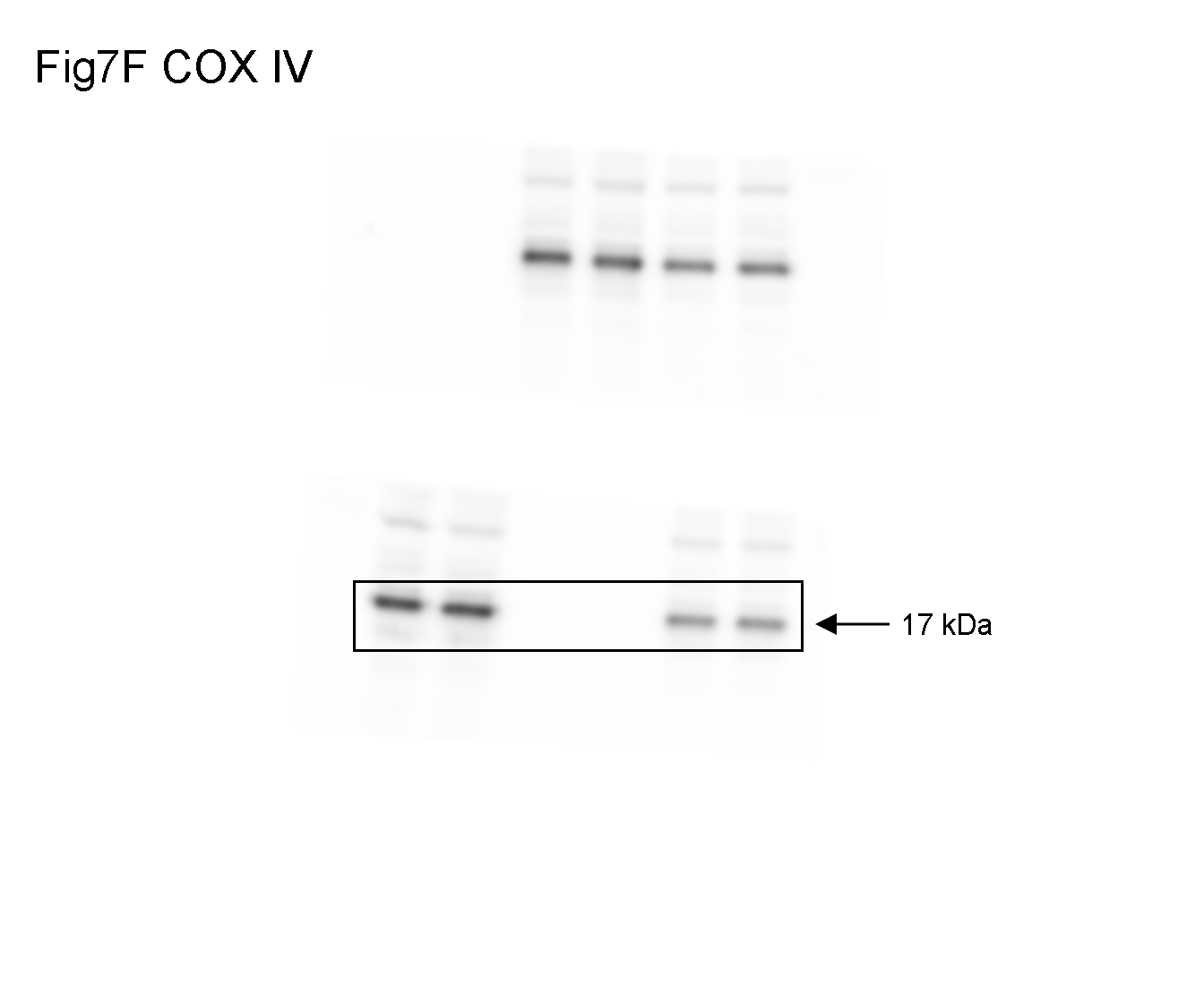

Supplement: Figure 7—source data 2. [file elife-98524-fig7-data2.zip › Fig 7-data2-v1/7F/COX IV.tif]

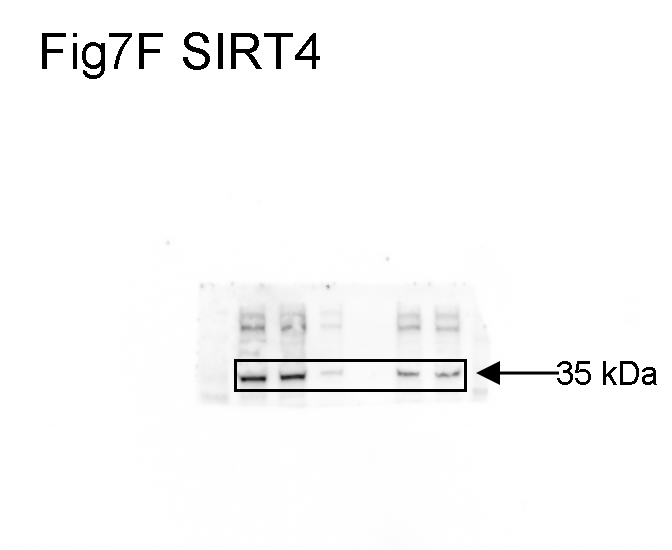

Supplement: Figure 7—source data 2. [file elife-98524-fig7-data2.zip › Fig 7-data2-v1/7F/SIRT4.tif]

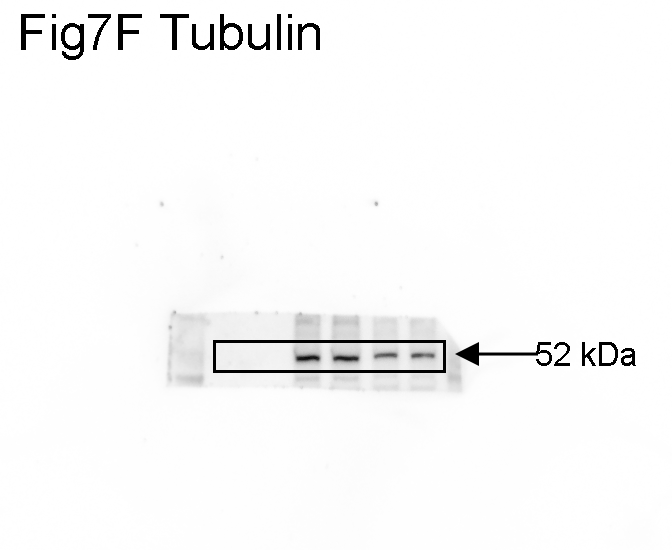

Supplement: Figure 7—source data 2. [file elife-98524-fig7-data2.zip › Fig 7-data2-v1/7F/Tubulin.tif]

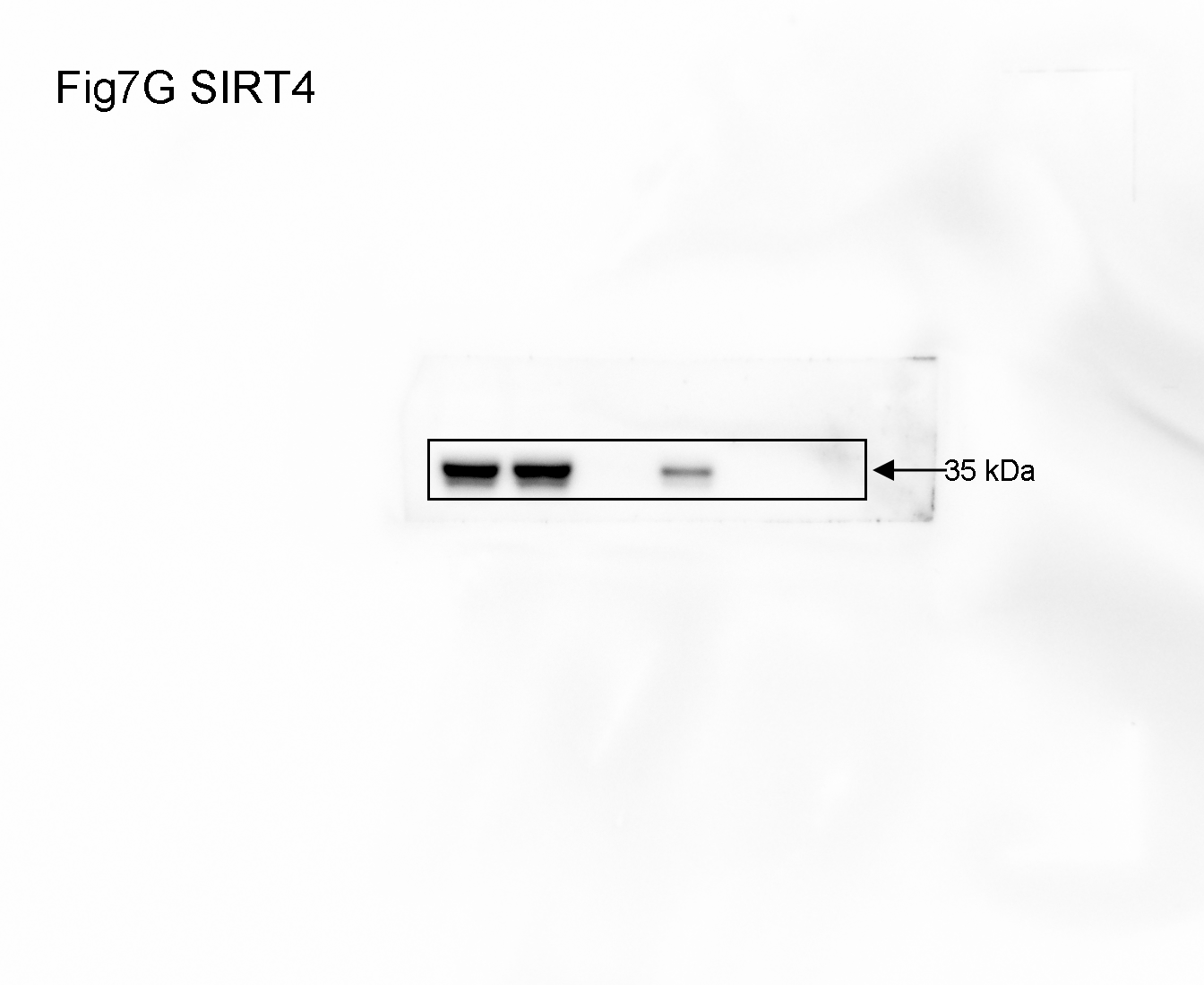

Supplement: Figure 7—source data 2. [file elife-98524-fig7-data2.zip › Fig 7-data2-v1/7G/SIRT4.tif]

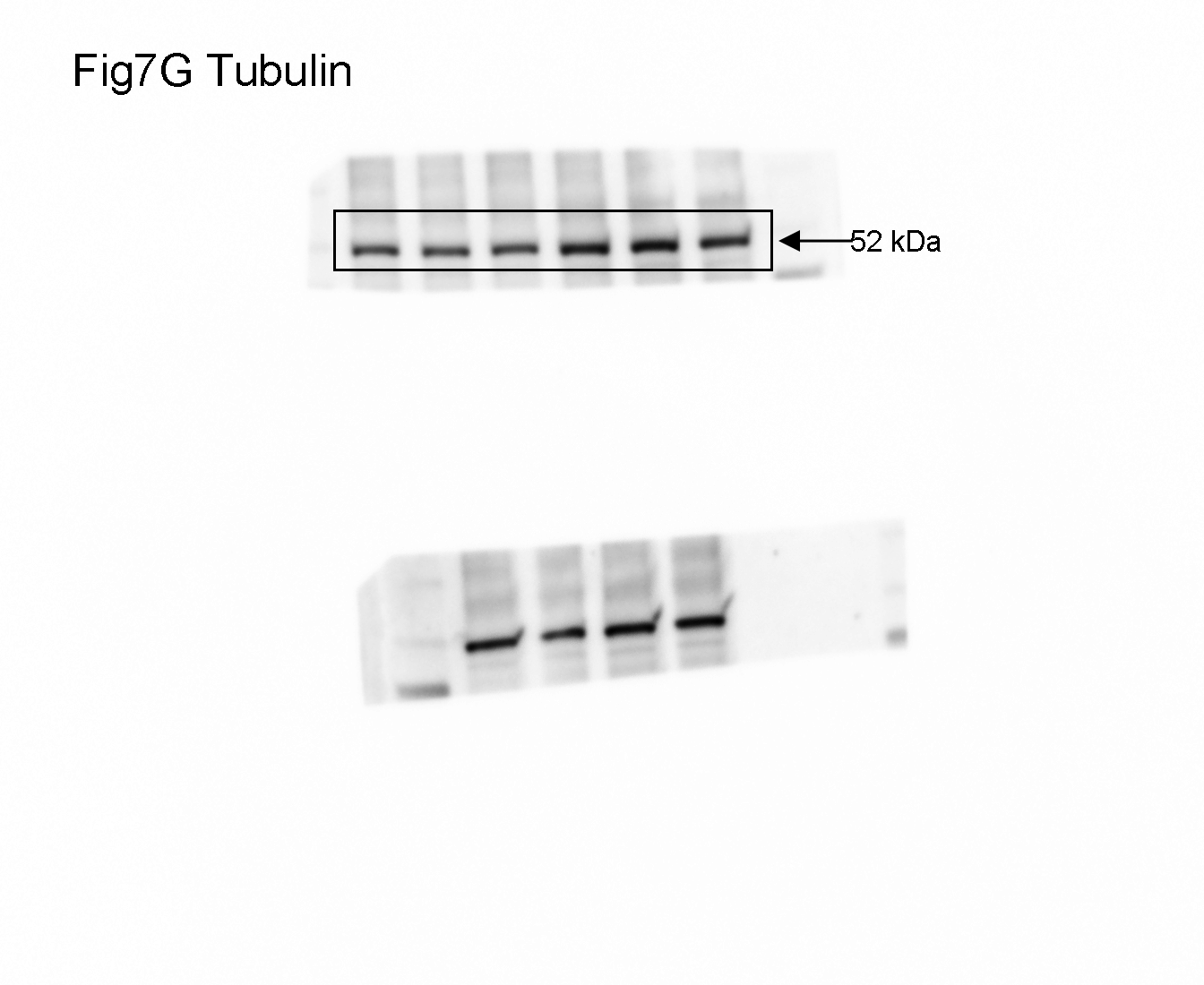

Supplement: Figure 7—source data 2. [file elife-98524-fig7-data2.zip › Fig 7-data2-v1/7G/Tubulin.tif]

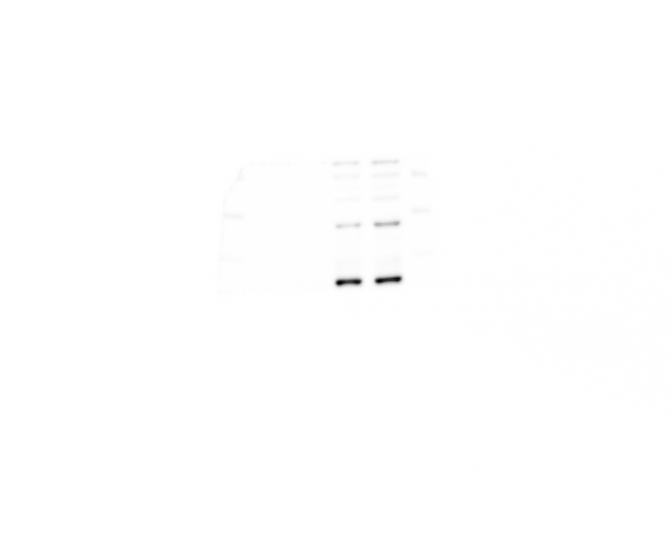

Supplement: Figure 8—source data 1. [file elife-98524-fig8-data1.zip › Fig 8-data1-v1/8A/bottom/COX IV.tif]

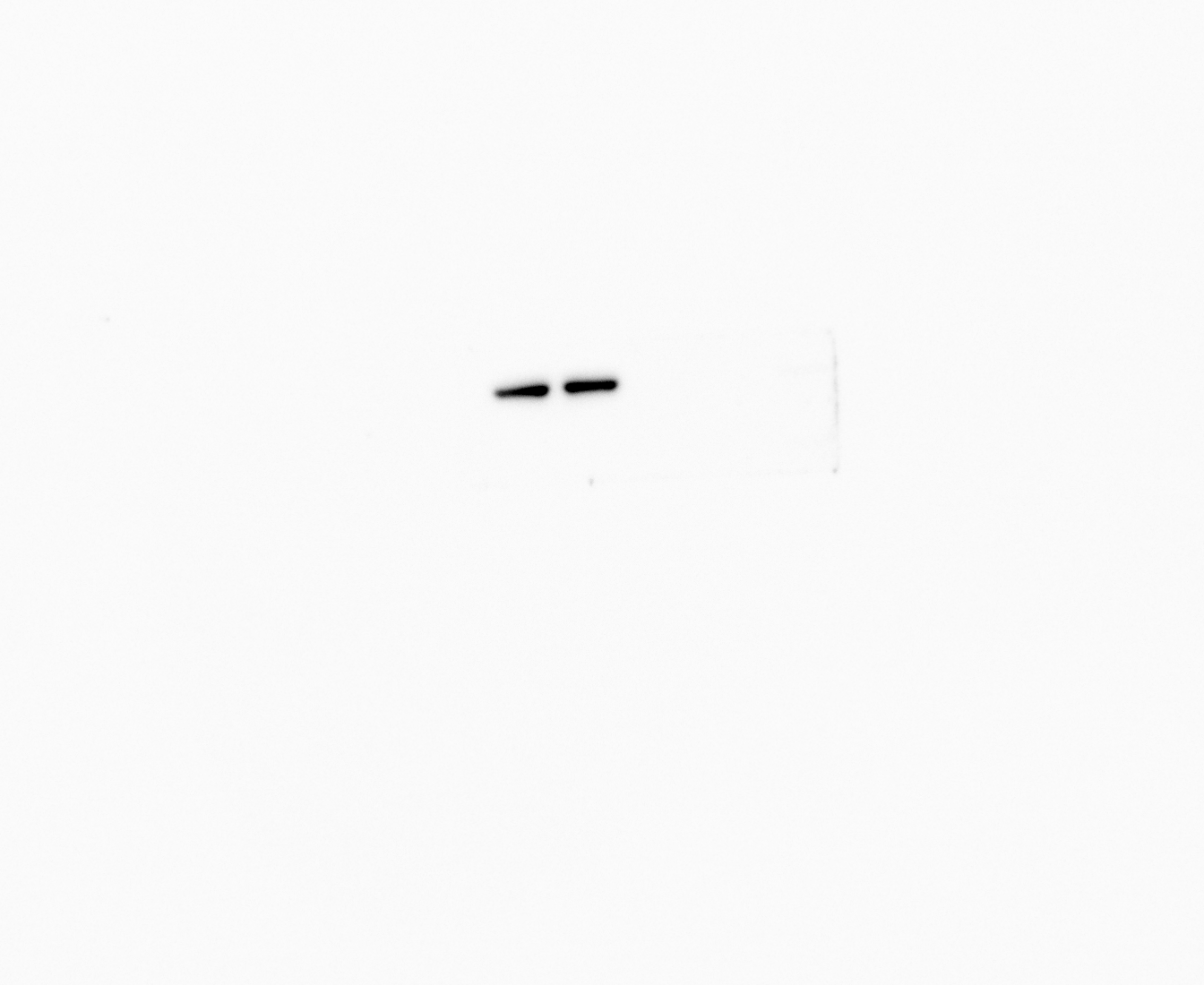

Supplement: Figure 8—source data 1. [file elife-98524-fig8-data1.zip › Fig 8-data1-v1/8A/bottom/PCNA.tif]

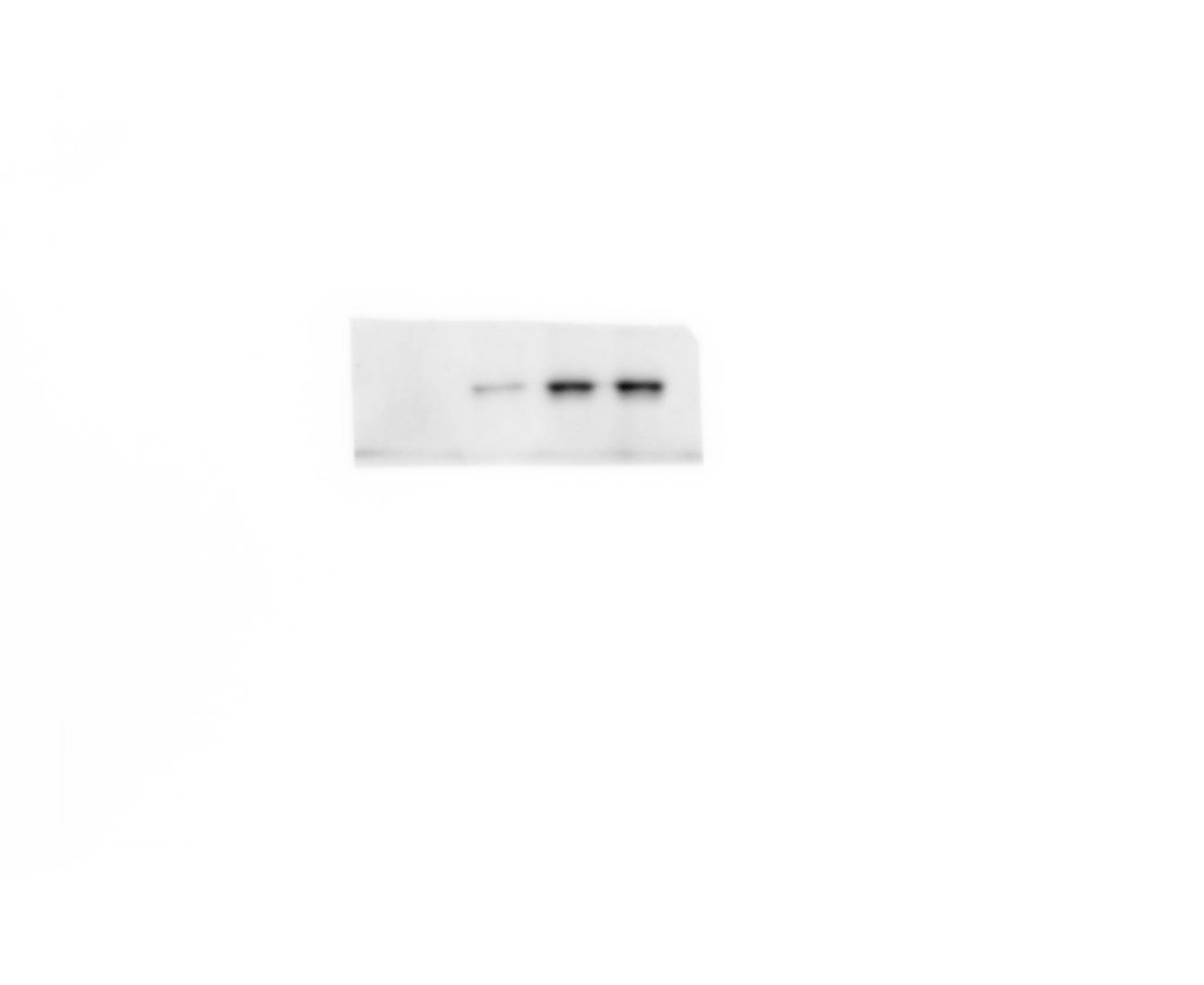

Supplement: Figure 8—source data 1. [file elife-98524-fig8-data1.zip › Fig 8-data1-v1/8A/bottom/SIRT4.tif]

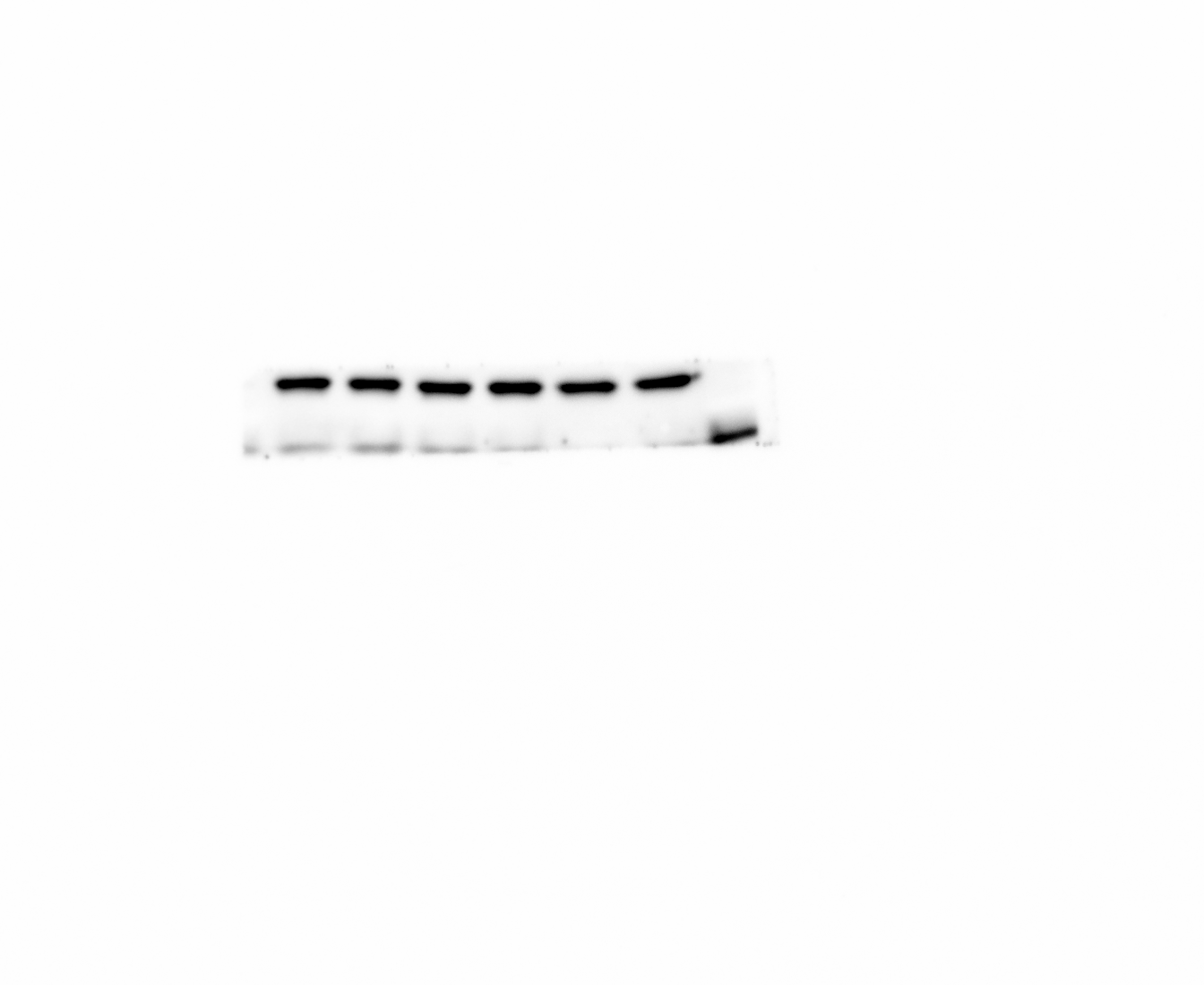

Supplement: Figure 8—source data 1. [file elife-98524-fig8-data1.zip › Fig 8-data1-v1/8A/upper/PCNA.tif]

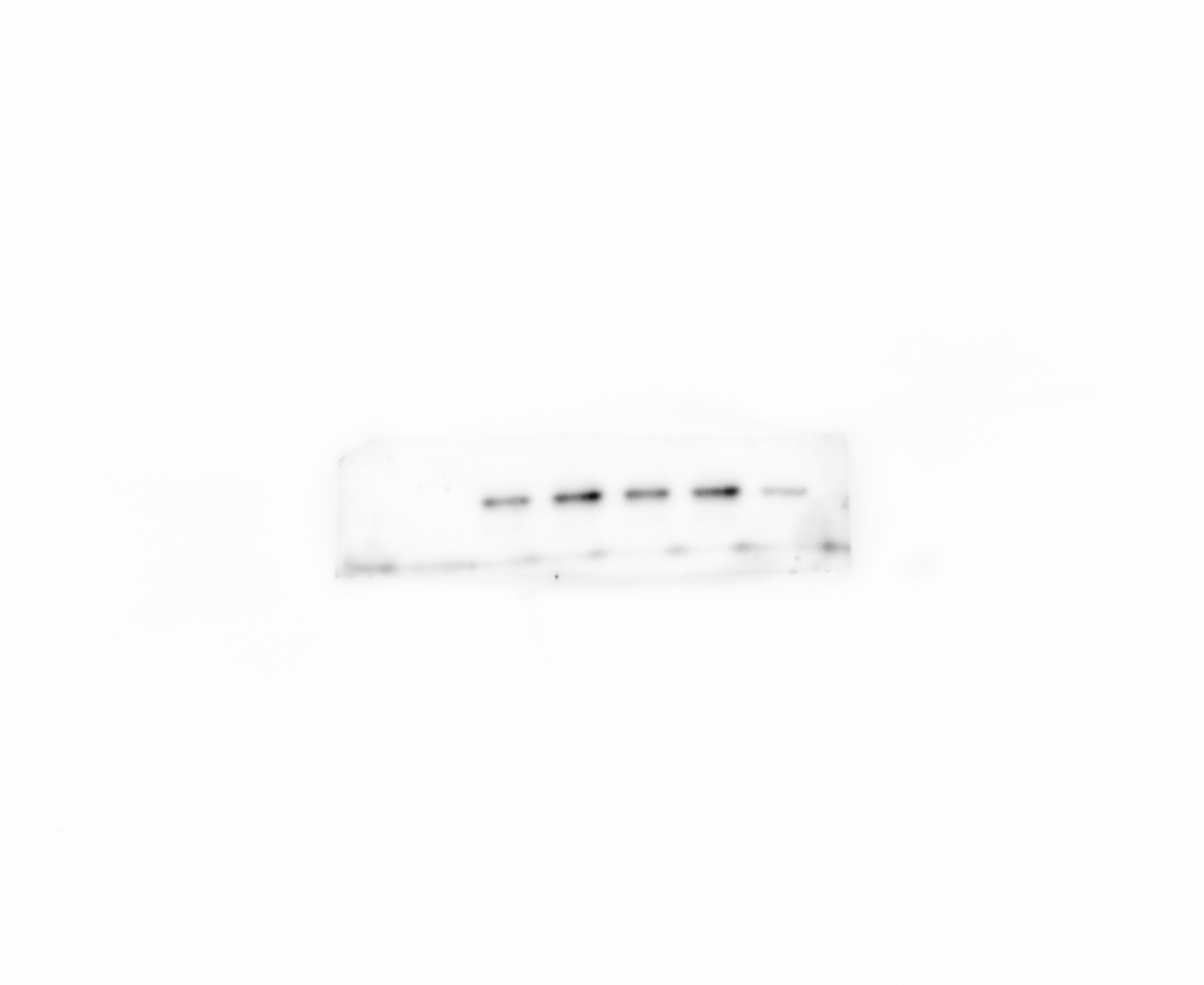

Supplement: Figure 8—source data 1. [file elife-98524-fig8-data1.zip › Fig 8-data1-v1/8A/upper/SIRT4.tif]

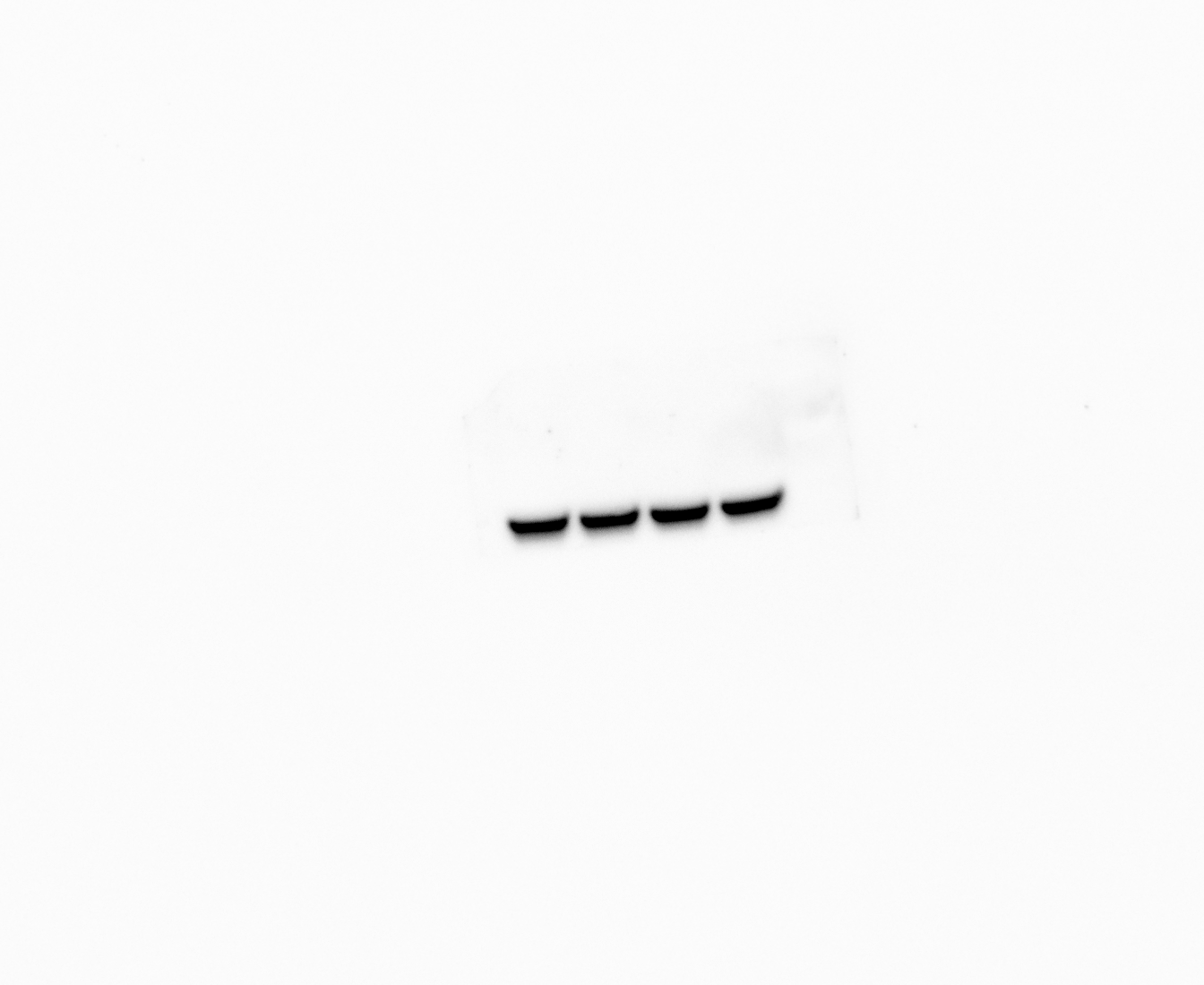

Supplement: Figure 8—source data 1. [file elife-98524-fig8-data1.zip › Fig 8-data1-v1/8C/bottom left/Flag.tif]

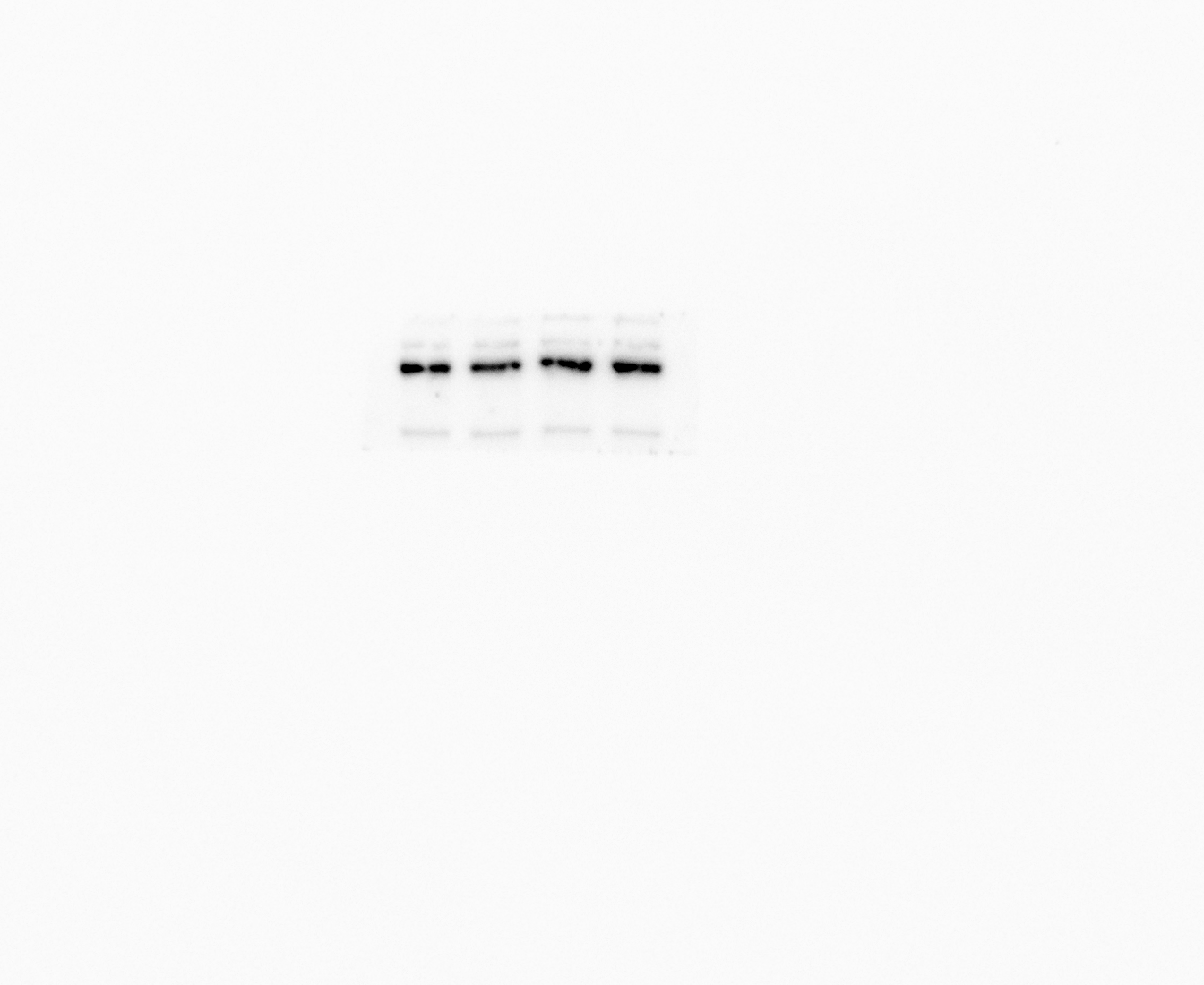

Supplement: Figure 8—source data 1. [file elife-98524-fig8-data1.zip › Fig 8-data1-v1/8C/bottom left/Tubulin.tif]

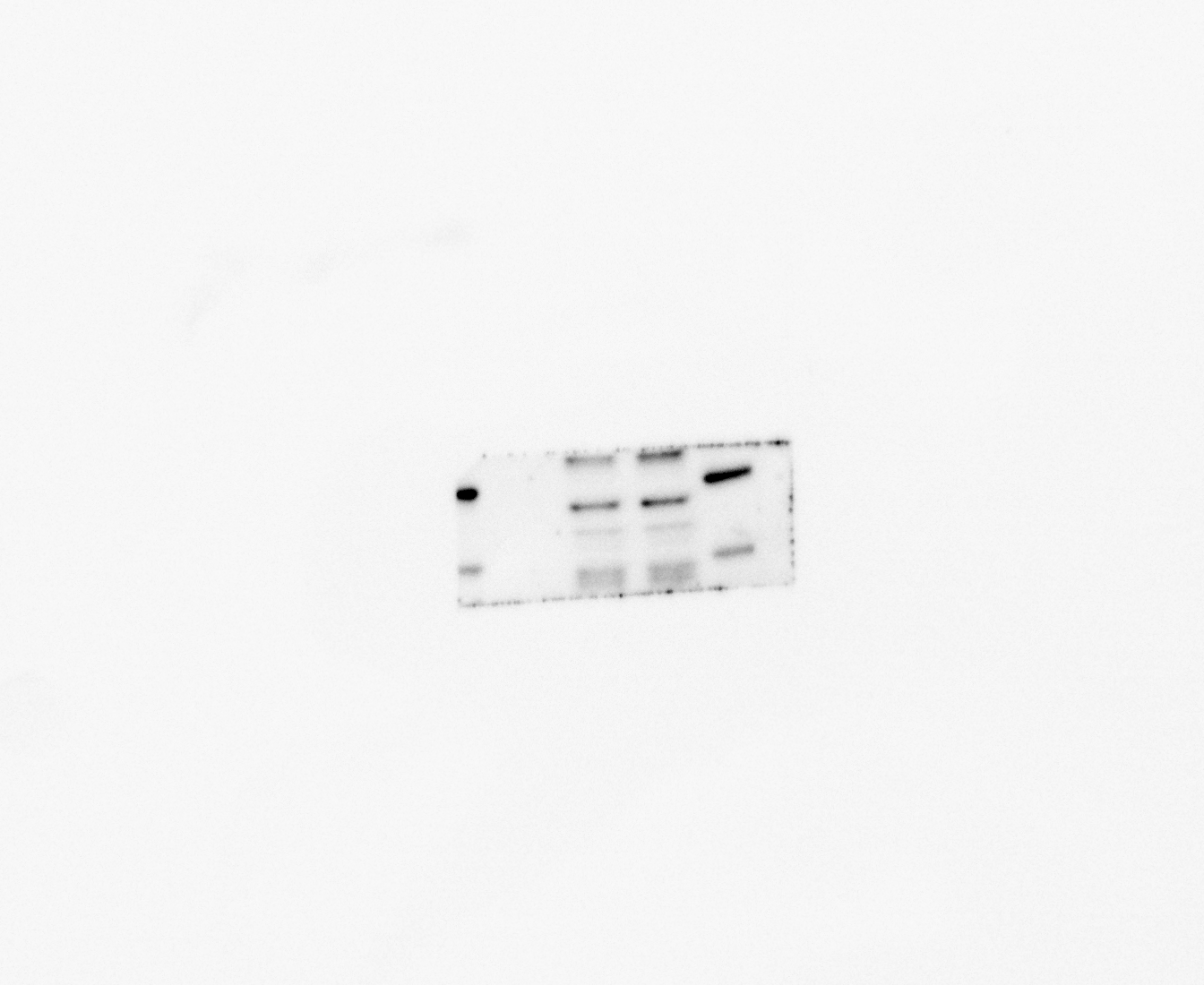

Supplement: Figure 8—source data 1. [file elife-98524-fig8-data1.zip › Fig 8-data1-v1/8C/bottom right/Flag.tif]

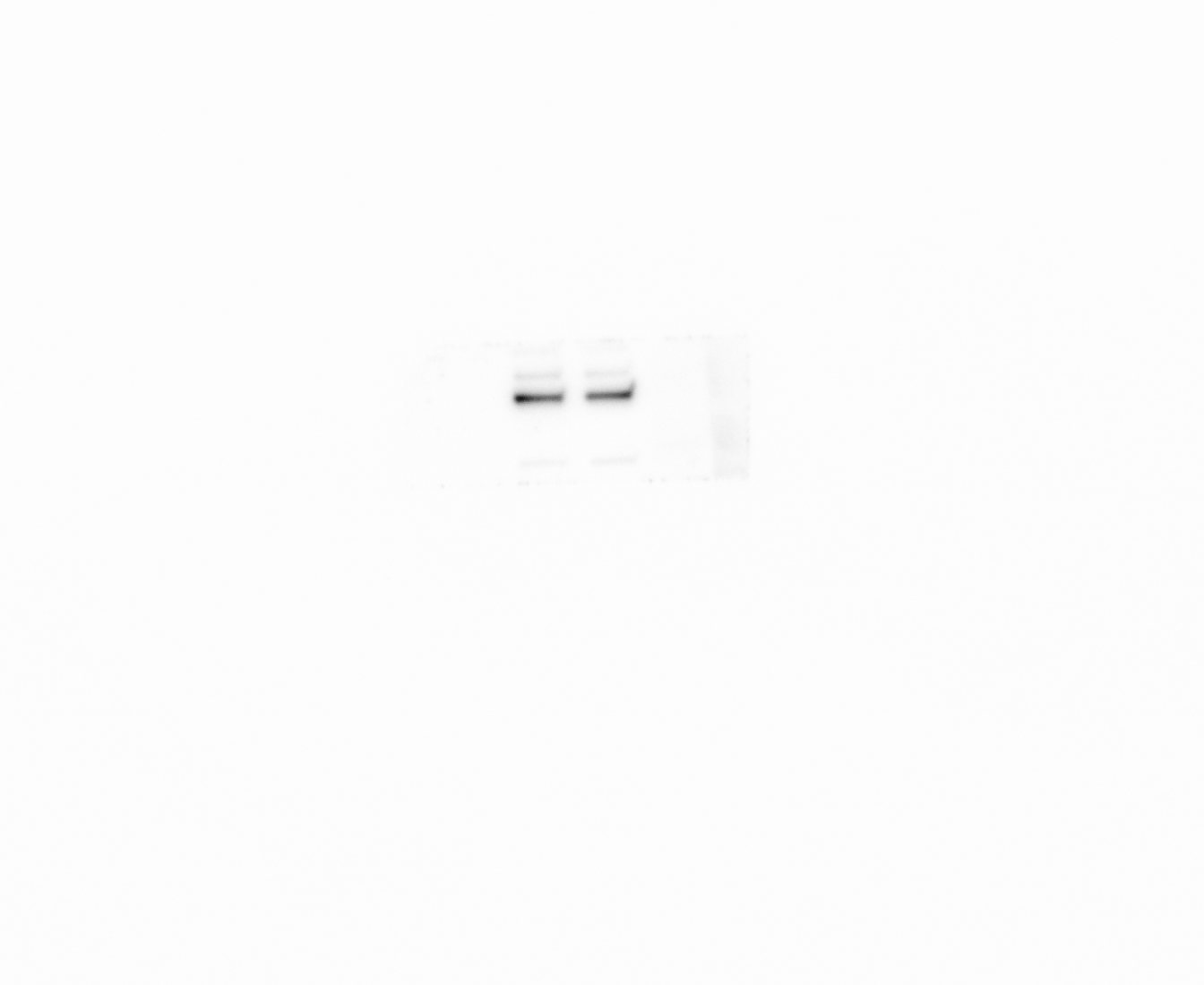

Supplement: Figure 8—source data 1. [file elife-98524-fig8-data1.zip › Fig 8-data1-v1/8C/bottom right/HA.tif]

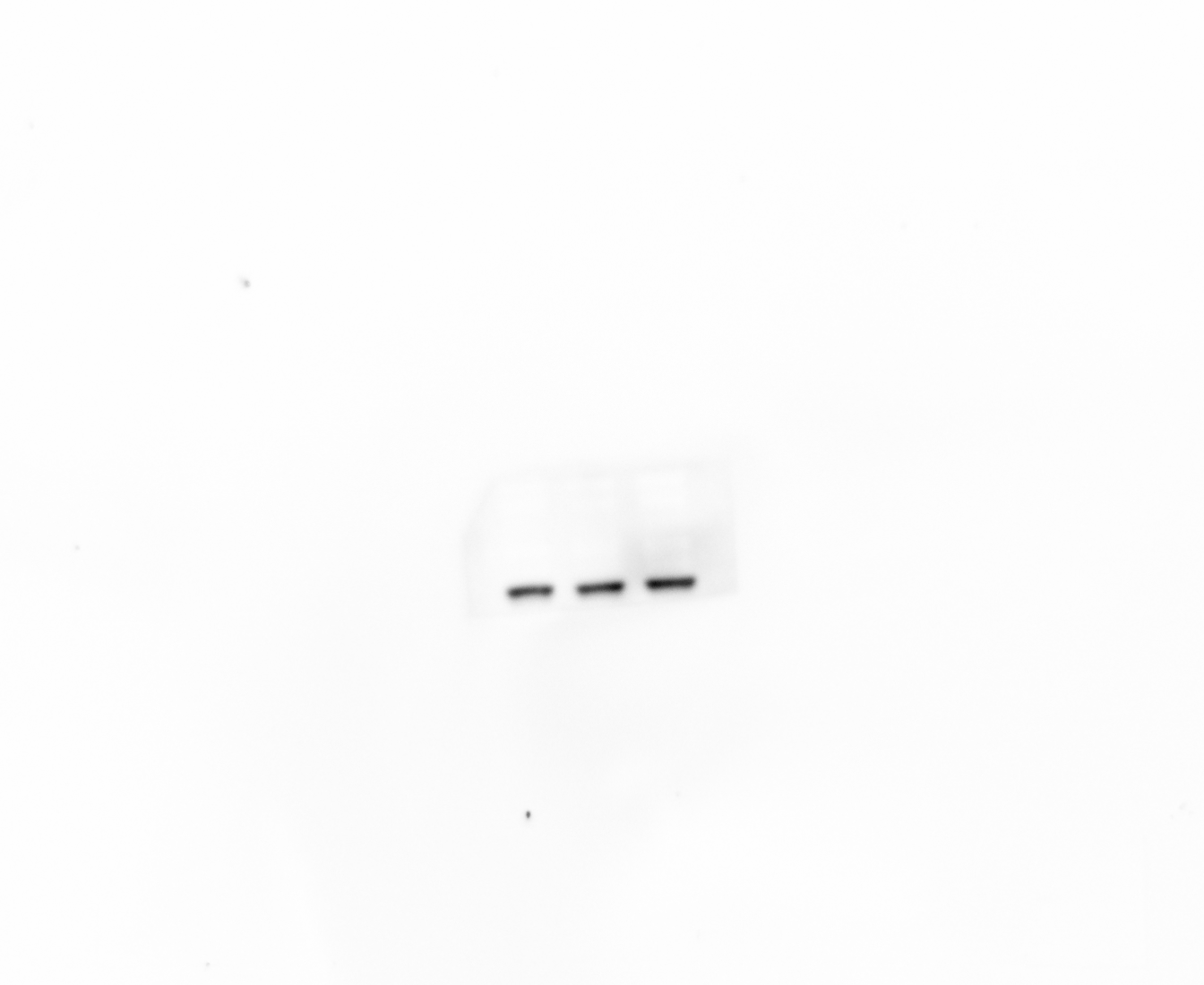

Supplement: Figure 8—source data 1. [file elife-98524-fig8-data1.zip › Fig 8-data1-v1/8C/bottom right/SIRT4.tif]

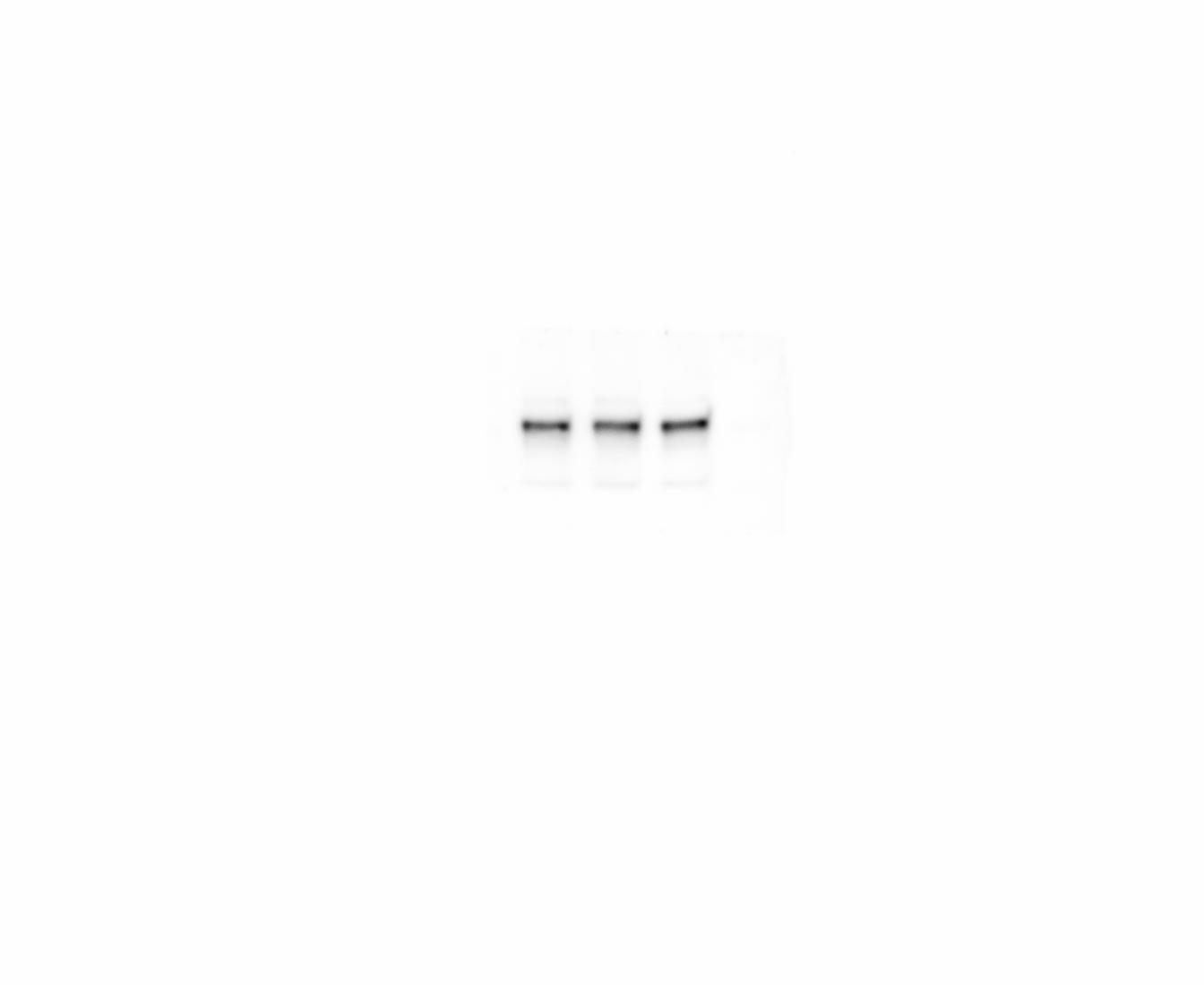

Supplement: Figure 8—source data 1. [file elife-98524-fig8-data1.zip › Fig 8-data1-v1/8C/bottom right/Tubulin.tif]

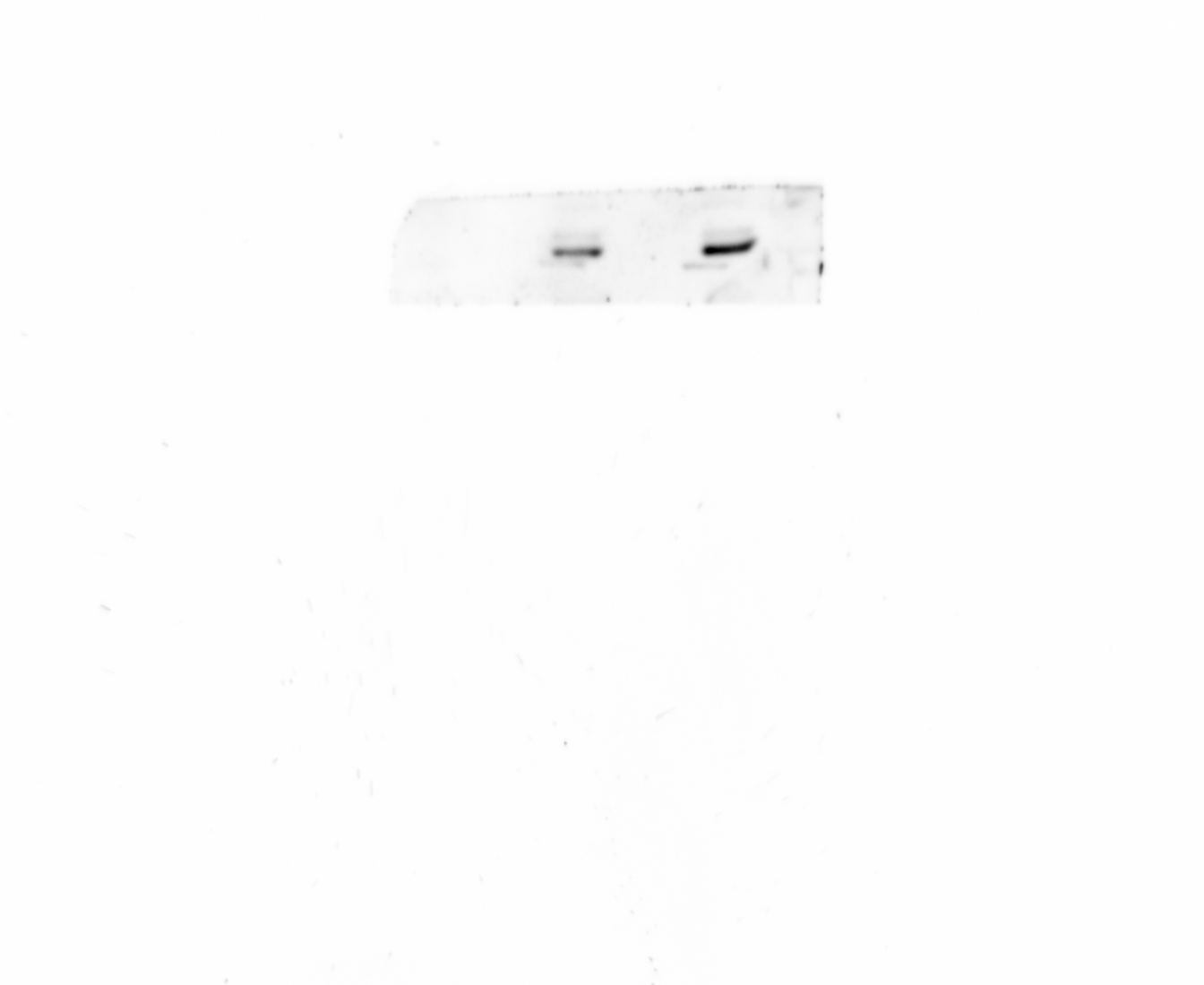

Supplement: Figure 8—source data 1. [file elife-98524-fig8-data1.zip › Fig 8-data1-v1/8C/middle left/SIRT4.tif]

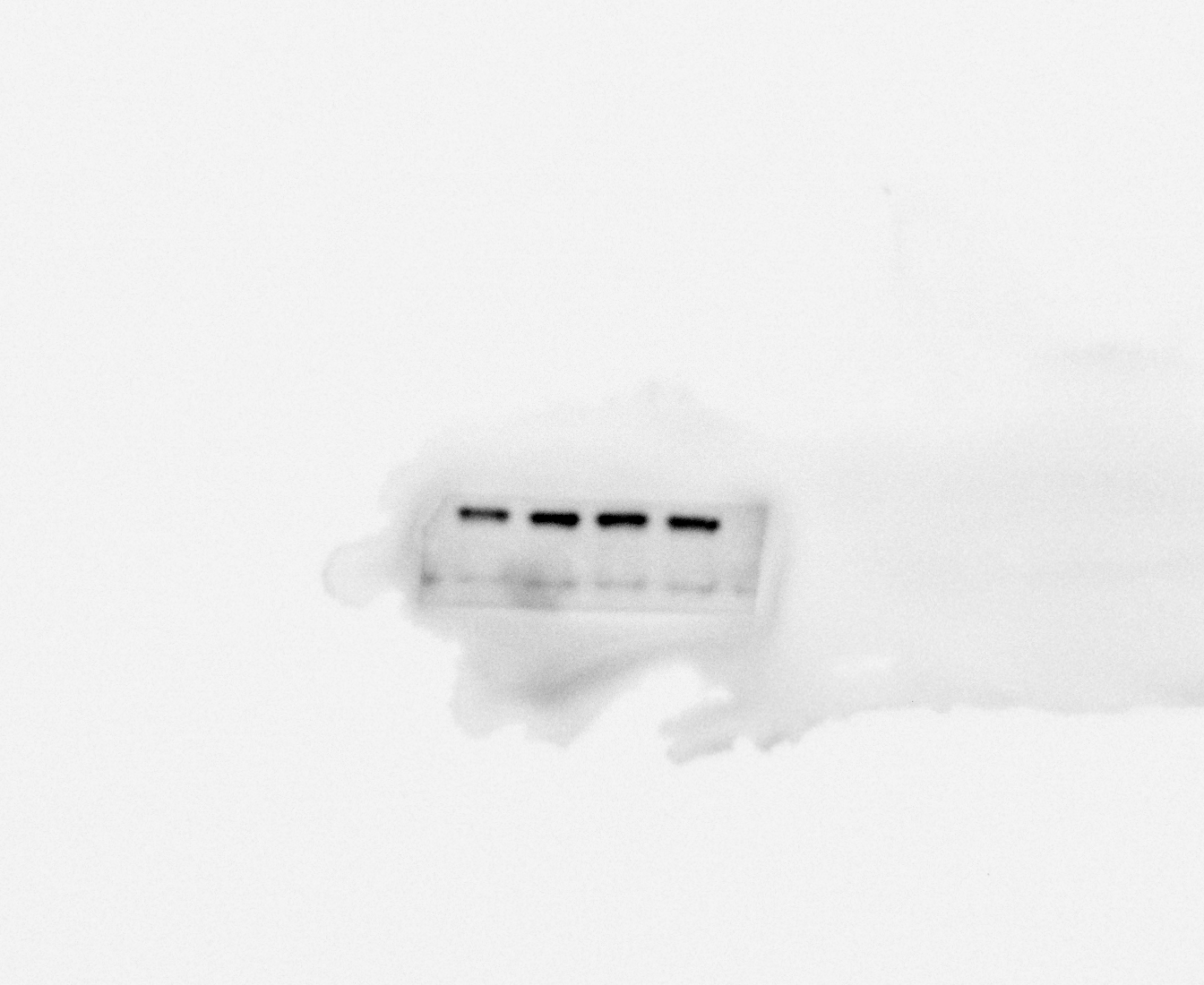

Supplement: Figure 8—source data 1. [file elife-98524-fig8-data1.zip › Fig 8-data1-v1/8C/middle left/Tubulin.tif]

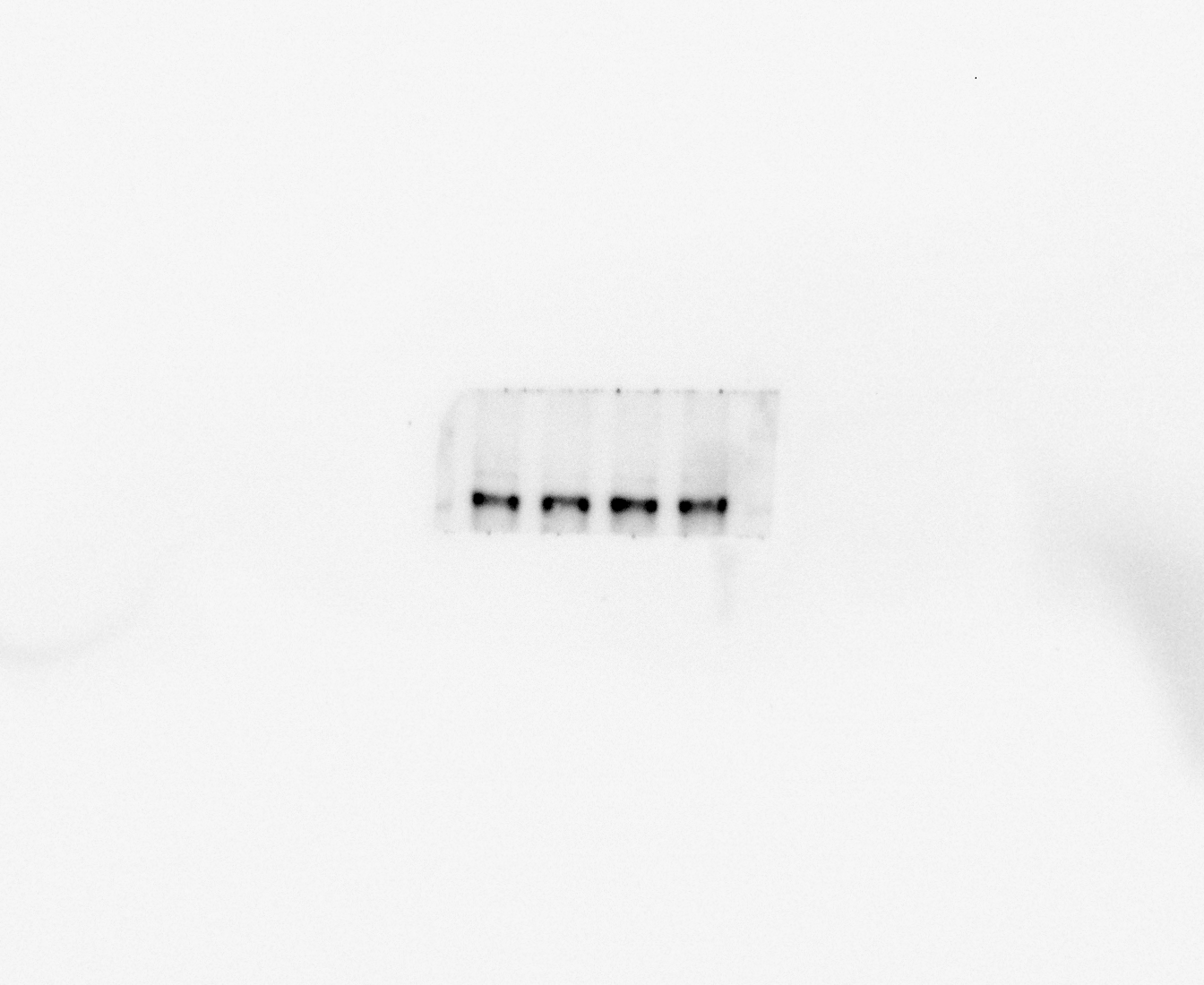

Supplement: Figure 8—source data 1. [file elife-98524-fig8-data1.zip › Fig 8-data1-v1/8C/upper left/PCNA.tif]

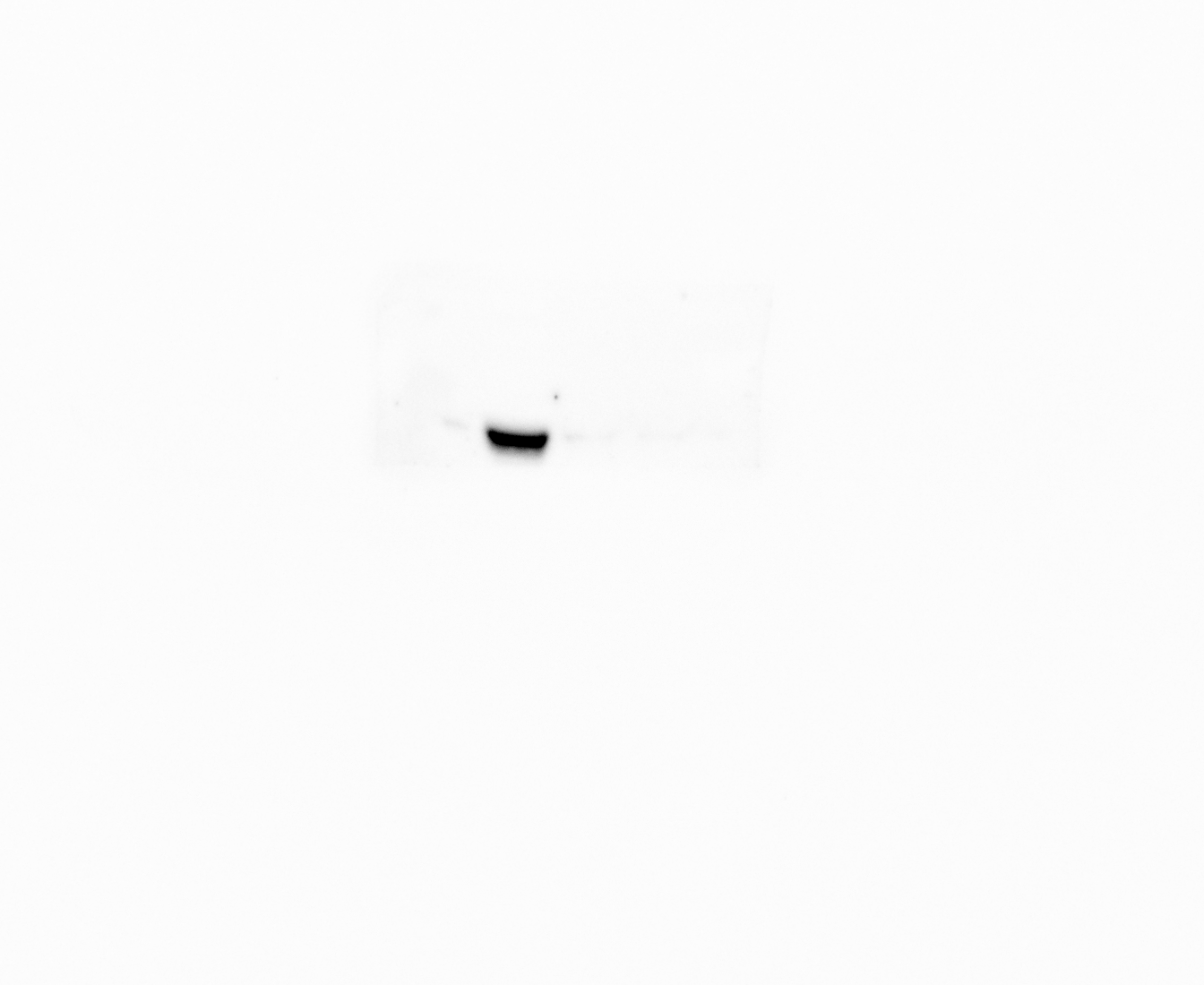

Supplement: Figure 8—source data 1. [file elife-98524-fig8-data1.zip › Fig 8-data1-v1/8C/upper left/SIRT4.tif]

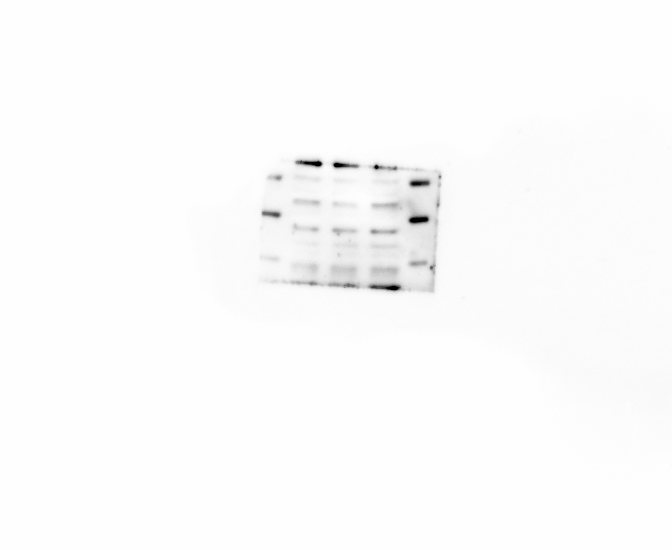

Supplement: Figure 8—source data 1. [file elife-98524-fig8-data1.zip › Fig 8-data1-v1/8C/upper right/PCNA.tif]

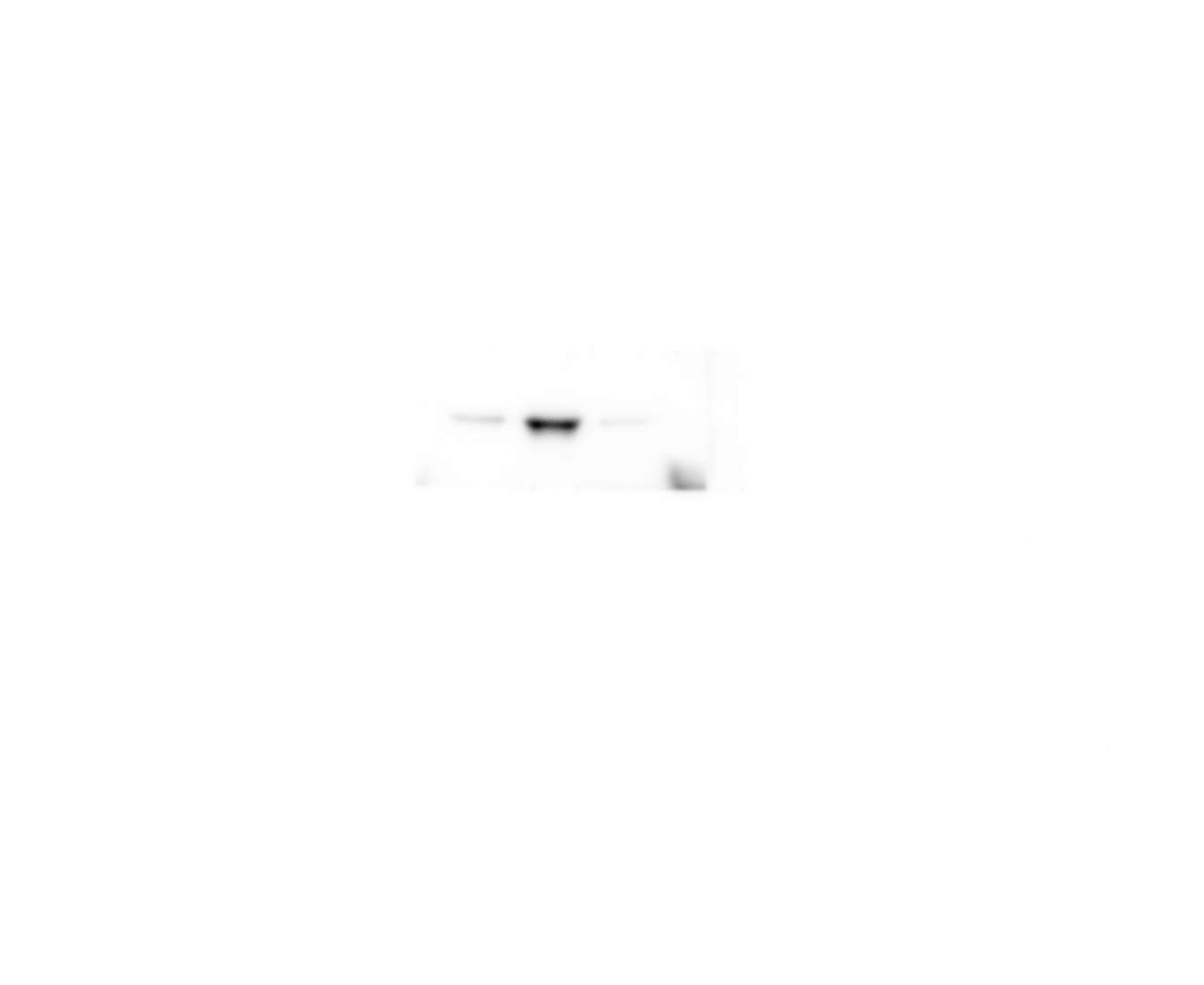

Supplement: Figure 8—source data 1. [file elife-98524-fig8-data1.zip › Fig 8-data1-v1/8C/upper right/SIRT4.tif]

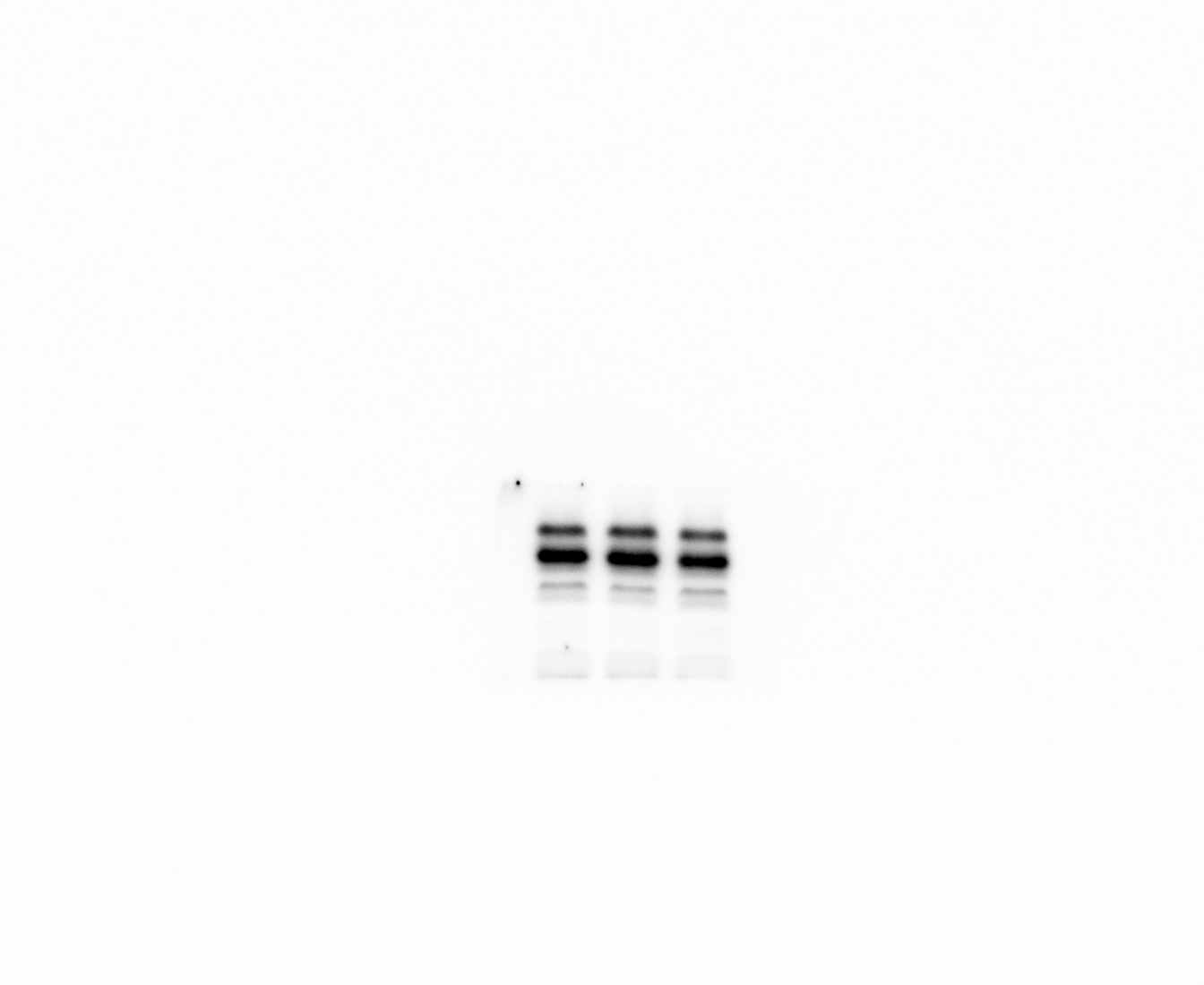

Supplement: Figure 8—source data 1. [file elife-98524-fig8-data1.zip › Fig 8-data1-v1/8D/bottom/ERK1 2.tif]

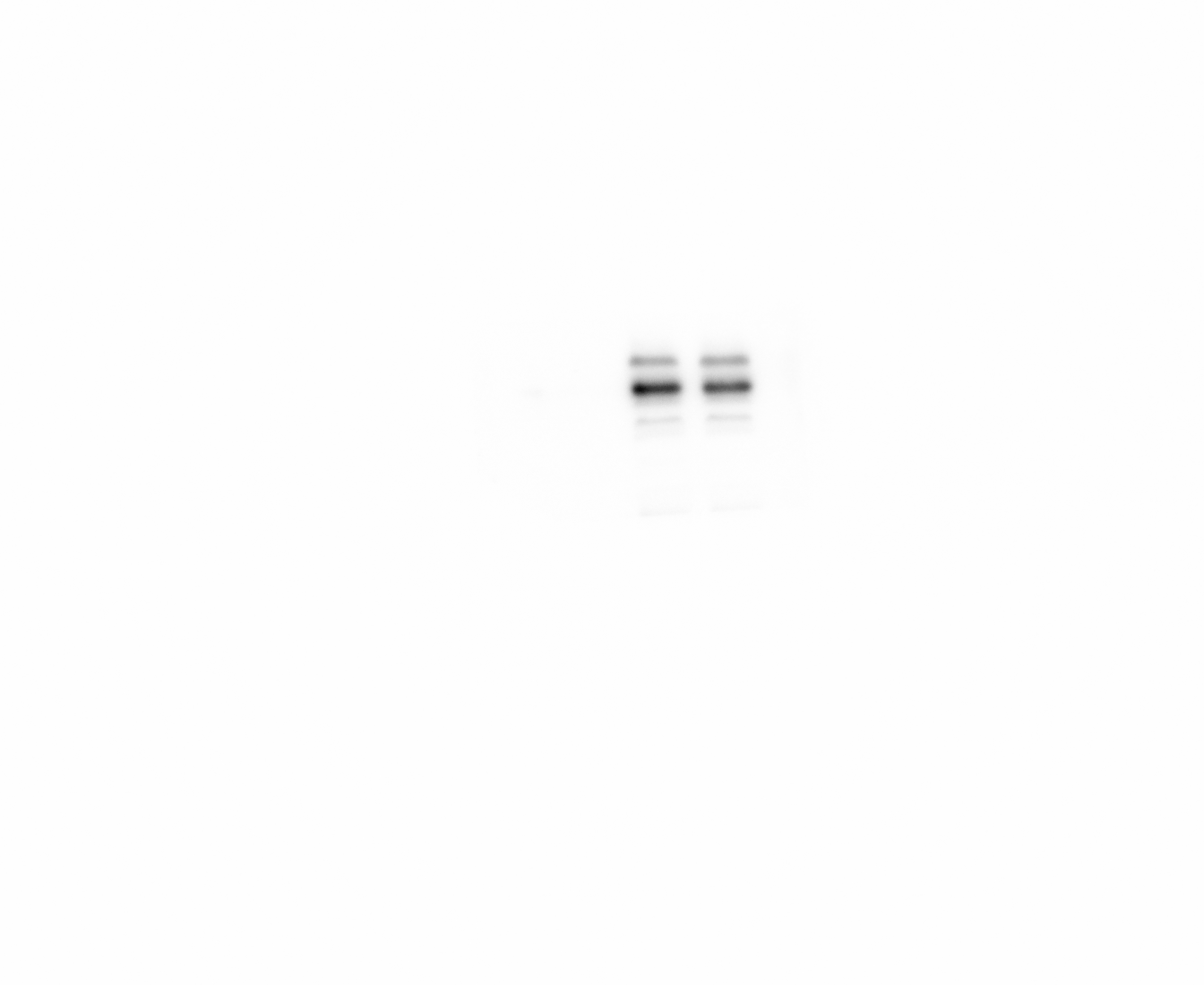

Supplement: Figure 8—source data 1. [file elife-98524-fig8-data1.zip › Fig 8-data1-v1/8D/bottom/pERK1 2.tif]

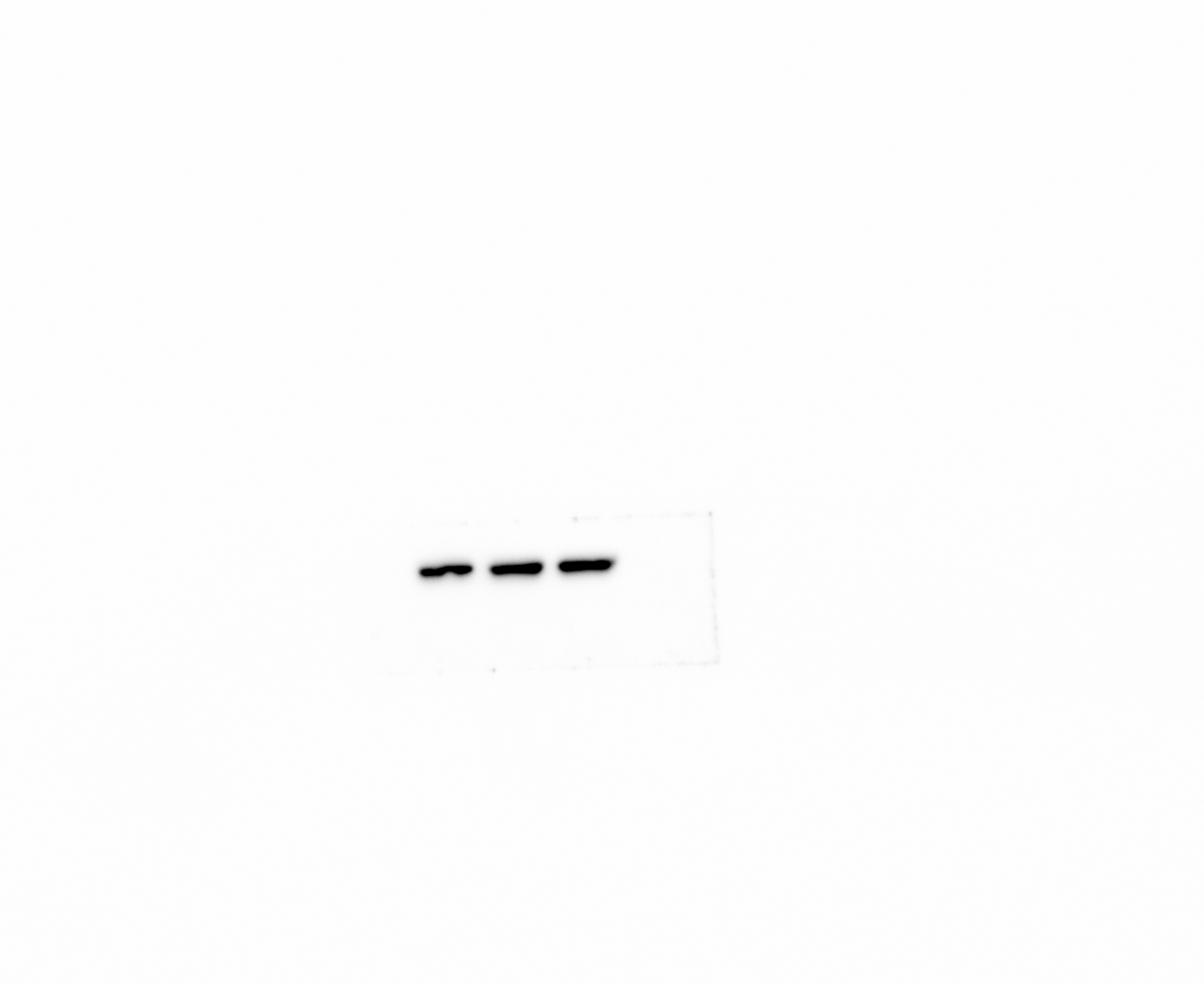

Supplement: Figure 8—source data 1. [file elife-98524-fig8-data1.zip › Fig 8-data1-v1/8D/bottom/SIRT4.tif]

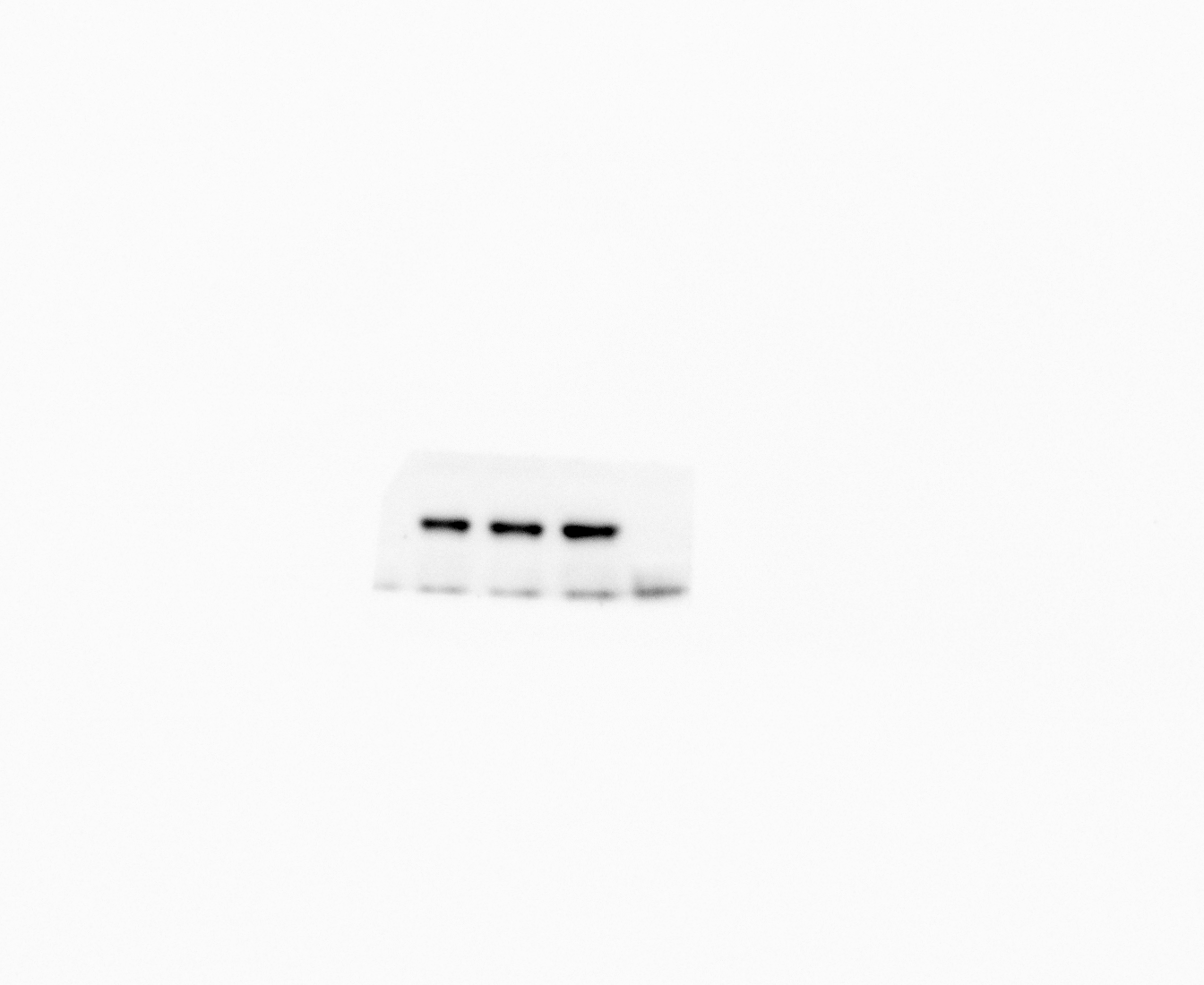

Supplement: Figure 8—source data 1. [file elife-98524-fig8-data1.zip › Fig 8-data1-v1/8D/bottom/Tubulin.tif]

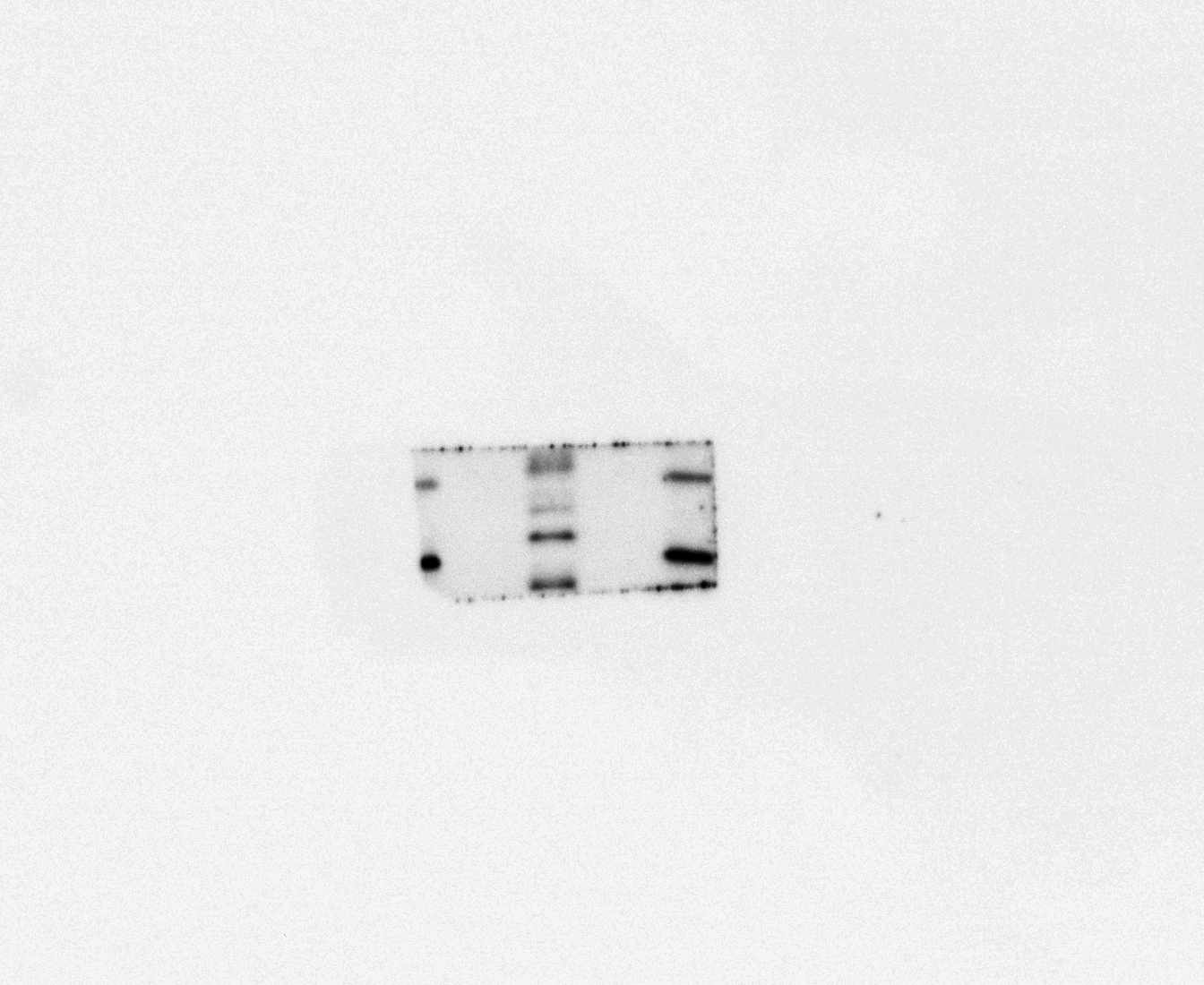

Supplement: Figure 8—source data 1. [file elife-98524-fig8-data1.zip › Fig 8-data1-v1/8D/upper/ERK1 2 .tif]

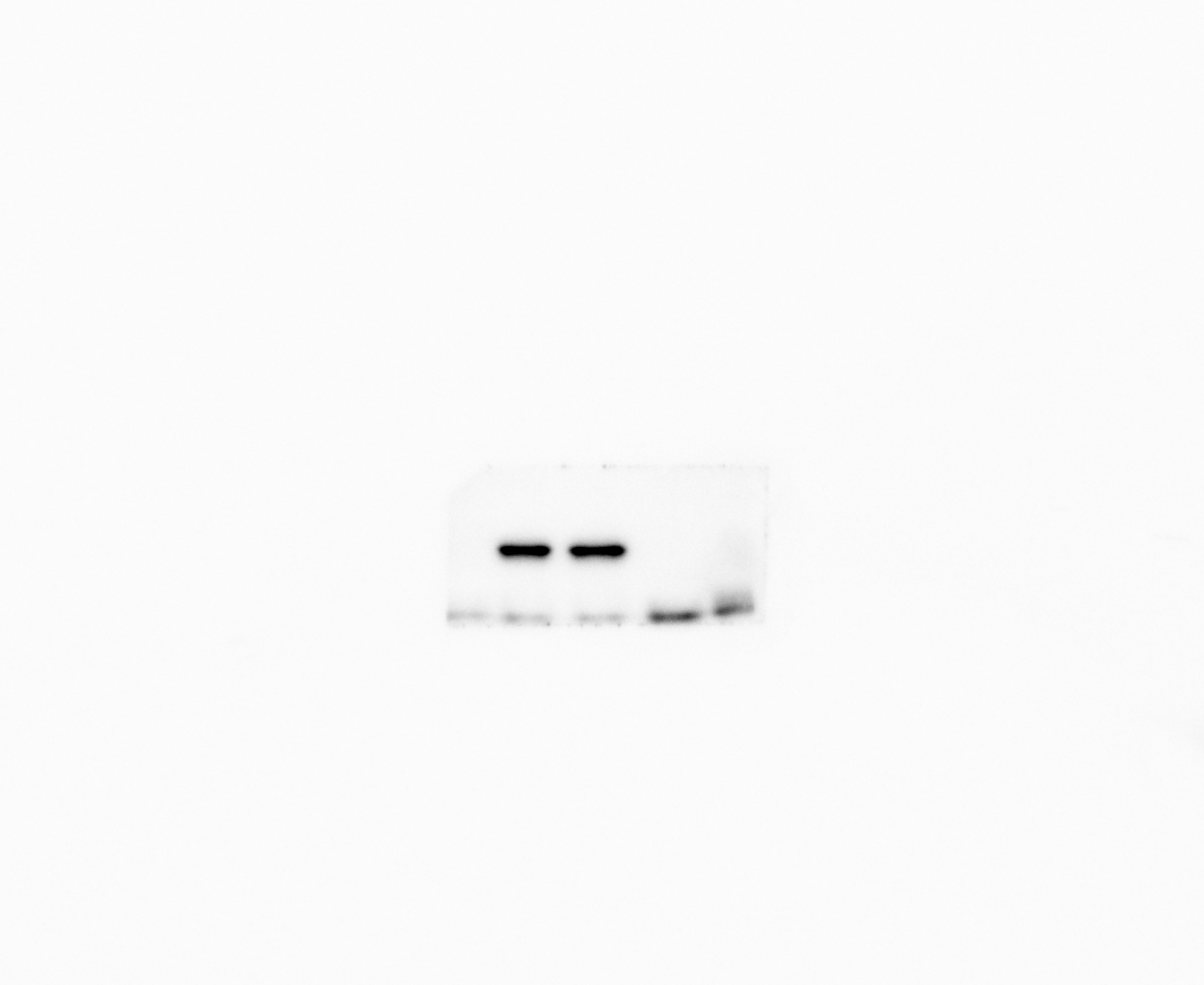

Supplement: Figure 8—source data 1. [file elife-98524-fig8-data1.zip › Fig 8-data1-v1/8D/upper/SIRT4.tif]

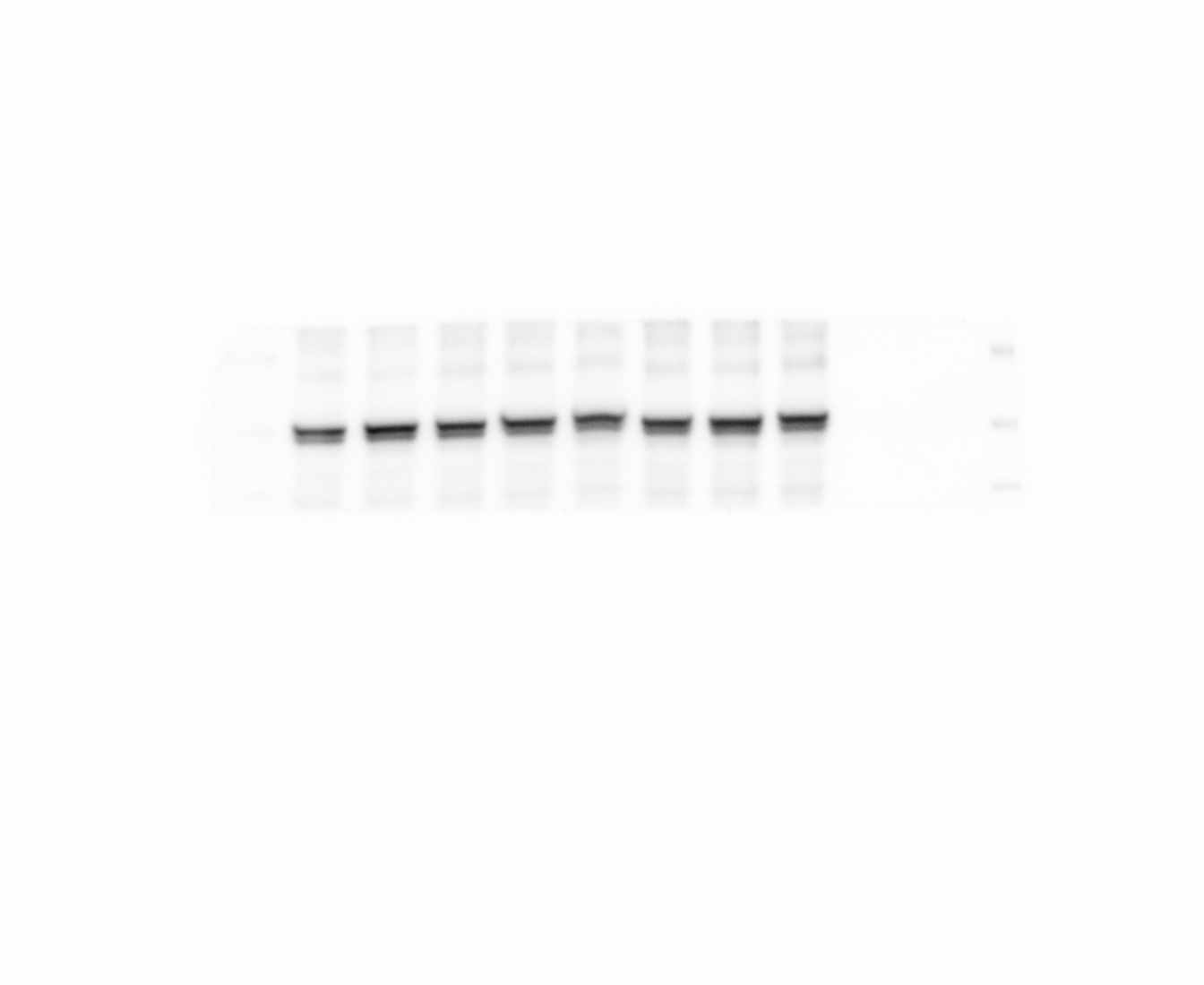

Supplement: Figure 8—source data 1. [file elife-98524-fig8-data1.zip › Fig 8-data1-v1/8E/bottom/Flag.tif]

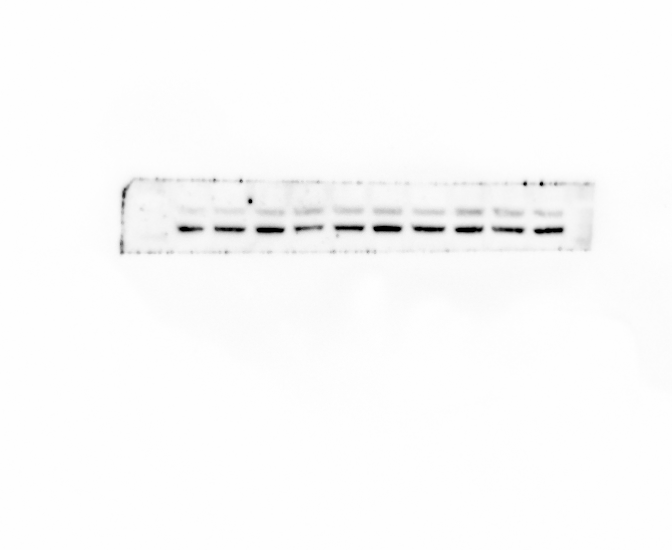

Supplement: Figure 8—source data 1. [file elife-98524-fig8-data1.zip › Fig 8-data1-v1/8E/bottom/SIRT4.tif]

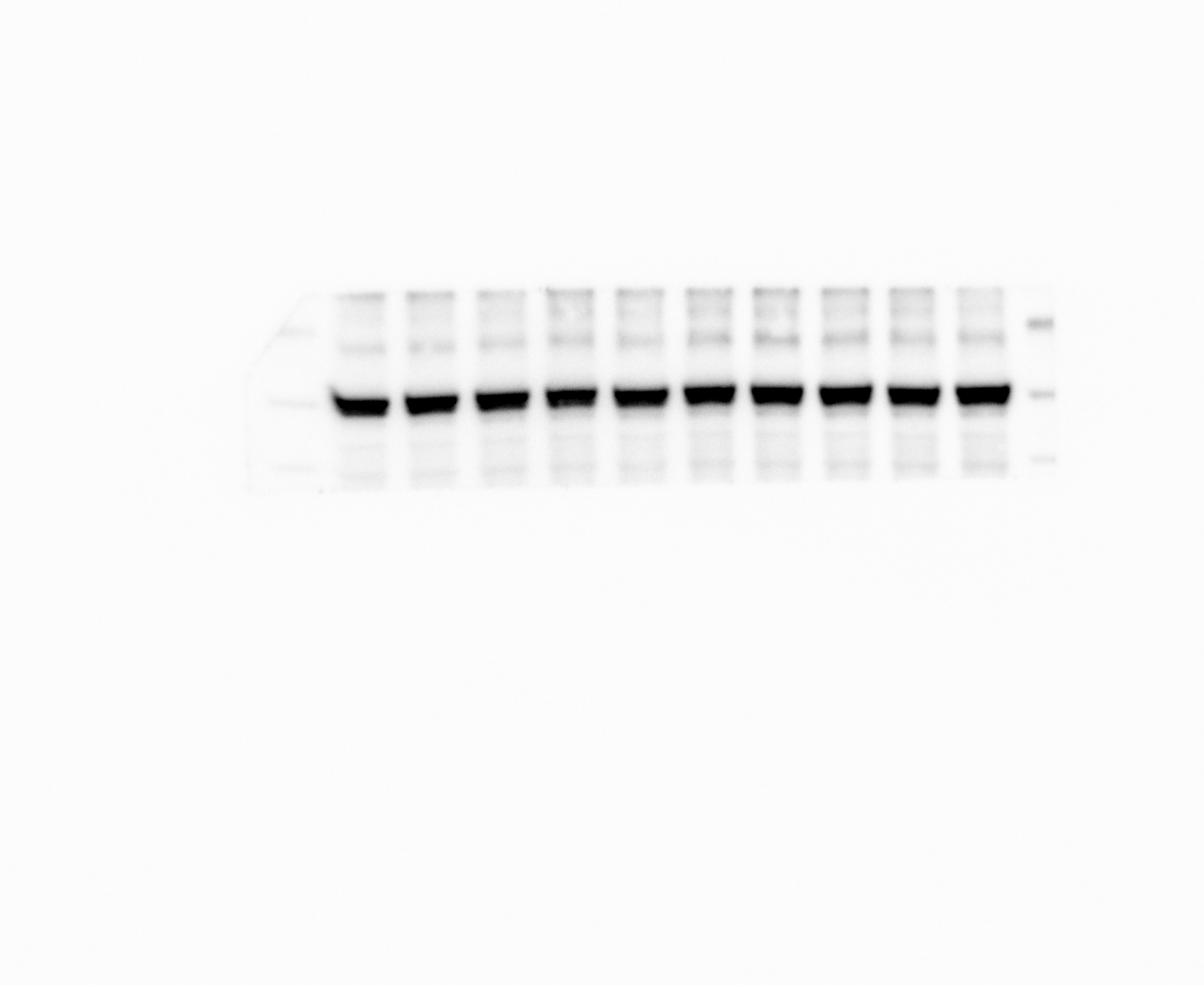

Supplement: Figure 8—source data 1. [file elife-98524-fig8-data1.zip › Fig 8-data1-v1/8E/bottom/Tubulin.tif]

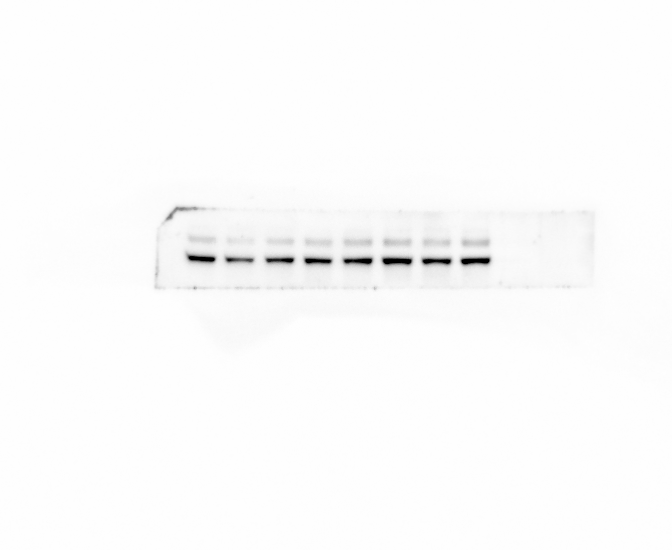

Supplement: Figure 8—source data 1. [file elife-98524-fig8-data1.zip › Fig 8-data1-v1/8E/upper/Flag.tif]

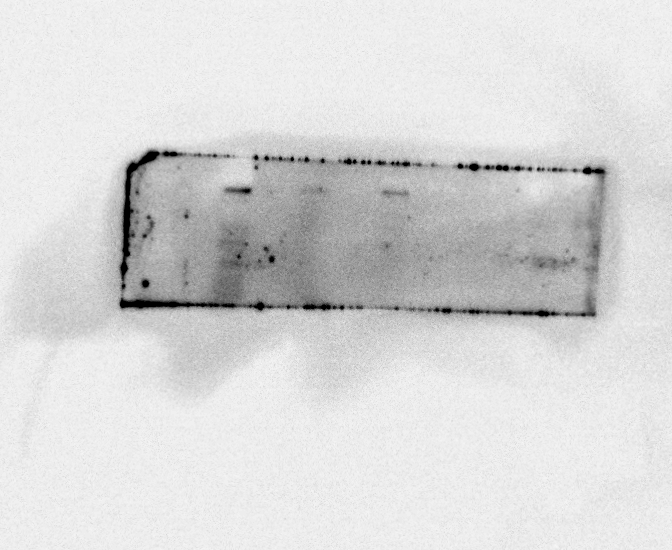

Supplement: Figure 8—source data 1. [file elife-98524-fig8-data1.zip › Fig 8-data1-v1/8E/upper/SIRT4.tif]

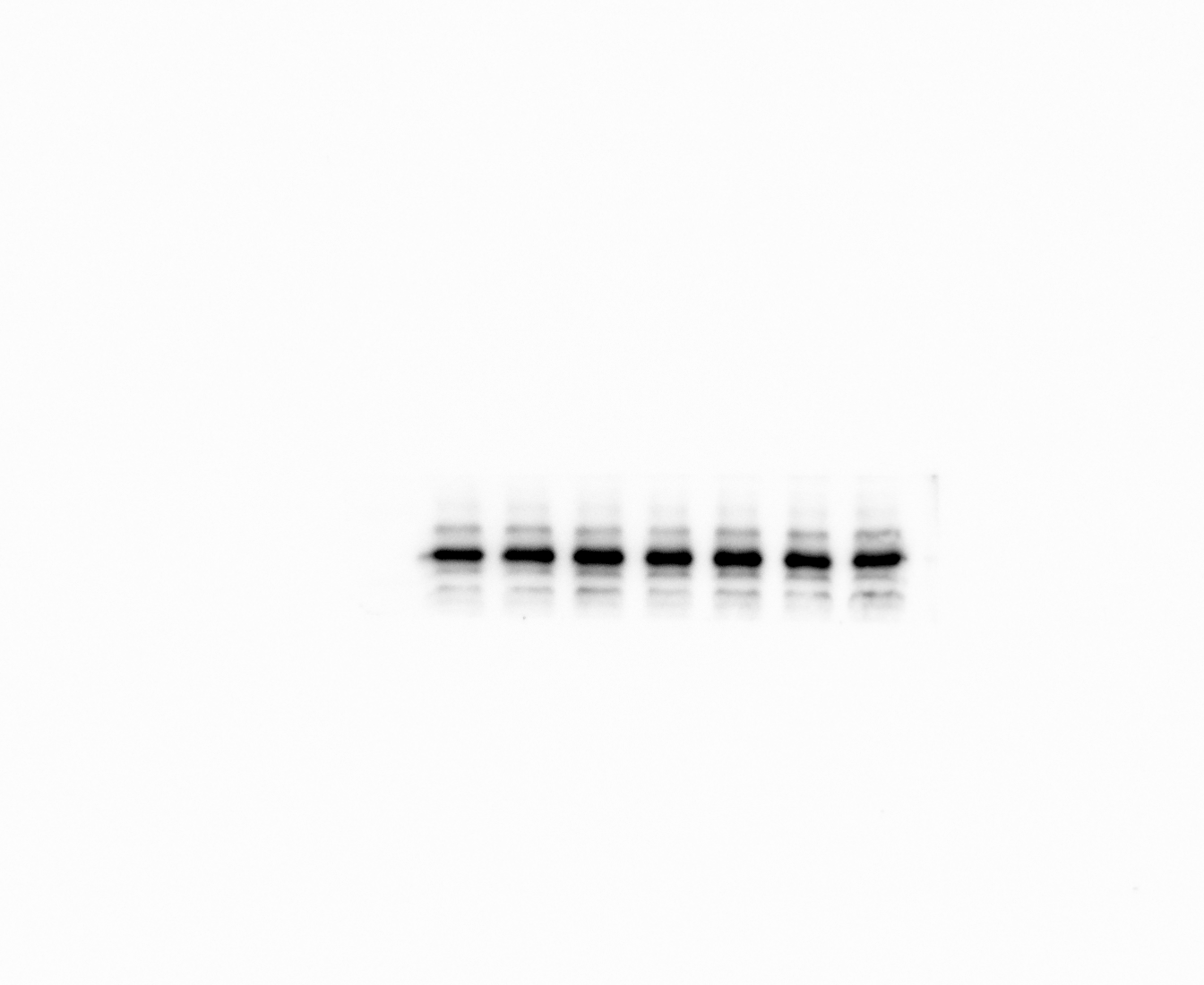

Supplement: Figure 8—source data 1. [file elife-98524-fig8-data1.zip › Fig 8-data1-v1/8G/bottom/ERK1 2.tif]

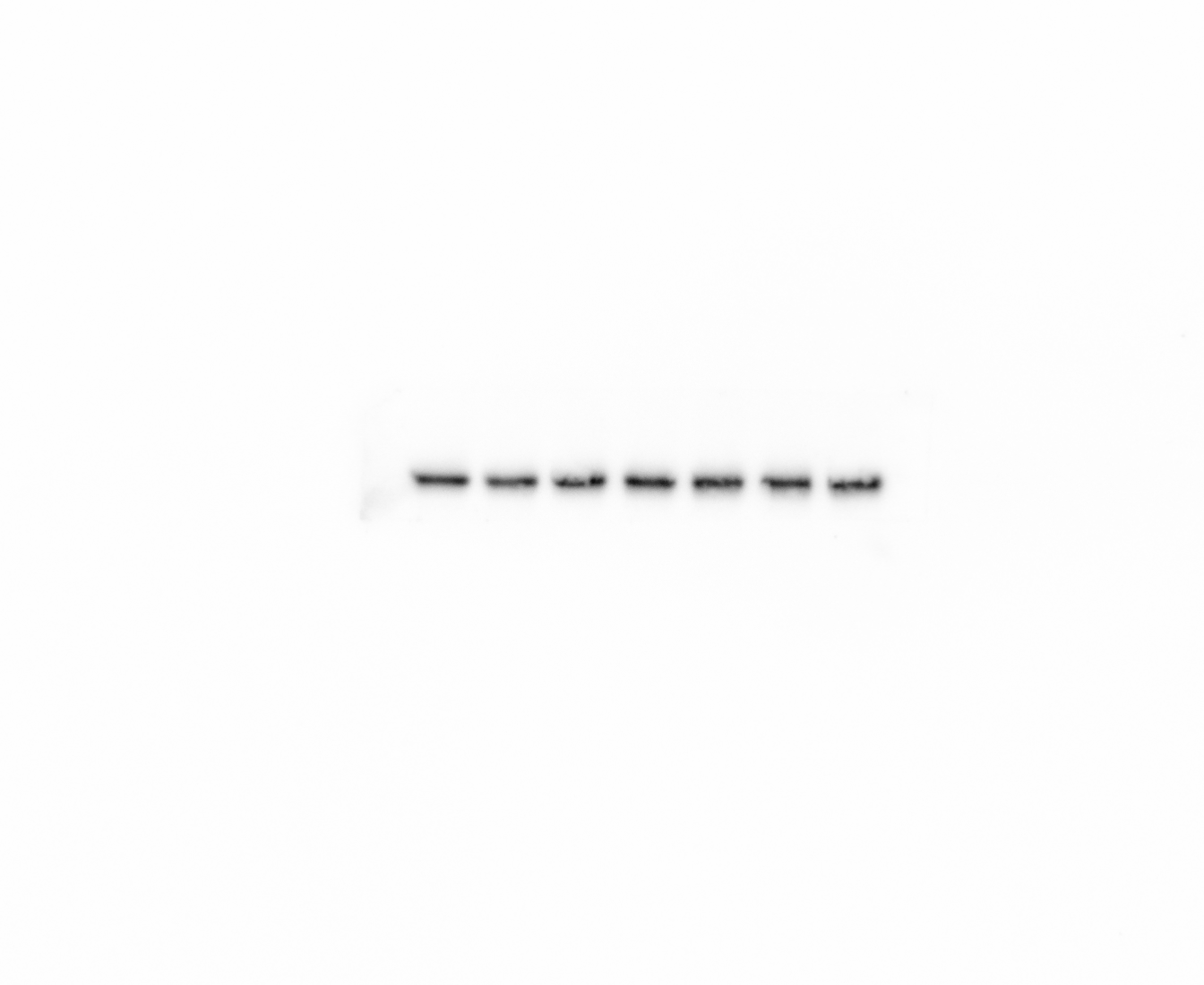

Supplement: Figure 8—source data 1. [file elife-98524-fig8-data1.zip › Fig 8-data1-v1/8G/bottom/Flag.tif]

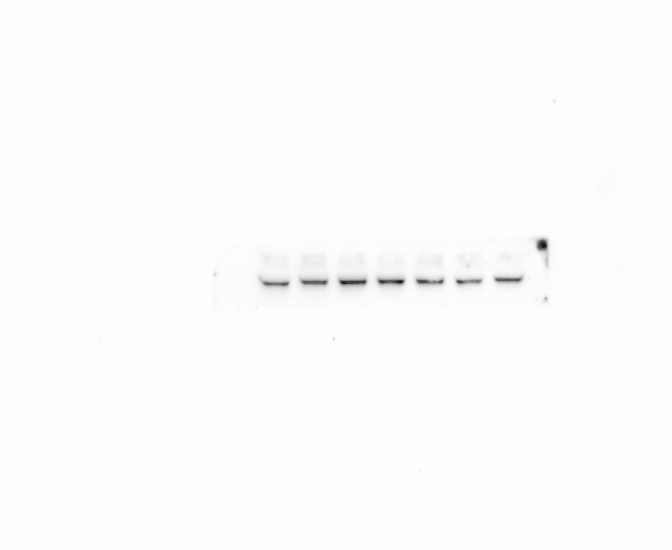

Supplement: Figure 8—source data 1. [file elife-98524-fig8-data1.zip › Fig 8-data1-v1/8G/bottom/Tubulin.tif]

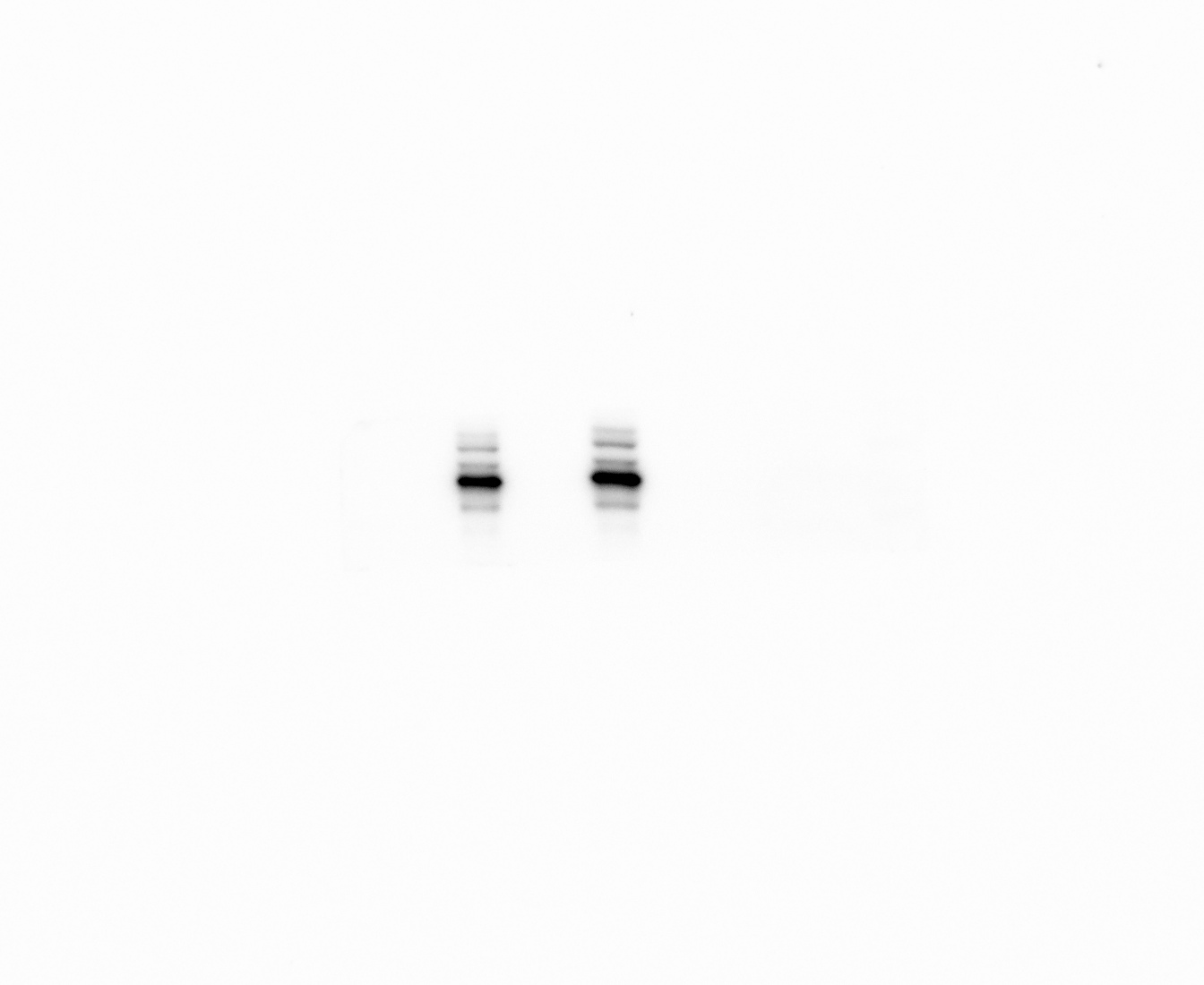

Supplement: Figure 8—source data 1. [file elife-98524-fig8-data1.zip › Fig 8-data1-v1/8G/upper/ERK1 2.tif]

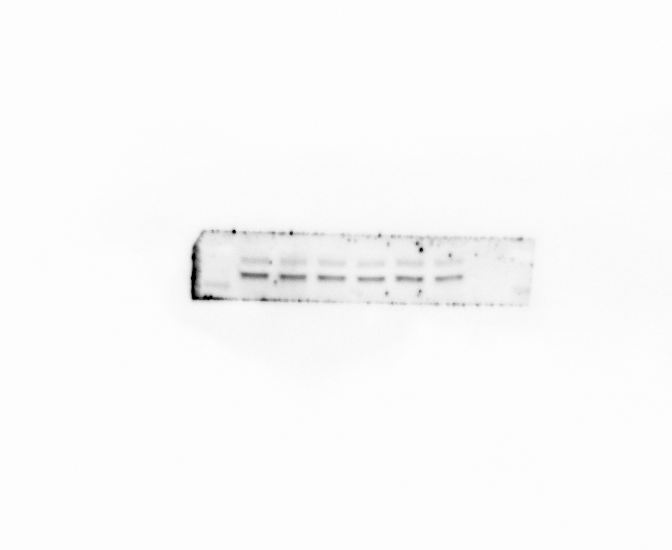

Supplement: Figure 8—source data 1. [file elife-98524-fig8-data1.zip › Fig 8-data1-v1/8G/upper/Flag.tif]

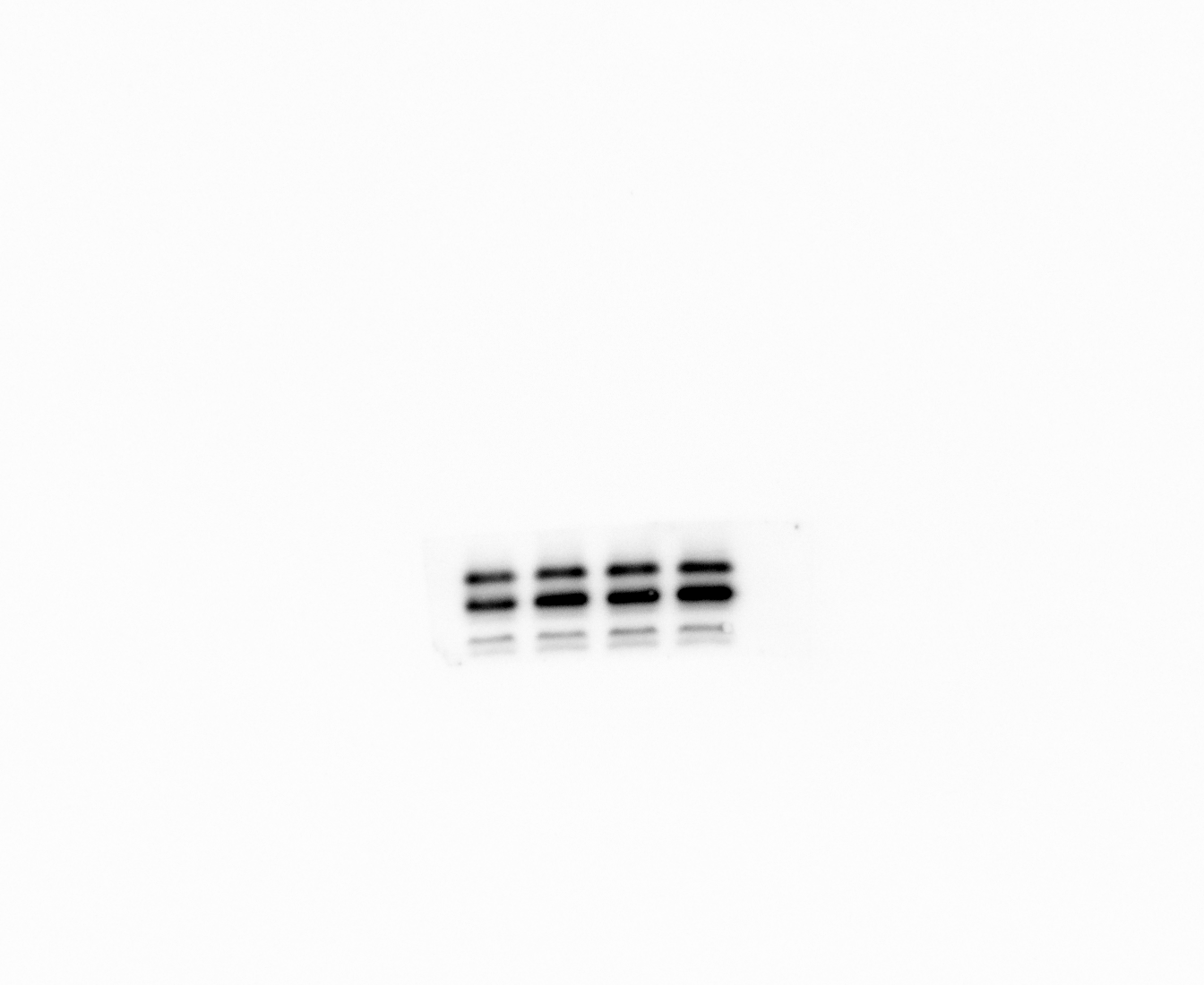

Supplement: Figure 8—source data 1. [file elife-98524-fig8-data1.zip › Fig 8-data1-v1/8H/bottom/ERK1 2.tif]

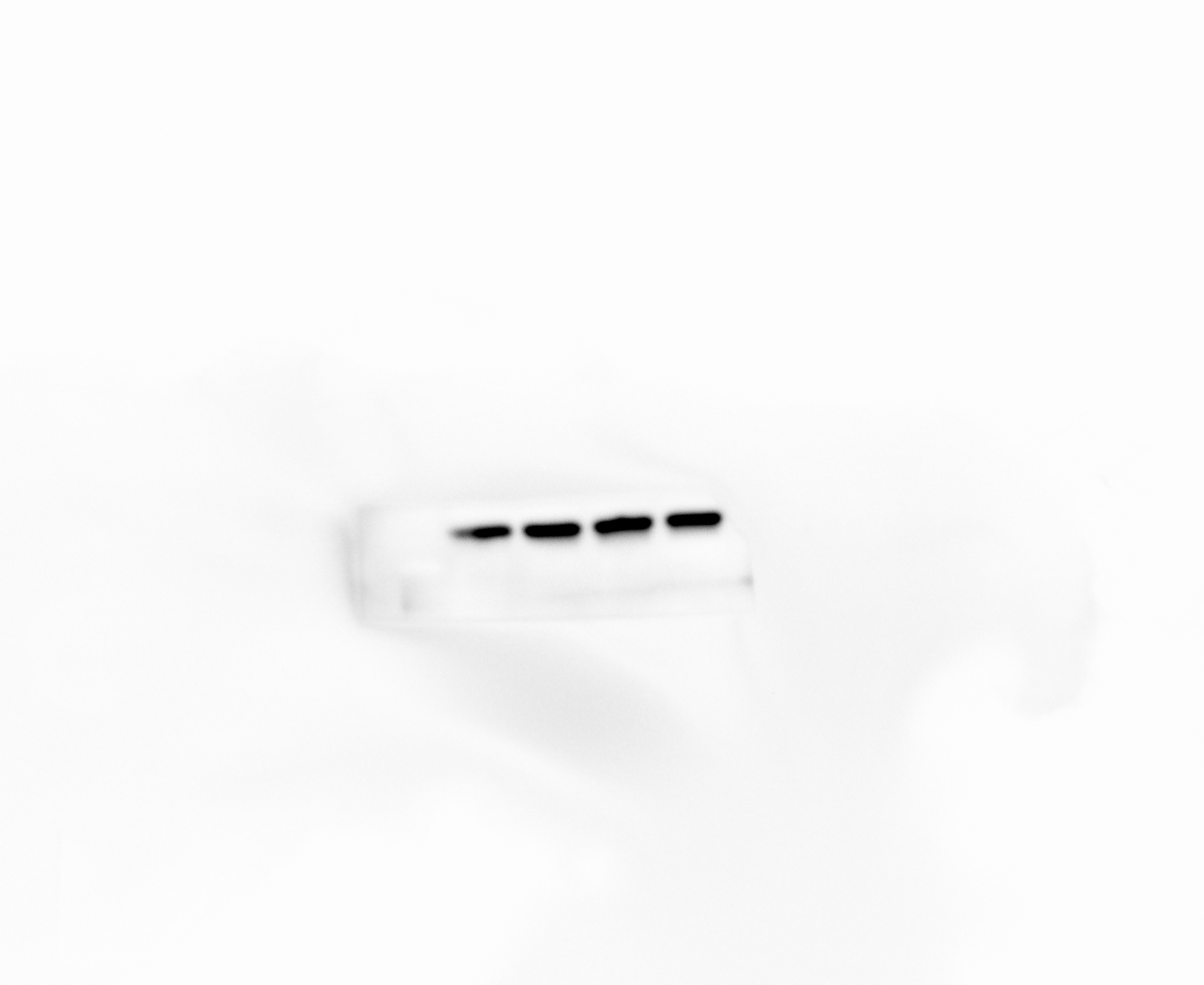

Supplement: Figure 8—source data 1. [file elife-98524-fig8-data1.zip › Fig 8-data1-v1/8H/bottom/Flag.tif]

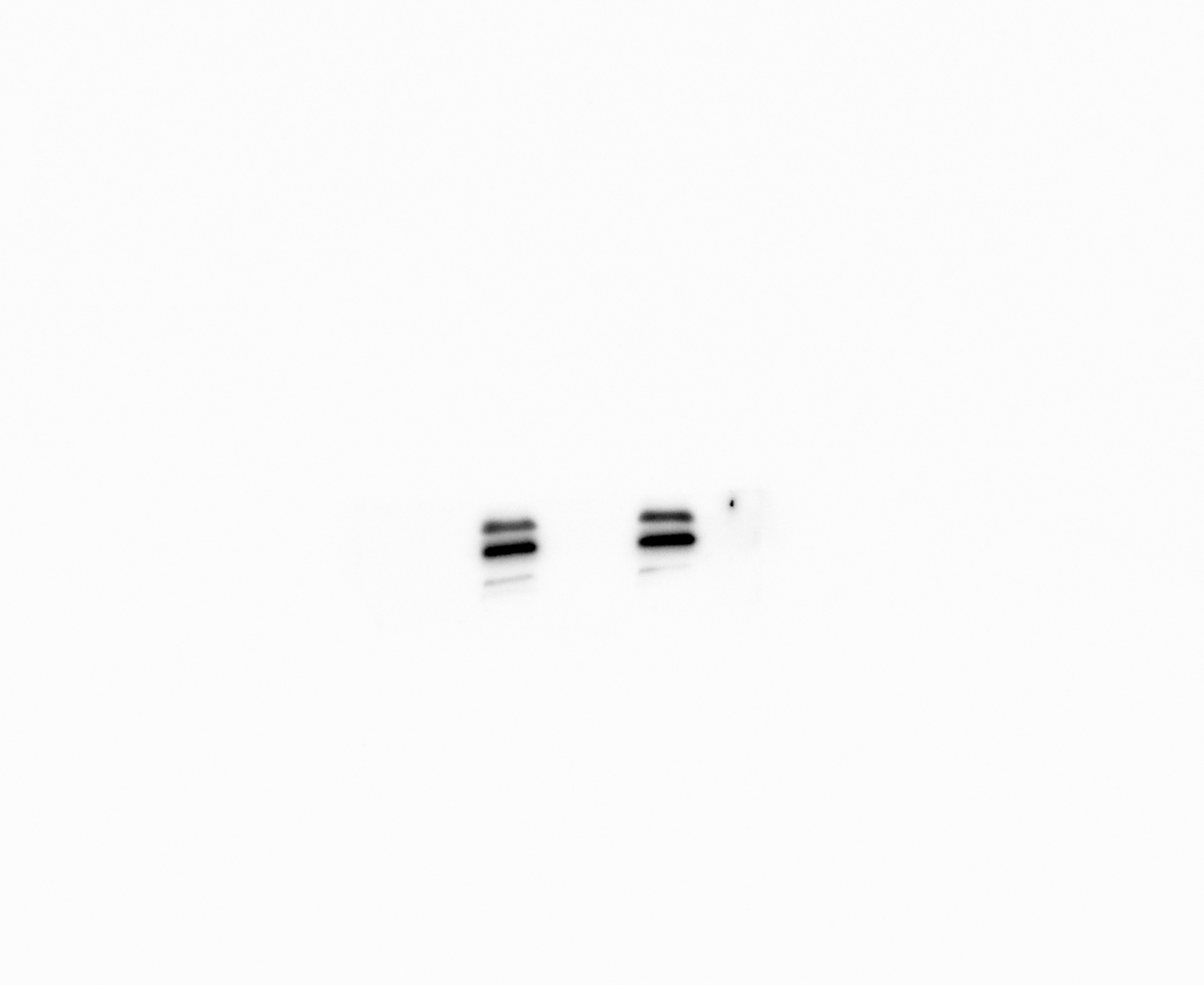

Supplement: Figure 8—source data 1. [file elife-98524-fig8-data1.zip › Fig 8-data1-v1/8H/bottom/pERK1 2.tif]

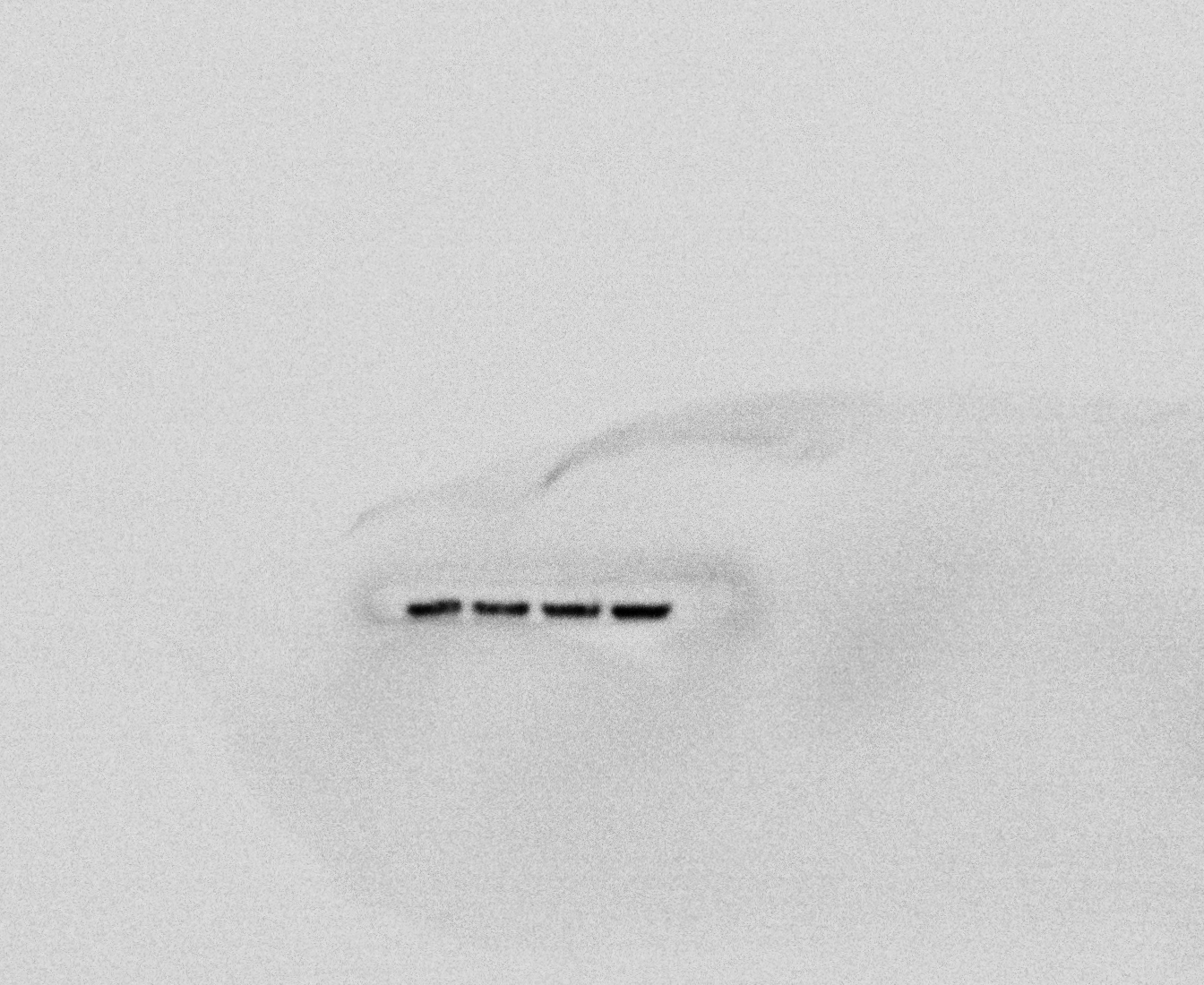

Supplement: Figure 8—source data 1. [file elife-98524-fig8-data1.zip › Fig 8-data1-v1/8H/bottom/Tubulin.tif]

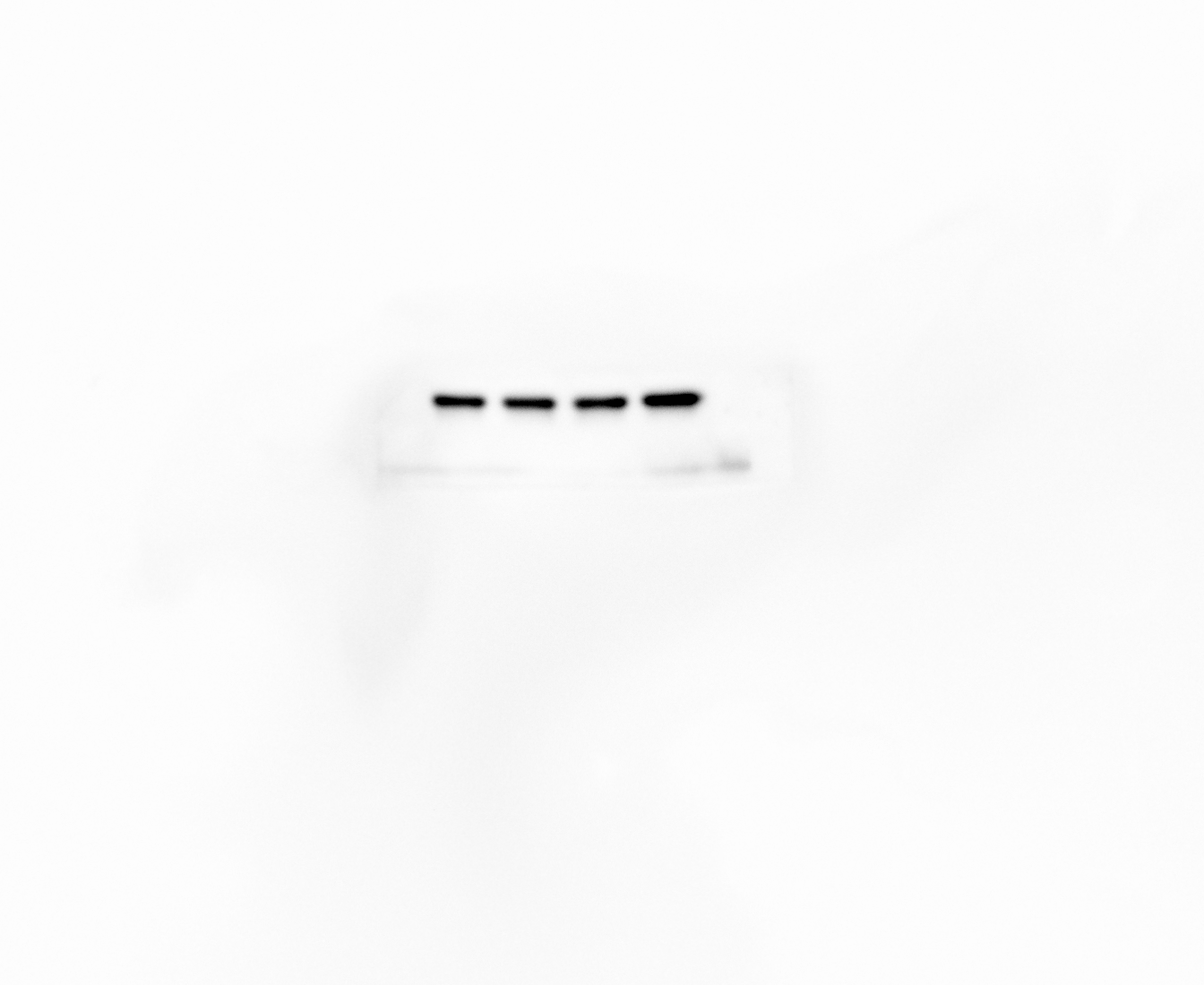

Supplement: Figure 8—source data 1. [file elife-98524-fig8-data1.zip › Fig 8-data1-v1/8H/upper/Flag.tif]

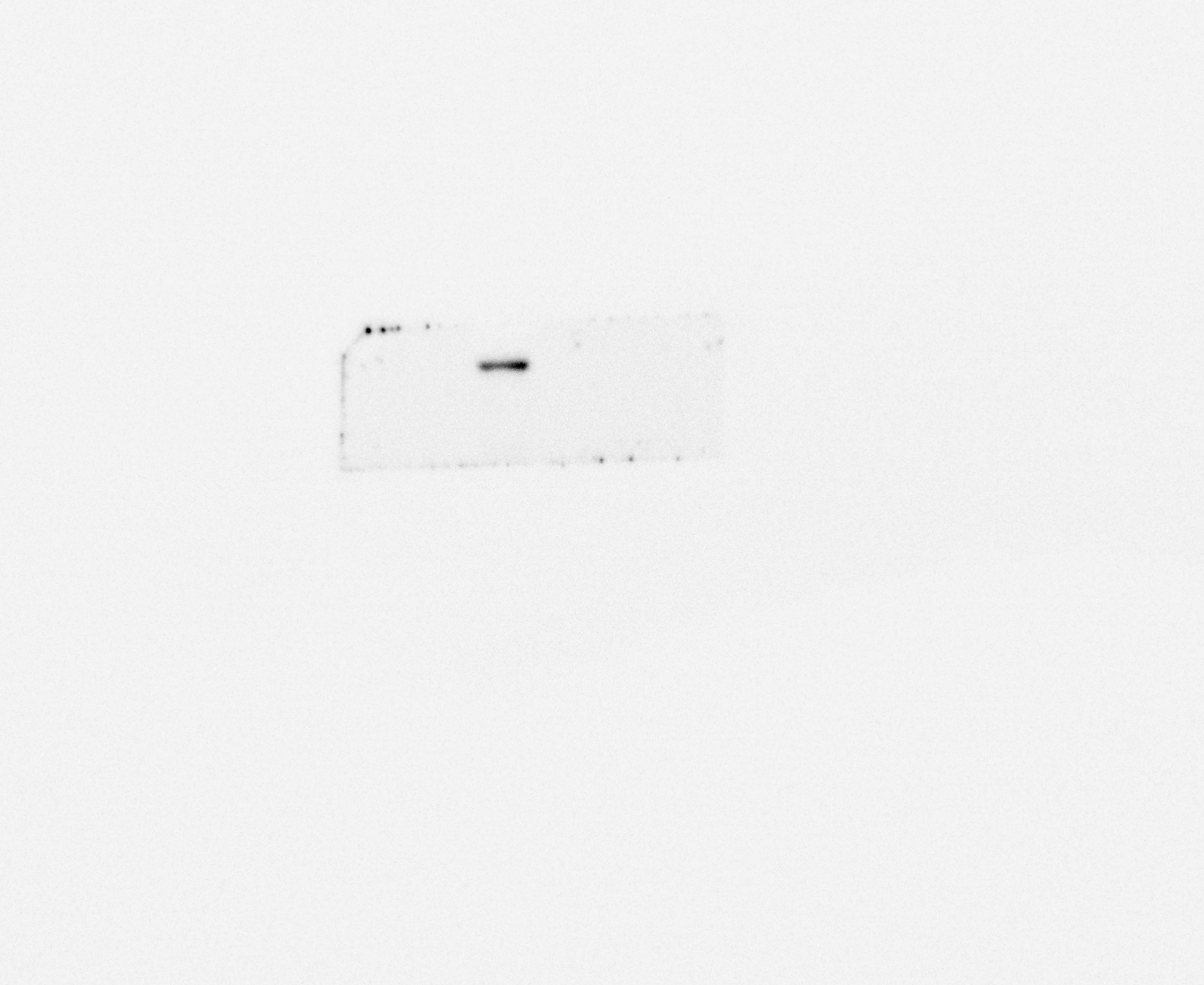

Supplement: Figure 8—source data 1. [file elife-98524-fig8-data1.zip › Fig 8-data1-v1/8H/upper/Phos.tif]

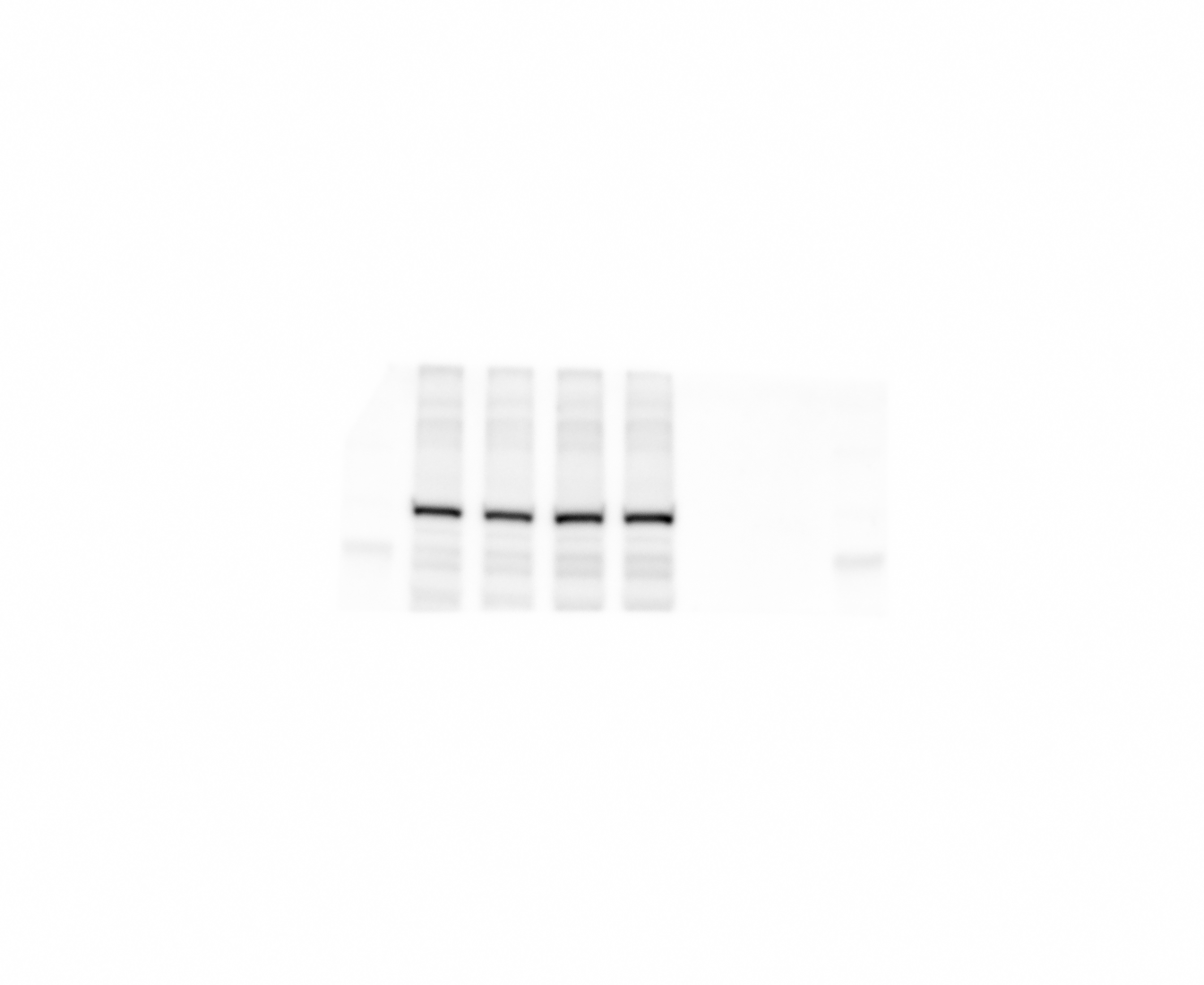

Supplement: Figure 8—source data 1. [file elife-98524-fig8-data1.zip › Fig 8-data1-v1/8I/bottom/Flag.tif]

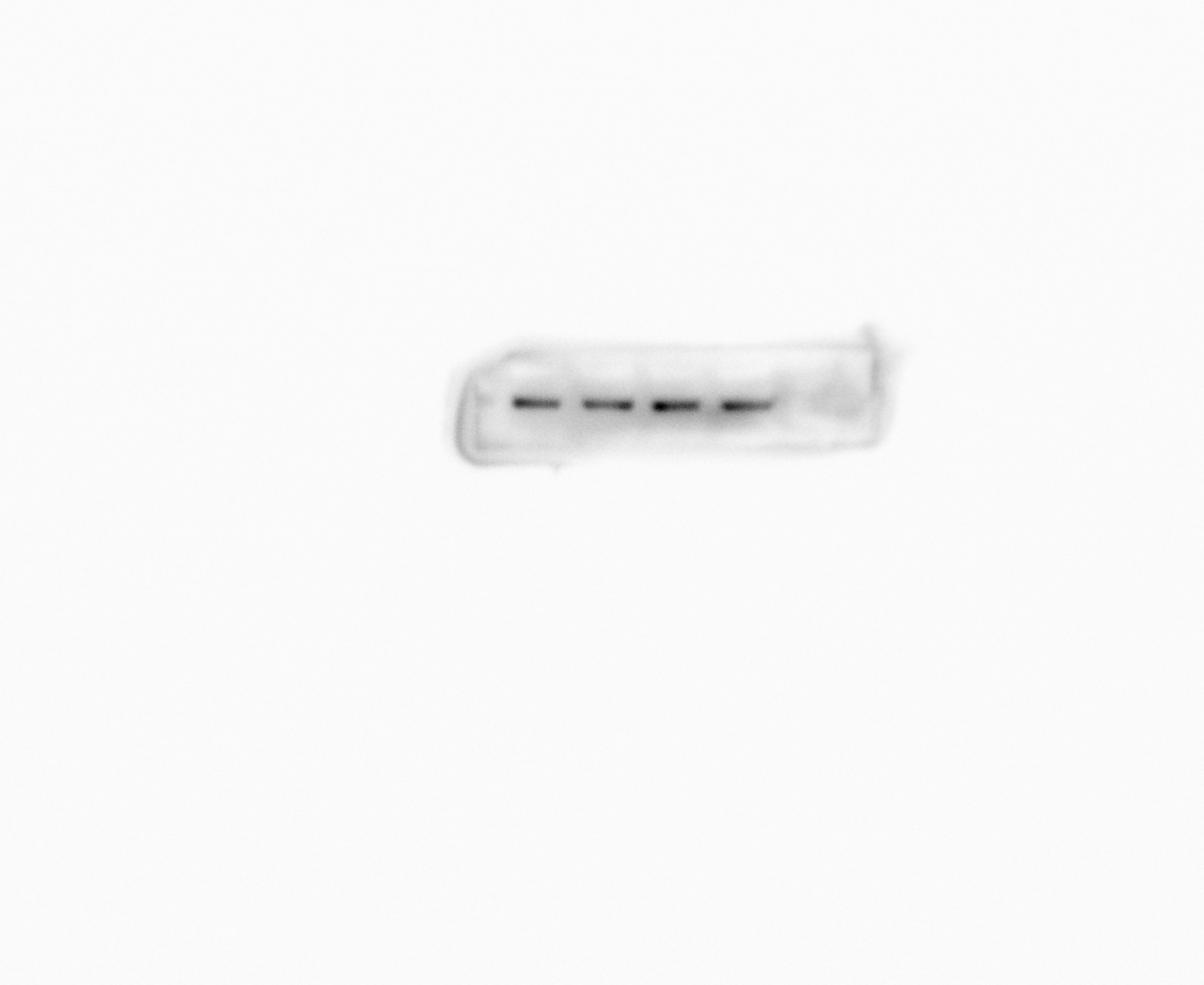

Supplement: Figure 8—source data 1. [file elife-98524-fig8-data1.zip › Fig 8-data1-v1/8I/bottom/Tubulin.tif]

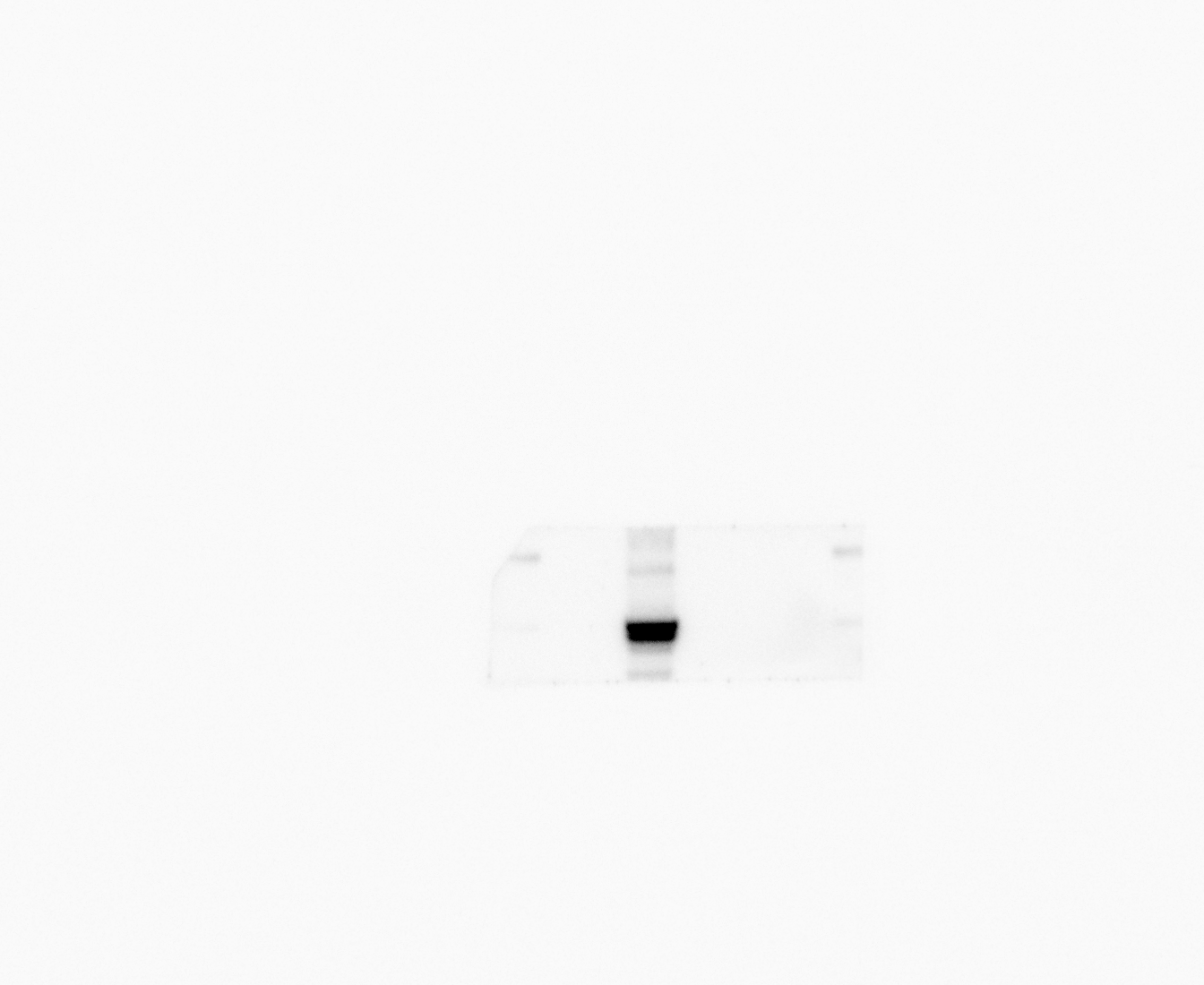

Supplement: Figure 8—source data 1. [file elife-98524-fig8-data1.zip › Fig 8-data1-v1/8I/middle/Flag.tif]

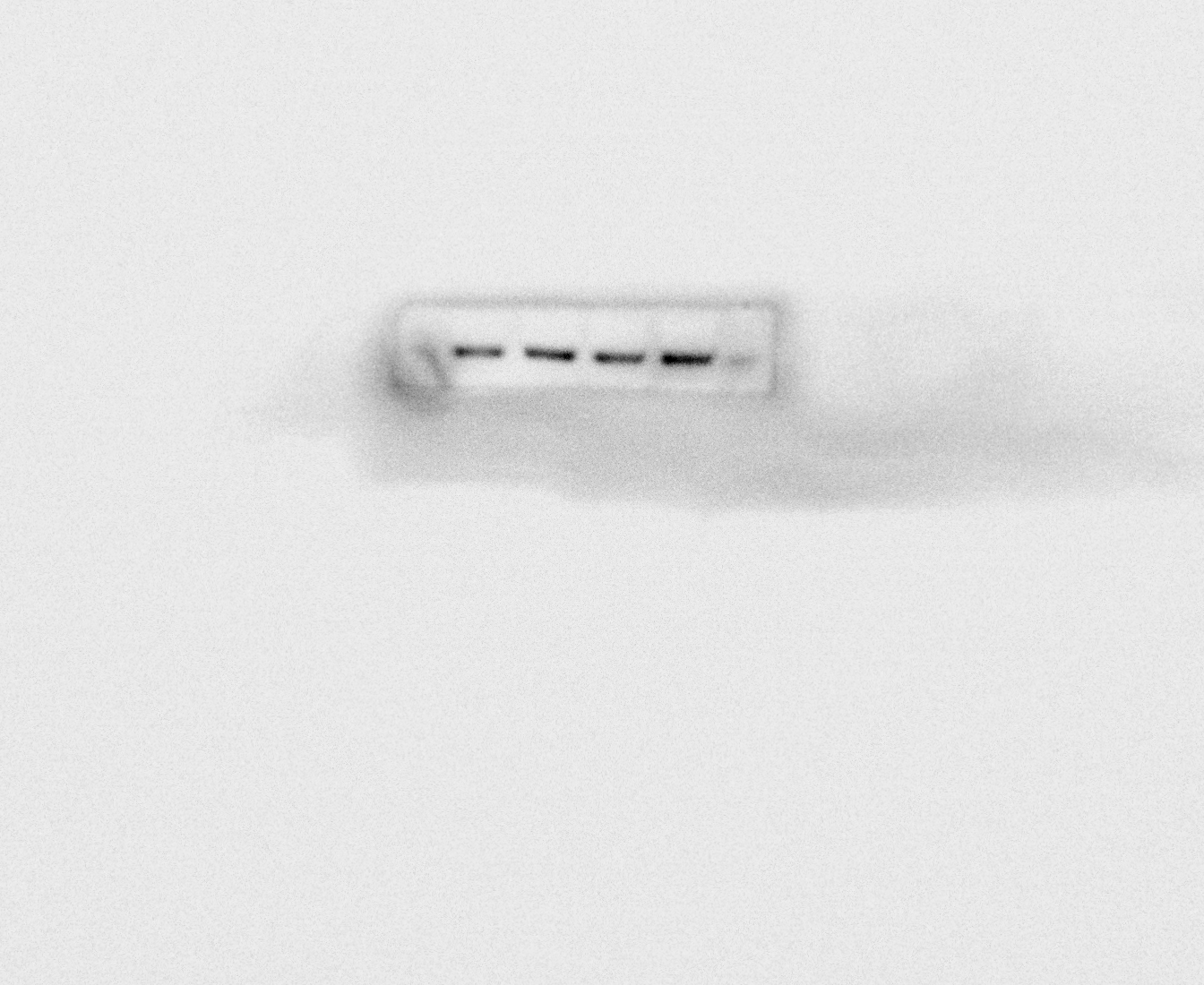

Supplement: Figure 8—source data 1. [file elife-98524-fig8-data1.zip › Fig 8-data1-v1/8I/middle/PCNA.tif]

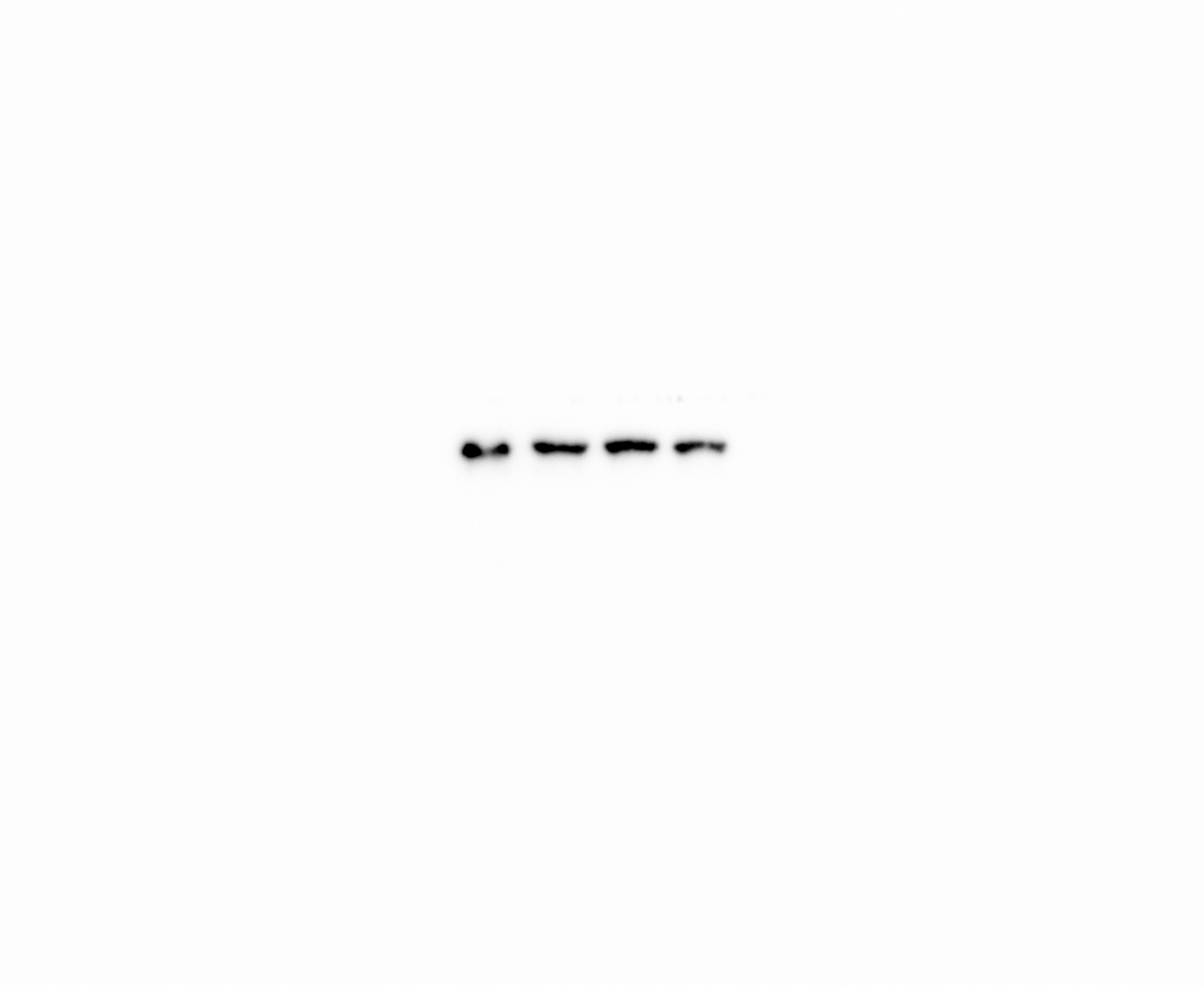

Supplement: Figure 8—source data 1. [file elife-98524-fig8-data1.zip › Fig 8-data1-v1/8I/upper/Flag.tif]

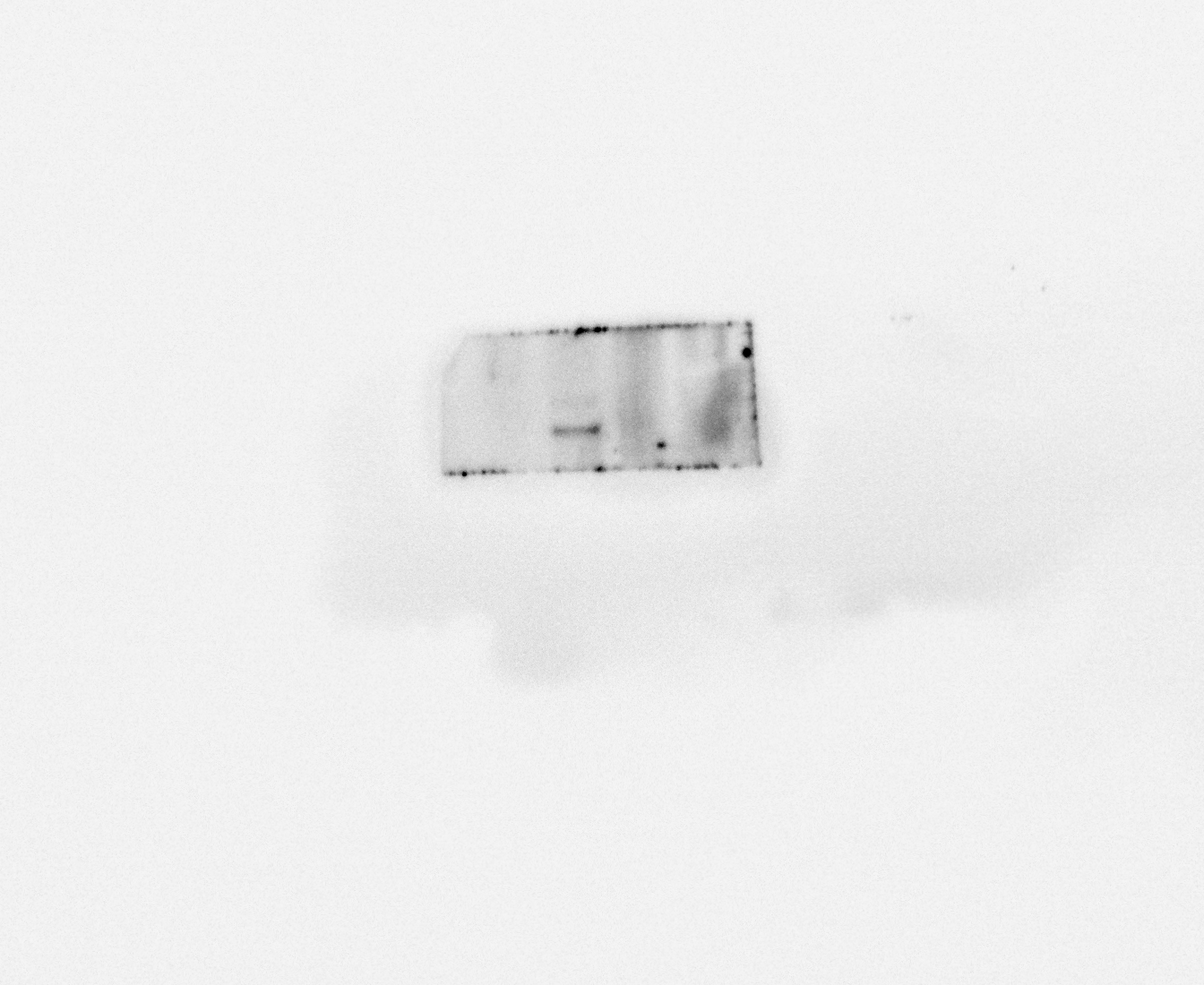

Supplement: Figure 8—source data 1. [file elife-98524-fig8-data1.zip › Fig 8-data1-v1/8I/upper/Phos.tif]

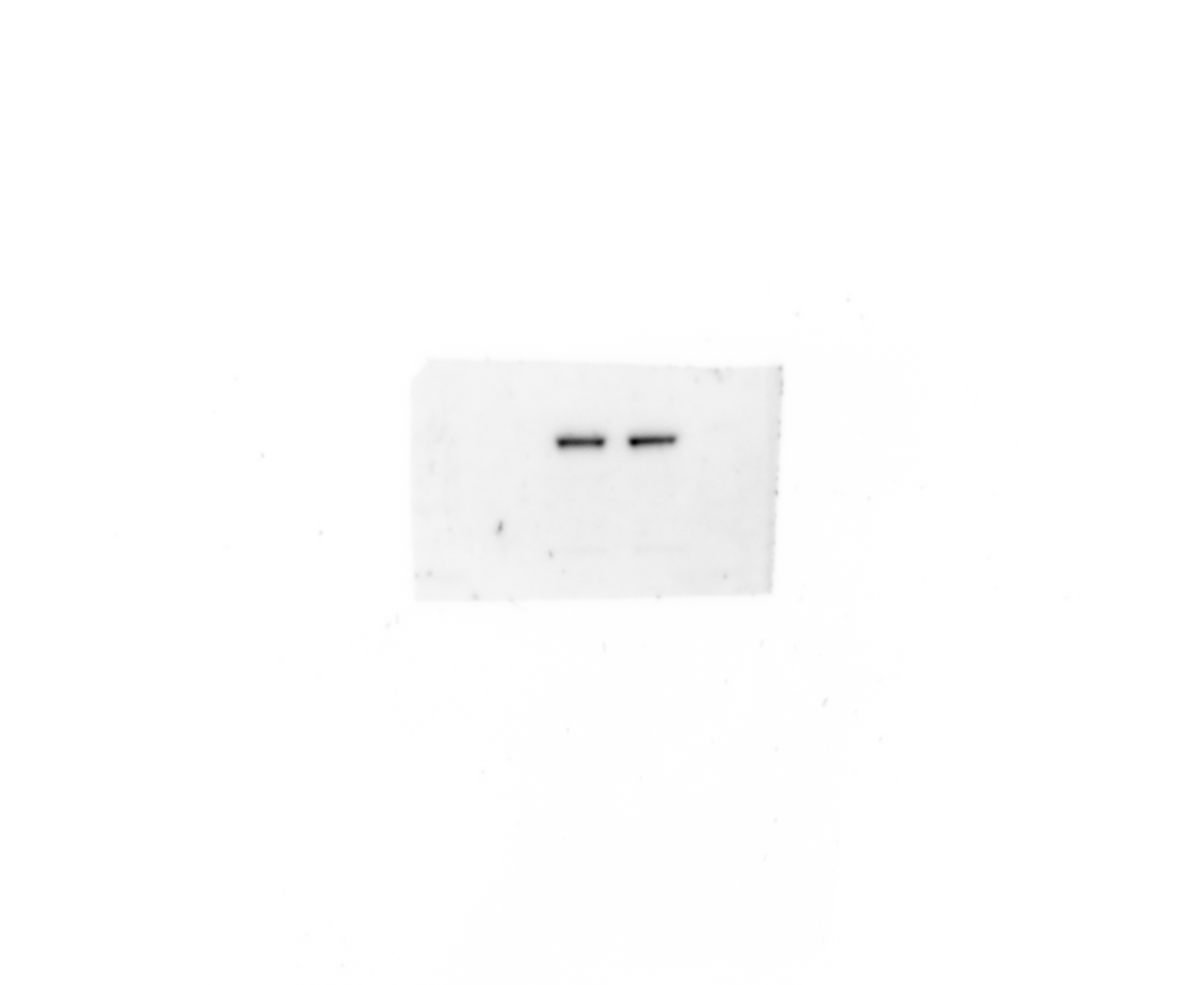

Supplement: Figure 8—source data 1. [file elife-98524-fig8-data1.zip › Fig 8-data1-v1/8J/bottom/Flag.tif]

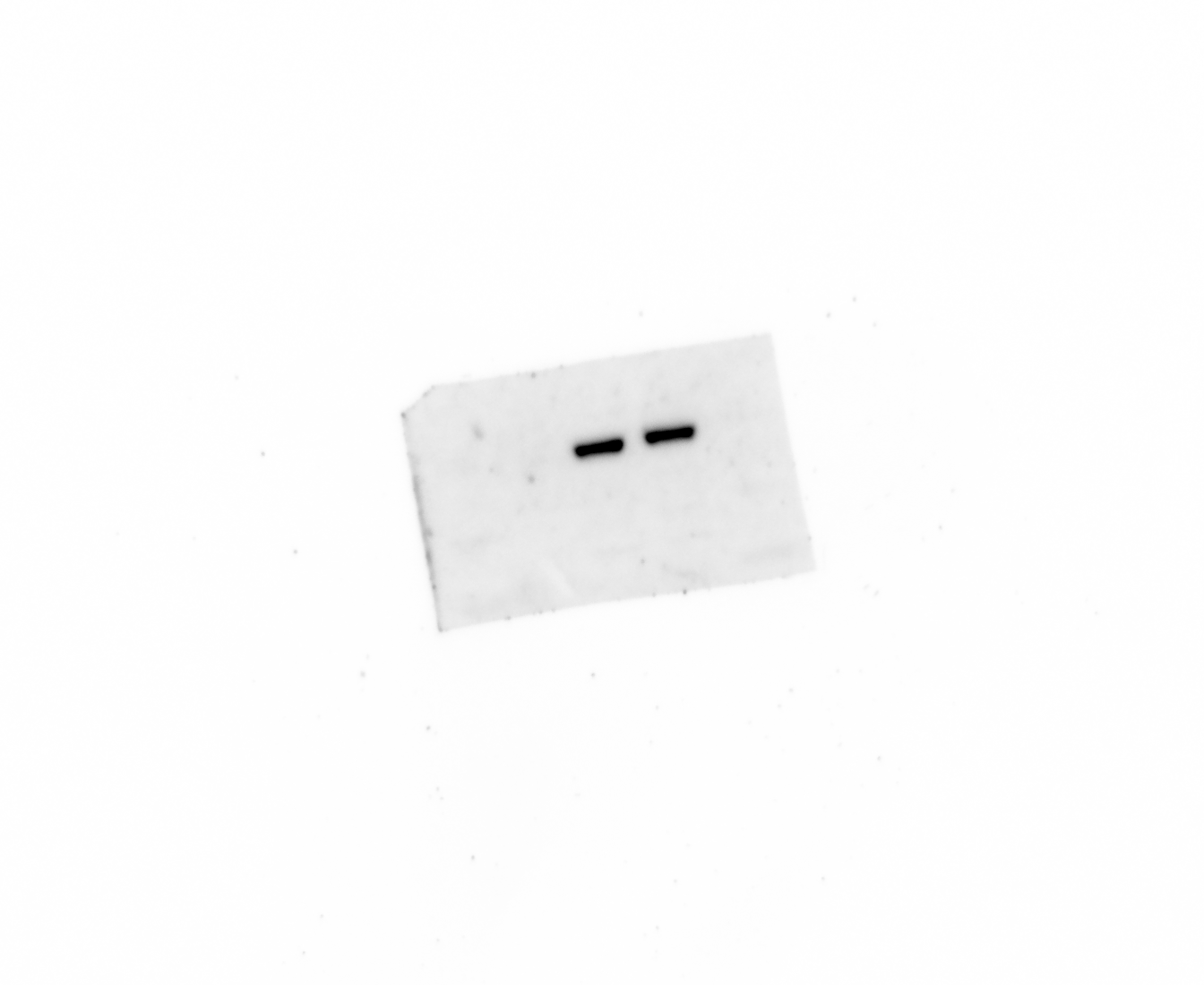

Supplement: Figure 8—source data 1. [file elife-98524-fig8-data1.zip › Fig 8-data1-v1/8J/bottom/HA.tif]

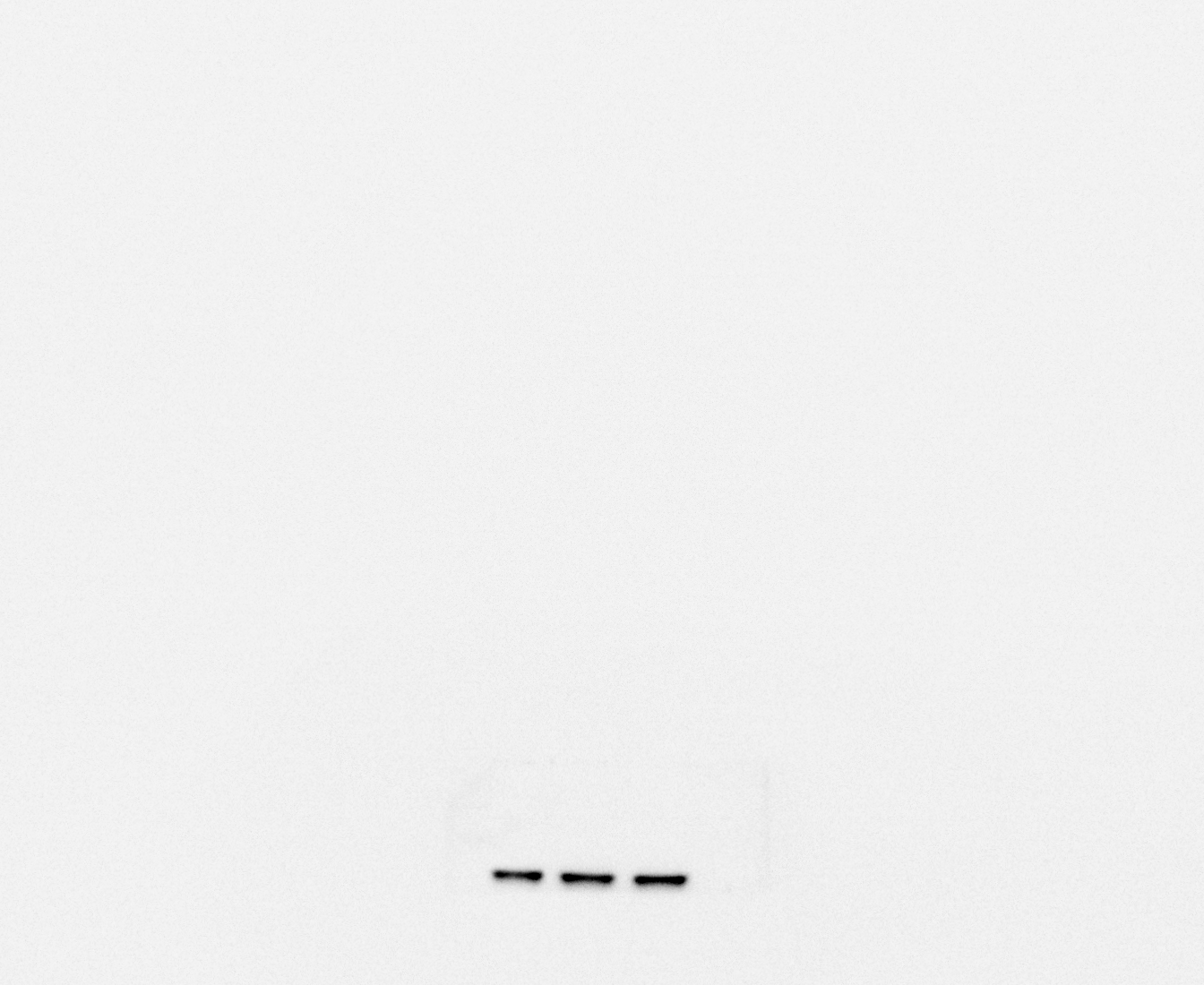

Supplement: Figure 8—source data 1. [file elife-98524-fig8-data1.zip › Fig 8-data1-v1/8J/bottom/Tubulin.tif]

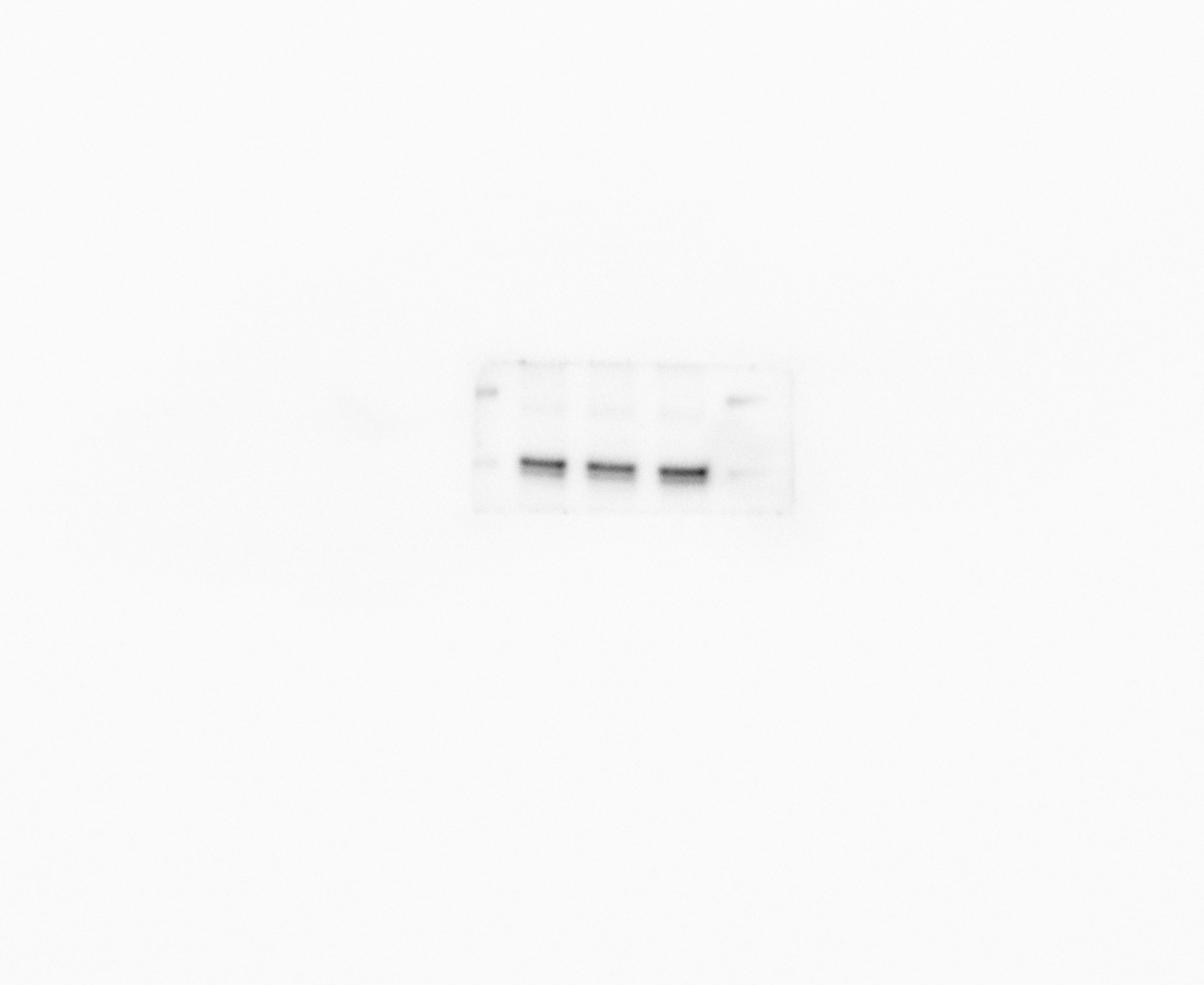

Supplement: Figure 8—source data 1. [file elife-98524-fig8-data1.zip › Fig 8-data1-v1/8J/middle/PCNA.tif]

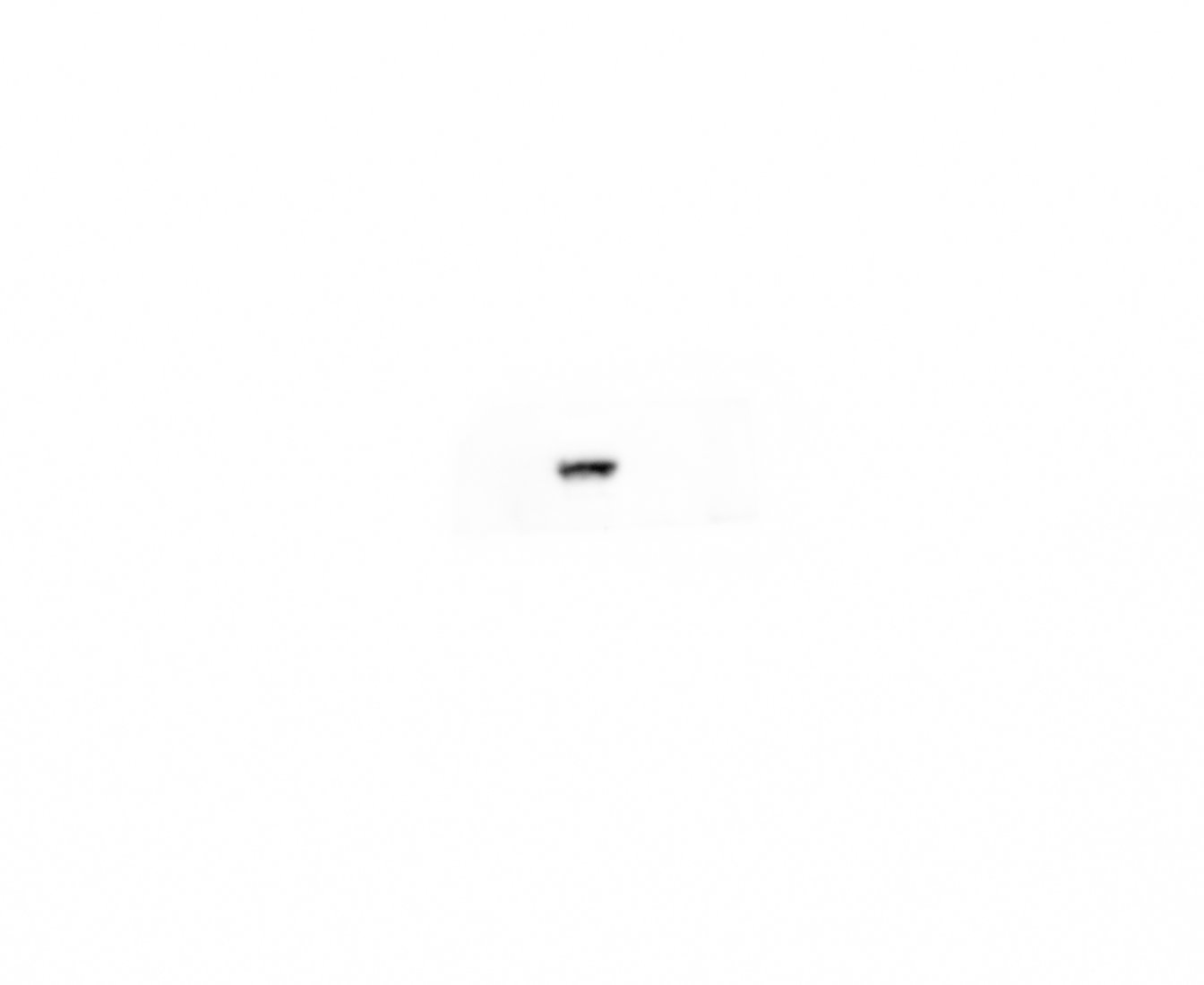

Supplement: Figure 8—source data 1. [file elife-98524-fig8-data1.zip › Fig 8-data1-v1/8J/middle/SIRT4.tif]

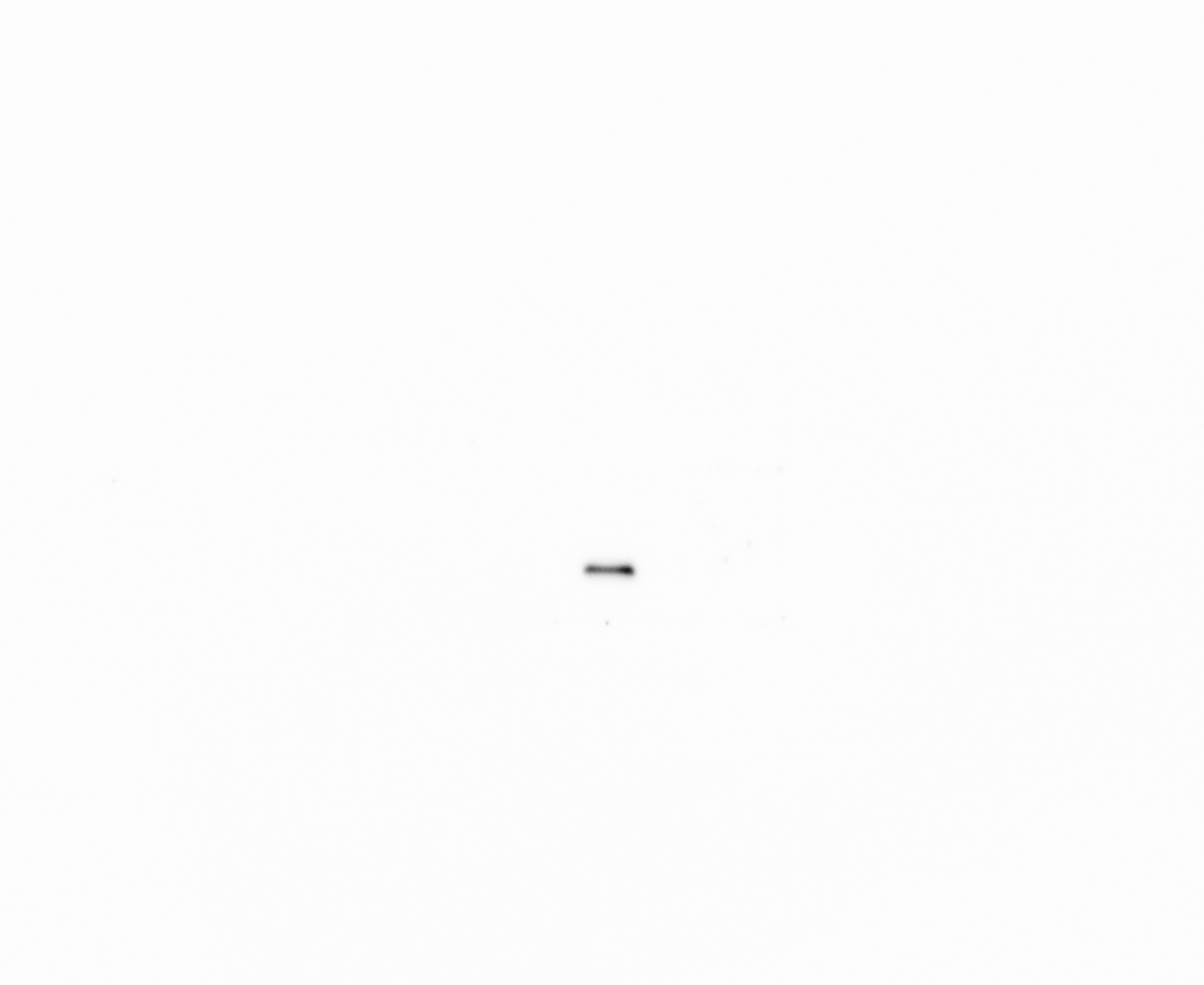

Supplement: Figure 8—source data 1. [file elife-98524-fig8-data1.zip › Fig 8-data1-v1/8J/upper/Phos.tif]

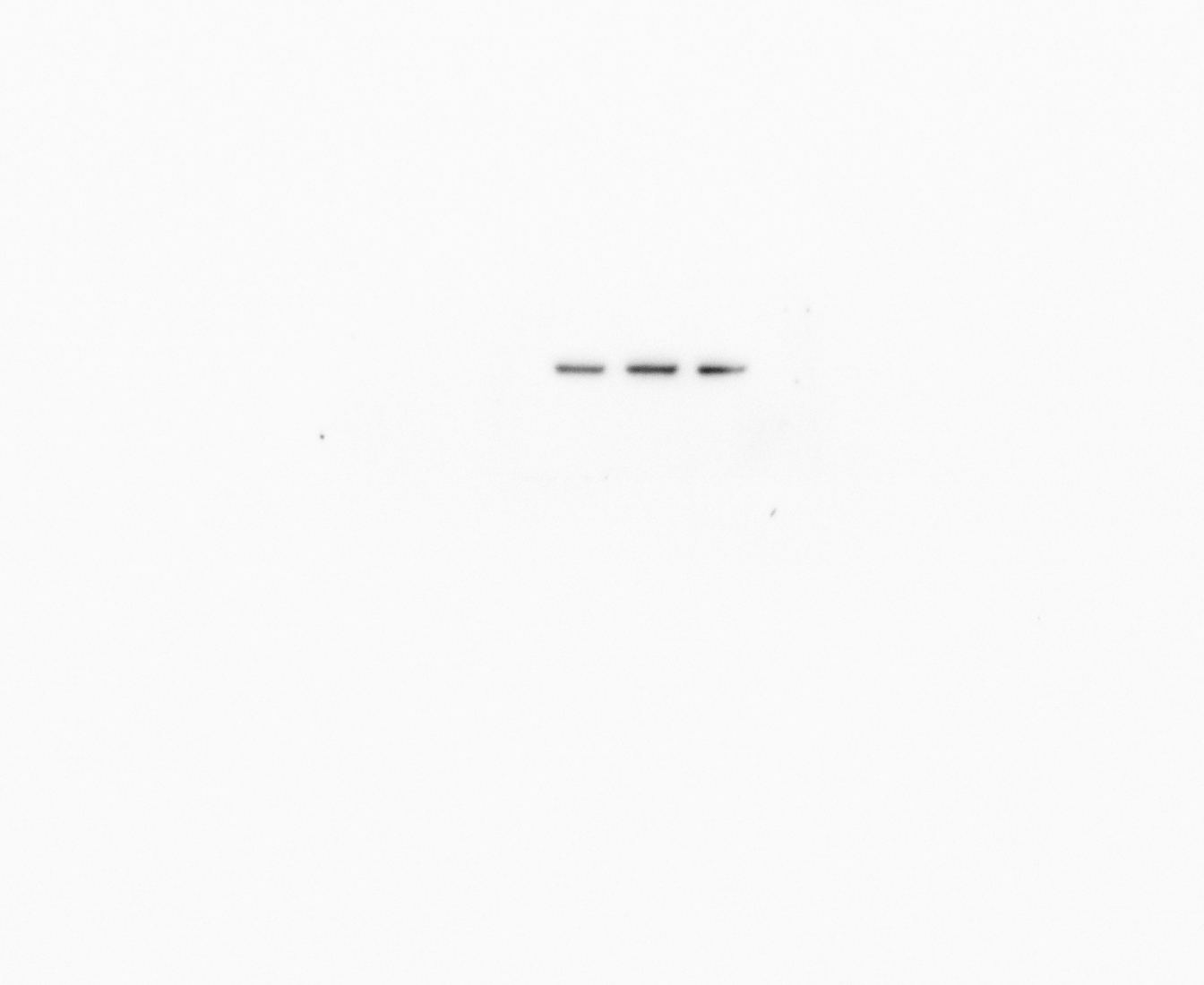

Supplement: Figure 8—source data 1. [file elife-98524-fig8-data1.zip › Fig 8-data1-v1/8J/upper/SIRT4.tif]

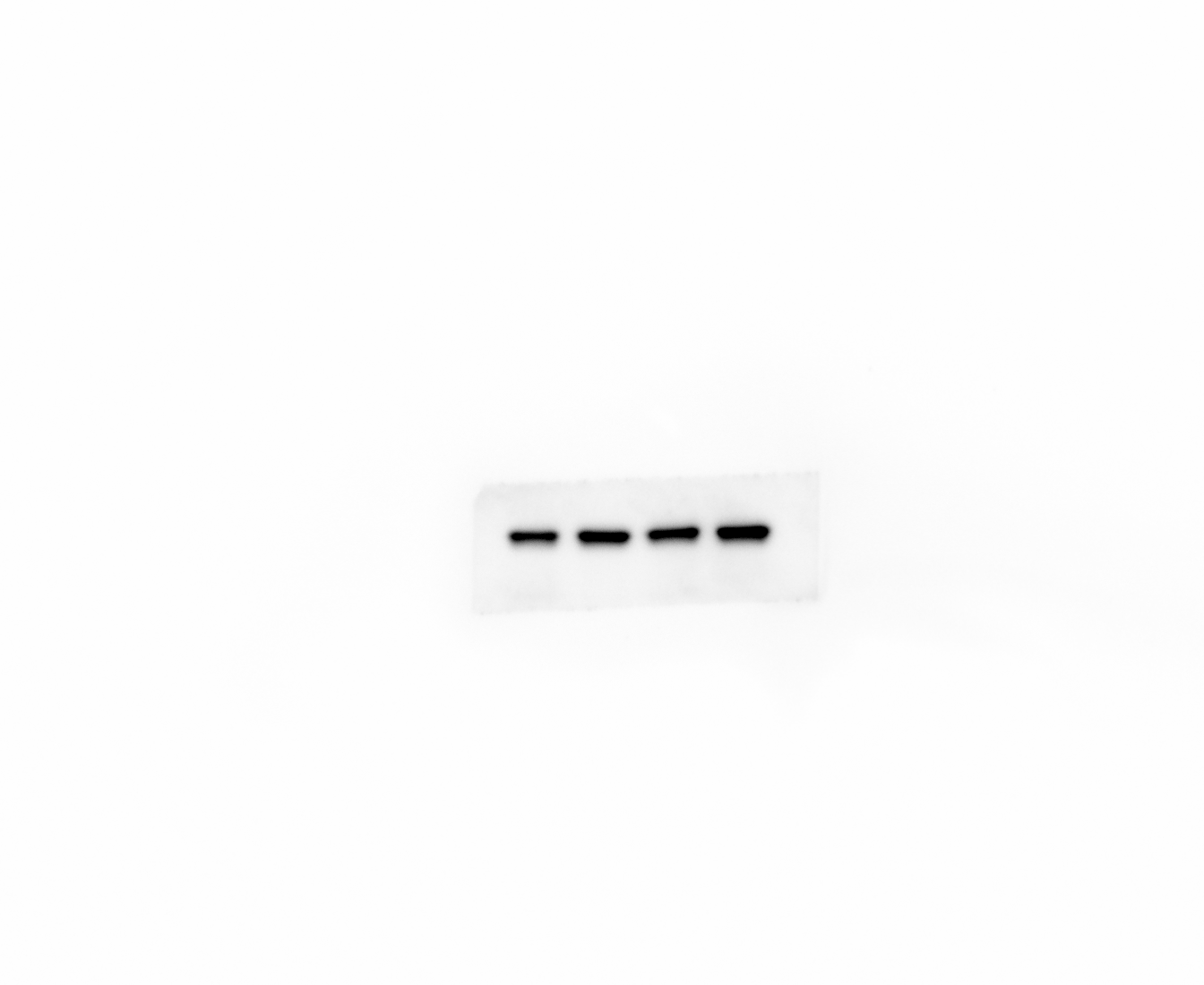

Supplement: Figure 8—source data 1. [file elife-98524-fig8-data1.zip › Fig 8-data1-v1/8K/bottom/Flag.tif]

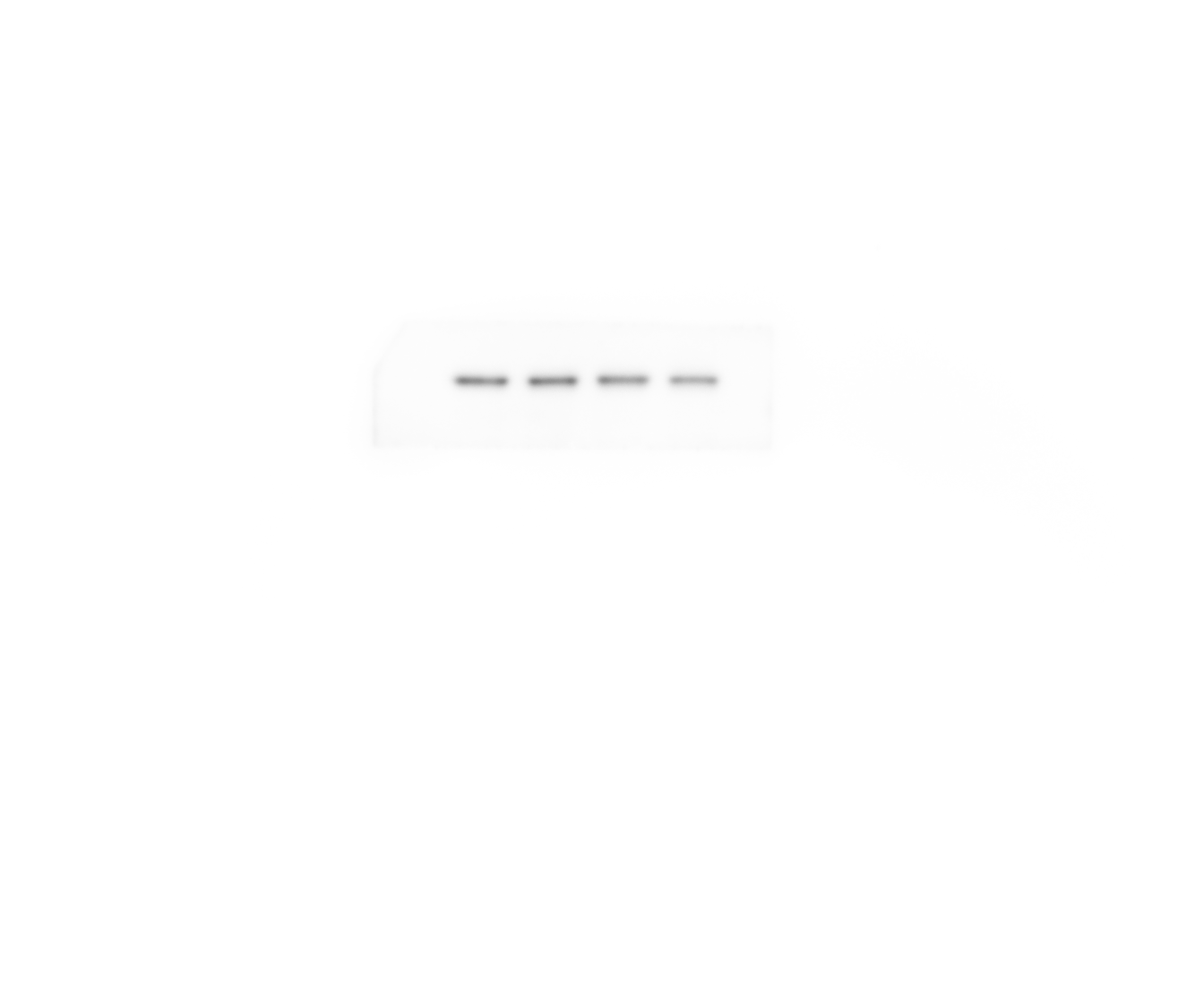

Supplement: Figure 8—source data 1. [file elife-98524-fig8-data1.zip › Fig 8-data1-v1/8K/bottom/Tubulin.tif]

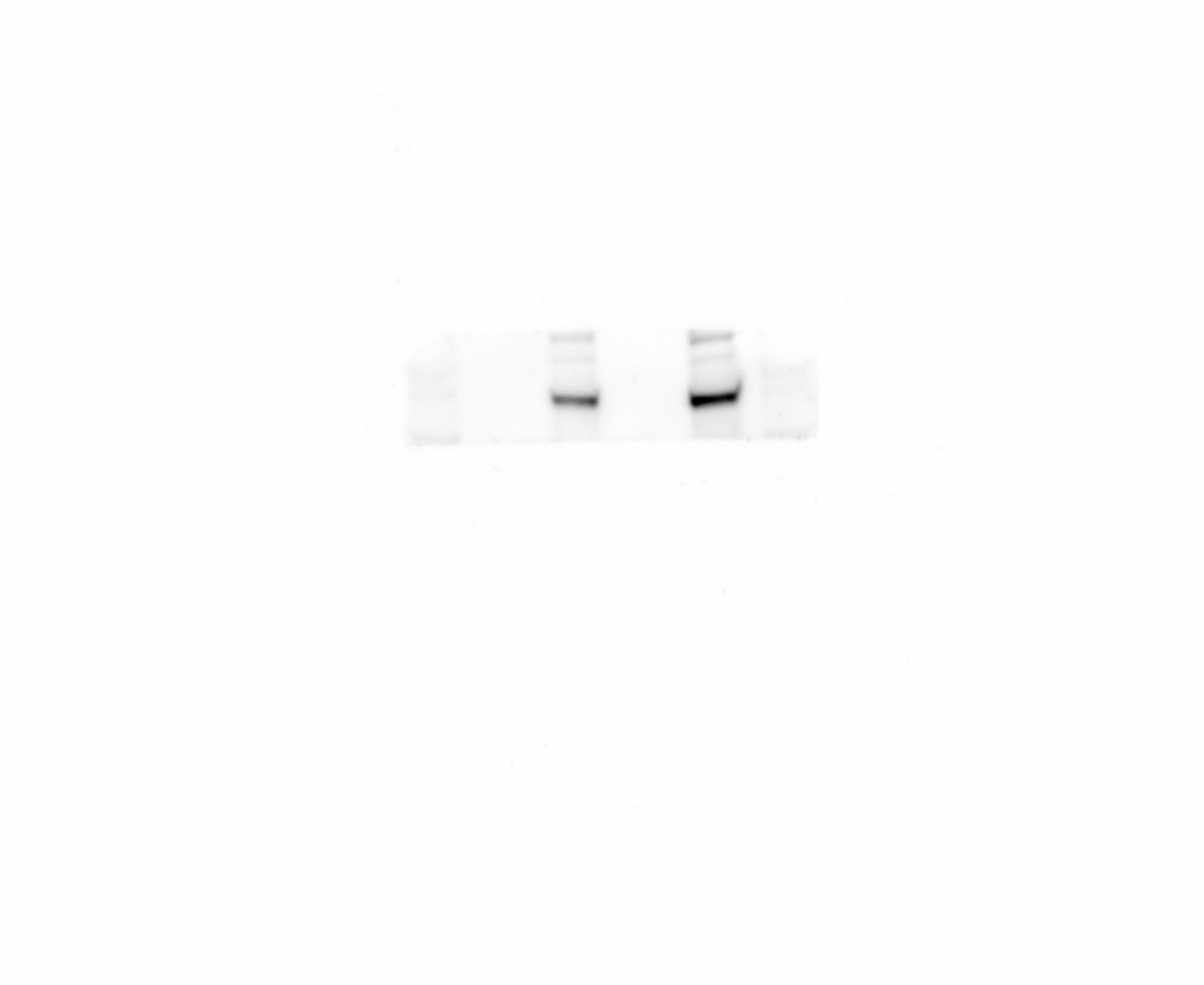

Supplement: Figure 8—source data 1. [file elife-98524-fig8-data1.zip › Fig 8-data1-v1/8K/middle/Flag.tif]

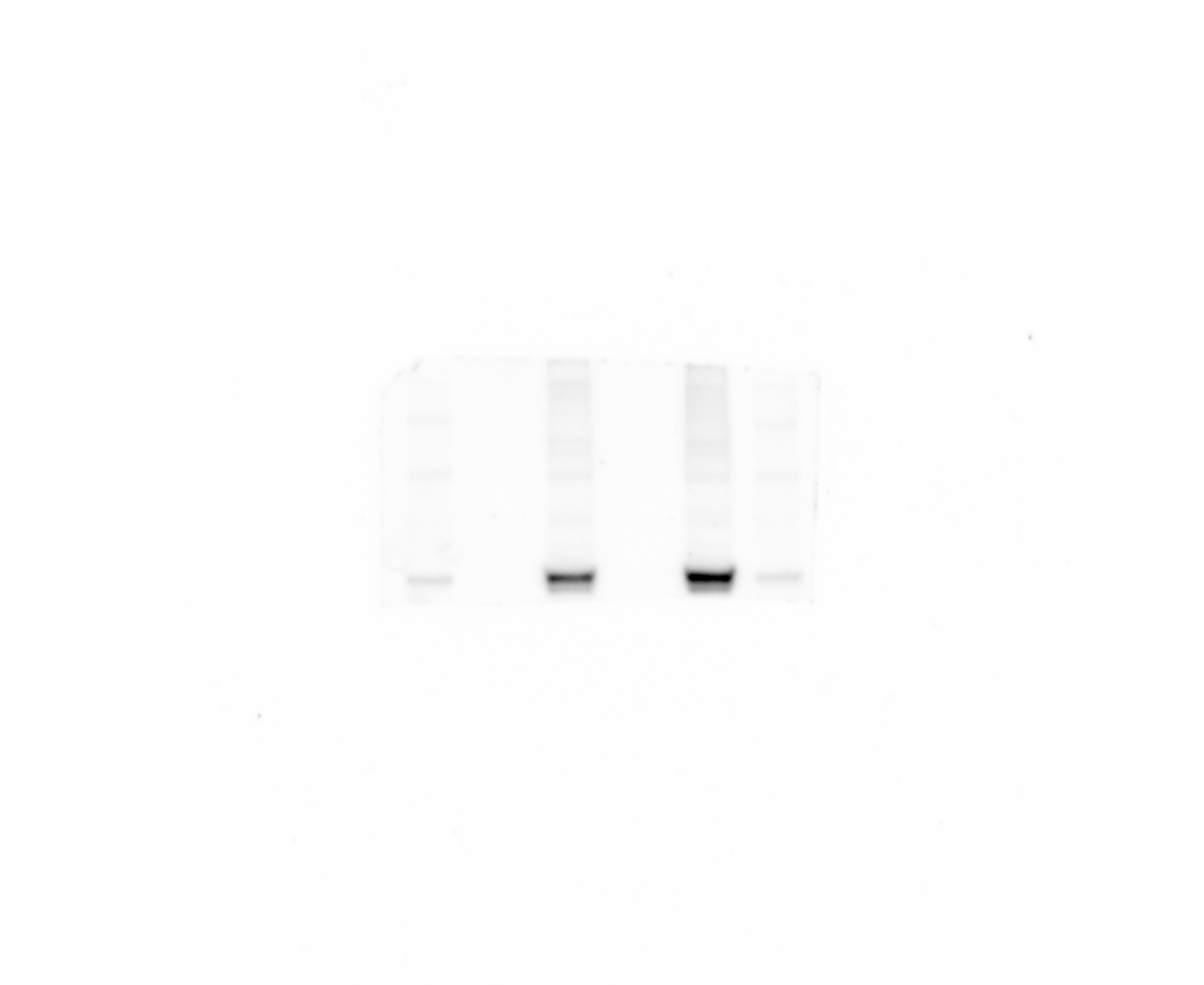

Supplement: Figure 8—source data 1. [file elife-98524-fig8-data1.zip › Fig 8-data1-v1/8K/middle/Phos.tif]

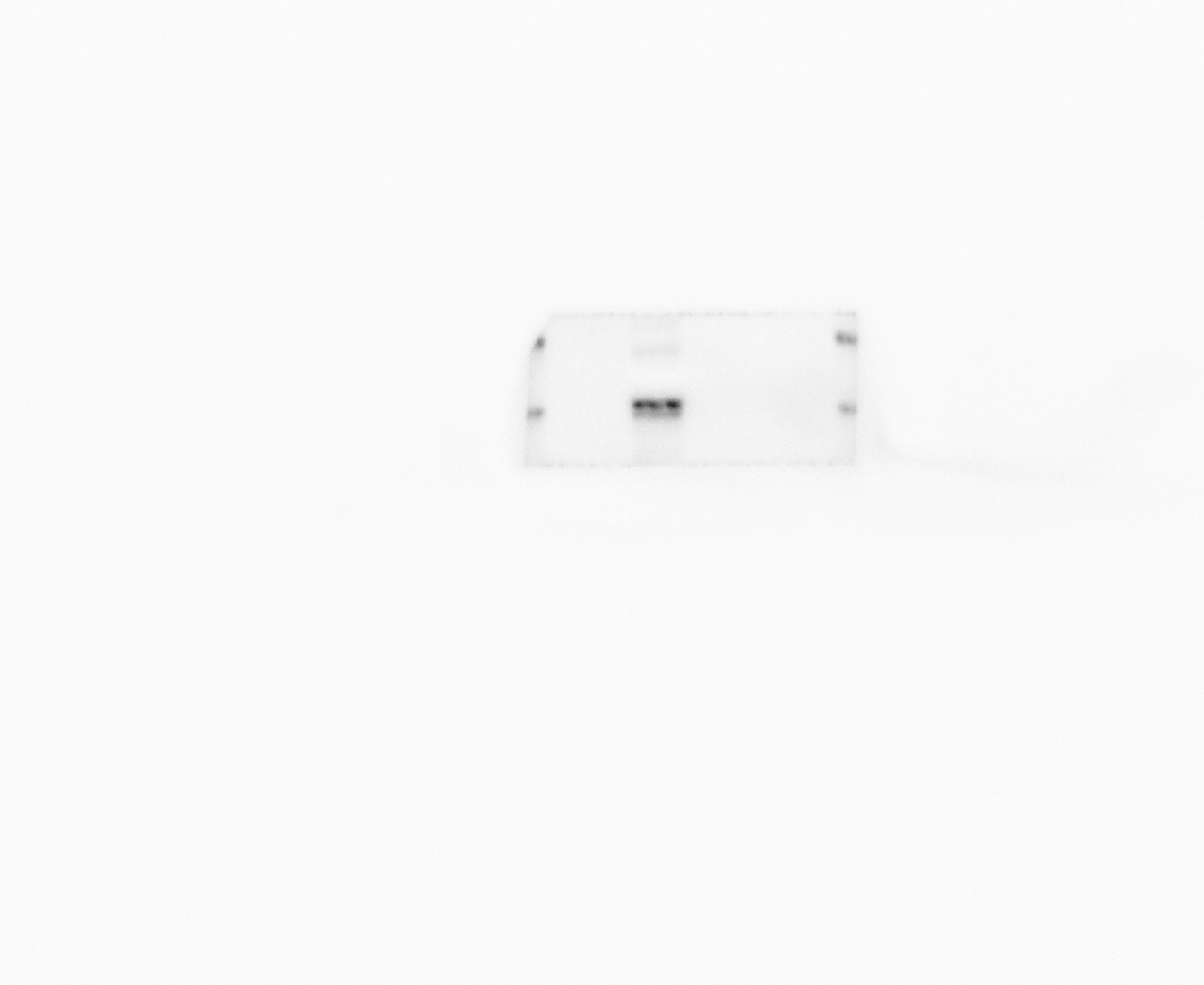

Supplement: Figure 8—source data 1. [file elife-98524-fig8-data1.zip › Fig 8-data1-v1/8K/upper/Flag.tif]

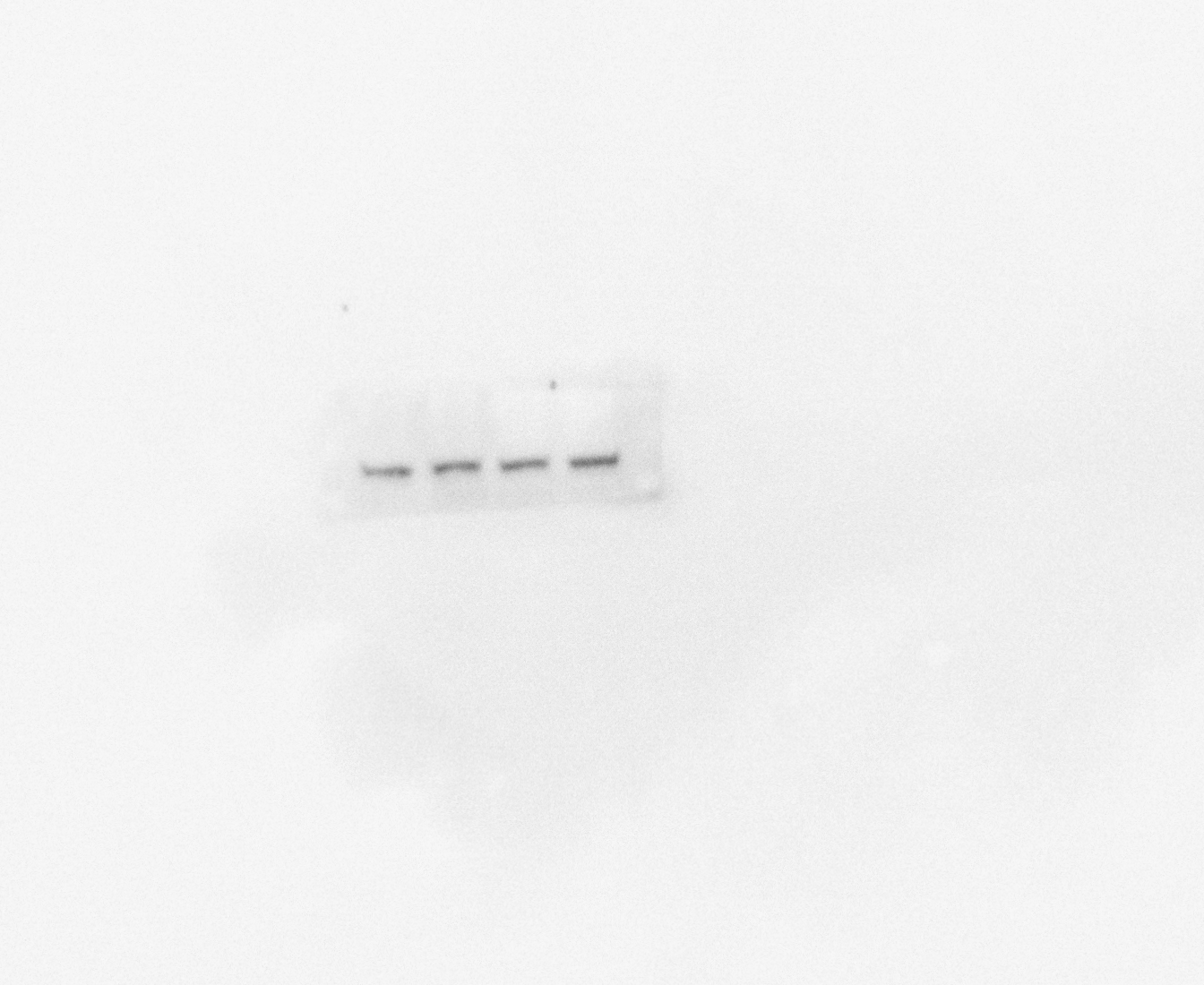

Supplement: Figure 8—source data 1. [file elife-98524-fig8-data1.zip › Fig 8-data1-v1/8K/upper/PCNA.tif]

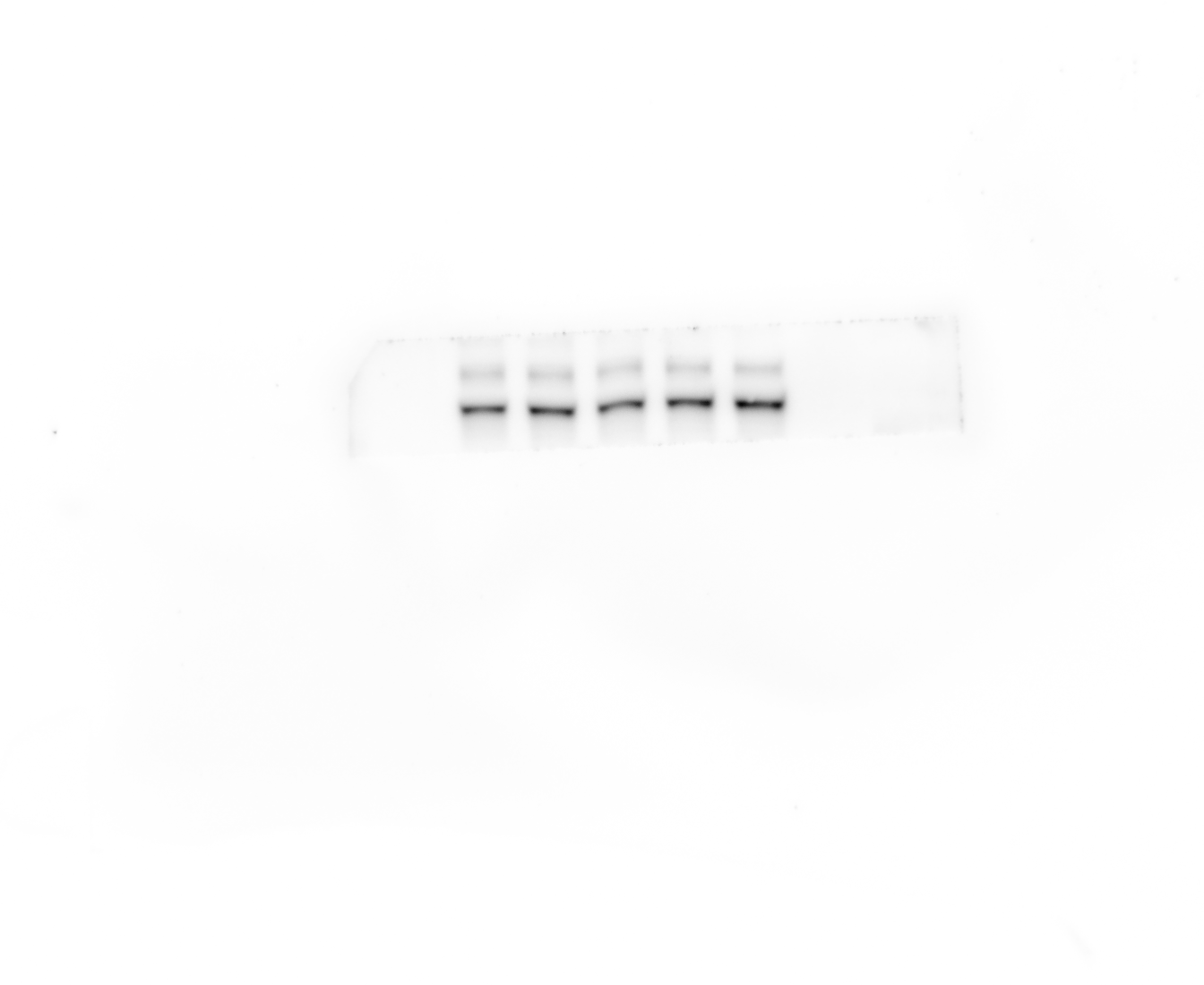

Supplement: Figure 8—source data 1. [file elife-98524-fig8-data1.zip › Fig 8-data1-v1/8M/bottom/Flag.tif]

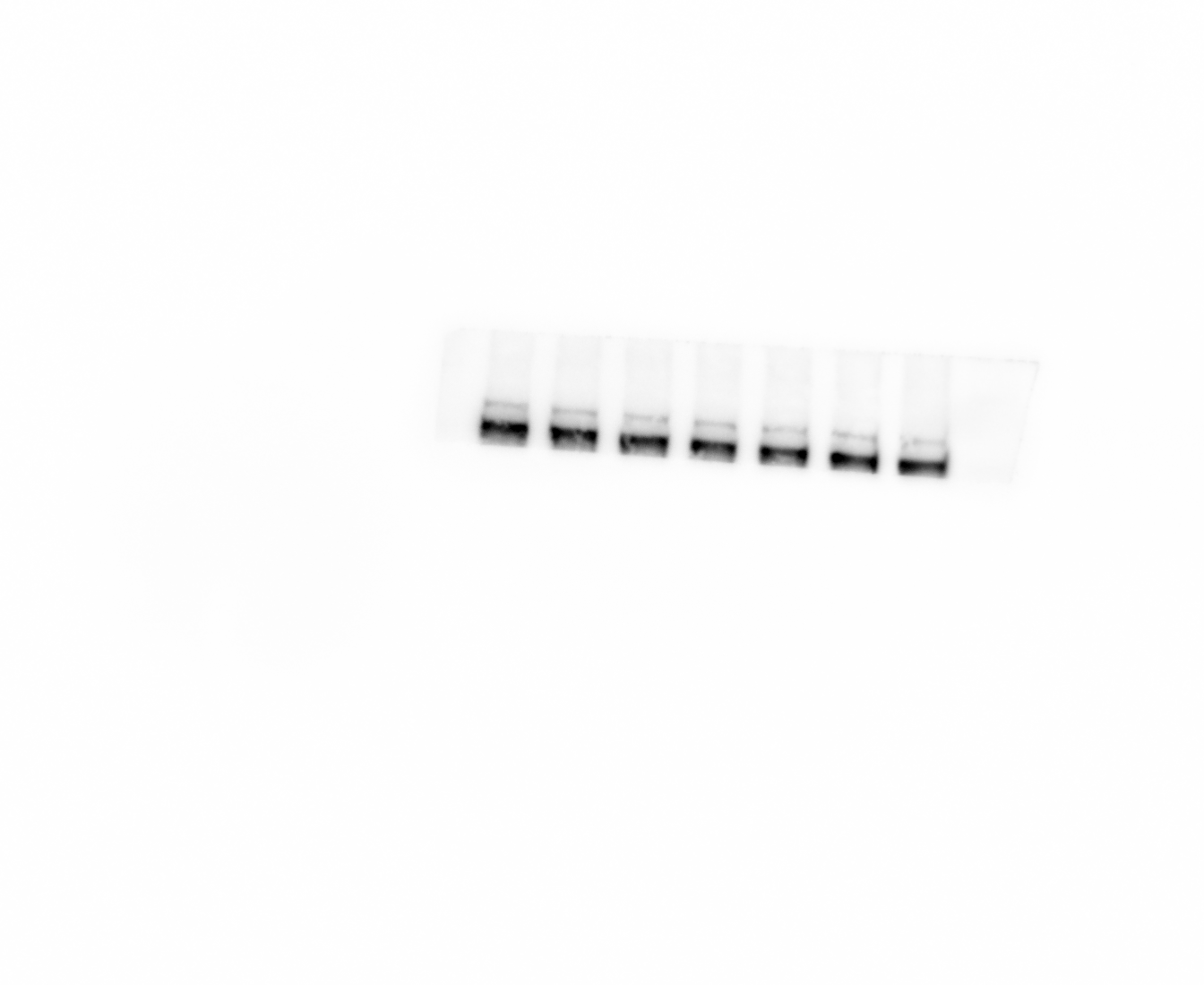

Supplement: Figure 8—source data 1. [file elife-98524-fig8-data1.zip › Fig 8-data1-v1/8M/bottom/SIRT4.tif]

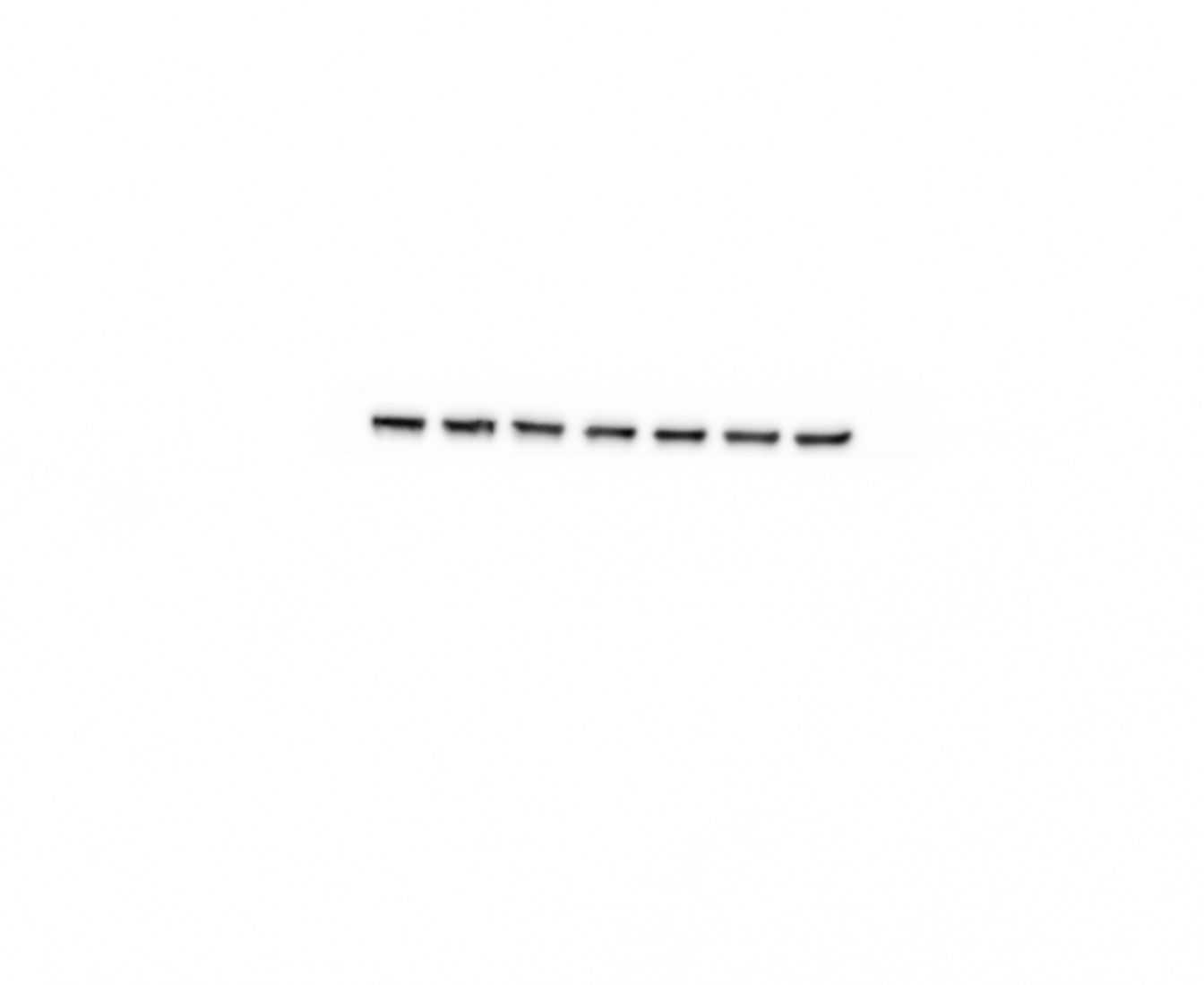

Supplement: Figure 8—source data 1. [file elife-98524-fig8-data1.zip › Fig 8-data1-v1/8M/bottom/Tubulin.tif]

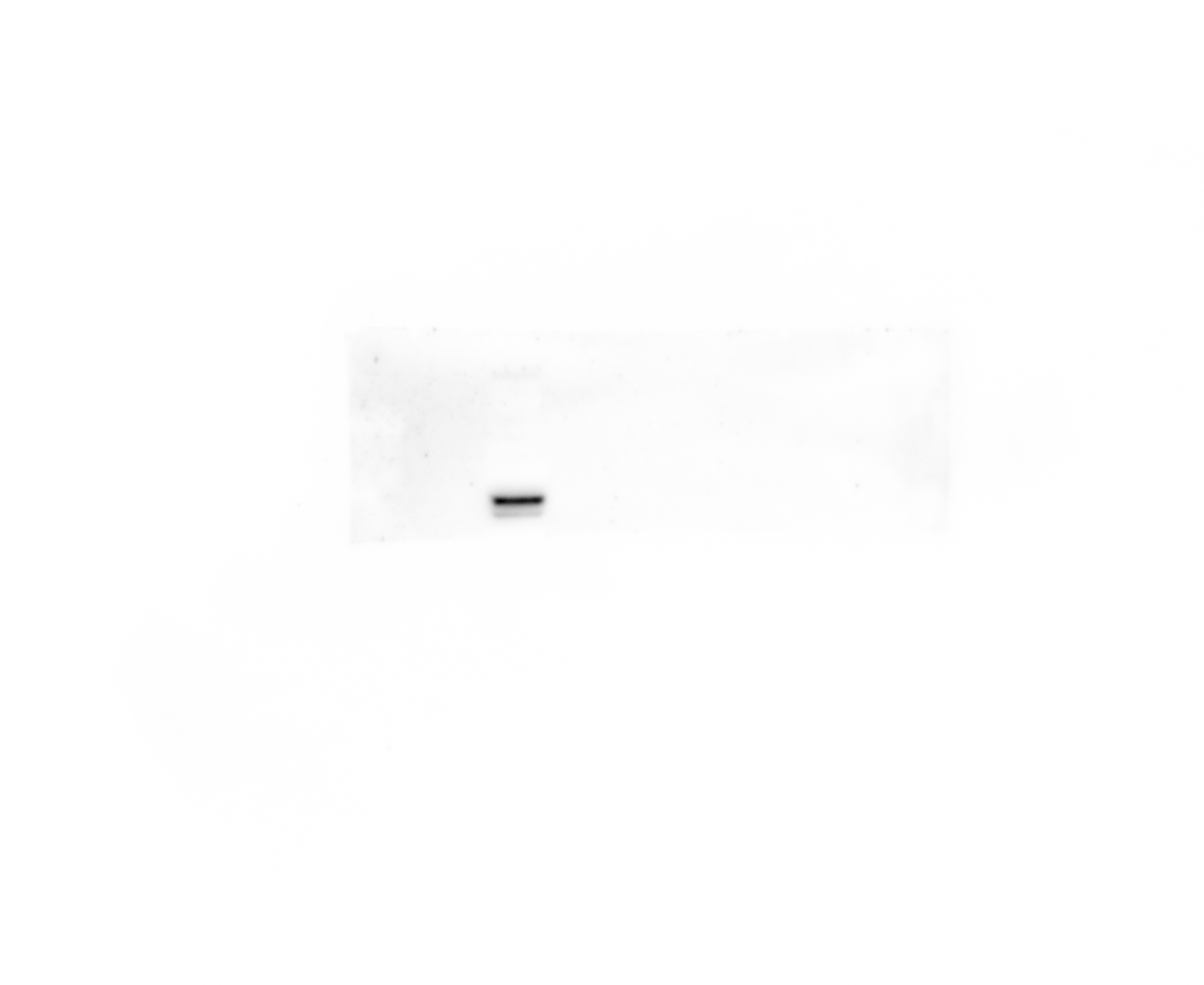

Supplement: Figure 8—source data 1. [file elife-98524-fig8-data1.zip › Fig 8-data1-v1/8M/upper/Flag.tif]

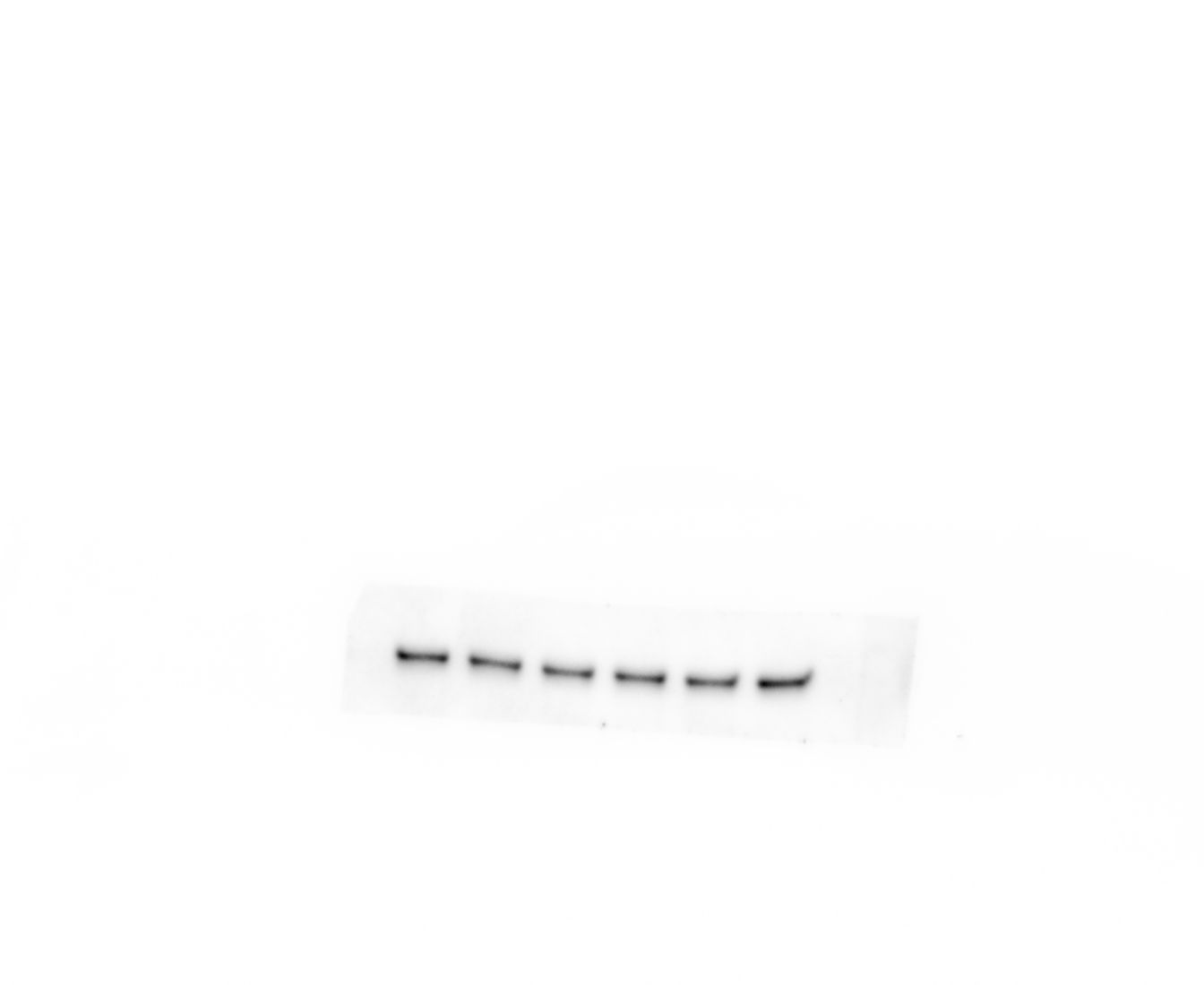

Supplement: Figure 8—source data 1. [file elife-98524-fig8-data1.zip › Fig 8-data1-v1/8M/upper/SIRT4.tif]

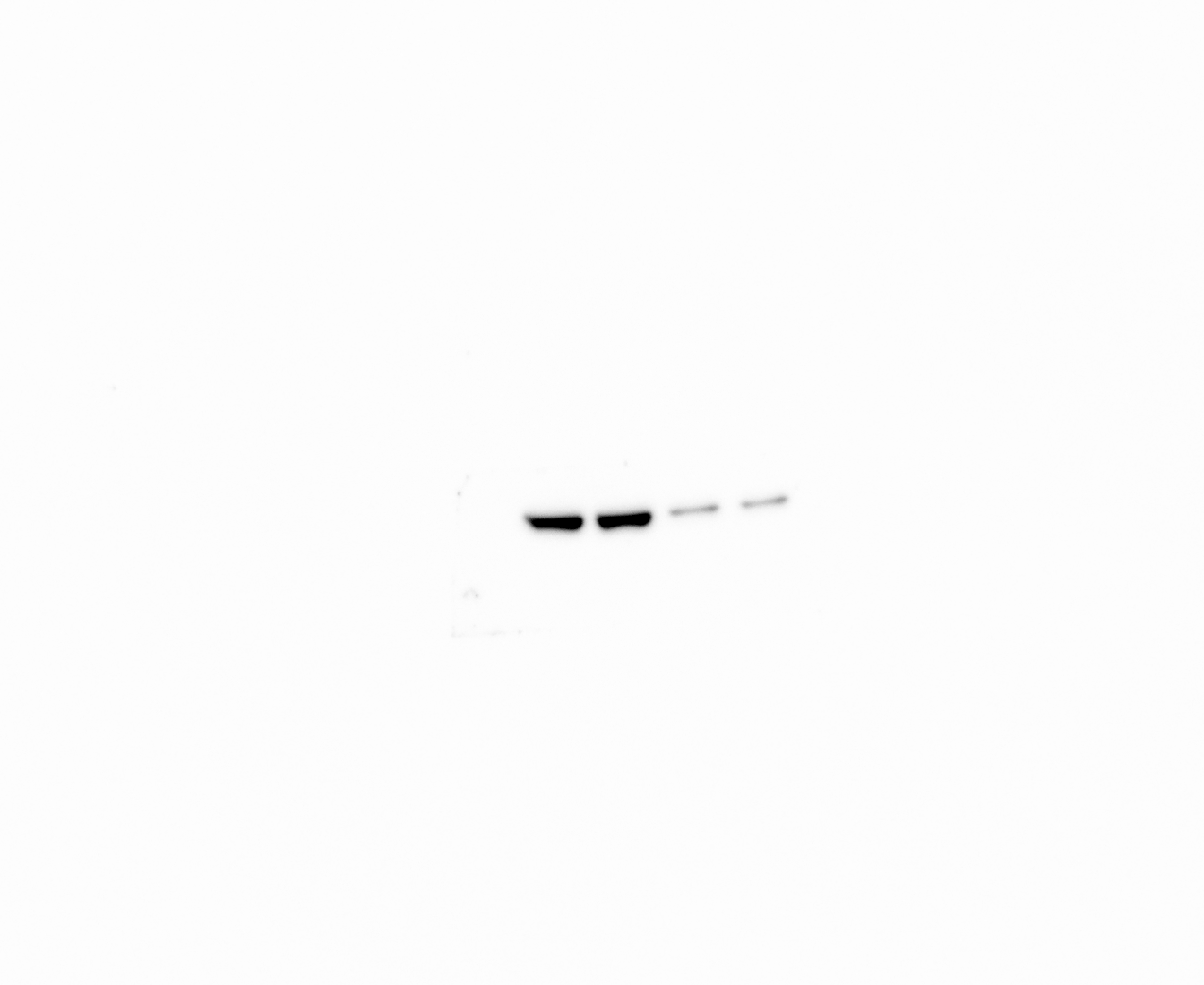

Supplement: Figure 8—source data 1. [file elife-98524-fig8-data1.zip › Fig 8-data1-v1/8N/bottom/Importin α1.tif]

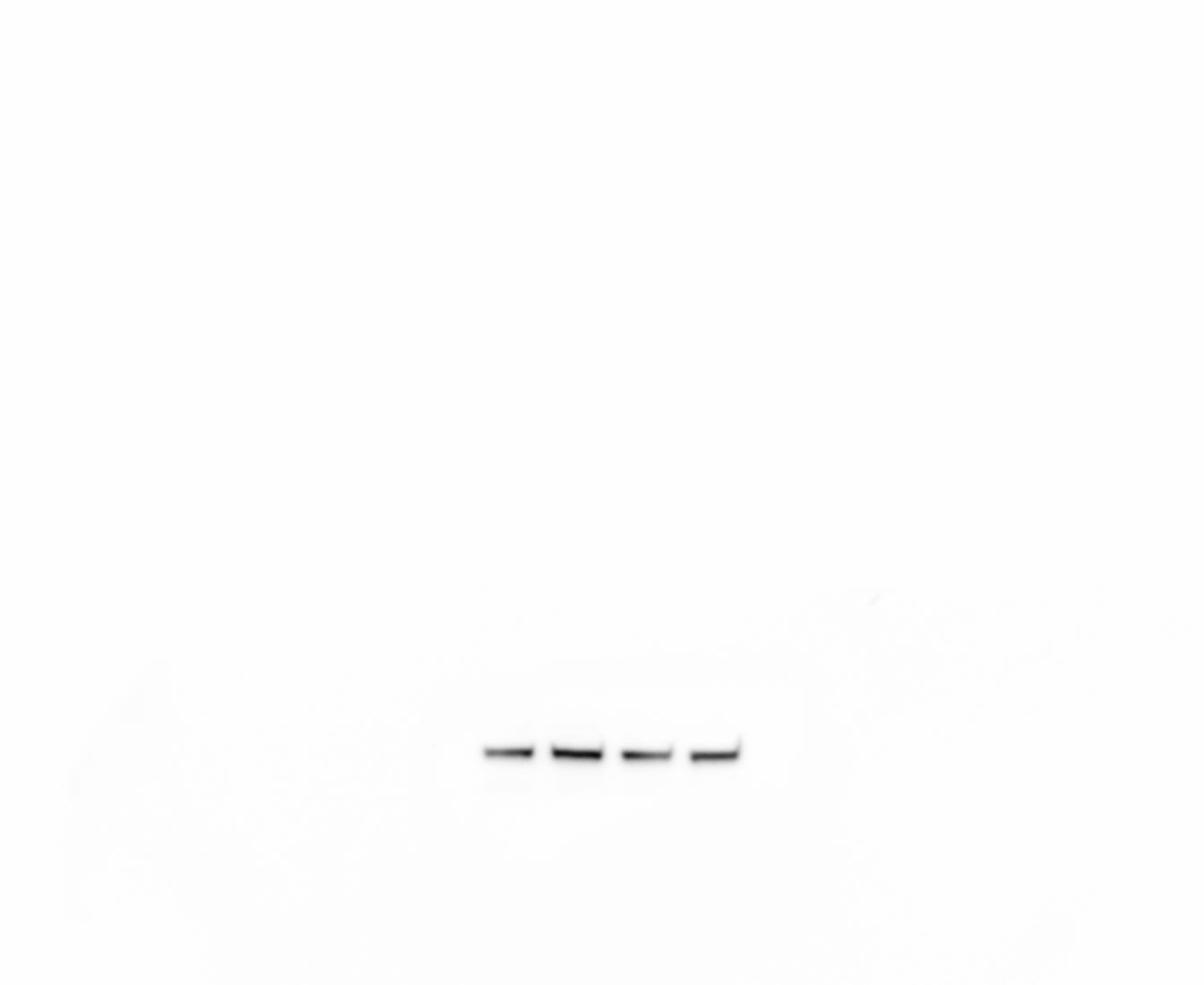

Supplement: Figure 8—source data 1. [file elife-98524-fig8-data1.zip › Fig 8-data1-v1/8N/bottom/Tubulin.tif]

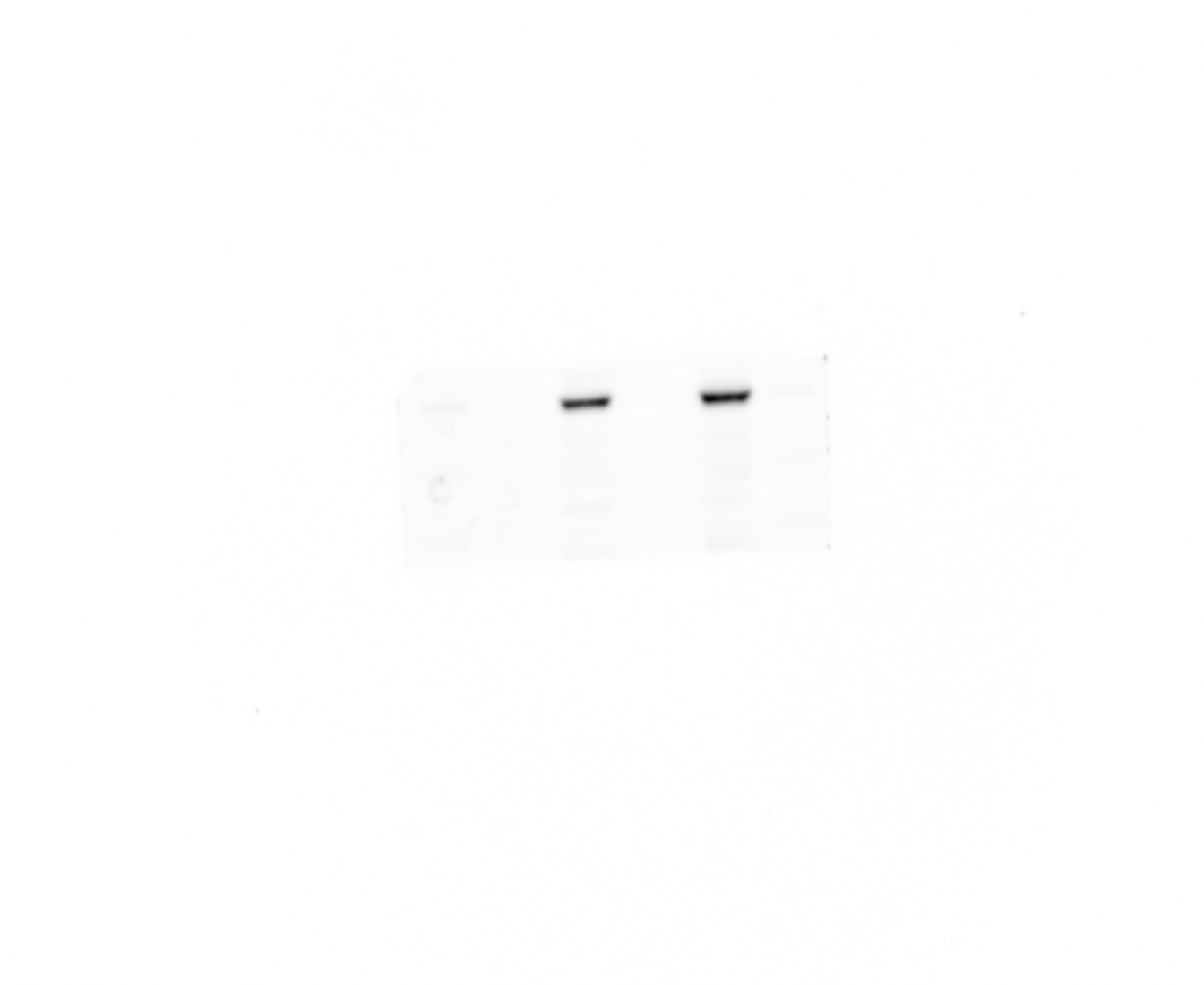

Supplement: Figure 8—source data 1. [file elife-98524-fig8-data1.zip › Fig 8-data1-v1/8N/middle/Phos.tif]

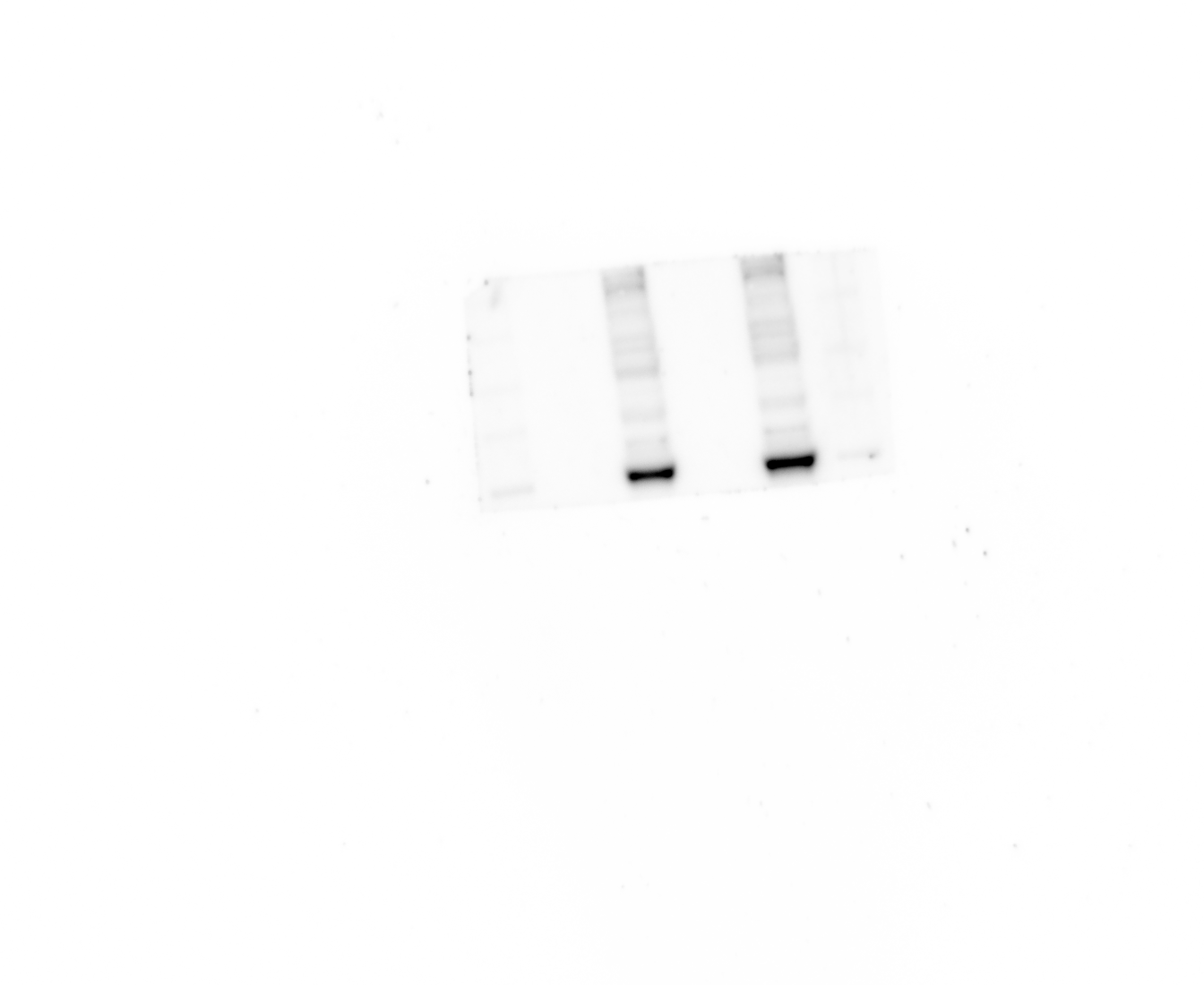

Supplement: Figure 8—source data 1. [file elife-98524-fig8-data1.zip › Fig 8-data1-v1/8N/middle/SIRT4.tif]

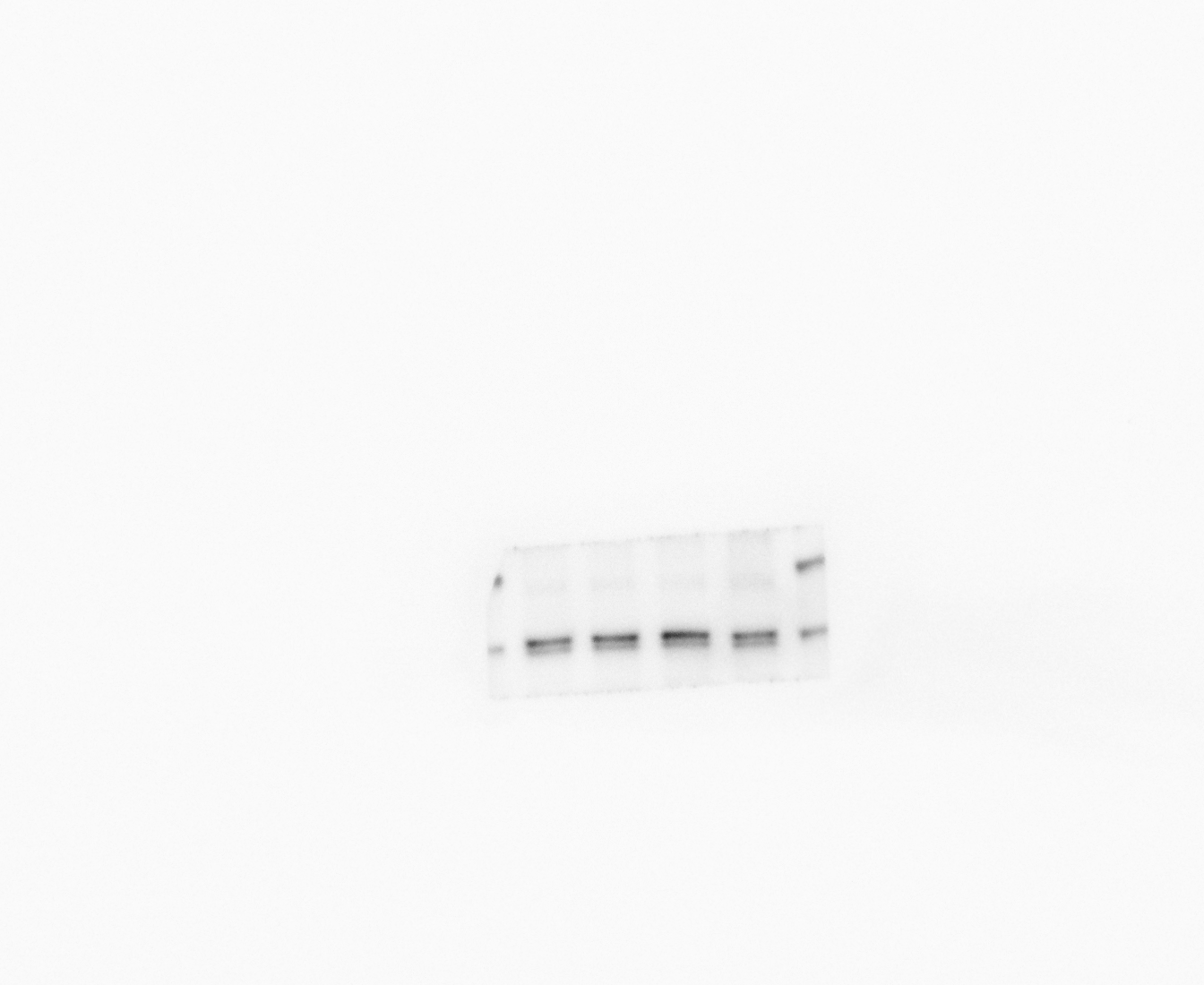

Supplement: Figure 8—source data 1. [file elife-98524-fig8-data1.zip › Fig 8-data1-v1/8N/upper/PCNA.tif]

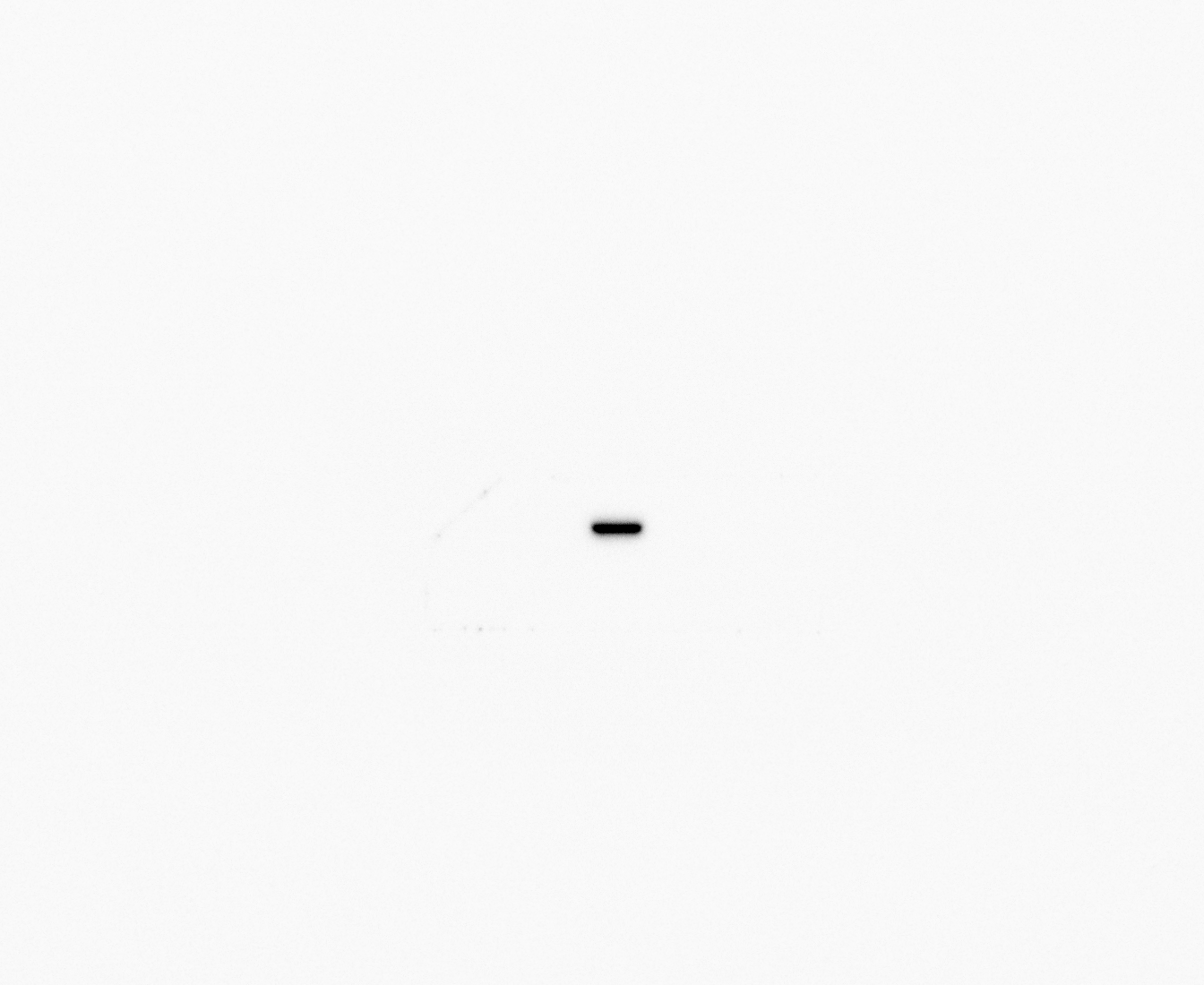

Supplement: Figure 8—source data 1. [file elife-98524-fig8-data1.zip › Fig 8-data1-v1/8N/upper/SIRT4.tif]

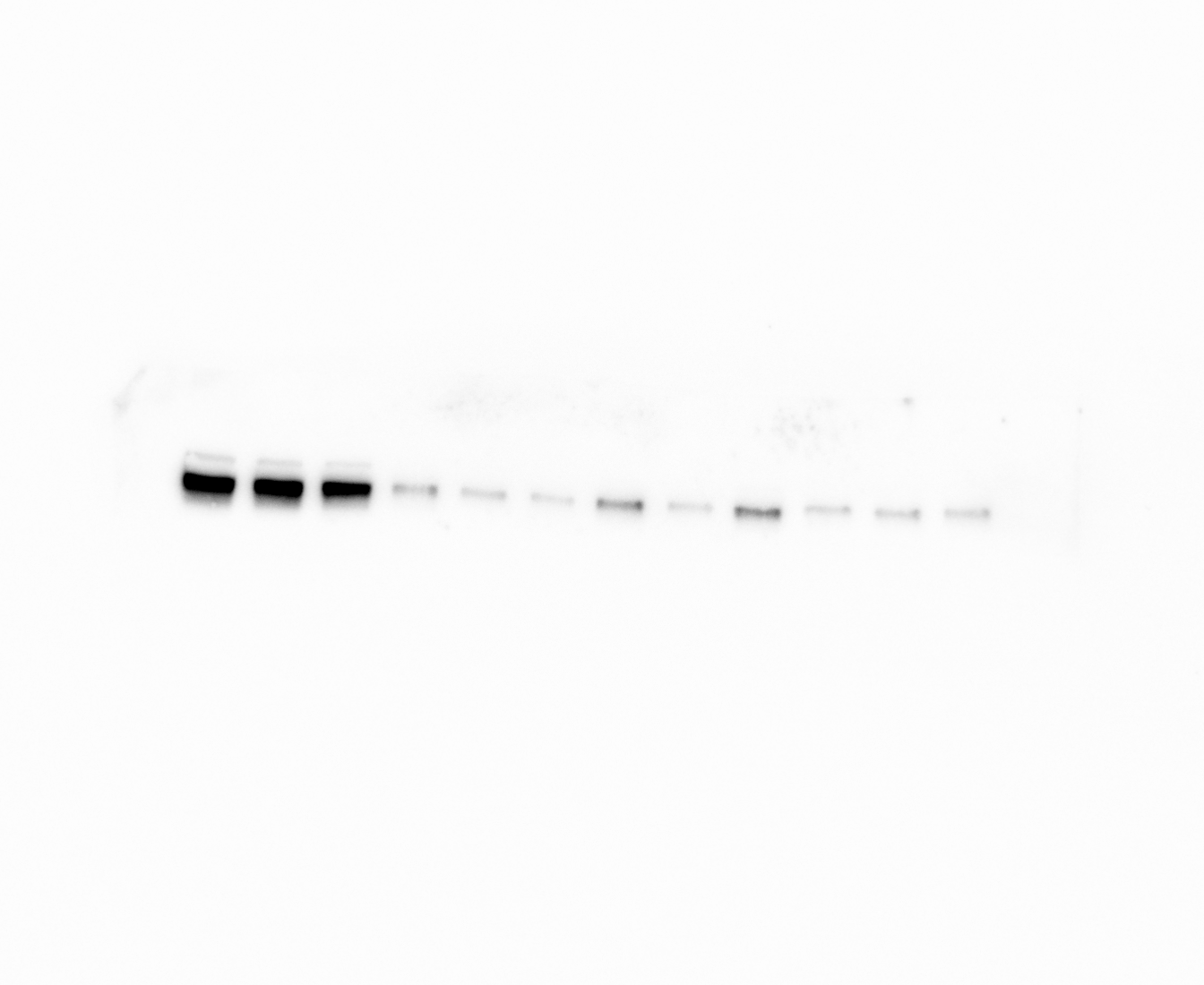

Supplement: Figure 8—source data 1. [file elife-98524-fig8-data1.zip › Fig 8-data1-v1/8O/CCN2.tif]

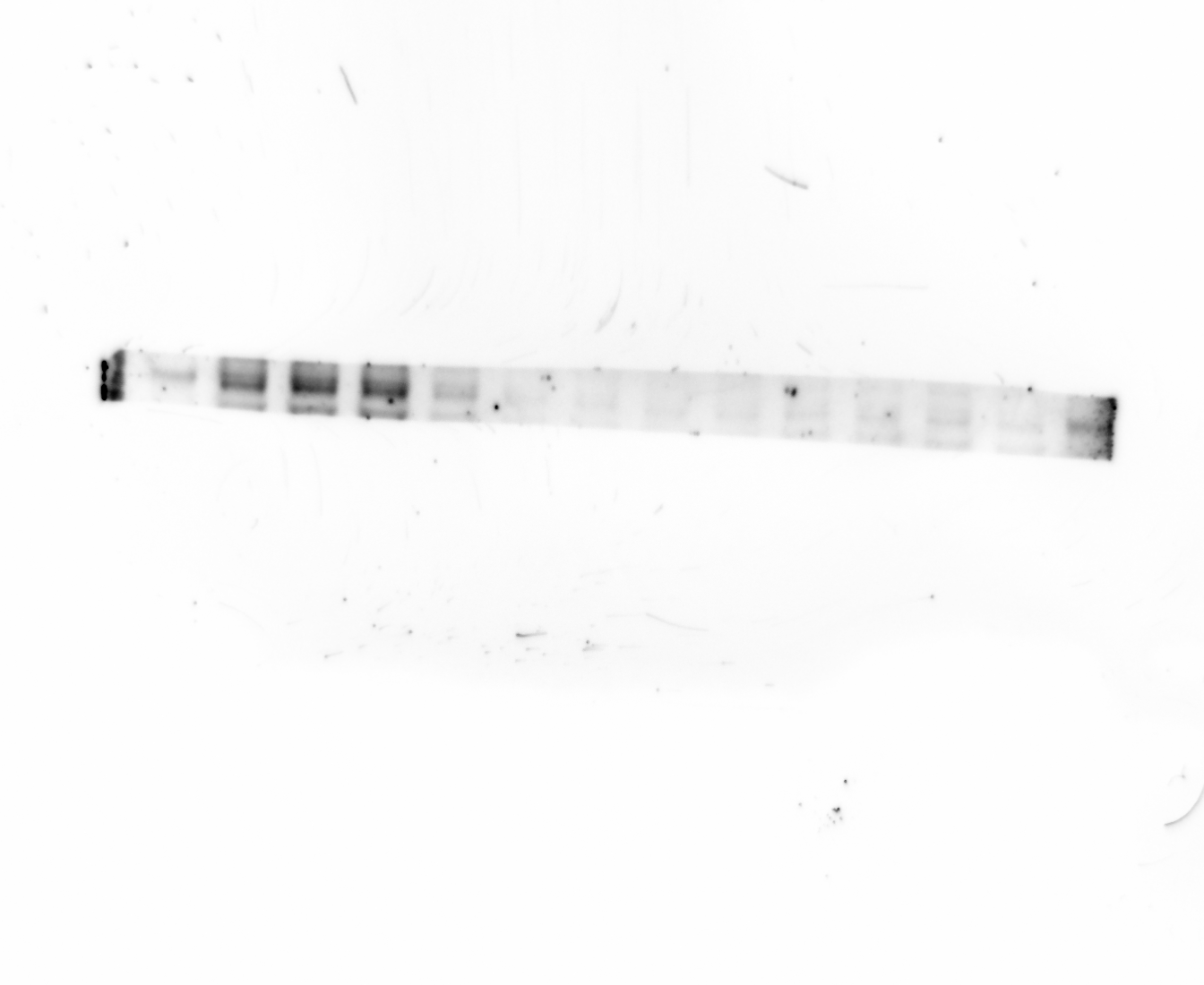

Supplement: Figure 8—source data 1. [file elife-98524-fig8-data1.zip › Fig 8-data1-v1/8O/COL3A1.tif]

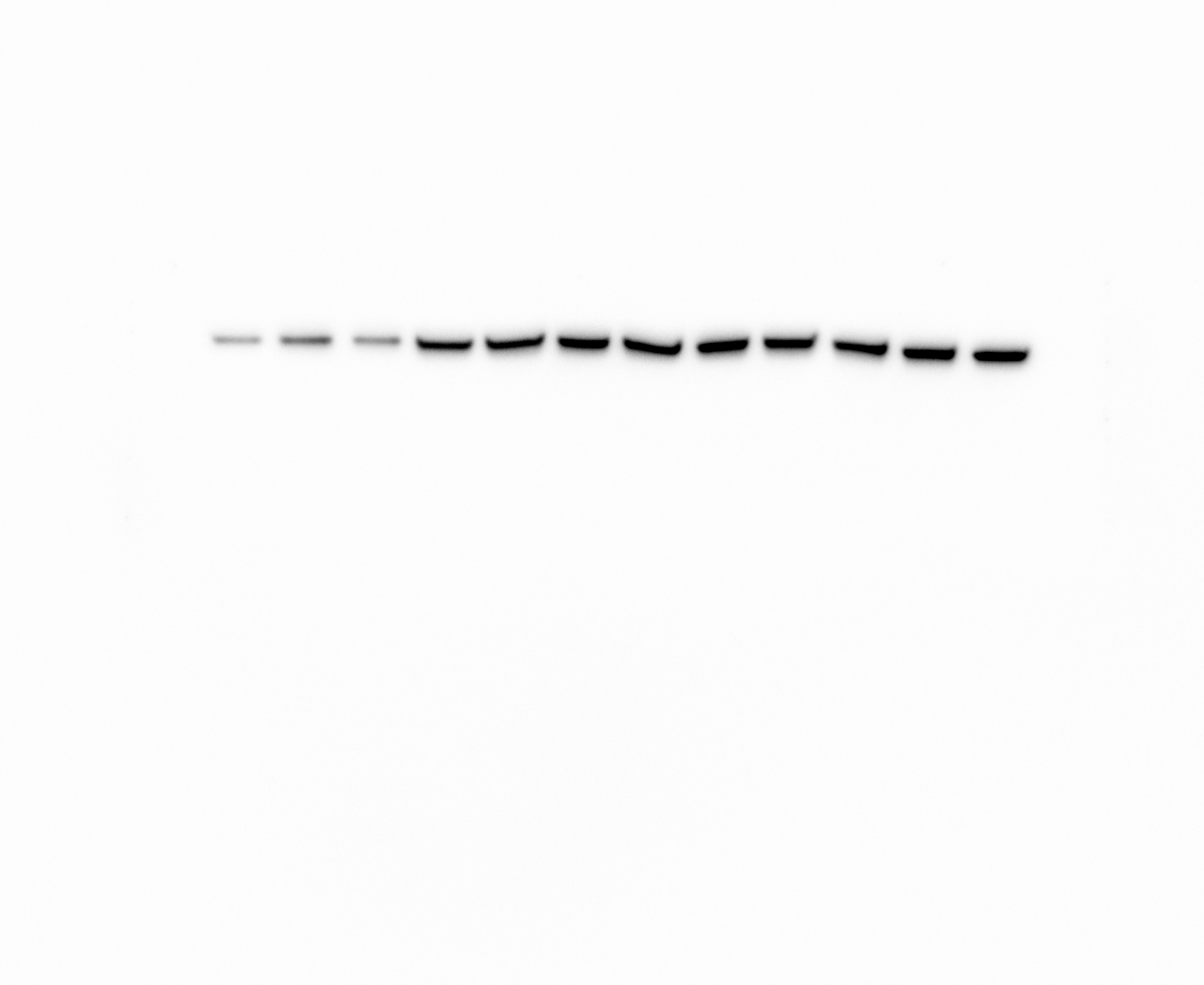

Supplement: Figure 8—source data 1. [file elife-98524-fig8-data1.zip › Fig 8-data1-v1/8O/E-cadherin.tif]

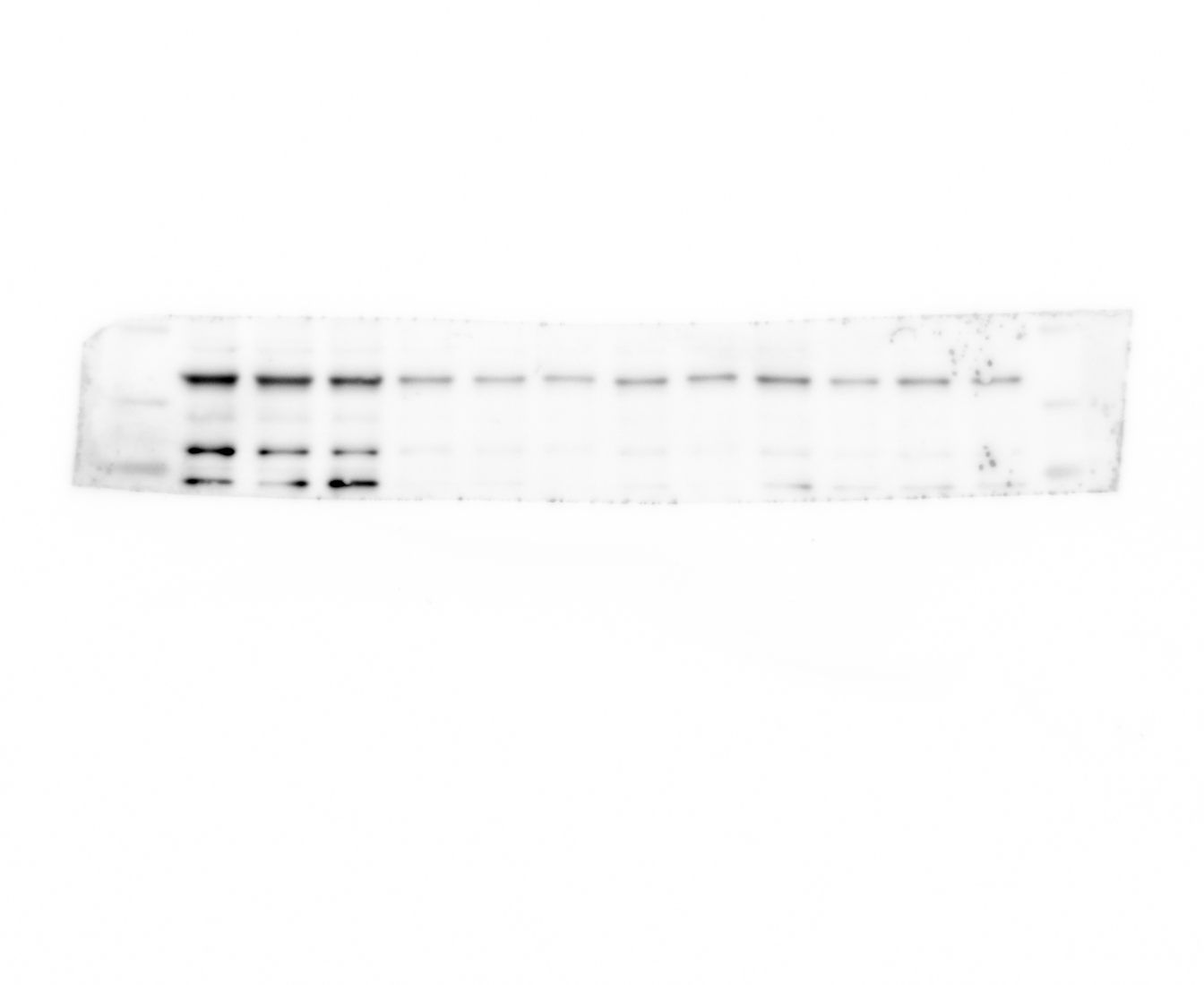

Supplement: Figure 8—source data 1. [file elife-98524-fig8-data1.zip › Fig 8-data1-v1/8O/FN1.tif]

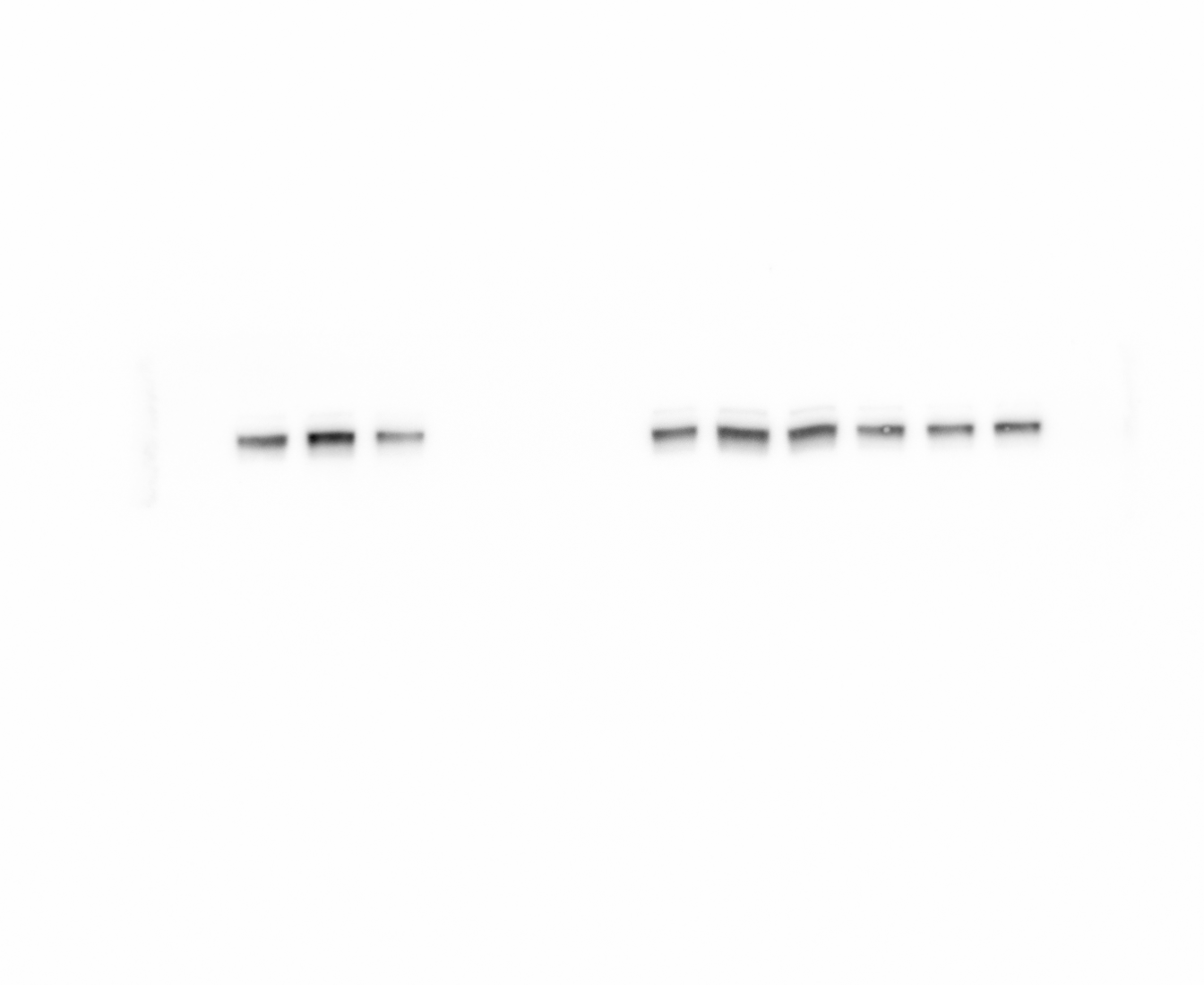

Supplement: Figure 8—source data 1. [file elife-98524-fig8-data1.zip › Fig 8-data1-v1/8O/SIRT4.tif]

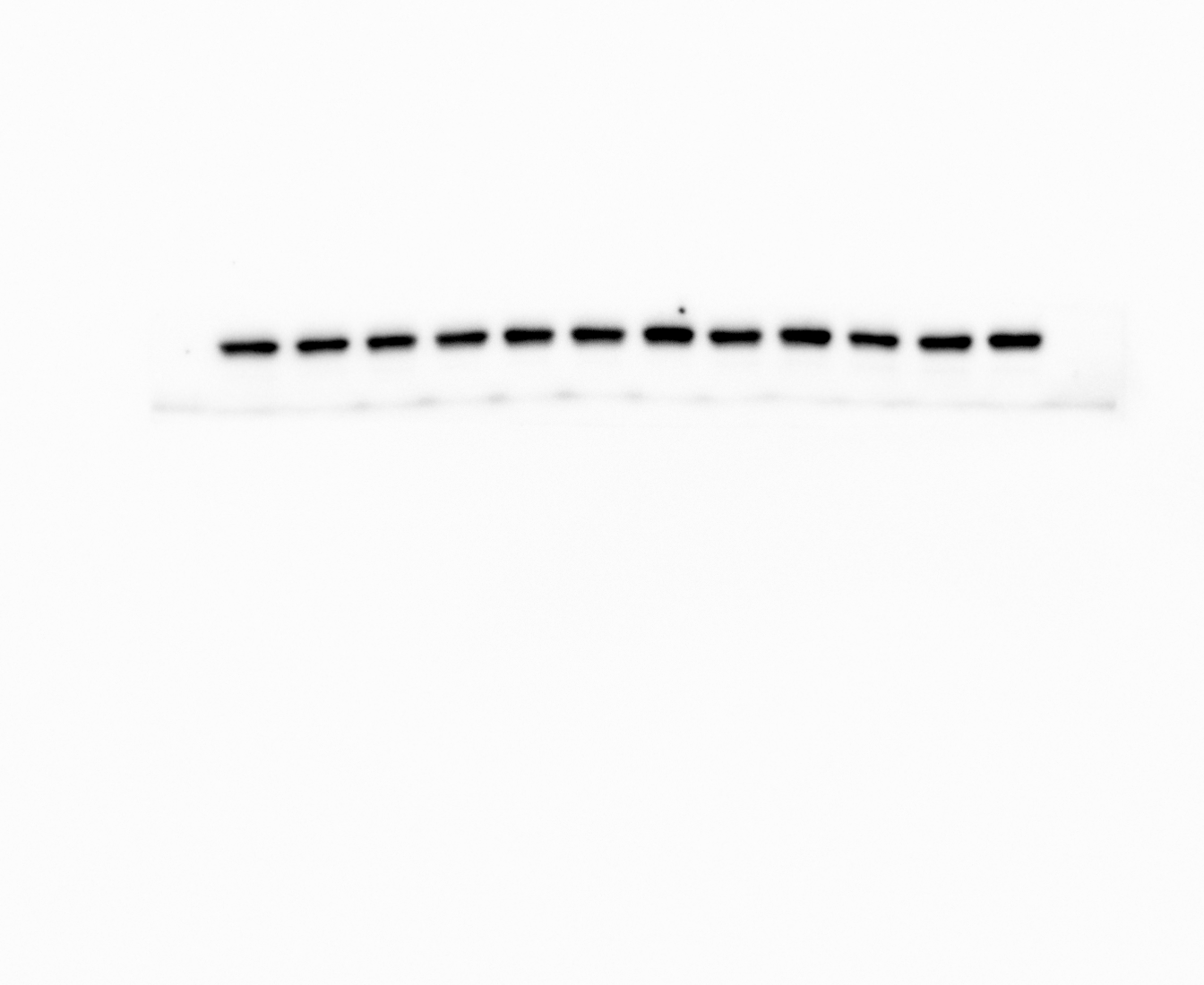

Supplement: Figure 8—source data 1. [file elife-98524-fig8-data1.zip › Fig 8-data1-v1/8O/Tubulin.tif]

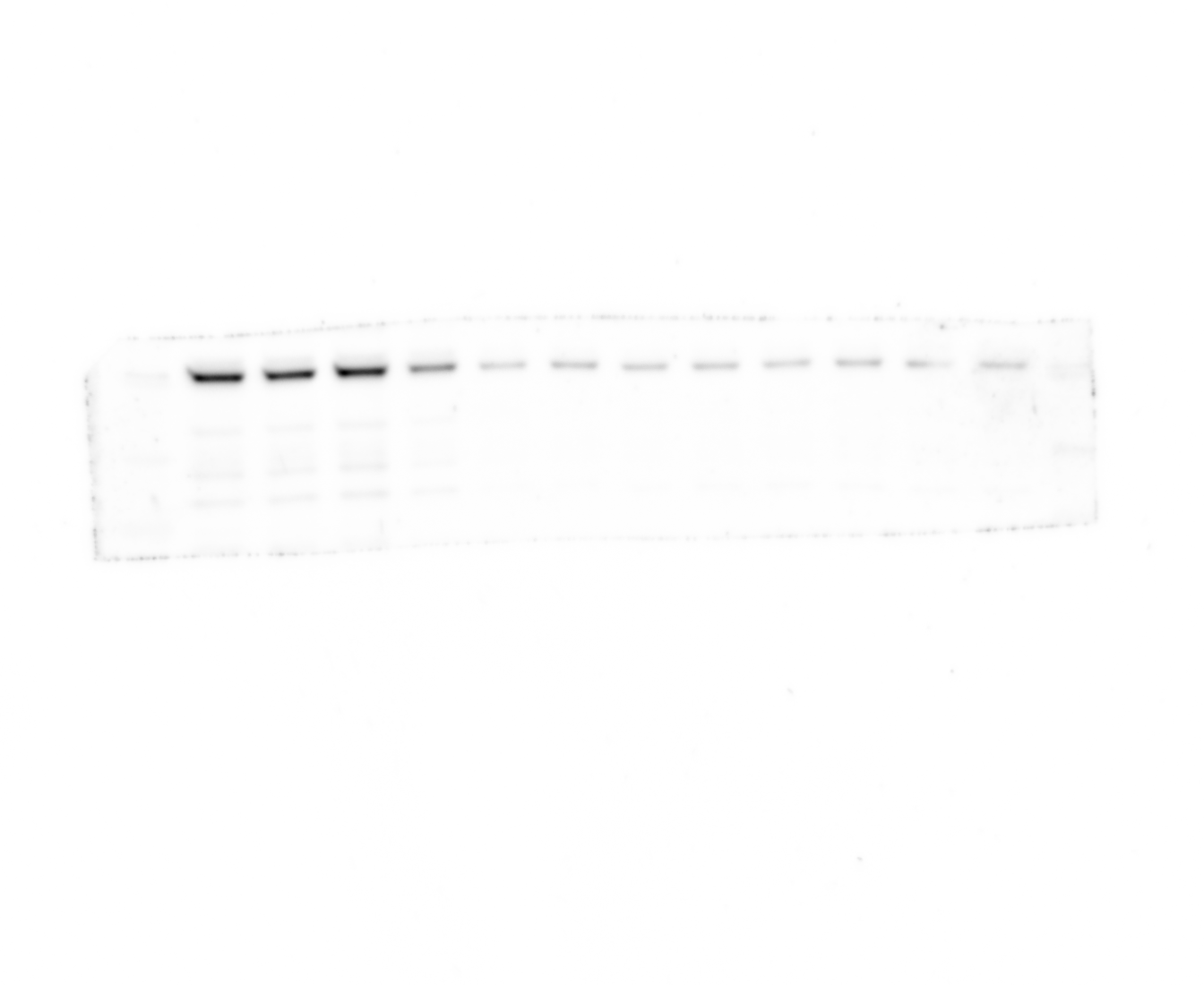

Supplement: Figure 8—source data 1. [file elife-98524-fig8-data1.zip › Fig 8-data1-v1/8O/α-SMA.tif]

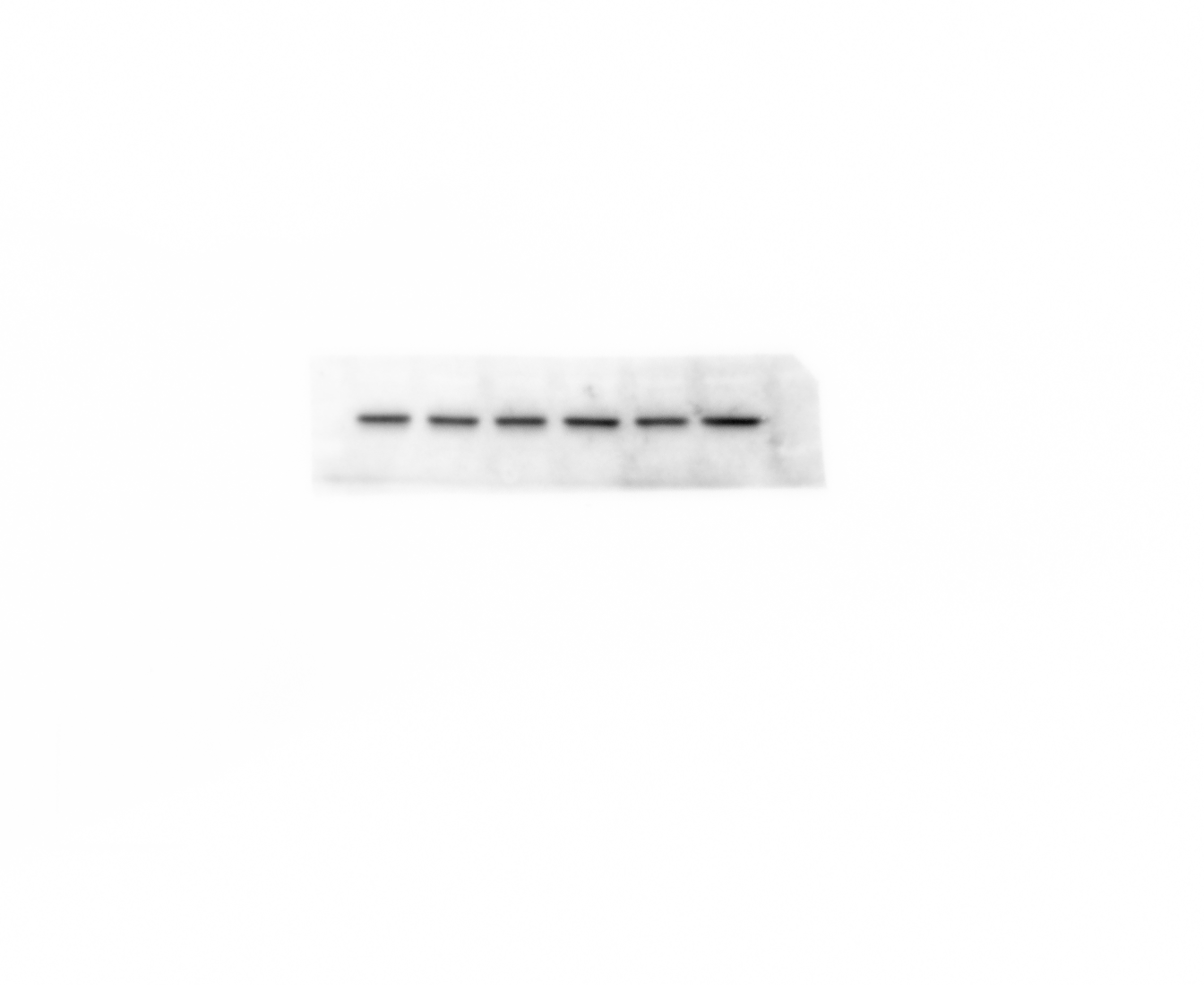

Supplement: Figure 8—source data 1. [file elife-98524-fig8-data1.zip › Fig 8-data1-v1/8R/PCNA.tif]

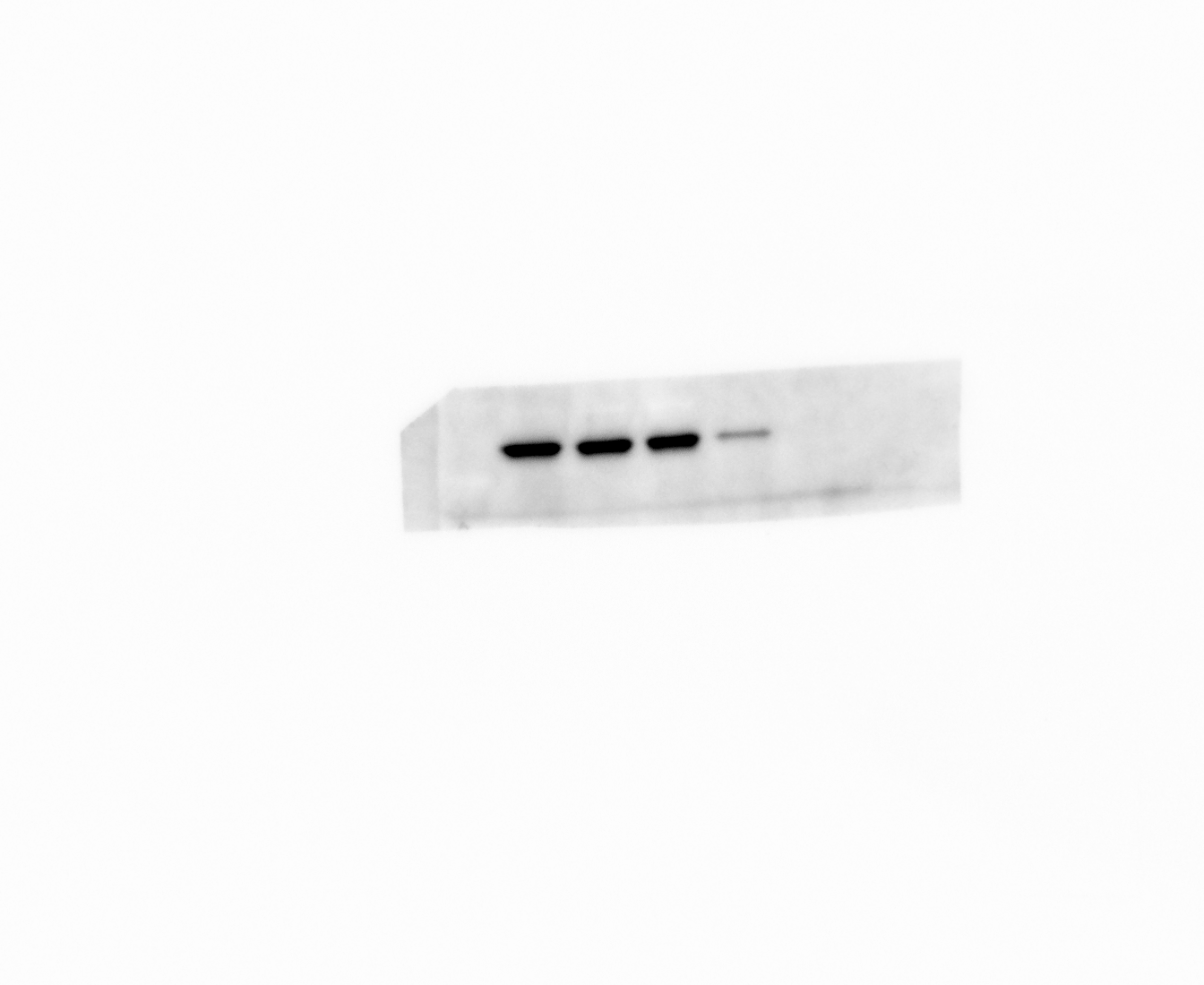

Supplement: Figure 8—source data 1. [file elife-98524-fig8-data1.zip › Fig 8-data1-v1/8R/SIRT4.tif]

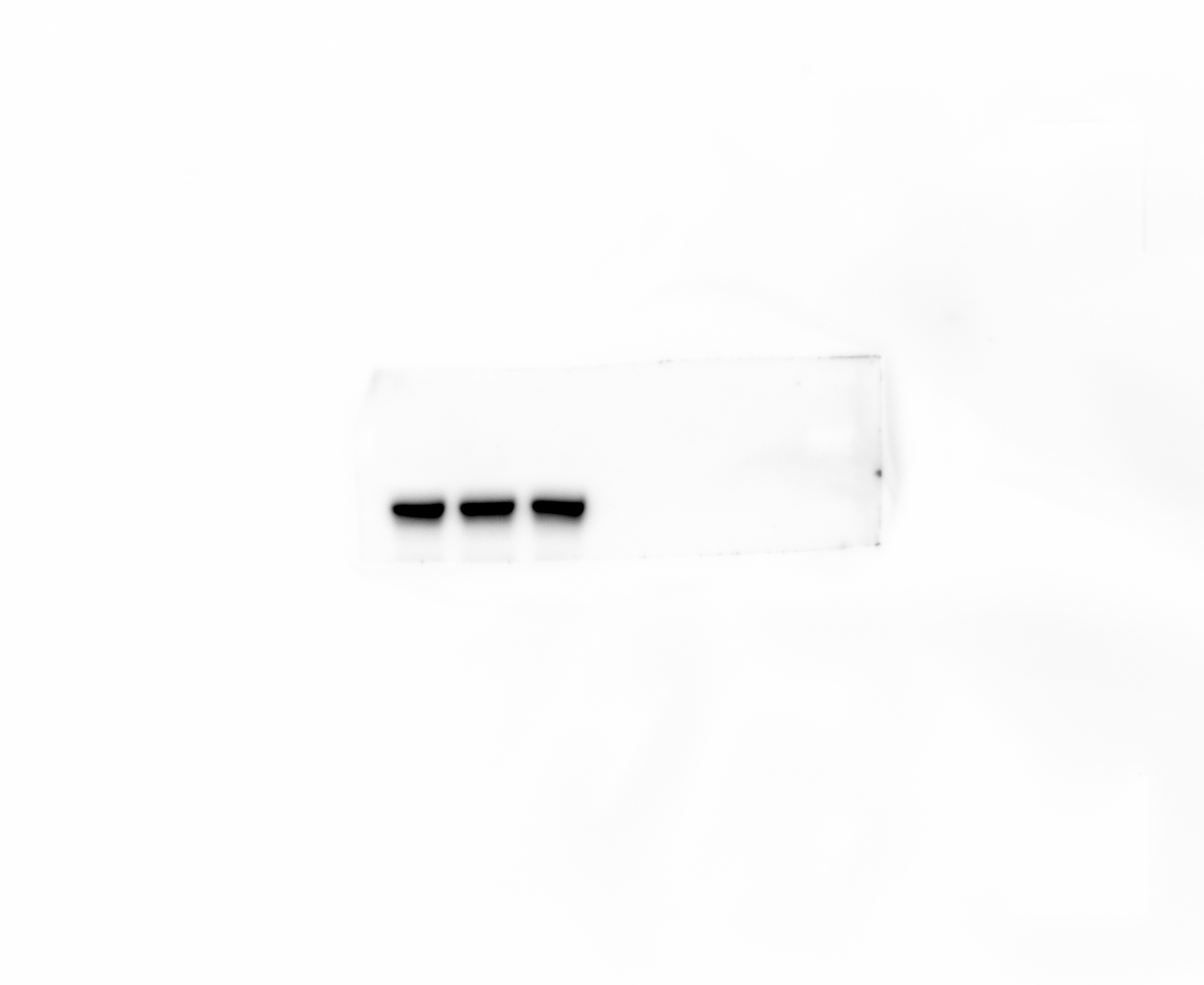

Supplement: Figure 8—source data 1. [file elife-98524-fig8-data1.zip › Fig 8-data1-v1/8R/Tubulin.tif]

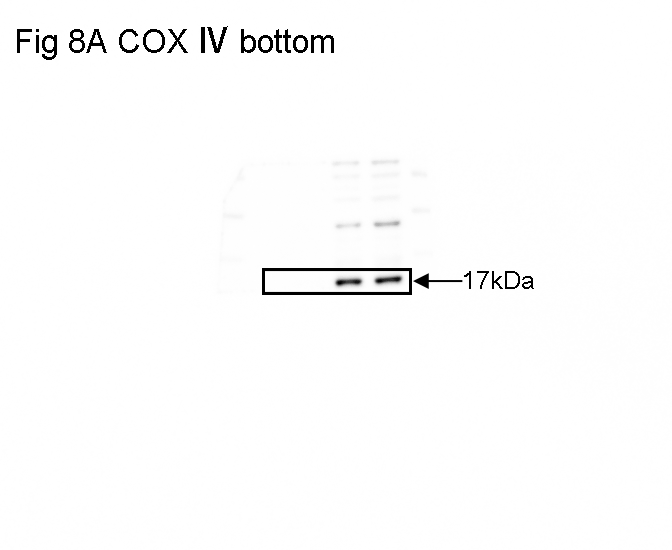

Supplement: Figure 8—source data 2. [file elife-98524-fig8-data2.zip › Fig 8-data2-v1/8A/bottom/COX IV.tif]
