# Supplementary figures and images for: Nuclear translocation of SIRT4 mediates deacetylation of U2AF2 to modulate renal fibrosis through alternative splicing-mediated upregulation of CCN2 (part 9 of 9)
Source: eLife. 2024 Nov 4;13:RP98524. doi: 10.7554/eLife.98524 (PMC11534337; doi:10.7554/eLife.98524)

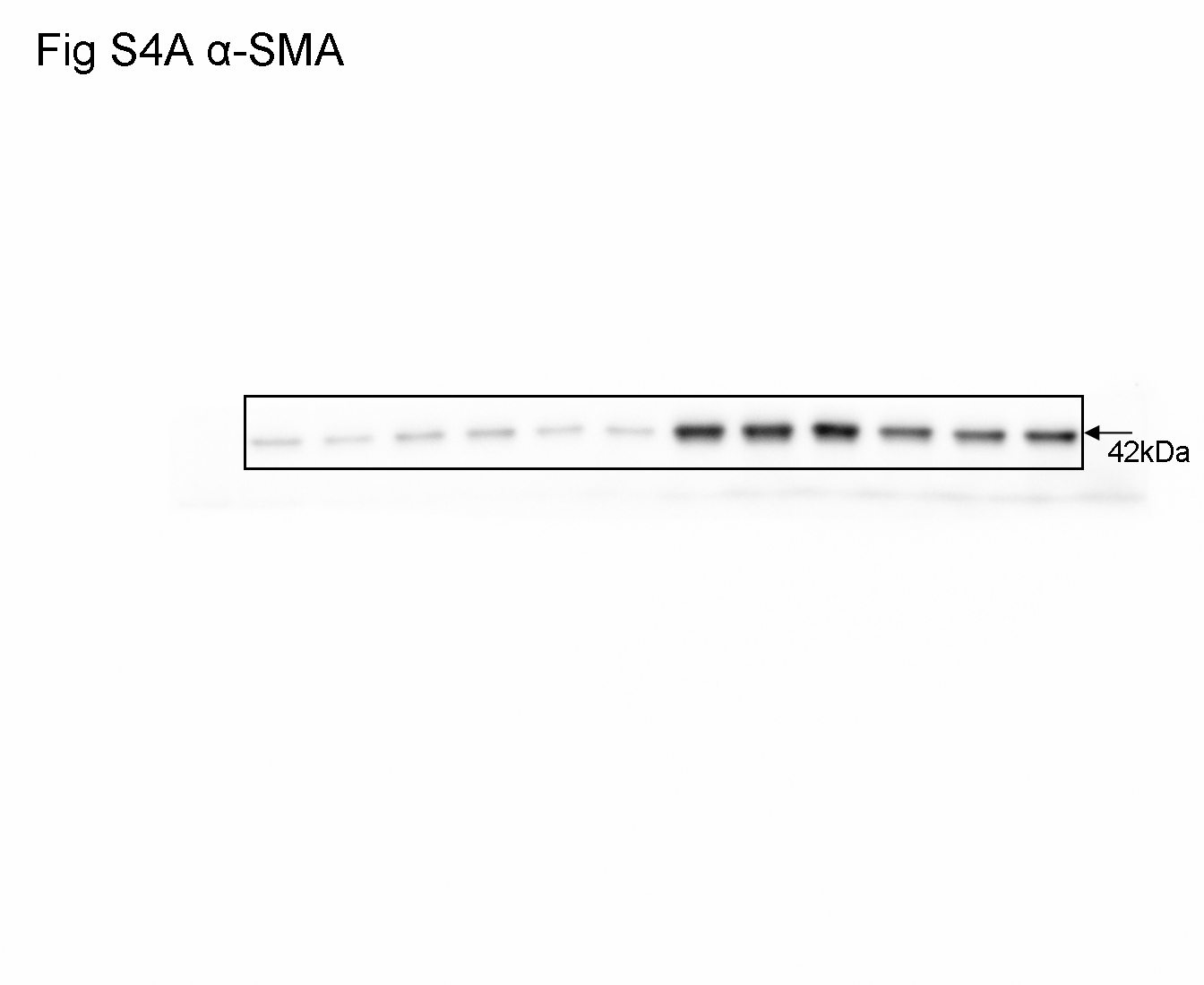

Supplement: Figure 8—figure supplement 2—source data 2. [file elife-98524-fig8-figsupp2-data2.zip › Fig 8-fig S4-data2-v1/S4A/α-SMA.tif]

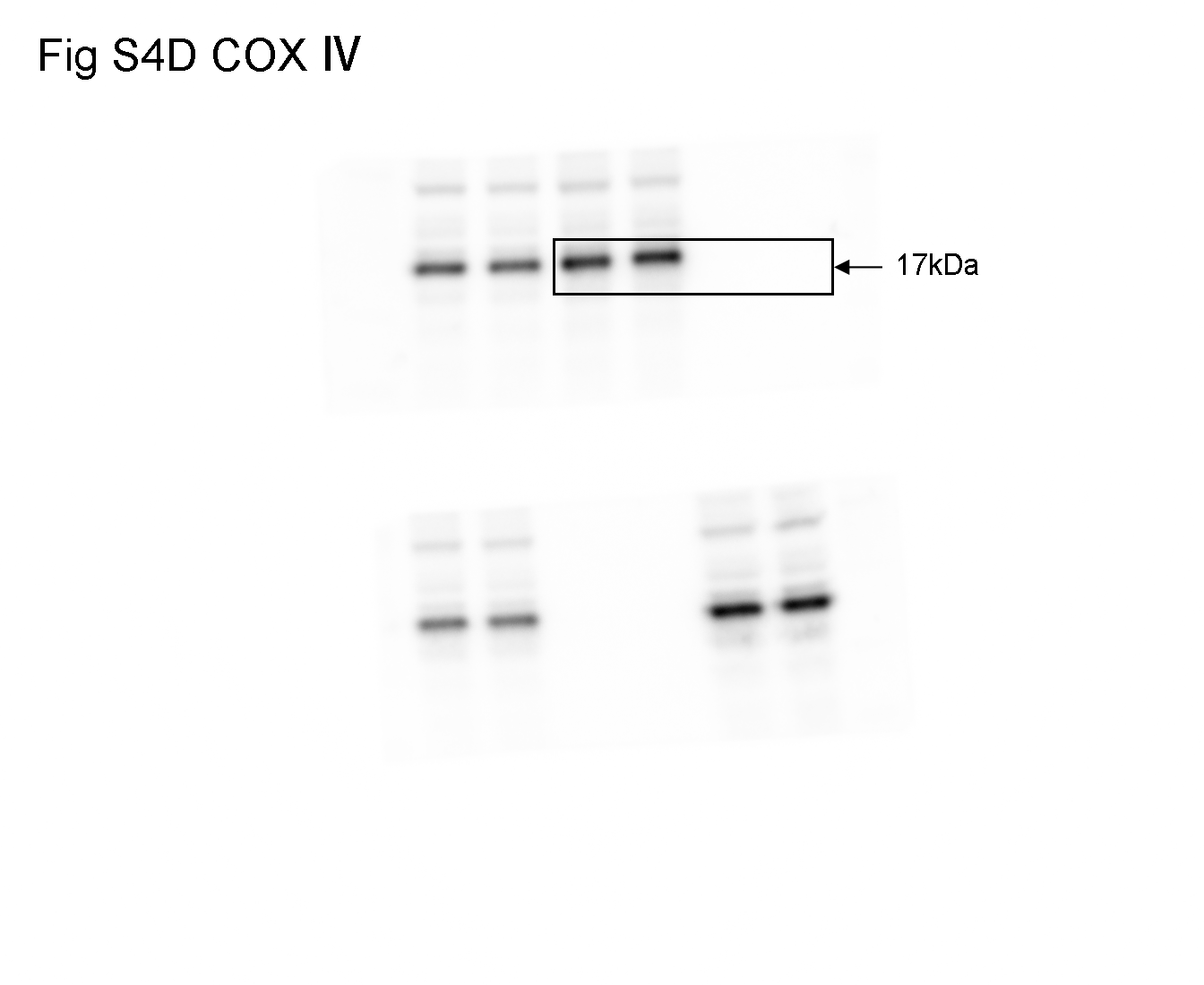

Supplement: Figure 8—figure supplement 2—source data 2. [file elife-98524-fig8-figsupp2-data2.zip › Fig 8-fig S4-data2-v1/S4D/COX IV.tif]

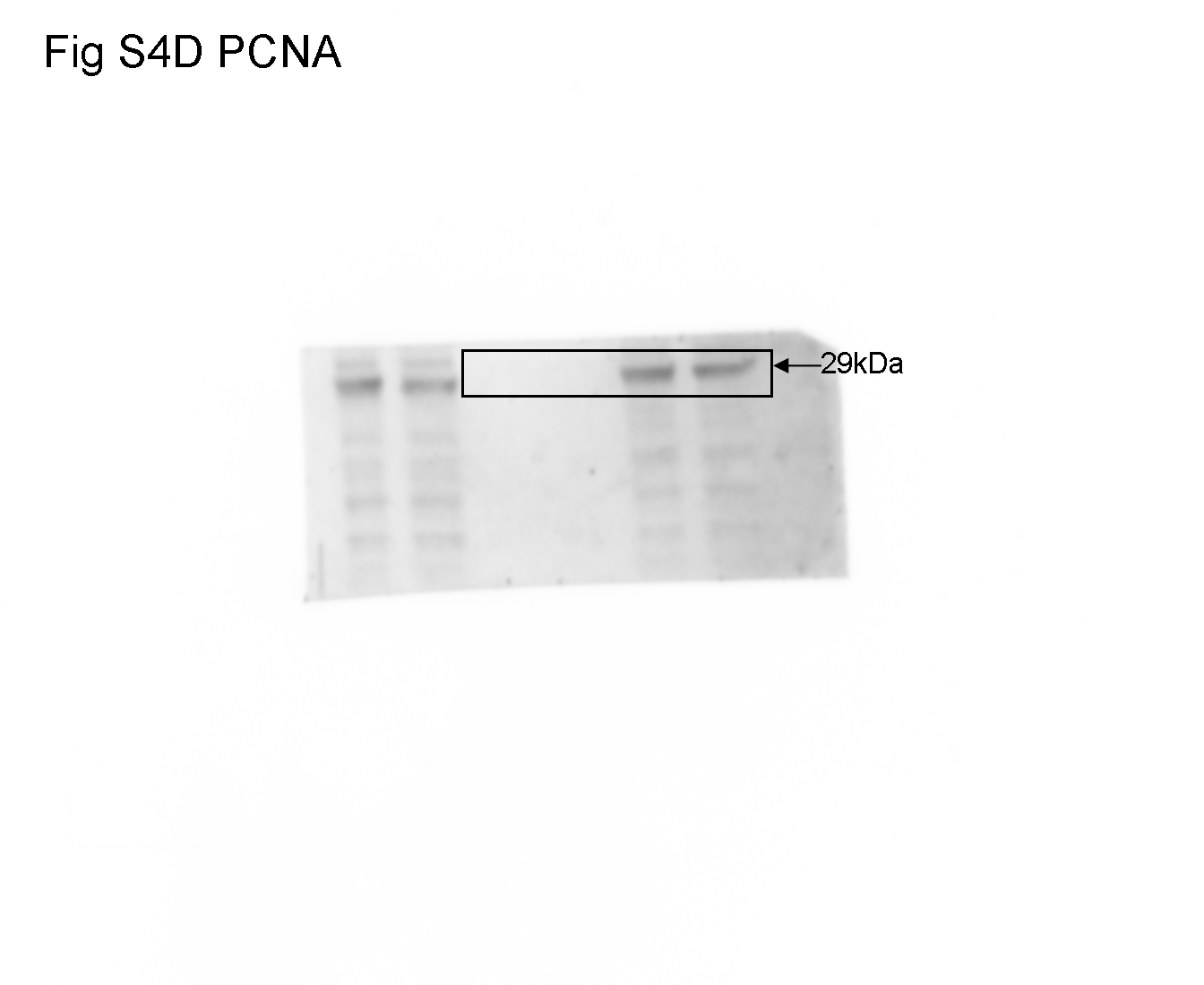

Supplement: Figure 8—figure supplement 2—source data 2. [file elife-98524-fig8-figsupp2-data2.zip › Fig 8-fig S4-data2-v1/S4D/PCNA.tif]

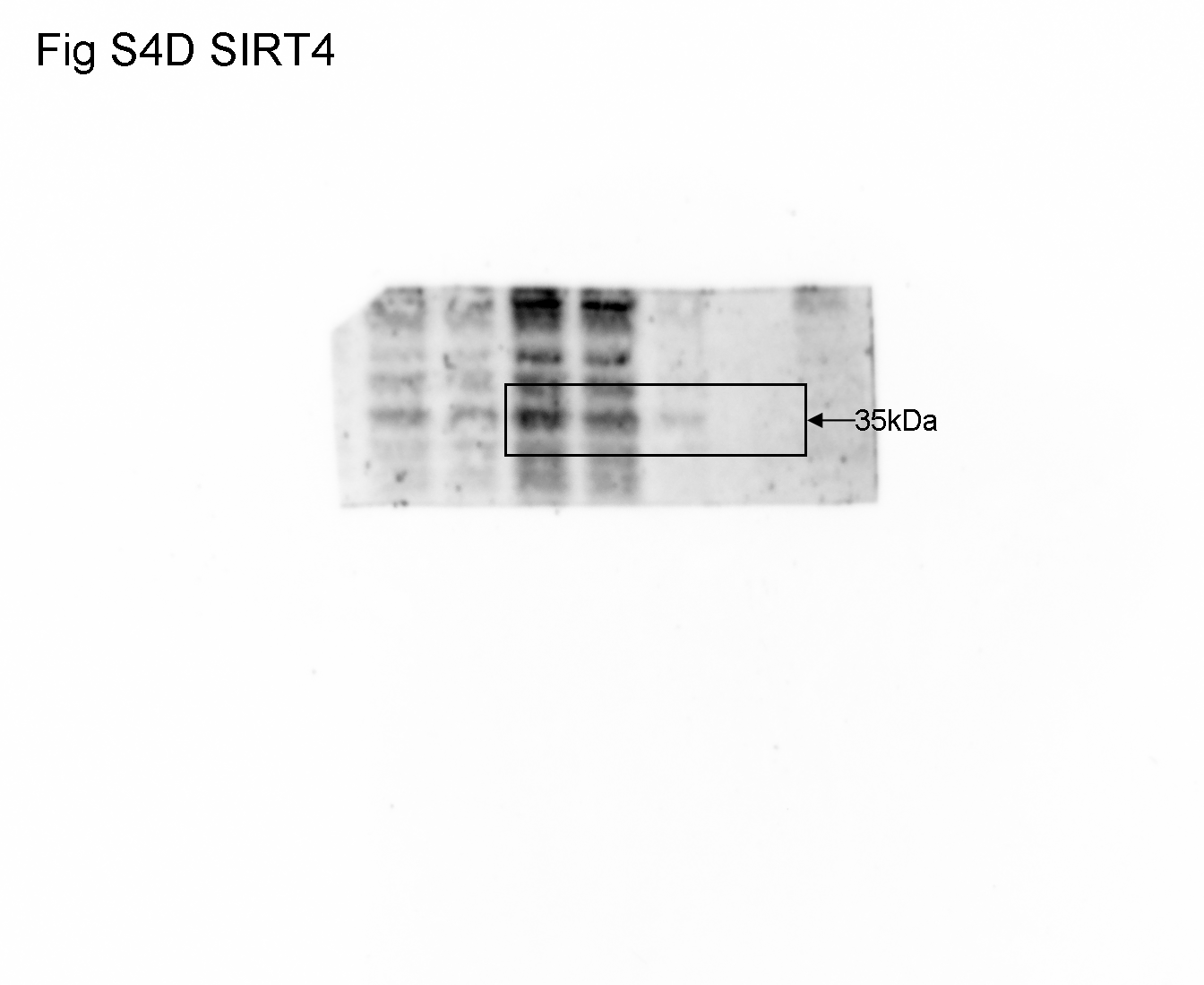

Supplement: Figure 8—figure supplement 2—source data 2. [file elife-98524-fig8-figsupp2-data2.zip › Fig 8-fig S4-data2-v1/S4D/SIRT4.tif]

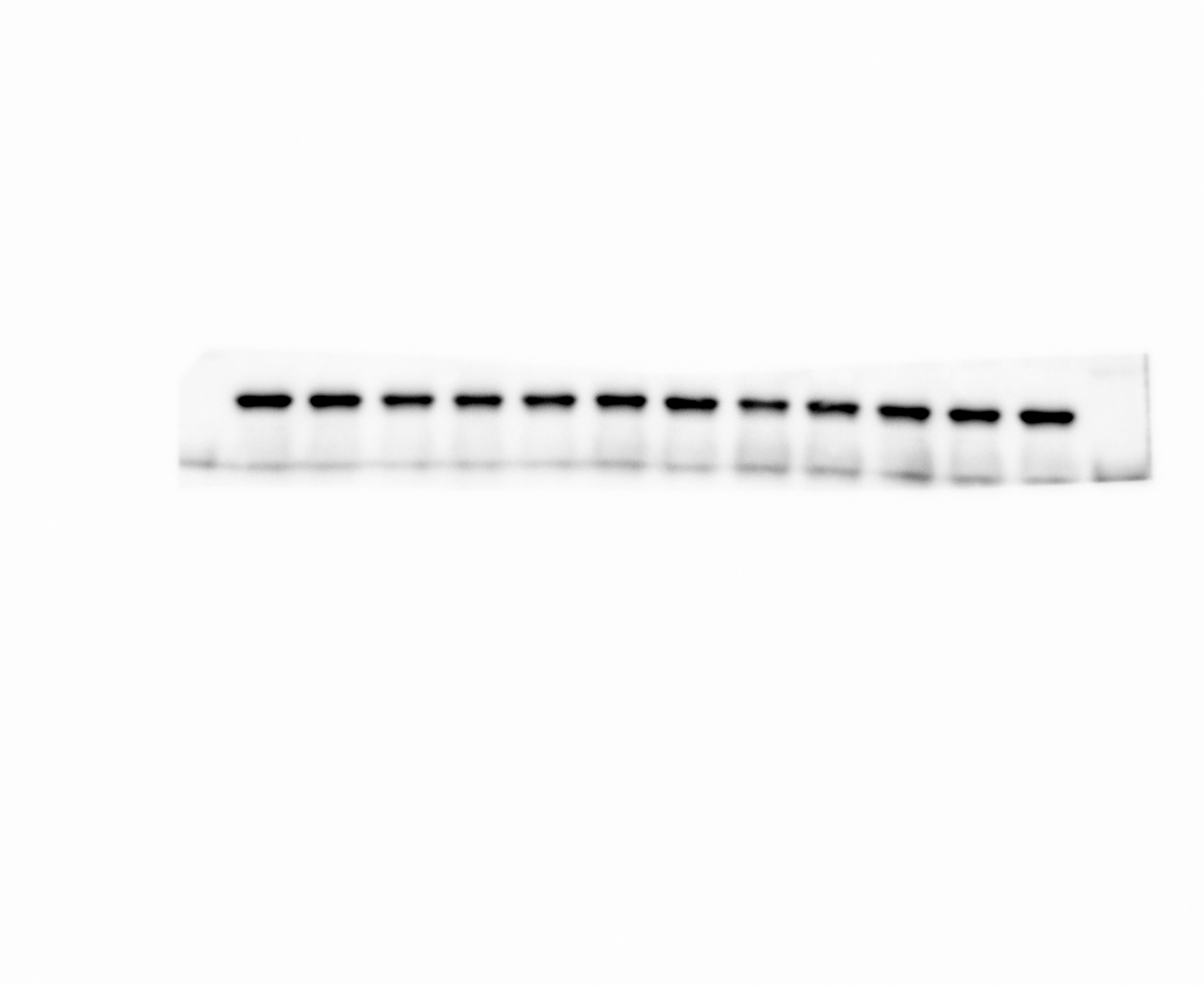

Supplement: Figure 8—figure supplement 3—source data 1. [file elife-98524-fig8-figsupp3-data1.zip › Fig 8-fig S5-data1-v1/S5A/bottom/PCNA.tif]

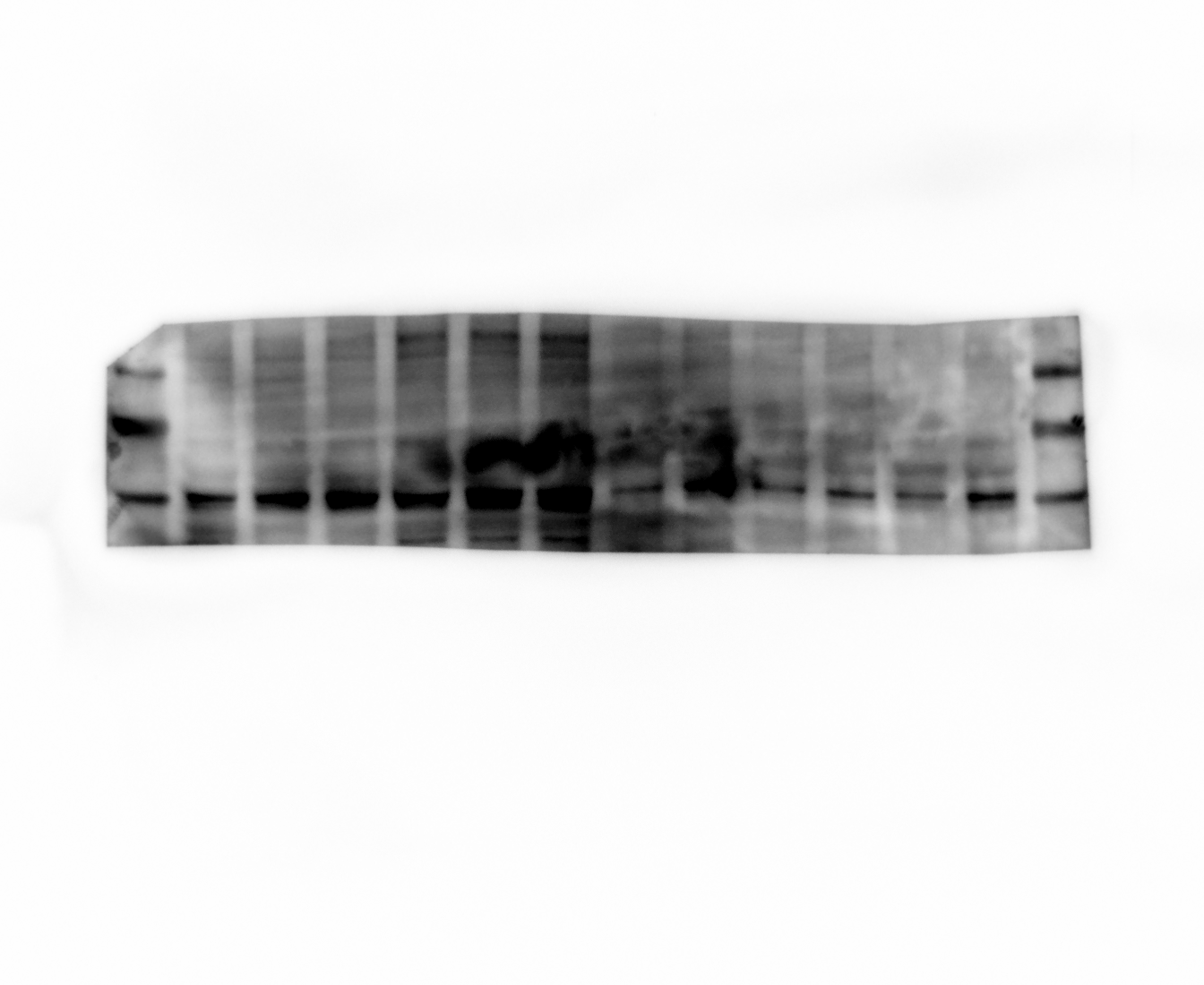

Supplement: Figure 8—figure supplement 3—source data 1. [file elife-98524-fig8-figsupp3-data1.zip › Fig 8-fig S5-data1-v1/S5A/bottom/β-catenin.tif]

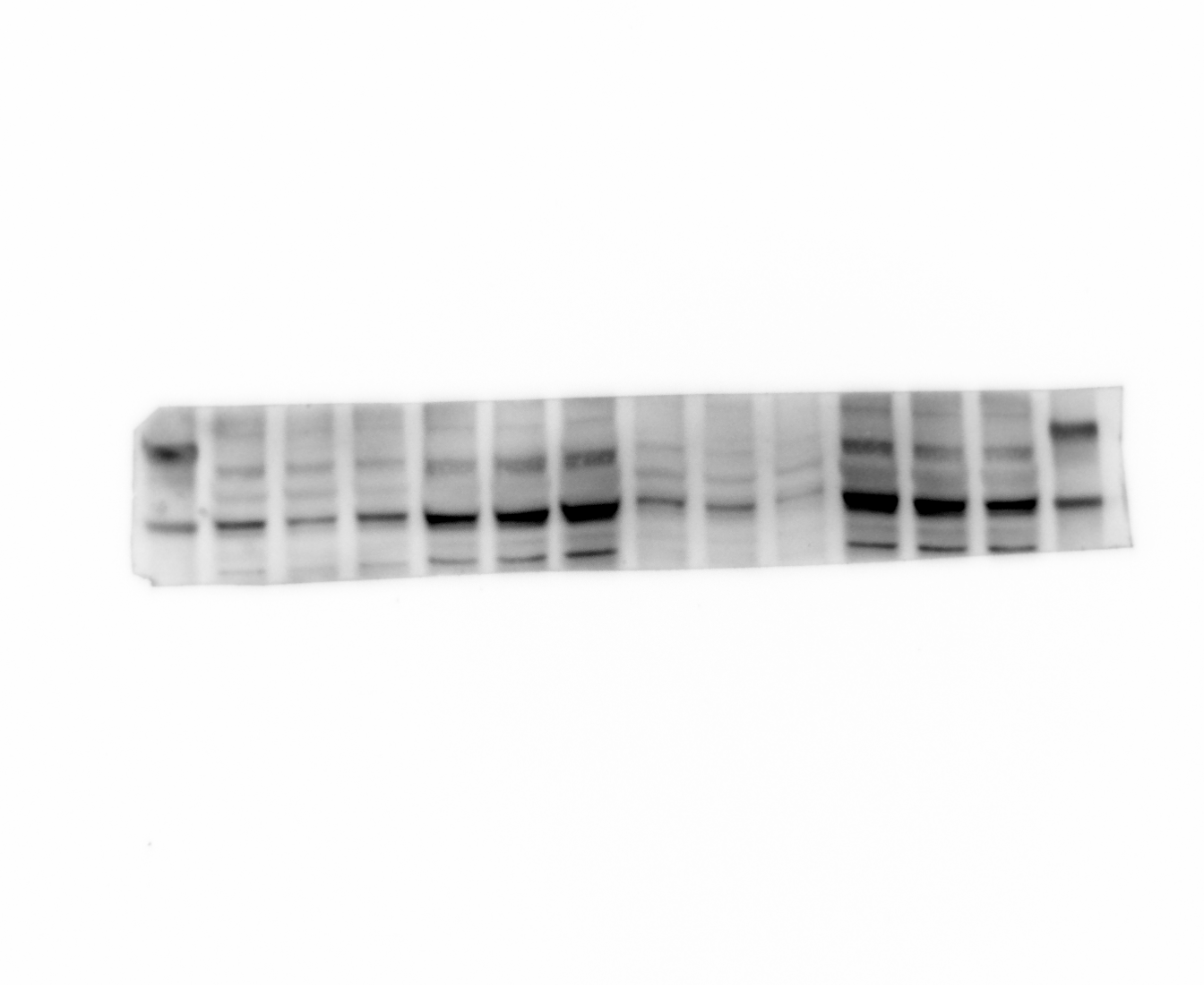

Supplement: Figure 8—figure supplement 3—source data 1. [file elife-98524-fig8-figsupp3-data1.zip › Fig 8-fig S5-data1-v1/S5A/upper/CCN2.tif]

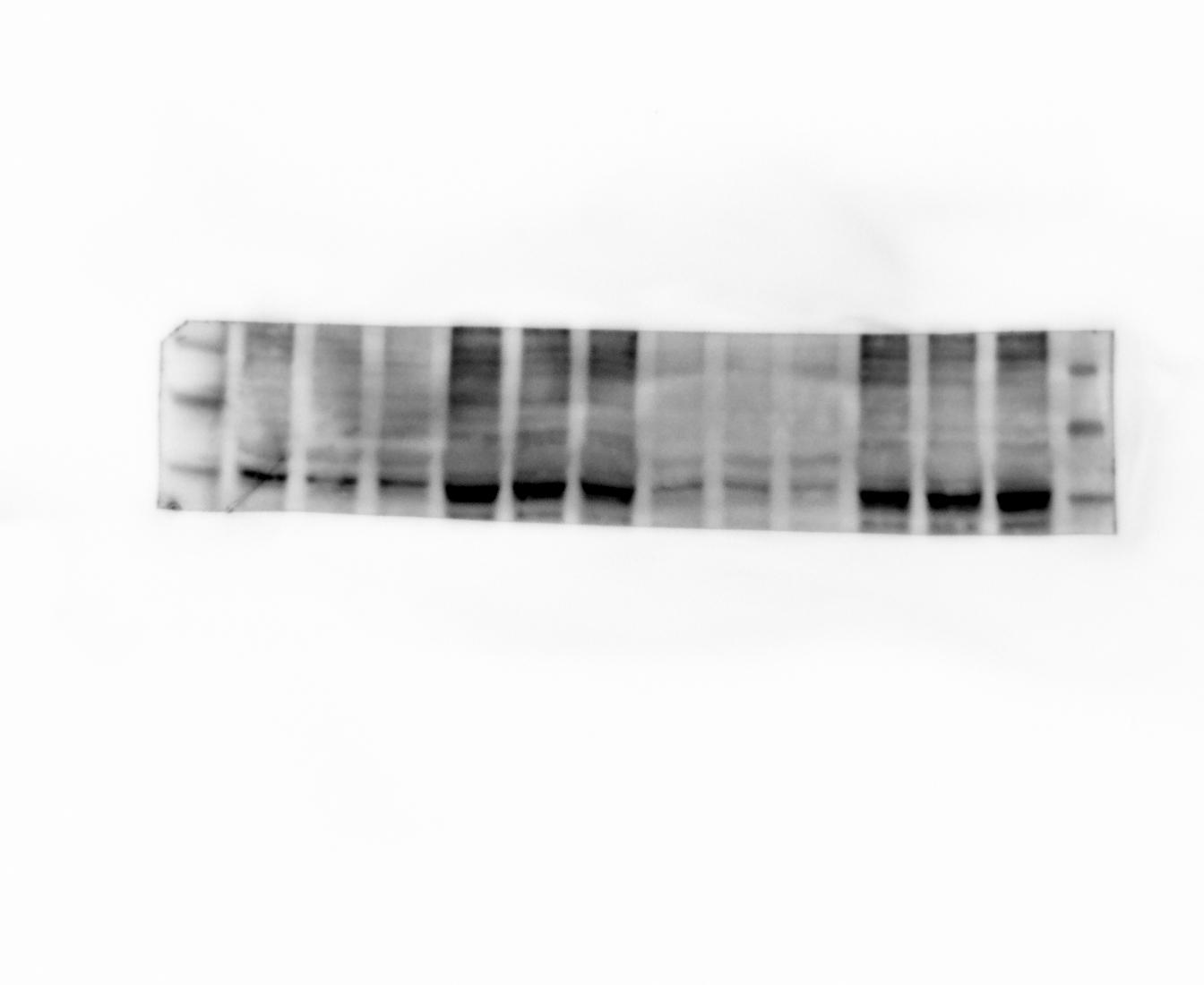

Supplement: Figure 8—figure supplement 3—source data 1. [file elife-98524-fig8-figsupp3-data1.zip › Fig 8-fig S5-data1-v1/S5A/upper/COL3A1.tif]

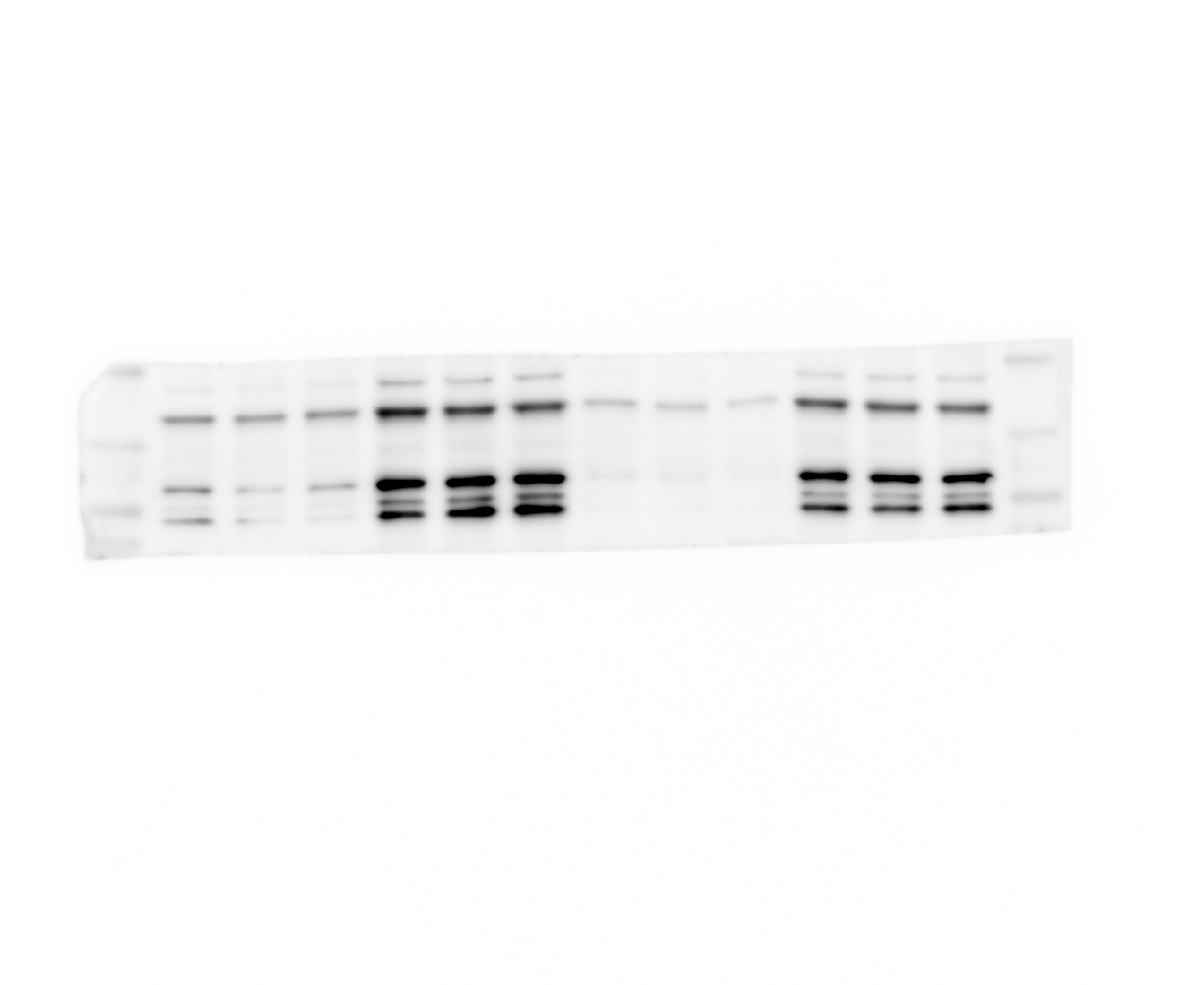

Supplement: Figure 8—figure supplement 3—source data 1. [file elife-98524-fig8-figsupp3-data1.zip › Fig 8-fig S5-data1-v1/S5A/upper/FN1.tif]

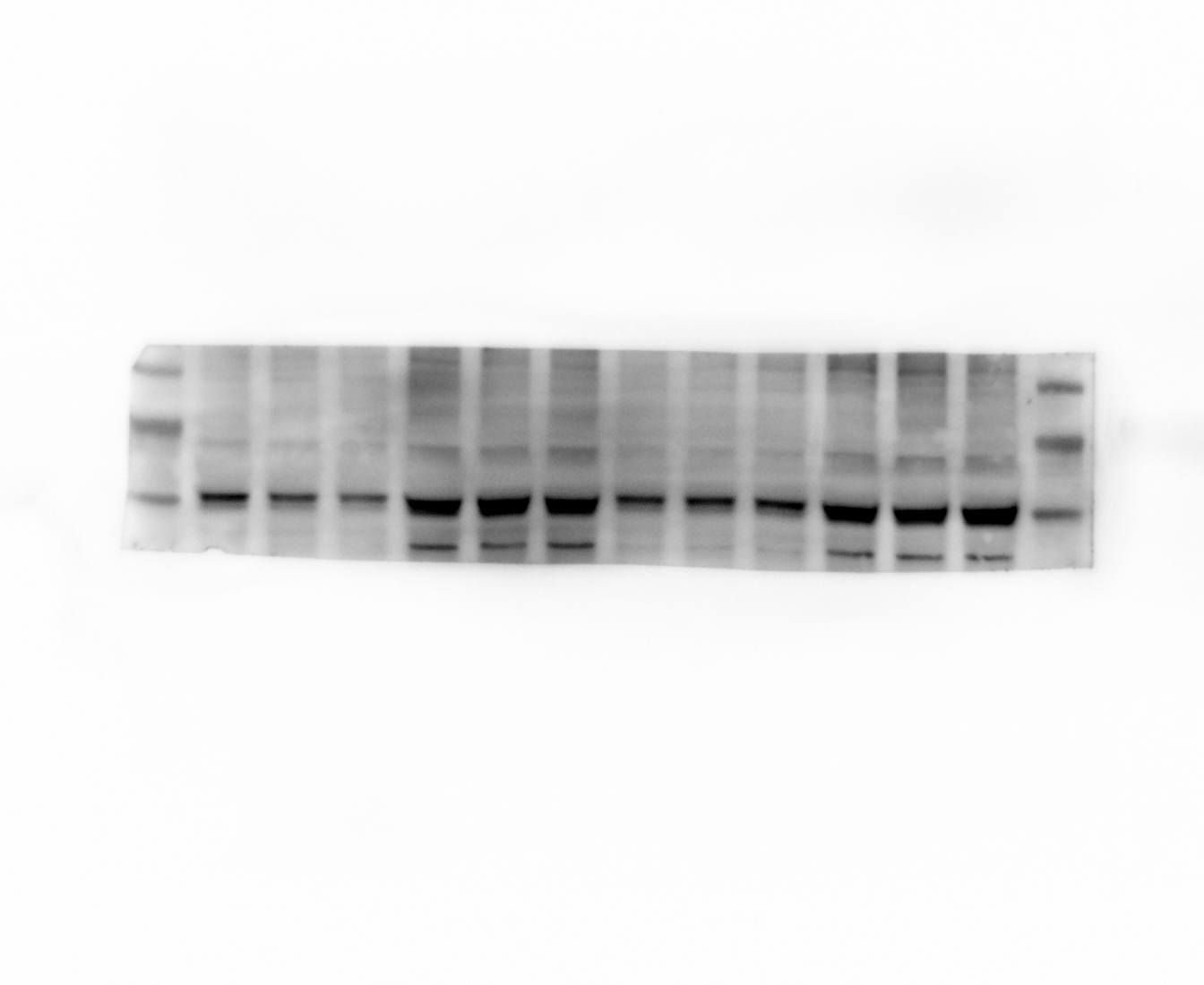

Supplement: Figure 8—figure supplement 3—source data 1. [file elife-98524-fig8-figsupp3-data1.zip › Fig 8-fig S5-data1-v1/S5A/upper/SIRT4.tif]

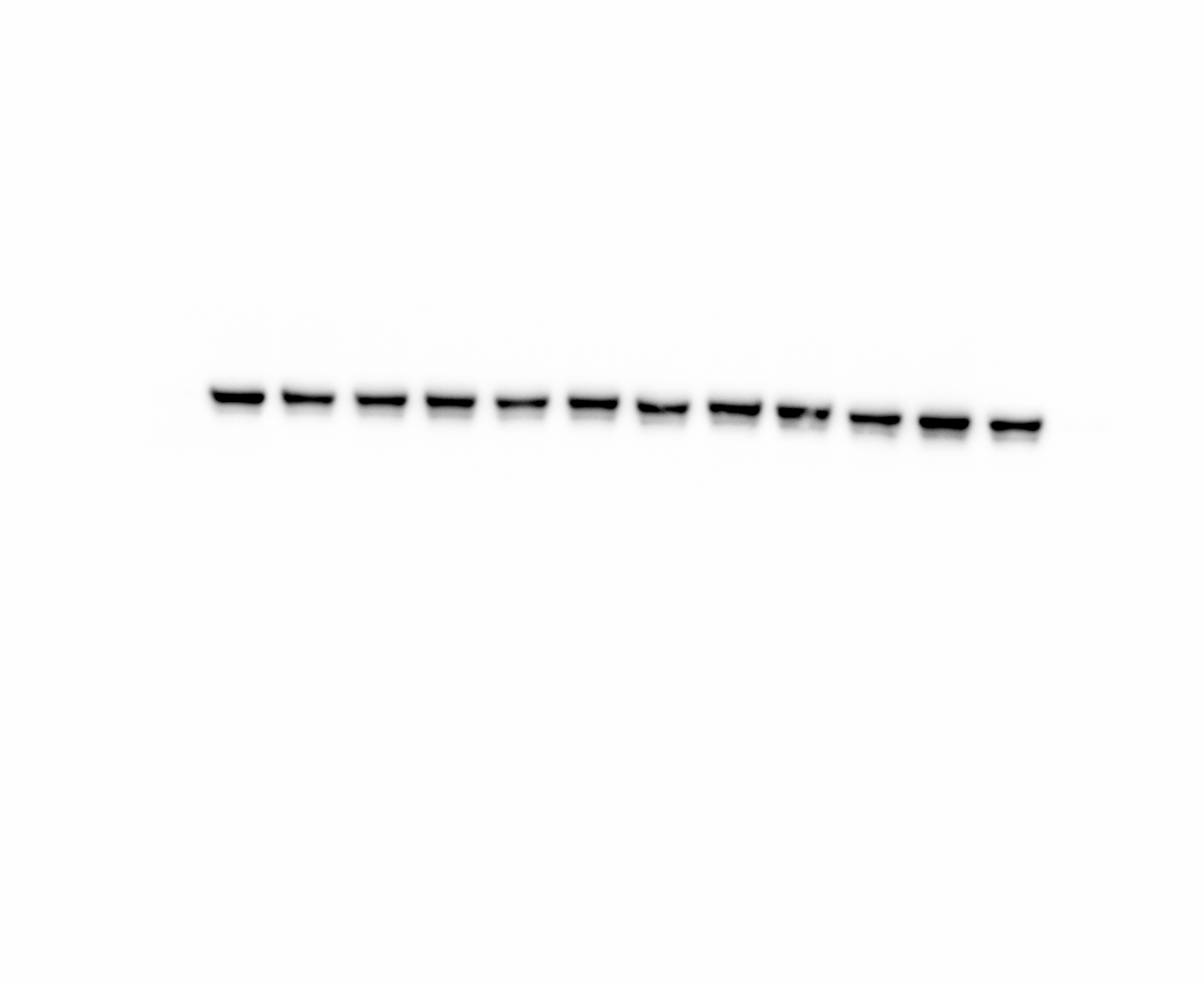

Supplement: Figure 8—figure supplement 3—source data 1. [file elife-98524-fig8-figsupp3-data1.zip › Fig 8-fig S5-data1-v1/S5A/upper/Tubulin.tif]

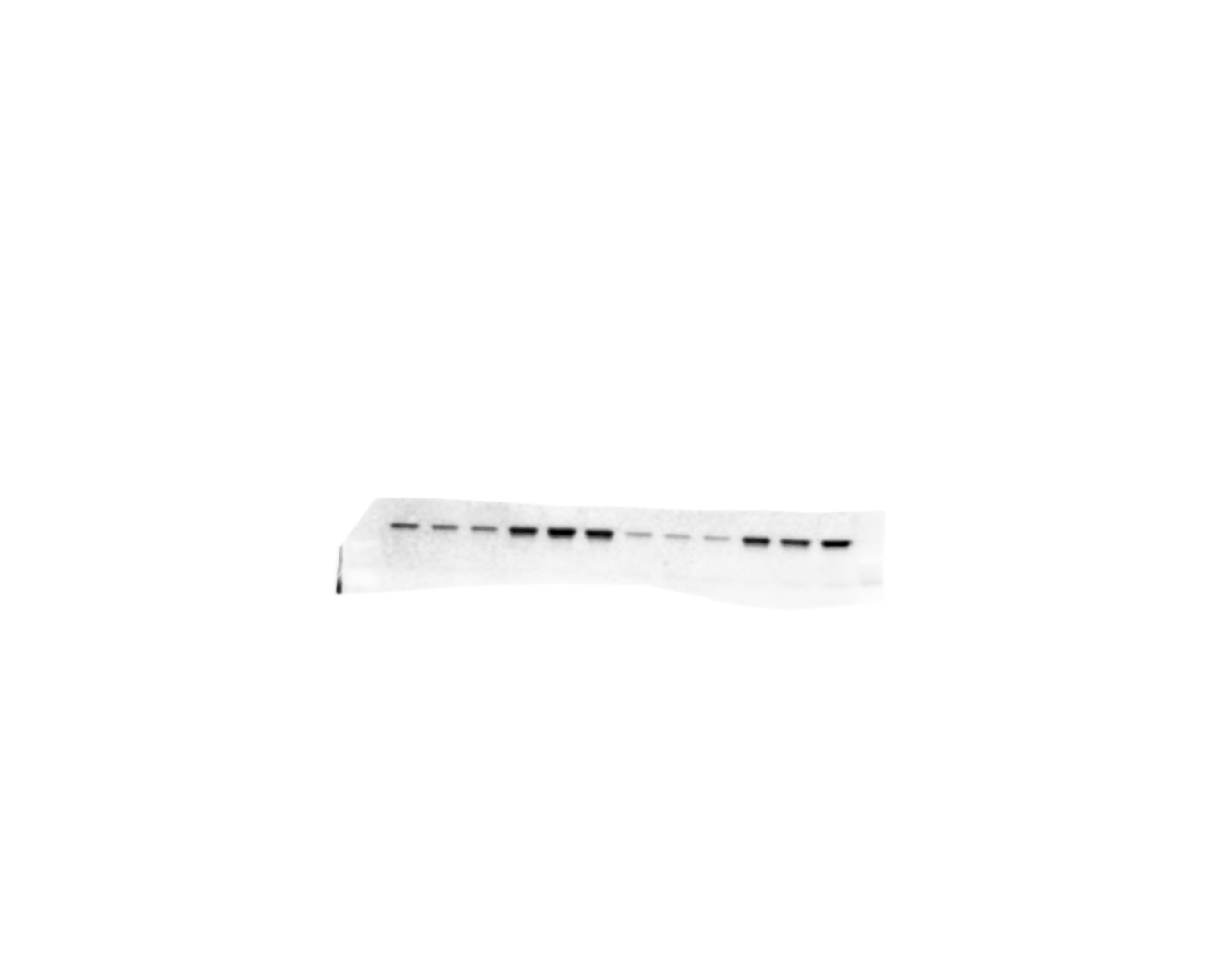

Supplement: Figure 8—figure supplement 3—source data 1. [file elife-98524-fig8-figsupp3-data1.zip › Fig 8-fig S5-data1-v1/S5A/upper/α-SMA.tiff]

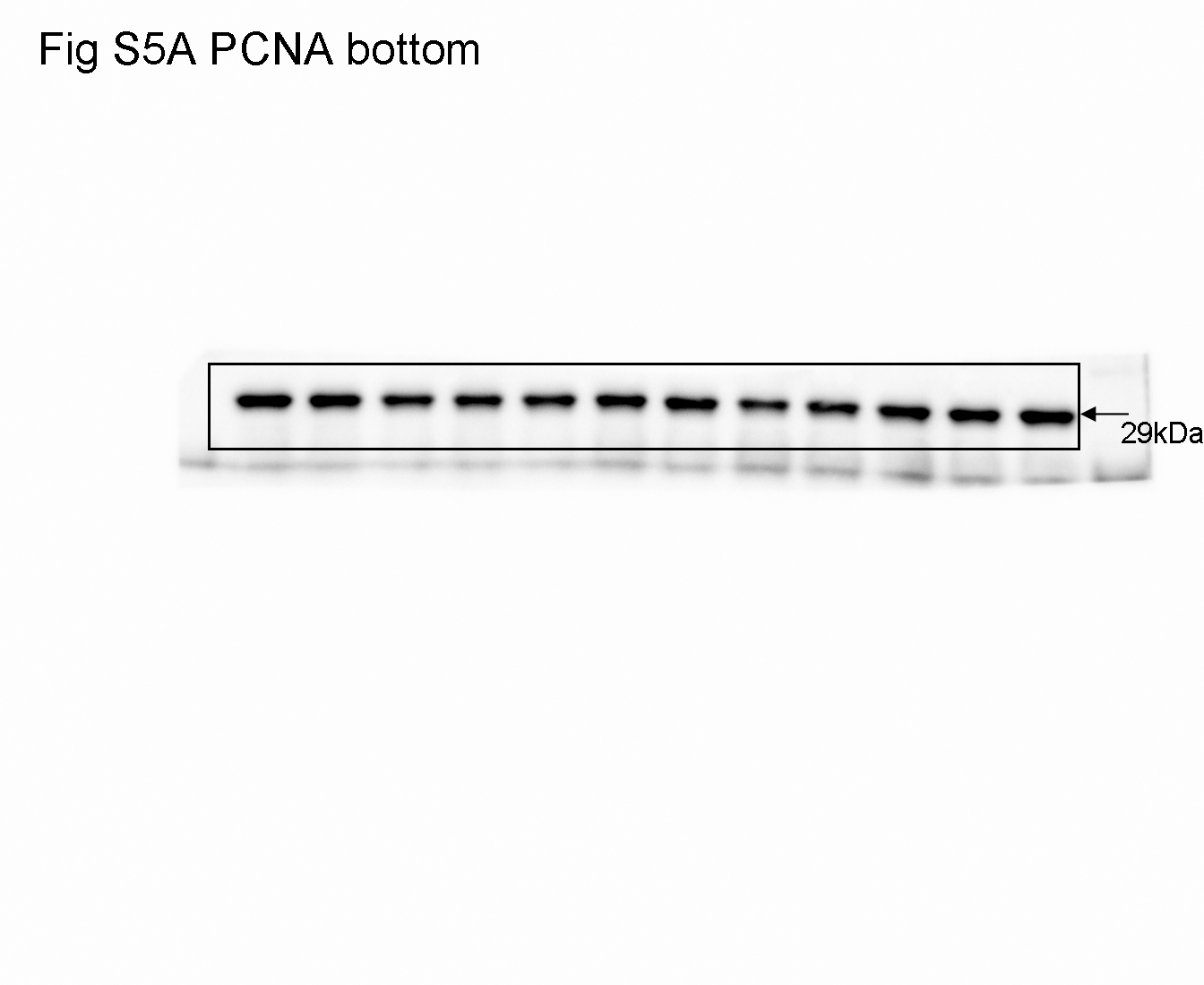

Supplement: Figure 8—figure supplement 3—source data 2. [file elife-98524-fig8-figsupp3-data2.zip › Fig 8-fig S5-data2-v1/S5A/bottom/PCNA.tif]

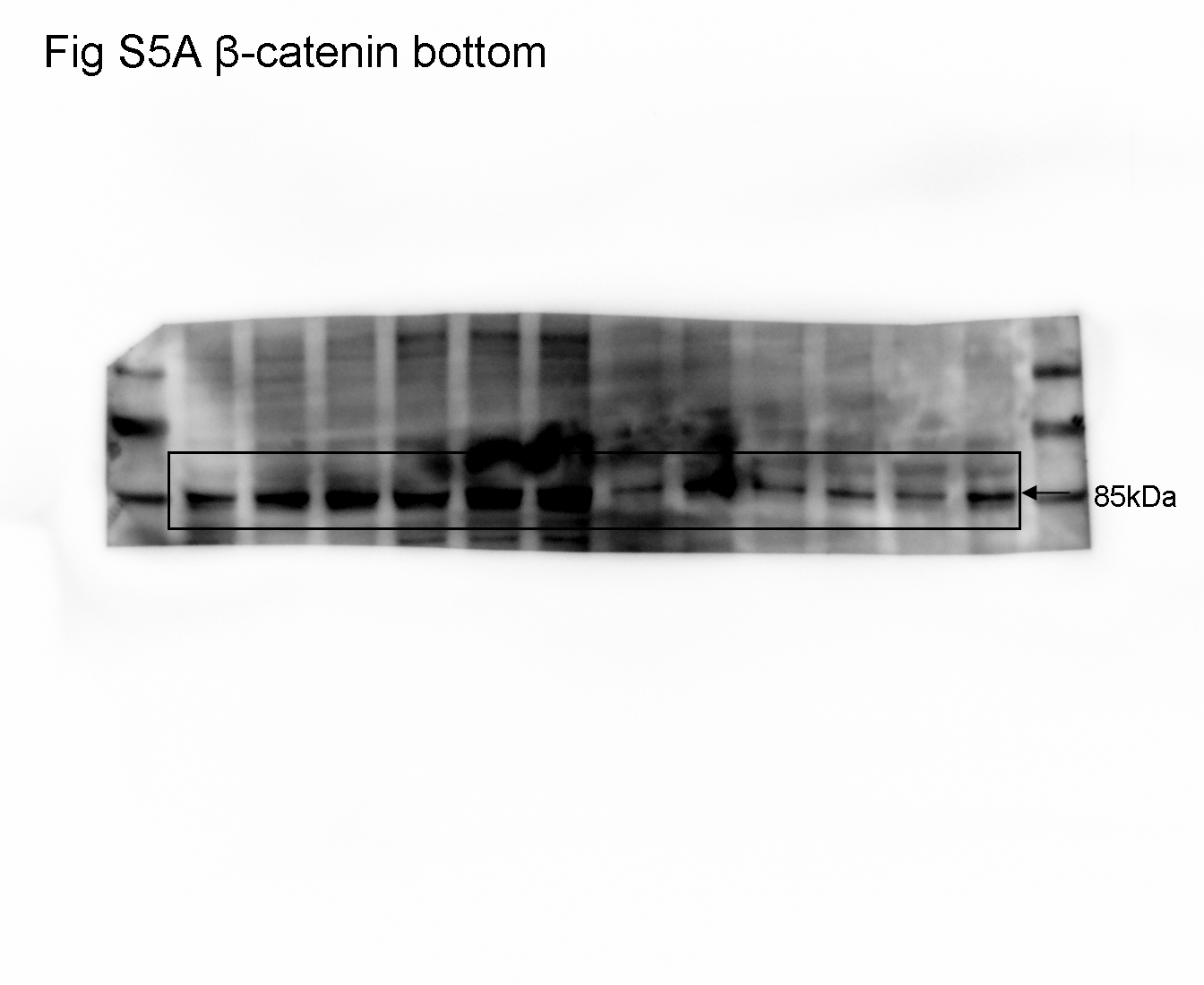

Supplement: Figure 8—figure supplement 3—source data 2. [file elife-98524-fig8-figsupp3-data2.zip › Fig 8-fig S5-data2-v1/S5A/bottom/β-catenin.tif]

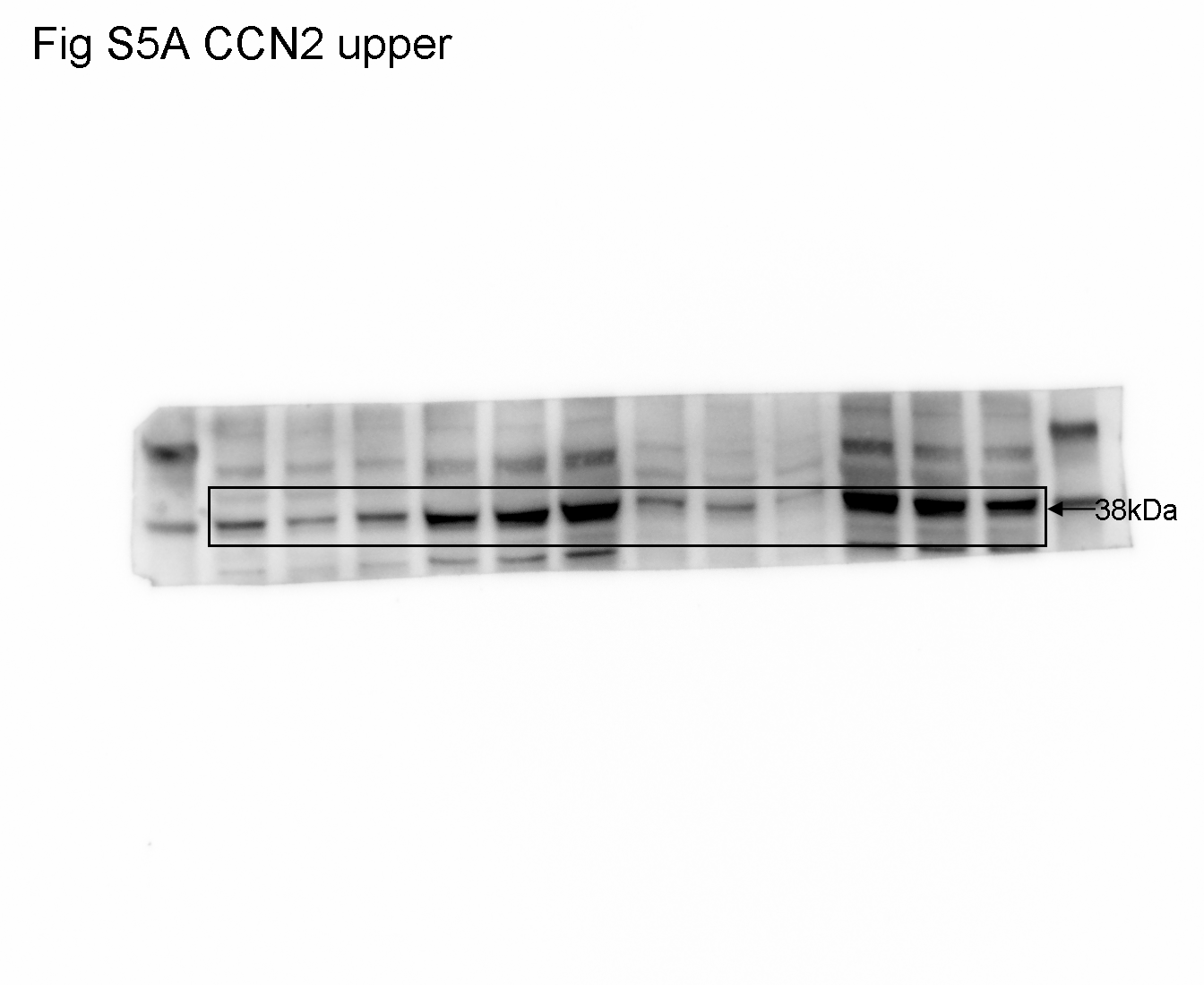

Supplement: Figure 8—figure supplement 3—source data 2. [file elife-98524-fig8-figsupp3-data2.zip › Fig 8-fig S5-data2-v1/S5A/upper/CCN2.tif]

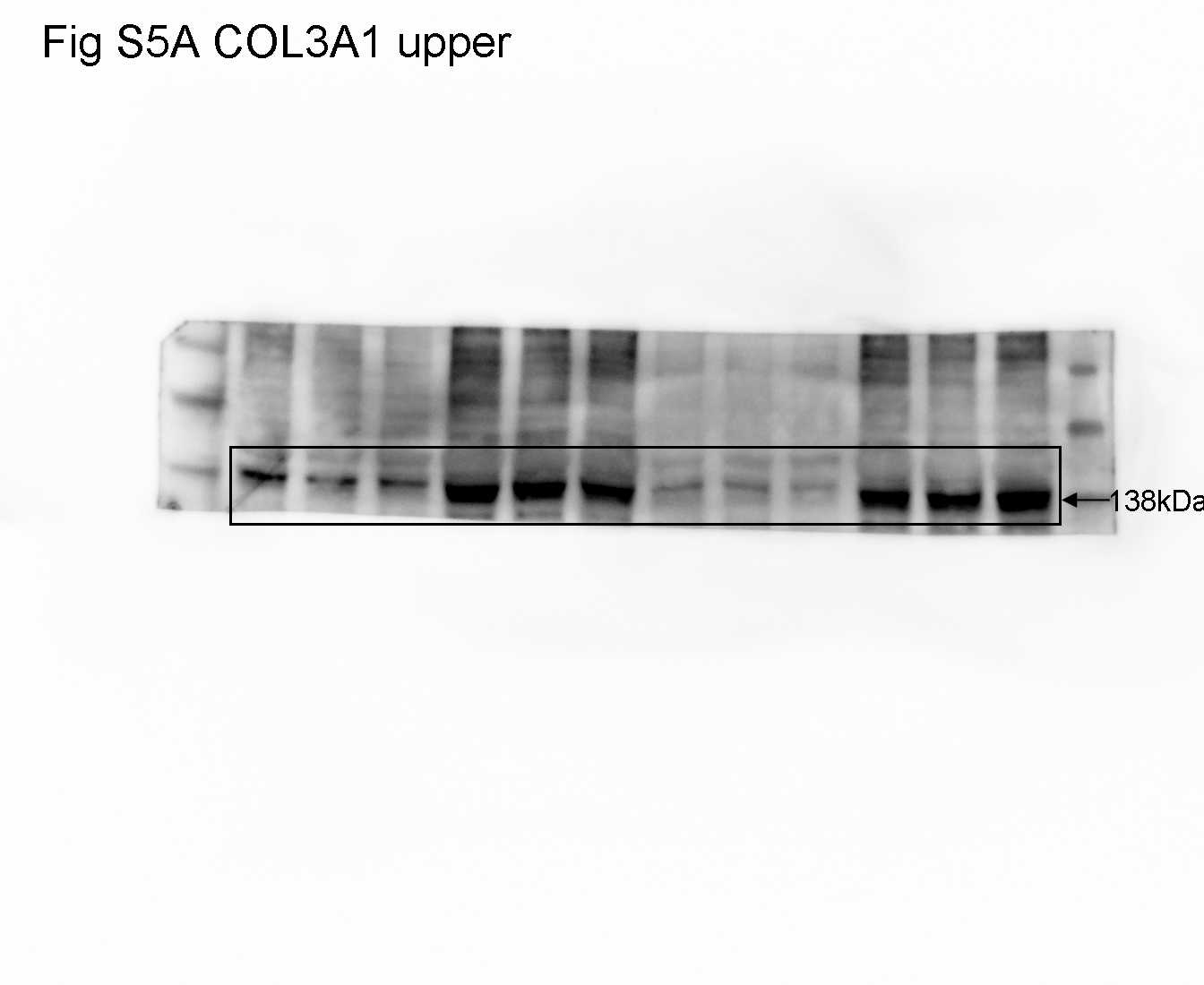

Supplement: Figure 8—figure supplement 3—source data 2. [file elife-98524-fig8-figsupp3-data2.zip › Fig 8-fig S5-data2-v1/S5A/upper/COL3A1.tif]

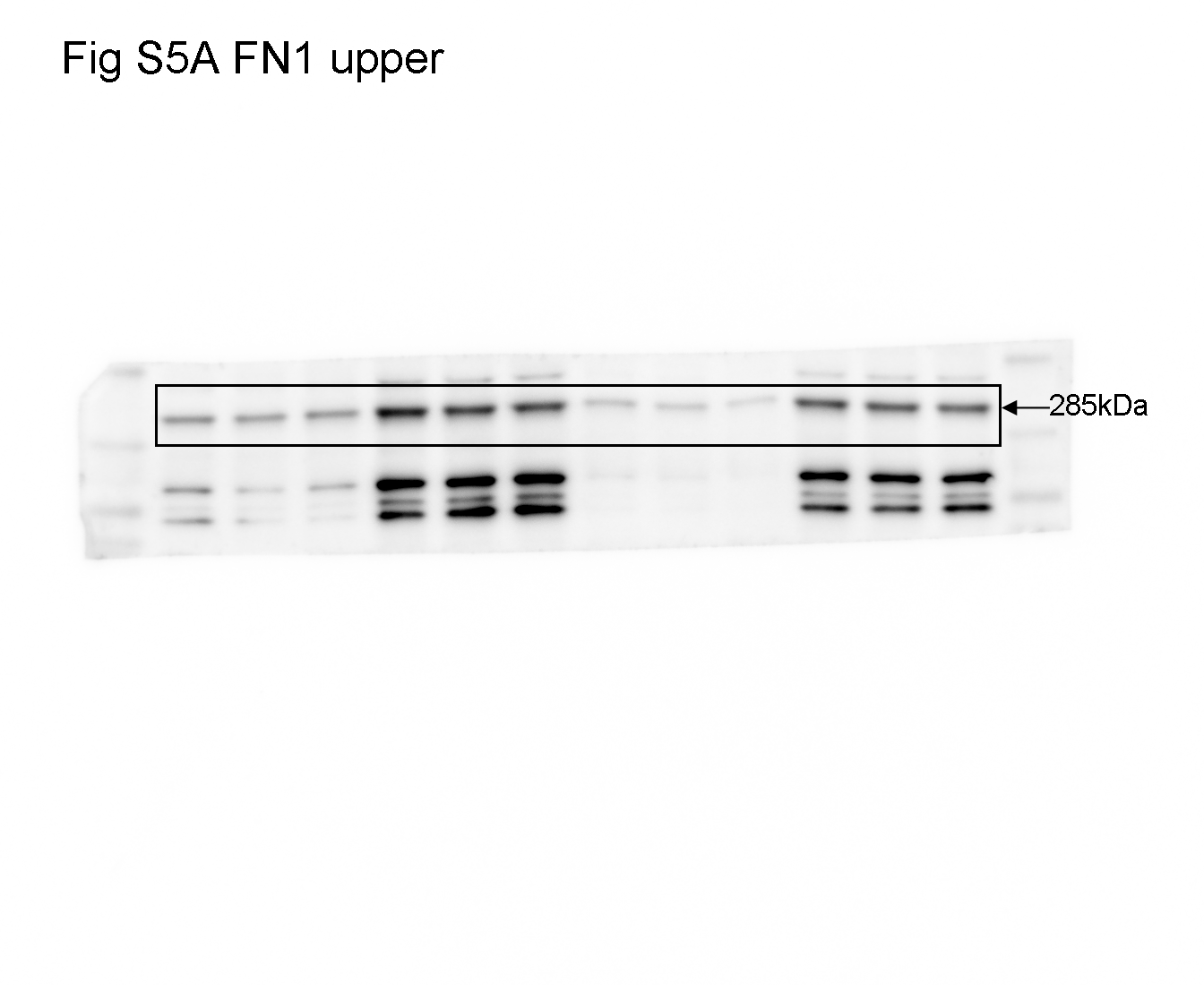

Supplement: Figure 8—figure supplement 3—source data 2. [file elife-98524-fig8-figsupp3-data2.zip › Fig 8-fig S5-data2-v1/S5A/upper/FN1.tif]

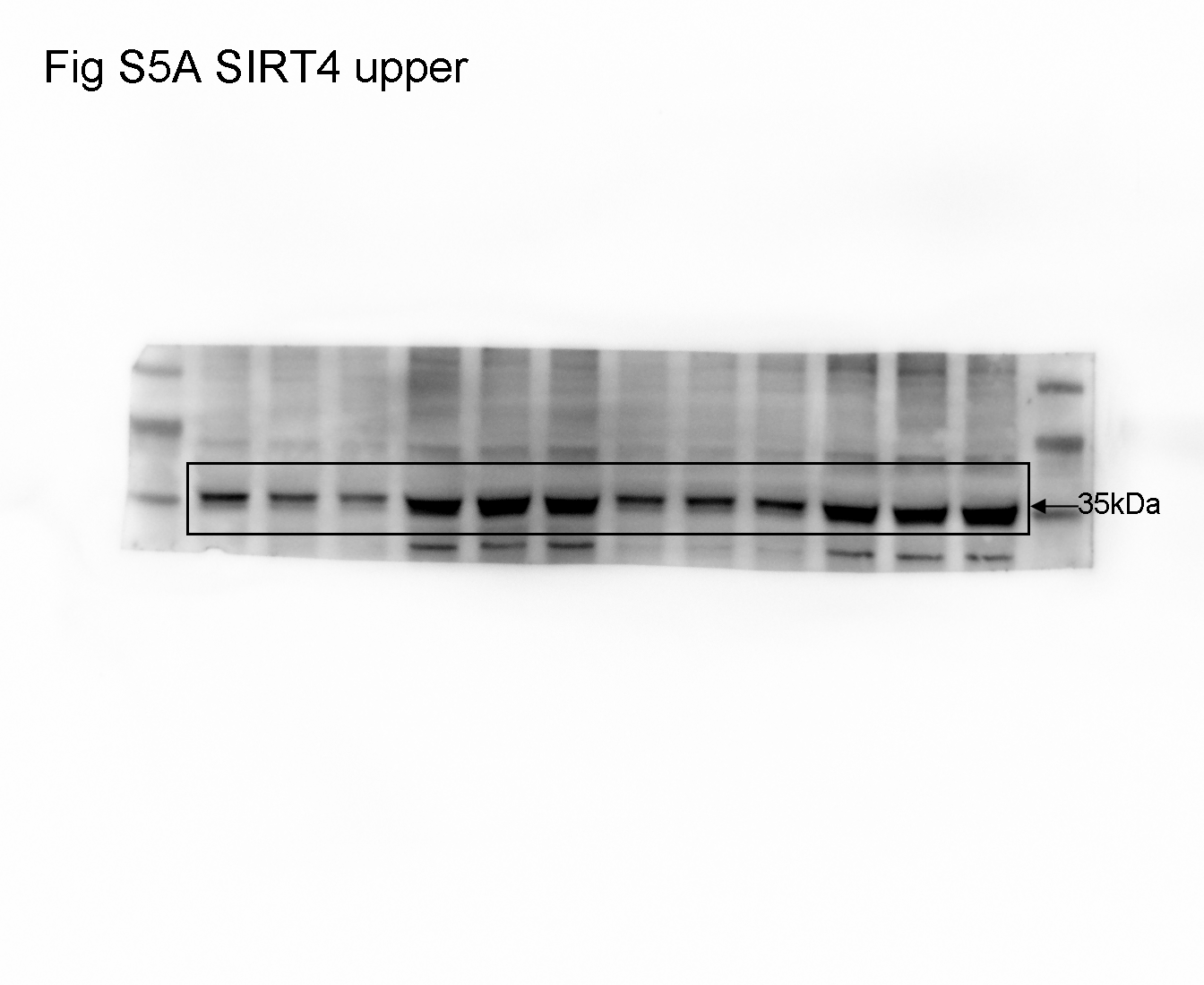

Supplement: Figure 8—figure supplement 3—source data 2. [file elife-98524-fig8-figsupp3-data2.zip › Fig 8-fig S5-data2-v1/S5A/upper/SIRT4.tif]

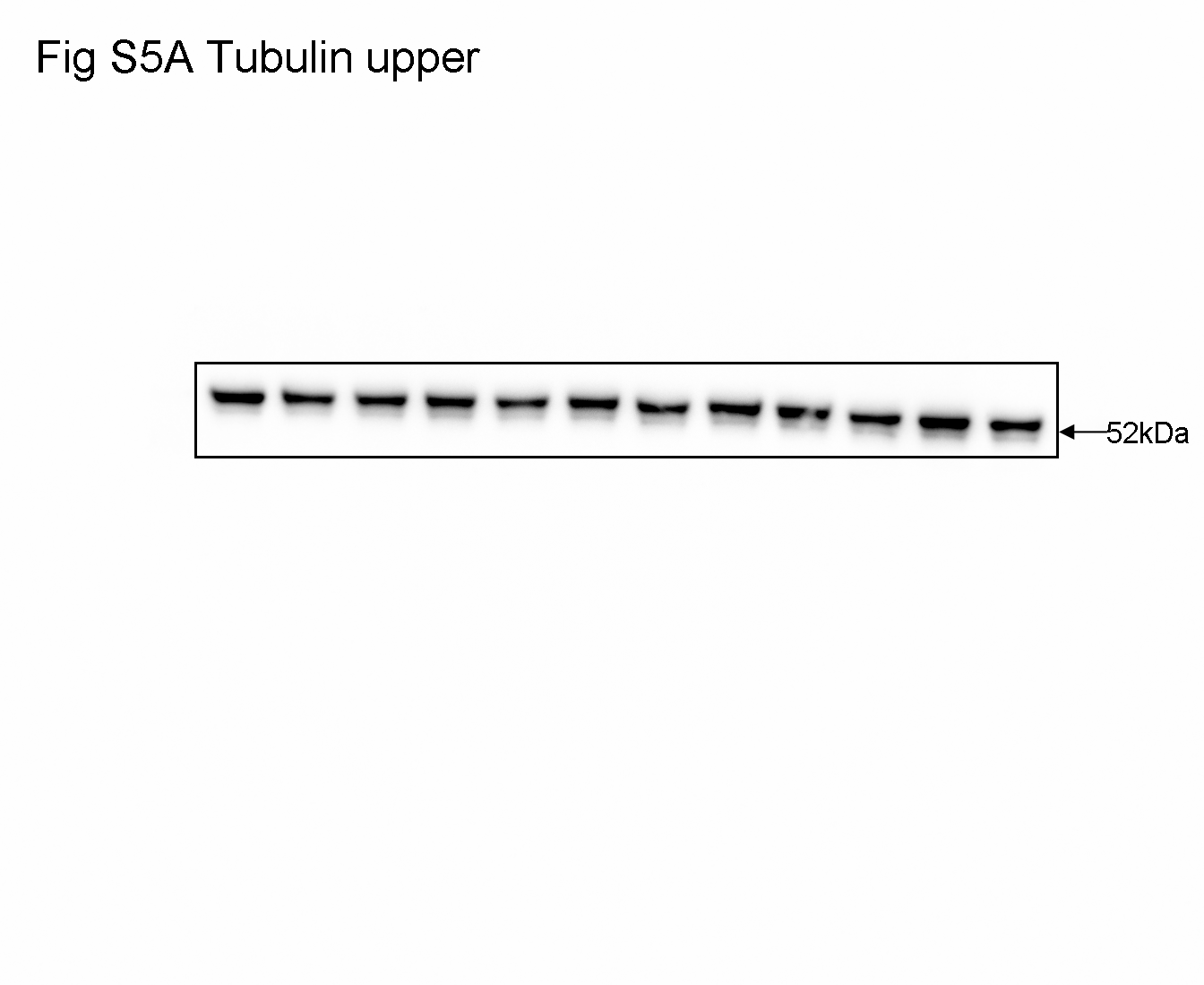

Supplement: Figure 8—figure supplement 3—source data 2. [file elife-98524-fig8-figsupp3-data2.zip › Fig 8-fig S5-data2-v1/S5A/upper/Tubulin.tif]

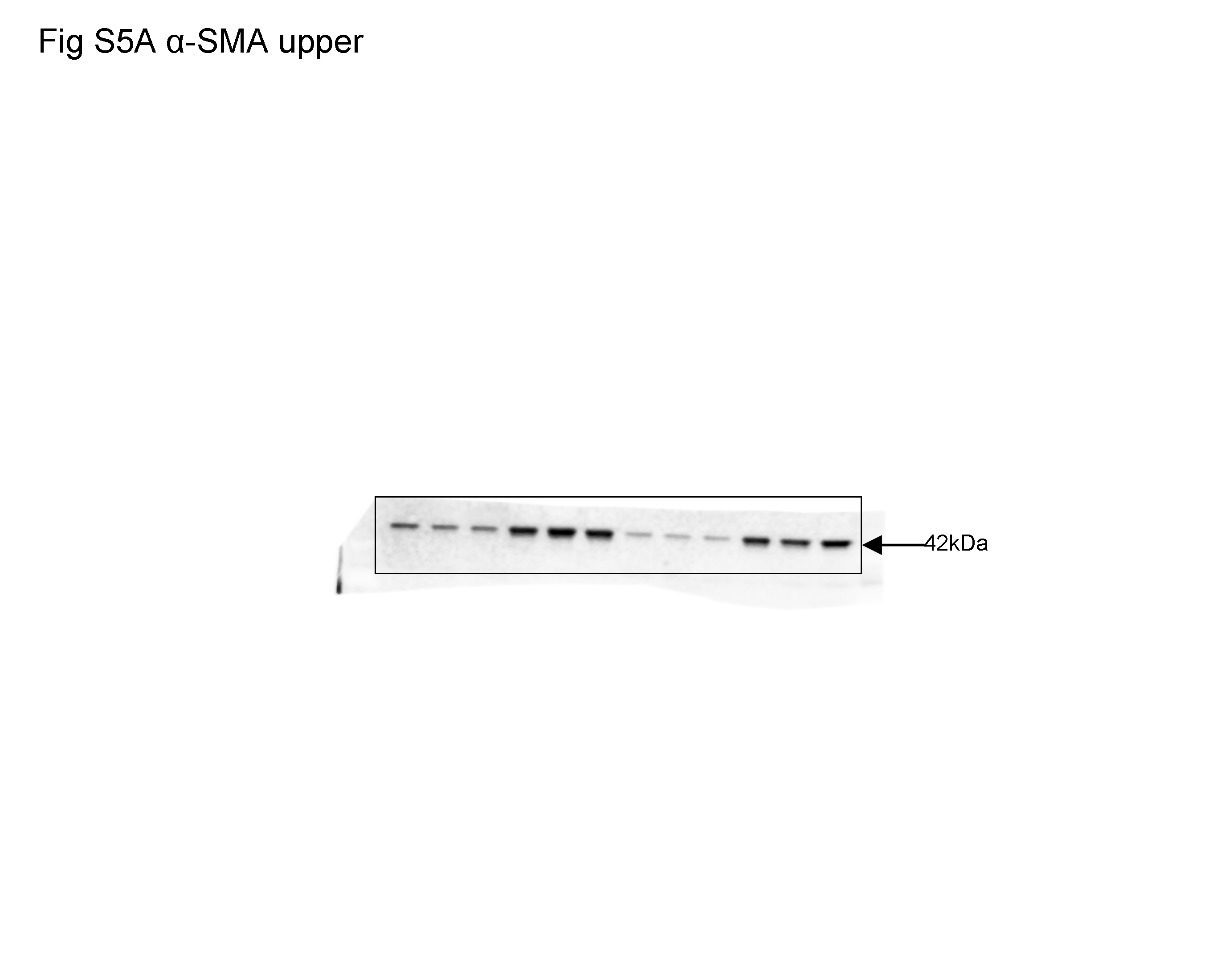

Supplement: Figure 8—figure supplement 3—source data 2. [file elife-98524-fig8-figsupp3-data2.zip › Fig 8-fig S5-data2-v1/S5A/upper/α-SMA.tiff]
